# An individual urinary proteome analysis in normal human beings to define the minimal sample number to represent the normal urinary proteome

**DOI:** 10.1186/1477-5956-10-70

**Published:** 2012-11-21

**Authors:** Xuejiao Liu, Chen Shao, Lilong Wei, Jindan Duan, Shuzhen Wu, Xuewang Li, Mingxi Li, Wei Sun

**Affiliations:** 1 Department of Nephrology, Peking Union Medical College Hospital, Chinese Academy of Medical Sciences, No. 1 Shuaifuyan, Wangfujing Street, Beijing, China; 2 Department of Physiology and Pathophysiology, National Key Laboratory of Medical Molecular Biology, Institute of Basic Medical Sciences Chinese Academy of Medical Sciences/School of Basic Medicine Peking Union Medical College, 5 Dong Dan San Tiao, Beijing, China; 3 Clinical laboratory, China-Japan Friendship Hospital, 2 Yinghua Dongjie, Hepingli, Beijing, China; 4 Core facility of instrument, Institute of Basic Medical Sciences Chinese Academy of Medical Sciences/School of Basic Medicine Peking Union Medical College, 5 Dong Dan San Tiao, Beijing, China

**Keywords:** Urinary proteome, Minimal sample number

## Abstract

**Background:**

The urinary proteome has been widely used for biomarker discovery. A urinary proteome database from normal humans can provide a background for discovery proteomics and candidate proteins/peptides for targeted proteomics. Therefore, it is necessary to define the minimum number of individuals required for sampling to represent the normal urinary proteome.

**Methods:**

In this study, inter-individual and inter-gender variations of urinary proteome were taken into consideration to achieve a representative database. An individual analysis was performed on overnight urine samples from 20 normal volunteers (10 males and 10 females) by 1DLC/MS/MS. To obtain a representative result of each sample, a replicate 1DLCMS/MS analysis was performed. The minimal sample number was estimated by statistical analysis.

**Results:**

For qualitative analysis, less than 5% of new proteins/peptides were identified in a male/female normal group by adding a new sample when the sample number exceeded nine. In addition, in a normal group, the percentage of newly identified proteins/peptides was less than 5% upon adding a new sample when the sample number reached 10. Furthermore, a statistical analysis indicated that urinary proteomes from normal males and females showed different patterns. For quantitative analysis, the variation of protein abundance was defined by spectrum count and western blotting methods. And then the minimal sample number for quantitative proteomic analysis was identified.

**Conclusions:**

For qualitative analysis, when considering the inter-individual and inter-gender variations, the minimum sample number is 10 and requires a balanced number of males and females in order to obtain a representative normal human urinary proteome. For quantitative analysis, the minimal sample number is much greater than that for qualitative analysis and depends on the experimental methods used for quantification.

## Introduction

Human urine is mainly composed of shed cells, debris, and secreted components from the urinary tract as well as blood components that have passed through glomerular filtration and renal tubule reabsorption. Therefore, urine contains useful information not only regarding the kidney and urinary tract, but also about more distant organs. Analysis of the urinary proteome could aid the discovery of biomarkers for both urogenital and systemic diseases. Moreover, compared to serum, human urine is relatively simple and easy to collect, which makes urinary proteome analysis an attractive approach in clinical proteomics research.

Because inherent and environmental factors may influence the components of the urinary proteome, the biological and technical variations are important issues for urinary proteome research. Many groups [1-6] have contributed data regarding this issue and found that (1) a considerable degree of variation can be found in intra-day (collection from one volunteer at different daily time points), intra-individual (collection from one volunteer on different days), and inter-individual (collection from different volunteers) samples; (2) the variation of five intra-day samples (first morning, second morning, 24 h, random, and water loading void) was similar; (3) the variation of intra-individual samples was less than that of inter-individual samples; and (4) technical variation was less than biological variation. Although great variations have been found in different urinary samples, a number of urinary proteins were demonstrated to be consistently present in urine samples collected at different time points and from different individuals [4]. Moreover, Nagaraj *et al.* used healthy volunteers to construct a common dataset of 500 urinary proteins [5]. Taken together, the findings to date indicate that the urinary proteome is relatively stable and a good source for disease biomarkers.

Since the first urinary proteome analysis was published in 2001 [7], many clinical urinary proteome differential analyses have been reported, including analyses of samples from urogenital diseases (kidney transplantation [8], diabetic nephropathy [9], obstructive nephropathy [10], bladder cancer [11], prostate cancer [12], and others) and non-urogenital diseases (hematopoietic stem cell transplantation [13], coronary artery disease [14], and others).

A urinary proteome database from normal human samples plays an important role in biomarker discovery. In the discovery stage, a database could be used as a control for a disease group. In the validation stage, the candidate proteins/peptides could be selected from the database for MS-based or immuno-based validation. Many groups have analyzed the normal human urinary proteome using various approaches and have identified more than 2500 urinary proteins to date [15]. Analyses of the normal urinary proteome have usually analyzed pooled or individual samples from several volunteers [15-17]. However, due to the variations in the urinary proteome, it is still unknown whether these data represent the true pattern of the normal urinary proteome. If the sample number was less than the minimal number required for a representative database in a group, then an analysis may only represent the pattern of selected individuals and not the entire group, which would be misleading for subsequent studies. Therefore, to obtain a representative urinary proteome, it is necessary to define the minimal urinary sample number needed. To the best of our knowledge, such an analysis has not been conducted to date.

**Figure 1:**
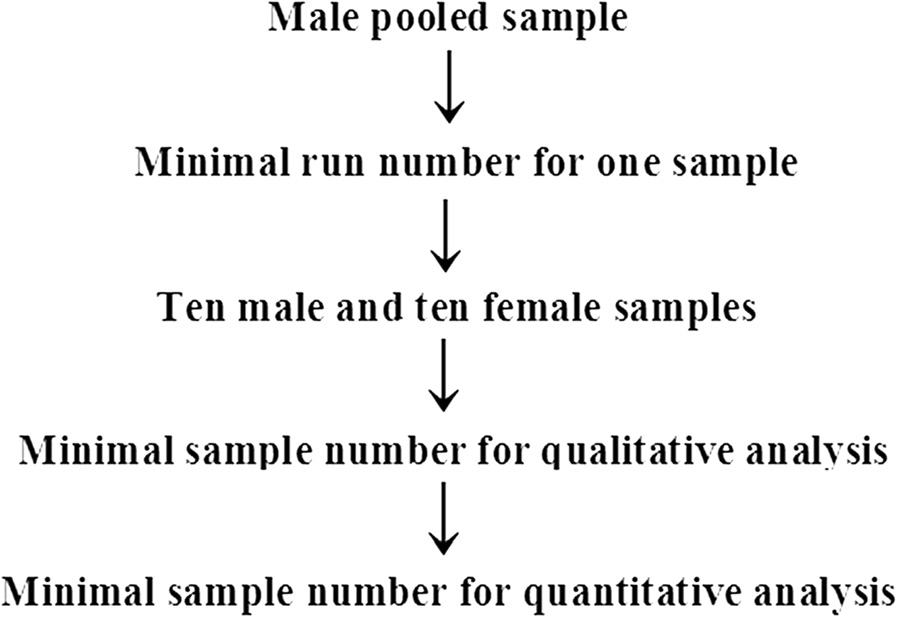
**The overall workflow of this study.** Pooled male samples were analyzed in replicate to define the minimal 1DLC/MS/MS number for a comprehensive analysis. After validating the analytical completeness in 10 males and 10 females, the minimal sample size in a male/female group was defined. Within a group, the minimal sample size and the ratio of males and females was further investigated.

In this study, inter-individual and inter-gender variations were taken into consideration to achieve a representative urinary proteome. An individual urinary proteome analysis of 10 male and 10 female normal overnight samples from healthy volunteers was used to define the minimal samples number required. Because the data-dependent acquisition mode in LC/MS/MS analysis is biased against low abundance proteins [18], replicate experimental strategies are often used to obtain a comprehensive analysis [18-20], and therefore this strategy was also adopted for this study. To determine how many runs are necessary to obtain a comprehensive result for one urine sample by 1DLC/MS/MS, a pooled sample from ten male samples was analyzed with forty runs. Based on these calculations, 10 male and 10 female urinary samples were then analyzed by replicate 1DLC/MS/MS. For qualitative analysis by intra-gender and inter-gender analysis, the minimal sample number for male, female, and normal groups was estimated. For quantitative analysis, the variation of protein abundance was defined by spectrum count and western blotting methods. And then the minimal sample number for quantitative proteomic analysis was estimated. The overall workflow is shown in Figure 1.

## Materials and methods

### Apparatus

An LTQ XL mass spectrometer was purchased from Thermo-Fisher (San Jose, CA). A 1200 nano-HPLC system was obtained from Agilent (Foster, CA). An ADVANCE CaptiveSpray source for Thermo and C18 reverse phase capillary column was purchased from Michrom Bioresources (Auburn, CA). The microwave oven used in this study was solid-state Whirlpool model VIP271 (Shanghai, China), and the maximum output power was 850 W.

### Reagents

Deionized water from a MilliQ RG ultrapure water system (Bedford, MA) was used at all times. HPLC grade acetonitrile (ACN) and formic acid, ammonium bicarbonate, iodoacetamide, dithiothreitol (DTT), sequencing grade modified trypsin, and protease inhibitor phenylmethylsulfonyl fluoride (PMSF) were purchased from Sigma-Aldrich (St. Louis, MO).

### Urine collection

Overnight urine samples were collected from twenty consenting individuals, including 10 males and 10 females (average age 28 and 31 y, respectively). The donors had no acute or chronic illnesses and were not taking any prescription or over-the-counter medications. No female was menstruating at the time of urine collection. Each specimen was collected in 250 mL conical tubes. The samples were immediately acidified to pH 2.7 with hydrochloric acid and then cooled to 4 °C to prevent bacterial growth and proteolysis.

### Acetone precipitation

All procedures were performed at 4 °C. Urine samples were centrifuged at 5,000 x g for 30 min and the pellets were removed. The supernatants were then precipitated with 75% v/v acetone for 16 h followed by centrifugation at 12,000 x g for 30 min. The pellets were resuspended in lysis buffer (7 M urea, 2 M thiourea, 50 mM Tris, and 50 mM dithioerythritol) and subjected to protein quantitation by the Bradford method. One pooled male sample was mixed using ten male samples with an “equal amounts of protein” criterion.

### Protein digestion

Each sample was digested with trypsin as previously described [21]. Briefly, each sample was reduced with DTT by heating at 100 °C for 5 min and then alkykated with iodoacetamide at room temperature in the dark for 45 min. The samples were then digested with trypsin (1:50) for 1 min under microwave irradiation at 850 W using the following method: samples were placed into 1.5 mL polypropylene vials and a container with 1,000 mL of water was placed beside the sample vials to absorb the extra microwave energy. The microwave oven was turned on for 1 min. After microwave irradiation, the vials were removed from the microwave oven and lyophilized to near dryness.

### 1DLC/MS/MS

All lyophilized samples were redissolved in 0.1% formic acid (buffer A) at a concentration of 5 mg/mL before MS analysis. All peptide mixtures were analyzed on a reverse phase C18 capillary LC column from Michrom Bioresources (100 μm x 150 mm, 3 μm, 0.5 μL/min). The elution gradient was 5-30% buffer B (0.1% formic acid, 99.9% ACN; flow rate, 0.5 μL/min) for 100 min. Eluted peptides were analyzed by an LTQ XL electrospray ion trap mass spectrometer. Ions were detected in a survey scan from 400 to 2000 amu followed by 10 data-dependent MS/MS scans (1 μscan each, isolation width 2 amu, 35% normalized collision energy, dynamic exclusion for 90 s) in a completely automated fashion.

### Western blot analysis

Western blots were performed for three proteins: alpha 1 antitrypsin, ceruloplasmin, and beta-2-microglobulin to confirm the variation in urine. For each protein, 16 normal human overnight urine samples (10 females and 6 males) were used. A urine sample from a stage IV diabetic nephropathy patient was used as a control. Thirty micrograms urine protein from each urine sample after acetone precipitation was separated on a 4-12% NuPAGE gel (Invitrogen). Proteins were then transferred to polyvinylidene difluoride (PVDF) membranes (Millipore, USA). The membranes were blocked for 1 h at room temperature in Tris-buffered saline (TBS) with 5% skim milk, and then incubated overnight at 4 °C in a 1% milk solution containing mouse monoclonal anti-alpha 1 antitrypsin (SERPINA1) antibody (1:1000, ab9400, Abcam), mouse monoclonal anti-ceruloplasmin antibody (1:1000, ab51083, Abcam), and rabbit monoclonal anti-beta-2-microglobulin antibody (1:1000, ab15976, Abcam), respectively. The membranes were washed three times for 5 min with TBST (Tris-buffered saline with 0.05% Tween-20), and then incubated with horseradish peroxidase-labeled goat anti-mouse or mouse anti-rabbit IgG secondary antibodies (diluted 1:5000, Abcam) at room temperature for 3 h. After washing three times for 5 min each in TBST, the membrane was visualized with an ECL detection kit (Millipore, Bedford, MA, USA) using a chemiluminescence imaging system (Millipore). Quantification of protein bands was performed for each sample by determining the relative optical density (ImageJ; National Institutes of Health, Bethesda, MD).

### Data processing

MS/MS spectra were extracted from raw files requiring a minimum of 50 signals with an intensity of at least 1 x 10^5^ U. Extracted MS/MS spectra were automatically assigned to the best-matching peptide sequences using the SEQUEST algorithm [22] and SEQUEST Browser software package Bioworks 3.3.1 SP1. SEQUEST searches were performed on a PC against an IPI human protein database (v3.70, released on 6th September, 2010) [23] containing 87,069 protein sequences downloaded as FASTA-formatted sequences from the Website of the European Bioinformatics Institute ( http://www.ebi.ac.uk/IPI/). To increase search speed, the protein database was preprocessed to create a binary database containing all possible tryptic peptides. A static modification of +57 Da on cysteine residues was used. The peptide mass search tolerance was set to 1.4 Da. Because the number of methionine is relatively few in the database and adding a variable modification maybe significantly increase random match, especially for low resolution instrument (such as LTQ XL used in the work), therefore we did not use the variable modification of methionine oxidation to achieve more accurate database searching results.

Stringent SEQUEST filter criteria were used and included the following: (1) DeltaCn score of at least 0.2; (2) Rsp of 1; and (3) the XCorr cutoff was adjusted to maintain the average false positive rate of all datasets at approximately 1%, leading to the following thresholds: 1.8, 2.8, and 3.3 for single, double, and triple-charged peptides, respectively.

To reduce redundancy in protein identification, peptides identified from tandem spectra were reassigned to proteins using the following procedure: (1) all peptides identified by MS/MS spectra were searched against protein databases to define whether each peptide was unique or shared (i.e., whether the peptide appeared in a single protein or in multiple proteins); (2) a protein identified by unique peptide(s) was marked as a single protein, and any peptide shared with this protein was also assigned to the protein; (3) the remaining shared peptides were redistributed to group proteins using the Occam’s razor constraint [24], which states that the least number of proteins yields the most peptide sequences; and (4) in a group of proteins identified by the same shared peptides, the longest protein and/or the protein annotated by the Swiss-Prot or Trembl databases was chosen to represent the group.

### Statistical analysis

To estimate the number of newly identified proteins along with the increased run/sample numbers, computer simulations were conducted by randomizing the order of each run/sample to be added to the existing run/sample pool. Means and standard deviations of the newly identified rates were calculated based on 5000 simulations to plot all of the saturation curves presented in this study. To further study the intra- and inter-gender variation, the overlap rate of identified proteins was calculated for each pair of samples. Hierarchical clustering analysis was also applied to this qualitative data. The distance between two samples was represented by one minus the overlap rate and the Ward’s minimum variance method was used as the clustering method. All of the statistical analyses were performed using the R program.

### Quantitative analysis and sample size calculation

Spectrum counts of proteins were calculated for the quantitative analysis. Since the LTQ XL mass spectrometer used in this study is of relative low sensitivity, only proteins that were reproducibly identified in at least 80% of the total 291 MS/MS runs were included in this study. To determine the minimal sample size needed to detect protein expression differences with a given level of statistical significance, the pwr.t.test function in the pwr package of the R program was used for power calculation of the two-sample *t* test [25].

## Results

### Overall identification of 21 samples

To achieve a comprehensive analysis of the urinary proteome by 1DLC/MS/MS, a replicate analysis was performed for 21 samples (one pooled male sample, 10 male samples, and 10 female samples). A total of 40 runs were performed for the pooled male sample, and an average of 12.5 runs were performed for each male/female sample, yielding a total of 291 runs for this study. To obtain high confidence results, a reverse database-searching method was used to evaluate random matches. All raw data were searched against the reverse database to estimate the false-positive rate (the false-positive rate = 2 x [spectrum count in reverse database]/[spectrum count in reverse database + spectrum count in forward database] x 100 [%]) [26]. The average false-positive rate of 291 results was approximately 1% based on a stringent SEQUEST criterion (Additional file 1).

In all, a total of 867 proteins, 2,804 peptides, and 152,449 spectra were identified from 21 samples, and an average of 219 proteins, 520 peptides, 7,259 spectra were found in each sample (Table 1, detailed information in Additional files 1,2,3,4). The inter-run overlap rates for protein and peptide identification were 74.12% and 71.23%, respectively, indicating a good reproducibility of the 1DLC/MS/MS analysis. In this study, a replicate 1DLC/MS/MS strategy was used to improve detection sensitivity and to balance the high-abundance protein identification bias of the data-dependent acquisition mode. Compared with a single run, more than 2-fold protein/peptides were identified with the replicate strategy in 21 samples, providing more information on the samples for further analysis (Table 1).

**Table 1:** The identification and inter-run overlap rates for 21 urinary samples

	**Protein**			**Peptide**			**Spectrum**		**Runs**
	**Mean±SD**	**Total**	**Overlap rate (%)**	**Mean±SD**	**Total**	**Overlap rate (%)**	**Mean±SD**	**Total**	
Pooled male sample	86±6	232	76.14±3.49	167±11	457	72.8±2.75	412±39	16,524	40
Male1	94±6	188	77.17±3.04	268±10	481	79.09±1.99	921±66	11,054	12
Male2	122±4	201	82.31±2.8	324±8	556	79.59±3.07	863±26	10,366	12
Male3	120±9	225	79.62±2.73	248±23	494	76.02±3.63	785±56	10,213	13
Male4	150±9	285	77.89±3.43	395±20	803	74.35±4.58	985±55	13,801	14
Male5	73±5	129	79.36±3.12	196±10	374	75.72±2.83	551±28	5519	10
Male6	75±3	132	78.17±2.64	203±10	363	76.89±1.91	785±40	7072	9
Male7	82±5	160	75.21±3.3	162±11	329	71.9±2.24	392±25	3920	10
Male8	82±4	162	74.34±3.2	164±8	336	71.09±2.56	366±26	3294	9
Male9	99±3	197	76.36±2.59	212±8	438	73.15±2.54	513±24	5647	11
Male10	83±5	167	77.23±2.38	192±8	397	73.82±1.78	417±14	5013	12
Female1	101±4	243	69.83±2.85	237±10	558	72.21±1.78	529±22	7941	15
Female2	97±5	212	69.6±3.19	245±10	565	67.85±2.52	446±23	4908	11
Female3	106±8	226	68.99±2.91	227±11	550	61.68±2.19	343±19	3439	10
Female4	104±4	217	72.42±2.61	197±9	479	67.75±1.77	358±14	3942	11
Female5	103±9	254	67.72±4.07	218±19	588	63.89±4.33	415±37	6235	15
Female6	79±4	180	75.13±2.74	152±8	363	70.85±2.51	333±14	5336	16
Female7	141±7	357	70.55±2.36	336±16	867	69.56±1.71	581±31	9310	16
Female8	119±7	297	68.27±2.84	267±15	723	64.17±2.38	443±23	6657	15
Female9	103±6	238	70.37±3.3	198±17	506	66.88±3.82	350±31	4912	14
Female10	120±5	296	69.97±3.75	264±13	701	66.67±3.98	459±22	7346	16
Total		867			2,804			152,449	291

### The minimal 1DLC/MS/MS number for a comprehensive analysis of one urine sample

To estimate the minimal 1DLC/MS/MS number of runs required for a comprehensive analysis using the present instrumentation and protein database, replicate 1DLC/MS/MS runs were statistically analyzed to determine at which point additional experiments produced fewer newly identified proteins. A total of 40 runs were performed in a pooled male sample and identified a total of 232 proteins and 457 peptides, with an average inter-run overlap rate of 76.14% (Table 1).

**Figure 2:**
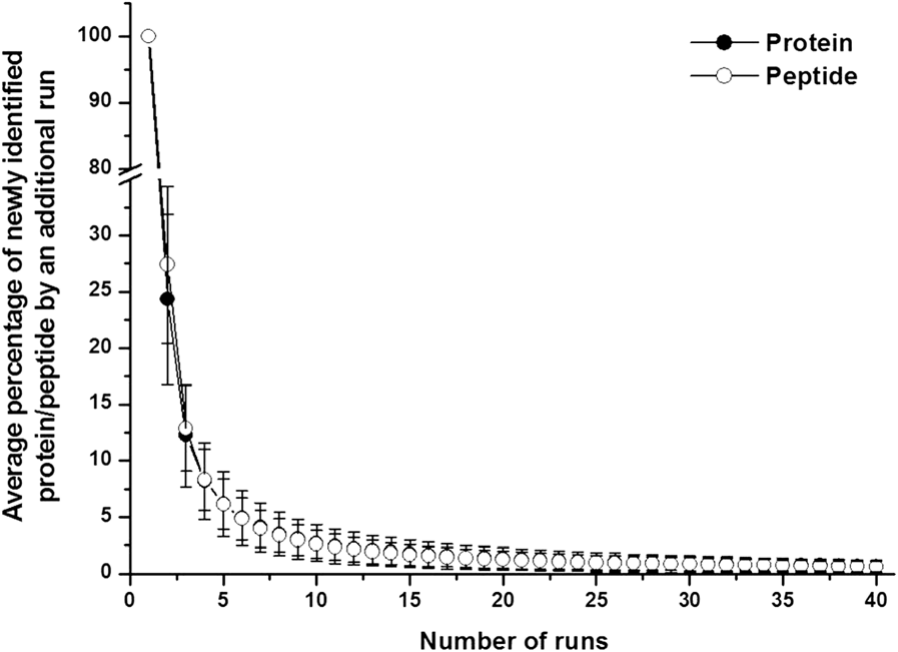
**The newly identified protein/peptide percentage versus run number in pooled male samples.** Each point represents the percentage of newly identified protein/peptides obtained from an additional run. When the run number reached six, the percentage was 4.9%, indicating six 1DLC/MS/MS runs may obtain 95% analytical completeness in one sample.

To estimate the percentage of newly identified proteins by increasing replicate run number, a saturation curve was plotted by computer simulation as described in the Method section. Figure 2 showed that approximately 76% of identified proteins in any run could be confirmed by any second run and 24% of proteins remained unconfirmed. Adding a third run decreased the newly identified protein percentage to 12%. After six runs, this number decreased to 4.9%, and after 25 runs, less than 1% of new proteins could be identified. Although 25 1DLC/MS/MS analyses provided more information than six analyses (208 proteins vs. 144 proteins, respectively), approximately 3-fold more experiments had to be performed, which is not suitable for analyzing larger numbers of samples. Therefore, six 1DLC/MS/MS analyses were empirically chosen as the point for the subsequent individual urinary proteome analyses. The newly identified peptide percentage with the increase in the number of runs was also plotted and showed almost the same trend as that of proteins (Figure 2).

### The analytical completeness of 20 urine samples

To define the minimal sample number for a group urinary proteome analysis, it is necessary to perform a comprehensive analysis of each sample. The term “analytical completeness” was used to describe the completeness of a urinary proteome obtained by LC/MS/MS analysis for one sample. The percentage of analytical completeness represented the percentage of newly identified protein/peptides gained by an additional run.

Overnight urine samples from 10 males and 10 females were analyzed by replicate 1DLC/MS/MS and each sample was subjected to at least nine 1DLC/MS/MS analyses. At a 1% false positive rate, a total of 836 proteins and 2,396 peptides (an average of 213 proteins and 522 peptides in each sample) were identified in these 20 samples, and the average inter-run overlap rate was 74.03% (Table 1).

To validate the analytical completeness of the 20 samples, the newly identified protein/peptide percentage was calculated as a function of run number. The results from the 20 samples (Additional file 5) showed that when the run number exceeded six, the newly identified protein/peptide percentage decreased to less than 5%, consistent with the male pooled sample, and at 12 and 15 1DLC/MS/MS runs, the final newly identified protein/peptide percentage was approximately 2%. These results indicate that by using replicate 1DLC/MS/MS analyses (9–16 runs), more than 95% analytical completeness was achieved for the 20 samples.

### The minimal sample number for a male/female group by qualitative analysis

**Figure 3:**
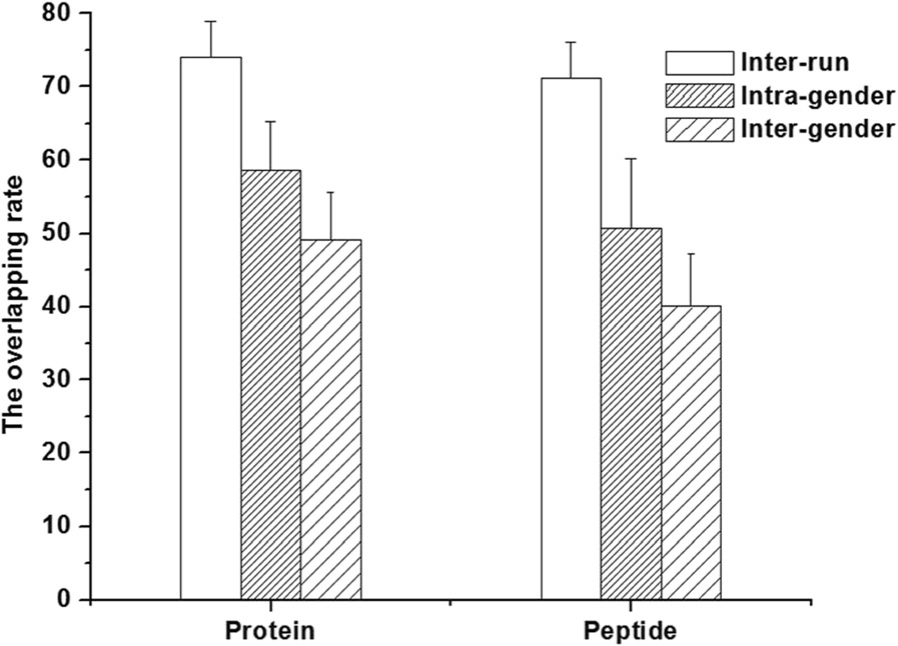
**The comparison of average protein/peptide overlap rate from intra-run, intra-gender, and inter-gender analyses.** The difference in the overlap rates of intra-run, intra-gender, and inter-gender analyses indicated there was intra- and inter-gender individual variation in the urinary proteome.

Before estimating the minimal sample number for a group, the sample number for a sub-group was defined, which in this case was a male/female group. The intra-gender individual variation was defined by protein/peptide overlap rate analysis between the 20 samples. The average intra-gender protein/peptide overlap rates from 10 male and 10 female samples was 58.62% and 50.71%, respectively, which were significantly lower than that of inter-run rates (Figure 3). These results showed that there was intra-gender individual variation in the urinary proteome, which were consistent with previous reports [15,16]. The intra-gender individual variation indicated that for a male/female group, multiple samples should be included in order to obtain a comprehensive urinary proteome analysis.

**Figure 4:**
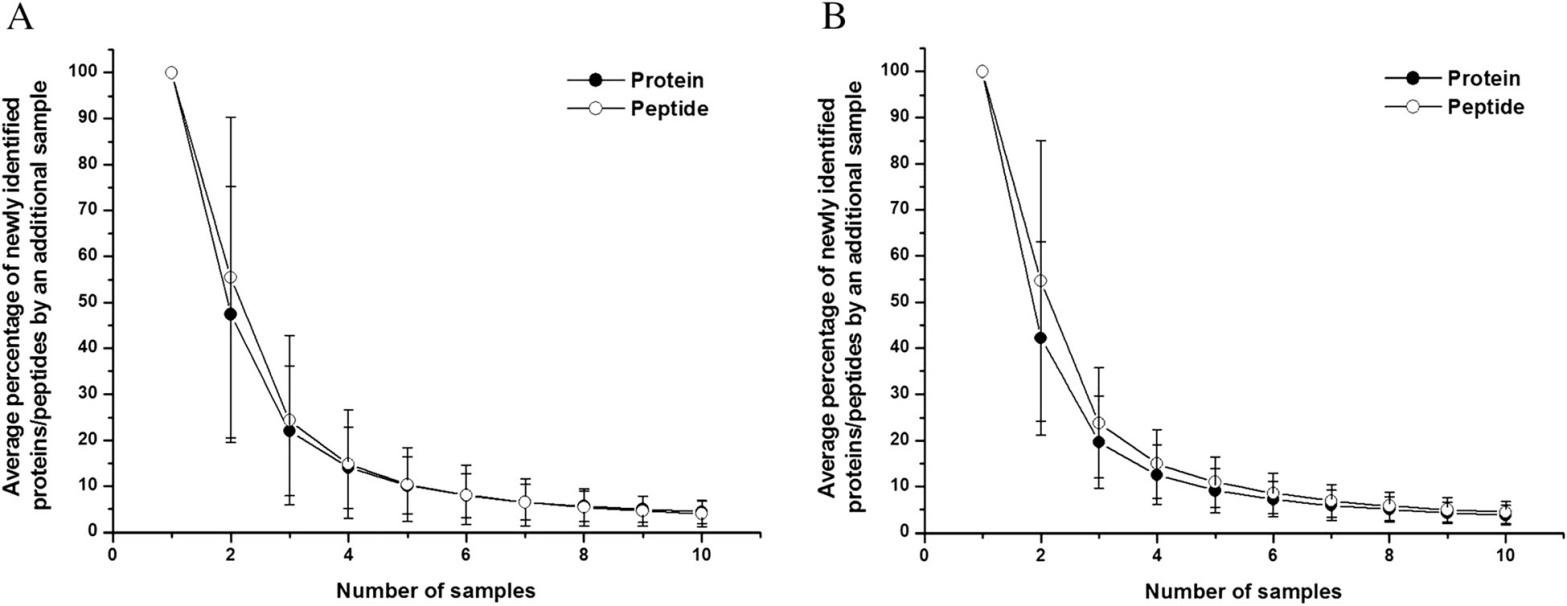
**The newly identified protein/peptide percentage versus sample size in 10 males (A) and 10 females (B).** Each point represents the percentage of newly identified protein/peptides obtained with an additional sample. When the sample number reached nine, the percentage was approximately 5%, indicating that nine samples may yield 95% analytical completeness in a male/female group.

The 10 male or 10 female 1DLC/MS/MS results were then used to define the minimal sample number for a male or female group, respectively. The term “analytical completeness” was also used to describe the completeness of a group urinary proteome by multiple sample statistical analysis. The newly identified protein/peptide percentage was calculated as a function of sample size. The saturation curves for male and female samples were plotted by computer simulation. When the sample number was six, the newly identified protein/peptide percentage was less than 10%, and when the sample number reached nine, the percentage decreased to less than 4%. The results for males and females showed the same trend (Figure 4). These data indicated that 6/9 samples may achieve approximately 90/95% analytical completeness, respectively, of a male/female group urinary proteome.

### The minimal sample number for a group by qualitative analysis

The inter-gender individual variations were also defined by a protein/peptide overlap rate analysis between all of the 20 samples. The overlap rate of inter-gender samples (49.12 and 40.02% for protein and peptide, respectively) was significantly lower than that of intra-gender samples (Figure 3), indicating that there was inter-gender individual variation in the urinary proteome. Moreover, a previous study [27] reported that gender-specific proteins could be found in male samples, suggesting that when the minimal sample number for a group is determined by inter-gender sample analysis, the selected samples should include at least one male/female sample.

**Figure 5:**
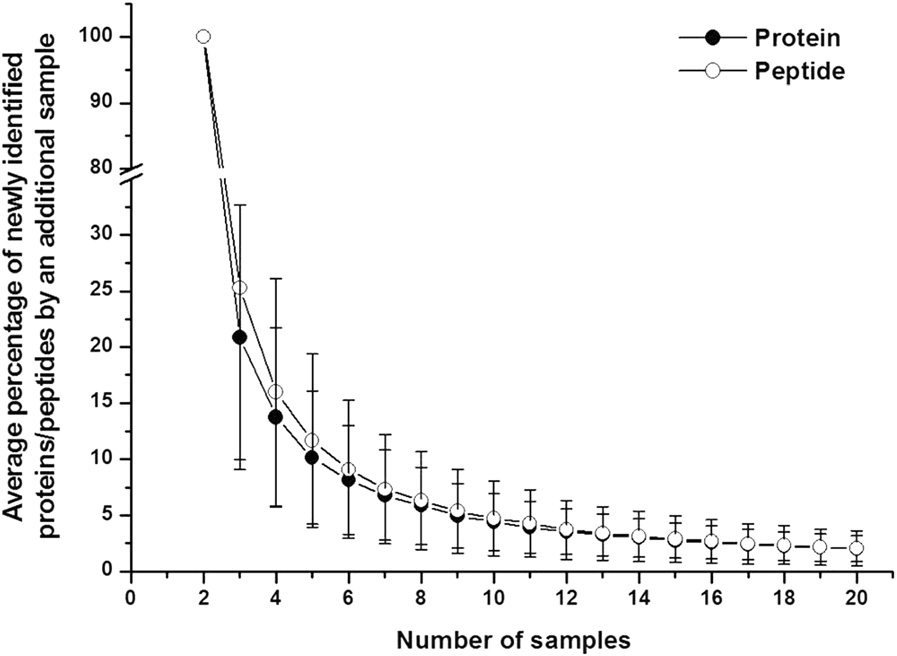
**The newly identified protein/peptide percentage versus sample size for 20 samples.** Each point represents the percentage of newly identified protein/peptides obtained with an additional sample. In each point, the samples included as least one male/female sample. When the sample number reached 10, the percentage was approximately 5%, indicating that 10 samples may yield 95% analytical completeness in a group.

Figure 5 showed that when the sample number was six, the newly identified protein/peptide percentage was less than 10%, and when the sample number reached 10, the percentage decreased to less than 5%. In addition, when the sample number was increased to 20, the newly identified protein/peptide percentage was approximately 2%. The above results showed that 6/10 male/female samples may contain approximately 90%/95% analytical completeness, respectively, of a group urinary proteome.

**Figure 6:**
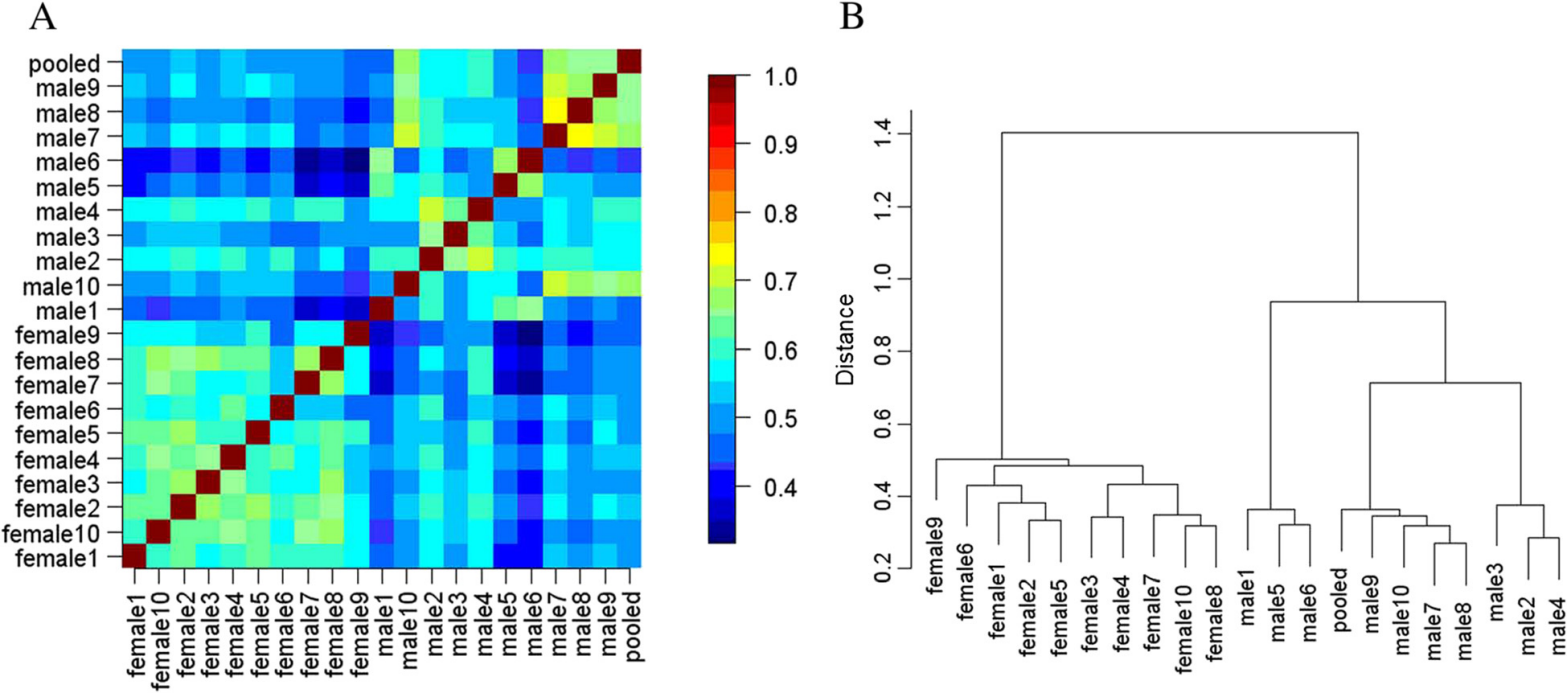
**Pairwise protein overlap rates and hierarchical clustering of 21 samples based on protein pattern.** A: Heatmap of the overlap rate between each sample pair. The higher overlap rates were clearly in the intra-gender region rather than in the inter-gender region. B: Hierarchical clustering of 21 samples also based on the protein overlap rates. The male/female samples clustered together. Both results showed that male and female urinary proteomes showed different patterns.

To define the inter-individual and inter-gender variation more comprehensively, further qualitative analysis was performed. Figure 6A shows the pairwise protein overlap rate of the 21 samples. For visualization, the rates were color-coded. The higher overlap rates were clearly in the intra-gender region, and the pooled male samples had higher overlap rates with male samples. Hierarchical clustering analysis based on the same qualitative data showed that the male/female samples clustered together (Figure 6B). These results indicate that the male and female urinary proteome may have different patterns.

### The minimal sample number for a group by quantitative analysis

Quantitative analysis has been widely used in clinical urinary proteomic studies. In these studies, hypothesis test (usually *t* test) is commonly used to identify whether a protein expressed significantly different between the disease and control group. Two types of error occur in a hypotheses test: the false positive error *α* and the false negative error *β*. *α* is also called the significance level, whereas 1- *β* refers to the power of the statistical test. Controlling both of these errors is crucial to the success of a proteomic study. To achieve this goal, enough number of samples must be included in the study. The minimal sample size in each group can be calculated based on the level of variation between samples, the expected fold change, the significance level and the statistical power [25].

In proteomic studies, typically hundreds or thousands of proteins/peaks are needed for the hypothesis test of their expression level. The chance of the false positive results increases significantly when multiple hypothesis tests are needed to be performed. Therefore false discovery rate (FDR) of the entire quantitative proteomic analysis should be calculated and controlled to an acceptable level [28].

In this report a semi-quantitative method (spectrum count, SC) was used to calculate the coefficient of variation (CV) between samples. Because SC was relatively accurate for evaluating highly abundant proteins, only proteins identified in more than 80% of the 291 LC-MS/MS runs were included, which was a total of 31 proteins. The median CV of these proteins was then used to calculate the minimal sample number. For male, female, and normal groups, the median CV was 66.2%, 58.2%, and 70.6%, respectively. The minimal sample number at a 5%FDR is shown in Table 2. When the fold change was 2, the average minimal sample number was 16, 13, and 18 for the male, female, and normal groups, respectively. However, when the fold change was 1.5, the average minimal sample number increased to 58, 46, and 66 for these groups, respectively.

**Table 2:** **The estimated minimal sample number per group for quantitative analysis based on spectrum count method, where power is the power of statistical test, *α *is the significance level, and *π *refers to the estimated proportion of truly differentially expressed proteins among all of the identified proteins**

**fold change**	**power**	***α***	***π***	**FDR**	**normal group**	**male group**	**female group**
2	0.8	0.001	0.05	2.31%	20	18	15
	0.8	0.005	0.1	5.33%	16	14	12
	0.8	0.01	0.2	4.76%	14	12	10
	0.9	0.001	0.05	2.06%	24	22	17
	0.9	0.005	0.1	4.76%	19	17	14
	0.9	0.01	0.2	4.26%	17	15	12
1.5	0.8	0.001	0.05	2.31%	71	63	49
	0.8	0.005	0.1	5.33%	56	49	39
	0.8	0.01	0.2	4.76%	49	43	34
	0.9	0.001	0.05	2.06%	87	77	60
	0.9	0.005	0.1	4.76%	69	61	48
	0.9	0.01	0.2	4.26%	62	54	43

**Figure 7:**
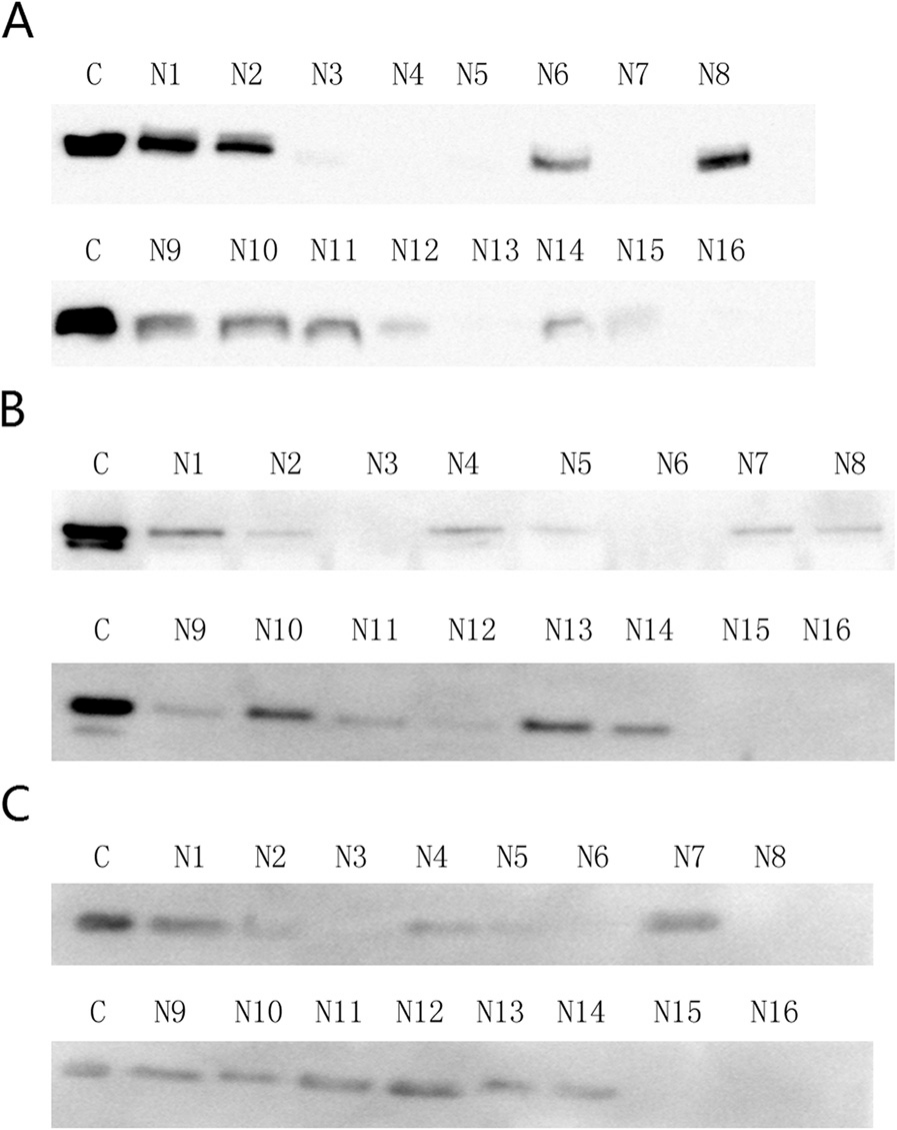
The western blot analysis of alpha 1 antitrypsin (A), ceruloplasmin (B), and beta-2-microglobulin (C) from 16 normal human overnight urine samples (10 females and 6 males, N1-N16) and one stage IV diabetic nephropathy urine sample as control (one male, C).

To estimate the minimal sample number for proteins with lower abundance, western blot was used to measure three proteins (alpha 1 antitrypsin, ceruloplasmin, and beta-2-microglobulin). These three proteins were identified in less than 40% of the LC-MS/MS runs and the CVs of SC were 170.6%, 279.1%, and 222%, respectively. However, based on the western blot analysis (Figure 7), the CVs were 81.2%, 101.1%, and 93%, respectively, indicating that quantitative western blot was more accurate than SC for proteins with low abundance. The average minimal sample number for these three proteins was 30 with a 2-fold change and 110 with a 1.5-fold change, indicating that more samples were necessary to obtain significant results for proteins of low abundance than those of high abundance (Table 3).

**Table 3:** **The estimated minimal sample number per group for five proteins based on western blot, where power is the power of statistical test, *α *is the significance level, and *π *refers to the estimated proportion of truly differentially expressed proteins among all of the identified proteins.**

**fold change**	**power**	***α***	***π***	**FDR**	**alpha 1**	**ceruloplasmin**	**beta-2**
					**antitrypsin**		**microglobulin**
2	0.8	0.001	0.05	2.31%	26	38	33
	0.8	0.005	0.1	5.33%	20	30	26
	0.8	0.01	0.2	4.76%	18	26	22
	0.9	0.001	0.05	2.06%	31	46	39
	0.9	0.005	0.1	4.76%	25	37	31
	0.9	0.01	0.2	4.26%	22	33	28
1.5	0.8	0.001	0.05	2.31%	93	143	121
	0.8	0.005	0.1	5.33%	73	111	95
	0.8	0.01	0.2	4.76%	64	98	83
	0.9	0.001	0.05	2.06%	113	174	148
	0.9	0.005	0.1	4.76%	91	139	118
	0.9	0.01	0.2	4.26%	81	124	105

## Discussion

In recent years, clinical urinary proteomic analyses have been widely used to discover biomarkers. A thorough and representative urinary proteome database of normal human samples is critically important as the background of a disease proteome for discovery proteomics and the source of candidate proteins/peptides for targeted proteomics. Since 2001, a number of groups have addressed this issue, and more than 2500 proteins have been identified from the normal human urinary proteome. However, there are still some important aspects that need to be defined.

To construct a representative urinary proteome, it is necessary to define the minimal sample number. Too few samples may present individual-specific proteins that do not represent the group pattern. Previous studies [15-17] have used various sample numbers ranging from one to over ten. However, because this issue has not been thoroughly assessed, the minimal sample number was unknown. In this study, inter-individual and inter-gender variations were taken into consideration for qualitative analysis to achieve a representative urinary proteome. We used replicate LC/MS/MS analyses of 20 urine samples from healthy volunteers to define the minimal sample number needed. The results showed that 9 male/female samples may contain approximately 95% analytical completeness of a male/female group. For a group, 10 samples can achieve 95% analytical completeness. Importantly, the results of this study may be helpful for constructing a new urinary proteome database or evaluating an existing database. The universal application of these conclusions should be cautioned for several reasons. First, technical variations factors, including sample preparation, LC separation, mass spectrometer detection, and data processing can affect the final identification results. The conclusions of this study are based on the results obtained with 1DLC separation and a low sensitivity and resolution mass spectrometer (LTQ XL). Any change in these factors, such as using an instrument with high sensitivity and high resolution (i.e. Orbitrap or TripleTOF 5600), might result in a different conclusion. For example, in this report a total of 867 proteins were identified with one-dimensional separation (1DLC) and low resolution instrument (LTQ XL). Kentsis *et al.*[29] identified 2362 proteins using three-dimensional separation (centrifugation, SDS-PAGE, and 1DLC) and a high resolution instrument (LTQ Orbitrap XL). Thus, with more separation approaches and a more accurate instrument, a substantially greater number of proteins could be identified and more useful information might be obtained. On the other hand, it is well known that the urinary proteome had great biological variation. In this study, only inter-individual and inter-gender variations were taken into consideration, and other biological variations (such as age, hormone level, exercise, and others [30]) may also have a marked impact on the results and increase the sample number. Therefore, the conclusion presented here represents a preliminary result that may be the minimal sample number needed. If other variation factors are included, the minimal sample number may increase.

Another important issue regarding a normal urinary proteome database is the quantitative information. For clinical research, the aim is generally focused on identifying disease-related biomarkers. The quantitative information of each protein would be helpful to define biological and technical variations so that the differential proteins with statistical significance in a group could be identified. In addition, the false differential proteins found due to the high variation in the group could be excluded. To date, most normal urinary proteome analyses have been qualitative studies, and only the study by Nagaraj *et al.*[15] provided overall quantitative information of each protein using a peak intensity method. In this report, we used SC and western blot to assess quantitative information of high and low abundance proteins, respectively, and to estimate the minimal sample number needed for quantitative analysis. For high abundance proteins, the average minimal sample number was 18 with a 2-fold change, and for the proteins of low abundance, the number was 30 with a 2-fold change. These results indicated that a higher minimal sample number is required to obtain statistical significance when detecting proteins of low abundance. We also attempted to estimate the minimal sample number using the Nagaraj *et al.*[15] data. With 66% inter-individual CV, the minimal average sample number was 16 with a 2-fold change and 58 with a 1.5-fold change, among all the acceptable levels of FDR and statistical power. These results were similar to our results for proteins of high abundance using the SC method. However, the sample number, separation method, MS instrument, data processing software and protein number used for quantitative analysis were different between these two studies. Considering that there were other quantitative methods, such as iTRAQ and TMT, it is difficult to conclude that the minimal sample number for urinary proteome quantitative analysis. Therefore, it is necessary to evaluate the variations of various quantitative methods in the future to define a proper minimal sample number for clinical research.

Our previous work [4] showed that it was hard to define the difference between the male and female urinary proteomes, except for the identification of several male-specific proteins. In addition, recent studies by both LC/MS/MS [5] and 2DE [6] approaches also failed to identify these differences. In this study, the protein overlap rates among the 21 samples and the result of hierarchical clustering analysis allowed us to separate male and female samples into two groups, indicating a difference between the male and female urinary proteome pattern. However, because this study was only based on 1DLC/MS/MS analysis and low-resolution mass spectrometry, the conclusion should be confirmed with additional experiments before being universally applied. In addition, considering the existence of male-specific proteins, it is important that the ratio of male and female samples is balanced when constructing a database.

The choice of pooled or individual sample was also an important issue. Since the proteome is known to have substantial biological variation, an appropriate number of samples should be analyzed for proteomic analysis. However, a few years ago the throughput of proteomic techniques was limited, and in order to circumvent this problem, samples were pooled [31]. Previous reports on cell lines [32] or tissues [33,34] by 2DE showed that pooling could reduce biological variation. On the other hand, Diz *et al.*[34] as well as our previous study [4] showed that pooling samples may lead to a loss of information through sample dilution. In this study, the pooled male sample was found to be clustered with male samples and closest to the samples from Male 7–10, indicating that a pooled sample may not adequately represent the pattern of all individual samples. Therefore, the results from a pooled sample should be carefully assessed before being applied to other experiments. In recent years, with the application of instruments having high sensitivity and high resolution (such as Orbitrap), high-throughput urinary proteome analysis has become possible. Nagaraj *et al.*[5] identified over 800 proteins in a 4h 1DLC/MS/MS analysis using an LTQ Orbitrap XL. Therefore, the use of individual samples is recommended in future work.

## Conclusion

With the wide application of the urinary proteome in clinical research, the construction of a representative and informative normal urinary proteome database has become critically important. Considering the inter-individual and inter-gender variation of the urinary proteome, we used replicate 1DLC/MS/MS to analyze 10 male and 10 female samples for qualitative analysis, and found that in order to achieve a representative urinary database the minimal sample number was estimated to be 10. In addition, the number of male and female samples in a group had better be balanced. For quantitative analysis, proteins of low abundance showed greater variation and required more samples to obtain statistical significance than proteins of high abundance. In addition, different quantitative analyses exhibited different technical variation, and therefore the minimal sample number should be evaluated in conjunction with the quantitative method being used. With the need of quantitative information for each protein in a group and the application of high sensitivity and resolution mass spectrometry, the high-throughput analysis of individual urinary proteomes using different quantitative methods would greatly benefit future clinical urinary proteome studies.

## Competing interests

The authors declare that they have no competing interests.

## Authors’ contributions

Xuejiao Liu participated in sample collection, sample preparations, data collection, and drafting of the manuscript. Chen Shao was involved in data collection, data analysis, and the drafting of the manuscript. Lilong Wei participated in sample preparations and data collection. Jindan Duan and Shuzhen Wu were involved in sample collection and sample preparations. Xuewang Li participated in the design of the experiments. Wei Sun and Mingxi Li participated in the design of the experiments and the drafting of the manuscript. All the authors approved the order of the authorship. All authors read and approved the final manuscript.

## Supplementary Material

Additional file 1 The identification of 291 1DLC/MS/MS analyses from 21 samples. Click here for file

Additional file 2 The protein list from 21 samples. Click here for file

Additional file 3 The peptide and spectrum list from 21 samples. Click here for file

Additional file 4 The spectra of single-peptide proteins in 21 samples. Click here for file

Additional file 5 The figures of the newly identified protein/peptide percentage versus run number in 10 males (1–10) and 10 females (11–20). Click here for file
