# Supplementary material for: An individual urinary proteome analysis in normal human beings to define the minimal sample number to represent the normal urinary proteome
Source: Proteome Sci. 2012 Nov 21;10:70. doi: 10.1186/1477-5956-10-70 (PMC3544588; doi:10.1186/1477-5956-10-70)

**Supporting information file 5**: The newly identified protein/peptide percentage versus run number in 10 males (1-10) and 10 female (11-20). Each point represents the percentage of newly identified protein/peptides obtained by an additional run. When the run number reached 6, the percentage was about 5%, indicating about 95% analytical completeness was achieved for 18 samples.

Figure 1 (Male 1)


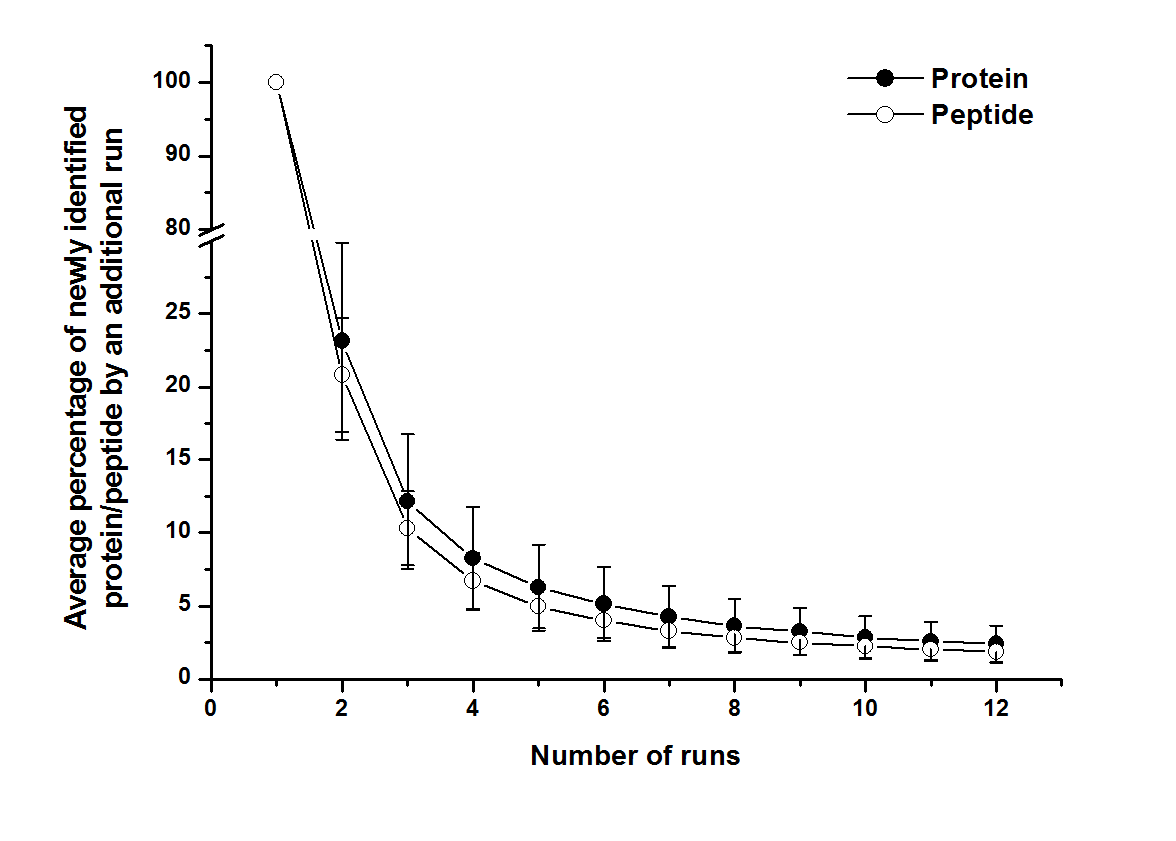


Figure 2 (Male 2)


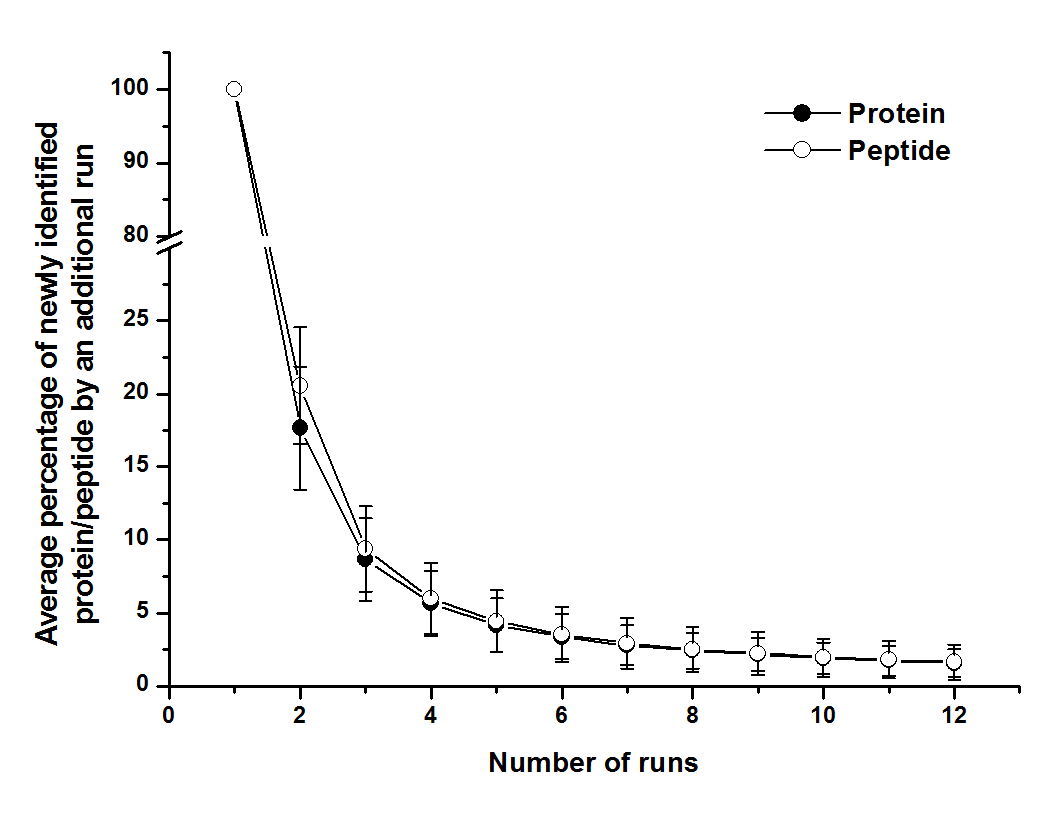


Figure 3 (Male 3)


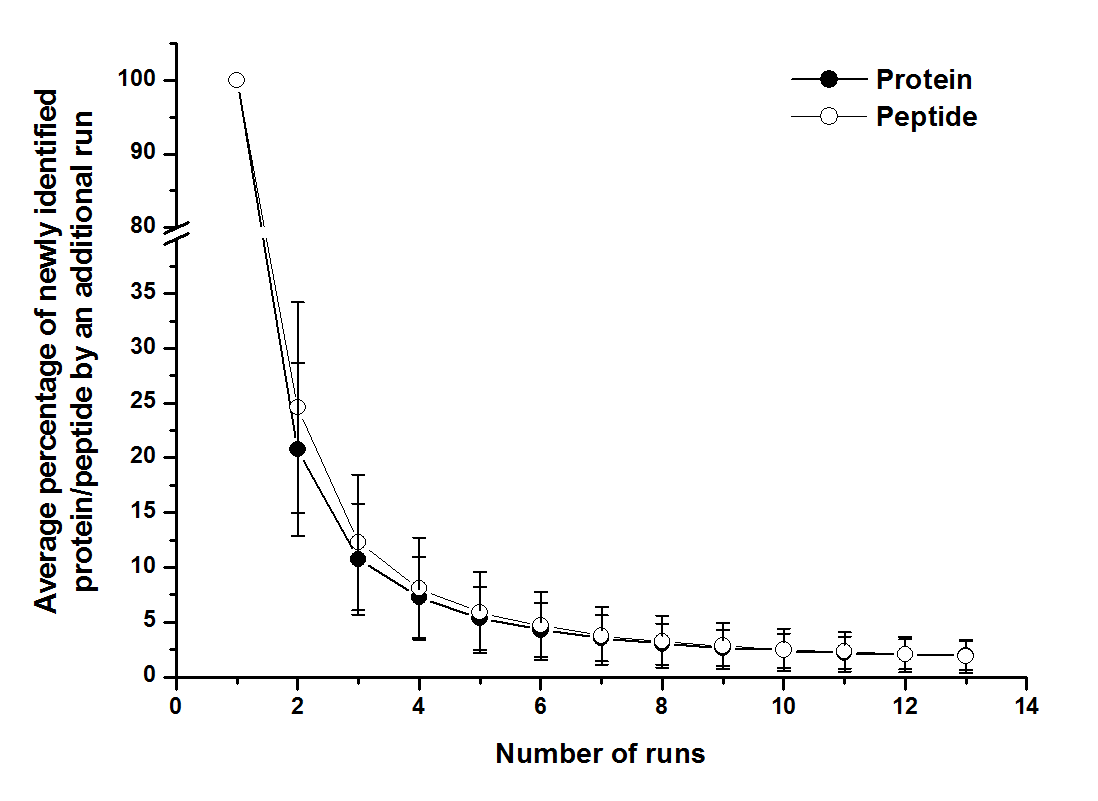


Figure 4 (Male 4)


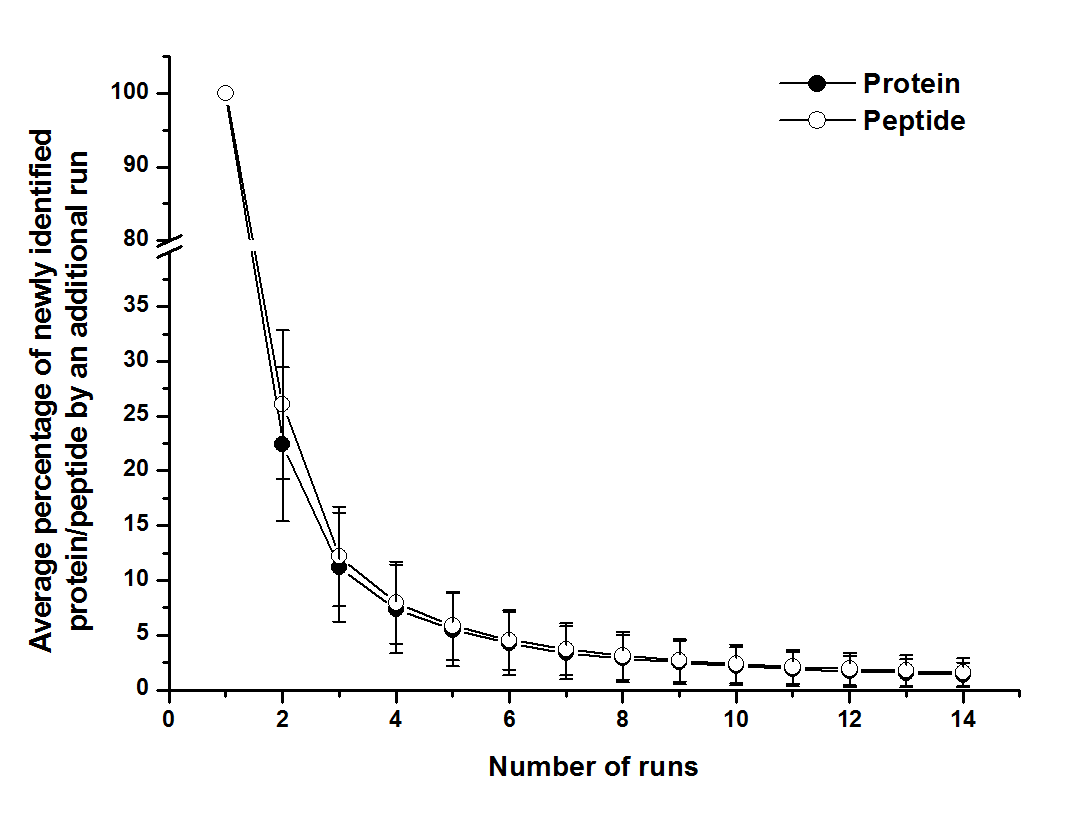


Figure 5 (Male 5)


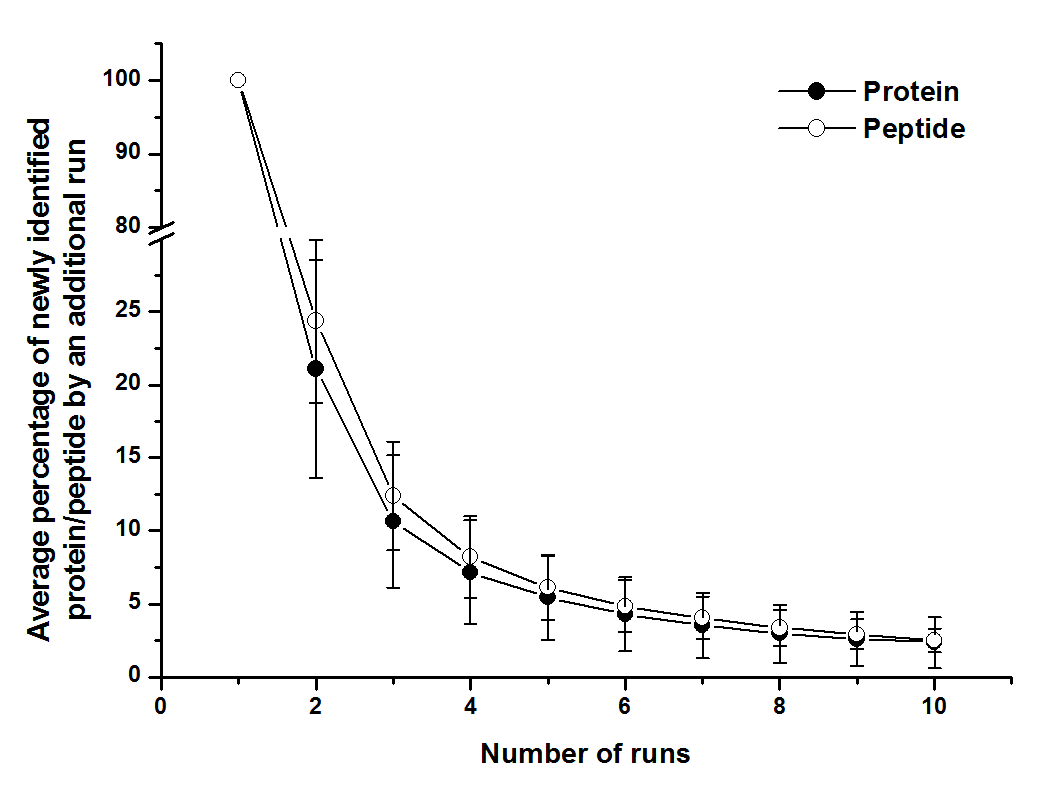


Figure 6 (Male 6)


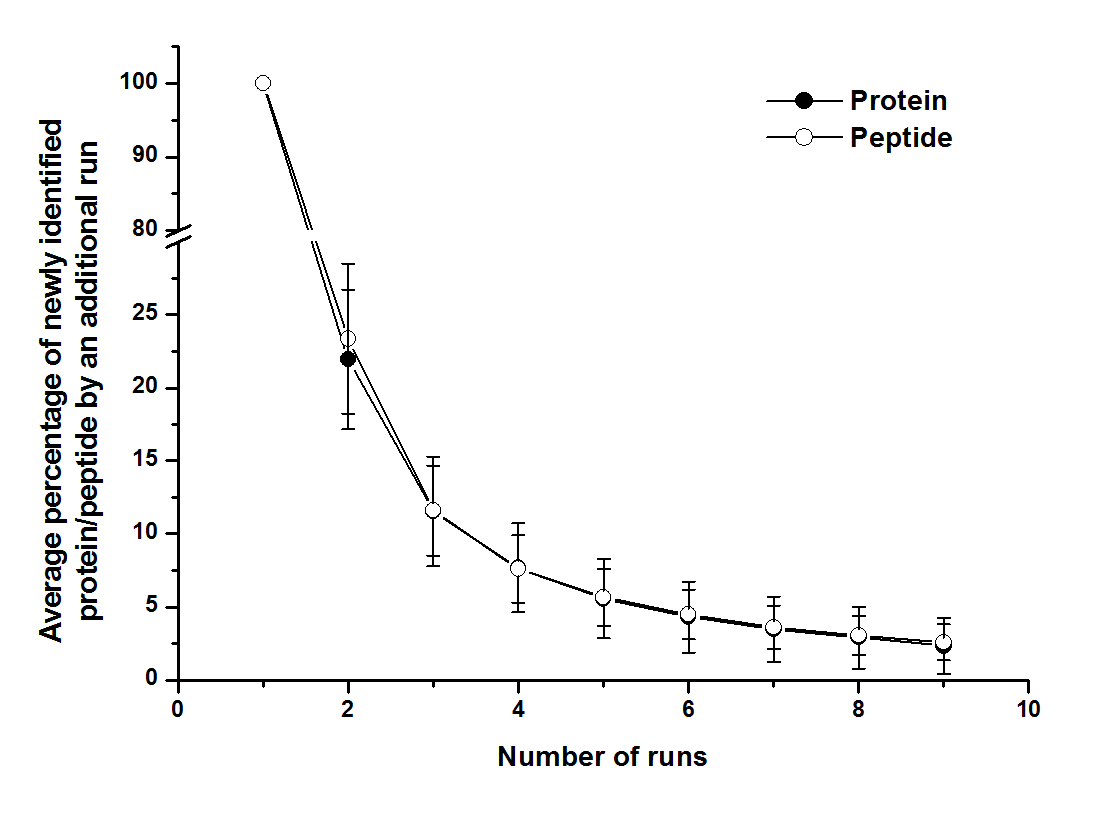


Figure 7 (Male 7)


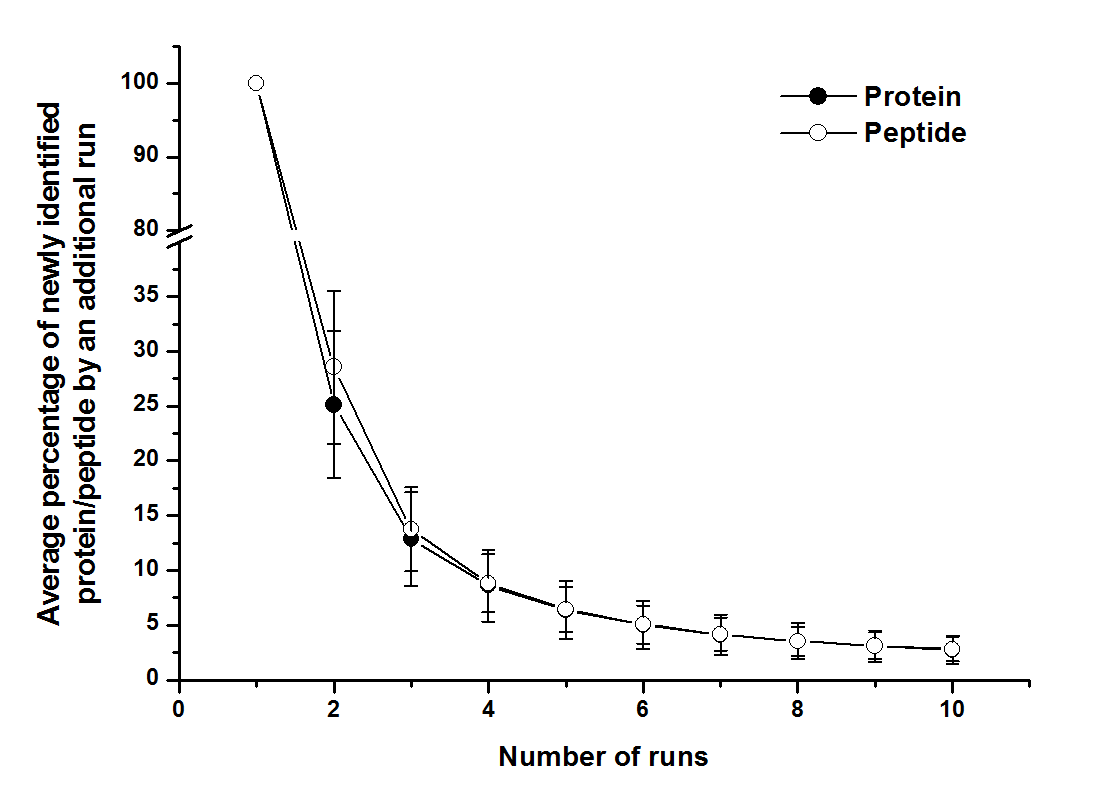


Figure 8 (Male 8)


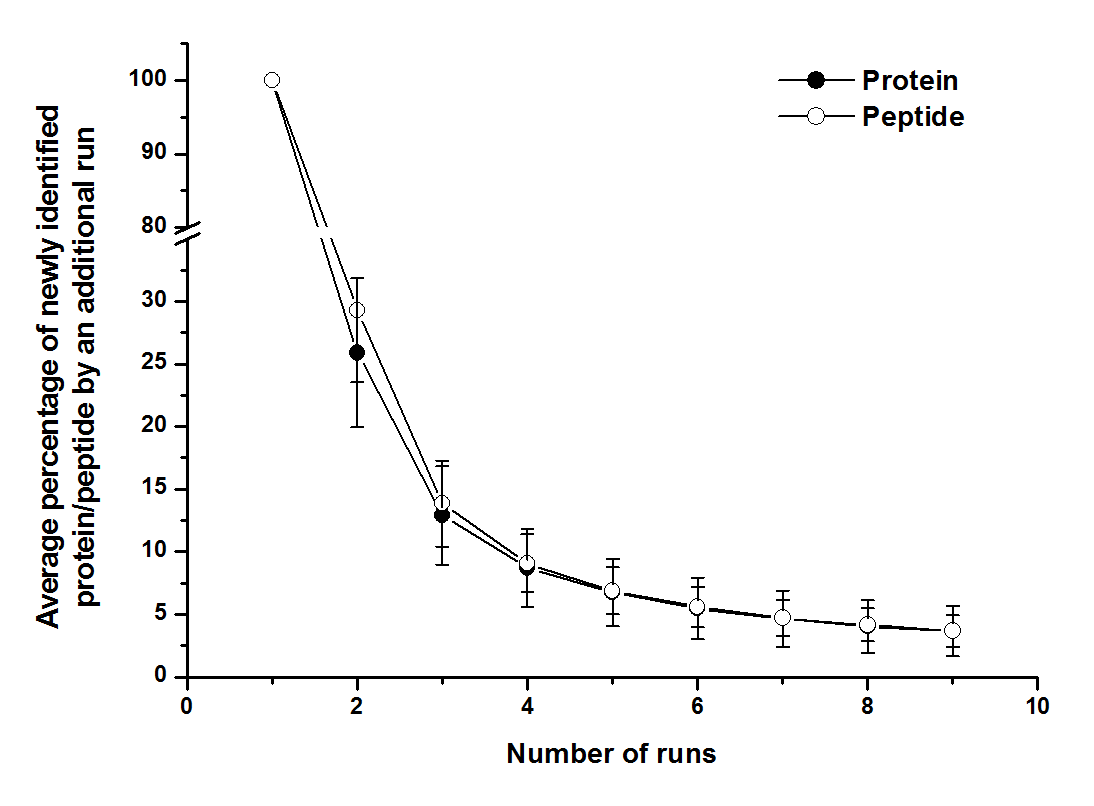


Figure 9 (Male 9)


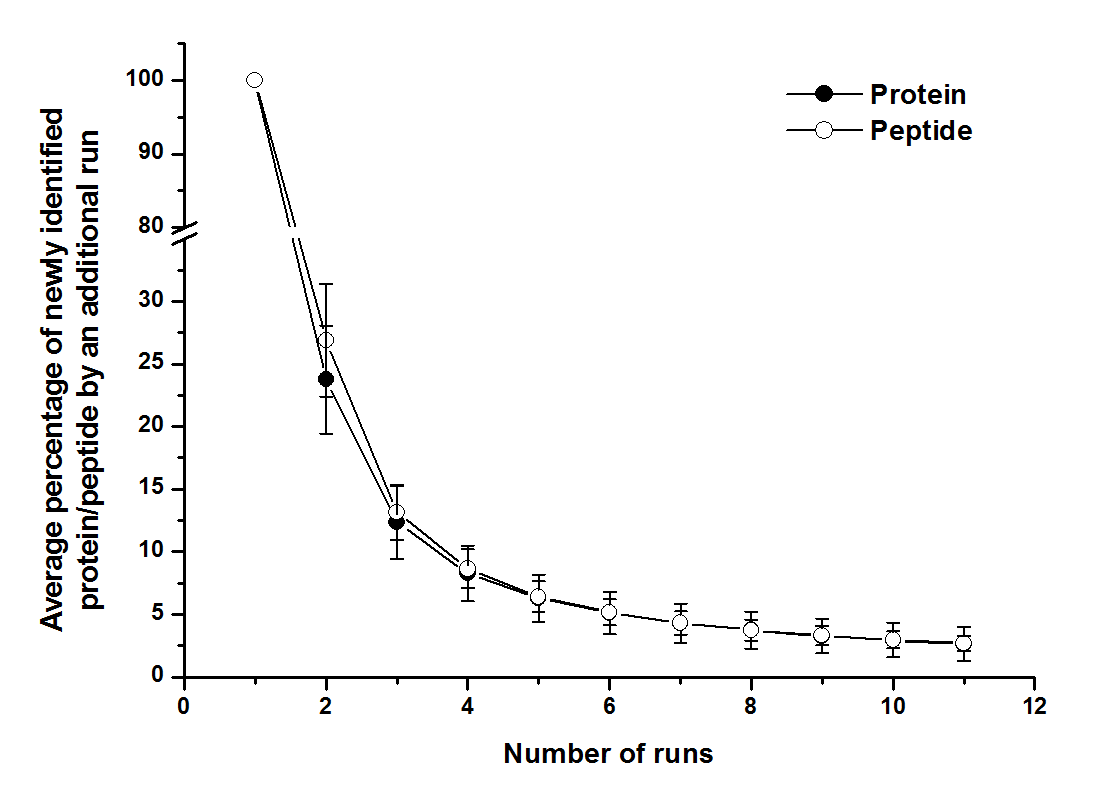


Figure 10 (Male 10)


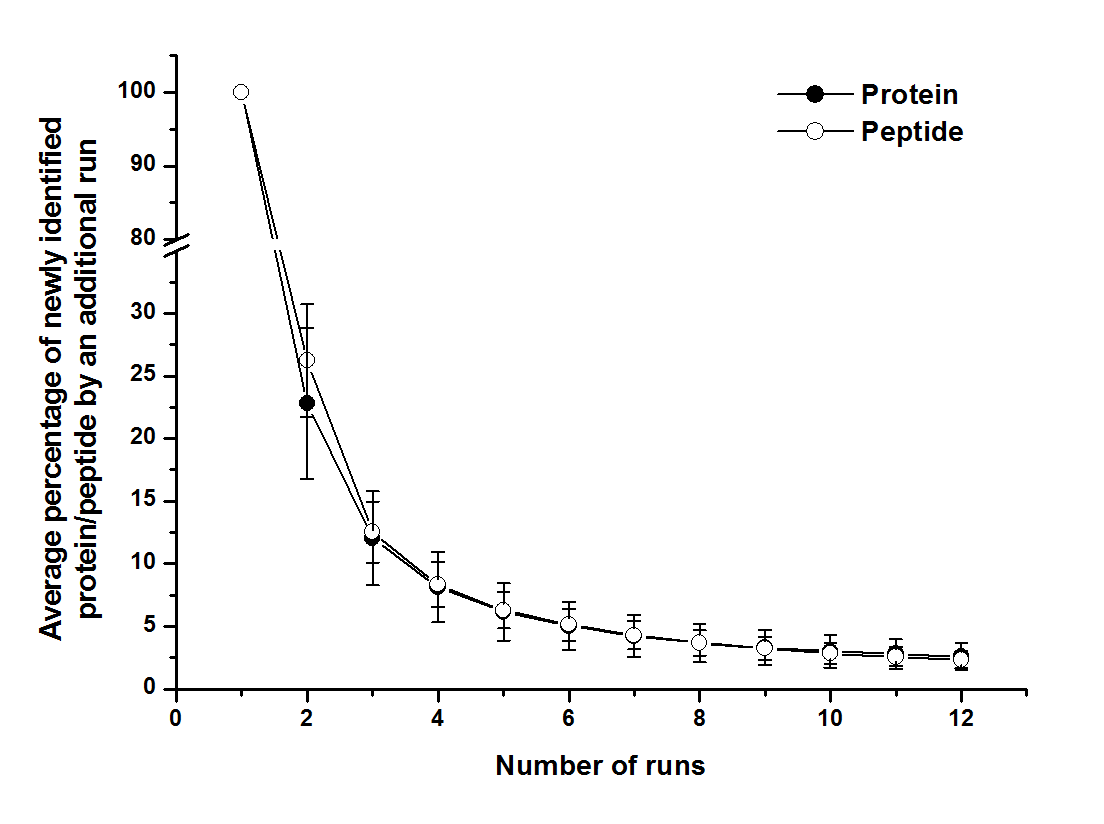


Figure 11 (Female 1)


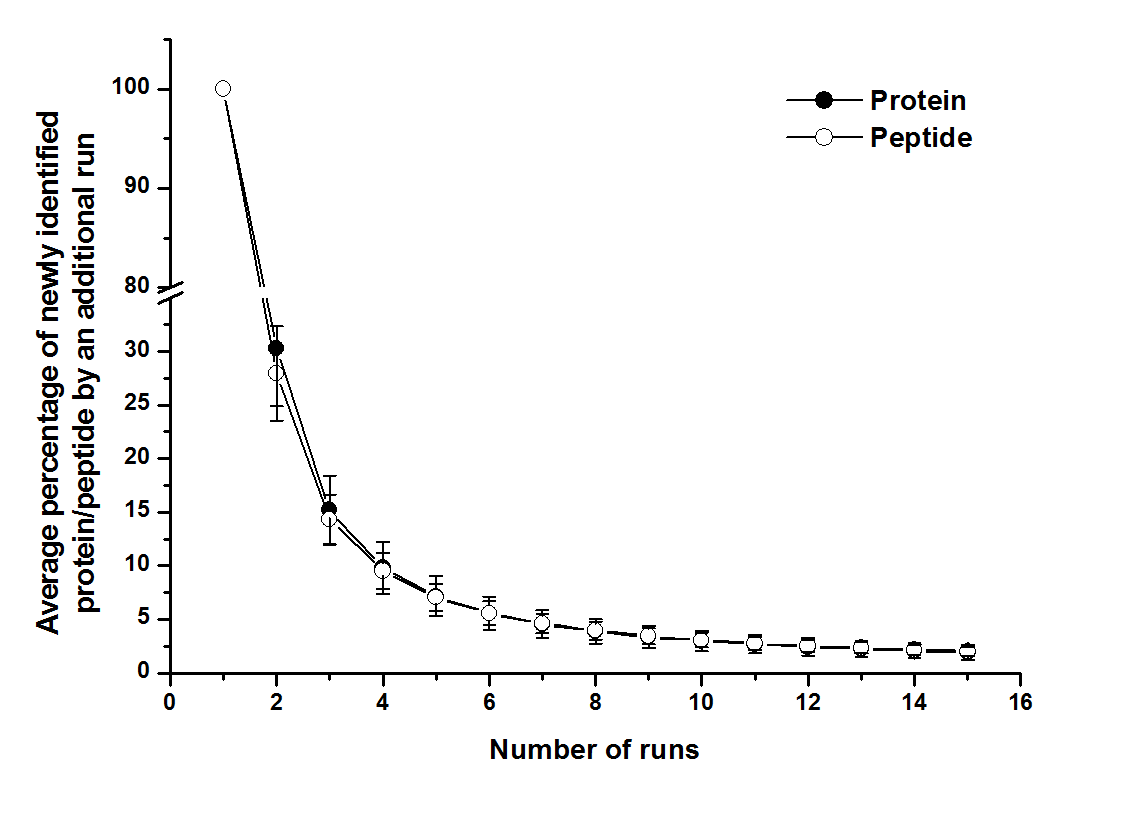


Figure 12 (Female 2)


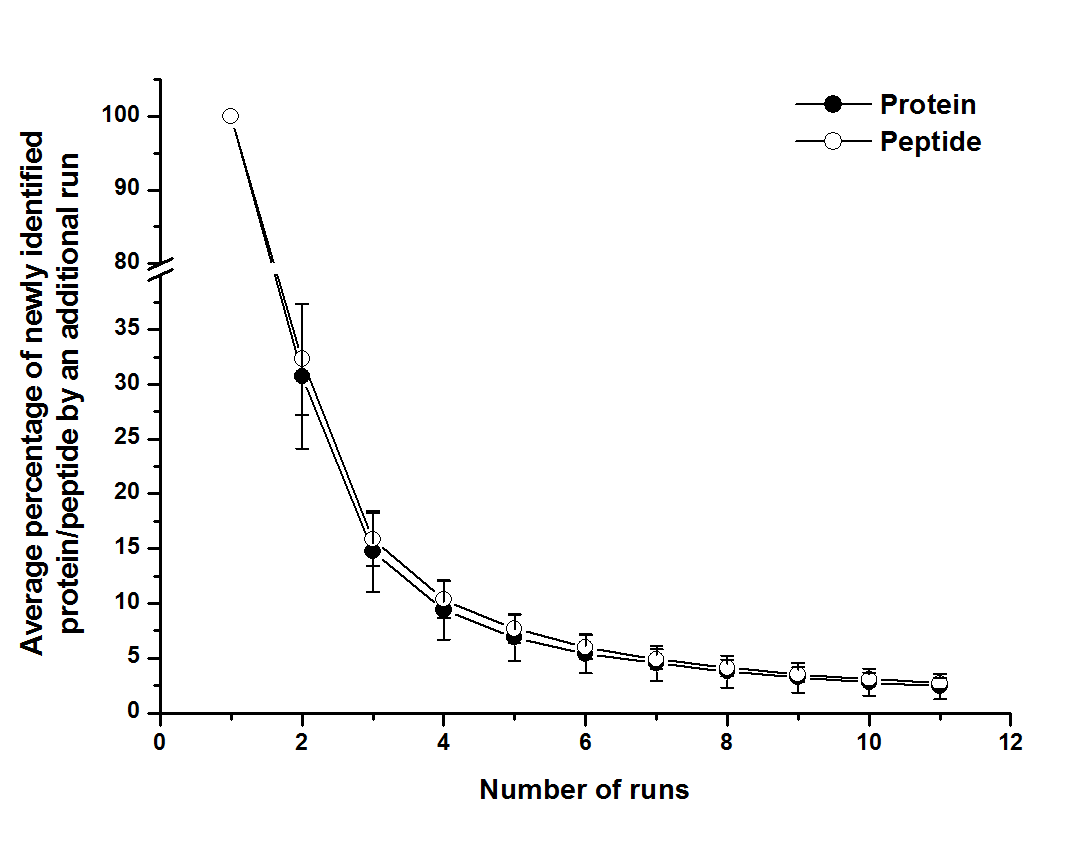


Figure 13 (Female 3)


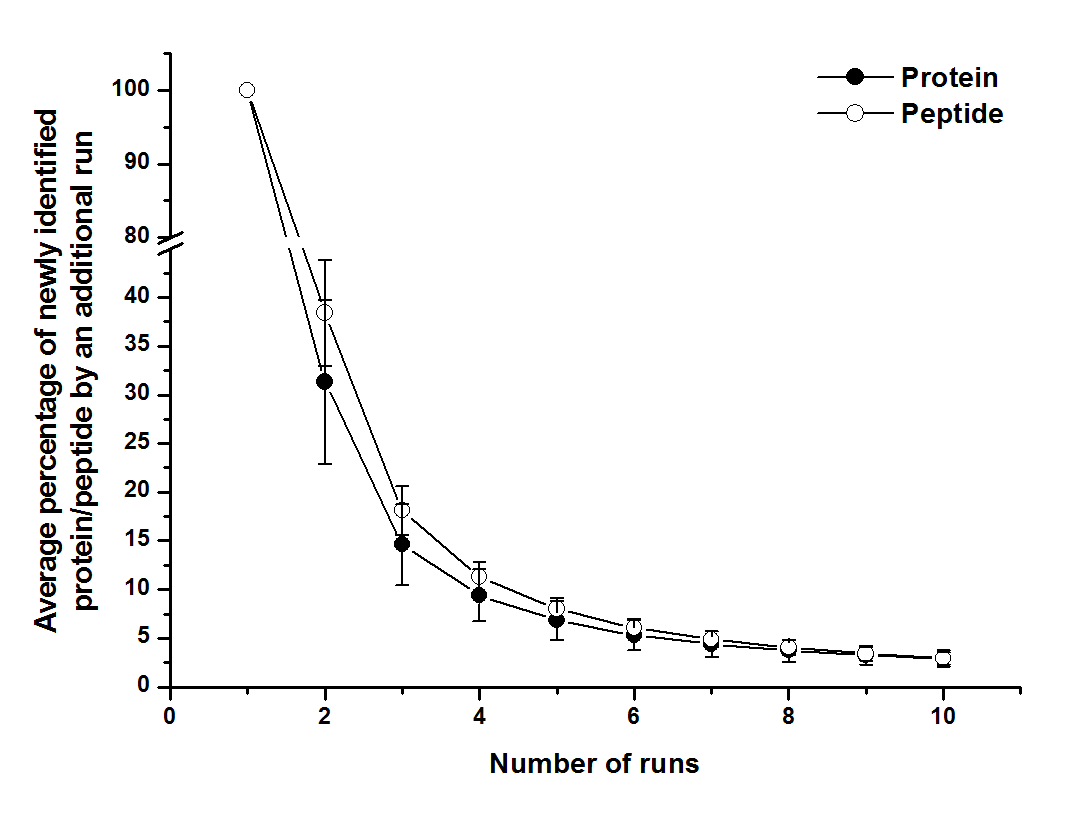


Figure 14 (Female 4)


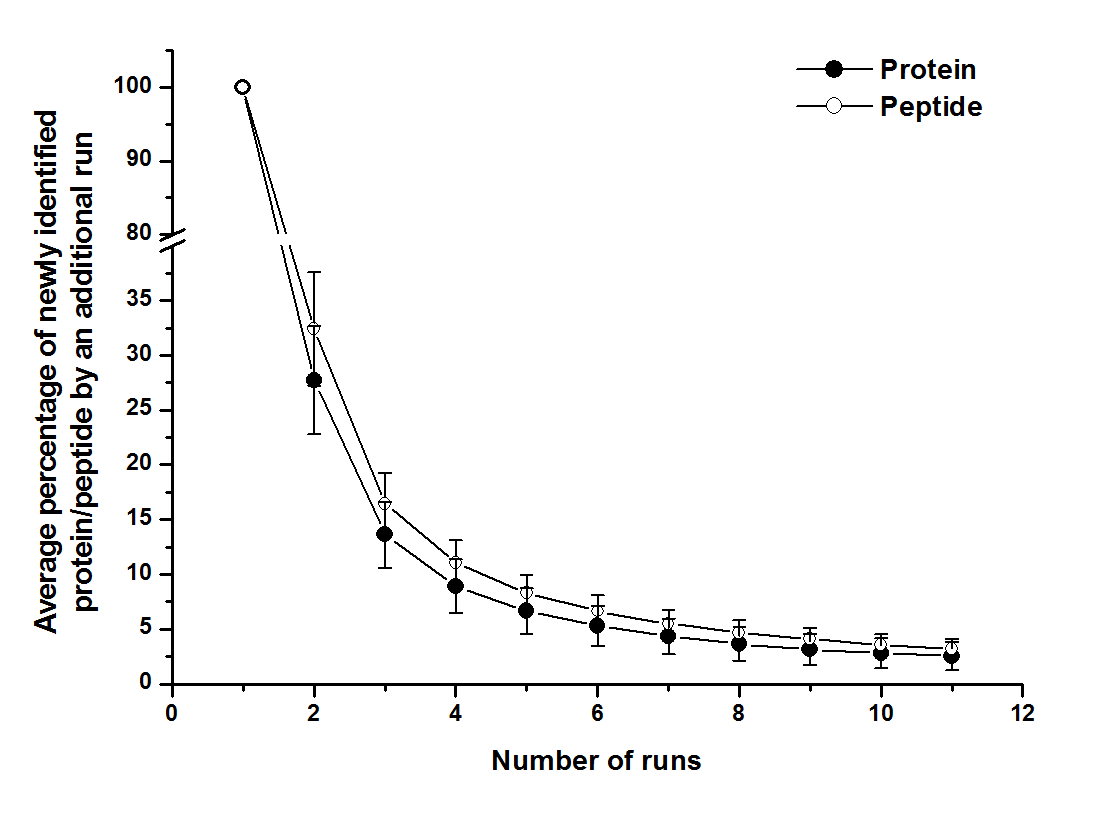


Figure 15 (Female 5)


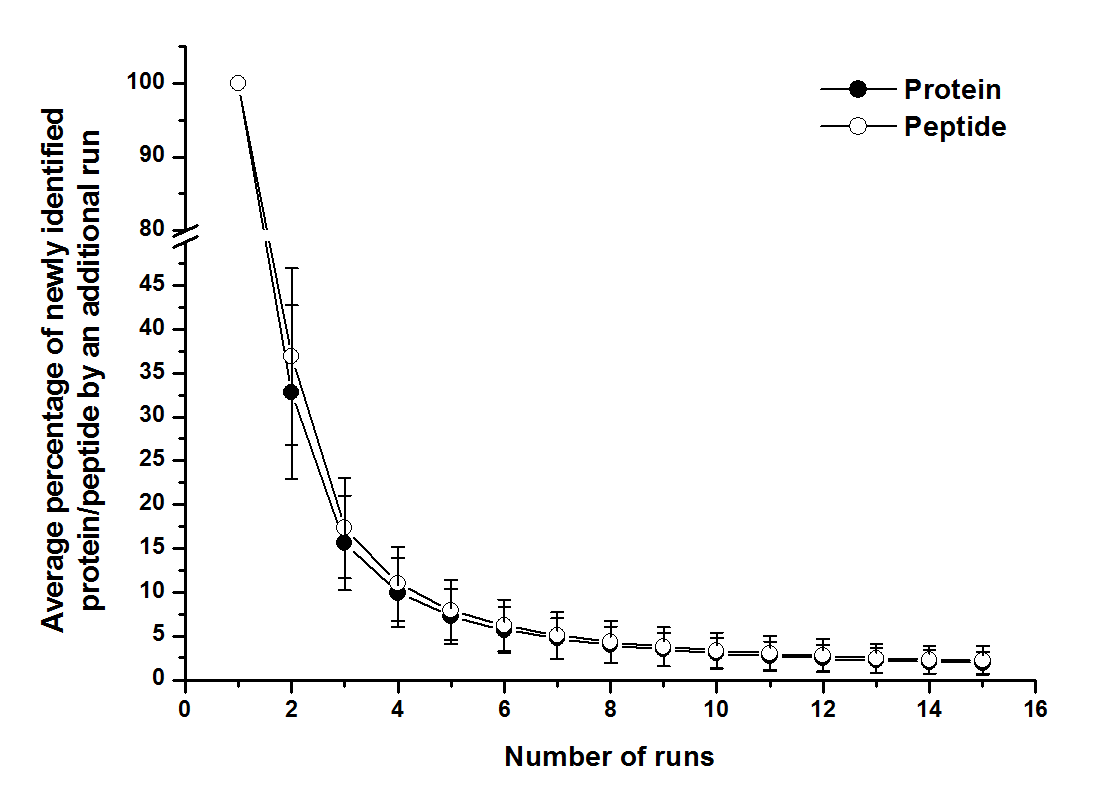


Figure 16 (Female 6)


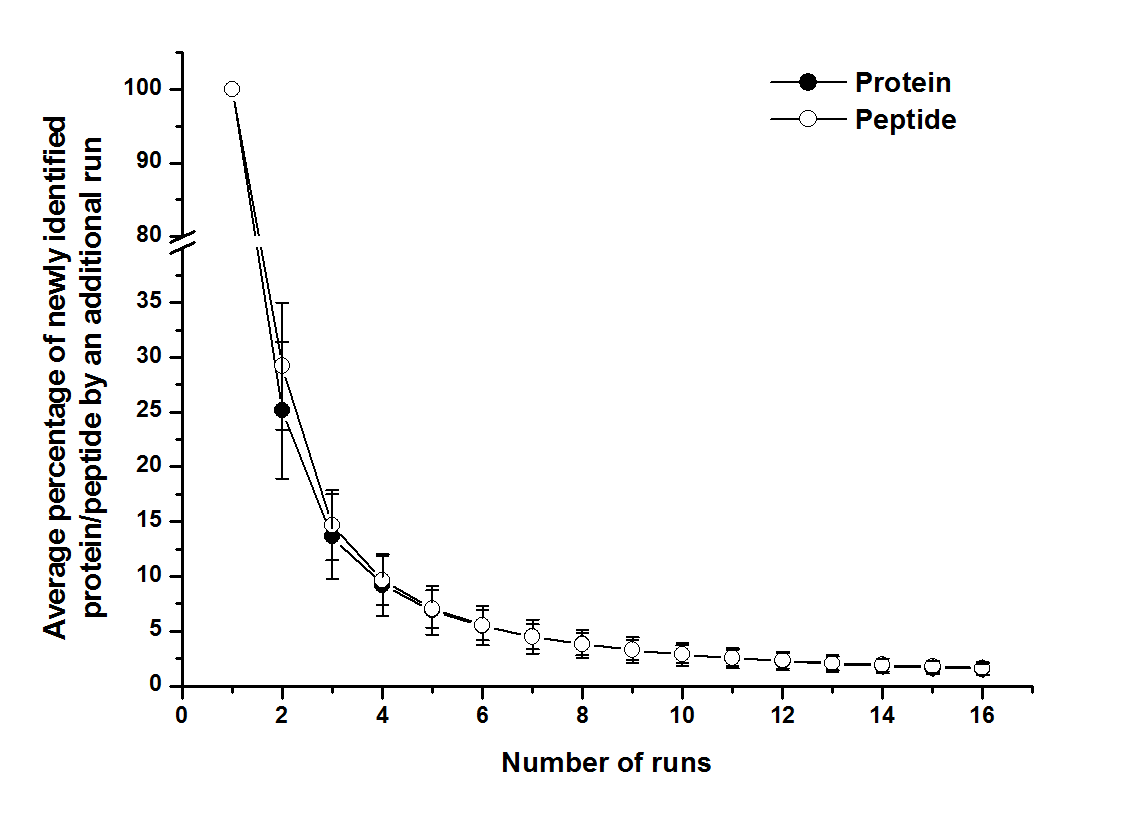


Figure 17 (Female 7)


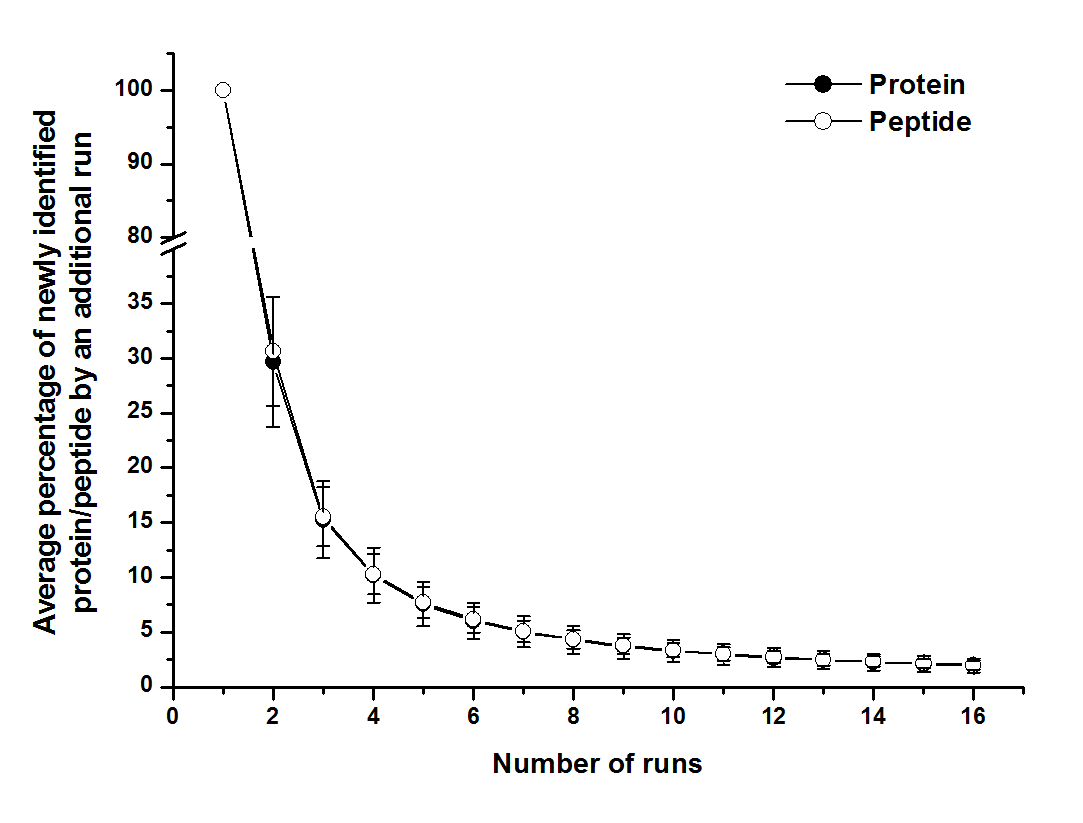


Figure 18 (Female 8)


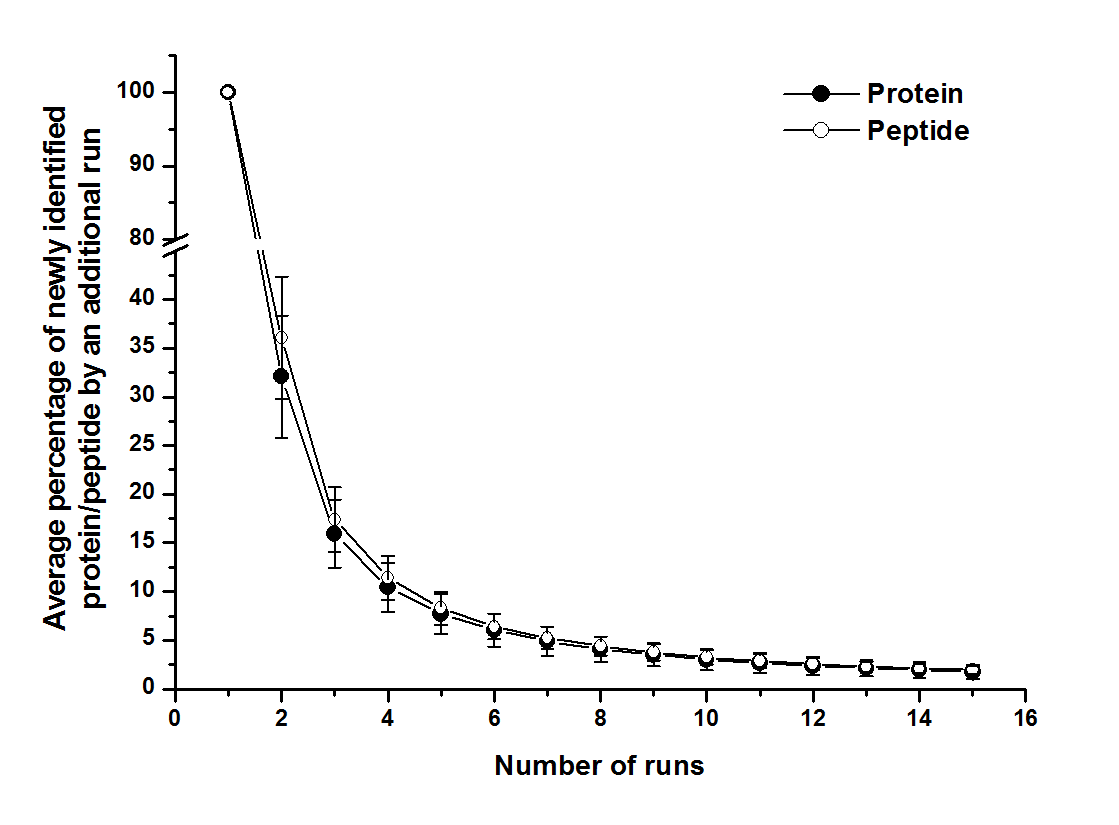


Figure 19 (Female 9)


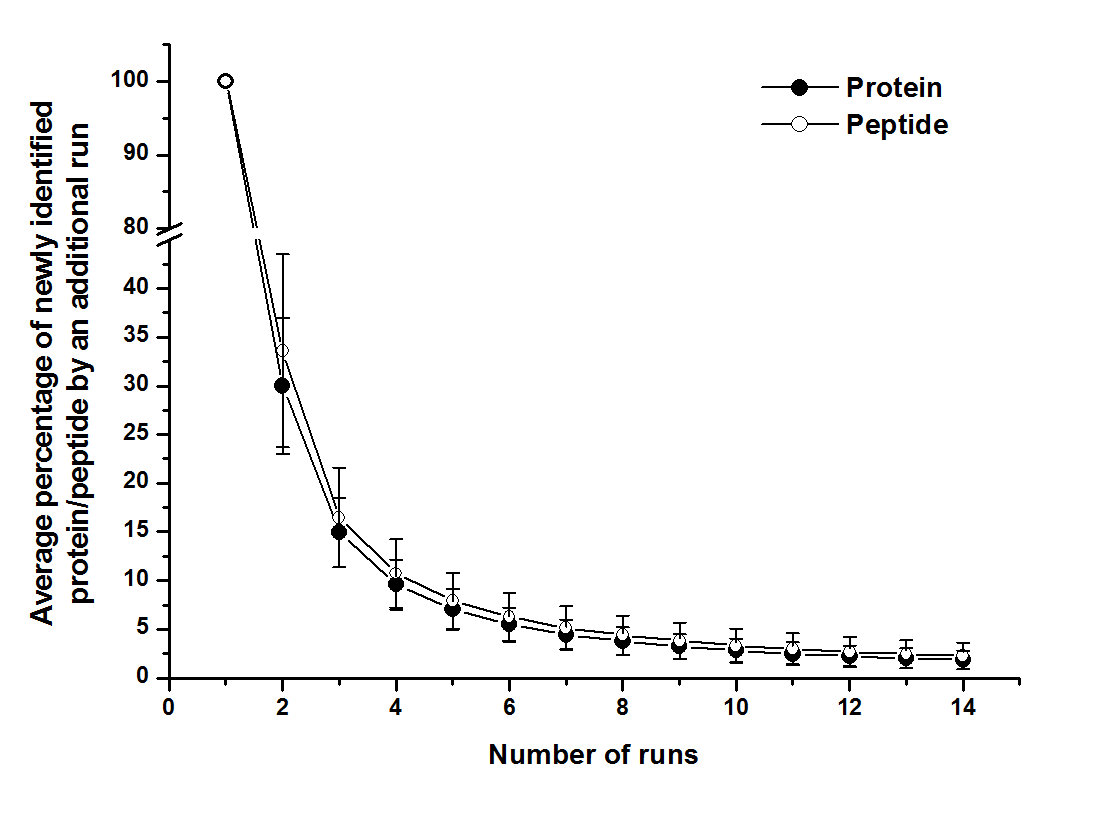


Figure 20 (Female 10)


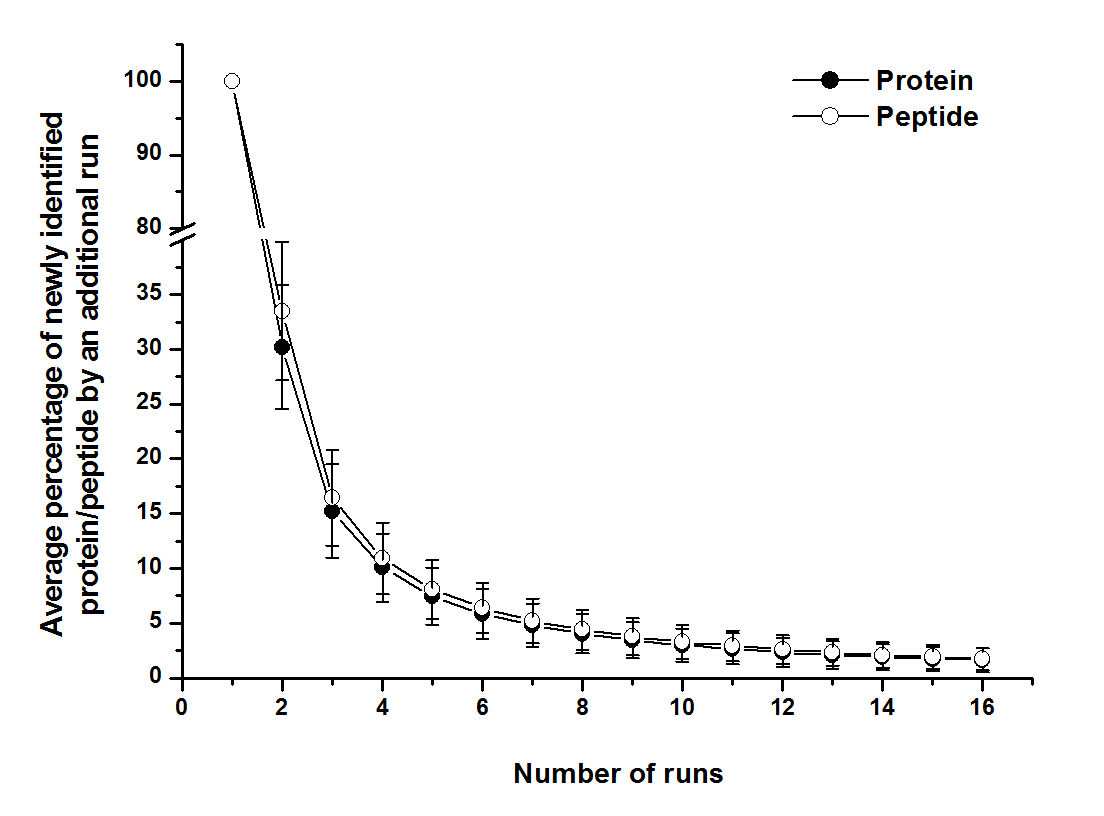

Supplement: Additional file 5 — The figures of the newly identified protein/peptide percentage versus run number in 10 males (1–10) and 10 females (11–20). [file 1477-5956-10-70-S5.doc]
