# Supplementary material for: An individual urinary proteome analysis in normal human beings to define the minimal sample number to represent the normal urinary proteome
Source: Proteome Sci. 2012 Nov 21;10:70. doi: 10.1186/1477-5956-10-70 (PMC3544588; doi:10.1186/1477-5956-10-70)
Supplement: Additional file 4 — The spectra of single-peptide proteins in 21 samples. [file 1477-5956-10-70-S4.doc]

**Supporting information file 4: The spectra of single-peptide proteins in 21 samples.**

**Female 1: page 2-322**

**Female 2: page 323-586**

**Female 3: page 587-888**

**Female 4: page 889-1192**

**Female 5: page 1193-1520**

**Female 6: page 1521-1772**

**Female 7: page 1773-2185**

**Female 8: page 2186-2537**

**Female 9: page 2538-2797**

**Female 10: page 2798-3123**

**Male 1: page 3124-3366**

**Male 2: page 3367-3578**

**Male 3: page 3579-3836**

**Male 4: page 3837-4108**

**Male 5: page 4109-4241**

**Male 6: page 4242-4399**

**Male 7: page 4400-4575**

**Male 8: page 4576-4768**

**Male 9: page 4769-5006**

**Male 10: page 5007-5194**

**Pooled male sample: page 5195-5481**

**Female 1:**

**1. IPI00022284 Major prion protein**

| Sequence | Charge | M+H+ | XCorr | DeltaCn |
| --- | --- | --- | --- | --- |
| R.VVEQMCITQYER.E | 2 | 1556.76 | 4.149 | 0.578 |

+1 charge

```
		      B   	        Y
  1	 V 	   100.08	        -	      12
  2	 V 	   199.14	  1456.79	 11
  3	 E 	   328.19	  1357.72	 10
  4	 Q 	   456.25	  1228.68	  9
  5	 M 	   587.29	  1100.62	  8
  6	 C 	   747.45	   969.58	  7
  7	 I 	   860.54	   809.42	  6
  8	 T 	   961.58	   696.33	  5
  9	 Q 	  1089.64	   595.28	  4
 10	 Y 	  1252.70	   467.22	  3
 11	 E 	  1381.75	   304.16	  2
 12	 R 	        -	        175.12	  1  _
```

+2 charge

```
		      B   	     Y
  1	 V 	    50.54	        -	      12
  2	 V 	   100.08	   728.90	 11
  3	 E 	   164.60	   679.36	 10
  4	 Q 	   228.63	   614.84	  9
  5	 M 	   294.15	   550.81	  8
  6	 C 	   374.23	   485.29	  7
  7	 I 	   430.77	   405.21	  6
  8	 T 	   481.30	   348.67	  5
  9	 Q 	   545.32	   298.15	  4
 10	 Y 	   626.86	   234.12	  3
 11	 E 	   691.38	   152.58	  2
 12	 R 	        -	         88.06	  1  _
```

**2. IPI00176125** Isoform 1 of Collagen alpha-5(VI) chain

| Sequence | Charge | M+H+ | XCorr | DeltaCn |
| --- | --- | --- | --- | --- |
| K.GDIGNPGIPGGPGPKGFR.G | 2 | 1693.888 | 2.805 | 0.339 |

+1 charge

```
		      B   	     Y
  1	 G 	    58.03	        -	     18
  2	 D 	   173.06	  1635.86	 17
  3	 I 	   286.14	  1520.83	 16
  4	 G 	   343.16	  1407.75	 15
  5	 N 	   457.20	  1350.73	 14
  6	 P 	   554.26	  1236.68	 13
  7	 G 	   611.28	  1139.63	 12
  8	 I 	   724.36	  1082.61	 11
  9	 P 	   821.42	   969.53	 10
 10	 G 	   878.44	   872.47	  9
 11	 G 	   935.46	   815.45	  8
 12	 P 	  1032.51	   758.43	  7
 13	 G 	  1089.53	   661.38	  6
 14	 P 	  1186.59	   604.36	  5
 15	 K 	  1314.68	   507.30	  4
 16	 G 	  1371.70	   379.21	  3
 17	 F 	  1518.77	   322.19	  2
 18	 R 	        -	        175.12	  1  _
```

+2 charge

```
		       B   	      Y
  1	 G 	    29.52	        -	      18
  2	 D 	    87.03	   818.43	 17
  3	 I 	   143.57	   760.92	 16
  4	 G 	   172.08	   704.38	 15
  5	 N 	   229.11	   675.87	 14
  6	 P 	   277.63	   618.85	 13
  7	 G 	   306.14	   570.32	 12
  8	 I 	   362.68	   541.81	 11
  9	 P 	   411.21	   485.27	 10
 10	 G 	   439.72	   436.74	  9
 11	 G 	   468.23	   408.23	  8
 12	 P 	   516.76	   379.72	  7
 13	 G 	   545.27	   331.19	  6
 14	 P 	   593.80	   302.68	  5
 15	 K 	   657.84	   254.16	  4
 16	 G 	   686.35	   190.11	  3
 17	 F 	   759.89	   161.60	  2
 18	 R 	        -	         88.06	  1  _
```

**3. IPI00329688 Protein YIPF3**

| Sequence | Charge | M+H+ | XCorr | DeltaCn |
| --- | --- | --- | --- | --- |
| R.DIPAMLPAAR.L | 1 | 1055.276 | 2.252 | 0.45 |

```
+1 charge

		      B   	       Y
  1	 D 	   116.03	        -  	 10
  2	 I 	   229.12	   939.54	  9
  3	 P 	   326.17	   826.46	  8
  4	 A 	   397.21	   729.41	  7
  5	 M 	   528.25	   658.37	  6
  6	 L 	   641.33	   527.33	  5
  7	 P 	   738.39	   414.25	  4
  8	 A 	   809.42	   317.19	  3
  9	 A 	   880.46	   246.16	  2
 10	 R 	        -  	   175.12	  1  _
```

**4. IPI00374563 Agrin**

| Sequence | Charge | M+H+ | XCorr | DeltaCn |
| --- | --- | --- | --- | --- |
| K.AYGTGFVGCLR.D | 2 | 1201.35 | 3.407 | 0.382 |

+1 charge

```
		       B   	      Y
  1	 A 	    72.04	        -	      11
  2	 Y 	   235.11	  1129.68	 10
  3	 G 	   292.13	   966.62	  9
  4	 T 	   393.18	   909.60	  8
  5	 G 	   450.20	   808.55	  7
  6	 F 	   597.27	   751.53	  6
  7	 V 	   696.34	   604.46	  5
  8	 G 	   753.36	   505.39	  4
  9	 C 	   913.52	   448.37	  3
 10	 L 	  1026.61	   288.20	  2
 11	 R 	        -	        175.12	  1  _
```

+2 charge

```
		      B   	     Y
  1	 A 	    36.53	        -	      11
  2	 Y 	   118.06	   565.34	 10
  3	 G 	   146.57	   483.81	  9
  4	 T 	   197.09	   455.30	  8
  5	 G 	   225.60	   404.78	  7
  6	 F 	   299.14	   376.27	  6
  7	 V 	   348.67	   302.73	  5
  8	 G 	   377.18	   253.20	  4
  9	 C 	   457.26	   224.69	  3
  10	 L 	   513.81	   144.61	  2
  11	 R 	        -	         88.06	  1
```

_

**5. IPI00414467 Isoform 1 of Collectin-12**

| Sequence | Charge | M+H+ | XCorr | DeltaCn |
| --- | --- | --- | --- | --- |
| K.WLDGTSPDYKNWK.A | 2 | 1610.751 | 3.002 | 0.529 |

```
+1 charge
		      B   	       Y
  1	 W 	   187.09	        -  	 13
  2	 L 	   300.17	  1423.69	 12
  3	 D 	   415.20	  1310.60	 11
  4	 G 	   472.22	  1195.57	 10
  5	 T 	   573.27	  1138.55	  9
  6	 S 	   660.30	  1037.51	  8
  7	 P 	   757.35	   950.47	  7
  8	 D 	   872.38	   853.42	  6
  9	 Y 	  1035.44	   738.39	  5
 10	 K 	  1163.54	   575.33	  4
 11	 N 	  1277.58	   447.24	  3
 12	 W 	  1463.66	   333.19	  2
 13	 K 	        -  	   147.11	  1  _
+2 charge
		      B   	       Y
  1	 W 	    94.05	        -  	 13
  2	 L 	   150.59	   712.35	 12
  3	 D 	   208.10	   655.80	 11
  4	 G 	   236.61	   598.29	 10
  5	 T 	   287.14	   569.78	  9
  6	 S 	   330.65	   519.26	  8
  7	 P 	   379.18	   475.74	  7
  8	 D 	   436.69	   427.21	  6
  9	 Y 	   518.22	   369.70	  5
 10	 K 	   582.27	   288.17	  4
 11	 N 	   639.29	   224.12	  3
 12	 W 	   732.33	   167.10	  2
 13	 K 	        -  	    74.06	  1  _
```

**6. IPI00043740 Isoform 1 of Sodium channel and clathrin linker 1**

| Sequence | Charge | M+H+ | XCorr | DeltaCn |
| --- | --- | --- | --- | --- |
| K.LHTQELLSQLEMANEK.V | 2 | 1885.132 | 3.557 | 0.228 |

```
+1 charge
		      B   	       Y
  1	 L 	   114.09	        -  	 16
  2	 H 	   251.15	  1770.87	 15
  3	 T 	   352.20	  1633.81	 14
  4	 Q 	   480.26	  1532.76	 13
  5	 E 	   609.30	  1404.70	 12
  6	 L 	   722.38	  1275.66	 11
  7	 L 	   835.47	  1162.58	 10
  8	 S 	   922.50	  1049.49	  9
  9	 Q 	  1050.56	   962.46	  8
 10	 L 	  1163.64	   834.40	  7
 11	 E 	  1292.68	   721.32	  6
 12	 M 	  1423.72	   592.28	  5
 13	 A 	  1494.76	   461.24	  4
 14	 N 	  1608.81	   390.20	  3
 15	 E 	  1737.85	   276.16	  2
 16	 K 	        -  	   147.11	  1  _
+2 charge
		      B   	       Y
  1	 L 	    57.55	        -  	 16
  2	 H 	   126.08	   885.94	 15
  3	 T 	   176.60	   817.41	 14
  4	 Q 	   240.63	   766.88	 13
  5	 E 	   305.15	   702.86	 12
  6	 L 	   361.70	   638.33	 11
  7	 L 	   418.24	   581.79	 10
  8	 S 	   461.75	   525.25	  9
  9	 Q 	   525.78	   481.73	  8
 10	 L 	   582.32	   417.70	  7
 11	 E 	   646.85	   361.16	  6
 12	 M 	   712.37	   296.64	  5
 13	 A 	   747.88	   231.12	  4
 14	 N 	   804.91	   195.60	  3
 15	 E 	   869.43	   138.58	  2
 16	 K 	        -  	    74.06	  1  _
```

**7. IPI00647357 Isoform 1 of Osteoclast-associated immunoglobulin-like receptor**

| Sequence | Charge | M+H+ | XCorr | DeltaCn |
| --- | --- | --- | --- | --- |
| R.FGLFKPGEIAPLLFR.D | 2 | 1706.066 | 3.489 | 0.374 |

+1 charge

```
		      B   	     Y
  1	 F 	   148.08	        -	      15
  2	 G 	   205.10	  1557.92	 14
  3	 L 	   318.18	  1500.89	 13
  4	 F 	   465.25	  1387.81	 12
  5	 K 	   593.34	  1240.74	 11
  6	 P 	   690.40	  1112.65	 10
  7	 G 	   747.42	  1015.59	  9
  8	 E 	   876.46	   958.57	  8
  9	 I 	   989.55	   829.53	  7
 10	 A 	  1060.58	   716.45	  6
 11	 P 	  1157.64	   645.41	  5
 12	 L 	  1270.72	   548.36	  4
 13	 L 	  1383.80	   435.27	  3
 14	 F 	  1530.87	   322.19	  2
 15	 R 	        -	        175.12	  1  _
```

+2 charge

```
		      B   	     Y
  1	 F 	    74.54	        -	      15
  2	 G 	   103.05	   779.46	 14
  3	 L 	   159.59	   750.95	 13
  4	 F 	   233.13	   694.41	 12
  5	 K 	   297.18	   620.87	 11
  6	 P 	   345.70	   556.83	 10
  7	 G 	   374.21	   508.30	  9
  8	 E 	   438.73	   479.79	  8
  9	 I 	   495.28	   415.27	  7
 10	 A 	   530.79	   358.73	  6
 11	 P 	   579.32	   323.21	  5
 12	 L 	   635.86	   274.68	  4
 13	 L 	   692.41	   218.14	  3
 14	 F 	   765.94	   161.60	  2
 15	 R 	        -	         88.06	  1
```

_

**8. IPI00375339 Isoform 1 of Sodium/potassium-transporting ATPase subunit alpha-4**

| Sequence | Charge | M+H+ | XCorr | DeltaCn |
| --- | --- | --- | --- | --- |
| K.MQINVQEVVLGDLVEIK.G | 2 | 1928.283 | 3.089 | 0.245 |

+1 charge

```
		      B   	     Y
  1	 M 	   132.05	        -	      17
  2	 Q 	   260.11	  1796.02	 16
  3	 I 	   373.19	  1667.96	 15
  4	 N 	   487.23	  1554.87	 14
  5	 V 	   586.30	  1440.83	 13
  6	 Q 	   714.36	  1341.76	 12
  7	 E 	   843.40	  1213.70	 11
  8	 V 	   942.47	  1084.66	 10
  9	 V 	  1041.54	   985.59	  9
 10	 L 	  1154.62	   886.52	  8
 11	 G 	  1211.65	   773.44	  7
 12	 D 	  1326.67	   716.42	  6
 13	 L 	  1439.76	   601.39	  5
 14	 V 	  1538.82	   488.31	  4
 15	 E 	  1667.87	   389.24	  3
 16	 I 	  1780.95	   260.20	  2
 17	 K 	        -	        147.11	  1  _
```

+2 charge

```
		      B   	     Y
  1	 M 	    66.53	        -	      17
  2	 Q 	   130.56	   898.51	 16
  3	 I 	   187.10	   834.48	 15
  4	 N 	   244.12	   777.94	 14
  5	 V 	   293.65	   720.92	 13
  6	 Q 	   357.68	   671.38	 12
  7	 E 	   422.21	   607.36	 11
  8	 V 	   471.74	   542.83	 10
  9	 V 	   521.27	   493.30	  9
 10	 L 	   577.82	   443.77	  8
 11	 G 	   606.33	   387.22	  7
 12	 D 	   663.84	   358.71	  6
 13	 L 	   720.38	   301.20	  5
 14	 V 	   769.92	   244.66	  4
 15	 E 	   834.44	   195.12	  3
 16	 I 	   890.98	   130.60	  2
 17	 K 	        -	         74.06	  1  _
```

**9. IPI00056309 Liver-expressed antimicrobial peptide 2**

| Sequence | Charge | M+H+ | XCorr | DeltaCn |
| --- | --- | --- | --- | --- |
| R.GVSLRPIGASCRDDSECITR.L | 3 | 2250.443 | 4.415 | 0.529 |

```
+1 charge
		      B   	       Y
  1	 G 	    58.03	        -  	 20
  2	 V 	   157.10	  2192.32	 19
  3	 S 	   244.13	  2093.26	 18
  4	 L 	   357.21	  2006.22	 17
  5	 R 	   513.31	  1893.14	 16
  6	 P 	   610.37	  1737.04	 15
  7	 I 	   723.45	  1639.99	 14
  8	 G 	   780.47	  1526.90	 13
  9	 A 	   851.51	  1469.88	 12
 10	 S 	   938.54	  1398.84	 11
 11	 C 	  1098.71	  1311.81	 10
 12	 R 	  1254.81	  1151.65	  9
 13	 D 	  1369.84	   995.54	  8
 14	 D 	  1484.86	   880.52	  7
 15	 S 	  1571.89	   765.49	  6
 16	 E 	  1700.94	   678.46	  5
 17	 C 	  1861.10	   549.42	  4
 18	 I 	  1974.19	   389.25	  3
 19	 T 	  2075.23	   276.17	  2
 20	 R 	        -  	   175.12	  1  _


+2 charge
		      B   	       Y
  1	 G 	    29.52	        -  	 20
  2	 V 	    79.05	  1096.67	 19
  3	 S 	   122.57	  1047.13	 18
  4	 L 	   179.11	  1003.62	 17
  5	 R 	   257.16	   947.07	 16
  6	 P 	   305.69	   869.02	 15
  7	 I 	   362.23	   820.50	 14
  8	 G 	   390.74	   763.95	 13
  9	 A 	   426.26	   735.44	 12
 10	 S 	   469.77	   699.93	 11
 11	 C 	   549.86	   656.41	 10
 12	 R 	   627.91	   576.33	  9
 13	 D 	   685.42	   498.28	  8
 14	 D 	   742.93	   440.76	  7
 15	 S 	   786.45	   383.25	  6
 16	 E 	   850.97	   339.73	  5
 17	 C 	   931.05	   275.21	  4
 18	 I 	   987.60	   195.13	  3
 19	 T 	  1038.12	   138.59	  2
 20	 R 	        -  	    88.06	  1  _
+3 charge
		      B   	       Y
  1	 G 	    20.01	        -  	 20
  2	 V 	    53.04	   731.45	 19
  3	 S 	    82.05	   698.42	 18
  4	 L 	   119.74	   669.41	 17
  5	 R 	   171.78	   631.72	 16
  6	 P 	   204.13	   579.68	 15
  7	 I 	   241.82	   547.33	 14
  8	 G 	   260.83	   509.64	 13
  9	 A 	   284.51	   490.63	 12
 10	 S 	   313.52	   466.95	 11
 11	 C 	   366.91	   437.94	 10
 12	 R 	   418.94	   384.55	  9
 13	 D 	   457.28	   332.52	  8
 14	 D 	   495.63	   294.18	  7
 15	 S 	   524.64	   255.84	  6
 16	 E 	   567.65	   226.82	  5
 17	 C 	   621.04	   183.81	  4
 18	 I 	   658.73	   130.42	  3
 19	 T 	   692.42	    92.73	  2
 20	 R 	        -  	    59.04	  1  _
```

**10. IPI00023315 Bone morphogenetic protein 3B**

| Sequence | Charge | M+H+ | XCorr | DeltaCn |
| --- | --- | --- | --- | --- |
| R.YDPFPAGDPEPR.A | 2 | 1361.441 | 2.88 | 0.447 |

```
+1 charge
		      B   	       Y
  1	 Y 	   164.07	        -  	 12
  2	 D 	   279.10	  1197.55	 11
  3	 P 	   376.15	  1082.53	 10
  4	 F 	   523.22	   985.47	  9
  5	 P 	   620.27	   838.41	  8
  6	 A 	   691.31	   741.35	  7
  7	 G 	   748.33	   670.32	  6
  8	 D 	   863.36	   613.29	  5
  9	 P 	   960.41	   498.27	  4
 10	 E 	  1089.45	   401.21	  3
 11	 P 	  1186.51	   272.17	  2
 12	 R 	        -  	   175.12	  1  _
+2 charge
		      B   	       Y
  1	 Y 	    82.54	        -  	 12
  2	 D 	   140.05	   599.28	 11
  3	 P 	   188.58	   541.77	 10
  4	 F 	   262.11	   493.24	  9
  5	 P 	   310.64	   419.71	  8
  6	 A 	   346.16	   371.18	  7
  7	 G 	   374.67	   335.66	  6
  8	 D 	   432.18	   307.15	  5
  9	 P 	   480.71	   249.64	  4
 10	 E 	   545.23	   201.11	  3
 11	 P 	   593.76	   136.59	  2
 12	 R 	        -  	    88.06	  1  _
```

**11. IPI00218803 Isoform B of Fibulin-1**

| Sequence | Charge | M+H+ | XCorr | DeltaCn |
| --- | --- | --- | --- | --- |
| R.LGESCINTVGSFR.C | 2 | 1440.577 | 3.085 | 0.549 |

```
+1 charge
		      B   	       Y
  1	 L 	   114.09	        -  	 13
  2	 G 	   171.11	  1326.75	 12
  3	 E 	   300.16	  1269.72	 11
  4	 S 	   387.19	  1140.68	 10
  5	 C 	   547.35	  1053.65	  9
  6	 I 	   660.44	   893.48	  8
  7	 N 	   774.48	   780.40	  7
  8	 T 	   875.53	   666.36	  6
  9	 V 	   974.60	   565.31	  5
 10	 G 	  1031.62	   466.24	  4
 11	 S 	  1118.65	   409.22	  3
 12	 F 	  1265.72	   322.19	  2
 13	 R 	        -  	   175.12	  1  _
+2 charge
		      B   	       Y
  1	 L 	    57.55	        -  	 13
  2	 G 	    86.06	   663.88	 12
  3	 E 	   150.58	   635.37	 11
  4	 S 	   194.10	   570.84	 10
  5	 C 	   274.18	   527.33	  9
  6	 I 	   330.72	   447.25	  8
  7	 N 	   387.74	   390.70	  7
  8	 T 	   438.27	   333.68	  6
  9	 V 	   487.80	   283.16	  5
 10	 G 	   516.31	   233.62	  4
 11	 S 	   559.83	   205.11	  3
 12	 F 	   633.36	   161.60	  2
 13	 R 	        -  	    88.06	  1  _
```

**12. IPI00006988 Resistin**

| Sequence | Charge | M+H+ | XCorr | DeltaCn |
| --- | --- | --- | --- | --- |
| R.IQEVAGSLIFR.A | 2 | 1233.442 | 3.111 | 0.517 |

```
+1 charge
		      B   	       Y
  1	 I 	   114.09	        -  	 11
  2	 Q 	   242.15	  1119.62	 10
  3	 E 	   371.19	   991.56	  9
  4	 V 	   470.26	   862.51	  8
  5	 A 	   541.30	   763.45	  7
  6	 G 	   598.32	   692.41	  6
  7	 S 	   685.35	   635.39	  5
  8	 L 	   798.44	   548.36	  4
  9	 I 	   911.52	   435.27	  3
 10	 F 	  1058.59	   322.19	  2
 11	 R 	        -  	   175.12	  1  _
+2 charge
		      B   	       Y
  1	 I 	    57.55	        -  	 11
  2	 Q 	   121.58	   560.31	 10
  3	 E 	   186.10	   496.28	  9
  4	 V 	   235.63	   431.76	  8
  5	 A 	   271.15	   382.23	  7
  6	 G 	   299.66	   346.71	  6
  7	 S 	   343.18	   318.20	  5
  8	 L 	   399.72	   274.68	  4
  9	 I 	   456.26	   218.14	  3
 10	 F 	   529.80	   161.60	  2
 11	 R 	        -  	    88.06	  1  _
```

**13. IPI00176222 Isoform 1 of Zinc finger protein 549**

| Sequence | Charge | M+H+ | XCorr | DeltaCn |
| --- | --- | --- | --- | --- |
| K.WFSFGSNLQQHQNQDSGEKHIRK.E | 3 | 2772.975 | 3.697 | 0.221 |

```
+1 charge
		      B   	       Y
  1	 W 	   187.09	        -  	 23
  2	 F 	   334.16	  2585.26	 22
  3	 S 	   421.19	  2438.19	 21
  4	 F 	   568.26	  2351.16	 20
  5	 G 	   625.28	  2204.09	 19
  6	 S 	   712.31	  2147.07	 18
  7	 N 	   826.35	  2060.04	 17
  8	 L 	   939.44	  1946.00	 16
  9	 Q 	  1067.49	  1832.91	 15
 10	 Q 	  1195.55	  1704.85	 14
 11	 H 	  1332.61	  1576.79	 13
 12	 Q 	  1460.67	  1439.73	 12
 13	 N 	  1574.71	  1311.68	 11
 14	 Q 	  1702.77	  1197.63	 10
 15	 D 	  1817.80	  1069.57	  9
 16	 S 	  1904.83	   954.55	  8
 17	 G 	  1961.85	   867.52	  7
 18	 E 	  2090.90	   810.49	  6
 19	 K 	  2218.99	   681.45	  5
 20	 H 	  2356.05	   553.36	  4
 21	 I 	  2469.13	   416.30	  3
 22	 R 	  2625.23	   303.21	  2
 23	 K 	        -  	   147.11	  1  _


+2 charge
		      B   	       Y
  1	 W 	    94.05	        -  	 23
  2	 F 	   167.58	  1293.13	 22
  3	 S 	   211.10	  1219.60	 21
  4	 F 	   284.63	  1176.08	 20
  5	 G 	   313.14	  1102.55	 19
  6	 S 	   356.66	  1074.04	 18
  7	 N 	   413.68	  1030.52	 17
  8	 L 	   470.22	   973.50	 16
  9	 Q 	   534.25	   916.96	 15
 10	 Q 	   598.28	   852.93	 14
 11	 H 	   666.81	   788.90	 13
 12	 Q 	   730.84	   720.37	 12
 13	 N 	   787.86	   656.34	 11
 14	 Q 	   851.89	   599.32	 10
 15	 D 	   909.40	   535.29	  9
 16	 S 	   952.92	   477.78	  8
 17	 G 	   981.43	   434.26	  7
 18	 E 	  1045.95	   405.75	  6
 19	 K 	  1110.00	   341.23	  5
 20	 H 	  1178.53	   277.18	  4
 21	 I 	  1235.07	   208.65	  3
 22	 R 	  1313.12	   152.11	  2
 23	 K 	        -  	    74.06	  1  _


+3 charge
		      B   	       Y
  1	 W 	    63.03	        -  	 23
  2	 F 	   112.06	   862.42	 22
  3	 S 	   141.07	   813.40	 21
  4	 F 	   190.09	   784.39	 20
  5	 G 	   209.10	   735.37	 19
  6	 S 	   238.11	   716.36	 18
  7	 N 	   276.12	   687.35	 17
  8	 L 	   313.82	   649.34	 16
  9	 Q 	   356.50	   611.64	 15
 10	 Q 	   399.19	   568.96	 14
 11	 H 	   444.88	   526.27	 13
 12	 Q 	   487.56	   480.58	 12
 13	 N 	   525.58	   437.90	 11
 14	 Q 	   568.26	   399.88	 10
 15	 D 	   606.60	   357.20	  9
 16	 S 	   635.62	   318.85	  8
 17	 G 	   654.62	   289.84	  7
 18	 E 	   697.64	   270.84	  6
 19	 K 	   740.33	   227.82	  5
 20	 H 	   786.02	   185.12	  4
 21	 I 	   823.72	   139.44	  3
 22	 R 	   875.75	   101.74	  2
 23	 K 	        -  	    49.71	  1  _
```

**14. IPI00289831 Isoform PTPS of Receptor-type tyrosine-protein phosphatase S**

| Sequence | Charge | M+H+ | XCorr | DeltaCn |
| --- | --- | --- | --- | --- |
| R.VLAFTSVGDGPLSDPIQVK.T | 2 | 1944.218 | 2.943 | 0.256 |

```
+1 charge
		      B   	       Y
  1	 V 	   100.08	        -  	 19
  2	 L 	   213.16	  1843.98	 18
  3	 A 	   284.20	  1730.90	 17
  4	 F 	   431.27	  1659.86	 16
  5	 T 	   532.31	  1512.79	 15
  6	 S 	   619.34	  1411.74	 14
  7	 V 	   718.41	  1324.71	 13
  8	 G 	   775.43	  1225.64	 12
  9	 D 	   890.46	  1168.62	 11
 10	 G 	   947.48	  1053.59	 10
 11	 P 	  1044.54	   996.57	  9
 12	 L 	  1157.62	   899.52	  8
 13	 S 	  1244.65	   786.44	  7
 14	 D 	  1359.68	   699.40	  6
 15	 P 	  1456.73	   584.38	  5
 16	 I 	  1569.82	   487.32	  4
 17	 Q 	  1697.87	   374.24	  3
 18	 V 	  1796.94	   246.18	  2
 19	 K 	        -  	   147.11	  1  _


+2 charge
		      B   	       Y
  1	 V 	    50.54	        -  	 19
  2	 L 	   107.08	   922.49	 18
  3	 A 	   142.60	   865.95	 17
  4	 F 	   216.14	   830.43	 16
  5	 T 	   266.66	   756.90	 15
  6	 S 	   310.18	   706.38	 14
  7	 V 	   359.71	   662.86	 13
  8	 G 	   388.22	   613.32	 12
  9	 D 	   445.73	   584.81	 11
 10	 G 	   474.25	   527.30	 10
 11	 P 	   522.77	   498.79	  9
 12	 L 	   579.31	   450.26	  8
 13	 S 	   622.83	   393.72	  7
 14	 D 	   680.34	   350.21	  6
 15	 P 	   728.87	   292.69	  5
 16	 I 	   785.41	   244.17	  4
 17	 Q 	   849.44	   187.62	  3
 18	 V 	   898.98	   123.59	  2
 19	 K 	        -  	    74.06	  1  _
```

**15. IPI00219788 Isoform A of Protein FAM3B**

| Sequence | Charge | M+H+ | XCorr | DeltaCn |
| --- | --- | --- | --- | --- |
| K.ICFEDNLLMGEQLGNVAR.G | 2 | 2080.345 | 3.079 | 0.457 |

```
+1 charge
		      B   	       Y
  1	 I 	   114.09	        -  	 18
  2	 C 	   274.26	  1966.05	 17
  3	 F 	   421.32	  1805.89	 16
  4	 E 	   550.37	  1658.82	 15
  5	 D 	   665.39	  1529.77	 14
  6	 N 	   779.44	  1414.75	 13
  7	 L 	   892.52	  1300.70	 12
  8	 L 	  1005.61	  1187.62	 11
  9	 M 	  1136.65	  1074.54	 10
 10	 G 	  1193.67	   943.50	  9
 11	 E 	  1322.71	   886.47	  8
 12	 Q 	  1450.77	   757.43	  7
 13	 L 	  1563.85	   629.37	  6
 14	 G 	  1620.87	   516.29	  5
 15	 N 	  1734.92	   459.27	  4
 16	 V 	  1833.99	   345.22	  3
 17	 A 	  1905.02	   246.16	  2
 18	 R 	        -  	   175.12	  1  _
+2 charge
		      B   	       Y
  1	 I 	    57.55	        -  	 18
  2	 C 	   137.63	   983.53	 17
  3	 F 	   211.17	   903.45	 16
  4	 E 	   275.69	   829.91	 15
  5	 D 	   333.20	   765.39	 14
  6	 N 	   390.22	   707.88	 13
  7	 L 	   446.76	   650.86	 12
  8	 L 	   503.31	   594.31	 11
  9	 M 	   568.83	   537.77	 10
 10	 G 	   597.34	   472.25	  9
 11	 E 	   661.86	   443.74	  8
 12	 Q 	   725.89	   379.22	  7
 13	 L 	   782.43	   315.19	  6
 14	 G 	   810.94	   258.65	  5
 15	 N 	   867.96	   230.14	  4
 16	 V 	   917.50	   173.12	  3
 17	 A 	   953.01	   123.58	  2
 18	 R 	        -  	    88.06	  1  _
```

**16. IPI00794461 Histone H2B type 1-N**

| Sequence | Charge | M+H+ | XCorr | DeltaCn |
| --- | --- | --- | --- | --- |
| K.AMGIMNSFVNDIFER.I | 2 | 1745.017 | 3.61 | 0.489 |

```
+1 charge
		      B   	       Y
  1	 A 	    72.04	        -  	 15
  2	 M 	   203.08	  1672.78	 14
  3	 G 	   260.11	  1541.74	 13
  4	 I 	   373.19	  1484.72	 12
  5	 M 	   504.23	  1371.64	 11
  6	 N 	   618.27	  1240.60	 10
  7	 S 	   705.31	  1126.55	  9
  8	 F 	   852.37	  1039.52	  8
  9	 V 	   951.44	   892.45	  7
 10	 N 	  1065.49	   793.38	  6
 11	 D 	  1180.51	   679.34	  5
 12	 I 	  1293.60	   564.31	  4
 13	 F 	  1440.67	   451.23	  3
 14	 E 	  1569.71	   304.16	  2
 15	 R 	        -  	   175.12	  1  _
+2 charge
		      B   	       Y
  1	 A 	    36.53	        -  	 15
  2	 M 	   102.05	   836.89	 14
  3	 G 	   130.56	   771.37	 13
  4	 I 	   187.10	   742.86	 12
  5	 M 	   252.62	   686.32	 11
  6	 N 	   309.64	   620.80	 10
  7	 S 	   353.16	   563.78	  9
  8	 F 	   426.69	   520.26	  8
  9	 V 	   476.22	   446.73	  7
 10	 N 	   533.25	   397.20	  6
 11	 D 	   590.76	   340.17	  5
 12	 I 	   647.30	   282.66	  4
 13	 F 	   720.84	   226.12	  3
 14	 E 	   785.36	   152.58	  2
 15	 R 	        -  	    88.06	  1  _
```

**17. IPI00167955 Putative uncharacterized protein FLJ35645**

| Sequence | Charge | M+H+ | XCorr | DeltaCn |
| --- | --- | --- | --- | --- |
| R.ERGGAVSRLNPETPTPSSPLPQEGGR.Q | 3 | 2690.911 | 3.577 | 0.302 |

```
+1 charge
		      B   	       Y
  1	 E 	   130.05	        -  	 26
  2	 R 	   286.15	  2560.32	 25
  3	 G 	   343.17	  2404.22	 24
  4	 G 	   400.19	  2347.20	 23
  5	 A 	   471.23	  2290.18	 22
  6	 V 	   570.30	  2219.14	 21
  7	 S 	   657.33	  2120.07	 20
  8	 R 	   813.43	  2033.04	 19
  9	 L 	   926.52	  1876.94	 18
 10	 N 	  1040.56	  1763.86	 17
 11	 P 	  1137.61	  1649.81	 16
 12	 E 	  1266.65	  1552.76	 15
 13	 T 	  1367.70	  1423.72	 14
 14	 P 	  1464.76	  1322.67	 13
 15	 T 	  1565.80	  1225.62	 12
 16	 P 	  1662.86	  1124.57	 11
 17	 S 	  1749.89	  1027.52	 10
 18	 S 	  1836.92	   940.48	  9
 19	 P 	  1933.97	   853.45	  8
 20	 L 	  2047.06	   756.40	  7
 21	 P 	  2144.11	   643.32	  6
 22	 Q 	  2272.17	   546.26	  5
 23	 E 	  2401.21	   418.20	  4
 24	 G 	  2458.23	   289.16	  3
 25	 G 	  2515.25	   232.14	  2
 26	 R 	        -  	   175.12	  1  _


+2 charge
		      B   	       Y
  1	 E 	    65.53	        -  	 26
  2	 R 	   143.58	  1280.66	 25
  3	 G 	   172.09	  1202.61	 24
  4	 G 	   200.60	  1174.10	 23
  5	 A 	   236.12	  1145.59	 22
  6	 V 	   285.65	  1110.07	 21
  7	 S 	   329.17	  1060.54	 20
  8	 R 	   407.22	  1017.02	 19
  9	 L 	   463.76	   938.97	 18
 10	 N 	   520.78	   882.43	 17
 11	 P 	   569.31	   825.41	 16
 12	 E 	   633.83	   776.88	 15
 13	 T 	   684.35	   712.36	 14
 14	 P 	   732.88	   661.84	 13
 15	 T 	   783.41	   613.31	 12
 16	 P 	   831.93	   562.79	 11
 17	 S 	   875.45	   514.26	 10
 18	 S 	   918.96	   470.75	  9
 19	 P 	   967.49	   427.23	  8
 20	 L 	  1024.03	   378.70	  7
 21	 P 	  1072.56	   322.16	  6
 22	 Q 	  1136.59	   273.64	  5
 23	 E 	  1201.11	   209.61	  4
 24	 G 	  1229.62	   145.08	  3
 25	 G 	  1258.13	   116.57	  2
 26	 R 	        -  	    88.06	  1  _


+3 charge
		      B   	       Y
  1	 E 	    44.02	        -  	 26
  2	 R 	    96.06	   854.11	 25
  3	 G 	   115.06	   802.08	 24
  4	 G 	   134.07	   783.07	 23
  5	 A 	   157.75	   764.06	 22
  6	 V 	   190.77	   740.39	 21
  7	 S 	   219.78	   707.36	 20
  8	 R 	   271.82	   678.35	 19
  9	 L 	   309.51	   626.32	 18
 10	 N 	   347.52	   588.62	 17
 11	 P 	   379.88	   550.61	 16
 12	 E 	   422.89	   518.26	 15
 13	 T 	   456.57	   475.24	 14
 14	 P 	   488.92	   441.56	 13
 15	 T 	   522.61	   409.21	 12
 16	 P 	   554.96	   375.53	 11
 17	 S 	   583.97	   343.18	 10
 18	 S 	   612.98	   314.17	  9
 19	 P 	   645.33	   285.16	  8
 20	 L 	   683.02	   252.80	  7
 21	 P 	   715.37	   215.11	  6
 22	 Q 	   758.06	   182.76	  5
 23	 E 	   801.08	   140.07	  4
 24	 G 	   820.08	    97.06	  3
 25	 G 	   839.09	    78.05	  2
 26	 R 	        -  	    59.04	  1  _
```

**18. IPI00007047 Protein S100-A8**

| Sequence | Charge | M+H+ | XCorr | DeltaCn |
| --- | --- | --- | --- | --- |
| K.KLLETECPQYIR.K | 2 | 1550.775 | 4.058 | 0.529 |

```
+1 charge
		      B   	       Y
  1	 K 	   129.10	        -  	 12
  2	 L 	   242.19	  1421.84	 11
  3	 L 	   355.27	  1308.76	 10
  4	 E 	   484.31	  1195.68	  9
  5	 T 	   585.36	  1066.63	  8
  6	 E 	   714.40	   965.59	  7
  7	 C 	   874.57	   836.54	  6
  8	 P 	   971.62	   676.38	  5
  9	 Q 	  1099.68	   579.32	  4
 10	 Y 	  1262.74	   451.27	  3
 11	 I 	  1375.83	   288.20	  2
 12	 R 	        -  	   175.12	  1  _
+2 charge
		      B   	       Y
  1	 K 	    65.05	        -  	 12
  2	 L 	   121.60	   711.43	 11
  3	 L 	   178.14	   654.88	 10
  4	 E 	   242.66	   598.34	  9
  5	 T 	   293.18	   533.82	  8
  6	 E 	   357.71	   483.30	  7
  7	 C 	   437.79	   418.78	  6
  8	 P 	   486.31	   338.69	  5
  9	 Q 	   550.34	   290.17	  4
 10	 Y 	   631.88	   226.14	  3
 11	 I 	   688.42	   144.61	  2
 12	 R 	        -  	    88.06	  1  _
```

**19. IPI00009010 tRNA methyltransferase 112 homolog**

| Sequence | Charge | M+H+ | XCorr | DeltaCn |
| --- | --- | --- | --- | --- |
| K.LLTHNLLSSHVRGVGSRGFPLR.L | 3 | 2417.801 | 3.477 | 0.201 |

```
+1 charge
		      B   	       Y
  1	 L 	   114.09	        -  	 22
  2	 L 	   227.18	  2303.28	 21
  3	 T 	   328.22	  2190.20	 20
  4	 H 	   465.28	  2089.15	 19
  5	 N 	   579.32	  1952.09	 18
  6	 L 	   692.41	  1838.05	 17
  7	 L 	   805.49	  1724.97	 16
  8	 S 	   892.53	  1611.88	 15
  9	 S 	   979.56	  1524.85	 14
 10	 H 	  1116.62	  1437.82	 13
 11	 V 	  1215.68	  1300.76	 12
 12	 R 	  1371.79	  1201.69	 11
 13	 G 	  1428.81	  1045.59	 10
 14	 V 	  1527.88	   988.57	  9
 15	 G 	  1584.90	   889.50	  8
 16	 S 	  1671.93	   832.48	  7
 17	 R 	  1828.03	   745.45	  6
 18	 G 	  1885.05	   589.35	  5
 19	 F 	  2032.12	   532.32	  4
 20	 P 	  2129.17	   385.26	  3
 21	 L 	  2242.26	   288.20	  2
 22	 R 	        -  	   175.12	  1  _


+2 charge
		      B   	       Y
  1	 L 	    57.55	        -  	 22
  2	 L 	   114.09	  1152.15	 21
  3	 T 	   164.62	  1095.60	 20
  4	 H 	   233.14	  1045.08	 19
  5	 N 	   290.17	   976.55	 18
  6	 L 	   346.71	   919.53	 17
  7	 L 	   403.25	   862.99	 16
  8	 S 	   446.77	   806.44	 15
  9	 S 	   490.28	   762.93	 14
 10	 H 	   558.81	   719.41	 13
 11	 V 	   608.35	   650.88	 12
 12	 R 	   686.40	   601.35	 11
 13	 G 	   714.91	   523.30	 10
 14	 V 	   764.44	   494.79	  9
 15	 G 	   792.95	   445.25	  8
 16	 S 	   836.47	   416.74	  7
 17	 R 	   914.52	   373.23	  6
 18	 G 	   943.03	   295.18	  5
 19	 F 	  1016.56	   266.67	  4
 20	 P 	  1065.09	   193.13	  3
 21	 L 	  1121.63	   144.61	  2
 22	 R 	        -  	    88.06	  1  _


+3 charge
		      B   	       Y
  1	 L 	    38.70	        -  	 22
  2	 L 	    76.40	   768.43	 21
  3	 T 	   110.08	   730.74	 20
  4	 H 	   155.77	   697.06	 19
  5	 N 	   193.78	   651.37	 18
  6	 L 	   231.47	   613.36	 17
  7	 L 	   269.17	   575.66	 16
  8	 S 	   298.18	   537.97	 15
  9	 S 	   327.19	   508.96	 14
 10	 H 	   372.88	   479.94	 13
 11	 V 	   405.90	   434.26	 12
 12	 R 	   457.93	   401.24	 11
 13	 G 	   476.94	   349.20	 10
 14	 V 	   509.96	   330.19	  9
 15	 G 	   528.97	   297.17	  8
 16	 S 	   557.98	   278.16	  7
 17	 R 	   610.01	   249.15	  6
 18	 G 	   629.02	   197.12	  5
 19	 F 	   678.04	   178.11	  4
 20	 P 	   710.40	   129.09	  3
 21	 L 	   748.09	    96.74	  2
 22	 R 	        -  	    59.04	  1  _
```

**20. IPI00294713 Isoform 1 of Mannan-binding lectin serine protease 2**

| Sequence | Charge | M+H+ | XCorr | DeltaCn |
| --- | --- | --- | --- | --- |
| R.LASPGFPGEYANDQER.R | 2 | 1751.834 | 3.531 | 0.495 |

```
+1 charge
		      B   	       Y
  1	 L 	   114.09	        -  	 16
  2	 A 	   185.13	  1637.72	 15
  3	 S 	   272.16	  1566.68	 14
  4	 P 	   369.21	  1479.65	 13
  5	 G 	   426.23	  1382.60	 12
  6	 F 	   573.30	  1325.58	 11
  7	 P 	   670.36	  1178.51	 10
  8	 G 	   727.38	  1081.45	  9
  9	 E 	   856.42	  1024.43	  8
 10	 Y 	  1019.48	   895.39	  7
 11	 A 	  1090.52	   732.33	  6
 12	 N 	  1204.56	   661.29	  5
 13	 D 	  1319.59	   547.25	  4
 14	 Q 	  1447.65	   432.22	  3
 15	 E 	  1576.69	   304.16	  2
 16	 R 	        -  	   175.12	  1  _
+2 charge
		      B   	       Y
  1	 L 	    57.55	        -  	 16
  2	 A 	    93.07	   819.36	 15
  3	 S 	   136.58	   783.84	 14
  4	 P 	   185.11	   740.33	 13
  5	 G 	   213.62	   691.80	 12
  6	 F 	   287.16	   663.29	 11
  7	 P 	   335.68	   589.76	 10
  8	 G 	   364.19	   541.23	  9
  9	 E 	   428.71	   512.72	  8
 10	 Y 	   510.25	   448.20	  7
 11	 A 	   545.76	   366.67	  6
 12	 N 	   602.79	   331.15	  5
 13	 D 	   660.30	   274.13	  4
 14	 Q 	   724.33	   216.61	  3
 15	 E 	   788.85	   152.58	  2
 16	 R 	        -  	    88.06	  1  _
```

**21. IPI00829834 Ig kappa chain V-III region VH (Fragment)**

| Sequence | Charge | M+H+ | XCorr | DeltaCn |
| --- | --- | --- | --- | --- |
| R.EIVMTQSPPTLSLSPGER.V | 2 | 1943.211 | 3.384 | 0.476 |

```
+1 charge
		      B   	       Y
  1	 E 	   130.05	        -  	 18
  2	 I 	   243.13	  1812.95	 17
  3	 V 	   342.20	  1699.87	 16
  4	 M 	   473.24	  1600.80	 15
  5	 T 	   574.29	  1469.76	 14
  6	 Q 	   702.35	  1368.71	 13
  7	 S 	   789.38	  1240.65	 12
  8	 P 	   886.43	  1153.62	 11
  9	 P 	   983.49	  1056.57	 10
 10	 T 	  1084.53	   959.52	  9
 11	 L 	  1197.62	   858.47	  8
 12	 S 	  1284.65	   745.38	  7
 13	 L 	  1397.73	   658.35	  6
 14	 S 	  1484.77	   545.27	  5
 15	 P 	  1581.82	   458.24	  4
 16	 G 	  1638.84	   361.18	  3
 17	 E 	  1767.88	   304.16	  2
 18	 R 	        -  	   175.12	  1  _
+2 charge
		      B   	       Y
  1	 E 	    65.53	        -  	 18
  2	 I 	   122.07	   906.98	 17
  3	 V 	   171.60	   850.44	 16
  4	 M 	   237.13	   800.90	 15
  5	 T 	   287.65	   735.38	 14
  6	 Q 	   351.68	   684.86	 13
  7	 S 	   395.19	   620.83	 12
  8	 P 	   443.72	   577.31	 11
  9	 P 	   492.25	   528.79	 10
 10	 T 	   542.77	   480.26	  9
 11	 L 	   599.31	   429.74	  8
 12	 S 	   642.83	   373.20	  7
 13	 L 	   699.37	   329.68	  6
 14	 S 	   742.89	   273.14	  5
 15	 P 	   791.41	   229.62	  4
 16	 G 	   819.92	   181.10	  3
 17	 E 	   884.45	   152.58	  2
 18	 R 	        -  	    88.06	  1  _
```

**22. IPI00336112 Isoform 1 of Mucin and cadherin-like protein**

| Sequence | Charge | M+H+ | XCorr | DeltaCn |
| --- | --- | --- | --- | --- |
| R.IQAQDPEFSDLNSAITYR.I | 2 | 2069.217 | 4.245 | 0.634 |

```
+1 charge
		      B   	       Y
  1	 I 	   114.09	        -  	 18
  2	 Q 	   242.15	  1954.91	 17
  3	 A 	   313.19	  1826.86	 16
  4	 Q 	   441.25	  1755.82	 15
  5	 D 	   556.27	  1627.76	 14
  6	 P 	   653.33	  1512.73	 13
  7	 E 	   782.37	  1415.68	 12
  8	 F 	   929.44	  1286.64	 11
  9	 S 	  1016.47	  1139.57	 10
 10	 D 	  1131.50	  1052.54	  9
 11	 L 	  1244.58	   937.51	  8
 12	 N 	  1358.62	   824.43	  7
 13	 S 	  1445.65	   710.38	  6
 14	 A 	  1516.69	   623.35	  5
 15	 I 	  1629.78	   552.31	  4
 16	 T 	  1730.82	   439.23	  3
 17	 Y 	  1893.89	   338.18	  2
 18	 R 	        -  	   175.12	  1  _
+2 charge
		      B   	       Y
  1	 I 	    57.55	        -  	 18
  2	 Q 	   121.58	   977.96	 17
  3	 A 	   157.10	   913.93	 16
  4	 Q 	   221.13	   878.41	 15
  5	 D 	   278.64	   814.38	 14
  6	 P 	   327.17	   756.87	 13
  7	 E 	   391.69	   708.34	 12
  8	 F 	   465.22	   643.82	 11
  9	 S 	   508.74	   570.29	 10
 10	 D 	   566.25	   526.77	  9
 11	 L 	   622.79	   469.26	  8
 12	 N 	   679.81	   412.72	  7
 13	 S 	   723.33	   355.70	  6
 14	 A 	   758.85	   312.18	  5
 15	 I 	   815.39	   276.66	  4
 16	 T 	   865.92	   220.12	  3
 17	 Y 	   947.45	   169.59	  2
 18	 R 	        -  	    88.06	  1  _
```

**23. IPI00022417 Leucine-rich alpha-2-glycoprotein**

| Sequence | Charge | M+H+ | XCorr | DeltaCn |
| --- | --- | --- | --- | --- |
| K.LQELHLSSNGLESLSPEFLRPVPQLR.V | 3 | 2961.365 | 4.898 | 0.629 |

```
+1 charge
		      B   	       Y
  1	 L 	   114.09	        -  	 26
  2	 Q 	   242.15	  2846.52	 25
  3	 E 	   371.19	  2718.46	 24
  4	 L 	   484.28	  2589.41	 23
  5	 H 	   621.34	  2476.33	 22
  6	 L 	   734.42	  2339.27	 21
  7	 S 	   821.45	  2226.19	 20
  8	 S 	   908.48	  2139.16	 19
  9	 N 	  1022.53	  2052.12	 18
 10	 G 	  1079.55	  1938.08	 17
 11	 L 	  1192.63	  1881.06	 16
 12	 E 	  1321.67	  1767.98	 15
 13	 S 	  1408.71	  1638.93	 14
 14	 L 	  1521.79	  1551.90	 13
 15	 S 	  1608.82	  1438.82	 12
 16	 P 	  1705.88	  1351.78	 11
 17	 E 	  1834.92	  1254.73	 10
 18	 F 	  1981.99	  1125.69	  9
 19	 L 	  2095.07	   978.62	  8
 20	 R 	  2251.17	   865.54	  7
 21	 P 	  2348.22	   709.44	  6
 22	 V 	  2447.29	   612.38	  5
 23	 P 	  2544.35	   513.31	  4
 24	 Q 	  2672.40	   416.26	  3
 25	 L 	  2785.49	   288.20	  2
 26	 R 	        -  	   175.12	  1  _


+2 charge
		      B   	       Y
  1	 L 	    57.55	        -  	 26
  2	 Q 	   121.58	  1423.76	 25
  3	 E 	   186.10	  1359.73	 24
  4	 L 	   242.64	  1295.21	 23
  5	 H 	   311.17	  1238.67	 22
  6	 L 	   367.71	  1170.14	 21
  7	 S 	   411.23	  1113.60	 20
  8	 S 	   454.75	  1070.08	 19
  9	 N 	   511.77	  1026.57	 18
 10	 G 	   540.28	   969.54	 17
 11	 L 	   596.82	   941.03	 16
 12	 E 	   661.34	   884.49	 15
 13	 S 	   704.86	   819.97	 14
 14	 L 	   761.40	   776.45	 13
 15	 S 	   804.92	   719.91	 12
 16	 P 	   853.44	   676.40	 11
 17	 E 	   917.96	   627.87	 10
 18	 F 	   991.50	   563.35	  9
 19	 L 	  1048.04	   489.81	  8
 20	 R 	  1126.09	   433.27	  7
 21	 P 	  1174.62	   355.22	  6
 22	 V 	  1224.15	   306.70	  5
 23	 P 	  1272.68	   257.16	  4
 24	 Q 	  1336.71	   208.63	  3
 25	 L 	  1393.25	   144.61	  2
 26	 R 	        -  	    88.06	  1  _


+3 charge
		      B   	       Y
  1	 L 	    38.70	        -  	 26
  2	 Q 	    81.39	   949.51	 25
  3	 E 	   124.40	   906.82	 24
  4	 L 	   162.10	   863.81	 23
  5	 H 	   207.78	   826.12	 22
  6	 L 	   245.48	   780.43	 21
  7	 S 	   274.49	   742.73	 20
  8	 S 	   303.50	   713.72	 19
  9	 N 	   341.51	   684.71	 18
 10	 G 	   360.52	   646.70	 17
 11	 L 	   398.22	   627.69	 16
 12	 E 	   441.23	   590.00	 15
 13	 S 	   470.24	   546.98	 14
 14	 L 	   507.94	   517.97	 13
 15	 S 	   536.95	   480.28	 12
 16	 P 	   569.30	   451.27	 11
 17	 E 	   612.31	   418.92	 10
 18	 F 	   661.33	   375.90	  9
 19	 L 	   699.03	   326.88	  8
 20	 R 	   751.06	   289.18	  7
 21	 P 	   783.41	   237.15	  6
 22	 V 	   816.44	   204.80	  5
 23	 P 	   848.79	   171.78	  4
 24	 Q 	   891.47	   139.43	  3
 25	 L 	   929.17	    96.74	  2
 26	 R 	        -  	    59.04	  1  _
```

**24. IPI00004310 Ly6/PLAUR domain-containing protein 3**

| Sequence | Charge | M+H+ | XCorr | DeltaCn |
| --- | --- | --- | --- | --- |
| R.GLDLHGLLAFIQLQQCAQDR.C | 3 | 2297.593 | 4.69 | 0.493 |

```
+1 charge
		      B   	       Y
  1	 G 	    58.03	        -  	 20
  2	 L 	   171.11	  2239.30	 19
  3	 D 	   286.14	  2126.22	 18
  4	 L 	   399.22	  2011.19	 17
  5	 H 	   536.28	  1898.10	 16
  6	 G 	   593.30	  1761.05	 15
  7	 L 	   706.39	  1704.02	 14
  8	 L 	   819.47	  1590.94	 13
  9	 A 	   890.51	  1477.86	 12
 10	 F 	  1037.58	  1406.82	 11
 11	 I 	  1150.66	  1259.75	 10
 12	 Q 	  1278.72	  1146.67	  9
 13	 L 	  1391.80	  1018.61	  8
 14	 Q 	  1519.86	   905.52	  7
 15	 Q 	  1647.92	   777.47	  6
 16	 C 	  1808.09	   649.41	  5
 17	 A 	  1879.12	   489.24	  4
 18	 Q 	  2007.18	   418.20	  3
 19	 D 	  2122.21	   290.15	  2
 20	 R 	        -  	   175.12	  1  _


+2 charge
		      B   	       Y
  1	 G 	    29.52	        -  	 20
  2	 L 	    86.06	  1120.15	 19
  3	 D 	   143.57	  1063.61	 18
  4	 L 	   200.12	  1006.10	 17
  5	 H 	   268.64	   949.56	 16
  6	 G 	   297.16	   881.03	 15
  7	 L 	   353.70	   852.52	 14
  8	 L 	   410.24	   795.97	 13
  9	 A 	   445.76	   739.43	 12
 10	 F 	   519.29	   703.91	 11
 11	 I 	   575.83	   630.38	 10
 12	 Q 	   639.86	   573.84	  9
 13	 L 	   696.41	   509.81	  8
 14	 Q 	   760.44	   453.27	  7
 15	 Q 	   824.46	   389.24	  6
 16	 C 	   904.55	   325.21	  5
 17	 A 	   940.07	   245.12	  4
 18	 Q 	  1004.09	   209.61	  3
 19	 D 	  1061.61	   145.58	  2
 20	 R 	        -  	    88.06	  1  _
+3 charge
		      B   	       Y
  1	 G 	    20.01	        -  	 20
  2	 L 	    57.71	   747.10	 19
  3	 D 	    96.05	   709.41	 18
  4	 L 	   133.75	   671.07	 17
  5	 H 	   179.43	   633.37	 16
  6	 G 	   198.44	   587.69	 15
  7	 L 	   236.13	   568.68	 14
  8	 L 	   273.83	   530.98	 13
  9	 A 	   297.51	   493.29	 12
 10	 F 	   346.53	   469.61	 11
 11	 I 	   384.23	   420.59	 10
 12	 Q 	   426.91	   382.89	  9
 13	 L 	   464.61	   340.21	  8
 14	 Q 	   507.29	   302.51	  7
 15	 Q 	   549.98	   259.83	  6
 16	 C 	   603.37	   217.14	  5
 17	 A 	   627.05	   163.75	  4
 18	 Q 	   669.73	   140.07	  3
 19	 D 	   708.07	    97.39	  2
 20	 R 	        -  	    59.04	  1  _
```

**25. IPI00009027 Lithostathine-1-alpha**

| Sequence | Charge | M+H+ | XCorr | DeltaCn |
| --- | --- | --- | --- | --- |
| R.ISCPEGTNAYR.S | 2 | 1268.352 | 2.979 | 0.484 |

```
+1 charge
		      B   	       Y
  1	 I 	   114.09	        -  	 11
  2	 S 	   201.12	  1154.62	 10
  3	 C 	   361.29	  1067.59	  9
  4	 P 	   458.34	   907.43	  8
  5	 E 	   587.38	   810.37	  7
  6	 G 	   644.41	   681.33	  6
  7	 T 	   745.45	   624.31	  5
  8	 N 	   859.50	   523.26	  4
  9	 A 	   930.53	   409.22	  3
 10	 Y 	  1093.60	   338.18	  2
 11	 R 	        -  	   175.12	  1  _
+2 charge
		      B   	       Y
  1	 I 	    57.55	        -  	 11
  2	 S 	   101.07	   577.82	 10
  3	 C 	   181.15	   534.30	  9
  4	 P 	   229.67	   454.22	  8
  5	 E 	   294.20	   405.69	  7
  6	 G 	   322.71	   341.17	  6
  7	 T 	   373.23	   312.66	  5
  8	 N 	   430.25	   262.13	  4
  9	 A 	   465.77	   205.11	  3
 10	 Y 	   547.30	   169.59	  2
 11	 R 	        -  	    88.06	  1  _
```

**26. IPI00009997 N-acetyllactosaminide beta-1,3-N-acetylglucosaminyltransferase**

| Sequence | Charge | M+H+ | XCorr | DeltaCn |
| --- | --- | --- | --- | --- |
| R.TALASGGVLDASGDYR.V | 2 | 1553.656 | 2.969 | 0.387 |

**+1 charge**

```
		      B   	       Y
  1	 T 	   102.05	        -  	 16
  2	 A 	   173.09	  1451.71	 15
  3	 L 	   286.18	  1380.68	 14
  4	 A 	   357.21	  1267.59	 13
  5	 S 	   444.25	  1196.55	 12
  6	 G 	   501.27	  1109.52	 11
  7	 G 	   558.29	  1052.50	 10
  8	 V 	   657.36	   995.48	  9
  9	 L 	   770.44	   896.41	  8
 10	 D 	   885.47	   783.33	  7
 11	 A 	   956.50	   668.30	  6
 12	 S 	  1043.54	   597.26	  5
 13	 G 	  1100.56	   510.23	  4
 14	 D 	  1215.59	   453.21	  3
 15	 Y 	  1378.65	   338.18	  2
 16	 R 	        -  	   175.12	  1  _
```

**+2 charge**

```
		      B   	       Y
  1	 T 	    51.53	        -  	 16
  2	 A 	    87.05	   726.36	 15
  3	 L 	   143.59	   690.84	 14
  4	 A 	   179.11	   634.30	 13
  5	 S 	   222.63	   598.78	 12
  6	 G 	   251.14	   555.26	 11
  7	 G 	   279.65	   526.75	 10
  8	 V 	   329.18	   498.24	  9
  9	 L 	   385.72	   448.71	  8
 10	 D 	   443.24	   392.17	  7
 11	 A 	   478.76	   334.65	  6
 12	 S 	   522.27	   299.13	  5
 13	 G 	   550.78	   255.62	  4
 14	 D 	   608.30	   227.11	  3
 15	 Y 	   689.83	   169.59	  2
 16	 R 	        -  	    88.06	  1  _
```

**27. IPI00936772 Isoform 1 of Golgi membrane protein 1**

| Sequence | Charge | M+H+ | XCorr | DeltaCn |
| --- | --- | --- | --- | --- |
| R.NIDVFNVEDQKR.D | 2 | 1477.604 | 2.904 | 0.379 |

**+1 charge**

```
		      B   	       Y
  1	 N 	   115.05	        -  	 12
  2	 I 	   228.13	  1362.70	 11
  3	 D 	   343.16	  1249.62	 10
  4	 V 	   442.23	  1134.59	  9
  5	 F 	   589.30	  1035.52	  8
  6	 N 	   703.34	   888.45	  7
  7	 V 	   802.41	   774.41	  6
  8	 E 	   931.45	   675.34	  5
  9	 D 	  1046.48	   546.30	  4
 10	 Q 	  1174.54	   431.27	  3
 11	 K 	  1302.63	   303.21	  2
 12	 R 	        -  	   175.12	  1  _
+2 charge
		      B   	       Y
  1	 N 	    58.03	        -  	 12
  2	 I 	   114.57	   681.85	 11
  3	 D 	   172.08	   625.31	 10
  4	 V 	   221.62	   567.80	  9
  5	 F 	   295.15	   518.26	  8
  6	 N 	   352.17	   444.73	  7
  7	 V 	   401.71	   387.71	  6
  8	 E 	   466.23	   338.17	  5
  9	 D 	   523.74	   273.65	  4
 10	 Q 	   587.77	   216.14	  3
 11	 K 	   651.82	   152.11	  2
 12	 R 	        -  	    88.06	  1  _
```

**28. IPI00219827 Secreted Ly-6/uPAR-related protein 2**

| Sequence | Charge | M+H+ | XCorr | DeltaCn |
| --- | --- | --- | --- | --- |
| R.VLSNTEDLPLVTK.M | 2 | 1429.641 | 4.381 | 0.622 |

**+1 charge**

```
		      B   	       Y
  1	 V 	   100.08	        -  	 13
  2	 L 	   213.16	  1329.73	 12
  3	 S 	   300.19	  1216.64	 11
  4	 N 	   414.23	  1129.61	 10
  5	 T 	   515.28	  1015.57	  9
  6	 E 	   644.32	   914.52	  8
  7	 D 	   759.35	   785.48	  7
  8	 L 	   872.44	   670.45	  6
  9	 P 	   969.49	   557.37	  5
 10	 L 	  1082.57	   460.31	  4
 11	 V 	  1181.64	   347.23	  3
 12	 T 	  1282.69	   248.16	  2
 13	 K 	        -  	   147.11	  1  _
+2 charge
		      B   	       Y
  1	 V 	    50.54	        -  	 13
  2	 L 	   107.08	   665.37	 12
  3	 S 	   150.60	   608.82	 11
  4	 N 	   207.62	   565.31	 10
  5	 T 	   258.14	   508.29	  9
  6	 E 	   322.67	   457.76	  8
  7	 D 	   380.18	   393.24	  7
  8	 L 	   436.72	   335.73	  6
  9	 P 	   485.25	   279.19	  5
 10	 L 	   541.79	   230.66	  4
 11	 V 	   591.32	   174.12	  3
 12	 T 	   641.85	   124.58	  2
 13	 K 	        -  	    74.06	  1  _
```

**29. IPI00553215 V1-5 protein**

| Sequence | Charge | M+H+ | XCorr | DeltaCn |
| --- | --- | --- | --- | --- |
| K.LMIYEVSNRPSGVPDRFSGSK.S | 3 | 2340.644 | 3.684 | 0.355 |

**+1 charge**

```
		      B   	       Y
  1	 L 	   114.09	        -  	 21
  2	 M 	   245.13	  2226.10	 20
  3	 I 	   358.22	  2095.06	 19
  4	 Y 	   521.28	  1981.97	 18
  5	 E 	   650.32	  1818.91	 17
  6	 V 	   749.39	  1689.87	 16
  7	 S 	   836.42	  1590.80	 15
  8	 N 	   950.47	  1503.77	 14
  9	 R 	  1106.57	  1389.72	 13
 10	 P 	  1203.62	  1233.62	 12
 11	 S 	  1290.65	  1136.57	 11
 12	 G 	  1347.67	  1049.54	 10
 13	 V 	  1446.74	   992.52	  9
 14	 P 	  1543.79	   893.45	  8
 15	 D 	  1658.82	   796.39	  7
 16	 R 	  1814.92	   681.37	  6
 17	 F 	  1961.99	   525.27	  5
 18	 S 	  2049.02	   378.20	  4
 19	 G 	  2106.04	   291.17	  3
 20	 S 	  2193.08	   234.14	  2
 21	 K 	        -  	   147.11	  1  _


+2 charge
		      B   	       Y
  1	 L 	    57.55	        -  	 21
  2	 M 	   123.07	  1113.55	 20
  3	 I 	   179.61	  1048.03	 19
  4	 Y 	   261.14	   991.49	 18
  5	 E 	   325.66	   909.96	 17
  6	 V 	   375.20	   845.44	 16
  7	 S 	   418.71	   795.90	 15
  8	 N 	   475.74	   752.39	 14
  9	 R 	   553.79	   695.37	 13
 10	 P 	   602.31	   617.31	 12
 11	 S 	   645.83	   568.79	 11
 12	 G 	   674.34	   525.27	 10
 13	 V 	   723.87	   496.76	  9
 14	 P 	   772.40	   447.23	  8
 15	 D 	   829.91	   398.70	  7
 16	 R 	   907.96	   341.19	  6
 17	 F 	   981.50	   263.14	  5
 18	 S 	  1025.01	   189.60	  4
 19	 G 	  1053.53	   146.09	  3
 20	 S 	  1097.04	   117.58	  2
 21	 K 	        -  	    74.06	  1  _


+3 charge
		      B   	       Y
  1	 L 	    38.70	        -  	 21
  2	 M 	    82.38	   742.70	 20
  3	 I 	   120.08	   699.02	 19
  4	 Y 	   174.43	   661.33	 18
  5	 E 	   217.45	   606.97	 17
  6	 V 	   250.47	   563.96	 16
  7	 S 	   279.48	   530.94	 15
  8	 N 	   317.49	   501.93	 14
  9	 R 	   369.53	   463.91	 13
 10	 P 	   401.88	   411.88	 12
 11	 S 	   430.89	   379.53	 11
 12	 G 	   449.90	   350.52	 10
 13	 V 	   482.92	   331.51	  9
 14	 P 	   515.27	   298.49	  8
 15	 D 	   553.61	   266.14	  7
 16	 R 	   605.65	   227.79	  6
 17	 F 	   654.67	   175.76	  5
 18	 S 	   683.68	   126.74	  4
 19	 G 	   702.69	    97.73	  3
 20	 S 	   731.70	    78.72	  2
 21	 K 	        -  	    49.71	  1  _
```

**30. IPI00011694 Trypsin-1**

| Sequence | Charge | M+H+ | XCorr | DeltaCn |
| --- | --- | --- | --- | --- |
| K.TLNNDIMLIK.L | 2 | 1175.424 | 3.549 | 0.281 |

**+1 charge**

```
		      B   	       Y
  1	 T 	   102.05	        -  	 10
  2	 L 	   215.14	  1073.60	  9
  3	 N 	   329.18	   960.52	  8
  4	 N 	   443.22	   846.48	  7
  5	 D 	   558.25	   732.43	  6
  6	 I 	   671.34	   617.41	  5
  7	 M 	   802.38	   504.32	  4
  8	 L 	   915.46	   373.28	  3
  9	 I 	  1028.54	   260.20	  2
 10	 K 	        -  	   147.11	  1  _
+2 charge
		      B   	       Y
  1	 T 	    51.53	        -  	 10
  2	 L 	   108.07	   537.30	  9
  3	 N 	   165.09	   480.76	  8
  4	 N 	   222.12	   423.74	  7
  5	 D 	   279.63	   366.72	  6
  6	 I 	   336.17	   309.21	  5
  7	 M 	   401.69	   252.66	  4
  8	 L 	   458.23	   187.14	  3
  9	 I 	   514.78	   130.60	  2
 10	 K 	        -  	    74.06	  1  _
```

**31. IPI00022488 Hemopexin**

| Sequence | Charge | M+H+ | XCorr | DeltaCn |
| --- | --- | --- | --- | --- |
| R.YYCFQGNQFLR.F | 2 | 1496.644 | 3.144 | 0.411 |

**+1 charge**

```
		      B   	       Y
  1	 Y 	   164.07	        -  	 11
  2	 Y 	   327.13	  1332.75	 10
  3	 C 	   487.30	  1169.69	  9
  4	 F 	   634.37	  1009.52	  8
  5	 Q 	   762.43	   862.45	  7
  6	 G 	   819.45	   734.39	  6
  7	 N 	   933.49	   677.37	  5
  8	 Q 	  1061.55	   563.33	  4
  9	 F 	  1208.62	   435.27	  3
 10	 L 	  1321.70	   288.20	  2
 11	 R 	        -  	   175.12	  1  _
+2 charge
		      B   	       Y
  1	 Y 	    82.54	        -  	 11
  2	 Y 	   164.07	   666.88	 10
  3	 C 	   244.15	   585.35	  9
  4	 F 	   317.69	   505.26	  8
  5	 Q 	   381.72	   431.73	  7
  6	 G 	   410.23	   367.70	  6
  7	 N 	   467.25	   339.19	  5
  8	 Q 	   531.28	   282.17	  4
  9	 F 	   604.81	   218.14	  3
 10	 L 	   661.35	   144.61	  2
 11	 R 	        -  	    88.06	  1  _
```

**32. IPI00013446 Prostate stem cell antigen**

| Sequence | Charge | M+H+ | XCorr | DeltaCn |
| --- | --- | --- | --- | --- |
| R.AVGLLTVISK.G | 2 | 1001.245 | 3.596 | 0.241 |

**+1 charge**

```
		      B   	       Y
  1	 A 	    72.04	        -  	 10
  2	 V 	   171.11	   929.60	  9
  3	 G 	   228.13	   830.53	  8
  4	 L 	   341.22	   773.51	  7
  5	 L 	   454.30	   660.43	  6
  6	 T 	   555.35	   547.34	  5
  7	 V 	   654.42	   446.30	  4
  8	 I 	   767.50	   347.23	  3
  9	 S 	   854.53	   234.14	  2
 10	 K 	        -  	   147.11	  1  _
+2 charge
		      B   	       Y
  1	 A 	    36.53	        -  	 10
  2	 V 	    86.06	   465.31	  9
  3	 G 	   114.57	   415.77	  8
  4	 L 	   171.11	   387.26	  7
  5	 L 	   227.65	   330.72	  6
  6	 T 	   278.18	   274.18	  5
  7	 V 	   327.71	   223.65	  4
  8	 I 	   384.25	   174.12	  3
  9	 S 	   427.77	   117.58	  2
 10	 K 	        -  	    74.06	  1  _.
```

**33. IPI00012102 N-acetylglucosamine-6-sulfatase**

| Sequence | Charge | M+H+ | XCorr | DeltaCn |
| --- | --- | --- | --- | --- |
| K.IQEPNTFPAILR.S | 2 | 1399.62 | 3.277 | 0.277 |

**+1 charge**

```
		      B   	       Y
  1	 I 	   114.09	        -  	 12
  2	 Q 	   242.15	  1285.69	 11
  3	 E 	   371.19	  1157.63	 10
  4	 P 	   468.25	  1028.59	  9
  5	 N 	   582.29	   931.54	  8
  6	 T 	   683.34	   817.49	  7
  7	 F 	   830.40	   716.45	  6
  8	 P 	   927.46	   569.38	  5
  9	 A 	   998.49	   472.32	  4
 10	 I 	  1111.58	   401.29	  3
 11	 L 	  1224.66	   288.20	  2
 12	 R 	        -  	   175.12	  1  _
+2 charge
		      B   	       Y
  1	 I 	    57.55	        -  	 12
  2	 Q 	   121.58	   643.35	 11
  3	 E 	   186.10	   579.32	 10
  4	 P 	   234.63	   514.80	  9
  5	 N 	   291.65	   466.27	  8
  6	 T 	   342.17	   409.25	  7
  7	 F 	   415.71	   358.73	  6
  8	 P 	   464.23	   285.19	  5
  9	 A 	   499.75	   236.67	  4
 10	 I 	   556.29	   201.15	  3
 11	 L 	   612.83	   144.61	  2
 12	 R 	        -  	    88.06	  1  _
```

**34. IPI00012887 Cathepsin L1**

| Sequence | Charge | M+H+ | XCorr | DeltaCn |
| --- | --- | --- | --- | --- |
| K.VFQEPLFYEAPR.S | 2 | 1496.691 | 2.855 | 0.33 |

**+1 charge**

```
		      B   	       Y
  1	 V 	   100.08	        -  	 12
  2	 F 	   247.14	  1396.69	 11
  3	 Q 	   375.20	  1249.62	 10
  4	 E 	   504.25	  1121.56	  9
  5	 P 	   601.30	   992.52	  8
  6	 L 	   714.38	   895.47	  7
  7	 F 	   861.45	   782.38	  6
  8	 Y 	  1024.51	   635.31	  5
  9	 E 	  1153.56	   472.25	  4
 10	 A 	  1224.59	   343.21	  3
 11	 P 	  1321.65	   272.17	  2
 12	 R 	        -  	   175.12	  1  _
+2 charge
		      B   	       Y
  1	 V 	    50.54	        -  	 12
  2	 F 	   124.08	   698.85	 11
  3	 Q 	   188.10	   625.31	 10
  4	 E 	   252.63	   561.28	  9
  5	 P 	   301.15	   496.76	  8
  6	 L 	   357.69	   448.24	  7
  7	 F 	   431.23	   391.70	  6
  8	 Y 	   512.76	   318.16	  5
  9	 E 	   577.28	   236.63	  4
 10	 A 	   612.80	   172.11	  3
 11	 P 	   661.33	   136.59	  2
 12	 R 	        -  	    88.06	  1  _
```

**35. IPI00843975 Ezrin**

| Sequence | Charge | M+H+ | XCorr | DeltaCn |
| --- | --- | --- | --- | --- |
| K.SQEQLAAELAEYTAK.I | 2 | 1652.784 | 3.77 | 0.527 |

**+1 charge**

```
		      B   	       Y
  1	 S 	    88.04	        -  	 15
  2	 Q 	   216.10	  1564.79	 14
  3	 E 	   345.14	  1436.73	 13
  4	 Q 	   473.20	  1307.68	 12
  5	 L 	   586.28	  1179.63	 11
  6	 A 	   657.32	  1066.54	 10
  7	 A 	   728.36	   995.50	  9
  8	 E 	   857.40	   924.47	  8
  9	 L 	   970.48	   795.42	  7
 10	 A 	  1041.52	   682.34	  6
 11	 E 	  1170.56	   611.30	  5
 12	 Y 	  1333.63	   482.26	  4
 13	 T 	  1434.67	   319.20	  3
 14	 A 	  1505.71	   218.15	  2
 15	 K 	        -  	   147.11	  1  _
+2 charge
		      B   	       Y
  1	 S 	    44.52	        -  	 15
  2	 Q 	   108.55	   782.90	 14
  3	 E 	   173.07	   718.87	 13
  4	 Q 	   237.10	   654.35	 12
  5	 L 	   293.65	   590.32	 11
  6	 A 	   329.16	   533.77	 10
  7	 A 	   364.68	   498.26	  9
  8	 E 	   429.20	   462.74	  8
  9	 L 	   485.75	   398.22	  7
 10	 A 	   521.26	   341.67	  6
 11	 E 	   585.79	   306.16	  5
 12	 Y 	   667.32	   241.63	  4
 13	 T 	   717.84	   160.10	  3
 14	 A 	   753.36	   109.58	  2
 15	 K 	        -  	    74.06	  1  _
```

**36. IPI00170635 Secreted and transmembrane protein 1**

| Sequence | Charge | M+H+ | XCorr | DeltaCn |
| --- | --- | --- | --- | --- |
| R.AHGQESAIFNEVAPGYFSR.D | 2 | 2081.233 | 4.231 | 0.657 |

**+1 charge**

```
		      B   	       Y
  1	 A 	    72.04	        -  	 19
  2	 H 	   209.10	  2008.95	 18
  3	 G 	   266.12	  1871.89	 17
  4	 Q 	   394.18	  1814.87	 16
  5	 E 	   523.23	  1686.81	 15
  6	 S 	   610.26	  1557.77	 14
  7	 A 	   681.30	  1470.74	 13
  8	 I 	   794.38	  1399.70	 12
  9	 F 	   941.45	  1286.62	 11
 10	 N 	  1055.49	  1139.55	 10
 11	 E 	  1184.53	  1025.51	  9
 12	 V 	  1283.60	   896.46	  8
 13	 A 	  1354.64	   797.39	  7
 14	 P 	  1451.69	   726.36	  6
 15	 G 	  1508.71	   629.30	  5
 16	 Y 	  1671.78	   572.28	  4
 17	 F 	  1818.84	   409.22	  3
 18	 S 	  1905.88	   262.15	  2
 19	 R 	        -  	   175.12	  1  _
+2 charge
		      B   	       Y
  1	 A 	    36.53	        -  	 19
  2	 H 	   105.06	  1004.98	 18
  3	 G 	   133.57	   936.45	 17
  4	 Q 	   197.60	   907.94	 16
  5	 E 	   262.12	   843.91	 15
  6	 S 	   305.63	   779.39	 14
  7	 A 	   341.15	   735.87	 13
  8	 I 	   397.69	   700.35	 12
  9	 F 	   471.23	   643.81	 11
 10	 N 	   528.25	   570.28	 10
 11	 E 	   592.77	   513.26	  9
 12	 V 	   642.30	   448.73	  8
 13	 A 	   677.82	   399.20	  7
 14	 P 	   726.35	   363.68	  6
 15	 G 	   754.86	   315.16	  5
 16	 Y 	   836.39	   286.64	  4
 17	 F 	   909.93	   205.11	  3
 18	 S 	   953.44	   131.58	  2
 19	 R 	        -  	    88.06	  1  _
```

**37. IPI00216065 Isoform 2 of Vitamin K-dependent protein Z**

| Sequence | Charge | M+H+ | XCorr | DeltaCn |
| --- | --- | --- | --- | --- |
| R.APDLQDLPWQVK.L | 2 | 1410.599 | 4.349 | 0.524 |

**+1 charge**

```
		      B   	       Y
  1	 A 	    72.04	        -  	 12
  2	 P 	   169.10	  1338.71	 11
  3	 D 	   284.12	  1241.65	 10
  4	 L 	   397.21	  1126.63	  9
  5	 Q 	   525.27	  1013.54	  8
  6	 D 	   640.29	   885.48	  7
  7	 L 	   753.38	   770.46	  6
  8	 P 	   850.43	   657.37	  5
  9	 W 	  1036.51	   560.32	  4
 10	 Q 	  1164.57	   374.24	  3
 11	 V 	  1263.64	   246.18	  2
 12	 K 	        -  	   147.11	  1  _
+2 charge
		      B   	       Y
  1	 A 	    36.53	        -  	 12
  2	 P 	    85.05	   669.86	 11
  3	 D 	   142.57	   621.33	 10
  4	 L 	   199.11	   563.82	  9
  5	 Q 	   263.14	   507.27	  8
  6	 D 	   320.65	   443.25	  7
  7	 L 	   377.19	   385.73	  6
  8	 P 	   425.72	   329.19	  5
  9	 W 	   518.76	   280.66	  4
 10	 Q 	   582.79	   187.62	  3
 11	 V 	   632.32	   123.59	  2
 12	 K 	        -  	    74.06	  1  _
```

**38. IPI00176776 Uncharacterized protein C11orf85**

| Sequence | Charge | M+H+ | XCorr | DeltaCn |
| --- | --- | --- | --- | --- |
| K.WFHENLSPGKPISDSPLGLVPVEKK.A | 3 | 2776.182 | 4.315 | 0.232 |

**+1 charge**

```
		      B   	       Y
  1	 W 	   187.09	        -  	 25
  2	 F 	   334.16	  2588.41	 24
  3	 H 	   471.21	  2441.34	 23
  4	 E 	   600.26	  2304.28	 22
  5	 N 	   714.30	  2175.24	 21
  6	 L 	   827.38	  2061.20	 20
  7	 S 	   914.42	  1948.11	 19
  8	 P 	  1011.47	  1861.08	 18
  9	 G 	  1068.49	  1764.03	 17
 10	 K 	  1196.58	  1707.01	 16
 11	 P 	  1293.64	  1578.91	 15
 12	 I 	  1406.72	  1481.86	 14
 13	 S 	  1493.75	  1368.77	 13
 14	 D 	  1608.78	  1281.74	 12
 15	 S 	  1695.81	  1166.71	 11
 16	 P 	  1792.87	  1079.68	 10
 17	 L 	  1905.95	   982.63	  9
 18	 G 	  1962.97	   869.55	  8
 19	 L 	  2076.05	   812.52	  7
 20	 V 	  2175.12	   699.44	  6
 21	 P 	  2272.18	   600.37	  5
 22	 V 	  2371.24	   503.32	  4
 23	 E 	  2500.29	   404.25	  3
 24	 K 	  2628.38	   275.21	  2
 25	 K 	        -  	   147.11	  1  _


+2 charge
		      B   	       Y
  1	 W 	    94.05	        -  	 25
  2	 F 	   167.58	  1294.71	 24
  3	 H 	   236.11	  1221.17	 23
  4	 E 	   300.63	  1152.64	 22
  5	 N 	   357.65	  1088.12	 21
  6	 L 	   414.20	  1031.10	 20
  7	 S 	   457.71	   974.56	 19
  8	 P 	   506.24	   931.04	 18
  9	 G 	   534.75	   882.52	 17
 10	 K 	   598.80	   854.01	 16
 11	 P 	   647.32	   789.96	 15
 12	 I 	   703.86	   741.43	 14
 13	 S 	   747.38	   684.89	 13
 14	 D 	   804.89	   641.37	 12
 15	 S 	   848.41	   583.86	 11
 16	 P 	   896.94	   540.34	 10
 17	 L 	   953.48	   491.82	  9
 18	 G 	   981.99	   435.28	  8
 19	 L 	  1038.53	   406.77	  7
 20	 V 	  1088.07	   350.22	  6
 21	 P 	  1136.59	   300.69	  5
 22	 V 	  1186.13	   252.16	  4
 23	 E 	  1250.65	   202.63	  3
 24	 K 	  1314.69	   138.11	  2
 25	 K 	        -  	    74.06	  1  _


+3 charge
		      B   	       Y
  1	 W 	    63.03	        -  	 25
  2	 F 	   112.06	   863.47	 24
  3	 H 	   157.74	   814.45	 23
  4	 E 	   200.76	   768.77	 22
  5	 N 	   238.77	   725.75	 21
  6	 L 	   276.47	   687.74	 20
  7	 S 	   305.48	   650.04	 19
  8	 P 	   337.83	   621.03	 18
  9	 G 	   356.83	   588.68	 17
 10	 K 	   399.53	   569.67	 16
 11	 P 	   431.88	   526.97	 15
 12	 I 	   469.58	   494.62	 14
 13	 S 	   498.59	   456.93	 13
 14	 D 	   536.93	   427.92	 12
 15	 S 	   565.94	   389.58	 11
 16	 P 	   598.29	   360.57	 10
 17	 L 	   635.99	   328.21	  9
 18	 G 	   655.00	   290.52	  8
 19	 L 	   692.69	   271.51	  7
 20	 V 	   725.71	   233.82	  6
 21	 P 	   758.06	   200.80	  5
 22	 V 	   791.09	   168.44	  4
 23	 E 	   834.10	   135.42	  3
 24	 K 	   876.80	    92.41	  2
 25	 K 	        -  	    49.71	  1  _
```

**39. IPI00022620 Secreted Ly-6/uPAR-related protein 1**

| Sequence | Charge | M+H+ | XCorr | DeltaCn |
| --- | --- | --- | --- | --- |
| R.SCSSSCVATDPDSIGAAHLIFCCFR.D | 3 | 2820.036 | 4.87 | 0.499 |

**+1 charge**

```
		      B   	       Y
  1	 S 	    88.04	        -  	 25
  2	 C 	   248.20	  2731.71	 24
  3	 S 	   335.24	  2571.55	 23
  4	 S 	   422.27	  2484.51	 22
  5	 S 	   509.30	  2397.48	 21
  6	 C 	   669.47	  2310.45	 20
  7	 V 	   768.53	  2150.28	 19
  8	 A 	   839.57	  2051.22	 18
  9	 T 	   940.62	  1980.18	 17
 10	 D 	  1055.65	  1879.13	 16
 11	 P 	  1152.70	  1764.10	 15
 12	 D 	  1267.73	  1667.05	 14
 13	 S 	  1354.76	  1552.03	 13
 14	 I 	  1467.84	  1464.99	 12
 15	 G 	  1524.86	  1351.91	 11
 16	 A 	  1595.90	  1294.89	 10
 17	 A 	  1666.94	  1223.85	  9
 18	 H 	  1804.00	  1152.81	  8
 19	 L 	  1917.08	  1015.75	  7
 20	 I 	  2030.16	   902.67	  6
 21	 F 	  2177.23	   789.59	  5
 22	 C 	  2337.40	   642.52	  4
 23	 C 	  2497.56	   482.35	  3
 24	 F 	  2644.63	   322.19	  2
 25	 R 	        -  	   175.12	  1  _


+2 charge
		      B   	       Y
  1	 S 	    44.52	        -  	 25
  2	 C 	   124.61	  1366.36	 24
  3	 S 	   168.12	  1286.28	 23
  4	 S 	   211.64	  1242.76	 22
  5	 S 	   255.15	  1199.24	 21
  6	 C 	   335.24	  1155.73	 20
  7	 V 	   384.77	  1075.65	 19
  8	 A 	   420.29	  1026.11	 18
  9	 T 	   470.81	   990.59	 17
 10	 D 	   528.33	   940.07	 16
 11	 P 	   576.85	   882.56	 15
 12	 D 	   634.37	   834.03	 14
 13	 S 	   677.88	   776.52	 13
 14	 I 	   734.42	   733.00	 12
 15	 G 	   762.94	   676.46	 11
 16	 A 	   798.45	   647.95	 10
 17	 A 	   833.97	   612.43	  9
 18	 H 	   902.50	   576.91	  8
 19	 L 	   959.04	   508.38	  7
 20	 I 	  1015.59	   451.84	  6
 21	 F 	  1089.12	   395.30	  5
 22	 C 	  1169.20	   321.76	  4
 23	 C 	  1249.29	   241.68	  3
 24	 F 	  1322.82	   161.60	  2
 25	 R 	        -  	    88.06	  1  _


+3 charge
		      B   	       Y
  1	 S 	    30.02	        -  	 25
  2	 C 	    83.41	   911.24	 24
  3	 S 	   112.42	   857.85	 23
  4	 S 	   141.43	   828.84	 22
  5	 S 	   170.44	   799.83	 21
  6	 C 	   223.83	   770.82	 20
  7	 V 	   256.85	   717.43	 19
  8	 A 	   280.53	   684.41	 18
  9	 T 	   314.21	   660.73	 17
 10	 D 	   352.55	   627.05	 16
 11	 P 	   384.90	   588.71	 15
 12	 D 	   423.25	   556.36	 14
 13	 S 	   452.26	   518.01	 13
 14	 I 	   489.95	   489.00	 12
 15	 G 	   508.96	   451.31	 11
 16	 A 	   532.64	   432.30	 10
 17	 A 	   556.32	   408.62	  9
 18	 H 	   602.00	   384.94	  8
 19	 L 	   639.70	   339.26	  7
 20	 I 	   677.39	   301.56	  6
 21	 F 	   726.42	   263.87	  5
 22	 C 	   779.80	   214.84	  4
 23	 C 	   833.19	   161.46	  3
 24	 F 	   882.22	   108.07	  2
 25	 R 	        -  	    59.04	  1  _
```

**40. IPI00007797 Fatty acid-binding protein, epidermal**

| Sequence | Charge | M+H+ | XCorr | DeltaCn |
| --- | --- | --- | --- | --- |
| R.LVDSKGFDEYMKELGVGIALR.K | 3 | 2341.712 | 4.671 | 0.543 |

**+1 charge**

```
		      B   	       Y
  1	 L 	   114.09	        -  	 21
  2	 V 	   213.16	  2227.14	 20
  3	 D 	   328.19	  2128.07	 19
  4	 S 	   415.22	  2013.05	 18
  5	 K 	   543.31	  1926.02	 17
  6	 G 	   600.34	  1797.92	 16
  7	 F 	   747.40	  1740.90	 15
  8	 D 	   862.43	  1593.83	 14
  9	 E 	   991.47	  1478.80	 13
 10	 Y 	  1154.54	  1349.76	 12
 11	 M 	  1285.58	  1186.70	 11
 12	 K 	  1413.67	  1055.66	 10
 13	 E 	  1542.71	   927.56	  9
 14	 L 	  1655.80	   798.52	  8
 15	 G 	  1712.82	   685.44	  7
 16	 V 	  1811.89	   628.41	  6
 17	 G 	  1868.91	   529.35	  5
 18	 I 	  1981.99	   472.32	  4
 19	 A 	  2053.03	   359.24	  3
 20	 L 	  2166.12	   288.20	  2
 21	 R 	        -  	   175.12	  1  _


+2 charge
		      B   	       Y
  1	 L 	    57.55	        -  	 21
  2	 V 	   107.08	  1114.08	 20
  3	 D 	   164.60	  1064.54	 19
  4	 S 	   208.11	  1007.03	 18
  5	 K 	   272.16	   963.51	 17
  6	 G 	   300.67	   899.46	 16
  7	 F 	   374.21	   870.95	 15
  8	 D 	   431.72	   797.42	 14
  9	 E 	   496.24	   739.91	 13
 10	 Y 	   577.77	   675.38	 12
 11	 M 	   643.29	   593.85	 11
 12	 K 	   707.34	   528.33	 10
 13	 E 	   771.86	   464.28	  9
 14	 L 	   828.40	   399.76	  8
 15	 G 	   856.91	   343.22	  7
 16	 V 	   906.45	   314.71	  6
 17	 G 	   934.96	   265.18	  5
 18	 I 	   991.50	   236.67	  4
 19	 A 	  1027.02	   180.12	  3
 20	 L 	  1083.56	   144.61	  2
 21	 R 	        -  	    88.06	  1  _


+3 charge
		      B   	       Y
  1	 L 	    38.70	        -  	 21
  2	 V 	    71.72	   743.05	 20
  3	 D 	   110.07	   710.03	 19
  4	 S 	   139.08	   671.69	 18
  5	 K 	   181.78	   642.68	 17
  6	 G 	   200.78	   599.98	 16
  7	 F 	   249.81	   580.97	 15
  8	 D 	   288.15	   531.95	 14
  9	 E 	   331.16	   493.61	 13
 10	 Y 	   385.52	   450.59	 12
 11	 M 	   429.20	   396.24	 11
 12	 K 	   471.90	   352.56	 10
 13	 E 	   514.91	   309.86	  9
 14	 L 	   552.60	   266.84	  8
 15	 G 	   571.61	   229.15	  7
 16	 V 	   604.63	   210.14	  6
 17	 G 	   623.64	   177.12	  5
 18	 I 	   661.34	   158.11	  4
 19	 A 	   685.02	   120.42	  3
 20	 L 	   722.71	    96.74	  2
 21	 R 	        -  	    59.04	  1  _
```

**41. IPI00418125 Isoform 1 of Layilin**

| Sequence | Charge | M+H+ | XCorr | DeltaCn |
| --- | --- | --- | --- | --- |
| K.FIENLLPSDGDFWIGLR.R | 2 | 1993.25 | 3.57 | 0.412 |

**+1 charge**

```
		      B   	       Y
  1	 F 	   148.08	        -  	 17
  2	 I 	   261.16	  1844.95	 16
  3	 E 	   390.20	  1731.87	 15
  4	 N 	   504.25	  1602.83	 14
  5	 L 	   617.33	  1488.78	 13
  6	 L 	   730.41	  1375.70	 12
  7	 P 	   827.47	  1262.62	 11
  8	 S 	   914.50	  1165.56	 10
  9	 D 	  1029.53	  1078.53	  9
 10	 G 	  1086.55	   963.50	  8
 11	 D 	  1201.57	   906.48	  7
 12	 F 	  1348.64	   791.46	  6
 13	 W 	  1534.72	   644.39	  5
 14	 I 	  1647.81	   458.31	  4
 15	 G 	  1704.83	   345.22	  3
 16	 L 	  1817.91	   288.20	  2
 17	 R 	        -  	   175.12	  1  _
+2 charge
		      B   	       Y
  1	 F 	    74.54	        -  	 17
  2	 I 	   131.08	   922.98	 16
  3	 E 	   195.60	   866.44	 15
  4	 N 	   252.63	   801.92	 14
  5	 L 	   309.17	   744.90	 13
  6	 L 	   365.71	   688.35	 12
  7	 P 	   414.24	   631.81	 11
  8	 S 	   457.75	   583.29	 10
  9	 D 	   515.27	   539.77	  9
 10	 G 	   543.78	   482.26	  8
 11	 D 	   601.29	   453.75	  7
 12	 F 	   674.82	   396.23	  6
 13	 W 	   767.86	   322.70	  5
 14	 I 	   824.41	   229.66	  4
 15	 G 	   852.92	   173.12	  3
 16	 L 	   909.46	   144.61	  2
 17	 R 	        -  	    88.06	  1  _
```

**42. IPI00027436 Tumor necrosis factor receptor superfamily member 16**

| Sequence | Charge | M+H+ | XCorr | DeltaCn |
| --- | --- | --- | --- | --- |
| R.WADAECEEIPGR.W | 2 | 1433.499 | 4.069 | 0.444 |

```
+1 charge
		      B   	       Y
  1	 W 	   187.09	        -  	 12
  2	 A 	   258.12	  1246.67	 11
  3	 D 	   373.15	  1175.63	 10
  4	 A 	   444.19	  1060.61	  9
  5	 E 	   573.23	   989.57	  8
  6	 C 	   733.40	   860.53	  7
  7	 E 	   862.44	   700.36	  6
  8	 E 	   991.48	   571.32	  5
  9	 I 	  1104.56	   442.28	  4
 10	 P 	  1201.62	   329.19	  3
 11	 G 	  1258.64	   232.14	  2
 12	 R 	        -  	   175.12	  1  _
+2 charge
		      B   	       Y
  1	 W 	    94.05	        -  	 12
  2	 A 	   129.57	   623.84	 11
  3	 D 	   187.08	   588.32	 10
  4	 A 	   222.60	   530.81	  9
  5	 E 	   287.12	   495.29	  8
  6	 C 	   367.20	   430.77	  7
  7	 E 	   431.72	   350.68	  6
  8	 E 	   496.24	   286.16	  5
  9	 I 	   552.79	   221.64	  4
 10	 P 	   601.31	   165.10	  3
 11	 G 	   629.82	   116.57	  2
 12	 R 	        -  	    88.06	  1  _
```

**43. IPI00011140 Protein NOV homolog**

| Sequence | Charge | M+H+ | XCorr | DeltaCn |
| --- | --- | --- | --- | --- |
| R.AVLDGCSCCLVCAR.Q | 2 | 1641.823 | 3.829 | 0.503 |

**+1 charge**

```
		      B   	       Y
  1	 A 	    72.04	        -  	 14
  2	 V 	   171.11	  1570.20	 13
  3	 L 	   284.20	  1471.13	 12
  4	 D 	   399.22	  1358.05	 11
  5	 G 	   456.25	  1243.02	 10
  6	 C 	   616.41	  1186.00	  9
  7	 S 	   703.44	  1025.84	  8
  8	 C 	   863.61	   938.80	  7
  9	 C 	  1023.77	   778.64	  6
 10	 L 	  1136.86	   618.47	  5
 11	 V 	  1235.93	   505.39	  4
 12	 C 	  1396.09	   406.32	  3
 13	 A 	  1467.13	   246.16	  2
 14	 R 	        -  	   175.12	  1  _
+2 charge

		      B   	       Y
  1	 A 	    36.53	        -  	 14
  2	 V 	    86.06	   785.60	 13
  3	 L 	   142.60	   736.07	 12
  4	 D 	   200.12	   679.53	 11
  5	 G 	   228.63	   622.02	 10
  6	 C 	   308.71	   593.50	  9
  7	 S 	   352.22	   513.42	  8
  8	 C 	   432.31	   469.91	  7
  9	 C 	   512.39	   389.82	  6
 10	 L 	   568.93	   309.74	  5
 11	 V 	   618.47	   253.20	  4
 12	 C 	   698.55	   203.66	  3
 13	 A 	   734.07	   123.58	  2
 14	 R 	        -  	    88.06	  1  _
```

**44. IPI00177884 Isoform 1 of Ras GTPase-activating protein SynGAP**

| Sequence | Charge | M+H+ | XCorr | DeltaCn |
| --- | --- | --- | --- | --- |
| R.PSQGFLSR.R | 1 | 891.9942 | 2.382 | 0.21 |

**+1 charge**

```
		      B   	       Y
  1	 P 	    98.06	        -  	  8
  2	 S 	   185.09	   794.42	  7
  3	 Q 	   313.15	   707.38	  6
  4	 G 	   370.17	   579.32	  5
  5	 F 	   517.24	   522.30	  4
  6	 L 	   630.32	   375.24	  3
  7	 S 	   717.36	   262.15	  2
  8	 R 	        -  	   175.12	  1  _
```

**45. IPI00384401 Myosin-reactive immunoglobulin kappa chain variable region (Fragment)**

| Sequence | Charge | M+H+ | XCorr | DeltaCn |
| --- | --- | --- | --- | --- |
| - .EIVMTQSPATLSVSPGER.A | 2 | 1903.147 | 4.547 | 0.516 |

**+1 charge**

```
		      B   	       Y
  1	 E 	   130.05	        -  	 18
  2	 I 	   243.13	  1772.92	 17
  3	 V 	   342.20	  1659.84	 16
  4	 M 	   473.24	  1560.77	 15
  5	 T 	   574.29	  1429.73	 14
  6	 Q 	   702.35	  1328.68	 13
  7	 S 	   789.38	  1200.62	 12
  8	 P 	   886.43	  1113.59	 11
  9	 A 	   957.47	  1016.54	 10
 10	 T 	  1058.52	   945.50	  9
 11	 L 	  1171.60	   844.45	  8
 12	 S 	  1258.63	   731.37	  7
 13	 V 	  1357.70	   644.34	  6
 14	 S 	  1444.74	   545.27	  5
 15	 P 	  1541.79	   458.24	  4
 16	 G 	  1598.81	   361.18	  3
 17	 E 	  1727.85	   304.16	  2
 18	 R 	        -  	   175.12	  1  _


+2 charge

		      B   	       Y
  1	 E 	    65.53	        -  	 18
  2	 I 	   122.07	   886.96	 17
  3	 V 	   171.60	   830.42	 16
  4	 M 	   237.13	   780.89	 15
  5	 T 	   287.65	   715.37	 14
  6	 Q 	   351.68	   664.84	 13
  7	 S 	   395.19	   600.81	 12
  8	 P 	   443.72	   557.30	 11
  9	 A 	   479.24	   508.77	 10
 10	 T 	   529.76	   473.25	  9
 11	 L 	   586.31	   422.73	  8
 12	 S 	   629.82	   366.19	  7
 13	 V 	   679.36	   322.67	  6
 14	 S 	   722.87	   273.14	  5
 15	 P 	   771.40	   229.62	  4
 16	 G 	   799.91	   181.10	  3
 17	 E 	   864.43	   152.58	  2
 18	 R 	        -  	    88.06	  1  _
```

**46. IPI00299738 Procollagen C-endopeptidase enhancer 1**

| Sequence | Charge | M+H+ | XCorr | DeltaCn |
| --- | --- | --- | --- | --- |
| R.FCGTFRPAPLVAPGNQVTLR.M | 3 | 2202.538 | 4.252 | 0.469 |

**+1 charge**

```
		      B   	       Y
  1	 F 	   148.08	        -  	 20
  2	 C 	   308.24	  2054.23	 19
  3	 G 	   365.26	  1894.07	 18
  4	 T 	   466.31	  1837.04	 17
  5	 F 	   613.38	  1736.00	 16
  6	 R 	   769.48	  1588.93	 15
  7	 P 	   866.53	  1432.83	 14
  8	 A 	   937.57	  1335.77	 13
  9	 P 	  1034.62	  1264.74	 12
 10	 L 	  1147.71	  1167.68	 11
 11	 V 	  1246.77	  1054.60	 10
 12	 A 	  1317.81	   955.53	  9
 13	 P 	  1414.86	   884.49	  8
 14	 G 	  1471.89	   787.44	  7
 15	 N 	  1585.93	   730.42	  6
 16	 Q 	  1713.99	   616.38	  5
 17	 V 	  1813.06	   488.32	  4
 18	 T 	  1914.10	   389.25	  3
 19	 L 	  2027.19	   288.20	  2
 20	 R 	        -  	   175.12	  1  _
+2 charge
		      B   	       Y
  1	 F 	    74.54	        -  	 20
  2	 C 	   154.62	  1027.62	 19
  3	 G 	   183.13	   947.54	 18
  4	 T 	   233.66	   919.03	 17
  5	 F 	   307.19	   868.50	 16
  6	 R 	   385.24	   794.97	 15
  7	 P 	   433.77	   716.92	 14
  8	 A 	   469.29	   668.39	 13
  9	 P 	   517.81	   632.87	 12
 10	 L 	   574.36	   584.35	 11
 11	 V 	   623.89	   527.80	 10
 12	 A 	   659.41	   478.27	  9
 13	 P 	   707.94	   442.75	  8
 14	 G 	   736.45	   394.22	  7
 15	 N 	   793.47	   365.71	  6
 16	 Q 	   857.50	   308.69	  5
 17	 V 	   907.03	   244.66	  4
 18	 T 	   957.56	   195.13	  3
 19	 L 	  1014.10	   144.61	  2
 20	 R 	        -  	    88.06	  1  _
+3 charge
		      B   	       Y
  1	 F 	    50.03	        -  	 20
  2	 C 	   103.42	   685.42	 19
  3	 G 	   122.43	   632.03	 18
  4	 T 	   156.11	   613.02	 17
  5	 F 	   205.13	   579.34	 16
  6	 R 	   257.16	   530.31	 15
  7	 P 	   289.52	   478.28	 14
  8	 A 	   313.19	   445.93	 13
  9	 P 	   345.55	   422.25	 12
 10	 L 	   383.24	   389.90	 11
 11	 V 	   416.26	   352.20	 10
 12	 A 	   439.94	   319.18	  9
 13	 P 	   472.29	   295.50	  8
 14	 G 	   491.30	   263.15	  7
 15	 N 	   529.31	   244.15	  6
 16	 Q 	   572.00	   206.13	  5
 17	 V 	   605.02	   163.44	  4
 18	 T 	   638.71	   130.42	  3
 19	 L 	   676.40	    96.74	  2
 20	 R 	        -  	    59.04	  1  _
```

**47. IPI00409698 Isoform 1 of Transportin-2**

| Sequence | Charge | M+H+ | XCorr | DeltaCn |
| --- | --- | --- | --- | --- |
| K.QECLNNIGDASSLIR.A | 2 | 1690.831 | 3.585 | 0.274 |

**+1 charge**

```
		      B   	       Y
  1	 Q 	   129.07	        -  	 15
  2	 E 	   258.11	  1561.90	 14
  3	 C 	   418.27	  1432.86	 13
  4	 L 	   531.36	  1272.69	 12
  5	 N 	   645.40	  1159.61	 11
  6	 N 	   759.44	  1045.56	 10
  7	 I 	   872.53	   931.52	  9
  8	 G 	   929.55	   818.44	  8
  9	 D 	  1044.58	   761.42	  7
 10	 A 	  1115.61	   646.39	  6
 11	 S 	  1202.65	   575.35	  5
 12	 S 	  1289.68	   488.32	  4
 13	 L 	  1402.76	   401.29	  3
 14	 I 	  1515.85	   288.20	  2
 15	 R 	        -  	   175.12	  1  _
+2 charge

		      B   	       Y
  1	 Q 	    65.04	        -  	 15
  2	 E 	   129.56	   781.45	 14
  3	 C 	   209.64	   716.93	 13
  4	 L 	   266.18	   636.85	 12
  5	 N 	   323.20	   580.31	 11
  6	 N 	   380.23	   523.29	 10
  7	 I 	   436.77	   466.26	  9
  8	 G 	   465.28	   409.72	  8
  9	 D 	   522.79	   381.21	  7
 10	 A 	   558.31	   323.70	  6
 11	 S 	   601.83	   288.18	  5
 12	 S 	   645.34	   244.66	  4
 13	 L 	   701.88	   201.15	  3
 14	 I 	   758.43	   144.61	  2
 15	 R 	        -  	    88.06	  1  _
```

**48. IPI00003091 Isoform 1 of Protein phosphatase 1 regulatory subunit 3A**

| Sequence | Charge | M+H+ | XCorr | DeltaCn |
| --- | --- | --- | --- | --- |
| K.EQEPEPVKPWKEVPNR.Q | 2 | 1963.183 | 3.686 | 0.241 |

**+1 charge**

```
		      B   	       Y
  1	 E 	   130.05	        -  	 16
  2	 Q 	   258.11	  1832.97	 15
  3	 E 	   387.15	  1704.91	 14
  4	 P 	   484.20	  1575.86	 13
  5	 E 	   613.25	  1478.81	 12
  6	 P 	   710.30	  1349.77	 11
  7	 V 	   809.37	  1252.72	 10
  8	 K 	   937.46	  1153.65	  9
  9	 P 	  1034.52	  1025.55	  8
 10	 W 	  1220.59	   928.50	  7
 11	 K 	  1348.69	   742.42	  6
 12	 E 	  1477.73	   614.33	  5
 13	 V 	  1576.80	   485.28	  4
 14	 P 	  1673.85	   386.21	  3
 15	 N 	  1787.90	   289.16	  2
 16	 R 	        -  	   175.12	  1  _
+2 charge

		      B   	       Y
  1	 E 	    65.53	        -  	 16
  2	 Q 	   129.56	   916.99	 15
  3	 E 	   194.08	   852.96	 14
  4	 P 	   242.61	   788.44	 13
  5	 E 	   307.13	   739.91	 12
  6	 P 	   355.65	   675.39	 11
  7	 V 	   405.19	   626.86	 10
  8	 K 	   469.23	   577.33	  9
  9	 P 	   517.76	   513.28	  8
 10	 W 	   610.80	   464.75	  7
 11	 K 	   674.85	   371.71	  6
 12	 E 	   739.37	   307.67	  5
 13	 V 	   788.90	   243.15	  4
 14	 P 	   837.43	   193.61	  3
 15	 N 	   894.45	   145.08	  2
 16	 R 	        -  	    88.06	  1  _
```

**49. IPI00025846 Isoform 2A of Desmocollin-2**

| Sequence | Charge | M+H+ | XCorr | DeltaCn |
| --- | --- | --- | --- | --- |
| R.LSYQNDPPFGSYVVPITVR.D | 2 | 2153.422 | 3.391 | 0.52 |

**+1 charge**

```
		      B   	       Y
  1	 L 	   114.09	        -  	 19
  2	 S 	   201.12	  2039.02	 18
  3	 Y 	   364.19	  1951.99	 17
  4	 Q 	   492.25	  1788.93	 16
  5	 N 	   606.29	  1660.87	 15
  6	 D 	   721.32	  1546.83	 14
  7	 P 	   818.37	  1431.80	 13
  8	 P 	   915.42	  1334.75	 12
  9	 F 	  1062.49	  1237.69	 11
 10	 G 	  1119.51	  1090.63	 10
 11	 S 	  1206.54	  1033.60	  9
 12	 Y 	  1369.61	   946.57	  8
 13	 V 	  1468.67	   783.51	  7
 14	 V 	  1567.74	   684.44	  6
 15	 P 	  1664.80	   585.37	  5
 16	 I 	  1777.88	   488.32	  4
 17	 T 	  1878.93	   375.24	  3
 18	 V 	  1978.00	   274.19	  2
 19	 R 	        -  	   175.12	  1  _
+2 charge
		      B   	       Y
  1	 L 	    57.55	        -  	 19
  2	 S 	   101.07	  1020.02	 18
  3	 Y 	   182.60	   976.50	 17
  4	 Q 	   246.63	   894.97	 16
  5	 N 	   303.65	   830.94	 15
  6	 D 	   361.16	   773.92	 14
  7	 P 	   409.69	   716.40	 13
  8	 P 	   458.21	   667.88	 12
  9	 F 	   531.75	   619.35	 11
 10	 G 	   560.26	   545.82	 10
 11	 S 	   603.77	   517.31	  9
 12	 Y 	   685.31	   473.79	  8
 13	 V 	   734.84	   392.26	  7
 14	 V 	   784.38	   342.72	  6
 15	 P 	   832.90	   293.19	  5
 16	 I 	   889.44	   244.66	  4
 17	 T 	   939.97	   188.12	  3
 18	 V 	   989.50	   137.60	  2
 19	 R 	        -  	    88.06	  1  _
```

**50. IPI00306400 Kinesin-like protein KIFC1**

| Sequence | Charge | M+H+ | XCorr | DeltaCn |
| --- | --- | --- | --- | --- |
| R.ERTQTLDQENQQLQDQLR.D | 3 | 2244.365 | 4.482 | 0.278 |

**+1 charge**

```
		      B   	       Y
  1	 E 	   130.05	        -  	 18
  2	 R 	   286.15	  2114.06	 17
  3	 T 	   387.20	  1957.96	 16
  4	 Q 	   515.26	  1856.91	 15
  5	 T 	   616.30	  1728.85	 14
  6	 L 	   729.39	  1627.80	 13
  7	 D 	   844.42	  1514.72	 12
  8	 Q 	   972.47	  1399.69	 11
  9	 E 	  1101.52	  1271.63	 10
 10	 N 	  1215.56	  1142.59	  9
 11	 Q 	  1343.62	  1028.55	  8
 12	 Q 	  1471.68	   900.49	  7
 13	 L 	  1584.76	   772.43	  6
 14	 Q 	  1712.82	   659.35	  5
 15	 D 	  1827.85	   531.29	  4
 16	 Q 	  1955.91	   416.26	  3
 17	 L 	  2068.99	   288.20	  2
 18	 R 	        -  	   175.12	  1  _
+2 charge

		      B   	       Y
  1	 E 	    65.53	        -  	 18
  2	 R 	   143.58	  1057.53	 17
  3	 T 	   194.10	   979.48	 16
  4	 Q 	   258.13	   928.96	 15
  5	 T 	   308.66	   864.93	 14
  6	 L 	   365.20	   814.41	 13
  7	 D 	   422.71	   757.86	 12
  8	 Q 	   486.74	   700.35	 11
  9	 E 	   551.26	   636.32	 10
 10	 N 	   608.28	   571.80	  9
 11	 Q 	   672.31	   514.78	  8
 12	 Q 	   736.34	   450.75	  7
 13	 L 	   792.88	   386.72	  6
 14	 Q 	   856.91	   330.18	  5
 15	 D 	   914.43	   266.15	  4
 16	 Q 	   978.46	   208.63	  3
 17	 L 	  1035.00	   144.61	  2
 18	 R 	        -  	    88.06	  1  _
+3 charge

		      B   	       Y
  1	 E 	    44.02	        -  	 18
  2	 R 	    96.06	   705.36	 17
  3	 T 	   129.74	   653.32	 16
  4	 Q 	   172.42	   619.64	 15
  5	 T 	   206.11	   576.96	 14
  6	 L 	   243.80	   543.27	 13
  7	 D 	   282.14	   505.58	 12
  8	 Q 	   324.83	   467.24	 11
  9	 E 	   367.84	   424.55	 10
 10	 N 	   405.86	   381.54	  9
 11	 Q 	   448.54	   343.52	  8
 12	 Q 	   491.23	   300.83	  7
 13	 L 	   528.93	   258.15	  6
 14	 Q 	   571.61	   220.45	  5
 15	 D 	   609.95	   177.77	  4
 16	 Q 	   652.64	   139.43	  3
 17	 L 	   690.33	    96.74	  2
 18	 R 	        -  	    59.04	  1  _
```

**51. IPI00929514 Similar to Cofilin-1**

| Sequence | Charge | M+H+ | XCorr | DeltaCn |
| --- | --- | --- | --- | --- |
| K.KILVSNVDQTIDDPYATFVKMLTVR.T | 3 | 2868.341 | 3.365 | 0.223 |

**+1 charge**

```
		      B   	       Y
  1	 K 	   129.10	        -  	 25
  2	 I 	   242.19	  2738.44	 24
  3	 L 	   355.27	  2625.36	 23
  4	 V 	   454.34	  2512.28	 22
  5	 S 	   541.37	  2413.21	 21
  6	 N 	   655.41	  2326.17	 20
  7	 V 	   754.48	  2212.13	 19
  8	 D 	   869.51	  2113.06	 18
  9	 Q 	   997.57	  1998.04	 17
 10	 T 	  1098.62	  1869.98	 16
 11	 I 	  1211.70	  1768.93	 15
 12	 D 	  1326.73	  1655.85	 14
 13	 D 	  1441.75	  1540.82	 13
 14	 P 	  1538.81	  1425.79	 12
 15	 Y 	  1701.87	  1328.74	 11
 16	 A 	  1772.91	  1165.68	 10
 17	 T 	  1873.95	  1094.64	  9
 18	 F 	  2021.02	   993.59	  8
 19	 V 	  2120.09	   846.52	  7
 20	 K 	  2248.19	   747.45	  6
 21	 M 	  2379.23	   619.36	  5
 22	 L 	  2492.31	   488.32	  4
 23	 T 	  2593.36	   375.24	  3
 24	 V 	  2692.43	   274.19	  2
 25	 R 	        -  	   175.12	  1  _


+2 charge
		      B   	       Y
  1	 K 	    65.05	        -  	 25
  2	 I 	   121.60	  1369.73	 24
  3	 L 	   178.14	  1313.18	 23
  4	 V 	   227.67	  1256.64	 22
  5	 S 	   271.19	  1207.11	 21
  6	 N 	   328.21	  1163.59	 20
  7	 V 	   377.74	  1106.57	 19
  8	 D 	   435.26	  1057.04	 18
  9	 Q 	   499.29	   999.52	 17
 10	 T 	   549.81	   935.49	 16
 11	 I 	   606.35	   884.97	 15
 12	 D 	   663.87	   828.43	 14
 13	 D 	   721.38	   770.91	 13
 14	 P 	   769.91	   713.40	 12
 15	 Y 	   851.44	   664.87	 11
 16	 A 	   886.96	   583.34	 10
 17	 T 	   937.48	   547.82	  9
 18	 F 	  1011.01	   497.30	  8
 19	 V 	  1060.55	   423.77	  7
 20	 K 	  1124.60	   374.23	  6
 21	 M 	  1190.12	   310.18	  5
 22	 L 	  1246.66	   244.66	  4
 23	 T 	  1297.18	   188.12	  3
 24	 V 	  1346.72	   137.60	  2
 25	 R 	        -  	    88.06	  1  _


+3 charge
		      B   	       Y
  1	 K 	    43.71	        -  	 25
  2	 I 	    81.40	   913.49	 24
  3	 L 	   119.09	   875.79	 23
  4	 V 	   152.12	   838.10	 22
  5	 S 	   181.13	   805.07	 21
  6	 N 	   219.14	   776.06	 20
  7	 V 	   252.17	   738.05	 19
  8	 D 	   290.51	   705.03	 18
  9	 Q 	   333.19	   666.68	 17
 10	 T 	   366.88	   624.00	 16
 11	 I 	   404.57	   590.31	 15
 12	 D 	   442.91	   552.62	 14
 13	 D 	   481.26	   514.28	 13
 14	 P 	   513.61	   475.94	 12
 15	 Y 	   567.96	   443.58	 11
 16	 A 	   591.64	   389.23	 10
 17	 T 	   625.32	   365.55	  9
 18	 F 	   674.35	   331.87	  8
 19	 V 	   707.37	   282.85	  7
 20	 K 	   750.07	   249.82	  6
 21	 M 	   793.75	   207.12	  5
 22	 L 	   831.44	   163.44	  4
 23	 T 	   865.12	   125.75	  3
 24	 V 	   898.15	    92.07	  2
 25	 R 	        -  	    59.04	  1  _
```

**52. IPI00855790 zinc finger protein 251**

| Sequence | Charge | M+H+ | XCorr | DeltaCn |
| --- | --- | --- | --- | --- |
| K.ELWVLNLLGAEEPDILK.S | 2 | 1953.268 | 3.029 | 0.234 |

**+1 charge**

```
		      B   	       Y
  1	 E 	   130.05	        -  	 17
  2	 L 	   243.13	  1823.03	 16
  3	 W 	   429.21	  1709.95	 15
  4	 V 	   528.28	  1523.87	 14
  5	 L 	   641.37	  1424.80	 13
  6	 N 	   755.41	  1311.72	 12
  7	 L 	   868.49	  1197.67	 11
  8	 L 	   981.58	  1084.59	 10
  9	 G 	  1038.60	   971.50	  9
 10	 A 	  1109.64	   914.48	  8
 11	 E 	  1238.68	   843.45	  7
 12	 E 	  1367.72	   714.40	  6
 13	 P 	  1464.77	   585.36	  5
 14	 D 	  1579.80	   488.31	  4
 15	 I 	  1692.88	   373.28	  3
 16	 L 	  1805.97	   260.20	  2
 17	 K 	        -  	   147.11	  1  _
+2 charge

		      B   	       Y
  1	 E 	    65.53	        -  	 17
  2	 L 	   122.07	   912.02	 16
  3	 W 	   215.11	   855.48	 15
  4	 V 	   264.64	   762.44	 14
  5	 L 	   321.19	   712.90	 13
  6	 N 	   378.21	   656.36	 12
  7	 L 	   434.75	   599.34	 11
  8	 L 	   491.29	   542.80	 10
  9	 G 	   519.80	   486.26	  9
 10	 A 	   555.32	   457.75	  8
 11	 E 	   619.84	   422.23	  7
 12	 E 	   684.36	   357.71	  6
 13	 P 	   732.89	   293.18	  5
 14	 D 	   790.40	   244.66	  4
 15	 I 	   846.95	   187.14	  3
 16	 L 	   903.49	   130.60	  2
 17	 K 	        -  	    74.06	  1  _
```

**53. IPI00001399 Adherens junction-associated protein 1**

| Sequence | Charge | M+H+ | XCorr | DeltaCn |
| --- | --- | --- | --- | --- |
| R.HLQGDGLSSFDSR.G | 2 | 1419.482 | 2.884 | 0.27 |

**+1 charge**

```
		      B   	       Y
  1	 H 	   138.07	        -  	 13
  2	 L 	   251.15	  1281.61	 12
  3	 Q 	   379.21	  1168.52	 11
  4	 G 	   436.23	  1040.46	 10
  5	 D 	   551.26	   983.44	  9
  6	 G 	   608.28	   868.42	  8
  7	 L 	   721.36	   811.39	  7
  8	 S 	   808.39	   698.31	  6
  9	 S 	   895.43	   611.28	  5
 10	 F 	  1042.50	   524.25	  4
 11	 D 	  1157.52	   377.18	  3
 12	 S 	  1244.55	   262.15	  2
 13	 R 	        -  	   175.12	  1  _
+2 charge

		      B   	       Y
  1	 H 	    69.54	        -  	 13
  2	 L 	   126.08	   641.31	 12
  3	 Q 	   190.11	   584.77	 11
  4	 G 	   218.62	   520.74	 10
  5	 D 	   276.13	   492.23	  9
  6	 G 	   304.64	   434.71	  8
  7	 L 	   361.19	   406.20	  7
  8	 S 	   404.70	   349.66	  6
  9	 S 	   448.22	   306.14	  5
 10	 F 	   521.75	   262.63	  4
 11	 D 	   579.26	   189.09	  3
 12	 S 	   622.78	   131.58	  2
 13	 R 	        -  	    88.06	  1  _
```

**54. IPI00002406 Basal cell adhesion molecule**

| Sequence | Charge | M+H+ | XCorr | DeltaCn |
| --- | --- | --- | --- | --- |
| R.VEDYDAADDVQLSK.T | 2 | 1568.621 | 3.407 | 0.52 |

**+1 charge**

```
		      B   	       Y
  1	 V 	   100.08	        -  	 14
  2	 E 	   229.12	  1468.64	 13
  3	 D 	   344.15	  1339.60	 12
  4	 Y 	   507.21	  1224.57	 11
  5	 D 	   622.24	  1061.51	 10
  6	 A 	   693.27	   946.48	  9
  7	 A 	   764.31	   875.45	  8
  8	 D 	   879.34	   804.41	  7
  9	 D 	   994.36	   689.38	  6
 10	 V 	  1093.43	   574.36	  5
 11	 Q 	  1221.49	   475.29	  4
 12	 L 	  1334.57	   347.23	  3
 13	 S 	  1421.61	   234.14	  2
 14	 K 	        -  	   147.11	  1  _
+2 charge

		      B   	       Y
  1	 V 	    50.54	        -  	 14
  2	 E 	   115.06	   734.83	 13
  3	 D 	   172.58	   670.30	 12
  4	 Y 	   254.11	   612.79	 11
  5	 D 	   311.62	   531.26	 10
  6	 A 	   347.14	   473.75	  9
  7	 A 	   382.66	   438.23	  8
  8	 D 	   440.17	   402.71	  7
  9	 D 	   497.69	   345.20	  6
 10	 V 	   547.22	   287.68	  5
 11	 Q 	   611.25	   238.15	  4
 12	 L 	   667.79	   174.12	  3
 13	 S 	   711.31	   117.58	  2
 14	 K 	        -  	    74.06	  1  _
```

**55. IPI00028911 Dystroglycan**

| Sequence | Charge | M+H+ | XCorr | DeltaCn |
| --- | --- | --- | --- | --- |
| K.LGCSLNQNSVPDIHGVEAPAR.E | 3 | 2235.437 | 4.456 | 0.571 |

**+1 charge**

```
		      B   	       Y
  1	 L 	   114.09	        -  	 21
  2	 G 	   171.11	  2121.15	 20
  3	 C 	   331.28	  2064.13	 19
  4	 S 	   418.31	  1903.96	 18
  5	 L 	   531.39	  1816.93	 17
  6	 N 	   645.44	  1703.85	 16
  7	 Q 	   773.50	  1589.80	 15
  8	 N 	   887.54	  1461.74	 14
  9	 S 	   974.57	  1347.70	 13
 10	 V 	  1073.64	  1260.67	 12
 11	 P 	  1170.69	  1161.60	 11
 12	 D 	  1285.72	  1064.55	 10
 13	 I 	  1398.80	   949.52	  9
 14	 H 	  1535.86	   836.44	  8
 15	 G 	  1592.88	   699.38	  7
 16	 V 	  1691.95	   642.36	  6
 17	 E 	  1820.99	   543.29	  5
 18	 A 	  1892.03	   414.25	  4
 19	 P 	  1989.08	   343.21	  3
 20	 A 	  2060.12	   246.16	  2
 21	 R 	        -  	   175.12	  1  _


+2 charge
		      B   	       Y
  1	 L 	    57.55	        -  	 21
  2	 G 	    86.06	  1061.08	 20
  3	 C 	   166.14	  1032.57	 19
  4	 S 	   209.66	   952.48	 18
  5	 L 	   266.20	   908.97	 17
  6	 N 	   323.22	   852.43	 16
  7	 Q 	   387.25	   795.41	 15
  8	 N 	   444.27	   731.38	 14
  9	 S 	   487.79	   674.35	 13
 10	 V 	   537.32	   630.84	 12
 11	 P 	   585.85	   581.30	 11
 12	 D 	   643.36	   532.78	 10
 13	 I 	   699.91	   475.26	  9
 14	 H 	   768.43	   418.72	  8
 15	 G 	   796.95	   350.19	  7
 16	 V 	   846.48	   321.68	  6
 17	 E 	   911.00	   272.15	  5
 18	 A 	   946.52	   207.63	  4
 19	 P 	   995.05	   172.11	  3
 20	 A 	  1030.56	   123.58	  2
 21	 R 	        -  	    88.06	  1  _
+3 charge

		      B   	       Y
  1	 L 	    38.70	        -  	 21
  2	 G 	    57.71	   707.72	 20
  3	 C 	   111.10	   688.71	 19
  4	 S 	   140.11	   635.33	 18
  5	 L 	   177.80	   606.31	 17
  6	 N 	   215.82	   568.62	 16
  7	 Q 	   258.50	   530.61	 15
  8	 N 	   296.52	   487.92	 14
  9	 S 	   325.53	   449.91	 13
 10	 V 	   358.55	   420.89	 12
 11	 P 	   390.90	   387.87	 11
 12	 D 	   429.24	   355.52	 10
 13	 I 	   466.94	   317.18	  9
 14	 H 	   512.63	   279.48	  8
 15	 G 	   531.63	   233.80	  7
 16	 V 	   564.66	   214.79	  6
 17	 E 	   607.67	   181.77	  5
 18	 A 	   631.35	   138.75	  4
 19	 P 	   663.70	   115.07	  3
 20	 A 	   687.38	    82.72	  2
 21	 R 	        -  	    59.04	  1  _
```

**56. IPI00028347 Isoform 1 of F-box only protein 31**

| Sequence | Charge | M+H+ | XCorr | DeltaCn |
| --- | --- | --- | --- | --- |
| R.IYLPPSRPDDLIKPGLFK.G | 2 | 2070.462 | 3.228 | 0.293 |

```
+1 charge

		      B   	       Y
  1	 I 	   114.09	        -  	 18
  2	 Y 	   277.15	  1956.10	 17
  3	 L 	   390.24	  1793.03	 16
  4	 P 	   487.29	  1679.95	 15
  5	 P 	   584.34	  1582.90	 14
  6	 S 	   671.38	  1485.84	 13
  7	 R 	   827.48	  1398.81	 12
  8	 P 	   924.53	  1242.71	 11
  9	 D 	  1039.56	  1145.66	 10
 10	 D 	  1154.58	  1030.63	  9
 11	 L 	  1267.67	   915.60	  8
 12	 I 	  1380.75	   802.52	  7
 13	 K 	  1508.85	   689.43	  6
 14	 P 	  1605.90	   561.34	  5
 15	 G 	  1662.92	   464.29	  4
 16	 L 	  1776.01	   407.27	  3
 17	 F 	  1923.07	   294.18	  2
 18	 K 	        -  	   147.11	  1  _
+2 charge

		      B   	       Y
  1	 I 	    57.55	        -  	 18
  2	 Y 	   139.08	   978.55	 17
  3	 L 	   195.62	   897.02	 16
  4	 P 	   244.15	   840.48	 15
  5	 P 	   292.68	   791.95	 14
  6	 S 	   336.19	   743.42	 13
  7	 R 	   414.24	   699.91	 12
  8	 P 	   462.77	   621.86	 11
  9	 D 	   520.28	   573.33	 10
 10	 D 	   577.80	   515.82	  9
 11	 L 	   634.34	   458.30	  8
 12	 I 	   690.88	   401.76	  7
 13	 K 	   754.93	   345.22	  6
 14	 P 	   803.45	   281.17	  5
 15	 G 	   831.96	   232.65	  4
 16	 L 	   888.51	   204.14	  3
 17	 F 	   962.04	   147.59	  2
 18	 K 	        -  	    74.06	  1  _
```

**57. IPI00473011 Hemoglobin subunit delta**

| Sequence | Charge | M+H+ | XCorr | DeltaCn |
| --- | --- | --- | --- | --- |
| K.VLGAFSDGLAHLDNLK.G | 2 | 1670.891 | 4.731 | 0.446 |

**+1 charge**

```
		      B   	       Y
  1	 V 	   100.08	        -  	 16
  2	 L 	   213.16	  1570.82	 15
  3	 G 	   270.18	  1457.74	 14
  4	 A 	   341.22	  1400.72	 13
  5	 F 	   488.29	  1329.68	 12
  6	 S 	   575.32	  1182.61	 11
  7	 D 	   690.35	  1095.58	 10
  8	 G 	   747.37	   980.55	  9
  9	 L 	   860.45	   923.53	  8
 10	 A 	   931.49	   810.45	  7
 11	 H 	  1068.55	   739.41	  6
 12	 L 	  1181.63	   602.35	  5
 13	 D 	  1296.66	   489.27	  4
 14	 N 	  1410.70	   374.24	  3
 15	 L 	  1523.79	   260.20	  2
 16	 K 	        -  	   147.11	  1  _
+2 charge

		      B   	       Y
  1	 V 	    50.54	        -  	 16
  2	 L 	   107.08	   785.91	 15
  3	 G 	   135.59	   729.37	 14
  4	 A 	   171.11	   700.86	 13
  5	 F 	   244.65	   665.34	 12
  6	 S 	   288.16	   591.81	 11
  7	 D 	   345.68	   548.29	 10
  8	 G 	   374.19	   490.78	  9
  9	 L 	   430.73	   462.27	  8
 10	 A 	   466.25	   405.73	  7
 11	 H 	   534.78	   370.21	  6
 12	 L 	   591.32	   301.68	  5
 13	 D 	   648.83	   245.14	  4
 14	 N 	   705.85	   187.62	  3
 15	 L 	   762.40	   130.60	  2
 16	 K 	        -  	    74.06	  1  _
```

**58. IPI00297263 Isoform 1 of Protein HEG homolog 1**

| Sequence | Charge | M+H+ | XCorr | DeltaCn |
| --- | --- | --- | --- | --- |
| R.SYSESSSTSSSESLNSSAPR.G | 2 | 2051.026 | 3.948 | 0.502 |

**+1 charge**

```
		      B   	       Y
  1	 S 	    88.04	        -  	 20
  2	 Y 	   251.10	  1962.85	 19
  3	 S 	   338.13	  1799.79	 18
  4	 E 	   467.18	  1712.76	 17
  5	 S 	   554.21	  1583.71	 16
  6	 S 	   641.24	  1496.68	 15
  7	 S 	   728.27	  1409.65	 14
  8	 T 	   829.32	  1322.62	 13
  9	 S 	   916.35	  1221.57	 12
 10	 S 	  1003.39	  1134.54	 11
 11	 S 	  1090.42	  1047.51	 10
 12	 E 	  1219.46	   960.47	  9
 13	 S 	  1306.49	   831.43	  8
 14	 L 	  1419.58	   744.40	  7
 15	 N 	  1533.62	   631.32	  6
 16	 S 	  1620.65	   517.27	  5
 17	 S 	  1707.68	   430.24	  4
 18	 A 	  1778.72	   343.21	  3
 19	 P 	  1875.77	   272.17	  2
 20	 R 	        -  	   175.12	  1  _
+2 charge
		      B   	       Y
  1	 S 	    44.52	        -  	 20
  2	 Y 	   126.05	   981.93	 19
  3	 S 	   169.57	   900.40	 18
  4	 E 	   234.09	   856.88	 17
  5	 S 	   277.61	   792.36	 16
  6	 S 	   321.12	   748.84	 15
  7	 S 	   364.64	   705.33	 14
  8	 T 	   415.16	   661.81	 13
  9	 S 	   458.68	   611.29	 12
 10	 S 	   502.20	   567.77	 11
 11	 S 	   545.71	   524.26	 10
 12	 E 	   610.23	   480.74	  9
 13	 S 	   653.75	   416.22	  8
 14	 L 	   710.29	   372.70	  7
 15	 N 	   767.31	   316.16	  6
 16	 S 	   810.83	   259.14	  5
 17	 S 	   854.35	   215.62	  4
 18	 A 	   889.86	   172.11	  3
 19	 P 	   938.39	   136.59	  2
 20	 R 	        -  	    88.06	  1  _
```

**59. IPI00478278 Isoform 2 of HAUS augmin-like complex subunit 7**

| Sequence | Charge | M+H+ | XCorr | DeltaCn |
| --- | --- | --- | --- | --- |
| K.QSDDWQWASASAK.S | 2 | 1480.52 | 2.81 | 0.243 |

**+1 charge**

```
		      B   	       Y
  1	 Q 	   129.07	        -  	 13
  2	 S 	   216.10	  1351.59	 12
  3	 D 	   331.12	  1264.56	 11
  4	 D 	   446.15	  1149.53	 10
  5	 W 	   632.23	  1034.51	  9
  6	 Q 	   760.29	   848.43	  8
  7	 W 	   946.37	   720.37	  7
  8	 A 	  1017.41	   534.29	  6
  9	 S 	  1104.44	   463.25	  5
 10	 A 	  1175.48	   376.22	  4
 11	 S 	  1262.51	   305.18	  3
 12	 A 	  1333.54	   218.15	  2
 13	 K 	        -  	   147.11	  1  _
+2 charge

		      B   	       Y
  1	 Q 	    65.04	        -  	 13
  2	 S 	   108.55	   676.30	 12
  3	 D 	   166.07	   632.78	 11
  4	 D 	   223.58	   575.27	 10
  5	 W 	   316.62	   517.76	  9
  6	 Q 	   380.65	   424.72	  8
  7	 W 	   473.69	   360.69	  7
  8	 A 	   509.21	   267.65	  6
  9	 S 	   552.72	   232.13	  5
 10	 A 	   588.24	   188.61	  4
 11	 S 	   631.76	   153.09	  3
 12	 A 	   667.28	   109.58	  2
 13	 K 	        -  	    74.06	  1  _
```

**60. IPI00003590 Isoform 1 of Sulfhydryl oxidase 1**

| Sequence | Charge | M+H+ | XCorr | DeltaCn |
| --- | --- | --- | --- | --- |
| R.AAPGQEPPEHMAELQR.N | 2 | 1761.941 | 3.169 | 0.447 |

**+1 charge**

```
		      B   	       Y
  1	 A 	    72.04	        -  	 16
  2	 A 	   143.08	  1689.80	 15
  3	 P 	   240.13	  1618.76	 14
  4	 G 	   297.16	  1521.71	 13
  5	 Q 	   425.21	  1464.69	 12
  6	 E 	   554.26	  1336.63	 11
  7	 P 	   651.31	  1207.59	 10
  8	 P 	   748.36	  1110.54	  9
  9	 E 	   877.41	  1013.48	  8
 10	 H 	  1014.46	   884.44	  7
 11	 M 	  1145.50	   747.38	  6
 12	 A 	  1216.54	   616.34	  5
 13	 E 	  1345.58	   545.30	  4
 14	 L 	  1458.67	   416.26	  3
 15	 Q 	  1586.73	   303.18	  2
 16	 R 	        -  	   175.12	  1  _
+2 charge

		      B   	       Y
  1	 A 	    36.53	        -  	 16
  2	 A 	    72.04	   845.40	 15
  3	 P 	   120.57	   809.89	 14
  4	 G 	   149.08	   761.36	 13
  5	 Q 	   213.11	   732.85	 12
  6	 E 	   277.63	   668.82	 11
  7	 P 	   326.16	   604.30	 10
  8	 P 	   374.68	   555.77	  9
  9	 E 	   439.21	   507.25	  8
 10	 H 	   507.74	   442.72	  7
 11	 M 	   573.26	   374.19	  6
 12	 A 	   608.77	   308.67	  5
 13	 E 	   673.30	   273.16	  4
 14	 L 	   729.84	   208.63	  3
 15	 Q 	   793.87	   152.09	  2
 16	 R 	        -  	    88.06	  1  _
```

**61. IPI00384404 Rheumatoid factor RF-ET9 (Fragment)**

| Sequence | Charge | M+H+ | XCorr | DeltaCn |
| --- | --- | --- | --- | --- |
| - .LVESGGGLVKPGGSLR.L | 2 | 1526.763 | 4.846 | 0.533 |

**+1 charge**

```
		      B   	       Y
  1	 L 	   114.09	        -  	 16
  2	 V 	   213.16	  1412.79	 15
  3	 E 	   342.20	  1313.72	 14
  4	 S 	   429.23	  1184.67	 13
  5	 G 	   486.26	  1097.64	 12
  6	 G 	   543.28	  1040.62	 11
  7	 G 	   600.30	   983.60	 10
  8	 L 	   713.38	   926.58	  9
  9	 V 	   812.45	   813.49	  8
 10	 K 	   940.55	   714.43	  7
 11	 P 	  1037.60	   586.33	  6
 12	 G 	  1094.62	   489.28	  5
 13	 G 	  1151.64	   432.26	  4
 14	 S 	  1238.67	   375.24	  3
 15	 L 	  1351.76	   288.20	  2
 16	 R 	        -  	   175.12	  1  _
+2 charge

		      B   	       Y
  1	 L 	    57.55	        -  	 16
  2	 V 	   107.08	   706.90	 15
  3	 E 	   171.60	   657.36	 14
  4	 S 	   215.12	   592.84	 13
  5	 G 	   243.63	   549.32	 12
  6	 G 	   272.14	   520.81	 11
  7	 G 	   300.65	   492.30	 10
  8	 L 	   357.20	   463.79	  9
  9	 V 	   406.73	   407.25	  8
 10	 K 	   470.78	   357.72	  7
 11	 P 	   519.30	   293.67	  6
 12	 G 	   547.81	   245.14	  5
 13	 G 	   576.32	   216.63	  4
 14	 S 	   619.84	   188.12	  3
 15	 L 	   676.38	   144.61	  2
 16	 R 	        -  	    88.06	  1  _
```

**62. IPI00024292 Low-density lipoprotein receptor-related protein 2**

| Sequence | Charge | M+H+ | XCorr | DeltaCn |
| --- | --- | --- | --- | --- |
| R.DVTIFDKQVQPR.S | 2 | 1446.633 | 2.804 | 0.443 |

**+1 charge**

```
		      B   	       Y
  1	 D 	   116.03	        -  	 12
  2	 V 	   215.10	  1330.75	 11
  3	 T 	   316.15	  1231.68	 10
  4	 I 	   429.23	  1130.63	  9
  5	 F 	   576.30	  1017.55	  8
  6	 D 	   691.33	   870.48	  7
  7	 K 	   819.42	   755.45	  6
  8	 Q 	   947.48	   627.36	  5
  9	 V 	  1046.55	   499.30	  4
 10	 Q 	  1174.61	   400.23	  3
 11	 P 	  1271.66	   272.17	  2
 12	 R 	        -  	   175.12	  1  _
+2 charge

		      B   	       Y
  1	 D 	    58.52	        -  	 12
  2	 V 	   108.05	   665.88	 11
  3	 T 	   158.58	   616.34	 10
  4	 I 	   215.12	   565.82	  9
  5	 F 	   288.66	   509.28	  8
  6	 D 	   346.17	   435.74	  7
  7	 K 	   410.22	   378.23	  6
  8	 Q 	   474.25	   314.18	  5
  9	 V 	   523.78	   250.15	  4
 10	 Q 	   587.81	   200.62	  3
 11	 P 	   636.34	   136.59	  2
 12	 R 	        -  	    88.06	  1  _
```

**63. IPI00420108 Dihydrolipoyllysine-residue succinyltransferase component of 2-oxoglutarate dehydrogenase complex, mitochondrial**

| Sequence | Charge | M+H+ | XCorr | DeltaCn |
| --- | --- | --- | --- | --- |
| K.TPAFAESVTEGDVR.W | 2 | 1479.574 | 2.877 | 0.345 |

**+1 charge**

```
		      B   	       Y
  1	 T 	   102.05	        -  	 14
  2	 P 	   199.11	  1377.66	 13
  3	 A 	   270.14	  1280.61	 12
  4	 F 	   417.21	  1209.57	 11
  5	 A 	   488.25	  1062.51	 10
  6	 E 	   617.29	   991.47	  9
  7	 S 	   704.32	   862.43	  8
  8	 V 	   803.39	   775.39	  7
  9	 T 	   904.44	   676.33	  6
 10	 E 	  1033.48	   575.28	  5
 11	 G 	  1090.51	   446.24	  4
 12	 D 	  1205.53	   389.21	  3
 13	 V 	  1304.60	   274.19	  2
 14	 R 	        -  	   175.12	  1  _
+2 charge

		      B   	       Y
  1	 T 	    51.53	        -  	 14
  2	 P 	   100.06	   689.34	 13
  3	 A 	   135.58	   640.81	 12
  4	 F 	   209.11	   605.29	 11
  5	 A 	   244.63	   531.76	 10
  6	 E 	   309.15	   496.24	  9
  7	 S 	   352.67	   431.72	  8
  8	 V 	   402.20	   388.20	  7
  9	 T 	   452.72	   338.67	  6
 10	 E 	   517.25	   288.14	  5
 11	 G 	   545.76	   223.62	  4
 12	 D 	   603.27	   195.11	  3
 13	 V 	   652.80	   137.60	  2
 14	 R 	        -  	    88.06	  1  _
```

**64. IPI00100796 Charged multivesicular body protein 5**

| Sequence | Charge | M+H+ | XCorr | DeltaCn |
| --- | --- | --- | --- | --- |
| K.APPPSLTDCIGTVDSR.A | 2 | 1686.839 | 3.266 | 0.595 |

**+1 charge**

```
		      B   	       Y
  1	 A 	    72.04	        -  	 16
  2	 P 	   169.10	  1614.91	 15
  3	 P 	   266.15	  1517.86	 14
  4	 P 	   363.20	  1420.81	 13
  5	 S 	   450.23	  1323.76	 12
  6	 L 	   563.32	  1236.72	 11
  7	 T 	   664.37	  1123.64	 10
  8	 D 	   779.39	  1022.59	  9
  9	 C 	   939.56	   907.56	  8
 10	 I 	  1052.64	   747.40	  7
 11	 G 	  1109.66	   634.32	  6
 12	 T 	  1210.71	   577.29	  5
 13	 V 	  1309.78	   476.25	  4
 14	 D 	  1424.81	   377.18	  3
 15	 S 	  1511.84	   262.15	  2
 16	 R 	        -  	   175.12	  1  _
+2 charge

		      B   	       Y
  1	 A 	    36.53	        -  	 16
  2	 P 	    85.05	   807.96	 15
  3	 P 	   133.58	   759.43	 14
  4	 P 	   182.10	   710.91	 13
  5	 S 	   225.62	   662.38	 12
  6	 L 	   282.16	   618.87	 11
  7	 T 	   332.69	   562.32	 10
  8	 D 	   390.20	   511.80	  9
  9	 C 	   470.28	   454.29	  8
 10	 I 	   526.82	   374.20	  7
 11	 G 	   555.34	   317.66	  6
 12	 T 	   605.86	   289.15	  5
 13	 V 	   655.39	   238.63	  4
 14	 D 	   712.91	   189.09	  3
 15	 S 	   756.42	   131.58	  2
 16	 R 	        -  	    88.06	  1  _
```

**65. IPI00009865 Keratin, type I cytoskeletal 10**

| Sequence | Charge | M+H+ | XCorr | DeltaCn |
| --- | --- | --- | --- | --- |
| K.GSLGGGFSSGGFSGGSFSR.G | 2 | 1708.77 | 4.377 | 0.54 |

**+1 charge**

```
		      B   	       Y
  1	 G 	    58.03	        -  	 19
  2	 S 	   145.06	  1650.75	 18
  3	 L 	   258.14	  1563.72	 17
  4	 G 	   315.17	  1450.63	 16
  5	 G 	   372.19	  1393.61	 15
  6	 G 	   429.21	  1336.59	 14
  7	 F 	   576.28	  1279.57	 13
  8	 S 	   663.31	  1132.50	 12
  9	 S 	   750.34	  1045.47	 11
 10	 G 	   807.36	   958.44	 10
 11	 G 	   864.38	   901.42	  9
 12	 F 	  1011.45	   844.39	  8
 13	 S 	  1098.49	   697.33	  7
 14	 G 	  1155.51	   610.29	  6
 15	 G 	  1212.53	   553.27	  5
 16	 S 	  1299.56	   496.25	  4
 17	 F 	  1446.63	   409.22	  3
 18	 S 	  1533.66	   262.15	  2
 19	 R 	        -  	   175.12	  1  _
+2 charge

		      B   	       Y
  1	 G 	    29.52	        -  	 19
  2	 S 	    73.03	   825.88	 18
  3	 L 	   129.58	   782.36	 17
  4	 G 	   158.09	   725.82	 16
  5	 G 	   186.60	   697.31	 15
  6	 G 	   215.11	   668.80	 14
  7	 F 	   288.64	   640.29	 13
  8	 S 	   332.16	   566.75	 12
  9	 S 	   375.67	   523.24	 11
 10	 G 	   404.19	   479.72	 10
 11	 G 	   432.70	   451.21	  9
 12	 F 	   506.23	   422.70	  8
 13	 S 	   549.75	   349.17	  7
 14	 G 	   578.26	   305.65	  6
 15	 G 	   606.77	   277.14	  5
 16	 S 	   650.28	   248.63	  4
 17	 F 	   723.82	   205.11	  3
 18	 S 	   767.33	   131.58	  2
 19	 R 	        -  	    88.06	  1  _
```

**66. IPI00795918 Isoform 1 of Neural cell adhesion molecule 1**

| Sequence | Charge | M+H+ | XCorr | DeltaCn |
| --- | --- | --- | --- | --- |
| K.FFLCQVAGDAK.D | 2 | 1256.426 | 3.185 | 0.394 |

**+1 charge**

```
		      B   	       Y
  1	 F 	   148.08	        -  	 11
  2	 F 	   295.14	  1108.68	 10
  3	 L 	   408.23	   961.61	  9
  4	 C 	   568.39	   848.53	  8
  5	 Q 	   696.45	   688.36	  7
  6	 V 	   795.52	   560.30	  6
  7	 A 	   866.56	   461.24	  5
  8	 G 	   923.58	   390.20	  4
  9	 D 	  1038.61	   333.18	  3
 10	 A 	  1109.64	   218.15	  2
 11	 K 	        -  	   147.11	  1  _
+2 charge
		      B   	       Y
  1	 F 	    74.54	        -  	 11
  2	 F 	   148.08	   554.84	 10
  3	 L 	   204.62	   481.31	  9
  4	 C 	   284.70	   424.77	  8
  5	 Q 	   348.73	   344.68	  7
  6	 V 	   398.26	   280.66	  6
  7	 A 	   433.78	   231.12	  5
  8	 G 	   462.29	   195.60	  4
  9	 D 	   519.81	   167.09	  3
 10	 A 	   555.33	   109.58	  2
 11	 K 	        -  	    74.06	  1  _
```

**67. IPI00218407 Fructose-bisphosphate aldolase B**

| Sequence | Charge | M+H+ | XCorr | DeltaCn |
| --- | --- | --- | --- | --- |
| K.ELSEIAQSIVANGK.G | 2 | 1459.627 | 4.641 | 0.537 |

**+1 charge**

```
		      B   	       Y
  1	 E 	   130.05	        -  	 14
  2	 L 	   243.13	  1329.74	 13
  3	 S 	   330.17	  1216.65	 12
  4	 E 	   459.21	  1129.62	 11
  5	 I 	   572.29	  1000.58	 10
  6	 A 	   643.33	   887.49	  9
  7	 Q 	   771.39	   816.46	  8
  8	 S 	   858.42	   688.40	  7
  9	 I 	   971.50	   601.37	  6
 10	 V 	  1070.57	   488.28	  5
 11	 A 	  1141.61	   389.21	  4
 12	 N 	  1255.65	   318.18	  3
 13	 G 	  1312.67	   204.13	  2
 14	 K 	        -  	   147.11	  1  _
+2 charge

		      B   	       Y
  1	 E 	    65.53	        -  	 14
  2	 L 	   122.07	   665.37	 13
  3	 S 	   165.59	   608.83	 12
  4	 E 	   230.11	   565.31	 11
  5	 I 	   286.65	   500.79	 10
  6	 A 	   322.17	   444.25	  9
  7	 Q 	   386.20	   408.73	  8
  8	 S 	   429.71	   344.70	  7
  9	 I 	   486.26	   301.19	  6
 10	 V 	   535.79	   244.64	  5
 11	 A 	   571.31	   195.11	  4
 12	 N 	   628.33	   159.59	  3
 13	 G 	   656.84	   102.57	  2
 14	 K 	        -  	    74.06	  1  _
```

**68. IPI00002441 Syndecan-1**

| Sequence | Charge | M+H+ | XCorr | DeltaCn |
| --- | --- | --- | --- | --- |
| K.EGEAVVLPEVEPGLTAR.E | 2 | 1766.974 | 3.367 | 0.429 |

**+1 charge**

```
		      B   	       Y
  1	 E 	   130.05	        -  	 17
  2	 G 	   187.07	  1636.89	 16
  3	 E 	   316.11	  1579.87	 15
  4	 A 	   387.15	  1450.83	 14
  5	 V 	   486.22	  1379.79	 13
  6	 V 	   585.29	  1280.72	 12
  7	 L 	   698.37	  1181.65	 11
  8	 P 	   795.42	  1068.57	 10
  9	 E 	   924.47	   971.52	  9
 10	 V 	  1023.54	   842.47	  8
 11	 E 	  1152.58	   743.40	  7
 12	 P 	  1249.63	   614.36	  6
 13	 G 	  1306.65	   517.31	  5
 14	 L 	  1419.74	   460.29	  4
 15	 T 	  1520.78	   347.20	  3
 16	 A 	  1591.82	   246.16	  2
 17	 R 	        -  	   175.12	  1  _
+2 charge

		      B   	       Y
  1	 E 	    65.53	        -  	 17
  2	 G 	    94.04	   818.95	 16
  3	 E 	   158.56	   790.44	 15
  4	 A 	   194.08	   725.92	 14
  5	 V 	   243.61	   690.40	 13
  6	 V 	   293.15	   640.86	 12
  7	 L 	   349.69	   591.33	 11
  8	 P 	   398.22	   534.79	 10
  9	 E 	   462.74	   486.26	  9
 10	 V 	   512.27	   421.74	  8
 11	 E 	   576.79	   372.21	  7
 12	 P 	   625.32	   307.68	  6
 13	 G 	   653.83	   259.16	  5
 14	 L 	   710.37	   230.65	  4
 15	 T 	   760.90	   174.11	  3
 16	 A 	   796.41	   123.58	  2
 17	 R 	        -  	    88.06	  1  _
```

**69. IPI00025276 Isoform XB of Tenascin-X**

| Sequence | Charge | M+H+ | XCorr | DeltaCn |
| --- | --- | --- | --- | --- |
| R.TLCSLHGVFDLSR.C | 2 | 1505.694 | 2.922 | 0.352 |

**+1 charge**

```
		      B   	       Y
  1	 T 	   102.05	        -  	 13
  2	 L 	   215.14	  1403.84	 12
  3	 C 	   375.30	  1290.76	 11
  4	 S 	   462.34	  1130.60	 10
  5	 L 	   575.42	  1043.56	  9
  6	 H 	   712.48	   930.48	  8
  7	 G 	   769.50	   793.42	  7
  8	 V 	   868.57	   736.40	  6
  9	 F 	  1015.64	   637.33	  5
 10	 D 	  1130.66	   490.26	  4
 11	 L 	  1243.75	   375.24	  3
 12	 S 	  1330.78	   262.15	  2
 13	 R 	        -  	   175.12	  1  _
+2 charge

		      B   	       Y
  1	 T 	    51.53	        -  	 13
  2	 L 	   108.07	   702.43	 12
  3	 C 	   188.16	   645.88	 11
  4	 S 	   231.67	   565.80	 10
  5	 L 	   288.21	   522.29	  9
  6	 H 	   356.74	   465.74	  8
  7	 G 	   385.25	   397.21	  7
  8	 V 	   434.79	   368.70	  6
  9	 F 	   508.32	   319.17	  5
 10	 D 	   565.84	   245.63	  4
 11	 L 	   622.38	   188.12	  3
 12	 S 	   665.89	   131.58	  2
 13	 R 	        -  	    88.06	  1  _
```

**70. IPI00011564 Syndecan-4**

| Sequence | Charge | M+H+ | XCorr | DeltaCn |
| --- | --- | --- | --- | --- |
| R.ETEVIDPQDLLEGR.Y | 2 | 1614.736 | 3.682 | 0.41 |

**+1 charge**

```
		      B   	       Y
  1	 E 	   130.05	        -  	 14
  2	 T 	   231.10	  1484.76	 13
  3	 E 	   360.14	  1383.71	 12
  4	 V 	   459.21	  1254.67	 11
  5	 I 	   572.29	  1155.60	 10
  6	 D 	   687.32	  1042.52	  9
  7	 P 	   784.37	   927.49	  8
  8	 Q 	   912.43	   830.44	  7
  9	 D 	  1027.46	   702.38	  6
 10	 L 	  1140.54	   587.35	  5
 11	 L 	  1253.63	   474.27	  4
 12	 E 	  1382.67	   361.18	  3
 13	 G 	  1439.69	   232.14	  2
 14	 R 	        -  	   175.12	  1  _
+2 charge

		      B   	       Y
  1	 E 	    65.53	        -  	 14
  2	 T 	   116.05	   742.88	 13
  3	 E 	   180.57	   692.36	 12
  4	 V 	   230.11	   627.84	 11
  5	 I 	   286.65	   578.30	 10
  6	 D 	   344.16	   521.76	  9
  7	 P 	   392.69	   464.25	  8
  8	 Q 	   456.72	   415.72	  7
  9	 D 	   514.23	   351.69	  6
 10	 L 	   570.77	   294.18	  5
 11	 L 	   627.32	   237.64	  4
 12	 E 	   691.84	   181.10	  3
 13	 G 	   720.35	   116.57	  2
 14	 R 	        -  	    88.06	  1  _
```

**71. IPI00289983 Isoform 2 of Prostatic acid phosphatase**

| Sequence | Charge | M+H+ | XCorr | DeltaCn |
| --- | --- | --- | --- | --- |
| R.LQGGVLVNEILNHMK.R | 2 | 1665.982 | 3.46 | 0.554 |

**+1 charge**

```
		      B   	       Y
  1	 L 	   114.09	        -  	 15
  2	 Q 	   242.15	  1551.83	 14
  3	 G 	   299.17	  1423.77	 13
  4	 G 	   356.19	  1366.75	 12
  5	 V 	   455.26	  1309.73	 11
  6	 L 	   568.35	  1210.66	 10
  7	 V 	   667.41	  1097.58	  9
  8	 N 	   781.46	   998.51	  8
  9	 E 	   910.50	   884.47	  7
 10	 I 	  1023.58	   755.42	  6
 11	 L 	  1136.67	   642.34	  5
 12	 N 	  1250.71	   529.26	  4
 13	 H 	  1387.77	   415.21	  3
 14	 M 	  1518.81	   278.15	  2
 15	 K 	        -  	   147.11	  1  _
+2 charge

		      B   	       Y
  1	 L 	    57.55	        -  	 15
  2	 Q 	   121.58	   776.42	 14
  3	 G 	   150.09	   712.39	 13
  4	 G 	   178.60	   683.88	 12
  5	 V 	   228.13	   655.37	 11
  6	 L 	   284.68	   605.83	 10
  7	 V 	   334.21	   549.29	  9
  8	 N 	   391.23	   499.76	  8
  9	 E 	   455.75	   442.74	  7
 10	 I 	   512.30	   378.22	  6
 11	 L 	   568.84	   321.67	  5
 12	 N 	   625.86	   265.13	  4
 13	 H 	   694.39	   208.11	  3
 14	 M 	   759.91	   139.58	  2
 15	 K 	        -  	    74.06	  1  _
```

**72. IPI00299024 Isoform 1 of Brain acid soluble protein 1**

| Sequence | Charge | M+H+ | XCorr | DeltaCn |
| --- | --- | --- | --- | --- |
| K.AEPPKAPEQEQAAPGPAAGGEAPK.A | 3 | 2299.482 | 4.018 | 0.434 |

**+1 charge**

```
		      B   	       Y
  1	 A 	    72.04	        -  	 24
  2	 E 	   201.09	  2227.10	 23
  3	 P 	   298.14	  2098.06	 22
  4	 P 	   395.19	  2001.00	 21
  5	 K 	   523.29	  1903.95	 20
  6	 A 	   594.32	  1775.86	 19
  7	 P 	   691.38	  1704.82	 18
  8	 E 	   820.42	  1607.77	 17
  9	 Q 	   948.48	  1478.72	 16
 10	 E 	  1077.52	  1350.66	 15
 11	 Q 	  1205.58	  1221.62	 14
 12	 A 	  1276.62	  1093.56	 13
 13	 A 	  1347.65	  1022.53	 12
 14	 P 	  1444.71	   951.49	 11
 15	 G 	  1501.73	   854.44	 10
 16	 P 	  1598.78	   797.42	  9
 17	 A 	  1669.82	   700.36	  8
 18	 A 	  1740.86	   629.33	  7
 19	 G 	  1797.88	   558.29	  6
 20	 G 	  1854.90	   501.27	  5
 21	 E 	  1983.94	   444.25	  4
 22	 A 	  2054.98	   315.20	  3
 23	 P 	  2152.03	   244.17	  2
 24	 K 	        -  	   147.11	  1  _


+2 charge

		      B   	       Y
  1	 A 	    36.53	        -  	 24
  2	 E 	   101.05	  1114.05	 23
  3	 P 	   149.57	  1049.53	 22
  4	 P 	   198.10	  1001.01	 21
  5	 K 	   262.15	   952.48	 20
  6	 A 	   297.67	   888.43	 19
  7	 P 	   346.19	   852.91	 18
  8	 E 	   410.71	   804.39	 17
  9	 Q 	   474.74	   739.87	 16
 10	 E 	   539.26	   675.84	 15
 11	 Q 	   603.29	   611.31	 14
 12	 A 	   638.81	   547.29	 13
 13	 A 	   674.33	   511.77	 12
 14	 P 	   722.86	   476.25	 11
 15	 G 	   751.37	   427.72	 10
 16	 P 	   799.89	   399.21	  9
 17	 A 	   835.41	   350.68	  8
 18	 A 	   870.93	   315.17	  7
 19	 G 	   899.44	   279.65	  6
 20	 G 	   927.95	   251.14	  5
 21	 E 	   992.47	   222.63	  4
 22	 A 	  1027.99	   158.10	  3
 23	 P 	  1076.52	   122.59	  2
 24	 K 	        -  	    74.06	  1  _


+3 charge

		      B   	       Y
  1	 A 	    24.69	        -  	 24
  2	 E 	    67.70	   743.04	 23
  3	 P 	   100.05	   700.02	 22
  4	 P 	   132.40	   667.67	 21
  5	 K 	   175.10	   635.32	 20
  6	 A 	   198.78	   592.62	 19
  7	 P 	   231.13	   568.94	 18
  8	 E 	   274.14	   536.59	 17
  9	 Q 	   316.83	   493.58	 16
 10	 E 	   359.85	   450.89	 15
 11	 Q 	   402.53	   407.88	 14
 12	 A 	   426.21	   365.19	 13
 13	 A 	   449.89	   341.51	 12
 14	 P 	   482.24	   317.83	 11
 15	 G 	   501.25	   285.48	 10
 16	 P 	   533.60	   266.48	  9
 17	 A 	   557.28	   234.13	  8
 18	 A 	   580.96	   210.45	  7
 19	 G 	   599.96	   186.77	  6
 20	 G 	   618.97	   167.76	  5
 21	 E 	   661.99	   148.75	  4
 22	 A 	   685.66	   105.74	  3
 23	 P 	   718.02	    82.06	  2
 24	 K 	        -  	    49.71	  1  _
```

**73. IPI00453473 Histone H4**

| Sequence | Charge | M+H+ | XCorr | DeltaCn |
| --- | --- | --- | --- | --- |
| R.DNIQGITKPAIR.R | 2 | 1326.527 | 3.169 | 0.213 |

**+1 charge**

```
		      B   	       Y
  1	 D 	   116.03	        -  	 12
  2	 N 	   230.08	  1210.73	 11
  3	 I 	   343.16	  1096.68	 10
  4	 Q 	   471.22	   983.60	  9
  5	 G 	   528.24	   855.54	  8
  6	 I 	   641.33	   798.52	  7
  7	 T 	   742.37	   685.44	  6
  8	 K 	   870.47	   584.39	  5
  9	 P 	   967.52	   456.29	  4
 10	 A 	  1038.56	   359.24	  3
 11	 I 	  1151.64	   288.20	  2
 12	 R 	        -  	   175.12	  1  _
+2 charge

		      B   	       Y
  1	 D 	    58.52	        -  	 12
  2	 N 	   115.54	   605.87	 11
  3	 I 	   172.08	   548.85	 10
  4	 Q 	   236.11	   492.30	  9
  5	 G 	   264.62	   428.27	  8
  6	 I 	   321.17	   399.76	  7
  7	 T 	   371.69	   343.22	  6
  8	 K 	   435.74	   292.70	  5
  9	 P 	   484.26	   228.65	  4
 10	 A 	   519.78	   180.12	  3
 11	 I 	   576.32	   144.61	  2
 12	 R 	        -  	    88.06	  1  _
```

**74. IPI00641251 CD320 antigen**

| Sequence | Charge | M+H+ | XCorr | DeltaCn |
| --- | --- | --- | --- | --- |
| R.LACLAGELR.C | 2 | 1003.171 | 3.141 | 0.331 |

**+1 charge**

```
		      B   	       Y
  1	 L 	   114.09	        -  	  9
  2	 A 	   185.13	   889.59	  8
  3	 C 	   345.29	   818.55	  7
  4	 L 	   458.38	   658.39	  6
  5	 A 	   529.41	   545.30	  5
  6	 G 	   586.44	   474.27	  4
  7	 E 	   715.48	   417.25	  3
  8	 L 	   828.56	   288.20	  2
  9	 R 	        -  	   175.12	  1  _
+2 charge

		      B   	       Y
  1	 L 	    57.55	        -  	  9
  2	 A 	    93.07	   445.30	  8
  3	 C 	   173.15	   409.78	  7
  4	 L 	   229.69	   329.70	  6
  5	 A 	   265.21	   273.16	  5
  6	 G 	   293.72	   237.64	  4
  7	 E 	   358.24	   209.13	  3
  8	 L 	   414.79	   144.61	  2
  9	 R 	        -  	    88.06	  1  _
```

**75.IPI00953689 Alpha-2-HS-glycoprotein**

| Sequence | Charge | M+H+ | XCorr | DeltaCn |
| --- | --- | --- | --- | --- |
| R.TVVQPSVGAAAGPVVPPCPGR.I | 2 | 2017.312 | 4.769 | 0.395 |

**+1 charge**

```
		      B   	       Y
  1	 T 	   102.05	        -  	 21
  2	 V 	   201.12	  1915.16	 20
  3	 V 	   300.19	  1816.09	 19
  4	 Q 	   428.25	  1717.02	 18
  5	 P 	   525.30	  1588.96	 17
  6	 S 	   612.34	  1491.91	 16
  7	 V 	   711.40	  1404.88	 15
  8	 G 	   768.43	  1305.81	 14
  9	 A 	   839.46	  1248.79	 13
 10	 A 	   910.50	  1177.75	 12
 11	 A 	   981.54	  1106.71	 11
 12	 G 	  1038.56	  1035.67	 10
 13	 P 	  1135.61	   978.65	  9
 14	 V 	  1234.68	   881.60	  8
 15	 V 	  1333.75	   782.53	  7
 16	 P 	  1430.80	   683.46	  6
 17	 P 	  1527.85	   586.41	  5
 18	 C 	  1688.02	   489.36	  4
 19	 P 	  1785.07	   329.19	  3
 20	 G 	  1842.09	   232.14	  2
 21	 R 	        -  	   175.12	  1  _
+2 charge
		      B   	       Y
  1	 T 	    51.53	        -  	 21
  2	 V 	   101.07	   958.08	 20
  3	 V 	   150.60	   908.55	 19
  4	 Q 	   214.63	   859.01	 18
  5	 P 	   263.16	   794.98	 17
  6	 S 	   306.67	   746.46	 16
  7	 V 	   356.21	   702.94	 15
  8	 G 	   384.72	   653.41	 14
  9	 A 	   420.23	   624.90	 13
 10	 A 	   455.75	   589.38	 12
 11	 A 	   491.27	   553.86	 11
 12	 G 	   519.78	   518.34	 10
 13	 P 	   568.31	   489.83	  9
 14	 V 	   617.84	   441.30	  8
 15	 V 	   667.38	   391.77	  7
 16	 P 	   715.90	   342.24	  6
 17	 P 	   764.43	   293.71	  5
 18	 C 	   844.51	   245.18	  4
 19	 P 	   893.04	   165.10	  3
 20	 G 	   921.55	   116.57	  2
 21	 R 	        -  	    88.06	  1  _
```

**76. IPI00002435 CD27 antigen**

| Sequence | Charge | M+H+ | XCorr | DeltaCn |
| --- | --- | --- | --- | --- |
| K.LCCQMCEPGTFLVK.D | 2 | 1744.027 | 4.148 | 0.485 |

```
+1 charge

		      B   	       Y
  1	 L 	   114.09	        -  	 14
  2	 C 	   274.26	  1630.09	 13
  3	 C 	   434.42	  1469.93	 12
  4	 Q 	   562.48	  1309.76	 11
  5	 M 	   693.52	  1181.70	 10
  6	 C 	   853.69	  1050.66	  9
  7	 E 	   982.73	   890.50	  8
  8	 P 	  1079.78	   761.46	  7
  9	 G 	  1136.80	   664.40	  6
 10	 T 	  1237.85	   607.38	  5
 11	 F 	  1384.92	   506.33	  4
 12	 L 	  1498.00	   359.27	  3
 13	 V 	  1597.07	   246.18	  2
 14	 K 	        -  	   147.11	  1  _
+2 charge

		      B   	       Y
  1	 L 	    57.55	        -  	 14
  2	 C 	   137.63	   815.55	 13
  3	 C 	   217.71	   735.47	 12
  4	 Q 	   281.74	   655.38	 11
  5	 M 	   347.26	   591.36	 10
  6	 C 	   427.35	   525.84	  9
  7	 E 	   491.87	   445.75	  8
  8	 P 	   540.39	   381.23	  7
  9	 G 	   568.91	   332.71	  6
 10	 T 	   619.43	   304.19	  5
 11	 F 	   692.96	   253.67	  4
 12	 L 	   749.51	   180.14	  3
 13	 V 	   799.04	   123.59	  2
 14	 K 	        -  	    74.06	  1  _
```

**77. IPI00651692 Stromal cell-derived factor 1 theta**

| Sequence | Charge | M+H+ | XCorr | DeltaCn |
| --- | --- | --- | --- | --- |
| K.ILNTPNCALQIVAR.L | 2 | 1583.851 | 4.231 | 0.476 |

**+1 charge**

```
		      B   	       Y
  1	 I 	   114.09	        -  	 14
  2	 L 	   227.18	  1469.92	 13
  3	 N 	   341.22	  1356.84	 12
  4	 T 	   442.27	  1242.80	 11
  5	 P 	   539.32	  1141.75	 10
  6	 N 	   653.36	  1044.70	  9
  7	 C 	   813.53	   930.65	  8
  8	 A 	   884.56	   770.49	  7
  9	 L 	   997.65	   699.45	  6
 10	 Q 	  1125.71	   586.37	  5
 11	 I 	  1238.79	   458.31	  4
 12	 V 	  1337.86	   345.22	  3
 13	 A 	  1408.90	   246.16	  2
 14	 R 	        -  	   175.12	  1  _
+2 charge

		      B   	       Y
  1	 I 	    57.55	        -  	 14
  2	 L 	   114.09	   735.47	 13
  3	 N 	   171.11	   678.92	 12
  4	 T 	   221.64	   621.90	 11
  5	 P 	   270.16	   571.38	 10
  6	 N 	   327.18	   522.85	  9
  7	 C 	   407.27	   465.83	  8
  8	 A 	   442.79	   385.75	  7
  9	 L 	   499.33	   350.23	  6
 10	 Q 	   563.36	   293.69	  5
 11	 I 	   619.90	   229.66	  4
 12	 V 	   669.43	   173.12	  3
 13	 A 	   704.95	   123.58	  2
 14	 R 	        -  	    88.06	  1  _
```

**78. IPI00019157 Chondroitin sulfate proteoglycan 4**

| Sequence | Charge | M+H+ | XCorr | DeltaCn |
| --- | --- | --- | --- | --- |
| R.GSLLLGGLDAEASR.H | 2 | 1359.511 | 3.284 | 0.479 |

**+1 charge**

```
		      B   	       Y
  1	 G 	    58.03	        -  	 14
  2	 S 	   145.06	  1301.71	 13
  3	 L 	   258.14	  1214.67	 12
  4	 L 	   371.23	  1101.59	 11
  5	 L 	   484.31	   988.51	 10
  6	 G 	   541.33	   875.42	  9
  7	 G 	   598.36	   818.40	  8
  8	 L 	   711.44	   761.38	  7
  9	 D 	   826.47	   648.29	  6
 10	 A 	   897.50	   533.27	  5
 11	 E 	  1026.55	   462.23	  4
 12	 A 	  1097.58	   333.19	  3
 13	 S 	  1184.62	   262.15	  2
 14	 R 	        -  	   175.12	  1  _
+2 charge

		      B   	       Y
  1	 G 	    29.52	        -  	 14
  2	 S 	    73.03	   651.36	 13
  3	 L 	   129.58	   607.84	 12
  4	 L 	   186.12	   551.30	 11
  5	 L 	   242.66	   494.76	 10
  6	 G 	   271.17	   438.21	  9
  7	 G 	   299.68	   409.70	  8
  8	 L 	   356.22	   381.19	  7
  9	 D 	   413.74	   324.65	  6
 10	 A 	   449.26	   267.14	  5
 11	 E 	   513.78	   231.62	  4
 12	 A 	   549.30	   167.10	  3
 13	 S 	   592.81	   131.58	  2
 14	 R 	        -  	    88.06	  1  _
```

**79. IPI00306543 Growth/differentiation factor 15**

| Sequence | Charge | M+H+ | XCorr | DeltaCn |
| --- | --- | --- | --- | --- |
| K.TDTGVSLQTYDDLLAK.D | 2 | 1740.89 | 4.086 | 0.489 |

**+1 charge**

```
		      B   	       Y
  1	 T 	   102.05	        -  	 16
  2	 D 	   217.08	  1638.82	 15
  3	 T 	   318.13	  1523.80	 14
  4	 G 	   375.15	  1422.75	 13
  5	 V 	   474.22	  1365.73	 12
  6	 S 	   561.25	  1266.66	 11
  7	 L 	   674.34	  1179.63	 10
  8	 Q 	   802.39	  1066.54	  9
  9	 T 	   903.44	   938.48	  8
 10	 Y 	  1066.51	   837.44	  7
 11	 D 	  1181.53	   674.37	  6
 12	 D 	  1296.56	   559.34	  5
 13	 L 	  1409.64	   444.32	  4
 14	 L 	  1522.73	   331.23	  3
 15	 A 	  1593.76	   218.15	  2
 16	 K 	        -  	   147.11	  1  _
+2 charge

		      B   	       Y
  1	 T 	    51.53	        -  	 16
  2	 D 	   109.04	   819.91	 15
  3	 T 	   159.57	   762.40	 14
  4	 G 	   188.08	   711.88	 13
  5	 V 	   237.61	   683.37	 12
  6	 S 	   281.13	   633.83	 11
  7	 L 	   337.67	   590.32	 10
  8	 Q 	   401.70	   533.77	  9
  9	 T 	   452.22	   469.75	  8
 10	 Y 	   533.76	   419.22	  7
 11	 D 	   591.27	   337.69	  6
 12	 D 	   648.78	   280.18	  5
 13	 L 	   705.33	   222.66	  4
 14	 L 	   761.87	   166.12	  3
 15	 A 	   797.39	   109.58	  2
 16	 K 	        -  	    74.06	  1  _
```

**80. IPI00027827 Extracellular superoxide dismutase [Cu-Zn]**

| Sequence | Charge | M+H+ | XCorr | DeltaCn |
| --- | --- | --- | --- | --- |
| K.VTEIWQEVMQR.R | 2 | 1419.631 | 2.991 | 0.408 |

**+1 charge**

```
		      B   	       Y
  1	 V 	   100.08	        -  	 11
  2	 T 	   201.12	  1319.64	 10
  3	 E 	   330.17	  1218.59	  9
  4	 I 	   443.25	  1089.55	  8
  5	 W 	   629.33	   976.47	  7
  6	 Q 	   757.39	   790.39	  6
  7	 E 	   886.43	   662.33	  5
  8	 V 	   985.50	   533.29	  4
  9	 M 	  1116.54	   434.22	  3
 10	 Q 	  1244.60	   303.18	  2
 11	 R 	        -  	   175.12	  1  _
+2 charge

		      B   	       Y
  1	 V 	    50.54	        -  	 11
  2	 T 	   101.07	   660.32	 10
  3	 E 	   165.59	   609.80	  9
  4	 I 	   222.13	   545.28	  8
  5	 W 	   315.17	   488.74	  7
  6	 Q 	   379.20	   395.70	  6
  7	 E 	   443.72	   331.67	  5
  8	 V 	   493.25	   267.15	  4
  9	 M 	   558.77	   217.61	  3
 10	 Q 	   622.80	   152.09	  2
 11	 R 	        -  	    88.06	  1  _
```

**81.** IPI00414888 Isoform 2 of ICOS ligand

| Sequence | Charge | M+H+ | XCorr | DeltaCn |
| --- | --- | --- | --- | --- |
| R.GLYDVVSVLR.I | 2 | 1121.311 | 3.986 | 0.387 |

**+1 charge**

```
		      B   	       Y
  1	 G 	    58.03	        -  	 10
  2	 L 	   171.11	  1063.61	  9
  3	 Y 	   334.18	   950.53	  8
  4	 D 	   449.20	   787.47	  7
  5	 V 	   548.27	   672.44	  6
  6	 V 	   647.34	   573.37	  5
  7	 S 	   734.37	   474.30	  4
  8	 V 	   833.44	   387.27	  3
  9	 L 	   946.52	   288.20	  2
 10	 R 	        -  	   175.12	  1  _
+2 charge

		      B   	       Y
  1	 G 	    29.52	        -  	 10
  2	 L 	    86.06	   532.31	  9
  3	 Y 	   167.59	   475.77	  8
  4	 D 	   225.11	   394.24	  7
  5	 V 	   274.64	   336.72	  6
  6	 V 	   324.17	   287.19	  5
  7	 S 	   367.69	   237.66	  4
  8	 V 	   417.22	   194.14	  3
  9	 L 	   473.77	   144.61	  2
 10	 R 	        -  	    88.06	  1  _
```

**82.** IPI00736885 Ig kappa chain V-II region TEW

| Sequence | Charge | M+H+ | XCorr | DeltaCn |
| --- | --- | --- | --- | --- |
| - .DIVMTQSPLSLPVTPGEPASISCR.S | 3 | 2556.911 | 4.441 | 0.406 |

**+1 charge**

```
		      B   	       Y
  1	 D 	   116.03	        -  	 24
  2	 I 	   229.12	  2440.39	 23
  3	 V 	   328.19	  2327.31	 22
  4	 M 	   459.23	  2228.24	 21
  5	 T 	   560.27	  2097.20	 20
  6	 Q 	   688.33	  1996.15	 19
  7	 S 	   775.37	  1868.09	 18
  8	 P 	   872.42	  1781.06	 17
  9	 L 	   985.50	  1684.01	 16
 10	 S 	  1072.53	  1570.92	 15
 11	 L 	  1185.62	  1483.89	 14
 12	 P 	  1282.67	  1370.81	 13
 13	 V 	  1381.74	  1273.76	 12
 14	 T 	  1482.79	  1174.69	 11
 15	 P 	  1579.84	  1073.64	 10
 16	 G 	  1636.86	   976.59	  9
 17	 E 	  1765.90	   919.56	  8
 18	 P 	  1862.96	   790.52	  7
 19	 A 	  1933.99	   693.47	  6
 20	 S 	  2021.03	   622.43	  5
 21	 I 	  2134.11	   535.40	  4
 22	 S 	  2221.14	   422.32	  3
 23	 C 	  2381.31	   335.28	  2
 24	 R 	        -  	   175.12	  1  _


+2 charge
		      B   	       Y
  1	 D 	    58.52	        -  	 24
  2	 I 	   115.06	  1220.70	 23
  3	 V 	   164.60	  1164.16	 22
  4	 M 	   230.12	  1114.62	 21
  5	 T 	   280.64	  1049.10	 20
  6	 Q 	   344.67	   998.58	 19
  7	 S 	   388.19	   934.55	 18
  8	 P 	   436.71	   891.03	 17
  9	 L 	   493.25	   842.51	 16
 10	 S 	   536.77	   785.97	 15
 11	 L 	   593.31	   742.45	 14
 12	 P 	   641.84	   685.91	 13
 13	 V 	   691.37	   637.38	 12
 14	 T 	   741.90	   587.85	 11
 15	 P 	   790.42	   537.32	 10
 16	 G 	   818.93	   488.80	  9
 17	 E 	   883.46	   460.29	  8
 18	 P 	   931.98	   395.76	  7
 19	 A 	   967.50	   347.24	  6
 20	 S 	  1011.02	   311.72	  5
 21	 I 	  1067.56	   268.20	  4
 22	 S 	  1111.07	   211.66	  3
 23	 C 	  1191.16	   168.15	  2
 24	 R 	        -  	    88.06	  1  _


+3 charge
		      B   	       Y
  1	 D 	    39.35	        -  	 24
  2	 I 	    77.04	   814.14	 23
  3	 V 	   110.07	   776.44	 22
  4	 M 	   153.75	   743.42	 21
  5	 T 	   187.43	   699.74	 20
  6	 Q 	   230.12	   666.06	 19
  7	 S 	   259.13	   623.37	 18
  8	 P 	   291.48	   594.36	 17
  9	 L 	   329.17	   562.01	 16
 10	 S 	   358.18	   524.31	 15
 11	 L 	   395.88	   495.30	 14
 12	 P 	   428.23	   457.61	 13
 13	 V 	   461.25	   425.26	 12
 14	 T 	   494.93	   392.23	 11
 15	 P 	   527.28	   358.55	 10
 16	 G 	   546.29	   326.20	  9
 17	 E 	   589.31	   307.19	  8
 18	 P 	   621.66	   264.18	  7
 19	 A 	   645.34	   231.83	  6
 20	 S 	   674.35	   208.15	  5
 21	 I 	   712.04	   179.14	  4
 22	 S 	   741.05	   141.44	  3
 23	 C 	   794.44	   112.43	  2
 24	 R 	        -  	    59.04	  1  _
```

**83. IPI00219960 Isoform 1L of Transcription factor 7**

| Sequence | Charge | M+H+ | XCorr | DeltaCn |
| --- | --- | --- | --- | --- |
| K.SSLVNESEGAAGGAGIPGVPGAGAGAR.G | 2 | 2310.467 | 2.929 | 0.432 |

**+1 charge**

```
		      B   	       Y
  1	 S 	    88.04	        -  	 27
  2	 S 	   175.07	  2222.12	 26
  3	 L 	   288.16	  2135.08	 25
  4	 V 	   387.22	  2022.00	 24
  5	 N 	   501.27	  1922.93	 23
  6	 E 	   630.31	  1808.89	 22
  7	 S 	   717.34	  1679.85	 21
  8	 E 	   846.38	  1592.81	 20
  9	 G 	   903.41	  1463.77	 19
 10	 A 	   974.44	  1406.75	 18
 11	 A 	  1045.48	  1335.71	 17
 12	 G 	  1102.50	  1264.68	 16
 13	 G 	  1159.52	  1207.65	 15
 14	 A 	  1230.56	  1150.63	 14
 15	 G 	  1287.58	  1079.60	 13
 16	 I 	  1400.67	  1022.57	 12
 17	 P 	  1497.72	   909.49	 11
 18	 G 	  1554.74	   812.44	 10
 19	 V 	  1653.81	   755.42	  9
 20	 P 	  1750.86	   656.35	  8
 21	 G 	  1807.88	   559.29	  7
 22	 A 	  1878.92	   502.27	  6
 23	 G 	  1935.94	   431.24	  5
 24	 A 	  2006.98	   374.21	  4
 25	 G 	  2064.00	   303.18	  3
 26	 A 	  2135.04	   246.16	  2
 27	 R 	        -  	   175.12	  1  _


+2 charge
		      B   	       Y
  1	 S 	    44.52	        -  	 27
  2	 S 	    88.04	  1111.56	 26
  3	 L 	   144.58	  1068.05	 25
  4	 V 	   194.12	  1011.50	 24
  5	 N 	   251.14	   961.97	 23
  6	 E 	   315.66	   904.95	 22
  7	 S 	   359.17	   840.43	 21
  8	 E 	   423.70	   796.91	 20
  9	 G 	   452.21	   732.39	 19
 10	 A 	   487.72	   703.88	 18
 11	 A 	   523.24	   668.36	 17
 12	 G 	   551.75	   632.84	 16
 13	 G 	   580.26	   604.33	 15
 14	 A 	   615.78	   575.82	 14
 15	 G 	   644.29	   540.30	 13
 16	 I 	   700.84	   511.79	 12
 17	 P 	   749.36	   455.25	 11
 18	 G 	   777.87	   406.72	 10
 19	 V 	   827.41	   378.21	  9
 20	 P 	   875.93	   328.68	  8
 21	 G 	   904.44	   280.15	  7
 22	 A 	   939.96	   251.64	  6
 23	 G 	   968.47	   216.12	  5
 24	 A 	  1003.99	   187.61	  4
 25	 G 	  1032.50	   152.09	  3
 26	 A 	  1068.02	   123.58	  2
 27	 R 	        -  	    88.06	  1  _
```

**84. IPI00178926 Immunoglobulin J chain**

| Sequence | Charge | M+H+ | XCorr | DeltaCn |
| --- | --- | --- | --- | --- |
| R.FVYHLSDLCK.K | 2 | 1282.463 | 3.376 | 0.547 |

**+1 charge**

```
		      B   	       Y
  1	 F 	   148.08	        -  	 10
  2	 V 	   247.14	  1134.70	  9
  3	 Y 	   410.21	  1035.63	  8
  4	 H 	   547.27	   872.56	  7
  5	 L 	   660.35	   735.51	  6
  6	 S 	   747.38	   622.42	  5
  7	 D 	   862.41	   535.39	  4
  8	 L 	   975.49	   420.36	  3
  9	 C 	  1135.66	   307.28	  2
 10	 K 	        -  	   147.11	  1  _
+2 charge

		      B   	       Y
  1	 F 	    74.54	        -  	 10
  2	 V 	   124.08	   567.85	  9
  3	 Y 	   205.61	   518.32	  8
  4	 H 	   274.14	   436.79	  7
  5	 L 	   330.68	   368.26	  6
  6	 S 	   374.19	   311.71	  5
  7	 D 	   431.71	   268.20	  4
  8	 L 	   488.25	   210.68	  3
  9	 C 	   568.33	   154.14	  2
 10	 K 	        -  	    74.06	  1  _
```

**85. IPI00066856 Ly6/PLAUR domain-containing protein 2**

| Sequence | Charge | M+H+ | XCorr | DeltaCn |
| --- | --- | --- | --- | --- |
| R.EIVYPFQGDSTVTK.S | 2 | 1584.751 | 3.439 | 0.421 |

**+1 charge**

```
		      B   	       Y
  1	 E 	   130.05	        -  	 14
  2	 I 	   243.13	  1454.75	 13
  3	 V 	   342.20	  1341.67	 12
  4	 Y 	   505.27	  1242.60	 11
  5	 P 	   602.32	  1079.54	 10
  6	 F 	   749.39	   982.48	  9
  7	 Q 	   877.45	   835.42	  8
  8	 G 	   934.47	   707.36	  7
  9	 D 	  1049.49	   650.34	  6
 10	 S 	  1136.53	   535.31	  5
 11	 T 	  1237.57	   448.28	  4
 12	 V 	  1336.64	   347.23	  3
 13	 T 	  1437.69	   248.16	  2
 14	 K 	        -  	   147.11	  1  _
+2 charge

		      B   	       Y
  1	 E 	    65.53	        -  	 14
  2	 I 	   122.07	   727.88	 13
  3	 V 	   171.60	   671.34	 12
  4	 Y 	   253.14	   621.80	 11
  5	 P 	   301.66	   540.27	 10
  6	 F 	   375.20	   491.75	  9
  7	 Q 	   439.23	   418.21	  8
  8	 G 	   467.74	   354.18	  7
  9	 D 	   525.25	   325.67	  6
 10	 S 	   568.77	   268.16	  5
 11	 T 	   619.29	   224.64	  4
 12	 V 	   668.82	   174.12	  3
 13	 T 	   719.35	   124.58	  2
 14	 K 	        -  	    74.06	  1  _
```

**86. IPI00031065 Deoxyribonuclease-1**

| Sequence | Charge | M+H+ | XCorr | DeltaCn |
| --- | --- | --- | --- | --- |
| K.LLDNLNQDAPDTYHYVVSEPLGR.N | 3 | 2630.851 | 5.143 | 0.567 |

```
+1 charge

		      B   	       Y
  1	 L 	   114.09	        -  	 23
  2	 L 	   227.18	  2516.21	 22
  3	 D 	   342.20	  2403.12	 21
  4	 N 	   456.25	  2288.09	 20
  5	 L 	   569.33	  2174.05	 19
  6	 N 	   683.37	  2060.97	 18
  7	 Q 	   811.43	  1946.92	 17
  8	 D 	   926.46	  1818.87	 16
  9	 A 	   997.49	  1703.84	 15
 10	 P 	  1094.55	  1632.80	 14
 11	 D 	  1209.57	  1535.75	 13
 12	 T 	  1310.62	  1420.72	 12
 13	 Y 	  1473.69	  1319.67	 11
 14	 H 	  1610.74	  1156.61	 10
 15	 Y 	  1773.81	  1019.55	  9
 16	 V 	  1872.88	   856.49	  8
 17	 V 	  1971.94	   757.42	  7
 18	 S 	  2058.98	   658.35	  6
 19	 E 	  2188.02	   571.32	  5
 20	 P 	  2285.07	   442.28	  4
 21	 L 	  2398.16	   345.22	  3
 22	 G 	  2455.18	   232.14	  2
 23	 R 	        -  	   175.12	  1  _


+2 charge
		      B   	       Y
  1	 L 	    57.55	        -  	 23
  2	 L 	   114.09	  1258.61	 22
  3	 D 	   171.60	  1202.06	 21
  4	 N 	   228.63	  1144.55	 20
  5	 L 	   285.17	  1087.53	 19
  6	 N 	   342.19	  1030.99	 18
  7	 Q 	   406.22	   973.97	 17
  8	 D 	   463.73	   909.94	 16
  9	 A 	   499.25	   852.42	 15
 10	 P 	   547.78	   816.90	 14
 11	 D 	   605.29	   768.38	 13
 12	 T 	   655.81	   710.86	 12
 13	 Y 	   737.35	   660.34	 11
 14	 H 	   805.88	   578.81	 10
 15	 Y 	   887.41	   510.28	  9
 16	 V 	   936.94	   428.75	  8
 17	 V 	   986.48	   379.21	  7
 18	 S 	  1029.99	   329.68	  6
 19	 E 	  1094.51	   286.16	  5
 20	 P 	  1143.04	   221.64	  4
 21	 L 	  1199.58	   173.12	  3
 22	 G 	  1228.09	   116.57	  2
 23	 R 	        -  	    88.06	  1  _
+3 charge
		      B   	       Y
  1	 L 	    38.70	        -  	 23
  2	 L 	    76.40	   839.41	 22
  3	 D 	   114.74	   801.71	 21
  4	 N 	   152.75	   763.37	 20
  5	 L 	   190.45	   725.36	 19
  6	 N 	   228.46	   687.66	 18
  7	 Q 	   271.15	   649.65	 17
  8	 D 	   309.49	   606.96	 16
  9	 A 	   333.17	   568.62	 15
 10	 P 	   365.52	   544.94	 14
 11	 D 	   403.86	   512.59	 13
 12	 T 	   437.55	   474.25	 12
 13	 Y 	   491.90	   440.56	 11
 14	 H 	   537.59	   386.21	 10
 15	 Y 	   591.94	   340.52	  9
 16	 V 	   624.96	   286.17	  8
 17	 V 	   657.99	   253.14	  7
 18	 S 	   687.00	   220.12	  6
 19	 E 	   730.01	   191.11	  5
 20	 P 	   762.36	   148.10	  4
 21	 L 	   800.06	   115.75	  3
 22	 G 	   819.06	    78.05	  2
 23	 R 	        -  	    59.04	  1  _
```

**87.IPI00005732 Isoform 1 of Activin receptor type-1B**

| Sequence | Charge | M+H+ | XCorr | DeltaCn |
| --- | --- | --- | --- | --- |
| K.VELVPAGKPFYCLSSEDLR.N | 3 | 2181.467 | 3.654 | 0.571 |

**+1 charge**

```
		      B   	       Y
  1	 V 	   100.08	        -  	 19
  2	 E 	   229.12	  2081.17	 18
  3	 L 	   342.20	  1952.13	 17
  4	 V 	   441.27	  1839.05	 16
  5	 P 	   538.32	  1739.98	 15
  6	 A 	   609.36	  1642.92	 14
  7	 G 	   666.38	  1571.89	 13
  8	 K 	   794.48	  1514.87	 12
  9	 P 	   891.53	  1386.77	 11
 10	 F 	  1038.60	  1289.72	 10
 11	 Y 	  1201.66	  1142.65	  9
 12	 C 	  1361.83	   979.59	  8
 13	 L 	  1474.91	   819.42	  7
 14	 S 	  1561.94	   706.34	  6
 15	 S 	  1648.97	   619.30	  5
 16	 E 	  1778.02	   532.27	  4
 17	 D 	  1893.04	   403.23	  3
 18	 L 	  2006.13	   288.20	  2
 19	 R 	        -  	   175.12	  1  _
+2 charge
		      B   	       Y
  1	 V 	    50.54	        -  	 19
  2	 E 	   115.06	  1041.09	 18
  3	 L 	   171.60	   976.57	 17
  4	 V 	   221.14	   920.03	 16
  5	 P 	   269.67	   870.49	 15
  6	 A 	   305.18	   821.97	 14
  7	 G 	   333.69	   786.45	 13
  8	 K 	   397.74	   757.94	 12
  9	 P 	   446.27	   693.89	 11
 10	 F 	   519.80	   645.36	 10
 11	 Y 	   601.33	   571.83	  9
 12	 C 	   681.42	   490.30	  8
 13	 L 	   737.96	   410.21	  7
 14	 S 	   781.48	   353.67	  6
 15	 S 	   824.99	   310.16	  5
 16	 E 	   889.51	   266.64	  4
 17	 D 	   947.03	   202.12	  3
 18	 L 	  1003.57	   144.61	  2
 19	 R 	        -  	    88.06	  1  _


+3 charge

		      B   	       Y
  1	 V 	    34.03	        -  	 19
  2	 E 	    77.04	   694.40	 18
  3	 L 	   114.74	   651.38	 17
  4	 V 	   147.76	   613.69	 16
  5	 P 	   180.11	   580.66	 15
  6	 A 	   203.79	   548.31	 14
  7	 G 	   222.80	   524.63	 13
  8	 K 	   265.50	   505.63	 12
  9	 P 	   297.85	   462.93	 11
 10	 F 	   346.87	   430.58	 10
 11	 Y 	   401.23	   381.55	  9
 12	 C 	   454.61	   327.20	  8
 13	 L 	   492.31	   273.81	  7
 14	 S 	   521.32	   236.12	  6
 15	 S 	   550.33	   207.11	  5
 16	 E 	   593.34	   178.10	  4
 17	 D 	   631.69	   135.08	  3
 18	 L 	   669.38	    96.74	  2
 19	 R 	        -  	    59.04	  1  _
```

**88.IPI00102300 Isoform 3 of Platelet glycoprotein VI**

| Sequence | Charge | M+H+ | XCorr | DeltaCn |
| --- | --- | --- | --- | --- |
| R.YQDQAVLFIPAMK.R | 2 | 1524.809 | 3.619 | 0.467 |

**+1 charge**

```
		      B   	       Y
  1	 Y 	   164.07	        -  	 13
  2	 Q 	   292.13	  1360.73	 12
  3	 D 	   407.16	  1232.67	 11
  4	 Q 	   535.21	  1117.64	 10
  5	 A 	   606.25	   989.59	  9
  6	 V 	   705.32	   918.55	  8
  7	 L 	   818.40	   819.48	  7
  8	 F 	   965.47	   706.40	  6
  9	 I 	  1078.56	   559.33	  5
 10	 P 	  1175.61	   446.24	  4
 11	 A 	  1246.65	   349.19	  3
 12	 M 	  1377.69	   278.15	  2
 13	 K 	        -  	   147.11	  1  _
+2 charge

		      B   	       Y
  1	 Y 	    82.54	        -  	 13
  2	 Q 	   146.57	   680.87	 12
  3	 D 	   204.08	   616.84	 11
  4	 Q 	   268.11	   559.33	 10
  5	 A 	   303.63	   495.30	  9
  6	 V 	   353.16	   459.78	  8
  7	 L 	   409.71	   410.24	  7
  8	 F 	   483.24	   353.70	  6
  9	 I 	   539.78	   280.17	  5
 10	 P 	   588.31	   223.63	  4
 11	 A 	   623.83	   175.10	  3
 12	 M 	   689.35	   139.58	  2
 13	 K 	        -  	    74.06	  1  _
```

**89. IPI00002460 Isoform 1 of Annexin A7**

| Sequence | Charge | M+H+ | XCorr | DeltaCn |
| --- | --- | --- | --- | --- |
| K.GFGTDEQAIVDVVANR.S | 2 | 1691.824 | 3.082 | 0.362 |

**+1 charge**

```
		      B   	       Y
  1	 G 	    58.03	        -  	 16
  2	 F 	   205.10	  1633.82	 15
  3	 G 	   262.12	  1486.75	 14
  4	 T 	   363.17	  1429.73	 13
  5	 D 	   478.19	  1328.68	 12
  6	 E 	   607.24	  1213.65	 11
  7	 Q 	   735.29	  1084.61	 10
  8	 A 	   806.33	   956.55	  9
  9	 I 	   919.42	   885.52	  8
 10	 V 	  1018.48	   772.43	  7
 11	 D 	  1133.51	   673.36	  6
 12	 V 	  1232.58	   558.34	  5
 13	 V 	  1331.65	   459.27	  4
 14	 A 	  1402.68	   360.20	  3
 15	 N 	  1516.73	   289.16	  2
 16	 R 	        -  	   175.12	  1  _
+2 charge

		      B   	       Y
  1	 G 	    29.52	        -  	 16
  2	 F 	   103.05	   817.41	 15
  3	 G 	   131.56	   743.88	 14
  4	 T 	   182.09	   715.37	 13
  5	 D 	   239.60	   664.84	 12
  6	 E 	   304.12	   607.33	 11
  7	 Q 	   368.15	   542.81	 10
  8	 A 	   403.67	   478.78	  9
  9	 I 	   460.21	   443.26	  8
 10	 V 	   509.75	   386.72	  7
 11	 D 	   567.26	   337.19	  6
 12	 V 	   616.79	   279.67	  5
 13	 V 	   666.33	   230.14	  4
 14	 A 	   701.85	   180.60	  3
 15	 N 	   758.87	   145.08	  2
 16	 R 	        -  	    88.06	  1  _
```

**90. IPI00021885 Isoform 1 of Fibrinogen alpha chain**

| Sequence | Charge | M+H+ | XCorr | DeltaCn |
| --- | --- | --- | --- | --- |
| R.GFGSLNDEGEGEFWLGNDYLHLLTQR.G | 3 | 2969.17 | 4.387 | 0.555 |

**+1 charge**

```
		      B   	       Y
  1	 G 	    58.03	        -  	 26
  2	 F 	   205.10	  2910.37	 25
  3	 G 	   262.12	  2763.30	 24
  4	 S 	   349.15	  2706.28	 23
  5	 L 	   462.23	  2619.25	 22
  6	 N 	   576.28	  2506.16	 21
  7	 D 	   691.30	  2392.12	 20
  8	 E 	   820.35	  2277.09	 19
  9	 G 	   877.37	  2148.05	 18
 10	 E 	  1006.41	  2091.03	 17
 11	 G 	  1063.43	  1961.99	 16
 12	 E 	  1192.48	  1904.97	 15
 13	 F 	  1339.54	  1775.92	 14
 14	 W 	  1525.62	  1628.85	 13
 15	 L 	  1638.71	  1442.78	 12
 16	 G 	  1695.73	  1329.69	 11
 17	 N 	  1809.77	  1272.67	 10
 18	 D 	  1924.80	  1158.63	  9
 19	 Y 	  2087.86	  1043.60	  8
 20	 L 	  2200.95	   880.54	  7
 21	 H 	  2338.00	   767.45	  6
 22	 L 	  2451.09	   630.39	  5
 23	 L 	  2564.17	   517.31	  4
 24	 T 	  2665.22	   404.23	  3
 25	 Q 	  2793.28	   303.18	  2
 26	 R 	        -  	   175.12	  1  _


+2 charge

		      B   	       Y
  1	 G 	    29.52	        -  	 26
  2	 F 	   103.05	  1455.69	 25
  3	 G 	   131.56	  1382.15	 24
  4	 S 	   175.08	  1353.64	 23
  5	 L 	   231.62	  1310.13	 22
  6	 N 	   288.64	  1253.59	 21
  7	 D 	   346.16	  1196.56	 20
  8	 E 	   410.68	  1139.05	 19
  9	 G 	   439.19	  1074.53	 18
 10	 E 	   503.71	  1046.02	 17
 11	 G 	   532.22	   981.50	 16
 12	 E 	   596.74	   952.99	 15
 13	 F 	   670.28	   888.47	 14
 14	 W 	   763.32	   814.93	 13
 15	 L 	   819.86	   721.89	 12
 16	 G 	   848.37	   665.35	 11
 17	 N 	   905.39	   636.84	 10
 18	 D 	   962.90	   579.82	  9
 19	 Y 	  1044.43	   522.30	  8
 20	 L 	  1100.98	   440.77	  7
 21	 H 	  1169.51	   384.23	  6
 22	 L 	  1226.05	   315.70	  5
 23	 L 	  1282.59	   259.16	  4
 24	 T 	  1333.11	   202.62	  3
 25	 Q 	  1397.14	   152.09	  2
 26	 R 	        -  	    88.06	  1  _


+3 charge

		      B   	       Y
  1	 G 	    20.01	        -  	 26
  2	 F 	    69.04	   970.79	 25
  3	 G 	    88.04	   921.77	 24
  4	 S 	   117.06	   902.76	 23
  5	 L 	   154.75	   873.75	 22
  6	 N 	   192.76	   836.06	 21
  7	 D 	   231.11	   798.04	 20
  8	 E 	   274.12	   759.70	 19
  9	 G 	   293.13	   716.69	 18
 10	 E 	   336.14	   697.68	 17
 11	 G 	   355.15	   654.67	 16
 12	 E 	   398.16	   635.66	 15
 13	 F 	   447.19	   592.65	 14
 14	 W 	   509.21	   543.62	 13
 15	 L 	   546.91	   481.60	 12
 16	 G 	   565.91	   443.90	 11
 17	 N 	   603.93	   424.89	 10
 18	 D 	   642.27	   386.88	  9
 19	 Y 	   696.63	   348.54	  8
 20	 L 	   734.32	   294.18	  7
 21	 H 	   780.01	   256.49	  6
 22	 L 	   817.70	   210.80	  5
 23	 L 	   855.40	   173.11	  4
 24	 T 	   889.08	   135.41	  3
 25	 Q 	   931.76	   101.73	  2
 26	 R 	        -  	    59.04	  1  _
```

**91. IPI00289931 MADCAM1 protein**

| Sequence | Charge | M+H+ | XCorr | DeltaCn |
| --- | --- | --- | --- | --- |
| R.GLDTSLGAVQSDTGR.S | 2 | 1477.559 | 3.509 | 0.502 |

+1 charge

```
		      B   	     Y
  1	 G 	    58.03	        -	      15
  2	 L 	   171.11	  1419.71	 14
  3	 D 	   286.14	  1306.62	 13
  4	 T 	   387.19	  1191.60	 12
  5	 S 	   474.22	  1090.55	 11
  6	 L 	   587.30	  1003.52	 10
  7	 G 	   644.32	   890.43	  9
  8	 A 	   715.36	   833.41	  8
  9	 V 	   814.43	   762.37	  7
 10	 Q 	   942.49	   663.31	  6
 11	 S 	  1029.52	   535.25	  5
 12	 D 	  1144.55	   448.22	  4
 13	 T 	  1245.60	   333.19	  3
 14	 G 	  1302.62	   232.14	  2
 15	 R 	        -	        175.12	  1
```

+2 charge

```
		       B   	     Y
  1	 G 	    29.52	        -	     15
  2	 L 	    86.06	   710.36	 14
  3	 D 	   143.57	   653.82	 13
  4	 T 	   194.10	   596.30	 12
  5	 S 	   237.61	   545.78	 11
  6	 L 	   294.16	   502.26	 10
  7	 G 	   322.67	   445.72	  9
  8	 A 	   358.18	   417.21	  8
  9	 V 	   407.72	   381.69	  7
 10	 Q 	   471.75	   332.16	  6
 11	 S 	   515.26	   268.13	  5
 12	 D 	   572.78	   224.61	  4
 13	 T 	   623.30	   167.10	  3
 14	 G 	   651.81	   116.57	  2
15	 R 	        -	         88.06	  1  _
```

**92. IPI00021434 Elafin**

| Sequence | Charge | M+H+ | XCorr | DeltaCn |
| --- | --- | --- | --- | --- |
| K.AQEPVKGPVSTKPGSCPIILIR.C | 3 | 2348.764 | 3.499 | 0.48 |

**+1 charge**

```
		      B   	       Y
  1	 A 	    72.04	        -  	 22
  2	 Q 	   200.10	  2276.41	 21
  3	 E 	   329.15	  2148.36	 20
  4	 P 	   426.20	  2019.31	 19
  5	 V 	   525.27	  1922.26	 18
  6	 K 	   653.36	  1823.19	 17
  7	 G 	   710.38	  1695.10	 16
  8	 P 	   807.44	  1638.08	 15
  9	 V 	   906.50	  1541.02	 14
 10	 S 	   993.54	  1441.95	 13
 11	 T 	  1094.58	  1354.92	 12
 12	 K 	  1222.68	  1253.87	 11
 13	 P 	  1319.73	  1125.78	 10
 14	 G 	  1376.75	  1028.73	  9
 15	 S 	  1463.79	   971.71	  8
 16	 C 	  1623.95	   884.67	  7
 17	 P 	  1721.00	   724.51	  6
 18	 I 	  1834.09	   627.46	  5
 19	 I 	  1947.17	   514.37	  4
 20	 L 	  2060.26	   401.29	  3
 21	 I 	  2173.34	   288.20	  2
 22	 R 	        -  	   175.12	  1  _


+2 charge
		      B   	       Y
  1	 A 	    36.53	        -  	 22
  2	 Q 	   100.56	  1138.71	 21
  3	 E 	   165.08	  1074.68	 20
  4	 P 	   213.60	  1010.16	 19
  5	 V 	   263.14	   961.63	 18
  6	 K 	   327.18	   912.10	 17
  7	 G 	   355.70	   848.05	 16
  8	 P 	   404.22	   819.54	 15
  9	 V 	   453.76	   771.01	 14
 10	 S 	   497.27	   721.48	 13
 11	 T 	   547.80	   677.96	 12
 12	 K 	   611.84	   627.44	 11
 13	 P 	   660.37	   563.39	 10
 14	 G 	   688.88	   514.87	  9
 15	 S 	   732.40	   486.36	  8
 16	 C 	   812.48	   442.84	  7
 17	 P 	   861.01	   362.76	  6
 18	 I 	   917.55	   314.23	  5
 19	 I 	   974.09	   257.69	  4
 20	 L 	  1030.63	   201.15	  3
 21	 I 	  1087.17	   144.61	  2
 22	 R 	        -  	    88.06	  1  _
+3 charge
		      B   	       Y
  1	 A 	    24.69	        -  	 22
  2	 Q 	    67.37	   759.48	 21
  3	 E 	   110.39	   716.79	 20
  4	 P 	   142.74	   673.78	 19
  5	 V 	   175.76	   641.42	 18
  6	 K 	   218.46	   608.40	 17
  7	 G 	   237.47	   565.70	 16
  8	 P 	   269.82	   546.70	 15
  9	 V 	   302.84	   514.35	 14
 10	 S 	   331.85	   481.32	 13
 11	 T 	   365.53	   452.31	 12
 12	 K 	   408.23	   418.63	 11
 13	 P 	   440.58	   375.93	 10
 14	 G 	   459.59	   343.58	  9
 15	 S 	   488.60	   324.57	  8
 16	 C 	   541.99	   295.56	  7
 17	 P 	   574.34	   242.17	  6
 18	 I 	   612.03	   209.82	  5
 19	 I 	   649.73	   172.13	  4
 20	 L 	   687.42	   134.43	  3
 21	 I 	   725.12	    96.74	  2
 22	 R 	        -  	    59.04	  1  _
```

**93. IPI00442297 Isoform 2 of Neurotrimin**

| Sequence | Charge | M+H+ | XCorr | DeltaCn |
| --- | --- | --- | --- | --- |
| K.VTVNYPPYISEAK.G | 2 | 1481.674 | 2.911 | 0.478 |

**+1 charge**

```
		      B   	       Y
  1	 V 	   100.08	        -  	 13
  2	 T 	   201.12	  1381.70	 12
  3	 V 	   300.19	  1280.65	 11
  4	 N 	   414.23	  1181.58	 10
  5	 Y 	   577.30	  1067.54	  9
  6	 P 	   674.35	   904.48	  8
  7	 P 	   771.40	   807.42	  7
  8	 Y 	   934.47	   710.37	  6
  9	 I 	  1047.55	   547.31	  5
 10	 S 	  1134.58	   434.22	  4
 11	 E 	  1263.63	   347.19	  3
 12	 A 	  1334.66	   218.15	  2
 13	 K 	        -  	   147.11	  1  _
+2 charge

		      B   	       Y
  1	 V 	    50.54	        -  	 13
  2	 T 	   101.07	   691.35	 12
  3	 V 	   150.60	   640.83	 11
  4	 N 	   207.62	   591.30	 10
  5	 Y 	   289.15	   534.27	  9
  6	 P 	   337.68	   452.74	  8
  7	 P 	   386.21	   404.22	  7
  8	 Y 	   467.74	   355.69	  6
  9	 I 	   524.28	   274.16	  5
 10	 S 	   567.80	   217.62	  4
 11	 E 	   632.32	   174.10	  3
 12	 A 	   667.83	   109.58	  2
 13	 K 	        -  	    74.06	  1  _
```

**94. IPI00784320 Protein FAM83H**

| Sequence | Charge | M+H+ | XCorr | DeltaCn |
| --- | --- | --- | --- | --- |
| R.EEGLGFPSFLDPDR.H | 2 | 1579.692 | 3.235 | 0.269 |

**+1 charge**

```
		      B   	       Y
  1	 E 	   130.05	        -  	 14
  2	 E 	   259.09	  1449.70	 13
  3	 G 	   316.11	  1320.66	 12
  4	 L 	   429.20	  1263.64	 11
  5	 G 	   486.22	  1150.55	 10
  6	 F 	   633.29	  1093.53	  9
  7	 P 	   730.34	   946.46	  8
  8	 S 	   817.37	   849.41	  7
  9	 F 	   964.44	   762.38	  6
 10	 L 	  1077.53	   615.31	  5
 11	 D 	  1192.55	   502.23	  4
 12	 P 	  1289.60	   387.20	  3
 13	 D 	  1404.63	   290.15	  2
 14	 R 	        -  	   175.12	  1  _
+2 charge

		      B   	       Y
  1	 E 	    65.53	        -  	 14
  2	 E 	   130.05	   725.35	 13
  3	 G 	   158.56	   660.83	 12
  4	 L 	   215.10	   632.32	 11
  5	 G 	   243.61	   575.78	 10
  6	 F 	   317.15	   547.27	  9
  7	 P 	   365.67	   473.74	  8
  8	 S 	   409.19	   425.21	  7
  9	 F 	   482.72	   381.69	  6
 10	 L 	   539.27	   308.16	  5
 11	 D 	   596.78	   251.62	  4
 12	 P 	   645.31	   194.10	  3
 13	 D 	   702.82	   145.58	  2
 14	 R 	        -  	    88.06	  1  _
```

**95. IPI00385252 Ig kappa chain V-III region GOL**

| Sequence | Charge | M+H+ | XCorr | DeltaCn |
| --- | --- | --- | --- | --- |
| - .EIVLTQSPGTLSLSPGER.A | 3 | 1885.108 | 4.745 | 0.329 |

**+1 charge**

```
		      B   	       Y
  1	 E 	   130.05	        -  	 18
  2	 I 	   243.13	  1754.96	 17
  3	 V 	   342.20	  1641.88	 16
  4	 L 	   455.29	  1542.81	 15
  5	 T 	   556.33	  1429.73	 14
  6	 Q 	   684.39	  1328.68	 13
  7	 S 	   771.42	  1200.62	 12
  8	 P 	   868.48	  1113.59	 11
  9	 G 	   925.50	  1016.54	 10
 10	 T 	  1026.55	   959.52	  9
 11	 L 	  1139.63	   858.47	  8
 12	 S 	  1226.66	   745.38	  7
 13	 L 	  1339.75	   658.35	  6
 14	 S 	  1426.78	   545.27	  5
 15	 P 	  1523.83	   458.24	  4
 16	 G 	  1580.85	   361.18	  3
 17	 E 	  1709.90	   304.16	  2
 18	 R 	        -  	   175.12	  1  _
+2 charge
		      B   	       Y
  1	 E 	    65.53	        -  	 18
  2	 I 	   122.07	   877.99	 17
  3	 V 	   171.60	   821.44	 16
  4	 L 	   228.15	   771.91	 15
  5	 T 	   278.67	   715.37	 14
  6	 Q 	   342.70	   664.84	 13
  7	 S 	   386.22	   600.81	 12
  8	 P 	   434.74	   557.30	 11
  9	 G 	   463.25	   508.77	 10
 10	 T 	   513.78	   480.26	  9
 11	 L 	   570.32	   429.74	  8
 12	 S 	   613.83	   373.20	  7
 13	 L 	   670.38	   329.68	  6
 14	 S 	   713.89	   273.14	  5
 15	 P 	   762.42	   229.62	  4
 16	 G 	   790.93	   181.10	  3
 17	 E 	   855.45	   152.58	  2
 18	 R 	        -  	    88.06	  1  _
+3 charge

		      B   	       Y
  1	 E 	    44.02	        -  	 18
  2	 I 	    81.72	   585.66	 17
  3	 V 	   114.74	   547.97	 16
  4	 L 	   152.43	   514.94	 15
  5	 T 	   186.12	   477.25	 14
  6	 Q 	   228.80	   443.57	 13
  7	 S 	   257.81	   400.88	 12
  8	 P 	   290.16	   371.87	 11
  9	 G 	   309.17	   339.52	 10
 10	 T 	   342.85	   320.51	  9
 11	 L 	   380.55	   286.83	  8
 12	 S 	   409.56	   249.13	  7
 13	 L 	   447.25	   220.12	  6
 14	 S 	   476.26	   182.43	  5
 15	 P 	   508.62	   153.42	  4
 16	 G 	   527.62	   121.07	  3
 17	 E 	   570.64	   102.06	  2
 18	 R 	        -  	    59.04	  1  _
```

**96. IPI00297420 Frizzled-4 precursor**

| Sequence | Charge | M+H+ | XCorr | DeltaCn |
| --- | --- | --- | --- | --- |
| K.INIPIGPCGGMCLSVK.R | 2 | 1717.054 | 2.955 | 0.286 |

```
+1 charge

		      B   	       Y
  1	 I 	   114.09	        -  	 16
  2	 N 	   228.13	  1603.05	 15
  3	 I 	   341.22	  1489.01	 14
  4	 P 	   438.27	  1375.92	 13
  5	 I 	   551.36	  1278.87	 12
  6	 G 	   608.38	  1165.79	 11
  7	 P 	   705.43	  1108.76	 10
  8	 C 	   865.59	  1011.71	  9
  9	 G 	   922.62	   851.55	  8
 10	 G 	   979.64	   794.52	  7
 11	 M 	  1110.68	   737.50	  6
 12	 C 	  1270.84	   606.46	  5
 13	 L 	  1383.93	   446.30	  4
 14	 S 	  1470.96	   333.21	  3
 15	 V 	  1570.03	   246.18	  2
 16	 K 	        -  	   147.11	  1  _
+2 charge

		      B   	       Y
  1	 I 	    57.55	        -  	 16
  2	 N 	   114.57	   802.03	 15
  3	 I 	   171.11	   745.01	 14
  4	 P 	   219.64	   688.46	 13
  5	 I 	   276.18	   639.94	 12
  6	 G 	   304.69	   583.40	 11
  7	 P 	   353.22	   554.89	 10
  8	 C 	   433.30	   506.36	  9
  9	 G 	   461.81	   426.28	  8
 10	 G 	   490.32	   397.77	  7
 11	 M 	   555.84	   369.26	  6
 12	 C 	   635.93	   303.73	  5
 13	 L 	   692.47	   223.65	  4
 14	 S 	   735.98	   167.11	  3
 15	 V 	   785.52	   123.59	  2
 16	 K 	        -  	    74.06	  1  _
```

**97. IPI00395337 Isoform 1 of Pre-mRNA 3'-end-processing factor FIP1**

| Sequence | Charge | M+H+ | XCorr | DeltaCn |
| --- | --- | --- | --- | --- |
| K.GVDLDAPGSINGVPLLEVDLDSFEDK.P | 3 | 2715.947 | 4.319 | 0.281 |

**+1 charge**

```
		      B   	       Y
  1	 G 	    58.03	        -  	 26
  2	 V 	   157.10	  2657.32	 25
  3	 D 	   272.12	  2558.25	 24
  4	 L 	   385.21	  2443.22	 23
  5	 D 	   500.24	  2330.14	 22
  6	 A 	   571.27	  2215.11	 21
  7	 P 	   668.32	  2144.08	 20
  8	 G 	   725.35	  2047.02	 19
  9	 S 	   812.38	  1990.00	 18
 10	 I 	   925.46	  1902.97	 17
 11	 N 	  1039.51	  1789.89	 16
 12	 G 	  1096.53	  1675.84	 15
 13	 V 	  1195.60	  1618.82	 14
 14	 P 	  1292.65	  1519.75	 13
 15	 L 	  1405.73	  1422.70	 12
 16	 L 	  1518.82	  1309.62	 11
 17	 E 	  1647.86	  1196.53	 10
 18	 V 	  1746.93	  1067.49	  9
 19	 D 	  1861.95	   968.42	  8
 20	 L 	  1975.04	   853.39	  7
 21	 D 	  2090.07	   740.31	  6
 22	 S 	  2177.10	   625.28	  5
 23	 F 	  2324.17	   538.25	  4
 24	 E 	  2453.21	   391.18	  3
 25	 D 	  2568.24	   262.14	  2
 26	 K 	        -  	   147.11	  1  _


+2 charge

		      B   	       Y
  1	 G 	    29.52	        -  	 26
  2	 V 	    79.05	  1329.16	 25
  3	 D 	   136.57	  1279.63	 24
  4	 L 	   193.11	  1222.12	 23
  5	 D 	   250.62	  1165.57	 22
  6	 A 	   286.14	  1108.06	 21
  7	 P 	   334.67	  1072.54	 20
  8	 G 	   363.18	  1024.02	 19
  9	 S 	   406.69	   995.50	 18
 10	 I 	   463.23	   951.99	 17
 11	 N 	   520.26	   895.45	 16
 12	 G 	   548.77	   838.42	 15
 13	 V 	   598.30	   809.91	 14
 14	 P 	   646.83	   760.38	 13
 15	 L 	   703.37	   711.85	 12
 16	 L 	   759.91	   655.31	 11
 17	 E 	   824.43	   598.77	 10
 18	 V 	   873.97	   534.25	  9
 19	 D 	   931.48	   484.71	  8
 20	 L 	   988.02	   427.20	  7
 21	 D 	  1045.54	   370.66	  6
 22	 S 	  1089.05	   313.15	  5
 23	 F 	  1162.59	   269.63	  4
 24	 E 	  1227.11	   196.09	  3
 25	 D 	  1284.62	   131.57	  2
 26	 K 	        -  	    74.06	  1  _


+3 charge
		      B   	       Y
  1	 G 	    20.01	        -  	 26
  2	 V 	    53.04	   886.44	 25
  3	 D 	    91.38	   853.42	 24
  4	 L 	   129.07	   815.08	 23
  5	 D 	   167.42	   777.38	 22
  6	 A 	   191.10	   739.04	 21
  7	 P 	   223.45	   715.36	 20
  8	 G 	   242.45	   683.01	 19
  9	 S 	   271.46	   664.01	 18
 10	 I 	   309.16	   634.99	 17
 11	 N 	   347.17	   597.30	 16
 12	 G 	   366.18	   559.29	 15
 13	 V 	   399.20	   540.28	 14
 14	 P 	   431.55	   507.26	 13
 15	 L 	   469.25	   474.90	 12
 16	 L 	   506.94	   437.21	 11
 17	 E 	   549.96	   399.52	 10
 18	 V 	   582.98	   356.50	  9
 19	 D 	   621.32	   323.48	  8
 20	 L 	   659.02	   285.14	  7
 21	 D 	   697.36	   247.44	  6
 22	 S 	   726.37	   209.10	  5
 23	 F 	   775.39	   180.09	  4
 24	 E 	   818.41	   131.07	  3
 25	 D 	   856.75	    88.05	  2
 26	 K 	        -  	    49.71	  1  _
```

**98.IPI00296180 Isoform 1 of Urokinase-type plasminogen activator**

| Sequence | Charge | M+H+ | XCorr | DeltaCn |
| --- | --- | --- | --- | --- |
| R.ECQQPHYYGSEVTTK.M | 2 | 1827.923 | 3.135 | 0.402 |

**+1 charge**

```
		      B   	       Y
  1	 E 	   130.05	        -  	 15
  2	 C 	   290.22	  1697.89	 14
  3	 Q 	   418.27	  1537.73	 13
  4	 Q 	   546.33	  1409.67	 12
  5	 P 	   643.39	  1281.61	 11
  6	 H 	   780.44	  1184.56	 10
  7	 Y 	   943.51	  1047.50	  9
  8	 Y 	  1106.57	   884.44	  8
  9	 G 	  1163.59	   721.37	  7
 10	 S 	  1250.62	   664.35	  6
 11	 E 	  1379.67	   577.32	  5
 12	 V 	  1478.74	   448.28	  4
 13	 T 	  1579.78	   349.21	  3
 14	 T 	  1680.83	   248.16	  2
 15	 K 	        -  	   147.11	  1  _
+2 charge

		      B   	       Y
  1	 E 	    65.53	        -  	 15
  2	 C 	   145.61	   849.45	 14
  3	 Q 	   209.64	   769.37	 13
  4	 Q 	   273.67	   705.34	 12
  5	 P 	   322.20	   641.31	 11
  6	 H 	   390.73	   592.78	 10
  7	 Y 	   472.26	   524.25	  9
  8	 Y 	   553.79	   442.72	  8
  9	 G 	   582.30	   361.19	  7
 10	 S 	   625.82	   332.68	  6
 11	 E 	   690.34	   289.16	  5
 12	 V 	   739.87	   224.64	  4
 13	 T 	   790.40	   175.11	  3
 14	 T 	   840.92	   124.58	  2
 15	 K 	        -  	    74.06	  1  _
```

**99. IPI00329538 Prostasin**

| Sequence | Charge | M+H+ | XCorr | DeltaCn |
| --- | --- | --- | --- | --- |
| K.LGAHQLDSYSEDAK.V | 2 | 1534.609 | 3.385 | 0.496 |

**+1 charge**

```
		      B   	       Y
  1	 L 	   114.09	        -  	 14
  2	 G 	   171.11	  1420.63	 13
  3	 A 	   242.15	  1363.61	 12
  4	 H 	   379.21	  1292.58	 11
  5	 Q 	   507.27	  1155.52	 10
  6	 L 	   620.35	  1027.46	  9
  7	 D 	   735.38	   914.37	  8
  8	 S 	   822.41	   799.35	  7
  9	 Y 	   985.47	   712.31	  6
 10	 S 	  1072.51	   549.25	  5
 11	 E 	  1201.55	   462.22	  4
 12	 D 	  1316.58	   333.18	  3
 13	 A 	  1387.61	   218.15	  2
 14	 K 	        -  	   147.11	  1  _
+2 charge

		      B   	       Y
  1	 L 	    57.55	        -  	 14
  2	 G 	    86.06	   710.82	 13
  3	 A 	   121.58	   682.31	 12
  4	 H 	   190.11	   646.79	 11
  5	 Q 	   254.14	   578.26	 10
  6	 L 	   310.68	   514.23	  9
  7	 D 	   368.19	   457.69	  8
  8	 S 	   411.71	   400.18	  7
  9	 Y 	   493.24	   356.66	  6
 10	 S 	   536.76	   275.13	  5
 11	 E 	   601.28	   231.61	  4
 12	 D 	   658.79	   167.09	  3
 13	 A 	   694.31	   109.58	  2
 14	 K 	        -  	    74.06	  1  _
```

**100. IPI00219018 Glyceraldehyde-3-phosphate dehydrogenase**

| Sequence | Charge | M+H+ | XCorr | DeltaCn |
| --- | --- | --- | --- | --- |
| K.IISNASCTTNCLAPLAK.V | 2 | 1835.079 | 3.213 | 0.416 |

**+1 charge**

```
		      B   	       Y
  1	 I 	   114.09	        -  	 17
  2	 I 	   227.18	  1721.10	 16
  3	 S 	   314.21	  1608.02	 15
  4	 N 	   428.25	  1520.99	 14
  5	 A 	   499.29	  1406.95	 13
  6	 S 	   586.32	  1335.91	 12
  7	 C 	   746.48	  1248.88	 11
  8	 T 	   847.53	  1088.71	 10
  9	 T 	   948.58	   987.66	  9
 10	 N 	  1062.62	   886.62	  8
 11	 C 	  1222.79	   772.57	  7
 12	 L 	  1335.87	   612.41	  6
 13	 A 	  1406.91	   499.32	  5
 14	 P 	  1503.96	   428.29	  4
 15	 L 	  1617.05	   331.23	  3
 16	 A 	  1688.08	   218.15	  2
 17	 K 	        -  	   147.11	  1  _
+2 charge

		      B   	       Y
  1	 I 	    57.55	        -  	 17
  2	 I 	   114.09	   861.06	 16
  3	 S 	   157.61	   804.51	 15
  4	 N 	   214.63	   761.00	 14
  5	 A 	   250.15	   703.98	 13
  6	 S 	   293.66	   668.46	 12
  7	 C 	   373.75	   624.94	 11
  8	 T 	   424.27	   544.86	 10
  9	 T 	   474.79	   494.34	  9
 10	 N 	   531.82	   443.81	  8
 11	 C 	   611.90	   386.79	  7
 12	 L 	   668.44	   306.71	  6
 13	 A 	   703.96	   250.17	  5
 14	 P 	   752.48	   214.65	  4
 15	 L 	   809.03	   166.12	  3
 16	 A 	   844.55	   109.58	  2
 17	 K 	        -  	    74.06	  1  _
```

**101. IPI00940464 Aminoacylase-1**

| Sequence | Charge | M+H+ | XCorr | DeltaCn |
| --- | --- | --- | --- | --- |
| R.AVGVPALGFSPMNR.T | 2 | 1416.674 | 3.326 | 0.53 |

**+1 charge**

```
		      B   	       Y
  1	 A 	    72.04	        -  	 14
  2	 V 	   171.11	  1344.71	 13
  3	 G 	   228.13	  1245.64	 12
  4	 V 	   327.20	  1188.62	 11
  5	 P 	   424.26	  1089.55	 10
  6	 A 	   495.29	   992.50	  9
  7	 L 	   608.38	   921.46	  8
  8	 G 	   665.40	   808.38	  7
  9	 F 	   812.47	   751.36	  6
 10	 S 	   899.50	   604.29	  5
 11	 P 	   996.55	   517.26	  4
 12	 M 	  1127.59	   420.20	  3
 13	 N 	  1241.63	   289.16	  2
 14	 R 	        -  	   175.12	  1  _
+2 charge

		      B   	       Y
  1	 A 	    36.53	        -  	 14
  2	 V 	    86.06	   672.86	 13
  3	 G 	   114.57	   623.32	 12
  4	 V 	   164.10	   594.81	 11
  5	 P 	   212.63	   545.28	 10
  6	 A 	   248.15	   496.75	  9
  7	 L 	   304.69	   461.23	  8
  8	 G 	   333.20	   404.69	  7
  9	 F 	   406.74	   376.18	  6
 10	 S 	   450.25	   302.65	  5
 11	 P 	   498.78	   259.13	  4
 12	 M 	   564.30	   210.60	  3
 13	 N 	   621.32	   145.08	  2
 14	 R 	        -  	    88.06	  1  _
```

**102. IPI00293331 Ribonucleases P/MRP protein subunit POP1**

| Sequence | Charge | M+H+ | XCorr | DeltaCn |
| --- | --- | --- | --- | --- |
| K.LGTLAPFCCPWEQLTQDWESR.V | 3 | 2595.835 | 3.409 | 0.288 |

**+1 charge**

```
		      B   	       Y
  1	 L 	   114.09	        -  	 21
  2	 G 	   171.11	  2481.37	 20
  3	 T 	   272.16	  2424.34	 19
  4	 L 	   385.24	  2323.30	 18
  5	 A 	   456.28	  2210.21	 17
  6	 P 	   553.33	  2139.18	 16
  7	 F 	   700.40	  2042.12	 15
  8	 C 	   860.57	  1895.05	 14
  9	 C 	  1020.73	  1734.89	 13
 10	 P 	  1117.79	  1574.72	 12
 11	 W 	  1303.87	  1477.67	 11
 12	 E 	  1432.91	  1291.59	 10
 13	 Q 	  1560.97	  1162.55	  9
 14	 L 	  1674.05	  1034.49	  8
 15	 T 	  1775.10	   921.41	  7
 16	 Q 	  1903.16	   820.36	  6
 17	 D 	  2018.18	   692.30	  5
 18	 W 	  2204.26	   577.27	  4
 19	 E 	  2333.31	   391.19	  3
 20	 S 	  2420.34	   262.15	  2
 21	 R 	        -  	   175.12	  1  _
+2 charge
		      B   	       Y
  1	 L 	    57.55	        -  	 21
  2	 G 	    86.06	  1241.19	 20
  3	 T 	   136.58	  1212.68	 19
  4	 L 	   193.13	  1162.15	 18
  5	 A 	   228.64	  1105.61	 17
  6	 P 	   277.17	  1070.09	 16
  7	 F 	   350.71	  1021.56	 15
  8	 C 	   430.79	   948.03	 14
  9	 C 	   510.87	   867.95	 13
 10	 P 	   559.40	   787.87	 12
 11	 W 	   652.44	   739.34	 11
 12	 E 	   716.96	   646.30	 10
 13	 Q 	   780.99	   581.78	  9
 14	 L 	   837.53	   517.75	  8
 15	 T 	   888.05	   461.21	  7
 16	 Q 	   952.08	   410.68	  6
 17	 D 	  1009.60	   346.65	  5
 18	 W 	  1102.64	   289.14	  4
 19	 E 	  1167.16	   196.10	  3
 20	 S 	  1210.67	   131.58	  2
 21	 R 	        -  	    88.06	  1  _
+3 charge

		      B   	       Y
  1	 L 	    38.70	        -  	 21
  2	 G 	    57.71	   827.79	 20
  3	 T 	    91.39	   808.79	 19
  4	 L 	   129.09	   775.10	 18
  5	 A 	   152.77	   737.41	 17
  6	 P 	   185.12	   713.73	 16
  7	 F 	   234.14	   681.38	 15
  8	 C 	   287.53	   632.36	 14
  9	 C 	   340.92	   578.97	 13
 10	 P 	   373.27	   525.58	 12
 11	 W 	   435.29	   493.23	 11
 12	 E 	   478.31	   431.20	 10
 13	 Q 	   520.99	   388.19	  9
 14	 L 	   558.69	   345.50	  8
 15	 T 	   592.37	   307.81	  7
 16	 Q 	   635.06	   274.12	  6
 17	 D 	   673.40	   231.44	  5
 18	 W 	   735.43	   193.10	  4
 19	 E 	   778.44	   131.07	  3
 20	 S 	   807.45	    88.06	  2
 21	 R 	        -  	    59.04	  1  _
```

**103. IPI00022331 Phosphatidylcholine-sterol acyltransferase**

| Sequence | Charge | M+H+ | XCorr | DeltaCn |
| --- | --- | --- | --- | --- |
| R.TYIYDHGFPYTDPVGVLYEDGDDTVATR.S | 3 | 3181.367 | 3.713 | 0.523 |

**+1 charge**

```
		      B   	       Y
  1	 T 	   102.05	        -  	 28
  2	 Y 	   265.12	  3078.40	 27
  3	 I 	   378.20	  2915.34	 26
  4	 Y 	   541.27	  2802.25	 25
  5	 D 	   656.29	  2639.19	 24
  6	 H 	   793.35	  2524.16	 23
  7	 G 	   850.37	  2387.10	 22
  8	 F 	   997.44	  2330.08	 21
  9	 P 	  1094.49	  2183.01	 20
 10	 Y 	  1257.56	  2085.96	 19
 11	 T 	  1358.61	  1922.90	 18
 12	 D 	  1473.63	  1821.85	 17
 13	 P 	  1570.68	  1706.82	 16
 14	 V 	  1669.75	  1609.77	 15
 15	 G 	  1726.77	  1510.70	 14
 16	 V 	  1825.84	  1453.68	 13
 17	 L 	  1938.93	  1354.61	 12
 18	 Y 	  2101.99	  1241.53	 11
 19	 E 	  2231.03	  1078.46	 10
 20	 D 	  2346.06	   949.42	  9
 21	 G 	  2403.08	   834.40	  8
 22	 D 	  2518.11	   777.37	  7
 23	 D 	  2633.14	   662.35	  6
 24	 T 	  2734.18	   547.32	  5
 25	 V 	  2833.25	   446.27	  4
 26	 A 	  2904.29	   347.20	  3
 27	 T 	  3005.34	   276.17	  2
 28	 R 	        -  	   175.12	  1  _


+2 charge

		      B   	       Y
  1	 T 	    51.53	        -  	 28
  2	 Y 	   133.06	  1539.70	 27
  3	 I 	   189.60	  1458.17	 26
  4	 Y 	   271.14	  1401.63	 25
  5	 D 	   328.65	  1320.10	 24
  6	 H 	   397.18	  1262.58	 23
  7	 G 	   425.69	  1194.06	 22
  8	 F 	   499.22	  1165.54	 21
  9	 P 	   547.75	  1092.01	 20
 10	 Y 	   629.28	  1043.48	 19
 11	 T 	   679.81	   961.95	 18
 12	 D 	   737.32	   911.43	 17
 13	 P 	   785.85	   853.92	 16
 14	 V 	   835.38	   805.39	 15
 15	 G 	   863.89	   755.85	 14
 16	 V 	   913.43	   727.34	 13
 17	 L 	   969.97	   677.81	 12
 18	 Y 	  1051.50	   621.27	 11
 19	 E 	  1116.02	   539.74	 10
 20	 D 	  1173.53	   475.21	  9
 21	 G 	  1202.04	   417.70	  8
 22	 D 	  1259.56	   389.19	  7
 23	 D 	  1317.07	   331.68	  6
 24	 T 	  1367.60	   274.16	  5
 25	 V 	  1417.13	   223.64	  4
 26	 A 	  1452.65	   174.11	  3
 27	 T 	  1503.17	   138.59	  2
 28	 R 	        -  	    88.06	  1  _


+3 charge

		      B   	       Y
  1	 T 	    34.69	        -  	 28
  2	 Y 	    89.04	  1026.80	 27
  3	 I 	   126.74	   972.45	 26
  4	 Y 	   181.09	   934.76	 25
  5	 D 	   219.44	   880.40	 24
  6	 H 	   265.12	   842.06	 23
  7	 G 	   284.13	   796.37	 22
  8	 F 	   333.15	   777.37	 21
  9	 P 	   365.50	   728.34	 20
 10	 Y 	   419.86	   695.99	 19
 11	 T 	   453.54	   641.64	 18
 12	 D 	   491.88	   607.95	 17
 13	 P 	   524.23	   569.61	 16
 14	 V 	   557.26	   537.26	 15
 15	 G 	   576.26	   504.24	 14
 16	 V 	   609.29	   485.23	 13
 17	 L 	   646.98	   452.21	 12
 18	 Y 	   701.34	   414.51	 11
 19	 E 	   744.35	   360.16	 10
 20	 D 	   782.69	   317.15	  9
 21	 G 	   801.70	   278.80	  8
 22	 D 	   840.04	   259.80	  7
 23	 D 	   878.38	   221.45	  6
 24	 T 	   912.07	   183.11	  5
 25	 V 	   945.09	   149.43	  4
 26	 A 	   968.77	   116.41	  3
 27	 T 	  1002.45	    92.73	  2
 28	 R 	        -  	    59.04	  1  _
```

**104. IPI00017557 Secreted frizzled-related protein 4**

| Sequence | Charge | M+H+ | XCorr | DeltaCn |
| --- | --- | --- | --- | --- |
| R.GVCISPEAIVTDLPEDVK.W | 2 | 1943.178 | 4.271 | 0.432 |

**+1 charge**

```
		      B   	       Y
  1	 G 	    58.03	        -  	 18
  2	 V 	   157.10	  1885.10	 17
  3	 C 	   317.26	  1786.03	 16
  4	 I 	   430.35	  1625.86	 15
  5	 S 	   517.38	  1512.78	 14
  6	 P 	   614.43	  1425.75	 13
  7	 E 	   743.47	  1328.69	 12
  8	 A 	   814.51	  1199.65	 11
  9	 I 	   927.60	  1128.61	 10
 10	 V 	  1026.66	  1015.53	  9
 11	 T 	  1127.71	   916.46	  8
 12	 D 	  1242.74	   815.41	  7
 13	 L 	  1355.82	   700.39	  6
 14	 P 	  1452.87	   587.30	  5
 15	 E 	  1581.92	   490.25	  4
 16	 D 	  1696.94	   361.21	  3
 17	 V 	  1796.01	   246.18	  2
 18	 K 	        -  	   147.11	  1  _
+2 charge
		      B   	       Y
  1	 G 	    29.52	        -  	 18
  2	 V 	    79.05	   943.05	 17
  3	 C 	   159.13	   893.52	 16
  4	 I 	   215.68	   813.44	 15
  5	 S 	   259.19	   756.89	 14
  6	 P 	   307.72	   713.38	 13
  7	 E 	   372.24	   664.85	 12
  8	 A 	   407.76	   600.33	 11
  9	 I 	   464.30	   564.81	 10
 10	 V 	   513.84	   508.27	  9
 11	 T 	   564.36	   458.73	  8
 12	 D 	   621.87	   408.21	  7
 13	 L 	   678.41	   350.70	  6
 14	 P 	   726.94	   294.16	  5
 15	 E 	   791.46	   245.63	  4
 16	 D 	   848.98	   181.11	  3
 17	 V 	   898.51	   123.59	  2
 18	 K 	        -  	    74.06	  1  _
```

**105. IPI00896419 Isoform 1 of Inter-alpha-trypsin inhibitor heavy chain H4**

| Sequence | Charge | M+H+ | XCorr | DeltaCn |
| --- | --- | --- | --- | --- |
| R.HRQGPVNLLSDPEQGVEVTGQYER.E | 3 | 2709.912 | 3.478 | 0.376 |

**+1 charge**

```
		      B   	       Y
  1	 H 	   138.07	        -  	 24
  2	 R 	   294.17	  2571.28	 23
  3	 Q 	   422.23	  2415.18	 22
  4	 G 	   479.25	  2287.12	 21
  5	 P 	   576.30	  2230.10	 20
  6	 V 	   675.37	  2133.05	 19
  7	 N 	   789.41	  2033.98	 18
  8	 L 	   902.50	  1919.93	 17
  9	 L 	  1015.58	  1806.85	 16
 10	 S 	  1102.61	  1693.77	 15
 11	 D 	  1217.64	  1606.73	 14
 12	 P 	  1314.69	  1491.71	 13
 13	 E 	  1443.73	  1394.65	 12
 14	 Q 	  1571.79	  1265.61	 11
 15	 G 	  1628.81	  1137.55	 10
 16	 V 	  1727.88	  1080.53	  9
 17	 E 	  1856.92	   981.46	  8
 18	 V 	  1955.99	   852.42	  7
 19	 T 	  2057.04	   753.35	  6
 20	 G 	  2114.06	   652.30	  5
 21	 Q 	  2242.12	   595.28	  4
 22	 Y 	  2405.18	   467.22	  3
 23	 E 	  2534.23	   304.16	  2
 24	 R 	        -  	   175.12	  1  _


+2 charge

		      B   	       Y
  1	 H 	    69.54	        -  	 24
  2	 R 	   147.59	  1286.14	 23
  3	 Q 	   211.62	  1208.09	 22
  4	 G 	   240.13	  1144.06	 21
  5	 P 	   288.65	  1115.55	 20
  6	 V 	   338.19	  1067.03	 19
  7	 N 	   395.21	  1017.49	 18
  8	 L 	   451.75	   960.47	 17
  9	 L 	   508.29	   903.93	 16
 10	 S 	   551.81	   847.39	 15
 11	 D 	   609.32	   803.87	 14
 12	 P 	   657.85	   746.36	 13
 13	 E 	   722.37	   697.83	 12
 14	 Q 	   786.40	   633.31	 11
 15	 G 	   814.91	   569.28	 10
 16	 V 	   864.44	   540.77	  9
 17	 E 	   928.97	   491.24	  8
 18	 V 	   978.50	   426.71	  7
 19	 T 	  1029.02	   377.18	  6
 20	 G 	  1057.53	   326.66	  5
 21	 Q 	  1121.56	   298.15	  4
 22	 Y 	  1203.10	   234.12	  3
 23	 E 	  1267.62	   152.58	  2
 24	 R 	        -  	    88.06	  1  _


+3 charge

		      B   	       Y
  1	 H 	    46.69	        -  	 24
  2	 R 	    98.73	   857.76	 23
  3	 Q 	   141.41	   805.73	 22
  4	 G 	   160.42	   763.04	 21
  5	 P 	   192.77	   744.04	 20
  6	 V 	   225.79	   711.69	 19
  7	 N 	   263.81	   678.66	 18
  8	 L 	   301.50	   640.65	 17
  9	 L 	   339.20	   602.96	 16
 10	 S 	   368.21	   565.26	 15
 11	 D 	   406.55	   536.25	 14
 12	 P 	   438.90	   497.91	 13
 13	 E 	   481.92	   465.56	 12
 14	 Q 	   524.60	   422.54	 11
 15	 G 	   543.61	   379.86	 10
 16	 V 	   576.63	   360.85	  9
 17	 E 	   619.65	   327.83	  8
 18	 V 	   652.67	   284.81	  7
 19	 T 	   686.35	   251.79	  6
 20	 G 	   705.36	   218.11	  5
 21	 Q 	   748.05	   199.10	  4
 22	 Y 	   802.40	   156.41	  3
 23	 E 	   845.41	   102.06	  2
 24	 R 	        -  	    59.04	  1  _
```

**106. IPI00939667 Isoform 1 of Tripeptidyl-peptidase 1**

| Sequence | Charge | M+H+ | XCorr | DeltaCn |
| --- | --- | --- | --- | --- |
| R.LFGGNFAHQASVAR.V | 2 | 1475.636 | 4.358 | 0.456 |

```
+1 charge

		      B   	       Y
  1	 L 	   114.09	        -  	 14
  2	 F 	   261.16	  1361.67	 13
  3	 G 	   318.18	  1214.60	 12
  4	 G 	   375.20	  1157.58	 11
  5	 N 	   489.25	  1100.56	 10
  6	 F 	   636.31	   986.52	  9
  7	 A 	   707.35	   839.45	  8
  8	 H 	   844.41	   768.41	  7
  9	 Q 	   972.47	   631.35	  6
 10	 A 	  1043.51	   503.29	  5
 11	 S 	  1130.54	   432.26	  4
 12	 V 	  1229.61	   345.22	  3
 13	 A 	  1300.64	   246.16	  2
 14	 R 	        -  	   175.12	  1  _
+2 charge

		      B   	       Y
  1	 L 	    57.55	        -  	 14
  2	 F 	   131.08	   681.34	 13
  3	 G 	   159.59	   607.80	 12
  4	 G 	   188.10	   579.29	 11
  5	 N 	   245.13	   550.78	 10
  6	 F 	   318.66	   493.76	  9
  7	 A 	   354.18	   420.23	  8
  8	 H 	   422.71	   384.71	  7
  9	 Q 	   486.74	   316.18	  6
 10	 A 	   522.26	   252.15	  5
 11	 S 	   565.77	   216.63	  4
 12	 V 	   615.31	   173.12	  3
 13	 A 	   650.83	   123.58	  2
 14	 R 	        -  	    88.06	  1  _
```

**107. IPI00025204 CD5 antigen-like**

| Sequence | Charge | M+H+ | XCorr | DeltaCn |
| --- | --- | --- | --- | --- |
| R.ELGCGAASGTPSGILYEPPAEKEQK.V | 3 | 2590.818 | 3.534 | 0.346 |

**+1 charge**

```
		      B   	       Y
  1	 E 	   130.05	        -  	 25
  2	 L 	   243.13	  2460.34	 24
  3	 G 	   300.16	  2347.26	 23
  4	 C 	   460.32	  2290.24	 22
  5	 G 	   517.34	  2130.07	 21
  6	 A 	   588.38	  2073.05	 20
  7	 A 	   659.42	  2002.01	 19
  8	 S 	   746.45	  1930.98	 18
  9	 G 	   803.47	  1843.94	 17
 10	 T 	   904.52	  1786.92	 16
 11	 P 	  1001.57	  1685.87	 15
 12	 S 	  1088.60	  1588.82	 14
 13	 G 	  1145.62	  1501.79	 13
 14	 I 	  1258.71	  1444.77	 12
 15	 L 	  1371.79	  1331.68	 11
 16	 Y 	  1534.86	  1218.60	 10
 17	 E 	  1663.90	  1055.54	  9
 18	 P 	  1760.95	   926.49	  8
 19	 P 	  1858.00	   829.44	  7
 20	 A 	  1929.04	   732.39	  6
 21	 E 	  2058.08	   661.35	  5
 22	 K 	  2186.18	   532.31	  4
 23	 E 	  2315.22	   404.21	  3
 24	 Q 	  2443.28	   275.17	  2
 25	 K 	        -  	   147.11	  1  _


+2 charge
		      B   	       Y
  1	 E 	    65.53	        -  	 25
  2	 L 	   122.07	  1230.67	 24
  3	 G 	   150.58	  1174.13	 23
  4	 C 	   230.66	  1145.62	 22
  5	 G 	   259.17	  1065.54	 21
  6	 A 	   294.69	  1037.03	 20
  7	 A 	   330.21	  1001.51	 19
  8	 S 	   373.73	   965.99	 18
  9	 G 	   402.24	   922.48	 17
 10	 T 	   452.76	   893.96	 16
 11	 P 	   501.29	   843.44	 15
 12	 S 	   544.80	   794.91	 14
 13	 G 	   573.32	   751.40	 13
 14	 I 	   629.86	   722.89	 12
 15	 L 	   686.40	   666.35	 11
 16	 Y 	   767.93	   609.80	 10
 17	 E 	   832.45	   528.27	  9
 18	 P 	   880.98	   463.75	  8
 19	 P 	   929.51	   415.22	  7
 20	 A 	   965.02	   366.70	  6
 21	 E 	  1029.55	   331.18	  5
 22	 K 	  1093.59	   266.66	  4
 23	 E 	  1158.11	   202.61	  3
 24	 Q 	  1222.14	   138.09	  2
 25	 K 	        -  	    74.06	  1  _


+3 charge
		      B   	       Y
  1	 E 	    44.02	        -  	 25
  2	 L 	    81.72	   820.79	 24
  3	 G 	   100.72	   783.09	 23
  4	 C 	   154.11	   764.08	 22
  5	 G 	   173.12	   710.70	 21
  6	 A 	   196.80	   691.69	 20
  7	 A 	   220.48	   668.01	 19
  8	 S 	   249.49	   644.33	 18
  9	 G 	   268.49	   615.32	 17
 10	 T 	   302.18	   596.31	 16
 11	 P 	   334.53	   562.63	 15
 12	 S 	   363.54	   530.28	 14
 13	 G 	   382.55	   501.27	 13
 14	 I 	   420.24	   482.26	 12
 15	 L 	   457.94	   444.57	 11
 16	 Y 	   512.29	   406.87	 10
 17	 E 	   555.30	   352.52	  9
 18	 P 	   587.66	   309.50	  8
 19	 P 	   620.01	   277.15	  7
 20	 A 	   643.68	   244.80	  6
 21	 E 	   686.70	   221.12	  5
 22	 K 	   729.40	   178.11	  4
 23	 E 	   772.41	   135.41	  3
 24	 Q 	   815.10	    92.40	  2
 25	 K 	        -  	    49.71	  1  _
```

**108. IPI00019581 Coagulation factor XII**

| Sequence | Charge | M+H+ | XCorr | DeltaCn |
| --- | --- | --- | --- | --- |
| R.LCHCPVGYTGPFCDVDTK.A | 3 | 2127.32 | 4.449 | 0.482 |

**+1 charge**

```
		      B   	       Y
  1	 L 	   114.09	        -  	 18
  2	 C 	   274.26	  2013.23	 17
  3	 H 	   411.32	  1853.07	 16
  4	 C 	   571.48	  1716.01	 15
  5	 P 	   668.53	  1555.84	 14
  6	 V 	   767.60	  1458.79	 13
  7	 G 	   824.62	  1359.72	 12
  8	 Y 	   987.69	  1302.70	 11
  9	 T 	  1088.73	  1139.64	 10
 10	 G 	  1145.76	  1038.59	  9
 11	 P 	  1242.81	   981.57	  8
 12	 F 	  1389.88	   884.52	  7
 13	 C 	  1550.04	   737.45	  6
 14	 D 	  1665.07	   577.28	  5
 15	 V 	  1764.14	   462.26	  4
 16	 D 	  1879.16	   363.19	  3
 17	 T 	  1980.21	   248.16	  2
 18	 K 	        -  	   147.11	  1  _
+2 charge

		      B   	       Y
  1	 L 	    57.55	        -  	 18
  2	 C 	   137.63	  1007.12	 17
  3	 H 	   206.16	   927.04	 16
  4	 C 	   286.24	   858.51	 15
  5	 P 	   334.77	   778.43	 14
  6	 V 	   384.30	   729.90	 13
  7	 G 	   412.82	   680.37	 12
  8	 Y 	   494.35	   651.85	 11
  9	 T 	   544.87	   570.32	 10
 10	 G 	   573.38	   519.80	  9
 11	 P 	   621.91	   491.29	  8
 12	 F 	   695.44	   442.76	  7
 13	 C 	   775.52	   369.23	  6
 14	 D 	   833.04	   289.15	  5
 15	 V 	   882.57	   231.63	  4
 16	 D 	   940.09	   182.10	  3
 17	 T 	   990.61	   124.58	  2
 18	 K 	        -  	    74.06	  1  _
+3 charge

		      B   	       Y
  1	 L 	    38.70	        -  	 18
  2	 C 	    92.09	   671.75	 17
  3	 H 	   137.78	   618.36	 16
  4	 C 	   191.17	   572.67	 15
  5	 P 	   223.52	   519.29	 14
  6	 V 	   256.54	   486.94	 13
  7	 G 	   275.55	   453.91	 12
  8	 Y 	   329.90	   434.91	 11
  9	 T 	   363.58	   380.55	 10
 10	 G 	   382.59	   346.87	  9
 11	 P 	   414.94	   327.86	  8
 12	 F 	   463.96	   295.51	  7
 13	 C 	   517.35	   246.49	  6
 14	 D 	   555.69	   193.10	  5
 15	 V 	   588.72	   154.76	  4
 16	 D 	   627.06	   121.73	  3
 17	 T 	   660.74	    83.39	  2
 18	 K 	        -  	    49.71	  1  _
```

**109. IPI00247063 Neprilysin**

| Sequence | Charge | M+H+ | XCorr | DeltaCn |
| --- | --- | --- | --- | --- |
| R.LIQNMDATTEPCTDFFK.Y | 2 | 2032.254 | 3.9 | 0.535 |

**+1 charge**

```
		      B   	       Y
  1	 L 	   114.09	        -  	 17
  2	 I 	   227.18	  1917.97	 16
  3	 Q 	   355.23	  1804.89	 15
  4	 N 	   469.28	  1676.83	 14
  5	 M 	   600.32	  1562.78	 13
  6	 D 	   715.34	  1431.74	 12
  7	 A 	   786.38	  1316.72	 11
  8	 T 	   887.43	  1245.68	 10
  9	 T 	   988.48	  1144.63	  9
 10	 E 	  1117.52	  1043.58	  8
 11	 P 	  1214.57	   914.54	  7
 12	 C 	  1374.74	   817.49	  6
 13	 T 	  1475.79	   657.32	  5
 14	 D 	  1590.81	   556.28	  4
 15	 F 	  1737.88	   441.25	  3
 16	 F 	  1884.95	   294.18	  2
 17	 K 	        -  	   147.11	  1  _
+2 charge

		      B   	       Y
  1	 L 	    57.55	        -  	 17
  2	 I 	   114.09	   959.49	 16
  3	 Q 	   178.12	   902.95	 15
  4	 N 	   235.14	   838.92	 14
  5	 M 	   300.66	   781.90	 13
  6	 D 	   358.18	   716.38	 12
  7	 A 	   393.69	   658.86	 11
  8	 T 	   444.22	   623.34	 10
  9	 T 	   494.74	   572.82	  9
 10	 E 	   559.26	   522.30	  8
 11	 P 	   607.79	   457.77	  7
 12	 C 	   687.87	   409.25	  6
 13	 T 	   738.40	   329.17	  5
 14	 D 	   795.91	   278.64	  4
 15	 F 	   869.44	   221.13	  3
 16	 F 	   942.98	   147.59	  2
 17	 K 	        -  	    74.06	  1  _
```

**110. IPI00219217 L-lactate dehydrogenase B chain**

| Sequence | Charge | M+H+ | XCorr | DeltaCn |
| --- | --- | --- | --- | --- |
| K.SADTLWDIQKDLK.D | 2 | 1533.708 | 2.973 | 0.27 |

**+1 charge**

```
		      B   	       Y
  1	 S 	    88.04	        -  	 13
  2	 A 	   159.08	  1445.76	 12
  3	 D 	   274.10	  1374.73	 11
  4	 T 	   375.15	  1259.70	 10
  5	 L 	   488.24	  1158.65	  9
  6	 W 	   674.31	  1045.57	  8
  7	 D 	   789.34	   859.49	  7
  8	 I 	   902.43	   744.46	  6
  9	 Q 	  1030.48	   631.38	  5
 10	 K 	  1158.58	   503.32	  4
 11	 D 	  1273.61	   375.22	  3
 12	 L 	  1386.69	   260.20	  2
 13	 K 	        -  	   147.11	  1  _
+2 charge

		      B   	       Y
  1	 S 	    44.52	        -  	 13
  2	 A 	    80.04	   723.39	 12
  3	 D 	   137.56	   687.87	 11
  4	 T 	   188.08	   630.35	 10
  5	 L 	   244.62	   579.83	  9
  6	 W 	   337.66	   523.29	  8
  7	 D 	   395.17	   430.25	  7
  8	 I 	   451.72	   372.73	  6
  9	 Q 	   515.75	   316.19	  5
 10	 K 	   579.79	   252.16	  4
 11	 D 	   637.31	   188.12	  3
 12	 L 	   693.85	   130.60	  2
 13	 K 	        -  	    74.06	  1  _
```

**111. IPI00219029 Aspartate aminotransferase, cytoplasmic**

| Sequence | Charge | M+H+ | XCorr | DeltaCn |
| --- | --- | --- | --- | --- |
| K.IANDNSLNHEYLPILGLAEFR.S | 3 | 2400.675 | 3.38 | 0.4 |

**+1 charge**

```
		      B   	       Y
  1	 I 	   114.09	        -  	 21
  2	 A 	   185.13	  2286.15	 20
  3	 N 	   299.17	  2215.11	 19
  4	 D 	   414.20	  2101.07	 18
  5	 N 	   528.24	  1986.04	 17
  6	 S 	   615.27	  1872.00	 16
  7	 L 	   728.36	  1784.97	 15
  8	 N 	   842.40	  1671.89	 14
  9	 H 	   979.46	  1557.84	 13
 10	 E 	  1108.50	  1420.78	 12
 11	 Y 	  1271.57	  1291.74	 11
 12	 L 	  1384.65	  1128.68	 10
 13	 P 	  1481.70	  1015.59	  9
 14	 I 	  1594.79	   918.54	  8
 15	 L 	  1707.87	   805.46	  7
 16	 G 	  1764.89	   692.37	  6
 17	 L 	  1877.98	   635.35	  5
 18	 A 	  1949.01	   522.27	  4
 19	 E 	  2078.06	   451.23	  3
 20	 F 	  2225.12	   322.19	  2
 21	 R 	        -  	   175.12	  1  _
+2 charge
		      B   	       Y
  1	 I 	    57.55	        -  	 21
  2	 A 	    93.07	  1143.58	 20
  3	 N 	   150.09	  1108.06	 19
  4	 D 	   207.60	  1051.04	 18
  5	 N 	   264.62	   993.53	 17
  6	 S 	   308.14	   936.50	 16
  7	 L 	   364.68	   892.99	 15
  8	 N 	   421.70	   836.45	 14
  9	 H 	   490.23	   779.42	 13
 10	 E 	   554.75	   710.90	 12
 11	 Y 	   636.29	   646.37	 11
 12	 L 	   692.83	   564.84	 10
 13	 P 	   741.35	   508.30	  9
 14	 I 	   797.90	   459.77	  8
 15	 L 	   854.44	   403.23	  7
 16	 G 	   882.95	   346.69	  6
 17	 L 	   939.49	   318.18	  5
 18	 A 	   975.01	   261.64	  4
 19	 E 	  1039.53	   226.12	  3
 20	 F 	  1113.07	   161.60	  2
 21	 R 	        -  	    88.06	  1  _
+3 charge
		      B   	       Y
  1	 I 	    38.70	        -  	 21
  2	 A 	    62.38	   762.72	 20
  3	 N 	   100.40	   739.04	 19
  4	 D 	   138.74	   701.03	 18
  5	 N 	   176.75	   662.69	 17
  6	 S 	   205.76	   624.67	 16
  7	 L 	   243.46	   595.66	 15
  8	 N 	   281.47	   557.97	 14
  9	 H 	   327.16	   519.95	 13
 10	 E 	   370.17	   474.27	 12
 11	 Y 	   424.53	   431.25	 11
 12	 L 	   462.22	   376.90	 10
 13	 P 	   494.57	   339.20	  9
 14	 I 	   532.27	   306.85	  8
 15	 L 	   569.96	   269.16	  7
 16	 G 	   588.97	   231.46	  6
 17	 L 	   626.66	   212.46	  5
 18	 A 	   650.34	   174.76	  4
 19	 E 	   693.36	   151.08	  3
 20	 F 	   742.38	   108.07	  2
 21	 R 	        -  	    59.04	  1  _
```

**112. IPI00418169 Isoform 2 of Annexin A2**

| Sequence | Charge | M+H+ | XCorr | DeltaCn |
| --- | --- | --- | --- | --- |
| K.SALSGHLETVILGLLK.T | 2 | 1651.972 | 4.62 | 0.456 |

**+1 charge**

```
		      B   	       Y
  1	 S 	    88.04	        -  	 16
  2	 A 	   159.08	  1563.95	 15
  3	 L 	   272.16	  1492.91	 14
  4	 S 	   359.19	  1379.83	 13
  5	 G 	   416.21	  1292.79	 12
  6	 H 	   553.27	  1235.77	 11
  7	 L 	   666.36	  1098.71	 10
  8	 E 	   795.40	   985.63	  9
  9	 T 	   896.45	   856.59	  8
 10	 V 	   995.52	   755.54	  7
 11	 I 	  1108.60	   656.47	  6
 12	 L 	  1221.68	   543.39	  5
 13	 G 	  1278.71	   430.30	  4
 14	 L 	  1391.79	   373.28	  3
 15	 L 	  1504.87	   260.20	  2
 16	 K 	        -  	   147.11	  1  _
+2 charge

		      B   	       Y
  1	 S 	    44.52	        -  	 16
  2	 A 	    80.04	   782.48	 15
  3	 L 	   136.58	   746.96	 14
  4	 S 	   180.10	   690.42	 13
  5	 G 	   208.61	   646.90	 12
  6	 H 	   277.14	   618.39	 11
  7	 L 	   333.68	   549.86	 10
  8	 E 	   398.20	   493.32	  9
  9	 T 	   448.73	   428.80	  8
 10	 V 	   498.26	   378.27	  7
 11	 I 	   554.80	   328.74	  6
 12	 L 	   611.35	   272.20	  5
 13	 G 	   639.86	   215.65	  4
 14	 L 	   696.40	   187.14	  3
 15	 L 	   752.94	   130.60	  2
 16	 K 	        -  	    74.06	  1  _
```

**113. IPI00784473 Isoform 1 of Vacuolar protein sorting-associated protein 13D**

| Sequence | Charge | M+H+ | XCorr | DeltaCn |
| --- | --- | --- | --- | --- |
| R.SHHYVLEPVFASALLK.R | 3 | 1812.104 | 3.379 | 0.211 |

**+1 charge**

```
		      B   	       Y
  1	 S 	    88.04	        -  	 16
  2	 H 	   225.10	  1723.95	 15
  3	 H 	   362.16	  1586.89	 14
  4	 Y 	   525.22	  1449.84	 13
  5	 V 	   624.29	  1286.77	 12
  6	 L 	   737.37	  1187.70	 11
  7	 E 	   866.42	  1074.62	 10
  8	 P 	   963.47	   945.58	  9
  9	 V 	  1062.54	   848.52	  8
 10	 F 	  1209.61	   749.46	  7
 11	 A 	  1280.64	   602.39	  6
 12	 S 	  1367.67	   531.35	  5
 13	 A 	  1438.71	   444.32	  4
 14	 L 	  1551.80	   373.28	  3
 15	 L 	  1664.88	   260.20	  2
 16	 K 	        -  	   147.11	  1  _
+2 charge

		      B   	       Y
  1	 S 	    44.52	        -  	 16
  2	 H 	   113.05	   862.48	 15
  3	 H 	   181.58	   793.95	 14
  4	 Y 	   263.11	   725.42	 13
  5	 V 	   312.65	   643.89	 12
  6	 L 	   369.19	   594.36	 11
  7	 E 	   433.71	   537.81	 10
  8	 P 	   482.24	   473.29	  9
  9	 V 	   531.77	   424.77	  8
 10	 F 	   605.31	   375.23	  7
 11	 A 	   640.82	   301.70	  6
 12	 S 	   684.34	   266.18	  5
 13	 A 	   719.86	   222.66	  4
 14	 L 	   776.40	   187.14	  3
 15	 L 	   832.94	   130.60	  2
 16	 K 	        -  	    74.06	  1  _


+3 charge

		      B   	       Y
  1	 S 	    30.02	        -  	 16
  2	 H 	    75.70	   575.32	 15
  3	 H 	   121.39	   529.64	 14
  4	 Y 	   175.75	   483.95	 13
  5	 V 	   208.77	   429.60	 12
  6	 L 	   246.46	   396.57	 11
  7	 E 	   289.48	   358.88	 10
  8	 P 	   321.83	   315.86	  9
  9	 V 	   354.85	   283.51	  8
 10	 F 	   403.87	   250.49	  7
 11	 A 	   427.55	   201.47	  6
 12	 S 	   456.56	   177.79	  5
 13	 A 	   480.24	   148.78	  4
 14	 L 	   517.94	   125.10	  3
 15	 L 	   555.63	    87.40	  2
 16	 K 	        -  	    49.71	  1  _
```

**114. IPI00030786 Isoform 1 of Matrilin-4**

| Sequence | Charge | M+H+ | XCorr | DeltaCn |
| --- | --- | --- | --- | --- |
| R.LEDLENQLANQK.- | 2 | 1415.531 | 3.351 | 0.317 |

**+1 charge**

```
		      B   	       Y
  1	 L 	   114.09	        -  	 12
  2	 E 	   243.13	  1301.63	 11
  3	 D 	   358.16	  1172.59	 10
  4	 L 	   471.24	  1057.56	  9
  5	 E 	   600.29	   944.48	  8
  6	 N 	   714.33	   815.44	  7
  7	 Q 	   842.39	   701.39	  6
  8	 L 	   955.47	   573.34	  5
  9	 A 	  1026.51	   460.25	  4
 10	 N 	  1140.55	   389.21	  3
 11	 Q 	  1268.61	   275.17	  2
 12	 K 	        -  	   147.11	  1  _
+2 charge

		      B   	       Y
  1	 L 	    57.55	        -  	 12
  2	 E 	   122.07	   651.32	 11
  3	 D 	   179.58	   586.80	 10
  4	 L 	   236.13	   529.29	  9
  5	 E 	   300.65	   472.74	  8
  6	 N 	   357.67	   408.22	  7
  7	 Q 	   421.70	   351.20	  6
  8	 L 	   478.24	   287.17	  5
  9	 A 	   513.76	   230.63	  4
 10	 N 	   570.78	   195.11	  3
 11	 Q 	   634.81	   138.09	  2
 12	 K 	        -  	    74.06	  1  _
```

**115. IPI00009950 Vesicular integral-membrane protein VIP36**

| Sequence | Charge | M+H+ | XCorr | DeltaCn |
| --- | --- | --- | --- | --- |
| R.WTELAGCTADFR.N | 2 | 1427.537 | 4.629 | 0.451 |

**+1 charge**

```
		      B   	       Y
  1	 W 	   187.09	        -  	 12
  2	 T 	   288.13	  1240.70	 11
  3	 E 	   417.18	  1139.65	 10
  4	 L 	   530.26	  1010.61	  9
  5	 A 	   601.30	   897.52	  8
  6	 G 	   658.32	   826.49	  7
  7	 C 	   818.48	   769.46	  6
  8	 T 	   919.53	   609.30	  5
  9	 A 	   990.57	   508.25	  4
 10	 D 	  1105.60	   437.21	  3
 11	 F 	  1252.66	   322.19	  2
 12	 R 	        -  	   175.12	  1  _
+2 charge

		      B   	       Y
  1	 W 	    94.05	        -  	 12
  2	 T 	   144.57	   620.85	 11
  3	 E 	   209.09	   570.33	 10
  4	 L 	   265.63	   505.81	  9
  5	 A 	   301.15	   449.27	  8
  6	 G 	   329.66	   413.75	  7
  7	 C 	   409.75	   385.24	  6
  8	 T 	   460.27	   305.15	  5
  9	 A 	   495.79	   254.63	  4
 10	 D 	   553.30	   219.11	  3
 11	 F 	   626.84	   161.60	  2
 12	 R 	        -  	    88.06	  1  _
```

**116. IPI00002910 Isoform 1 of Transmembrane protein 149**

| Sequence | Charge | M+H+ | XCorr | DeltaCn |
| --- | --- | --- | --- | --- |
| R.FGPPPCPDYEFR.E | 2 | 1482.614 | 2.972 | 0.439 |

```
+1 charge

		      B   	       Y
  1	 F 	   148.08	        -  	 12
  2	 G 	   205.10	  1334.72	 11
  3	 P 	   302.15	  1277.70	 10
  4	 P 	   399.20	  1180.64	  9
  5	 P 	   496.26	  1083.59	  8
  6	 C 	   656.42	   986.54	  7
  7	 P 	   753.47	   826.37	  6
  8	 D 	   868.50	   729.32	  5
  9	 Y 	  1031.56	   614.29	  4
 10	 E 	  1160.61	   451.23	  3
 11	 F 	  1307.67	   322.19	  2
 12	 R 	        -  	   175.12	  1  _
+2 charge

		      B   	       Y
  1	 F 	    74.54	        -  	 12
  2	 G 	   103.05	   667.86	 11
  3	 P 	   151.58	   639.35	 10
  4	 P 	   200.10	   590.83	  9
  5	 P 	   248.63	   542.30	  8
  6	 C 	   328.71	   493.77	  7
  7	 P 	   377.24	   413.69	  6
  8	 D 	   434.75	   365.16	  5
  9	 Y 	   516.29	   307.65	  4
 10	 E 	   580.81	   226.12	  3
 11	 F 	   654.34	   161.60	  2
 12	 R 	        -  	    88.06	  1  _
```

**117. IPI00387113 Ig kappa chain V-III region B6**

| Sequence | Charge | M+H+ | XCorr | DeltaCn |
| --- | --- | --- | --- | --- |
| - .ZIVLTZSPGTLSLSPGZR.A | 2 | 1884.616 | 3.482 | 0.589 |

**+1 charge**

```
		      B   	       Y
  1	 Z 	   129.56	        -  	 18
  2	 I 	   242.64	  1754.96	 17
  3	 V 	   341.71	  1641.88	 16
  4	 L 	   454.79	  1542.81	 15
  5	 T 	   555.84	  1429.73	 14
  6	 Z 	   684.39	  1328.68	 13
  7	 S 	   771.42	  1200.13	 12
  8	 P 	   868.48	  1113.10	 11
  9	 G 	   925.50	  1016.05	 10
 10	 T 	  1026.55	   959.02	  9
 11	 L 	  1139.63	   857.98	  8
 12	 S 	  1226.66	   744.89	  7
 13	 L 	  1339.75	   657.86	  6
 14	 S 	  1426.78	   544.78	  5
 15	 P 	  1523.83	   457.74	  4
 16	 G 	  1580.85	   360.69	  3
 17	 Z 	  1709.40	   303.67	  2
 18	 R 	        -  	   175.12	  1  _
+2 charge
		      B   	       Y
  1	 Z 	    65.28	        -  	 18
  2	 I 	   121.82	   877.99	 17
  3	 V 	   171.36	   821.44	 16
  4	 L 	   227.90	   771.91	 15
  5	 T 	   278.42	   715.37	 14
  6	 Z 	   342.70	   664.84	 13
  7	 S 	   386.22	   600.57	 12
  8	 P 	   434.74	   557.05	 11
  9	 G 	   463.25	   508.53	 10
 10	 T 	   513.78	   480.02	  9
 11	 L 	   570.32	   429.49	  8
 12	 S 	   613.83	   372.95	  7
 13	 L 	   670.38	   329.43	  6
 14	 S 	   713.89	   272.89	  5
 15	 P 	   762.42	   229.38	  4
 16	 G 	   790.93	   180.85	  3
 17	 Z 	   855.21	   152.34	  2
 18	 R 	        -  	    88.06	  1  _
```

**118. IPI00008787 Alpha-N-acetylglucosaminidase**

| Sequence | Charge | M+H+ | XCorr | DeltaCn |
| --- | --- | --- | --- | --- |
| R.LLGPGPAADFSVSVER.A | 2 | 1615.812 | 4.539 | 0.478 |

**+1 charge**

```
		      B   	       Y
  1	 L 	   114.09	        -  	 16
  2	 L 	   227.18	  1501.76	 15
  3	 G 	   284.20	  1388.68	 14
  4	 P 	   381.25	  1331.66	 13
  5	 G 	   438.27	  1234.61	 12
  6	 P 	   535.32	  1177.58	 11
  7	 A 	   606.36	  1080.53	 10
  8	 A 	   677.40	  1009.49	  9
  9	 D 	   792.43	   938.46	  8
 10	 F 	   939.49	   823.43	  7
 11	 S 	  1026.53	   676.36	  6
 12	 V 	  1125.59	   589.33	  5
 13	 S 	  1212.63	   490.26	  4
 14	 V 	  1311.69	   403.23	  3
 15	 E 	  1440.74	   304.16	  2
 16	 R 	        -  	   175.12	  1  _
+2 charge
		      B   	       Y
  1	 L 	    57.55	        -  	 16
  2	 L 	   114.09	   751.39	 15
  3	 G 	   142.60	   694.84	 14
  4	 P 	   191.13	   666.33	 13
  5	 G 	   219.64	   617.81	 12
  6	 P 	   268.17	   589.30	 11
  7	 A 	   303.68	   540.77	 10
  8	 A 	   339.20	   505.25	  9
  9	 D 	   396.72	   469.73	  8
 10	 F 	   470.25	   412.22	  7
 11	 S 	   513.77	   338.68	  6
 12	 V 	   563.30	   295.17	  5
 13	 S 	   606.82	   245.63	  4
 14	 V 	   656.35	   202.12	  3
 15	 E 	   720.87	   152.58	  2
 16	 R 	        -  	    88.06	  1  _
```

**119. IPI00387120 Ig kappa chain V-IV region Len**

| Sequence | Charge | M+H+ | XCorr | DeltaCn |
| --- | --- | --- | --- | --- |
| - .DIVMTQSPDSLAVSLGER.A | 2 | 1919.147 | 5.66 | 0.548 |

**+1 charge**

```
		      B   	       Y
  1	 D 	   116.03	        -  	 18
  2	 I 	   229.12	  1802.93	 17
  3	 V 	   328.19	  1689.85	 16
  4	 M 	   459.23	  1590.78	 15
  5	 T 	   560.27	  1459.74	 14
  6	 Q 	   688.33	  1358.69	 13
  7	 S 	   775.37	  1230.63	 12
  8	 P 	   872.42	  1143.60	 11
  9	 D 	   987.45	  1046.55	 10
 10	 S 	  1074.48	   931.52	  9
 11	 L 	  1187.56	   844.49	  8
 12	 A 	  1258.60	   731.40	  7
 13	 V 	  1357.67	   660.37	  6
 14	 S 	  1444.70	   561.30	  5
 15	 L 	  1557.78	   474.27	  4
 16	 G 	  1614.80	   361.18	  3
 17	 E 	  1743.85	   304.16	  2
 18	 R 	        -  	   175.12	  1  _
+2 charge

		      B   	       Y
  1	 D 	    58.52	        -  	 18
  2	 I 	   115.06	   901.97	 17
  3	 V 	   164.60	   845.43	 16
  4	 M 	   230.12	   795.89	 15
  5	 T 	   280.64	   730.37	 14
  6	 Q 	   344.67	   679.85	 13
  7	 S 	   388.19	   615.82	 12
  8	 P 	   436.71	   572.30	 11
  9	 D 	   494.23	   523.78	 10
 10	 S 	   537.74	   466.26	  9
 11	 L 	   594.28	   422.75	  8
 12	 A 	   629.80	   366.21	  7
 13	 V 	   679.34	   330.69	  6
 14	 S 	   722.85	   281.15	  5
 15	 L 	   779.40	   237.64	  4
 16	 G 	   807.91	   181.10	  3
 17	 E 	   872.43	   152.58	  2
 18	 R 	        -  	    88.06	  1  _
```

**120. IPI00296058 EGF-containing fibulin-like extracellular matrix protein 2**

| Sequence | Charge | M+H+ | XCorr | DeltaCn |
| --- | --- | --- | --- | --- |
| K.CINHYGGYLCLPR.S | 2 | 1623.824 | 3.27 | 0.392 |

**+1 charge**

```
		      B   	       Y
  1	 C 	   161.17	        -  	 13
  2	 I 	   274.26	  1462.86	 12
  3	 N 	   388.30	  1349.78	 11
  4	 H 	   525.36	  1235.73	 10
  5	 Y 	   688.42	  1098.67	  9
  6	 G 	   745.44	   935.61	  8
  7	 G 	   802.46	   878.59	  7
  8	 Y 	   965.53	   821.57	  6
  9	 L 	  1078.61	   658.51	  5
 10	 C 	  1238.78	   545.42	  4
 11	 L 	  1351.86	   385.26	  3
 12	 P 	  1448.91	   272.17	  2
 13	 R 	        -  	   175.12	  1  _
+2 charge

		      B   	       Y
  1	 C 	    81.09	        -  	 13
  2	 I 	   137.63	   731.93	 12
  3	 N 	   194.65	   675.39	 11
  4	 H 	   263.18	   618.37	 10
  5	 Y 	   344.71	   549.84	  9
  6	 G 	   373.23	   468.31	  8
  7	 G 	   401.74	   439.80	  7
  8	 Y 	   483.27	   411.29	  6
  9	 L 	   539.81	   329.76	  5
 10	 C 	   619.89	   273.21	  4
 11	 L 	   676.43	   193.13	  3
 12	 P 	   724.96	   136.59	  2
 13	 R 	        -  	    88.06	  1  _
```

**121. IPI00005142 Isoform 1 of Basic fibroblast growth factor receptor 1**

| Sequence | Charge | M+H+ | XCorr | DeltaCn |
| --- | --- | --- | --- | --- |
| K.IGPDNLPYVQILK.T | 2 | 1470.738 | 3.444 | 0.342 |

**+1 charge**

```
		      B   	       Y
  1	 I 	   114.09	        -  	 13
  2	 G 	   171.11	  1356.75	 12
  3	 P 	   268.17	  1299.73	 11
  4	 D 	   383.19	  1202.68	 10
  5	 N 	   497.24	  1087.65	  9
  6	 L 	   610.32	   973.61	  8
  7	 P 	   707.37	   860.52	  7
  8	 Y 	   870.44	   763.47	  6
  9	 V 	   969.50	   600.41	  5
 10	 Q 	  1097.56	   501.34	  4
 11	 I 	  1210.65	   373.28	  3
 12	 L 	  1323.73	   260.20	  2
 13	 K 	        -  	   147.11	  1  _
+2 charge

		      B   	       Y
  1	 I 	    57.55	        -  	 13
  2	 G 	    86.06	   678.88	 12
  3	 P 	   134.59	   650.37	 11
  4	 D 	   192.10	   601.84	 10
  5	 N 	   249.12	   544.33	  9
  6	 L 	   305.66	   487.31	  8
  7	 P 	   354.19	   430.77	  7
  8	 Y 	   435.72	   382.24	  6
  9	 V 	   485.26	   300.71	  5
 10	 Q 	   549.28	   251.17	  4
 11	 I 	   605.83	   187.14	  3
 12	 L 	   662.37	   130.60	  2
 13	 K 	        -  	    74.06	  1  _
```

**122. IPI00001593 Lysosomal Pro-X carboxypeptidase**

| Sequence | Charge | M+H+ | XCorr | DeltaCn |
| --- | --- | --- | --- | --- |
| K.NALDPMSVLLAR.S | 2 | 1300.553 | 2.901 | 0.458 |

**+1 charge**

```
		      B   	       Y
  1	 N 	   115.05	        -  	 12
  2	 A 	   186.09	  1185.67	 11
  3	 L 	   299.17	  1114.63	 10
  4	 D 	   414.20	  1001.54	  9
  5	 P 	   511.25	   886.52	  8
  6	 M 	   642.29	   789.47	  7
  7	 S 	   729.32	   658.42	  6
  8	 V 	   828.39	   571.39	  5
  9	 L 	   941.48	   472.32	  4
 10	 L 	  1054.56	   359.24	  3
 11	 A 	  1125.60	   246.16	  2
 12	 R 	        -  	   175.12	  1  _
+2 charge

		      B   	       Y
  1	 N 	    58.03	        -  	 12
  2	 A 	    93.55	   593.34	 11
  3	 L 	   150.09	   557.82	 10
  4	 D 	   207.60	   501.28	  9
  5	 P 	   256.13	   443.76	  8
  6	 M 	   321.65	   395.24	  7
  7	 S 	   365.17	   329.72	  6
  8	 V 	   414.70	   286.20	  5
  9	 L 	   471.24	   236.67	  4
 10	 L 	   527.78	   180.12	  3
 11	 A 	   563.30	   123.58	  2
 12	 R 	        -  	    88.06	  1  _
```

**123. IPI00296777 SPARC-like protein 1**

| Sequence | Charge | M+H+ | XCorr | DeltaCn |
| --- | --- | --- | --- | --- |
| K.SIPTCTDFEVIQFPLR.M | 2 | 1924.18 | 3.008 | 0.4 |

**+1 charge**

```
		      B   	       Y
  1	 S 	    88.04	        -  	 16
  2	 I 	   201.12	  1836.07	 15
  3	 P 	   298.18	  1722.99	 14
  4	 T 	   399.22	  1625.93	 13
  5	 C 	   559.39	  1524.89	 12
  6	 T 	   660.44	  1364.72	 11
  7	 D 	   775.46	  1263.67	 10
  8	 F 	   922.53	  1148.65	  9
  9	 E 	  1051.57	  1001.58	  8
 10	 V 	  1150.64	   872.54	  7
 11	 I 	  1263.73	   773.47	  6
 12	 Q 	  1391.79	   660.38	  5
 13	 F 	  1538.85	   532.32	  4
 14	 P 	  1635.91	   385.26	  3
 15	 L 	  1748.99	   288.20	  2
 16	 R 	        -  	   175.12	  1  _
+2 charge

		      B   	       Y
  1	 S 	    44.52	        -  	 16
  2	 I 	   101.07	   918.54	 15
  3	 P 	   149.59	   862.00	 14
  4	 T 	   200.12	   813.47	 13
  5	 C 	   280.20	   762.95	 12
  6	 T 	   330.72	   682.86	 11
  7	 D 	   388.24	   632.34	 10
  8	 F 	   461.77	   574.83	  9
  9	 E 	   526.29	   501.29	  8
 10	 V 	   575.83	   436.77	  7
 11	 I 	   632.37	   387.24	  6
 12	 Q 	   696.40	   330.70	  5
 13	 F 	   769.93	   266.67	  4
 14	 P 	   818.46	   193.13	  3
 15	 L 	   875.00	   144.61	  2
 16	 R 	        -  	    88.06	  1  _
```

**124. IPI00103871 Isoform 1 of Roundabout homolog 4**

| Sequence | Charge | M+H+ | XCorr | DeltaCn |
| --- | --- | --- | --- | --- |
| R.MSCQASGQPPPTIR.W | 2 | 1530.725 | 3.351 | 0.398 |

**+1 charge**

```
		      B   	       Y
  1	 M 	   132.05	        -  	 14
  2	 S 	   219.08	  1398.81	 13
  3	 C 	   379.25	  1311.78	 12
  4	 Q 	   507.30	  1151.62	 11
  5	 A 	   578.34	  1023.56	 10
  6	 S 	   665.37	   952.52	  9
  7	 G 	   722.39	   865.49	  8
  8	 Q 	   850.45	   808.47	  7
  9	 P 	   947.51	   680.41	  6
 10	 P 	  1044.56	   583.36	  5
 11	 P 	  1141.61	   486.30	  4
 12	 T 	  1242.66	   389.25	  3
 13	 I 	  1355.74	   288.20	  2
 14	 R 	        -  	   175.12	  1  _
+2 charge

		      B   	       Y
  1	 M 	    66.53	        -  	 14
  2	 S 	   110.04	   699.91	 13
  3	 C 	   190.13	   656.39	 12
  4	 Q 	   254.16	   576.31	 11
  5	 A 	   289.67	   512.28	 10
  6	 S 	   333.19	   476.76	  9
  7	 G 	   361.70	   433.25	  8
  8	 Q 	   425.73	   404.74	  7
  9	 P 	   474.26	   340.71	  6
 10	 P 	   522.78	   292.18	  5
 11	 P 	   571.31	   243.66	  4
 12	 T 	   621.83	   195.13	  3
 13	 I 	   678.38	   144.61	  2
 14	 R 	        -  	    88.06	  1  _
```

**125. IPI00297252 Isoform 1 of Extracellular sulfatase Sulf-2**

| Sequence | Charge | M+H+ | XCorr | DeltaCn |
| --- | --- | --- | --- | --- |
| R.VYHVGLGDAAQPR.N | 3 | 1383.537 | 3.7 | 0.337 |

**+1 charge**

```
		      B   	       Y
  1	 V 	   100.08	        -  	 13
  2	 Y 	   263.14	  1283.65	 12
  3	 H 	   400.20	  1120.59	 11
  4	 V 	   499.27	   983.53	 10
  5	 G 	   556.29	   884.46	  9
  6	 L 	   669.37	   827.44	  8
  7	 G 	   726.39	   714.35	  7
  8	 D 	   841.42	   657.33	  6
  9	 A 	   912.46	   542.30	  5
 10	 A 	   983.49	   471.27	  4
 11	 Q 	  1111.55	   400.23	  3
 12	 P 	  1208.61	   272.17	  2
 13	 R 	        -  	   175.12	  1  _
+2 charge

		      B   	       Y
  1	 V 	    50.54	        -  	 13
  2	 Y 	   132.07	   642.33	 12
  3	 H 	   200.60	   560.80	 11
  4	 V 	   250.14	   492.27	 10
  5	 G 	   278.65	   442.73	  9
  6	 L 	   335.19	   414.22	  8
  7	 G 	   363.70	   357.68	  7
  8	 D 	   421.21	   329.17	  6
  9	 A 	   456.73	   271.66	  5
 10	 A 	   492.25	   236.14	  4
 11	 Q 	   556.28	   200.62	  3
 12	 P 	   604.81	   136.59	  2
 13	 R 	        -  	    88.06	  1  _


+3 charge

		      B   	       Y
  1	 V 	    34.03	        -  	 13
  2	 Y 	    88.38	   428.55	 12
  3	 H 	   134.07	   374.20	 11
  4	 V 	   167.09	   328.51	 10
  5	 G 	   186.10	   295.49	  9
  6	 L 	   223.80	   276.48	  8
  7	 G 	   242.80	   238.79	  7
  8	 D 	   281.14	   219.78	  6
  9	 A 	   304.82	   181.44	  5
 10	 A 	   328.50	   157.76	  4
 11	 Q 	   371.19	   134.08	  3
 12	 P 	   403.54	    91.40	  2
 13	 R 	        -  	    59.04	  1  _
```

**126. IPI00953695 Ig kappa chain V-II region GM607 (Fragment)**

| Sequence | Charge | M+H+ | XCorr | DeltaCn |
| --- | --- | --- | --- | --- |
| R.ASGVPDRFSGSGSGTDFTLK.I | 2 | 1987.116 | 4.005 | 0.456 |

```
+1 charge
		      B   	       Y
  1	 A 	    72.04	        -  	 20
  2	 S 	   159.08	  1914.92	 19
  3	 G 	   216.10	  1827.89	 18
  4	 V 	   315.17	  1770.87	 17
  5	 P 	   412.22	  1671.80	 16
  6	 D 	   527.25	  1574.74	 15
  7	 R 	   683.35	  1459.72	 14
  8	 F 	   830.42	  1303.62	 13
  9	 S 	   917.45	  1156.55	 12
 10	 G 	   974.47	  1069.52	 11
 11	 S 	  1061.50	  1012.49	 10
 12	 G 	  1118.52	   925.46	  9
 13	 S 	  1205.55	   868.44	  8
 14	 G 	  1262.58	   781.41	  7
 15	 T 	  1363.62	   724.39	  6
 16	 D 	  1478.65	   623.34	  5
 17	 F 	  1625.72	   508.31	  4
 18	 T 	  1726.77	   361.24	  3
 19	 L 	  1839.85	   260.20	  2
 20	 K 	        -  	   147.11	  1  _
+2 charge
		      B   	       Y
  1	 A 	    36.53	        -  	 20
  2	 S 	    80.04	   957.96	 19
  3	 G 	   108.55	   914.45	 18
  4	 V 	   158.09	   885.94	 17
  5	 P 	   206.61	   836.40	 16
  6	 D 	   264.13	   787.88	 15
  7	 R 	   342.18	   730.36	 14
  8	 F 	   415.71	   652.31	 13
  9	 S 	   459.23	   578.78	 12
 10	 G 	   487.74	   535.26	 11
 11	 S 	   531.25	   506.75	 10
 12	 G 	   559.76	   463.23	  9
 13	 S 	   603.28	   434.72	  8
 14	 G 	   631.79	   391.21	  7
 15	 T 	   682.32	   362.70	  6
 16	 D 	   739.83	   312.17	  5
 17	 F 	   813.36	   254.66	  4
 18	 T 	   863.89	   181.13	  3
 19	 L 	   920.43	   130.60	  2
 20	 K 	        -  	    74.06	  1  _
```

**127. IPI00008239 cDNA FLJ55176, highly similar to G-protein coupled receptor family C group 5 member B**

| Sequence | Charge | M+H+ | XCorr | DeltaCn |
| --- | --- | --- | --- | --- |
| R.ETAFEEDVQLPR.A | 2 | 1434.533 | 3.834 | 0.232 |

**+1 charge**

```
		      B   	       Y
  1	 E 	   130.05	        -  	 12
  2	 T 	   231.10	  1304.65	 11
  3	 A 	   302.13	  1203.60	 10
  4	 F 	   449.20	  1132.56	  9
  5	 E 	   578.25	   985.49	  8
  6	 E 	   707.29	   856.45	  7
  7	 D 	   822.32	   727.41	  6
  8	 V 	   921.38	   612.38	  5
  9	 Q 	  1049.44	   513.31	  4
 10	 L 	  1162.53	   385.26	  3
 11	 P 	  1259.58	   272.17	  2
 12	 R 	        -  	   175.12	  1  _
+2 charge

		      B   	       Y
  1	 E 	    65.53	        -  	 12
  2	 T 	   116.05	   652.83	 11
  3	 A 	   151.57	   602.30	 10
  4	 F 	   225.11	   566.79	  9
  5	 E 	   289.63	   493.25	  8
  6	 E 	   354.15	   428.73	  7
  7	 D 	   411.66	   364.21	  6
  8	 V 	   461.20	   306.70	  5
  9	 Q 	   525.22	   257.16	  4
 10	 L 	   581.77	   193.13	  3
 11	 P 	   630.29	   136.59	  2
 12	 R 	        -  	    88.06	  1  _
```

**128. IPI00004416 Charged multivesicular body protein 2a**

| Sequence | Charge | M+H+ | XCorr | DeltaCn |
| --- | --- | --- | --- | --- |
| K.AEAAASALADADADLEER.L | 2 | 1789.836 | 3.621 | 0.361 |

**+1 charge**

```
		      B   	       Y
  1	 A 	    72.04	        -  	 18
  2	 E 	   201.09	  1717.79	 17
  3	 A 	   272.12	  1588.74	 16
  4	 A 	   343.16	  1517.71	 15
  5	 A 	   414.20	  1446.67	 14
  6	 S 	   501.23	  1375.63	 13
  7	 A 	   572.27	  1288.60	 12
  8	 L 	   685.35	  1217.56	 11
  9	 A 	   756.39	  1104.48	 10
 10	 D 	   871.42	  1033.44	  9
 11	 A 	   942.45	   918.42	  8
 12	 D 	  1057.48	   847.38	  7
 13	 A 	  1128.52	   732.35	  6
 14	 D 	  1243.54	   661.32	  5
 15	 L 	  1356.63	   546.29	  4
 16	 E 	  1485.67	   433.20	  3
 17	 E 	  1614.71	   304.16	  2
 18	 R 	        -  	   175.12	  1  _
+2 charge
		      B   	       Y
  1	 A 	    36.53	        -  	 18
  2	 E 	   101.05	   859.40	 17
  3	 A 	   136.57	   794.88	 16
  4	 A 	   172.08	   759.36	 15
  5	 A 	   207.60	   723.84	 14
  6	 S 	   251.12	   688.32	 13
  7	 A 	   286.64	   644.80	 12
  8	 L 	   343.18	   609.29	 11
  9	 A 	   378.70	   552.74	 10
 10	 D 	   436.21	   517.23	  9
 11	 A 	   471.73	   459.71	  8
 12	 D 	   529.24	   424.19	  7
 13	 A 	   564.76	   366.68	  6
 14	 D 	   622.28	   331.16	  5
 15	 L 	   678.82	   273.65	  4
 16	 E 	   743.34	   217.11	  3
 17	 E 	   807.86	   152.58	  2
 18	 R 	        -  	    88.06	  1  _
```

**129. IPI00414320 cDNA FLJ55482, highly similar to Annexin A11**

| Sequence | Charge | M+H+ | XCorr | DeltaCn |
| --- | --- | --- | --- | --- |
| K.GFGTDEQAIIDCLGSR.S | 2 | 1739.859 | 3.083 | 0.44 |

**+1 charge**

```
		      B   	       Y
  1	 G 	    58.03	        -  	 16
  2	 F 	   205.10	  1681.92	 15
  3	 G 	   262.12	  1534.85	 14
  4	 T 	   363.17	  1477.83	 13
  5	 D 	   478.19	  1376.78	 12
  6	 E 	   607.24	  1261.76	 11
  7	 Q 	   735.29	  1132.71	 10
  8	 A 	   806.33	  1004.65	  9
  9	 I 	   919.42	   933.62	  8
 10	 I 	  1032.50	   820.53	  7
 11	 D 	  1147.53	   707.45	  6
 12	 C 	  1307.69	   592.42	  5
 13	 L 	  1420.78	   432.26	  4
 14	 G 	  1477.80	   319.17	  3
 15	 S 	  1564.83	   262.15	  2
 16	 R 	        -  	   175.12	  1  _
+2 charge

		      B   	       Y
  1	 G 	    29.52	        -  	 16
  2	 F 	   103.05	   841.46	 15
  3	 G 	   131.56	   767.93	 14
  4	 T 	   182.09	   739.42	 13
  5	 D 	   239.60	   688.89	 12
  6	 E 	   304.12	   631.38	 11
  7	 Q 	   368.15	   566.86	 10
  8	 A 	   403.67	   502.83	  9
  9	 I 	   460.21	   467.31	  8
 10	 I 	   516.75	   410.77	  7
 11	 D 	   574.27	   354.23	  6
 12	 C 	   654.35	   296.71	  5
 13	 L 	   710.89	   216.63	  4
 14	 G 	   739.40	   160.09	  3
 15	 S 	   782.92	   131.58	  2
 16	 R 	        -  	    88.06	  1  _
```

**130. IPI00745701 Isoform 6 of Deleted in malignant brain tumors 1 protein**

| Sequence | Charge | M+H+ | XCorr | DeltaCn |
| --- | --- | --- | --- | --- |
| K.VDVVLGPIQLQTPPR.R | 2 | 1632.929 | 2.9 | 0.355 |

**+1 charge**

```
		      B   	       Y
  1	 V 	   100.08	        -  	 15
  2	 D 	   215.10	  1532.88	 14
  3	 V 	   314.17	  1417.85	 13
  4	 V 	   413.24	  1318.78	 12
  5	 L 	   526.32	  1219.72	 11
  6	 G 	   583.34	  1106.63	 10
  7	 P 	   680.40	  1049.61	  9
  8	 I 	   793.48	   952.56	  8
  9	 Q 	   921.54	   839.47	  7
 10	 L 	  1034.62	   711.41	  6
 11	 Q 	  1162.68	   598.33	  5
 12	 T 	  1263.73	   470.27	  4
 13	 P 	  1360.78	   369.22	  3
 14	 P 	  1457.84	   272.17	  2
 15	 R 	        -  	   175.12	  1  _
+2 charge

		      B   	       Y
  1	 V 	    50.54	        -  	 15
  2	 D 	   108.05	   766.94	 14
  3	 V 	   157.59	   709.43	 13
  4	 V 	   207.12	   659.90	 12
  5	 L 	   263.67	   610.36	 11
  6	 G 	   292.18	   553.82	 10
  7	 P 	   340.70	   525.31	  9
  8	 I 	   397.24	   476.78	  8
  9	 Q 	   461.27	   420.24	  7
 10	 L 	   517.82	   356.21	  6
 11	 Q 	   581.85	   299.67	  5
 12	 T 	   632.37	   235.64	  4
 13	 P 	   680.90	   185.12	  3
 14	 P 	   729.42	   136.59	  2
 15	 R 	        -  	    88.06	  1  _
```

**131. IPI00218834 Low affinity immunoglobulin gamma Fc region receptor III-A**

| Sequence | Charge | M+H+ | XCorr | DeltaCn |
| --- | --- | --- | --- | --- |
| K.AVVFLEPQWYR.V | 2 | 1408.628 | 3.43 | 0.293 |

**+1 charge**

```
		      B   	       Y
  1	 A 	    72.04	        -  	 11
  2	 V 	   171.11	  1336.70	 10
  3	 V 	   270.18	  1237.64	  9
  4	 F 	   417.25	  1138.57	  8
  5	 L 	   530.33	   991.50	  7
  6	 E 	   659.38	   878.42	  6
  7	 P 	   756.43	   749.37	  5
  8	 Q 	   884.49	   652.32	  4
  9	 W 	  1070.57	   524.26	  3
 10	 Y 	  1233.63	   338.18	  2
 11	 R 	        -  	   175.12	  1  _
+2 charge

		      B   	       Y
  1	 A 	    36.53	        -  	 11
  2	 V 	    86.06	   668.86	 10
  3	 V 	   135.59	   619.32	  9
  4	 F 	   209.13	   569.79	  8
  5	 L 	   265.67	   496.25	  7
  6	 E 	   330.19	   439.71	  6
  7	 P 	   378.72	   375.19	  5
  8	 Q 	   442.75	   326.66	  4
  9	 W 	   535.79	   262.63	  3
 10	 Y 	   617.32	   169.59	  2
 11	 R 	        -  	    88.06	  1  _
```

**132. IPI00017601 Ceruloplasmin**

| Sequence | Charge | M+H+ | XCorr | DeltaCn |
| --- | --- | --- | --- | --- |
| K.MYYSAVDPTKDIFTGLIGPMK.I | 3 | 2348.767 | 4.382 | 0.457 |

**+1 charge**

```
		      B   	       Y
  1	 M 	   132.05	        -  	 21
  2	 Y 	   295.11	  2216.13	 20
  3	 Y 	   458.17	  2053.07	 19
  4	 S 	   545.21	  1890.00	 18
  5	 A 	   616.24	  1802.97	 17
  6	 V 	   715.31	  1731.93	 16
  7	 D 	   830.34	  1632.87	 15
  8	 P 	   927.39	  1517.84	 14
  9	 T 	  1028.44	  1420.79	 13
 10	 K 	  1156.53	  1319.74	 12
 11	 D 	  1271.56	  1191.64	 11
 12	 I 	  1384.65	  1076.62	 10
 13	 F 	  1531.71	   963.53	  9
 14	 T 	  1632.76	   816.46	  8
 15	 G 	  1689.78	   715.42	  7
 16	 L 	  1802.87	   658.40	  6
 17	 I 	  1915.95	   545.31	  5
 18	 G 	  1972.97	   432.23	  4
 19	 P 	  2070.03	   375.21	  3
 20	 M 	  2201.07	   278.15	  2
 21	 K 	        -  	   147.11	  1  _
+2 charge
		      B   	       Y
  1	 M 	    66.53	        -  	 21
  2	 Y 	   148.06	  1108.57	 20
  3	 Y 	   229.59	  1027.04	 19
  4	 S 	   273.11	   945.51	 18
  5	 A 	   308.63	   901.99	 17
  6	 V 	   358.16	   866.47	 16
  7	 D 	   415.67	   816.94	 15
  8	 P 	   464.20	   759.42	 14
  9	 T 	   514.72	   710.90	 13
 10	 K 	   578.77	   660.37	 12
 11	 D 	   636.28	   596.33	 11
 12	 I 	   692.83	   538.81	 10
 13	 F 	   766.36	   482.27	  9
 14	 T 	   816.88	   408.74	  8
 15	 G 	   845.40	   358.21	  7
 16	 L 	   901.94	   329.70	  6
 17	 I 	   958.48	   273.16	  5
 18	 G 	   986.99	   216.62	  4
 19	 P 	  1035.52	   188.11	  3
 20	 M 	  1101.04	   139.58	  2
 21	 K 	        -  	    74.06	  1  _
+3 charge
		      B   	       Y
  1	 M 	    44.69	        -  	 21
  2	 Y 	    99.04	   739.38	 20
  3	 Y 	   153.40	   685.03	 19
  4	 S 	   182.41	   630.67	 18
  5	 A 	   206.09	   601.66	 17
  6	 V 	   239.11	   577.98	 16
  7	 D 	   277.45	   544.96	 15
  8	 P 	   309.80	   506.62	 14
  9	 T 	   343.48	   474.27	 13
 10	 K 	   386.18	   440.58	 12
 11	 D 	   424.53	   397.89	 11
 12	 I 	   462.22	   359.54	 10
 13	 F 	   511.24	   321.85	  9
 14	 T 	   544.93	   272.83	  8
 15	 G 	   563.93	   239.14	  7
 16	 L 	   601.63	   220.14	  6
 17	 I 	   639.32	   182.44	  5
 18	 G 	   658.33	   144.75	  4
 19	 P 	   690.68	   125.74	  3
 20	 M 	   734.36	    93.39	  2
 21	 K 	        -  	    49.71	  1  _
```

**133. IPI00218413 Biotinidase**

| Sequence | Charge | M+H+ | XCorr | DeltaCn |
| --- | --- | --- | --- | --- |
| R.TSIYPFLDFMPSPQVVR.W | 2 | 1998.334 | 3.654 | 0.611 |

**+1 charge**

```
		      B   	       Y
  1	 T 	   102.05	        -  	 17
  2	 S 	   189.09	  1895.97	 16
  3	 I 	   302.17	  1808.94	 15
  4	 Y 	   465.23	  1695.86	 14
  5	 P 	   562.29	  1532.79	 13
  6	 F 	   709.36	  1435.74	 12
  7	 L 	   822.44	  1288.67	 11
  8	 D 	   937.47	  1175.59	 10
  9	 F 	  1084.53	  1060.56	  9
 10	 M 	  1215.58	   913.49	  8
 11	 P 	  1312.63	   782.45	  7
 12	 S 	  1399.66	   685.40	  6
 13	 P 	  1496.71	   598.37	  5
 14	 Q 	  1624.77	   501.31	  4
 15	 V 	  1723.84	   373.26	  3
 16	 V 	  1822.91	   274.19	  2
 17	 R 	        -  	   175.12	  1  _
+2 charge

		      B   	       Y
  1	 T 	    51.53	        -  	 17
  2	 S 	    95.05	   948.49	 16
  3	 I 	   151.59	   904.97	 15
  4	 Y 	   233.12	   848.43	 14
  5	 P 	   281.65	   766.90	 13
  6	 F 	   355.18	   718.37	 12
  7	 L 	   411.72	   644.84	 11
  8	 D 	   469.24	   588.30	 10
  9	 F 	   542.77	   530.78	  9
 10	 M 	   608.29	   457.25	  8
 11	 P 	   656.82	   391.73	  7
 12	 S 	   700.33	   343.20	  6
 13	 P 	   748.86	   299.69	  5
 14	 Q 	   812.89	   251.16	  4
 15	 V 	   862.42	   187.13	  3
 16	 V 	   911.96	   137.60	  2
 17	 R 	        -  	    88.06	  1  _
```

**134. IPI00394992 Isoform 2 of N-acetylmuramoyl-L-alanine amidase**

| Sequence | Charge | M+H+ | XCorr | DeltaCn |
| --- | --- | --- | --- | --- |
| R.EGKEYGVVLAPDGSTVAVEPLLAGLEAGLQGR.R | 3 | 3197.585 | 6.216 | 0.584 |

**+1 charge**

```
		      B   	       Y
  1	 E 	   130.05	        -  	 32
  2	 G 	   187.07	  3066.65	 31
  3	 K 	   315.17	  3009.63	 30
  4	 E 	   444.21	  2881.53	 29
  5	 Y 	   607.27	  2752.49	 28
  6	 G 	   664.29	  2589.42	 27
  7	 V 	   763.36	  2532.40	 26
  8	 V 	   862.43	  2433.33	 25
  9	 L 	   975.51	  2334.27	 24
 10	 A 	  1046.55	  2221.18	 23
 11	 P 	  1143.60	  2150.15	 22
 12	 D 	  1258.63	  2053.09	 21
 13	 G 	  1315.65	  1938.07	 20
 14	 S 	  1402.68	  1881.04	 19
 15	 T 	  1503.73	  1794.01	 18
 16	 V 	  1602.80	  1692.96	 17
 17	 A 	  1673.84	  1593.90	 16
 18	 V 	  1772.91	  1522.86	 15
 19	 E 	  1901.95	  1423.79	 14
 20	 P 	  1999.00	  1294.75	 13
 21	 L 	  2112.09	  1197.69	 12
 22	 L 	  2225.17	  1084.61	 11
 23	 A 	  2296.21	   971.53	 10
 24	 G 	  2353.23	   900.49	  9
 25	 L 	  2466.31	   843.47	  8
 26	 E 	  2595.36	   730.38	  7
 27	 A 	  2666.39	   601.34	  6
 28	 G 	  2723.41	   530.30	  5
 29	 L 	  2836.50	   473.28	  4
 30	 Q 	  2964.56	   360.20	  3
 31	 G 	  3021.58	   232.14	  2
 32	 R 	        -  	   175.12	  1  _


+2 charge

		      B   	       Y
  1	 E 	    65.53	        -  	 32
  2	 G 	    94.04	  1533.83	 31
  3	 K 	   158.09	  1505.32	 30
  4	 E 	   222.61	  1441.27	 29
  5	 Y 	   304.14	  1376.75	 28
  6	 G 	   332.65	  1295.22	 27
  7	 V 	   382.18	  1266.71	 26
  8	 V 	   431.72	  1217.17	 25
  9	 L 	   488.26	  1167.64	 24
 10	 A 	   523.78	  1111.09	 23
 11	 P 	   572.31	  1075.58	 22
 12	 D 	   629.82	  1027.05	 21
 13	 G 	   658.33	   969.54	 20
 14	 S 	   701.85	   941.03	 19
 15	 T 	   752.37	   897.51	 18
 16	 V 	   801.90	   846.99	 17
 17	 A 	   837.42	   797.45	 16
 18	 V 	   886.96	   761.93	 15
 19	 E 	   951.48	   712.40	 14
 20	 P 	  1000.00	   647.88	 13
 21	 L 	  1056.55	   599.35	 12
 22	 L 	  1113.09	   542.81	 11
 23	 A 	  1148.61	   486.27	 10
 24	 G 	  1177.12	   450.75	  9
 25	 L 	  1233.66	   422.24	  8
 26	 E 	  1298.18	   365.70	  7
 27	 A 	  1333.70	   301.17	  6
 28	 G 	  1362.21	   265.66	  5
 29	 L 	  1418.75	   237.15	  4
 30	 Q 	  1482.78	   180.60	  3
 31	 G 	  1511.29	   116.57	  2
 32	 R 	        -  	    88.06	  1  _


+3 charge

		      B   	       Y
  1	 E 	    44.02	        -  	 32
  2	 G 	    63.03	  1022.89	 31
  3	 K 	   105.73	  1003.88	 30
  4	 E 	   148.74	   961.18	 29
  5	 Y 	   203.10	   918.17	 28
  6	 G 	   222.10	   863.81	 27
  7	 V 	   255.13	   844.81	 26
  8	 V 	   288.15	   811.78	 25
  9	 L 	   325.84	   778.76	 24
 10	 A 	   349.52	   741.07	 23
 11	 P 	   381.87	   717.39	 22
 12	 D 	   420.22	   685.04	 21
 13	 G 	   439.22	   646.69	 20
 14	 S 	   468.23	   627.69	 19
 15	 T 	   501.92	   598.68	 18
 16	 V 	   534.94	   564.99	 17
 17	 A 	   558.62	   531.97	 16
 18	 V 	   591.64	   508.29	 15
 19	 E 	   634.65	   475.27	 14
 20	 P 	   667.01	   432.25	 13
 21	 L 	   704.70	   399.90	 12
 22	 L 	   742.39	   362.21	 11
 23	 A 	   766.07	   324.51	 10
 24	 G 	   785.08	   300.83	  9
 25	 L 	   822.78	   281.83	  8
 26	 E 	   865.79	   244.13	  7
 27	 A 	   889.47	   201.12	  6
 28	 G 	   908.48	   177.44	  5
 29	 L 	   946.17	   158.43	  4
 30	 Q 	   988.86	   120.74	  3
 31	 G 	  1007.86	    78.05	  2
 32	 R 	        -  	    59.04	  1  _
```

**135. IPI00006601 Secretogranin-1**

| Sequence | Charge | M+H+ | XCorr | DeltaCn |
| --- | --- | --- | --- | --- |
| R.GEAGAPGEEDIQGPTK.A | 2 | 1556.613 | 3.901 | 0.478 |

**+1 charge**

```
		      B   	       Y
  1	 G 	    58.03	        -  	 16
  2	 E 	   187.07	  1498.70	 15
  3	 A 	   258.11	  1369.66	 14
  4	 G 	   315.13	  1298.62	 13
  5	 A 	   386.17	  1241.60	 12
  6	 P 	   483.22	  1170.56	 11
  7	 G 	   540.24	  1073.51	 10
  8	 E 	   669.28	  1016.49	  9
  9	 E 	   798.33	   887.45	  8
 10	 D 	   913.35	   758.40	  7
 11	 I 	  1026.44	   643.38	  6
 12	 Q 	  1154.50	   530.29	  5
 13	 G 	  1211.52	   402.23	  4
 14	 P 	  1308.57	   345.21	  3
 15	 T 	  1409.62	   248.16	  2
 16	 K 	        -  	   147.11	  1  _
+2 charge

		      B   	       Y
  1	 G 	    29.52	        -  	 16
  2	 E 	    94.04	   749.85	 15
  3	 A 	   129.56	   685.33	 14
  4	 G 	   158.07	   649.81	 13
  5	 A 	   193.59	   621.30	 12
  6	 P 	   242.11	   585.79	 11
  7	 G 	   270.62	   537.26	 10
  8	 E 	   335.15	   508.75	  9
  9	 E 	   399.67	   444.23	  8
 10	 D 	   457.18	   379.71	  7
 11	 I 	   513.72	   322.19	  6
 12	 Q 	   577.75	   265.65	  5
 13	 G 	   606.26	   201.62	  4
 14	 P 	   654.79	   173.11	  3
 15	 T 	   705.31	   124.58	  2
 16	 K 	        -  	    74.06	  1  _
```

**136. IPI00297646 Collagen alpha-1(I) chain**

| Sequence | Charge | M+H+ | XCorr | DeltaCn |
| --- | --- | --- | --- | --- |
| K.SGDRGETGPAGPAGPVGPVGAR.G | 2 | 1963.101 | 3.688 | 0.533 |

```
+1 charge
		      B   	       Y
  1	 S 	    88.04	        -  	 22
  2	 G 	   145.06	  1874.95	 21
  3	 D 	   260.09	  1817.93	 20
  4	 R 	   416.19	  1702.90	 19
  5	 G 	   473.21	  1546.80	 18
  6	 E 	   602.25	  1489.78	 17
  7	 T 	   703.30	  1360.73	 16
  8	 G 	   760.32	  1259.69	 15
  9	 P 	   857.37	  1202.66	 14
 10	 A 	   928.41	  1105.61	 13
 11	 G 	   985.43	  1034.57	 12
 12	 P 	  1082.49	   977.55	 11
 13	 A 	  1153.52	   880.50	 10
 14	 G 	  1210.54	   809.46	  9
 15	 P 	  1307.60	   752.44	  8
 16	 V 	  1406.67	   655.39	  7
 17	 G 	  1463.69	   556.32	  6
 18	 P 	  1560.74	   499.30	  5
 19	 V 	  1659.81	   402.25	  4
 20	 G 	  1716.83	   303.18	  3
 21	 A 	  1787.87	   246.16	  2
 22	 R 	        -  	   175.12	  1  _


+2 charge
		      B   	       Y
  1	 S 	    44.52	        -  	 22
  2	 G 	    73.03	   937.98	 21
  3	 D 	   130.55	   909.47	 20
  4	 R 	   208.60	   851.95	 19
  5	 G 	   237.11	   773.90	 18
  6	 E 	   301.63	   745.39	 17
  7	 T 	   352.15	   680.87	 16
  8	 G 	   380.66	   630.35	 15
  9	 P 	   429.19	   601.84	 14
 10	 A 	   464.71	   553.31	 13
 11	 G 	   493.22	   517.79	 12
 12	 P 	   541.75	   489.28	 11
 13	 A 	   577.27	   440.75	 10
 14	 G 	   605.78	   405.24	  9
 15	 P 	   654.30	   376.72	  8
 16	 V 	   703.84	   328.20	  7
 17	 G 	   732.35	   278.66	  6
 18	 P 	   780.87	   250.15	  5
 19	 V 	   830.41	   201.63	  4
 20	 G 	   858.92	   152.09	  3
 21	 A 	   894.44	   123.58	  2
 22	 R 	        -  	    88.06	  1  _
```

**137. IPI00297056 Cornulin**

| Sequence | Charge | M+H+ | XCorr | DeltaCn |
| --- | --- | --- | --- | --- |
| R.ISPQIQLSGQTEQTQK.A | 2 | 1786.965 | 4.962 | 0.572 |

**+1 charge**

```
		      B   	       Y
  1	 I 	   114.09	        -  	 16
  2	 S 	   201.12	  1672.85	 15
  3	 P 	   298.18	  1585.82	 14
  4	 Q 	   426.23	  1488.77	 13
  5	 I 	   539.32	  1360.71	 12
  6	 Q 	   667.38	  1247.62	 11
  7	 L 	   780.46	  1119.56	 10
  8	 S 	   867.49	  1006.48	  9
  9	 G 	   924.51	   919.45	  8
 10	 Q 	  1052.57	   862.43	  7
 11	 T 	  1153.62	   734.37	  6
 12	 E 	  1282.66	   633.32	  5
 13	 Q 	  1410.72	   504.28	  4
 14	 T 	  1511.77	   376.22	  3
 15	 Q 	  1639.83	   275.17	  2
 16	 K 	        -  	   147.11	  1  _
+2 charge

		      B   	       Y
  1	 I 	    57.55	        -  	 16
  2	 S 	   101.07	   836.93	 15
  3	 P 	   149.59	   793.41	 14
  4	 Q 	   213.62	   744.89	 13
  5	 I 	   270.16	   680.86	 12
  6	 Q 	   334.19	   624.31	 11
  7	 L 	   390.73	   560.29	 10
  8	 S 	   434.25	   503.74	  9
  9	 G 	   462.76	   460.23	  8
 10	 Q 	   526.79	   431.72	  7
 11	 T 	   577.31	   367.69	  6
 12	 E 	   641.84	   317.16	  5
 13	 Q 	   705.86	   252.64	  4
 14	 T 	   756.39	   188.61	  3
 15	 Q 	   820.42	   138.09	  2
 16	 K 	        -  	    74.06	  1  _
```

**138. IPI00896380 Isoform 2 of Ig mu chain C region**

| Sequence | Charge | M+H+ | XCorr | DeltaCn |
| --- | --- | --- | --- | --- |
| K.YAATSQVLLPSKDVMQGTDEHVVCK.V | 3 | 2778.125 | 6.348 | 0.574 |

**+1 charge**

```
		      B   	       Y
  1	 Y 	   164.07	        -  	 25
  2	 A 	   235.11	  2613.44	 24
  3	 A 	   306.14	  2542.40	 23
  4	 T 	   407.19	  2471.36	 22
  5	 S 	   494.22	  2370.31	 21
  6	 Q 	   622.28	  2283.28	 20
  7	 V 	   721.35	  2155.22	 19
  8	 L 	   834.44	  2056.15	 18
  9	 L 	   947.52	  1943.07	 17
 10	 P 	  1044.57	  1829.99	 16
 11	 S 	  1131.60	  1732.93	 15
 12	 K 	  1259.70	  1645.90	 14
 13	 D 	  1374.73	  1517.81	 13
 14	 V 	  1473.79	  1402.78	 12
 15	 M 	  1604.84	  1303.71	 11
 16	 Q 	  1732.89	  1172.67	 10
 17	 G 	  1789.92	  1044.61	  9
 18	 T 	  1890.96	   987.59	  8
 19	 D 	  2005.99	   886.54	  7
 20	 E 	  2135.03	   771.52	  6
 21	 H 	  2272.09	   642.47	  5
 22	 V 	  2371.16	   505.41	  4
 23	 V 	  2470.23	   406.35	  3
 24	 C 	  2630.39	   307.28	  2
 25	 K 	        -  	   147.11	  1  _


+2 charge
		      B   	       Y
  1	 Y 	    82.54	        -  	 25
  2	 A 	   118.06	  1307.22	 24
  3	 A 	   153.58	  1271.70	 23
  4	 T 	   204.10	  1236.18	 22
  5	 S 	   247.62	  1185.66	 21
  6	 Q 	   311.65	  1142.14	 20
  7	 V 	   361.18	  1078.12	 19
  8	 L 	   417.72	  1028.58	 18
  9	 L 	   474.26	   972.04	 17
 10	 P 	   522.79	   915.50	 16
 11	 S 	   566.31	   866.97	 15
 12	 K 	   630.35	   823.45	 14
 13	 D 	   687.87	   759.41	 13
 14	 V 	   737.40	   701.89	 12
 15	 M 	   802.92	   652.36	 11
 16	 Q 	   866.95	   586.84	 10
 17	 G 	   895.46	   522.81	  9
 18	 T 	   945.99	   494.30	  8
 19	 D 	  1003.50	   443.78	  7
 20	 E 	  1068.02	   386.26	  6
 21	 H 	  1136.55	   321.74	  5
 22	 V 	  1186.08	   253.21	  4
 23	 V 	  1235.62	   203.68	  3
 24	 C 	  1315.70	   154.14	  2
 25	 K 	        -  	    74.06	  1  _


+3 charge
		      B   	       Y
  1	 Y 	    55.36	        -  	 25
  2	 A 	    79.04	   871.82	 24
  3	 A 	   102.72	   848.14	 23
  4	 T 	   136.40	   824.46	 22
  5	 S 	   165.41	   790.78	 21
  6	 Q 	   208.10	   761.77	 20
  7	 V 	   241.12	   719.08	 19
  8	 L 	   278.82	   686.06	 18
  9	 L 	   316.51	   648.36	 17
 10	 P 	   348.86	   610.67	 16
 11	 S 	   377.87	   578.32	 15
 12	 K 	   420.57	   549.31	 14
 13	 D 	   458.91	   506.61	 13
 14	 V 	   491.94	   468.26	 12
 15	 M 	   535.62	   435.24	 11
 16	 Q 	   578.30	   391.56	 10
 17	 G 	   597.31	   348.88	  9
 18	 T 	   630.99	   329.87	  8
 19	 D 	   669.33	   296.19	  7
 20	 E 	   712.35	   257.84	  6
 21	 H 	   758.04	   214.83	  5
 22	 V 	   791.06	   169.14	  4
 23	 V 	   824.08	   136.12	  3
 24	 C 	   877.47	   103.10	  2
 25	 K 	        -  	    49.71	  1  _
```

**139. IPI00290315 Chromogranin-A**

| Sequence | Charge | M+H+ | XCorr | DeltaCn |
| --- | --- | --- | --- | --- |
| R.RPEDQELESLSAIEAELEK.V | 2 | 2187.347 | 2.954 | 0.486 |

**+1 charge**

```
		      B   	       Y
  1	 R 	   157.11	        -  	 19
  2	 P 	   254.16	  2029.98	 18
  3	 E 	   383.20	  1932.93	 17
  4	 D 	   498.23	  1803.89	 16
  5	 Q 	   626.29	  1688.86	 15
  6	 E 	   755.33	  1560.80	 14
  7	 L 	   868.42	  1431.76	 13
  8	 E 	   997.46	  1318.67	 12
  9	 S 	  1084.49	  1189.63	 11
 10	 L 	  1197.57	  1102.60	 10
 11	 S 	  1284.61	   989.51	  9
 12	 A 	  1355.64	   902.48	  8
 13	 I 	  1468.73	   831.45	  7
 14	 E 	  1597.77	   718.36	  6
 15	 A 	  1668.81	   589.32	  5
 16	 E 	  1797.85	   518.28	  4
 17	 L 	  1910.93	   389.24	  3
 18	 E 	  2039.98	   276.16	  2
 19	 K 	        -  	   147.11	  1  _
+2 charge
		      B   	       Y
  1	 R 	    79.06	        -  	 19
  2	 P 	   127.58	  1015.49	 18
  3	 E 	   192.11	   966.97	 17
  4	 D 	   249.62	   902.45	 16
  5	 Q 	   313.65	   844.93	 15
  6	 E 	   378.17	   780.90	 14
  7	 L 	   434.71	   716.38	 13
  8	 E 	   499.23	   659.84	 12
  9	 S 	   542.75	   595.32	 11
 10	 L 	   599.29	   551.80	 10
 11	 S 	   642.81	   495.26	  9
 12	 A 	   678.33	   451.75	  8
 13	 I 	   734.87	   416.23	  7
 14	 E 	   799.39	   359.68	  6
 15	 A 	   834.91	   295.16	  5
 16	 E 	   899.43	   259.64	  4
 17	 L 	   955.97	   195.12	  3
 18	 E 	  1020.49	   138.58	  2
 19	 K 	        -  	    74.06	  1  _
```

**140. IPI00018236 Ganglioside GM2 activator**

| Sequence | Charge | M+H+ | XCorr | DeltaCn |
| --- | --- | --- | --- | --- |
| K.SEFVVPDLELPSWLTTGNYR.I | 2 | 2324.573 | 2.899 | 0.593 |

**+1 charge**

```
		      B   	       Y
  1	 S 	    88.04	        -  	 20
  2	 E 	   217.08	  2236.13	 19
  3	 F 	   364.15	  2107.09	 18
  4	 V 	   463.22	  1960.02	 17
  5	 V 	   562.29	  1860.95	 16
  6	 P 	   659.34	  1761.88	 15
  7	 D 	   774.37	  1664.83	 14
  8	 L 	   887.45	  1549.80	 13
  9	 E 	  1016.49	  1436.72	 12
 10	 L 	  1129.58	  1307.67	 11
 11	 P 	  1226.63	  1194.59	 10
 12	 S 	  1313.66	  1097.54	  9
 13	 W 	  1499.74	  1010.51	  8
 14	 L 	  1612.83	   824.43	  7
 15	 T 	  1713.87	   711.34	  6
 16	 T 	  1814.92	   610.29	  5
 17	 G 	  1871.94	   509.25	  4
 18	 N 	  1985.99	   452.23	  3
 19	 Y 	  2149.05	   338.18	  2
 20	 R 	        -  	   175.12	  1  _
+2 charge
		      B   	       Y
  1	 S 	    44.52	        -  	 20
  2	 E 	   109.04	  1118.57	 19
  3	 F 	   182.58	  1054.05	 18
  4	 V 	   232.11	   980.51	 17
  5	 V 	   281.65	   930.98	 16
  6	 P 	   330.17	   881.44	 15
  7	 D 	   387.69	   832.92	 14
  8	 L 	   444.23	   775.40	 13
  9	 E 	   508.75	   718.86	 12
 10	 L 	   565.29	   654.34	 11
 11	 P 	   613.82	   597.80	 10
 12	 S 	   657.33	   549.27	  9
 13	 W 	   750.37	   505.76	  8
 14	 L 	   806.92	   412.72	  7
 15	 T 	   857.44	   356.17	  6
 16	 T 	   907.96	   305.65	  5
 17	 G 	   936.47	   255.13	  4
 18	 N 	   993.50	   226.62	  3
 19	 Y 	  1075.03	   169.59	  2
 20	 R 	        -  	    88.06	  1  _
```

**141. IPI00218075 Protein FAM9B**

| Sequence | Charge | M+H+ | XCorr | DeltaCn |
| --- | --- | --- | --- | --- |
| R.DYIHSLKLLNVLEEYITDEQK.E | 3 | 2564.872 | 3.547 | 0.237 |

**+1 charge**

```
		      B   	       Y
  1	 D 	   116.03	        -  	 21
  2	 Y 	   279.10	  2448.30	 20
  3	 I 	   392.18	  2285.24	 19
  4	 H 	   529.24	  2172.15	 18
  5	 S 	   616.27	  2035.10	 17
  6	 L 	   729.36	  1948.06	 16
  7	 K 	   857.45	  1834.98	 15
  8	 L 	   970.54	  1706.88	 14
  9	 L 	  1083.62	  1593.80	 13
 10	 N 	  1197.66	  1480.72	 12
 11	 V 	  1296.73	  1366.67	 11
 12	 L 	  1409.82	  1267.61	 10
 13	 E 	  1538.86	  1154.52	  9
 14	 E 	  1667.90	  1025.48	  8
 15	 Y 	  1830.96	   896.44	  7
 16	 I 	  1944.05	   733.37	  6
 17	 T 	  2045.10	   620.29	  5
 18	 D 	  2160.12	   519.24	  4
 19	 E 	  2289.16	   404.21	  3
 20	 Q 	  2417.22	   275.17	  2
 21	 K 	        -  	   147.11	  1  _
+2 charge
		      B   	       Y
  1	 D 	    58.52	        -  	 21
  2	 Y 	   140.05	  1224.65	 20
  3	 I 	   196.59	  1143.12	 19
  4	 H 	   265.12	  1086.58	 18
  5	 S 	   308.64	  1018.05	 17
  6	 L 	   365.18	   974.54	 16
  7	 K 	   429.23	   917.99	 15
  8	 L 	   485.77	   853.95	 14
  9	 L 	   542.31	   797.40	 13
 10	 N 	   599.33	   740.86	 12
 11	 V 	   648.87	   683.84	 11
 12	 L 	   705.41	   634.31	 10
 13	 E 	   769.93	   577.76	  9
 14	 E 	   834.45	   513.24	  8
 15	 Y 	   915.99	   448.72	  7
 16	 I 	   972.53	   367.19	  6
 17	 T 	  1023.05	   310.65	  5
 18	 D 	  1080.56	   260.12	  4
 19	 E 	  1145.09	   202.61	  3
 20	 Q 	  1209.12	   138.09	  2
 21	 K 	        -  	    74.06	  1  _
+3 charge

		      B   	       Y
  1	 D 	    39.35	        -  	 21
  2	 Y 	    93.70	   816.77	 20
  3	 I 	   131.40	   762.42	 19
  4	 H 	   177.09	   724.72	 18
  5	 S 	   206.10	   679.04	 17
  6	 L 	   243.79	   650.03	 16
  7	 K 	   286.49	   612.33	 15
  8	 L 	   324.18	   569.63	 14
  9	 L 	   361.88	   531.94	 13
 10	 N 	   399.89	   494.24	 12
 11	 V 	   432.92	   456.23	 11
 12	 L 	   470.61	   423.21	 10
 13	 E 	   513.62	   385.51	  9
 14	 E 	   556.64	   342.50	  8
 15	 Y 	   610.99	   299.48	  7
 16	 I 	   648.69	   245.13	  6
 17	 T 	   682.37	   207.43	  5
 18	 D 	   720.71	   173.75	  4
 19	 E 	   763.73	   135.41	  3
 20	 Q 	   806.41	    92.40	  2
 21	 K 	        -  	    49.71	  1  _
```

**Female 2.**

**1. IPI00027235 Isoform 1 of Attractin**

| Sequence | Charge | M+H+ | XCorr | DeltaCn |
| --- | --- | --- | --- | --- |
| R.SCALDQNCQWEPR.N | 2 | 1664.745 | 3.754 | 0.359 |

+1 charge

```
		      B   	       Y
  1	 S 	    88.04	        -  	13
  2	 C 	   248.20	  1576.93	 12
  3	 A 	   319.24	  1416.77	 11
  4	 L 	   432.33	  1345.73	 10
  5	 D 	   547.35	  1232.65	  9
  6	 Q 	   675.41	  1117.62	  8
  7	 N 	   789.45	   989.56	  7
  8	 C 	   949.62	   875.52	  6
  9	 Q 	  1077.68	   715.35	  5
 10	 W 	  1263.76	   587.29	  4
 11	 E 	  1392.80	   401.21	  3
 12	 P 	  1489.85	   272.17	  2
 13	 R 	        -  	   175.12	  1  _
```

+2 charge

```
		      B   	       Y
  1	 S 	    44.52	        -  	 13
  2	 C 	   124.61	   788.97	 12
  3	 A 	   160.12	   708.89	 11
  4	 L 	   216.67	   673.37	 10
  5	 D 	   274.18	   616.83	  9
  6	 Q 	   338.21	   559.31	  8
  7	 N 	   395.23	   495.28	  7
  8	 C 	   475.31	   438.26	  6
  9	 Q 	   539.34	   358.18	  5
 10	 W 	   632.38	   294.15	  4
 11	 E 	   696.90	   201.11	  3
 12	 P 	   745.43	   136.59	  2
 13	 R 	        -  	    88.06	  1  _
```

**2. IPI00024331 Tumor necrosis factor receptor superfamily member 14**

| Sequence | Charge | M+H+ | XCorr | DeltaCn |
| --- | --- | --- | --- | --- |
| K.CLQCQMCDPAMGLR.A | 2 | 1741.008 | 3.982 | 0.384 |

+1 charge

```
		      B   	       Y
  1	 C 	   161.17	        -  	 14
  2	 L 	   274.26	  1579.95	 13
  3	 Q 	   402.32	  1466.87	 12
  4	 C 	   562.48	  1338.81	 11
  5	 Q 	   690.54	  1178.65	 10
  6	 M 	   821.58	  1050.59	  9
  7	 C 	   981.74	   919.55	  8
  8	 D 	  1096.77	   759.38	  7
  9	 P 	  1193.82	   644.35	  6
 10	 A 	  1264.86	   547.30	  5
 11	 M 	  1395.90	   476.26	  4
 12	 G 	  1452.92	   345.22	  3
 13	 L 	  1566.01	   288.20	  2
 14	 R 	        -  	   175.12	  1  _
+2 charge

		      B   	       Y
  1	 C 	    81.09	        -  	 14
  2	 L 	   137.63	   790.48	 13
  3	 Q 	   201.66	   733.94	 12
  4	 C 	   281.74	   669.91	 11
  5	 Q 	   345.77	   589.83	 10
  6	 M 	   411.29	   525.80	  9
  7	 C 	   491.38	   460.28	  8
  8	 D 	   548.89	   380.19	  7
  9	 P 	   597.42	   322.68	  6
 10	 A 	   632.93	   274.15	  5
 11	 M 	   698.45	   238.64	  4
 12	 G 	   726.97	   173.12	  3
 13	 L 	   783.51	   144.61	  2
 14	 R 	        -  	    88.06	  1  _
```

**3. IPI00553215 V1-5 protein**

| Sequence | Charge | M+H+ | XCorr | DeltaCn |
| --- | --- | --- | --- | --- |
| K.LMIYEVSNRPSGVPDRFSGSK.S | 3 | 2340.644 | 3.66 | 0.46 |

+1 charge

```
		      B   	     Y
  1	 L 	   114.09	        -	      21
  2	 M 	   245.13	  2226.10	 20
  3	 I 	   358.22	  2095.06	 19
  4	 Y 	   521.28	  1981.97	 18
  5	 E 	   650.32	  1818.91	 17
  6	 V 	   749.39	  1689.87	 16
  7	 S 	   836.42	  1590.80	 15
  8	 N 	   950.47	  1503.77	 14
  9	 R 	  1106.57	  1389.72	 13
 10	 P 	  1203.62	  1233.62	 12
 11	 S 	  1290.65	  1136.57	 11
 12	 G 	  1347.67	  1049.54	 10
 13	 V 	  1446.74	   992.52	  9
 14	 P 	  1543.79	   893.45	  8
 15	 D 	  1658.82	   796.39	  7
 16	 R 	  1814.92	   681.37	  6
 17	 F 	  1961.99	   525.27	  5
 18	 S 	  2049.02	   378.20	  4
 19	 G 	  2106.04	   291.17	  3
 20	 S 	  2193.08	   234.14	  2
 21	 K 	        -	        147.11	  1  _
```

+2 charge

```
		      B   	     Y
  1	 L 	    57.55	        -	      21
  2	 M 	   123.07	  1113.55	 20
  3	 I 	   179.61	  1048.03	 19
  4	 Y 	   261.14	   991.49	 18
  5	 E 	   325.66	   909.96	 17
  6	 V 	   375.20	   845.44	 16
  7	 S 	   418.71	   795.90	 15
  8	 N 	   475.74	   752.39	 14
  9	 R 	   553.79	   695.37	 13
 10	 P 	   602.31	   617.31	 12
 11	 S 	   645.83	   568.79	 11
 12	 G 	   674.34	   525.27	 10
 13	 V 	   723.87	   496.76	  9
 14	 P 	   772.40	   447.23	  8
 15	 D 	   829.91	   398.70	  7
 16	 R 	   907.96	   341.19	  6
 17	 F 	   981.50	   263.14	  5
 18	 S 	  1025.01	   189.60	  4
 19	 G 	  1053.53	   146.09	  3
 20	 S 	  1097.04	   117.58	  2
 21	 K 	        -	         74.06	  1  _
```

+3 charge

```
		       B   	      Y
  1	 L 	    38.70	        -	      21
  2	 M 	    82.38	   742.70	 20
  3	 I 	   120.08	   699.02	 19
  4	 Y 	   174.43	   661.33	 18
  5	 E 	   217.45	   606.97	 17
  6	 V 	   250.47	   563.96	 16
  7	 S 	   279.48	   530.94	 15
  8	 N 	   317.49	   501.93	 14
  9	 R 	   369.53	   463.91	 13
 10	 P 	   401.88	   411.88	 12
 11	 S 	   430.89	   379.53	 11
 12	 G 	   449.90	   350.52	 10
 13	 V 	   482.92	   331.51	  9
 14	 P 	   515.27	   298.49	  8
 15	 D 	   553.61	   266.14	  7
 16	 R 	   605.65	   227.79	  6
 17	 F 	   654.67	   175.76	  5
 18	 S 	   683.68	   126.74	  4
 19	 G 	   702.69	    97.73	  3
 20	 S 	   731.70	    78.72	  2
```

21	K	-	49.71	1 _

**4. IPI00009950 Vesicular integral-membrane protein VIP36**

| Sequence | Charge | M+H+ | XCorr | DeltaCn |
| --- | --- | --- | --- | --- |
| R.WTELAGCTADFR.N | 2 | 1427.537 | 3.125 | 0.454 |

+1 charge

```
		      B   	       Y
  1	 W 	   187.09	        -  	 12
  2	 T 	   288.13	  1240.70	 11
  3	 E 	   417.18	  1139.65	 10
  4	 L 	   530.26	  1010.61	  9
  5	 A 	   601.30	   897.52	  8
  6	 G 	   658.32	   826.49	  7
  7	 C 	   818.48	   769.46	  6
  8	 T 	   919.53	   609.30	  5
  9	 A 	   990.57	   508.25	  4
 10	 D 	  1105.60	   437.21	  3
 11	 F 	  1252.66	   322.19	  2
 12	 R 	        -  	   175.12	  1  _
+2 charge
		      B   	       Y
  1	 W 	    94.05	        -  	 12
  2	 T 	   144.57	   620.85	 11
  3	 E 	   209.09	   570.33	 10
  4	 L 	   265.63	   505.81	  9
  5	 A 	   301.15	   449.27	  8
  6	 G 	   329.66	   413.75	  7
  7	 C 	   409.75	   385.24	  6
  8	 T 	   460.27	   305.15	  5
  9	 A 	   495.79	   254.63	  4
 10	 D 	   553.30	   219.11	  3
 11	 F 	   626.84	   161.60	  2
 12	 R 	        -  	    88.06	  1  _
```

**5. IPI00019157 Chondroitin sulfate proteoglycan 4**

| Sequence | Charge | M+H+ | XCorr | DeltaCn |
| --- | --- | --- | --- | --- |
| R.GSLLLGGLDAEASR.H | 2 | 1359.511 | 4.037 | 0.52 |

+1 charge

```
		      B   	       Y
  1	 G 	    58.03	        -  	 14
  2	 S 	   145.06	  1301.71	 13
  3	 L 	   258.14	  1214.67	 12
  4	 L 	   371.23	  1101.59	 11
  5	 L 	   484.31	   988.51	 10
  6	 G 	   541.33	   875.42	  9
  7	 G 	   598.36	   818.40	  8
  8	 L 	   711.44	   761.38	  7
  9	 D 	   826.47	   648.29	  6
 10	 A 	   897.50	   533.27	  5
 11	 E 	  1026.55	   462.23	  4
 12	 A 	  1097.58	   333.19	  3
 13	 S 	  1184.62	   262.15	  2
 14	 R 	        -  	   175.12	  1  _
+2 charge
		      B   	       Y
  1	 G 	    29.52	        -  	 14
  2	 S 	    73.03	   651.36	 13
  3	 L 	   129.58	   607.84	 12
  4	 L 	   186.12	   551.30	 11
  5	 L 	   242.66	   494.76	 10
  6	 G 	   271.17	   438.21	  9
  7	 G 	   299.68	   409.70	  8
  8	 L 	   356.22	   381.19	  7
  9	 D 	   413.74	   324.65	  6
 10	 A 	   449.26	   267.14	  5
 11	 E 	   513.78	   231.62	  4
 12	 A 	   549.30	   167.10	  3
 13	 S 	   592.81	   131.58	  2
 14	 R 	        -  	    88.06	  1  _
```

**6. IPI00387120 Ig kappa chain V-IV region Len**

| Sequence | Charge | M+H+ | XCorr | DeltaCn |
| --- | --- | --- | --- | --- |
| - .DIVMTQSPDSLAVSLGER.A | 2 | 1919.147 | 5.805 | 0.565 |

+1 charge

```
		      B   	       Y
  1	 D 	   116.03	        -  	 18
  2	 I 	   229.12	  1802.93	 17
  3	 V 	   328.19	  1689.85	 16
  4	 M 	   459.23	  1590.78	 15
  5	 T 	   560.27	  1459.74	 14
  6	 Q 	   688.33	  1358.69	 13
  7	 S 	   775.37	  1230.63	 12
  8	 P 	   872.42	  1143.60	 11
  9	 D 	   987.45	  1046.55	 10
 10	 S 	  1074.48	   931.52	  9
 11	 L 	  1187.56	   844.49	  8
 12	 A 	  1258.60	   731.40	  7
 13	 V 	  1357.67	   660.37	  6
 14	 S 	  1444.70	   561.30	  5
 15	 L 	  1557.78	   474.27	  4
 16	 G 	  1614.80	   361.18	  3
 17	 E 	  1743.85	   304.16	  2
 18	 R 	        -  	   175.12	  1  _
+2 charge
		      B   	       Y
  1	 D 	    58.52	        -  	 18
  2	 I 	   115.06	   901.97	 17
  3	 V 	   164.60	   845.43	 16
  4	 M 	   230.12	   795.89	 15
  5	 T 	   280.64	   730.37	 14
  6	 Q 	   344.67	   679.85	 13
  7	 S 	   388.19	   615.82	 12
  8	 P 	   436.71	   572.30	 11
  9	 D 	   494.23	   523.78	 10
 10	 S 	   537.74	   466.26	  9
 11	 L 	   594.28	   422.75	  8
 12	 A 	   629.80	   366.21	  7
 13	 V 	   679.34	   330.69	  6
 14	 S 	   722.85	   281.15	  5
 15	 L 	   779.40	   237.64	  4
 16	 G 	   807.91	   181.10	  3
 17	 E 	   872.43	   152.58	  2
 18	 R 	        -  	    88.06	  1  _
```

**7. IPI00736885 Ig kappa chain V-II region TEW**

| Sequence | Charge | M+H+ | XCorr | DeltaCn |
| --- | --- | --- | --- | --- |
| - .DIVMTQSPLSLPVTPGEPASISCR.S | 3 | 2556.911 | 3.452 | 0.485 |

+1 charge

```
		      B   	       Y
  1	 D 	   116.03	        -  	 24
  2	 I 	   229.12	  2440.39	 23
  3	 V 	   328.19	  2327.31	 22
  4	 M 	   459.23	  2228.24	 21
  5	 T 	   560.27	  2097.20	 20
  6	 Q 	   688.33	  1996.15	 19
  7	 S 	   775.37	  1868.09	 18
  8	 P 	   872.42	  1781.06	 17
  9	 L 	   985.50	  1684.01	 16
 10	 S 	  1072.53	  1570.92	 15
 11	 L 	  1185.62	  1483.89	 14
 12	 P 	  1282.67	  1370.81	 13
 13	 V 	  1381.74	  1273.76	 12
 14	 T 	  1482.79	  1174.69	 11
 15	 P 	  1579.84	  1073.64	 10
 16	 G 	  1636.86	   976.59	  9
 17	 E 	  1765.90	   919.56	  8
 18	 P 	  1862.96	   790.52	  7
 19	 A 	  1933.99	   693.47	  6
 20	 S 	  2021.03	   622.43	  5
 21	 I 	  2134.11	   535.40	  4
 22	 S 	  2221.14	   422.32	  3
 23	 C 	  2381.31	   335.28	  2
 24	 R 	        -  	   175.12	  1  _


+2 charge
		      B   	       Y
  1	 D 	    58.52	        -  	 24
  2	 I 	   115.06	  1220.70	 23
  3	 V 	   164.60	  1164.16	 22
  4	 M 	   230.12	  1114.62	 21
  5	 T 	   280.64	  1049.10	 20
  6	 Q 	   344.67	   998.58	 19
  7	 S 	   388.19	   934.55	 18
  8	 P 	   436.71	   891.03	 17
  9	 L 	   493.25	   842.51	 16
 10	 S 	   536.77	   785.97	 15
 11	 L 	   593.31	   742.45	 14
 12	 P 	   641.84	   685.91	 13
 13	 V 	   691.37	   637.38	 12
 14	 T 	   741.90	   587.85	 11
 15	 P 	   790.42	   537.32	 10
 16	 G 	   818.93	   488.80	  9
 17	 E 	   883.46	   460.29	  8
 18	 P 	   931.98	   395.76	  7
 19	 A 	   967.50	   347.24	  6
 20	 S 	  1011.02	   311.72	  5
 21	 I 	  1067.56	   268.20	  4
 22	 S 	  1111.07	   211.66	  3
 23	 C 	  1191.16	   168.15	  2
 24	 R 	        -  	    88.06	  1  _


+3 charge
		      B   	       Y
  1	 D 	    39.35	        -  	 24
  2	 I 	    77.04	   814.14	 23
  3	 V 	   110.07	   776.44	 22
  4	 M 	   153.75	   743.42	 21
  5	 T 	   187.43	   699.74	 20
  6	 Q 	   230.12	   666.06	 19
  7	 S 	   259.13	   623.37	 18
  8	 P 	   291.48	   594.36	 17
  9	 L 	   329.17	   562.01	 16
 10	 S 	   358.18	   524.31	 15
 11	 L 	   395.88	   495.30	 14
 12	 P 	   428.23	   457.61	 13
 13	 V 	   461.25	   425.26	 12
 14	 T 	   494.93	   392.23	 11
 15	 P 	   527.28	   358.55	 10
 16	 G 	   546.29	   326.20	  9
 17	 E 	   589.31	   307.19	  8
 18	 P 	   621.66	   264.18	  7
 19	 A 	   645.34	   231.83	  6
 20	 S 	   674.35	   208.15	  5
 21	 I 	   712.04	   179.14	  4
 22	 S 	   741.05	   141.44	  3
 23	 C 	   794.44	   112.43	  2
 24	 R 	        -  	    59.04	  1  _
```

**8. IPI00032179 Antithrombin-III**

| Sequence | Charge | M+H+ | XCorr | DeltaCn |
| --- | --- | --- | --- | --- |
| R.EVPLNTIIFMGR.V | 2 | 1390.676 | 3.135 | 0.559 |

+1 charge

```
		      B   	       Y
  1	 E 	   130.05	        -  	 12
  2	 V 	   229.12	  1260.71	 11
  3	 P 	   326.17	  1161.64	 10
  4	 L 	   439.26	  1064.59	  9
  5	 N 	   553.30	   951.51	  8
  6	 T 	   654.35	   837.47	  7
  7	 I 	   767.43	   736.42	  6
  8	 I 	   880.51	   623.33	  5
  9	 F 	  1027.58	   510.25	  4
 10	 M 	  1158.62	   363.18	  3
 11	 G 	  1215.64	   232.14	  2
 12	 R 	        -  	   175.12	  1  _
+2 charge
		      B   	       Y
  1	 E 	    65.53	        -  	 12
  2	 V 	   115.06	   630.86	 11
  3	 P 	   163.59	   581.33	 10
  4	 L 	   220.13	   532.80	  9
  5	 N 	   277.15	   476.26	  8
  6	 T 	   327.68	   419.24	  7
  7	 I 	   384.22	   368.71	  6
  8	 I 	   440.76	   312.17	  5
  9	 F 	   514.29	   255.63	  4
 10	 M 	   579.82	   182.09	  3
 11	 G 	   608.33	   116.57	  2
 12	 R 	        -  	    88.06	  1  _
```

**9. IPI00032258 Complement C4-A**

| Sequence | Charge | M+H+ | XCorr | DeltaCn |
| --- | --- | --- | --- | --- |
| R.VTASDPLDTLGSEGALSPGGVASLLRLPR.G | 3 | 2851.205 | 3.899 | 0.505 |

+1 charge

```
		      B   	       Y
  1	 V 	   100.08	        -  	 29
  2	 T 	   201.12	  2750.47	 28
  3	 A 	   272.16	  2649.42	 27
  4	 S 	   359.19	  2578.38	 26
  5	 D 	   474.22	  2491.35	 25
  6	 P 	   571.27	  2376.32	 24
  7	 L 	   684.36	  2279.27	 23
  8	 D 	   799.38	  2166.19	 22
  9	 T 	   900.43	  2051.16	 21
 10	 L 	  1013.51	  1950.11	 20
 11	 G 	  1070.54	  1837.03	 19
 12	 S 	  1157.57	  1780.01	 18
 13	 E 	  1286.61	  1692.98	 17
 14	 G 	  1343.63	  1563.93	 16
 15	 A 	  1414.67	  1506.91	 15
 16	 L 	  1527.75	  1435.87	 14
 17	 S 	  1614.79	  1322.79	 13
 18	 P 	  1711.84	  1235.76	 12
 19	 G 	  1768.86	  1138.71	 11
 20	 G 	  1825.88	  1081.68	 10
 21	 V 	  1924.95	  1024.66	  9
 22	 A 	  1995.99	   925.59	  8
 23	 S 	  2083.02	   854.56	  7
 24	 L 	  2196.10	   767.53	  6
 25	 L 	  2309.19	   654.44	  5
 26	 R 	  2465.29	   541.36	  4
 27	 L 	  2578.37	   385.26	  3
 28	 P 	  2675.43	   272.17	  2
 29	 R 	        -  	   175.12	  1  _


+2 charge
		      B   	       Y
  1	 V 	    50.54	        -  	 29
  2	 T 	   101.07	  1375.74	 28
  3	 A 	   136.58	  1325.21	 27
  4	 S 	   180.10	  1289.70	 26
  5	 D 	   237.61	  1246.18	 25
  6	 P 	   286.14	  1188.67	 24
  7	 L 	   342.68	  1140.14	 23
  8	 D 	   400.20	  1083.60	 22
  9	 T 	   450.72	  1026.08	 21
 10	 L 	   507.26	   975.56	 20
 11	 G 	   535.77	   919.02	 19
 12	 S 	   579.29	   890.51	 18
 13	 E 	   643.81	   846.99	 17
 14	 G 	   672.32	   782.47	 16
 15	 A 	   707.84	   753.96	 15
 16	 L 	   764.38	   718.44	 14
 17	 S 	   807.90	   661.90	 13
 18	 P 	   856.42	   618.38	 12
 19	 G 	   884.93	   569.86	 11
 20	 G 	   913.44	   541.35	 10
 21	 V 	   962.98	   512.83	  9
 22	 A 	   998.50	   463.30	  8
 23	 S 	  1042.01	   427.78	  7
 24	 L 	  1098.56	   384.27	  6
 25	 L 	  1155.10	   327.72	  5
 26	 R 	  1233.15	   271.18	  4
 27	 L 	  1289.69	   193.13	  3
 28	 P 	  1338.22	   136.59	  2
 29	 R 	        -  	    88.06	  1  _


+3 charge
		      B   	       Y
  1	 V 	    34.03	        -  	 29
  2	 T 	    67.71	   917.49	 28
  3	 A 	    91.39	   883.81	 27
  4	 S 	   120.40	   860.13	 26
  5	 D 	   158.74	   831.12	 25
  6	 P 	   191.10	   792.78	 24
  7	 L 	   228.79	   760.43	 23
  8	 D 	   267.13	   722.73	 22
  9	 T 	   300.82	   684.39	 21
 10	 L 	   338.51	   650.71	 20
 11	 G 	   357.52	   613.01	 19
 12	 S 	   386.53	   594.01	 18
 13	 E 	   429.54	   565.00	 17
 14	 G 	   448.55	   521.98	 16
 15	 A 	   472.23	   502.98	 15
 16	 L 	   509.92	   479.30	 14
 17	 S 	   538.93	   441.60	 13
 18	 P 	   571.28	   412.59	 12
 19	 G 	   590.29	   380.24	 11
 20	 G 	   609.30	   361.23	 10
 21	 V 	   642.32	   342.23	  9
 22	 A 	   666.00	   309.20	  8
 23	 S 	   695.01	   285.52	  7
 24	 L 	   732.71	   256.51	  6
 25	 L 	   770.40	   218.82	  5
 26	 R 	   822.43	   181.12	  4
 27	 L 	   860.13	   129.09	  3
 28	 P 	   892.48	    91.40	  2
 29	 R 	        -  	    59.04	  1  _
```

**10. IPI00017601 Ceruloplasmin**

| Sequence | Charge | M+H+ | XCorr | DeltaCn |
| --- | --- | --- | --- | --- |
| R.FNKNNEGTYYSPNYNPQSR.S | 3 | 2294.381 | 3.46 | 0.286 |

+1 charge

```
		      B   	       Y
  1	 F 	   148.08	        -  	 19
  2	 N 	   262.12	  2145.96	 18
  3	 K 	   390.21	  2031.92	 17
  4	 N 	   504.26	  1903.82	 16
  5	 N 	   618.30	  1789.78	 15
  6	 E 	   747.34	  1675.73	 14
  7	 G 	   804.36	  1546.69	 13
  8	 T 	   905.41	  1489.67	 12
  9	 Y 	  1068.47	  1388.62	 11
 10	 Y 	  1231.54	  1225.56	 10
 11	 S 	  1318.57	  1062.50	  9
 12	 P 	  1415.62	   975.46	  8
 13	 N 	  1529.67	   878.41	  7
 14	 Y 	  1692.73	   764.37	  6
 15	 N 	  1806.77	   601.31	  5
 16	 P 	  1903.82	   487.26	  4
 17	 Q 	  2031.88	   390.21	  3
 18	 S 	  2118.92	   262.15	  2
 19	 R 	        -  	   175.12	  1  _
+2 charge
		      B   	       Y
  1	 F 	    74.54	        -  	 19
  2	 N 	   131.56	  1073.48	 18
  3	 K 	   195.61	  1016.46	 17
  4	 N 	   252.63	   952.41	 16
  5	 N 	   309.65	   895.39	 15
  6	 E 	   374.17	   838.37	 14
  7	 G 	   402.69	   773.85	 13
  8	 T 	   453.21	   745.34	 12
  9	 Y 	   534.74	   694.82	 11
 10	 Y 	   616.27	   613.28	 10
 11	 S 	   659.79	   531.75	  9
 12	 P 	   708.31	   488.24	  8
 13	 N 	   765.34	   439.71	  7
 14	 Y 	   846.87	   382.69	  6
 15	 N 	   903.89	   301.16	  5
 16	 P 	   952.42	   244.13	  4
 17	 Q 	  1016.45	   195.61	  3
 18	 S 	  1059.96	   131.58	  2
 19	 R 	        -  	    88.06	  1  _

+3 charge
		      B   	       Y
  1	 F 	    50.03	        -  	 19
  2	 N 	    88.04	   715.99	 18
  3	 K 	   130.74	   677.98	 17
  4	 N 	   168.76	   635.28	 16
  5	 N 	   206.77	   597.26	 15
  6	 E 	   249.79	   559.25	 14
  7	 G 	   268.79	   516.24	 13
  8	 T 	   302.48	   497.23	 12
  9	 Y 	   356.83	   463.55	 11
 10	 Y 	   411.18	   409.19	 10
 11	 S 	   440.19	   354.84	  9
 12	 P 	   472.55	   325.83	  8
 13	 N 	   510.56	   293.48	  7
 14	 Y 	   564.91	   255.46	  6
 15	 N 	   602.93	   201.11	  5
 16	 P 	   635.28	   163.09	  4
 17	 Q 	   677.97	   130.74	  3
 18	 S 	   706.98	    88.06	  2
 19	 R 	        -  	    59.04	  1  _
```

**11. IPI00152018 Isoform 1 of CMRF35-like molecule 9**

| Sequence | Charge | M+H+ | XCorr | DeltaCn |
| --- | --- | --- | --- | --- |
| R.CSGTIYAEEEGQETMKGR.V | 2 | 2047.184 | 3.087 | 0.599 |

+1 charge

```
		      B   	       Y
  1	 C 	   161.17	        -  	 18
  2	 S 	   248.20	  1885.86	 17
  3	 G 	   305.23	  1798.83	 16
  4	 T 	   406.27	  1741.81	 15
  5	 I 	   519.36	  1640.76	 14
  6	 Y 	   682.42	  1527.67	 13
  7	 A 	   753.46	  1364.61	 12
  8	 E 	   882.50	  1293.57	 11
  9	 E 	  1011.54	  1164.53	 10
 10	 E 	  1140.59	  1035.49	  9
 11	 G 	  1197.61	   906.45	  8
 12	 Q 	  1325.67	   849.42	  7
 13	 E 	  1454.71	   721.37	  6
 14	 T 	  1555.76	   592.32	  5
 15	 M 	  1686.80	   491.28	  4
 16	 K 	  1814.89	   360.24	  3
 17	 G 	  1871.91	   232.14	  2
 18	 R 	        -  	   175.12	  1  _
+2 charge
		      B   	       Y
  1	 C 	    81.09	        -  	 18
  2	 S 	   124.61	   943.43	 17
  3	 G 	   153.12	   899.92	 16
  4	 T 	   203.64	   871.41	 15
  5	 I 	   260.18	   820.88	 14
  6	 Y 	   341.71	   764.34	 13
  7	 A 	   377.23	   682.81	 12
  8	 E 	   441.75	   647.29	 11
  9	 E 	   506.28	   582.77	 10
 10	 E 	   570.80	   518.25	  9
 11	 G 	   599.31	   453.73	  8
 12	 Q 	   663.34	   425.22	  7
 13	 E 	   727.86	   361.19	  6
 14	 T 	   778.38	   296.67	  5
 15	 M 	   843.90	   246.14	  4
 16	 K 	   907.95	   180.62	  3
 17	 G 	   936.46	   116.57	  2
 18	 R 	        -  	    88.06	  1  _
```

**12. IPI00004101 Betaine--homocysteine S-methyltransferase 1**

| Sequence | Charge | M+H+ | XCorr | DeltaCn |
| --- | --- | --- | --- | --- |
| K.ISGQEVNEAACDIAR.Q | 2 | 1633.736 | 3.154 | 0.389 |

+1 charge

```
		      B   	       Y
  1	 I 	   114.09	        -  	 15
  2	 S 	   201.12	  1519.82	 14
  3	 G 	   258.14	  1432.78	 13
  4	 Q 	   386.20	  1375.76	 12
  5	 E 	   515.25	  1247.70	 11
  6	 V 	   614.31	  1118.66	 10
  7	 N 	   728.36	  1019.59	  9
  8	 E 	   857.40	   905.55	  8
  9	 A 	   928.44	   776.51	  7
 10	 A 	   999.47	   705.47	  6
 11	 C 	  1159.64	   634.43	  5
 12	 D 	  1274.67	   474.27	  4
 13	 I 	  1387.75	   359.24	  3
 14	 A 	  1458.79	   246.16	  2
 15	 R 	        -  	   175.12	  1  _
+2 charge
		      B   	       Y
  1	 I 	    57.55	        -  	 15
  2	 S 	   101.07	   760.41	 14
  3	 G 	   129.58	   716.90	 13
  4	 Q 	   193.61	   688.38	 12
  5	 E 	   258.13	   624.36	 11
  6	 V 	   307.66	   559.83	 10
  7	 N 	   364.68	   510.30	  9
  8	 E 	   429.20	   453.28	  8
  9	 A 	   464.72	   388.76	  7
 10	 A 	   500.24	   353.24	  6
 11	 C 	   580.32	   317.72	  5
 12	 D 	   637.84	   237.64	  4
 13	 I 	   694.38	   180.12	  3
 14	 A 	   729.90	   123.58	  2
 15	 R 	        -  	    88.06	  1  _
```

**13. IPI00289342 Ephrin type-B receptor 4**

| Sequence | Charge | M+H+ | XCorr | DeltaCn |  |
| --- | --- | --- | --- | --- | --- |
| R.TYEVCDVQRAPGQAHWLR.T | 3 | 2187.397 | 3.839 | 0.504 |  |

+1 charge

```
		      B   	       Y
  1	 T 	   102.05	        -  	 18
  2	 Y 	   265.12	  2085.14	 17
  3	 E 	   394.16	  1922.08	 16
  4	 V 	   493.23	  1793.04	 15
  5	 C 	   653.39	  1693.97	 14
  6	 D 	   768.42	  1533.80	 13
  7	 V 	   867.49	  1418.78	 12
  8	 Q 	   995.55	  1319.71	 11
  9	 R 	  1151.65	  1191.65	 10
 10	 A 	  1222.69	  1035.55	  9
 11	 P 	  1319.74	   964.51	  8
 12	 G 	  1376.76	   867.46	  7
 13	 Q 	  1504.82	   810.44	  6
 14	 A 	  1575.86	   682.38	  5
 15	 H 	  1712.92	   611.34	  4
 16	 W 	  1898.99	   474.28	  3
 17	 L 	  2012.08	   288.20	  2
 18	 R 	        -  	   175.12	  1  _
+2 charge
		      B   	       Y
  1	 T 	    51.53	        -  	 18
  2	 Y 	   133.06	  1043.08	 17
  3	 E 	   197.58	   961.54	 16
  4	 V 	   247.12	   897.02	 15
  5	 C 	   327.20	   847.49	 14
  6	 D 	   384.71	   767.41	 13
  7	 V 	   434.25	   709.89	 12
  8	 Q 	   498.28	   660.36	 11
  9	 R 	   576.33	   596.33	 10
 10	 A 	   611.85	   518.28	  9
 11	 P 	   660.37	   482.76	  8
 12	 G 	   688.88	   434.23	  7
 13	 Q 	   752.91	   405.72	  6
 14	 A 	   788.43	   341.69	  5
 15	 H 	   856.96	   306.17	  4
 16	 W 	   950.00	   237.64	  3
 17	 L 	  1006.54	   144.61	  2
 18	 R 	        -  	    88.06	  1  _
+3 charge
		      B   	       Y
  1	 T 	    34.69	        -  	 18
  2	 Y 	    89.04	   695.72	 17
  3	 E 	   132.06	   641.36	 16
  4	 V 	   165.08	   598.35	 15
  5	 C 	   218.47	   565.33	 14
  6	 D 	   256.81	   511.94	 13
  7	 V 	   289.83	   473.60	 12
  8	 Q 	   332.52	   440.57	 11
  9	 R 	   384.55	   397.89	 10
 10	 A 	   408.23	   345.85	  9
 11	 P 	   440.58	   322.18	  8
 12	 G 	   459.59	   289.82	  7
 13	 Q 	   502.28	   270.82	  6
 14	 A 	   525.96	   228.13	  5
 15	 H 	   571.64	   204.45	  4
 16	 W 	   633.67	   158.77	  3
 17	 L 	   671.36	    96.74	  2
 18	 R 	        -  	    59.04	  1  _
```

**14. IPI00178926 Immunoglobulin J chain**

| Sequence | Charge | M+H+ | XCorr | DeltaCn |
| --- | --- | --- | --- | --- |
| R.FVYHLSDLCK.K | 2 | 1282.463 | 3.219 | 0.46 |

+1 charge

```
		      B   	       Y
  1	 F 	   148.08	        -  	 10
  2	 V 	   247.14	  1134.70	  9
  3	 Y 	   410.21	  1035.63	  8
  4	 H 	   547.27	   872.56	  7
  5	 L 	   660.35	   735.51	  6
  6	 S 	   747.38	   622.42	  5
  7	 D 	   862.41	   535.39	  4
  8	 L 	   975.49	   420.36	  3
  9	 C 	  1135.66	   307.28	  2
 10	 K 	        -  	   147.11	  1  _
+2 charge
		      B   	       Y
  1	 F 	    74.54	        -  	 10
  2	 V 	   124.08	   567.85	  9
  3	 Y 	   205.61	   518.32	  8
  4	 H 	   274.14	   436.79	  7
  5	 L 	   330.68	   368.26	  6
  6	 S 	   374.19	   311.71	  5
  7	 D 	   431.71	   268.20	  4
  8	 L 	   488.25	   210.68	  3
  9	 C 	   568.33	   154.14	  2
 10	 K 	        -  	    74.06	  1  _
```

**15. IPI00953695 Ig kappa chain V-II region GM607 (Fragment)**

| Sequence | Charge | M+H+ | XCorr | DeltaCn |
| --- | --- | --- | --- | --- |
| R.ASGVPDRFSGSGSGTDFTLK.I | 2 | 1987.116 | 4.15 | 0.528 |

+1 charge

```
		      B   	       Y
  1	 A 	    72.04	        -  	 20
  2	 S 	   159.08	  1914.92	 19
  3	 G 	   216.10	  1827.89	 18
  4	 V 	   315.17	  1770.87	 17
  5	 P 	   412.22	  1671.80	 16
  6	 D 	   527.25	  1574.74	 15
  7	 R 	   683.35	  1459.72	 14
  8	 F 	   830.42	  1303.62	 13
  9	 S 	   917.45	  1156.55	 12
 10	 G 	   974.47	  1069.52	 11
 11	 S 	  1061.50	  1012.49	 10
 12	 G 	  1118.52	   925.46	  9
 13	 S 	  1205.55	   868.44	  8
 14	 G 	  1262.58	   781.41	  7
 15	 T 	  1363.62	   724.39	  6
 16	 D 	  1478.65	   623.34	  5
 17	 F 	  1625.72	   508.31	  4
 18	 T 	  1726.77	   361.24	  3
 19	 L 	  1839.85	   260.20	  2
 20	 K 	        -  	   147.11	  1  _
+2 charge
		      B   	       Y
  1	 A 	    36.53	        -  	 20
  2	 S 	    80.04	   957.96	 19
  3	 G 	   108.55	   914.45	 18
  4	 V 	   158.09	   885.94	 17
  5	 P 	   206.61	   836.40	 16
  6	 D 	   264.13	   787.88	 15
  7	 R 	   342.18	   730.36	 14
  8	 F 	   415.71	   652.31	 13
  9	 S 	   459.23	   578.78	 12
 10	 G 	   487.74	   535.26	 11
 11	 S 	   531.25	   506.75	 10
 12	 G 	   559.76	   463.23	  9
 13	 S 	   603.28	   434.72	  8
 14	 G 	   631.79	   391.21	  7
 15	 T 	   682.32	   362.70	  6
 16	 D 	   739.83	   312.17	  5
 17	 F 	   813.36	   254.66	  4
 18	 T 	   863.89	   181.13	  3
 19	 L 	   920.43	   130.60	  2
 20	 K 	        -  	    74.06	  1  _
```

**16. IPI00329688 Protein YIPF3**

| Sequence | Charge | M+H+ | XCorr | DeltaCn |
| --- | --- | --- | --- | --- |
| R.DIPAMLPAAR.L | 1 | 1055.276 | 2.509 | 0.277 |

+1 charge

```
		      B   	       Y
  1	 D 	   116.03	        -  	 10
  2	 I 	   229.12	   939.54	  9
  3	 P 	   326.17	   826.46	  8
  4	 A 	   397.21	   729.41	  7
  5	 M 	   528.25	   658.37	  6
  6	 L 	   641.33	   527.33	  5
  7	 P 	   738.39	   414.25	  4
  8	 A 	   809.42	   317.19	  3
  9	 A 	   880.46	   246.16	  2
 10	 R 	        -  	   175.12	  1  _
```

**17. IPI00936772 Isoform 1 of Golgi membrane protein 1**

| Sequence | Charge | M+H+ | XCorr | DeltaCn |
| --- | --- | --- | --- | --- |
| R.KFSYDLSQCINQMK.E | 2 | 1763.002 | 3.552 | 0.428 |

+1 charge

```
		      B   	       Y
  1	 K 	   129.10	        -  	 14
  2	 F 	   276.17	  1633.87	 13
  3	 S 	   363.20	  1486.80	 12
  4	 Y 	   526.27	  1399.77	 11
  5	 D 	   641.29	  1236.71	 10
  6	 L 	   754.38	  1121.68	  9
  7	 S 	   841.41	  1008.59	  8
  8	 Q 	   969.47	   921.56	  7
  9	 C 	  1129.63	   793.50	  6
 10	 I 	  1242.72	   633.34	  5
 11	 N 	  1356.76	   520.25	  4
 12	 Q 	  1484.82	   406.21	  3
 13	 M 	  1615.86	   278.15	  2
 14	 K 	        -  	   147.11	  1  _
+2 charge
		      B   	       Y
  1	 K 	    65.05	        -  	 14
  2	 F 	   138.59	   817.44	 13
  3	 S 	   182.10	   743.90	 12
  4	 Y 	   263.64	   700.39	 11
  5	 D 	   321.15	   618.86	 10
  6	 L 	   377.69	   561.34	  9
  7	 S 	   421.21	   504.80	  8
  8	 Q 	   485.24	   461.28	  7
  9	 C 	   565.32	   397.26	  6
 10	 I 	   621.86	   317.17	  5
 11	 N 	   678.88	   260.63	  4
 12	 Q 	   742.91	   203.61	  3
 13	 M 	   808.43	   139.58	  2
 14	 K 	        -  	    74.06	  1  _
```

**18. IPI00479743 Isoform 1 of POTE ankyrin domain family member E**

| Sequence | Charge | M+H+ | XCorr | DeltaCn |
| --- | --- | --- | --- | --- |
| R.SYELPDGQVITIGNER.F | 2 | 1791.94 | 3.234 | 0.278 |

+1 charge

```
		      B   	       Y
  1	 S 	    88.04	        -  	 16
  2	 Y 	   251.10	  1703.86	 15
  3	 E 	   380.15	  1540.80	 14
  4	 L 	   493.23	  1411.75	 13
  5	 P 	   590.28	  1298.67	 12
  6	 D 	   705.31	  1201.62	 11
  7	 G 	   762.33	  1086.59	 10
  8	 Q 	   890.39	  1029.57	  9
  9	 V 	   989.46	   901.51	  8
 10	 I 	  1102.54	   802.44	  7
 11	 T 	  1203.59	   689.36	  6
 12	 I 	  1316.67	   588.31	  5
 13	 G 	  1373.69	   475.23	  4
 14	 N 	  1487.74	   418.20	  3
 15	 E 	  1616.78	   304.16	  2
 16	 R 	        -  	   175.12	  1  _
+2 charge
		      B   	       Y
  1	 S 	    44.52	        -  	 16
  2	 Y 	   126.05	   852.43	 15
  3	 E 	   190.58	   770.90	 14
  4	 L 	   247.12	   706.38	 13
  5	 P 	   295.64	   649.84	 12
  6	 D 	   353.16	   601.31	 11
  7	 G 	   381.67	   543.80	 10
  8	 Q 	   445.70	   515.29	  9
  9	 V 	   495.23	   451.26	  8
 10	 I 	   551.77	   401.72	  7
 11	 T 	   602.30	   345.18	  6
 12	 I 	   658.84	   294.66	  5
 13	 G 	   687.35	   238.12	  4
 14	 N 	   744.37	   209.61	  3
 15	 E 	   808.89	   152.58	  2
 16	 R 	        -  	    88.06	  1  _
```

**19. IPI00025346 Isoform 1 of Peroxisomal membrane protein PEX14**

| Sequence | Charge | M+H+ | XCorr | DeltaCn |
| --- | --- | --- | --- | --- |
| R.DYGALAIIMAGIAFGFHQLYK.K | 3 | 2300.707 | 3.672 | 0.204 |

+1 charge

```
		      B   	       Y
  1	 D 	   116.03	        -  	 21
  2	 Y 	   279.10	  2184.17	 20
  3	 G 	   336.12	  2021.10	 19
  4	 A 	   407.16	  1964.08	 18
  5	 L 	   520.24	  1893.05	 17
  6	 A 	   591.28	  1779.96	 16
  7	 I 	   704.36	  1708.92	 15
  8	 I 	   817.45	  1595.84	 14
  9	 M 	   948.49	  1482.76	 13
 10	 A 	  1019.52	  1351.72	 12
 11	 G 	  1076.54	  1280.68	 11
 12	 I 	  1189.63	  1223.66	 10
 13	 A 	  1260.67	  1110.57	  9
 14	 F 	  1407.73	  1039.54	  8
 15	 G 	  1464.76	   892.47	  7
 16	 F 	  1611.82	   835.45	  6
 17	 H 	  1748.88	   688.38	  5
 18	 Q 	  1876.94	   551.32	  4
 19	 L 	  1990.03	   423.26	  3
 20	 Y 	  2153.09	   310.18	  2
 21	 K 	        -  	   147.11	  1  _


+2 charge
		      B   	       Y
  1	 D 	    58.52	        -  	 21
  2	 Y 	   140.05	  1092.59	 20
  3	 G 	   168.56	  1011.06	 19
  4	 A 	   204.08	   982.54	 18
  5	 L 	   260.62	   947.03	 17
  6	 A 	   296.14	   890.48	 16
  7	 I 	   352.68	   854.97	 15
  8	 I 	   409.23	   798.42	 14
  9	 M 	   474.75	   741.88	 13
 10	 A 	   510.27	   676.36	 12
 11	 G 	   538.78	   640.84	 11
 12	 I 	   595.32	   612.33	 10
 13	 A 	   630.84	   555.79	  9
 14	 F 	   704.37	   520.27	  8
 15	 G 	   732.88	   446.74	  7
 16	 F 	   806.42	   418.23	  6
 17	 H 	   874.95	   344.69	  5
 18	 Q 	   938.97	   276.16	  4
 19	 L 	   995.52	   212.13	  3
 20	 Y 	  1077.05	   155.59	  2
 21	 K 	        -  	    74.06	  1  _
+3 charge
		      B   	       Y
  1	 D 	    39.35	        -  	 21
  2	 Y 	    93.70	   728.73	 20
  3	 G 	   112.71	   674.37	 19
  4	 A 	   136.39	   655.37	 18
  5	 L 	   174.08	   631.69	 17
  6	 A 	   197.76	   593.99	 16
  7	 I 	   235.46	   570.31	 15
  8	 I 	   273.15	   532.62	 14
  9	 M 	   316.83	   494.92	 13
 10	 A 	   340.51	   451.24	 12
 11	 G 	   359.52	   427.56	 11
 12	 I 	   397.21	   408.56	 10
 13	 A 	   420.89	   370.86	  9
 14	 F 	   469.92	   347.18	  8
 15	 G 	   488.92	   298.16	  7
 16	 F 	   537.95	   279.15	  6
 17	 H 	   583.63	   230.13	  5
 18	 Q 	   626.32	   184.44	  4
 19	 L 	   664.01	   141.76	  3
 20	 Y 	   718.37	   104.06	  2
 21	 K 	        -  	    49.71	  1  _
```

**20. IPI00441344 Isoform 1 of Beta-galactosidase**

| Sequence | Charge | M+H+ | XCorr | DeltaCn |
| --- | --- | --- | --- | --- |
| K.AGATLDLLVENMGR.V | 2 | 1460.682 | 3.55 | 0.379 |

+1 charge

```
		      B   	       Y
  1	 A 	    72.04	        -  	 14
  2	 G 	   129.07	  1388.72	 13
  3	 A 	   200.10	  1331.70	 12
  4	 T 	   301.15	  1260.66	 11
  5	 L 	   414.23	  1159.61	 10
  6	 D 	   529.26	  1046.53	  9
  7	 L 	   642.35	   931.50	  8
  8	 L 	   755.43	   818.42	  7
  9	 V 	   854.50	   705.33	  6
 10	 E 	   983.54	   606.27	  5
 11	 N 	  1097.58	   477.22	  4
 12	 M 	  1228.62	   363.18	  3
 13	 G 	  1285.65	   232.14	  2
 14	 R 	        -  	   175.12	  1  _
+2 charge
		      B   	       Y
  1	 A 	    36.53	        -  	 14
  2	 G 	    65.04	   694.86	 13
  3	 A 	   100.56	   666.35	 12
  4	 T 	   151.08	   630.83	 11
  5	 L 	   207.62	   580.31	 10
  6	 D 	   265.13	   523.77	  9
  7	 L 	   321.68	   466.26	  8
  8	 L 	   378.22	   409.71	  7
  9	 V 	   427.75	   353.17	  6
 10	 E 	   492.27	   303.64	  5
 11	 N 	   549.30	   239.12	  4
 12	 M 	   614.82	   182.09	  3
 13	 G 	   643.33	   116.57	  2
 14	 R 	        -  	    88.06	  1  _
```

**21. IPI00021304 Keratin, type II cytoskeletal 2 epidermal**

| Sequence | Charge | M+H+ | XCorr | DeltaCn |
| --- | --- | --- | --- | --- |
| R.FLEQQNQVLQTKWELLQQMNVGTR.P | 3 | 2933.335 | 4.656 | 0.509 |

+1 charge

```
		      B   	       Y
  1	 F 	   148.08	        -  	 24
  2	 L 	   261.16	  2784.45	 23
  3	 E 	   390.20	  2671.36	 22
  4	 Q 	   518.26	  2542.32	 21
  5	 Q 	   646.32	  2414.26	 20
  6	 N 	   760.36	  2286.20	 19
  7	 Q 	   888.42	  2172.16	 18
  8	 V 	   987.49	  2044.10	 17
  9	 L 	  1100.57	  1945.03	 16
 10	 Q 	  1228.63	  1831.95	 15
 11	 T 	  1329.68	  1703.89	 14
 12	 K 	  1457.77	  1602.84	 13
 13	 W 	  1643.85	  1474.75	 12
 14	 E 	  1772.90	  1288.67	 11
 15	 L 	  1885.98	  1159.63	 10
 16	 L 	  1999.06	  1046.54	  9
 17	 Q 	  2127.12	   933.46	  8
 18	 Q 	  2255.18	   805.40	  7
 19	 M 	  2386.22	   677.34	  6
 20	 N 	  2500.27	   546.30	  5
 21	 V 	  2599.33	   432.26	  4
 22	 G 	  2656.36	   333.19	  3
 23	 T 	  2757.40	   276.17	  2
 24	 R 	        -  	   175.12	  1  _


+2 charge
		      B   	       Y
  1	 F 	    74.54	        -  	 24
  2	 L 	   131.08	  1392.73	 23
  3	 E 	   195.60	  1336.18	 22
  4	 Q 	   259.63	  1271.66	 21
  5	 Q 	   323.66	  1207.63	 20
  6	 N 	   380.68	  1143.60	 19
  7	 Q 	   444.71	  1086.58	 18
  8	 V 	   494.25	  1022.55	 17
  9	 L 	   550.79	   973.02	 16
 10	 Q 	   614.82	   916.48	 15
 11	 T 	   665.34	   852.45	 14
 12	 K 	   729.39	   801.92	 13
 13	 W 	   822.43	   737.88	 12
 14	 E 	   886.95	   644.84	 11
 15	 L 	   943.49	   580.32	 10
 16	 L 	  1000.04	   523.77	  9
 17	 Q 	  1064.07	   467.23	  8
 18	 Q 	  1128.09	   403.20	  7
 19	 M 	  1193.61	   339.17	  6
 20	 N 	  1250.64	   273.65	  5
 21	 V 	  1300.17	   216.63	  4
 22	 G 	  1328.68	   167.10	  3
 23	 T 	  1379.21	   138.59	  2
 24	 R 	        -  	    88.06	  1  _


+3 charge
		      B   	       Y
  1	 F 	    50.03	        -  	 24
  2	 L 	    87.72	   928.82	 23
  3	 E 	   130.74	   891.13	 22
  4	 Q 	   173.43	   848.11	 21
  5	 Q 	   216.11	   805.43	 20
  6	 N 	   254.13	   762.74	 19
  7	 Q 	   296.81	   724.72	 18
  8	 V 	   329.83	   682.04	 17
  9	 L 	   367.53	   649.02	 16
 10	 Q 	   410.22	   611.32	 15
 11	 T 	   443.90	   568.63	 14
 12	 K 	   486.60	   534.95	 13
 13	 W 	   548.62	   492.25	 12
 14	 E 	   591.64	   430.23	 11
 15	 L 	   629.33	   387.21	 10
 16	 L 	   667.03	   349.52	  9
 17	 Q 	   709.71	   311.82	  8
 18	 Q 	   752.40	   269.14	  7
 19	 M 	   796.08	   226.45	  6
 20	 N 	   834.09	   182.77	  5
 21	 V 	   867.12	   144.76	  4
 22	 G 	   886.12	   111.73	  3
 23	 T 	   919.81	    92.73	  2
 24	 R 	        -  	    59.04	  1  _
```

**22. IPI00005707 C-type mannose receptor 2**

| Sequence | Charge | M+H+ | XCorr | DeltaCn |
| --- | --- | --- | --- | --- |
| R.TLGDQLSLLLGAR.T | 2 | 1357.581 | 3.843 | 0.456 |

+1 charge

```
		      B   	       Y
  1	 T 	   102.05	        -  	 13
  2	 L 	   215.14	  1255.74	 12
  3	 G 	   272.16	  1142.65	 11
  4	 D 	   387.19	  1085.63	 10
  5	 Q 	   515.25	   970.60	  9
  6	 L 	   628.33	   842.55	  8
  7	 S 	   715.36	   729.46	  7
  8	 L 	   828.45	   642.43	  6
  9	 L 	   941.53	   529.35	  5
 10	 L 	  1054.61	   416.26	  4
 11	 G 	  1111.64	   303.18	  3
 12	 A 	  1182.67	   246.16	  2
 13	 R 	        -  	   175.12	  1  _
+2 charge
		      B   	       Y
  1	 T 	    51.53	        -  	 13
  2	 L 	   108.07	   628.37	 12
  3	 G 	   136.58	   571.83	 11
  4	 D 	   194.10	   543.32	 10
  5	 Q 	   258.13	   485.81	  9
  6	 L 	   314.67	   421.78	  8
  7	 S 	   358.18	   365.23	  7
  8	 L 	   414.73	   321.72	  6
  9	 L 	   471.27	   265.18	  5
 10	 L 	   527.81	   208.63	  4
 11	 G 	   556.32	   152.09	  3
 12	 A 	   591.84	   123.58	  2
 13	 R 	        -  	    88.06	  1  _
```

**23. IPI00246058 Programmed cell death 6-interacting protein**

| Sequence | Charge | M+H+ | XCorr | DeltaCn |
| --- | --- | --- | --- | --- |
| R.SVIEQGGIQTVDQLIKELPELLQR.N | 3 | 2708.104 | 4.275 | 0.4 |

+1 charge

```
		      B   	       Y
  1	 S 	    88.04	        -  	 24
  2	 V 	   187.11	  2619.47	 23
  3	 I 	   300.19	  2520.40	 22
  4	 E 	   429.23	  2407.32	 21
  5	 Q 	   557.29	  2278.28	 20
  6	 G 	   614.31	  2150.22	 19
  7	 G 	   671.34	  2093.20	 18
  8	 I 	   784.42	  2036.18	 17
  9	 Q 	   912.48	  1923.09	 16
 10	 T 	  1013.53	  1795.03	 15
 11	 V 	  1112.59	  1693.98	 14
 12	 D 	  1227.62	  1594.92	 13
 13	 Q 	  1355.68	  1479.89	 12
 14	 L 	  1468.76	  1351.83	 11
 15	 I 	  1581.85	  1238.75	 10
 16	 K 	  1709.94	  1125.66	  9
 17	 E 	  1838.99	   997.57	  8
 18	 L 	  1952.07	   868.53	  7
 19	 P 	  2049.12	   755.44	  6
 20	 E 	  2178.17	   658.39	  5
 21	 L 	  2291.25	   529.35	  4
 22	 L 	  2404.33	   416.26	  3
 23	 Q 	  2532.39	   303.18	  2
 24	 R 	        -  	   175.12	  1  _


+2 charge
		      B   	       Y
  1	 S 	    44.52	        -  	 24
  2	 V 	    94.06	  1310.24	 23
  3	 I 	   150.60	  1260.71	 22
  4	 E 	   215.12	  1204.16	 21
  5	 Q 	   279.15	  1139.64	 20
  6	 G 	   307.66	  1075.61	 19
  7	 G 	   336.17	  1047.10	 18
  8	 I 	   392.71	  1018.59	 17
  9	 Q 	   456.74	   962.05	 16
 10	 T 	   507.27	   898.02	 15
 11	 V 	   556.80	   847.50	 14
 12	 D 	   614.31	   797.96	 13
 13	 Q 	   678.34	   740.45	 12
 14	 L 	   734.89	   676.42	 11
 15	 I 	   791.43	   619.88	 10
 16	 K 	   855.48	   563.33	  9
 17	 E 	   920.00	   499.29	  8
 18	 L 	   976.54	   434.77	  7
 19	 P 	  1025.06	   378.22	  6
 20	 E 	  1089.59	   329.70	  5
 21	 L 	  1146.13	   265.18	  4
 22	 L 	  1202.67	   208.63	  3
 23	 Q 	  1266.70	   152.09	  2
 24	 R 	        -  	    88.06	  1  _


+3 charge
		      B   	       Y
  1	 S 	    30.02	        -  	 24
  2	 V 	    63.04	   873.83	 23
  3	 I 	   100.74	   840.81	 22
  4	 E 	   143.75	   803.11	 21
  5	 Q 	   186.44	   760.10	 20
  6	 G 	   205.44	   717.41	 19
  7	 G 	   224.45	   698.40	 18
  8	 I 	   262.14	   679.40	 17
  9	 Q 	   304.83	   641.70	 16
 10	 T 	   338.51	   599.02	 15
 11	 V 	   371.54	   565.33	 14
 12	 D 	   409.88	   532.31	 13
 13	 Q 	   452.56	   493.97	 12
 14	 L 	   490.26	   451.28	 11
 15	 I 	   527.95	   413.59	 10
 16	 K 	   570.65	   375.89	  9
 17	 E 	   613.67	   333.19	  8
 18	 L 	   651.36	   290.18	  7
 19	 P 	   683.71	   252.49	  6
 20	 E 	   726.73	   220.13	  5
 21	 L 	   764.42	   177.12	  4
 22	 L 	   802.12	   139.43	  3
 23	 Q 	   844.80	   101.73	  2
 24	 R 	        -  	    59.04	  1  _
```

**24. IPI00008239 cDNA FLJ55176, highly similar to G-protein coupled receptor family C group 5 member B**

| Sequence | Charge | M+H+ | XCorr | DeltaCn |
| --- | --- | --- | --- | --- |
| K.AFSMDEHNAALR.T | 2 | 1362.497 | 3.317 | 0.48 |

+1 charge

```
		      B   	       Y
  1	 A 	    72.04	        -  	 12
  2	 F 	   219.11	  1290.59	 11
  3	 S 	   306.14	  1143.52	 10
  4	 M 	   437.19	  1056.49	  9
  5	 D 	   552.21	   925.45	  8
  6	 E 	   681.25	   810.42	  7
  7	 H 	   818.31	   681.38	  6
  8	 N 	   932.36	   544.32	  5
  9	 A 	  1003.39	   430.28	  4
 10	 A 	  1074.43	   359.24	  3
 11	 L 	  1187.51	   288.20	  2
 12	 R 	        -  	   175.12	  1  _
+2 charge
		      B   	       Y
  1	 A 	    36.53	        -  	 12
  2	 F 	   110.06	   645.80	 11
  3	 S 	   153.58	   572.26	 10
  4	 M 	   219.10	   528.75	  9
  5	 D 	   276.61	   463.23	  8
  6	 E 	   341.13	   405.71	  7
  7	 H 	   409.66	   341.19	  6
  8	 N 	   466.68	   272.66	  5
  9	 A 	   502.20	   215.64	  4
 10	 A 	   537.72	   180.12	  3
 11	 L 	   594.26	   144.61	  2
 12	 R 	        -  	    88.06	  1  _
```

**25. IPI00056309 Liver-expressed antimicrobial peptide 2**

| Sequence | Charge | M+H+ | XCorr | DeltaCn |
| --- | --- | --- | --- | --- |
| R.GVSLRPIGASCRDDSECITR.L | 3 | 2250.443 | 4.783 | 0.503 |

+1 charge

```
		      B   	       Y
  1	 G 	    58.03	        -  	 20
  2	 V 	   157.10	  2192.32	 19
  3	 S 	   244.13	  2093.26	 18
  4	 L 	   357.21	  2006.22	 17
  5	 R 	   513.31	  1893.14	 16
  6	 P 	   610.37	  1737.04	 15
  7	 I 	   723.45	  1639.99	 14
  8	 G 	   780.47	  1526.90	 13
  9	 A 	   851.51	  1469.88	 12
 10	 S 	   938.54	  1398.84	 11
 11	 C 	  1098.71	  1311.81	 10
 12	 R 	  1254.81	  1151.65	  9
 13	 D 	  1369.84	   995.54	  8
 14	 D 	  1484.86	   880.52	  7
 15	 S 	  1571.89	   765.49	  6
 16	 E 	  1700.94	   678.46	  5
 17	 C 	  1861.10	   549.42	  4
 18	 I 	  1974.19	   389.25	  3
 19	 T 	  2075.23	   276.17	  2
 20	 R 	        -  	   175.12	  1  _
+2 charge
		      B   	       Y
  1	 G 	    29.52	        -  	 20
  2	 V 	    79.05	  1096.67	 19
  3	 S 	   122.57	  1047.13	 18
  4	 L 	   179.11	  1003.62	 17
  5	 R 	   257.16	   947.07	 16
  6	 P 	   305.69	   869.02	 15
  7	 I 	   362.23	   820.50	 14
  8	 G 	   390.74	   763.95	 13
  9	 A 	   426.26	   735.44	 12
 10	 S 	   469.77	   699.93	 11
 11	 C 	   549.86	   656.41	 10
 12	 R 	   627.91	   576.33	  9
 13	 D 	   685.42	   498.28	  8
 14	 D 	   742.93	   440.76	  7
 15	 S 	   786.45	   383.25	  6
 16	 E 	   850.97	   339.73	  5
 17	 C 	   931.05	   275.21	  4
 18	 I 	   987.60	   195.13	  3
 19	 T 	  1038.12	   138.59	  2
 20	 R 	        -  	    88.06	  1  _

+3 charge
		      B   	       Y
  1	 G 	    20.01	        -  	 20
  2	 V 	    53.04	   731.45	 19
  3	 S 	    82.05	   698.42	 18
  4	 L 	   119.74	   669.41	 17
  5	 R 	   171.78	   631.72	 16
  6	 P 	   204.13	   579.68	 15
  7	 I 	   241.82	   547.33	 14
  8	 G 	   260.83	   509.64	 13
  9	 A 	   284.51	   490.63	 12
 10	 S 	   313.52	   466.95	 11
 11	 C 	   366.91	   437.94	 10
 12	 R 	   418.94	   384.55	  9
 13	 D 	   457.28	   332.52	  8
 14	 D 	   495.63	   294.18	  7
 15	 S 	   524.64	   255.84	  6
 16	 E 	   567.65	   226.82	  5
 17	 C 	   621.04	   183.81	  4
 18	 I 	   658.73	   130.42	  3
 19	 T 	   692.42	    92.73	  2
 20	 R 	        -  	    59.04	  1  _
```

**26. IPI00022284 Major prion protein**

| Sequence | Charge | M+H+ | XCorr | DeltaCn |
| --- | --- | --- | --- | --- |
| R.VVEQMCITQYER.E | 2 | 1556.76 | 4.635 | 0.491 |

+1 charge

```
		      B   	       Y
  1	 V 	   100.08	        -  	 12
  2	 V 	   199.14	  1456.79	 11
  3	 E 	   328.19	  1357.72	 10
  4	 Q 	   456.25	  1228.68	  9
  5	 M 	   587.29	  1100.62	  8
  6	 C 	   747.45	   969.58	  7
  7	 I 	   860.54	   809.42	  6
  8	 T 	   961.58	   696.33	  5
  9	 Q 	  1089.64	   595.28	  4
 10	 Y 	  1252.70	   467.22	  3
 11	 E 	  1381.75	   304.16	  2
 12	 R 	        -  	   175.12	  1  _
+2 charge
		      B   	       Y
  1	 V 	    50.54	        -  	 12
  2	 V 	   100.08	   728.90	 11
  3	 E 	   164.60	   679.36	 10
  4	 Q 	   228.63	   614.84	  9
  5	 M 	   294.15	   550.81	  8
  6	 C 	   374.23	   485.29	  7
  7	 I 	   430.77	   405.21	  6
  8	 T 	   481.30	   348.67	  5
  9	 Q 	   545.32	   298.15	  4
 10	 Y 	   626.86	   234.12	  3
 11	 E 	   691.38	   152.58	  2
 12	 R 	        -  	    88.06	  1  _
```

**27. IPI00299158 Isoform Alpha of Poliovirus receptor**

| Sequence | Charge | M+H+ | XCorr | DeltaCn |
| --- | --- | --- | --- | --- |
| R.VLAKPQNTAEVQKVQLTGEPVPMAR.C | 3 | 2706.158 | 5.23 | 0.545 |

+1 charge

```
		      B   	       Y
  1	 V 	   100.08	        -  	 25
  2	 L 	   213.16	  2605.41	 24
  3	 A 	   284.20	  2492.33	 23
  4	 K 	   412.29	  2421.29	 22
  5	 P 	   509.34	  2293.20	 21
  6	 Q 	   637.40	  2196.14	 20
  7	 N 	   751.45	  2068.09	 19
  8	 T 	   852.49	  1954.04	 18
  9	 A 	   923.53	  1852.99	 17
 10	 E 	  1052.57	  1781.96	 16
 11	 V 	  1151.64	  1652.92	 15
 12	 Q 	  1279.70	  1553.85	 14
 13	 K 	  1407.80	  1425.79	 13
 14	 V 	  1506.86	  1297.69	 12
 15	 Q 	  1634.92	  1198.62	 11
 16	 L 	  1748.01	  1070.57	 10
 17	 T 	  1849.05	   957.48	  9
 18	 G 	  1906.08	   856.43	  8
 19	 E 	  2035.12	   799.41	  7
 20	 P 	  2132.17	   670.37	  6
 21	 V 	  2231.24	   573.32	  5
 22	 P 	  2328.29	   474.25	  4
 23	 M 	  2459.33	   377.20	  3
 24	 A 	  2530.37	   246.16	  2
 25	 R 	        -  	   175.12	  1  _


+2 charge
		      B   	       Y
  1	 V 	    50.54	        -  	 25
  2	 L 	   107.08	  1303.21	 24
  3	 A 	   142.60	  1246.67	 23
  4	 K 	   206.65	  1211.15	 22
  5	 P 	   255.18	  1147.10	 21
  6	 Q 	   319.21	  1098.58	 20
  7	 N 	   376.23	  1034.55	 19
  8	 T 	   426.75	   977.52	 18
  9	 A 	   462.27	   927.00	 17
 10	 E 	   526.79	   891.48	 16
 11	 V 	   576.32	   826.96	 15
 12	 Q 	   640.35	   777.43	 14
 13	 K 	   704.40	   713.40	 13
 14	 V 	   753.94	   649.35	 12
 15	 Q 	   817.96	   599.82	 11
 16	 L 	   874.51	   535.79	 10
 17	 T 	   925.03	   479.24	  9
 18	 G 	   953.54	   428.72	  8
 19	 E 	  1018.06	   400.21	  7
 20	 P 	  1066.59	   335.69	  6
 21	 V 	  1116.12	   287.16	  5
 22	 P 	  1164.65	   237.63	  4
 23	 M 	  1230.17	   189.10	  3
 24	 A 	  1265.69	   123.58	  2
 25	 R 	        -  	    88.06	  1  _


+3 charge
		      B   	       Y
  1	 V 	    34.03	        -  	 25
  2	 L 	    71.72	   869.14	 24
  3	 A 	    95.40	   831.45	 23
  4	 K 	   138.10	   807.77	 22
  5	 P 	   170.45	   765.07	 21
  6	 Q 	   213.14	   732.72	 20
  7	 N 	   251.15	   690.03	 19
  8	 T 	   284.84	   652.02	 18
  9	 A 	   308.52	   618.34	 17
 10	 E 	   351.53	   594.66	 16
 11	 V 	   384.55	   551.64	 15
 12	 Q 	   427.24	   518.62	 14
 13	 K 	   469.94	   475.93	 13
 14	 V 	   502.96	   433.24	 12
 15	 Q 	   545.65	   400.21	 11
 16	 L 	   583.34	   357.53	 10
 17	 T 	   617.02	   319.83	  9
 18	 G 	   636.03	   286.15	  8
 19	 E 	   679.04	   267.14	  7
 20	 P 	   711.40	   224.13	  6
 21	 V 	   744.42	   191.78	  5
 22	 P 	   776.77	   158.75	  4
 23	 M 	   820.45	   126.40	  3
 24	 A 	   844.13	    82.72	  2
 25	 R 	        -  	    59.04	  1  _
```

**28. IPI00019943 Afamin**

| Sequence | Charge | M+H+ | XCorr | DeltaCn |
| --- | --- | --- | --- | --- |
| R.RHPDLSIPELLR.I | 2 | 1446.68 | 2.938 | 0.411 |

+1 charge

```
		      B   	       Y
  1	 R 	   157.11	        -  	 12
  2	 H 	   294.17	  1289.72	 11
  3	 P 	   391.22	  1152.66	 10
  4	 D 	   506.25	  1055.61	  9
  5	 L 	   619.33	   940.58	  8
  6	 S 	   706.36	   827.50	  7
  7	 I 	   819.45	   740.47	  6
  8	 P 	   916.50	   627.38	  5
  9	 E 	  1045.54	   530.33	  4
 10	 L 	  1158.63	   401.29	  3
 11	 L 	  1271.71	   288.20	  2
 12	 R 	        -  	   175.12	  1  _
+2 charge
		      B   	       Y
  1	 R 	    79.06	        -  	 12
  2	 H 	   147.59	   645.36	 11
  3	 P 	   196.11	   576.83	 10
  4	 D 	   253.63	   528.31	  9
  5	 L 	   310.17	   470.79	  8
  6	 S 	   353.69	   414.25	  7
  7	 I 	   410.23	   370.74	  6
  8	 P 	   458.75	   314.19	  5
  9	 E 	   523.27	   265.67	  4
 10	 L 	   579.82	   201.15	  3
 11	 L 	   636.36	   144.61	  2
 12	 R 	        -  	    88.06	  1  _
```

**29. IPI00031065 Deoxyribonuclease-1**

| Sequence | Charge | M+H+ | XCorr | DeltaCn |
| --- | --- | --- | --- | --- |
| K.LLDNLNQDAPDTYHYVVSEPLGR.N | 3 | 2630.851 | 4.683 | 0.449 |

+1 charge

```
		      B   	       Y
  1	 L 	   114.09	        -  	 23
  2	 L 	   227.18	  2516.21	 22
  3	 D 	   342.20	  2403.12	 21
  4	 N 	   456.25	  2288.09	 20
  5	 L 	   569.33	  2174.05	 19
  6	 N 	   683.37	  2060.97	 18
  7	 Q 	   811.43	  1946.92	 17
  8	 D 	   926.46	  1818.87	 16
  9	 A 	   997.49	  1703.84	 15
 10	 P 	  1094.55	  1632.80	 14
 11	 D 	  1209.57	  1535.75	 13
 12	 T 	  1310.62	  1420.72	 12
 13	 Y 	  1473.69	  1319.67	 11
 14	 H 	  1610.74	  1156.61	 10
 15	 Y 	  1773.81	  1019.55	  9
 16	 V 	  1872.88	   856.49	  8
 17	 V 	  1971.94	   757.42	  7
 18	 S 	  2058.98	   658.35	  6
 19	 E 	  2188.02	   571.32	  5
 20	 P 	  2285.07	   442.28	  4
 21	 L 	  2398.16	   345.22	  3
 22	 G 	  2455.18	   232.14	  2
 23	 R 	        -  	   175.12	  1  _


+2 charge
		      B   	       Y
  1	 L 	    57.55	        -  	 23
  2	 L 	   114.09	  1258.61	 22
  3	 D 	   171.60	  1202.06	 21
  4	 N 	   228.63	  1144.55	 20
  5	 L 	   285.17	  1087.53	 19
  6	 N 	   342.19	  1030.99	 18
  7	 Q 	   406.22	   973.97	 17
  8	 D 	   463.73	   909.94	 16
  9	 A 	   499.25	   852.42	 15
 10	 P 	   547.78	   816.90	 14
 11	 D 	   605.29	   768.38	 13
 12	 T 	   655.81	   710.86	 12
 13	 Y 	   737.35	   660.34	 11
 14	 H 	   805.88	   578.81	 10
 15	 Y 	   887.41	   510.28	  9
 16	 V 	   936.94	   428.75	  8
 17	 V 	   986.48	   379.21	  7
 18	 S 	  1029.99	   329.68	  6
 19	 E 	  1094.51	   286.16	  5
 20	 P 	  1143.04	   221.64	  4
 21	 L 	  1199.58	   173.12	  3
 22	 G 	  1228.09	   116.57	  2
 23	 R 	        -  	    88.06	  1  _


+3 charge
		      B   	       Y
  1	 L 	    38.70	        -  	 23
  2	 L 	    76.40	   839.41	 22
  3	 D 	   114.74	   801.71	 21
  4	 N 	   152.75	   763.37	 20
  5	 L 	   190.45	   725.36	 19
  6	 N 	   228.46	   687.66	 18
  7	 Q 	   271.15	   649.65	 17
  8	 D 	   309.49	   606.96	 16
  9	 A 	   333.17	   568.62	 15
 10	 P 	   365.52	   544.94	 14
 11	 D 	   403.86	   512.59	 13
 12	 T 	   437.55	   474.25	 12
 13	 Y 	   491.90	   440.56	 11
 14	 H 	   537.59	   386.21	 10
 15	 Y 	   591.94	   340.52	  9
 16	 V 	   624.96	   286.17	  8
 17	 V 	   657.99	   253.14	  7
 18	 S 	   687.00	   220.12	  6
 19	 E 	   730.01	   191.11	  5
 20	 P 	   762.36	   148.10	  4
 21	 L 	   800.06	   115.75	  3
 22	 G 	   819.06	    78.05	  2
 23	 R 	        -  	    59.04	  1  _
```

**30. IPI00005573 Isoform 1 of 5'(3')-deoxyribonucleotidase, cytosolic type**

| Sequence | Charge | M+H+ | XCorr | DeltaCn |
| --- | --- | --- | --- | --- |
| R.VLVDMDGVLADFEAGLLR.G | 2 | 1934.246 | 5.112 | 0.575 |

+1 charge

```
		      B   	       Y
  1	 V 	   100.08	        -  	 18
  2	 L 	   213.16	  1833.94	 17
  3	 V 	   312.23	  1720.86	 16
  4	 D 	   427.26	  1621.79	 15
  5	 M 	   558.30	  1506.76	 14
  6	 D 	   673.32	  1375.72	 13
  7	 G 	   730.34	  1260.69	 12
  8	 V 	   829.41	  1203.67	 11
  9	 L 	   942.50	  1104.60	 10
 10	 A 	  1013.53	   991.52	  9
 11	 D 	  1128.56	   920.48	  8
 12	 F 	  1275.63	   805.46	  7
 13	 E 	  1404.67	   658.39	  6
 14	 A 	  1475.71	   529.35	  5
 15	 G 	  1532.73	   458.31	  4
 16	 L 	  1645.81	   401.29	  3
 17	 L 	  1758.90	   288.20	  2
 18	 R 	        -  	   175.12	  1  _
+2 charge
		      B   	       Y
  1	 V 	    50.54	        -  	 18
  2	 L 	   107.08	   917.47	 17
  3	 V 	   156.62	   860.93	 16
  4	 D 	   214.13	   811.40	 15
  5	 M 	   279.65	   753.88	 14
  6	 D 	   337.16	   688.36	 13
  7	 G 	   365.68	   630.85	 12
  8	 V 	   415.21	   602.34	 11
  9	 L 	   471.75	   552.81	 10
 10	 A 	   507.27	   496.26	  9
 11	 D 	   564.78	   460.75	  8
 12	 F 	   638.32	   403.23	  7
 13	 E 	   702.84	   329.70	  6
 14	 A 	   738.36	   265.18	  5
 15	 G 	   766.87	   229.66	  4
 16	 L 	   823.41	   201.15	  3
 17	 L 	   879.95	   144.61	  2
 18	 R 	        -  	    88.06	  1  _
```

**31. IPI00009867 Keratin, type II cytoskeletal 5**

| Sequence | Charge | M+H+ | XCorr | DeltaCn |
| --- | --- | --- | --- | --- |
| R.VSLAGACGVGGYGSR.S | 2 | 1411.539 | 3.611 | 0.596 |

+1 charge

```
		      B   	       Y
  1	 V 	   100.08	        -  	 15
  2	 S 	   187.11	  1311.75	 14
  3	 L 	   300.19	  1224.71	 13
  4	 A 	   371.23	  1111.63	 12
  5	 G 	   428.25	  1040.59	 11
  6	 A 	   499.29	   983.57	 10
  7	 C 	   659.45	   912.53	  9
  8	 G 	   716.47	   752.37	  8
  9	 V 	   815.54	   695.35	  7
 10	 G 	   872.56	   596.28	  6
 11	 G 	   929.59	   539.26	  5
 12	 Y 	  1092.65	   482.24	  4
 13	 G 	  1149.67	   319.17	  3
 14	 S 	  1236.70	   262.15	  2
 15	 R 	        -  	   175.12	  1  _
+2 charge
		      B   	       Y
  1	 V 	    50.54	        -  	 15
  2	 S 	    94.06	   656.38	 14
  3	 L 	   150.60	   612.86	 13
  4	 A 	   186.12	   556.32	 12
  5	 G 	   214.63	   520.80	 11
  6	 A 	   250.15	   492.29	 10
  7	 C 	   330.23	   456.77	  9
  8	 G 	   358.74	   376.69	  8
  9	 V 	   408.27	   348.18	  7
 10	 G 	   436.79	   298.64	  6
 11	 G 	   465.30	   270.13	  5
 12	 Y 	   546.83	   241.62	  4
 13	 G 	   575.34	   160.09	  3
 14	 S 	   618.85	   131.58	  2
 15	 R 	        -  	    88.06	  1  _
```

**32. IPI00289931 MADCAM1 protein**

| Sequence | Charge | M+H+ | XCorr | DeltaCn |
| --- | --- | --- | --- | --- |
| R.GLDTSLGAVQSDTGR.S | 2 | 1477.559 | 3.096 | 0.523 |

+1 charge

```
		      B   	       Y
  1	 G 	    58.03	        -  	 15
  2	 L 	   171.11	  1419.71	 14
  3	 D 	   286.14	  1306.62	 13
  4	 T 	   387.19	  1191.60	 12
  5	 S 	   474.22	  1090.55	 11
  6	 L 	   587.30	  1003.52	 10
  7	 G 	   644.32	   890.43	  9
  8	 A 	   715.36	   833.41	  8
  9	 V 	   814.43	   762.37	  7
 10	 Q 	   942.49	   663.31	  6
 11	 S 	  1029.52	   535.25	  5
 12	 D 	  1144.55	   448.22	  4
 13	 T 	  1245.60	   333.19	  3
 14	 G 	  1302.62	   232.14	  2
 15	 R 	        -  	   175.12	  1  _
+2 charge
		      B   	       Y
  1	 G 	    29.52	        -  	 15
  2	 L 	    86.06	   710.36	 14
  3	 D 	   143.57	   653.82	 13
  4	 T 	   194.10	   596.30	 12
  5	 S 	   237.61	   545.78	 11
  6	 L 	   294.16	   502.26	 10
  7	 G 	   322.67	   445.72	  9
  8	 A 	   358.18	   417.21	  8
  9	 V 	   407.72	   381.69	  7
 10	 Q 	   471.75	   332.16	  6
 11	 S 	   515.26	   268.13	  5
 12	 D 	   572.78	   224.61	  4
 13	 T 	   623.30	   167.10	  3
 14	 G 	   651.81	   116.57	  2
 15	 R 	        -  	    88.06	  1  _
```

**33. IPI00384404 Rheumatoid factor RF-ET9 (Fragment)**

| Sequence | Charge | M+H+ | XCorr | DeltaCn |
| --- | --- | --- | --- | --- |
| - .LVESGGGLVKPGGSLR.L | 2 | 1526.763 | 4.914 | 0.464 |

+1 charge

```
		      B   	       Y
  1	 L 	   114.09	        -  	 16
  2	 V 	   213.16	  1412.79	 15
  3	 E 	   342.20	  1313.72	 14
  4	 S 	   429.23	  1184.67	 13
  5	 G 	   486.26	  1097.64	 12
  6	 G 	   543.28	  1040.62	 11
  7	 G 	   600.30	   983.60	 10
  8	 L 	   713.38	   926.58	  9
  9	 V 	   812.45	   813.49	  8
 10	 K 	   940.55	   714.43	  7
 11	 P 	  1037.60	   586.33	  6
 12	 G 	  1094.62	   489.28	  5
 13	 G 	  1151.64	   432.26	  4
 14	 S 	  1238.67	   375.24	  3
 15	 L 	  1351.76	   288.20	  2
 16	 R 	        -  	   175.12	  1  _
+2 charge
		      B   	       Y
  1	 L 	    57.55	        -  	 16
  2	 V 	   107.08	   706.90	 15
  3	 E 	   171.60	   657.36	 14
  4	 S 	   215.12	   592.84	 13
  5	 G 	   243.63	   549.32	 12
  6	 G 	   272.14	   520.81	 11
  7	 G 	   300.65	   492.30	 10
  8	 L 	   357.20	   463.79	  9
  9	 V 	   406.73	   407.25	  8
 10	 K 	   470.78	   357.72	  7
 11	 P 	   519.30	   293.67	  6
 12	 G 	   547.81	   245.14	  5
 13	 G 	   576.32	   216.63	  4
 14	 S 	   619.84	   188.12	  3
 15	 L 	   676.38	   144.61	  2
 16	 R 	        -  	    88.06	  1  _
```

**34. IPI00939667 Isoform 1 of Tripeptidyl-peptidase 1**

| Sequence | Charge | M+H+ | XCorr | DeltaCn |
| --- | --- | --- | --- | --- |
| R.LFGGNFAHQASVAR.V | 2 | 1475.636 | 3.912 | 0.479 |

+1 charge

```
		      B   	       Y
  1	 L 	   114.09	        -  	 14
  2	 F 	   261.16	  1361.67	 13
  3	 G 	   318.18	  1214.60	 12
  4	 G 	   375.20	  1157.58	 11
  5	 N 	   489.25	  1100.56	 10
  6	 F 	   636.31	   986.52	  9
  7	 A 	   707.35	   839.45	  8
  8	 H 	   844.41	   768.41	  7
  9	 Q 	   972.47	   631.35	  6
 10	 A 	  1043.51	   503.29	  5
 11	 S 	  1130.54	   432.26	  4
 12	 V 	  1229.61	   345.22	  3
 13	 A 	  1300.64	   246.16	  2
 14	 R 	        -  	   175.12	  1  _
+2 charge
		      B   	       Y
  1	 L 	    57.55	        -  	 14
  2	 F 	   131.08	   681.34	 13
  3	 G 	   159.59	   607.80	 12
  4	 G 	   188.10	   579.29	 11
  5	 N 	   245.13	   550.78	 10
  6	 F 	   318.66	   493.76	  9
  7	 A 	   354.18	   420.23	  8
  8	 H 	   422.71	   384.71	  7
  9	 Q 	   486.74	   316.18	  6
 10	 A 	   522.26	   252.15	  5
 11	 S 	   565.77	   216.63	  4
 12	 V 	   615.31	   173.12	  3
 13	 A 	   650.83	   123.58	  2
 14	 R 	        -  	    88.06	  1  _
```

**35. IPI00298388 Isoform 1 of Phosphoinositide-3-kinase-interacting protein 1**

| Sequence | Charge | M+H+ | XCorr | DeltaCn |
| --- | --- | --- | --- | --- |
| R.SEAAAVQPVIGISQR.V | 2 | 1526.72 | 4.289 | 0.515 |

+1 charge

```
		      B   	       Y
  1	 S 	    88.04	        -  	 15
  2	 E 	   217.08	  1438.80	 14
  3	 A 	   288.12	  1309.76	 13
  4	 A 	   359.16	  1238.72	 12
  5	 A 	   430.19	  1167.68	 11
  6	 V 	   529.26	  1096.65	 10
  7	 Q 	   657.32	   997.58	  9
  8	 P 	   754.37	   869.52	  8
  9	 V 	   853.44	   772.47	  7
 10	 I 	   966.53	   673.40	  6
 11	 G 	  1023.55	   560.32	  5
 12	 I 	  1136.63	   503.29	  4
 13	 S 	  1223.66	   390.21	  3
 14	 Q 	  1351.72	   303.18	  2
 15	 R 	        -  	   175.12	  1  _
+2 charge
		      B   	       Y
  1	 S 	    44.52	        -  	 15
  2	 E 	   109.04	   719.90	 14
  3	 A 	   144.56	   655.38	 13
  4	 A 	   180.08	   619.86	 12
  5	 A 	   215.60	   584.35	 11
  6	 V 	   265.13	   548.83	 10
  7	 Q 	   329.16	   499.29	  9
  8	 P 	   377.69	   435.26	  8
  9	 V 	   427.22	   386.74	  7
 10	 I 	   483.77	   337.20	  6
 11	 G 	   512.28	   280.66	  5
 12	 I 	   568.82	   252.15	  4
 13	 S 	   612.34	   195.61	  3
 14	 Q 	   676.36	   152.09	  2
 15	 R 	        -  	    88.06	  1  _
```

**36. IPI00479116 Carboxypeptidase N subunit 2**

| Sequence | Charge | M+H+ | XCorr | DeltaCn |
| --- | --- | --- | --- | --- |
| R.SQCTYSNPEGTVVLACDQAQCR.W | 3 | 2545.683 | 3.799 | 0.299 |

+1 charge

```
		      B   	       Y
  1	 S 	    88.04	        -  	 22
  2	 Q 	   216.10	  2457.46	 21
  3	 C 	   376.26	  2329.40	 20
  4	 T 	   477.31	  2169.24	 19
  5	 Y 	   640.37	  2068.19	 18
  6	 S 	   727.41	  1905.13	 17
  7	 N 	   841.45	  1818.10	 16
  8	 P 	   938.50	  1704.05	 15
  9	 E 	  1067.54	  1607.00	 14
 10	 G 	  1124.57	  1477.96	 13
 11	 T 	  1225.61	  1420.94	 12
 12	 V 	  1324.68	  1319.89	 11
 13	 V 	  1423.75	  1220.82	 10
 14	 L 	  1536.83	  1121.75	  9
 15	 A 	  1607.87	  1008.67	  8
 16	 C 	  1768.04	   937.63	  7
 17	 D 	  1883.06	   777.47	  6
 18	 Q 	  2011.12	   662.44	  5
 19	 A 	  2082.16	   534.38	  4
 20	 Q 	  2210.22	   463.34	  3
 21	 C 	  2370.38	   335.28	  2
 22	 R 	        -  	   175.12	  1  _


+2 charge
		      B   	       Y
  1	 S 	    44.52	        -  	 22
  2	 Q 	   108.55	  1229.24	 21
  3	 C 	   188.64	  1165.21	 20
  4	 T 	   239.16	  1085.12	 19
  5	 Y 	   320.69	  1034.60	 18
  6	 S 	   364.21	   953.07	 17
  7	 N 	   421.23	   909.55	 16
  8	 P 	   469.75	   852.53	 15
  9	 E 	   534.28	   804.00	 14
 10	 G 	   562.79	   739.48	 13
 11	 T 	   613.31	   710.97	 12
 12	 V 	   662.84	   660.45	 11
 13	 V 	   712.38	   610.91	 10
 14	 L 	   768.92	   561.38	  9
 15	 A 	   804.44	   504.84	  8
 16	 C 	   884.52	   469.32	  7
 17	 D 	   942.04	   389.24	  6
 18	 Q 	  1006.06	   331.72	  5
 19	 A 	  1041.58	   267.69	  4
 20	 Q 	  1105.61	   232.18	  3
 21	 C 	  1185.70	   168.15	  2
 22	 R 	        -  	    88.06	  1  _


+3 charge
		      B   	       Y
  1	 S 	    30.02	        -  	 22
  2	 Q 	    72.70	   819.83	 21
  3	 C 	   126.09	   777.14	 20
  4	 T 	   159.78	   723.75	 19
  5	 Y 	   214.13	   690.07	 18
  6	 S 	   243.14	   635.71	 17
  7	 N 	   281.15	   606.70	 16
  8	 P 	   313.51	   568.69	 15
  9	 E 	   356.52	   536.34	 14
 10	 G 	   375.53	   493.32	 13
 11	 T 	   409.21	   474.32	 12
 12	 V 	   442.23	   440.63	 11
 13	 V 	   475.25	   407.61	 10
 14	 L 	   512.95	   374.59	  9
 15	 A 	   536.63	   336.89	  8
 16	 C 	   590.02	   313.22	  7
 17	 D 	   628.36	   259.83	  6
 18	 Q 	   671.05	   221.48	  5
 19	 A 	   694.72	   178.80	  4
 20	 Q 	   737.41	   155.12	  3
 21	 C 	   790.80	   112.43	  2
 22	 R 	        -  	    59.04	  1  _
```

**37. IPI00219827 Secreted Ly-6/uPAR-related protein 2**

| Sequence | Charge | M+H+ | XCorr | DeltaCn |
| --- | --- | --- | --- | --- |
| R.VLSNTEDLPLVTK.M | 2 | 1429.641 | 4.682 | 0.581 |

+1 charge

```
		      B   	       Y
  1	 V 	   100.08	        -  	 13
  2	 L 	   213.16	  1329.73	 12
  3	 S 	   300.19	  1216.64	 11
  4	 N 	   414.23	  1129.61	 10
  5	 T 	   515.28	  1015.57	  9
  6	 E 	   644.32	   914.52	  8
  7	 D 	   759.35	   785.48	  7
  8	 L 	   872.44	   670.45	  6
  9	 P 	   969.49	   557.37	  5
 10	 L 	  1082.57	   460.31	  4
 11	 V 	  1181.64	   347.23	  3
 12	 T 	  1282.69	   248.16	  2
 13	 K 	        -  	   147.11	  1  _
+2 charge
		      B   	       Y
  1	 V 	    50.54	        -  	 13
  2	 L 	   107.08	   665.37	 12
  3	 S 	   150.60	   608.82	 11
  4	 N 	   207.62	   565.31	 10
  5	 T 	   258.14	   508.29	  9
  6	 E 	   322.67	   457.76	  8
  7	 D 	   380.18	   393.24	  7
  8	 L 	   436.72	   335.73	  6
  9	 P 	   485.25	   279.19	  5
 10	 L 	   541.79	   230.66	  4
 11	 V 	   591.32	   174.12	  3
 12	 T 	   641.85	   124.58	  2
 13	 K 	        -  	    74.06	  1  _
```

**38. IPI00384401 Myosin-reactive immunoglobulin kappa chain variable region (Fragment)**

| Sequence | Charge | M+H+ | XCorr | DeltaCn |
| --- | --- | --- | --- | --- |
| - .EIVMTQSPATLSVSPGER.A | 2 | 1903.147 | 5.113 | 0.492 |

+1 charge

```
		      B   	       Y
  1	 E 	   130.05	        -  	 18
  2	 I 	   243.13	  1772.92	 17
  3	 V 	   342.20	  1659.84	 16
  4	 M 	   473.24	  1560.77	 15
  5	 T 	   574.29	  1429.73	 14
  6	 Q 	   702.35	  1328.68	 13
  7	 S 	   789.38	  1200.62	 12
  8	 P 	   886.43	  1113.59	 11
  9	 A 	   957.47	  1016.54	 10
 10	 T 	  1058.52	   945.50	  9
 11	 L 	  1171.60	   844.45	  8
 12	 S 	  1258.63	   731.37	  7
 13	 V 	  1357.70	   644.34	  6
 14	 S 	  1444.74	   545.27	  5
 15	 P 	  1541.79	   458.24	  4
 16	 G 	  1598.81	   361.18	  3
 17	 E 	  1727.85	   304.16	  2
 18	 R 	        -  	   175.12	  1  _
+2 charge
		      B   	       Y
  1	 E 	    65.53	        -  	 18
  2	 I 	   122.07	   886.96	 17
  3	 V 	   171.60	   830.42	 16
  4	 M 	   237.13	   780.89	 15
  5	 T 	   287.65	   715.37	 14
  6	 Q 	   351.68	   664.84	 13
  7	 S 	   395.19	   600.81	 12
  8	 P 	   443.72	   557.30	 11
  9	 A 	   479.24	   508.77	 10
 10	 T 	   529.76	   473.25	  9
 11	 L 	   586.31	   422.73	  8
 12	 S 	   629.82	   366.19	  7
 13	 V 	   679.36	   322.67	  6
 14	 S 	   722.87	   273.14	  5
 15	 P 	   771.40	   229.62	  4
 16	 G 	   799.91	   181.10	  3
 17	 E 	   864.43	   152.58	  2
 18	 R 	        -  	    88.06	  1  _
```

**39. IPI00011694 Trypsin-1**

| Sequence | Charge | M+H+ | XCorr | DeltaCn |
| --- | --- | --- | --- | --- |
| K.TLNNDIMLIK.L | 2 | 1175.424 | 3.59 | 0.226 |

+1 charge

```
		      B   	       Y
  1	 T 	   102.05	        -  	 10
  2	 L 	   215.14	  1073.60	  9
  3	 N 	   329.18	   960.52	  8
  4	 N 	   443.22	   846.48	  7
  5	 D 	   558.25	   732.43	  6
  6	 I 	   671.34	   617.41	  5
  7	 M 	   802.38	   504.32	  4
  8	 L 	   915.46	   373.28	  3
  9	 I 	  1028.54	   260.20	  2
 10	 K 	        -  	   147.11	  1  _
+2 charge
		      B   	       Y
  1	 T 	    51.53	        -  	 10
  2	 L 	   108.07	   537.30	  9
  3	 N 	   165.09	   480.76	  8
  4	 N 	   222.12	   423.74	  7
  5	 D 	   279.63	   366.72	  6
  6	 I 	   336.17	   309.21	  5
  7	 M 	   401.69	   252.66	  4
  8	 L 	   458.23	   187.14	  3
  9	 I 	   514.78	   130.60	  2
 10	 K 	        -  	    74.06	  1  _
```

**40. IPI00387113 Ig kappa chain V-III region B6**

| Sequence | Charge | M+H+ | XCorr | DeltaCn |
| --- | --- | --- | --- | --- |
| - .ZIVLTZSPGTLSLSPGZR.A | 2 | 1884.616 | 4.236 | 0.566 |

+1 charge

```
		      B   	       Y
  1	 Z 	   129.56	        -  	 18
  2	 I 	   242.64	  1754.96	 17
  3	 V 	   341.71	  1641.88	 16
  4	 L 	   454.79	  1542.81	 15
  5	 T 	   555.84	  1429.73	 14
  6	 Z 	   684.39	  1328.68	 13
  7	 S 	   771.42	  1200.13	 12
  8	 P 	   868.48	  1113.10	 11
  9	 G 	   925.50	  1016.05	 10
 10	 T 	  1026.55	   959.02	  9
 11	 L 	  1139.63	   857.98	  8
 12	 S 	  1226.66	   744.89	  7
 13	 L 	  1339.75	   657.86	  6
 14	 S 	  1426.78	   544.78	  5
 15	 P 	  1523.83	   457.74	  4
 16	 G 	  1580.85	   360.69	  3
 17	 Z 	  1709.40	   303.67	  2
 18	 R 	        -  	   175.12	  1  _
+2 charge
		      B   	       Y
  1	 Z 	    65.28	        -  	 18
  2	 I 	   121.82	   877.99	 17
  3	 V 	   171.36	   821.44	 16
  4	 L 	   227.90	   771.91	 15
  5	 T 	   278.42	   715.37	 14
  6	 Z 	   342.70	   664.84	 13
  7	 S 	   386.22	   600.57	 12
  8	 P 	   434.74	   557.05	 11
  9	 G 	   463.25	   508.53	 10
 10	 T 	   513.78	   480.02	  9
 11	 L 	   570.32	   429.49	  8
 12	 S 	   613.83	   372.95	  7
 13	 L 	   670.38	   329.43	  6
 14	 S 	   713.89	   272.89	  5
 15	 P 	   762.42	   229.38	  4
 16	 G 	   790.93	   180.85	  3
 17	 Z 	   855.21	   152.34	  2
 18	 R 	        -  	    88.06	  1  _
```

**41. IPI00003865 Isoform 1 of Heat shock cognate 71 kDa protein**

| Sequence | Charge | M+H+ | XCorr | DeltaCn |
| --- | --- | --- | --- | --- |
| R.IINEPTAAAIAYGLDKK.V | 2 | 1789.066 | 2.854 | 0.494 |

+1 charge

```
		      B   	       Y
  1	 I 	   114.09	        -  	 17
  2	 I 	   227.18	  1674.91	 16
  3	 N 	   341.22	  1561.82	 15
  4	 E 	   470.26	  1447.78	 14
  5	 P 	   567.31	  1318.74	 13
  6	 T 	   668.36	  1221.68	 12
  7	 A 	   739.40	  1120.64	 11
  8	 A 	   810.44	  1049.60	 10
  9	 A 	   881.47	   978.56	  9
 10	 I 	   994.56	   907.52	  8
 11	 A 	  1065.59	   794.44	  7
 12	 Y 	  1228.66	   723.40	  6
 13	 G 	  1285.68	   560.34	  5
 14	 L 	  1398.76	   503.32	  4
 15	 D 	  1513.79	   390.23	  3
 16	 K 	  1641.88	   275.21	  2
 17	 K 	        -  	   147.11	  1  _
+2 charge
		      B   	       Y
  1	 I 	    57.55	        -  	 17
  2	 I 	   114.09	   837.96	 16
  3	 N 	   171.11	   781.41	 15
  4	 E 	   235.63	   724.39	 14
  5	 P 	   284.16	   659.87	 13
  6	 T 	   334.68	   611.35	 12
  7	 A 	   370.20	   560.82	 11
  8	 A 	   405.72	   525.30	 10
  9	 A 	   441.24	   489.78	  9
 10	 I 	   497.78	   454.27	  8
 11	 A 	   533.30	   397.72	  7
 12	 Y 	   614.83	   362.21	  6
 13	 G 	   643.34	   280.67	  5
 14	 L 	   699.89	   252.16	  4
 15	 D 	   757.40	   195.62	  3
 16	 K 	   821.45	   138.11	  2
 17	 K 	        -  	    74.06	  1  _
```

**42. IPI00000874 Peroxiredoxin-1**

| Sequence | Charge | M+H+ | XCorr | DeltaCn |
| --- | --- | --- | --- | --- |
| R.TIAQDYGVLKADEGISFR.G | 2 | 1984.199 | 3.348 | 0.617 |

+1 charge

```
		      B   	       Y
  1	 T 	   102.05	        -  	 18
  2	 I 	   215.14	  1881.97	 17
  3	 A 	   286.18	  1768.89	 16
  4	 Q 	   414.23	  1697.85	 15
  5	 D 	   529.26	  1569.79	 14
  6	 Y 	   692.32	  1454.76	 13
  7	 G 	   749.35	  1291.70	 12
  8	 V 	   848.41	  1234.68	 11
  9	 L 	   961.50	  1135.61	 10
 10	 K 	  1089.59	  1022.53	  9
 11	 A 	  1160.63	   894.43	  8
 12	 D 	  1275.66	   823.39	  7
 13	 E 	  1404.70	   708.37	  6
 14	 G 	  1461.72	   579.32	  5
 15	 I 	  1574.81	   522.30	  4
 16	 S 	  1661.84	   409.22	  3
 17	 F 	  1808.91	   322.19	  2
 18	 R 	        -  	   175.12	  1  _
+2 charge
		      B   	       Y
  1	 T 	    51.53	        -  	 18
  2	 I 	   108.07	   941.49	 17
  3	 A 	   143.59	   884.95	 16
  4	 Q 	   207.62	   849.43	 15
  5	 D 	   265.13	   785.40	 14
  6	 Y 	   346.67	   727.89	 13
  7	 G 	   375.18	   646.35	 12
  8	 V 	   424.71	   617.84	 11
  9	 L 	   481.25	   568.31	 10
 10	 K 	   545.30	   511.77	  9
 11	 A 	   580.82	   447.72	  8
 12	 D 	   638.33	   412.20	  7
 13	 E 	   702.85	   354.69	  6
 14	 G 	   731.36	   290.17	  5
 15	 I 	   787.91	   261.66	  4
 16	 S 	   831.42	   205.11	  3
 17	 F 	   904.96	   161.60	  2
 18	 R 	        -  	    88.06	  1  _
```

**43. IPI00005526 PRO0419**

| Sequence | Charge | M+H+ | XCorr | DeltaCn |
| --- | --- | --- | --- | --- |
| R.RSELFILPQDDLDPPNFPCSR.P | 3 | 2517.772 | 3.857 | 0.285 |

+1 charge

```
		      B   	       Y
  1	 R 	   157.11	        -  	 21
  2	 S 	   244.14	  2360.26	 20
  3	 E 	   373.18	  2273.23	 19
  4	 L 	   486.27	  2144.18	 18
  5	 F 	   633.34	  2031.10	 17
  6	 I 	   746.42	  1884.03	 16
  7	 L 	   859.50	  1770.95	 15
  8	 P 	   956.56	  1657.86	 14
  9	 Q 	  1084.61	  1560.81	 13
 10	 D 	  1199.64	  1432.75	 12
 11	 D 	  1314.67	  1317.72	 11
 12	 L 	  1427.75	  1202.70	 10
 13	 D 	  1542.78	  1089.61	  9
 14	 P 	  1639.83	   974.59	  8
 15	 P 	  1736.89	   877.53	  7
 16	 N 	  1850.93	   780.48	  6
 17	 F 	  1998.00	   666.44	  5
 18	 P 	  2095.05	   519.37	  4
 19	 C 	  2255.21	   422.32	  3
 20	 S 	  2342.25	   262.15	  2
 21	 R 	        -  	   175.12	  1  _


+2 charge
		      B   	       Y
  1	 R 	    79.06	        -  	 21
  2	 S 	   122.57	  1180.63	 20
  3	 E 	   187.10	  1137.12	 19
  4	 L 	   243.64	  1072.59	 18
  5	 F 	   317.17	  1016.05	 17
  6	 I 	   373.71	   942.52	 16
  7	 L 	   430.26	   885.98	 15
  8	 P 	   478.78	   829.43	 14
  9	 Q 	   542.81	   780.91	 13
 10	 D 	   600.32	   716.88	 12
 11	 D 	   657.84	   659.37	 11
 12	 L 	   714.38	   601.85	 10
 13	 D 	   771.89	   545.31	  9
 14	 P 	   820.42	   487.80	  8
 15	 P 	   868.95	   439.27	  7
 16	 N 	   925.97	   390.74	  6
 17	 F 	   999.50	   333.72	  5
 18	 P 	  1048.03	   260.19	  4
 19	 C 	  1128.11	   211.66	  3
 20	 S 	  1171.63	   131.58	  2
 21	 R 	        -  	    88.06	  1  _


+3 charge
		      B   	       Y
  1	 R 	    53.04	        -  	 21
  2	 S 	    82.05	   787.42	 20
  3	 E 	   125.07	   758.41	 19
  4	 L 	   162.76	   715.40	 18
  5	 F 	   211.78	   677.70	 17
  6	 I 	   249.48	   628.68	 16
  7	 L 	   287.17	   590.99	 15
  8	 P 	   319.52	   553.29	 14
  9	 Q 	   362.21	   520.94	 13
 10	 D 	   400.55	   478.26	 12
 11	 D 	   438.89	   439.91	 11
 12	 L 	   476.59	   401.57	 10
 13	 D 	   514.93	   363.88	  9
 14	 P 	   547.28	   325.53	  8
 15	 P 	   579.63	   293.18	  7
 16	 N 	   617.65	   260.83	  6
 17	 F 	   666.67	   222.82	  5
 18	 P 	   699.02	   173.79	  4
 19	 C 	   752.41	   141.44	  3
 20	 S 	   781.42	    88.06	  2
 21	 R 	        -  	    59.04	  1  _
```

**44. IPI00018901 Isoform 1 of Gamma-glutamyltranspeptidase 1**

| Sequence | Charge | M+H+ | XCorr | DeltaCn |
| --- | --- | --- | --- | --- |
| R.TVIEQQPVLCEVFCR.D | 2 | 1879.134 | 3.879 | 0.381 |

+1 charge

```
		      B   	       Y
  1	 T 	   102.05	        -  	 15
  2	 V 	   201.12	  1777.15	 14
  3	 I 	   314.21	  1678.08	 13
  4	 E 	   443.25	  1564.99	 12
  5	 Q 	   571.31	  1435.95	 11
  6	 Q 	   699.37	  1307.89	 10
  7	 P 	   796.42	  1179.83	  9
  8	 V 	   895.49	  1082.78	  8
  9	 L 	  1008.57	   983.71	  7
 10	 C 	  1168.74	   870.63	  6
 11	 E 	  1297.78	   710.46	  5
 12	 V 	  1396.85	   581.42	  4
 13	 F 	  1543.92	   482.35	  3
 14	 C 	  1704.08	   335.28	  2
 15	 R 	        -  	   175.12	  1  _
+2 charge
		      B   	       Y
  1	 T 	    51.53	        -  	 15
  2	 V 	   101.07	   889.08	 14
  3	 I 	   157.61	   839.54	 13
  4	 E 	   222.13	   783.00	 12
  5	 Q 	   286.16	   718.48	 11
  6	 Q 	   350.19	   654.45	 10
  7	 P 	   398.71	   590.42	  9
  8	 V 	   448.25	   541.89	  8
  9	 L 	   504.79	   492.36	  7
 10	 C 	   584.87	   435.82	  6
 11	 E 	   649.39	   355.74	  5
 12	 V 	   698.93	   291.21	  4
 13	 F 	   772.46	   241.68	  3
 14	 C 	   852.54	   168.15	  2
 15	 R 	        -  	    88.06	  1  _
```

**45. IPI00290085 Cadherin-2**

| Sequence | Charge | M+H+ | XCorr | DeltaCn |
| --- | --- | --- | --- | --- |
| R.FAIQTDPNSNDGLVTVVKPIDFETNR.M | 3 | 2892.17 | 4.532 | 0.573 |

+1 charge

```
		      B   	       Y
  1	 F 	   148.08	        -  	 26
  2	 A 	   219.11	  2743.39	 25
  3	 I 	   332.20	  2672.35	 24
  4	 Q 	   460.26	  2559.27	 23
  5	 T 	   561.30	  2431.21	 22
  6	 D 	   676.33	  2330.16	 21
  7	 P 	   773.38	  2215.14	 20
  8	 N 	   887.43	  2118.08	 19
  9	 S 	   974.46	  2004.04	 18
 10	 N 	  1088.50	  1917.01	 17
 11	 D 	  1203.53	  1802.96	 16
 12	 G 	  1260.55	  1687.94	 15
 13	 L 	  1373.63	  1630.92	 14
 14	 V 	  1472.70	  1517.83	 13
 15	 T 	  1573.75	  1418.76	 12
 16	 V 	  1672.82	  1317.72	 11
 17	 V 	  1771.89	  1218.65	 10
 18	 K 	  1899.98	  1119.58	  9
 19	 P 	  1997.03	   991.48	  8
 20	 I 	  2110.12	   894.43	  7
 21	 D 	  2225.14	   781.35	  6
 22	 F 	  2372.21	   666.32	  5
 23	 E 	  2501.26	   519.25	  4
 24	 T 	  2602.30	   390.21	  3
 25	 N 	  2716.35	   289.16	  2
 26	 R 	        -  	   175.12	  1  _


+2 charge
		      B   	       Y
  1	 F 	    74.54	        -  	 26
  2	 A 	   110.06	  1372.20	 25
  3	 I 	   166.60	  1336.68	 24
  4	 Q 	   230.63	  1280.14	 23
  5	 T 	   281.16	  1216.11	 22
  6	 D 	   338.67	  1165.58	 21
  7	 P 	   387.20	  1108.07	 20
  8	 N 	   444.22	  1059.54	 19
  9	 S 	   487.73	  1002.52	 18
 10	 N 	   544.75	   959.01	 17
 11	 D 	   602.27	   901.99	 16
 12	 G 	   630.78	   844.47	 15
 13	 L 	   687.32	   815.96	 14
 14	 V 	   736.85	   759.42	 13
 15	 T 	   787.38	   709.89	 12
 16	 V 	   836.91	   659.36	 11
 17	 V 	   886.45	   609.83	 10
 18	 K 	   950.49	   560.29	  9
 19	 P 	   999.02	   496.25	  8
 20	 I 	  1055.56	   447.72	  7
 21	 D 	  1113.08	   391.18	  6
 22	 F 	  1186.61	   333.66	  5
 23	 E 	  1251.13	   260.13	  4
 24	 T 	  1301.66	   195.61	  3
 25	 N 	  1358.68	   145.08	  2
 26	 R 	        -  	    88.06	  1  _


+3 charge
		      B   	       Y
  1	 F 	    50.03	        -  	 26
  2	 A 	    73.71	   915.13	 25
  3	 I 	   111.40	   891.46	 24
  4	 Q 	   154.09	   853.76	 23
  5	 T 	   187.77	   811.07	 22
  6	 D 	   226.11	   777.39	 21
  7	 P 	   258.47	   739.05	 20
  8	 N 	   296.48	   706.70	 19
  9	 S 	   325.49	   668.68	 18
 10	 N 	   363.51	   639.67	 17
 11	 D 	   401.85	   601.66	 16
 12	 G 	   420.85	   563.32	 15
 13	 L 	   458.55	   544.31	 14
 14	 V 	   491.57	   506.62	 13
 15	 T 	   525.25	   473.59	 12
 16	 V 	   558.28	   439.91	 11
 17	 V 	   591.30	   406.89	 10
 18	 K 	   634.00	   373.86	  9
 19	 P 	   666.35	   331.17	  8
 20	 I 	   704.04	   298.82	  7
 21	 D 	   742.39	   261.12	  6
 22	 F 	   791.41	   222.78	  5
 23	 E 	   834.42	   173.76	  4
 24	 T 	   868.11	   130.74	  3
 25	 N 	   906.12	    97.06	  2
 26	 R 	        -  	    59.04	  1  _
```

**46. IPI00872780 Annexin A4**

| Sequence | Charge | M+H+ | XCorr | DeltaCn |
| --- | --- | --- | --- | --- |
| K.GLGTDEDAIISVLAYR.N | 2 | 1693.879 | 2.814 | 0.356 |

+1 charge

```
		      B   	       Y
  1	 G 	    58.03	        -  	 16
  2	 L 	   171.11	  1635.86	 15
  3	 G 	   228.13	  1522.77	 14
  4	 T 	   329.18	  1465.75	 13
  5	 D 	   444.21	  1364.71	 12
  6	 E 	   573.25	  1249.68	 11
  7	 D 	   688.28	  1120.64	 10
  8	 A 	   759.32	  1005.61	  9
  9	 I 	   872.40	   934.57	  8
 10	 I 	   985.48	   821.49	  7
 11	 S 	  1072.52	   708.40	  6
 12	 V 	  1171.58	   621.37	  5
 13	 L 	  1284.67	   522.30	  4
 14	 A 	  1355.71	   409.22	  3
 15	 Y 	  1518.77	   338.18	  2
 16	 R 	        -  	   175.12	  1  _
+2 charge
		      B   	       Y
  1	 G 	    29.52	        -  	 16
  2	 L 	    86.06	   818.43	 15
  3	 G 	   114.57	   761.89	 14
  4	 T 	   165.09	   733.38	 13
  5	 D 	   222.61	   682.86	 12
  6	 E 	   287.13	   625.34	 11
  7	 D 	   344.64	   560.82	 10
  8	 A 	   380.16	   503.31	  9
  9	 I 	   436.70	   467.79	  8
 10	 I 	   493.25	   411.25	  7
 11	 S 	   536.76	   354.71	  6
 12	 V 	   586.30	   311.19	  5
 13	 L 	   642.84	   261.66	  4
 14	 A 	   678.36	   205.11	  3
 15	 Y 	   759.89	   169.59	  2
 16	 R 	        -  	    88.06	  1  _
```

**47. IPI00019954 Cystatin-M**

| Sequence | Charge | M+H+ | XCorr | DeltaCn |
| --- | --- | --- | --- | --- |
| K.AQSQLVAGIKYFLTMEMGSTDCR.K | 3 | 2607.982 | 3.613 | 0.486 |

+1 charge

```
		      B   	       Y
  1	 A 	    72.04	        -  	 23
  2	 Q 	   200.10	  2535.34	 22
  3	 S 	   287.13	  2407.28	 21
  4	 Q 	   415.19	  2320.25	 20
  5	 L 	   528.28	  2192.19	 19
  6	 V 	   627.35	  2079.11	 18
  7	 A 	   698.38	  1980.04	 17
  8	 G 	   755.40	  1909.00	 16
  9	 I 	   868.49	  1851.98	 15
 10	 K 	   996.58	  1738.89	 14
 11	 Y 	  1159.65	  1610.80	 13
 12	 F 	  1306.72	  1447.74	 12
 13	 L 	  1419.80	  1300.67	 11
 14	 T 	  1520.85	  1187.58	 10
 15	 M 	  1651.89	  1086.54	  9
 16	 E 	  1780.93	   955.50	  8
 17	 M 	  1911.97	   826.45	  7
 18	 G 	  1968.99	   695.41	  6
 19	 S 	  2056.02	   638.39	  5
 20	 T 	  2157.07	   551.36	  4
 21	 D 	  2272.10	   450.31	  3
 22	 C 	  2432.26	   335.28	  2
 23	 R 	        -  	   175.12	  1  _


+2 charge
		      B   	       Y
  1	 A 	    36.53	        -  	 23
  2	 Q 	   100.56	  1268.17	 22
  3	 S 	   144.07	  1204.14	 21
  4	 Q 	   208.10	  1160.63	 20
  5	 L 	   264.64	  1096.60	 19
  6	 V 	   314.18	  1040.06	 18
  7	 A 	   349.70	   990.52	 17
  8	 G 	   378.21	   955.00	 16
  9	 I 	   434.75	   926.49	 15
 10	 K 	   498.80	   869.95	 14
 11	 Y 	   580.33	   805.90	 13
 12	 F 	   653.86	   724.37	 12
 13	 L 	   710.40	   650.84	 11
 14	 T 	   760.93	   594.30	 10
 15	 M 	   826.45	   543.77	  9
 16	 E 	   890.97	   478.25	  8
 17	 M 	   956.49	   413.73	  7
 18	 G 	   985.00	   348.21	  6
 19	 S 	  1028.52	   319.70	  5
 20	 T 	  1079.04	   276.18	  4
 21	 D 	  1136.55	   225.66	  3
 22	 C 	  1216.64	   168.15	  2
 23	 R 	        -  	    88.06	  1  _


+3 charge
		      B   	       Y
  1	 A 	    24.69	        -  	 23
  2	 Q 	    67.37	   845.78	 22
  3	 S 	    96.38	   803.10	 21
  4	 Q 	   139.07	   774.09	 20
  5	 L 	   176.76	   731.40	 19
  6	 V 	   209.79	   693.71	 18
  7	 A 	   233.47	   660.68	 17
  8	 G 	   252.47	   637.00	 16
  9	 I 	   290.17	   618.00	 15
 10	 K 	   332.87	   580.30	 14
 11	 Y 	   387.22	   537.60	 13
 12	 F 	   436.24	   483.25	 12
 13	 L 	   473.94	   434.23	 11
 14	 T 	   507.62	   396.53	 10
 15	 M 	   551.30	   362.85	  9
 16	 E 	   594.31	   319.17	  8
 17	 M 	   638.00	   276.16	  7
 18	 G 	   657.00	   232.48	  6
 19	 S 	   686.01	   213.47	  5
 20	 T 	   719.70	   184.46	  4
 21	 D 	   758.04	   150.78	  3
 22	 C 	   811.43	   112.43	  2
 23	 R 	        -  	    59.04	  1  _
```

**48. IPI00292150 Latent-transforming growth factor beta-binding protein 2**

| Sequence | Charge | M+H+ | XCorr | DeltaCn |
| --- | --- | --- | --- | --- |
| R.CHCSPGYVAEAGPPHCTAKE.- | 3 | 2229.373 | 6.001 | 0.386 |

+1 charge

```
		      B   	       Y
  1	 C 	   161.17	        -  	 20
  2	 H 	   298.23	  2068.17	 19
  3	 C 	   458.40	  1931.11	 18
  4	 S 	   545.43	  1770.95	 17
  5	 P 	   642.48	  1683.91	 16
  6	 G 	   699.50	  1586.86	 15
  7	 Y 	   862.57	  1529.84	 14
  8	 V 	   961.63	  1366.78	 13
  9	 A 	  1032.67	  1267.71	 12
 10	 E 	  1161.71	  1196.67	 11
 11	 A 	  1232.75	  1067.63	 10
 12	 G 	  1289.77	   996.59	  9
 13	 P 	  1386.83	   939.57	  8
 14	 P 	  1483.88	   842.52	  7
 15	 H 	  1620.94	   745.46	  6
 16	 C 	  1781.10	   608.41	  5
 17	 T 	  1882.15	   448.24	  4
 18	 A 	  1953.19	   347.19	  3
 19	 K 	  2081.28	   276.16	  2
 20	 E 	        -  	   148.06	  1  _
+2 charge
		      B   	       Y
  1	 C 	    81.09	        -  	 20
  2	 H 	   149.62	  1034.59	 19
  3	 C 	   229.70	   966.06	 18
  4	 S 	   273.22	   885.98	 17
  5	 P 	   321.74	   842.46	 16
  6	 G 	   350.26	   793.93	 15
  7	 Y 	   431.79	   765.42	 14
  8	 V 	   481.32	   683.89	 13
  9	 A 	   516.84	   634.36	 12
 10	 E 	   581.36	   598.84	 11
 11	 A 	   616.88	   534.32	 10
 12	 G 	   645.39	   498.80	  9
 13	 P 	   693.92	   470.29	  8
 14	 P 	   742.44	   421.76	  7
 15	 H 	   810.97	   373.24	  6
 16	 C 	   891.05	   304.71	  5
 17	 T 	   941.58	   224.62	  4
 18	 A 	   977.10	   174.10	  3
 19	 K 	  1041.14	   138.58	  2
 20	 E 	        -  	    74.53	  1  _
+3 charge
		      B   	       Y
  1	 C 	    54.40	        -  	 20
  2	 H 	   100.08	   690.06	 19
  3	 C 	   153.47	   644.38	 18
  4	 S 	   182.48	   590.99	 17
  5	 P 	   214.83	   561.98	 16
  6	 G 	   233.84	   529.63	 15
  7	 Y 	   288.19	   510.62	 14
  8	 V 	   321.22	   456.26	 13
  9	 A 	   344.90	   423.24	 12
 10	 E 	   387.91	   399.56	 11
 11	 A 	   411.59	   356.55	 10
 12	 G 	   430.60	   332.87	  9
 13	 P 	   462.95	   313.86	  8
 14	 P 	   495.30	   281.51	  7
 15	 H 	   540.98	   249.16	  6
 16	 C 	   594.37	   203.47	  5
 17	 T 	   628.05	   150.08	  4
 18	 A 	   651.73	   116.40	  3
 19	 K 	   694.43	    92.72	  2
 20	 E 	        -  	    50.02	  1  _
```

**49. IPI00216914 Vitelline membrane outer layer protein 1 homolog**

| Sequence | Charge | M+H+ | XCorr | DeltaCn |
| --- | --- | --- | --- | --- |
| R.VEAPTTLGDNTAANNVR.F | 2 | 1743.857 | 3.793 | 0.426 |

+1 charge

```
		      B   	       Y
  1	 V 	   100.08	        -  	 17
  2	 E 	   229.12	  1643.80	 16
  3	 A 	   300.16	  1514.76	 15
  4	 P 	   397.21	  1443.72	 14
  5	 T 	   498.26	  1346.67	 13
  6	 T 	   599.30	  1245.62	 12
  7	 L 	   712.39	  1144.57	 11
  8	 G 	   769.41	  1031.49	 10
  9	 D 	   884.44	   974.46	  9
 10	 N 	   998.48	   859.44	  8
 11	 T 	  1099.53	   745.40	  7
 12	 A 	  1170.56	   644.35	  6
 13	 A 	  1241.60	   573.31	  5
 14	 N 	  1355.64	   502.27	  4
 15	 N 	  1469.69	   388.23	  3
 16	 V 	  1568.76	   274.19	  2
 17	 R 	        -  	   175.12	  1  _
+2 charge
		      B   	       Y
  1	 V 	    50.54	        -  	 17
  2	 E 	   115.06	   822.40	 16
  3	 A 	   150.58	   757.88	 15
  4	 P 	   199.11	   722.36	 14
  5	 T 	   249.63	   673.84	 13
  6	 T 	   300.16	   623.31	 12
  7	 L 	   356.70	   572.79	 11
  8	 G 	   385.21	   516.25	 10
  9	 D 	   442.72	   487.74	  9
 10	 N 	   499.74	   430.22	  8
 11	 T 	   550.27	   373.20	  7
 12	 A 	   585.79	   322.68	  6
 13	 A 	   621.30	   287.16	  5
 14	 N 	   678.33	   251.64	  4
 15	 N 	   735.35	   194.62	  3
 16	 V 	   784.88	   137.60	  2
 17	 R 	        -  	    88.06	  1  _
```

**50. IPI00028030 Cartilage oligomeric matrix protein**

| Sequence | Charge | M+H+ | XCorr | DeltaCn |
| --- | --- | --- | --- | --- |
| K.NTVMECDACGMQQSVR.T | 2 | 1887.073 | 3.496 | 0.363 |

+1 charge

```
		      B   	       Y
  1	 N 	   115.05	        -  	 16
  2	 T 	   216.10	  1771.99	 15
  3	 V 	   315.17	  1670.94	 14
  4	 M 	   446.21	  1571.88	 13
  5	 E 	   575.25	  1440.84	 12
  6	 C 	   735.41	  1311.79	 11
  7	 D 	   850.44	  1151.63	 10
  8	 A 	   921.48	  1036.60	  9
  9	 C 	  1081.64	   965.56	  8
 10	 G 	  1138.67	   805.40	  7
 11	 M 	  1269.71	   748.38	  6
 12	 Q 	  1397.76	   617.34	  5
 13	 Q 	  1525.82	   489.28	  4
 14	 S 	  1612.86	   361.22	  3
 15	 V 	  1711.92	   274.19	  2
 16	 R 	        -  	   175.12	  1  _
+2 charge
		      B   	       Y
  1	 N 	    58.03	        -  	 16
  2	 T 	   108.55	   886.50	 15
  3	 V 	   158.09	   835.98	 14
  4	 M 	   223.61	   786.44	 13
  5	 E 	   288.13	   720.92	 12
  6	 C 	   368.21	   656.40	 11
  7	 D 	   425.72	   576.32	 10
  8	 A 	   461.24	   518.80	  9
  9	 C 	   541.33	   483.29	  8
 10	 G 	   569.84	   403.20	  7
 11	 M 	   635.36	   374.69	  6
 12	 Q 	   699.39	   309.17	  5
 13	 Q 	   763.42	   245.14	  4
 14	 S 	   806.93	   181.11	  3
 15	 V 	   856.47	   137.60	  2
 16	 R 	        -  	    88.06	  1  _
```

**51. IPI00009997 N-acetyllactosaminide beta-1,3-N-acetylglucosaminyltransferase**

| Sequence | Charge | M+H+ | XCorr | DeltaCn |
| --- | --- | --- | --- | --- |
| R.SCQEVFDKLAR.V | 2 | 1353.5 | 3.109 | 0.392 |

+1 charge

```
		      B   	       Y
  1	 S 	    88.04	        -  	 11
  2	 C 	   248.20	  1265.77	 10
  3	 Q 	   376.26	  1105.60	  9
  4	 E 	   505.31	   977.54	  8
  5	 V 	   604.37	   848.50	  7
  6	 F 	   751.44	   749.43	  6
  7	 D 	   866.47	   602.36	  5
  8	 K 	   994.56	   487.34	  4
  9	 L 	  1107.65	   359.24	  3
 10	 A 	  1178.69	   246.16	  2
 11	 R 	        -  	   175.12	  1  _
+2 charge
		      B   	       Y
  1	 S 	    44.52	        -  	 11
  2	 C 	   124.61	   633.39	 10
  3	 Q 	   188.64	   553.30	  9
  4	 E 	   253.16	   489.27	  8
  5	 V 	   302.69	   424.75	  7
  6	 F 	   376.22	   375.22	  6
  7	 D 	   433.74	   301.68	  5
  8	 K 	   497.79	   244.17	  4
  9	 L 	   554.33	   180.12	  3
 10	 A 	   589.85	   123.58	  2
 11	 R 	        -  	    88.06	  1  _
```

**52. IPI00026944 Isoform 1 of Nidogen-1**

| Sequence | Charge | M+H+ | XCorr | DeltaCn |
| --- | --- | --- | --- | --- |
| K.VYYREDLSPSITQR.A | 2 | 1727.899 | 4.481 | 0.419 |

+1 charge

```
		      B   	       Y
  1	 V 	   100.08	        -  	 14
  2	 Y 	   263.14	  1627.81	 13
  3	 Y 	   426.20	  1464.74	 12
  4	 R 	   582.30	  1301.68	 11
  5	 E 	   711.35	  1145.58	 10
  6	 D 	   826.37	  1016.54	  9
  7	 L 	   939.46	   901.51	  8
  8	 S 	  1026.49	   788.43	  7
  9	 P 	  1123.54	   701.39	  6
 10	 S 	  1210.57	   604.34	  5
 11	 I 	  1323.66	   517.31	  4
 12	 T 	  1424.71	   404.23	  3
 13	 Q 	  1552.76	   303.18	  2
 14	 R 	        -  	   175.12	  1  _
+2 charge
		      B   	       Y
  1	 V 	    50.54	        -  	 14
  2	 Y 	   132.07	   814.41	 13
  3	 Y 	   213.60	   732.88	 12
  4	 R 	   291.66	   651.34	 11
  5	 E 	   356.18	   573.29	 10
  6	 D 	   413.69	   508.77	  9
  7	 L 	   470.23	   451.26	  8
  8	 S 	   513.75	   394.72	  7
  9	 P 	   562.27	   351.20	  6
 10	 S 	   605.79	   302.67	  5
 11	 I 	   662.33	   259.16	  4
 12	 T 	   712.86	   202.62	  3
 13	 Q 	   776.89	   152.09	  2
 14	 R 	        -  	    88.06	  1  _
```

**53. IPI00433028 Isoform 1 of Leukocyte-associated immunoglobulin-like receptor 1**

| Sequence | Charge | M+H+ | XCorr | DeltaCn |
| --- | --- | --- | --- | --- |
| R.FRIDSVSEGNAGPYR.C | 2 | 1668.792 | 4.513 | 0.363 |

+1 charge

```
		      B   	       Y
  1	 F 	   148.08	        -  	 15
  2	 R 	   304.18	  1520.75	 14
  3	 I 	   417.26	  1364.64	 13
  4	 D 	   532.29	  1251.56	 12
  5	 S 	   619.32	  1136.53	 11
  6	 V 	   718.39	  1049.50	 10
  7	 S 	   805.42	   950.43	  9
  8	 E 	   934.46	   863.40	  8
  9	 G 	   991.48	   734.36	  7
 10	 N 	  1105.53	   677.34	  6
 11	 A 	  1176.56	   563.29	  5
 12	 G 	  1233.59	   492.26	  4
 13	 P 	  1330.64	   435.24	  3
 14	 Y 	  1493.70	   338.18	  2
 15	 R 	        -  	   175.12	  1  _
+2 charge
		      B   	       Y
  1	 F 	    74.54	        -  	 15
  2	 R 	   152.59	   760.88	 14
  3	 I 	   209.13	   682.83	 13
  4	 D 	   266.65	   626.28	 12
  5	 S 	   310.16	   568.77	 11
  6	 V 	   359.70	   525.25	 10
  7	 S 	   403.21	   475.72	  9
  8	 E 	   467.74	   432.20	  8
  9	 G 	   496.25	   367.68	  7
 10	 N 	   553.27	   339.17	  6
 11	 A 	   588.79	   282.15	  5
 12	 G 	   617.30	   246.63	  4
 13	 P 	   665.82	   218.12	  3
 14	 Y 	   747.35	   169.59	  2
 15	 R 	        -  	    88.06	  1  _
```

**54. IPI00896419 Isoform 1 of Inter-alpha-trypsin inhibitor heavy chain H4**

| Sequence | Charge | M+H+ | XCorr | DeltaCn |
| --- | --- | --- | --- | --- |
| R.QGPVNLLSDPEQGVEVTGQYEREK.A | 3 | 2673.874 | 4.003 | 0.531 |

+1 charge

```
		      B   	       Y
  1	 Q 	   129.07	        -  	 24
  2	 G 	   186.09	  2544.26	 23
  3	 P 	   283.14	  2487.24	 22
  4	 V 	   382.21	  2390.18	 21
  5	 N 	   496.25	  2291.11	 20
  6	 L 	   609.34	  2177.07	 19
  7	 L 	   722.42	  2063.99	 18
  8	 S 	   809.45	  1950.90	 17
  9	 D 	   924.48	  1863.87	 16
 10	 P 	  1021.53	  1748.84	 15
 11	 E 	  1150.57	  1651.79	 14
 12	 Q 	  1278.63	  1522.75	 13
 13	 G 	  1335.65	  1394.69	 12
 14	 V 	  1434.72	  1337.67	 11
 15	 E 	  1563.76	  1238.60	 10
 16	 V 	  1662.83	  1109.56	  9
 17	 T 	  1763.88	  1010.49	  8
 18	 G 	  1820.90	   909.44	  7
 19	 Q 	  1948.96	   852.42	  6
 20	 Y 	  2112.02	   724.36	  5
 21	 E 	  2241.07	   561.30	  4
 22	 R 	  2397.17	   432.26	  3
 23	 E 	  2526.21	   276.16	  2
 24	 K 	        -  	   147.11	  1  _


+2 charge
		      B   	       Y
  1	 Q 	    65.04	        -  	 24
  2	 G 	    93.55	  1272.63	 23
  3	 P 	   142.07	  1244.12	 22
  4	 V 	   191.61	  1195.60	 21
  5	 N 	   248.63	  1146.06	 20
  6	 L 	   305.17	  1089.04	 19
  7	 L 	   361.71	  1032.50	 18
  8	 S 	   405.23	   975.96	 17
  9	 D 	   462.74	   932.44	 16
 10	 P 	   511.27	   874.93	 15
 11	 E 	   575.79	   826.40	 14
 12	 Q 	   639.82	   761.88	 13
 13	 G 	   668.33	   697.85	 12
 14	 V 	   717.86	   669.34	 11
 15	 E 	   782.39	   619.80	 10
 16	 V 	   831.92	   555.28	  9
 17	 T 	   882.44	   505.75	  8
 18	 G 	   910.95	   455.22	  7
 19	 Q 	   974.98	   426.71	  6
 20	 Y 	  1056.52	   362.68	  5
 21	 E 	  1121.04	   281.15	  4
 22	 R 	  1199.09	   216.63	  3
 23	 E 	  1263.61	   138.58	  2
 24	 K 	        -  	    74.06	  1  _


+3 charge
		      B   	       Y
  1	 Q 	    43.69	        -  	 24
  2	 G 	    62.70	   848.76	 23
  3	 P 	    95.05	   829.75	 22
  4	 V 	   128.07	   797.40	 21
  5	 N 	   166.09	   764.38	 20
  6	 L 	   203.78	   726.36	 19
  7	 L 	   241.48	   688.67	 18
  8	 S 	   270.49	   650.97	 17
  9	 D 	   308.83	   621.96	 16
 10	 P 	   341.18	   583.62	 15
 11	 E 	   384.20	   551.27	 14
 12	 Q 	   426.88	   508.25	 13
 13	 G 	   445.89	   465.57	 12
 14	 V 	   478.91	   446.56	 11
 15	 E 	   521.93	   413.54	 10
 16	 V 	   554.95	   370.52	  9
 17	 T 	   588.63	   337.50	  8
 18	 G 	   607.64	   303.82	  7
 19	 Q 	   650.33	   284.81	  6
 20	 Y 	   704.68	   242.13	  5
 21	 E 	   747.69	   187.77	  4
 22	 R 	   799.73	   144.76	  3
 23	 E 	   842.74	    92.72	  2
 24	 K 	        -  	    49.71	  1  _
```

**55. IPI00025861 Cadherin-1**

| Sequence | Charge | M+H+ | XCorr | DeltaCn |
| --- | --- | --- | --- | --- |
| K.MALEVGDYKINLK.L | 2 | 1494.781 | 3.872 | 0.473 |

+1 charge

```
		      B   	       Y
  1	 M 	   132.05	        -  	 13
  2	 A 	   203.08	  1362.76	 12
  3	 L 	   316.17	  1291.73	 11
  4	 E 	   445.21	  1178.64	 10
  5	 V 	   544.28	  1049.60	  9
  6	 G 	   601.30	   950.53	  8
  7	 D 	   716.33	   893.51	  7
  8	 Y 	   879.39	   778.48	  6
  9	 K 	  1007.49	   615.42	  5
 10	 I 	  1120.57	   487.32	  4
 11	 N 	  1234.61	   374.24	  3
 12	 L 	  1347.70	   260.20	  2
 13	 K 	        -  	   147.11	  1  _
+2 charge
		      B   	       Y
  1	 M 	    66.53	        -  	 13
  2	 A 	   102.05	   681.89	 12
  3	 L 	   158.59	   646.37	 11
  4	 E 	   223.11	   589.82	 10
  5	 V 	   272.64	   525.30	  9
  6	 G 	   301.15	   475.77	  8
  7	 D 	   358.67	   447.26	  7
  8	 Y 	   440.20	   389.74	  6
  9	 K 	   504.25	   308.21	  5
 10	 I 	   560.79	   244.17	  4
 11	 N 	   617.81	   187.62	  3
 12	 L 	   674.35	   130.60	  2
 13	 K 	        -  	    74.06	  1  _
```

**56. IPI00166392 Similar to Cell adhesion molecule 1 precursor**

| Sequence | Charge | M+H+ | XCorr | DeltaCn |
| --- | --- | --- | --- | --- |
| R.YLEVQYKPQVHIQMTYPLQGLTR.E | 3 | 2807.262 | 4.52 | 0.545 |

+1 charge

```
		      B   	       Y
  1	 Y 	   164.07	        -  	 23
  2	 L 	   277.15	  2642.41	 22
  3	 E 	   406.20	  2529.33	 21
  4	 V 	   505.27	  2400.29	 20
  5	 Q 	   633.32	  2301.22	 19
  6	 Y 	   796.39	  2173.16	 18
  7	 K 	   924.48	  2010.10	 17
  8	 P 	  1021.54	  1882.00	 16
  9	 Q 	  1149.59	  1784.95	 15
 10	 V 	  1248.66	  1656.89	 14
 11	 H 	  1385.72	  1557.82	 13
 12	 I 	  1498.81	  1420.76	 12
 13	 Q 	  1626.86	  1307.68	 11
 14	 M 	  1757.90	  1179.62	 10
 15	 T 	  1858.95	  1048.58	  9
 16	 Y 	  2022.02	   947.53	  8
 17	 P 	  2119.07	   784.47	  7
 18	 L 	  2232.15	   687.41	  6
 19	 Q 	  2360.21	   574.33	  5
 20	 G 	  2417.23	   446.27	  4
 21	 L 	  2530.32	   389.25	  3
 22	 T 	  2631.36	   276.17	  2
 23	 R 	        -  	   175.12	  1  _


+2 charge
		      B   	       Y
  1	 Y 	    82.54	        -  	 23
  2	 L 	   139.08	  1321.71	 22
  3	 E 	   203.60	  1265.17	 21
  4	 V 	   253.14	  1200.65	 20
  5	 Q 	   317.17	  1151.11	 19
  6	 Y 	   398.70	  1087.08	 18
  7	 K 	   462.74	  1005.55	 17
  8	 P 	   511.27	   941.50	 16
  9	 Q 	   575.30	   892.98	 15
 10	 V 	   624.83	   828.95	 14
 11	 H 	   693.36	   779.41	 13
 12	 I 	   749.91	   710.88	 12
 13	 Q 	   813.94	   654.34	 11
 14	 M 	   879.46	   590.31	 10
 15	 T 	   929.98	   524.79	  9
 16	 Y 	  1011.51	   474.27	  8
 17	 P 	  1060.04	   392.74	  7
 18	 L 	  1116.58	   344.21	  6
 19	 Q 	  1180.61	   287.67	  5
 20	 G 	  1209.12	   223.64	  4
 21	 L 	  1265.66	   195.13	  3
 22	 T 	  1316.19	   138.59	  2
 23	 R 	        -  	    88.06	  1  _


+3 charge
		      B   	       Y
  1	 Y 	    55.36	        -  	 23
  2	 L 	    93.06	   881.48	 22
  3	 E 	   136.07	   843.78	 21
  4	 V 	   169.09	   800.77	 20
  5	 Q 	   211.78	   767.74	 19
  6	 Y 	   266.13	   725.06	 18
  7	 K 	   308.83	   670.70	 17
  8	 P 	   341.18	   628.00	 16
  9	 Q 	   383.87	   595.65	 15
 10	 V 	   416.89	   552.97	 14
 11	 H 	   462.58	   519.95	 13
 12	 I 	   500.27	   474.26	 12
 13	 Q 	   542.96	   436.56	 11
 14	 M 	   586.64	   393.88	 10
 15	 T 	   620.32	   350.20	  9
 16	 Y 	   674.68	   316.52	  8
 17	 P 	   707.03	   262.16	  7
 18	 L 	   744.72	   229.81	  6
 19	 Q 	   787.41	   192.12	  5
 20	 G 	   806.42	   149.43	  4
 21	 L 	   844.11	   130.42	  3
 22	 T 	   877.79	    92.73	  2
 23	 R 	        -  	    59.04	  1  _
```

**57. IPI00303161 Endothelial cell-selective adhesion molecule**

| Sequence | Charge | M+H+ | XCorr | DeltaCn |
| --- | --- | --- | --- | --- |
| R.QLPSFQTFFAPALDVIR.G | 2 | 1951.257 | 4.041 | 0.595 |

+1 charge

```
		      B   	       Y
  1	 Q 	   129.07	        -  	 17
  2	 L 	   242.15	  1821.99	 16
  3	 P 	   339.20	  1708.91	 15
  4	 S 	   426.23	  1611.85	 14
  5	 F 	   573.30	  1524.82	 13
  6	 Q 	   701.36	  1377.75	 12
  7	 T 	   802.41	  1249.69	 11
  8	 F 	   949.48	  1148.65	 10
  9	 F 	  1096.55	  1001.58	  9
 10	 A 	  1167.58	   854.51	  8
 11	 P 	  1264.64	   783.47	  7
 12	 A 	  1335.67	   686.42	  6
 13	 L 	  1448.76	   615.38	  5
 14	 D 	  1563.78	   502.30	  4
 15	 V 	  1662.85	   387.27	  3
 16	 I 	  1775.94	   288.20	  2
 17	 R 	        -  	   175.12	  1  _
+2 charge
		      B   	       Y
  1	 Q 	    65.04	        -  	 17
  2	 L 	   121.58	   911.50	 16
  3	 P 	   170.10	   854.96	 15
  4	 S 	   213.62	   806.43	 14
  5	 F 	   287.16	   762.91	 13
  6	 Q 	   351.18	   689.38	 12
  7	 T 	   401.71	   625.35	 11
  8	 F 	   475.24	   574.83	 10
  9	 F 	   548.78	   501.29	  9
 10	 A 	   584.30	   427.76	  8
 11	 P 	   632.82	   392.24	  7
 12	 A 	   668.34	   343.71	  6
 13	 L 	   724.88	   308.19	  5
 14	 D 	   782.40	   251.65	  4
 15	 V 	   831.93	   194.14	  3
 16	 I 	   888.47	   144.61	  2
 17	 R 	        -  	    88.06	  1  _
```

**58. IPI00555812 Isoform 1 of Vitamin D-binding protein**

| Sequence | Charge | M+H+ | XCorr | DeltaCn |
| --- | --- | --- | --- | --- |
| K.LAQKVPTADLEDVLPLAEDITNILSK.C | 3 | 2808.217 | 5.124 | 0.513 |

+1 charge

```
		      B   	       Y
  1	 L 	   114.09	        -  	 26
  2	 A 	   185.13	  2693.46	 25
  3	 Q 	   313.19	  2622.42	 24
  4	 K 	   441.28	  2494.37	 23
  5	 V 	   540.35	  2366.27	 22
  6	 P 	   637.40	  2267.20	 21
  7	 T 	   738.45	  2170.15	 20
  8	 A 	   809.49	  2069.10	 19
  9	 D 	   924.51	  1998.06	 18
 10	 L 	  1037.60	  1883.04	 17
 11	 E 	  1166.64	  1769.95	 16
 12	 D 	  1281.67	  1640.91	 15
 13	 V 	  1380.74	  1525.88	 14
 14	 L 	  1493.82	  1426.82	 13
 15	 P 	  1590.87	  1313.73	 12
 16	 L 	  1703.96	  1216.68	 11
 17	 A 	  1774.99	  1103.59	 10
 18	 E 	  1904.04	  1032.56	  9
 19	 D 	  2019.06	   903.51	  8
 20	 I 	  2132.15	   788.49	  7
 21	 T 	  2233.20	   675.40	  6
 22	 N 	  2347.24	   574.36	  5
 23	 I 	  2460.32	   460.31	  4
 24	 L 	  2573.41	   347.23	  3
 25	 S 	  2660.44	   234.14	  2
 26	 K 	        -  	   147.11	  1  _


+2 charge
		      B   	       Y
  1	 L 	    57.55	        -  	 26
  2	 A 	    93.07	  1347.23	 25
  3	 Q 	   157.10	  1311.72	 24
  4	 K 	   221.14	  1247.69	 23
  5	 V 	   270.68	  1183.64	 22
  6	 P 	   319.21	  1134.10	 21
  7	 T 	   369.73	  1085.58	 20
  8	 A 	   405.25	  1035.05	 19
  9	 D 	   462.76	   999.54	 18
 10	 L 	   519.30	   942.02	 17
 11	 E 	   583.82	   885.48	 16
 12	 D 	   641.34	   820.96	 15
 13	 V 	   690.87	   763.45	 14
 14	 L 	   747.41	   713.91	 13
 15	 P 	   795.94	   657.37	 12
 16	 L 	   852.48	   608.84	 11
 17	 A 	   888.00	   552.30	 10
 18	 E 	   952.52	   516.78	  9
 19	 D 	  1010.04	   452.26	  8
 20	 I 	  1066.58	   394.75	  7
 21	 T 	  1117.10	   338.21	  6
 22	 N 	  1174.12	   287.68	  5
 23	 I 	  1230.67	   230.66	  4
 24	 L 	  1287.21	   174.12	  3
 25	 S 	  1330.72	   117.58	  2
 26	 K 	        -  	    74.06	  1  _


+3 charge
		      B   	       Y
  1	 L 	    38.70	        -  	 26
  2	 A 	    62.38	   898.49	 25
  3	 Q 	   105.07	   874.81	 24
  4	 K 	   147.77	   832.13	 23
  5	 V 	   180.79	   789.43	 22
  6	 P 	   213.14	   756.41	 21
  7	 T 	   246.82	   724.05	 20
  8	 A 	   270.50	   690.37	 19
  9	 D 	   308.84	   666.69	 18
 10	 L 	   346.54	   628.35	 17
 11	 E 	   389.55	   590.66	 16
 12	 D 	   427.89	   547.64	 15
 13	 V 	   460.92	   509.30	 14
 14	 L 	   498.61	   476.28	 13
 15	 P 	   530.96	   438.58	 12
 16	 L 	   568.66	   406.23	 11
 17	 A 	   592.34	   368.54	 10
 18	 E 	   635.35	   344.86	  9
 19	 D 	   673.69	   301.84	  8
 20	 I 	   711.39	   263.50	  7
 21	 T 	   745.07	   225.81	  6
 22	 N 	   783.08	   192.12	  5
 23	 I 	   820.78	   154.11	  4
 24	 L 	   858.47	   116.41	  3
 25	 S 	   887.48	    78.72	  2
 26	 K 	        -  	    49.71	  1  _
```

**59. IPI00441498 Folate receptor alpha**

| Sequence | Charge | M+H+ | XCorr | DeltaCn |
| --- | --- | --- | --- | --- |
| R.FNWNHCGEMAPACKR.H | 3 | 1879.079 | 4.157 | 0.435 |

+1 charge

```
		      B   	       Y
  1	 F 	   148.08	        -  	 15
  2	 N 	   262.12	  1731.00	 14
  3	 W 	   448.20	  1616.96	 13
  4	 N 	   562.24	  1430.88	 12
  5	 H 	   699.30	  1316.83	 11
  6	 C 	   859.47	  1179.78	 10
  7	 G 	   916.49	  1019.61	  9
  8	 E 	  1045.53	   962.59	  8
  9	 M 	  1176.57	   833.55	  7
 10	 A 	  1247.61	   702.51	  6
 11	 P 	  1344.66	   631.47	  5
 12	 A 	  1415.70	   534.42	  4
 13	 C 	  1575.86	   463.38	  3
 14	 K 	  1703.96	   303.21	  2
 15	 R 	        -  	   175.12	  1  _
+2 charge
		      B   	       Y
  1	 F 	    74.54	        -  	 15
  2	 N 	   131.56	   866.00	 14
  3	 W 	   224.60	   808.98	 13
  4	 N 	   281.62	   715.94	 12
  5	 H 	   350.15	   658.92	 11
  6	 C 	   430.24	   590.39	 10
  7	 G 	   458.75	   510.31	  9
  8	 E 	   523.27	   481.80	  8
  9	 M 	   588.79	   417.28	  7
 10	 A 	   624.31	   351.76	  6
 11	 P 	   672.83	   316.24	  5
 12	 A 	   708.35	   267.71	  4
 13	 C 	   788.43	   232.19	  3
 14	 K 	   852.48	   152.11	  2
 15	 R 	        -  	    88.06	  1  _


+3 charge
		      B   	       Y
  1	 F 	    50.03	        -  	 15
  2	 N 	    88.04	   577.67	 14
  3	 W 	   150.07	   539.66	 13
  4	 N 	   188.09	   477.63	 12
  5	 H 	   233.77	   439.62	 11
  6	 C 	   287.16	   393.93	 10
  7	 G 	   306.17	   340.54	  9
  8	 E 	   349.18	   321.53	  8
  9	 M 	   392.86	   278.52	  7
 10	 A 	   416.54	   234.84	  6
 11	 P 	   448.89	   211.16	  5
 12	 A 	   472.57	   178.81	  4
 13	 C 	   525.96	   155.13	  3
 14	 K 	   568.66	   101.74	  2
 15	 R 	        -  	    59.04	  1  _
```

**60. IPI00028347 Isoform 1 of F-box only protein 31**

| Sequence | Charge | M+H+ | XCorr | DeltaCn |
| --- | --- | --- | --- | --- |
| R.IYLPPSRPDDLIKPGLFK.G | 2 | 2070.462 | 3.131 | 0.331 |

+1 charge

```
		      B   	       Y
  1	 I 	   114.09	        -  	 18
  2	 Y 	   277.15	  1956.10	 17
  3	 L 	   390.24	  1793.03	 16
  4	 P 	   487.29	  1679.95	 15
  5	 P 	   584.34	  1582.90	 14
  6	 S 	   671.38	  1485.84	 13
  7	 R 	   827.48	  1398.81	 12
  8	 P 	   924.53	  1242.71	 11
  9	 D 	  1039.56	  1145.66	 10
 10	 D 	  1154.58	  1030.63	  9
 11	 L 	  1267.67	   915.60	  8
 12	 I 	  1380.75	   802.52	  7
 13	 K 	  1508.85	   689.43	  6
 14	 P 	  1605.90	   561.34	  5
 15	 G 	  1662.92	   464.29	  4
 16	 L 	  1776.01	   407.27	  3
 17	 F 	  1923.07	   294.18	  2
 18	 K 	        -  	   147.11	  1  _
+2 charge
		      B   	       Y
  1	 I 	    57.55	        -  	 18
  2	 Y 	   139.08	   978.55	 17
  3	 L 	   195.62	   897.02	 16
  4	 P 	   244.15	   840.48	 15
  5	 P 	   292.68	   791.95	 14
  6	 S 	   336.19	   743.42	 13
  7	 R 	   414.24	   699.91	 12
  8	 P 	   462.77	   621.86	 11
  9	 D 	   520.28	   573.33	 10
 10	 D 	   577.80	   515.82	  9
 11	 L 	   634.34	   458.30	  8
 12	 I 	   690.88	   401.76	  7
 13	 K 	   754.93	   345.22	  6
 14	 P 	   803.45	   281.17	  5
 15	 G 	   831.96	   232.65	  4
 16	 L 	   888.51	   204.14	  3
 17	 F 	   962.04	   147.59	  2
 18	 K 	        -  	    74.06	  1  _
```

**61. IPI00385985 Ig lambda chain V-III region LOI**

| Sequence | Charge | M+H+ | XCorr | DeltaCn |
| --- | --- | --- | --- | --- |
| - .YVLTQPPSVSVAPGETAR.L | 2 | 1873.099 | 3.862 | 0.532 |

+1 charge

```
		      B   	       Y
  1	 Y 	   164.07	        -  	 18
  2	 V 	   263.14	  1708.92	 17
  3	 L 	   376.22	  1609.85	 16
  4	 T 	   477.27	  1496.77	 15
  5	 Q 	   605.33	  1395.72	 14
  6	 P 	   702.38	  1267.66	 13
  7	 P 	   799.43	  1170.61	 12
  8	 S 	   886.47	  1073.56	 11
  9	 V 	   985.54	   986.53	 10
 10	 S 	  1072.57	   887.46	  9
 11	 V 	  1171.64	   800.43	  8
 12	 A 	  1242.67	   701.36	  7
 13	 P 	  1339.73	   630.32	  6
 14	 G 	  1396.75	   533.27	  5
 15	 E 	  1525.79	   476.25	  4
 16	 T 	  1626.84	   347.20	  3
 17	 A 	  1697.87	   246.16	  2
 18	 R 	        -  	   175.12	  1  _
+2 charge
		      B   	       Y
  1	 Y 	    82.54	        -  	 18
  2	 V 	   132.07	   854.97	 17
  3	 L 	   188.62	   805.43	 16
  4	 T 	   239.14	   748.89	 15
  5	 Q 	   303.17	   698.36	 14
  6	 P 	   351.69	   634.34	 13
  7	 P 	   400.22	   585.81	 12
  8	 S 	   443.74	   537.28	 11
  9	 V 	   493.27	   493.77	 10
 10	 S 	   536.79	   444.23	  9
 11	 V 	   586.32	   400.72	  8
 12	 A 	   621.84	   351.18	  7
 13	 P 	   670.37	   315.66	  6
 14	 G 	   698.88	   267.14	  5
 15	 E 	   763.40	   238.63	  4
 16	 T 	   813.92	   174.11	  3
 17	 A 	   849.44	   123.58	  2
 18	 R 	        -  	    88.06	  1  _
```

**62. IPI00641737 Haptoglobin**

| Sequence | Charge | M+H+ | XCorr | DeltaCn |
| --- | --- | --- | --- | --- |
| K.YVMLPVADQDQCIR.H | 2 | 1708.954 | 3.076 | 0.272 |

+1 charge

```
		      B   	       Y
  1	 Y 	   164.07	        -  	 14
  2	 V 	   263.14	  1544.89	 13
  3	 M 	   394.18	  1445.82	 12
  4	 L 	   507.26	  1314.78	 11
  5	 P 	   604.32	  1201.70	 10
  6	 V 	   703.38	  1104.64	  9
  7	 A 	   774.42	  1005.58	  8
  8	 D 	   889.45	   934.54	  7
  9	 Q 	  1017.51	   819.51	  6
 10	 D 	  1132.53	   691.45	  5
 11	 Q 	  1260.59	   576.43	  4
 12	 C 	  1420.76	   448.37	  3
 13	 I 	  1533.84	   288.20	  2
 14	 R 	        -  	   175.12	  1  _
+2 charge
		      B   	       Y
  1	 Y 	    82.54	        -  	 14
  2	 V 	   132.07	   772.95	 13
  3	 M 	   197.59	   723.41	 12
  4	 L 	   254.14	   657.89	 11
  5	 P 	   302.66	   601.35	 10
  6	 V 	   352.20	   552.83	  9
  7	 A 	   387.71	   503.29	  8
  8	 D 	   445.23	   467.77	  7
  9	 Q 	   509.26	   410.26	  6
 10	 D 	   566.77	   346.23	  5
 11	 Q 	   630.80	   288.72	  4
 12	 C 	   710.88	   224.69	  3
 13	 I 	   767.42	   144.61	  2
 14	 R 	        -  	    88.06	  1  _
```

**63. IPI00385252 Ig kappa chain V-III region GOL**

| Sequence | Charge | M+H+ | XCorr | DeltaCn |
| --- | --- | --- | --- | --- |
| - .EIVLTQSPGTLSLSPGER.A | 2 | 1885.108 | 4.821 | 0.516 |

+1 charge

```
		      B   	       Y
  1	 E 	   130.05	        -  	 18
  2	 I 	   243.13	  1754.96	 17
  3	 V 	   342.20	  1641.88	 16
  4	 L 	   455.29	  1542.81	 15
  5	 T 	   556.33	  1429.73	 14
  6	 Q 	   684.39	  1328.68	 13
  7	 S 	   771.42	  1200.62	 12
  8	 P 	   868.48	  1113.59	 11
  9	 G 	   925.50	  1016.54	 10
 10	 T 	  1026.55	   959.52	  9
 11	 L 	  1139.63	   858.47	  8
 12	 S 	  1226.66	   745.38	  7
 13	 L 	  1339.75	   658.35	  6
 14	 S 	  1426.78	   545.27	  5
 15	 P 	  1523.83	   458.24	  4
 16	 G 	  1580.85	   361.18	  3
 17	 E 	  1709.90	   304.16	  2
 18	 R 	        -  	   175.12	  1  _
+2 charge
		      B   	       Y
  1	 E 	    65.53	        -  	 18
  2	 I 	   122.07	   877.99	 17
  3	 V 	   171.60	   821.44	 16
  4	 L 	   228.15	   771.91	 15
  5	 T 	   278.67	   715.37	 14
  6	 Q 	   342.70	   664.84	 13
  7	 S 	   386.22	   600.81	 12
  8	 P 	   434.74	   557.30	 11
  9	 G 	   463.25	   508.77	 10
 10	 T 	   513.78	   480.26	  9
 11	 L 	   570.32	   429.74	  8
 12	 S 	   613.83	   373.20	  7
 13	 L 	   670.38	   329.68	  6
 14	 S 	   713.89	   273.14	  5
 15	 P 	   762.42	   229.62	  4
 16	 G 	   790.93	   181.10	  3
 17	 E 	   855.45	   152.58	  2
 18	 R 	        -  	    88.06	  1  _
```

**64. IPI00022488 Hemopexin**

| Sequence | Charge | M+H+ | XCorr | DeltaCn |
| --- | --- | --- | --- | --- |
| R.YYCFQGNQFLR.F | 2 | 1496.644 | 3.25 | 0.477 |

+1 charge

```
		      B   	       Y
  1	 Y 	   164.07	        -  	 11
  2	 Y 	   327.13	  1332.75	 10
  3	 C 	   487.30	  1169.69	  9
  4	 F 	   634.37	  1009.52	  8
  5	 Q 	   762.43	   862.45	  7
  6	 G 	   819.45	   734.39	  6
  7	 N 	   933.49	   677.37	  5
  8	 Q 	  1061.55	   563.33	  4
  9	 F 	  1208.62	   435.27	  3
 10	 L 	  1321.70	   288.20	  2
 11	 R 	        -  	   175.12	  1  _
+2 charge
		      B   	       Y
  1	 Y 	    82.54	        -  	 11
  2	 Y 	   164.07	   666.88	 10
  3	 C 	   244.15	   585.35	  9
  4	 F 	   317.69	   505.26	  8
  5	 Q 	   381.72	   431.73	  7
  6	 G 	   410.23	   367.70	  6
  7	 N 	   467.25	   339.19	  5
  8	 Q 	   531.28	   282.17	  4
  9	 F 	   604.81	   218.14	  3
 10	 L 	   661.35	   144.61	  2
 11	 R 	        -  	    88.06	  1  _
```

**65. IPI00099239 Isoform 1 of SLAM family member 5**

| Sequence | Charge | M+H+ | XCorr | DeltaCn |
| --- | --- | --- | --- | --- |
| R.IHALGPNYNLVISDLR.M | 2 | 1796.063 | 3.047 | 0.531 |

+1 charge

```
		      B   	       Y
  1	 I 	   114.09	        -  	 16
  2	 H 	   251.15	  1681.90	 15
  3	 A 	   322.19	  1544.84	 14
  4	 L 	   435.27	  1473.81	 13
  5	 G 	   492.29	  1360.72	 12
  6	 P 	   589.35	  1303.70	 11
  7	 N 	   703.39	  1206.65	 10
  8	 Y 	   866.45	  1092.60	  9
  9	 N 	   980.49	   929.54	  8
 10	 L 	  1093.58	   815.50	  7
 11	 V 	  1192.65	   702.41	  6
 12	 I 	  1305.73	   603.35	  5
 13	 S 	  1392.76	   490.26	  4
 14	 D 	  1507.79	   403.23	  3
 15	 L 	  1620.87	   288.20	  2
 16	 R 	        -  	   175.12	  1  _
+2 charge
		      B   	       Y
  1	 I 	    57.55	        -  	 16
  2	 H 	   126.08	   841.45	 15
  3	 A 	   161.60	   772.93	 14
  4	 L 	   218.14	   737.41	 13
  5	 G 	   246.65	   680.86	 12
  6	 P 	   295.18	   652.35	 11
  7	 N 	   352.20	   603.83	 10
  8	 Y 	   433.73	   546.81	  9
  9	 N 	   490.75	   465.27	  8
 10	 L 	   547.29	   408.25	  7
 11	 V 	   596.83	   351.71	  6
 12	 I 	   653.37	   302.18	  5
 13	 S 	   696.89	   245.63	  4
 14	 D 	   754.40	   202.12	  3
 15	 L 	   810.94	   144.61	  2
 16	 R 	        -  	    88.06	  1  _
```

**66. IPI00409698 Isoform 1 of Transportin-2**

| Sequence | Charge | M+H+ | XCorr | DeltaCn |
| --- | --- | --- | --- | --- |
| K.QECLNNIGDASSLIR.A | 2 | 1690.831 | 3.168 | 0.278 |

+1 charge

```
		      B   	       Y
  1	 Q 	   129.07	        -  	 15
  2	 E 	   258.11	  1561.90	 14
  3	 C 	   418.27	  1432.86	 13
  4	 L 	   531.36	  1272.69	 12
  5	 N 	   645.40	  1159.61	 11
  6	 N 	   759.44	  1045.56	 10
  7	 I 	   872.53	   931.52	  9
  8	 G 	   929.55	   818.44	  8
  9	 D 	  1044.58	   761.42	  7
 10	 A 	  1115.61	   646.39	  6
 11	 S 	  1202.65	   575.35	  5
 12	 S 	  1289.68	   488.32	  4
 13	 L 	  1402.76	   401.29	  3
 14	 I 	  1515.85	   288.20	  2
 15	 R 	        -  	   175.12	  1  _
+2 charge
		      B   	       Y
  1	 Q 	    65.04	        -  	 15
  2	 E 	   129.56	   781.45	 14
  3	 C 	   209.64	   716.93	 13
  4	 L 	   266.18	   636.85	 12
  5	 N 	   323.20	   580.31	 11
  6	 N 	   380.23	   523.29	 10
  7	 I 	   436.77	   466.26	  9
  8	 G 	   465.28	   409.72	  8
  9	 D 	   522.79	   381.21	  7
 10	 A 	   558.31	   323.70	  6
 11	 S 	   601.83	   288.18	  5
 12	 S 	   645.34	   244.66	  4
 13	 L 	   701.88	   201.15	  3
 14	 I 	   758.43	   144.61	  2
 15	 R 	        -  	    88.06	  1  _
```

**67. IPI00784430 Similar to Ig kappa chain V-III region VG precursor**

| Sequence | Charge | M+H+ | XCorr | DeltaCn |
| --- | --- | --- | --- | --- |
| R.LLIYDASNR.A | 2 | 1065.205 | 3.206 | 0.482 |

+1 charge

```
		      B   	       Y
  1	 L 	   114.09	        -  	  9
  2	 L 	   227.18	   951.49	  8
  3	 I 	   340.26	   838.41	  7
  4	 Y 	   503.32	   725.32	  6
  5	 D 	   618.35	   562.26	  5
  6	 A 	   689.39	   447.23	  4
  7	 S 	   776.42	   376.19	  3
  8	 N 	   890.46	   289.16	  2
  9	 R 	        -  	   175.12	  1  _
+2 charge
		      B   	       Y
  1	 L 	    57.55	        -  	  9
  2	 L 	   114.09	   476.25	  8
  3	 I 	   170.63	   419.71	  7
  4	 Y 	   252.17	   363.16	  6
  5	 D 	   309.68	   281.63	  5
  6	 A 	   345.20	   224.12	  4
  7	 S 	   388.71	   188.60	  3
  8	 N 	   445.73	   145.08	  2
  9	 R 	        -  	    88.06	  1  _
```

**68. IPI00382452 Isoform 1 of Charged multivesicular body protein 1a**

| Sequence | Charge | M+H+ | XCorr | DeltaCn |
| --- | --- | --- | --- | --- |
| K.ALDKALSTMDLQK.V | 2 | 1434.684 | 2.946 | 0.376 |

+1 charge

```
		      B   	       Y
  1	 A 	    72.04	        -  	 13
  2	 L 	   185.13	  1362.73	 12
  3	 D 	   300.16	  1249.65	 11
  4	 K 	   428.25	  1134.62	 10
  5	 A 	   499.29	  1006.52	  9
  6	 L 	   612.37	   935.49	  8
  7	 S 	   699.40	   822.40	  7
  8	 T 	   800.45	   735.37	  6
  9	 M 	   931.49	   634.32	  5
 10	 D 	  1046.52	   503.28	  4
 11	 L 	  1159.60	   388.26	  3
 12	 Q 	  1287.66	   275.17	  2
 13	 K 	        -  	   147.11	  1  _
+2 charge
		      B   	       Y
  1	 A 	    36.53	        -  	 13
  2	 L 	    93.07	   681.87	 12
  3	 D 	   150.58	   625.33	 11
  4	 K 	   214.63	   567.81	 10
  5	 A 	   250.15	   503.77	  9
  6	 L 	   306.69	   468.25	  8
  7	 S 	   350.21	   411.70	  7
  8	 T 	   400.73	   368.19	  6
  9	 M 	   466.25	   317.67	  5
 10	 D 	   523.76	   252.14	  4
 11	 L 	   580.31	   194.63	  3
 12	 Q 	   644.33	   138.09	  2
 13	 K 	        -  	    74.06	  1  _
```

**69. IPI00304273 Apolipoprotein A-IV**

| Sequence | Charge | M+H+ | XCorr | DeltaCn |
| --- | --- | --- | --- | --- |
| K.SLAELGGHLDQQVEEFRR.R | 3 | 2085.266 | 3.521 | 0.286 |

+1 charge

```
		      B   	       Y
  1	 S 	    88.04	        -  	 18
  2	 L 	   201.12	  1997.02	 17
  3	 A 	   272.16	  1883.94	 16
  4	 E 	   401.20	  1812.90	 15
  5	 L 	   514.29	  1683.86	 14
  6	 G 	   571.31	  1570.77	 13
  7	 G 	   628.33	  1513.75	 12
  8	 H 	   765.39	  1456.73	 11
  9	 L 	   878.47	  1319.67	 10
 10	 D 	   993.50	  1206.59	  9
 11	 Q 	  1121.56	  1091.56	  8
 12	 Q 	  1249.62	   963.50	  7
 13	 V 	  1348.69	   835.44	  6
 14	 E 	  1477.73	   736.37	  5
 15	 E 	  1606.77	   607.33	  4
 16	 F 	  1753.84	   478.29	  3
 17	 R 	  1909.94	   331.22	  2
 18	 R 	        -  	   175.12	  1  _
+2 charge
		      B   	       Y
  1	 S 	    44.52	        -  	 18
  2	 L 	   101.07	   999.01	 17
  3	 A 	   136.58	   942.47	 16
  4	 E 	   201.11	   906.95	 15
  5	 L 	   257.65	   842.43	 14
  6	 G 	   286.16	   785.89	 13
  7	 G 	   314.67	   757.38	 12
  8	 H 	   383.20	   728.87	 11
  9	 L 	   439.74	   660.34	 10
 10	 D 	   497.25	   603.80	  9
 11	 Q 	   561.28	   546.28	  8
 12	 Q 	   625.31	   482.25	  7
 13	 V 	   674.85	   418.22	  6
 14	 E 	   739.37	   368.69	  5
 15	 E 	   803.89	   304.17	  4
 16	 F 	   877.42	   239.65	  3
 17	 R 	   955.47	   166.11	  2
 18	 R 	        -  	    88.06	  1  _
+3 charge
		      B   	       Y
  1	 S 	    30.02	        -  	 18
  2	 L 	    67.71	   666.34	 17
  3	 A 	    91.39	   628.65	 16
  4	 E 	   134.41	   604.97	 15
  5	 L 	   172.10	   561.96	 14
  6	 G 	   191.11	   524.26	 13
  7	 G 	   210.11	   505.26	 12
  8	 H 	   255.80	   486.25	 11
  9	 L 	   293.50	   440.56	 10
 10	 D 	   331.84	   402.87	  9
 11	 Q 	   374.52	   364.52	  8
 12	 Q 	   417.21	   321.84	  7
 13	 V 	   450.23	   279.15	  6
 14	 E 	   493.25	   246.13	  5
 15	 E 	   536.26	   203.12	  4
 16	 F 	   585.28	   160.10	  3
 17	 R 	   637.32	   111.08	  2
 18	 R 	        -  	    59.04	  1  _
```

**70. IPI00004416 Charged multivesicular body protein 2a**

| Sequence | Charge | M+H+ | XCorr | DeltaCn |
| --- | --- | --- | --- | --- |
| K.AEAAASALADADADLEERLK.N | 2 | 2031.167 | 4.453 | 0.451 |

+1 charge

```
		      B   	       Y
  1	 A 	    72.04	        -  	 20
  2	 E 	   201.09	  1958.97	 19
  3	 A 	   272.12	  1829.92	 18
  4	 A 	   343.16	  1758.89	 17
  5	 A 	   414.20	  1687.85	 16
  6	 S 	   501.23	  1616.81	 15
  7	 A 	   572.27	  1529.78	 14
  8	 L 	   685.35	  1458.74	 13
  9	 A 	   756.39	  1345.66	 12
 10	 D 	   871.42	  1274.62	 11
 11	 A 	   942.45	  1159.60	 10
 12	 D 	  1057.48	  1088.56	  9
 13	 A 	  1128.52	   973.53	  8
 14	 D 	  1243.54	   902.49	  7
 15	 L 	  1356.63	   787.47	  6
 16	 E 	  1485.67	   674.38	  5
 17	 E 	  1614.71	   545.34	  4
 18	 R 	  1770.81	   416.30	  3
 19	 L 	  1883.90	   260.20	  2
 20	 K 	        -  	   147.11	  1  _


+2 charge
		      B   	       Y
  1	 A 	    36.53	        -  	 20
  2	 E 	   101.05	   979.99	 19
  3	 A 	   136.57	   915.47	 18
  4	 A 	   172.08	   879.95	 17
  5	 A 	   207.60	   844.43	 16
  6	 S 	   251.12	   808.91	 15
  7	 A 	   286.64	   765.39	 14
  8	 L 	   343.18	   729.88	 13
  9	 A 	   378.70	   673.33	 12
 10	 D 	   436.21	   637.81	 11
 11	 A 	   471.73	   580.30	 10
 12	 D 	   529.24	   544.78	  9
 13	 A 	   564.76	   487.27	  8
 14	 D 	   622.28	   451.75	  7
 15	 L 	   678.82	   394.24	  6
 16	 E 	   743.34	   337.70	  5
 17	 E 	   807.86	   273.17	  4
 18	 R 	   885.91	   208.65	  3
 19	 L 	   942.45	   130.60	  2
 20	 K 	        -  	    74.06	  1  _
```

**71. IPI00019641 Pepsin A**

| Sequence | Charge | M+H+ | XCorr | DeltaCn |
| --- | --- | --- | --- | --- |
| R.QYFTVFDRANNQVGLAPVA.- | 2 | 2111.345 | 3.843 | 0.583 |

+1 charge

```
		      B   	       Y
  1	 Q 	   129.07	        -  	 19
  2	 Y 	   292.13	  1982.01	 18
  3	 F 	   439.20	  1818.95	 17
  4	 T 	   540.25	  1671.88	 16
  5	 V 	   639.31	  1570.83	 15
  6	 F 	   786.38	  1471.77	 14
  7	 D 	   901.41	  1324.70	 13
  8	 R 	  1057.51	  1209.67	 12
  9	 A 	  1128.55	  1053.57	 11
 10	 N 	  1242.59	   982.53	 10
 11	 N 	  1356.63	   868.49	  9
 12	 Q 	  1484.69	   754.45	  8
 13	 V 	  1583.76	   626.39	  7
 14	 G 	  1640.78	   527.32	  6
 15	 L 	  1753.87	   470.30	  5
 16	 A 	  1824.90	   357.21	  4
 17	 P 	  1921.96	   286.18	  3
 18	 V 	  2021.02	   189.12	  2
 19	 A 	        -  	    90.05	  1  _
+2 charge
		      B   	       Y
  1	 Q 	    65.04	        -  	 19
  2	 Y 	   146.57	   991.51	 18
  3	 F 	   220.10	   909.98	 17
  4	 T 	   270.63	   836.44	 16
  5	 V 	   320.16	   785.92	 15
  6	 F 	   393.69	   736.39	 14
  7	 D 	   451.21	   662.85	 13
  8	 R 	   529.26	   605.34	 12
  9	 A 	   564.78	   527.29	 11
 10	 N 	   621.80	   491.77	 10
 11	 N 	   678.82	   434.75	  9
 12	 Q 	   742.85	   377.73	  8
 13	 V 	   792.38	   313.70	  7
 14	 G 	   820.89	   264.16	  6
 15	 L 	   877.44	   235.65	  5
 16	 A 	   912.96	   179.11	  4
 17	 P 	   961.48	   143.59	  3
 18	 V 	  1011.02	    95.07	  2
 19	 A 	        -  	    45.53	  1  _
```

**72. IPI00216007 Isoform 2 of Collagen alpha-5(IV) chain**

| Sequence | Charge | M+H+ | XCorr | DeltaCn |
| --- | --- | --- | --- | --- |
| K.GDTGPPGPPGLVIPR.P | 2 | 1430.634 | 2.897 | 0.353 |

+1 charge

```
		      B   	       Y
  1	 G 	    58.03	        -  	 15
  2	 D 	   173.06	  1372.76	 14
  3	 T 	   274.10	  1257.73	 13
  4	 G 	   331.12	  1156.68	 12
  5	 P 	   428.18	  1099.66	 11
  6	 P 	   525.23	  1002.61	 10
  7	 G 	   582.25	   905.56	  9
  8	 P 	   679.30	   848.54	  8
  9	 P 	   776.36	   751.48	  7
 10	 G 	   833.38	   654.43	  6
 11	 L 	   946.46	   597.41	  5
 12	 V 	  1045.53	   484.32	  4
 13	 I 	  1158.62	   385.26	  3
 14	 P 	  1255.67	   272.17	  2
 15	 R 	        -  	   175.12	  1  _
+2 charge
		      B   	       Y
  1	 G 	    29.52	        -  	 15
  2	 D 	    87.03	   686.88	 14
  3	 T 	   137.56	   629.37	 13
  4	 G 	   166.07	   578.85	 12
  5	 P 	   214.59	   550.33	 11
  6	 P 	   263.12	   501.81	 10
  7	 G 	   291.63	   453.28	  9
  8	 P 	   340.16	   424.77	  8
  9	 P 	   388.68	   376.24	  7
 10	 G 	   417.19	   327.72	  6
 11	 L 	   473.74	   299.21	  5
 12	 V 	   523.27	   242.67	  4
 13	 I 	   579.81	   193.13	  3
 14	 P 	   628.34	   136.59	  2
 15	 R 	        -  	    88.06	  1  _
```

**73. IPI00022371 Histidine-rich glycoprotein**

| Sequence | Charge | M+H+ | XCorr | DeltaCn |
| --- | --- | --- | --- | --- |
| R.ADLFYDVEALDLESPK.N | 2 | 1825.993 | 3.258 | 0.483 |

+1 charge

```
		      B   	       Y
  1	 A 	    72.04	        -  	 16
  2	 D 	   187.07	  1753.85	 15
  3	 L 	   300.16	  1638.83	 14
  4	 F 	   447.22	  1525.74	 13
  5	 Y 	   610.29	  1378.67	 12
  6	 D 	   725.31	  1215.61	 11
  7	 V 	   824.38	  1100.58	 10
  8	 E 	   953.43	  1001.51	  9
  9	 A 	  1024.46	   872.47	  8
 10	 L 	  1137.55	   801.44	  7
 11	 D 	  1252.57	   688.35	  6
 12	 L 	  1365.66	   573.32	  5
 13	 E 	  1494.70	   460.24	  4
 14	 S 	  1581.73	   331.20	  3
 15	 P 	  1678.78	   244.17	  2
 16	 K 	        -  	   147.11	  1  _
+2 charge
		      B   	       Y
  1	 A 	    36.53	        -  	 16
  2	 D 	    94.04	   877.43	 15
  3	 L 	   150.58	   819.92	 14
  4	 F 	   224.12	   763.37	 13
  5	 Y 	   305.65	   689.84	 12
  6	 D 	   363.16	   608.31	 11
  7	 V 	   412.69	   550.80	 10
  8	 E 	   477.22	   501.26	  9
  9	 A 	   512.73	   436.74	  8
 10	 L 	   569.28	   401.22	  7
 11	 D 	   626.79	   344.68	  6
 12	 L 	   683.33	   287.17	  5
 13	 E 	   747.85	   230.62	  4
 14	 S 	   791.37	   166.10	  3
 15	 P 	   839.90	   122.59	  2
 16	 K 	        -  	    74.06	  1  _
```

**74. IPI00940158 trefoil factor 3 precursor**

| Sequence | Charge | M+H+ | XCorr | DeltaCn |
| --- | --- | --- | --- | --- |
| R.IPGVPWCFKPLQEAECTF.- | 2 | 2180.474 | 3.168 | 0.545 |

+1 charge

```
		      B   	       Y
  1	 I 	   114.09	        -  	 18
  2	 P 	   211.14	  2066.22	 17
  3	 G 	   268.17	  1969.17	 16
  4	 V 	   367.23	  1912.15	 15
  5	 P 	   464.29	  1813.08	 14
  6	 W 	   650.37	  1716.02	 13
  7	 C 	   810.53	  1529.95	 12
  8	 F 	   957.60	  1369.78	 11
  9	 K 	  1085.69	  1222.71	 10
 10	 P 	  1182.75	  1094.62	  9
 11	 L 	  1295.83	   997.56	  8
 12	 Q 	  1423.89	   884.48	  7
 13	 E 	  1552.93	   756.42	  6
 14	 A 	  1623.97	   627.38	  5
 15	 E 	  1753.01	   556.34	  4
 16	 C 	  1913.18	   427.30	  3
 17	 T 	  2014.23	   267.13	  2
 18	 F 	        -  	   166.09	  1  _
+2 charge
		      B   	       Y
  1	 I 	    57.55	        -  	 18
  2	 P 	   106.08	  1033.61	 17
  3	 G 	   134.59	   985.09	 16
  4	 V 	   184.12	   956.58	 15
  5	 P 	   232.65	   907.04	 14
  6	 W 	   325.69	   858.52	 13
  7	 C 	   405.77	   765.48	 12
  8	 F 	   479.30	   685.39	 11
  9	 K 	   543.35	   611.86	 10
 10	 P 	   591.88	   547.81	  9
 11	 L 	   648.42	   499.29	  8
 12	 Q 	   712.45	   442.74	  7
 13	 E 	   776.97	   378.71	  6
 14	 A 	   812.49	   314.19	  5
 15	 E 	   877.01	   278.67	  4
 16	 C 	   957.09	   214.15	  3
 17	 T 	  1007.62	   134.07	  2
 18	 F 	        -  	    83.55	  1  _
```

**75. IPI00896380 Isoform 2 of Ig mu chain C region**

| Sequence | Charge | M+H+ | XCorr | DeltaCn |
| --- | --- | --- | --- | --- |
| K.LICQATGFSPR.Q | 2 | 1250.423 | 2.922 | 0.379 |

+1 charge

```
		      B   	       Y
  1	 L 	   114.09	        -  	 11
  2	 I 	   227.18	  1136.69	 10
  3	 C 	   387.34	  1023.60	  9
  4	 Q 	   515.40	   863.44	  8
  5	 A 	   586.44	   735.38	  7
  6	 T 	   687.48	   664.34	  6
  7	 G 	   744.51	   563.29	  5
  8	 F 	   891.57	   506.27	  4
  9	 S 	   978.61	   359.20	  3
 10	 P 	  1075.66	   272.17	  2
 11	 R 	        -  	   175.12	  1  _
+2 charge
		      B   	       Y
  1	 L 	    57.55	        -  	 11
  2	 I 	   114.09	   568.85	 10
  3	 C 	   194.17	   512.30	  9
  4	 Q 	   258.20	   432.22	  8
  5	 A 	   293.72	   368.19	  7
  6	 T 	   344.25	   332.67	  6
  7	 G 	   372.76	   282.15	  5
  8	 F 	   446.29	   253.64	  4
  9	 S 	   489.81	   180.11	  3
 10	 P 	   538.33	   136.59	  2
 11	 R 	        -  	    88.06	  1  _
```

**76. IPI00921523 Isoform 1 of Complement factor B (Fragment)**

| Sequence | Charge | M+H+ | XCorr | DeltaCn |
| --- | --- | --- | --- | --- |
| R.ALRLPPTTTCQQQKEELLPAQDIK.A | 3 | 2780.164 | 3.394 | 0.328 |

+1 charge

```
		      B   	       Y
  1	 A 	    72.04	        -  	 24
  2	 L 	   185.13	  2707.58	 23
  3	 R 	   341.23	  2594.50	 22
  4	 L 	   454.31	  2438.39	 21
  5	 P 	   551.37	  2325.31	 20
  6	 P 	   648.42	  2228.26	 19
  7	 T 	   749.47	  2131.20	 18
  8	 T 	   850.51	  2030.16	 17
  9	 T 	   951.56	  1929.11	 16
 10	 C 	  1111.73	  1828.06	 15
 11	 Q 	  1239.79	  1667.90	 14
 12	 Q 	  1367.84	  1539.84	 13
 13	 Q 	  1495.90	  1411.78	 12
 14	 K 	  1624.00	  1283.72	 11
 15	 E 	  1753.04	  1155.63	 10
 16	 E 	  1882.08	  1026.58	  9
 17	 L 	  1995.17	   897.54	  8
 18	 L 	  2108.25	   784.46	  7
 19	 P 	  2205.30	   671.37	  6
 20	 A 	  2276.34	   574.32	  5
 21	 Q 	  2404.40	   503.28	  4
 22	 D 	  2519.43	   375.22	  3
 23	 I 	  2632.51	   260.20	  2
 24	 K 	        -  	   147.11	  1  _


+2 charge
		      B   	       Y
  1	 A 	    36.53	        -  	 24
  2	 L 	    93.07	  1354.29	 23
  3	 R 	   171.12	  1297.75	 22
  4	 L 	   227.66	  1219.70	 21
  5	 P 	   276.19	  1163.16	 20
  6	 P 	   324.71	  1114.63	 19
  7	 T 	   375.24	  1066.11	 18
  8	 T 	   425.76	  1015.58	 17
  9	 T 	   476.28	   965.06	 16
 10	 C 	   556.37	   914.53	 15
 11	 Q 	   620.40	   834.45	 14
 12	 Q 	   684.43	   770.42	 13
 13	 Q 	   748.46	   706.39	 12
 14	 K 	   812.50	   642.36	 11
 15	 E 	   877.02	   578.32	 10
 16	 E 	   941.55	   513.80	  9
 17	 L 	   998.09	   449.27	  8
 18	 L 	  1054.63	   392.73	  7
 19	 P 	  1103.16	   336.19	  6
 20	 A 	  1138.67	   287.66	  5
 21	 Q 	  1202.70	   252.14	  4
 22	 D 	  1260.22	   188.12	  3
 23	 I 	  1316.76	   130.60	  2
 24	 K 	        -  	    74.06	  1  _


+3 charge
		      B   	       Y
  1	 A 	    24.69	        -  	 24
  2	 L 	    62.38	   903.20	 23
  3	 R 	   114.41	   865.50	 22
  4	 L 	   152.11	   813.47	 21
  5	 P 	   184.46	   775.77	 20
  6	 P 	   216.81	   743.42	 19
  7	 T 	   250.49	   711.07	 18
  8	 T 	   284.18	   677.39	 17
  9	 T 	   317.86	   643.71	 16
 10	 C 	   371.25	   610.03	 15
 11	 Q 	   413.93	   556.64	 14
 12	 Q 	   456.62	   513.95	 13
 13	 Q 	   499.31	   471.26	 12
 14	 K 	   542.00	   428.58	 11
 15	 E 	   585.02	   385.88	 10
 16	 E 	   628.03	   342.87	  9
 17	 L 	   665.73	   299.85	  8
 18	 L 	   703.42	   262.16	  7
 19	 P 	   735.77	   224.46	  6
 20	 A 	   759.45	   192.11	  5
 21	 Q 	   802.14	   168.43	  4
 22	 D 	   840.48	   125.75	  3
 23	 I 	   878.18	    87.40	  2
 24	 K 	        -  	    49.71	  1  _
```

**77. IPI00009901 Nuclear transport factor 2**

| Sequence | Charge | M+H+ | XCorr | DeltaCn |
| --- | --- | --- | --- | --- |
| K.AAIVEKLSSLPFQK.I | 2 | 1531.821 | 4.192 | 0.484 |

+1 charge

```
		      B   	       Y
  1	 A 	    72.04	        -  	 14
  2	 A 	   143.08	  1459.85	 13
  3	 I 	   256.17	  1388.81	 12
  4	 V 	   355.23	  1275.73	 11
  5	 E 	   484.28	  1176.66	 10
  6	 K 	   612.37	  1047.62	  9
  7	 L 	   725.46	   919.52	  8
  8	 S 	   812.49	   806.44	  7
  9	 S 	   899.52	   719.41	  6
 10	 L 	  1012.60	   632.38	  5
 11	 P 	  1109.66	   519.29	  4
 12	 F 	  1256.72	   422.24	  3
 13	 Q 	  1384.78	   275.17	  2
 14	 K 	        -  	   147.11	  1  _
+2 charge
		      B   	       Y
  1	 A 	    36.53	        -  	 14
  2	 A 	    72.04	   730.43	 13
  3	 I 	   128.59	   694.91	 12
  4	 V 	   178.12	   638.37	 11
  5	 E 	   242.64	   588.83	 10
  6	 K 	   306.69	   524.31	  9
  7	 L 	   363.23	   460.27	  8
  8	 S 	   406.75	   403.72	  7
  9	 S 	   450.26	   360.21	  6
 10	 L 	   506.81	   316.69	  5
 11	 P 	   555.33	   260.15	  4
 12	 F 	   628.87	   211.62	  3
 13	 Q 	   692.90	   138.09	  2
 14	 K 	        -  	    74.06	  1  _
```

**78. IPI00879999 ATP-dependent RNA helicase DDX54**

| Sequence | Charge | M+H+ | XCorr | DeltaCn |
| --- | --- | --- | --- | --- |
| R.DQEFYIPYRPKDFDSER.G | 2 | 2206.355 | 3.862 | 0.295 |

+1 charge

```
		      B   	       Y
  1	 D 	   116.03	        -  	 17
  2	 Q 	   244.09	  2090.00	 16
  3	 E 	   373.14	  1961.94	 15
  4	 F 	   520.20	  1832.90	 14
  5	 Y 	   683.27	  1685.83	 13
  6	 I 	   796.35	  1522.76	 12
  7	 P 	   893.40	  1409.68	 11
  8	 Y 	  1056.47	  1312.63	 10
  9	 R 	  1212.57	  1149.56	  9
 10	 P 	  1309.62	   993.46	  8
 11	 K 	  1437.72	   896.41	  7
 12	 D 	  1552.74	   768.32	  6
 13	 F 	  1699.81	   653.29	  5
 14	 D 	  1814.84	   506.22	  4
 15	 S 	  1901.87	   391.19	  3
 16	 E 	  2030.91	   304.16	  2
 17	 R 	        -  	   175.12	  1  _
+2 charge
		      B   	       Y
  1	 D 	    58.52	        -  	 17
  2	 Q 	   122.55	  1045.50	 16
  3	 E 	   187.07	   981.47	 15
  4	 F 	   260.61	   916.95	 14
  5	 Y 	   342.14	   843.42	 13
  6	 I 	   398.68	   761.89	 12
  7	 P 	   447.21	   705.34	 11
  8	 Y 	   528.74	   656.82	 10
  9	 R 	   606.79	   575.29	  9
 10	 P 	   655.31	   497.24	  8
 11	 K 	   719.36	   448.71	  7
 12	 D 	   776.88	   384.66	  6
 13	 F 	   850.41	   327.15	  5
 14	 D 	   907.92	   253.61	  4
 15	 S 	   951.44	   196.10	  3
 16	 E 	  1015.96	   152.58	  2
 17	 R 	        -  	    88.06	  1  _
```

**79. IPI00641251 CD320 antigen**

| Sequence | Charge | M+H+ | XCorr | DeltaCn |
| --- | --- | --- | --- | --- |
| R.TSGLCVPLTWR.C | 2 | 1290.487 | 3.102 | 0.304 |

+1 charge

```
		      B   	       Y
  1	 T 	   102.05	        -  	 11
  2	 S 	   189.09	  1188.75	 10
  3	 G 	   246.11	  1101.72	  9
  4	 L 	   359.19	  1044.70	  8
  5	 C 	   519.36	   931.62	  7
  6	 V 	   618.43	   771.45	  6
  7	 P 	   715.48	   672.38	  5
  8	 L 	   828.56	   575.33	  4
  9	 T 	   929.61	   462.25	  3
 10	 W 	  1115.69	   361.20	  2
 11	 R 	        -  	   175.12	  1  _
+2 charge
		      B   	       Y
  1	 T 	    51.53	        -  	 11
  2	 S 	    95.05	   594.88	 10
  3	 G 	   123.56	   551.36	  9
  4	 L 	   180.10	   522.85	  8
  5	 C 	   260.18	   466.31	  7
  6	 V 	   309.72	   386.23	  6
  7	 P 	   358.24	   336.70	  5
  8	 L 	   414.79	   288.17	  4
  9	 T 	   465.31	   231.63	  3
 10	 W 	   558.35	   181.10	  2
 11	 R 	        -  	    88.06	  1  _
```

**80. IPI00216065 Isoform 2 of Vitamin K-dependent protein Z**

| Sequence | Charge | M+H+ | XCorr | DeltaCn |
| --- | --- | --- | --- | --- |
| R.APDLQDLPWQVK.L | 2 | 1410.599 | 4.158 | 0.516 |

+1 charge

```
		      B   	       Y
  1	 A 	    72.04	        -  	 12
  2	 P 	   169.10	  1338.71	 11
  3	 D 	   284.12	  1241.65	 10
  4	 L 	   397.21	  1126.63	  9
  5	 Q 	   525.27	  1013.54	  8
  6	 D 	   640.29	   885.48	  7
  7	 L 	   753.38	   770.46	  6
  8	 P 	   850.43	   657.37	  5
  9	 W 	  1036.51	   560.32	  4
 10	 Q 	  1164.57	   374.24	  3
 11	 V 	  1263.64	   246.18	  2
 12	 K 	        -  	   147.11	  1  _
+2 charge
		      B   	       Y
  1	 A 	    36.53	        -  	 12
  2	 P 	    85.05	   669.86	 11
  3	 D 	   142.57	   621.33	 10
  4	 L 	   199.11	   563.82	  9
  5	 Q 	   263.14	   507.27	  8
  6	 D 	   320.65	   443.25	  7
  7	 L 	   377.19	   385.73	  6
  8	 P 	   425.72	   329.19	  5
  9	 W 	   518.76	   280.66	  4
 10	 Q 	   582.79	   187.62	  3
 11	 V 	   632.32	   123.59	  2
 12	 K 	        -  	    74.06	  1  _
```

**81. IPI00942979 Transketolase**

| Sequence | Charge | M+H+ | XCorr | DeltaCn |
| --- | --- | --- | --- | --- |
| K.NMAEQIIQEIYSQIQSK.K | 2 | 2024.285 | 2.869 | 0.45 |

+1 charge

```
		      B   	       Y
  1	 N 	   115.05	        -  	 17
  2	 M 	   246.09	  1908.97	 16
  3	 A 	   317.13	  1777.93	 15
  4	 E 	   446.17	  1706.90	 14
  5	 Q 	   574.23	  1577.85	 13
  6	 I 	   687.31	  1449.79	 12
  7	 I 	   800.40	  1336.71	 11
  8	 Q 	   928.46	  1223.63	 10
  9	 E 	  1057.50	  1095.57	  9
 10	 I 	  1170.58	   966.53	  8
 11	 Y 	  1333.65	   853.44	  7
 12	 S 	  1420.68	   690.38	  6
 13	 Q 	  1548.74	   603.35	  5
 14	 I 	  1661.82	   475.29	  4
 15	 Q 	  1789.88	   362.20	  3
 16	 S 	  1876.91	   234.14	  2
 17	 K 	        -  	   147.11	  1  _
+2 charge
		      B   	       Y
  1	 N 	    58.03	        -  	 17
  2	 M 	   123.55	   954.99	 16
  3	 A 	   159.07	   889.47	 15
  4	 E 	   223.59	   853.95	 14
  5	 Q 	   287.62	   789.43	 13
  6	 I 	   344.16	   725.40	 12
  7	 I 	   400.70	   668.86	 11
  8	 Q 	   464.73	   612.32	 10
  9	 E 	   529.25	   548.29	  9
 10	 I 	   585.79	   483.77	  8
 11	 Y 	   667.33	   427.22	  7
 12	 S 	   710.84	   345.69	  6
 13	 Q 	   774.87	   302.18	  5
 14	 I 	   831.41	   238.15	  4
 15	 Q 	   895.44	   181.61	  3
 16	 S 	   938.96	   117.58	  2
 17	 K 	        -  	    74.06	  1  _
```

**82. IPI00297252 Isoform 1 of Extracellular sulfatase Sulf-2**

| Sequence | Charge | M+H+ | XCorr | DeltaCn |
| --- | --- | --- | --- | --- |
| R.VYHVGLGDAAQPR.N | 2 | 1383.537 | 3.89 | 0.405 |

+1 charge

```
		      B   	       Y
  1	 V 	   100.08	        -  	 13
  2	 Y 	   263.14	  1283.65	 12
  3	 H 	   400.20	  1120.59	 11
  4	 V 	   499.27	   983.53	 10
  5	 G 	   556.29	   884.46	  9
  6	 L 	   669.37	   827.44	  8
  7	 G 	   726.39	   714.35	  7
  8	 D 	   841.42	   657.33	  6
  9	 A 	   912.46	   542.30	  5
 10	 A 	   983.49	   471.27	  4
 11	 Q 	  1111.55	   400.23	  3
 12	 P 	  1208.61	   272.17	  2
 13	 R 	        -  	   175.12	  1  _
+2 charge
		      B   	       Y
  1	 V 	    50.54	        -  	 13
  2	 Y 	   132.07	   642.33	 12
  3	 H 	   200.60	   560.80	 11
  4	 V 	   250.14	   492.27	 10
  5	 G 	   278.65	   442.73	  9
  6	 L 	   335.19	   414.22	  8
  7	 G 	   363.70	   357.68	  7
  8	 D 	   421.21	   329.17	  6
  9	 A 	   456.73	   271.66	  5
 10	 A 	   492.25	   236.14	  4
 11	 Q 	   556.28	   200.62	  3
 12	 P 	   604.81	   136.59	  2
 13	 R 	        -  	    88.06	  1  _
```

**83. IPI00027166 Metalloproteinase inhibitor 2**

| Sequence | Charge | M+H+ | XCorr | DeltaCn |
| --- | --- | --- | --- | --- |
| K.AVSEKEVDSGNDIYGNPIKR.I | 3 | 2192.372 | 4.807 | 0.542 |

+1 charge

```
		      B   	       Y
  1	 A 	    72.04	        -  	 20
  2	 V 	   171.11	  2120.06	 19
  3	 S 	   258.14	  2020.99	 18
  4	 E 	   387.19	  1933.96	 17
  5	 K 	   515.28	  1804.92	 16
  6	 E 	   644.32	  1676.82	 15
  7	 V 	   743.39	  1547.78	 14
  8	 D 	   858.42	  1448.71	 13
  9	 S 	   945.45	  1333.69	 12
 10	 G 	  1002.47	  1246.65	 11
 11	 N 	  1116.52	  1189.63	 10
 12	 D 	  1231.54	  1075.59	  9
 13	 I 	  1344.63	   960.56	  8
 14	 Y 	  1507.69	   847.48	  7
 15	 G 	  1564.71	   684.42	  6
 16	 N 	  1678.76	   627.39	  5
 17	 P 	  1775.81	   513.35	  4
 18	 I 	  1888.89	   416.30	  3
 19	 K 	  2016.99	   303.21	  2
 20	 R 	        -  	   175.12	  1  _
+2 charge
		      B   	       Y
  1	 A 	    36.53	        -  	 20
  2	 V 	    86.06	  1060.53	 19
  3	 S 	   129.58	  1011.00	 18
  4	 E 	   194.10	   967.48	 17
  5	 K 	   258.14	   902.96	 16
  6	 E 	   322.67	   838.92	 15
  7	 V 	   372.20	   774.39	 14
  8	 D 	   429.71	   724.86	 13
  9	 S 	   473.23	   667.35	 12
 10	 G 	   501.74	   623.83	 11
 11	 N 	   558.76	   595.32	 10
 12	 D 	   616.28	   538.30	  9
 13	 I 	   672.82	   480.78	  8
 14	 Y 	   754.35	   424.24	  7
 15	 G 	   782.86	   342.71	  6
 16	 N 	   839.88	   314.20	  5
 17	 P 	   888.41	   257.18	  4
 18	 I 	   944.95	   208.65	  3
 19	 K 	  1009.00	   152.11	  2
 20	 R 	        -  	    88.06	  1  _

+3 charge
		      B   	       Y
  1	 A 	    24.69	        -  	 20
  2	 V 	    57.71	   707.36	 19
  3	 S 	    86.72	   674.34	 18
  4	 E 	   129.73	   645.33	 17
  5	 K 	   172.43	   602.31	 16
  6	 E 	   215.45	   559.61	 15
  7	 V 	   248.47	   516.60	 14
  8	 D 	   286.81	   483.58	 13
  9	 S 	   315.82	   445.23	 12
 10	 G 	   334.83	   416.22	 11
 11	 N 	   372.84	   397.22	 10
 12	 D 	   411.19	   359.20	  9
 13	 I 	   448.88	   320.86	  8
 14	 Y 	   503.24	   283.16	  7
 15	 G 	   522.24	   228.81	  6
 16	 N 	   560.26	   209.80	  5
 17	 P 	   592.61	   171.79	  4
 18	 I 	   630.30	   139.44	  3
 19	 K 	   673.00	   101.74	  2
 20	 R 	        -  	    59.04	  1  _
```

**84. IPI00386839 Amyloid lambda 6 light chain variable region SAR (Fragment)**

| Sequence | Charge | M+H+ | XCorr | DeltaCn |
| --- | --- | --- | --- | --- |
| - .NFMLTQPHSVSESPGK.T | 2 | 1759.965 | 3.218 | 0.526 |

+1 charge

```
		      B   	       Y
  1	 N 	   115.05	        -  	 16
  2	 F 	   262.12	  1644.81	 15
  3	 M 	   393.16	  1497.74	 14
  4	 L 	   506.24	  1366.70	 13
  5	 T 	   607.29	  1253.61	 12
  6	 Q 	   735.35	  1152.56	 11
  7	 P 	   832.40	  1024.51	 10
  8	 H 	   969.46	   927.45	  9
  9	 S 	  1056.49	   790.39	  8
 10	 V 	  1155.56	   703.36	  7
 11	 S 	  1242.59	   604.29	  6
 12	 E 	  1371.64	   517.26	  5
 13	 S 	  1458.67	   388.22	  4
 14	 P 	  1555.72	   301.19	  3
 15	 G 	  1612.74	   204.13	  2
 16	 K 	        -  	   147.11	  1  _
+2 charge
		      B   	       Y
  1	 N 	    58.03	        -  	 16
  2	 F 	   131.56	   822.91	 15
  3	 M 	   197.08	   749.37	 14
  4	 L 	   253.63	   683.85	 13
  5	 T 	   304.15	   627.31	 12
  6	 Q 	   368.18	   576.79	 11
  7	 P 	   416.70	   512.76	 10
  8	 H 	   485.23	   464.23	  9
  9	 S 	   528.75	   395.70	  8
 10	 V 	   578.28	   352.18	  7
 11	 S 	   621.80	   302.65	  6
 12	 E 	   686.32	   259.13	  5
 13	 S 	   729.84	   194.61	  4
 14	 P 	   778.36	   151.10	  3
 15	 G 	   806.87	   102.57	  2
 16	 K 	        -  	    74.06	  1  _
```

**85. IPI00011140 Protein NOV homolog**

| Sequence | Charge | M+H+ | XCorr | DeltaCn |
| --- | --- | --- | --- | --- |
| R.AVLDGCSCCLVCAR.Q | 2 | 1641.823 | 4.324 | 0.446 |

+1 charge

```
		      B   	       Y
  1	 A 	    72.04	        -  	 14
  2	 V 	   171.11	  1570.20	 13
  3	 L 	   284.20	  1471.13	 12
  4	 D 	   399.22	  1358.05	 11
  5	 G 	   456.25	  1243.02	 10
  6	 C 	   616.41	  1186.00	  9
  7	 S 	   703.44	  1025.84	  8
  8	 C 	   863.61	   938.80	  7
  9	 C 	  1023.77	   778.64	  6
 10	 L 	  1136.86	   618.47	  5
 11	 V 	  1235.93	   505.39	  4
 12	 C 	  1396.09	   406.32	  3
 13	 A 	  1467.13	   246.16	  2
 14	 R 	        -  	   175.12	  1  _
+2 charge
		      B   	       Y
  1	 A 	    36.53	        -  	 14
  2	 V 	    86.06	   785.60	 13
  3	 L 	   142.60	   736.07	 12
  4	 D 	   200.12	   679.53	 11
  5	 G 	   228.63	   622.02	 10
  6	 C 	   308.71	   593.50	  9
  7	 S 	   352.22	   513.42	  8
  8	 C 	   432.31	   469.91	  7
  9	 C 	   512.39	   389.82	  6
 10	 L 	   568.93	   309.74	  5
 11	 V 	   618.47	   253.20	  4
 12	 C 	   698.55	   203.66	  3
 13	 A 	   734.07	   123.58	  2
 14	 R 	        -  	    88.06	  1  _
```

**86. IPI00419585 Peptidyl-prolyl cis-trans isomerase A**

| Sequence | Charge | M+H+ | XCorr | DeltaCn |
| --- | --- | --- | --- | --- |
| R.VSFELFADKVPK.T | 2 | 1380.613 | 2.887 | 0.399 |

+1 charge

```
		      B   	       Y
  1	 V 	   100.08	        -  	 12
  2	 S 	   187.11	  1280.69	 11
  3	 F 	   334.18	  1193.66	 10
  4	 E 	   463.22	  1046.59	  9
  5	 L 	   576.30	   917.55	  8
  6	 F 	   723.37	   804.46	  7
  7	 A 	   794.41	   657.39	  6
  8	 D 	   909.44	   586.36	  5
  9	 K 	  1037.53	   471.33	  4
 10	 V 	  1136.60	   343.23	  3
 11	 P 	  1233.65	   244.17	  2
 12	 K 	        -  	   147.11	  1  _
+2 charge
		      B   	       Y
  1	 V 	    50.54	        -  	 12
  2	 S 	    94.06	   640.85	 11
  3	 F 	   167.59	   597.33	 10
  4	 E 	   232.11	   523.80	  9
  5	 L 	   288.66	   459.28	  8
  6	 F 	   362.19	   402.73	  7
  7	 A 	   397.71	   329.20	  6
  8	 D 	   455.22	   293.68	  5
  9	 K 	   519.27	   236.17	  4
 10	 V 	   568.80	   172.12	  3
 11	 P 	   617.33	   122.59	  2
 12	 K 	        -  	    74.06	  1  _
```

**87. IPI00297420 Frizzled-4 precursor**

| Sequence | Charge | M+H+ | XCorr | DeltaCn |
| --- | --- | --- | --- | --- |
| K.INIPIGPCGGMCLSVK.R | 2 | 1717.054 | 3.445 | 0.494 |

+1 charge

```
		      B   	       Y
  1	 I 	   114.09	        -  	 16
  2	 N 	   228.13	  1603.05	 15
  3	 I 	   341.22	  1489.01	 14
  4	 P 	   438.27	  1375.92	 13
  5	 I 	   551.36	  1278.87	 12
  6	 G 	   608.38	  1165.79	 11
  7	 P 	   705.43	  1108.76	 10
  8	 C 	   865.59	  1011.71	  9
  9	 G 	   922.62	   851.55	  8
 10	 G 	   979.64	   794.52	  7
 11	 M 	  1110.68	   737.50	  6
 12	 C 	  1270.84	   606.46	  5
 13	 L 	  1383.93	   446.30	  4
 14	 S 	  1470.96	   333.21	  3
 15	 V 	  1570.03	   246.18	  2
 16	 K 	        -  	   147.11	  1  _
+2 charge

		      B   	       Y
  1	 I 	    57.55	        -  	 16
  2	 N 	   114.57	   802.03	 15
  3	 I 	   171.11	   745.01	 14
  4	 P 	   219.64	   688.46	 13
  5	 I 	   276.18	   639.94	 12
  6	 G 	   304.69	   583.40	 11
  7	 P 	   353.22	   554.89	 10
  8	 C 	   433.30	   506.36	  9
  9	 G 	   461.81	   426.28	  8
 10	 G 	   490.32	   397.77	  7
 11	 M 	   555.84	   369.26	  6
 12	 C 	   635.93	   303.73	  5
 13	 L 	   692.47	   223.65	  4
 14	 S 	   735.98	   167.11	  3
 15	 V 	   785.52	   123.59	  2
 16	 K 	        -  	    74.06	  1  _
```

**88. IPI00409566 Isoform 1 of Polycystin-1**

| Sequence | Charge | M+H+ | XCorr | DeltaCn |
| --- | --- | --- | --- | --- |
| R.TLGPALRIPADATALDVSHNLLR.A | 3 | 2415.777 | 4.445 | 0.504 |

+1 charge

```
		      B   	       Y
  1	 T 	   102.05	        -  	 23
  2	 L 	   215.14	  2313.30	 22
  3	 G 	   272.16	  2200.22	 21
  4	 P 	   369.21	  2143.20	 20
  5	 A 	   440.25	  2046.15	 19
  6	 L 	   553.33	  1975.11	 18
  7	 R 	   709.44	  1862.02	 17
  8	 I 	   822.52	  1705.92	 16
  9	 P 	   919.57	  1592.84	 15
 10	 A 	   990.61	  1495.79	 14
 11	 D 	  1105.64	  1424.75	 13
 12	 A 	  1176.67	  1309.72	 12
 13	 T 	  1277.72	  1238.69	 11
 14	 A 	  1348.76	  1137.64	 10
 15	 L 	  1461.84	  1066.60	  9
 16	 D 	  1576.87	   953.52	  8
 17	 V 	  1675.94	   838.49	  7
 18	 S 	  1762.97	   739.42	  6
 19	 H 	  1900.03	   652.39	  5
 20	 N 	  2014.07	   515.33	  4
 21	 L 	  2127.16	   401.29	  3
 22	 L 	  2240.24	   288.20	  2
 23	 R 	        -  	   175.12	  1  _


+2 charge
		      B   	       Y
  1	 T 	    51.53	        -  	 23
  2	 L 	   108.07	  1157.16	 22
  3	 G 	   136.58	  1100.61	 21
  4	 P 	   185.11	  1072.10	 20
  5	 A 	   220.63	  1023.58	 19
  6	 L 	   277.17	   988.06	 18
  7	 R 	   355.22	   931.52	 17
  8	 I 	   411.76	   853.47	 16
  9	 P 	   460.29	   796.92	 15
 10	 A 	   495.81	   748.40	 14
 11	 D 	   553.32	   712.88	 13
 12	 A 	   588.84	   655.36	 12
 13	 T 	   639.36	   619.85	 11
 14	 A 	   674.88	   569.32	 10
 15	 L 	   731.42	   533.80	  9
 16	 D 	   788.94	   477.26	  8
 17	 V 	   838.47	   419.75	  7
 18	 S 	   881.99	   370.21	  6
 19	 H 	   950.52	   326.70	  5
 20	 N 	  1007.54	   258.17	  4
 21	 L 	  1064.08	   201.15	  3
 22	 L 	  1120.62	   144.61	  2
 23	 R 	        -  	    88.06	  1  _


+3 charge
		      B   	       Y
  1	 T 	    34.69	        -  	 23
  2	 L 	    72.38	   771.77	 22
  3	 G 	    91.39	   734.08	 21
  4	 P 	   123.74	   715.07	 20
  5	 A 	   147.42	   682.72	 19
  6	 L 	   185.12	   659.04	 18
  7	 R 	   237.15	   621.35	 17
  8	 I 	   274.84	   569.31	 16
  9	 P 	   307.20	   531.62	 15
 10	 A 	   330.87	   499.27	 14
 11	 D 	   369.22	   475.59	 13
 12	 A 	   392.90	   437.25	 12
 13	 T 	   426.58	   413.57	 11
 14	 A 	   450.26	   379.88	 10
 15	 L 	   487.95	   356.20	  9
 16	 D 	   526.29	   318.51	  8
 17	 V 	   559.32	   280.17	  7
 18	 S 	   588.33	   247.15	  6
 19	 H 	   634.01	   218.13	  5
 20	 N 	   672.03	   172.45	  4
 21	 L 	   709.72	   134.43	  3
 22	 L 	   747.42	    96.74	  2
 23	 R 	        -  	    59.04	  1  _
```

**89. IPI00167710 Isoform 1 of Fibulin-7**

| Sequence | Charge | M+H+ | XCorr | DeltaCn |
| --- | --- | --- | --- | --- |
| K.TISFHYLSLPSNLKTPITLFR.M | 3 | 2449.875 | 4.507 | 0.542 |

+1 charge

```
		      B   	       Y
  1	 T 	   102.05	        -  	 21
  2	 I 	   215.14	  2347.32	 20
  3	 S 	   302.17	  2234.23	 19
  4	 F 	   449.24	  2147.20	 18
  5	 H 	   586.30	  2000.13	 17
  6	 Y 	   749.36	  1863.07	 16
  7	 L 	   862.45	  1700.01	 15
  8	 S 	   949.48	  1586.93	 14
  9	 L 	  1062.56	  1499.89	 13
 10	 P 	  1159.61	  1386.81	 12
 11	 S 	  1246.65	  1289.76	 11
 12	 N 	  1360.69	  1202.73	 10
 13	 L 	  1473.77	  1088.68	  9
 14	 K 	  1601.87	   975.60	  8
 15	 T 	  1702.92	   847.50	  7
 16	 P 	  1799.97	   746.46	  6
 17	 I 	  1913.05	   649.40	  5
 18	 T 	  2014.10	   536.32	  4
 19	 L 	  2127.18	   435.27	  3
 20	 F 	  2274.25	   322.19	  2
 21	 R 	        -  	   175.12	  1  _


+2 charge
		      B   	       Y
  1	 T 	    51.53	        -  	 21
  2	 I 	   108.07	  1174.16	 20
  3	 S 	   151.59	  1117.62	 19
  4	 F 	   225.12	  1074.10	 18
  5	 H 	   293.65	  1000.57	 17
  6	 Y 	   375.18	   932.04	 16
  7	 L 	   431.73	   850.51	 15
  8	 S 	   475.24	   793.97	 14
  9	 L 	   531.78	   750.45	 13
 10	 P 	   580.31	   693.91	 12
 11	 S 	   623.83	   645.38	 11
 12	 N 	   680.85	   601.87	 10
 13	 L 	   737.39	   544.84	  9
 14	 K 	   801.44	   488.30	  8
 15	 T 	   851.96	   424.26	  7
 16	 P 	   900.49	   373.73	  6
 17	 I 	   957.03	   325.21	  5
 18	 T 	  1007.55	   268.66	  4
 19	 L 	  1064.10	   218.14	  3
 20	 F 	  1137.63	   161.60	  2
 21	 R 	        -  	    88.06	  1  _


+3 charge
		      B   	       Y
  1	 T 	    34.69	        -  	 21
  2	 I 	    72.38	   783.11	 20
  3	 S 	   101.40	   745.42	 19
  4	 F 	   150.42	   716.41	 18
  5	 H 	   196.10	   667.38	 17
  6	 Y 	   250.46	   621.70	 16
  7	 L 	   288.15	   567.34	 15
  8	 S 	   317.16	   529.65	 14
  9	 L 	   354.86	   500.64	 13
 10	 P 	   387.21	   462.94	 12
 11	 S 	   416.22	   430.59	 11
 12	 N 	   454.23	   401.58	 10
 13	 L 	   491.93	   363.57	  9
 14	 K 	   534.63	   325.87	  8
 15	 T 	   568.31	   283.17	  7
 16	 P 	   600.66	   249.49	  6
 17	 I 	   638.36	   217.14	  5
 18	 T 	   672.04	   179.44	  4
 19	 L 	   709.73	   145.76	  3
 20	 F 	   758.76	   108.07	  2
 21	 R 	        -  	    59.04	  1  _
```

**90. IPI00744184 Arylsulfatase A**

| Sequence | Charge | M+H+ | XCorr | DeltaCn |
| --- | --- | --- | --- | --- |
| R.GGLPLEEVTVAEVLAAR.G | 2 | 1724.98 | 4.156 | 0.429 |

+1 charge

```
		      B   	       Y
  1	 G 	    58.03	        -  	 17
  2	 G 	   115.05	  1666.94	 16
  3	 L 	   228.13	  1609.92	 15
  4	 P 	   325.19	  1496.83	 14
  5	 L 	   438.27	  1399.78	 13
  6	 E 	   567.31	  1286.70	 12
  7	 E 	   696.36	  1157.65	 11
  8	 V 	   795.42	  1028.61	 10
  9	 T 	   896.47	   929.54	  9
 10	 V 	   995.54	   828.49	  8
 11	 A 	  1066.58	   729.43	  7
 12	 E 	  1195.62	   658.39	  6
 13	 V 	  1294.69	   529.35	  5
 14	 L 	  1407.77	   430.28	  4
 15	 A 	  1478.81	   317.19	  3
 16	 A 	  1549.85	   246.16	  2
 17	 R 	        -  	   175.12	  1  _
+2 charge
		      B   	       Y
  1	 G 	    29.52	        -  	 17
  2	 G 	    58.03	   833.97	 16
  3	 L 	   114.57	   805.46	 15
  4	 P 	   163.10	   748.92	 14
  5	 L 	   219.64	   700.39	 13
  6	 E 	   284.16	   643.85	 12
  7	 E 	   348.68	   579.33	 11
  8	 V 	   398.22	   514.81	 10
  9	 T 	   448.74	   465.27	  9
 10	 V 	   498.27	   414.75	  8
 11	 A 	   533.79	   365.22	  7
 12	 E 	   598.31	   329.70	  6
 13	 V 	   647.85	   265.18	  5
 14	 L 	   704.39	   215.64	  4
 15	 A 	   739.91	   159.10	  3
 16	 A 	   775.43	   123.58	  2
 17	 R 	        -  	    88.06	  1  _
```

**91. IPI00218501 Isoform 2 of Ryanodine receptor 2**

| Sequence | Charge | M+H+ | XCorr | DeltaCn |
| --- | --- | --- | --- | --- |
| K.EAATPEEESDTLEK.E | 2 | 1549.573 | 3.361 | 0.201 |

+1 charge

```
		      B   	       Y
  1	 E 	   130.05	        -  	 14
  2	 A 	   201.09	  1419.65	 13
  3	 A 	   272.12	  1348.61	 12
  4	 T 	   373.17	  1277.57	 11
  5	 P 	   470.22	  1176.53	 10
  6	 E 	   599.27	  1079.47	  9
  7	 E 	   728.31	   950.43	  8
  8	 E 	   857.35	   821.39	  7
  9	 S 	   944.38	   692.35	  6
 10	 D 	  1059.41	   605.31	  5
 11	 T 	  1160.46	   490.29	  4
 12	 L 	  1273.54	   389.24	  3
 13	 E 	  1402.59	   276.16	  2
 14	 K 	        -  	   147.11	  1  _
+2 charge
		      B   	       Y
  1	 E 	    65.53	        -  	 14
  2	 A 	   101.05	   710.33	 13
  3	 A 	   136.57	   674.81	 12
  4	 T 	   187.09	   639.29	 11
  5	 P 	   235.62	   588.77	 10
  6	 E 	   300.14	   540.24	  9
  7	 E 	   364.66	   475.72	  8
  8	 E 	   429.18	   411.20	  7
  9	 S 	   472.70	   346.68	  6
 10	 D 	   530.21	   303.16	  5
 11	 T 	   580.73	   245.65	  4
 12	 L 	   637.28	   195.12	  3
 13	 E 	   701.80	   138.58	  2
 14	 K 	        -  	    74.06	  1  _
```

**92. IPI00296713 Isoform 1 of Granulins**

| Sequence | Charge | M+H+ | XCorr | DeltaCn |
| --- | --- | --- | --- | --- |
| K.CDMEVSCPDGYTCCR.L | 2 | 1911.012 | 5.061 | 0.611 |

+1 charge

```
		      B   	       Y
  1	 C 	   161.17	        -  	 15
  2	 D 	   276.20	  1750.04	 14
  3	 M 	   407.24	  1635.01	 13
  4	 E 	   536.28	  1503.97	 12
  5	 V 	   635.35	  1374.93	 11
  6	 S 	   722.38	  1275.86	 10
  7	 C 	   882.55	  1188.83	  9
  8	 P 	   979.60	  1028.66	  8
  9	 D 	  1094.63	   931.61	  7
 10	 G 	  1151.65	   816.58	  6
 11	 Y 	  1314.71	   759.56	  5
 12	 T 	  1415.76	   596.50	  4
 13	 C 	  1575.93	   495.45	  3
 14	 C 	  1736.09	   335.28	  2
 15	 R 	        -  	   175.12	  1  _
+2 charge
		      B   	       Y
  1	 C 	    81.09	        -  	 15
  2	 D 	   138.60	   875.52	 14
  3	 M 	   204.12	   818.01	 13
  4	 E 	   268.64	   752.49	 12
  5	 V 	   318.18	   687.97	 11
  6	 S 	   361.70	   638.43	 10
  7	 C 	   441.78	   594.92	  9
  8	 P 	   490.30	   514.83	  8
  9	 D 	   547.82	   466.31	  7
 10	 G 	   576.33	   408.79	  6
 11	 Y 	   657.86	   380.28	  5
 12	 T 	   708.38	   298.75	  4
 13	 C 	   788.47	   248.23	  3
 14	 C 	   868.55	   168.15	  2
 15	 R 	        -  	    88.06	  1  _
```

**93. IPI00303832 Paf1/RNA polymerase II complex component**

| Sequence | Charge | M+H+ | XCorr | DeltaCn |
| --- | --- | --- | --- | --- |
| K.DKDLNSKSASDLSEDLFK.V | 2 | 2013.149 | 3.138 | 0.248 |

+1 charge

```
		      B   	       Y
  1	 D 	   116.03	        -  	 18
  2	 K 	   244.13	  1896.95	 17
  3	 D 	   359.16	  1768.86	 16
  4	 L 	   472.24	  1653.83	 15
  5	 N 	   586.28	  1540.75	 14
  6	 S 	   673.32	  1426.71	 13
  7	 K 	   801.41	  1339.67	 12
  8	 S 	   888.44	  1211.58	 11
  9	 A 	   959.48	  1124.55	 10
 10	 S 	  1046.51	  1053.51	  9
 11	 D 	  1161.54	   966.48	  8
 12	 L 	  1274.62	   851.45	  7
 13	 S 	  1361.65	   738.37	  6
 14	 E 	  1490.70	   651.33	  5
 15	 D 	  1605.72	   522.29	  4
 16	 L 	  1718.81	   407.27	  3
 17	 F 	  1865.88	   294.18	  2
 18	 K 	        -  	   147.11	  1  _
+2 charge
		      B   	       Y
  1	 D 	    58.52	        -  	 18
  2	 K 	   122.57	   948.98	 17
  3	 D 	   180.08	   884.93	 16
  4	 L 	   236.62	   827.42	 15
  5	 N 	   293.65	   770.88	 14
  6	 S 	   337.16	   713.86	 13
  7	 K 	   401.21	   670.34	 12
  8	 S 	   444.72	   606.29	 11
  9	 A 	   480.24	   562.78	 10
 10	 S 	   523.76	   527.26	  9
 11	 D 	   581.27	   483.74	  8
 12	 L 	   637.81	   426.23	  7
 13	 S 	   681.33	   369.69	  6
 14	 E 	   745.85	   326.17	  5
 15	 D 	   803.37	   261.65	  4
 16	 L 	   859.91	   204.14	  3
 17	 F 	   933.44	   147.59	  2
 18	 K 	        -  	    74.06	  1  _
```

**94. IPI00002435 CD27 antigen**

| Sequence | Charge | M+H+ | XCorr | DeltaCn |
| --- | --- | --- | --- | --- |
| K.LCCQMCEPGTFLVK.D | 2 | 1744.027 | 4.455 | 0.557 |

+1 charge

```
		      B   	       Y
  1	 L 	   114.09	        -  	 14
  2	 C 	   274.26	  1630.09	 13
  3	 C 	   434.42	  1469.93	 12
  4	 Q 	   562.48	  1309.76	 11
  5	 M 	   693.52	  1181.70	 10
  6	 C 	   853.69	  1050.66	  9
  7	 E 	   982.73	   890.50	  8
  8	 P 	  1079.78	   761.46	  7
  9	 G 	  1136.80	   664.40	  6
 10	 T 	  1237.85	   607.38	  5
 11	 F 	  1384.92	   506.33	  4
 12	 L 	  1498.00	   359.27	  3
 13	 V 	  1597.07	   246.18	  2
 14	 K 	        -  	   147.11	  1  _
+2 charge
		      B   	       Y
  1	 L 	    57.55	        -  	 14
  2	 C 	   137.63	   815.55	 13
  3	 C 	   217.71	   735.47	 12
  4	 Q 	   281.74	   655.38	 11
  5	 M 	   347.26	   591.36	 10
  6	 C 	   427.35	   525.84	  9
  7	 E 	   491.87	   445.75	  8
  8	 P 	   540.39	   381.23	  7
  9	 G 	   568.91	   332.71	  6
 10	 T 	   619.43	   304.19	  5
 11	 F 	   692.96	   253.67	  4
 12	 L 	   749.51	   180.14	  3
 13	 V 	   799.04	   123.59	  2
 14	 K 	        -  	    74.06	  1  _
```

**95. IPI00015199 T-cell antigen CD7**

| Sequence | Charge | M+H+ | XCorr | DeltaCn |
| --- | --- | --- | --- | --- |
| R.QLGPQPQDIIYYEDGVVPTTDRR.F | 3 | 2661.908 | 3.511 | 0.423 |

+1 charge

```
		      B   	       Y
  1	 Q 	   129.07	        -  	 23
  2	 L 	   242.15	  2532.27	 22
  3	 G 	   299.17	  2419.19	 21
  4	 P 	   396.22	  2362.17	 20
  5	 Q 	   524.28	  2265.11	 19
  6	 P 	   621.34	  2137.06	 18
  7	 Q 	   749.39	  2040.00	 17
  8	 D 	   864.42	  1911.94	 16
  9	 I 	   977.51	  1796.92	 15
 10	 I 	  1090.59	  1683.83	 14
 11	 Y 	  1253.65	  1570.75	 13
 12	 Y 	  1416.72	  1407.69	 12
 13	 E 	  1545.76	  1244.62	 11
 14	 D 	  1660.79	  1115.58	 10
 15	 G 	  1717.81	  1000.55	  9
 16	 V 	  1816.88	   943.53	  8
 17	 V 	  1915.94	   844.46	  7
 18	 P 	  2013.00	   745.40	  6
 19	 T 	  2114.04	   648.34	  5
 20	 T 	  2215.09	   547.29	  4
 21	 D 	  2330.12	   446.25	  3
 22	 R 	  2486.22	   331.22	  2
 23	 R 	        -  	   175.12	  1  _


+2 charge
		      B   	       Y
  1	 Q 	    65.04	        -  	 23
  2	 L 	   121.58	  1266.64	 22
  3	 G 	   150.09	  1210.10	 21
  4	 P 	   198.62	  1181.59	 20
  5	 Q 	   262.64	  1133.06	 19
  6	 P 	   311.17	  1069.03	 18
  7	 Q 	   375.20	  1020.51	 17
  8	 D 	   432.71	   956.48	 16
  9	 I 	   489.26	   898.96	 15
 10	 I 	   545.80	   842.42	 14
 11	 Y 	   627.33	   785.88	 13
 12	 Y 	   708.86	   704.35	 12
 13	 E 	   773.38	   622.82	 11
 14	 D 	   830.90	   558.29	 10
 15	 G 	   859.41	   500.78	  9
 16	 V 	   908.94	   472.27	  8
 17	 V 	   958.48	   422.74	  7
 18	 P 	  1007.00	   373.20	  6
 19	 T 	  1057.53	   324.67	  5
 20	 T 	  1108.05	   274.15	  4
 21	 D 	  1165.56	   223.63	  3
 22	 R 	  1243.61	   166.11	  2
 23	 R 	        -  	    88.06	  1  _


+3 charge
		      B   	       Y
  1	 Q 	    43.69	        -  	 23
  2	 L 	    81.39	   844.76	 22
  3	 G 	   100.40	   807.07	 21
  4	 P 	   132.75	   788.06	 20
  5	 Q 	   175.43	   755.71	 19
  6	 P 	   207.78	   713.02	 18
  7	 Q 	   250.47	   680.67	 17
  8	 D 	   288.81	   637.99	 16
  9	 I 	   326.51	   599.64	 15
 10	 I 	   364.20	   561.95	 14
 11	 Y 	   418.56	   524.25	 13
 12	 Y 	   472.91	   469.90	 12
 13	 E 	   515.92	   415.55	 11
 14	 D 	   554.27	   372.53	 10
 15	 G 	   573.27	   334.19	  9
 16	 V 	   606.30	   315.18	  8
 17	 V 	   639.32	   282.16	  7
 18	 P 	   671.67	   249.14	  6
 19	 T 	   705.35	   216.79	  5
 20	 T 	   739.04	   183.10	  4
 21	 D 	   777.38	   149.42	  3
 22	 R 	   829.41	   111.08	  2
 23	 R 	        -  	    59.04	  1  _
```

**96. IPI00018136 Isoform 1 of Vascular cell adhesion protein 1**

| Sequence | Charge | M+H+ | XCorr | DeltaCn |
| --- | --- | --- | --- | --- |
| K.SQEFLEDADRKSLETK.S | 2 | 1897.033 | 3.524 | 0.314 |

+1 charge

```
		      B   	       Y
  1	 S 	    88.04	        -  	 16
  2	 Q 	   216.10	  1808.90	 15
  3	 E 	   345.14	  1680.84	 14
  4	 F 	   492.21	  1551.80	 13
  5	 L 	   605.29	  1404.73	 12
  6	 E 	   734.34	  1291.65	 11
  7	 D 	   849.36	  1162.61	 10
  8	 A 	   920.40	  1047.58	  9
  9	 D 	  1035.43	   976.54	  8
 10	 R 	  1191.53	   861.52	  7
 11	 K 	  1319.62	   705.41	  6
 12	 S 	  1406.65	   577.32	  5
 13	 L 	  1519.74	   490.29	  4
 14	 E 	  1648.78	   377.20	  3
 15	 T 	  1749.83	   248.16	  2
 16	 K 	        -  	   147.11	  1  _
+2 charge
		      B   	       Y
  1	 S 	    44.52	        -  	 16
  2	 Q 	   108.55	   904.95	 15
  3	 E 	   173.07	   840.93	 14
  4	 F 	   246.61	   776.40	 13
  5	 L 	   303.15	   702.87	 12
  6	 E 	   367.67	   646.33	 11
  7	 D 	   425.18	   581.81	 10
  8	 A 	   460.70	   524.29	  9
  9	 D 	   518.22	   488.77	  8
 10	 R 	   596.27	   431.26	  7
 11	 K 	   660.31	   353.21	  6
 12	 S 	   703.83	   289.16	  5
 13	 L 	   760.37	   245.65	  4
 14	 E 	   824.89	   189.11	  3
 15	 T 	   875.42	   124.58	  2
 16	 K 	        -  	    74.06	  1  _
```

**97. IPI00176776 Uncharacterized protein C11orf85**

| Sequence | Charge | M+H+ | XCorr | DeltaCn |
| --- | --- | --- | --- | --- |
| K.WFHENLSPGKPISDSPLGLVPVEKK.A | 3 | 2776.182 | 4.054 | 0.222 |

+1 charge

```
		      B   	       Y
  1	 W 	   187.09	        -  	 25
  2	 F 	   334.16	  2588.41	 24
  3	 H 	   471.21	  2441.34	 23
  4	 E 	   600.26	  2304.28	 22
  5	 N 	   714.30	  2175.24	 21
  6	 L 	   827.38	  2061.20	 20
  7	 S 	   914.42	  1948.11	 19
  8	 P 	  1011.47	  1861.08	 18
  9	 G 	  1068.49	  1764.03	 17
 10	 K 	  1196.58	  1707.01	 16
 11	 P 	  1293.64	  1578.91	 15
 12	 I 	  1406.72	  1481.86	 14
 13	 S 	  1493.75	  1368.77	 13
 14	 D 	  1608.78	  1281.74	 12
 15	 S 	  1695.81	  1166.71	 11
 16	 P 	  1792.87	  1079.68	 10
 17	 L 	  1905.95	   982.63	  9
 18	 G 	  1962.97	   869.55	  8
 19	 L 	  2076.05	   812.52	  7
 20	 V 	  2175.12	   699.44	  6
 21	 P 	  2272.18	   600.37	  5
 22	 V 	  2371.24	   503.32	  4
 23	 E 	  2500.29	   404.25	  3
 24	 K 	  2628.38	   275.21	  2
 25	 K 	        -  	   147.11	  1  _


+2 charge
		      B   	       Y
  1	 W 	    94.05	        -  	 25
  2	 F 	   167.58	  1294.71	 24
  3	 H 	   236.11	  1221.17	 23
  4	 E 	   300.63	  1152.64	 22
  5	 N 	   357.65	  1088.12	 21
  6	 L 	   414.20	  1031.10	 20
  7	 S 	   457.71	   974.56	 19
  8	 P 	   506.24	   931.04	 18
  9	 G 	   534.75	   882.52	 17
 10	 K 	   598.80	   854.01	 16
 11	 P 	   647.32	   789.96	 15
 12	 I 	   703.86	   741.43	 14
 13	 S 	   747.38	   684.89	 13
 14	 D 	   804.89	   641.37	 12
 15	 S 	   848.41	   583.86	 11
 16	 P 	   896.94	   540.34	 10
 17	 L 	   953.48	   491.82	  9
 18	 G 	   981.99	   435.28	  8
 19	 L 	  1038.53	   406.77	  7
 20	 V 	  1088.07	   350.22	  6
 21	 P 	  1136.59	   300.69	  5
 22	 V 	  1186.13	   252.16	  4
 23	 E 	  1250.65	   202.63	  3
 24	 K 	  1314.69	   138.11	  2
 25	 K 	        -  	    74.06	  1  _


+3 charge
		      B   	       Y
  1	 W 	    63.03	        -  	 25
  2	 F 	   112.06	   863.47	 24
  3	 H 	   157.74	   814.45	 23
  4	 E 	   200.76	   768.77	 22
  5	 N 	   238.77	   725.75	 21
  6	 L 	   276.47	   687.74	 20
  7	 S 	   305.48	   650.04	 19
  8	 P 	   337.83	   621.03	 18
  9	 G 	   356.83	   588.68	 17
 10	 K 	   399.53	   569.67	 16
 11	 P 	   431.88	   526.97	 15
 12	 I 	   469.58	   494.62	 14
 13	 S 	   498.59	   456.93	 13
 14	 D 	   536.93	   427.92	 12
 15	 S 	   565.94	   389.58	 11
 16	 P 	   598.29	   360.57	 10
 17	 L 	   635.99	   328.21	  9
 18	 G 	   655.00	   290.52	  8
 19	 L 	   692.69	   271.51	  7
 20	 V 	   725.71	   233.82	  6
 21	 P 	   758.06	   200.80	  5
 22	 V 	   791.09	   168.44	  4
 23	 E 	   834.10	   135.42	  3
 24	 K 	   876.80	    92.41	  2
 25	 K 	        -  	    49.71	  1  _
```

**98. IPI00005732 Isoform 1 of Activin receptor type-1B**

| Sequence | Charge | M+H+ | XCorr | DeltaCn |
| --- | --- | --- | --- | --- |
| R.TCIPKVELVPAGKPFYCLSSEDLR.N | 3 | 2781.184 | 4.746 | 0.572 |

+1 charge

```
		      B   	       Y
  1	 T 	   102.05	        -  	 24
  2	 C 	   262.22	  2678.64	 23
  3	 I 	   375.30	  2518.47	 22
  4	 P 	   472.36	  2405.39	 21
  5	 K 	   600.45	  2308.34	 20
  6	 V 	   699.52	  2180.24	 19
  7	 E 	   828.56	  2081.17	 18
  8	 L 	   941.65	  1952.13	 17
  9	 V 	  1040.72	  1839.05	 16
 10	 P 	  1137.77	  1739.98	 15
 11	 A 	  1208.81	  1642.92	 14
 12	 G 	  1265.83	  1571.89	 13
 13	 K 	  1393.92	  1514.87	 12
 14	 P 	  1490.97	  1386.77	 11
 15	 F 	  1638.04	  1289.72	 10
 16	 Y 	  1801.11	  1142.65	  9
 17	 C 	  1961.27	   979.59	  8
 18	 L 	  2074.36	   819.42	  7
 19	 S 	  2161.39	   706.34	  6
 20	 S 	  2248.42	   619.30	  5
 21	 E 	  2377.46	   532.27	  4
 22	 D 	  2492.49	   403.23	  3
 23	 L 	  2605.57	   288.20	  2
 24	 R 	        -  	   175.12	  1  _


+2 charge
		      B   	       Y
  1	 T 	    51.53	        -  	 24
  2	 C 	   131.61	  1339.82	 23
  3	 I 	   188.16	  1259.74	 22
  4	 P 	   236.68	  1203.20	 21
  5	 K 	   300.73	  1154.67	 20
  6	 V 	   350.26	  1090.62	 19
  7	 E 	   414.79	  1041.09	 18
  8	 L 	   471.33	   976.57	 17
  9	 V 	   520.86	   920.03	 16
 10	 P 	   569.39	   870.49	 15
 11	 A 	   604.91	   821.97	 14
 12	 G 	   633.42	   786.45	 13
 13	 K 	   697.46	   757.94	 12
 14	 P 	   745.99	   693.89	 11
 15	 F 	   819.53	   645.36	 10
 16	 Y 	   901.06	   571.83	  9
 17	 C 	   981.14	   490.30	  8
 18	 L 	  1037.68	   410.21	  7
 19	 S 	  1081.20	   353.67	  6
 20	 S 	  1124.71	   310.16	  5
 21	 E 	  1189.23	   266.64	  4
 22	 D 	  1246.75	   202.12	  3
 23	 L 	  1303.29	   144.61	  2
 24	 R 	        -  	    88.06	  1  _


+3 charge
		      B   	       Y
  1	 T 	    34.69	        -  	 24
  2	 C 	    88.08	   893.55	 23
  3	 I 	   125.77	   840.16	 22
  4	 P 	   158.12	   802.47	 21
  5	 K 	   200.82	   770.12	 20
  6	 V 	   233.84	   727.42	 19
  7	 E 	   276.86	   694.40	 18
  8	 L 	   314.55	   651.38	 17
  9	 V 	   347.58	   613.69	 16
 10	 P 	   379.93	   580.66	 15
 11	 A 	   403.61	   548.31	 14
 12	 G 	   422.61	   524.63	 13
 13	 K 	   465.31	   505.63	 12
 14	 P 	   497.66	   462.93	 11
 15	 F 	   546.69	   430.58	 10
 16	 Y 	   601.04	   381.55	  9
 17	 C 	   654.43	   327.20	  8
 18	 L 	   692.12	   273.81	  7
 19	 S 	   721.13	   236.12	  6
 20	 S 	   750.14	   207.11	  5
 21	 E 	   793.16	   178.10	  4
 22	 D 	   831.50	   135.08	  3
 23	 L 	   869.20	    96.74	  2
 24	 R 	        -  	    59.04	  1  _
```

**99. IPI00009793 Complement C1r subcomponent-like protein**

| Sequence | Charge | M+H+ | XCorr | DeltaCn |
| --- | --- | --- | --- | --- |
| K.LGNFPWQAFTSIHGR.G | 2 | 1731.938 | 3.224 | 0.585 |

+1 charge

```
		      B   	       Y
  1	 L 	   114.09	        -  	 15
  2	 G 	   171.11	  1617.79	 14
  3	 N 	   285.16	  1560.77	 13
  4	 F 	   432.22	  1446.73	 12
  5	 P 	   529.28	  1299.66	 11
  6	 W 	   715.36	  1202.61	 10
  7	 Q 	   843.41	  1016.53	  9
  8	 A 	   914.45	   888.47	  8
  9	 F 	  1061.52	   817.43	  7
 10	 T 	  1162.57	   670.36	  6
 11	 S 	  1249.60	   569.32	  5
 12	 I 	  1362.68	   482.28	  4
 13	 H 	  1499.74	   369.20	  3
 14	 G 	  1556.76	   232.14	  2
 15	 R 	        -  	   175.12	  1  _
+2 charge
		      B   	       Y
  1	 L 	    57.55	        -  	 15
  2	 G 	    86.06	   809.40	 14
  3	 N 	   143.08	   780.89	 13
  4	 F 	   216.62	   723.87	 12
  5	 P 	   265.14	   650.33	 11
  6	 W 	   358.18	   601.81	 10
  7	 Q 	   422.21	   508.77	  9
  8	 A 	   457.73	   444.74	  8
  9	 F 	   531.26	   409.22	  7
 10	 T 	   581.79	   335.69	  6
 11	 S 	   625.30	   285.16	  5
 12	 I 	   681.85	   241.65	  4
 13	 H 	   750.38	   185.10	  3
 14	 G 	   778.89	   116.57	  2
 15	 R 	        -  	    88.06	  1  _
```

**100. IPI00940960 Epididymal secretory protein E1**

| Sequence | Charge | M+H+ | XCorr | DeltaCn |
| --- | --- | --- | --- | --- |
| K.AVVHGILMGVPVPFPIPEPDGCK.S | 3 | 2430.887 | 3.435 | 0.432 |

+1 charge

```
		      B   	       Y
  1	 A 	    72.04	        -  	 23
  2	 V 	   171.11	  2358.37	 22
  3	 V 	   270.18	  2259.30	 21
  4	 H 	   407.24	  2160.23	 20
  5	 G 	   464.26	  2023.17	 19
  6	 I 	   577.35	  1966.15	 18
  7	 L 	   690.43	  1853.07	 17
  8	 M 	   821.47	  1739.98	 16
  9	 G 	   878.49	  1608.94	 15
 10	 V 	   977.56	  1551.92	 14
 11	 P 	  1074.61	  1452.85	 13
 12	 V 	  1173.68	  1355.80	 12
 13	 P 	  1270.73	  1256.73	 11
 14	 F 	  1417.80	  1159.68	 10
 15	 P 	  1514.86	  1012.61	  9
 16	 I 	  1627.94	   915.56	  8
 17	 P 	  1724.99	   802.47	  7
 18	 E 	  1854.03	   705.42	  6
 19	 P 	  1951.09	   576.38	  5
 20	 D 	  2066.11	   479.33	  4
 21	 G 	  2123.14	   364.30	  3
 22	 C 	  2283.30	   307.28	  2
 23	 K 	        -  	   147.11	  1  _


+2 charge
		      B   	       Y
  1	 A 	    36.53	        -  	 23
  2	 V 	    86.06	  1179.69	 22
  3	 V 	   135.59	  1130.15	 21
  4	 H 	   204.12	  1080.62	 20
  5	 G 	   232.63	  1012.09	 19
  6	 I 	   289.18	   983.58	 18
  7	 L 	   345.72	   927.04	 17
  8	 M 	   411.24	   870.50	 16
  9	 G 	   439.75	   804.98	 15
 10	 V 	   489.28	   776.46	 14
 11	 P 	   537.81	   726.93	 13
 12	 V 	   587.34	   678.40	 12
 13	 P 	   635.87	   628.87	 11
 14	 F 	   709.40	   580.34	 10
 15	 P 	   757.93	   506.81	  9
 16	 I 	   814.47	   458.28	  8
 17	 P 	   863.00	   401.74	  7
 18	 E 	   927.52	   353.21	  6
 19	 P 	   976.05	   288.69	  5
 20	 D 	  1033.56	   240.17	  4
 21	 G 	  1062.07	   182.65	  3
 22	 C 	  1142.15	   154.14	  2
 23	 K 	        -  	    74.06	  1  _


+3 charge
		      B   	       Y
  1	 A 	    24.69	        -  	 23
  2	 V 	    57.71	   786.79	 22
  3	 V 	    90.73	   753.77	 21
  4	 H 	   136.42	   720.75	 20
  5	 G 	   155.43	   675.06	 19
  6	 I 	   193.12	   656.06	 18
  7	 L 	   230.81	   618.36	 17
  8	 M 	   274.49	   580.67	 16
  9	 G 	   293.50	   536.99	 15
 10	 V 	   326.52	   517.98	 14
 11	 P 	   358.88	   484.96	 13
 12	 V 	   391.90	   452.61	 12
 13	 P 	   424.25	   419.58	 11
 14	 F 	   473.27	   387.23	 10
 15	 P 	   505.62	   338.21	  9
 16	 I 	   543.32	   305.86	  8
 17	 P 	   575.67	   268.16	  7
 18	 E 	   618.68	   235.81	  6
 19	 P 	   651.03	   192.80	  5
 20	 D 	   689.38	   160.45	  4
 21	 G 	   708.38	   122.10	  3
 22	 C 	   761.77	   103.10	  2
 23	 K 	        -  	    49.71	  1  _
```

**101. IPI00293665 Keratin, type II cytoskeletal 6B**

| Sequence | Charge | M+H+ | XCorr | DeltaCn |
| --- | --- | --- | --- | --- |
| R.ISIGGGSCAISGGYGSR.A | 2 | 1599.721 | 3.842 | 0.501 |

+1 charge

```
		      B   	       Y
  1	 I 	   114.09	        -  	 17
  2	 S 	   201.12	  1485.81	 16
  3	 I 	   314.21	  1398.78	 15
  4	 G 	   371.23	  1285.69	 14
  5	 G 	   428.25	  1228.67	 13
  6	 G 	   485.27	  1171.65	 12
  7	 S 	   572.30	  1114.63	 11
  8	 C 	   732.47	  1027.60	 10
  9	 A 	   803.51	   867.43	  9
 10	 I 	   916.59	   796.39	  8
 11	 S 	  1003.62	   683.31	  7
 12	 G 	  1060.64	   596.28	  6
 13	 G 	  1117.67	   539.26	  5
 14	 Y 	  1280.73	   482.24	  4
 15	 G 	  1337.75	   319.17	  3
 16	 S 	  1424.78	   262.15	  2
 17	 R 	        -  	   175.12	  1  _
+2 charge
		      B   	       Y
  1	 I 	    57.55	        -  	 17
  2	 S 	   101.07	   743.41	 16
  3	 I 	   157.61	   699.89	 15
  4	 G 	   186.12	   643.35	 14
  5	 G 	   214.63	   614.84	 13
  6	 G 	   243.14	   586.33	 12
  7	 S 	   286.66	   557.82	 11
  8	 C 	   366.74	   514.30	 10
  9	 A 	   402.26	   434.22	  9
 10	 I 	   458.80	   398.70	  8
 11	 S 	   502.31	   342.16	  7
 12	 G 	   530.83	   298.64	  6
 13	 G 	   559.34	   270.13	  5
 14	 Y 	   640.87	   241.62	  4
 15	 G 	   669.38	   160.09	  3
 16	 S 	   712.89	   131.58	  2
 17	 R 	        -  	    88.06	  1  _
```

**102. IPI00382440 Ig lambda chain V-IV region Hil**

| Sequence | Charge | M+H+ | XCorr | DeltaCn |
| --- | --- | --- | --- | --- |
| - .SYELTQPPSVSVSPGQTAR.I | 2 | 2005.175 | 3.397 | 0.431 |

+1 charge

```
		      B   	       Y
  1	 S 	    88.04	        -  	 19
  2	 Y 	   251.10	  1916.97	 18
  3	 E 	   380.15	  1753.91	 17
  4	 L 	   493.23	  1624.87	 16
  5	 T 	   594.28	  1511.78	 15
  6	 Q 	   722.34	  1410.73	 14
  7	 P 	   819.39	  1282.67	 13
  8	 P 	   916.44	  1185.62	 12
  9	 S 	  1003.47	  1088.57	 11
 10	 V 	  1102.54	  1001.54	 10
 11	 S 	  1189.57	   902.47	  9
 12	 V 	  1288.64	   815.44	  8
 13	 S 	  1375.67	   716.37	  7
 14	 P 	  1472.73	   629.34	  6
 15	 G 	  1529.75	   532.28	  5
 16	 Q 	  1657.81	   475.26	  4
 17	 T 	  1758.85	   347.20	  3
 18	 A 	  1829.89	   246.16	  2
 19	 R 	        -  	   175.12	  1  _
+2 charge
		      B   	       Y
  1	 S 	    44.52	        -  	 19
  2	 Y 	   126.05	   958.99	 18
  3	 E 	   190.58	   877.46	 17
  4	 L 	   247.12	   812.94	 16
  5	 T 	   297.64	   756.39	 15
  6	 Q 	   361.67	   705.87	 14
  7	 P 	   410.20	   641.84	 13
  8	 P 	   458.72	   593.31	 12
  9	 S 	   502.24	   544.79	 11
 10	 V 	   551.77	   501.27	 10
 11	 S 	   595.29	   451.74	  9
 12	 V 	   644.82	   408.22	  8
 13	 S 	   688.34	   358.69	  7
 14	 P 	   736.87	   315.17	  6
 15	 G 	   765.38	   266.65	  5
 16	 Q 	   829.41	   238.13	  4
 17	 T 	   879.93	   174.11	  3
 18	 A 	   915.45	   123.58	  2
 19	 R 	        -  	    88.06	  1  _
```

**103. IPI00643114 70 kDa protein**

| Sequence | Charge | M+H+ | XCorr | DeltaCn |
| --- | --- | --- | --- | --- |
| R.LLKARAPPTPPHPPGPR.I | 3 | 1803.147 | 3.349 | 0.214 |

+1 charge

```
		      B   	       Y
  1	 L 	   114.09	        -  	 17
  2	 L 	   227.18	  1688.97	 16
  3	 K 	   355.27	  1575.89	 15
  4	 A 	   426.31	  1447.79	 14
  5	 R 	   582.41	  1376.75	 13
  6	 A 	   653.45	  1220.65	 12
  7	 P 	   750.50	  1149.62	 11
  8	 P 	   847.55	  1052.56	 10
  9	 T 	   948.60	   955.51	  9
 10	 P 	  1045.65	   854.46	  8
 11	 P 	  1142.70	   757.41	  7
 12	 H 	  1279.76	   660.36	  6
 13	 P 	  1376.82	   523.30	  5
 14	 P 	  1473.87	   426.25	  4
 15	 G 	  1530.89	   329.19	  3
 16	 P 	  1627.94	   272.17	  2
 17	 R 	        -  	   175.12	  1  _
+2 charge
		      B   	       Y
  1	 L 	    57.55	        -  	 17
  2	 L 	   114.09	   844.99	 16
  3	 K 	   178.14	   788.45	 15
  4	 A 	   213.66	   724.40	 14
  5	 R 	   291.71	   688.88	 13
  6	 A 	   327.23	   610.83	 12
  7	 P 	   375.75	   575.31	 11
  8	 P 	   424.28	   526.79	 10
  9	 T 	   474.80	   478.26	  9
 10	 P 	   523.33	   427.74	  8
 11	 P 	   571.86	   379.21	  7
 12	 H 	   640.39	   330.68	  6
 13	 P 	   688.91	   262.15	  5
 14	 P 	   737.44	   213.63	  4
 15	 G 	   765.95	   165.10	  3
 16	 P 	   814.48	   136.59	  2
 17	 R 	        -  	    88.06	  1  _


+3 charge
		      B   	       Y
  1	 L 	    38.70	        -  	 17
  2	 L 	    76.40	   563.66	 16
  3	 K 	   119.09	   525.97	 15
  4	 A 	   142.77	   483.27	 14
  5	 R 	   194.81	   459.59	 13
  6	 A 	   218.49	   407.56	 12
  7	 P 	   250.84	   383.88	 11
  8	 P 	   283.19	   351.53	 10
  9	 T 	   316.87	   319.18	  9
 10	 P 	   349.22	   285.49	  8
 11	 P 	   381.57	   253.14	  7
 12	 H 	   427.26	   220.79	  6
 13	 P 	   459.61	   175.10	  5
 14	 P 	   491.96	   142.75	  4
 15	 G 	   510.97	   110.40	  3
 16	 P 	   543.32	    91.40	  2
 17	 R 	        -  	    59.04	  1  _
```

**104. IPI00153049 Isoform 2 of Matrix-remodeling-associated protein 8**

| Sequence | Charge | M+H+ | XCorr | DeltaCn |
| --- | --- | --- | --- | --- |
| R.LLDLYSAGEQR.V | 2 | 1265.397 | 3.606 | 0.335 |

+1 charge

```
		      B   	       Y
  1	 L 	   114.09	        -  	 11
  2	 L 	   227.18	  1151.57	 10
  3	 D 	   342.20	  1038.49	  9
  4	 L 	   455.29	   923.46	  8
  5	 Y 	   618.35	   810.37	  7
  6	 S 	   705.38	   647.31	  6
  7	 A 	   776.42	   560.28	  5
  8	 G 	   833.44	   489.24	  4
  9	 E 	   962.48	   432.22	  3
 10	 Q 	  1090.54	   303.18	  2
 11	 R 	        -  	   175.12	  1  _
+2 charge
		      B   	       Y
  1	 L 	    57.55	        -  	 11
  2	 L 	   114.09	   576.29	 10
  3	 D 	   171.60	   519.75	  9
  4	 L 	   228.15	   462.23	  8
  5	 Y 	   309.68	   405.69	  7
  6	 S 	   353.19	   324.16	  6
  7	 A 	   388.71	   280.64	  5
  8	 G 	   417.22	   245.12	  4
  9	 E 	   481.75	   216.61	  3
 10	 Q 	   545.77	   152.09	  2
 11	 R 	        -  	    88.06	  1  _
```

**105. IPI00102300 Isoform 3 of Platelet glycoprotein VI**

| Sequence | Charge | M+H+ | XCorr | DeltaCn |
| --- | --- | --- | --- | --- |
| R.ASFPIITVTAAHSGTYR.C | 2 | 1793.016 | 3.721 | 0.578 |

+1 charge

```
		      B   	       Y
  1	 A 	    72.04	        -  	 17
  2	 S 	   159.08	  1720.90	 16
  3	 F 	   306.14	  1633.87	 15
  4	 P 	   403.20	  1486.80	 14
  5	 I 	   516.28	  1389.75	 13
  6	 I 	   629.37	  1276.66	 12
  7	 T 	   730.41	  1163.58	 11
  8	 V 	   829.48	  1062.53	 10
  9	 T 	   930.53	   963.46	  9
 10	 A 	  1001.57	   862.42	  8
 11	 A 	  1072.60	   791.38	  7
 12	 H 	  1209.66	   720.34	  6
 13	 S 	  1296.69	   583.28	  5
 14	 G 	  1353.72	   496.25	  4
 15	 T 	  1454.76	   439.23	  3
 16	 Y 	  1617.83	   338.18	  2
 17	 R 	        -  	   175.12	  1  _
+2 charge
		      B   	       Y
  1	 A 	    36.53	        -  	 17
  2	 S 	    80.04	   860.95	 16
  3	 F 	   153.58	   817.44	 15
  4	 P 	   202.10	   743.90	 14
  5	 I 	   258.64	   695.38	 13
  6	 I 	   315.19	   638.84	 12
  7	 T 	   365.71	   582.29	 11
  8	 V 	   415.24	   531.77	 10
  9	 T 	   465.77	   482.24	  9
 10	 A 	   501.29	   431.71	  8
 11	 A 	   536.81	   396.19	  7
 12	 H 	   605.33	   360.67	  6
 13	 S 	   648.85	   292.15	  5
 14	 G 	   677.36	   248.63	  4
 15	 T 	   727.89	   220.12	  3
 16	 Y 	   809.42	   169.59	  2
 17	 R 	        -  	    88.06	  1  _
```

**106. IPI00023648 Immunoglobulin superfamily containing leucine-rich repeat protein**

| Sequence | Charge | M+H+ | XCorr | DeltaCn |
| --- | --- | --- | --- | --- |
| R.TVAAGALASLSHLK.S | 2 | 1339.566 | 3.432 | 0.49 |

+1 charge

```
		      B   	       Y
  1	 T 	   102.05	        -  	 14
  2	 V 	   201.12	  1237.73	 13
  3	 A 	   272.16	  1138.66	 12
  4	 A 	   343.20	  1067.62	 11
  5	 G 	   400.22	   996.58	 10
  6	 A 	   471.26	   939.56	  9
  7	 L 	   584.34	   868.53	  8
  8	 A 	   655.38	   755.44	  7
  9	 S 	   742.41	   684.40	  6
 10	 L 	   855.49	   597.37	  5
 11	 S 	   942.53	   484.29	  4
 12	 H 	  1079.58	   397.26	  3
 13	 L 	  1192.67	   260.20	  2
 14	 K 	        -  	   147.11	  1  _
+2 charge
		      B   	       Y
  1	 T 	    51.53	        -  	 14
  2	 V 	   101.07	   619.37	 13
  3	 A 	   136.58	   569.83	 12
  4	 A 	   172.10	   534.31	 11
  5	 G 	   200.61	   498.80	 10
  6	 A 	   236.13	   470.28	  9
  7	 L 	   292.67	   434.77	  8
  8	 A 	   328.19	   378.22	  7
  9	 S 	   371.71	   342.71	  6
 10	 L 	   428.25	   299.19	  5
 11	 S 	   471.77	   242.65	  4
 12	 H 	   540.30	   199.13	  3
 13	 L 	   596.84	   130.60	  2
 14	 K 	        -  	    74.06	  1  _
```

**107. IPI00816799 Rheumatoid factor D5 light chain (Fragment)**

| Sequence | Charge | M+H+ | XCorr | DeltaCn |
| --- | --- | --- | --- | --- |
| - .EIVLTQSPATLSLSPGER.A | 2 | 1899.135 | 4.869 | 0.468 |

+1 charge

```
		      B   	       Y
  1	 E 	   130.05	        -  	 18
  2	 I 	   243.13	  1768.98	 17
  3	 V 	   342.20	  1655.90	 16
  4	 L 	   455.29	  1556.83	 15
  5	 T 	   556.33	  1443.74	 14
  6	 Q 	   684.39	  1342.70	 13
  7	 S 	   771.42	  1214.64	 12
  8	 P 	   868.48	  1127.61	 11
  9	 A 	   939.51	  1030.55	 10
 10	 T 	  1040.56	   959.52	  9
 11	 L 	  1153.65	   858.47	  8
 12	 S 	  1240.68	   745.38	  7
 13	 L 	  1353.76	   658.35	  6
 14	 S 	  1440.79	   545.27	  5
 15	 P 	  1537.85	   458.24	  4
 16	 G 	  1594.87	   361.18	  3
 17	 E 	  1723.91	   304.16	  2
 18	 R 	        -  	   175.12	  1  _
+2 charge
		      B   	       Y
  1	 E 	    65.53	        -  	 18
  2	 I 	   122.07	   884.99	 17
  3	 V 	   171.60	   828.45	 16
  4	 L 	   228.15	   778.92	 15
  5	 T 	   278.67	   722.38	 14
  6	 Q 	   342.70	   671.85	 13
  7	 S 	   386.22	   607.82	 12
  8	 P 	   434.74	   564.31	 11
  9	 A 	   470.26	   515.78	 10
 10	 T 	   520.78	   480.26	  9
 11	 L 	   577.33	   429.74	  8
 12	 S 	   620.84	   373.20	  7
 13	 L 	   677.38	   329.68	  6
 14	 S 	   720.90	   273.14	  5
 15	 P 	   769.43	   229.62	  4
 16	 G 	   797.94	   181.10	  3
 17	 E 	   862.46	   152.58	  2
 18	 R 	        -  	    88.06	  1  _
```

**108. IPI00003091 Isoform 1 of Protein phosphatase 1 regulatory subunit 3A**

| Sequence | Charge | M+H+ | XCorr | DeltaCn |
| --- | --- | --- | --- | --- |
| K.EQEPEPVKPWKEVPNR.Q | 2 | 1963.183 | 3.454 | 0.248 |

+1 charge

```
		      B   	       Y
  1	 E 	   130.05	        -  	 16
  2	 Q 	   258.11	  1832.97	 15
  3	 E 	   387.15	  1704.91	 14
  4	 P 	   484.20	  1575.86	 13
  5	 E 	   613.25	  1478.81	 12
  6	 P 	   710.30	  1349.77	 11
  7	 V 	   809.37	  1252.72	 10
  8	 K 	   937.46	  1153.65	  9
  9	 P 	  1034.52	  1025.55	  8
 10	 W 	  1220.59	   928.50	  7
 11	 K 	  1348.69	   742.42	  6
 12	 E 	  1477.73	   614.33	  5
 13	 V 	  1576.80	   485.28	  4
 14	 P 	  1673.85	   386.21	  3
 15	 N 	  1787.90	   289.16	  2
 16	 R 	        -  	   175.12	  1  _
+2 charge
		      B   	       Y
  1	 E 	    65.53	        -  	 16
  2	 Q 	   129.56	   916.99	 15
  3	 E 	   194.08	   852.96	 14
  4	 P 	   242.61	   788.44	 13
  5	 E 	   307.13	   739.91	 12
  6	 P 	   355.65	   675.39	 11
  7	 V 	   405.19	   626.86	 10
  8	 K 	   469.23	   577.33	  9
  9	 P 	   517.76	   513.28	  8
 10	 W 	   610.80	   464.75	  7
 11	 K 	   674.85	   371.71	  6
 12	 E 	   739.37	   307.67	  5
 13	 V 	   788.90	   243.15	  4
 14	 P 	   837.43	   193.61	  3
 15	 N 	   894.45	   145.08	  2
 16	 R 	        -  	    88.06	  1  _
```

**109. IPI00029235 Insulin-like growth factor-binding protein 6**

| Sequence | Charge | M+H+ | XCorr | DeltaCn |
| --- | --- | --- | --- | --- |
| R.HLDSVLQQLQTEVYR.G | 2 | 1830.035 | 3.223 | 0.407 |

+1 charge

```
		      B   	       Y
  1	 H 	   138.07	        -  	 15
  2	 L 	   251.15	  1691.90	 14
  3	 D 	   366.18	  1578.81	 13
  4	 S 	   453.21	  1463.79	 12
  5	 V 	   552.28	  1376.75	 11
  6	 L 	   665.36	  1277.68	 10
  7	 Q 	   793.42	  1164.60	  9
  8	 Q 	   921.48	  1036.54	  8
  9	 L 	  1034.56	   908.48	  7
 10	 Q 	  1162.62	   795.40	  6
 11	 T 	  1263.67	   667.34	  5
 12	 E 	  1392.71	   566.29	  4
 13	 V 	  1491.78	   437.25	  3
 14	 Y 	  1654.84	   338.18	  2
 15	 R 	        -  	   175.12	  1  _
+2 charge
		      B   	       Y
  1	 H 	    69.54	        -  	 15
  2	 L 	   126.08	   846.45	 14
  3	 D 	   183.59	   789.91	 13
  4	 S 	   227.11	   732.40	 12
  5	 V 	   276.64	   688.88	 11
  6	 L 	   333.18	   639.35	 10
  7	 Q 	   397.21	   582.80	  9
  8	 Q 	   461.24	   518.77	  8
  9	 L 	   517.79	   454.75	  7
 10	 Q 	   581.81	   398.20	  6
 11	 T 	   632.34	   334.17	  5
 12	 E 	   696.86	   283.65	  4
 13	 V 	   746.39	   219.13	  3
 14	 Y 	   827.93	   169.59	  2
 15	 R 	        -  	    88.06	  1  _
```

**110. IPI00025846 Isoform 2A of Desmocollin-2**

| Sequence | Charge | M+H+ | XCorr | DeltaCn |
| --- | --- | --- | --- | --- |
| R.LSYQNDPPFGSYVVPITVR.D | 2 | 2153.422 | 3.188 | 0.434 |

+1 charge

```
		      B   	       Y
  1	 L 	   114.09	        -  	 19
  2	 S 	   201.12	  2039.02	 18
  3	 Y 	   364.19	  1951.99	 17
  4	 Q 	   492.25	  1788.93	 16
  5	 N 	   606.29	  1660.87	 15
  6	 D 	   721.32	  1546.83	 14
  7	 P 	   818.37	  1431.80	 13
  8	 P 	   915.42	  1334.75	 12
  9	 F 	  1062.49	  1237.69	 11
 10	 G 	  1119.51	  1090.63	 10
 11	 S 	  1206.54	  1033.60	  9
 12	 Y 	  1369.61	   946.57	  8
 13	 V 	  1468.67	   783.51	  7
 14	 V 	  1567.74	   684.44	  6
 15	 P 	  1664.80	   585.37	  5
 16	 I 	  1777.88	   488.32	  4
 17	 T 	  1878.93	   375.24	  3
 18	 V 	  1978.00	   274.19	  2
 19	 R 	        -  	   175.12	  1  _
+2 charge
		      B   	       Y
  1	 L 	    57.55	        -  	 19
  2	 S 	   101.07	  1020.02	 18
  3	 Y 	   182.60	   976.50	 17
  4	 Q 	   246.63	   894.97	 16
  5	 N 	   303.65	   830.94	 15
  6	 D 	   361.16	   773.92	 14
  7	 P 	   409.69	   716.40	 13
  8	 P 	   458.21	   667.88	 12
  9	 F 	   531.75	   619.35	 11
 10	 G 	   560.26	   545.82	 10
 11	 S 	   603.77	   517.31	  9
 12	 Y 	   685.31	   473.79	  8
 13	 V 	   734.84	   392.26	  7
 14	 V 	   784.38	   342.72	  6
 15	 P 	   832.90	   293.19	  5
 16	 I 	   889.44	   244.66	  4
 17	 T 	   939.97	   188.12	  3
 18	 V 	   989.50	   137.60	  2
 19	 R 	        -  	    88.06	  1  _
```

**111. IPI00027462 Protein S100-A9**

| Sequence | Charge | M+H+ | XCorr | DeltaCn |
| --- | --- | --- | --- | --- |
| R.NIETIINTFHQYSVK.L | 2 | 1808.027 | 4.649 | 0.528 |

+1 charge

```
		      B   	       Y
  1	 N 	   115.05	        -  	 15
  2	 I 	   228.13	  1692.90	 14
  3	 E 	   357.18	  1579.81	 13
  4	 T 	   458.22	  1450.77	 12
  5	 I 	   571.31	  1349.72	 11
  6	 I 	   684.39	  1236.64	 10
  7	 N 	   798.44	  1123.55	  9
  8	 T 	   899.48	  1009.51	  8
  9	 F 	  1046.55	   908.46	  7
 10	 H 	  1183.61	   761.39	  6
 11	 Q 	  1311.67	   624.34	  5
 12	 Y 	  1474.73	   496.28	  4
 13	 S 	  1561.76	   333.21	  3
 14	 V 	  1660.83	   246.18	  2
 15	 K 	        -  	   147.11	  1  _
+2 charge
		      B   	       Y
  1	 N 	    58.03	        -  	 15
  2	 I 	   114.57	   846.95	 14
  3	 E 	   179.09	   790.41	 13
  4	 T 	   229.62	   725.89	 12
  5	 I 	   286.16	   675.36	 11
  6	 I 	   342.70	   618.82	 10
  7	 N 	   399.72	   562.28	  9
  8	 T 	   450.25	   505.26	  8
  9	 F 	   523.78	   454.73	  7
 10	 H 	   592.31	   381.20	  6
 11	 Q 	   656.34	   312.67	  5
 12	 Y 	   737.87	   248.64	  4
 13	 S 	   781.39	   167.11	  3
 14	 V 	   830.92	   123.59	  2
 15	 K 	        -  	    74.06	  1  _
```

**112. IPI00000828 Proenkephalin-A**

| Sequence | Charge | M+H+ | XCorr | DeltaCn |
| --- | --- | --- | --- | --- |
| K.DAEEDDSLANSSDLLKELLETGDNRER.S | 3 | 3036.123 | 6.224 | 0.589 |

+1 charge

```
		      B   	       Y
  1	 D 	   116.03	        -  	 27
  2	 A 	   187.07	  2919.38	 26
  3	 E 	   316.11	  2848.34	 25
  4	 E 	   445.16	  2719.30	 24
  5	 D 	   560.18	  2590.26	 23
  6	 D 	   675.21	  2475.23	 22
  7	 S 	   762.24	  2360.21	 21
  8	 L 	   875.33	  2273.17	 20
  9	 A 	   946.36	  2160.09	 19
 10	 N 	  1060.41	  2089.05	 18
 11	 S 	  1147.44	  1975.01	 17
 12	 S 	  1234.47	  1887.98	 16
 13	 D 	  1349.50	  1800.95	 15
 14	 L 	  1462.58	  1685.92	 14
 15	 L 	  1575.67	  1572.83	 13
 16	 K 	  1703.76	  1459.75	 12
 17	 E 	  1832.80	  1331.65	 11
 18	 L 	  1945.89	  1202.61	 10
 19	 L 	  2058.97	  1089.53	  9
 20	 E 	  2188.01	   976.44	  8
 21	 T 	  2289.06	   847.40	  7
 22	 G 	  2346.08	   746.35	  6
 23	 D 	  2461.11	   689.33	  5
 24	 N 	  2575.15	   574.31	  4
 25	 R 	  2731.25	   460.26	  3
 26	 E 	  2860.30	   304.16	  2
 27	 R 	        -  	   175.12	  1  _


+2 charge
		      B   	       Y
  1	 D 	    58.52	        -  	 27
  2	 A 	    94.04	  1460.19	 26
  3	 E 	   158.56	  1424.68	 25
  4	 E 	   223.08	  1360.15	 24
  5	 D 	   280.60	  1295.63	 23
  6	 D 	   338.11	  1238.12	 22
  7	 S 	   381.62	  1180.61	 21
  8	 L 	   438.17	  1137.09	 20
  9	 A 	   473.69	  1080.55	 19
 10	 N 	   530.71	  1045.03	 18
 11	 S 	   574.22	   988.01	 17
 12	 S 	   617.74	   944.49	 16
 13	 D 	   675.25	   900.98	 15
 14	 L 	   731.79	   843.46	 14
 15	 L 	   788.34	   786.92	 13
 16	 K 	   852.38	   730.38	 12
 17	 E 	   916.91	   666.33	 11
 18	 L 	   973.45	   601.81	 10
 19	 L 	  1029.99	   545.27	  9
 20	 E 	  1094.51	   488.73	  8
 21	 T 	  1145.03	   424.20	  7
 22	 G 	  1173.55	   373.68	  6
 23	 D 	  1231.06	   345.17	  5
 24	 N 	  1288.08	   287.66	  4
 25	 R 	  1366.13	   230.63	  3
 26	 E 	  1430.65	   152.58	  2
 27	 R 	        -  	    88.06	  1  _


+3 charge
		      B   	       Y
  1	 D 	    39.35	        -  	 27
  2	 A 	    63.03	   973.80	 26
  3	 E 	   106.04	   950.12	 25
  4	 E 	   149.06	   907.11	 24
  5	 D 	   187.40	   864.09	 23
  6	 D 	   225.74	   825.75	 22
  7	 S 	   254.75	   787.41	 21
  8	 L 	   292.45	   758.40	 20
  9	 A 	   316.13	   720.70	 19
 10	 N 	   354.14	   697.02	 18
 11	 S 	   383.15	   659.01	 17
 12	 S 	   412.16	   630.00	 16
 13	 D 	   450.50	   600.99	 15
 14	 L 	   488.20	   562.64	 14
 15	 L 	   525.89	   524.95	 13
 16	 K 	   568.59	   487.25	 12
 17	 E 	   611.61	   444.56	 11
 18	 L 	   649.30	   401.54	 10
 19	 L 	   687.00	   363.85	  9
 20	 E 	   730.01	   326.15	  8
 21	 T 	   763.69	   283.14	  7
 22	 G 	   782.70	   249.46	  6
 23	 D 	   821.04	   230.45	  5
 24	 N 	   859.06	   192.11	  4
 25	 R 	   911.09	   154.09	  3
 26	 E 	   954.10	   102.06	  2
 27	 R 	        -  	    59.04	  1  _
```

**113. IPI00465248 Isoform alpha-enolase of Alpha-enolase**

| Sequence | Charge | M+H+ | XCorr | DeltaCn |
| --- | --- | --- | --- | --- |
| K.LAMQEFMILPVGAANFR.E | 2 | 1909.308 | 2.916 | 0.435 |

+1 charge

```
		      B   	       Y
  1	 L 	   114.09	        -  	 17
  2	 A 	   185.13	  1794.90	 16
  3	 M 	   316.17	  1723.87	 15
  4	 Q 	   444.23	  1592.83	 14
  5	 E 	   573.27	  1464.77	 13
  6	 F 	   720.34	  1335.72	 12
  7	 M 	   851.38	  1188.66	 11
  8	 I 	   964.46	  1057.62	 10
  9	 L 	  1077.55	   944.53	  9
 10	 P 	  1174.60	   831.45	  8
 11	 V 	  1273.67	   734.39	  7
 12	 G 	  1330.69	   635.33	  6
 13	 A 	  1401.73	   578.30	  5
 14	 A 	  1472.76	   507.27	  4
 15	 N 	  1586.81	   436.23	  3
 16	 F 	  1733.88	   322.19	  2
 17	 R 	        -  	   175.12	  1  _
+2 charge
		      B   	       Y
  1	 L 	    57.55	        -  	 17
  2	 A 	    93.07	   897.96	 16
  3	 M 	   158.59	   862.44	 15
  4	 Q 	   222.62	   796.92	 14
  5	 E 	   287.14	   732.89	 13
  6	 F 	   360.67	   668.37	 12
  7	 M 	   426.19	   594.83	 11
  8	 I 	   482.74	   529.31	 10
  9	 L 	   539.28	   472.77	  9
 10	 P 	   587.80	   416.23	  8
 11	 V 	   637.34	   367.70	  7
 12	 G 	   665.85	   318.17	  6
 13	 A 	   701.37	   289.66	  5
 14	 A 	   736.89	   254.14	  4
 15	 N 	   793.91	   218.62	  3
 16	 F 	   867.44	   161.60	  2
 17	 R 	        -  	    88.06	  1  _
```

**114. IPI00844026 Isoform 1 of BRICHOS domain-containing protein C16orf79**

| Sequence | Charge | M+H+ | XCorr | DeltaCn |
| --- | --- | --- | --- | --- |
| R.AVGLLLLLLLLVLAAVGIVAGGLLGSAQGPPKPR.L | 3 | 3262.062 | 3.373 | 0.341 |

+1 charge

```
		      B   	     Y
  1	 A 	    72.04	        -	     34
  2	 V 	   171.11	  3189.02	 33
  3	 G 	   228.13	  3089.95	 32
  4	 L 	   341.22	  3032.93	 31
  5	 L 	   454.30	  2919.85	 30
  6	 L 	   567.39	  2806.76	 29
  7	 L 	   680.47	  2693.68	 28
  8	 L 	   793.55	  2580.60	 27
  9	 L 	   906.64	  2467.51	 26
 10	 L 	  1019.72	  2354.43	 25
 11	 L 	  1132.81	  2241.34	 24
 12	 V 	  1231.88	  2128.26	 23
 13	 L 	  1344.96	  2029.19	 22
 14	 A 	  1416.00	  1916.11	 21
 15	 A 	  1487.03	  1845.07	 20
 16	 V 	  1586.10	  1774.03	 19
 17	 G 	  1643.12	  1674.96	 18
 18	 I 	  1756.21	  1617.94	 17
 19	 V 	  1855.28	  1504.86	 16
 20	 A 	  1926.31	  1405.79	 15
 21	 G 	  1983.33	  1334.75	 14
 22	 G 	  2040.36	  1277.73	 13
 23	 L 	  2153.44	  1220.71	 12
 24	 L 	  2266.52	  1107.63	 11
 25	 G 	  2323.55	   994.54	 10
 26	 S 	  2410.58	   937.52	  9
 27	 A 	  2481.61	   850.49	  8
 28	 Q 	  2609.67	   779.45	  7
 29	 G 	  2666.69	   651.39	  6
 30	 P 	  2763.75	   594.37	  5
 31	 P 	  2860.80	   497.32	  4
 32	 K 	  2988.90	   400.27	  3
 33	 P 	  3085.95	   272.17	  2
 34	 R 	        -	        175.12	  1  _
```

+2 charge

```
		       B   	     Y
  1	 A 	    36.53	        -	      34
  2	 V 	    86.06	  1595.01	 33
  3	 G 	   114.57	  1545.48	 32
  4	 L 	   171.11	  1516.97	 31
  5	 L 	   227.65	  1460.43	 30
  6	 L 	   284.20	  1403.89	 29
  7	 L 	   340.74	  1347.34	 28
  8	 L 	   397.28	  1290.80	 27
  9	 L 	   453.82	  1234.26	 26
 10	 L 	   510.36	  1177.72	 25
 11	 L 	   566.91	  1121.18	 24
 12	 V 	   616.44	  1064.63	 23
 13	 L 	   672.98	  1015.10	 22
 14	 A 	   708.50	   958.56	 21
 15	 A 	   744.02	   923.04	 20
 16	 V 	   793.55	   887.52	 19
 17	 G 	   822.07	   837.99	 18
 18	 I 	   878.61	   809.48	 17
 19	 V 	   928.14	   752.93	 16
 20	 A 	   963.66	   703.40	 15
 21	 G 	   992.17	   667.88	 14
 22	 G 	  1020.68	   639.37	 13
 23	 L 	  1077.22	   610.86	 12
 24	 L 	  1133.77	   554.32	 11
 25	 G 	  1162.28	   497.78	 10
 26	 S 	  1205.79	   469.26	  9
 27	 A 	  1241.31	   425.75	  8
 28	 Q 	  1305.34	   390.23	  7
 29	 G 	  1333.85	   326.20	  6
 30	 P 	  1382.38	   297.69	  5
 31	 P 	  1430.90	   249.16	  4
 32	 K 	  1494.95	   200.64	  3
 33	 P 	  1543.48	   136.59	  2
 34	 R 	        -	        88.06	  1  _
```

+3 charge

```
		       B   	     Y
  1	 A 	    24.69	        -	      34
  2	 V 	    57.71	  1063.68	 33
  3	 G 	    76.72	  1030.66	 32
  4	 L 	   114.41	  1011.65	 31
  5	 L 	   152.11	   973.95	 30
  6	 L 	   189.80	   936.26	 29
  7	 L 	   227.50	   898.56	 28
  8	 L 	   265.19	   860.87	 27
  9	 L 	   302.88	   823.18	 26
 10	 L 	   340.58	   785.48	 25
 11	 L 	   378.27	   747.79	 24
 12	 V 	   411.30	   710.09	 23
 13	 L 	   448.99	   677.07	 22
 14	 A 	   472.67	   639.37	 21
 15	 A 	   496.35	   615.70	 20
 16	 V 	   529.37	   592.02	 19
 17	 G 	   548.38	   558.99	 18
 18	 I 	   586.07	   539.99	 17
 19	 V 	   619.10	   502.29	 16
 20	 A 	   642.78	   469.27	 15
 21	 G 	   661.78	   445.59	 14
 22	 G 	   680.79	   426.58	 13
 23	 L 	   718.48	   407.58	 12
 24	 L 	   756.18	   369.88	 11
 25	 G 	   775.19	   332.19	 10
 26	 S 	   804.20	   313.18	  9
 27	 A 	   827.88	   284.17	  8
 28	 Q 	   870.56	   260.49	  7
 29	 G 	   889.57	   217.80	  6
 30	 P 	   921.92	   198.80	  5
 31	 P 	   954.27	   166.44	  4
 32	 K 	   996.97	   134.09	  3
 33	 P 	  1029.32	    91.40	  2
```

34	R	-	59.04	1 _

**115. IPI00030786 Isoform 1 of Matrilin-4**

| Sequence | Charge | M+H+ | XCorr | DeltaCn |
| --- | --- | --- | --- | --- |
| R.TLGALESLTLNLAQLTAR.L | 2 | 1886.183 | 4.522 | 0.6 |

+1 charge

```
		      B   	       Y
  1	 T 	   102.05	        -  	 18
  2	 L 	   215.14	  1784.03	 17
  3	 G 	   272.16	  1670.94	 16
  4	 A 	   343.20	  1613.92	 15
  5	 L 	   456.28	  1542.88	 14
  6	 E 	   585.32	  1429.80	 13
  7	 S 	   672.36	  1300.76	 12
  8	 L 	   785.44	  1213.73	 11
  9	 T 	   886.49	  1100.64	 10
 10	 L 	   999.57	   999.59	  9
 11	 N 	  1113.62	   886.51	  8
 12	 L 	  1226.70	   772.47	  7
 13	 A 	  1297.74	   659.38	  6
 14	 Q 	  1425.79	   588.35	  5
 15	 L 	  1538.88	   460.29	  4
 16	 T 	  1639.93	   347.20	  3
 17	 A 	  1710.96	   246.16	  2
 18	 R 	        -  	   175.12	  1  _
+2 charge
		      B   	       Y
  1	 T 	    51.53	        -  	 18
  2	 L 	   108.07	   892.52	 17
  3	 G 	   136.58	   835.98	 16
  4	 A 	   172.10	   807.46	 15
  5	 L 	   228.64	   771.95	 14
  6	 E 	   293.17	   715.40	 13
  7	 S 	   336.68	   650.88	 12
  8	 L 	   393.22	   607.37	 11
  9	 T 	   443.75	   550.82	 10
 10	 L 	   500.29	   500.30	  9
 11	 N 	   557.31	   443.76	  8
 12	 L 	   613.85	   386.74	  7
 13	 A 	   649.37	   330.20	  6
 14	 Q 	   713.40	   294.68	  5
 15	 L 	   769.94	   230.65	  4
 16	 T 	   820.47	   174.11	  3
 17	 A 	   855.99	   123.58	  2
 18	 R 	        -  	    88.06	  1  _
```

**Female 3.**

**1. IPI00056309 Liver-expressed antimicrobial peptide 2**

| Sequence | Charge | M+H+ | XCorr | DeltaCn |
| --- | --- | --- | --- | --- |
| R.GVSLRPIGASCRDDSECITR.L | 3 | 2250.443 | 4.868 | 0.52 |

+1 charge

```
		      B   	       Y
  1	 G 	    58.03	        -  	 20
  2	 V 	   157.10	  2192.32	 19
  3	 S 	   244.13	  2093.26	 18
  4	 L 	   357.21	  2006.22	 17
  5	 R 	   513.31	  1893.14	 16
  6	 P 	   610.37	  1737.04	 15
  7	 I 	   723.45	  1639.99	 14
  8	 G 	   780.47	  1526.90	 13
  9	 A 	   851.51	  1469.88	 12
 10	 S 	   938.54	  1398.84	 11
 11	 C 	  1098.71	  1311.81	 10
 12	 R 	  1254.81	  1151.65	  9
 13	 D 	  1369.84	   995.54	  8
 14	 D 	  1484.86	   880.52	  7
 15	 S 	  1571.89	   765.49	  6
 16	 E 	  1700.94	   678.46	  5
 17	 C 	  1861.10	   549.42	  4
 18	 I 	  1974.19	   389.25	  3
 19	 T 	  2075.23	   276.17	  2
 20	 R 	        -  	   175.12	  1  _


+2 charge
		      B   	       Y
  1	 G 	    29.52	        -  	 20
  2	 V 	    79.05	  1096.67	 19
  3	 S 	   122.57	  1047.13	 18
  4	 L 	   179.11	  1003.62	 17
  5	 R 	   257.16	   947.07	 16
  6	 P 	   305.69	   869.02	 15
  7	 I 	   362.23	   820.50	 14
  8	 G 	   390.74	   763.95	 13
  9	 A 	   426.26	   735.44	 12
 10	 S 	   469.77	   699.93	 11
 11	 C 	   549.86	   656.41	 10
 12	 R 	   627.91	   576.33	  9
 13	 D 	   685.42	   498.28	  8
 14	 D 	   742.93	   440.76	  7
 15	 S 	   786.45	   383.25	  6
 16	 E 	   850.97	   339.73	  5
 17	 C 	   931.05	   275.21	  4
 18	 I 	   987.60	   195.13	  3
 19	 T 	  1038.12	   138.59	  2
 20	 R 	        -  	    88.06	  1  _
+3 charge
		      B   	       Y
  1	 G 	    20.01	        -  	 20
  2	 V 	    53.04	   731.45	 19
  3	 S 	    82.05	   698.42	 18
  4	 L 	   119.74	   669.41	 17
  5	 R 	   171.78	   631.72	 16
  6	 P 	   204.13	   579.68	 15
  7	 I 	   241.82	   547.33	 14
  8	 G 	   260.83	   509.64	 13
  9	 A 	   284.51	   490.63	 12
 10	 S 	   313.52	   466.95	 11
 11	 C 	   366.91	   437.94	 10
 12	 R 	   418.94	   384.55	  9
 13	 D 	   457.28	   332.52	  8
 14	 D 	   495.63	   294.18	  7
 15	 S 	   524.64	   255.84	  6
 16	 E 	   567.65	   226.82	  5
 17	 C 	   621.04	   183.81	  4
 18	 I 	   658.73	   130.42	  3
 19	 T 	   692.42	    92.73	  2
 20	 R 	        -  	    59.04	  1  _
```

**2. IPI00892695 Putative uncharacterized protein BRE**

| Sequence | Charge | M+H+ | XCorr | DeltaCn |
| --- | --- | --- | --- | --- |
| R.IEQLSLSPCHTDFLLTR.F | 2 | 2031.292 | 2.9 | 0.278 |

+1 charge

```
		      B   	       Y
  1	 I 	   114.09	        -  	 17
  2	 E 	   243.13	  1917.09	 16
  3	 Q 	   371.19	  1788.05	 15
  4	 L 	   484.28	  1659.99	 14
  5	 S 	   571.31	  1546.90	 13
  6	 L 	   684.39	  1459.87	 12
  7	 S 	   771.42	  1346.79	 11
  8	 P 	   868.48	  1259.75	 10
  9	 C 	  1028.64	  1162.70	  9
 10	 H 	  1165.70	  1002.54	  8
 11	 T 	  1266.75	   865.48	  7
 12	 D 	  1381.78	   764.43	  6
 13	 F 	  1528.84	   649.40	  5
 14	 L 	  1641.93	   502.33	  4
 15	 L 	  1755.01	   389.25	  3
 16	 T 	  1856.06	   276.17	  2
 17	 R 	        -  	   175.12	  1  _
+2 charge
		      B   	       Y
  1	 I 	    57.55	        -  	 17
  2	 E 	   122.07	   959.05	 16
  3	 Q 	   186.10	   894.53	 15
  4	 L 	   242.64	   830.50	 14
  5	 S 	   286.16	   773.96	 13
  6	 L 	   342.70	   730.44	 12
  7	 S 	   386.22	   673.90	 11
  8	 P 	   434.74	   630.38	 10
  9	 C 	   514.82	   581.85	  9
 10	 H 	   583.35	   501.77	  8
 11	 T 	   633.88	   433.24	  7
 12	 D 	   691.39	   382.72	  6
 13	 F 	   764.93	   325.21	  5
 14	 L 	   821.47	   251.67	  4
 15	 L 	   878.01	   195.13	  3
 16	 T 	   928.53	   138.59	  2
 17	 R 	        -  	    88.06	  1  _
```

**3. IPI00219827 Secreted Ly-6/uPAR-related protein 2**

| Sequence | Charge | M+H+ | XCorr | DeltaCn |
| --- | --- | --- | --- | --- |
| R.VLSNTEDLPLVTK.M | 2 | 1429.641 | 4.394 | 0.624 |

+1 charge

```
		      B   	       Y
  1	 V 	   100.08	        -  	 13
  2	 L 	   213.16	  1329.73	 12
  3	 S 	   300.19	  1216.64	 11
  4	 N 	   414.23	  1129.61	 10
  5	 T 	   515.28	  1015.57	  9
  6	 E 	   644.32	   914.52	  8
  7	 D 	   759.35	   785.48	  7
  8	 L 	   872.44	   670.45	  6
  9	 P 	   969.49	   557.37	  5
 10	 L 	  1082.57	   460.31	  4
 11	 V 	  1181.64	   347.23	  3
 12	 T 	  1282.69	   248.16	  2
 13	 K 	        -  	   147.11	  1  _
+2 charge
		      B   	       Y
  1	 V 	    50.54	        -  	 13
  2	 L 	   107.08	   665.37	 12
  3	 S 	   150.60	   608.82	 11
  4	 N 	   207.62	   565.31	 10
  5	 T 	   258.14	   508.29	  9
  6	 E 	   322.67	   457.76	  8
  7	 D 	   380.18	   393.24	  7
  8	 L 	   436.72	   335.73	  6
  9	 P 	   485.25	   279.19	  5
 10	 L 	   541.79	   230.66	  4
 11	 V 	   591.32	   174.12	  3
 12	 T 	   641.85	   124.58	  2
 13	 K 	        -  	    74.06	  1  _
```

**4. IPI00465431 Galectin-3**

| Sequence | Charge | M+H+ | XCorr | DeltaCn |
| --- | --- | --- | --- | --- |
| R.MLITILGTVKPNANR.I | 2 | 1642.004 | 2.825 | 0.4 |

+1 charge

```
		      B   	       Y
  1	 M 	   132.05	        -  	 15
  2	 L 	   245.13	  1509.91	 14
  3	 I 	   358.22	  1396.83	 13
  4	 T 	   459.26	  1283.74	 12
  5	 I 	   572.35	  1182.70	 11
  6	 L 	   685.43	  1069.61	 10
  7	 G 	   742.45	   956.53	  9
  8	 T 	   843.50	   899.51	  8
  9	 V 	   942.57	   798.46	  7
 10	 K 	  1070.66	   699.39	  6
 11	 P 	  1167.72	   571.29	  5
 12	 N 	  1281.76	   474.24	  4
 13	 A 	  1352.80	   360.20	  3
 14	 N 	  1466.84	   289.16	  2
 15	 R 	        -  	   175.12	  1  _
+2 charge
		      B   	       Y
  1	 M 	    66.53	        -  	 15
  2	 L 	   123.07	   755.46	 14
  3	 I 	   179.61	   698.92	 13
  4	 T 	   230.14	   642.38	 12
  5	 I 	   286.68	   591.85	 11
  6	 L 	   343.22	   535.31	 10
  7	 G 	   371.73	   478.77	  9
  8	 T 	   422.25	   450.26	  8
  9	 V 	   471.79	   399.73	  7
 10	 K 	   535.84	   350.20	  6
 11	 P 	   584.36	   286.15	  5
 12	 N 	   641.38	   237.62	  4
 13	 A 	   676.90	   180.60	  3
 14	 N 	   733.92	   145.08	  2
 15	 R 	        -  	    88.06	  1  _
```

**5. IPI00018136 Isoform 1 of Vascular cell adhesion protein 1**

| Sequence | Charge | M+H+ | XCorr | DeltaCn |
| --- | --- | --- | --- | --- |
| K.SQEFLEDADRKSLETK.S | 2 | 1897.033 | 3.591 | 0.279 |

+1 charge

```
		      B   	       Y
  1	 S 	    88.04	        -  	 16
  2	 Q 	   216.10	  1808.90	 15
  3	 E 	   345.14	  1680.84	 14
  4	 F 	   492.21	  1551.80	 13
  5	 L 	   605.29	  1404.73	 12
  6	 E 	   734.34	  1291.65	 11
  7	 D 	   849.36	  1162.61	 10
  8	 A 	   920.40	  1047.58	  9
  9	 D 	  1035.43	   976.54	  8
 10	 R 	  1191.53	   861.52	  7
 11	 K 	  1319.62	   705.41	  6
 12	 S 	  1406.65	   577.32	  5
 13	 L 	  1519.74	   490.29	  4
 14	 E 	  1648.78	   377.20	  3
 15	 T 	  1749.83	   248.16	  2
 16	 K 	        -  	   147.11	  1  _
+2 charge
		      B   	       Y
  1	 S 	    44.52	        -  	 16
  2	 Q 	   108.55	   904.95	 15
  3	 E 	   173.07	   840.93	 14
  4	 F 	   246.61	   776.40	 13
  5	 L 	   303.15	   702.87	 12
  6	 E 	   367.67	   646.33	 11
  7	 D 	   425.18	   581.81	 10
  8	 A 	   460.70	   524.29	  9
  9	 D 	   518.22	   488.77	  8
 10	 R 	   596.27	   431.26	  7
 11	 K 	   660.31	   353.21	  6
 12	 S 	   703.83	   289.16	  5
 13	 L 	   760.37	   245.65	  4
 14	 E 	   824.89	   189.11	  3
 15	 T 	   875.42	   124.58	  2
 16	 K 	        -  	    74.06	  1  _
```

**6. IPI00290085 Cadherin-2**

| Sequence | Charge | M+H+ | XCorr | DeltaCn |
| --- | --- | --- | --- | --- |
| R.FAIQTDPNSNDGLVTVVKPIDFETNR.M | 3 | 2892.17 | 4.564 | 0.537 |

+1 charge

```
		      B   	       Y
  1	 F 	   148.08	        -  	 26
  2	 A 	   219.11	  2743.39	 25
  3	 I 	   332.20	  2672.35	 24
  4	 Q 	   460.26	  2559.27	 23
  5	 T 	   561.30	  2431.21	 22
  6	 D 	   676.33	  2330.16	 21
  7	 P 	   773.38	  2215.14	 20
  8	 N 	   887.43	  2118.08	 19
  9	 S 	   974.46	  2004.04	 18
 10	 N 	  1088.50	  1917.01	 17
 11	 D 	  1203.53	  1802.96	 16
 12	 G 	  1260.55	  1687.94	 15
 13	 L 	  1373.63	  1630.92	 14
 14	 V 	  1472.70	  1517.83	 13
 15	 T 	  1573.75	  1418.76	 12
 16	 V 	  1672.82	  1317.72	 11
 17	 V 	  1771.89	  1218.65	 10
 18	 K 	  1899.98	  1119.58	  9
 19	 P 	  1997.03	   991.48	  8
 20	 I 	  2110.12	   894.43	  7
 21	 D 	  2225.14	   781.35	  6
 22	 F 	  2372.21	   666.32	  5
 23	 E 	  2501.26	   519.25	  4
 24	 T 	  2602.30	   390.21	  3
 25	 N 	  2716.35	   289.16	  2
 26	 R 	        -  	   175.12	  1  _


+2 charge
		      B   	       Y
  1	 F 	    74.54	        -  	 26
  2	 A 	   110.06	  1372.20	 25
  3	 I 	   166.60	  1336.68	 24
  4	 Q 	   230.63	  1280.14	 23
  5	 T 	   281.16	  1216.11	 22
  6	 D 	   338.67	  1165.58	 21
  7	 P 	   387.20	  1108.07	 20
  8	 N 	   444.22	  1059.54	 19
  9	 S 	   487.73	  1002.52	 18
 10	 N 	   544.75	   959.01	 17
 11	 D 	   602.27	   901.99	 16
 12	 G 	   630.78	   844.47	 15
 13	 L 	   687.32	   815.96	 14
 14	 V 	   736.85	   759.42	 13
 15	 T 	   787.38	   709.89	 12
 16	 V 	   836.91	   659.36	 11
 17	 V 	   886.45	   609.83	 10
 18	 K 	   950.49	   560.29	  9
 19	 P 	   999.02	   496.25	  8
 20	 I 	  1055.56	   447.72	  7
 21	 D 	  1113.08	   391.18	  6
 22	 F 	  1186.61	   333.66	  5
 23	 E 	  1251.13	   260.13	  4
 24	 T 	  1301.66	   195.61	  3
 25	 N 	  1358.68	   145.08	  2
 26	 R 	        -  	    88.06	  1  _


+3 charge
		      B   	       Y
  1	 F 	    50.03	        -  	 26
  2	 A 	    73.71	   915.13	 25
  3	 I 	   111.40	   891.46	 24
  4	 Q 	   154.09	   853.76	 23
  5	 T 	   187.77	   811.07	 22
  6	 D 	   226.11	   777.39	 21
  7	 P 	   258.47	   739.05	 20
  8	 N 	   296.48	   706.70	 19
  9	 S 	   325.49	   668.68	 18
 10	 N 	   363.51	   639.67	 17
 11	 D 	   401.85	   601.66	 16
 12	 G 	   420.85	   563.32	 15
 13	 L 	   458.55	   544.31	 14
 14	 V 	   491.57	   506.62	 13
 15	 T 	   525.25	   473.59	 12
 16	 V 	   558.28	   439.91	 11
 17	 V 	   591.30	   406.89	 10
 18	 K 	   634.00	   373.86	  9
 19	 P 	   666.35	   331.17	  8
 20	 I 	   704.04	   298.82	  7
 21	 D 	   742.39	   261.12	  6
 22	 F 	   791.41	   222.78	  5
 23	 E 	   834.42	   173.76	  4
 24	 T 	   868.11	   130.74	  3
 25	 N 	   906.12	    97.06	  2
 26	 R 	        -  	    59.04	  1  _
```

**7. IPI00759466 Isoform 4 of Epidermal growth factor receptor kinase substrate 8-like protein 1**

| Sequence | Charge | M+H+ | XCorr | DeltaCn |
| --- | --- | --- | --- | --- |
| R.LGAELIREDIQGALHNYR.S | 3 | 2069.31 | 3.325 | 0.425 |

+1 charge

```
		      B   	       Y
  1	 L 	   114.09	        -  	 18
  2	 G 	   171.11	  1955.01	 17
  3	 A 	   242.15	  1897.99	 16
  4	 E 	   371.19	  1826.95	 15
  5	 L 	   484.28	  1697.91	 14
  6	 I 	   597.36	  1584.82	 13
  7	 R 	   753.46	  1471.74	 12
  8	 E 	   882.50	  1315.64	 11
  9	 D 	   997.53	  1186.60	 10
 10	 I 	  1110.62	  1071.57	  9
 11	 Q 	  1238.67	   958.49	  8
 12	 G 	  1295.70	   830.43	  7
 13	 A 	  1366.73	   773.41	  6
 14	 L 	  1479.82	   702.37	  5
 15	 H 	  1616.88	   589.28	  4
 16	 N 	  1730.92	   452.23	  3
 17	 Y 	  1893.98	   338.18	  2
 18	 R 	        -  	   175.12	  1  _
+2 charge
		      B   	       Y
  1	 L 	    57.55	        -  	 18
  2	 G 	    86.06	   978.01	 17
  3	 A 	   121.58	   949.50	 16
  4	 E 	   186.10	   913.98	 15
  5	 L 	   242.64	   849.46	 14
  6	 I 	   299.18	   792.92	 13
  7	 R 	   377.23	   736.37	 12
  8	 E 	   441.76	   658.32	 11
  9	 D 	   499.27	   593.80	 10
 10	 I 	   555.81	   536.29	  9
 11	 Q 	   619.84	   479.75	  8
 12	 G 	   648.35	   415.72	  7
 13	 A 	   683.87	   387.21	  6
 14	 L 	   740.41	   351.69	  5
 15	 H 	   808.94	   295.15	  4
 16	 N 	   865.96	   226.62	  3
 17	 Y 	   947.49	   169.59	  2
 18	 R 	        -  	    88.06	  1  _


+3 charge
		      B   	       Y
  1	 L 	    38.70	        -  	 18
  2	 G 	    57.71	   652.34	 17
  3	 A 	    81.39	   633.33	 16
  4	 E 	   124.40	   609.66	 15
  5	 L 	   162.10	   566.64	 14
  6	 I 	   199.79	   528.95	 13
  7	 R 	   251.83	   491.25	 12
  8	 E 	   294.84	   439.22	 11
  9	 D 	   333.18	   396.20	 10
 10	 I 	   370.88	   357.86	  9
 11	 Q 	   413.56	   320.17	  8
 12	 G 	   432.57	   277.48	  7
 13	 A 	   456.25	   258.47	  6
 14	 L 	   493.94	   234.79	  5
 15	 H 	   539.63	   197.10	  4
 16	 N 	   577.64	   151.41	  3
 17	 Y 	   632.00	   113.40	  2
 18	 R 	        -  	    59.04	  1  _
```

**8. IPI00291866 Plasma protease C1 inhibitor**

| Sequence | Charge | M+H+ | XCorr | DeltaCn |
| --- | --- | --- | --- | --- |
| K.AIMEKLEMSKFQPTLLTLPR.I | 3 | 2347.87 | 5.483 | 0.513 |

+1 charge

```
		      B   	       Y
  1	 A 	    72.04	        -  	 20
  2	 I 	   185.13	  2275.26	 19
  3	 M 	   316.17	  2162.17	 18
  4	 E 	   445.21	  2031.13	 17
  5	 K 	   573.31	  1902.09	 16
  6	 L 	   686.39	  1773.99	 15
  7	 E 	   815.43	  1660.91	 14
  8	 M 	   946.47	  1531.87	 13
  9	 S 	  1033.51	  1400.83	 12
 10	 K 	  1161.60	  1313.79	 11
 11	 F 	  1308.67	  1185.70	 10
 12	 Q 	  1436.73	  1038.63	  9
 13	 P 	  1533.78	   910.57	  8
 14	 T 	  1634.83	   813.52	  7
 15	 L 	  1747.91	   712.47	  6
 16	 L 	  1861.00	   599.39	  5
 17	 T 	  1962.04	   486.30	  4
 18	 L 	  2075.13	   385.26	  3
 19	 P 	  2172.18	   272.17	  2
 20	 R 	        -  	   175.12	  1  _


+2 charge
		      B   	       Y
  1	 A 	    36.53	        -  	 20
  2	 I 	    93.07	  1138.13	 19
  3	 M 	   158.59	  1081.59	 18
  4	 E 	   223.11	  1016.07	 17
  5	 K 	   287.16	   951.55	 16
  6	 L 	   343.70	   887.50	 15
  7	 E 	   408.22	   830.96	 14
  8	 M 	   473.74	   766.44	 13
  9	 S 	   517.26	   700.92	 12
 10	 K 	   581.30	   657.40	 11
 11	 F 	   654.84	   593.35	 10
 12	 Q 	   718.87	   519.82	  9
 13	 P 	   767.39	   455.79	  8
 14	 T 	   817.92	   407.26	  7
 15	 L 	   874.46	   356.74	  6
 16	 L 	   931.00	   300.20	  5
 17	 T 	   981.53	   243.66	  4
 18	 L 	  1038.07	   193.13	  3
 19	 P 	  1086.59	   136.59	  2
 20	 R 	        -  	    88.06	  1  _
+3 charge
		      B   	       Y
  1	 A 	    24.69	        -  	 20
  2	 I 	    62.38	   759.09	 19
  3	 M 	   106.06	   721.40	 18
  4	 E 	   149.08	   677.72	 17
  5	 K 	   191.77	   634.70	 16
  6	 L 	   229.47	   592.00	 15
  7	 E 	   272.48	   554.31	 14
  8	 M 	   316.16	   511.29	 13
  9	 S 	   345.17	   467.61	 12
 10	 K 	   387.87	   438.60	 11
 11	 F 	   436.89	   395.90	 10
 12	 Q 	   479.58	   346.88	  9
 13	 P 	   511.93	   304.20	  8
 14	 T 	   545.61	   271.84	  7
 15	 L 	   583.31	   238.16	  6
 16	 L 	   621.00	   200.47	  5
 17	 T 	   654.69	   162.77	  4
 18	 L 	   692.38	   129.09	  3
 19	 P 	   724.73	    91.40	  2
 20	 R 	        -  	    59.04	  1  _
```

**9. IPI00479186 Isoform M2 of Pyruvate kinase isozymes M1/M2**

| Sequence | Charge | M+H+ | XCorr | DeltaCn |
| --- | --- | --- | --- | --- |
| R.LAPITSDPTEATAVGAVEASFK.C | 2 | 2176.409 | 3.135 | 0.36 |

+1 charge

```
		      B   	       Y
  1	 L 	   114.09	        -  	 22
  2	 A 	   185.13	  2062.03	 21
  3	 P 	   282.18	  1991.00	 20
  4	 I 	   395.27	  1893.94	 19
  5	 T 	   496.31	  1780.86	 18
  6	 S 	   583.34	  1679.81	 17
  7	 D 	   698.37	  1592.78	 16
  8	 P 	   795.42	  1477.75	 15
  9	 T 	   896.47	  1380.70	 14
 10	 E 	  1025.51	  1279.65	 13
 11	 A 	  1096.55	  1150.61	 12
 12	 T 	  1197.60	  1079.57	 11
 13	 A 	  1268.64	   978.53	 10
 14	 V 	  1367.71	   907.49	  9
 15	 G 	  1424.73	   808.42	  8
 16	 A 	  1495.76	   751.40	  7
 17	 V 	  1594.83	   680.36	  6
 18	 E 	  1723.87	   581.29	  5
 19	 A 	  1794.91	   452.25	  4
 20	 S 	  1881.94	   381.21	  3
 21	 F 	  2029.01	   294.18	  2
 22	 K 	        -  	   147.11	  1  _


+2 charge
		      B   	       Y
  1	 L 	    57.55	        -  	 22
  2	 A 	    93.07	  1031.52	 21
  3	 P 	   141.59	   996.00	 20
  4	 I 	   198.14	   947.48	 19
  5	 T 	   248.66	   890.93	 18
  6	 S 	   292.18	   840.41	 17
  7	 D 	   349.69	   796.89	 16
  8	 P 	   398.22	   739.38	 15
  9	 T 	   448.74	   690.85	 14
 10	 E 	   513.26	   640.33	 13
 11	 A 	   548.78	   575.81	 12
 12	 T 	   599.30	   540.29	 11
 13	 A 	   634.82	   489.77	 10
 14	 V 	   684.36	   454.25	  9
 15	 G 	   712.87	   404.71	  8
 16	 A 	   748.39	   376.20	  7
 17	 V 	   797.92	   340.68	  6
 18	 E 	   862.44	   291.15	  5
 19	 A 	   897.96	   226.63	  4
 20	 S 	   941.48	   191.11	  3
 21	 F 	  1015.01	   147.59	  2
 22	 K 	        -  	    74.06	  1  _
```

**10. IPI00007797 Fatty acid-binding protein, epidermal**

| Sequence | Charge | M+H+ | XCorr | DeltaCn |
| --- | --- | --- | --- | --- |
| R.LVDSKGFDEYMKELGVGIALR.K | 3 | 2341.712 | 4.658 | 0.513 |

+1 charge

```
		      B   	       Y
  1	 L 	   114.09	        -  	 21
  2	 V 	   213.16	  2227.14	 20
  3	 D 	   328.19	  2128.07	 19
  4	 S 	   415.22	  2013.05	 18
  5	 K 	   543.31	  1926.02	 17
  6	 G 	   600.34	  1797.92	 16
  7	 F 	   747.40	  1740.90	 15
  8	 D 	   862.43	  1593.83	 14
  9	 E 	   991.47	  1478.80	 13
 10	 Y 	  1154.54	  1349.76	 12
 11	 M 	  1285.58	  1186.70	 11
 12	 K 	  1413.67	  1055.66	 10
 13	 E 	  1542.71	   927.56	  9
 14	 L 	  1655.80	   798.52	  8
 15	 G 	  1712.82	   685.44	  7
 16	 V 	  1811.89	   628.41	  6
 17	 G 	  1868.91	   529.35	  5
 18	 I 	  1981.99	   472.32	  4
 19	 A 	  2053.03	   359.24	  3
 20	 L 	  2166.12	   288.20	  2
 21	 R 	        -  	   175.12	  1  _


+2 charge
		      B   	       Y
  1	 L 	    57.55	        -  	 21
  2	 V 	   107.08	  1114.08	 20
  3	 D 	   164.60	  1064.54	 19
  4	 S 	   208.11	  1007.03	 18
  5	 K 	   272.16	   963.51	 17
  6	 G 	   300.67	   899.46	 16
  7	 F 	   374.21	   870.95	 15
  8	 D 	   431.72	   797.42	 14
  9	 E 	   496.24	   739.91	 13
 10	 Y 	   577.77	   675.38	 12
 11	 M 	   643.29	   593.85	 11
 12	 K 	   707.34	   528.33	 10
 13	 E 	   771.86	   464.28	  9
 14	 L 	   828.40	   399.76	  8
 15	 G 	   856.91	   343.22	  7
 16	 V 	   906.45	   314.71	  6
 17	 G 	   934.96	   265.18	  5
 18	 I 	   991.50	   236.67	  4
 19	 A 	  1027.02	   180.12	  3
 20	 L 	  1083.56	   144.61	  2
 21	 R 	        -  	    88.06	  1  _
+3 charge
		      B   	       Y
  1	 L 	    38.70	        -  	 21
  2	 V 	    71.72	   743.05	 20
  3	 D 	   110.07	   710.03	 19
  4	 S 	   139.08	   671.69	 18
  5	 K 	   181.78	   642.68	 17
  6	 G 	   200.78	   599.98	 16
  7	 F 	   249.81	   580.97	 15
  8	 D 	   288.15	   531.95	 14
  9	 E 	   331.16	   493.61	 13
 10	 Y 	   385.52	   450.59	 12
 11	 M 	   429.20	   396.24	 11
 12	 K 	   471.90	   352.56	 10
 13	 E 	   514.91	   309.86	  9
 14	 L 	   552.60	   266.84	  8
 15	 G 	   571.61	   229.15	  7
 16	 V 	   604.63	   210.14	  6
 17	 G 	   623.64	   177.12	  5
 18	 I 	   661.34	   158.11	  4
 19	 A 	   685.02	   120.42	  3
 20	 L 	   722.71	    96.74	  2
 21	 R 	        -  	    59.04	  1  _
```

**11. IPI00873924 Isoform A of Interleukin-18-binding protein**

| Sequence | Charge | M+H+ | XCorr | DeltaCn |
| --- | --- | --- | --- | --- |
| K.ALVLEQLTPALHSTNFSCVLVDPEQVVQR.H | 3 | 3265.699 | 3.321 | 0.356 |

```
+1 charge
		      B   	       Y
  1	 A 	    72.04	        -  	 29
  2	 L 	   185.13	  3192.81	 28
  3	 V 	   284.20	  3079.72	 27
  4	 L 	   397.28	  2980.65	 26
  5	 E 	   526.32	  2867.57	 25
  6	 Q 	   654.38	  2738.53	 24
  7	 L 	   767.47	  2610.47	 23
  8	 T 	   868.51	  2497.38	 22
  9	 P 	   965.57	  2396.34	 21
 10	 A 	  1036.60	  2299.28	 20
 11	 L 	  1149.69	  2228.25	 19
 12	 H 	  1286.75	  2115.16	 18
 13	 S 	  1373.78	  1978.10	 17
 14	 T 	  1474.83	  1891.07	 16
 15	 N 	  1588.87	  1790.02	 15
 16	 F 	  1735.94	  1675.98	 14
 17	 S 	  1822.97	  1528.91	 13
 18	 C 	  1983.14	  1441.88	 12
 19	 V 	  2082.20	  1281.72	 11
 20	 L 	  2195.29	  1182.65	 10
 21	 V 	  2294.36	  1069.56	  9
 22	 D 	  2409.38	   970.50	  8
 23	 P 	  2506.44	   855.47	  7
 24	 E 	  2635.48	   758.42	  6
 25	 Q 	  2763.54	   629.37	  5
 26	 V 	  2862.61	   501.31	  4
 27	 V 	  2961.67	   402.25	  3
 28	 Q 	  3089.73	   303.18	  2
 29	 R 	        -  	   175.12	  1  _


+2 charge
		      B   	       Y
  1	 A 	    36.53	        -  	 29
  2	 L 	    93.07	  1596.91	 28
  3	 V 	   142.60	  1540.36	 27
  4	 L 	   199.14	  1490.83	 26
  5	 E 	   263.67	  1434.29	 25
  6	 Q 	   327.69	  1369.77	 24
  7	 L 	   384.24	  1305.74	 23
  8	 T 	   434.76	  1249.20	 22
  9	 P 	   483.29	  1198.67	 21
 10	 A 	   518.81	  1150.15	 20
 11	 L 	   575.35	  1114.63	 19
 12	 H 	   643.88	  1058.09	 18
 13	 S 	   687.39	   989.56	 17
 14	 T 	   737.92	   946.04	 16
 15	 N 	   794.94	   895.52	 15
 16	 F 	   868.47	   838.49	 14
 17	 S 	   911.99	   764.96	 13
 18	 C 	   992.07	   721.44	 12
 19	 V 	  1041.61	   641.36	 11
 20	 L 	  1098.15	   591.83	 10
 21	 V 	  1147.68	   535.29	  9
 22	 D 	  1205.20	   485.75	  8
 23	 P 	  1253.72	   428.24	  7
 24	 E 	  1318.24	   379.71	  6
 25	 Q 	  1382.27	   315.19	  5
 26	 V 	  1431.81	   251.16	  4
 27	 V 	  1481.34	   201.63	  3
 28	 Q 	  1545.37	   152.09	  2
 29	 R 	        -  	    88.06	  1  _


+3 charge
		      B   	       Y
  1	 A 	    24.69	        -  	 29
  2	 L 	    62.38	  1064.94	 28
  3	 V 	    95.40	  1027.25	 27
  4	 L 	   133.10	   994.22	 26
  5	 E 	   176.11	   956.53	 25
  6	 Q 	   218.80	   913.51	 24
  7	 L 	   256.49	   870.83	 23
  8	 T 	   290.18	   833.13	 22
  9	 P 	   322.53	   799.45	 21
 10	 A 	   346.21	   767.10	 20
 11	 L 	   383.90	   743.42	 19
 12	 H 	   429.59	   705.73	 18
 13	 S 	   458.60	   660.04	 17
 14	 T 	   492.28	   631.03	 16
 15	 N 	   530.29	   597.35	 15
 16	 F 	   579.32	   559.33	 14
 17	 S 	   608.33	   510.31	 13
 18	 C 	   661.72	   481.30	 12
 19	 V 	   694.74	   427.91	 11
 20	 L 	   732.43	   394.89	 10
 21	 V 	   765.46	   357.19	  9
 22	 D 	   803.80	   324.17	  8
 23	 P 	   836.15	   285.83	  7
 24	 E 	   879.16	   253.48	  6
 25	 Q 	   921.85	   210.46	  5
 26	 V 	   954.87	   167.78	  4
 27	 V 	   987.90	   134.75	  3
 28	 Q 	  1030.58	   101.73	  2
 29	 R 	        -  	    59.04	  1  _
```

**12. IPI00304808 Isoform 1 of Kallikrein-1**

| Sequence | Charge | M+H+ | XCorr | DeltaCn |
| --- | --- | --- | --- | --- |
| R.VLSYVKWIEDTIAENS.- | 2 | 1868.076 | 4.079 | 0.512 |

+1 charge

```
		      B   	       Y
  1	 V 	   100.08	        -  	 16
  2	 L 	   213.16	  1767.88	 15
  3	 S 	   300.19	  1654.80	 14
  4	 Y 	   463.26	  1567.76	 13
  5	 V 	   562.32	  1404.70	 12
  6	 K 	   690.42	  1305.63	 11
  7	 W 	   876.50	  1177.54	 10
  8	 I 	   989.58	   991.46	  9
  9	 E 	  1118.62	   878.37	  8
 10	 D 	  1233.65	   749.33	  7
 11	 T 	  1334.70	   634.30	  6
 12	 I 	  1447.78	   533.26	  5
 13	 A 	  1518.82	   420.17	  4
 14	 E 	  1647.86	   349.14	  3
 15	 N 	  1761.91	   220.09	  2
 16	 S 	        -  	   106.05	  1  _
+2 charge
		      B   	       Y
  1	 V 	    50.54	        -  	 16
  2	 L 	   107.08	   884.44	 15
  3	 S 	   150.60	   827.90	 14
  4	 Y 	   232.13	   784.39	 13
  5	 V 	   281.67	   702.85	 12
  6	 K 	   345.71	   653.32	 11
  7	 W 	   438.75	   589.27	 10
  8	 I 	   495.29	   496.23	  9
  9	 E 	   559.82	   439.69	  8
 10	 D 	   617.33	   375.17	  7
 11	 T 	   667.85	   317.66	  6
 12	 I 	   724.40	   267.13	  5
 13	 A 	   759.91	   210.59	  4
 14	 E 	   824.44	   175.07	  3
 15	 N 	   881.46	   110.55	  2
 16	 S 	        -  	    53.53	  1  _
```

**13. IPI00246058 Programmed cell death 6-interacting protein**

| Sequence | Charge | M+H+ | XCorr | DeltaCn |
| --- | --- | --- | --- | --- |
| R.SVIEQGGIQTVDQLIKELPELLQR.N | 3 | 2708.104 | 4.49 | 0.581 |

+1 charge

```
		      B   	       Y
  1	 S 	    88.04	        -  	 24
  2	 V 	   187.11	  2619.47	 23
  3	 I 	   300.19	  2520.40	 22
  4	 E 	   429.23	  2407.32	 21
  5	 Q 	   557.29	  2278.28	 20
  6	 G 	   614.31	  2150.22	 19
  7	 G 	   671.34	  2093.20	 18
  8	 I 	   784.42	  2036.18	 17
  9	 Q 	   912.48	  1923.09	 16
 10	 T 	  1013.53	  1795.03	 15
 11	 V 	  1112.59	  1693.98	 14
 12	 D 	  1227.62	  1594.92	 13
 13	 Q 	  1355.68	  1479.89	 12
 14	 L 	  1468.76	  1351.83	 11
 15	 I 	  1581.85	  1238.75	 10
 16	 K 	  1709.94	  1125.66	  9
 17	 E 	  1838.99	   997.57	  8
 18	 L 	  1952.07	   868.53	  7
 19	 P 	  2049.12	   755.44	  6
 20	 E 	  2178.17	   658.39	  5
 21	 L 	  2291.25	   529.35	  4
 22	 L 	  2404.33	   416.26	  3
 23	 Q 	  2532.39	   303.18	  2
 24	 R 	        -  	   175.12	  1  _


+2 charge
		      B   	       Y
  1	 S 	    44.52	        -  	 24
  2	 V 	    94.06	  1310.24	 23
  3	 I 	   150.60	  1260.71	 22
  4	 E 	   215.12	  1204.16	 21
  5	 Q 	   279.15	  1139.64	 20
  6	 G 	   307.66	  1075.61	 19
  7	 G 	   336.17	  1047.10	 18
  8	 I 	   392.71	  1018.59	 17
  9	 Q 	   456.74	   962.05	 16
 10	 T 	   507.27	   898.02	 15
 11	 V 	   556.80	   847.50	 14
 12	 D 	   614.31	   797.96	 13
 13	 Q 	   678.34	   740.45	 12
 14	 L 	   734.89	   676.42	 11
 15	 I 	   791.43	   619.88	 10
 16	 K 	   855.48	   563.33	  9
 17	 E 	   920.00	   499.29	  8
 18	 L 	   976.54	   434.77	  7
 19	 P 	  1025.06	   378.22	  6
 20	 E 	  1089.59	   329.70	  5
 21	 L 	  1146.13	   265.18	  4
 22	 L 	  1202.67	   208.63	  3
 23	 Q 	  1266.70	   152.09	  2
 24	 R 	        -  	    88.06	  1  _


+3 charge
		      B   	       Y
  1	 S 	    30.02	        -  	 24
  2	 V 	    63.04	   873.83	 23
  3	 I 	   100.74	   840.81	 22
  4	 E 	   143.75	   803.11	 21
  5	 Q 	   186.44	   760.10	 20
  6	 G 	   205.44	   717.41	 19
  7	 G 	   224.45	   698.40	 18
  8	 I 	   262.14	   679.40	 17
  9	 Q 	   304.83	   641.70	 16
 10	 T 	   338.51	   599.02	 15
 11	 V 	   371.54	   565.33	 14
 12	 D 	   409.88	   532.31	 13
 13	 Q 	   452.56	   493.97	 12
 14	 L 	   490.26	   451.28	 11
 15	 I 	   527.95	   413.59	 10
 16	 K 	   570.65	   375.89	  9
 17	 E 	   613.67	   333.19	  8
 18	 L 	   651.36	   290.18	  7
 19	 P 	   683.71	   252.49	  6
 20	 E 	   726.73	   220.13	  5
 21	 L 	   764.42	   177.12	  4
 22	 L 	   802.12	   139.43	  3
 23	 Q 	   844.80	   101.73	  2
 24	 R 	        -  	    59.04	  1  _
```

**14. IPI00555812 Isoform 1 of Vitamin D-binding protein**

| Sequence | Charge | M+H+ | XCorr | DeltaCn |
| --- | --- | --- | --- | --- |
| K.LAQKVPTADLEDVLPLAEDITNILSK.C | 3 | 2808.217 | 5.306 | 0.585 |

+1 charge

```
		      B   	       Y
  1	 L 	   114.09	        -  	 26
  2	 A 	   185.13	  2693.46	 25
  3	 Q 	   313.19	  2622.42	 24
  4	 K 	   441.28	  2494.37	 23
  5	 V 	   540.35	  2366.27	 22
  6	 P 	   637.40	  2267.20	 21
  7	 T 	   738.45	  2170.15	 20
  8	 A 	   809.49	  2069.10	 19
  9	 D 	   924.51	  1998.06	 18
 10	 L 	  1037.60	  1883.04	 17
 11	 E 	  1166.64	  1769.95	 16
 12	 D 	  1281.67	  1640.91	 15
 13	 V 	  1380.74	  1525.88	 14
 14	 L 	  1493.82	  1426.82	 13
 15	 P 	  1590.87	  1313.73	 12
 16	 L 	  1703.96	  1216.68	 11
 17	 A 	  1774.99	  1103.59	 10
 18	 E 	  1904.04	  1032.56	  9
 19	 D 	  2019.06	   903.51	  8
 20	 I 	  2132.15	   788.49	  7
 21	 T 	  2233.20	   675.40	  6
 22	 N 	  2347.24	   574.36	  5
 23	 I 	  2460.32	   460.31	  4
 24	 L 	  2573.41	   347.23	  3
 25	 S 	  2660.44	   234.14	  2
 26	 K 	        -  	   147.11	  1  _


+2 charge
		      B   	       Y
  1	 L 	    57.55	        -  	 26
  2	 A 	    93.07	  1347.23	 25
  3	 Q 	   157.10	  1311.72	 24
  4	 K 	   221.14	  1247.69	 23
  5	 V 	   270.68	  1183.64	 22
  6	 P 	   319.21	  1134.10	 21
  7	 T 	   369.73	  1085.58	 20
  8	 A 	   405.25	  1035.05	 19
  9	 D 	   462.76	   999.54	 18
 10	 L 	   519.30	   942.02	 17
 11	 E 	   583.82	   885.48	 16
 12	 D 	   641.34	   820.96	 15
 13	 V 	   690.87	   763.45	 14
 14	 L 	   747.41	   713.91	 13
 15	 P 	   795.94	   657.37	 12
 16	 L 	   852.48	   608.84	 11
 17	 A 	   888.00	   552.30	 10
 18	 E 	   952.52	   516.78	  9
 19	 D 	  1010.04	   452.26	  8
 20	 I 	  1066.58	   394.75	  7
 21	 T 	  1117.10	   338.21	  6
 22	 N 	  1174.12	   287.68	  5
 23	 I 	  1230.67	   230.66	  4
 24	 L 	  1287.21	   174.12	  3
 25	 S 	  1330.72	   117.58	  2
 26	 K 	        -  	    74.06	  1  _


+3 charge
		      B   	       Y
  1	 L 	    38.70	        -  	 26
  2	 A 	    62.38	   898.49	 25
  3	 Q 	   105.07	   874.81	 24
  4	 K 	   147.77	   832.13	 23
  5	 V 	   180.79	   789.43	 22
  6	 P 	   213.14	   756.41	 21
  7	 T 	   246.82	   724.05	 20
  8	 A 	   270.50	   690.37	 19
  9	 D 	   308.84	   666.69	 18
 10	 L 	   346.54	   628.35	 17
 11	 E 	   389.55	   590.66	 16
 12	 D 	   427.89	   547.64	 15
 13	 V 	   460.92	   509.30	 14
 14	 L 	   498.61	   476.28	 13
 15	 P 	   530.96	   438.58	 12
 16	 L 	   568.66	   406.23	 11
 17	 A 	   592.34	   368.54	 10
 18	 E 	   635.35	   344.86	  9
 19	 D 	   673.69	   301.84	  8
 20	 I 	   711.39	   263.50	  7
 21	 T 	   745.07	   225.81	  6
 22	 N 	   783.08	   192.12	  5
 23	 I 	   820.78	   154.11	  4
 24	 L 	   858.47	   116.41	  3
 25	 S 	   887.48	    78.72	  2
 26	 K 	        -  	    49.71	  1  _
```

**15. IPI00217466 Histone H1.3**

| Sequence | Charge | M+H+ | XCorr | DeltaCn |
| --- | --- | --- | --- | --- |
| K.ALAAAGYDVEKNNSR.I | 2 | 1579.697 | 3.641 | 0.532 |

+1 charge

```
		      B   	       Y
  1	 A 	    72.04	        -  	 15
  2	 L 	   185.13	  1507.75	 14
  3	 A 	   256.17	  1394.67	 13
  4	 A 	   327.20	  1323.63	 12
  5	 A 	   398.24	  1252.59	 11
  6	 G 	   455.26	  1181.55	 10
  7	 Y 	   618.32	  1124.53	  9
  8	 D 	   733.35	   961.47	  8
  9	 V 	   832.42	   846.44	  7
 10	 E 	   961.46	   747.37	  6
 11	 K 	  1089.56	   618.33	  5
 12	 N 	  1203.60	   490.24	  4
 13	 N 	  1317.64	   376.19	  3
 14	 S 	  1404.68	   262.15	  2
 15	 R 	        -  	   175.12	  1  _
+2 charge
		      B   	       Y
  1	 A 	    36.53	        -  	 15
  2	 L 	    93.07	   754.38	 14
  3	 A 	   128.59	   697.84	 13
  4	 A 	   164.10	   662.32	 12
  5	 A 	   199.62	   626.80	 11
  6	 G 	   228.13	   591.28	 10
  7	 Y 	   309.67	   562.77	  9
  8	 D 	   367.18	   481.24	  8
  9	 V 	   416.71	   423.73	  7
 10	 E 	   481.23	   374.19	  6
 11	 K 	   545.28	   309.67	  5
 12	 N 	   602.30	   245.62	  4
 13	 N 	   659.33	   188.60	  3
 14	 S 	   702.84	   131.58	  2
 15	 R 	        -  	    88.06	  1  _
```

**16. IPI00017601 Ceruloplasmin**

| Sequence | Charge | M+H+ | XCorr | DeltaCn |
| --- | --- | --- | --- | --- |
| R.FNKNNEGTYYSPNYNPQSR.S | 3 | 2294.381 | 3.555 | 0.285 |

+1 charge

```
		      B   	       Y
  1	 F 	   148.08	        -  	 19
  2	 N 	   262.12	  2145.96	 18
  3	 K 	   390.21	  2031.92	 17
  4	 N 	   504.26	  1903.82	 16
  5	 N 	   618.30	  1789.78	 15
  6	 E 	   747.34	  1675.73	 14
  7	 G 	   804.36	  1546.69	 13
  8	 T 	   905.41	  1489.67	 12
  9	 Y 	  1068.47	  1388.62	 11
 10	 Y 	  1231.54	  1225.56	 10
 11	 S 	  1318.57	  1062.50	  9
 12	 P 	  1415.62	   975.46	  8
 13	 N 	  1529.67	   878.41	  7
 14	 Y 	  1692.73	   764.37	  6
 15	 N 	  1806.77	   601.31	  5
 16	 P 	  1903.82	   487.26	  4
 17	 Q 	  2031.88	   390.21	  3
 18	 S 	  2118.92	   262.15	  2
 19	 R 	        -  	   175.12	  1  _
+2 charge
		      B   	       Y
  1	 F 	    74.54	        -  	 19
  2	 N 	   131.56	  1073.48	 18
  3	 K 	   195.61	  1016.46	 17
  4	 N 	   252.63	   952.41	 16
  5	 N 	   309.65	   895.39	 15
  6	 E 	   374.17	   838.37	 14
  7	 G 	   402.69	   773.85	 13
  8	 T 	   453.21	   745.34	 12
  9	 Y 	   534.74	   694.82	 11
 10	 Y 	   616.27	   613.28	 10
 11	 S 	   659.79	   531.75	  9
 12	 P 	   708.31	   488.24	  8
 13	 N 	   765.34	   439.71	  7
 14	 Y 	   846.87	   382.69	  6
 15	 N 	   903.89	   301.16	  5
 16	 P 	   952.42	   244.13	  4
 17	 Q 	  1016.45	   195.61	  3
 18	 S 	  1059.96	   131.58	  2
 19	 R 	        -  	    88.06	  1  _


+3 charge
		      B   	       Y
  1	 F 	    50.03	        -  	 19
  2	 N 	    88.04	   715.99	 18
  3	 K 	   130.74	   677.98	 17
  4	 N 	   168.76	   635.28	 16
  5	 N 	   206.77	   597.26	 15
  6	 E 	   249.79	   559.25	 14
  7	 G 	   268.79	   516.24	 13
  8	 T 	   302.48	   497.23	 12
  9	 Y 	   356.83	   463.55	 11
 10	 Y 	   411.18	   409.19	 10
 11	 S 	   440.19	   354.84	  9
 12	 P 	   472.55	   325.83	  8
 13	 N 	   510.56	   293.48	  7
 14	 Y 	   564.91	   255.46	  6
 15	 N 	   602.93	   201.11	  5
 16	 P 	   635.28	   163.09	  4
 17	 Q 	   677.97	   130.74	  3
 18	 S 	   706.98	    88.06	  2
 19	 R 	        -  	    59.04	  1  _
```

**17. IPI00027409 Myeloblastin**

| Sequence | Charge | M+H+ | XCorr | DeltaCn |
| --- | --- | --- | --- | --- |
| R.LVNVVLGAHNVR.T | 2 | 1291.528 | 3.679 | 0.364 |

+1 charge

```
		      B   	       Y
  1	 L 	   114.09	        -  	 12
  2	 V 	   213.16	  1177.68	 11
  3	 N 	   327.20	  1078.61	 10
  4	 V 	   426.27	   964.57	  9
  5	 V 	   525.34	   865.50	  8
  6	 L 	   638.42	   766.43	  7
  7	 G 	   695.45	   653.35	  6
  8	 A 	   766.48	   596.33	  5
  9	 H 	   903.54	   525.29	  4
 10	 N 	  1017.58	   388.23	  3
 11	 V 	  1116.65	   274.19	  2
 12	 R 	        -  	   175.12	  1  _
+2 charge
		      B   	       Y
  1	 L 	    57.55	        -  	 12
  2	 V 	   107.08	   589.34	 11
  3	 N 	   164.10	   539.81	 10
  4	 V 	   213.64	   482.79	  9
  5	 V 	   263.17	   433.25	  8
  6	 L 	   319.72	   383.72	  7
  7	 G 	   348.23	   327.18	  6
  8	 A 	   383.74	   298.67	  5
  9	 H 	   452.27	   263.15	  4
 10	 N 	   509.30	   194.62	  3
 11	 V 	   558.83	   137.60	  2
 12	 R 	        -  	    88.06	  1  _
```

**18. IPI00784430 Similar to Ig kappa chain V-III region VG precursor**

| Sequence | Charge | M+H+ | XCorr | DeltaCn |
| --- | --- | --- | --- | --- |
| R.LLIYDASNR.A | 2 | 1065.205 | 3.142 | 0.435 |

+1 charge

```
		      B   	       Y
  1	 L 	   114.09	        -  	  9
  2	 L 	   227.18	   951.49	  8
  3	 I 	   340.26	   838.41	  7
  4	 Y 	   503.32	   725.32	  6
  5	 D 	   618.35	   562.26	  5
  6	 A 	   689.39	   447.23	  4
  7	 S 	   776.42	   376.19	  3
  8	 N 	   890.46	   289.16	  2
  9	 R 	        -  	   175.12	  1  _
+2 charge
		      B   	       Y
  1	 L 	    57.55	        -  	  9
  2	 L 	   114.09	   476.25	  8
  3	 I 	   170.63	   419.71	  7
  4	 Y 	   252.17	   363.16	  6
  5	 D 	   309.68	   281.63	  5
  6	 A 	   345.20	   224.12	  4
  7	 S 	   388.71	   188.60	  3
  8	 N 	   445.73	   145.08	  2
  9	 R 	        -  	    88.06	  1  _
```

**19. IPI00013808 Alpha-actinin-4**

| Sequence | Charge | M+H+ | XCorr | DeltaCn |
| --- | --- | --- | --- | --- |
| R.TINEVENQILTR.D | 2 | 1430.589 | 2.873 | 0.383 |

+1 charge

```
		      B   	       Y
  1	 T 	   102.05	        -  	 12
  2	 I 	   215.14	  1328.72	 11
  3	 N 	   329.18	  1215.63	 10
  4	 E 	   458.22	  1101.59	  9
  5	 V 	   557.29	   972.55	  8
  6	 E 	   686.34	   873.48	  7
  7	 N 	   800.38	   744.44	  6
  8	 Q 	   928.44	   630.39	  5
  9	 I 	  1041.52	   502.33	  4
 10	 L 	  1154.61	   389.25	  3
 11	 T 	  1255.65	   276.17	  2
 12	 R 	        -  	   175.12	  1  _
+2 charge
		      B   	       Y
  1	 T 	    51.53	        -  	 12
  2	 I 	   108.07	   664.86	 11
  3	 N 	   165.09	   608.32	 10
  4	 E 	   229.62	   551.30	  9
  5	 V 	   279.15	   486.78	  8
  6	 E 	   343.67	   437.24	  7
  7	 N 	   400.69	   372.72	  6
  8	 Q 	   464.72	   315.70	  5
  9	 I 	   521.26	   251.67	  4
 10	 L 	   577.81	   195.13	  3
 11	 T 	   628.33	   138.59	  2
 12	 R 	        -  	    88.06	  1  _
```

**20. IPI00056478 Isoform 1 of Immunoglobulin superfamily member 8**

| Sequence | Charge | M+H+ | XCorr | DeltaCn |
| --- | --- | --- | --- | --- |
| R.VVAGEVQVQRLQGDAVVLK.I | 2 | 2009.339 | 3.116 | 0.316 |

+1 charge

```
		      B   	       Y
  1	 V 	   100.08	        -  	 19
  2	 V 	   199.14	  1909.09	 18
  3	 A 	   270.18	  1810.02	 17
  4	 G 	   327.20	  1738.98	 16
  5	 E 	   456.25	  1681.96	 15
  6	 V 	   555.31	  1552.92	 14
  7	 Q 	   683.37	  1453.85	 13
  8	 V 	   782.44	  1325.79	 12
  9	 Q 	   910.50	  1226.72	 11
 10	 R 	  1066.60	  1098.66	 10
 11	 L 	  1179.68	   942.56	  9
 12	 Q 	  1307.74	   829.48	  8
 13	 G 	  1364.76	   701.42	  7
 14	 D 	  1479.79	   644.40	  6
 15	 A 	  1550.83	   529.37	  5
 16	 V 	  1649.90	   458.33	  4
 17	 V 	  1748.97	   359.27	  3
 18	 L 	  1862.05	   260.20	  2
 19	 K 	        -  	   147.11	  1  _
+2 charge
		      B   	       Y
  1	 V 	    50.54	        -  	 19
  2	 V 	   100.08	   955.05	 18
  3	 A 	   135.59	   905.51	 17
  4	 G 	   164.10	   869.99	 16
  5	 E 	   228.63	   841.48	 15
  6	 V 	   278.16	   776.96	 14
  7	 Q 	   342.19	   727.43	 13
  8	 V 	   391.72	   663.40	 12
  9	 Q 	   455.75	   613.86	 11
 10	 R 	   533.80	   549.84	 10
 11	 L 	   590.35	   471.78	  9
 12	 Q 	   654.38	   415.24	  8
 13	 G 	   682.89	   351.21	  7
 14	 D 	   740.40	   322.70	  6
 15	 A 	   775.92	   265.19	  5
 16	 V 	   825.45	   229.67	  4
 17	 V 	   874.99	   180.14	  3
 18	 L 	   931.53	   130.60	  2
 19	 K 	        -  	    74.06	  1  _
```

**21. IPI00299738 Procollagen C-endopeptidase enhancer 1**

| Sequence | Charge | M+H+ | XCorr | DeltaCn |
| --- | --- | --- | --- | --- |
| R.LGRFCGTFRPAPLVAPGNQVTLR.M | 3 | 2528.934 | 4.578 | 0.352 |

+1 charge

```
		      B   	       Y
  1	 L 	   114.09	        -  	 23
  2	 G 	   171.11	  2414.42	 22
  3	 R 	   327.21	  2357.40	 21
  4	 F 	   474.28	  2201.30	 20
  5	 C 	   634.45	  2054.23	 19
  6	 G 	   691.47	  1894.07	 18
  7	 T 	   792.52	  1837.04	 17
  8	 F 	   939.59	  1736.00	 16
  9	 R 	  1095.69	  1588.93	 15
 10	 P 	  1192.74	  1432.83	 14
 11	 A 	  1263.78	  1335.77	 13
 12	 P 	  1360.83	  1264.74	 12
 13	 L 	  1473.91	  1167.68	 11
 14	 V 	  1572.98	  1054.60	 10
 15	 A 	  1644.02	   955.53	  9
 16	 P 	  1741.07	   884.49	  8
 17	 G 	  1798.09	   787.44	  7
 18	 N 	  1912.14	   730.42	  6
 19	 Q 	  2040.19	   616.38	  5
 20	 V 	  2139.26	   488.32	  4
 21	 T 	  2240.31	   389.25	  3
 22	 L 	  2353.39	   288.20	  2
 23	 R 	        -  	   175.12	  1  _


+2 charge
		      B   	       Y
  1	 L 	    57.55	        -  	 23
  2	 G 	    86.06	  1207.71	 22
  3	 R 	   164.11	  1179.20	 21
  4	 F 	   237.64	  1101.15	 20
  5	 C 	   317.73	  1027.62	 19
  6	 G 	   346.24	   947.54	 18
  7	 T 	   396.76	   919.03	 17
  8	 F 	   470.30	   868.50	 16
  9	 R 	   548.35	   794.97	 15
 10	 P 	   596.87	   716.92	 14
 11	 A 	   632.39	   668.39	 13
 12	 P 	   680.92	   632.87	 12
 13	 L 	   737.46	   584.35	 11
 14	 V 	   786.99	   527.80	 10
 15	 A 	   822.51	   478.27	  9
 16	 P 	   871.04	   442.75	  8
 17	 G 	   899.55	   394.22	  7
 18	 N 	   956.57	   365.71	  6
 19	 Q 	  1020.60	   308.69	  5
 20	 V 	  1070.13	   244.66	  4
 21	 T 	  1120.66	   195.13	  3
 22	 L 	  1177.20	   144.61	  2
 23	 R 	        -  	    88.06	  1  _


+3 charge
		      B   	       Y
  1	 L 	    38.70	        -  	 23
  2	 G 	    57.71	   805.48	 22
  3	 R 	   109.74	   786.47	 21
  4	 F 	   158.77	   734.44	 20
  5	 C 	   212.15	   685.42	 19
  6	 G 	   231.16	   632.03	 18
  7	 T 	   264.84	   613.02	 17
  8	 F 	   313.87	   579.34	 16
  9	 R 	   365.90	   530.31	 15
 10	 P 	   398.25	   478.28	 14
 11	 A 	   421.93	   445.93	 13
 12	 P 	   454.28	   422.25	 12
 13	 L 	   491.98	   389.90	 11
 14	 V 	   525.00	   352.20	 10
 15	 A 	   548.68	   319.18	  9
 16	 P 	   581.03	   295.50	  8
 17	 G 	   600.04	   263.15	  7
 18	 N 	   638.05	   244.15	  6
 19	 Q 	   680.74	   206.13	  5
 20	 V 	   713.76	   163.44	  4
 21	 T 	   747.44	   130.42	  3
 22	 L 	   785.14	    96.74	  2
 23	 R 	        -  	    59.04	  1  _
```

**22. IPI00029658 Isoform 1 of EGF-containing fibulin-like extracellular matrix protein 1**

| Sequence | Charge | M+H+ | XCorr | DeltaCn |
| --- | --- | --- | --- | --- |
| K.CVNHYGGYLCLPK.T | 2 | 1581.784 | 3.828 | 0.45 |

+1 charge

```
		      B   	       Y
  1	 C 	   161.17	        -  	 13
  2	 V 	   260.24	  1420.84	 12
  3	 N 	   374.28	  1321.77	 11
  4	 H 	   511.34	  1207.73	 10
  5	 Y 	   674.41	  1070.67	  9
  6	 G 	   731.43	   907.61	  8
  7	 G 	   788.45	   850.58	  7
  8	 Y 	   951.51	   793.56	  6
  9	 L 	  1064.60	   630.50	  5
 10	 C 	  1224.76	   517.41	  4
 11	 L 	  1337.85	   357.25	  3
 12	 P 	  1434.90	   244.17	  2
 13	 K 	        -  	   147.11	  1  _
+2 charge
		      B   	       Y
  1	 C 	    81.09	        -  	 13
  2	 V 	   130.62	   710.92	 12
  3	 N 	   187.65	   661.39	 11
  4	 H 	   256.18	   604.37	 10
  5	 Y 	   337.71	   535.84	  9
  6	 G 	   366.22	   454.31	  8
  7	 G 	   394.73	   425.80	  7
  8	 Y 	   476.26	   397.28	  6
  9	 L 	   532.80	   315.75	  5
 10	 C 	   612.88	   259.21	  4
 11	 L 	   669.43	   179.13	  3
 12	 P 	   717.95	   122.59	  2
 13	 K 	        -  	    74.06	  1  _
```

**23. IPI00216065 Isoform 2 of Vitamin K-dependent protein Z**

| Sequence | Charge | M+H+ | XCorr | DeltaCn |
| --- | --- | --- | --- | --- |
| R.APDLQDLPWQVK.L | 2 | 1410.599 | 3.348 | 0.412 |

+1 charge

```
		      B   	       Y
  1	 A 	    72.04	        -  	 12
  2	 P 	   169.10	  1338.71	 11
  3	 D 	   284.12	  1241.65	 10
  4	 L 	   397.21	  1126.63	  9
  5	 Q 	   525.27	  1013.54	  8
  6	 D 	   640.29	   885.48	  7
  7	 L 	   753.38	   770.46	  6
  8	 P 	   850.43	   657.37	  5
  9	 W 	  1036.51	   560.32	  4
 10	 Q 	  1164.57	   374.24	  3
 11	 V 	  1263.64	   246.18	  2
 12	 K 	        -  	   147.11	  1  _
+2 charge
		      B   	       Y
  1	 A 	    36.53	        -  	 12
  2	 P 	    85.05	   669.86	 11
  3	 D 	   142.57	   621.33	 10
  4	 L 	   199.11	   563.82	  9
  5	 Q 	   263.14	   507.27	  8
  6	 D 	   320.65	   443.25	  7
  7	 L 	   377.19	   385.73	  6
  8	 P 	   425.72	   329.19	  5
  9	 W 	   518.76	   280.66	  4
 10	 Q 	   582.79	   187.62	  3
 11	 V 	   632.32	   123.59	  2
 12	 K 	        -  	    74.06	  1  _
```

**24. IPI00000828 Proenkephalin-A**

| Sequence | Charge | M+H+ | XCorr | DeltaCn |
| --- | --- | --- | --- | --- |
| K.DAEEDDSLANSSDLLKELLETGDNRER.S | 3 | 3036.123 | 3.4 | 0.303 |

+1 charge

```
		      B   	       Y
  1	 D 	   116.03	        -  	 27
  2	 A 	   187.07	  2919.38	 26
  3	 E 	   316.11	  2848.34	 25
  4	 E 	   445.16	  2719.30	 24
  5	 D 	   560.18	  2590.26	 23
  6	 D 	   675.21	  2475.23	 22
  7	 S 	   762.24	  2360.21	 21
  8	 L 	   875.33	  2273.17	 20
  9	 A 	   946.36	  2160.09	 19
 10	 N 	  1060.41	  2089.05	 18
 11	 S 	  1147.44	  1975.01	 17
 12	 S 	  1234.47	  1887.98	 16
 13	 D 	  1349.50	  1800.95	 15
 14	 L 	  1462.58	  1685.92	 14
 15	 L 	  1575.67	  1572.83	 13
 16	 K 	  1703.76	  1459.75	 12
 17	 E 	  1832.80	  1331.65	 11
 18	 L 	  1945.89	  1202.61	 10
 19	 L 	  2058.97	  1089.53	  9
 20	 E 	  2188.01	   976.44	  8
 21	 T 	  2289.06	   847.40	  7
 22	 G 	  2346.08	   746.35	  6
 23	 D 	  2461.11	   689.33	  5
 24	 N 	  2575.15	   574.31	  4
 25	 R 	  2731.25	   460.26	  3
 26	 E 	  2860.30	   304.16	  2
 27	 R 	        -  	   175.12	  1  _


+2 charge
		      B   	       Y
  1	 D 	    58.52	        -  	 27
  2	 A 	    94.04	  1460.19	 26
  3	 E 	   158.56	  1424.68	 25
  4	 E 	   223.08	  1360.15	 24
  5	 D 	   280.60	  1295.63	 23
  6	 D 	   338.11	  1238.12	 22
  7	 S 	   381.62	  1180.61	 21
  8	 L 	   438.17	  1137.09	 20
  9	 A 	   473.69	  1080.55	 19
 10	 N 	   530.71	  1045.03	 18
 11	 S 	   574.22	   988.01	 17
 12	 S 	   617.74	   944.49	 16
 13	 D 	   675.25	   900.98	 15
 14	 L 	   731.79	   843.46	 14
 15	 L 	   788.34	   786.92	 13
 16	 K 	   852.38	   730.38	 12
 17	 E 	   916.91	   666.33	 11
 18	 L 	   973.45	   601.81	 10
 19	 L 	  1029.99	   545.27	  9
 20	 E 	  1094.51	   488.73	  8
 21	 T 	  1145.03	   424.20	  7
 22	 G 	  1173.55	   373.68	  6
 23	 D 	  1231.06	   345.17	  5
 24	 N 	  1288.08	   287.66	  4
 25	 R 	  1366.13	   230.63	  3
 26	 E 	  1430.65	   152.58	  2
 27	 R 	        -  	    88.06	  1  _


+3 charge
		      B   	       Y
  1	 D 	    39.35	        -  	 27
  2	 A 	    63.03	   973.80	 26
  3	 E 	   106.04	   950.12	 25
  4	 E 	   149.06	   907.11	 24
  5	 D 	   187.40	   864.09	 23
  6	 D 	   225.74	   825.75	 22
  7	 S 	   254.75	   787.41	 21
  8	 L 	   292.45	   758.40	 20
  9	 A 	   316.13	   720.70	 19
 10	 N 	   354.14	   697.02	 18
 11	 S 	   383.15	   659.01	 17
 12	 S 	   412.16	   630.00	 16
 13	 D 	   450.50	   600.99	 15
 14	 L 	   488.20	   562.64	 14
 15	 L 	   525.89	   524.95	 13
 16	 K 	   568.59	   487.25	 12
 17	 E 	   611.61	   444.56	 11
 18	 L 	   649.30	   401.54	 10
 19	 L 	   687.00	   363.85	  9
 20	 E 	   730.01	   326.15	  8
 21	 T 	   763.69	   283.14	  7
 22	 G 	   782.70	   249.46	  6
 23	 D 	   821.04	   230.45	  5
 24	 N 	   859.06	   192.11	  4
 25	 R 	   911.09	   154.09	  3
 26	 E 	   954.10	   102.06	  2
 27	 R 	        -  	    59.04	  1  _
```

**25. IPI00646304 Peptidyl-prolyl cis-trans isomerase B**

| Sequence | Charge | M+H+ | XCorr | DeltaCn |
| --- | --- | --- | --- | --- |
| R.IGDEDVGRVIFGLFGK.T | 2 | 1722.966 | 2.823 | 0.526 |

+1 charge

```
		      B   	       Y
  1	 I 	   114.09	        -  	 16
  2	 G 	   171.11	  1608.84	 15
  3	 D 	   286.14	  1551.82	 14
  4	 E 	   415.18	  1436.79	 13
  5	 D 	   530.21	  1307.75	 12
  6	 V 	   629.28	  1192.72	 11
  7	 G 	   686.30	  1093.65	 10
  8	 R 	   842.40	  1036.63	  9
  9	 V 	   941.47	   880.53	  8
 10	 I 	  1054.55	   781.46	  7
 11	 F 	  1201.62	   668.38	  6
 12	 G 	  1258.64	   521.31	  5
 13	 L 	  1371.73	   464.29	  4
 14	 F 	  1518.80	   351.20	  3
 15	 G 	  1575.82	   204.13	  2
 16	 K 	        -  	   147.11	  1  _
+2 charge
		      B   	       Y
  1	 I 	    57.55	        -  	 16
  2	 G 	    86.06	   804.92	 15
  3	 D 	   143.57	   776.41	 14
  4	 E 	   208.09	   718.90	 13
  5	 D 	   265.61	   654.38	 12
  6	 V 	   315.14	   596.86	 11
  7	 G 	   343.65	   547.33	 10
  8	 R 	   421.70	   518.82	  9
  9	 V 	   471.24	   440.77	  8
 10	 I 	   527.78	   391.23	  7
 11	 F 	   601.31	   334.69	  6
 12	 G 	   629.82	   261.16	  5
 13	 L 	   686.37	   232.65	  4
 14	 F 	   759.90	   176.10	  3
 15	 G 	   788.41	   102.57	  2
 16	 K 	        -  	    74.06	  1  _
```

**26. IPI00306543 Growth/differentiation factor 15**

| Sequence | Charge | M+H+ | XCorr | DeltaCn |
| --- | --- | --- | --- | --- |
| R.ASLEDLGWADWVLSPR.E | 2 | 1816.006 | 4.421 | 0.449 |

+1 charge

```
		      B   	       Y
  1	 A 	    72.04	        -  	 16
  2	 S 	   159.08	  1743.87	 15
  3	 L 	   272.16	  1656.84	 14
  4	 E 	   401.20	  1543.75	 13
  5	 D 	   516.23	  1414.71	 12
  6	 L 	   629.31	  1299.68	 11
  7	 G 	   686.34	  1186.60	 10
  8	 W 	   872.41	  1129.58	  9
  9	 A 	   943.45	   943.50	  8
 10	 D 	  1058.48	   872.46	  7
 11	 W 	  1244.56	   757.44	  6
 12	 V 	  1343.63	   571.36	  5
 13	 L 	  1456.71	   472.29	  4
 14	 S 	  1543.74	   359.20	  3
 15	 P 	  1640.80	   272.17	  2
 16	 R 	        -  	   175.12	  1  _
+2 charge
		      B   	       Y
  1	 A 	    36.53	        -  	 16
  2	 S 	    80.04	   872.44	 15
  3	 L 	   136.58	   828.92	 14
  4	 E 	   201.11	   772.38	 13
  5	 D 	   258.62	   707.86	 12
  6	 L 	   315.16	   650.35	 11
  7	 G 	   343.67	   593.80	 10
  8	 W 	   436.71	   565.29	  9
  9	 A 	   472.23	   472.25	  8
 10	 D 	   529.74	   436.73	  7
 11	 W 	   622.78	   379.22	  6
 12	 V 	   672.32	   286.18	  5
 13	 L 	   728.86	   236.65	  4
 14	 S 	   772.38	   180.11	  3
 15	 P 	   820.90	   136.59	  2
 16	 R 	        -  	    88.06	  1  _
```

**27. IPI00292150 Latent-transforming growth factor beta-binding protein 2**

| Sequence | Charge | M+H+ | XCorr | DeltaCn |
| --- | --- | --- | --- | --- |
| R.CHCSPGYVAEAGPPHCTAKE.- | 3 | 2229.373 | 6.104 | 0.517 |

+1 charge

```
		      B   	       Y
  1	 C 	   161.17	        -  	 20
  2	 H 	   298.23	  2068.17	 19
  3	 C 	   458.40	  1931.11	 18
  4	 S 	   545.43	  1770.95	 17
  5	 P 	   642.48	  1683.91	 16
  6	 G 	   699.50	  1586.86	 15
  7	 Y 	   862.57	  1529.84	 14
  8	 V 	   961.63	  1366.78	 13
  9	 A 	  1032.67	  1267.71	 12
 10	 E 	  1161.71	  1196.67	 11
 11	 A 	  1232.75	  1067.63	 10
 12	 G 	  1289.77	   996.59	  9
 13	 P 	  1386.83	   939.57	  8
 14	 P 	  1483.88	   842.52	  7
 15	 H 	  1620.94	   745.46	  6
 16	 C 	  1781.10	   608.41	  5
 17	 T 	  1882.15	   448.24	  4
 18	 A 	  1953.19	   347.19	  3
 19	 K 	  2081.28	   276.16	  2
 20	 E 	        -  	   148.06	  1  _
+2 charge
		      B   	       Y
  1	 C 	    81.09	        -  	 20
  2	 H 	   149.62	  1034.59	 19
  3	 C 	   229.70	   966.06	 18
  4	 S 	   273.22	   885.98	 17
  5	 P 	   321.74	   842.46	 16
  6	 G 	   350.26	   793.93	 15
  7	 Y 	   431.79	   765.42	 14
  8	 V 	   481.32	   683.89	 13
  9	 A 	   516.84	   634.36	 12
 10	 E 	   581.36	   598.84	 11
 11	 A 	   616.88	   534.32	 10
 12	 G 	   645.39	   498.80	  9
 13	 P 	   693.92	   470.29	  8
 14	 P 	   742.44	   421.76	  7
 15	 H 	   810.97	   373.24	  6
 16	 C 	   891.05	   304.71	  5
 17	 T 	   941.58	   224.62	  4
 18	 A 	   977.10	   174.10	  3
 19	 K 	  1041.14	   138.58	  2
 20	 E 	        -  	    74.53	  1  _

+3 charge
		      B   	       Y
  1	 C 	    54.40	        -  	 20
  2	 H 	   100.08	   690.06	 19
  3	 C 	   153.47	   644.38	 18
  4	 S 	   182.48	   590.99	 17
  5	 P 	   214.83	   561.98	 16
  6	 G 	   233.84	   529.63	 15
  7	 Y 	   288.19	   510.62	 14
  8	 V 	   321.22	   456.26	 13
  9	 A 	   344.90	   423.24	 12
 10	 E 	   387.91	   399.56	 11
 11	 A 	   411.59	   356.55	 10
 12	 G 	   430.60	   332.87	  9
 13	 P 	   462.95	   313.86	  8
 14	 P 	   495.30	   281.51	  7
 15	 H 	   540.98	   249.16	  6
 16	 C 	   594.37	   203.47	  5
 17	 T 	   628.05	   150.08	  4
 18	 A 	   651.73	   116.40	  3
 19	 K 	   694.43	    92.72	  2
 20	 E 	        -  	    50.02	  1  _
```

**28. IPI00064377 Tumor necrosis factor receptor superfamily member 19L**

| Sequence | Charge | M+H+ | XCorr | DeltaCn |
| --- | --- | --- | --- | --- |
| R.RLEAQVGMATR.D | 2 | 1232.439 | 3.18 | 0.376 |

+1 charge

```
		      B   	       Y
  1	 R 	   157.11	        -  	 11
  2	 L 	   270.19	  1075.56	 10
  3	 E 	   399.24	   962.47	  9
  4	 A 	   470.27	   833.43	  8
  5	 Q 	   598.33	   762.39	  7
  6	 V 	   697.40	   634.33	  6
  7	 G 	   754.42	   535.27	  5
  8	 M 	   885.46	   478.24	  4
  9	 A 	   956.50	   347.20	  3
 10	 T 	  1057.55	   276.17	  2
 11	 R 	        -  	   175.12	  1  _
+2 charge
		      B   	       Y
  1	 R 	    79.06	        -  	 11
  2	 L 	   135.60	   538.28	 10
  3	 E 	   200.12	   481.74	  9
  4	 A 	   235.64	   417.22	  8
  5	 Q 	   299.67	   381.70	  7
  6	 V 	   349.20	   317.67	  6
  7	 G 	   377.71	   268.14	  5
  8	 M 	   443.23	   239.63	  4
  9	 A 	   478.75	   174.11	  3
 10	 T 	   529.28	   138.59	  2
 11	 R 	        -  	    88.06	  1  _
```

**29. IPI00003929 Glutathione S-transferase A3**

| Sequence | Charge | M+H+ | XCorr | DeltaCn |
| --- | --- | --- | --- | --- |
| K.SHGQDYLVGNKLSR.A | 2 | 1574.723 | 3.132 | 0.478 |

```
+1 charge
		      B   	       Y
  1	 S 	    88.04	        -  	 14
  2	 H 	   225.10	  1486.78	 13
  3	 G 	   282.12	  1349.72	 12
  4	 Q 	   410.18	  1292.70	 11
  5	 D 	   525.21	  1164.64	 10
  6	 Y 	   688.27	  1049.61	  9
  7	 L 	   801.35	   886.55	  8
  8	 V 	   900.42	   773.46	  7
  9	 G 	   957.44	   674.39	  6
 10	 N 	  1071.49	   617.37	  5
 11	 K 	  1199.58	   503.33	  4
 12	 L 	  1312.66	   375.24	  3
 13	 S 	  1399.70	   262.15	  2
 14	 R 	        -  	   175.12	  1  _
+2 charge
		      B   	       Y
  1	 S 	    44.52	        -  	 14
  2	 H 	   113.05	   743.89	 13
  3	 G 	   141.56	   675.36	 12
  4	 Q 	   205.59	   646.85	 11
  5	 D 	   263.11	   582.82	 10
  6	 Y 	   344.64	   525.31	  9
  7	 L 	   401.18	   443.78	  8
  8	 V 	   450.71	   387.24	  7
  9	 G 	   479.22	   337.70	  6
 10	 N 	   536.25	   309.19	  5
 11	 K 	   600.29	   252.17	  4
 12	 L 	   656.84	   188.12	  3
 13	 S 	   700.35	   131.58	  2
 14	 R 	        -  	    88.06	  1  _
```

**30. IPI00384404 Rheumatoid factor RF-ET9 (Fragment)**

| Sequence | Charge | M+H+ | XCorr | DeltaCn |
| --- | --- | --- | --- | --- |
| - .LVESGGGLVKPGGSLR.L | 2 | 1526.763 | 5.068 | 0.505 |

+1 charge

```
		      B   	       Y
  1	 L 	   114.09	        -  	 16
  2	 V 	   213.16	  1412.79	 15
  3	 E 	   342.20	  1313.72	 14
  4	 S 	   429.23	  1184.67	 13
  5	 G 	   486.26	  1097.64	 12
  6	 G 	   543.28	  1040.62	 11
  7	 G 	   600.30	   983.60	 10
  8	 L 	   713.38	   926.58	  9
  9	 V 	   812.45	   813.49	  8
 10	 K 	   940.55	   714.43	  7
 11	 P 	  1037.60	   586.33	  6
 12	 G 	  1094.62	   489.28	  5
 13	 G 	  1151.64	   432.26	  4
 14	 S 	  1238.67	   375.24	  3
 15	 L 	  1351.76	   288.20	  2
 16	 R 	        -  	   175.12	  1  _
+2 charge
		      B   	       Y
  1	 L 	    57.55	        -  	 16
  2	 V 	   107.08	   706.90	 15
  3	 E 	   171.60	   657.36	 14
  4	 S 	   215.12	   592.84	 13
  5	 G 	   243.63	   549.32	 12
  6	 G 	   272.14	   520.81	 11
  7	 G 	   300.65	   492.30	 10
  8	 L 	   357.20	   463.79	  9
  9	 V 	   406.73	   407.25	  8
 10	 K 	   470.78	   357.72	  7
 11	 P 	   519.30	   293.67	  6
 12	 G 	   547.81	   245.14	  5
 13	 G 	   576.32	   216.63	  4
 14	 S 	   619.84	   188.12	  3
 15	 L 	   676.38	   144.61	  2
 16	 R 	        -  	    88.06	  1  _
```

**31. IPI00009865 Keratin, type I cytoskeletal 10**

| Sequence | Charge | M+H+ | XCorr | DeltaCn |
| --- | --- | --- | --- | --- |
| K.IRLENEIQTYR.S | 2 | 1435.61 | 2.949 | 0.324 |

+1 charge

```
		      B   	       Y
  1	 I 	   114.09	        -  	 11
  2	 R 	   270.19	  1321.69	 10
  3	 L 	   383.28	  1165.58	  9
  4	 E 	   512.32	  1052.50	  8
  5	 N 	   626.36	   923.46	  7
  6	 E 	   755.40	   809.42	  6
  7	 I 	   868.49	   680.37	  5
  8	 Q 	   996.55	   567.29	  4
  9	 T 	  1097.59	   439.23	  3
 10	 Y 	  1260.66	   338.18	  2
 11	 R 	        -  	   175.12	  1  _
+2 charge
		      B   	       Y
  1	 I 	    57.55	        -  	 11
  2	 R 	   135.60	   661.35	 10
  3	 L 	   192.14	   583.30	  9
  4	 E 	   256.66	   526.75	  8
  5	 N 	   313.68	   462.23	  7
  6	 E 	   378.21	   405.21	  6
  7	 I 	   434.75	   340.69	  5
  8	 Q 	   498.78	   284.15	  4
  9	 T 	   549.30	   220.12	  3
 10	 Y 	   630.83	   169.59	  2
 11	 R 	        -  	    88.06	  1  _
```

**32. IPI00916111 Malate dehydrogenase**

| Sequence | Charge | M+H+ | XCorr | DeltaCn |
| --- | --- | --- | --- | --- |
| K.VIVVGNPANTNCLTASK.S | 2 | 1758.991 | 2.941 | 0.493 |

+1 charge

```
		      B   	       Y
  1	 V 	   100.08	        -  	 17
  2	 I 	   213.16	  1658.99	 16
  3	 V 	   312.23	  1545.90	 15
  4	 V 	   411.30	  1446.84	 14
  5	 G 	   468.32	  1347.77	 13
  6	 N 	   582.36	  1290.75	 12
  7	 P 	   679.41	  1176.70	 11
  8	 A 	   750.45	  1079.65	 10
  9	 N 	   864.49	  1008.61	  9
 10	 T 	   965.54	   894.57	  8
 11	 N 	  1079.58	   793.52	  7
 12	 C 	  1239.75	   679.48	  6
 13	 L 	  1352.83	   519.31	  5
 14	 T 	  1453.88	   406.23	  4
 15	 A 	  1524.92	   305.18	  3
 16	 S 	  1611.95	   234.14	  2
 17	 K 	        -  	   147.11	  1  _
+2 charge
		      B   	       Y
  1	 V 	    50.54	        -  	 17
  2	 I 	   107.08	   830.00	 16
  3	 V 	   156.62	   773.46	 15
  4	 V 	   206.15	   723.92	 14
  5	 G 	   234.66	   674.39	 13
  6	 N 	   291.68	   645.88	 12
  7	 P 	   340.21	   588.85	 11
  8	 A 	   375.73	   540.33	 10
  9	 N 	   432.75	   504.81	  9
 10	 T 	   483.27	   447.79	  8
 11	 N 	   540.30	   397.26	  7
 12	 C 	   620.38	   340.24	  6
 13	 L 	   676.92	   260.16	  5
 14	 T 	   727.44	   203.62	  4
 15	 A 	   762.96	   153.09	  3
 16	 S 	   806.48	   117.58	  2
 17	 K 	        -  	    74.06	  1  _
```

**33. IPI00011140 Protein NOV homolog**

| Sequence | Charge | M+H+ | XCorr | DeltaCn |
| --- | --- | --- | --- | --- |
| R.AVLDGCSCCLVCAR.Q | 2 | 1641.823 | 4.083 | 0.556 |

+1 charge

```
		      B   	       Y
  1	 A 	    72.04	        -  	 14
  2	 V 	   171.11	  1570.20	 13
  3	 L 	   284.20	  1471.13	 12
  4	 D 	   399.22	  1358.05	 11
  5	 G 	   456.25	  1243.02	 10
  6	 C 	   616.41	  1186.00	  9
  7	 S 	   703.44	  1025.84	  8
  8	 C 	   863.61	   938.80	  7
  9	 C 	  1023.77	   778.64	  6
 10	 L 	  1136.86	   618.47	  5
 11	 V 	  1235.93	   505.39	  4
 12	 C 	  1396.09	   406.32	  3
 13	 A 	  1467.13	   246.16	  2
 14	 R 	        -  	   175.12	  1  _
+2 charge
		      B   	       Y
  1	 A 	    36.53	        -  	 14
  2	 V 	    86.06	   785.60	 13
  3	 L 	   142.60	   736.07	 12
  4	 D 	   200.12	   679.53	 11
  5	 G 	   228.63	   622.02	 10
  6	 C 	   308.71	   593.50	  9
  7	 S 	   352.22	   513.42	  8
  8	 C 	   432.31	   469.91	  7
  9	 C 	   512.39	   389.82	  6
 10	 L 	   568.93	   309.74	  5
 11	 V 	   618.47	   253.20	  4
 12	 C 	   698.55	   203.66	  3
 13	 A 	   734.07	   123.58	  2
 14	 R 	        -  	    88.06	  1  _
```

**34. IPI00019954 Cystatin-M**

| Sequence | Charge | M+H+ | XCorr | DeltaCn |
| --- | --- | --- | --- | --- |
| R.VTGDHVDLTTCPLAAGAQQEKLR.C | 3 | 2481.741 | 5.601 | 0.599 |

+1 charge

```
		      B   	       Y
  1	 V 	   100.08	        -  	 23
  2	 T 	   201.12	  2381.32	 22
  3	 G 	   258.14	  2280.27	 21
  4	 D 	   373.17	  2223.25	 20
  5	 H 	   510.23	  2108.23	 19
  6	 V 	   609.30	  1971.17	 18
  7	 D 	   724.33	  1872.10	 17
  8	 L 	   837.41	  1757.07	 16
  9	 T 	   938.46	  1643.99	 15
 10	 T 	  1039.51	  1542.94	 14
 11	 C 	  1199.67	  1441.89	 13
 12	 P 	  1296.72	  1281.73	 12
 13	 L 	  1409.81	  1184.67	 11
 14	 A 	  1480.84	  1071.59	 10
 15	 A 	  1551.88	  1000.55	  9
 16	 G 	  1608.90	   929.52	  8
 17	 A 	  1679.94	   872.49	  7
 18	 Q 	  1808.00	   801.46	  6
 19	 Q 	  1936.06	   673.40	  5
 20	 E 	  2065.10	   545.34	  4
 21	 K 	  2193.20	   416.30	  3
 22	 L 	  2306.28	   288.20	  2
 23	 R 	        -  	   175.12	  1  _


+2 charge
		      B   	       Y
  1	 V 	    50.54	        -  	 23
  2	 T 	   101.07	  1191.16	 22
  3	 G 	   129.58	  1140.64	 21
  4	 D 	   187.09	  1112.13	 20
  5	 H 	   255.62	  1054.62	 19
  6	 V 	   305.15	   986.09	 18
  7	 D 	   362.67	   936.55	 17
  8	 L 	   419.21	   879.04	 16
  9	 T 	   469.73	   822.50	 15
 10	 T 	   520.26	   771.97	 14
 11	 C 	   600.34	   721.45	 13
 12	 P 	   648.87	   641.37	 12
 13	 L 	   705.41	   592.84	 11
 14	 A 	   740.93	   536.30	 10
 15	 A 	   776.44	   500.78	  9
 16	 G 	   804.96	   465.26	  8
 17	 A 	   840.47	   436.75	  7
 18	 Q 	   904.50	   401.23	  6
 19	 Q 	   968.53	   337.20	  5
 20	 E 	  1033.05	   273.17	  4
 21	 K 	  1097.10	   208.65	  3
 22	 L 	  1153.64	   144.61	  2
 23	 R 	        -  	    88.06	  1  _
+3 charge
		      B   	       Y
  1	 V 	    34.03	        -  	 23
  2	 T 	    67.71	   794.45	 22
  3	 G 	    86.72	   760.76	 21
  4	 D 	   125.06	   741.76	 20
  5	 H 	   170.75	   703.41	 19
  6	 V 	   203.77	   657.73	 18
  7	 D 	   242.11	   624.70	 17
  8	 L 	   279.81	   586.36	 16
  9	 T 	   313.49	   548.67	 15
 10	 T 	   347.17	   514.98	 14
 11	 C 	   400.56	   481.30	 13
 12	 P 	   432.91	   427.91	 12
 13	 L 	   470.61	   395.56	 11
 14	 A 	   494.29	   357.87	 10
 15	 A 	   517.97	   334.19	  9
 16	 G 	   536.97	   310.51	  8
 17	 A 	   560.65	   291.50	  7
 18	 Q 	   603.34	   267.82	  6
 19	 Q 	   646.02	   225.14	  5
 20	 E 	   689.04	   182.45	  4
 21	 K 	   731.74	   139.44	  3
 22	 L 	   769.43	    96.74	  2
 23	 R 	        -  	    59.04	  1  _
```

**35. IPI00384401 Myosin-reactive immunoglobulin kappa chain variable region (Fragment)**

| Sequence | Charge | M+H+ | XCorr | DeltaCn |
| --- | --- | --- | --- | --- |
| - .EIVMTQSPATLSVSPGER.A | 2 | 1903.147 | 5.059 | 0.59 |

+1 charge

```
		      B   	       Y
  1	 E 	   130.05	        -  	 18
  2	 I 	   243.13	  1772.92	 17
  3	 V 	   342.20	  1659.84	 16
  4	 M 	   473.24	  1560.77	 15
  5	 T 	   574.29	  1429.73	 14
  6	 Q 	   702.35	  1328.68	 13
  7	 S 	   789.38	  1200.62	 12
  8	 P 	   886.43	  1113.59	 11
  9	 A 	   957.47	  1016.54	 10
 10	 T 	  1058.52	   945.50	  9
 11	 L 	  1171.60	   844.45	  8
 12	 S 	  1258.63	   731.37	  7
 13	 V 	  1357.70	   644.34	  6
 14	 S 	  1444.74	   545.27	  5
 15	 P 	  1541.79	   458.24	  4
 16	 G 	  1598.81	   361.18	  3
 17	 E 	  1727.85	   304.16	  2
 18	 R 	        -  	   175.12	  1  _
+2 charge
		      B   	       Y
  1	 E 	    65.53	        -  	 18
  2	 I 	   122.07	   886.96	 17
  3	 V 	   171.60	   830.42	 16
  4	 M 	   237.13	   780.89	 15
  5	 T 	   287.65	   715.37	 14
  6	 Q 	   351.68	   664.84	 13
  7	 S 	   395.19	   600.81	 12
  8	 P 	   443.72	   557.30	 11
  9	 A 	   479.24	   508.77	 10
 10	 T 	   529.76	   473.25	  9
 11	 L 	   586.31	   422.73	  8
 12	 S 	   629.82	   366.19	  7
 13	 V 	   679.36	   322.67	  6
 14	 S 	   722.87	   273.14	  5
 15	 P 	   771.40	   229.62	  4
 16	 G 	   799.91	   181.10	  3
 17	 E 	   864.43	   152.58	  2
 18	 R 	        -  	    88.06	  1  _
```

**36. IPI00297030 Transmembrane protease serine 11E**

| Sequence | Charge | M+H+ | XCorr | DeltaCn |
| --- | --- | --- | --- | --- |
| R.VTALRDWITSK.T | 2 | 1290.493 | 3.039 | 0.326 |

+1 charge

```
		      B   	       Y
  1	 V 	   100.08	        -  	 11
  2	 T 	   201.12	  1190.65	 10
  3	 A 	   272.16	  1089.61	  9
  4	 L 	   385.24	  1018.57	  8
  5	 R 	   541.35	   905.48	  7
  6	 D 	   656.37	   749.38	  6
  7	 W 	   842.45	   634.36	  5
  8	 I 	   955.54	   448.28	  4
  9	 T 	  1056.58	   335.19	  3
 10	 S 	  1143.62	   234.14	  2
 11	 K 	        -  	   147.11	  1  _
+2 charge
		      B   	       Y
  1	 V 	    50.54	        -  	 11
  2	 T 	   101.07	   595.83	 10
  3	 A 	   136.58	   545.31	  9
  4	 L 	   193.13	   509.79	  8
  5	 R 	   271.18	   453.25	  7
  6	 D 	   328.69	   375.20	  6
  7	 W 	   421.73	   317.68	  5
  8	 I 	   478.27	   224.64	  4
  9	 T 	   528.80	   168.10	  3
 10	 S 	   572.31	   117.58	  2
 11	 K 	        -  	    74.06	  1  _
```

**37. IPI00027983 Cytidine deaminase**

| Sequence | Charge | M+H+ | XCorr | DeltaCn |
| --- | --- | --- | --- | --- |
| R.IFKGCNIENACYPLGICAER.T | 3 | 2386.67 | 3.772 | 0.38 |

+1 charge

```
		      B   	       Y
  1	 I 	   114.09	        -  	 20
  2	 F 	   261.16	  2272.43	 19
  3	 K 	   389.25	  2125.37	 18
  4	 G 	   446.28	  1997.27	 17
  5	 C 	   606.44	  1940.25	 16
  6	 N 	   720.48	  1780.08	 15
  7	 I 	   833.57	  1666.04	 14
  8	 E 	   962.61	  1552.96	 13
  9	 N 	  1076.65	  1423.91	 12
 10	 A 	  1147.69	  1309.87	 11
 11	 C 	  1307.86	  1238.83	 10
 12	 Y 	  1470.92	  1078.67	  9
 13	 P 	  1567.97	   915.61	  8
 14	 L 	  1681.06	   818.55	  7
 15	 G 	  1738.08	   705.47	  6
 16	 I 	  1851.16	   648.45	  5
 17	 C 	  2011.33	   535.36	  4
 18	 A 	  2082.36	   375.20	  3
 19	 E 	  2211.41	   304.16	  2
 20	 R 	        -  	   175.12	  1  _
+2 charge
		      B   	       Y
  1	 I 	    57.55	        -  	 20
  2	 F 	   131.08	  1136.72	 19
  3	 K 	   195.13	  1063.19	 18
  4	 G 	   223.64	   999.14	 17
  5	 C 	   303.72	   970.63	 16
  6	 N 	   360.75	   890.55	 15
  7	 I 	   417.29	   833.52	 14
  8	 E 	   481.81	   776.98	 13
  9	 N 	   538.83	   712.46	 12
 10	 A 	   574.35	   655.44	 11
 11	 C 	   654.43	   619.92	 10
 12	 Y 	   735.96	   539.84	  9
 13	 P 	   784.49	   458.31	  8
 14	 L 	   841.03	   409.78	  7
 15	 G 	   869.54	   353.24	  6
 16	 I 	   926.08	   324.73	  5
 17	 C 	  1006.17	   268.19	  4
 18	 A 	  1041.69	   188.10	  3
 19	 E 	  1106.21	   152.58	  2
 20	 R 	        -  	    88.06	  1  _

+3 charge
		      B   	       Y
  1	 I 	    38.70	        -  	 20
  2	 F 	    87.72	   758.15	 19
  3	 K 	   130.42	   709.13	 18
  4	 G 	   149.43	   666.43	 17
  5	 C 	   202.82	   647.42	 16
  6	 N 	   240.83	   594.03	 15
  7	 I 	   278.53	   556.02	 14
  8	 E 	   321.54	   518.32	 13
  9	 N 	   359.56	   475.31	 12
 10	 A 	   383.24	   437.30	 11
 11	 C 	   436.62	   413.62	 10
 12	 Y 	   490.98	   360.23	  9
 13	 P 	   523.33	   305.87	  8
 14	 L 	   561.02	   273.52	  7
 15	 G 	   580.03	   235.83	  6
 16	 I 	   617.73	   216.82	  5
 17	 C 	   671.11	   179.13	  4
 18	 A 	   694.79	   125.74	  3
 19	 E 	   737.81	   102.06	  2
 20	 R 	        -  	    59.04	  1  _
```

**38. IPI00024331 Tumor necrosis factor receptor superfamily member 14**

| Sequence | Charge | M+H+ | XCorr | DeltaCn |
| --- | --- | --- | --- | --- |
| R.VKEACGELTGTVCEPCPPGTYIAHLNGLSK.C | 3 | 3260.621 | 3.36 | 0.577 |

+1 charge

```
		      B   	       Y
  1	 V 	   100.08	        -  	 30
  2	 K 	   228.17	  3159.89	 29
  3	 E 	   357.21	  3031.80	 28
  4	 A 	   428.25	  2902.76	 27
  5	 C 	   588.42	  2831.72	 26
  6	 G 	   645.44	  2671.55	 25
  7	 E 	   774.48	  2614.53	 24
  8	 L 	   887.56	  2485.49	 23
  9	 T 	   988.61	  2372.41	 22
 10	 G 	  1045.63	  2271.36	 21
 11	 T 	  1146.68	  2214.34	 20
 12	 V 	  1245.75	  2113.29	 19
 13	 C 	  1405.91	  2014.22	 18
 14	 E 	  1534.96	  1854.06	 17
 15	 P 	  1632.01	  1725.01	 16
 16	 C 	  1792.17	  1627.96	 15
 17	 P 	  1889.23	  1467.80	 14
 18	 P 	  1986.28	  1370.74	 13
 19	 G 	  2043.30	  1273.69	 12
 20	 T 	  2144.35	  1216.67	 11
 21	 Y 	  2307.41	  1115.62	 10
 22	 I 	  2420.50	   952.56	  9
 23	 A 	  2491.53	   839.47	  8
 24	 H 	  2628.59	   768.44	  7
 25	 L 	  2741.68	   631.38	  6
 26	 N 	  2855.72	   518.29	  5
 27	 G 	  2912.74	   404.25	  4
 28	 L 	  3025.83	   347.23	  3
 29	 S 	  3112.86	   234.14	  2
 30	 K 	        -  	   147.11	  1  _


+2 charge
		      B   	       Y
  1	 V 	    50.54	        -  	 30
  2	 K 	   114.59	  1580.45	 29
  3	 E 	   179.11	  1516.40	 28
  4	 A 	   214.63	  1451.88	 27
  5	 C 	   294.71	  1416.36	 26
  6	 G 	   323.22	  1336.28	 25
  7	 E 	   387.74	  1307.77	 24
  8	 L 	   444.29	  1243.25	 23
  9	 T 	   494.81	  1186.71	 22
 10	 G 	   523.32	  1136.18	 21
 11	 T 	   573.84	  1107.67	 20
 12	 V 	   623.38	  1057.15	 19
 13	 C 	   703.46	  1007.61	 18
 14	 E 	   767.98	   927.53	 17
 15	 P 	   816.51	   863.01	 16
 16	 C 	   896.59	   814.48	 15
 17	 P 	   945.12	   734.40	 14
 18	 P 	   993.64	   685.87	 13
 19	 G 	  1022.15	   637.35	 12
 20	 T 	  1072.68	   608.84	 11
 21	 Y 	  1154.21	   558.31	 10
 22	 I 	  1210.75	   476.78	  9
 23	 A 	  1246.27	   420.24	  8
 24	 H 	  1314.80	   384.72	  7
 25	 L 	  1371.34	   316.19	  6
 26	 N 	  1428.36	   259.65	  5
 27	 G 	  1456.87	   202.63	  4
 28	 L 	  1513.42	   174.12	  3
 29	 S 	  1556.93	   117.58	  2
 30	 K 	        -  	    74.06	  1  _


+3 charge
		      B   	       Y
  1	 V 	    34.03	        -  	 30
  2	 K 	    76.73	  1053.97	 29
  3	 E 	   119.74	  1011.27	 28
  4	 A 	   143.42	   968.26	 27
  5	 C 	   196.81	   944.58	 26
  6	 G 	   215.82	   891.19	 25
  7	 E 	   258.83	   872.18	 24
  8	 L 	   296.53	   829.17	 23
  9	 T 	   330.21	   791.47	 22
 10	 G 	   349.22	   757.79	 21
 11	 T 	   382.90	   738.78	 20
 12	 V 	   415.92	   705.10	 19
 13	 C 	   469.31	   672.08	 18
 14	 E 	   512.32	   618.69	 17
 15	 P 	   544.67	   575.68	 16
 16	 C 	   598.06	   543.33	 15
 17	 P 	   630.41	   489.94	 14
 18	 P 	   662.76	   457.59	 13
 19	 G 	   681.77	   425.23	 12
 20	 T 	   715.45	   406.23	 11
 21	 Y 	   769.81	   372.55	 10
 22	 I 	   807.50	   318.19	  9
 23	 A 	   831.18	   280.50	  8
 24	 H 	   876.87	   256.82	  7
 25	 L 	   914.56	   211.13	  6
 26	 N 	   952.58	   173.44	  5
 27	 G 	   971.59	   135.42	  4
 28	 L 	  1009.28	   116.41	  3
 29	 S 	  1038.29	    78.72	  2
 30	 K 	        -  	    49.71	  1  _
```

**39. IPI00410487 Isoform 1 of Twisted gastrulation protein homolog 1**

| Sequence | Charge | M+H+ | XCorr | DeltaCn |
| --- | --- | --- | --- | --- |
| K.STVEELHEPIPSLFR.A | 2 | 1754.964 | 4.262 | 0.566 |

+1 charge

```
		      B   	       Y
  1	 S 	    88.04	        -  	 15
  2	 T 	   189.09	  1666.88	 14
  3	 V 	   288.16	  1565.83	 13
  4	 E 	   417.20	  1466.76	 12
  5	 E 	   546.24	  1337.72	 11
  6	 L 	   659.32	  1208.68	 10
  7	 H 	   796.38	  1095.59	  9
  8	 E 	   925.43	   958.54	  8
  9	 P 	  1022.48	   829.49	  7
 10	 I 	  1135.56	   732.44	  6
 11	 P 	  1232.62	   619.36	  5
 12	 S 	  1319.65	   522.30	  4
 13	 L 	  1432.73	   435.27	  3
 14	 F 	  1579.80	   322.19	  2
 15	 R 	        -  	   175.12	  1  _
+2 charge
		      B   	       Y
  1	 S 	    44.52	        -  	 15
  2	 T 	    95.05	   833.94	 14
  3	 V 	   144.58	   783.42	 13
  4	 E 	   209.10	   733.89	 12
  5	 E 	   273.62	   669.36	 11
  6	 L 	   330.17	   604.84	 10
  7	 H 	   398.70	   548.30	  9
  8	 E 	   463.22	   479.77	  8
  9	 P 	   511.74	   415.25	  7
 10	 I 	   568.29	   366.72	  6
 11	 P 	   616.81	   310.18	  5
 12	 S 	   660.33	   261.66	  4
 13	 L 	   716.87	   218.14	  3
 14	 F 	   790.40	   161.60	  2
 15	 R 	        -  	    88.06	  1  _
```

**40. IPI00019988 N-sulphoglucosamine sulphohydrolase**

| Sequence | Charge | M+H+ | | XCorr | DeltaCn |
| --- | --- | --- | --- | --- | --- |
| R.SLPLLLSQAGVR.T | 2 | | 1254.504 | 2.824 | 0.444 |

+1 charge

```
		      B   	       Y
  1	 S 	    88.04	        -  	 12
  2	 L 	   201.12	  1166.73	 11
  3	 P 	   298.18	  1053.64	 10
  4	 L 	   411.26	   956.59	  9
  5	 L 	   524.34	   843.50	  8
  6	 L 	   637.43	   730.42	  7
  7	 S 	   724.46	   617.34	  6
  8	 Q 	   852.52	   530.30	  5
  9	 A 	   923.56	   402.25	  4
 10	 G 	   980.58	   331.21	  3
 11	 V 	  1079.65	   274.19	  2
 12	 R 	        -  	   175.12	  1  _
+2 charge
		      B   	       Y
  1	 S 	    44.52	        -  	 12
  2	 L 	   101.07	   583.87	 11
  3	 P 	   149.59	   527.32	 10
  4	 L 	   206.13	   478.80	  9
  5	 L 	   262.68	   422.26	  8
  6	 L 	   319.22	   365.71	  7
  7	 S 	   362.73	   309.17	  6
  8	 Q 	   426.76	   265.66	  5
  9	 A 	   462.28	   201.63	  4
 10	 G 	   490.79	   166.11	  3
 11	 V 	   540.33	   137.60	  2
 12	 R 	        -  	    88.06	  1  _
```

**41. IPI00025861 Cadherin-1**

| Sequence | Charge | M+H+ | XCorr | DeltaCn |
| --- | --- | --- | --- | --- |
| K.MALEVGDYKINLK.L | 2 | 1494.781 | 3.815 | 0.419 |

+1 charge

```
		      B   	       Y
  1	 M 	   132.05	        -  	 13
  2	 A 	   203.08	  1362.76	 12
  3	 L 	   316.17	  1291.73	 11
  4	 E 	   445.21	  1178.64	 10
  5	 V 	   544.28	  1049.60	  9
  6	 G 	   601.30	   950.53	  8
  7	 D 	   716.33	   893.51	  7
  8	 Y 	   879.39	   778.48	  6
  9	 K 	  1007.49	   615.42	  5
 10	 I 	  1120.57	   487.32	  4
 11	 N 	  1234.61	   374.24	  3
 12	 L 	  1347.70	   260.20	  2
 13	 K 	        -  	   147.11	  1  _
+2 charge
		      B   	       Y
  1	 M 	    66.53	        -  	 13
  2	 A 	   102.05	   681.89	 12
  3	 L 	   158.59	   646.37	 11
  4	 E 	   223.11	   589.82	 10
  5	 V 	   272.64	   525.30	  9
  6	 G 	   301.15	   475.77	  8
  7	 D 	   358.67	   447.26	  7
  8	 Y 	   440.20	   389.74	  6
  9	 K 	   504.25	   308.21	  5
 10	 I 	   560.79	   244.17	  4
 11	 N 	   617.81	   187.62	  3
 12	 L 	   674.35	   130.60	  2
 13	 K 	        -  	    74.06	  1  _
```

**42. IPI00021841 Isoform 1 of Gamma-glutamyltransferase 6**

| Sequence | Charge | M+H+ | XCorr | DeltaCn |
| --- | --- | --- | --- | --- |
| K.LLDNWDSVTSTFSK.L | 2 | 1613.75 | 3.551 | 0.519 |

+1 charge

```
		      B   	       Y
  1	 L 	   114.09	        -  	 14
  2	 L 	   227.18	  1499.70	 13
  3	 D 	   342.20	  1386.62	 12
  4	 N 	   456.25	  1271.59	 11
  5	 W 	   642.32	  1157.55	 10
  6	 D 	   757.35	   971.47	  9
  7	 S 	   844.38	   856.44	  8
  8	 V 	   943.45	   769.41	  7
  9	 T 	  1044.50	   670.34	  6
 10	 S 	  1131.53	   569.29	  5
 11	 T 	  1232.58	   482.26	  4
 12	 F 	  1379.65	   381.21	  3
 13	 S 	  1466.68	   234.14	  2
 14	 K 	        -  	   147.11	  1  _
+2 charge
		      B   	       Y
  1	 L 	    57.55	        -  	 14
  2	 L 	   114.09	   750.35	 13
  3	 D 	   171.60	   693.81	 12
  4	 N 	   228.63	   636.30	 11
  5	 W 	   321.67	   579.28	 10
  6	 D 	   379.18	   486.24	  9
  7	 S 	   422.70	   428.72	  8
  8	 V 	   472.23	   385.21	  7
  9	 T 	   522.75	   335.67	  6
 10	 S 	   566.27	   285.15	  5
 11	 T 	   616.79	   241.63	  4
 12	 F 	   690.33	   191.11	  3
 13	 S 	   733.84	   117.58	  2
 14	 K 	        -  	    74.06	  1  _
```

**43. IPI00647357 Isoform 1 of Osteoclast-associated immunoglobulin-like receptor**

| Sequence | Charge | M+H+ | XCorr | DeltaCn |
| --- | --- | --- | --- | --- |
| R.HSAQPWADFTLLGAR.A | 2 | 1670.853 | 3.402 | 0.434 |

+1 charge

```
		      B   	       Y
  1	 H 	   138.07	        -  	 15
  2	 S 	   225.10	  1532.79	 14
  3	 A 	   296.14	  1445.75	 13
  4	 Q 	   424.19	  1374.72	 12
  5	 P 	   521.25	  1246.66	 11
  6	 W 	   707.33	  1149.61	 10
  7	 A 	   778.36	   963.53	  9
  8	 D 	   893.39	   892.49	  8
  9	 F 	  1040.46	   777.46	  7
 10	 T 	  1141.51	   630.39	  6
 11	 L 	  1254.59	   529.35	  5
 12	 L 	  1367.67	   416.26	  4
 13	 G 	  1424.70	   303.18	  3
 14	 A 	  1495.73	   246.16	  2
 15	 R 	        -  	   175.12	  1  _
+2 charge
		      B   	       Y
  1	 H 	    69.54	        -  	 15
  2	 S 	   113.05	   766.90	 14
  3	 A 	   148.57	   723.38	 13
  4	 Q 	   212.60	   687.86	 12
  5	 P 	   261.13	   623.83	 11
  6	 W 	   354.17	   575.31	 10
  7	 A 	   389.69	   482.27	  9
  8	 D 	   447.20	   446.75	  8
  9	 F 	   520.73	   389.23	  7
 10	 T 	   571.26	   315.70	  6
 11	 L 	   627.80	   265.18	  5
 12	 L 	   684.34	   208.63	  4
 13	 G 	   712.85	   152.09	  3
 14	 A 	   748.37	   123.58	  2
 15	 R 	        -  	    88.06	  1  _
```

**44. IPI00009276 Endothelial protein C receptor precursor**

| Sequence | Charge | M+H+ | XCorr | DeltaCn |
| --- | --- | --- | --- | --- |
| R.TQSGLQSYLLQFHGLVR.L | 2 | 1948.214 | 4.11 | 0.5 |

+1 charge

```
		      B   	       Y
  1	 T 	   102.05	        -  	 17
  2	 Q 	   230.11	  1846.00	 16
  3	 S 	   317.15	  1717.94	 15
  4	 G 	   374.17	  1630.91	 14
  5	 L 	   487.25	  1573.88	 13
  6	 Q 	   615.31	  1460.80	 12
  7	 S 	   702.34	  1332.74	 11
  8	 Y 	   865.41	  1245.71	 10
  9	 L 	   978.49	  1082.65	  9
 10	 L 	  1091.57	   969.56	  8
 11	 Q 	  1219.63	   856.48	  7
 12	 F 	  1366.70	   728.42	  6
 13	 H 	  1503.76	   581.35	  5
 14	 G 	  1560.78	   444.29	  4
 15	 L 	  1673.86	   387.27	  3
 16	 V 	  1772.93	   274.19	  2
 17	 R 	        -  	   175.12	  1  _
+2 charge
		      B   	       Y
  1	 T 	    51.53	        -  	 17
  2	 Q 	   115.56	   923.50	 16
  3	 S 	   159.08	   859.47	 15
  4	 G 	   187.59	   815.96	 14
  5	 L 	   244.13	   787.45	 13
  6	 Q 	   308.16	   730.90	 12
  7	 S 	   351.67	   666.87	 11
  8	 Y 	   433.21	   623.36	 10
  9	 L 	   489.75	   541.83	  9
 10	 L 	   546.29	   485.29	  8
 11	 Q 	   610.32	   428.74	  7
 12	 F 	   683.85	   364.71	  6
 13	 H 	   752.38	   291.18	  5
 14	 G 	   780.89	   222.65	  4
 15	 L 	   837.44	   194.14	  3
 16	 V 	   886.97	   137.60	  2
 17	 R 	        -  	    88.06	  1  _
```

**45. IPI00007047 Protein S100-A8**

| Sequence | Charge | M+H+ | XCorr | DeltaCn |
| --- | --- | --- | --- | --- |
| K.LLETECPQYIR.K | 2 | 1422.602 | 3.084 | 0.296 |

+1 charge

```
		      B   	       Y
  1	 L 	   114.09	        -  	 11
  2	 L 	   227.18	  1308.76	 10
  3	 E 	   356.22	  1195.68	  9
  4	 T 	   457.27	  1066.63	  8
  5	 E 	   586.31	   965.59	  7
  6	 C 	   746.47	   836.54	  6
  7	 P 	   843.53	   676.38	  5
  8	 Q 	   971.58	   579.32	  4
  9	 Y 	  1134.65	   451.27	  3
 10	 I 	  1247.73	   288.20	  2
 11	 R 	        -  	   175.12	  1  _
+2 charge
		      B   	       Y
  1	 L 	    57.55	        -  	 11
  2	 L 	   114.09	   654.88	 10
  3	 E 	   178.61	   598.34	  9
  4	 T 	   229.14	   533.82	  8
  5	 E 	   293.66	   483.30	  7
  6	 C 	   373.74	   418.78	  6
  7	 P 	   422.27	   338.69	  5
  8	 Q 	   486.30	   290.17	  4
  9	 Y 	   567.83	   226.14	  3
 10	 I 	   624.37	   144.61	  2
 11	 R 	        -  	    88.06	  1  _
```

**46. IPI00008239 cDNA FLJ55176, highly similar to G-protein coupled receptor family C group 5 member B**

| Sequence | Charge | M+H+ | XCorr | DeltaCn |
| --- | --- | --- | --- | --- |
| R.AYMENKAFSMDEHNAALR.T | 3 | 2099.337 | 4.2 | 0.575 |

+1 charge

```
		      B   	       Y
  1	 A 	    72.04	        -  	 18
  2	 Y 	   235.11	  2026.91	 17
  3	 M 	   366.15	  1863.85	 16
  4	 E 	   495.19	  1732.81	 15
  5	 N 	   609.23	  1603.76	 14
  6	 K 	   737.33	  1489.72	 13
  7	 A 	   808.37	  1361.63	 12
  8	 F 	   955.43	  1290.59	 11
  9	 S 	  1042.47	  1143.52	 10
 10	 M 	  1173.51	  1056.49	  9
 11	 D 	  1288.53	   925.45	  8
 12	 E 	  1417.58	   810.42	  7
 13	 H 	  1554.64	   681.38	  6
 14	 N 	  1668.68	   544.32	  5
 15	 A 	  1739.72	   430.28	  4
 16	 A 	  1810.75	   359.24	  3
 17	 L 	  1923.84	   288.20	  2
 18	 R 	        -  	   175.12	  1  _
+2 charge
		      B   	       Y
  1	 A 	    36.53	        -  	 18
  2	 Y 	   118.06	  1013.96	 17
  3	 M 	   183.58	   932.43	 16
  4	 E 	   248.10	   866.91	 15
  5	 N 	   305.12	   802.39	 14
  6	 K 	   369.17	   745.36	 13
  7	 A 	   404.69	   681.32	 12
  8	 F 	   478.22	   645.80	 11
  9	 S 	   521.74	   572.26	 10
 10	 M 	   587.26	   528.75	  9
 11	 D 	   644.77	   463.23	  8
 12	 E 	   709.29	   405.71	  7
 13	 H 	   777.82	   341.19	  6
 14	 N 	   834.84	   272.66	  5
 15	 A 	   870.36	   215.64	  4
 16	 A 	   905.88	   180.12	  3
 17	 L 	   962.42	   144.61	  2
 18	 R 	        -  	    88.06	  1  _


+3 charge
		      B   	       Y
  1	 A 	    24.69	        -  	 18
  2	 Y 	    79.04	   676.31	 17
  3	 M 	   122.72	   621.95	 16
  4	 E 	   165.74	   578.27	 15
  5	 N 	   203.75	   535.26	 14
  6	 K 	   246.45	   497.25	 13
  7	 A 	   270.13	   454.55	 12
  8	 F 	   319.15	   430.87	 11
  9	 S 	   348.16	   381.85	 10
 10	 M 	   391.84	   352.83	  9
 11	 D 	   430.18	   309.15	  8
 12	 E 	   473.20	   270.81	  7
 13	 H 	   518.88	   227.80	  6
 14	 N 	   556.90	   182.11	  5
 15	 A 	   580.58	   144.10	  4
 16	 A 	   604.26	   120.42	  3
 17	 L 	   641.95	    96.74	  2
 18	 R 	        -  	    59.04	  1  _
```

**47. IPI00021828 Cystatin-B**

| Sequence | Charge | M+H+ | XCorr | DeltaCn |
| --- | --- | --- | --- | --- |
| K.VHVGDEDFVHLR.V | 3 | 1423.558 | 3.648 | 0.43 |

+1 charge

```
		      B   	       Y
  1	 V 	   100.08	        -  	 12
  2	 H 	   237.13	  1323.64	 11
  3	 V 	   336.20	  1186.59	 10
  4	 G 	   393.22	  1087.52	  9
  5	 D 	   508.25	  1030.50	  8
  6	 E 	   637.29	   915.47	  7
  7	 D 	   752.32	   786.43	  6
  8	 F 	   899.39	   671.40	  5
  9	 V 	   998.46	   524.33	  4
 10	 H 	  1135.52	   425.26	  3
 11	 L 	  1248.60	   288.20	  2
 12	 R 	        -  	   175.12	  1  _
+2 charge
		      B   	       Y
  1	 V 	    50.54	        -  	 12
  2	 H 	   119.07	   662.33	 11
  3	 V 	   168.61	   593.80	 10
  4	 G 	   197.12	   544.26	  9
  5	 D 	   254.63	   515.75	  8
  6	 E 	   319.15	   458.24	  7
  7	 D 	   376.66	   393.72	  6
  8	 F 	   450.20	   336.20	  5
  9	 V 	   499.73	   262.67	  4
 10	 H 	   568.26	   213.13	  3
 11	 L 	   624.80	   144.61	  2
 12	 R 	        -  	    88.06	  1  _
+3 charge
		      B   	       Y
  1	 V 	    34.03	        -  	 12
  2	 H 	    79.72	   441.89	 11
  3	 V 	   112.74	   396.20	 10
  4	 G 	   131.75	   363.18	  9
  5	 D 	   170.09	   344.17	  8
  6	 E 	   213.10	   305.83	  7
  7	 D 	   251.45	   262.81	  6
  8	 F 	   300.47	   224.47	  5
  9	 V 	   333.49	   175.45	  4
 10	 H 	   379.18	   142.43	  3
 11	 L 	   416.87	    96.74	  2
 12	 R 	        -  	    59.04	  1  _
```

**48. IPI00382488 Ig heavy chain V-III region HIL**

| Sequence | Charge | M+H+ | XCorr | DeltaCn |
| --- | --- | --- | --- | --- |
| - .QVKLVQAGGGVVQPGR.S | 2 | 1593.856 | 2.956 | 0.373 |

+1 charge

```
		      B   	       Y
  1	 Q 	   129.07	        -  	 16
  2	 V 	   228.13	  1464.86	 15
  3	 K 	   356.23	  1365.80	 14
  4	 L 	   469.31	  1237.70	 13
  5	 V 	   568.38	  1124.62	 12
  6	 Q 	   696.44	  1025.55	 11
  7	 A 	   767.48	   897.49	 10
  8	 G 	   824.50	   826.45	  9
  9	 G 	   881.52	   769.43	  8
 10	 G 	   938.54	   712.41	  7
 11	 V 	  1037.61	   655.39	  6
 12	 V 	  1136.68	   556.32	  5
 13	 Q 	  1264.74	   457.25	  4
 14	 P 	  1361.79	   329.19	  3
 15	 G 	  1418.81	   232.14	  2
 16	 R 	        -  	   175.12	  1  _
+2 charge
		      B   	       Y
  1	 Q 	    65.04	        -  	 16
  2	 V 	   114.57	   732.94	 15
  3	 K 	   178.62	   683.40	 14
  4	 L 	   235.16	   619.35	 13
  5	 V 	   284.69	   562.81	 12
  6	 Q 	   348.72	   513.28	 11
  7	 A 	   384.24	   449.25	 10
  8	 G 	   412.75	   413.73	  9
  9	 G 	   441.26	   385.22	  8
 10	 G 	   469.77	   356.71	  7
 11	 V 	   519.31	   328.20	  6
 12	 V 	   568.84	   278.66	  5
 13	 Q 	   632.87	   229.13	  4
 14	 P 	   681.40	   165.10	  3
 15	 G 	   709.91	   116.57	  2
 16	 R 	        -  	    88.06	  1  _
```

**49. IPI00013890 Isoform 1 of 14-3-3 protein sigma**

| Sequence | Charge | M+H+ | XCorr | DeltaCn |
| --- | --- | --- | --- | --- |
| K.LAEQAERYEDMAAFMK.G | 2 | 1904.157 | 3.259 | 0.41 |

+1 charge

```
		      B   	       Y
  1	 L 	   114.09	        -  	 16
  2	 A 	   185.13	  1789.79	 15
  3	 E 	   314.17	  1718.75	 14
  4	 Q 	   442.23	  1589.71	 13
  5	 A 	   513.27	  1461.65	 12
  6	 E 	   642.31	  1390.61	 11
  7	 R 	   798.41	  1261.57	 10
  8	 Y 	   961.47	  1105.47	  9
  9	 E 	  1090.52	   942.41	  8
 10	 D 	  1205.54	   813.36	  7
 11	 M 	  1336.58	   698.34	  6
 12	 A 	  1407.62	   567.30	  5
 13	 A 	  1478.66	   496.26	  4
 14	 F 	  1625.73	   425.22	  3
 15	 M 	  1756.77	   278.15	  2
 16	 K 	        -  	   147.11	  1  _
+2 charge
		      B   	       Y
  1	 L 	    57.55	        -  	 16
  2	 A 	    93.07	   895.40	 15
  3	 E 	   157.59	   859.88	 14
  4	 Q 	   221.62	   795.36	 13
  5	 A 	   257.14	   731.33	 12
  6	 E 	   321.66	   695.81	 11
  7	 R 	   399.71	   631.29	 10
  8	 Y 	   481.24	   553.24	  9
  9	 E 	   545.76	   471.71	  8
 10	 D 	   603.28	   407.19	  7
 11	 M 	   668.80	   349.67	  6
 12	 A 	   704.31	   284.15	  5
 13	 A 	   739.83	   248.63	  4
 14	 F 	   813.37	   213.11	  3
 15	 M 	   878.89	   139.58	  2
 16	 K 	        -  	    74.06	  1  _
```

**50. IPI00746352 SH3 domain-binding glutamic acid-rich-like protein 3**

| Sequence | Charge | M+H+ | XCorr | DeltaCn |
| --- | --- | --- | --- | --- |
| K.RIQYQLVDISQDNALRDEMR.A | 3 | 2464.744 | 3.635 | 0.461 |

+1 charge

```
		      B   	       Y
  1	 R 	   157.11	        -  	 20
  2	 I 	   270.19	  2307.14	 19
  3	 Q 	   398.25	  2194.06	 18
  4	 Y 	   561.31	  2066.00	 17
  5	 Q 	   689.37	  1902.93	 16
  6	 L 	   802.46	  1774.88	 15
  7	 V 	   901.53	  1661.79	 14
  8	 D 	  1016.55	  1562.72	 13
  9	 I 	  1129.64	  1447.70	 12
 10	 S 	  1216.67	  1334.61	 11
 11	 Q 	  1344.73	  1247.58	 10
 12	 D 	  1459.75	  1119.52	  9
 13	 N 	  1573.80	  1004.49	  8
 14	 A 	  1644.83	   890.45	  7
 15	 L 	  1757.92	   819.41	  6
 16	 R 	  1914.02	   706.33	  5
 17	 D 	  2029.05	   550.23	  4
 18	 E 	  2158.09	   435.20	  3
 19	 M 	  2289.13	   306.16	  2
 20	 R 	        -  	   175.12	  1  _
+2 charge
		      B   	       Y
  1	 R 	    79.06	        -  	 20
  2	 I 	   135.60	  1154.07	 19
  3	 Q 	   199.63	  1097.53	 18
  4	 Y 	   281.16	  1033.50	 17
  5	 Q 	   345.19	   951.97	 16
  6	 L 	   401.73	   887.94	 15
  7	 V 	   451.27	   831.40	 14
  8	 D 	   508.78	   781.87	 13
  9	 I 	   565.32	   724.35	 12
 10	 S 	   608.84	   667.81	 11
 11	 Q 	   672.87	   624.29	 10
 12	 D 	   730.38	   560.26	  9
 13	 N 	   787.40	   502.75	  8
 14	 A 	   822.92	   445.73	  7
 15	 L 	   879.46	   410.21	  6
 16	 R 	   957.51	   353.67	  5
 17	 D 	  1015.03	   275.62	  4
 18	 E 	  1079.55	   218.10	  3
 19	 M 	  1145.07	   153.58	  2
 20	 R 	        -  	    88.06	  1  _

+3 charge
		      B   	       Y
  1	 R 	    53.04	        -  	 20
  2	 I 	    90.74	   769.72	 19
  3	 Q 	   133.42	   732.02	 18
  4	 Y 	   187.78	   689.34	 17
  5	 Q 	   230.46	   634.98	 16
  6	 L 	   268.16	   592.30	 15
  7	 V 	   301.18	   554.60	 14
  8	 D 	   339.52	   521.58	 13
  9	 I 	   377.22	   483.24	 12
 10	 S 	   406.23	   445.54	 11
 11	 Q 	   448.91	   416.53	 10
 12	 D 	   487.26	   373.85	  9
 13	 N 	   525.27	   335.50	  8
 14	 A 	   548.95	   297.49	  7
 15	 L 	   586.64	   273.81	  6
 16	 R 	   638.68	   236.11	  5
 17	 D 	   677.02	   184.08	  4
 18	 E 	   720.03	   145.74	  3
 19	 M 	   763.71	   102.72	  2
 20	 R 	        -  	    59.04	  1  _
```

**51. IPI00005732 Isoform 1 of Activin receptor type-1B**

| Sequence | Charge | M+H+ | XCorr | DeltaCn |
| --- | --- | --- | --- | --- |
| R.TCIPKVELVPAGKPFYCLSSEDLR.N | 3 | 2781.184 | 4.734 | 0.546 |

+1 charge

```
		      B   	       Y
  1	 T 	   102.05	        -  	 24
  2	 C 	   262.22	  2678.64	 23
  3	 I 	   375.30	  2518.47	 22
  4	 P 	   472.36	  2405.39	 21
  5	 K 	   600.45	  2308.34	 20
  6	 V 	   699.52	  2180.24	 19
  7	 E 	   828.56	  2081.17	 18
  8	 L 	   941.65	  1952.13	 17
  9	 V 	  1040.72	  1839.05	 16
 10	 P 	  1137.77	  1739.98	 15
 11	 A 	  1208.81	  1642.92	 14
 12	 G 	  1265.83	  1571.89	 13
 13	 K 	  1393.92	  1514.87	 12
 14	 P 	  1490.97	  1386.77	 11
 15	 F 	  1638.04	  1289.72	 10
 16	 Y 	  1801.11	  1142.65	  9
 17	 C 	  1961.27	   979.59	  8
 18	 L 	  2074.36	   819.42	  7
 19	 S 	  2161.39	   706.34	  6
 20	 S 	  2248.42	   619.30	  5
 21	 E 	  2377.46	   532.27	  4
 22	 D 	  2492.49	   403.23	  3
 23	 L 	  2605.57	   288.20	  2
 24	 R 	        -  	   175.12	  1  _


+2 charge
		      B   	       Y
  1	 T 	    51.53	        -  	 24
  2	 C 	   131.61	  1339.82	 23
  3	 I 	   188.16	  1259.74	 22
  4	 P 	   236.68	  1203.20	 21
  5	 K 	   300.73	  1154.67	 20
  6	 V 	   350.26	  1090.62	 19
  7	 E 	   414.79	  1041.09	 18
  8	 L 	   471.33	   976.57	 17
  9	 V 	   520.86	   920.03	 16
 10	 P 	   569.39	   870.49	 15
 11	 A 	   604.91	   821.97	 14
 12	 G 	   633.42	   786.45	 13
 13	 K 	   697.46	   757.94	 12
 14	 P 	   745.99	   693.89	 11
 15	 F 	   819.53	   645.36	 10
 16	 Y 	   901.06	   571.83	  9
 17	 C 	   981.14	   490.30	  8
 18	 L 	  1037.68	   410.21	  7
 19	 S 	  1081.20	   353.67	  6
 20	 S 	  1124.71	   310.16	  5
 21	 E 	  1189.23	   266.64	  4
 22	 D 	  1246.75	   202.12	  3
 23	 L 	  1303.29	   144.61	  2
 24	 R 	        -  	    88.06	  1  _


+3 charge
		      B   	       Y
  1	 T 	    34.69	        -  	 24
  2	 C 	    88.08	   893.55	 23
  3	 I 	   125.77	   840.16	 22
  4	 P 	   158.12	   802.47	 21
  5	 K 	   200.82	   770.12	 20
  6	 V 	   233.84	   727.42	 19
  7	 E 	   276.86	   694.40	 18
  8	 L 	   314.55	   651.38	 17
  9	 V 	   347.58	   613.69	 16
 10	 P 	   379.93	   580.66	 15
 11	 A 	   403.61	   548.31	 14
 12	 G 	   422.61	   524.63	 13
 13	 K 	   465.31	   505.63	 12
 14	 P 	   497.66	   462.93	 11
 15	 F 	   546.69	   430.58	 10
 16	 Y 	   601.04	   381.55	  9
 17	 C 	   654.43	   327.20	  8
 18	 L 	   692.12	   273.81	  7
 19	 S 	   721.13	   236.12	  6
 20	 S 	   750.14	   207.11	  5
 21	 E 	   793.16	   178.10	  4
 22	 D 	   831.50	   135.08	  3
 23	 L 	   869.20	    96.74	  2
 24	 R 	        -  	    59.04	  1  _
```

**52. IPI00018901 Isoform 1 of Gamma-glutamyltranspeptidase 1**

| Sequence | Charge | M+H+ | XCorr | DeltaCn |
| --- | --- | --- | --- | --- |
| R.TVIEQQPVLCEVFCR.D | 2 | 1879.134 | 3.675 | 0.336 |

+1 charge

```
		      B   	       Y
  1	 T 	   102.05	        -  	 15
  2	 V 	   201.12	  1777.15	 14
  3	 I 	   314.21	  1678.08	 13
  4	 E 	   443.25	  1564.99	 12
  5	 Q 	   571.31	  1435.95	 11
  6	 Q 	   699.37	  1307.89	 10
  7	 P 	   796.42	  1179.83	  9
  8	 V 	   895.49	  1082.78	  8
  9	 L 	  1008.57	   983.71	  7
 10	 C 	  1168.74	   870.63	  6
 11	 E 	  1297.78	   710.46	  5
 12	 V 	  1396.85	   581.42	  4
 13	 F 	  1543.92	   482.35	  3
 14	 C 	  1704.08	   335.28	  2
 15	 R 	        -  	   175.12	  1  _
+2 charge
		      B   	       Y
  1	 T 	    51.53	        -  	 15
  2	 V 	   101.07	   889.08	 14
  3	 I 	   157.61	   839.54	 13
  4	 E 	   222.13	   783.00	 12
  5	 Q 	   286.16	   718.48	 11
  6	 Q 	   350.19	   654.45	 10
  7	 P 	   398.71	   590.42	  9
  8	 V 	   448.25	   541.89	  8
  9	 L 	   504.79	   492.36	  7
 10	 C 	   584.87	   435.82	  6
 11	 E 	   649.39	   355.74	  5
 12	 V 	   698.93	   291.21	  4
 13	 F 	   772.46	   241.68	  3
 14	 C 	   852.54	   168.15	  2
 15	 R 	        -  	    88.06	  1  _
```

**53. IPI00152018 Isoform 1 of CMRF35-like molecule 9**

| Sequence | Charge | M+H+ | XCorr | DeltaCn |
| --- | --- | --- | --- | --- |
| R.KGGILFSR.C | 2 | 878.0538 | 3.206 | 0.387 |

+1 charge

```
		      B   	       Y
  1	 K 	   129.10	        -  	  8
  2	 G 	   186.12	   749.43	  7
  3	 G 	   243.15	   692.41	  6
  4	 I 	   356.23	   635.39	  5
  5	 L 	   469.31	   522.30	  4
  6	 F 	   616.38	   409.22	  3
  7	 S 	   703.41	   262.15	  2
  8	 R 	        -  	   175.12	  1  _
+2 charge
		      B   	       Y
  1	 K 	    65.05	        -  	  8
  2	 G 	    93.57	   375.22	  7
  3	 G 	   122.08	   346.71	  6
  4	 I 	   178.62	   318.20	  5
  5	 L 	   235.16	   261.66	  4
  6	 F 	   308.69	   205.11	  3
  7	 S 	   352.21	   131.58	  2
  8	 R 	        -  	    88.06	  1  _
```

**54. IPI00289342 Ephrin type-B receptor 4**

| Sequence | Charge | M+H+ | XCorr | DeltaCn |
| --- | --- | --- | --- | --- |
| R.TYEVCDVQRAPGQAHWLR.T | 3 | 2187.397 | 3.795 | 0.47 |

+1 charge

```
		      B   	       Y
  1	 T 	   102.05	        -  	 18
  2	 Y 	   265.12	  2085.14	 17
  3	 E 	   394.16	  1922.08	 16
  4	 V 	   493.23	  1793.04	 15
  5	 C 	   653.39	  1693.97	 14
  6	 D 	   768.42	  1533.80	 13
  7	 V 	   867.49	  1418.78	 12
  8	 Q 	   995.55	  1319.71	 11
  9	 R 	  1151.65	  1191.65	 10
 10	 A 	  1222.69	  1035.55	  9
 11	 P 	  1319.74	   964.51	  8
 12	 G 	  1376.76	   867.46	  7
 13	 Q 	  1504.82	   810.44	  6
 14	 A 	  1575.86	   682.38	  5
 15	 H 	  1712.92	   611.34	  4
 16	 W 	  1898.99	   474.28	  3
 17	 L 	  2012.08	   288.20	  2
 18	 R 	        -  	   175.12	  1  _
+2 charge
		      B   	       Y
  1	 T 	    51.53	        -  	 18
  2	 Y 	   133.06	  1043.08	 17
  3	 E 	   197.58	   961.54	 16
  4	 V 	   247.12	   897.02	 15
  5	 C 	   327.20	   847.49	 14
  6	 D 	   384.71	   767.41	 13
  7	 V 	   434.25	   709.89	 12
  8	 Q 	   498.28	   660.36	 11
  9	 R 	   576.33	   596.33	 10
 10	 A 	   611.85	   518.28	  9
 11	 P 	   660.37	   482.76	  8
 12	 G 	   688.88	   434.23	  7
 13	 Q 	   752.91	   405.72	  6
 14	 A 	   788.43	   341.69	  5
 15	 H 	   856.96	   306.17	  4
 16	 W 	   950.00	   237.64	  3
 17	 L 	  1006.54	   144.61	  2
 18	 R 	        -  	    88.06	  1  _
+3 charge
		      B   	       Y
  1	 T 	    34.69	        -  	 18
  2	 Y 	    89.04	   695.72	 17
  3	 E 	   132.06	   641.36	 16
  4	 V 	   165.08	   598.35	 15
  5	 C 	   218.47	   565.33	 14
  6	 D 	   256.81	   511.94	 13
  7	 V 	   289.83	   473.60	 12
  8	 Q 	   332.52	   440.57	 11
  9	 R 	   384.55	   397.89	 10
 10	 A 	   408.23	   345.85	  9
 11	 P 	   440.58	   322.18	  8
 12	 G 	   459.59	   289.82	  7
 13	 Q 	   502.28	   270.82	  6
 14	 A 	   525.96	   228.13	  5
 15	 H 	   571.64	   204.45	  4
 16	 W 	   633.67	   158.77	  3
 17	 L 	   671.36	    96.74	  2
 18	 R 	        -  	    59.04	  1  _
```

**55. IPI00382440 Ig lambda chain V-IV region Hil**

| Sequence | Charge | M+H+ | XCorr | DeltaCn |
| --- | --- | --- | --- | --- |
| - .SYELTQPPSVSVSPGQTAR.I | 2 | 2005.175 | 3.203 | 0.573 |

+1 charge

```
		      B   	       Y
  1	 S 	    88.04	        -  	 19
  2	 Y 	   251.10	  1916.97	 18
  3	 E 	   380.15	  1753.91	 17
  4	 L 	   493.23	  1624.87	 16
  5	 T 	   594.28	  1511.78	 15
  6	 Q 	   722.34	  1410.73	 14
  7	 P 	   819.39	  1282.67	 13
  8	 P 	   916.44	  1185.62	 12
  9	 S 	  1003.47	  1088.57	 11
 10	 V 	  1102.54	  1001.54	 10
 11	 S 	  1189.57	   902.47	  9
 12	 V 	  1288.64	   815.44	  8
 13	 S 	  1375.67	   716.37	  7
 14	 P 	  1472.73	   629.34	  6
 15	 G 	  1529.75	   532.28	  5
 16	 Q 	  1657.81	   475.26	  4
 17	 T 	  1758.85	   347.20	  3
 18	 A 	  1829.89	   246.16	  2
 19	 R 	        -  	   175.12	  1  _
+2 charge
		      B   	       Y
  1	 S 	    44.52	        -  	 19
  2	 Y 	   126.05	   958.99	 18
  3	 E 	   190.58	   877.46	 17
  4	 L 	   247.12	   812.94	 16
  5	 T 	   297.64	   756.39	 15
  6	 Q 	   361.67	   705.87	 14
  7	 P 	   410.20	   641.84	 13
  8	 P 	   458.72	   593.31	 12
  9	 S 	   502.24	   544.79	 11
 10	 V 	   551.77	   501.27	 10
 11	 S 	   595.29	   451.74	  9
 12	 V 	   644.82	   408.22	  8
 13	 S 	   688.34	   358.69	  7
 14	 P 	   736.87	   315.17	  6
 15	 G 	   765.38	   266.65	  5
 16	 Q 	   829.41	   238.13	  4
 17	 T 	   879.93	   174.11	  3
 18	 A 	   915.45	   123.58	  2
 19	 R 	        -  	    88.06	  1  _
```

**56. IPI00028347 Isoform 1 of F-box only protein 31**

| Sequence | Charge | M+H+ | XCorr | DeltaCn |
| --- | --- | --- | --- | --- |
| R.IYLPPSRPDDLIKPGLFK.G | 2 | 2070.462 | 2.893 | 0.213 |

+1 charge

```
		      B   	       Y
  1	 I 	   114.09	        -  	 18
  2	 Y 	   277.15	  1956.10	 17
  3	 L 	   390.24	  1793.03	 16
  4	 P 	   487.29	  1679.95	 15
  5	 P 	   584.34	  1582.90	 14
  6	 S 	   671.38	  1485.84	 13
  7	 R 	   827.48	  1398.81	 12
  8	 P 	   924.53	  1242.71	 11
  9	 D 	  1039.56	  1145.66	 10
 10	 D 	  1154.58	  1030.63	  9
 11	 L 	  1267.67	   915.60	  8
 12	 I 	  1380.75	   802.52	  7
 13	 K 	  1508.85	   689.43	  6
 14	 P 	  1605.90	   561.34	  5
 15	 G 	  1662.92	   464.29	  4
 16	 L 	  1776.01	   407.27	  3
 17	 F 	  1923.07	   294.18	  2
 18	 K 	        -  	   147.11	  1  _
+2 charge
		      B   	       Y
  1	 I 	    57.55	        -  	 18
  2	 Y 	   139.08	   978.55	 17
  3	 L 	   195.62	   897.02	 16
  4	 P 	   244.15	   840.48	 15
  5	 P 	   292.68	   791.95	 14
  6	 S 	   336.19	   743.42	 13
  7	 R 	   414.24	   699.91	 12
  8	 P 	   462.77	   621.86	 11
  9	 D 	   520.28	   573.33	 10
 10	 D 	   577.80	   515.82	  9
 11	 L 	   634.34	   458.30	  8
 12	 I 	   690.88	   401.76	  7
 13	 K 	   754.93	   345.22	  6
 14	 P 	   803.45	   281.17	  5
 15	 G 	   831.96	   232.65	  4
 16	 L 	   888.51	   204.14	  3
 17	 F 	   962.04	   147.59	  2
 18	 K 	        -  	    74.06	  1  _
```

**57. IPI00387113 Ig kappa chain V-III region B6**

| Sequence | Charge | M+H+ | XCorr | DeltaCn |
| --- | --- | --- | --- | --- |
| - .ZIVLTZSPGTLSLSPGZR.A | 2 | 1884.616 | 3.73 | 0.611 |

+1 charge

```
		      B   	       Y
  1	 Z 	   129.56	        -  	 18
  2	 I 	   242.64	  1754.96	 17
  3	 V 	   341.71	  1641.88	 16
  4	 L 	   454.79	  1542.81	 15
  5	 T 	   555.84	  1429.73	 14
  6	 Z 	   684.39	  1328.68	 13
  7	 S 	   771.42	  1200.13	 12
  8	 P 	   868.48	  1113.10	 11
  9	 G 	   925.50	  1016.05	 10
 10	 T 	  1026.55	   959.02	  9
 11	 L 	  1139.63	   857.98	  8
 12	 S 	  1226.66	   744.89	  7
 13	 L 	  1339.75	   657.86	  6
 14	 S 	  1426.78	   544.78	  5
 15	 P 	  1523.83	   457.74	  4
 16	 G 	  1580.85	   360.69	  3
 17	 Z 	  1709.40	   303.67	  2
 18	 R 	        -  	   175.12	  1  _
+2 charge
		      B   	       Y
  1	 Z 	    65.28	        -  	 18
  2	 I 	   121.82	   877.99	 17
  3	 V 	   171.36	   821.44	 16
  4	 L 	   227.90	   771.91	 15
  5	 T 	   278.42	   715.37	 14
  6	 Z 	   342.70	   664.84	 13
  7	 S 	   386.22	   600.57	 12
  8	 P 	   434.74	   557.05	 11
  9	 G 	   463.25	   508.53	 10
 10	 T 	   513.78	   480.02	  9
 11	 L 	   570.32	   429.49	  8
 12	 S 	   613.83	   372.95	  7
 13	 L 	   670.38	   329.43	  6
 14	 S 	   713.89	   272.89	  5
 15	 P 	   762.42	   229.38	  4
 16	 G 	   790.93	   180.85	  3
 17	 Z 	   855.21	   152.34	  2
 18	 R 	        -  	    88.06	  1  _
```

**58. IPI00296058 EGF-containing fibulin-like extracellular matrix protein 2**

| Sequence | Charge | M+H+ | XCorr | DeltaCn |
| --- | --- | --- | --- | --- |
| K.CINHYGGYLCLPR.S | 2 | 1623.824 | 4.465 | 0.468 |

+1 charge

```
		      B   	       Y
  1	 C 	   161.17	        -  	 13
  2	 I 	   274.26	  1462.86	 12
  3	 N 	   388.30	  1349.78	 11
  4	 H 	   525.36	  1235.73	 10
  5	 Y 	   688.42	  1098.67	  9
  6	 G 	   745.44	   935.61	  8
  7	 G 	   802.46	   878.59	  7
  8	 Y 	   965.53	   821.57	  6
  9	 L 	  1078.61	   658.51	  5
 10	 C 	  1238.78	   545.42	  4
 11	 L 	  1351.86	   385.26	  3
 12	 P 	  1448.91	   272.17	  2
 13	 R 	        -  	   175.12	  1  _
+2 charge
		      B   	       Y
  1	 C 	    81.09	        -  	 13
  2	 I 	   137.63	   731.93	 12
  3	 N 	   194.65	   675.39	 11
  4	 H 	   263.18	   618.37	 10
  5	 Y 	   344.71	   549.84	  9
  6	 G 	   373.23	   468.31	  8
  7	 G 	   401.74	   439.80	  7
  8	 Y 	   483.27	   411.29	  6
  9	 L 	   539.81	   329.76	  5
 10	 C 	   619.89	   273.21	  4
 11	 L 	   676.43	   193.13	  3
 12	 P 	   724.96	   136.59	  2
 13	 R 	        -  	    88.06	  1  _
```

**59. IPI00023728 Gamma-glutamyl hydrolase**

| Sequence | Charge | M+H+ | XCorr | DeltaCn |
| --- | --- | --- | --- | --- |
| R.VVPVRLDLTEKDYEILFK.S | 3 | 2178.556 | 3.939 | 0.387 |

+1 charge

```
		      B   	       Y
  1	 V 	   100.08	        -  	 18
  2	 V 	   199.14	  2078.15	 17
  3	 P 	   296.20	  1979.08	 16
  4	 V 	   395.27	  1882.03	 15
  5	 R 	   551.37	  1782.96	 14
  6	 L 	   664.45	  1626.86	 13
  7	 D 	   779.48	  1513.78	 12
  8	 L 	   892.56	  1398.75	 11
  9	 T 	   993.61	  1285.67	 10
 10	 E 	  1122.65	  1184.62	  9
 11	 K 	  1250.75	  1055.58	  8
 12	 D 	  1365.77	   927.48	  7
 13	 Y 	  1528.84	   812.46	  6
 14	 E 	  1657.88	   649.39	  5
 15	 I 	  1770.96	   520.35	  4
 16	 L 	  1884.05	   407.27	  3
 17	 F 	  2031.12	   294.18	  2
 18	 K 	        -  	   147.11	  1  _
+2 charge
		      B   	       Y
  1	 V 	    50.54	        -  	 18
  2	 V 	   100.08	  1039.58	 17
  3	 P 	   148.60	   990.05	 16
  4	 V 	   198.14	   941.52	 15
  5	 R 	   276.19	   891.99	 14
  6	 L 	   332.73	   813.93	 13
  7	 D 	   390.24	   757.39	 12
  8	 L 	   446.78	   699.88	 11
  9	 T 	   497.31	   643.34	 10
 10	 E 	   561.83	   592.81	  9
 11	 K 	   625.88	   528.29	  8
 12	 D 	   683.39	   464.24	  7
 13	 Y 	   764.92	   406.73	  6
 14	 E 	   829.44	   325.20	  5
 15	 I 	   885.99	   260.68	  4
 16	 L 	   942.53	   204.14	  3
 17	 F 	  1016.06	   147.59	  2
 18	 K 	        -  	    74.06	  1  _
+3 charge
		      B   	       Y
  1	 V 	    34.03	        -  	 18
  2	 V 	    67.05	   693.39	 17
  3	 P 	    99.40	   660.37	 16
  4	 V 	   132.43	   628.02	 15
  5	 R 	   184.46	   594.99	 14
  6	 L 	   222.16	   542.96	 13
  7	 D 	   260.50	   505.26	 12
  8	 L 	   298.19	   466.92	 11
  9	 T 	   331.87	   429.23	 10
 10	 E 	   374.89	   395.54	  9
 11	 K 	   417.59	   352.53	  8
 12	 D 	   455.93	   309.83	  7
 13	 Y 	   510.28	   271.49	  6
 14	 E 	   553.30	   217.14	  5
 15	 I 	   590.99	   174.12	  4
 16	 L 	   628.69	   136.43	  3
 17	 F 	   677.71	    98.73	  2
 18	 K 	        -  	    49.71	  1  _
```

**60. IPI00297252 Isoform 1 of Extracellular sulfatase Sulf-2**

| Sequence | Charge | M+H+ | XCorr | DeltaCn |
| --- | --- | --- | --- | --- |
| R.VYHVGLGDAAQPR.N | 3 | 1383.537 | 4.234 | 0.429 |

+1 charge

```
		      B   	       Y
  1	 V 	   100.08	        -  	 13
  2	 Y 	   263.14	  1283.65	 12
  3	 H 	   400.20	  1120.59	 11
  4	 V 	   499.27	   983.53	 10
  5	 G 	   556.29	   884.46	  9
  6	 L 	   669.37	   827.44	  8
  7	 G 	   726.39	   714.35	  7
  8	 D 	   841.42	   657.33	  6
  9	 A 	   912.46	   542.30	  5
 10	 A 	   983.49	   471.27	  4
 11	 Q 	  1111.55	   400.23	  3
 12	 P 	  1208.61	   272.17	  2
 13	 R 	        -  	   175.12	  1  _
+2 charge
		      B   	       Y
  1	 V 	    50.54	        -  	 13
  2	 Y 	   132.07	   642.33	 12
  3	 H 	   200.60	   560.80	 11
  4	 V 	   250.14	   492.27	 10
  5	 G 	   278.65	   442.73	  9
  6	 L 	   335.19	   414.22	  8
  7	 G 	   363.70	   357.68	  7
  8	 D 	   421.21	   329.17	  6
  9	 A 	   456.73	   271.66	  5
 10	 A 	   492.25	   236.14	  4
 11	 Q 	   556.28	   200.62	  3
 12	 P 	   604.81	   136.59	  2
 13	 R 	        -  	    88.06	  1  _


+3 charge
		      B   	       Y
  1	 V 	    34.03	        -  	 13
  2	 Y 	    88.38	   428.55	 12
  3	 H 	   134.07	   374.20	 11
  4	 V 	   167.09	   328.51	 10
  5	 G 	   186.10	   295.49	  9
  6	 L 	   223.80	   276.48	  8
  7	 G 	   242.80	   238.79	  7
  8	 D 	   281.14	   219.78	  6
  9	 A 	   304.82	   181.44	  5
 10	 A 	   328.50	   157.76	  4
 11	 Q 	   371.19	   134.08	  3
 12	 P 	   403.54	    91.40	  2
 13	 R 	        -  	    59.04	  1  _
```

**61. IPI00896380 Isoform 2 of Ig mu chain C region**

| Sequence | Charge | M+H+ | XCorr | DeltaCn |
| --- | --- | --- | --- | --- |
| K.LICQATGFSPR.Q | 2 | 1250.423 | 3.248 | 0.423 |

+1 charge

```
		      B   	       Y
  1	 L 	   114.09	        -  	 11
  2	 I 	   227.18	  1136.69	 10
  3	 C 	   387.34	  1023.60	  9
  4	 Q 	   515.40	   863.44	  8
  5	 A 	   586.44	   735.38	  7
  6	 T 	   687.48	   664.34	  6
  7	 G 	   744.51	   563.29	  5
  8	 F 	   891.57	   506.27	  4
  9	 S 	   978.61	   359.20	  3
 10	 P 	  1075.66	   272.17	  2
 11	 R 	        -  	   175.12	  1  _
+2 charge
		      B   	       Y
  1	 L 	    57.55	        -  	 11
  2	 I 	   114.09	   568.85	 10
  3	 C 	   194.17	   512.30	  9
  4	 Q 	   258.20	   432.22	  8
  5	 A 	   293.72	   368.19	  7
  6	 T 	   344.25	   332.67	  6
  7	 G 	   372.76	   282.15	  5
  8	 F 	   446.29	   253.64	  4
  9	 S 	   489.81	   180.11	  3
 10	 P 	   538.33	   136.59	  2
 11	 R 	        -  	    88.06	  1  _
```

**62. IPI00289931 MADCAM1 protein**

| Sequence | Charge | M+H+ | XCorr | DeltaCn |
| --- | --- | --- | --- | --- |
| R.GLDTSLGAVQSDTGR.S | 2 | 1477.559 | 4.158 | 0.569 |

+1 charge

```
		      B   	       Y
  1	 G 	    58.03	        -  	 15
  2	 L 	   171.11	  1419.71	 14
  3	 D 	   286.14	  1306.62	 13
  4	 T 	   387.19	  1191.60	 12
  5	 S 	   474.22	  1090.55	 11
  6	 L 	   587.30	  1003.52	 10
  7	 G 	   644.32	   890.43	  9
  8	 A 	   715.36	   833.41	  8
  9	 V 	   814.43	   762.37	  7
 10	 Q 	   942.49	   663.31	  6
 11	 S 	  1029.52	   535.25	  5
 12	 D 	  1144.55	   448.22	  4
 13	 T 	  1245.60	   333.19	  3
 14	 G 	  1302.62	   232.14	  2
 15	 R 	        -  	   175.12	  1  _
+2 charge
		      B   	       Y
  1	 G 	    29.52	        -  	 15
  2	 L 	    86.06	   710.36	 14
  3	 D 	   143.57	   653.82	 13
  4	 T 	   194.10	   596.30	 12
  5	 S 	   237.61	   545.78	 11
  6	 L 	   294.16	   502.26	 10
  7	 G 	   322.67	   445.72	  9
  8	 A 	   358.18	   417.21	  8
  9	 V 	   407.72	   381.69	  7
 10	 Q 	   471.75	   332.16	  6
 11	 S 	   515.26	   268.13	  5
 12	 D 	   572.78	   224.61	  4
 13	 T 	   623.30	   167.10	  3
 14	 G 	   651.81	   116.57	  2
 15	 R 	        -  	    88.06	  1  _
```

**63. IPI00022284 Major prion protein**

| Sequence | Charge | M+H+ | XCorr | DeltaCn |
| --- | --- | --- | --- | --- |
| R.VVEQMCITQYER.E | 2 | 1556.76 | 3.795 | 0.425 |

+1 charge

```
		      B   	       Y
  1	 V 	   100.08	        -  	 12
  2	 V 	   199.14	  1456.79	 11
  3	 E 	   328.19	  1357.72	 10
  4	 Q 	   456.25	  1228.68	  9
  5	 M 	   587.29	  1100.62	  8
  6	 C 	   747.45	   969.58	  7
  7	 I 	   860.54	   809.42	  6
  8	 T 	   961.58	   696.33	  5
  9	 Q 	  1089.64	   595.28	  4
 10	 Y 	  1252.70	   467.22	  3
 11	 E 	  1381.75	   304.16	  2
 12	 R 	        -  	   175.12	  1  _
+2 charge
		      B   	       Y
  1	 V 	    50.54	        -  	 12
  2	 V 	   100.08	   728.90	 11
  3	 E 	   164.60	   679.36	 10
  4	 Q 	   228.63	   614.84	  9
  5	 M 	   294.15	   550.81	  8
  6	 C 	   374.23	   485.29	  7
  7	 I 	   430.77	   405.21	  6
  8	 T 	   481.30	   348.67	  5
  9	 Q 	   545.32	   298.15	  4
 10	 Y 	   626.86	   234.12	  3
 11	 E 	   691.38	   152.58	  2
 12	 R 	        -  	    88.06	  1  _
```

**64. IPI00043992 Poliovirus receptor-related protein 4**

| Sequence | Charge | M+H+ | XCorr | DeltaCn |
| --- | --- | --- | --- | --- |
| R.VDAGEGAQELALLHSK.Y | 2 | 1638.804 | 2.857 | 0.388 |

+1 charge

```
		      B   	       Y
  1	 V 	   100.08	        -  	 16
  2	 D 	   215.10	  1538.78	 15
  3	 A 	   286.14	  1423.75	 14
  4	 G 	   343.16	  1352.72	 13
  5	 E 	   472.20	  1295.70	 12
  6	 G 	   529.23	  1166.65	 11
  7	 A 	   600.26	  1109.63	 10
  8	 Q 	   728.32	  1038.59	  9
  9	 E 	   857.36	   910.54	  8
 10	 L 	   970.45	   781.49	  7
 11	 A 	  1041.48	   668.41	  6
 12	 L 	  1154.57	   597.37	  5
 13	 L 	  1267.65	   484.29	  4
 14	 H 	  1404.71	   371.20	  3
 15	 S 	  1491.74	   234.14	  2
 16	 K 	        -  	   147.11	  1  _
+2 charge
		      B   	       Y
  1	 V 	    50.54	        -  	 16
  2	 D 	   108.05	   769.89	 15
  3	 A 	   143.57	   712.38	 14
  4	 G 	   172.08	   676.86	 13
  5	 E 	   236.61	   648.35	 12
  6	 G 	   265.12	   583.83	 11
  7	 A 	   300.63	   555.32	 10
  8	 Q 	   364.66	   519.80	  9
  9	 E 	   429.19	   455.77	  8
 10	 L 	   485.73	   391.25	  7
 11	 A 	   521.25	   334.71	  6
 12	 L 	   577.79	   299.19	  5
 13	 L 	   634.33	   242.65	  4
 14	 H 	   702.86	   186.11	  3
 15	 S 	   746.38	   117.58	  2
 16	 K 	        -  	    74.06	  1  _
```

**65. IPI00020986 Lumican**

| Sequence | Charge | M+H+ | XCorr | DeltaCn |
| --- | --- | --- | --- | --- |
| K.RFNALQYLR.L | 2 | 1181.372 | 2.823 | 0.24 |

+1 charge

```
		      B   	       Y
  1	 R 	   157.11	        -  	  9
  2	 F 	   304.18	  1024.56	  8
  3	 N 	   418.22	   877.49	  7
  4	 A 	   489.26	   763.45	  6
  5	 L 	   602.34	   692.41	  5
  6	 Q 	   730.40	   579.32	  4
  7	 Y 	   893.46	   451.27	  3
  8	 L 	  1006.55	   288.20	  2
  9	 R 	        -  	   175.12	  1  _
+2 charge
		      B   	       Y
  1	 R 	    79.06	        -  	  9
  2	 F 	   152.59	   512.78	  8
  3	 N 	   209.61	   439.25	  7
  4	 A 	   245.13	   382.23	  6
  5	 L 	   301.67	   346.71	  5
  6	 Q 	   365.70	   290.17	  4
  7	 Y 	   447.24	   226.14	  3
  8	 L 	   503.78	   144.61	  2
  9	 R 	        -  	    88.06	  1  _
```

**66. IPI00382436 Ig lambda chain V-III region SH**

| Sequence | Charge | M+H+ | XCorr | DeltaCn |
| --- | --- | --- | --- | --- |
| - .SELTQDPAVSVALGQTVR.I | 2 | 1872.07 | 6.424 | 0.519 |

+1 charge

```
		      B   	       Y
  1	 S 	    88.04	        -  	 18
  2	 E 	   217.08	  1783.95	 17
  3	 L 	   330.17	  1654.91	 16
  4	 T 	   431.21	  1541.83	 15
  5	 Q 	   559.27	  1440.78	 14
  6	 D 	   674.30	  1312.72	 13
  7	 P 	   771.35	  1197.69	 12
  8	 A 	   842.39	  1100.64	 11
  9	 V 	   941.46	  1029.61	 10
 10	 S 	  1028.49	   930.54	  9
 11	 V 	  1127.56	   843.50	  8
 12	 A 	  1198.60	   744.44	  7
 13	 L 	  1311.68	   673.40	  6
 14	 G 	  1368.70	   560.32	  5
 15	 Q 	  1496.76	   503.29	  4
 16	 T 	  1597.81	   375.24	  3
 17	 V 	  1696.88	   274.19	  2
 18	 R 	        -  	   175.12	  1  _
+2 charge
		      B   	       Y
  1	 S 	    44.52	        -  	 18
  2	 E 	   109.04	   892.48	 17
  3	 L 	   165.59	   827.96	 16
  4	 T 	   216.11	   771.42	 15
  5	 Q 	   280.14	   720.89	 14
  6	 D 	   337.65	   656.86	 13
  7	 P 	   386.18	   599.35	 12
  8	 A 	   421.70	   550.82	 11
  9	 V 	   471.23	   515.31	 10
 10	 S 	   514.75	   465.77	  9
 11	 V 	   564.28	   422.26	  8
 12	 A 	   599.80	   372.72	  7
 13	 L 	   656.34	   337.20	  6
 14	 G 	   684.85	   280.66	  5
 15	 Q 	   748.88	   252.15	  4
 16	 T 	   799.41	   188.12	  3
 17	 V 	   848.94	   137.60	  2
 18	 R 	        -  	    88.06	  1  _
```

**67. IPI00816799 Rheumatoid factor D5 light chain (Fragment)**

| Sequence | Charge | M+H+ | XCorr | DeltaCn |
| --- | --- | --- | --- | --- |
| - .EIVLTQSPATLSLSPGER.A | 2 | 1899.135 | 4.807 | 0.462 |

+1 charge

```
		      B   	       Y
  1	 E 	   130.05	        -  	 18
  2	 I 	   243.13	  1768.98	 17
  3	 V 	   342.20	  1655.90	 16
  4	 L 	   455.29	  1556.83	 15
  5	 T 	   556.33	  1443.74	 14
  6	 Q 	   684.39	  1342.70	 13
  7	 S 	   771.42	  1214.64	 12
  8	 P 	   868.48	  1127.61	 11
  9	 A 	   939.51	  1030.55	 10
 10	 T 	  1040.56	   959.52	  9
 11	 L 	  1153.65	   858.47	  8
 12	 S 	  1240.68	   745.38	  7
 13	 L 	  1353.76	   658.35	  6
 14	 S 	  1440.79	   545.27	  5
 15	 P 	  1537.85	   458.24	  4
 16	 G 	  1594.87	   361.18	  3
 17	 E 	  1723.91	   304.16	  2
 18	 R 	        -  	   175.12	  1  _
+2 charge
		      B   	       Y
  1	 E 	    65.53	        -  	 18
  2	 I 	   122.07	   884.99	 17
  3	 V 	   171.60	   828.45	 16
  4	 L 	   228.15	   778.92	 15
  5	 T 	   278.67	   722.38	 14
  6	 Q 	   342.70	   671.85	 13
  7	 S 	   386.22	   607.82	 12
  8	 P 	   434.74	   564.31	 11
  9	 A 	   470.26	   515.78	 10
 10	 T 	   520.78	   480.26	  9
 11	 L 	   577.33	   429.74	  8
 12	 S 	   620.84	   373.20	  7
 13	 L 	   677.38	   329.68	  6
 14	 S 	   720.90	   273.14	  5
 15	 P 	   769.43	   229.62	  4
 16	 G 	   797.94	   181.10	  3
 17	 E 	   862.46	   152.58	  2
 18	 R 	        -  	    88.06	  1  _
```

**68. IPI00008787 Alpha-N-acetylglucosaminidase**

| Sequence | Charge | M+H+ | XCorr | DeltaCn |
| --- | --- | --- | --- | --- |
| R.LLGPGPAADFSVSVER.A | 2 | 1615.812 | 4.581 | 0.567 |

+1 charge

```
		      B   	       Y
  1	 L 	   114.09	        -  	 16
  2	 L 	   227.18	  1501.76	 15
  3	 G 	   284.20	  1388.68	 14
  4	 P 	   381.25	  1331.66	 13
  5	 G 	   438.27	  1234.61	 12
  6	 P 	   535.32	  1177.58	 11
  7	 A 	   606.36	  1080.53	 10
  8	 A 	   677.40	  1009.49	  9
  9	 D 	   792.43	   938.46	  8
 10	 F 	   939.49	   823.43	  7
 11	 S 	  1026.53	   676.36	  6
 12	 V 	  1125.59	   589.33	  5
 13	 S 	  1212.63	   490.26	  4
 14	 V 	  1311.69	   403.23	  3
 15	 E 	  1440.74	   304.16	  2
 16	 R 	        -  	   175.12	  1  _
+2 charge
		      B   	       Y
  1	 L 	    57.55	        -  	 16
  2	 L 	   114.09	   751.39	 15
  3	 G 	   142.60	   694.84	 14
  4	 P 	   191.13	   666.33	 13
  5	 G 	   219.64	   617.81	 12
  6	 P 	   268.17	   589.30	 11
  7	 A 	   303.68	   540.77	 10
  8	 A 	   339.20	   505.25	  9
  9	 D 	   396.72	   469.73	  8
 10	 F 	   470.25	   412.22	  7
 11	 S 	   513.77	   338.68	  6
 12	 V 	   563.30	   295.17	  5
 13	 S 	   606.82	   245.63	  4
 14	 V 	   656.35	   202.12	  3
 15	 E 	   720.87	   152.58	  2
 16	 R 	        -  	    88.06	  1  _
```

**69. IPI00387120 Ig kappa chain V-IV region Len**

| Sequence | Charge | M+H+ | XCorr | DeltaCn |
| --- | --- | --- | --- | --- |
| - .DIVMTQSPDSLAVSLGER.A | 2 | 1919.147 | 5.001 | 0.458 |

+1 charge

```
		      B   	       Y
  1	 D 	   116.03	        -  	 18
  2	 I 	   229.12	  1802.93	 17
  3	 V 	   328.19	  1689.85	 16
  4	 M 	   459.23	  1590.78	 15
  5	 T 	   560.27	  1459.74	 14
  6	 Q 	   688.33	  1358.69	 13
  7	 S 	   775.37	  1230.63	 12
  8	 P 	   872.42	  1143.60	 11
  9	 D 	   987.45	  1046.55	 10
 10	 S 	  1074.48	   931.52	  9
 11	 L 	  1187.56	   844.49	  8
 12	 A 	  1258.60	   731.40	  7
 13	 V 	  1357.67	   660.37	  6
 14	 S 	  1444.70	   561.30	  5
 15	 L 	  1557.78	   474.27	  4
 16	 G 	  1614.80	   361.18	  3
 17	 E 	  1743.85	   304.16	  2
 18	 R 	        -  	   175.12	  1  _
+2 charge
		      B   	       Y
  1	 D 	    58.52	        -  	 18
  2	 I 	   115.06	   901.97	 17
  3	 V 	   164.60	   845.43	 16
  4	 M 	   230.12	   795.89	 15
  5	 T 	   280.64	   730.37	 14
  6	 Q 	   344.67	   679.85	 13
  7	 S 	   388.19	   615.82	 12
  8	 P 	   436.71	   572.30	 11
  9	 D 	   494.23	   523.78	 10
 10	 S 	   537.74	   466.26	  9
 11	 L 	   594.28	   422.75	  8
 12	 A 	   629.80	   366.21	  7
 13	 V 	   679.34	   330.69	  6
 14	 S 	   722.85	   281.15	  5
 15	 L 	   779.40	   237.64	  4
 16	 G 	   807.91	   181.10	  3
 17	 E 	   872.43	   152.58	  2
 18	 R 	        -  	    88.06	  1  _
```

**70. IPI00005526 PRO0419**

| Sequence | Charge | M+H+ | XCorr | DeltaCn |
| --- | --- | --- | --- | --- |
| R.RSELFILPQDDLDPPNFPCSR.P | 3 | 2517.772 | 4.673 | 0.225 |

+1 charge

```
		      B   	       Y
  1	 R 	   157.11	        -  	 21
  2	 S 	   244.14	  2360.26	 20
  3	 E 	   373.18	  2273.23	 19
  4	 L 	   486.27	  2144.18	 18
  5	 F 	   633.34	  2031.10	 17
  6	 I 	   746.42	  1884.03	 16
  7	 L 	   859.50	  1770.95	 15
  8	 P 	   956.56	  1657.86	 14
  9	 Q 	  1084.61	  1560.81	 13
 10	 D 	  1199.64	  1432.75	 12
 11	 D 	  1314.67	  1317.72	 11
 12	 L 	  1427.75	  1202.70	 10
 13	 D 	  1542.78	  1089.61	  9
 14	 P 	  1639.83	   974.59	  8
 15	 P 	  1736.89	   877.53	  7
 16	 N 	  1850.93	   780.48	  6
 17	 F 	  1998.00	   666.44	  5
 18	 P 	  2095.05	   519.37	  4
 19	 C 	  2255.21	   422.32	  3
 20	 S 	  2342.25	   262.15	  2
 21	 R 	        -  	   175.12	  1  _


+2 charge
		      B   	       Y
  1	 R 	    79.06	        -  	 21
  2	 S 	   122.57	  1180.63	 20
  3	 E 	   187.10	  1137.12	 19
  4	 L 	   243.64	  1072.59	 18
  5	 F 	   317.17	  1016.05	 17
  6	 I 	   373.71	   942.52	 16
  7	 L 	   430.26	   885.98	 15
  8	 P 	   478.78	   829.43	 14
  9	 Q 	   542.81	   780.91	 13
 10	 D 	   600.32	   716.88	 12
 11	 D 	   657.84	   659.37	 11
 12	 L 	   714.38	   601.85	 10
 13	 D 	   771.89	   545.31	  9
 14	 P 	   820.42	   487.80	  8
 15	 P 	   868.95	   439.27	  7
 16	 N 	   925.97	   390.74	  6
 17	 F 	   999.50	   333.72	  5
 18	 P 	  1048.03	   260.19	  4
 19	 C 	  1128.11	   211.66	  3
 20	 S 	  1171.63	   131.58	  2
 21	 R 	        -  	    88.06	  1  _
+3 charge
		      B   	       Y
  1	 R 	    53.04	        -  	 21
  2	 S 	    82.05	   787.42	 20
  3	 E 	   125.07	   758.41	 19
  4	 L 	   162.76	   715.40	 18
  5	 F 	   211.78	   677.70	 17
  6	 I 	   249.48	   628.68	 16
  7	 L 	   287.17	   590.99	 15
  8	 P 	   319.52	   553.29	 14
  9	 Q 	   362.21	   520.94	 13
 10	 D 	   400.55	   478.26	 12
 11	 D 	   438.89	   439.91	 11
 12	 L 	   476.59	   401.57	 10
 13	 D 	   514.93	   363.88	  9
 14	 P 	   547.28	   325.53	  8
 15	 P 	   579.63	   293.18	  7
 16	 N 	   617.65	   260.83	  6
 17	 F 	   666.67	   222.82	  5
 18	 P 	   699.02	   173.79	  4
 19	 C 	   752.41	   141.44	  3
 20	 S 	   781.42	    88.06	  2
 21	 R 	        -  	    59.04	  1  _
```

**71. IPI00304273 Apolipoprotein A-IV**

| Sequence | Charge | M+H+ | XCorr | DeltaCn |
| --- | --- | --- | --- | --- |
| R.RVEPYGENFNKALVQQMEQLR.Q | 3 | 2550.878 | 3.573 | 0.388 |

+1 charge

```
		      B   	       Y
  1	 R 	   157.11	        -  	 21
  2	 V 	   256.18	  2393.19	 20
  3	 E 	   385.22	  2294.12	 19
  4	 P 	   482.27	  2165.08	 18
  5	 Y 	   645.34	  2068.03	 17
  6	 G 	   702.36	  1904.96	 16
  7	 E 	   831.40	  1847.94	 15
  8	 N 	   945.44	  1718.90	 14
  9	 F 	  1092.51	  1604.86	 13
 10	 N 	  1206.55	  1457.79	 12
 11	 K 	  1334.65	  1343.75	 11
 12	 A 	  1405.69	  1215.65	 10
 13	 L 	  1518.77	  1144.61	  9
 14	 V 	  1617.84	  1031.53	  8
 15	 Q 	  1745.90	   932.46	  7
 16	 Q 	  1873.96	   804.40	  6
 17	 M 	  2005.00	   676.34	  5
 18	 E 	  2134.04	   545.30	  4
 19	 Q 	  2262.10	   416.26	  3
 20	 L 	  2375.18	   288.20	  2
 21	 R 	        -  	   175.12	  1  _


+2 charge
		      B   	       Y
  1	 R 	    79.06	        -  	 21
  2	 V 	   128.59	  1197.10	 20
  3	 E 	   193.11	  1147.57	 19
  4	 P 	   241.64	  1083.04	 18
  5	 Y 	   323.17	  1034.52	 17
  6	 G 	   351.68	   952.99	 16
  7	 E 	   416.20	   924.48	 15
  8	 N 	   473.22	   859.95	 14
  9	 F 	   546.76	   802.93	 13
 10	 N 	   603.78	   729.40	 12
 11	 K 	   667.83	   672.38	 11
 12	 A 	   703.35	   608.33	 10
 13	 L 	   759.89	   572.81	  9
 14	 V 	   809.42	   516.27	  8
 15	 Q 	   873.45	   466.73	  7
 16	 Q 	   937.48	   402.71	  6
 17	 M 	  1003.00	   338.68	  5
 18	 E 	  1067.52	   273.16	  4
 19	 Q 	  1131.55	   208.63	  3
 20	 L 	  1188.09	   144.61	  2
 21	 R 	        -  	    88.06	  1  _
+3 charge
		      B   	       Y
  1	 R 	    53.04	        -  	 21
  2	 V 	    86.06	   798.40	 20
  3	 E 	   129.08	   765.38	 19
  4	 P 	   161.43	   722.37	 18
  5	 Y 	   215.78	   690.01	 17
  6	 G 	   234.79	   635.66	 16
  7	 E 	   277.80	   616.65	 15
  8	 N 	   315.82	   573.64	 14
  9	 F 	   364.84	   535.62	 13
 10	 N 	   402.86	   486.60	 12
 11	 K 	   445.55	   448.59	 11
 12	 A 	   469.23	   405.89	 10
 13	 L 	   506.93	   382.21	  9
 14	 V 	   539.95	   344.51	  8
 15	 Q 	   582.64	   311.49	  7
 16	 Q 	   625.32	   268.81	  6
 17	 M 	   669.00	   226.12	  5
 18	 E 	   712.02	   182.44	  4
 19	 Q 	   754.70	   139.43	  3
 20	 L 	   792.40	    96.74	  2
 21	 R 	        -  	    59.04	  1  _
```

**72. IPI00414888 Isoform 2 of ICOS ligand**

| Sequence | Charge | M+H+ | XCorr | DeltaCn |
| --- | --- | --- | --- | --- |
| R.GLYDVVSVLR.I | 2 | 1121.311 | 3.859 | 0.394 |

+1 charge

```
		      B   	       Y
  1	 G 	    58.03	        -  	 10
  2	 L 	   171.11	  1063.61	  9
  3	 Y 	   334.18	   950.53	  8
  4	 D 	   449.20	   787.47	  7
  5	 V 	   548.27	   672.44	  6
  6	 V 	   647.34	   573.37	  5
  7	 S 	   734.37	   474.30	  4
  8	 V 	   833.44	   387.27	  3
  9	 L 	   946.52	   288.20	  2
 10	 R 	        -  	   175.12	  1  _
+2 charge
		      B   	       Y
  1	 G 	    29.52	        -  	 10
  2	 L 	    86.06	   532.31	  9
  3	 Y 	   167.59	   475.77	  8
  4	 D 	   225.11	   394.24	  7
  5	 V 	   274.64	   336.72	  6
  6	 V 	   324.17	   287.19	  5
  7	 S 	   367.69	   237.66	  4
  8	 V 	   417.22	   194.14	  3
  9	 L 	   473.77	   144.61	  2
 10	 R 	        -  	    88.06	  1  _
```

**73. IPI00297420 Frizzled-4 precursor**

| Sequence | Charge | M+H+ | XCorr | DeltaCn |
| --- | --- | --- | --- | --- |
| K.INIPIGPCGGMCLSVK.R | 2 | 1717.054 | 3.024 | 0.383 |

+1 charge

```
		      B   	       Y
  1	 I 	   114.09	        -  	 16
  2	 N 	   228.13	  1603.05	 15
  3	 I 	   341.22	  1489.01	 14
  4	 P 	   438.27	  1375.92	 13
  5	 I 	   551.36	  1278.87	 12
  6	 G 	   608.38	  1165.79	 11
  7	 P 	   705.43	  1108.76	 10
  8	 C 	   865.59	  1011.71	  9
  9	 G 	   922.62	   851.55	  8
 10	 G 	   979.64	   794.52	  7
 11	 M 	  1110.68	   737.50	  6
 12	 C 	  1270.84	   606.46	  5
 13	 L 	  1383.93	   446.30	  4
 14	 S 	  1470.96	   333.21	  3
 15	 V 	  1570.03	   246.18	  2
 16	 K 	        -  	   147.11	  1  _
+2 charge
		      B   	       Y
  1	 I 	    57.55	        -  	 16
  2	 N 	   114.57	   802.03	 15
  3	 I 	   171.11	   745.01	 14
  4	 P 	   219.64	   688.46	 13
  5	 I 	   276.18	   639.94	 12
  6	 G 	   304.69	   583.40	 11
  7	 P 	   353.22	   554.89	 10
  8	 C 	   433.30	   506.36	  9
  9	 G 	   461.81	   426.28	  8
 10	 G 	   490.32	   397.77	  7
 11	 M 	   555.84	   369.26	  6
 12	 C 	   635.93	   303.73	  5
 13	 L 	   692.47	   223.65	  4
 14	 S 	   735.98	   167.11	  3
 15	 V 	   785.52	   123.59	  2
 16	 K 	        -  	    74.06	  1  _
```

**74. IPI00736885 Ig kappa chain V-II region TEW**

| Sequence | Charge | M+H+ | XCorr | DeltaCn |
| --- | --- | --- | --- | --- |
| - .DIVMTQSPLSLPVTPGEPASISCR.S | 3 | 2556.911 | 3.712 | 0.437 |

+1 charge

```
		      B   	       Y
  1	 D 	   116.03	        -  	 24
  2	 I 	   229.12	  2440.39	 23
  3	 V 	   328.19	  2327.31	 22
  4	 M 	   459.23	  2228.24	 21
  5	 T 	   560.27	  2097.20	 20
  6	 Q 	   688.33	  1996.15	 19
  7	 S 	   775.37	  1868.09	 18
  8	 P 	   872.42	  1781.06	 17
  9	 L 	   985.50	  1684.01	 16
 10	 S 	  1072.53	  1570.92	 15
 11	 L 	  1185.62	  1483.89	 14
 12	 P 	  1282.67	  1370.81	 13
 13	 V 	  1381.74	  1273.76	 12
 14	 T 	  1482.79	  1174.69	 11
 15	 P 	  1579.84	  1073.64	 10
 16	 G 	  1636.86	   976.59	  9
 17	 E 	  1765.90	   919.56	  8
 18	 P 	  1862.96	   790.52	  7
 19	 A 	  1933.99	   693.47	  6
 20	 S 	  2021.03	   622.43	  5
 21	 I 	  2134.11	   535.40	  4
 22	 S 	  2221.14	   422.32	  3
 23	 C 	  2381.31	   335.28	  2
 24	 R 	        -  	   175.12	  1  _


+2 charge
		      B   	       Y
  1	 D 	    58.52	        -  	 24
  2	 I 	   115.06	  1220.70	 23
  3	 V 	   164.60	  1164.16	 22
  4	 M 	   230.12	  1114.62	 21
  5	 T 	   280.64	  1049.10	 20
  6	 Q 	   344.67	   998.58	 19
  7	 S 	   388.19	   934.55	 18
  8	 P 	   436.71	   891.03	 17
  9	 L 	   493.25	   842.51	 16
 10	 S 	   536.77	   785.97	 15
 11	 L 	   593.31	   742.45	 14
 12	 P 	   641.84	   685.91	 13
 13	 V 	   691.37	   637.38	 12
 14	 T 	   741.90	   587.85	 11
 15	 P 	   790.42	   537.32	 10
 16	 G 	   818.93	   488.80	  9
 17	 E 	   883.46	   460.29	  8
 18	 P 	   931.98	   395.76	  7
 19	 A 	   967.50	   347.24	  6
 20	 S 	  1011.02	   311.72	  5
 21	 I 	  1067.56	   268.20	  4
 22	 S 	  1111.07	   211.66	  3
 23	 C 	  1191.16	   168.15	  2
 24	 R 	        -  	    88.06	  1  _


+3 charge
		      B   	       Y
  1	 D 	    39.35	        -  	 24
  2	 I 	    77.04	   814.14	 23
  3	 V 	   110.07	   776.44	 22
  4	 M 	   153.75	   743.42	 21
  5	 T 	   187.43	   699.74	 20
  6	 Q 	   230.12	   666.06	 19
  7	 S 	   259.13	   623.37	 18
  8	 P 	   291.48	   594.36	 17
  9	 L 	   329.17	   562.01	 16
 10	 S 	   358.18	   524.31	 15
 11	 L 	   395.88	   495.30	 14
 12	 P 	   428.23	   457.61	 13
 13	 V 	   461.25	   425.26	 12
 14	 T 	   494.93	   392.23	 11
 15	 P 	   527.28	   358.55	 10
 16	 G 	   546.29	   326.20	  9
 17	 E 	   589.31	   307.19	  8
 18	 P 	   621.66	   264.18	  7
 19	 A 	   645.34	   231.83	  6
 20	 S 	   674.35	   208.15	  5
 21	 I 	   712.04	   179.14	  4
 22	 S 	   741.05	   141.44	  3
 23	 C 	   794.44	   112.43	  2
 24	 R 	        -  	    59.04	  1  _
```

**75. IPI00374975 Probable phosphoglycerate mutase 4**

| Sequence | Charge | M+H+ | XCorr | DeltaCn |
| --- | --- | --- | --- | --- |
| R.ALPFWNEEIVPQIK.A | 2 | 1684.959 | 2.851 | 0.288 |

+1 charge

```
		      B   	       Y
  1	 A 	    72.04	        -  	 14
  2	 L 	   185.13	  1612.87	 13
  3	 P 	   282.18	  1499.79	 12
  4	 F 	   429.25	  1402.74	 11
  5	 W 	   615.33	  1255.67	 10
  6	 N 	   729.37	  1069.59	  9
  7	 E 	   858.41	   955.55	  8
  8	 E 	   987.46	   826.50	  7
  9	 I 	  1100.54	   697.46	  6
 10	 V 	  1199.61	   584.38	  5
 11	 P 	  1296.66	   485.31	  4
 12	 Q 	  1424.72	   388.26	  3
 13	 I 	  1537.80	   260.20	  2
 14	 K 	        -  	   147.11	  1  _
+2 charge
		      B   	       Y
  1	 A 	    36.53	        -  	 14
  2	 L 	    93.07	   806.94	 13
  3	 P 	   141.59	   750.40	 12
  4	 F 	   215.13	   701.87	 11
  5	 W 	   308.17	   628.34	 10
  6	 N 	   365.19	   535.30	  9
  7	 E 	   429.71	   478.28	  8
  8	 E 	   494.23	   413.76	  7
  9	 I 	   550.77	   349.23	  6
 10	 V 	   600.31	   292.69	  5
 11	 P 	   648.83	   243.16	  4
 12	 Q 	   712.86	   194.63	  3
 13	 I 	   769.41	   130.60	  2
 14	 K 	        -  	    74.06	  1  _
```

**76. IPI00219217 L-lactate dehydrogenase B chain**

| Sequence | Charge | M+H+ | XCorr | DeltaCn |
| --- | --- | --- | --- | --- |
| K.SADTLWDIQKDLKDL.- | 2 | 1761.954 | 3.12 | 0.362 |

+1 charge

```
		      B   	       Y
  1	 S 	    88.04	        -  	 15
  2	 A 	   159.08	  1673.87	 14
  3	 D 	   274.10	  1602.84	 13
  4	 T 	   375.15	  1487.81	 12
  5	 L 	   488.24	  1386.76	 11
  6	 W 	   674.31	  1273.68	 10
  7	 D 	   789.34	  1087.60	  9
  8	 I 	   902.43	   972.57	  8
  9	 Q 	  1030.48	   859.49	  7
 10	 K 	  1158.58	   731.43	  6
 11	 D 	  1273.61	   603.33	  5
 12	 L 	  1386.69	   488.31	  4
 13	 K 	  1514.78	   375.22	  3
 14	 D 	  1629.81	   247.13	  2
 15	 L 	        -  	   132.10	  1  _
+2 charge
		      B   	       Y
  1	 S 	    44.52	        -  	 15
  2	 A 	    80.04	   837.44	 14
  3	 D 	   137.56	   801.92	 13
  4	 T 	   188.08	   744.41	 12
  5	 L 	   244.62	   693.89	 11
  6	 W 	   337.66	   637.34	 10
  7	 D 	   395.17	   544.30	  9
  8	 I 	   451.72	   486.79	  8
  9	 Q 	   515.75	   430.25	  7
 10	 K 	   579.79	   366.22	  6
 11	 D 	   637.31	   302.17	  5
 12	 L 	   693.85	   244.66	  4
 13	 K 	   757.90	   188.12	  3
 14	 D 	   815.41	   124.07	  2
 15	 L 	        -  	    66.55	  1  _
```

**77. IPI00940158 trefoil factor 3 precursor**

| Sequence | Charge | M+H+ | XCorr | DeltaCn |
| --- | --- | --- | --- | --- |
| R.IPGVPWCFKPLQEAECTF.- | 2 | 2180.474 | 3.298 | 0.491 |

+1 charge

```
		      B   	       Y
  1	 I 	   114.09	        -  	 18
  2	 P 	   211.14	  2066.22	 17
  3	 G 	   268.17	  1969.17	 16
  4	 V 	   367.23	  1912.15	 15
  5	 P 	   464.29	  1813.08	 14
  6	 W 	   650.37	  1716.02	 13
  7	 C 	   810.53	  1529.95	 12
  8	 F 	   957.60	  1369.78	 11
  9	 K 	  1085.69	  1222.71	 10
 10	 P 	  1182.75	  1094.62	  9
 11	 L 	  1295.83	   997.56	  8
 12	 Q 	  1423.89	   884.48	  7
 13	 E 	  1552.93	   756.42	  6
 14	 A 	  1623.97	   627.38	  5
 15	 E 	  1753.01	   556.34	  4
 16	 C 	  1913.18	   427.30	  3
 17	 T 	  2014.23	   267.13	  2
 18	 F 	        -  	   166.09	  1  _
+2 charge
		      B   	       Y
  1	 I 	    57.55	        -  	 18
  2	 P 	   106.08	  1033.61	 17
  3	 G 	   134.59	   985.09	 16
  4	 V 	   184.12	   956.58	 15
  5	 P 	   232.65	   907.04	 14
  6	 W 	   325.69	   858.52	 13
  7	 C 	   405.77	   765.48	 12
  8	 F 	   479.30	   685.39	 11
  9	 K 	   543.35	   611.86	 10
 10	 P 	   591.88	   547.81	  9
 11	 L 	   648.42	   499.29	  8
 12	 Q 	   712.45	   442.74	  7
 13	 E 	   776.97	   378.71	  6
 14	 A 	   812.49	   314.19	  5
 15	 E 	   877.01	   278.67	  4
 16	 C 	   957.09	   214.15	  3
 17	 T 	  1007.62	   134.07	  2
 18	 F 	        -  	    83.55	  1  _
```

**78. IPI00012503 Isoform Sap-mu-0 of Proactivator polypeptide**

| Sequence | Charge | M+H+ | XCorr | DeltaCn |
| --- | --- | --- | --- | --- |
| K.NYISQYSEIAIQMMMHMQPK.E | 3 | 2444.903 | 3.519 | 0.546 |

+1 charge

```
		      B   	       Y
  1	 N 	   115.05	        -  	 20
  2	 Y 	   278.11	  2329.08	 19
  3	 I 	   391.20	  2166.02	 18
  4	 S 	   478.23	  2052.94	 17
  5	 Q 	   606.29	  1965.91	 16
  6	 Y 	   769.35	  1837.85	 15
  7	 S 	   856.38	  1674.78	 14
  8	 E 	   985.43	  1587.75	 13
  9	 I 	  1098.51	  1458.71	 12
 10	 A 	  1169.55	  1345.62	 11
 11	 I 	  1282.63	  1274.59	 10
 12	 Q 	  1410.69	  1161.50	  9
 13	 M 	  1541.73	  1033.44	  8
 14	 M 	  1672.77	   902.40	  7
 15	 M 	  1803.81	   771.36	  6
 16	 H 	  1940.87	   640.32	  5
 17	 M 	  2071.91	   503.26	  4
 18	 Q 	  2199.97	   372.22	  3
 19	 P 	  2297.02	   244.17	  2
 20	 K 	        -  	   147.11	  1  _
+2 charge
		      B   	       Y
  1	 N 	    58.03	        -  	 20
  2	 Y 	   139.56	  1165.05	 19
  3	 I 	   196.10	  1083.51	 18
  4	 S 	   239.62	  1026.97	 17
  5	 Q 	   303.65	   983.46	 16
  6	 Y 	   385.18	   919.43	 15
  7	 S 	   428.70	   837.90	 14
  8	 E 	   493.22	   794.38	 13
  9	 I 	   549.76	   729.86	 12
 10	 A 	   585.28	   673.32	 11
 11	 I 	   641.82	   637.80	 10
 12	 Q 	   705.85	   581.26	  9
 13	 M 	   771.37	   517.23	  8
 14	 M 	   836.89	   451.71	  7
 15	 M 	   902.41	   386.19	  6
 16	 H 	   970.94	   320.67	  5
 17	 M 	  1036.46	   252.14	  4
 18	 Q 	  1100.49	   186.62	  3
 19	 P 	  1149.01	   122.59	  2
 20	 K 	        -  	    74.06	  1  _

+3 charge
		      B   	       Y
  1	 N 	    39.02	        -  	 20
  2	 Y 	    93.38	   777.03	 19
  3	 I 	   131.07	   722.68	 18
  4	 S 	   160.08	   684.98	 17
  5	 Q 	   202.77	   655.97	 16
  6	 Y 	   257.12	   613.29	 15
  7	 S 	   286.13	   558.93	 14
  8	 E 	   329.15	   529.92	 13
  9	 I 	   366.84	   486.91	 12
 10	 A 	   390.52	   449.21	 11
 11	 I 	   428.22	   425.53	 10
 12	 Q 	   470.90	   387.84	  9
 13	 M 	   514.58	   345.15	  8
 14	 M 	   558.26	   301.47	  7
 15	 M 	   601.94	   257.79	  6
 16	 H 	   647.63	   214.11	  5
 17	 M 	   691.31	   168.43	  4
 18	 Q 	   733.99	   124.75	  3
 19	 P 	   766.35	    82.06	  2
 20	 K 	        -  	    49.71	  1  _
```

**79. IPI00011662 Kunitz-type protease inhibitor 2**

| Sequence | Charge | M+H+ | XCorr | DeltaCn |
| --- | --- | --- | --- | --- |
| R.SIHDFCLVSK.V | 2 | 1206.367 | 3.035 | 0.309 |

+1 charge

```
		      B   	       Y
  1	 S 	    88.04	        -  	 10
  2	 I 	   201.12	  1118.70	  9
  3	 H 	   338.18	  1005.62	  8
  4	 D 	   453.21	   868.56	  7
  5	 F 	   600.28	   753.53	  6
  6	 C 	   760.44	   606.46	  5
  7	 L 	   873.53	   446.30	  4
  8	 V 	   972.60	   333.21	  3
  9	 S 	  1059.63	   234.14	  2
 10	 K 	        -  	   147.11	  1  _
+2 charge
		      B   	       Y
  1	 S 	    44.52	        -  	 10
  2	 I 	   101.07	   559.85	  9
  3	 H 	   169.59	   503.31	  8
  4	 D 	   227.11	   434.78	  7
  5	 F 	   300.64	   377.27	  6
  6	 C 	   380.73	   303.73	  5
  7	 L 	   437.27	   223.65	  4
  8	 V 	   486.80	   167.11	  3
  9	 S 	   530.32	   117.58	  2
 10	 K 	        -  	    74.06	  1  _
```

**80. IPI00433028 Isoform 1 of Leukocyte-associated immunoglobulin-like receptor 1**

| Sequence | Charge | M+H+ | XCorr | DeltaCn |
| --- | --- | --- | --- | --- |
| R.FRIDSVSEGNAGPYR.C | 2 | 1668.792 | 4.485 | 0.414 |

+1 charge

```
		      B   	       Y
  1	 F 	   148.08	        -  	 15
  2	 R 	   304.18	  1520.75	 14
  3	 I 	   417.26	  1364.64	 13
  4	 D 	   532.29	  1251.56	 12
  5	 S 	   619.32	  1136.53	 11
  6	 V 	   718.39	  1049.50	 10
  7	 S 	   805.42	   950.43	  9
  8	 E 	   934.46	   863.40	  8
  9	 G 	   991.48	   734.36	  7
 10	 N 	  1105.53	   677.34	  6
 11	 A 	  1176.56	   563.29	  5
 12	 G 	  1233.59	   492.26	  4
 13	 P 	  1330.64	   435.24	  3
 14	 Y 	  1493.70	   338.18	  2
 15	 R 	        -  	   175.12	  1  _
+2 charge
		      B   	       Y
  1	 F 	    74.54	        -  	 15
  2	 R 	   152.59	   760.88	 14
  3	 I 	   209.13	   682.83	 13
  4	 D 	   266.65	   626.28	 12
  5	 S 	   310.16	   568.77	 11
  6	 V 	   359.70	   525.25	 10
  7	 S 	   403.21	   475.72	  9
  8	 E 	   467.74	   432.20	  8
  9	 G 	   496.25	   367.68	  7
 10	 N 	   553.27	   339.17	  6
 11	 A 	   588.79	   282.15	  5
 12	 G 	   617.30	   246.63	  4
 13	 P 	   665.82	   218.12	  3
 14	 Y 	   747.35	   169.59	  2
 15	 R 	        -  	    88.06	  1  _
```

**81. IPI00298388 Isoform 1 of Phosphoinositide-3-kinase-interacting protein 1**

| Sequence | Charge | M+H+ | XCorr | DeltaCn |
| --- | --- | --- | --- | --- |
| R.SEAAAVQPVIGISQR.V | 2 | 1526.72 | 3.748 | 0.45 |

+1 charge

```
		      B   	       Y
  1	 S 	    88.04	        -  	 15
  2	 E 	   217.08	  1438.80	 14
  3	 A 	   288.12	  1309.76	 13
  4	 A 	   359.16	  1238.72	 12
  5	 A 	   430.19	  1167.68	 11
  6	 V 	   529.26	  1096.65	 10
  7	 Q 	   657.32	   997.58	  9
  8	 P 	   754.37	   869.52	  8
  9	 V 	   853.44	   772.47	  7
 10	 I 	   966.53	   673.40	  6
 11	 G 	  1023.55	   560.32	  5
 12	 I 	  1136.63	   503.29	  4
 13	 S 	  1223.66	   390.21	  3
 14	 Q 	  1351.72	   303.18	  2
 15	 R 	        -  	   175.12	  1  _
+2 charge
		      B   	       Y
  1	 S 	    44.52	        -  	 15
  2	 E 	   109.04	   719.90	 14
  3	 A 	   144.56	   655.38	 13
  4	 A 	   180.08	   619.86	 12
  5	 A 	   215.60	   584.35	 11
  6	 V 	   265.13	   548.83	 10
  7	 Q 	   329.16	   499.29	  9
  8	 P 	   377.69	   435.26	  8
  9	 V 	   427.22	   386.74	  7
 10	 I 	   483.77	   337.20	  6
 11	 G 	   512.28	   280.66	  5
 12	 I 	   568.82	   252.15	  4
 13	 S 	   612.34	   195.61	  3
 14	 Q 	   676.36	   152.09	  2
 15	 R 	        -  	    88.06	  1  _
```

**82. IPI00478816 Serine protease inhibitor Kazal-type 5**

| Sequence | Charge | M+H+ | XCorr | DeltaCn |
| --- | --- | --- | --- | --- |
| K.DLKPVCGDDGQTYNNPCMLCHENLIR.Q | 3 | 3121.409 | 4.213 | 0.493 |

+1 charge

```
		      B   	       Y
  1	 D 	   116.03	        -  	 26
  2	 L 	   229.12	  3004.76	 25
  3	 K 	   357.21	  2891.67	 24
  4	 P 	   454.27	  2763.58	 23
  5	 V 	   553.33	  2666.53	 22
  6	 C 	   713.50	  2567.46	 21
  7	 G 	   770.52	  2407.29	 20
  8	 D 	   885.55	  2350.27	 19
  9	 D 	  1000.58	  2235.24	 18
 10	 G 	  1057.60	  2120.22	 17
 11	 Q 	  1185.66	  2063.19	 16
 12	 T 	  1286.70	  1935.14	 15
 13	 Y 	  1449.77	  1834.09	 14
 14	 N 	  1563.81	  1671.03	 13
 15	 N 	  1677.85	  1556.98	 12
 16	 P 	  1774.90	  1442.94	 11
 17	 C 	  1935.07	  1345.89	 10
 18	 M 	  2066.11	  1185.72	  9
 19	 L 	  2179.19	  1054.68	  8
 20	 C 	  2339.36	   941.60	  7
 21	 H 	  2476.42	   781.43	  6
 22	 E 	  2605.46	   644.37	  5
 23	 N 	  2719.50	   515.33	  4
 24	 L 	  2832.59	   401.29	  3
 25	 I 	  2945.67	   288.20	  2
 26	 R 	        -  	   175.12	  1  _


+2 charge
		      B   	       Y
  1	 D 	    58.52	        -  	 26
  2	 L 	   115.06	  1502.88	 25
  3	 K 	   179.11	  1446.34	 24
  4	 P 	   227.64	  1382.29	 23
  5	 V 	   277.17	  1333.77	 22
  6	 C 	   357.25	  1284.23	 21
  7	 G 	   385.76	  1204.15	 20
  8	 D 	   443.28	  1175.64	 19
  9	 D 	   500.79	  1118.13	 18
 10	 G 	   529.30	  1060.61	 17
 11	 Q 	   593.33	  1032.10	 16
 12	 T 	   643.86	   968.07	 15
 13	 Y 	   725.39	   917.55	 14
 14	 N 	   782.41	   836.02	 13
 15	 N 	   839.43	   778.99	 12
 16	 P 	   887.96	   721.97	 11
 17	 C 	   968.04	   673.45	 10
 18	 M 	  1033.56	   593.36	  9
 19	 L 	  1090.10	   527.84	  8
 20	 C 	  1170.18	   471.30	  7
 21	 H 	  1238.71	   391.22	  6
 22	 E 	  1303.23	   322.69	  5
 23	 N 	  1360.26	   258.17	  4
 24	 L 	  1416.80	   201.15	  3
 25	 I 	  1473.34	   144.61	  2
 26	 R 	        -  	    88.06	  1  _


+3 charge
		      B   	       Y
  1	 D 	    39.35	        -  	 26
  2	 L 	    77.04	  1002.26	 25
  3	 K 	   119.74	   964.56	 24
  4	 P 	   152.09	   921.86	 23
  5	 V 	   185.12	   889.51	 22
  6	 C 	   238.50	   856.49	 21
  7	 G 	   257.51	   803.10	 20
  8	 D 	   295.85	   784.09	 19
  9	 D 	   334.20	   745.75	 18
 10	 G 	   353.20	   707.41	 17
 11	 Q 	   395.89	   688.40	 16
 12	 T 	   429.57	   645.72	 15
 13	 Y 	   483.93	   612.03	 14
 14	 N 	   521.94	   557.68	 13
 15	 N 	   559.96	   519.67	 12
 16	 P 	   592.31	   481.65	 11
 17	 C 	   645.69	   449.30	 10
 18	 M 	   689.37	   395.91	  9
 19	 L 	   727.07	   352.23	  8
 20	 C 	   780.46	   314.54	  7
 21	 H 	   826.14	   261.15	  6
 22	 E 	   869.16	   215.46	  5
 23	 N 	   907.17	   172.45	  4
 24	 L 	   944.87	   134.43	  3
 25	 I 	   982.56	    96.74	  2
 26	 R 	        -  	    59.04	  1  _
```

**83. IPI00011694 Trypsin-1**

| Sequence | Charge | M+H+ | XCorr | DeltaCn |
| --- | --- | --- | --- | --- |
| K.TLNNDIMLIK.L | 2 | 1175.424 | 3.513 | 0.313 |

+1 charge

```
		      B   	       Y
  1	 T 	   102.05	        -  	 10
  2	 L 	   215.14	  1073.60	  9
  3	 N 	   329.18	   960.52	  8
  4	 N 	   443.22	   846.48	  7
  5	 D 	   558.25	   732.43	  6
  6	 I 	   671.34	   617.41	  5
  7	 M 	   802.38	   504.32	  4
  8	 L 	   915.46	   373.28	  3
  9	 I 	  1028.54	   260.20	  2
 10	 K 	        -  	   147.11	  1  _
+2 charge
		      B   	       Y
  1	 T 	    51.53	        -  	 10
  2	 L 	   108.07	   537.30	  9
  3	 N 	   165.09	   480.76	  8
  4	 N 	   222.12	   423.74	  7
  5	 D 	   279.63	   366.72	  6
  6	 I 	   336.17	   309.21	  5
  7	 M 	   401.69	   252.66	  4
  8	 L 	   458.23	   187.14	  3
  9	 I 	   514.78	   130.60	  2
 10	 K 	        -  	    74.06	  1  _
```

**84. IPI00829834 Ig kappa chain V-III region VH (Fragment)**

| Sequence | Charge | M+H+ | XCorr | DeltaCn |
| --- | --- | --- | --- | --- |
| R.EIVMTQSPPTLSLSPGER.V | 2 | 1943.211 | 3.259 | 0.434 |

+1 charge

```
		      B   	       Y
  1	 E 	   130.05	        -  	 18
  2	 I 	   243.13	  1812.95	 17
  3	 V 	   342.20	  1699.87	 16
  4	 M 	   473.24	  1600.80	 15
  5	 T 	   574.29	  1469.76	 14
  6	 Q 	   702.35	  1368.71	 13
  7	 S 	   789.38	  1240.65	 12
  8	 P 	   886.43	  1153.62	 11
  9	 P 	   983.49	  1056.57	 10
 10	 T 	  1084.53	   959.52	  9
 11	 L 	  1197.62	   858.47	  8
 12	 S 	  1284.65	   745.38	  7
 13	 L 	  1397.73	   658.35	  6
 14	 S 	  1484.77	   545.27	  5
 15	 P 	  1581.82	   458.24	  4
 16	 G 	  1638.84	   361.18	  3
 17	 E 	  1767.88	   304.16	  2
 18	 R 	        -  	   175.12	  1  _
+2 charge
		      B   	       Y
  1	 E 	    65.53	        -  	 18
  2	 I 	   122.07	   906.98	 17
  3	 V 	   171.60	   850.44	 16
  4	 M 	   237.13	   800.90	 15
  5	 T 	   287.65	   735.38	 14
  6	 Q 	   351.68	   684.86	 13
  7	 S 	   395.19	   620.83	 12
  8	 P 	   443.72	   577.31	 11
  9	 P 	   492.25	   528.79	 10
 10	 T 	   542.77	   480.26	  9
 11	 L 	   599.31	   429.74	  8
 12	 S 	   642.83	   373.20	  7
 13	 L 	   699.37	   329.68	  6
 14	 S 	   742.89	   273.14	  5
 15	 P 	   791.41	   229.62	  4
 16	 G 	   819.92	   181.10	  3
 17	 E 	   884.45	   152.58	  2
 18	 R 	        -  	    88.06	  1  _
```

**85. IPI00382478 Ig heavy chain V-III region TIL**

| Sequence | Charge | M+H+ | XCorr | DeltaCn |
| --- | --- | --- | --- | --- |
| - .EVQLLESGGGLVQPGGSLR.L | 2 | 1897.122 | 4.414 | 0.272 |

+1 charge

```
		      B   	       Y
  1	 E 	   130.05	        -  	 19
  2	 V 	   229.12	  1766.98	 18
  3	 Q 	   357.18	  1667.91	 17
  4	 L 	   470.26	  1539.85	 16
  5	 L 	   583.34	  1426.76	 15
  6	 E 	   712.39	  1313.68	 14
  7	 S 	   799.42	  1184.64	 13
  8	 G 	   856.44	  1097.61	 12
  9	 G 	   913.46	  1040.58	 11
 10	 G 	   970.48	   983.56	 10
 11	 L 	  1083.57	   926.54	  9
 12	 V 	  1182.64	   813.46	  8
 13	 Q 	  1310.70	   714.39	  7
 14	 P 	  1407.75	   586.33	  6
 15	 G 	  1464.77	   489.28	  5
 16	 G 	  1521.79	   432.26	  4
 17	 S 	  1608.82	   375.24	  3
 18	 L 	  1721.91	   288.20	  2
 19	 R 	        -  	   175.12	  1  _
+2 charge
		      B   	       Y
  1	 E 	    65.53	        -  	 19
  2	 V 	   115.06	   883.99	 18
  3	 Q 	   179.09	   834.46	 17
  4	 L 	   235.63	   770.43	 16
  5	 L 	   292.18	   713.89	 15
  6	 E 	   356.70	   657.34	 14
  7	 S 	   400.21	   592.82	 13
  8	 G 	   428.72	   549.31	 12
  9	 G 	   457.23	   520.80	 11
 10	 G 	   485.75	   492.29	 10
 11	 L 	   542.29	   463.77	  9
 12	 V 	   591.82	   407.23	  8
 13	 Q 	   655.85	   357.70	  7
 14	 P 	   704.38	   293.67	  6
 15	 G 	   732.89	   245.14	  5
 16	 G 	   761.40	   216.63	  4
 17	 S 	   804.92	   188.12	  3
 18	 L 	   861.46	   144.61	  2
 19	 R 	        -  	    88.06	  1  _
```

**86. IPI00419585 Peptidyl-prolyl cis-trans isomerase A**

| Sequence | Charge | M+H+ | XCorr | DeltaCn |
| --- | --- | --- | --- | --- |
| R.VSFELFADKVPK.T | 2 | 1380.613 | 3.506 | 0.493 |

+1 charge

```
		      B   	       Y
  1	 V 	   100.08	        -  	 12
  2	 S 	   187.11	  1280.69	 11
  3	 F 	   334.18	  1193.66	 10
  4	 E 	   463.22	  1046.59	  9
  5	 L 	   576.30	   917.55	  8
  6	 F 	   723.37	   804.46	  7
  7	 A 	   794.41	   657.39	  6
  8	 D 	   909.44	   586.36	  5
  9	 K 	  1037.53	   471.33	  4
 10	 V 	  1136.60	   343.23	  3
 11	 P 	  1233.65	   244.17	  2
 12	 K 	        -  	   147.11	  1  _
+2 charge
		      B   	       Y
  1	 V 	    50.54	        -  	 12
  2	 S 	    94.06	   640.85	 11
  3	 F 	   167.59	   597.33	 10
  4	 E 	   232.11	   523.80	  9
  5	 L 	   288.66	   459.28	  8
  6	 F 	   362.19	   402.73	  7
  7	 A 	   397.71	   329.20	  6
  8	 D 	   455.22	   293.68	  5
  9	 K 	   519.27	   236.17	  4
 10	 V 	   568.80	   172.12	  3
 11	 P 	   617.33	   122.59	  2
 12	 K 	        -  	    74.06	  1  _
```

**87. IPI00031065 Deoxyribonuclease-1**

| Sequence | Charge | M+H+ | XCorr | DeltaCn |
| --- | --- | --- | --- | --- |
| K.LLDNLNQDAPDTYHYVVSEPLGR.N | 3 | 2630.851 | 5.059 | 0.395 |

+1 charge

```
		      B   	       Y
  1	 L 	   114.09	        -  	 23
  2	 L 	   227.18	  2516.21	 22
  3	 D 	   342.20	  2403.12	 21
  4	 N 	   456.25	  2288.09	 20
  5	 L 	   569.33	  2174.05	 19
  6	 N 	   683.37	  2060.97	 18
  7	 Q 	   811.43	  1946.92	 17
  8	 D 	   926.46	  1818.87	 16
  9	 A 	   997.49	  1703.84	 15
 10	 P 	  1094.55	  1632.80	 14
 11	 D 	  1209.57	  1535.75	 13
 12	 T 	  1310.62	  1420.72	 12
 13	 Y 	  1473.69	  1319.67	 11
 14	 H 	  1610.74	  1156.61	 10
 15	 Y 	  1773.81	  1019.55	  9
 16	 V 	  1872.88	   856.49	  8
 17	 V 	  1971.94	   757.42	  7
 18	 S 	  2058.98	   658.35	  6
 19	 E 	  2188.02	   571.32	  5
 20	 P 	  2285.07	   442.28	  4
 21	 L 	  2398.16	   345.22	  3
 22	 G 	  2455.18	   232.14	  2
 23	 R 	        -  	   175.12	  1  _


+2 charge
		      B   	       Y
  1	 L 	    57.55	        -  	 23
  2	 L 	   114.09	  1258.61	 22
  3	 D 	   171.60	  1202.06	 21
  4	 N 	   228.63	  1144.55	 20
  5	 L 	   285.17	  1087.53	 19
  6	 N 	   342.19	  1030.99	 18
  7	 Q 	   406.22	   973.97	 17
  8	 D 	   463.73	   909.94	 16
  9	 A 	   499.25	   852.42	 15
 10	 P 	   547.78	   816.90	 14
 11	 D 	   605.29	   768.38	 13
 12	 T 	   655.81	   710.86	 12
 13	 Y 	   737.35	   660.34	 11
 14	 H 	   805.88	   578.81	 10
 15	 Y 	   887.41	   510.28	  9
 16	 V 	   936.94	   428.75	  8
 17	 V 	   986.48	   379.21	  7
 18	 S 	  1029.99	   329.68	  6
 19	 E 	  1094.51	   286.16	  5
 20	 P 	  1143.04	   221.64	  4
 21	 L 	  1199.58	   173.12	  3
 22	 G 	  1228.09	   116.57	  2
 23	 R 	        -  	    88.06	  1  _


+3 charge
		      B   	       Y
  1	 L 	    38.70	        -  	 23
  2	 L 	    76.40	   839.41	 22
  3	 D 	   114.74	   801.71	 21
  4	 N 	   152.75	   763.37	 20
  5	 L 	   190.45	   725.36	 19
  6	 N 	   228.46	   687.66	 18
  7	 Q 	   271.15	   649.65	 17
  8	 D 	   309.49	   606.96	 16
  9	 A 	   333.17	   568.62	 15
 10	 P 	   365.52	   544.94	 14
 11	 D 	   403.86	   512.59	 13
 12	 T 	   437.55	   474.25	 12
 13	 Y 	   491.90	   440.56	 11
 14	 H 	   537.59	   386.21	 10
 15	 Y 	   591.94	   340.52	  9
 16	 V 	   624.96	   286.17	  8
 17	 V 	   657.99	   253.14	  7
 18	 S 	   687.00	   220.12	  6
 19	 E 	   730.01	   191.11	  5
 20	 P 	   762.36	   148.10	  4
 21	 L 	   800.06	   115.75	  3
 22	 G 	   819.06	    78.05	  2
 23	 R 	        -  	    59.04	  1  _
```

**88. IPI00219446 Phosphatidylethanolamine-binding protein 1**

| Sequence | Charge | M+H+ | XCorr | DeltaCn |
| --- | --- | --- | --- | --- |
| R.YVWLVYEQDRPLKCDEPILSNR.S | 3 | 2795.136 | 4.059 | 0.475 |

+1 charge

```
		      B   	       Y
  1	 Y 	   164.07	        -  	 22
  2	 V 	   263.14	  2630.47	 21
  3	 W 	   449.22	  2531.41	 20
  4	 L 	   562.30	  2345.33	 19
  5	 V 	   661.37	  2232.24	 18
  6	 Y 	   824.43	  2133.17	 17
  7	 E 	   953.48	  1970.11	 16
  8	 Q 	  1081.54	  1841.07	 15
  9	 D 	  1196.56	  1713.01	 14
 10	 R 	  1352.66	  1597.98	 13
 11	 P 	  1449.72	  1441.88	 12
 12	 L 	  1562.80	  1344.83	 11
 13	 K 	  1690.90	  1231.74	 10
 14	 C 	  1851.06	  1103.65	  9
 15	 D 	  1966.09	   943.48	  8
 16	 E 	  2095.13	   828.46	  7
 17	 P 	  2192.18	   699.41	  6
 18	 I 	  2305.27	   602.36	  5
 19	 L 	  2418.35	   489.28	  4
 20	 S 	  2505.38	   376.19	  3
 21	 N 	  2619.43	   289.16	  2
 22	 R 	        -  	   175.12	  1  _


+2 charge
		      B   	       Y
  1	 Y 	    82.54	        -  	 22
  2	 V 	   132.07	  1315.74	 21
  3	 W 	   225.11	  1266.21	 20
  4	 L 	   281.65	  1173.17	 19
  5	 V 	   331.19	  1116.62	 18
  6	 Y 	   412.72	  1067.09	 17
  7	 E 	   477.24	   985.56	 16
  8	 Q 	   541.27	   921.04	 15
  9	 D 	   598.78	   857.01	 14
 10	 R 	   676.84	   799.49	 13
 11	 P 	   725.36	   721.44	 12
 12	 L 	   781.90	   672.92	 11
 13	 K 	   845.95	   616.38	 10
 14	 C 	   926.03	   552.33	  9
 15	 D 	   983.55	   472.25	  8
 16	 E 	  1048.07	   414.73	  7
 17	 P 	  1096.59	   350.21	  6
 18	 I 	  1153.14	   301.68	  5
 19	 L 	  1209.68	   245.14	  4
 20	 S 	  1253.20	   188.60	  3
 21	 N 	  1310.22	   145.08	  2
 22	 R 	        -  	    88.06	  1  _


+3 charge
		      B   	       Y
  1	 Y 	    55.36	        -  	 22
  2	 V 	    88.38	   877.50	 21
  3	 W 	   150.41	   844.47	 20
  4	 L 	   188.11	   782.45	 19
  5	 V 	   221.13	   744.75	 18
  6	 Y 	   275.48	   711.73	 17
  7	 E 	   318.50	   657.38	 16
  8	 Q 	   361.18	   614.36	 15
  9	 D 	   399.53	   571.67	 14
 10	 R 	   451.56	   533.33	 13
 11	 P 	   483.91	   481.30	 12
 12	 L 	   521.60	   448.95	 11
 13	 K 	   564.30	   411.25	 10
 14	 C 	   617.69	   368.55	  9
 15	 D 	   656.03	   315.17	  8
 16	 E 	   699.05	   276.82	  7
 17	 P 	   731.40	   233.81	  6
 18	 I 	   769.09	   201.46	  5
 19	 L 	   806.79	   163.76	  4
 20	 S 	   835.80	   126.07	  3
 21	 N 	   873.81	    97.06	  2
 22	 R 	        -  	    59.04	  1  _
```

**89. IPI00409698 Isoform 1 of Transportin-2**

| Sequence | Charge | M+H+ | XCorr | DeltaCn |
| --- | --- | --- | --- | --- |
| K.QECLNNIGDASSLIR.A | 2 | 1690.831 | 3.151 | 0.263 |

+1 charge

```
		      B   	       Y
  1	 Q 	   129.07	        -  	 15
  2	 E 	   258.11	  1561.90	 14
  3	 C 	   418.27	  1432.86	 13
  4	 L 	   531.36	  1272.69	 12
  5	 N 	   645.40	  1159.61	 11
  6	 N 	   759.44	  1045.56	 10
  7	 I 	   872.53	   931.52	  9
  8	 G 	   929.55	   818.44	  8
  9	 D 	  1044.58	   761.42	  7
 10	 A 	  1115.61	   646.39	  6
 11	 S 	  1202.65	   575.35	  5
 12	 S 	  1289.68	   488.32	  4
 13	 L 	  1402.76	   401.29	  3
 14	 I 	  1515.85	   288.20	  2
 15	 R 	        -  	   175.12	  1  _
+2 charge
		      B   	       Y
  1	 Q 	    65.04	        -  	 15
  2	 E 	   129.56	   781.45	 14
  3	 C 	   209.64	   716.93	 13
  4	 L 	   266.18	   636.85	 12
  5	 N 	   323.20	   580.31	 11
  6	 N 	   380.23	   523.29	 10
  7	 I 	   436.77	   466.26	  9
  8	 G 	   465.28	   409.72	  8
  9	 D 	   522.79	   381.21	  7
 10	 A 	   558.31	   323.70	  6
 11	 S 	   601.83	   288.18	  5
 12	 S 	   645.34	   244.66	  4
 13	 L 	   701.88	   201.15	  3
 14	 I 	   758.43	   144.61	  2
 15	 R 	        -  	    88.06	  1  _
```

**90. IPI00000854 Lutropin subunit beta**

| Sequence | Charge | M+H+ | XCorr | DeltaCn |
| --- | --- | --- | --- | --- |
| R.GVDPVVSFPVALSCR.C | 2 | 1603.838 | 3.378 | 0.47 |

+1 charge

```
		      B   	       Y
  1	 G 	    58.03	        -  	 15
  2	 V 	   157.10	  1545.94	 14
  3	 D 	   272.12	  1446.88	 13
  4	 P 	   369.18	  1331.85	 12
  5	 V 	   468.25	  1234.80	 11
  6	 V 	   567.31	  1135.73	 10
  7	 S 	   654.35	  1036.66	  9
  8	 F 	   801.41	   949.63	  8
  9	 P 	   898.47	   802.56	  7
 10	 V 	   997.54	   705.51	  6
 11	 A 	  1068.57	   606.44	  5
 12	 L 	  1181.66	   535.40	  4
 13	 S 	  1268.69	   422.32	  3
 14	 C 	  1428.85	   335.28	  2
 15	 R 	        -  	   175.12	  1  _
+2 charge
		      B   	       Y
  1	 G 	    29.52	        -  	 15
  2	 V 	    79.05	   773.48	 14
  3	 D 	   136.57	   723.94	 13
  4	 P 	   185.09	   666.43	 12
  5	 V 	   234.63	   617.90	 11
  6	 V 	   284.16	   568.37	 10
  7	 S 	   327.68	   518.83	  9
  8	 F 	   401.21	   475.32	  8
  9	 P 	   449.74	   401.78	  7
 10	 V 	   499.27	   353.26	  6
 11	 A 	   534.79	   303.72	  5
 12	 L 	   591.33	   268.20	  4
 13	 S 	   634.85	   211.66	  3
 14	 C 	   714.93	   168.15	  2
 15	 R 	        -  	    88.06	  1  _
```

**91. IPI00003865 Isoform 1 of Heat shock cognate 71 kDa protein**

| Sequence | Charge | M+H+ | XCorr | DeltaCn |
| --- | --- | --- | --- | --- |
| K.SFYPEEVSSMVLTK.M | 2 | 1617.845 | 2.838 | 0.478 |

+1 charge

```
		      B   	       Y
  1	 S 	    88.04	        -  	 14
  2	 F 	   235.11	  1529.76	 13
  3	 Y 	   398.17	  1382.69	 12
  4	 P 	   495.22	  1219.62	 11
  5	 E 	   624.27	  1122.57	 10
  6	 E 	   753.31	   993.53	  9
  7	 V 	   852.38	   864.49	  8
  8	 S 	   939.41	   765.42	  7
  9	 S 	  1026.44	   678.39	  6
 10	 M 	  1157.48	   591.35	  5
 11	 V 	  1256.55	   460.31	  4
 12	 L 	  1369.63	   361.24	  3
 13	 T 	  1470.68	   248.16	  2
 14	 K 	        -  	   147.11	  1  _
+2 charge
		      B   	       Y
  1	 S 	    44.52	        -  	 14
  2	 F 	   118.06	   765.38	 13
  3	 Y 	   199.59	   691.85	 12
  4	 P 	   248.12	   610.32	 11
  5	 E 	   312.64	   561.79	 10
  6	 E 	   377.16	   497.27	  9
  7	 V 	   426.69	   432.75	  8
  8	 S 	   470.21	   383.21	  7
  9	 S 	   513.72	   339.70	  6
 10	 M 	   579.24	   296.18	  5
 11	 V 	   628.78	   230.66	  4
 12	 L 	   685.32	   181.13	  3
 13	 T 	   735.84	   124.58	  2
 14	 K 	        -  	    74.06	  1  _
```

**92. IPI00019641 Pepsin A**

| Sequence | Charge | M+H+ | XCorr | DeltaCn |
| --- | --- | --- | --- | --- |
| R.QYFTVFDRANNQVGLAPVA.- | 2 | 2111.345 | 4.149 | 0.542 |

+1 charge

```
		      B   	       Y
  1	 Q 	   129.07	        -  	 19
  2	 Y 	   292.13	  1982.01	 18
  3	 F 	   439.20	  1818.95	 17
  4	 T 	   540.25	  1671.88	 16
  5	 V 	   639.31	  1570.83	 15
  6	 F 	   786.38	  1471.77	 14
  7	 D 	   901.41	  1324.70	 13
  8	 R 	  1057.51	  1209.67	 12
  9	 A 	  1128.55	  1053.57	 11
 10	 N 	  1242.59	   982.53	 10
 11	 N 	  1356.63	   868.49	  9
 12	 Q 	  1484.69	   754.45	  8
 13	 V 	  1583.76	   626.39	  7
 14	 G 	  1640.78	   527.32	  6
 15	 L 	  1753.87	   470.30	  5
 16	 A 	  1824.90	   357.21	  4
 17	 P 	  1921.96	   286.18	  3
 18	 V 	  2021.02	   189.12	  2
 19	 A 	        -  	    90.05	  1  _
+2 charge
		      B   	       Y
  1	 Q 	    65.04	        -  	 19
  2	 Y 	   146.57	   991.51	 18
  3	 F 	   220.10	   909.98	 17
  4	 T 	   270.63	   836.44	 16
  5	 V 	   320.16	   785.92	 15
  6	 F 	   393.69	   736.39	 14
  7	 D 	   451.21	   662.85	 13
  8	 R 	   529.26	   605.34	 12
  9	 A 	   564.78	   527.29	 11
 10	 N 	   621.80	   491.77	 10
 11	 N 	   678.82	   434.75	  9
 12	 Q 	   742.85	   377.73	  8
 13	 V 	   792.38	   313.70	  7
 14	 G 	   820.89	   264.16	  6
 15	 L 	   877.44	   235.65	  5
 16	 A 	   912.96	   179.11	  4
 17	 P 	   961.48	   143.59	  3
 18	 V 	  1011.02	    95.07	  2
 19	 A 	        -  	    45.53	  1  _
```

**93. IPI00409566 Isoform 1 of Polycystin-1**

| Sequence | Charge | M+H+ | XCorr | DeltaCn |
| --- | --- | --- | --- | --- |
| R.TLGPALRIPADATALDVSHNLLR.A | 3 | 2415.777 | 4.766 | 0.497 |

+1 charge

```
		      B   	       Y
  1	 T 	   102.05	        -  	 23
  2	 L 	   215.14	  2313.30	 22
  3	 G 	   272.16	  2200.22	 21
  4	 P 	   369.21	  2143.20	 20
  5	 A 	   440.25	  2046.15	 19
  6	 L 	   553.33	  1975.11	 18
  7	 R 	   709.44	  1862.02	 17
  8	 I 	   822.52	  1705.92	 16
  9	 P 	   919.57	  1592.84	 15
 10	 A 	   990.61	  1495.79	 14
 11	 D 	  1105.64	  1424.75	 13
 12	 A 	  1176.67	  1309.72	 12
 13	 T 	  1277.72	  1238.69	 11
 14	 A 	  1348.76	  1137.64	 10
 15	 L 	  1461.84	  1066.60	  9
 16	 D 	  1576.87	   953.52	  8
 17	 V 	  1675.94	   838.49	  7
 18	 S 	  1762.97	   739.42	  6
 19	 H 	  1900.03	   652.39	  5
 20	 N 	  2014.07	   515.33	  4
 21	 L 	  2127.16	   401.29	  3
 22	 L 	  2240.24	   288.20	  2
 23	 R 	        -  	   175.12	  1  _


+2 charge
		      B   	       Y
  1	 T 	    51.53	        -  	 23
  2	 L 	   108.07	  1157.16	 22
  3	 G 	   136.58	  1100.61	 21
  4	 P 	   185.11	  1072.10	 20
  5	 A 	   220.63	  1023.58	 19
  6	 L 	   277.17	   988.06	 18
  7	 R 	   355.22	   931.52	 17
  8	 I 	   411.76	   853.47	 16
  9	 P 	   460.29	   796.92	 15
 10	 A 	   495.81	   748.40	 14
 11	 D 	   553.32	   712.88	 13
 12	 A 	   588.84	   655.36	 12
 13	 T 	   639.36	   619.85	 11
 14	 A 	   674.88	   569.32	 10
 15	 L 	   731.42	   533.80	  9
 16	 D 	   788.94	   477.26	  8
 17	 V 	   838.47	   419.75	  7
 18	 S 	   881.99	   370.21	  6
 19	 H 	   950.52	   326.70	  5
 20	 N 	  1007.54	   258.17	  4
 21	 L 	  1064.08	   201.15	  3
 22	 L 	  1120.62	   144.61	  2
 23	 R 	        -  	    88.06	  1  _


+3 charge
		      B   	       Y
  1	 T 	    34.69	        -  	 23
  2	 L 	    72.38	   771.77	 22
  3	 G 	    91.39	   734.08	 21
  4	 P 	   123.74	   715.07	 20
  5	 A 	   147.42	   682.72	 19
  6	 L 	   185.12	   659.04	 18
  7	 R 	   237.15	   621.35	 17
  8	 I 	   274.84	   569.31	 16
  9	 P 	   307.20	   531.62	 15
 10	 A 	   330.87	   499.27	 14
 11	 D 	   369.22	   475.59	 13
 12	 A 	   392.90	   437.25	 12
 13	 T 	   426.58	   413.57	 11
 14	 A 	   450.26	   379.88	 10
 15	 L 	   487.95	   356.20	  9
 16	 D 	   526.29	   318.51	  8
 17	 V 	   559.32	   280.17	  7
 18	 S 	   588.33	   247.15	  6
 19	 H 	   634.01	   218.13	  5
 20	 N 	   672.03	   172.45	  4
 21	 L 	   709.72	   134.43	  3
 22	 L 	   747.42	    96.74	  2
 23	 R 	        -  	    59.04	  1  _
```

**94. IPI00025846 Isoform 2A of Desmocollin-2**

| Sequence | Charge | M+H+ | XCorr | DeltaCn |
| --- | --- | --- | --- | --- |
| R.LSYQNDPPFGSYVVPITVR.D | 2 | 2153.422 | 4.187 | 0.529 |

+1 charge

```
		      B   	       Y
  1	 L 	   114.09	        -  	 19
  2	 S 	   201.12	  2039.02	 18
  3	 Y 	   364.19	  1951.99	 17
  4	 Q 	   492.25	  1788.93	 16
  5	 N 	   606.29	  1660.87	 15
  6	 D 	   721.32	  1546.83	 14
  7	 P 	   818.37	  1431.80	 13
  8	 P 	   915.42	  1334.75	 12
  9	 F 	  1062.49	  1237.69	 11
 10	 G 	  1119.51	  1090.63	 10
 11	 S 	  1206.54	  1033.60	  9
 12	 Y 	  1369.61	   946.57	  8
 13	 V 	  1468.67	   783.51	  7
 14	 V 	  1567.74	   684.44	  6
 15	 P 	  1664.80	   585.37	  5
 16	 I 	  1777.88	   488.32	  4
 17	 T 	  1878.93	   375.24	  3
 18	 V 	  1978.00	   274.19	  2
 19	 R 	        -  	   175.12	  1  _
+2 charge
		      B   	       Y
  1	 L 	    57.55	        -  	 19
  2	 S 	   101.07	  1020.02	 18
  3	 Y 	   182.60	   976.50	 17
  4	 Q 	   246.63	   894.97	 16
  5	 N 	   303.65	   830.94	 15
  6	 D 	   361.16	   773.92	 14
  7	 P 	   409.69	   716.40	 13
  8	 P 	   458.21	   667.88	 12
  9	 F 	   531.75	   619.35	 11
 10	 G 	   560.26	   545.82	 10
 11	 S 	   603.77	   517.31	  9
 12	 Y 	   685.31	   473.79	  8
 13	 V 	   734.84	   392.26	  7
 14	 V 	   784.38	   342.72	  6
 15	 P 	   832.90	   293.19	  5
 16	 I 	   889.44	   244.66	  4
 17	 T 	   939.97	   188.12	  3
 18	 V 	   989.50	   137.60	  2
 19	 R 	        -  	    88.06	  1  _
```

**95. IPI00021263 14-3-3 protein zeta/delta**

| Sequence | Charge | M+H+ | XCorr | DeltaCn |
| --- | --- | --- | --- | --- |
| K.SVTEQGAELSNEERNLLSVAYKNVVGAR.R | 3 | 3035.316 | 4.377 | 0.482 |

+1 charge

```
		      B   	       Y
  1	 S 	    88.04	        -  	 28
  2	 V 	   187.11	  2946.53	 27
  3	 T 	   288.16	  2847.46	 26
  4	 E 	   417.20	  2746.41	 25
  5	 Q 	   545.26	  2617.37	 24
  6	 G 	   602.28	  2489.31	 23
  7	 A 	   673.32	  2432.29	 22
  8	 E 	   802.36	  2361.25	 21
  9	 L 	   915.44	  2232.21	 20
 10	 S 	  1002.47	  2119.13	 19
 11	 N 	  1116.52	  2032.09	 18
 12	 E 	  1245.56	  1918.05	 17
 13	 E 	  1374.60	  1789.01	 16
 14	 R 	  1530.70	  1659.97	 15
 15	 N 	  1644.75	  1503.86	 14
 16	 L 	  1757.83	  1389.82	 13
 17	 L 	  1870.91	  1276.74	 12
 18	 S 	  1957.95	  1163.65	 11
 19	 V 	  2057.01	  1076.62	 10
 20	 A 	  2128.05	   977.55	  9
 21	 Y 	  2291.11	   906.52	  8
 22	 K 	  2419.21	   743.45	  7
 23	 N 	  2533.25	   615.36	  6
 24	 V 	  2632.32	   501.31	  5
 25	 V 	  2731.39	   402.25	  4
 26	 G 	  2788.41	   303.18	  3
 27	 A 	  2859.45	   246.16	  2
 28	 R 	        -  	   175.12	  1  _


+2 charge
		      B   	       Y
  1	 S 	    44.52	        -  	 28
  2	 V 	    94.06	  1473.77	 27
  3	 T 	   144.58	  1424.23	 26
  4	 E 	   209.10	  1373.71	 25
  5	 Q 	   273.13	  1309.19	 24
  6	 G 	   301.64	  1245.16	 23
  7	 A 	   337.16	  1216.65	 22
  8	 E 	   401.68	  1181.13	 21
  9	 L 	   458.22	  1116.61	 20
 10	 S 	   501.74	  1060.07	 19
 11	 N 	   558.76	  1016.55	 18
 12	 E 	   623.28	   959.53	 17
 13	 E 	   687.80	   895.01	 16
 14	 R 	   765.86	   830.49	 15
 15	 N 	   822.88	   752.44	 14
 16	 L 	   879.42	   695.41	 13
 17	 L 	   935.96	   638.87	 12
 18	 S 	   979.48	   582.33	 11
 19	 V 	  1029.01	   538.81	 10
 20	 A 	  1064.53	   489.28	  9
 21	 Y 	  1146.06	   453.76	  8
 22	 K 	  1210.11	   372.23	  7
 23	 N 	  1267.13	   308.18	  6
 24	 V 	  1316.66	   251.16	  5
 25	 V 	  1366.20	   201.63	  4
 26	 G 	  1394.71	   152.09	  3
 27	 A 	  1430.23	   123.58	  2
 28	 R 	        -  	    88.06	  1  _


+3 charge
		      B   	       Y
  1	 S 	    30.02	        -  	 28
  2	 V 	    63.04	   982.85	 27
  3	 T 	    96.72	   949.82	 26
  4	 E 	   139.74	   916.14	 25
  5	 Q 	   182.42	   873.13	 24
  6	 G 	   201.43	   830.44	 23
  7	 A 	   225.11	   811.43	 22
  8	 E 	   268.12	   787.76	 21
  9	 L 	   305.82	   744.74	 20
 10	 S 	   334.83	   707.05	 19
 11	 N 	   372.84	   678.04	 18
 12	 E 	   415.86	   640.02	 17
 13	 E 	   458.87	   597.01	 16
 14	 R 	   510.91	   553.99	 15
 15	 N 	   548.92	   501.96	 14
 16	 L 	   586.61	   463.95	 13
 17	 L 	   624.31	   426.25	 12
 18	 S 	   653.32	   388.56	 11
 19	 V 	   686.34	   359.55	 10
 20	 A 	   710.02	   326.52	  9
 21	 Y 	   764.38	   302.84	  8
 22	 K 	   807.07	   248.49	  7
 23	 N 	   845.09	   205.79	  6
 24	 V 	   878.11	   167.78	  5
 25	 V 	   911.13	   134.75	  4
 26	 G 	   930.14	   101.73	  3
 27	 A 	   953.82	    82.72	  2
 28	 R 	        -  	    59.04	  1  _
```

**96. IPI00873231 cDNA FLJ60768, moderately similar to RNA polymerase I-specific transcriptioninitiation factor RRN3**

| Sequence | Charge | M+H+ | XCorr | DeltaCn |
| --- | --- | --- | --- | --- |
| K.NQLLDPDIKRLPWLNR.S | 3 | 1992.313 | 3.322 | 0.305 |

+1 charge

```
		      B   	       Y
  1	 N 	   115.05	        -  	 16
  2	 Q 	   243.11	  1877.08	 15
  3	 L 	   356.19	  1749.02	 14
  4	 L 	   469.28	  1635.93	 13
  5	 D 	   584.30	  1522.85	 12
  6	 P 	   681.36	  1407.82	 11
  7	 D 	   796.38	  1310.77	 10
  8	 I 	   909.47	  1195.74	  9
  9	 K 	  1037.56	  1082.66	  8
 10	 R 	  1193.66	   954.56	  7
 11	 L 	  1306.75	   798.46	  6
 12	 P 	  1403.80	   685.38	  5
 13	 W 	  1589.88	   588.33	  4
 14	 L 	  1702.96	   402.25	  3
 15	 N 	  1817.01	   289.16	  2
 16	 R 	        -  	   175.12	  1  _
+2 charge
		      B   	       Y
  1	 N 	    58.03	        -  	 16
  2	 Q 	   122.06	   939.04	 15
  3	 L 	   178.60	   875.01	 14
  4	 L 	   235.14	   818.47	 13
  5	 D 	   292.66	   761.93	 12
  6	 P 	   341.18	   704.41	 11
  7	 D 	   398.70	   655.89	 10
  8	 I 	   455.24	   598.37	  9
  9	 K 	   519.28	   541.83	  8
 10	 R 	   597.34	   477.79	  7
 11	 L 	   653.88	   399.73	  6
 12	 P 	   702.40	   343.19	  5
 13	 W 	   795.44	   294.67	  4
 14	 L 	   851.99	   201.63	  3
 15	 N 	   909.01	   145.08	  2
 16	 R 	        -  	    88.06	  1  _


+3 charge
		      B   	       Y
  1	 N 	    39.02	        -  	 16
  2	 Q 	    81.71	   626.36	 15
  3	 L 	   119.40	   583.68	 14
  4	 L 	   157.10	   545.98	 13
  5	 D 	   195.44	   508.29	 12
  6	 P 	   227.79	   469.95	 11
  7	 D 	   266.13	   437.59	 10
  8	 I 	   303.83	   399.25	  9
  9	 K 	   346.53	   361.56	  8
 10	 R 	   398.56	   318.86	  7
 11	 L 	   436.25	   266.83	  6
 12	 P 	   468.61	   229.13	  5
 13	 W 	   530.63	   196.78	  4
 14	 L 	   568.33	   134.75	  3
 15	 N 	   606.34	    97.06	  2
 16	 R 	        -  	    59.04	  1  _
```

**97. IPI00026944 Isoform 1 of Nidogen-1**

| Sequence | Charge | M+H+ | XCorr | DeltaCn |
| --- | --- | --- | --- | --- |
| K.VYYREDLSPSITQR.A | 2 | 1727.899 | 4.452 | 0.499 |

+1 charge

```
		      B   	       Y
  1	 V 	   100.08	        -  	 14
  2	 Y 	   263.14	  1627.81	 13
  3	 Y 	   426.20	  1464.74	 12
  4	 R 	   582.30	  1301.68	 11
  5	 E 	   711.35	  1145.58	 10
  6	 D 	   826.37	  1016.54	  9
  7	 L 	   939.46	   901.51	  8
  8	 S 	  1026.49	   788.43	  7
  9	 P 	  1123.54	   701.39	  6
 10	 S 	  1210.57	   604.34	  5
 11	 I 	  1323.66	   517.31	  4
 12	 T 	  1424.71	   404.23	  3
 13	 Q 	  1552.76	   303.18	  2
 14	 R 	        -  	   175.12	  1  _
+2 charge
		      B   	       Y
  1	 V 	    50.54	        -  	 14
  2	 Y 	   132.07	   814.41	 13
  3	 Y 	   213.60	   732.88	 12
  4	 R 	   291.66	   651.34	 11
  5	 E 	   356.18	   573.29	 10
  6	 D 	   413.69	   508.77	  9
  7	 L 	   470.23	   451.26	  8
  8	 S 	   513.75	   394.72	  7
  9	 P 	   562.27	   351.20	  6
 10	 S 	   605.79	   302.67	  5
 11	 I 	   662.33	   259.16	  4
 12	 T 	   712.86	   202.62	  3
 13	 Q 	   776.89	   152.09	  2
 14	 R 	        -  	    88.06	  1  _
```

**98. IPI00854644 similar to hCG1686089**

| Sequence | Charge | M+H+ | XCorr | DeltaCn |
| --- | --- | --- | --- | --- |
| R.LLIYDASSR.A | 2 | 1038.179 | 2.834 | 0.377 |

+1 charge

```
		      B   	       Y
  1	 L 	   114.09	        -  	  9
  2	 L 	   227.18	   924.48	  8
  3	 I 	   340.26	   811.39	  7
  4	 Y 	   503.32	   698.31	  6
  5	 D 	   618.35	   535.25	  5
  6	 A 	   689.39	   420.22	  4
  7	 S 	   776.42	   349.18	  3
  8	 S 	   863.45	   262.15	  2
  9	 R 	        -  	   175.12	  1  _
+2 charge
		      B   	       Y
  1	 L 	    57.55	        -  	  9
  2	 L 	   114.09	   462.74	  8
  3	 I 	   170.63	   406.20	  7
  4	 Y 	   252.17	   349.66	  6
  5	 D 	   309.68	   268.13	  5
  6	 A 	   345.20	   210.61	  4
  7	 S 	   388.71	   175.10	  3
  8	 S 	   432.23	   131.58	  2
  9	 R 	        -  	    88.06	  1  _
```

**99. IPI00009901 Nuclear transport factor 2**

| Sequence | Charge | M+H+ | XCorr | DeltaCn |
| --- | --- | --- | --- | --- |
| K.AAIVEKLSSLPFQK.I | 2 | 1531.821 | 4.448 | 0.527 |

+1 charge

```
		      B   	       Y
  1	 A 	    72.04	        -  	 14
  2	 A 	   143.08	  1459.85	 13
  3	 I 	   256.17	  1388.81	 12
  4	 V 	   355.23	  1275.73	 11
  5	 E 	   484.28	  1176.66	 10
  6	 K 	   612.37	  1047.62	  9
  7	 L 	   725.46	   919.52	  8
  8	 S 	   812.49	   806.44	  7
  9	 S 	   899.52	   719.41	  6
 10	 L 	  1012.60	   632.38	  5
 11	 P 	  1109.66	   519.29	  4
 12	 F 	  1256.72	   422.24	  3
 13	 Q 	  1384.78	   275.17	  2
 14	 K 	        -  	   147.11	  1  _
+2 charge
		      B   	       Y
  1	 A 	    36.53	        -  	 14
  2	 A 	    72.04	   730.43	 13
  3	 I 	   128.59	   694.91	 12
  4	 V 	   178.12	   638.37	 11
  5	 E 	   242.64	   588.83	 10
  6	 K 	   306.69	   524.31	  9
  7	 L 	   363.23	   460.27	  8
  8	 S 	   406.75	   403.72	  7
  9	 S 	   450.26	   360.21	  6
 10	 L 	   506.81	   316.69	  5
 11	 P 	   555.33	   260.15	  4
 12	 F 	   628.87	   211.62	  3
 13	 Q 	   692.90	   138.09	  2
 14	 K 	        -  	    74.06	  1  _
```

**100. IPI00003348 Guanine nucleotide-binding protein G(I)/G(S)/G(T) subunit beta-2**

| Sequence | Charge | M+H+ | XCorr | DeltaCn |
| --- | --- | --- | --- | --- |
| R.KACGDSTLTQITAGLDPVGR.I | 2 | 2061.276 | 3.388 | 0.426 |

+1 charge

```
		      B   	       Y
  1	 K 	   129.10	        -  	 20
  2	 A 	   200.14	  1932.08	 19
  3	 C 	   360.30	  1861.05	 18
  4	 G 	   417.33	  1700.88	 17
  5	 D 	   532.35	  1643.86	 16
  6	 S 	   619.39	  1528.83	 15
  7	 T 	   720.43	  1441.80	 14
  8	 L 	   833.52	  1340.75	 13
  9	 T 	   934.56	  1227.67	 12
 10	 Q 	  1062.62	  1126.62	 11
 11	 I 	  1175.71	   998.56	 10
 12	 T 	  1276.75	   885.48	  9
 13	 A 	  1347.79	   784.43	  8
 14	 G 	  1404.81	   713.39	  7
 15	 L 	  1517.90	   656.37	  6
 16	 D 	  1632.92	   543.29	  5
 17	 P 	  1729.98	   428.26	  4
 18	 V 	  1829.05	   331.21	  3
 19	 G 	  1886.07	   232.14	  2
 20	 R 	        -  	   175.12	  1  _


+2 charge
		      B   	       Y
  1	 K 	    65.05	        -  	 20
  2	 A 	   100.57	   966.55	 19
  3	 C 	   180.66	   931.03	 18
  4	 G 	   209.17	   850.94	 17
  5	 D 	   266.68	   822.43	 16
  6	 S 	   310.20	   764.92	 15
  7	 T 	   360.72	   721.40	 14
  8	 L 	   417.26	   670.88	 13
  9	 T 	   467.79	   614.34	 12
 10	 Q 	   531.82	   563.81	 11
 11	 I 	   588.36	   499.79	 10
 12	 T 	   638.88	   443.24	  9
 13	 A 	   674.40	   392.72	  8
 14	 G 	   702.91	   357.20	  7
 15	 L 	   759.45	   328.69	  6
 16	 D 	   816.97	   272.15	  5
 17	 P 	   865.49	   214.63	  4
 18	 V 	   915.03	   166.11	  3
 19	 G 	   943.54	   116.57	  2
 20	 R 	        -  	    88.06	  1  _
```

**101. IPI00921523 Isoform 1 of Complement factor B (Fragment)**

| Sequence | Charge | M+H+ | XCorr | DeltaCn |
| --- | --- | --- | --- | --- |
| R.LLQEGQALEYVCPSGFYPYPVQTR.T | 3 | 2817.138 | 3.588 | 0.463 |

+1 charge

```
		      B   	       Y
  1	 L 	   114.09	        -  	 24
  2	 L 	   227.18	  2702.43	 23
  3	 Q 	   355.23	  2589.34	 22
  4	 E 	   484.28	  2461.28	 21
  5	 G 	   541.30	  2332.24	 20
  6	 Q 	   669.36	  2275.22	 19
  7	 A 	   740.39	  2147.16	 18
  8	 L 	   853.48	  2076.12	 17
  9	 E 	   982.52	  1963.04	 16
 10	 Y 	  1145.58	  1834.00	 15
 11	 V 	  1244.65	  1670.93	 14
 12	 C 	  1404.82	  1571.87	 13
 13	 P 	  1501.87	  1411.70	 12
 14	 S 	  1588.90	  1314.65	 11
 15	 G 	  1645.92	  1227.62	 10
 16	 F 	  1792.99	  1170.59	  9
 17	 Y 	  1956.06	  1023.53	  8
 18	 P 	  2053.11	   860.46	  7
 19	 Y 	  2216.17	   763.41	  6
 20	 P 	  2313.22	   600.35	  5
 21	 V 	  2412.29	   503.29	  4
 22	 Q 	  2540.35	   404.23	  3
 23	 T 	  2641.40	   276.17	  2
 24	 R 	        -  	   175.12	  1  _


+2 charge
		      B   	       Y
  1	 L 	    57.55	        -  	 24
  2	 L 	   114.09	  1351.72	 23
  3	 Q 	   178.12	  1295.17	 22
  4	 E 	   242.64	  1231.15	 21
  5	 G 	   271.15	  1166.62	 20
  6	 Q 	   335.18	  1138.11	 19
  7	 A 	   370.70	  1074.08	 18
  8	 L 	   427.24	  1038.57	 17
  9	 E 	   491.76	   982.02	 16
 10	 Y 	   573.30	   917.50	 15
 11	 V 	   622.83	   835.97	 14
 12	 C 	   702.91	   786.44	 13
 13	 P 	   751.44	   706.35	 12
 14	 S 	   794.95	   657.83	 11
 15	 G 	   823.47	   614.31	 10
 16	 F 	   897.00	   585.80	  9
 17	 Y 	   978.53	   512.27	  8
 18	 P 	  1027.06	   430.73	  7
 19	 Y 	  1108.59	   382.21	  6
 20	 P 	  1157.12	   300.68	  5
 21	 V 	  1206.65	   252.15	  4
 22	 Q 	  1270.68	   202.62	  3
 23	 T 	  1321.20	   138.59	  2
 24	 R 	        -  	    88.06	  1  _


+3 charge
		      B   	       Y
  1	 L 	    38.70	        -  	 24
  2	 L 	    76.40	   901.48	 23
  3	 Q 	   119.08	   863.79	 22
  4	 E 	   162.10	   821.10	 21
  5	 G 	   181.10	   778.09	 20
  6	 Q 	   223.79	   759.08	 19
  7	 A 	   247.47	   716.39	 18
  8	 L 	   285.16	   692.71	 17
  9	 E 	   328.18	   655.02	 16
 10	 Y 	   382.53	   612.00	 15
 11	 V 	   415.56	   557.65	 14
 12	 C 	   468.94	   524.63	 13
 13	 P 	   501.29	   471.24	 12
 14	 S 	   530.31	   438.89	 11
 15	 G 	   549.31	   409.88	 10
 16	 F 	   598.34	   390.87	  9
 17	 Y 	   652.69	   341.85	  8
 18	 P 	   685.04	   287.49	  7
 19	 Y 	   739.40	   255.14	  6
 20	 P 	   771.75	   200.79	  5
 21	 V 	   804.77	   168.44	  4
 22	 Q 	   847.46	   135.41	  3
 23	 T 	   881.14	    92.73	  2
 24	 R 	        -  	    59.04	  1  _
```

**102. IPI00167710 Isoform 1 of Fibulin-7**

| Sequence | Charge | M+H+ | XCorr | DeltaCn |
| --- | --- | --- | --- | --- |
| K.TISFHYLSLPSNLKTPITLFR.M | 3 | 2449.875 | 4.416 | 0.421 |

+1 charge

```
		      B   	       Y
  1	 T 	   102.05	        -  	 21
  2	 I 	   215.14	  2347.32	 20
  3	 S 	   302.17	  2234.23	 19
  4	 F 	   449.24	  2147.20	 18
  5	 H 	   586.30	  2000.13	 17
  6	 Y 	   749.36	  1863.07	 16
  7	 L 	   862.45	  1700.01	 15
  8	 S 	   949.48	  1586.93	 14
  9	 L 	  1062.56	  1499.89	 13
 10	 P 	  1159.61	  1386.81	 12
 11	 S 	  1246.65	  1289.76	 11
 12	 N 	  1360.69	  1202.73	 10
 13	 L 	  1473.77	  1088.68	  9
 14	 K 	  1601.87	   975.60	  8
 15	 T 	  1702.92	   847.50	  7
 16	 P 	  1799.97	   746.46	  6
 17	 I 	  1913.05	   649.40	  5
 18	 T 	  2014.10	   536.32	  4
 19	 L 	  2127.18	   435.27	  3
 20	 F 	  2274.25	   322.19	  2
 21	 R 	        -  	   175.12	  1  _


+2 charge
		      B   	       Y
  1	 T 	    51.53	        -  	 21
  2	 I 	   108.07	  1174.16	 20
  3	 S 	   151.59	  1117.62	 19
  4	 F 	   225.12	  1074.10	 18
  5	 H 	   293.65	  1000.57	 17
  6	 Y 	   375.18	   932.04	 16
  7	 L 	   431.73	   850.51	 15
  8	 S 	   475.24	   793.97	 14
  9	 L 	   531.78	   750.45	 13
 10	 P 	   580.31	   693.91	 12
 11	 S 	   623.83	   645.38	 11
 12	 N 	   680.85	   601.87	 10
 13	 L 	   737.39	   544.84	  9
 14	 K 	   801.44	   488.30	  8
 15	 T 	   851.96	   424.26	  7
 16	 P 	   900.49	   373.73	  6
 17	 I 	   957.03	   325.21	  5
 18	 T 	  1007.55	   268.66	  4
 19	 L 	  1064.10	   218.14	  3
 20	 F 	  1137.63	   161.60	  2
 21	 R 	        -  	    88.06	  1  _
+3 charge
		      B   	       Y
  1	 T 	    34.69	        -  	 21
  2	 I 	    72.38	   783.11	 20
  3	 S 	   101.40	   745.42	 19
  4	 F 	   150.42	   716.41	 18
  5	 H 	   196.10	   667.38	 17
  6	 Y 	   250.46	   621.70	 16
  7	 L 	   288.15	   567.34	 15
  8	 S 	   317.16	   529.65	 14
  9	 L 	   354.86	   500.64	 13
 10	 P 	   387.21	   462.94	 12
 11	 S 	   416.22	   430.59	 11
 12	 N 	   454.23	   401.58	 10
 13	 L 	   491.93	   363.57	  9
 14	 K 	   534.63	   325.87	  8
 15	 T 	   568.31	   283.17	  7
 16	 P 	   600.66	   249.49	  6
 17	 I 	   638.36	   217.14	  5
 18	 T 	   672.04	   179.44	  4
 19	 L 	   709.73	   145.76	  3
 20	 F 	   758.76	   108.07	  2
 21	 R 	        -  	    59.04	  1  _
```

**103. IPI00027166 Metalloproteinase inhibitor 2**

| Sequence | Charge | M+H+ | XCorr | DeltaCn |
| --- | --- | --- | --- | --- |
| K.AVSEKEVDSGNDIYGNPIKR.I | 3 | 2192.372 | 4.271 | 0.342 |

+1 charge

```
		      B   	       Y
  1	 A 	    72.04	        -  	 20
  2	 V 	   171.11	  2120.06	 19
  3	 S 	   258.14	  2020.99	 18
  4	 E 	   387.19	  1933.96	 17
  5	 K 	   515.28	  1804.92	 16
  6	 E 	   644.32	  1676.82	 15
  7	 V 	   743.39	  1547.78	 14
  8	 D 	   858.42	  1448.71	 13
  9	 S 	   945.45	  1333.69	 12
 10	 G 	  1002.47	  1246.65	 11
 11	 N 	  1116.52	  1189.63	 10
 12	 D 	  1231.54	  1075.59	  9
 13	 I 	  1344.63	   960.56	  8
 14	 Y 	  1507.69	   847.48	  7
 15	 G 	  1564.71	   684.42	  6
 16	 N 	  1678.76	   627.39	  5
 17	 P 	  1775.81	   513.35	  4
 18	 I 	  1888.89	   416.30	  3
 19	 K 	  2016.99	   303.21	  2
 20	 R 	        -  	   175.12	  1  _


+2 charge
		      B   	       Y
  1	 A 	    36.53	        -  	 20
  2	 V 	    86.06	  1060.53	 19
  3	 S 	   129.58	  1011.00	 18
  4	 E 	   194.10	   967.48	 17
  5	 K 	   258.14	   902.96	 16
  6	 E 	   322.67	   838.92	 15
  7	 V 	   372.20	   774.39	 14
  8	 D 	   429.71	   724.86	 13
  9	 S 	   473.23	   667.35	 12
 10	 G 	   501.74	   623.83	 11
 11	 N 	   558.76	   595.32	 10
 12	 D 	   616.28	   538.30	  9
 13	 I 	   672.82	   480.78	  8
 14	 Y 	   754.35	   424.24	  7
 15	 G 	   782.86	   342.71	  6
 16	 N 	   839.88	   314.20	  5
 17	 P 	   888.41	   257.18	  4
 18	 I 	   944.95	   208.65	  3
 19	 K 	  1009.00	   152.11	  2
 20	 R 	        -  	    88.06	  1  _
+3 charge
		      B   	       Y
  1	 A 	    24.69	        -  	 20
  2	 V 	    57.71	   707.36	 19
  3	 S 	    86.72	   674.34	 18
  4	 E 	   129.73	   645.33	 17
  5	 K 	   172.43	   602.31	 16
  6	 E 	   215.45	   559.61	 15
  7	 V 	   248.47	   516.60	 14
  8	 D 	   286.81	   483.58	 13
  9	 S 	   315.82	   445.23	 12
 10	 G 	   334.83	   416.22	 11
 11	 N 	   372.84	   397.22	 10
 12	 D 	   411.19	   359.20	  9
 13	 I 	   448.88	   320.86	  8
 14	 Y 	   503.24	   283.16	  7
 15	 G 	   522.24	   228.81	  6
 16	 N 	   560.26	   209.80	  5
 17	 P 	   592.61	   171.79	  4
 18	 I 	   630.30	   139.44	  3
 19	 K 	   673.00	   101.74	  2
 20	 R 	        -  	    59.04	  1  _
```

**104. IPI00296713 Isoform 1 of Granulins**

| Sequence | Charge | M+H+ | XCorr | DeltaCn |
| --- | --- | --- | --- | --- |
| K.CDMEVSCPDGYTCCR.L | 2 | 1911.012 | 4.5 | 0.497 |

+1 charge

```
		      B   	       Y
  1	 C 	   161.17	        -  	 15
  2	 D 	   276.20	  1750.04	 14
  3	 M 	   407.24	  1635.01	 13
  4	 E 	   536.28	  1503.97	 12
  5	 V 	   635.35	  1374.93	 11
  6	 S 	   722.38	  1275.86	 10
  7	 C 	   882.55	  1188.83	  9
  8	 P 	   979.60	  1028.66	  8
  9	 D 	  1094.63	   931.61	  7
 10	 G 	  1151.65	   816.58	  6
 11	 Y 	  1314.71	   759.56	  5
 12	 T 	  1415.76	   596.50	  4
 13	 C 	  1575.93	   495.45	  3
 14	 C 	  1736.09	   335.28	  2
 15	 R 	        -  	   175.12	  1  _
+2 charge
		      B   	       Y
  1	 C 	    81.09	        -  	 15
  2	 D 	   138.60	   875.52	 14
  3	 M 	   204.12	   818.01	 13
  4	 E 	   268.64	   752.49	 12
  5	 V 	   318.18	   687.97	 11
  6	 S 	   361.70	   638.43	 10
  7	 C 	   441.78	   594.92	  9
  8	 P 	   490.30	   514.83	  8
  9	 D 	   547.82	   466.31	  7
 10	 G 	   576.33	   408.79	  6
 11	 Y 	   657.86	   380.28	  5
 12	 T 	   708.38	   298.75	  4
 13	 C 	   788.47	   248.23	  3
 14	 C 	   868.55	   168.15	  2
 15	 R 	        -  	    88.06	  1  _
```

**105. IPI00441498 Folate receptor alpha**

| Sequence | Charge | M+H+ | XCorr | DeltaCn |
| --- | --- | --- | --- | --- |
| R.FNWNHCGEMAPACKR.H | 3 | 1879.079 | 3.894 | 0.395 |

+1 charge

```
		      B   	       Y
  1	 F 	   148.08	        -  	 15
  2	 N 	   262.12	  1731.00	 14
  3	 W 	   448.20	  1616.96	 13
  4	 N 	   562.24	  1430.88	 12
  5	 H 	   699.30	  1316.83	 11
  6	 C 	   859.47	  1179.78	 10
  7	 G 	   916.49	  1019.61	  9
  8	 E 	  1045.53	   962.59	  8
  9	 M 	  1176.57	   833.55	  7
 10	 A 	  1247.61	   702.51	  6
 11	 P 	  1344.66	   631.47	  5
 12	 A 	  1415.70	   534.42	  4
 13	 C 	  1575.86	   463.38	  3
 14	 K 	  1703.96	   303.21	  2
 15	 R 	        -  	   175.12	  1  _
+2 charge
		      B   	       Y
  1	 F 	    74.54	        -  	 15
  2	 N 	   131.56	   866.00	 14
  3	 W 	   224.60	   808.98	 13
  4	 N 	   281.62	   715.94	 12
  5	 H 	   350.15	   658.92	 11
  6	 C 	   430.24	   590.39	 10
  7	 G 	   458.75	   510.31	  9
  8	 E 	   523.27	   481.80	  8
  9	 M 	   588.79	   417.28	  7
 10	 A 	   624.31	   351.76	  6
 11	 P 	   672.83	   316.24	  5
 12	 A 	   708.35	   267.71	  4
 13	 C 	   788.43	   232.19	  3
 14	 K 	   852.48	   152.11	  2
 15	 R 	        -  	    88.06	  1  _
+3 charge
		      B   	       Y
  1	 F 	    50.03	        -  	 15
  2	 N 	    88.04	   577.67	 14
  3	 W 	   150.07	   539.66	 13
  4	 N 	   188.09	   477.63	 12
  5	 H 	   233.77	   439.62	 11
  6	 C 	   287.16	   393.93	 10
  7	 G 	   306.17	   340.54	  9
  8	 E 	   349.18	   321.53	  8
  9	 M 	   392.86	   278.52	  7
 10	 A 	   416.54	   234.84	  6
 11	 P 	   448.89	   211.16	  5
 12	 A 	   472.57	   178.81	  4
 13	 C 	   525.96	   155.13	  3
 14	 K 	   568.66	   101.74	  2
 15	 R 	        -  	    59.04	  1  _
```

**106. IPI00900332 AIG2-like domain-containing protein 1**

| Sequence | Charge | M+H+ | XCorr | DeltaCn |
| --- | --- | --- | --- | --- |
| R.LVEGEVYAVDER.M | 2 | 1379.497 | 3.626 | 0.426 |

+1 charge

```
		      B   	       Y
  1	 L 	   114.09	        -  	 12
  2	 V 	   213.16	  1265.60	 11
  3	 E 	   342.20	  1166.53	 10
  4	 G 	   399.22	  1037.49	  9
  5	 E 	   528.27	   980.47	  8
  6	 V 	   627.33	   851.43	  7
  7	 Y 	   790.40	   752.36	  6
  8	 A 	   861.44	   589.29	  5
  9	 V 	   960.50	   518.26	  4
 10	 D 	  1075.53	   419.19	  3
 11	 E 	  1204.57	   304.16	  2
 12	 R 	        -  	   175.12	  1  _
+2 charge
		      B   	       Y
  1	 L 	    57.55	        -  	 12
  2	 V 	   107.08	   633.30	 11
  3	 E 	   171.60	   583.77	 10
  4	 G 	   200.12	   519.25	  9
  5	 E 	   264.64	   490.74	  8
  6	 V 	   314.17	   426.22	  7
  7	 Y 	   395.70	   376.68	  6
  8	 A 	   431.22	   295.15	  5
  9	 V 	   480.76	   259.63	  4
 10	 D 	   538.27	   210.10	  3
 11	 E 	   602.79	   152.58	  2
 12	 R 	        -  	    88.06	  1  _
```

**107. IPI00012540 Prominin-1**

| Sequence | Charge | M+H+ | XCorr | DeltaCn |
| --- | --- | --- | --- | --- |
| R.LSLSQLNSNPELR.Q | 2 | 1471.641 | 2.833 | 0.391 |

+1 charge

```
		      B   	       Y
  1	 L 	   114.09	        -  	 13
  2	 S 	   201.12	  1357.71	 12
  3	 L 	   314.21	  1270.67	 11
  4	 S 	   401.24	  1157.59	 10
  5	 Q 	   529.30	  1070.56	  9
  6	 L 	   642.38	   942.50	  8
  7	 N 	   756.43	   829.42	  7
  8	 S 	   843.46	   715.37	  6
  9	 N 	   957.50	   628.34	  5
 10	 P 	  1054.55	   514.30	  4
 11	 E 	  1183.60	   417.25	  3
 12	 L 	  1296.68	   288.20	  2
 13	 R 	        -  	   175.12	  1  _
+2 charge
		      B   	       Y
  1	 L 	    57.55	        -  	 13
  2	 S 	   101.07	   679.36	 12
  3	 L 	   157.61	   635.84	 11
  4	 S 	   201.12	   579.30	 10
  5	 Q 	   265.15	   535.78	  9
  6	 L 	   321.69	   471.75	  8
  7	 N 	   378.72	   415.21	  7
  8	 S 	   422.23	   358.19	  6
  9	 N 	   479.25	   314.67	  5
 10	 P 	   527.78	   257.65	  4
 11	 E 	   592.30	   209.13	  3
 12	 L 	   648.84	   144.61	  2
 13	 R 	        -  	    88.06	  1  _
```

**108. IPI00007221 Plasma serine protease inhibitor**

| Sequence | Charge | M+H+ | XCorr | DeltaCn |
| --- | --- | --- | --- | --- |
| R.AAAATGTIFTFR.S | 2 | 1227.394 | 3.362 | 0.461 |

+1 charge

```
		      B   	       Y
  1	 A 	    72.04	        -  	 12
  2	 A 	   143.08	  1155.62	 11
  3	 A 	   214.12	  1084.58	 10
  4	 A 	   285.16	  1013.54	  9
  5	 T 	   386.20	   942.50	  8
  6	 G 	   443.22	   841.46	  7
  7	 T 	   544.27	   784.44	  6
  8	 I 	   657.36	   683.39	  5
  9	 F 	   804.43	   570.30	  4
 10	 T 	   905.47	   423.24	  3
 11	 F 	  1052.54	   322.19	  2
 12	 R 	        -  	   175.12	  1  _
+2 charge
		      B   	       Y
  1	 A 	    36.53	        -  	 12
  2	 A 	    72.04	   578.31	 11
  3	 A 	   107.56	   542.79	 10
  4	 A 	   143.08	   507.27	  9
  5	 T 	   193.61	   471.76	  8
  6	 G 	   222.12	   421.23	  7
  7	 T 	   272.64	   392.72	  6
  8	 I 	   329.18	   342.20	  5
  9	 F 	   402.72	   285.66	  4
 10	 T 	   453.24	   212.12	  3
 11	 F 	   526.77	   161.60	  2
 12	 R 	        -  	    88.06	  1  _
```

**109. IPI00942979 Transketolase**

| Sequence | Charge | M+H+ | XCorr | DeltaCn |
| --- | --- | --- | --- | --- |
| K.MFGIDRDAIAQAVR.G | 2 | 1563.807 | 2.855 | 0.265 |

+1 charge

```
		      B   	       Y
  1	 M 	   132.05	        -  	 14
  2	 F 	   279.12	  1431.77	 13
  3	 G 	   336.14	  1284.70	 12
  4	 I 	   449.22	  1227.68	 11
  5	 D 	   564.25	  1114.60	 10
  6	 R 	   720.35	   999.57	  9
  7	 D 	   835.38	   843.47	  8
  8	 A 	   906.41	   728.44	  7
  9	 I 	  1019.50	   657.40	  6
 10	 A 	  1090.53	   544.32	  5
 11	 Q 	  1218.59	   473.28	  4
 12	 A 	  1289.63	   345.22	  3
 13	 V 	  1388.70	   274.19	  2
 14	 R 	        -  	   175.12	  1  _
+2 charge
		      B   	       Y
  1	 M 	    66.53	        -  	 14
  2	 F 	   140.06	   716.39	 13
  3	 G 	   168.57	   642.85	 12
  4	 I 	   225.11	   614.34	 11
  5	 D 	   282.63	   557.80	 10
  6	 R 	   360.68	   500.29	  9
  7	 D 	   418.19	   422.24	  8
  8	 A 	   453.71	   364.72	  7
  9	 I 	   510.25	   329.21	  6
 10	 A 	   545.77	   272.66	  5
 11	 Q 	   609.80	   237.15	  4
 12	 A 	   645.32	   173.12	  3
 13	 V 	   694.85	   137.60	  2
 14	 R 	        -  	    88.06	  1  _
```

**110. IPI00152453 HCG2042771**

| Sequence | Charge | M+H+ | XCorr | DeltaCn |
| --- | --- | --- | --- | --- |
| R.AILVDLEPGTMDSVR.S | 2 | 1616.862 | 2.917 | 0.229 |

+1 charge

```
		      B   	       Y
  1	 A 	    72.04	        -  	 15
  2	 I 	   185.13	  1544.80	 14
  3	 L 	   298.21	  1431.71	 13
  4	 V 	   397.28	  1318.63	 12
  5	 D 	   512.31	  1219.56	 11
  6	 L 	   625.39	  1104.54	 10
  7	 E 	   754.43	   991.45	  9
  8	 P 	   851.49	   862.41	  8
  9	 G 	   908.51	   765.36	  7
 10	 T 	  1009.56	   708.33	  6
 11	 M 	  1140.60	   607.29	  5
 12	 D 	  1255.62	   476.25	  4
 13	 S 	  1342.66	   361.22	  3
 14	 V 	  1441.72	   274.19	  2
 15	 R 	        -  	   175.12	  1  _
+2 charge
		      B   	       Y
  1	 A 	    36.53	        -  	 15
  2	 I 	    93.07	   772.90	 14
  3	 L 	   149.61	   716.36	 13
  4	 V 	   199.14	   659.82	 12
  5	 D 	   256.66	   610.28	 11
  6	 L 	   313.20	   552.77	 10
  7	 E 	   377.72	   496.23	  9
  8	 P 	   426.25	   431.71	  8
  9	 G 	   454.76	   383.18	  7
 10	 T 	   505.28	   354.67	  6
 11	 M 	   570.80	   304.15	  5
 12	 D 	   628.32	   238.63	  4
 13	 S 	   671.83	   181.11	  3
 14	 V 	   721.37	   137.60	  2
 15	 R 	        -  	    88.06	  1  _
```

**111. IPI00022432 Transthyretin**

| Sequence | Charge | M+H+ | XCorr | DeltaCn |
| --- | --- | --- | --- | --- |
| K.VLDAVRGSPAINVAVHVFR.K | 3 | 2021.355 | 5.807 | 0.484 |

+1 charge

```
		      B   	       Y
  1	 V 	   100.08	        -  	 19
  2	 L 	   213.16	  1921.08	 18
  3	 D 	   328.19	  1807.99	 17
  4	 A 	   399.22	  1692.97	 16
  5	 V 	   498.29	  1621.93	 15
  6	 R 	   654.39	  1522.86	 14
  7	 G 	   711.41	  1366.76	 13
  8	 S 	   798.45	  1309.74	 12
  9	 P 	   895.50	  1222.71	 11
 10	 A 	   966.54	  1125.65	 10
 11	 I 	  1079.62	  1054.62	  9
 12	 N 	  1193.66	   941.53	  8
 13	 V 	  1292.73	   827.49	  7
 14	 A 	  1363.77	   728.42	  6
 15	 V 	  1462.84	   657.38	  5
 16	 H 	  1599.90	   558.31	  4
 17	 V 	  1698.96	   421.26	  3
 18	 F 	  1846.03	   322.19	  2
 19	 R 	        -  	   175.12	  1  _
+2 charge
		      B   	       Y
  1	 V 	    50.54	        -  	 19
  2	 L 	   107.08	   961.04	 18
  3	 D 	   164.60	   904.50	 17
  4	 A 	   200.12	   846.99	 16
  5	 V 	   249.65	   811.47	 15
  6	 R 	   327.70	   761.93	 14
  7	 G 	   356.21	   683.88	 13
  8	 S 	   399.73	   655.37	 12
  9	 P 	   448.25	   611.86	 11
 10	 A 	   483.77	   563.33	 10
 11	 I 	   540.31	   527.81	  9
 12	 N 	   597.34	   471.27	  8
 13	 V 	   646.87	   414.25	  7
 14	 A 	   682.39	   364.71	  6
 15	 V 	   731.92	   329.20	  5
 16	 H 	   800.45	   279.66	  4
 17	 V 	   849.99	   211.13	  3
 18	 F 	   923.52	   161.60	  2
 19	 R 	        -  	    88.06	  1  _


+3 charge
		      B   	       Y
  1	 V 	    34.03	        -  	 19
  2	 L 	    71.72	   641.03	 18
  3	 D 	   110.07	   603.34	 17
  4	 A 	   133.75	   564.99	 16
  5	 V 	   166.77	   541.31	 15
  6	 R 	   218.80	   508.29	 14
  7	 G 	   237.81	   456.26	 13
  8	 S 	   266.82	   437.25	 12
  9	 P 	   299.17	   408.24	 11
 10	 A 	   322.85	   375.89	 10
 11	 I 	   360.55	   352.21	  9
 12	 N 	   398.56	   314.52	  8
 13	 V 	   431.58	   276.50	  7
 14	 A 	   455.26	   243.48	  6
 15	 V 	   488.28	   219.80	  5
 16	 H 	   533.97	   186.78	  4
 17	 V 	   566.99	   141.09	  3
 18	 F 	   616.02	   108.07	  2
 19	 R 	        -  	    59.04	  1  _
```

**112. IPI00166392 Similar to Cell adhesion molecule 1 precursor**

| Sequence | Charge | M+H+ | XCorr | DeltaCn |
| --- | --- | --- | --- | --- |
| R.YLEVQYKPQVHIQMTYPLQGLTR.E | 3 | 2807.262 | 4.235 | 0.504 |

+1 charge

```
		      B   	       Y
  1	 Y 	   164.07	        -  	 23
  2	 L 	   277.15	  2642.41	 22
  3	 E 	   406.20	  2529.33	 21
  4	 V 	   505.27	  2400.29	 20
  5	 Q 	   633.32	  2301.22	 19
  6	 Y 	   796.39	  2173.16	 18
  7	 K 	   924.48	  2010.10	 17
  8	 P 	  1021.54	  1882.00	 16
  9	 Q 	  1149.59	  1784.95	 15
 10	 V 	  1248.66	  1656.89	 14
 11	 H 	  1385.72	  1557.82	 13
 12	 I 	  1498.81	  1420.76	 12
 13	 Q 	  1626.86	  1307.68	 11
 14	 M 	  1757.90	  1179.62	 10
 15	 T 	  1858.95	  1048.58	  9
 16	 Y 	  2022.02	   947.53	  8
 17	 P 	  2119.07	   784.47	  7
 18	 L 	  2232.15	   687.41	  6
 19	 Q 	  2360.21	   574.33	  5
 20	 G 	  2417.23	   446.27	  4
 21	 L 	  2530.32	   389.25	  3
 22	 T 	  2631.36	   276.17	  2
 23	 R 	        -  	   175.12	  1  _


+2 charge
		      B   	       Y
  1	 Y 	    82.54	        -  	 23
  2	 L 	   139.08	  1321.71	 22
  3	 E 	   203.60	  1265.17	 21
  4	 V 	   253.14	  1200.65	 20
  5	 Q 	   317.17	  1151.11	 19
  6	 Y 	   398.70	  1087.08	 18
  7	 K 	   462.74	  1005.55	 17
  8	 P 	   511.27	   941.50	 16
  9	 Q 	   575.30	   892.98	 15
 10	 V 	   624.83	   828.95	 14
 11	 H 	   693.36	   779.41	 13
 12	 I 	   749.91	   710.88	 12
 13	 Q 	   813.94	   654.34	 11
 14	 M 	   879.46	   590.31	 10
 15	 T 	   929.98	   524.79	  9
 16	 Y 	  1011.51	   474.27	  8
 17	 P 	  1060.04	   392.74	  7
 18	 L 	  1116.58	   344.21	  6
 19	 Q 	  1180.61	   287.67	  5
 20	 G 	  1209.12	   223.64	  4
 21	 L 	  1265.66	   195.13	  3
 22	 T 	  1316.19	   138.59	  2
 23	 R 	        -  	    88.06	  1  _


+3 charge
		      B   	       Y
  1	 Y 	    55.36	        -  	 23
  2	 L 	    93.06	   881.48	 22
  3	 E 	   136.07	   843.78	 21
  4	 V 	   169.09	   800.77	 20
  5	 Q 	   211.78	   767.74	 19
  6	 Y 	   266.13	   725.06	 18
  7	 K 	   308.83	   670.70	 17
  8	 P 	   341.18	   628.00	 16
  9	 Q 	   383.87	   595.65	 15
 10	 V 	   416.89	   552.97	 14
 11	 H 	   462.58	   519.95	 13
 12	 I 	   500.27	   474.26	 12
 13	 Q 	   542.96	   436.56	 11
 14	 M 	   586.64	   393.88	 10
 15	 T 	   620.32	   350.20	  9
 16	 Y 	   674.68	   316.52	  8
 17	 P 	   707.03	   262.16	  7
 18	 L 	   744.72	   229.81	  6
 19	 Q 	   787.41	   192.12	  5
 20	 G 	   806.42	   149.43	  4
 21	 L 	   844.11	   130.42	  3
 22	 T 	   877.79	    92.73	  2
 23	 R 	        -  	    59.04	  1  _
```

**113. IPI00022620 Secreted Ly-6/uPAR-related protein 1**

| Sequence | Charge | M+H+ | XCorr | DeltaCn |
| --- | --- | --- | --- | --- |
| R.SCSSSCVATDPDSIGAAHLIFCCFR.D | 3 | 2820.036 | 4.908 | 0.596 |

+1 charge

```
		      B   	       Y
  1	 S 	    88.04	        -  	 25
  2	 C 	   248.20	  2731.71	 24
  3	 S 	   335.24	  2571.55	 23
  4	 S 	   422.27	  2484.51	 22
  5	 S 	   509.30	  2397.48	 21
  6	 C 	   669.47	  2310.45	 20
  7	 V 	   768.53	  2150.28	 19
  8	 A 	   839.57	  2051.22	 18
  9	 T 	   940.62	  1980.18	 17
 10	 D 	  1055.65	  1879.13	 16
 11	 P 	  1152.70	  1764.10	 15
 12	 D 	  1267.73	  1667.05	 14
 13	 S 	  1354.76	  1552.03	 13
 14	 I 	  1467.84	  1464.99	 12
 15	 G 	  1524.86	  1351.91	 11
 16	 A 	  1595.90	  1294.89	 10
 17	 A 	  1666.94	  1223.85	  9
 18	 H 	  1804.00	  1152.81	  8
 19	 L 	  1917.08	  1015.75	  7
 20	 I 	  2030.16	   902.67	  6
 21	 F 	  2177.23	   789.59	  5
 22	 C 	  2337.40	   642.52	  4
 23	 C 	  2497.56	   482.35	  3
 24	 F 	  2644.63	   322.19	  2
 25	 R 	        -  	   175.12	  1  _


+2 charge
		      B   	       Y
  1	 S 	    44.52	        -  	 25
  2	 C 	   124.61	  1366.36	 24
  3	 S 	   168.12	  1286.28	 23
  4	 S 	   211.64	  1242.76	 22
  5	 S 	   255.15	  1199.24	 21
  6	 C 	   335.24	  1155.73	 20
  7	 V 	   384.77	  1075.65	 19
  8	 A 	   420.29	  1026.11	 18
  9	 T 	   470.81	   990.59	 17
 10	 D 	   528.33	   940.07	 16
 11	 P 	   576.85	   882.56	 15
 12	 D 	   634.37	   834.03	 14
 13	 S 	   677.88	   776.52	 13
 14	 I 	   734.42	   733.00	 12
 15	 G 	   762.94	   676.46	 11
 16	 A 	   798.45	   647.95	 10
 17	 A 	   833.97	   612.43	  9
 18	 H 	   902.50	   576.91	  8
 19	 L 	   959.04	   508.38	  7
 20	 I 	  1015.59	   451.84	  6
 21	 F 	  1089.12	   395.30	  5
 22	 C 	  1169.20	   321.76	  4
 23	 C 	  1249.29	   241.68	  3
 24	 F 	  1322.82	   161.60	  2
 25	 R 	        -  	    88.06	  1  _


+3 charge
		      B   	       Y
  1	 S 	    30.02	        -  	 25
  2	 C 	    83.41	   911.24	 24
  3	 S 	   112.42	   857.85	 23
  4	 S 	   141.43	   828.84	 22
  5	 S 	   170.44	   799.83	 21
  6	 C 	   223.83	   770.82	 20
  7	 V 	   256.85	   717.43	 19
  8	 A 	   280.53	   684.41	 18
  9	 T 	   314.21	   660.73	 17
 10	 D 	   352.55	   627.05	 16
 11	 P 	   384.90	   588.71	 15
 12	 D 	   423.25	   556.36	 14
 13	 S 	   452.26	   518.01	 13
 14	 I 	   489.95	   489.00	 12
 15	 G 	   508.96	   451.31	 11
 16	 A 	   532.64	   432.30	 10
 17	 A 	   556.32	   408.62	  9
 18	 H 	   602.00	   384.94	  8
 19	 L 	   639.70	   339.26	  7
 20	 I 	   677.39	   301.56	  6
 21	 F 	   726.42	   263.87	  5
 22	 C 	   779.80	   214.84	  4
 23	 C 	   833.19	   161.46	  3
 24	 F 	   882.22	   108.07	  2
 25	 R 	        -  	    59.04	  1  _
```

**114. IPI00165360 3-mercaptopyruvate sulfurtransfera**se

| Sequence | Charge | M+H+ | XCorr | DeltaCn |
| --- | --- | --- | --- | --- |
| R.ALVSAQWVAEALRAPR.A | 2 | 1739.015 | 3.497 | 0.454 |

+1 charge

```
		      B   	       Y
  1	 A 	    72.04	        -  	 16
  2	 L 	   185.13	  1666.94	 15
  3	 V 	   284.20	  1553.85	 14
  4	 S 	   371.23	  1454.79	 13
  5	 A 	   442.27	  1367.75	 12
  6	 Q 	   570.32	  1296.72	 11
  7	 W 	   756.40	  1168.66	 10
  8	 V 	   855.47	   982.58	  9
  9	 A 	   926.51	   883.51	  8
 10	 E 	  1055.55	   812.47	  7
 11	 A 	  1126.59	   683.43	  6
 12	 L 	  1239.67	   612.39	  5
 13	 R 	  1395.77	   499.31	  4
 14	 A 	  1466.81	   343.21	  3
 15	 P 	  1563.86	   272.17	  2
 16	 R 	        -  	   175.12	  1  _
+2 charge
		      B   	       Y
  1	 A 	    36.53	        -  	 16
  2	 L 	    93.07	   833.97	 15
  3	 V 	   142.60	   777.43	 14
  4	 S 	   186.12	   727.90	 13
  5	 A 	   221.64	   684.38	 12
  6	 Q 	   285.67	   648.86	 11
  7	 W 	   378.71	   584.83	 10
  8	 V 	   428.24	   491.79	  9
  9	 A 	   463.76	   442.26	  8
 10	 E 	   528.28	   406.74	  7
 11	 A 	   563.80	   342.22	  6
 12	 L 	   620.34	   306.70	  5
 13	 R 	   698.39	   250.16	  4
 14	 A 	   733.91	   172.11	  3
 15	 P 	   782.44	   136.59	  2
 16	 R 	        -  	    88.06	  1  _
```

**115. IPI00937967 similar to hCG2039165**

| Sequence | Charge | M+H+ | XCorr | DeltaCn |
| --- | --- | --- | --- | --- |
| - .MRTEDLPKAVVFLEPQWYR.V | 3 | 2379.765 | 3.52 | 0.449 |

+1 charge

```
		      B   	       Y
  1	 M 	   132.05	        -  	 19
  2	 R 	   288.15	  2247.19	 18
  3	 T 	   389.20	  2091.09	 17
  4	 E 	   518.24	  1990.04	 16
  5	 D 	   633.27	  1861.00	 15
  6	 L 	   746.35	  1745.97	 14
  7	 P 	   843.40	  1632.89	 13
  8	 K 	   971.50	  1535.84	 12
  9	 A 	  1042.53	  1407.74	 11
 10	 V 	  1141.60	  1336.70	 10
 11	 V 	  1240.67	  1237.64	  9
 12	 F 	  1387.74	  1138.57	  8
 13	 L 	  1500.82	   991.50	  7
 14	 E 	  1629.87	   878.42	  6
 15	 P 	  1726.92	   749.37	  5
 16	 Q 	  1854.98	   652.32	  4
 17	 W 	  2041.06	   524.26	  3
 18	 Y 	  2204.12	   338.18	  2
 19	 R 	        -  	   175.12	  1  _
+2 charge
		      B   	       Y
  1	 M 	    66.53	        -  	 19
  2	 R 	   144.58	  1124.10	 18
  3	 T 	   195.10	  1046.05	 17
  4	 E 	   259.62	   995.53	 16
  5	 D 	   317.14	   931.00	 15
  6	 L 	   373.68	   873.49	 14
  7	 P 	   422.21	   816.95	 13
  8	 K 	   486.25	   768.42	 12
  9	 A 	   521.77	   704.37	 11
 10	 V 	   571.31	   668.86	 10
 11	 V 	   620.84	   619.32	  9
 12	 F 	   694.37	   569.79	  8
 13	 L 	   750.92	   496.25	  7
 14	 E 	   815.44	   439.71	  6
 15	 P 	   863.96	   375.19	  5
 16	 Q 	   927.99	   326.66	  4
 17	 W 	  1021.03	   262.63	  3
 18	 Y 	  1102.56	   169.59	  2
 19	 R 	        -  	    88.06	  1  _


+3 charge
		      B   	       Y
  1	 M 	    44.69	        -  	 19
  2	 R 	    96.72	   749.74	 18
  3	 T 	   130.40	   697.70	 17
  4	 E 	   173.42	   664.02	 16
  5	 D 	   211.76	   621.01	 15
  6	 L 	   249.45	   582.66	 14
  7	 P 	   281.81	   544.97	 13
  8	 K 	   324.50	   512.62	 12
  9	 A 	   348.18	   469.92	 11
 10	 V 	   381.21	   446.24	 10
 11	 V 	   414.23	   413.22	  9
 12	 F 	   463.25	   380.19	  8
 13	 L 	   500.95	   331.17	  7
 14	 E 	   543.96	   293.48	  6
 15	 P 	   576.31	   250.46	  5
 16	 Q 	   619.00	   218.11	  4
 17	 W 	   681.02	   175.43	  3
 18	 Y 	   735.38	   113.40	  2
 19	 R 	        -  	    59.04	  1  _
```

**116. IPI00940353 Isoform 1 of Gamma-glutamyltransferase 6**

| Sequence | Charge | M+H+ | XCorr | DeltaCn |
| --- | --- | --- | --- | --- |
| R.LLVGPTTLAQEGFLVDTPLAR.A | 2 | 2212.574 | 3.396 | 0.562 |

+1 charge

```
		      B   	       Y
  1	 L 	   114.09	        -  	 21
  2	 L 	   227.18	  2098.15	 20
  3	 V 	   326.24	  1985.07	 19
  4	 G 	   383.27	  1886.00	 18
  5	 P 	   480.32	  1828.98	 17
  6	 T 	   581.37	  1731.93	 16
  7	 T 	   682.41	  1630.88	 15
  8	 L 	   795.50	  1529.83	 14
  9	 A 	   866.53	  1416.75	 13
 10	 Q 	   994.59	  1345.71	 12
 11	 E 	  1123.64	  1217.65	 11
 12	 G 	  1180.66	  1088.61	 10
 13	 F 	  1327.73	  1031.59	  9
 14	 L 	  1440.81	   884.52	  8
 15	 V 	  1539.88	   771.44	  7
 16	 D 	  1654.91	   672.37	  6
 17	 T 	  1755.95	   557.34	  5
 18	 P 	  1853.01	   456.29	  4
 19	 L 	  1966.09	   359.24	  3
 20	 A 	  2037.13	   246.16	  2
 21	 R 	        -  	   175.12	  1  _


+2 charge
		      B   	       Y
  1	 L 	    57.55	        -  	 21
  2	 L 	   114.09	  1049.58	 20
  3	 V 	   163.63	   993.04	 19
  4	 G 	   192.14	   943.50	 18
  5	 P 	   240.66	   914.99	 17
  6	 T 	   291.19	   866.47	 16
  7	 T 	   341.71	   815.94	 15
  8	 L 	   398.25	   765.42	 14
  9	 A 	   433.77	   708.88	 13
 10	 Q 	   497.80	   673.36	 12
 11	 E 	   562.32	   609.33	 11
 12	 G 	   590.83	   544.81	 10
 13	 F 	   664.37	   516.30	  9
 14	 L 	   720.91	   442.76	  8
 15	 V 	   770.44	   386.22	  7
 16	 D 	   827.96	   336.69	  6
 17	 T 	   878.48	   279.17	  5
 18	 P 	   927.01	   228.65	  4
 19	 L 	   983.55	   180.12	  3
 20	 A 	  1019.07	   123.58	  2
 21	 R 	        -  	    88.06	  1  _
```

**117. IPI00032258	Complement C4-A**

| Sequence | Charge | M+H+ | XCorr | DeltaCn |
| --- | --- | --- | --- | --- |
| R.VTASDPLDTLGSEGALSPGGVASLLRLPR.G | 3 | 2851.205 | 3.588 | 0.595 |

+1 charge

```
		      B   	       Y
  1	 V 	   100.08	        -  	 29
  2	 T 	   201.12	  2750.47	 28
  3	 A 	   272.16	  2649.42	 27
  4	 S 	   359.19	  2578.38	 26
  5	 D 	   474.22	  2491.35	 25
  6	 P 	   571.27	  2376.32	 24
  7	 L 	   684.36	  2279.27	 23
  8	 D 	   799.38	  2166.19	 22
  9	 T 	   900.43	  2051.16	 21
 10	 L 	  1013.51	  1950.11	 20
 11	 G 	  1070.54	  1837.03	 19
 12	 S 	  1157.57	  1780.01	 18
 13	 E 	  1286.61	  1692.98	 17
 14	 G 	  1343.63	  1563.93	 16
 15	 A 	  1414.67	  1506.91	 15
 16	 L 	  1527.75	  1435.87	 14
 17	 S 	  1614.79	  1322.79	 13
 18	 P 	  1711.84	  1235.76	 12
 19	 G 	  1768.86	  1138.71	 11
 20	 G 	  1825.88	  1081.68	 10
 21	 V 	  1924.95	  1024.66	  9
 22	 A 	  1995.99	   925.59	  8
 23	 S 	  2083.02	   854.56	  7
 24	 L 	  2196.10	   767.53	  6
 25	 L 	  2309.19	   654.44	  5
 26	 R 	  2465.29	   541.36	  4
 27	 L 	  2578.37	   385.26	  3
 28	 P 	  2675.43	   272.17	  2
 29	 R 	        -  	   175.12	  1  _


+2 charge
		      B   	       Y
  1	 V 	    50.54	        -  	 29
  2	 T 	   101.07	  1375.74	 28
  3	 A 	   136.58	  1325.21	 27
  4	 S 	   180.10	  1289.70	 26
  5	 D 	   237.61	  1246.18	 25
  6	 P 	   286.14	  1188.67	 24
  7	 L 	   342.68	  1140.14	 23
  8	 D 	   400.20	  1083.60	 22
  9	 T 	   450.72	  1026.08	 21
 10	 L 	   507.26	   975.56	 20
 11	 G 	   535.77	   919.02	 19
 12	 S 	   579.29	   890.51	 18
 13	 E 	   643.81	   846.99	 17
 14	 G 	   672.32	   782.47	 16
 15	 A 	   707.84	   753.96	 15
 16	 L 	   764.38	   718.44	 14
 17	 S 	   807.90	   661.90	 13
 18	 P 	   856.42	   618.38	 12
 19	 G 	   884.93	   569.86	 11
 20	 G 	   913.44	   541.35	 10
 21	 V 	   962.98	   512.83	  9
 22	 A 	   998.50	   463.30	  8
 23	 S 	  1042.01	   427.78	  7
 24	 L 	  1098.56	   384.27	  6
 25	 L 	  1155.10	   327.72	  5
 26	 R 	  1233.15	   271.18	  4
 27	 L 	  1289.69	   193.13	  3
 28	 P 	  1338.22	   136.59	  2
 29	 R 	        -  	    88.06	  1  _


+3 charge
		      B   	       Y
  1	 V 	    34.03	        -  	 29
  2	 T 	    67.71	   917.49	 28
  3	 A 	    91.39	   883.81	 27
  4	 S 	   120.40	   860.13	 26
  5	 D 	   158.74	   831.12	 25
  6	 P 	   191.10	   792.78	 24
  7	 L 	   228.79	   760.43	 23
  8	 D 	   267.13	   722.73	 22
  9	 T 	   300.82	   684.39	 21
 10	 L 	   338.51	   650.71	 20
 11	 G 	   357.52	   613.01	 19
 12	 S 	   386.53	   594.01	 18
 13	 E 	   429.54	   565.00	 17
 14	 G 	   448.55	   521.98	 16
 15	 A 	   472.23	   502.98	 15
 16	 L 	   509.92	   479.30	 14
 17	 S 	   538.93	   441.60	 13
 18	 P 	   571.28	   412.59	 12
 19	 G 	   590.29	   380.24	 11
 20	 G 	   609.30	   361.23	 10
 21	 V 	   642.32	   342.23	  9
 22	 A 	   666.00	   309.20	  8
 23	 S 	   695.01	   285.52	  7
 24	 L 	   732.71	   256.51	  6
 25	 L 	   770.40	   218.82	  5
 26	 R 	   822.43	   181.12	  4
 27	 L 	   860.13	   129.09	  3
 28	 P 	   892.48	    91.40	  2
 29	 R 	        -  	    59.04	  1  _
```

**118. IPI00021885 Isoform 1 of Fibrinogen alpha chain**

| Sequence | Charge | M+H+ | XCorr | DeltaCn |
| --- | --- | --- | --- | --- |
| R.GFGSLNDEGEGEFWLGNDYLHLLTQR.G | 3 | 2969.17 | 4.728 | 0.528 |

+1 charge

```
		      B   	       Y
  1	 G 	    58.03	        -  	 26
  2	 F 	   205.10	  2910.37	 25
  3	 G 	   262.12	  2763.30	 24
  4	 S 	   349.15	  2706.28	 23
  5	 L 	   462.23	  2619.25	 22
  6	 N 	   576.28	  2506.16	 21
  7	 D 	   691.30	  2392.12	 20
  8	 E 	   820.35	  2277.09	 19
  9	 G 	   877.37	  2148.05	 18
 10	 E 	  1006.41	  2091.03	 17
 11	 G 	  1063.43	  1961.99	 16
 12	 E 	  1192.48	  1904.97	 15
 13	 F 	  1339.54	  1775.92	 14
 14	 W 	  1525.62	  1628.85	 13
 15	 L 	  1638.71	  1442.78	 12
 16	 G 	  1695.73	  1329.69	 11
 17	 N 	  1809.77	  1272.67	 10
 18	 D 	  1924.80	  1158.63	  9
 19	 Y 	  2087.86	  1043.60	  8
 20	 L 	  2200.95	   880.54	  7
 21	 H 	  2338.00	   767.45	  6
 22	 L 	  2451.09	   630.39	  5
 23	 L 	  2564.17	   517.31	  4
 24	 T 	  2665.22	   404.23	  3
 25	 Q 	  2793.28	   303.18	  2
 26	 R 	        -  	   175.12	  1  _


+2 charge
		      B   	       Y
  1	 G 	    29.52	        -  	 26
  2	 F 	   103.05	  1455.69	 25
  3	 G 	   131.56	  1382.15	 24
  4	 S 	   175.08	  1353.64	 23
  5	 L 	   231.62	  1310.13	 22
  6	 N 	   288.64	  1253.59	 21
  7	 D 	   346.16	  1196.56	 20
  8	 E 	   410.68	  1139.05	 19
  9	 G 	   439.19	  1074.53	 18
 10	 E 	   503.71	  1046.02	 17
 11	 G 	   532.22	   981.50	 16
 12	 E 	   596.74	   952.99	 15
 13	 F 	   670.28	   888.47	 14
 14	 W 	   763.32	   814.93	 13
 15	 L 	   819.86	   721.89	 12
 16	 G 	   848.37	   665.35	 11
 17	 N 	   905.39	   636.84	 10
 18	 D 	   962.90	   579.82	  9
 19	 Y 	  1044.43	   522.30	  8
 20	 L 	  1100.98	   440.77	  7
 21	 H 	  1169.51	   384.23	  6
 22	 L 	  1226.05	   315.70	  5
 23	 L 	  1282.59	   259.16	  4
 24	 T 	  1333.11	   202.62	  3
 25	 Q 	  1397.14	   152.09	  2
 26	 R 	        -  	    88.06	  1  _


+3 charge
		      B   	       Y
  1	 G 	    20.01	        -  	 26
  2	 F 	    69.04	   970.79	 25
  3	 G 	    88.04	   921.77	 24
  4	 S 	   117.06	   902.76	 23
  5	 L 	   154.75	   873.75	 22
  6	 N 	   192.76	   836.06	 21
  7	 D 	   231.11	   798.04	 20
  8	 E 	   274.12	   759.70	 19
  9	 G 	   293.13	   716.69	 18
 10	 E 	   336.14	   697.68	 17
 11	 G 	   355.15	   654.67	 16
 12	 E 	   398.16	   635.66	 15
 13	 F 	   447.19	   592.65	 14
 14	 W 	   509.21	   543.62	 13
 15	 L 	   546.91	   481.60	 12
 16	 G 	   565.91	   443.90	 11
 17	 N 	   603.93	   424.89	 10
 18	 D 	   642.27	   386.88	  9
 19	 Y 	   696.63	   348.54	  8
 20	 L 	   734.32	   294.18	  7
 21	 H 	   780.01	   256.49	  6
 22	 L 	   817.70	   210.80	  5
 23	 L 	   855.40	   173.11	  4
 24	 T 	   889.08	   135.41	  3
 25	 Q 	   931.76	   101.73	  2
 26	 R 	        -  	    59.04	  1  _
```

**119. IPI00034319 Isoform A of Protein CutA**

| Sequence | Charge | M+H+ | XCorr | DeltaCn |
| --- | --- | --- | --- | --- |
| R.SVHPYEVAEVIALPVEQGNFPYLQWVR.Q | 3 | 3142.554 | 5.494 | 0.551 |

+1 charge

```
		      B   	       Y
  1	 S 	    88.04	        -  	 27
  2	 V 	   187.11	  3053.59	 26
  3	 H 	   324.17	  2954.52	 25
  4	 P 	   421.22	  2817.46	 24
  5	 Y 	   584.28	  2720.41	 23
  6	 E 	   713.33	  2557.34	 22
  7	 V 	   812.39	  2428.30	 21
  8	 A 	   883.43	  2329.23	 20
  9	 E 	  1012.47	  2258.20	 19
 10	 V 	  1111.54	  2129.15	 18
 11	 I 	  1224.63	  2030.09	 17
 12	 A 	  1295.66	  1917.00	 16
 13	 L 	  1408.75	  1845.96	 15
 14	 P 	  1505.80	  1732.88	 14
 15	 V 	  1604.87	  1635.83	 13
 16	 E 	  1733.91	  1536.76	 12
 17	 Q 	  1861.97	  1407.72	 11
 18	 G 	  1918.99	  1279.66	 10
 19	 N 	  2033.03	  1222.64	  9
 20	 F 	  2180.10	  1108.59	  8
 21	 P 	  2277.16	   961.53	  7
 22	 Y 	  2440.22	   864.47	  6
 23	 L 	  2553.30	   701.41	  5
 24	 Q 	  2681.36	   588.33	  4
 25	 W 	  2867.44	   460.27	  3
 26	 V 	  2966.51	   274.19	  2
 27	 R 	        -  	   175.12	  1  _


+2 charge
		      B   	       Y
  1	 S 	    44.52	        -  	 27
  2	 V 	    94.06	  1527.30	 26
  3	 H 	   162.59	  1477.76	 25
  4	 P 	   211.11	  1409.23	 24
  5	 Y 	   292.64	  1360.71	 23
  6	 E 	   357.17	  1279.18	 22
  7	 V 	   406.70	  1214.65	 21
  8	 A 	   442.22	  1165.12	 20
  9	 E 	   506.74	  1129.60	 19
 10	 V 	   556.27	  1065.08	 18
 11	 I 	   612.82	  1015.55	 17
 12	 A 	   648.34	   959.00	 16
 13	 L 	   704.88	   923.49	 15
 14	 P 	   753.40	   866.94	 14
 15	 V 	   802.94	   818.42	 13
 16	 E 	   867.46	   768.88	 12
 17	 Q 	   931.49	   704.36	 11
 18	 G 	   960.00	   640.33	 10
 19	 N 	  1017.02	   611.82	  9
 20	 F 	  1090.55	   554.80	  8
 21	 P 	  1139.08	   481.27	  7
 22	 Y 	  1220.61	   432.74	  6
 23	 L 	  1277.15	   351.21	  5
 24	 Q 	  1341.18	   294.67	  4
 25	 W 	  1434.22	   230.64	  3
 26	 V 	  1483.76	   137.60	  2
 27	 R 	        -  	    88.06	  1  _


+3 charge
		      B   	       Y
  1	 S 	    30.02	        -  	 27
  2	 V 	    63.04	  1018.53	 26
  3	 H 	   108.73	   985.51	 25
  4	 P 	   141.08	   939.83	 24
  5	 Y 	   195.43	   907.47	 23
  6	 E 	   238.45	   853.12	 22
  7	 V 	   271.47	   810.11	 21
  8	 A 	   295.15	   777.08	 20
  9	 E 	   338.16	   753.40	 19
 10	 V 	   371.19	   710.39	 18
 11	 I 	   408.88	   677.37	 17
 12	 A 	   432.56	   639.67	 16
 13	 L 	   470.25	   615.99	 15
 14	 P 	   502.60	   578.30	 14
 15	 V 	   535.63	   545.95	 13
 16	 E 	   578.64	   512.92	 12
 17	 Q 	   621.33	   469.91	 11
 18	 G 	   640.34	   427.22	 10
 19	 N 	   678.35	   408.22	  9
 20	 F 	   727.37	   370.20	  8
 21	 P 	   759.72	   321.18	  7
 22	 Y 	   814.08	   288.83	  6
 23	 L 	   851.77	   234.47	  5
 24	 Q 	   894.46	   196.78	  4
 25	 W 	   956.48	   154.09	  3
 26	 V 	   989.51	    92.07	  2
 27	 R 	        -  	    59.04	  1  _
```

**120. IPI00021434 Elafin**

| Sequence | Charge | M+H+ | XCorr | DeltaCn |
| --- | --- | --- | --- | --- |
| K.AQEPVKGPVSTKPGSCPIILIR.C | 3 | 2348.764 | 3.494 | 0.358 |

+1 charge

```
		      B   	       Y
  1	 A 	    72.04	        -  	 22
  2	 Q 	   200.10	  2276.41	 21
  3	 E 	   329.15	  2148.36	 20
  4	 P 	   426.20	  2019.31	 19
  5	 V 	   525.27	  1922.26	 18
  6	 K 	   653.36	  1823.19	 17
  7	 G 	   710.38	  1695.10	 16
  8	 P 	   807.44	  1638.08	 15
  9	 V 	   906.50	  1541.02	 14
 10	 S 	   993.54	  1441.95	 13
 11	 T 	  1094.58	  1354.92	 12
 12	 K 	  1222.68	  1253.87	 11
 13	 P 	  1319.73	  1125.78	 10
 14	 G 	  1376.75	  1028.73	  9
 15	 S 	  1463.79	   971.71	  8
 16	 C 	  1623.95	   884.67	  7
 17	 P 	  1721.00	   724.51	  6
 18	 I 	  1834.09	   627.46	  5
 19	 I 	  1947.17	   514.37	  4
 20	 L 	  2060.26	   401.29	  3
 21	 I 	  2173.34	   288.20	  2
 22	 R 	        -  	   175.12	  1  _


+2 charge
		      B   	       Y
  1	 A 	    36.53	        -  	 22
  2	 Q 	   100.56	  1138.71	 21
  3	 E 	   165.08	  1074.68	 20
  4	 P 	   213.60	  1010.16	 19
  5	 V 	   263.14	   961.63	 18
  6	 K 	   327.18	   912.10	 17
  7	 G 	   355.70	   848.05	 16
  8	 P 	   404.22	   819.54	 15
  9	 V 	   453.76	   771.01	 14
 10	 S 	   497.27	   721.48	 13
 11	 T 	   547.80	   677.96	 12
 12	 K 	   611.84	   627.44	 11
 13	 P 	   660.37	   563.39	 10
 14	 G 	   688.88	   514.87	  9
 15	 S 	   732.40	   486.36	  8
 16	 C 	   812.48	   442.84	  7
 17	 P 	   861.01	   362.76	  6
 18	 I 	   917.55	   314.23	  5
 19	 I 	   974.09	   257.69	  4
 20	 L 	  1030.63	   201.15	  3
 21	 I 	  1087.17	   144.61	  2
 22	 R 	        -  	    88.06	  1  _


+3 charge
		      B   	       Y
  1	 A 	    24.69	        -  	 22
  2	 Q 	    67.37	   759.48	 21
  3	 E 	   110.39	   716.79	 20
  4	 P 	   142.74	   673.78	 19
  5	 V 	   175.76	   641.42	 18
  6	 K 	   218.46	   608.40	 17
  7	 G 	   237.47	   565.70	 16
  8	 P 	   269.82	   546.70	 15
  9	 V 	   302.84	   514.35	 14
 10	 S 	   331.85	   481.32	 13
 11	 T 	   365.53	   452.31	 12
 12	 K 	   408.23	   418.63	 11
 13	 P 	   440.58	   375.93	 10
 14	 G 	   459.59	   343.58	  9
 15	 S 	   488.60	   324.57	  8
 16	 C 	   541.99	   295.56	  7
 17	 P 	   574.34	   242.17	  6
 18	 I 	   612.03	   209.82	  5
 19	 I 	   649.73	   172.13	  4
 20	 L 	   687.42	   134.43	  3
 21	 I 	   725.12	    96.74	  2
 22	 R 	        -  	    59.04	  1  _
```

**121. IPI00001952 Endonuclease domain-containing 1 protein**

| Sequence | Charge | M+H+ | XCorr | DeltaCn |
| --- | --- | --- | --- | --- |
| K.ILEVVNQIQDEER.M | 2 | 1585.741 | 3.862 | 0.463 |

+1 charge

```
		      B   	       Y
  1	 I 	   114.09	        -  	 13
  2	 L 	   227.18	  1471.74	 12
  3	 E 	   356.22	  1358.65	 11
  4	 V 	   455.29	  1229.61	 10
  5	 V 	   554.35	  1130.54	  9
  6	 N 	   668.40	  1031.48	  8
  7	 Q 	   796.46	   917.43	  7
  8	 I 	   909.54	   789.37	  6
  9	 Q 	  1037.60	   676.29	  5
 10	 D 	  1152.63	   548.23	  4
 11	 E 	  1281.67	   433.20	  3
 12	 E 	  1410.71	   304.16	  2
 13	 R 	        -  	   175.12	  1  _
+2 charge
		      B   	       Y
  1	 I 	    57.55	        -  	 13
  2	 L 	   114.09	   736.37	 12
  3	 E 	   178.61	   679.83	 11
  4	 V 	   228.15	   615.31	 10
  5	 V 	   277.68	   565.78	  9
  6	 N 	   334.70	   516.24	  8
  7	 Q 	   398.73	   459.22	  7
  8	 I 	   455.27	   395.19	  6
  9	 Q 	   519.30	   338.65	  5
 10	 D 	   576.82	   274.62	  4
 11	 E 	   641.34	   217.11	  3
 12	 E 	   705.86	   152.58	  2
 13	 R 	        -  	    88.06	  1  _
```

**122. IPI00102300 Isoform 3 of Platelet glycoprotein VI**

| Sequence | Charge | M+H+ | XCorr | DeltaCn |
| --- | --- | --- | --- | --- |
| R.ASFPIITVTAAHSGTYR.C | 2 | 1793.016 | 3.778 | 0.497 |

+1 charge

```
		      B   	       Y
  1	 A 	    72.04	        -  	 17
  2	 S 	   159.08	  1720.90	 16
  3	 F 	   306.14	  1633.87	 15
  4	 P 	   403.20	  1486.80	 14
  5	 I 	   516.28	  1389.75	 13
  6	 I 	   629.37	  1276.66	 12
  7	 T 	   730.41	  1163.58	 11
  8	 V 	   829.48	  1062.53	 10
  9	 T 	   930.53	   963.46	  9
 10	 A 	  1001.57	   862.42	  8
 11	 A 	  1072.60	   791.38	  7
 12	 H 	  1209.66	   720.34	  6
 13	 S 	  1296.69	   583.28	  5
 14	 G 	  1353.72	   496.25	  4
 15	 T 	  1454.76	   439.23	  3
 16	 Y 	  1617.83	   338.18	  2
 17	 R 	        -  	   175.12	  1  _
+2 charge
		      B   	       Y
  1	 A 	    36.53	        -  	 17
  2	 S 	    80.04	   860.95	 16
  3	 F 	   153.58	   817.44	 15
  4	 P 	   202.10	   743.90	 14
  5	 I 	   258.64	   695.38	 13
  6	 I 	   315.19	   638.84	 12
  7	 T 	   365.71	   582.29	 11
  8	 V 	   415.24	   531.77	 10
  9	 T 	   465.77	   482.24	  9
 10	 A 	   501.29	   431.71	  8
 11	 A 	   536.81	   396.19	  7
 12	 H 	   605.33	   360.67	  6
 13	 S 	   648.85	   292.15	  5
 14	 G 	   677.36	   248.63	  4
 15	 T 	   727.89	   220.12	  3
 16	 Y 	   809.42	   169.59	  2
 17	 R 	        -  	    88.06	  1  _
```

**123. IPI00385252 Ig kappa chain V-III region GOL**

| Sequence | Charge | M+H+ | XCorr | DeltaCn |
| --- | --- | --- | --- | --- |
| - .EIVLTQSPGTLSLSPGER.A | 2 | 1885.108 | 3.021 | 0.35 |

+1 charge

```
		      B   	       Y
  1	 E 	   130.05	        -  	 18
  2	 I 	   243.13	  1754.96	 17
  3	 V 	   342.20	  1641.88	 16
  4	 L 	   455.29	  1542.81	 15
  5	 T 	   556.33	  1429.73	 14
  6	 Q 	   684.39	  1328.68	 13
  7	 S 	   771.42	  1200.62	 12
  8	 P 	   868.48	  1113.59	 11
  9	 G 	   925.50	  1016.54	 10
 10	 T 	  1026.55	   959.52	  9
 11	 L 	  1139.63	   858.47	  8
 12	 S 	  1226.66	   745.38	  7
 13	 L 	  1339.75	   658.35	  6
 14	 S 	  1426.78	   545.27	  5
 15	 P 	  1523.83	   458.24	  4
 16	 G 	  1580.85	   361.18	  3
 17	 E 	  1709.90	   304.16	  2
 18	 R 	        -  	   175.12	  1  _
+2 charge
		      B   	       Y
  1	 E 	    65.53	        -  	 18
  2	 I 	   122.07	   877.99	 17
  3	 V 	   171.60	   821.44	 16
  4	 L 	   228.15	   771.91	 15
  5	 T 	   278.67	   715.37	 14
  6	 Q 	   342.70	   664.84	 13
  7	 S 	   386.22	   600.81	 12
  8	 P 	   434.74	   557.30	 11
  9	 G 	   463.25	   508.77	 10
 10	 T 	   513.78	   480.26	  9
 11	 L 	   570.32	   429.74	  8
 12	 S 	   613.83	   373.20	  7
 13	 L 	   670.38	   329.68	  6
 14	 S 	   713.89	   273.14	  5
 15	 P 	   762.42	   229.62	  4
 16	 G 	   790.93	   181.10	  3
 17	 E 	   855.45	   152.58	  2
 18	 R 	        -  	    88.06	  1  _
```

**124. IPI00651692 Stromal cell-derived factor 1 theta**

| Sequence | Charge | M+H+ | XCorr | DeltaCn |
| --- | --- | --- | --- | --- |
| K.ILNTPNCALQIVAR.L | 2 | 1583.851 | 4.089 | 0.406 |

+1 charge

```
		      B   	       Y
  1	 I 	   114.09	        -  	 14
  2	 L 	   227.18	  1469.92	 13
  3	 N 	   341.22	  1356.84	 12
  4	 T 	   442.27	  1242.80	 11
  5	 P 	   539.32	  1141.75	 10
  6	 N 	   653.36	  1044.70	  9
  7	 C 	   813.53	   930.65	  8
  8	 A 	   884.56	   770.49	  7
  9	 L 	   997.65	   699.45	  6
 10	 Q 	  1125.71	   586.37	  5
 11	 I 	  1238.79	   458.31	  4
 12	 V 	  1337.86	   345.22	  3
 13	 A 	  1408.90	   246.16	  2
 14	 R 	        -  	   175.12	  1  _
+2 charge
		      B   	       Y
  1	 I 	    57.55	        -  	 14
  2	 L 	   114.09	   735.47	 13
  3	 N 	   171.11	   678.92	 12
  4	 T 	   221.64	   621.90	 11
  5	 P 	   270.16	   571.38	 10
  6	 N 	   327.18	   522.85	  9
  7	 C 	   407.27	   465.83	  8
  8	 A 	   442.79	   385.75	  7
  9	 L 	   499.33	   350.23	  6
 10	 Q 	   563.36	   293.69	  5
 11	 I 	   619.90	   229.66	  4
 12	 V 	   669.43	   173.12	  3
 13	 A 	   704.95	   123.58	  2
 14	 R 	        -  	    88.06	  1  _
```

**125. IPI00013446 Prostate stem cell antigen**

| Sequence | Charge | M+H+ | XCorr | DeltaCn |
| --- | --- | --- | --- | --- |
| R.IRAVGLLTVISK.G | 2 | 1270.59 | 3.079 | 0.395 |

+1 charge

```
		      B   	       Y
  1	 I 	   114.09	        -  	 12
  2	 R 	   270.19	  1156.74	 11
  3	 A 	   341.23	  1000.64	 10
  4	 V 	   440.30	   929.60	  9
  5	 G 	   497.32	   830.53	  8
  6	 L 	   610.40	   773.51	  7
  7	 L 	   723.49	   660.43	  6
  8	 T 	   824.54	   547.34	  5
  9	 V 	   923.60	   446.30	  4
 10	 I 	  1036.69	   347.23	  3
 11	 S 	  1123.72	   234.14	  2
 12	 K 	        -  	   147.11	  1  _
+2 charge
		      B   	       Y
  1	 I 	    57.55	        -  	 12
  2	 R 	   135.60	   578.87	 11
  3	 A 	   171.12	   500.82	 10
  4	 V 	   220.65	   465.31	  9
  5	 G 	   249.16	   415.77	  8
  6	 L 	   305.71	   387.26	  7
  7	 L 	   362.25	   330.72	  6
  8	 T 	   412.77	   274.18	  5
  9	 V 	   462.31	   223.65	  4
 10	 I 	   518.85	   174.12	  3
 11	 S 	   562.36	   117.58	  2
 12	 K 	        -  	    74.06	  1  _
```

**126. IPI00000874 Peroxiredoxin-1**

| Sequence | Charge | M+H+ | XCorr | DeltaCn |
| --- | --- | --- | --- | --- |
| R.TIAQDYGVLKADEGISFR.G | 2 | 1984.199 | 3.694 | 0.563 |

+1 charge

```
		      B   	       Y
  1	 T 	   102.05	        -  	 18
  2	 I 	   215.14	  1881.97	 17
  3	 A 	   286.18	  1768.89	 16
  4	 Q 	   414.23	  1697.85	 15
  5	 D 	   529.26	  1569.79	 14
  6	 Y 	   692.32	  1454.76	 13
  7	 G 	   749.35	  1291.70	 12
  8	 V 	   848.41	  1234.68	 11
  9	 L 	   961.50	  1135.61	 10
 10	 K 	  1089.59	  1022.53	  9
 11	 A 	  1160.63	   894.43	  8
 12	 D 	  1275.66	   823.39	  7
 13	 E 	  1404.70	   708.37	  6
 14	 G 	  1461.72	   579.32	  5
 15	 I 	  1574.81	   522.30	  4
 16	 S 	  1661.84	   409.22	  3
 17	 F 	  1808.91	   322.19	  2
 18	 R 	        -  	   175.12	  1  _
+2 charge
		      B   	       Y
  1	 T 	    51.53	        -  	 18
  2	 I 	   108.07	   941.49	 17
  3	 A 	   143.59	   884.95	 16
  4	 Q 	   207.62	   849.43	 15
  5	 D 	   265.13	   785.40	 14
  6	 Y 	   346.67	   727.89	 13
  7	 G 	   375.18	   646.35	 12
  8	 V 	   424.71	   617.84	 11
  9	 L 	   481.25	   568.31	 10
 10	 K 	   545.30	   511.77	  9
 11	 A 	   580.82	   447.72	  8
 12	 D 	   638.33	   412.20	  7
 13	 E 	   702.85	   354.69	  6
 14	 G 	   731.36	   290.17	  5
 15	 I 	   787.91	   261.66	  4
 16	 S 	   831.42	   205.11	  3
 17	 F 	   904.96	   161.60	  2
 18	 R 	        -  	    88.06	  1  _
```

**127. IPI00855900 Isoform 3 of Proline-rich acidic protein 1**

| Sequence | Charge | M+H+ | XCorr | DeltaCn |
| --- | --- | --- | --- | --- |
| K.DDQLVVLFPVQKPK.L | 2 | 1626.921 | 3.127 | 0.369 |

+1 charge

```
		      B   	       Y
  1	 D 	   116.03	        -  	 14
  2	 D 	   231.06	  1510.90	 13
  3	 Q 	   359.12	  1395.87	 12
  4	 L 	   472.20	  1267.81	 11
  5	 V 	   571.27	  1154.73	 10
  6	 V 	   670.34	  1055.66	  9
  7	 L 	   783.42	   956.59	  8
  8	 F 	   930.49	   843.51	  7
  9	 P 	  1027.55	   696.44	  6
 10	 V 	  1126.61	   599.39	  5
 11	 Q 	  1254.67	   500.32	  4
 12	 K 	  1382.77	   372.26	  3
 13	 P 	  1479.82	   244.17	  2
 14	 K 	        -  	   147.11	  1  _
+2 charge
		      B   	       Y
  1	 D 	    58.52	        -  	 14
  2	 D 	   116.03	   755.95	 13
  3	 Q 	   180.06	   698.44	 12
  4	 L 	   236.61	   634.41	 11
  5	 V 	   286.14	   577.87	 10
  6	 V 	   335.67	   528.33	  9
  7	 L 	   392.22	   478.80	  8
  8	 F 	   465.75	   422.26	  7
  9	 P 	   514.28	   348.72	  6
 10	 V 	   563.81	   300.20	  5
 11	 Q 	   627.84	   250.66	  4
 12	 K 	   691.89	   186.63	  3
 13	 P 	   740.41	   122.59	  2
 14	 K 	        -  	    74.06	  1  _
```

**128. IPI00007664 Plasma glutamate carboxypeptidase**

| Sequence | Charge | M+H+ | XCorr | DeltaCn |
| --- | --- | --- | --- | --- |
| R.TQGAVEAAKVGALASLIR.S | 2 | 1756.04 | 3.246 | 0.357 |

+1 charge

```
		      B   	       Y
  1	 T 	   102.05	        -  	 18
  2	 Q 	   230.11	  1653.96	 17
  3	 G 	   287.13	  1525.91	 16
  4	 A 	   358.17	  1468.88	 15
  5	 V 	   457.24	  1397.85	 14
  6	 E 	   586.28	  1298.78	 13
  7	 A 	   657.32	  1169.74	 12
  8	 A 	   728.36	  1098.70	 11
  9	 K 	   856.45	  1027.66	 10
 10	 V 	   955.52	   899.57	  9
 11	 G 	  1012.54	   800.50	  8
 12	 A 	  1083.58	   743.48	  7
 13	 L 	  1196.66	   672.44	  6
 14	 A 	  1267.70	   559.36	  5
 15	 S 	  1354.73	   488.32	  4
 16	 L 	  1467.82	   401.29	  3
 17	 I 	  1580.90	   288.20	  2
 18	 R 	        -  	   175.12	  1  _
+2 charge
		      B   	       Y
  1	 T 	    51.53	        -  	 18
  2	 Q 	   115.56	   827.49	 17
  3	 G 	   144.07	   763.46	 16
  4	 A 	   179.59	   734.95	 15
  5	 V 	   229.12	   699.43	 14
  6	 E 	   293.65	   649.89	 13
  7	 A 	   329.16	   585.37	 12
  8	 A 	   364.68	   549.85	 11
  9	 K 	   428.73	   514.33	 10
 10	 V 	   478.26	   450.29	  9
 11	 G 	   506.77	   400.75	  8
 12	 A 	   542.29	   372.24	  7
 13	 L 	   598.84	   336.72	  6
 14	 A 	   634.35	   280.18	  5
 15	 S 	   677.87	   244.66	  4
 16	 L 	   734.41	   201.15	  3
 17	 I 	   790.95	   144.61	  2
 18	 R 	        -  	    88.06	  1  _
```

**129. IPI00333913 Isoform 1 of Neuroblastoma-amplified sequence**

| Sequence | Charge | M+H+ | XCorr | DeltaCn |
| --- | --- | --- | --- | --- |
| K.QINWHLVLASNGKLLAAVQDQCVEIR.S | 3 | 2977.404 | 3.518 | 0.251 |

+1 charge

```
		      B   	       Y
  1	 Q 	   129.07	        -  	 26
  2	 I 	   242.15	  2847.66	 25
  3	 N 	   356.19	  2734.58	 24
  4	 W 	   542.27	  2620.54	 23
  5	 H 	   679.33	  2434.46	 22
  6	 L 	   792.42	  2297.40	 21
  7	 V 	   891.48	  2184.32	 20
  8	 L 	  1004.57	  2085.25	 19
  9	 A 	  1075.60	  1972.16	 18
 10	 S 	  1162.64	  1901.13	 17
 11	 N 	  1276.68	  1814.09	 16
 12	 G 	  1333.70	  1700.05	 15
 13	 K 	  1461.80	  1643.03	 14
 14	 L 	  1574.88	  1514.93	 13
 15	 L 	  1687.96	  1401.85	 12
 16	 A 	  1759.00	  1288.77	 11
 17	 A 	  1830.04	  1217.73	 10
 18	 V 	  1929.11	  1146.69	  9
 19	 Q 	  2057.17	  1047.62	  8
 20	 D 	  2172.19	   919.56	  7
 21	 Q 	  2300.25	   804.54	  6
 22	 C 	  2460.42	   676.48	  5
 23	 V 	  2559.48	   516.31	  4
 24	 E 	  2688.53	   417.25	  3
 25	 I 	  2801.61	   288.20	  2
 26	 R 	        -  	   175.12	  1  _


+2 charge
		      B   	       Y
  1	 Q 	    65.04	        -  	 26
  2	 I 	   121.58	  1424.34	 25
  3	 N 	   178.60	  1367.79	 24
  4	 W 	   271.64	  1310.77	 23
  5	 H 	   340.17	  1217.73	 22
  6	 L 	   396.71	  1149.20	 21
  7	 V 	   446.25	  1092.66	 20
  8	 L 	   502.79	  1043.13	 19
  9	 A 	   538.31	   986.58	 18
 10	 S 	   581.82	   951.07	 17
 11	 N 	   638.84	   907.55	 16
 12	 G 	   667.35	   850.53	 15
 13	 K 	   731.40	   822.02	 14
 14	 L 	   787.94	   757.97	 13
 15	 L 	   844.49	   701.43	 12
 16	 A 	   880.00	   644.89	 11
 17	 A 	   915.52	   609.37	 10
 18	 V 	   965.06	   573.85	  9
 19	 Q 	  1029.09	   524.32	  8
 20	 D 	  1086.60	   460.29	  7
 21	 Q 	  1150.63	   402.77	  6
 22	 C 	  1230.71	   338.74	  5
 23	 V 	  1280.25	   258.66	  4
 24	 E 	  1344.77	   209.13	  3
 25	 I 	  1401.31	   144.61	  2
 26	 R 	        -  	    88.06	  1  _


+3 charge
		      B   	       Y
  1	 Q 	    43.69	        -  	 26
  2	 I 	    81.39	   949.89	 25
  3	 N 	   119.40	   912.20	 24
  4	 W 	   181.43	   874.18	 23
  5	 H 	   227.12	   812.16	 22
  6	 L 	   264.81	   766.47	 21
  7	 V 	   297.83	   728.78	 20
  8	 L 	   335.53	   695.75	 19
  9	 A 	   359.21	   658.06	 18
 10	 S 	   388.22	   634.38	 17
 11	 N 	   426.23	   605.37	 16
 12	 G 	   445.24	   567.36	 15
 13	 K 	   487.94	   548.35	 14
 14	 L 	   525.63	   505.65	 13
 15	 L 	   563.33	   467.95	 12
 16	 A 	   587.01	   430.26	 11
 17	 A 	   610.68	   406.58	 10
 18	 V 	   643.71	   382.90	  9
 19	 Q 	   686.39	   349.88	  8
 20	 D 	   724.74	   307.19	  7
 21	 Q 	   767.42	   268.85	  6
 22	 C 	   820.81	   226.16	  5
 23	 V 	   853.83	   172.78	  4
 24	 E 	   896.85	   139.75	  3
 25	 I 	   934.54	    96.74	  2
 26	 R 	        -  	    59.04	  1  _
```

**Female 4**

**1.IPI00291867 Complement factor I**

| Sequence | Charge | M+H+ | XCorr | DeltaCn |
| --- | --- | --- | --- | --- |
| K.LVDQDKTMFICK.S | 2 | 1498.763 | 3.306 | 0.471 |

```
+1 charge
		      B   	       Y
  1	 L 	   114.09	        -  	 12
  2	 V 	   213.16	  1384.79	 11
  3	 D 	   328.19	  1285.73	 10
  4	 Q 	   456.25	  1170.70	  9
  5	 D 	   571.27	  1042.64	  8
  6	 K 	   699.37	   927.61	  7
  7	 T 	   800.41	   799.52	  6
  8	 M 	   931.46	   698.47	  5
  9	 F 	  1078.52	   567.43	  4
 10	 I 	  1191.61	   420.36	  3
 11	 C 	  1351.77	   307.28	  2
 12	 K 	        -  	   147.11	  1  _
+2 charge
		      B   	       Y
  1	 L 	    57.55	        -  	 12
  2	 V 	   107.08	   692.90	 11
  3	 D 	   164.60	   643.37	 10
  4	 Q 	   228.63	   585.85	  9
  5	 D 	   286.14	   521.82	  8
  6	 K 	   350.19	   464.31	  7
  7	 T 	   400.71	   400.26	  6
  8	 M 	   466.23	   349.74	  5
  9	 F 	   539.77	   284.22	  4
 10	 I 	   596.31	   210.68	  3
 11	 C 	   676.39	   154.14	  2
 12	 K 	        -  	    74.06	  1  _
```

**2. IPI00004101 Betaine--homocysteine S-methyltransferase 1**

| Sequence | Charge | M+H+ | XCorr | DeltaCn |
| --- | --- | --- | --- | --- |
| R.IASGRPYNPSMSKPDGWGVTK.G | 3 | 2249.535 | 3.503 | 0.297 |

+1 charge

```
		      B   	       Y
  1	 I 	   114.09	        -  	 21
  2	 A 	   185.13	  2135.03	 20
  3	 S 	   272.16	  2064.00	 19
  4	 G 	   329.18	  1976.96	 18
  5	 R 	   485.28	  1919.94	 17
  6	 P 	   582.34	  1763.84	 16
  7	 Y 	   745.40	  1666.79	 15
  8	 N 	   859.44	  1503.73	 14
  9	 P 	   956.49	  1389.68	 13
 10	 S 	  1043.53	  1292.63	 12
 11	 M 	  1174.57	  1205.60	 11
 12	 S 	  1261.60	  1074.56	 10
 13	 K 	  1389.69	   987.53	  9
 14	 P 	  1486.75	   859.43	  8
 15	 D 	  1601.77	   762.38	  7
 16	 G 	  1658.80	   647.35	  6
 17	 W 	  1844.87	   590.33	  5
 18	 G 	  1901.90	   404.25	  4
 19	 V 	  2000.96	   347.23	  3
 20	 T 	  2102.01	   248.16	  2
 21	 K 	        -  	   147.11	  1  _
+2 charge
		      B   	       Y
  1	 I 	    57.55	        -  	 21
  2	 A 	    93.07	  1068.02	 20
  3	 S 	   136.58	  1032.50	 19
  4	 G 	   165.09	   988.99	 18
  5	 R 	   243.15	   960.48	 17
  6	 P 	   291.67	   882.42	 16
  7	 Y 	   373.20	   833.90	 15
  8	 N 	   430.22	   752.37	 14
  9	 P 	   478.75	   695.35	 13
 10	 S 	   522.27	   646.82	 12
 11	 M 	   587.79	   603.30	 11
 12	 S 	   631.30	   537.78	 10
 13	 K 	   695.35	   494.27	  9
 14	 P 	   743.88	   430.22	  8
 15	 D 	   801.39	   381.69	  7
 16	 G 	   829.90	   324.18	  6
 17	 W 	   922.94	   295.67	  5
 18	 G 	   951.45	   202.63	  4
 19	 V 	  1000.99	   174.12	  3
 20	 T 	  1051.51	   124.58	  2
 21	 K 	        -  	    74.06	  1  _
+3 charge
		      B   	       Y
  1	 I 	    38.70	        -  	 21
  2	 A 	    62.38	   712.35	 20
  3	 S 	    91.39	   688.67	 19
  4	 G 	   110.40	   659.66	 18
  5	 R 	   162.43	   640.65	 17
  6	 P 	   194.78	   588.62	 16
  7	 Y 	   249.14	   556.27	 15
  8	 N 	   287.15	   501.91	 14
  9	 P 	   319.50	   463.90	 13
 10	 S 	   348.51	   431.55	 12
 11	 M 	   392.19	   402.54	 11
 12	 S 	   421.20	   358.86	 10
 13	 K 	   463.90	   329.85	  9
 14	 P 	   496.25	   287.15	  8
 15	 D 	   534.60	   254.80	  7
 16	 G 	   553.60	   216.46	  6
 17	 W 	   615.63	   197.45	  5
 18	 G 	   634.64	   135.42	  4
 19	 V 	   667.66	   116.41	  3
 20	 T 	   701.34	    83.39	  2
 21	 K 	        -  	    49.71	  1  _
```

**3. IPI00022284 Major prion protein**

| Sequence | Charge | M+H+ | XCorr | DeltaCn |
| --- | --- | --- | --- | --- |
| R.VVEQMCITQYER.E | 2 | 1556.76 | 3.071 | 0.538 |

+1 charge

```
		      B   	       Y
  1	 V 	   100.08	        -  	 12
  2	 V 	   199.14	  1456.79	 11
  3	 E 	   328.19	  1357.72	 10
  4	 Q 	   456.25	  1228.68	  9
  5	 M 	   587.29	  1100.62	  8
  6	 C 	   747.45	   969.58	  7
  7	 I 	   860.54	   809.42	  6
  8	 T 	   961.58	   696.33	  5
  9	 Q 	  1089.64	   595.28	  4
 10	 Y 	  1252.70	   467.22	  3
 11	 E 	  1381.75	   304.16	  2
 12	 R 	        -  	   175.12	  1  _
+2 charge
		      B   	       Y
  1	 V 	    50.54	        -  	 12
  2	 V 	   100.08	   728.90	 11
  3	 E 	   164.60	   679.36	 10
  4	 Q 	   228.63	   614.84	  9
  5	 M 	   294.15	   550.81	  8
  6	 C 	   374.23	   485.29	  7
  7	 I 	   430.77	   405.21	  6
  8	 T 	   481.30	   348.67	  5
  9	 Q 	   545.32	   298.15	  4
 10	 Y 	   626.86	   234.12	  3
 11	 E 	   691.38	   152.58	  2
 12	 R 	        -  	    88.06	  1  _
```

**4. IPI00219827 Secreted Ly-6/uPAR-related protein 2**

| Sequence | Charge | M+H+ | XCorr | DeltaCn |
| --- | --- | --- | --- | --- |
| R.VLSNTEDLPLVTK.M | 2 | 1429.641 | 4.663 | 0.538 |

+1 charge

```
		      B   	       Y
  1	 V 	   100.08	        -  	 13
  2	 L 	   213.16	  1329.73	 12
  3	 S 	   300.19	  1216.64	 11
  4	 N 	   414.23	  1129.61	 10
  5	 T 	   515.28	  1015.57	  9
  6	 E 	   644.32	   914.52	  8
  7	 D 	   759.35	   785.48	  7
  8	 L 	   872.44	   670.45	  6
  9	 P 	   969.49	   557.37	  5
 10	 L 	  1082.57	   460.31	  4
 11	 V 	  1181.64	   347.23	  3
 12	 T 	  1282.69	   248.16	  2
 13	 K 	        -  	   147.11	  1  _
+2 charge
		      B   	       Y
  1	 V 	    50.54	        -  	 13
  2	 L 	   107.08	   665.37	 12
  3	 S 	   150.60	   608.82	 11
  4	 N 	   207.62	   565.31	 10
  5	 T 	   258.14	   508.29	  9
  6	 E 	   322.67	   457.76	  8
  7	 D 	   380.18	   393.24	  7
  8	 L 	   436.72	   335.73	  6
  9	 P 	   485.25	   279.19	  5
 10	 L 	   541.79	   230.66	  4
 11	 V 	   591.32	   174.12	  3
 12	 T 	   641.85	   124.58	  2
 13	 K 	        -  	    74.06	  1  _
```

**5. IPI00409698 Isoform 1 of Transportin-2**

| Sequence | Charge | M+H+ | XCorr | DeltaCn |
| --- | --- | --- | --- | --- |
| K.QECLNNIGDASSLIR.A | 2 | 1690.831 | 3.153 | 0.276 |

+1 charge

```
		      B   	       Y
  1	 Q 	   129.07	        -  	 15
  2	 E 	   258.11	  1561.90	 14
  3	 C 	   418.27	  1432.86	 13
  4	 L 	   531.36	  1272.69	 12
  5	 N 	   645.40	  1159.61	 11
  6	 N 	   759.44	  1045.56	 10
  7	 I 	   872.53	   931.52	  9
  8	 G 	   929.55	   818.44	  8
  9	 D 	  1044.58	   761.42	  7
 10	 A 	  1115.61	   646.39	  6
 11	 S 	  1202.65	   575.35	  5
 12	 S 	  1289.68	   488.32	  4
 13	 L 	  1402.76	   401.29	  3
 14	 I 	  1515.85	   288.20	  2
 15	 R 	        -  	   175.12	  1  _
+2 charge
		      B   	       Y
  1	 Q 	    65.04	        -  	 15
  2	 E 	   129.56	   781.45	 14
  3	 C 	   209.64	   716.93	 13
  4	 L 	   266.18	   636.85	 12
  5	 N 	   323.20	   580.31	 11
  6	 N 	   380.23	   523.29	 10
  7	 I 	   436.77	   466.26	  9
  8	 G 	   465.28	   409.72	  8
  9	 D 	   522.79	   381.21	  7
 10	 A 	   558.31	   323.70	  6
 11	 S 	   601.83	   288.18	  5
 12	 S 	   645.34	   244.66	  4
 13	 L 	   701.88	   201.15	  3
 14	 I 	   758.43	   144.61	  2
 15	 R 	        -  	    88.06	  1  _
```

**6. IPI00003091 Isoform 1 of Protein phosphatase 1 regulatory subunit 3A**

| Sequence | Charge | M+H+ | XCorr | DeltaCn |
| --- | --- | --- | --- | --- |
| K.EQEPEPVKPWKEVPNR.Q | 2 | 1963.183 | 3.384 | 0.306 |

+1 charge

```
		      B   	       Y
  1	 E 	   130.05	        -  	 16
  2	 Q 	   258.11	  1832.97	 15
  3	 E 	   387.15	  1704.91	 14
  4	 P 	   484.20	  1575.86	 13
  5	 E 	   613.25	  1478.81	 12
  6	 P 	   710.30	  1349.77	 11
  7	 V 	   809.37	  1252.72	 10
  8	 K 	   937.46	  1153.65	  9
  9	 P 	  1034.52	  1025.55	  8
 10	 W 	  1220.59	   928.50	  7
 11	 K 	  1348.69	   742.42	  6
 12	 E 	  1477.73	   614.33	  5
 13	 V 	  1576.80	   485.28	  4
 14	 P 	  1673.85	   386.21	  3
 15	 N 	  1787.90	   289.16	  2
 16	 R 	        -  	   175.12	  1  _
+2 charge
		      B   	       Y
  1	 E 	    65.53	        -  	 16
  2	 Q 	   129.56	   916.99	 15
  3	 E 	   194.08	   852.96	 14
  4	 P 	   242.61	   788.44	 13
  5	 E 	   307.13	   739.91	 12
  6	 P 	   355.65	   675.39	 11
  7	 V 	   405.19	   626.86	 10
  8	 K 	   469.23	   577.33	  9
  9	 P 	   517.76	   513.28	  8
 10	 W 	   610.80	   464.75	  7
 11	 K 	   674.85	   371.71	  6
 12	 E 	   739.37	   307.67	  5
 13	 V 	   788.90	   243.15	  4
 14	 P 	   837.43	   193.61	  3
 15	 N 	   894.45	   145.08	  2
 16	 R 	        -  	    88.06	  1  _
```

**7. IPI00216171 Gamma-enolase**

| Sequence | Charge | M+H+ | XCorr | DeltaCn |
| --- | --- | --- | --- | --- |
| R.AAVPSGASTGIYEALELR.D | 2 | 1806.01 | 4.318 | 0.598 |

+1 charge

```
		      B   	       Y
  1	 A 	    72.04	        -  	 18
  2	 A 	   143.08	  1733.91	 17
  3	 V 	   242.15	  1662.87	 16
  4	 P 	   339.20	  1563.80	 15
  5	 S 	   426.23	  1466.75	 14
  6	 G 	   483.26	  1379.72	 13
  7	 A 	   554.29	  1322.70	 12
  8	 S 	   641.33	  1251.66	 11
  9	 T 	   742.37	  1164.63	 10
 10	 G 	   799.39	  1063.58	  9
 11	 I 	   912.48	  1006.56	  8
 12	 Y 	  1075.54	   893.47	  7
 13	 E 	  1204.58	   730.41	  6
 14	 A 	  1275.62	   601.37	  5
 15	 L 	  1388.71	   530.33	  4
 16	 E 	  1517.75	   417.25	  3
 17	 L 	  1630.83	   288.20	  2
 18	 R 	        -  	   175.12	  1  _
+2 charge
		      B   	       Y
  1	 A 	    36.53	        -  	 18
  2	 A 	    72.04	   867.46	 17
  3	 V 	   121.58	   831.94	 16
  4	 P 	   170.10	   782.40	 15
  5	 S 	   213.62	   733.88	 14
  6	 G 	   242.13	   690.36	 13
  7	 A 	   277.65	   661.85	 12
  8	 S 	   321.17	   626.33	 11
  9	 T 	   371.69	   582.82	 10
 10	 G 	   400.20	   532.29	  9
 11	 I 	   456.74	   503.78	  8
 12	 Y 	   538.27	   447.24	  7
 13	 E 	   602.80	   365.71	  6
 14	 A 	   638.31	   301.19	  5
 15	 L 	   694.86	   265.67	  4
 16	 E 	   759.38	   209.13	  3
 17	 L 	   815.92	   144.61	  2
 18	 R 	        -  	    88.06	  1  _
```

**8. IPI00290085 Cadherin-2**

| Sequence | Charge | M+H+ | XCorr | DeltaCn |
| --- | --- | --- | --- | --- |
| R.FAIQTDPNSNDGLVTVVKPIDFETNR.M | 3 | 2892.17 | 3.452 | 0.406 |

+1 charge

```
		      B   	       Y
  1	 F 	   148.08	        -  	 26
  2	 A 	   219.11	  2743.39	 25
  3	 I 	   332.20	  2672.35	 24
  4	 Q 	   460.26	  2559.27	 23
  5	 T 	   561.30	  2431.21	 22
  6	 D 	   676.33	  2330.16	 21
  7	 P 	   773.38	  2215.14	 20
  8	 N 	   887.43	  2118.08	 19
  9	 S 	   974.46	  2004.04	 18
 10	 N 	  1088.50	  1917.01	 17
 11	 D 	  1203.53	  1802.96	 16
 12	 G 	  1260.55	  1687.94	 15
 13	 L 	  1373.63	  1630.92	 14
 14	 V 	  1472.70	  1517.83	 13
 15	 T 	  1573.75	  1418.76	 12
 16	 V 	  1672.82	  1317.72	 11
 17	 V 	  1771.89	  1218.65	 10
 18	 K 	  1899.98	  1119.58	  9
 19	 P 	  1997.03	   991.48	  8
 20	 I 	  2110.12	   894.43	  7
 21	 D 	  2225.14	   781.35	  6
 22	 F 	  2372.21	   666.32	  5
 23	 E 	  2501.26	   519.25	  4
 24	 T 	  2602.30	   390.21	  3
 25	 N 	  2716.35	   289.16	  2
 26	 R 	        -  	   175.12	  1  _


+2 charge
		      B   	       Y
  1	 F 	    74.54	        -  	 26
  2	 A 	   110.06	  1372.20	 25
  3	 I 	   166.60	  1336.68	 24
  4	 Q 	   230.63	  1280.14	 23
  5	 T 	   281.16	  1216.11	 22
  6	 D 	   338.67	  1165.58	 21
  7	 P 	   387.20	  1108.07	 20
  8	 N 	   444.22	  1059.54	 19
  9	 S 	   487.73	  1002.52	 18
 10	 N 	   544.75	   959.01	 17
 11	 D 	   602.27	   901.99	 16
 12	 G 	   630.78	   844.47	 15
 13	 L 	   687.32	   815.96	 14
 14	 V 	   736.85	   759.42	 13
 15	 T 	   787.38	   709.89	 12
 16	 V 	   836.91	   659.36	 11
 17	 V 	   886.45	   609.83	 10
 18	 K 	   950.49	   560.29	  9
 19	 P 	   999.02	   496.25	  8
 20	 I 	  1055.56	   447.72	  7
 21	 D 	  1113.08	   391.18	  6
 22	 F 	  1186.61	   333.66	  5
 23	 E 	  1251.13	   260.13	  4
 24	 T 	  1301.66	   195.61	  3
 25	 N 	  1358.68	   145.08	  2
 26	 R 	        -  	    88.06	  1  _


+3 charge
		      B   	       Y
  1	 F 	    50.03	        -  	 26
  2	 A 	    73.71	   915.13	 25
  3	 I 	   111.40	   891.46	 24
  4	 Q 	   154.09	   853.76	 23
  5	 T 	   187.77	   811.07	 22
  6	 D 	   226.11	   777.39	 21
  7	 P 	   258.47	   739.05	 20
  8	 N 	   296.48	   706.70	 19
  9	 S 	   325.49	   668.68	 18
 10	 N 	   363.51	   639.67	 17
 11	 D 	   401.85	   601.66	 16
 12	 G 	   420.85	   563.32	 15
 13	 L 	   458.55	   544.31	 14
 14	 V 	   491.57	   506.62	 13
 15	 T 	   525.25	   473.59	 12
 16	 V 	   558.28	   439.91	 11
 17	 V 	   591.30	   406.89	 10
 18	 K 	   634.00	   373.86	  9
 19	 P 	   666.35	   331.17	  8
 20	 I 	   704.04	   298.82	  7
 21	 D 	   742.39	   261.12	  6
 22	 F 	   791.41	   222.78	  5
 23	 E 	   834.42	   173.76	  4
 24	 T 	   868.11	   130.74	  3
 25	 N 	   906.12	    97.06	  2
 26	 R 	        -  	    59.04	  1  _
```

**9. IPI0004371 Isoform 1 of Ligand-dependent nuclear receptor corepressor-like protein**

| Sequence | Charge | M+H+ | XCorr | DeltaCn |
| --- | --- | --- | --- | --- |
| K.EKNESLQYETSNPTVQLKIPQLR.V | 3 | 2717.028 | 3.621 | 0.25 |

+1 charge

```
		      B   	       Y
  1	 E 	   130.05	        -  	 23
  2	 K 	   258.14	  2586.39	 22
  3	 N 	   372.19	  2458.29	 21
  4	 E 	   501.23	  2344.25	 20
  5	 S 	   588.26	  2215.21	 19
  6	 L 	   701.35	  2128.18	 18
  7	 Q 	   829.41	  2015.09	 17
  8	 Y 	   992.47	  1887.03	 16
  9	 E 	  1121.51	  1723.97	 15
 10	 T 	  1222.56	  1594.93	 14
 11	 S 	  1309.59	  1493.88	 13
 12	 N 	  1423.63	  1406.85	 12
 13	 P 	  1520.69	  1292.80	 11
 14	 T 	  1621.73	  1195.75	 10
 15	 V 	  1720.80	  1094.70	  9
 16	 Q 	  1848.86	   995.64	  8
 17	 L 	  1961.95	   867.58	  7
 18	 K 	  2090.04	   754.49	  6
 19	 I 	  2203.12	   626.40	  5
 20	 P 	  2300.18	   513.31	  4
 21	 Q 	  2428.24	   416.26	  3
 22	 L 	  2541.32	   288.20	  2
 23	 R 	        -  	   175.12	  1  _


+2 charge
		      B   	       Y
  1	 E 	    65.53	        -  	 23
  2	 K 	   129.58	  1293.70	 22
  3	 N 	   186.60	  1229.65	 21
  4	 E 	   251.12	  1172.63	 20
  5	 S 	   294.63	  1108.11	 19
  6	 L 	   351.18	  1064.59	 18
  7	 Q 	   415.21	  1008.05	 17
  8	 Y 	   496.74	   944.02	 16
  9	 E 	   561.26	   862.49	 15
 10	 T 	   611.78	   797.97	 14
 11	 S 	   655.30	   747.44	 13
 12	 N 	   712.32	   703.93	 12
 13	 P 	   760.85	   646.91	 11
 14	 T 	   811.37	   598.38	 10
 15	 V 	   860.90	   547.86	  9
 16	 Q 	   924.93	   498.32	  8
 17	 L 	   981.48	   434.29	  7
 18	 K 	  1045.52	   377.75	  6
 19	 I 	  1102.07	   313.70	  5
 20	 P 	  1150.59	   257.16	  4
 21	 Q 	  1214.62	   208.63	  3
 22	 L 	  1271.16	   144.61	  2
 23	 R 	        -  	    88.06	  1  _
+3 charge
		      B   	       Y
  1	 E 	    44.02	        -  	 23
  2	 K 	    86.72	   862.80	 22
  3	 N 	   124.73	   820.10	 21
  4	 E 	   167.75	   782.09	 20
  5	 S 	   196.76	   739.07	 19
  6	 L 	   234.45	   710.06	 18
  7	 Q 	   277.14	   672.37	 17
  8	 Y 	   331.49	   629.68	 16
  9	 E 	   374.51	   575.33	 15
 10	 T 	   408.19	   532.31	 14
 11	 S 	   437.20	   498.63	 13
 12	 N 	   475.22	   469.62	 12
 13	 P 	   507.57	   431.61	 11
 14	 T 	   541.25	   399.26	 10
 15	 V 	   574.27	   365.57	  9
 16	 Q 	   616.96	   332.55	  8
 17	 L 	   654.65	   289.86	  7
 18	 K 	   697.35	   252.17	  6
 19	 I 	   735.05	   209.47	  5
 20	 P 	   767.40	   171.78	  4
 21	 Q 	   810.08	   139.43	  3
 22	 L 	   847.78	    96.74	  2
 23	 R 	        -  	    59.04	  1  _
```

**10. IPI00410585 Isoform 1 of Crumbs homolog 2**

| Sequence | Charge | M+H+ | XCorr | DeltaCn |
| --- | --- | --- | --- | --- |
| R.SDPALYGGVQAAFPGAFSFR.H | 2 | 2059.269 | 3.145 | 0.445 |

+1 charge

```
		      B   	       Y
  1	 S 	    88.04	        -  	 20
  2	 D 	   203.07	  1970.98	 19
  3	 P 	   300.12	  1855.95	 18
  4	 A 	   371.16	  1758.90	 17
  5	 L 	   484.24	  1687.86	 16
  6	 Y 	   647.30	  1574.78	 15
  7	 G 	   704.32	  1411.71	 14
  8	 G 	   761.35	  1354.69	 13
  9	 V 	   860.41	  1297.67	 12
 10	 Q 	   988.47	  1198.60	 11
 11	 A 	  1059.51	  1070.54	 10
 12	 A 	  1130.55	   999.50	  9
 13	 F 	  1277.62	   928.47	  8
 14	 P 	  1374.67	   781.40	  7
 15	 G 	  1431.69	   684.35	  6
 16	 A 	  1502.73	   627.32	  5
 17	 F 	  1649.80	   556.29	  4
 18	 S 	  1736.83	   409.22	  3
 19	 F 	  1883.90	   322.19	  2
 20	 R 	        -  	   175.12	  1  _
+2 charge
		      B   	       Y
  1	 S 	    44.52	        -  	 20
  2	 D 	   102.04	   985.99	 19
  3	 P 	   150.56	   928.48	 18
  4	 A 	   186.08	   879.95	 17
  5	 L 	   242.62	   844.43	 16
  6	 Y 	   324.16	   787.89	 15
  7	 G 	   352.67	   706.36	 14
  8	 G 	   381.18	   677.85	 13
  9	 V 	   430.71	   649.34	 12
 10	 Q 	   494.74	   599.80	 11
 11	 A 	   530.26	   535.77	 10
 12	 A 	   565.78	   500.26	  9
 13	 F 	   639.31	   464.74	  8
 14	 P 	   687.84	   391.20	  7
 15	 G 	   716.35	   342.68	  6
 16	 A 	   751.87	   314.17	  5
 17	 F 	   825.40	   278.65	  4
 18	 S 	   868.92	   205.11	  3
 19	 F 	   942.45	   161.60	  2
 20	 R 	        -  	    88.06	  1  _
```

**11. IPI00017601 Ceruloplasmin**

| Sequence | Charge | M+H+ | XCorr | DeltaCn |
| --- | --- | --- | --- | --- |
| K.MYYSAVDPTKDIFTGLIGPMKICK.K | 3 | 2750.263 | 4.864 | 0.42 |

+1 charge

```
		      B   	       Y
  1	 M 	   132.05	        -  	 24
  2	 Y 	   295.11	  2617.48	 23
  3	 Y 	   458.17	  2454.41	 22
  4	 S 	   545.21	  2291.35	 21
  5	 A 	   616.24	  2204.32	 20
  6	 V 	   715.31	  2133.28	 19
  7	 D 	   830.34	  2034.21	 18
  8	 P 	   927.39	  1919.18	 17
  9	 T 	  1028.44	  1822.13	 16
 10	 K 	  1156.53	  1721.08	 15
 11	 D 	  1271.56	  1592.99	 14
 12	 I 	  1384.65	  1477.96	 13
 13	 F 	  1531.71	  1364.88	 12
 14	 T 	  1632.76	  1217.81	 11
 15	 G 	  1689.78	  1116.76	 10
 16	 L 	  1802.87	  1059.74	  9
 17	 I 	  1915.95	   946.66	  8
 18	 G 	  1972.97	   833.57	  7
 19	 P 	  2070.03	   776.55	  6
 20	 M 	  2201.07	   679.50	  5
 21	 K 	  2329.16	   548.46	  4
 22	 I 	  2442.24	   420.36	  3
 23	 C 	  2602.41	   307.28	  2
 24	 K 	        -  	   147.11	  1  _


+2 charge
		      B   	       Y
  1	 M 	    66.53	        -  	 24
  2	 Y 	   148.06	  1309.24	 23
  3	 Y 	   229.59	  1227.71	 22
  4	 S 	   273.11	  1146.18	 21
  5	 A 	   308.63	  1102.66	 20
  6	 V 	   358.16	  1067.14	 19
  7	 D 	   415.67	  1017.61	 18
  8	 P 	   464.20	   960.10	 17
  9	 T 	   514.72	   911.57	 16
 10	 K 	   578.77	   861.05	 15
 11	 D 	   636.28	   797.00	 14
 12	 I 	   692.83	   739.48	 13
 13	 F 	   766.36	   682.94	 12
 14	 T 	   816.88	   609.41	 11
 15	 G 	   845.40	   558.88	 10
 16	 L 	   901.94	   530.37	  9
 17	 I 	   958.48	   473.83	  8
 18	 G 	   986.99	   417.29	  7
 19	 P 	  1035.52	   388.78	  6
 20	 M 	  1101.04	   340.25	  5
 21	 K 	  1165.08	   274.73	  4
 22	 I 	  1221.63	   210.68	  3
 23	 C 	  1301.71	   154.14	  2
 24	 K 	        -  	    74.06	  1  _


+3 charge
		      B   	       Y
  1	 M 	    44.69	        -  	 24
  2	 Y 	    99.04	   873.16	 23
  3	 Y 	   153.40	   818.81	 22
  4	 S 	   182.41	   764.45	 21
  5	 A 	   206.09	   735.44	 20
  6	 V 	   239.11	   711.76	 19
  7	 D 	   277.45	   678.74	 18
  8	 P 	   309.80	   640.40	 17
  9	 T 	   343.48	   608.05	 16
 10	 K 	   386.18	   574.37	 15
 11	 D 	   424.53	   531.67	 14
 12	 I 	   462.22	   493.33	 13
 13	 F 	   511.24	   455.63	 12
 14	 T 	   544.93	   406.61	 11
 15	 G 	   563.93	   372.93	 10
 16	 L 	   601.63	   353.92	  9
 17	 I 	   639.32	   316.22	  8
 18	 G 	   658.33	   278.53	  7
 19	 P 	   690.68	   259.52	  6
 20	 M 	   734.36	   227.17	  5
 21	 K 	   777.06	   183.49	  4
 22	 I 	   814.75	   140.79	  3
 23	 C 	   868.14	   103.10	  2
 24	 K 	        -  	    49.71	  1  _
```

**12. IPI00010737 Thrombomodulin**

| Sequence | Charge | M+H+ | XCorr | DeltaCn |
| --- | --- | --- | --- | --- |
| R.SSVAADVISLLLNGDGGVGR.R | 2 | 1901.111 | 4.958 | 0.613 |

+1 charge

```
		      B   	       Y
  1	 S 	    88.04	        -  	 20
  2	 S 	   175.07	  1812.98	 19
  3	 V 	   274.14	  1725.95	 18
  4	 A 	   345.18	  1626.88	 17
  5	 A 	   416.21	  1555.84	 16
  6	 D 	   531.24	  1484.81	 15
  7	 V 	   630.31	  1369.78	 14
  8	 I 	   743.39	  1270.71	 13
  9	 S 	   830.43	  1157.63	 12
 10	 L 	   943.51	  1070.60	 11
 11	 L 	  1056.59	   957.51	 10
 12	 L 	  1169.68	   844.43	  9
 13	 N 	  1283.72	   731.34	  8
 14	 G 	  1340.74	   617.30	  7
 15	 D 	  1455.77	   560.28	  6
 16	 G 	  1512.79	   445.25	  5
 17	 G 	  1569.81	   388.23	  4
 18	 V 	  1668.88	   331.21	  3
 19	 G 	  1725.90	   232.14	  2
 20	 R 	        -  	   175.12	  1  _
+2 charge
		      B   	       Y
  1	 S 	    44.52	        -  	 20
  2	 S 	    88.04	   906.99	 19
  3	 V 	   137.57	   863.48	 18
  4	 A 	   173.09	   813.94	 17
  5	 A 	   208.61	   778.43	 16
  6	 D 	   266.12	   742.91	 15
  7	 V 	   315.66	   685.39	 14
  8	 I 	   372.20	   635.86	 13
  9	 S 	   415.72	   579.32	 12
 10	 L 	   472.26	   535.80	 11
 11	 L 	   528.80	   479.26	 10
 12	 L 	   585.34	   422.72	  9
 13	 N 	   642.36	   366.18	  8
 14	 G 	   670.87	   309.15	  7
 15	 D 	   728.39	   280.64	  6
 16	 G 	   756.90	   223.13	  5
 17	 G 	   785.41	   194.62	  4
 18	 V 	   834.94	   166.11	  3
 19	 G 	   863.45	   116.57	  2
 20	 R 	        -  	    88.06	  1  _
```

**13. IPI00953695 Ig kappa chain V-II region GM607 (Fragment)**

| Sequence | Charge | M+H+ | XCorr | DeltaCn |
| --- | --- | --- | --- | --- |
| R.ASGVPDRFSGSGSGTDFTLK.I | 2 | 1987.116 | 3.985 | 0.487 |

+1 charge

```
		      B   	       Y
  1	 A 	    72.04	        -  	 20
  2	 S 	   159.08	  1914.92	 19
  3	 G 	   216.10	  1827.89	 18
  4	 V 	   315.17	  1770.87	 17
  5	 P 	   412.22	  1671.80	 16
  6	 D 	   527.25	  1574.74	 15
  7	 R 	   683.35	  1459.72	 14
  8	 F 	   830.42	  1303.62	 13
  9	 S 	   917.45	  1156.55	 12
 10	 G 	   974.47	  1069.52	 11
 11	 S 	  1061.50	  1012.49	 10
 12	 G 	  1118.52	   925.46	  9
 13	 S 	  1205.55	   868.44	  8
 14	 G 	  1262.58	   781.41	  7
 15	 T 	  1363.62	   724.39	  6
 16	 D 	  1478.65	   623.34	  5
 17	 F 	  1625.72	   508.31	  4
 18	 T 	  1726.77	   361.24	  3
 19	 L 	  1839.85	   260.20	  2
 20	 K 	        -  	   147.11	  1  _
+2 charge
		      B   	       Y
  1	 A 	    36.53	        -  	 20
  2	 S 	    80.04	   957.96	 19
  3	 G 	   108.55	   914.45	 18
  4	 V 	   158.09	   885.94	 17
  5	 P 	   206.61	   836.40	 16
  6	 D 	   264.13	   787.88	 15
  7	 R 	   342.18	   730.36	 14
  8	 F 	   415.71	   652.31	 13
  9	 S 	   459.23	   578.78	 12
 10	 G 	   487.74	   535.26	 11
 11	 S 	   531.25	   506.75	 10
 12	 G 	   559.76	   463.23	  9
 13	 S 	   603.28	   434.72	  8
 14	 G 	   631.79	   391.21	  7
 15	 T 	   682.32	   362.70	  6
 16	 D 	   739.83	   312.17	  5
 17	 F 	   813.36	   254.66	  4
 18	 T 	   863.89	   181.13	  3
 19	 L 	   920.43	   130.60	  2
 20	 K 	        -  	    74.06	  1  _
```

**14. IPI00011694 Trypsin-1**

| Sequence | Charge | M+H+ | XCorr | DeltaCn |
| --- | --- | --- | --- | --- |
| K.TLNNDIMLIK.L | 2 | 1175.424 | 3.302 | 0.326 |

+1 charge

```
		      B   	       Y
  1	 T 	   102.05	        -  	 10
  2	 L 	   215.14	  1073.60	  9
  3	 N 	   329.18	   960.52	  8
  4	 N 	   443.22	   846.48	  7
  5	 D 	   558.25	   732.43	  6
  6	 I 	   671.34	   617.41	  5
  7	 M 	   802.38	   504.32	  4
  8	 L 	   915.46	   373.28	  3
  9	 I 	  1028.54	   260.20	  2
 10	 K 	        -  	   147.11	  1  _
+2 charge
		      B   	       Y
  1	 T 	    51.53	        -  	 10
  2	 L 	   108.07	   537.30	  9
  3	 N 	   165.09	   480.76	  8
  4	 N 	   222.12	   423.74	  7
  5	 D 	   279.63	   366.72	  6
  6	 I 	   336.17	   309.21	  5
  7	 M 	   401.69	   252.66	  4
  8	 L 	   458.23	   187.14	  3
  9	 I 	   514.78	   130.60	  2
 10	 K 	        -  	    74.06	  1  _
```

**15. IPI00217223 Multifunctional protein ADE2**

| Sequence | Charge | M+H+ | XCorr | DeltaCn |
| --- | --- | --- | --- | --- |
| - .MLPPGRQDLSSASLPGAVAALSPLR.I | 3 | 2505.922 | 3.5 | 0.37 |

+1 charge

```
		      B   	       Y
  1	 M 	   132.05	        -  	 25
  2	 L 	   245.13	  2373.32	 24
  3	 P 	   342.18	  2260.24	 23
  4	 P 	   439.24	  2163.19	 22
  5	 G 	   496.26	  2066.14	 21
  6	 R 	   652.36	  2009.11	 20
  7	 Q 	   780.42	  1853.01	 19
  8	 D 	   895.45	  1724.95	 18
  9	 L 	  1008.53	  1609.93	 17
 10	 S 	  1095.56	  1496.84	 16
 11	 S 	  1182.59	  1409.81	 15
 12	 A 	  1253.63	  1322.78	 14
 13	 S 	  1340.66	  1251.74	 13
 14	 L 	  1453.75	  1164.71	 12
 15	 P 	  1550.80	  1051.63	 11
 16	 G 	  1607.82	   954.57	 10
 17	 A 	  1678.86	   897.55	  9
 18	 V 	  1777.93	   826.51	  8
 19	 A 	  1848.96	   727.45	  7
 20	 A 	  1920.00	   656.41	  6
 21	 L 	  2033.08	   585.37	  5
 22	 S 	  2120.12	   472.29	  4
 23	 P 	  2217.17	   385.26	  3
 24	 L 	  2330.25	   288.20	  2
 25	 R 	        -  	   175.12	  1  _


+2 charge
		      B   	       Y
  1	 M 	    66.53	        -  	 25
  2	 L 	   123.07	  1187.17	 24
  3	 P 	   171.60	  1130.62	 23
  4	 P 	   220.12	  1082.10	 22
  5	 G 	   248.63	  1033.57	 21
  6	 R 	   326.68	  1005.06	 20
  7	 Q 	   390.71	   927.01	 19
  8	 D 	   448.23	   862.98	 18
  9	 L 	   504.77	   805.47	 17
 10	 S 	   548.28	   748.93	 16
 11	 S 	   591.80	   705.41	 15
 12	 A 	   627.32	   661.89	 14
 13	 S 	   670.83	   626.37	 13
 14	 L 	   727.38	   582.86	 12
 15	 P 	   775.90	   526.32	 11
 16	 G 	   804.41	   477.79	 10
 17	 A 	   839.93	   449.28	  9
 18	 V 	   889.47	   413.76	  8
 19	 A 	   924.99	   364.23	  7
 20	 A 	   960.50	   328.71	  6
 21	 L 	  1017.05	   293.19	  5
 22	 S 	  1060.56	   236.65	  4
 23	 P 	  1109.09	   193.13	  3
 24	 L 	  1165.63	   144.61	  2
 25	 R 	        -  	    88.06	  1  _


+3 charge
		      B   	       Y
  1	 M 	    44.69	        -  	 25
  2	 L 	    82.38	   791.78	 24
  3	 P 	   114.73	   754.09	 23
  4	 P 	   147.08	   721.73	 22
  5	 G 	   166.09	   689.38	 21
  6	 R 	   218.12	   670.38	 20
  7	 Q 	   260.81	   618.34	 19
  8	 D 	   299.15	   575.66	 18
  9	 L 	   336.85	   537.31	 17
 10	 S 	   365.86	   499.62	 16
 11	 S 	   394.87	   470.61	 15
 12	 A 	   418.55	   441.60	 14
 13	 S 	   447.56	   417.92	 13
 14	 L 	   485.25	   388.91	 12
 15	 P 	   517.60	   351.21	 11
 16	 G 	   536.61	   318.86	 10
 17	 A 	   560.29	   299.86	  9
 18	 V 	   593.31	   276.18	  8
 19	 A 	   616.99	   243.15	  7
 20	 A 	   640.67	   219.47	  6
 21	 L 	   678.37	   195.80	  5
 22	 S 	   707.38	   158.10	  4
 23	 P 	   739.73	   129.09	  3
 24	 L 	   777.42	    96.74	  2
 25	 R 	        -  	    59.04	  1  _
```

**16. IPI00816799 Rheumatoid factor D5 light chain (Fragment)**

| Sequence | Charge | M+H+ | XCorr | DeltaCn |
| --- | --- | --- | --- | --- |
| - .EIVLTQSPATLSLSPGER.A | 2 | 1899.135 | 5.263 | 0.482 |

+1 charge

```
		      B   	       Y
  1	 E 	   130.05	        -  	 18
  2	 I 	   243.13	  1768.98	 17
  3	 V 	   342.20	  1655.90	 16
  4	 L 	   455.29	  1556.83	 15
  5	 T 	   556.33	  1443.74	 14
  6	 Q 	   684.39	  1342.70	 13
  7	 S 	   771.42	  1214.64	 12
  8	 P 	   868.48	  1127.61	 11
  9	 A 	   939.51	  1030.55	 10
 10	 T 	  1040.56	   959.52	  9
 11	 L 	  1153.65	   858.47	  8
 12	 S 	  1240.68	   745.38	  7
 13	 L 	  1353.76	   658.35	  6
 14	 S 	  1440.79	   545.27	  5
 15	 P 	  1537.85	   458.24	  4
 16	 G 	  1594.87	   361.18	  3
 17	 E 	  1723.91	   304.16	  2
 18	 R 	        -  	   175.12	  1  _
+2 charge
		      B   	       Y
  1	 E 	    65.53	        -  	 18
  2	 I 	   122.07	   884.99	 17
  3	 V 	   171.60	   828.45	 16
  4	 L 	   228.15	   778.92	 15
  5	 T 	   278.67	   722.38	 14
  6	 Q 	   342.70	   671.85	 13
  7	 S 	   386.22	   607.82	 12
  8	 P 	   434.74	   564.31	 11
  9	 A 	   470.26	   515.78	 10
 10	 T 	   520.78	   480.26	  9
 11	 L 	   577.33	   429.74	  8
 12	 S 	   620.84	   373.20	  7
 13	 L 	   677.38	   329.68	  6
 14	 S 	   720.90	   273.14	  5
 15	 P 	   769.43	   229.62	  4
 16	 G 	   797.94	   181.10	  3
 17	 E 	   862.46	   152.58	  2
 18	 R 	        -  	    88.06	  1  _
```

**17. IPI00024046 cDNA FLJ52398, highly similar to Cadherin-13**

| Sequence | Charge | M+H+ | XCorr | DeltaCn |
| --- | --- | --- | --- | --- |
| R.MTAFDADDPATDNALLR.Y | 2 | 1837.989 | 5.774 | 0.567 |

+1 charge

```
		      B   	       Y
  1	 M 	   132.05	        -  	 17
  2	 T 	   233.10	  1705.80	 16
  3	 A 	   304.13	  1604.76	 15
  4	 F 	   451.20	  1533.72	 14
  5	 D 	   566.23	  1386.65	 13
  6	 A 	   637.27	  1271.62	 12
  7	 D 	   752.29	  1200.59	 11
  8	 D 	   867.32	  1085.56	 10
  9	 P 	   964.37	   970.53	  9
 10	 A 	  1035.41	   873.48	  8
 11	 T 	  1136.46	   802.44	  7
 12	 D 	  1251.48	   701.39	  6
 13	 N 	  1365.53	   586.37	  5
 14	 A 	  1436.56	   472.32	  4
 15	 L 	  1549.65	   401.29	  3
 16	 L 	  1662.73	   288.20	  2
 17	 R 	        -  	   175.12	  1  _
+2 charge
		      B   	       Y
  1	 M 	    66.53	        -  	 17
  2	 T 	   117.05	   853.41	 16
  3	 A 	   152.57	   802.88	 15
  4	 F 	   226.10	   767.36	 14
  5	 D 	   283.62	   693.83	 13
  6	 A 	   319.14	   636.31	 12
  7	 D 	   376.65	   600.80	 11
  8	 D 	   434.16	   543.28	 10
  9	 P 	   482.69	   485.77	  9
 10	 A 	   518.21	   437.24	  8
 11	 T 	   568.73	   401.72	  7
 12	 D 	   626.25	   351.20	  6
 13	 N 	   683.27	   293.69	  5
 14	 A 	   718.79	   236.67	  4
 15	 L 	   775.33	   201.15	  3
 16	 L 	   831.87	   144.61	  2
 17	 R 	        -  	    88.06	  1  _
```

**18. IPI00008787 Alpha-N-acetylglucosaminidase**

| Sequence | Charge | M+H+ | XCorr | DeltaCn |
| --- | --- | --- | --- | --- |
| R.LLGPGPAADFSVSVER.A | 2 | 1615.812 | 4.671 | 0.576 |

+1 charge

```
		      B   	       Y
  1	 L 	   114.09	        -  	 16
  2	 L 	   227.18	  1501.76	 15
  3	 G 	   284.20	  1388.68	 14
  4	 P 	   381.25	  1331.66	 13
  5	 G 	   438.27	  1234.61	 12
  6	 P 	   535.32	  1177.58	 11
  7	 A 	   606.36	  1080.53	 10
  8	 A 	   677.40	  1009.49	  9
  9	 D 	   792.43	   938.46	  8
 10	 F 	   939.49	   823.43	  7
 11	 S 	  1026.53	   676.36	  6
 12	 V 	  1125.59	   589.33	  5
 13	 S 	  1212.63	   490.26	  4
 14	 V 	  1311.69	   403.23	  3
 15	 E 	  1440.74	   304.16	  2
 16	 R 	        -  	   175.12	  1  _
+2 charge
		      B   	       Y
  1	 L 	    57.55	        -  	 16
  2	 L 	   114.09	   751.39	 15
  3	 G 	   142.60	   694.84	 14
  4	 P 	   191.13	   666.33	 13
  5	 G 	   219.64	   617.81	 12
  6	 P 	   268.17	   589.30	 11
  7	 A 	   303.68	   540.77	 10
  8	 A 	   339.20	   505.25	  9
  9	 D 	   396.72	   469.73	  8
 10	 F 	   470.25	   412.22	  7
 11	 S 	   513.77	   338.68	  6
 12	 V 	   563.30	   295.17	  5
 13	 S 	   606.82	   245.63	  4
 14	 V 	   656.35	   202.12	  3
 15	 E 	   720.87	   152.58	  2
 16	 R 	        -  	    88.06	  1  _
```

**19. IPI00022420 Retinol-binding protein 4**

| Sequence | Charge | M+H+ | XCorr | DeltaCn |
| --- | --- | --- | --- | --- |
| R.LLNLDGTCADSYSFVFSRDPNGLPPEAQK.I | 3 | 3213.493 | 4.707 | 0.515 |

+1 charge

```
		      B   	       Y
  1	 L 	   114.09	        -  	 29
  2	 L 	   227.18	  3098.59	 28
  3	 N 	   341.22	  2985.50	 27
  4	 L 	   454.30	  2871.46	 26
  5	 D 	   569.33	  2758.38	 25
  6	 G 	   626.35	  2643.35	 24
  7	 T 	   727.40	  2586.33	 23
  8	 C 	   887.56	  2485.28	 22
  9	 A 	   958.60	  2325.11	 21
 10	 D 	  1073.63	  2254.08	 20
 11	 S 	  1160.66	  2139.05	 19
 12	 Y 	  1323.72	  2052.02	 18
 13	 S 	  1410.76	  1888.96	 17
 14	 F 	  1557.82	  1801.92	 16
 15	 V 	  1656.89	  1654.85	 15
 16	 F 	  1803.96	  1555.79	 14
 17	 S 	  1890.99	  1408.72	 13
 18	 R 	  2047.09	  1321.69	 12
 19	 D 	  2162.12	  1165.58	 11
 20	 P 	  2259.17	  1050.56	 10
 21	 N 	  2373.22	   953.51	  9
 22	 G 	  2430.24	   839.46	  8
 23	 L 	  2543.32	   782.44	  7
 24	 P 	  2640.37	   669.36	  6
 25	 P 	  2737.43	   572.30	  5
 26	 E 	  2866.47	   475.25	  4
 27	 A 	  2937.51	   346.21	  3
 28	 Q 	  3065.57	   275.17	  2
 29	 K 	        -  	   147.11	  1  _


+2 charge
		      B   	       Y
  1	 L 	    57.55	        -  	 29
  2	 L 	   114.09	  1549.80	 28
  3	 N 	   171.11	  1493.26	 27
  4	 L 	   227.65	  1436.23	 26
  5	 D 	   285.17	  1379.69	 25
  6	 G 	   313.68	  1322.18	 24
  7	 T 	   364.20	  1293.67	 23
  8	 C 	   444.29	  1243.14	 22
  9	 A 	   479.80	  1163.06	 21
 10	 D 	   537.32	  1127.54	 20
 11	 S 	   580.83	  1070.03	 19
 12	 Y 	   662.37	  1026.51	 18
 13	 S 	   705.88	   944.98	 17
 14	 F 	   779.42	   901.47	 16
 15	 V 	   828.95	   827.93	 15
 16	 F 	   902.48	   778.40	 14
 17	 S 	   946.00	   704.86	 13
 18	 R 	  1024.05	   661.35	 12
 19	 D 	  1081.56	   583.30	 11
 20	 P 	  1130.09	   525.78	 10
 21	 N 	  1187.11	   477.26	  9
 22	 G 	  1215.62	   420.23	  8
 23	 L 	  1272.16	   391.72	  7
 24	 P 	  1320.69	   335.18	  6
 25	 P 	  1369.22	   286.66	  5
 26	 E 	  1433.74	   238.13	  4
 27	 A 	  1469.26	   173.61	  3
 28	 Q 	  1533.29	   138.09	  2
 29	 K 	        -  	    74.06	  1  _


+3 charge
		      B   	       Y
  1	 L 	    38.70	        -  	 29
  2	 L 	    76.40	  1033.53	 28
  3	 N 	   114.41	   995.84	 27
  4	 L 	   152.11	   957.82	 26
  5	 D 	   190.45	   920.13	 25
  6	 G 	   209.46	   881.79	 24
  7	 T 	   243.14	   862.78	 23
  8	 C 	   296.53	   829.10	 22
  9	 A 	   320.21	   775.71	 21
 10	 D 	   358.55	   752.03	 20
 11	 S 	   387.56	   713.69	 19
 12	 Y 	   441.91	   684.68	 18
 13	 S 	   470.92	   630.32	 17
 14	 F 	   519.95	   601.31	 16
 15	 V 	   552.97	   552.29	 15
 16	 F 	   601.99	   519.27	 14
 17	 S 	   631.00	   470.24	 13
 18	 R 	   683.04	   441.23	 12
 19	 D 	   721.38	   389.20	 11
 20	 P 	   753.73	   350.86	 10
 21	 N 	   791.74	   318.51	  9
 22	 G 	   810.75	   280.49	  8
 23	 L 	   848.45	   261.49	  7
 24	 P 	   880.80	   223.79	  6
 25	 P 	   913.15	   191.44	  5
 26	 E 	   956.16	   159.09	  4
 27	 A 	   979.84	   116.07	  3
 28	 Q 	  1022.53	    92.40	  2
 29	 K 	        -  	    49.71	  1  _
```

**20. IPI00012011 Cofilin-1**

| Sequence | Charge | M+H+ | XCorr | DeltaCn |
| --- | --- | --- | --- | --- |
| K.NIILEEGKEILVGDVGQTVDDPYATFVK.M | 3 | 3064.432 | 4.252 | 0.543 |

+1 charge

```
		      B   	       Y
  1	 N 	   115.05	        -  	 28
  2	 I 	   228.13	  2948.55	 27
  3	 I 	   341.22	  2835.47	 26
  4	 L 	   454.30	  2722.38	 25
  5	 E 	   583.34	  2609.30	 24
  6	 E 	   712.39	  2480.26	 23
  7	 G 	   769.41	  2351.21	 22
  8	 K 	   897.50	  2294.19	 21
  9	 E 	  1026.55	  2166.10	 20
 10	 I 	  1139.63	  2037.05	 19
 11	 L 	  1252.71	  1923.97	 18
 12	 V 	  1351.78	  1810.89	 17
 13	 G 	  1408.80	  1711.82	 16
 14	 D 	  1523.83	  1654.80	 15
 15	 V 	  1622.90	  1539.77	 14
 16	 G 	  1679.92	  1440.70	 13
 17	 Q 	  1807.98	  1383.68	 12
 18	 T 	  1909.03	  1255.62	 11
 19	 V 	  2008.10	  1154.57	 10
 20	 D 	  2123.12	  1055.50	  9
 21	 D 	  2238.15	   940.48	  8
 22	 P 	  2335.20	   825.45	  7
 23	 Y 	  2498.27	   728.40	  6
 24	 A 	  2569.30	   565.33	  5
 25	 T 	  2670.35	   494.30	  4
 26	 F 	  2817.42	   393.25	  3
 27	 V 	  2916.49	   246.18	  2
 28	 K 	        -  	   147.11	  1  _


+2 charge
		      B   	       Y
  1	 N 	    58.03	        -  	 28
  2	 I 	   114.57	  1474.78	 27
  3	 I 	   171.11	  1418.24	 26
  4	 L 	   227.65	  1361.69	 25
  5	 E 	   292.18	  1305.15	 24
  6	 E 	   356.70	  1240.63	 23
  7	 G 	   385.21	  1176.11	 22
  8	 K 	   449.26	  1147.60	 21
  9	 E 	   513.78	  1083.55	 20
 10	 I 	   570.32	  1019.03	 19
 11	 L 	   626.86	   962.49	 18
 12	 V 	   676.40	   905.95	 17
 13	 G 	   704.91	   856.41	 16
 14	 D 	   762.42	   827.90	 15
 15	 V 	   811.95	   770.39	 14
 16	 G 	   840.46	   720.85	 13
 17	 Q 	   904.49	   692.34	 12
 18	 T 	   955.02	   628.31	 11
 19	 V 	  1004.55	   577.79	 10
 20	 D 	  1062.07	   528.26	  9
 21	 D 	  1119.58	   470.74	  8
 22	 P 	  1168.11	   413.23	  7
 23	 Y 	  1249.64	   364.70	  6
 24	 A 	  1285.16	   283.17	  5
 25	 T 	  1335.68	   247.65	  4
 26	 F 	  1409.21	   197.13	  3
 27	 V 	  1458.75	   123.59	  2
 28	 K 	        -  	    74.06	  1  _


+3 charge
		      B   	       Y
  1	 N 	    39.02	        -  	 28
  2	 I 	    76.72	   983.52	 27
  3	 I 	   114.41	   945.83	 26
  4	 L 	   152.11	   908.13	 25
  5	 E 	   195.12	   870.44	 24
  6	 E 	   238.13	   827.42	 23
  7	 G 	   257.14	   784.41	 22
  8	 K 	   299.84	   765.40	 21
  9	 E 	   342.85	   722.70	 20
 10	 I 	   380.55	   679.69	 19
 11	 L 	   418.24	   641.99	 18
 12	 V 	   451.27	   604.30	 17
 13	 G 	   470.27	   571.28	 16
 14	 D 	   508.62	   552.27	 15
 15	 V 	   541.64	   513.93	 14
 16	 G 	   560.65	   480.91	 13
 17	 Q 	   603.33	   461.90	 12
 18	 T 	   637.01	   419.21	 11
 19	 V 	   670.04	   385.53	 10
 20	 D 	   708.38	   352.51	  9
 21	 D 	   746.72	   314.16	  8
 22	 P 	   779.07	   275.82	  7
 23	 Y 	   833.43	   243.47	  6
 24	 A 	   857.11	   189.12	  5
 25	 T 	   890.79	   165.44	  4
 26	 F 	   939.81	   131.75	  3
 27	 V 	   972.83	    82.73	  2
 28	 K 	        -  	    49.71	  1  _
```

**21. IPI00219018 Glyceraldehyde-3-phosphate dehydrogenase**

| Sequence | Charge | M+H+ | XCorr | DeltaCn |
| --- | --- | --- | --- | --- |
| K.VIHDNFGIVEGLMTTVHAITATQK.T | 3 | 2596.987 | 3.387 | 0.393 |

+1 charge

```
		      B   	       Y
  1	 V 	   100.08	        -  	 24
  2	 I 	   213.16	  2496.29	 23
  3	 H 	   350.22	  2383.21	 22
  4	 D 	   465.25	  2246.15	 21
  5	 N 	   579.29	  2131.12	 20
  6	 F 	   726.36	  2017.08	 19
  7	 G 	   783.38	  1870.01	 18
  8	 I 	   896.46	  1812.99	 17
  9	 V 	   995.53	  1699.90	 16
 10	 E 	  1124.57	  1600.84	 15
 11	 G 	  1181.59	  1471.79	 14
 12	 L 	  1294.68	  1414.77	 13
 13	 M 	  1425.72	  1301.69	 12
 14	 T 	  1526.77	  1170.65	 11
 15	 T 	  1627.81	  1069.60	 10
 16	 V 	  1726.88	   968.55	  9
 17	 H 	  1863.94	   869.48	  8
 18	 A 	  1934.98	   732.43	  7
 19	 I 	  2048.06	   661.39	  6
 20	 T 	  2149.11	   548.30	  5
 21	 A 	  2220.15	   447.26	  4
 22	 T 	  2321.20	   376.22	  3
 23	 Q 	  2449.25	   275.17	  2
 24	 K 	        -  	   147.11	  1  _


+2 charge
		      B   	       Y
  1	 V 	    50.54	        -  	 24
  2	 I 	   107.08	  1248.65	 23
  3	 H 	   175.61	  1192.11	 22
  4	 D 	   233.13	  1123.58	 21
  5	 N 	   290.15	  1066.06	 20
  6	 F 	   363.68	  1009.04	 19
  7	 G 	   392.19	   935.51	 18
  8	 I 	   448.73	   907.00	 17
  9	 V 	   498.27	   850.46	 16
 10	 E 	   562.79	   800.92	 15
 11	 G 	   591.30	   736.40	 14
 12	 L 	   647.84	   707.89	 13
 13	 M 	   713.36	   651.35	 12
 14	 T 	   763.89	   585.83	 11
 15	 T 	   814.41	   535.30	 10
 16	 V 	   863.95	   484.78	  9
 17	 H 	   932.47	   435.25	  8
 18	 A 	   967.99	   366.72	  7
 19	 I 	  1024.54	   331.20	  6
 20	 T 	  1075.06	   274.66	  5
 21	 A 	  1110.58	   224.13	  4
 22	 T 	  1161.10	   188.61	  3
 23	 Q 	  1225.13	   138.09	  2
 24	 K 	        -  	    74.06	  1  _


+3 charge
		      B   	       Y
  1	 V 	    34.03	        -  	 24
  2	 I 	    71.72	   832.77	 23
  3	 H 	   117.41	   795.07	 22
  4	 D 	   155.75	   749.39	 21
  5	 N 	   193.77	   711.05	 20
  6	 F 	   242.79	   673.03	 19
  7	 G 	   261.80	   624.01	 18
  8	 I 	   299.49	   605.00	 17
  9	 V 	   332.52	   567.31	 16
 10	 E 	   375.53	   534.28	 15
 11	 G 	   394.54	   491.27	 14
 12	 L 	   432.23	   472.26	 13
 13	 M 	   475.91	   434.57	 12
 14	 T 	   509.59	   390.89	 11
 15	 T 	   543.28	   357.20	 10
 16	 V 	   576.30	   323.52	  9
 17	 H 	   621.99	   290.50	  8
 18	 A 	   645.66	   244.81	  7
 19	 I 	   683.36	   221.13	  6
 20	 T 	   717.04	   183.44	  5
 21	 A 	   740.72	   149.76	  4
 22	 T 	   774.40	   126.08	  3
 23	 Q 	   817.09	    92.40	  2
 24	 K 	        -  	    49.71	  1  _
```

**22. IPI00382452 Isoform 1 of Charged multivesicular body protein 1a**

| Sequence | Charge | M+H+ | XCorr | DeltaCn |
| --- | --- | --- | --- | --- |
| K.ALDKALSTMDLQK.V | 2 | 1434.684 | 2.995 | 0.354 |

+1 charge

```
		      B   	       Y
  1	 A 	    72.04	        -  	 13
  2	 L 	   185.13	  1362.73	 12
  3	 D 	   300.16	  1249.65	 11
  4	 K 	   428.25	  1134.62	 10
  5	 A 	   499.29	  1006.52	  9
  6	 L 	   612.37	   935.49	  8
  7	 S 	   699.40	   822.40	  7
  8	 T 	   800.45	   735.37	  6
  9	 M 	   931.49	   634.32	  5
 10	 D 	  1046.52	   503.28	  4
 11	 L 	  1159.60	   388.26	  3
 12	 Q 	  1287.66	   275.17	  2
 13	 K 	        -  	   147.11	  1  _
+2 charge
		      B   	       Y
  1	 A 	    36.53	        -  	 13
  2	 L 	    93.07	   681.87	 12
  3	 D 	   150.58	   625.33	 11
  4	 K 	   214.63	   567.81	 10
  5	 A 	   250.15	   503.77	  9
  6	 L 	   306.69	   468.25	  8
  7	 S 	   350.21	   411.70	  7
  8	 T 	   400.73	   368.19	  6
  9	 M 	   466.25	   317.67	  5
 10	 D 	   523.76	   252.14	  4
 11	 L 	   580.31	   194.63	  3
 12	 Q 	   644.33	   138.09	  2
 13	 K 	        -  	    74.06	  1  _
```

**23. IPI00183968 tropomyosin 3 isoform 1**

| Sequence | Charge | M+H+ | XCorr | DeltaCn |
| --- | --- | --- | --- | --- |
| R.LATALQKLEEAEKAADESER.G | 3 | 2203.393 | 3.981 | 0.468 |

+1 charge

```
		      B   	       Y
  1	 L 	   114.09	        -  	 20
  2	 A 	   185.13	  2089.04	 19
  3	 T 	   286.18	  2018.00	 18
  4	 A 	   357.21	  1916.96	 17
  5	 L 	   470.30	  1845.92	 16
  6	 Q 	   598.36	  1732.83	 15
  7	 K 	   726.45	  1604.78	 14
  8	 L 	   839.53	  1476.68	 13
  9	 E 	   968.58	  1363.60	 12
 10	 E 	  1097.62	  1234.55	 11
 11	 A 	  1168.66	  1105.51	 10
 12	 E 	  1297.70	  1034.47	  9
 13	 K 	  1425.79	   905.43	  8
 14	 A 	  1496.83	   777.34	  7
 15	 A 	  1567.87	   706.30	  6
 16	 D 	  1682.90	   635.26	  5
 17	 E 	  1811.94	   520.24	  4
 18	 S 	  1898.97	   391.19	  3
 19	 E 	  2028.01	   304.16	  2
 20	 R 	        -  	   175.12	  1  _
+2 charge
		      B   	       Y
  1	 L 	    57.55	        -  	 20
  2	 A 	    93.07	  1045.02	 19
  3	 T 	   143.59	  1009.51	 18
  4	 A 	   179.11	   958.98	 17
  5	 L 	   235.65	   923.46	 16
  6	 Q 	   299.68	   866.92	 15
  7	 K 	   363.73	   802.89	 14
  8	 L 	   420.27	   738.84	 13
  9	 E 	   484.79	   682.30	 12
 10	 E 	   549.31	   617.78	 11
 11	 A 	   584.83	   553.26	 10
 12	 E 	   649.35	   517.74	  9
 13	 K 	   713.40	   453.22	  8
 14	 A 	   748.92	   389.17	  7
 15	 A 	   784.44	   353.65	  6
 16	 D 	   841.95	   318.14	  5
 17	 E 	   906.47	   260.62	  4
 18	 S 	   949.99	   196.10	  3
 19	 E 	  1014.51	   152.58	  2
 20	 R 	        -  	    88.06	  1  _

+3 charge
		      B   	       Y
  1	 L 	    38.70	        -  	 20
  2	 A 	    62.38	   697.02	 19
  3	 T 	    96.06	   673.34	 18
  4	 A 	   119.74	   639.66	 17
  5	 L 	   157.44	   615.98	 16
  6	 Q 	   200.12	   578.28	 15
  7	 K 	   242.82	   535.60	 14
  8	 L 	   280.52	   492.90	 13
  9	 E 	   323.53	   455.20	 12
 10	 E 	   366.54	   412.19	 11
 11	 A 	   390.22	   369.18	 10
 12	 E 	   433.24	   345.50	  9
 13	 K 	   475.94	   302.48	  8
 14	 A 	   499.62	   259.78	  7
 15	 A 	   523.29	   236.10	  6
 16	 D 	   561.64	   212.43	  5
 17	 E 	   604.65	   174.08	  4
 18	 S 	   633.66	   131.07	  3
 19	 E 	   676.68	   102.06	  2
 20	 R 	        -  	    59.04	  1  _
```

**24. IPI00019449 Non-secretory ribonuclease**

| Sequence | Charge | M+H+ | XCorr | DeltaCn |
| --- | --- | --- | --- | --- |
| R.YAQTPANMFYIVACDNR.D | 2 | 2035.263 | 5.121 | 0.584 |

+1 charge

```
		      B   	       Y
  1	 Y 	   164.07	        -  	 17
  2	 A 	   235.11	  1870.99	 16
  3	 Q 	   363.17	  1799.95	 15
  4	 T 	   464.21	  1671.90	 14
  5	 P 	   561.27	  1570.85	 13
  6	 A 	   632.30	  1473.80	 12
  7	 N 	   746.35	  1402.76	 11
  8	 M 	   877.39	  1288.72	 10
  9	 F 	  1024.46	  1157.68	  9
 10	 Y 	  1187.52	  1010.61	  8
 11	 I 	  1300.60	   847.54	  7
 12	 V 	  1399.67	   734.46	  6
 13	 A 	  1470.71	   635.39	  5
 14	 C 	  1630.87	   564.35	  4
 15	 D 	  1745.90	   404.19	  3
 16	 N 	  1859.94	   289.16	  2
 17	 R 	        -  	   175.12	  1  _
+2 charge
		      B   	       Y
  1	 Y 	    82.54	        -  	 17
  2	 A 	   118.06	   936.00	 16
  3	 Q 	   182.09	   900.48	 15
  4	 T 	   232.61	   836.45	 14
  5	 P 	   281.14	   785.93	 13
  6	 A 	   316.66	   737.40	 12
  7	 N 	   373.68	   701.88	 11
  8	 M 	   439.20	   644.86	 10
  9	 F 	   512.73	   579.34	  9
 10	 Y 	   594.26	   505.81	  8
 11	 I 	   650.81	   424.28	  7
 12	 V 	   700.34	   367.73	  6
 13	 A 	   735.86	   318.20	  5
 14	 C 	   815.94	   282.68	  4
 15	 D 	   873.45	   202.60	  3
 16	 N 	   930.48	   145.08	  2
 17	 R 	        -  	    88.06	  1  _
```

**25. IPI00219446 Phosphatidylethanolamine-binding protein 1**

| Sequence | Charge | M+H+ | XCorr | DeltaCn |
| --- | --- | --- | --- | --- |
| R.APVAGTCYQAEWDDYVPKLYEQLSGK.- | 3 | 2990.262 | 4.093 | 0.583 |

+1 charge

```
		      B   	       Y
  1	 A 	    72.04	        -  	 26
  2	 P 	   169.10	  2917.51	 25
  3	 V 	   268.17	  2820.45	 24
  4	 A 	   339.20	  2721.38	 23
  5	 G 	   396.22	  2650.35	 22
  6	 T 	   497.27	  2593.33	 21
  7	 C 	   657.44	  2492.28	 20
  8	 Y 	   820.50	  2332.11	 19
  9	 Q 	   948.56	  2169.05	 18
 10	 A 	  1019.60	  2040.99	 17
 11	 E 	  1148.64	  1969.95	 16
 12	 W 	  1334.72	  1840.91	 15
 13	 D 	  1449.74	  1654.83	 14
 14	 D 	  1564.77	  1539.81	 13
 15	 Y 	  1727.84	  1424.78	 12
 16	 V 	  1826.90	  1261.72	 11
 17	 P 	  1923.96	  1162.65	 10
 18	 K 	  2052.05	  1065.59	  9
 19	 L 	  2165.14	   937.50	  8
 20	 Y 	  2328.20	   824.41	  7
 21	 E 	  2457.24	   661.35	  6
 22	 Q 	  2585.30	   532.31	  5
 23	 L 	  2698.38	   404.25	  4
 24	 S 	  2785.42	   291.17	  3
 25	 G 	  2842.44	   204.13	  2
 26	 K 	        -  	   147.11	  1  _


+2 charge
		      B   	       Y
  1	 A 	    36.53	        -  	 26
  2	 P 	    85.05	  1459.26	 25
  3	 V 	   134.59	  1410.73	 24
  4	 A 	   170.10	  1361.20	 23
  5	 G 	   198.62	  1325.68	 22
  6	 T 	   249.14	  1297.17	 21
  7	 C 	   329.22	  1246.64	 20
  8	 Y 	   410.75	  1166.56	 19
  9	 Q 	   474.78	  1085.03	 18
 10	 A 	   510.30	  1021.00	 17
 11	 E 	   574.82	   985.48	 16
 12	 W 	   667.86	   920.96	 15
 13	 D 	   725.38	   827.92	 14
 14	 D 	   782.89	   770.41	 13
 15	 Y 	   864.42	   712.89	 12
 16	 V 	   913.96	   631.36	 11
 17	 P 	   962.48	   581.83	 10
 18	 K 	  1026.53	   533.30	  9
 19	 L 	  1083.07	   469.25	  8
 20	 Y 	  1164.60	   412.71	  7
 21	 E 	  1229.12	   331.18	  6
 22	 Q 	  1293.15	   266.66	  5
 23	 L 	  1349.70	   202.63	  4
 24	 S 	  1393.21	   146.09	  3
 25	 G 	  1421.72	   102.57	  2
 26	 K 	        -  	    74.06	  1  _


+3 charge
		      B   	       Y
  1	 A 	    24.69	        -  	 26
  2	 P 	    57.04	   973.17	 25
  3	 V 	    90.06	   940.82	 24
  4	 A 	   113.74	   907.80	 23
  5	 G 	   132.75	   884.12	 22
  6	 T 	   166.43	   865.11	 21
  7	 C 	   219.82	   831.43	 20
  8	 Y 	   274.17	   778.04	 19
  9	 Q 	   316.86	   723.69	 18
 10	 A 	   340.54	   681.00	 17
 11	 E 	   383.55	   657.32	 16
 12	 W 	   445.58	   614.31	 15
 13	 D 	   483.92	   552.28	 14
 14	 D 	   522.26	   513.94	 13
 15	 Y 	   576.62	   475.60	 12
 16	 V 	   609.64	   421.24	 11
 17	 P 	   641.99	   388.22	 10
 18	 K 	   684.69	   355.87	  9
 19	 L 	   722.38	   313.17	  8
 20	 Y 	   776.74	   275.48	  7
 21	 E 	   819.75	   221.12	  6
 22	 Q 	   862.44	   178.11	  5
 23	 L 	   900.13	   135.42	  4
 24	 S 	   929.14	    97.73	  3
 25	 G 	   948.15	    68.72	  2
 26	 K 	        -  	    49.71	  1  _
```

**26. IPI00032258 Complement C4-A**

| Sequence | Charge | M+H+ | XCorr | DeltaCn |
| --- | --- | --- | --- | --- |
| R.VTASDPLDTLGSEGALSPGGVASLLRLPR.G | 3 | 2851.205 | 5.373 | 0.601 |

+1 charge

```
		      B   	       Y
  1	 V 	   100.08	        -  	 29
  2	 T 	   201.12	  2750.47	 28
  3	 A 	   272.16	  2649.42	 27
  4	 S 	   359.19	  2578.38	 26
  5	 D 	   474.22	  2491.35	 25
  6	 P 	   571.27	  2376.32	 24
  7	 L 	   684.36	  2279.27	 23
  8	 D 	   799.38	  2166.19	 22
  9	 T 	   900.43	  2051.16	 21
 10	 L 	  1013.51	  1950.11	 20
 11	 G 	  1070.54	  1837.03	 19
 12	 S 	  1157.57	  1780.01	 18
 13	 E 	  1286.61	  1692.98	 17
 14	 G 	  1343.63	  1563.93	 16
 15	 A 	  1414.67	  1506.91	 15
 16	 L 	  1527.75	  1435.87	 14
 17	 S 	  1614.79	  1322.79	 13
 18	 P 	  1711.84	  1235.76	 12
 19	 G 	  1768.86	  1138.71	 11
 20	 G 	  1825.88	  1081.68	 10
 21	 V 	  1924.95	  1024.66	  9
 22	 A 	  1995.99	   925.59	  8
 23	 S 	  2083.02	   854.56	  7
 24	 L 	  2196.10	   767.53	  6
 25	 L 	  2309.19	   654.44	  5
 26	 R 	  2465.29	   541.36	  4
 27	 L 	  2578.37	   385.26	  3
 28	 P 	  2675.43	   272.17	  2
 29	 R 	        -  	   175.12	  1  _


+2 charge
		      B   	       Y
  1	 V 	    50.54	        -  	 29
  2	 T 	   101.07	  1375.74	 28
  3	 A 	   136.58	  1325.21	 27
  4	 S 	   180.10	  1289.70	 26
  5	 D 	   237.61	  1246.18	 25
  6	 P 	   286.14	  1188.67	 24
  7	 L 	   342.68	  1140.14	 23
  8	 D 	   400.20	  1083.60	 22
  9	 T 	   450.72	  1026.08	 21
 10	 L 	   507.26	   975.56	 20
 11	 G 	   535.77	   919.02	 19
 12	 S 	   579.29	   890.51	 18
 13	 E 	   643.81	   846.99	 17
 14	 G 	   672.32	   782.47	 16
 15	 A 	   707.84	   753.96	 15
 16	 L 	   764.38	   718.44	 14
 17	 S 	   807.90	   661.90	 13
 18	 P 	   856.42	   618.38	 12
 19	 G 	   884.93	   569.86	 11
 20	 G 	   913.44	   541.35	 10
 21	 V 	   962.98	   512.83	  9
 22	 A 	   998.50	   463.30	  8
 23	 S 	  1042.01	   427.78	  7
 24	 L 	  1098.56	   384.27	  6
 25	 L 	  1155.10	   327.72	  5
 26	 R 	  1233.15	   271.18	  4
 27	 L 	  1289.69	   193.13	  3
 28	 P 	  1338.22	   136.59	  2
 29	 R 	        -  	    88.06	  1  _


+3 charge
		      B   	       Y
  1	 V 	    34.03	        -  	 29
  2	 T 	    67.71	   917.49	 28
  3	 A 	    91.39	   883.81	 27
  4	 S 	   120.40	   860.13	 26
  5	 D 	   158.74	   831.12	 25
  6	 P 	   191.10	   792.78	 24
  7	 L 	   228.79	   760.43	 23
  8	 D 	   267.13	   722.73	 22
  9	 T 	   300.82	   684.39	 21
 10	 L 	   338.51	   650.71	 20
 11	 G 	   357.52	   613.01	 19
 12	 S 	   386.53	   594.01	 18
 13	 E 	   429.54	   565.00	 17
 14	 G 	   448.55	   521.98	 16
 15	 A 	   472.23	   502.98	 15
 16	 L 	   509.92	   479.30	 14
 17	 S 	   538.93	   441.60	 13
 18	 P 	   571.28	   412.59	 12
 19	 G 	   590.29	   380.24	 11
 20	 G 	   609.30	   361.23	 10
 21	 V 	   642.32	   342.23	  9
 22	 A 	   666.00	   309.20	  8
 23	 S 	   695.01	   285.52	  7
 24	 L 	   732.71	   256.51	  6
 25	 L 	   770.40	   218.82	  5
 26	 R 	   822.43	   181.12	  4
 27	 L 	   860.13	   129.09	  3
 28	 P 	   892.48	    91.40	  2
 29	 R 	        -  	    59.04	  1  _
```

**27. IPI00005573 Isoform 1 of 5'(3')-deoxyribonucleotidase, cytosolic type**

| Sequence | Charge | M+H+ | XCorr | DeltaCn |
| --- | --- | --- | --- | --- |
| R.VLVDMDGVLADFEAGLLR.G | 2 | 1934.246 | 3.481 | 0.447 |

+1 charge

```
		      B   	       Y
  1	 V 	   100.08	        -  	 18
  2	 L 	   213.16	  1833.94	 17
  3	 V 	   312.23	  1720.86	 16
  4	 D 	   427.26	  1621.79	 15
  5	 M 	   558.30	  1506.76	 14
  6	 D 	   673.32	  1375.72	 13
  7	 G 	   730.34	  1260.69	 12
  8	 V 	   829.41	  1203.67	 11
  9	 L 	   942.50	  1104.60	 10
 10	 A 	  1013.53	   991.52	  9
 11	 D 	  1128.56	   920.48	  8
 12	 F 	  1275.63	   805.46	  7
 13	 E 	  1404.67	   658.39	  6
 14	 A 	  1475.71	   529.35	  5
 15	 G 	  1532.73	   458.31	  4
 16	 L 	  1645.81	   401.29	  3
 17	 L 	  1758.90	   288.20	  2
 18	 R 	        -  	   175.12	  1  _
+2 charge
		      B   	       Y
  1	 V 	    50.54	        -  	 18
  2	 L 	   107.08	   917.47	 17
  3	 V 	   156.62	   860.93	 16
  4	 D 	   214.13	   811.40	 15
  5	 M 	   279.65	   753.88	 14
  6	 D 	   337.16	   688.36	 13
  7	 G 	   365.68	   630.85	 12
  8	 V 	   415.21	   602.34	 11
  9	 L 	   471.75	   552.81	 10
 10	 A 	   507.27	   496.26	  9
 11	 D 	   564.78	   460.75	  8
 12	 F 	   638.32	   403.23	  7
 13	 E 	   702.84	   329.70	  6
 14	 A 	   738.36	   265.18	  5
 15	 G 	   766.87	   229.66	  4
 16	 L 	   823.41	   201.15	  3
 17	 L 	   879.95	   144.61	  2
 18	 R 	        -  	    88.06	  1  _
```

**28. IPI0002143 Actin, cytoplasmic 1**

| Sequence | Charge | M+H+ | XCorr | DeltaCn |
| --- | --- | --- | --- | --- |
| K.AQEPVKGPVSTKPGSCPIILIR.C | 3 | 2348.764 | 3.649 | 0.524 |

+1 charge

```
		      B   	       Y
  1	 A 	    72.04	        -  	 22
  2	 Q 	   200.10	  2276.41	 21
  3	 E 	   329.15	  2148.36	 20
  4	 P 	   426.20	  2019.31	 19
  5	 V 	   525.27	  1922.26	 18
  6	 K 	   653.36	  1823.19	 17
  7	 G 	   710.38	  1695.10	 16
  8	 P 	   807.44	  1638.08	 15
  9	 V 	   906.50	  1541.02	 14
 10	 S 	   993.54	  1441.95	 13
 11	 T 	  1094.58	  1354.92	 12
 12	 K 	  1222.68	  1253.87	 11
 13	 P 	  1319.73	  1125.78	 10
 14	 G 	  1376.75	  1028.73	  9
 15	 S 	  1463.79	   971.71	  8
 16	 C 	  1623.95	   884.67	  7
 17	 P 	  1721.00	   724.51	  6
 18	 I 	  1834.09	   627.46	  5
 19	 I 	  1947.17	   514.37	  4
 20	 L 	  2060.26	   401.29	  3
 21	 I 	  2173.34	   288.20	  2
 22	 R 	        -  	   175.12	  1  _


+2 charge
		      B   	       Y
  1	 A 	    36.53	        -  	 22
  2	 Q 	   100.56	  1138.71	 21
  3	 E 	   165.08	  1074.68	 20
  4	 P 	   213.60	  1010.16	 19
  5	 V 	   263.14	   961.63	 18
  6	 K 	   327.18	   912.10	 17
  7	 G 	   355.70	   848.05	 16
  8	 P 	   404.22	   819.54	 15
  9	 V 	   453.76	   771.01	 14
 10	 S 	   497.27	   721.48	 13
 11	 T 	   547.80	   677.96	 12
 12	 K 	   611.84	   627.44	 11
 13	 P 	   660.37	   563.39	 10
 14	 G 	   688.88	   514.87	  9
 15	 S 	   732.40	   486.36	  8
 16	 C 	   812.48	   442.84	  7
 17	 P 	   861.01	   362.76	  6
 18	 I 	   917.55	   314.23	  5
 19	 I 	   974.09	   257.69	  4
 20	 L 	  1030.63	   201.15	  3
 21	 I 	  1087.17	   144.61	  2
 22	 R 	        -  	    88.06	  1  _
+3 charge
		      B   	       Y
  1	 A 	    24.69	        -  	 22
  2	 Q 	    67.37	   759.48	 21
  3	 E 	   110.39	   716.79	 20
  4	 P 	   142.74	   673.78	 19
  5	 V 	   175.76	   641.42	 18
  6	 K 	   218.46	   608.40	 17
  7	 G 	   237.47	   565.70	 16
  8	 P 	   269.82	   546.70	 15
  9	 V 	   302.84	   514.35	 14
 10	 S 	   331.85	   481.32	 13
 11	 T 	   365.53	   452.31	 12
 12	 K 	   408.23	   418.63	 11
 13	 P 	   440.58	   375.93	 10
 14	 G 	   459.59	   343.58	  9
 15	 S 	   488.60	   324.57	  8
 16	 C 	   541.99	   295.56	  7
 17	 P 	   574.34	   242.17	  6
 18	 I 	   612.03	   209.82	  5
 19	 I 	   649.73	   172.13	  4
 20	 L 	   687.42	   134.43	  3
 21	 I 	   725.12	    96.74	  2
 22	 R 	        -  	    59.04	  1  _
```

**29. IPI00301143 Isoform 1 of Peptidase inhibitor 16**

| Sequence | Charge | M+H+ | XCorr | DeltaCn |
| --- | --- | --- | --- | --- |
| R.LMVELHNLYR.A | 2 | 1288.544 | 3.604 | 0.361 |

+1 charge

```
		      B   	       Y
  1	 L 	   114.09	        -  	 10
  2	 M 	   245.13	  1174.60	  9
  3	 V 	   344.20	  1043.56	  8
  4	 E 	   473.24	   944.49	  7
  5	 L 	   586.33	   815.45	  6
  6	 H 	   723.39	   702.37	  5
  7	 N 	   837.43	   565.31	  4
  8	 L 	   950.51	   451.27	  3
  9	 Y 	  1113.58	   338.18	  2
 10	 R 	        -  	   175.12	  1  _
+2 charge
		      B   	       Y
  1	 L 	    57.55	        -  	 10
  2	 M 	   123.07	   587.81	  9
  3	 V 	   172.60	   522.29	  8
  4	 E 	   237.13	   472.75	  7
  5	 L 	   293.67	   408.23	  6
  6	 H 	   362.20	   351.69	  5
  7	 N 	   419.22	   283.16	  4
  8	 L 	   475.76	   226.14	  3
  9	 Y 	   557.29	   169.59	  2
 10	 R 	        -  	    88.06	  1  _
```

**30. IPI00387113 Ig kappa chain V-III region B6**

| Sequence | Charge | M+H+ | XCorr | DeltaCn |
| --- | --- | --- | --- | --- |
| - .ZIVLTZSPGTLSLSPGZR.A | 2 | 1884.616 | 3.524 | 0.473 |

+1 charge

```
		      B   	       Y
  1	 Z 	   129.56	        -  	 18
  2	 I 	   242.64	  1754.96	 17
  3	 V 	   341.71	  1641.88	 16
  4	 L 	   454.79	  1542.81	 15
  5	 T 	   555.84	  1429.73	 14
  6	 Z 	   684.39	  1328.68	 13
  7	 S 	   771.42	  1200.13	 12
  8	 P 	   868.48	  1113.10	 11
  9	 G 	   925.50	  1016.05	 10
 10	 T 	  1026.55	   959.02	  9
 11	 L 	  1139.63	   857.98	  8
 12	 S 	  1226.66	   744.89	  7
 13	 L 	  1339.75	   657.86	  6
 14	 S 	  1426.78	   544.78	  5
 15	 P 	  1523.83	   457.74	  4
 16	 G 	  1580.85	   360.69	  3
 17	 Z 	  1709.40	   303.67	  2
 18	 R 	        -  	   175.12	  1  _
+2 charge
		      B   	       Y
  1	 Z 	    65.28	        -  	 18
  2	 I 	   121.82	   877.99	 17
  3	 V 	   171.36	   821.44	 16
  4	 L 	   227.90	   771.91	 15
  5	 T 	   278.42	   715.37	 14
  6	 Z 	   342.70	   664.84	 13
  7	 S 	   386.22	   600.57	 12
  8	 P 	   434.74	   557.05	 11
  9	 G 	   463.25	   508.53	 10
 10	 T 	   513.78	   480.02	  9
 11	 L 	   570.32	   429.49	  8
 12	 S 	   613.83	   372.95	  7
 13	 L 	   670.38	   329.43	  6
 14	 S 	   713.89	   272.89	  5
 15	 P 	   762.42	   229.38	  4
 16	 G 	   790.93	   180.85	  3
 17	 Z 	   855.21	   152.34	  2
 18	 R 	        -  	    88.06	  1  _
```

**31. IPI00024307 Ephrin-B1**

| Sequence | Charge | M+H+ | XCorr | DeltaCn |
| --- | --- | --- | --- | --- |
| K.NLEPVSWSSLNPK.F | 2 | 1471.64 | 3.239 | 0.31 |

+1 charge

```
		      B   	       Y
  1	 N 	   115.05	        -  	 13
  2	 L 	   228.13	  1356.72	 12
  3	 E 	   357.18	  1243.63	 11
  4	 P 	   454.23	  1114.59	 10
  5	 V 	   553.30	  1017.54	  9
  6	 S 	   640.33	   918.47	  8
  7	 W 	   826.41	   831.44	  7
  8	 S 	   913.44	   645.36	  6
  9	 S 	  1000.47	   558.32	  5
 10	 L 	  1113.56	   471.29	  4
 11	 N 	  1227.60	   358.21	  3
 12	 P 	  1324.65	   244.17	  2
 13	 K 	        -  	   147.11	  1  _
+2 charge
		      B   	       Y
  1	 N 	    58.03	        -  	 13
  2	 L 	   114.57	   678.86	 12
  3	 E 	   179.09	   622.32	 11
  4	 P 	   227.62	   557.80	 10
  5	 V 	   277.15	   509.27	  9
  6	 S 	   320.67	   459.74	  8
  7	 W 	   413.71	   416.22	  7
  8	 S 	   457.22	   323.18	  6
  9	 S 	   500.74	   279.67	  5
 10	 L 	   557.28	   236.15	  4
 11	 N 	   614.30	   179.61	  3
 12	 P 	   662.83	   122.59	  2
 13	 K 	        -  	    74.06	  1  _
```

**32. IPI00215767 Isoform Long of Beta-1,4-galactosyltransferase 1**

| Sequence | Charge | M+H+ | XCorr | DeltaCn |
| --- | --- | --- | --- | --- |
| R.DLSRLPQLVGVSTPLQGGSNSAAAIGQSSGELR.T | 3 | 3267.597 | 3.646 | 0.518 |

+1 charge

```
		      B   	       Y
  1	 D 	   116.03	        -  	 33
  2	 L 	   229.12	  3150.69	 32
  3	 S 	   316.15	  3037.60	 31
  4	 R 	   472.25	  2950.57	 30
  5	 L 	   585.34	  2794.47	 29
  6	 P 	   682.39	  2681.39	 28
  7	 Q 	   810.45	  2584.33	 27
  8	 L 	   923.53	  2456.27	 26
  9	 V 	  1022.60	  2343.19	 25
 10	 G 	  1079.62	  2244.12	 24
 11	 V 	  1178.69	  2187.10	 23
 12	 S 	  1265.72	  2088.03	 22
 13	 T 	  1366.77	  2001.00	 21
 14	 P 	  1463.82	  1899.95	 20
 15	 L 	  1576.91	  1802.90	 19
 16	 Q 	  1704.96	  1689.82	 18
 17	 G 	  1761.99	  1561.76	 17
 18	 G 	  1819.01	  1504.74	 16
 19	 S 	  1906.04	  1447.71	 15
 20	 N 	  2020.08	  1360.68	 14
 21	 S 	  2107.11	  1246.64	 13
 22	 A 	  2178.15	  1159.61	 12
 23	 A 	  2249.19	  1088.57	 11
 24	 A 	  2320.23	  1017.53	 10
 25	 I 	  2433.31	   946.50	  9
 26	 G 	  2490.33	   833.41	  8
 27	 Q 	  2618.39	   776.39	  7
 28	 S 	  2705.42	   648.33	  6
 29	 S 	  2792.45	   561.30	  5
 30	 G 	  2849.48	   474.27	  4
 31	 E 	  2978.52	   417.25	  3
 32	 L 	  3091.60	   288.20	  2
 33	 R 	        -  	   175.12	  1  _


+2 charge
		      B   	       Y
  1	 D 	    58.52	        -  	 33
  2	 L 	   115.06	  1575.85	 32
  3	 S 	   158.58	  1519.30	 31
  4	 R 	   236.63	  1475.79	 30
  5	 L 	   293.17	  1397.74	 29
  6	 P 	   341.70	  1341.20	 28
  7	 Q 	   405.73	  1292.67	 27
  8	 L 	   462.27	  1228.64	 26
  9	 V 	   511.80	  1172.10	 25
 10	 G 	   540.31	  1122.56	 24
 11	 V 	   589.85	  1094.05	 23
 12	 S 	   633.36	  1044.52	 22
 13	 T 	   683.89	  1001.00	 21
 14	 P 	   732.41	   950.48	 20
 15	 L 	   788.96	   901.95	 19
 16	 Q 	   852.99	   845.41	 18
 17	 G 	   881.50	   781.38	 17
 18	 G 	   910.01	   752.87	 16
 19	 S 	   953.52	   724.36	 15
 20	 N 	  1010.54	   680.84	 14
 21	 S 	  1054.06	   623.82	 13
 22	 A 	  1089.58	   580.31	 12
 23	 A 	  1125.10	   544.79	 11
 24	 A 	  1160.62	   509.27	 10
 25	 I 	  1217.16	   473.75	  9
 26	 G 	  1245.67	   417.21	  8
 27	 Q 	  1309.70	   388.70	  7
 28	 S 	  1353.21	   324.67	  6
 29	 S 	  1396.73	   281.15	  5
 30	 G 	  1425.24	   237.64	  4
 31	 E 	  1489.76	   209.13	  3
 32	 L 	  1546.30	   144.61	  2
 33	 R 	        -  	    88.06	  1  _


+3 charge
		      B   	       Y
  1	 D 	    39.35	        -  	 33
  2	 L 	    77.04	  1050.90	 32
  3	 S 	   106.05	  1013.21	 31
  4	 R 	   158.09	   984.20	 30
  5	 L 	   195.78	   932.16	 29
  6	 P 	   228.13	   894.47	 28
  7	 Q 	   270.82	   862.12	 27
  8	 L 	   308.52	   819.43	 26
  9	 V 	   341.54	   781.73	 25
 10	 G 	   360.55	   748.71	 24
 11	 V 	   393.57	   729.70	 23
 12	 S 	   422.58	   696.68	 22
 13	 T 	   456.26	   667.67	 21
 14	 P 	   488.61	   633.99	 20
 15	 L 	   526.31	   601.64	 19
 16	 Q 	   568.99	   563.94	 18
 17	 G 	   588.00	   521.26	 17
 18	 G 	   607.01	   502.25	 16
 19	 S 	   636.02	   483.24	 15
 20	 N 	   674.03	   454.23	 14
 21	 S 	   703.04	   416.22	 13
 22	 A 	   726.72	   387.21	 12
 23	 A 	   750.40	   363.53	 11
 24	 A 	   774.08	   339.85	 10
 25	 I 	   811.77	   316.17	  9
 26	 G 	   830.78	   278.48	  8
 27	 Q 	   873.47	   259.47	  7
 28	 S 	   902.48	   216.78	  6
 29	 S 	   931.49	   187.77	  5
 30	 G 	   950.50	   158.76	  4
 31	 E 	   993.51	   139.75	  3
 32	 L 	  1031.21	    96.74	  2
 33	 R 	        -  	    59.04	  1  _
```

**33. IPI00297263 Isoform 1 of Protein HEG homolog 1**

| Sequence | Charge | M+H+ | XCorr | DeltaCn |
| --- | --- | --- | --- | --- |
| R.ALSLAPLAGAGLELQLER.R | 2 | 1823.127 | 4.498 | 0.57 |

+1 charge

```
		      B   	       Y
  1	 A 	    72.04	        -  	 18
  2	 L 	   185.13	  1751.01	 17
  3	 S 	   272.16	  1637.92	 16
  4	 L 	   385.24	  1550.89	 15
  5	 A 	   456.28	  1437.81	 14
  6	 P 	   553.33	  1366.77	 13
  7	 L 	   666.42	  1269.72	 12
  8	 A 	   737.46	  1156.63	 11
  9	 G 	   794.48	  1085.59	 10
 10	 A 	   865.51	  1028.57	  9
 11	 G 	   922.54	   957.54	  8
 12	 L 	  1035.62	   900.51	  7
 13	 E 	  1164.66	   787.43	  6
 14	 L 	  1277.75	   658.39	  5
 15	 Q 	  1405.80	   545.30	  4
 16	 L 	  1518.89	   417.25	  3
 17	 E 	  1647.93	   304.16	  2
 18	 R 	        -  	   175.12	  1  _
+2 charge
		      B   	       Y
  1	 A 	    36.53	        -  	 18
  2	 L 	    93.07	   876.01	 17
  3	 S 	   136.58	   819.46	 16
  4	 L 	   193.13	   775.95	 15
  5	 A 	   228.64	   719.41	 14
  6	 P 	   277.17	   683.89	 13
  7	 L 	   333.71	   635.36	 12
  8	 A 	   369.23	   578.82	 11
  9	 G 	   397.74	   543.30	 10
 10	 A 	   433.26	   514.79	  9
 11	 G 	   461.77	   479.27	  8
 12	 L 	   518.31	   450.76	  7
 13	 E 	   582.83	   394.22	  6
 14	 L 	   639.38	   329.70	  5
 15	 Q 	   703.41	   273.16	  4
 16	 L 	   759.95	   209.13	  3
 17	 E 	   824.47	   152.58	  2
 18	 R 	        -  	    88.06	  1  _
```

**34. IPI00062047 Kinesin-like protein KIF12**

| Sequence | Charge | M+H+ | XCorr | DeltaCn |
| --- | --- | --- | --- | --- |
| R.NLYGMLQEFMLENER.L | 2 | 1888.158 | 2.962 | 0.295 |

+1 charge

```
		      B   	       Y
  1	 N 	   115.05	        -  	 15
  2	 L 	   228.13	  1772.83	 14
  3	 Y 	   391.20	  1659.75	 13
  4	 G 	   448.22	  1496.69	 12
  5	 M 	   579.26	  1439.67	 11
  6	 L 	   692.34	  1308.63	 10
  7	 Q 	   820.40	  1195.54	  9
  8	 E 	   949.44	  1067.48	  8
  9	 F 	  1096.51	   938.44	  7
 10	 M 	  1227.55	   791.37	  6
 11	 L 	  1340.64	   660.33	  5
 12	 E 	  1469.68	   547.25	  4
 13	 N 	  1583.72	   418.20	  3
 14	 E 	  1712.77	   304.16	  2
 15	 R 	        -  	   175.12	  1  _
+2 charge
		      B   	       Y
  1	 N 	    58.03	        -  	 15
  2	 L 	   114.57	   886.92	 14
  3	 Y 	   196.10	   830.38	 13
  4	 G 	   224.61	   748.85	 12
  5	 M 	   290.13	   720.34	 11
  6	 L 	   346.68	   654.82	 10
  7	 Q 	   410.70	   598.27	  9
  8	 E 	   475.23	   534.24	  8
  9	 F 	   548.76	   469.72	  7
 10	 M 	   614.28	   396.19	  6
 11	 L 	   670.82	   330.67	  5
 12	 E 	   735.34	   274.13	  4
 13	 N 	   792.37	   209.61	  3
 14	 E 	   856.89	   152.58	  2
 15	 R 	        -  	    88.06	  1  _
```

**35. IPI00246058 Programmed cell death 6-interacting protein**

| Sequence | Charge | M+H+ | XCorr | DeltaCn |
| --- | --- | --- | --- | --- |
| R.SVIEQGGIQTVDQLIKELPELLQR.N | 3 | 2708.104 | 3.609 | 0.355 |

+1 charge

```
		      B   	       Y
  1	 S 	    88.04	        -  	 24
  2	 V 	   187.11	  2619.47	 23
  3	 I 	   300.19	  2520.40	 22
  4	 E 	   429.23	  2407.32	 21
  5	 Q 	   557.29	  2278.28	 20
  6	 G 	   614.31	  2150.22	 19
  7	 G 	   671.34	  2093.20	 18
  8	 I 	   784.42	  2036.18	 17
  9	 Q 	   912.48	  1923.09	 16
 10	 T 	  1013.53	  1795.03	 15
 11	 V 	  1112.59	  1693.98	 14
 12	 D 	  1227.62	  1594.92	 13
 13	 Q 	  1355.68	  1479.89	 12
 14	 L 	  1468.76	  1351.83	 11
 15	 I 	  1581.85	  1238.75	 10
 16	 K 	  1709.94	  1125.66	  9
 17	 E 	  1838.99	   997.57	  8
 18	 L 	  1952.07	   868.53	  7
 19	 P 	  2049.12	   755.44	  6
 20	 E 	  2178.17	   658.39	  5
 21	 L 	  2291.25	   529.35	  4
 22	 L 	  2404.33	   416.26	  3
 23	 Q 	  2532.39	   303.18	  2
 24	 R 	        -  	   175.12	  1  _


+2 charge
		      B   	       Y
  1	 S 	    44.52	        -  	 24
  2	 V 	    94.06	  1310.24	 23
  3	 I 	   150.60	  1260.71	 22
  4	 E 	   215.12	  1204.16	 21
  5	 Q 	   279.15	  1139.64	 20
  6	 G 	   307.66	  1075.61	 19
  7	 G 	   336.17	  1047.10	 18
  8	 I 	   392.71	  1018.59	 17
  9	 Q 	   456.74	   962.05	 16
 10	 T 	   507.27	   898.02	 15
 11	 V 	   556.80	   847.50	 14
 12	 D 	   614.31	   797.96	 13
 13	 Q 	   678.34	   740.45	 12
 14	 L 	   734.89	   676.42	 11
 15	 I 	   791.43	   619.88	 10
 16	 K 	   855.48	   563.33	  9
 17	 E 	   920.00	   499.29	  8
 18	 L 	   976.54	   434.77	  7
 19	 P 	  1025.06	   378.22	  6
 20	 E 	  1089.59	   329.70	  5
 21	 L 	  1146.13	   265.18	  4
 22	 L 	  1202.67	   208.63	  3
 23	 Q 	  1266.70	   152.09	  2
 24	 R 	        -  	    88.06	  1  _


+3 charge
		      B   	       Y
  1	 S 	    30.02	        -  	 24
  2	 V 	    63.04	   873.83	 23
  3	 I 	   100.74	   840.81	 22
  4	 E 	   143.75	   803.11	 21
  5	 Q 	   186.44	   760.10	 20
  6	 G 	   205.44	   717.41	 19
  7	 G 	   224.45	   698.40	 18
  8	 I 	   262.14	   679.40	 17
  9	 Q 	   304.83	   641.70	 16
 10	 T 	   338.51	   599.02	 15
 11	 V 	   371.54	   565.33	 14
 12	 D 	   409.88	   532.31	 13
 13	 Q 	   452.56	   493.97	 12
 14	 L 	   490.26	   451.28	 11
 15	 I 	   527.95	   413.59	 10
 16	 K 	   570.65	   375.89	  9
 17	 E 	   613.67	   333.19	  8
 18	 L 	   651.36	   290.18	  7
 19	 P 	   683.71	   252.49	  6
 20	 E 	   726.73	   220.13	  5
 21	 L 	   764.42	   177.12	  4
 22	 L 	   802.12	   139.43	  3
 23	 Q 	   844.80	   101.73	  2
 24	 R 	        -  	    59.04	  1  _
```

**36. IPI00795918 Isoform 1 of Neural cell adhesion molecule 1**

| Sequence | Charge | M+H+ | XCorr | DeltaCn |
| --- | --- | --- | --- | --- |
| K.CVVTGEDGSESEATVNVK.I | 2 | 1881.967 | 3.539 | 0.542 |

+1 charge

```
		      B   	       Y
  1	 C 	   161.17	        -  	 18
  2	 V 	   260.24	  1720.82	 17
  3	 V 	   359.31	  1621.76	 16
  4	 T 	   460.36	  1522.69	 15
  5	 G 	   517.38	  1421.64	 14
  6	 E 	   646.42	  1364.62	 13
  7	 D 	   761.45	  1235.58	 12
  8	 G 	   818.47	  1120.55	 11
  9	 S 	   905.50	  1063.53	 10
 10	 E 	  1034.54	   976.49	  9
 11	 S 	  1121.58	   847.45	  8
 12	 E 	  1250.62	   760.42	  7
 13	 A 	  1321.66	   631.38	  6
 14	 T 	  1422.70	   560.34	  5
 15	 V 	  1521.77	   459.29	  4
 16	 N 	  1635.81	   360.22	  3
 17	 V 	  1734.88	   246.18	  2
 18	 K 	        -  	   147.11	  1  _
+2 charge
		      B   	       Y
  1	 C 	    81.09	        -  	 18
  2	 V 	   130.62	   860.92	 17
  3	 V 	   180.16	   811.38	 16
  4	 T 	   230.68	   761.85	 15
  5	 G 	   259.19	   711.32	 14
  6	 E 	   323.71	   682.81	 13
  7	 D 	   381.23	   618.29	 12
  8	 G 	   409.74	   560.78	 11
  9	 S 	   453.25	   532.27	 10
 10	 E 	   517.78	   488.75	  9
 11	 S 	   561.29	   424.23	  8
 12	 E 	   625.81	   380.71	  7
 13	 A 	   661.33	   316.19	  6
 14	 T 	   711.86	   280.67	  5
 15	 V 	   761.39	   230.15	  4
 16	 N 	   818.41	   180.62	  3
 17	 V 	   867.95	   123.59	  2
 18	 K 	        -  	    74.06	  1  _
```

**37. IPI00433028 Isoform 1 of Leukocyte-associated immunoglobulin-like receptor 1**

| Sequence | Charge | M+H+ | XCorr | DeltaCn |
| --- | --- | --- | --- | --- |
| R.FRIDSVSEGNAGPYR.C | 2 | 1668.792 | 4.233 | 0.442 |

+1 charge

```
		      B   	       Y
  1	 F 	   148.08	        -  	 15
  2	 R 	   304.18	  1520.75	 14
  3	 I 	   417.26	  1364.64	 13
  4	 D 	   532.29	  1251.56	 12
  5	 S 	   619.32	  1136.53	 11
  6	 V 	   718.39	  1049.50	 10
  7	 S 	   805.42	   950.43	  9
  8	 E 	   934.46	   863.40	  8
  9	 G 	   991.48	   734.36	  7
 10	 N 	  1105.53	   677.34	  6
 11	 A 	  1176.56	   563.29	  5
 12	 G 	  1233.59	   492.26	  4
 13	 P 	  1330.64	   435.24	  3
 14	 Y 	  1493.70	   338.18	  2
 15	 R 	        -  	   175.12	  1  _
+2 charge
		      B   	       Y
  1	 F 	    74.54	        -  	 15
  2	 R 	   152.59	   760.88	 14
  3	 I 	   209.13	   682.83	 13
  4	 D 	   266.65	   626.28	 12
  5	 S 	   310.16	   568.77	 11
  6	 V 	   359.70	   525.25	 10
  7	 S 	   403.21	   475.72	  9
  8	 E 	   467.74	   432.20	  8
  9	 G 	   496.25	   367.68	  7
 10	 N 	   553.27	   339.17	  6
 11	 A 	   588.79	   282.15	  5
 12	 G 	   617.30	   246.63	  4
 13	 P 	   665.82	   218.12	  3
 14	 Y 	   747.35	   169.59	  2
 15	 R 	        -  	    88.06	  1  _
```

**38. IPI00295414 Collagen alpha-1(XV) chain**

| Sequence | Charge | M+H+ | XCorr | DeltaCn |
| --- | --- | --- | --- | --- |
| R.FTGSLQQLTVHPDPR.T | 2 | 1696.888 | 2.996 | 0.508 |

+1 charge

```
		      B   	       Y
  1	 F 	   148.08	        -  	 15
  2	 T 	   249.12	  1548.81	 14
  3	 G 	   306.14	  1447.77	 13
  4	 S 	   393.18	  1390.74	 12
  5	 L 	   506.26	  1303.71	 11
  6	 Q 	   634.32	  1190.63	 10
  7	 Q 	   762.38	  1062.57	  9
  8	 L 	   875.46	   934.51	  8
  9	 T 	   976.51	   821.43	  7
 10	 V 	  1075.58	   720.38	  6
 11	 H 	  1212.64	   621.31	  5
 12	 P 	  1309.69	   484.25	  4
 13	 D 	  1424.72	   387.20	  3
 14	 P 	  1521.77	   272.17	  2
 15	 R 	        -  	   175.12	  1  _
+2 charge
		      B   	       Y
  1	 F 	    74.54	        -  	 15
  2	 T 	   125.07	   774.91	 14
  3	 G 	   153.58	   724.39	 13
  4	 S 	   197.09	   695.88	 12
  5	 L 	   253.63	   652.36	 11
  6	 Q 	   317.66	   595.82	 10
  7	 Q 	   381.69	   531.79	  9
  8	 L 	   438.23	   467.76	  8
  9	 T 	   488.76	   411.22	  7
 10	 V 	   538.29	   360.69	  6
 11	 H 	   606.82	   311.16	  5
 12	 P 	   655.35	   242.63	  4
 13	 D 	   712.86	   194.10	  3
 14	 P 	   761.39	   136.59	  2
 15	 R 	        -  	    88.06	  1  _
```

**39. IPI00384401 Myosin-reactive immunoglobulin kappa chain variable region (Fragment)**

| Sequence | Charge | M+H+ | XCorr | DeltaCn |
| --- | --- | --- | --- | --- |
| - .EIVMTQSPATLSVSPGER.A | 2 | 1903.147 | 4.549 | 0.55 |

+1 charge

```
		      B   	       Y
  1	 E 	   130.05	        -  	 18
  2	 I 	   243.13	  1772.92	 17
  3	 V 	   342.20	  1659.84	 16
  4	 M 	   473.24	  1560.77	 15
  5	 T 	   574.29	  1429.73	 14
  6	 Q 	   702.35	  1328.68	 13
  7	 S 	   789.38	  1200.62	 12
  8	 P 	   886.43	  1113.59	 11
  9	 A 	   957.47	  1016.54	 10
 10	 T 	  1058.52	   945.50	  9
 11	 L 	  1171.60	   844.45	  8
 12	 S 	  1258.63	   731.37	  7
 13	 V 	  1357.70	   644.34	  6
 14	 S 	  1444.74	   545.27	  5
 15	 P 	  1541.79	   458.24	  4
 16	 G 	  1598.81	   361.18	  3
 17	 E 	  1727.85	   304.16	  2
 18	 R 	        -  	   175.12	  1  _
+2 charge
		      B   	       Y
  1	 E 	    65.53	        -  	 18
  2	 I 	   122.07	   886.96	 17
  3	 V 	   171.60	   830.42	 16
  4	 M 	   237.13	   780.89	 15
  5	 T 	   287.65	   715.37	 14
  6	 Q 	   351.68	   664.84	 13
  7	 S 	   395.19	   600.81	 12
  8	 P 	   443.72	   557.30	 11
  9	 A 	   479.24	   508.77	 10
 10	 T 	   529.76	   473.25	  9
 11	 L 	   586.31	   422.73	  8
 12	 S 	   629.82	   366.19	  7
 13	 V 	   679.36	   322.67	  6
 14	 S 	   722.87	   273.14	  5
 15	 P 	   771.40	   229.62	  4
 16	 G 	   799.91	   181.10	  3
 17	 E 	   864.43	   152.58	  2
 18	 R 	        -  	    88.06	  1  _
```

**40. IPI00384195 Isoform 3 of Plexin-B1**

| Sequence | Charge | M+H+ | XCorr | DeltaCn |
| --- | --- | --- | --- | --- |
| R.PASTTSMTPPGSGSWKRR.P | 2 | 1905.128 | 3.024 | 0.238 |

+1 charge

```
		      B   	       Y
  1	 P 	    98.06	        -  	 18
  2	 A 	   169.10	  1806.89	 17
  3	 S 	   256.13	  1735.85	 16
  4	 T 	   357.18	  1648.82	 15
  5	 T 	   458.22	  1547.77	 14
  6	 S 	   545.26	  1446.73	 13
  7	 M 	   676.30	  1359.70	 12
  8	 T 	   777.34	  1228.65	 11
  9	 P 	   874.40	  1127.61	 10
 10	 P 	   971.45	  1030.55	  9
 11	 G 	  1028.47	   933.50	  8
 12	 S 	  1115.50	   876.48	  7
 13	 G 	  1172.53	   789.45	  6
 14	 S 	  1259.56	   732.43	  5
 15	 W 	  1445.64	   645.39	  4
 16	 K 	  1573.73	   459.32	  3
 17	 R 	  1729.83	   331.22	  2
 18	 R 	        -  	   175.12	  1  _
+2 charge
		      B   	       Y
  1	 P 	    49.53	        -  	 18
  2	 A 	    85.05	   903.95	 17
  3	 S 	   128.57	   868.43	 16
  4	 T 	   179.09	   824.91	 15
  5	 T 	   229.62	   774.39	 14
  6	 S 	   273.13	   723.87	 13
  7	 M 	   338.65	   680.35	 12
  8	 T 	   389.18	   614.83	 11
  9	 P 	   437.70	   564.31	 10
 10	 P 	   486.23	   515.78	  9
 11	 G 	   514.74	   467.25	  8
 12	 S 	   558.26	   438.74	  7
 13	 G 	   586.77	   395.23	  6
 14	 S 	   630.28	   366.72	  5
 15	 W 	   723.32	   323.20	  4
 16	 K 	   787.37	   230.16	  3
 17	 R 	   865.42	   166.11	  2
 18	 R 	        -  	    88.06	  1  _
```

**41. IPI00183445 Isoform 1 of Latrophilin-1**

| Sequence | Charge | M+H+ | XCorr | DeltaCn |
| --- | --- | --- | --- | --- |
| R.ELACEGYPIELR.C | 2 | 1450.612 | 3.589 | 0.322 |

+1 charge

```
		      B   	       Y
  1	 E 	   130.05	        -  	 12
  2	 L 	   243.13	  1320.80	 11
  3	 A 	   314.17	  1207.71	 10
  4	 C 	   474.34	  1136.68	  9
  5	 E 	   603.38	   976.51	  8
  6	 G 	   660.40	   847.47	  7
  7	 Y 	   823.46	   790.45	  6
  8	 P 	   920.52	   627.38	  5
  9	 I 	  1033.60	   530.33	  4
 10	 E 	  1162.64	   417.25	  3
 11	 L 	  1275.73	   288.20	  2
 12	 R 	        -  	   175.12	  1  _
+2 charge
		      B   	       Y
  1	 E 	    65.53	        -  	 12
  2	 L 	   122.07	   660.90	 11
  3	 A 	   157.59	   604.36	 10
  4	 C 	   237.67	   568.84	  9
  5	 E 	   302.19	   488.76	  8
  6	 G 	   330.70	   424.24	  7
  7	 Y 	   412.24	   395.73	  6
  8	 P 	   460.76	   314.19	  5
  9	 I 	   517.30	   265.67	  4
 10	 E 	   581.83	   209.13	  3
 11	 L 	   638.37	   144.61	  2
 12	 R 	        -  	    88.06	  1  _
```

**42. IPI00651692 Stromal cell-derived factor 1 theta**

| Sequence | Charge | M+H+ | XCorr | DeltaCn |
| --- | --- | --- | --- | --- |
| K.ILNTPNCALQIVAR.L | 2 | 1583.851 | 4.093 | 0.505 |

+1 charge

```
		      B   	       Y
  1	 I 	   114.09	        -  	 14
  2	 L 	   227.18	  1469.92	 13
  3	 N 	   341.22	  1356.84	 12
  4	 T 	   442.27	  1242.80	 11
  5	 P 	   539.32	  1141.75	 10
  6	 N 	   653.36	  1044.70	  9
  7	 C 	   813.53	   930.65	  8
  8	 A 	   884.56	   770.49	  7
  9	 L 	   997.65	   699.45	  6
 10	 Q 	  1125.71	   586.37	  5
 11	 I 	  1238.79	   458.31	  4
 12	 V 	  1337.86	   345.22	  3
 13	 A 	  1408.90	   246.16	  2
 14	 R 	        -  	   175.12	  1  _
+2 charge
		      B   	       Y
  1	 I 	    57.55	        -  	 14
  2	 L 	   114.09	   735.47	 13
  3	 N 	   171.11	   678.92	 12
  4	 T 	   221.64	   621.90	 11
  5	 P 	   270.16	   571.38	 10
  6	 N 	   327.18	   522.85	  9
  7	 C 	   407.27	   465.83	  8
  8	 A 	   442.79	   385.75	  7
  9	 L 	   499.33	   350.23	  6
 10	 Q 	   563.36	   293.69	  5
 11	 I 	   619.90	   229.66	  4
 12	 V 	   669.43	   173.12	  3
 13	 A 	   704.95	   123.58	  2
 14	 R 	        -  	    88.06	  1  _
```

**43. IPI0038712 Ig kappa chain V-IV region Len**

| Sequence | Charge | M+H+ | XCorr | DeltaCn |
| --- | --- | --- | --- | --- |
| - .DIVMTQSPDSLAVSLGER.A | 2 | 1919.147 | 5.549 | 0.574 |

+1 charge

```
		      B   	       Y
  1	 D 	   116.03	        -  	 18
  2	 I 	   229.12	  1802.93	 17
  3	 V 	   328.19	  1689.85	 16
  4	 M 	   459.23	  1590.78	 15
  5	 T 	   560.27	  1459.74	 14
  6	 Q 	   688.33	  1358.69	 13
  7	 S 	   775.37	  1230.63	 12
  8	 P 	   872.42	  1143.60	 11
  9	 D 	   987.45	  1046.55	 10
 10	 S 	  1074.48	   931.52	  9
 11	 L 	  1187.56	   844.49	  8
 12	 A 	  1258.60	   731.40	  7
 13	 V 	  1357.67	   660.37	  6
 14	 S 	  1444.70	   561.30	  5
 15	 L 	  1557.78	   474.27	  4
 16	 G 	  1614.80	   361.18	  3
 17	 E 	  1743.85	   304.16	  2
 18	 R 	        -  	   175.12	  1  _
+2 charge
		      B   	       Y
  1	 D 	    58.52	        -  	 18
  2	 I 	   115.06	   901.97	 17
  3	 V 	   164.60	   845.43	 16
  4	 M 	   230.12	   795.89	 15
  5	 T 	   280.64	   730.37	 14
  6	 Q 	   344.67	   679.85	 13
  7	 S 	   388.19	   615.82	 12
  8	 P 	   436.71	   572.30	 11
  9	 D 	   494.23	   523.78	 10
 10	 S 	   537.74	   466.26	  9
 11	 L 	   594.28	   422.75	  8
 12	 A 	   629.80	   366.21	  7
 13	 V 	   679.34	   330.69	  6
 14	 S 	   722.85	   281.15	  5
 15	 L 	   779.40	   237.64	  4
 16	 G 	   807.91	   181.10	  3
 17	 E 	   872.43	   152.58	  2
 18	 R 	        -  	    88.06	  1  _
```

**44. IPI00009028 Tetranectin**

| Sequence | Charge | M+H+ | XCorr | DeltaCn |
| --- | --- | --- | --- | --- |
| R.GGTLSTPQTGSENDALYEYLR.Q | 2 | 2273.399 | 3.142 | 0.532 |

+1 charge

```
		      B   	       Y
  1	 G 	    58.03	        -  	 21
  2	 G 	   115.05	  2215.05	 20
  3	 T 	   216.10	  2158.03	 19
  4	 L 	   329.18	  2056.98	 18
  5	 S 	   416.21	  1943.90	 17
  6	 T 	   517.26	  1856.87	 16
  7	 P 	   614.31	  1755.82	 15
  8	 Q 	   742.37	  1658.77	 14
  9	 T 	   843.42	  1530.71	 13
 10	 G 	   900.44	  1429.66	 12
 11	 S 	   987.47	  1372.64	 11
 12	 E 	  1116.52	  1285.61	 10
 13	 N 	  1230.56	  1156.56	  9
 14	 D 	  1345.59	  1042.52	  8
 15	 A 	  1416.62	   927.49	  7
 16	 L 	  1529.71	   856.46	  6
 17	 Y 	  1692.77	   743.37	  5
 18	 E 	  1821.81	   580.31	  4
 19	 Y 	  1984.88	   451.27	  3
 20	 L 	  2097.96	   288.20	  2
 21	 R 	        -  	   175.12	  1  _
+2 charge
		      B   	       Y
  1	 G 	    29.52	        -  	 21
  2	 G 	    58.03	  1108.03	 20
  3	 T 	   108.55	  1079.52	 19
  4	 L 	   165.09	  1028.99	 18
  5	 S 	   208.61	   972.45	 17
  6	 T 	   259.13	   928.94	 16
  7	 P 	   307.66	   878.41	 15
  8	 Q 	   371.69	   829.89	 14
  9	 T 	   422.21	   765.86	 13
 10	 G 	   450.72	   715.33	 12
 11	 S 	   494.24	   686.82	 11
 12	 E 	   558.76	   643.31	 10
 13	 N 	   615.78	   578.79	  9
 14	 D 	   673.30	   521.76	  8
 15	 A 	   708.82	   464.25	  7
 16	 L 	   765.36	   428.73	  6
 17	 Y 	   846.89	   372.19	  5
 18	 E 	   911.41	   290.66	  4
 19	 Y 	   992.94	   226.14	  3
 20	 L 	  1049.48	   144.61	  2
 21	 R 	        -  	    88.06	  1  _
```

**45. IPI00018901 Isoform 1 of Gamma-glutamyltranspeptidase 1**

| Sequence | Charge | M+H+ | XCorr | DeltaCn |
| --- | --- | --- | --- | --- |
| R.TVIEQQPVLCEVFCR.D | 2 | 1879.134 | 3.584 | 0.539 |

+1 charge

```
		      B   	       Y
  1	 T 	   102.05	        -  	 15
  2	 V 	   201.12	  1777.15	 14
  3	 I 	   314.21	  1678.08	 13
  4	 E 	   443.25	  1564.99	 12
  5	 Q 	   571.31	  1435.95	 11
  6	 Q 	   699.37	  1307.89	 10
  7	 P 	   796.42	  1179.83	  9
  8	 V 	   895.49	  1082.78	  8
  9	 L 	  1008.57	   983.71	  7
 10	 C 	  1168.74	   870.63	  6
 11	 E 	  1297.78	   710.46	  5
 12	 V 	  1396.85	   581.42	  4
 13	 F 	  1543.92	   482.35	  3
 14	 C 	  1704.08	   335.28	  2
 15	 R 	        -  	   175.12	  1  _
+2 charge
		      B   	       Y
  1	 T 	    51.53	        -  	 15
  2	 V 	   101.07	   889.08	 14
  3	 I 	   157.61	   839.54	 13
  4	 E 	   222.13	   783.00	 12
  5	 Q 	   286.16	   718.48	 11
  6	 Q 	   350.19	   654.45	 10
  7	 P 	   398.71	   590.42	  9
  8	 V 	   448.25	   541.89	  8
  9	 L 	   504.79	   492.36	  7
 10	 C 	   584.87	   435.82	  6
 11	 E 	   649.39	   355.74	  5
 12	 V 	   698.93	   291.21	  4
 13	 F 	   772.46	   241.68	  3
 14	 C 	   852.54	   168.15	  2
 15	 R 	        -  	    88.06	  1  _
```

**46. IPI00647357 Isoform 1 of Osteoclast-associated immunoglobulin-like receptor**

| Sequence | Charge | M+H+ | XCorr | DeltaCn |
| --- | --- | --- | --- | --- |
| R.HSAQPWADFTLLGAR.A | 2 | 1670.853 | 3.672 | 0.514 |

+1 charge

```
		      B   	       Y
  1	 H 	   138.07	        -  	 15
  2	 S 	   225.10	  1532.79	 14
  3	 A 	   296.14	  1445.75	 13
  4	 Q 	   424.19	  1374.72	 12
  5	 P 	   521.25	  1246.66	 11
  6	 W 	   707.33	  1149.61	 10
  7	 A 	   778.36	   963.53	  9
  8	 D 	   893.39	   892.49	  8
  9	 F 	  1040.46	   777.46	  7
 10	 T 	  1141.51	   630.39	  6
 11	 L 	  1254.59	   529.35	  5
 12	 L 	  1367.67	   416.26	  4
 13	 G 	  1424.70	   303.18	  3
 14	 A 	  1495.73	   246.16	  2
 15	 R 	        -  	   175.12	  1  _
+2 charge
		      B   	       Y
  1	 H 	    69.54	        -  	 15
  2	 S 	   113.05	   766.90	 14
  3	 A 	   148.57	   723.38	 13
  4	 Q 	   212.60	   687.86	 12
  5	 P 	   261.13	   623.83	 11
  6	 W 	   354.17	   575.31	 10
  7	 A 	   389.69	   482.27	  9
  8	 D 	   447.20	   446.75	  8
  9	 F 	   520.73	   389.23	  7
 10	 T 	   571.26	   315.70	  6
 11	 L 	   627.80	   265.18	  5
 12	 L 	   684.34	   208.63	  4
 13	 G 	   712.85	   152.09	  3
 14	 A 	   748.37	   123.58	  2
 15	 R 	        -  	    88.06	  1  _
```

**47. IPI00000828 Proenkephalin-A**

| Sequence | Charge | M+H+ | XCorr | DeltaCn |
| --- | --- | --- | --- | --- |
| K.DAEEDDSLANSSDLLKELLETGDNRER.S | 3 | 3036.123 | 5.819 | 0.538 |

+1 charge

```
		      B   	       Y
  1	 D 	   116.03	        -  	 27
  2	 A 	   187.07	  2919.38	 26
  3	 E 	   316.11	  2848.34	 25
  4	 E 	   445.16	  2719.30	 24
  5	 D 	   560.18	  2590.26	 23
  6	 D 	   675.21	  2475.23	 22
  7	 S 	   762.24	  2360.21	 21
  8	 L 	   875.33	  2273.17	 20
  9	 A 	   946.36	  2160.09	 19
 10	 N 	  1060.41	  2089.05	 18
 11	 S 	  1147.44	  1975.01	 17
 12	 S 	  1234.47	  1887.98	 16
 13	 D 	  1349.50	  1800.95	 15
 14	 L 	  1462.58	  1685.92	 14
 15	 L 	  1575.67	  1572.83	 13
 16	 K 	  1703.76	  1459.75	 12
 17	 E 	  1832.80	  1331.65	 11
 18	 L 	  1945.89	  1202.61	 10
 19	 L 	  2058.97	  1089.53	  9
 20	 E 	  2188.01	   976.44	  8
 21	 T 	  2289.06	   847.40	  7
 22	 G 	  2346.08	   746.35	  6
 23	 D 	  2461.11	   689.33	  5
 24	 N 	  2575.15	   574.31	  4
 25	 R 	  2731.25	   460.26	  3
 26	 E 	  2860.30	   304.16	  2
 27	 R 	        -  	   175.12	  1  _


+2 charge
		      B   	       Y
  1	 D 	    58.52	        -  	 27
  2	 A 	    94.04	  1460.19	 26
  3	 E 	   158.56	  1424.68	 25
  4	 E 	   223.08	  1360.15	 24
  5	 D 	   280.60	  1295.63	 23
  6	 D 	   338.11	  1238.12	 22
  7	 S 	   381.62	  1180.61	 21
  8	 L 	   438.17	  1137.09	 20
  9	 A 	   473.69	  1080.55	 19
 10	 N 	   530.71	  1045.03	 18
 11	 S 	   574.22	   988.01	 17
 12	 S 	   617.74	   944.49	 16
 13	 D 	   675.25	   900.98	 15
 14	 L 	   731.79	   843.46	 14
 15	 L 	   788.34	   786.92	 13
 16	 K 	   852.38	   730.38	 12
 17	 E 	   916.91	   666.33	 11
 18	 L 	   973.45	   601.81	 10
 19	 L 	  1029.99	   545.27	  9
 20	 E 	  1094.51	   488.73	  8
 21	 T 	  1145.03	   424.20	  7
 22	 G 	  1173.55	   373.68	  6
 23	 D 	  1231.06	   345.17	  5
 24	 N 	  1288.08	   287.66	  4
 25	 R 	  1366.13	   230.63	  3
 26	 E 	  1430.65	   152.58	  2
 27	 R 	        -  	    88.06	  1  _


+3 charge
		      B   	       Y
  1	 D 	    39.35	        -  	 27
  2	 A 	    63.03	   973.80	 26
  3	 E 	   106.04	   950.12	 25
  4	 E 	   149.06	   907.11	 24
  5	 D 	   187.40	   864.09	 23
  6	 D 	   225.74	   825.75	 22
  7	 S 	   254.75	   787.41	 21
  8	 L 	   292.45	   758.40	 20
  9	 A 	   316.13	   720.70	 19
 10	 N 	   354.14	   697.02	 18
 11	 S 	   383.15	   659.01	 17
 12	 S 	   412.16	   630.00	 16
 13	 D 	   450.50	   600.99	 15
 14	 L 	   488.20	   562.64	 14
 15	 L 	   525.89	   524.95	 13
 16	 K 	   568.59	   487.25	 12
 17	 E 	   611.61	   444.56	 11
 18	 L 	   649.30	   401.54	 10
 19	 L 	   687.00	   363.85	  9
 20	 E 	   730.01	   326.15	  8
 21	 T 	   763.69	   283.14	  7
 22	 G 	   782.70	   249.46	  6
 23	 D 	   821.04	   230.45	  5
 24	 N 	   859.06	   192.11	  4
 25	 R 	   911.09	   154.09	  3
 26	 E 	   954.10	   102.06	  2
 27	 R 	        -  	    59.04	  1  _
```

**48. IPI00167710 Isoform 1 of Fibulin-7**

| Sequence | Charge | M+H+ | XCorr | DeltaCn |
| --- | --- | --- | --- | --- |
| K.TISFHYLSLPSNLKTPITLFR.M | 3 | 2449.875 | 4.196 | 0.498 |

+1 charge

```
		      B   	       Y
  1	 T 	   102.05	        -  	 21
  2	 I 	   215.14	  2347.32	 20
  3	 S 	   302.17	  2234.23	 19
  4	 F 	   449.24	  2147.20	 18
  5	 H 	   586.30	  2000.13	 17
  6	 Y 	   749.36	  1863.07	 16
  7	 L 	   862.45	  1700.01	 15
  8	 S 	   949.48	  1586.93	 14
  9	 L 	  1062.56	  1499.89	 13
 10	 P 	  1159.61	  1386.81	 12
 11	 S 	  1246.65	  1289.76	 11
 12	 N 	  1360.69	  1202.73	 10
 13	 L 	  1473.77	  1088.68	  9
 14	 K 	  1601.87	   975.60	  8
 15	 T 	  1702.92	   847.50	  7
 16	 P 	  1799.97	   746.46	  6
 17	 I 	  1913.05	   649.40	  5
 18	 T 	  2014.10	   536.32	  4
 19	 L 	  2127.18	   435.27	  3
 20	 F 	  2274.25	   322.19	  2
 21	 R 	        -  	   175.12	  1  _
+2 charge
		      B   	       Y
  1	 T 	    51.53	        -  	 21
  2	 I 	   108.07	  1174.16	 20
  3	 S 	   151.59	  1117.62	 19
  4	 F 	   225.12	  1074.10	 18
  5	 H 	   293.65	  1000.57	 17
  6	 Y 	   375.18	   932.04	 16
  7	 L 	   431.73	   850.51	 15
  8	 S 	   475.24	   793.97	 14
  9	 L 	   531.78	   750.45	 13
 10	 P 	   580.31	   693.91	 12
 11	 S 	   623.83	   645.38	 11
 12	 N 	   680.85	   601.87	 10
 13	 L 	   737.39	   544.84	  9
 14	 K 	   801.44	   488.30	  8
 15	 T 	   851.96	   424.26	  7
 16	 P 	   900.49	   373.73	  6
 17	 I 	   957.03	   325.21	  5
 18	 T 	  1007.55	   268.66	  4
 19	 L 	  1064.10	   218.14	  3
 20	 F 	  1137.63	   161.60	  2
 21	 R 	        -  	    88.06	  1  _
+3 charge
		      B   	       Y
  1	 T 	    34.69	        -  	 21
  2	 I 	    72.38	   783.11	 20
  3	 S 	   101.40	   745.42	 19
  4	 F 	   150.42	   716.41	 18
  5	 H 	   196.10	   667.38	 17
  6	 Y 	   250.46	   621.70	 16
  7	 L 	   288.15	   567.34	 15
  8	 S 	   317.16	   529.65	 14
  9	 L 	   354.86	   500.64	 13
 10	 P 	   387.21	   462.94	 12
 11	 S 	   416.22	   430.59	 11
 12	 N 	   454.23	   401.58	 10
 13	 L 	   491.93	   363.57	  9
 14	 K 	   534.63	   325.87	  8
 15	 T 	   568.31	   283.17	  7
 16	 P 	   600.66	   249.49	  6
 17	 I 	   638.36	   217.14	  5
 18	 T 	   672.04	   179.44	  4
 19	 L 	   709.73	   145.76	  3
 20	 F 	   758.76	   108.07	  2
 21	 R 	        -  	    59.04	  1  _
```

**49. IPI00794461 Histone H2B type 1-N**

| Sequence | Charge | M+H+ | XCorr | DeltaCn |
| --- | --- | --- | --- | --- |
| K.AMGIMNSFVNDIFER.I | 2 | 1745.017 | 4.865 | 0.57 |

+1 charge

```
		      B   	       Y
  1	 A 	    72.04	        -  	 15
  2	 M 	   203.08	  1672.78	 14
  3	 G 	   260.11	  1541.74	 13
  4	 I 	   373.19	  1484.72	 12
  5	 M 	   504.23	  1371.64	 11
  6	 N 	   618.27	  1240.60	 10
  7	 S 	   705.31	  1126.55	  9
  8	 F 	   852.37	  1039.52	  8
  9	 V 	   951.44	   892.45	  7
 10	 N 	  1065.49	   793.38	  6
 11	 D 	  1180.51	   679.34	  5
 12	 I 	  1293.60	   564.31	  4
 13	 F 	  1440.67	   451.23	  3
 14	 E 	  1569.71	   304.16	  2
 15	 R 	        -  	   175.12	  1  _
+2 charge
		      B   	       Y
  1	 A 	    36.53	        -  	 15
  2	 M 	   102.05	   836.89	 14
  3	 G 	   130.56	   771.37	 13
  4	 I 	   187.10	   742.86	 12
  5	 M 	   252.62	   686.32	 11
  6	 N 	   309.64	   620.80	 10
  7	 S 	   353.16	   563.78	  9
  8	 F 	   426.69	   520.26	  8
  9	 V 	   476.22	   446.73	  7
 10	 N 	   533.25	   397.20	  6
 11	 D 	   590.76	   340.17	  5
 12	 I 	   647.30	   282.66	  4
 13	 F 	   720.84	   226.12	  3
 14	 E 	   785.36	   152.58	  2
 15	 R 	        -  	    88.06	  1  _
```

**50. IPI00418125 Isoform 1 of Layilin**

| Sequence | Charge | M+H+ | XCorr | DeltaCn |
| --- | --- | --- | --- | --- |
| K.FIENLLPSDGDFWIGLR.R | 2 | 1993.25 | 2.996 | 0.231 |

+1 charge

```
		      B   	       Y
  1	 F 	   148.08	        -  	 17
  2	 I 	   261.16	  1844.95	 16
  3	 E 	   390.20	  1731.87	 15
  4	 N 	   504.25	  1602.83	 14
  5	 L 	   617.33	  1488.78	 13
  6	 L 	   730.41	  1375.70	 12
  7	 P 	   827.47	  1262.62	 11
  8	 S 	   914.50	  1165.56	 10
  9	 D 	  1029.53	  1078.53	  9
 10	 G 	  1086.55	   963.50	  8
 11	 D 	  1201.57	   906.48	  7
 12	 F 	  1348.64	   791.46	  6
 13	 W 	  1534.72	   644.39	  5
 14	 I 	  1647.81	   458.31	  4
 15	 G 	  1704.83	   345.22	  3
 16	 L 	  1817.91	   288.20	  2
 17	 R 	        -  	   175.12	  1  _
+2 charge
		      B   	       Y
  1	 F 	    74.54	        -  	 17
  2	 I 	   131.08	   922.98	 16
  3	 E 	   195.60	   866.44	 15
  4	 N 	   252.63	   801.92	 14
  5	 L 	   309.17	   744.90	 13
  6	 L 	   365.71	   688.35	 12
  7	 P 	   414.24	   631.81	 11
  8	 S 	   457.75	   583.29	 10
  9	 D 	   515.27	   539.77	  9
 10	 G 	   543.78	   482.26	  8
 11	 D 	   601.29	   453.75	  7
 12	 F 	   674.82	   396.23	  6
 13	 W 	   767.86	   322.70	  5
 14	 I 	   824.41	   229.66	  4
 15	 G 	   852.92	   173.12	  3
 16	 L 	   909.46	   144.61	  2
 17	 R 	        -  	    88.06	  1  _
```

**51. IPI00555812 Isoform 1 of Vitamin D-binding protein**

| Sequence | Charge | M+H+ | XCorr | DeltaCn |
| --- | --- | --- | --- | --- |
| K.LAQKVPTADLEDVLPLAEDITNILSK.C | 3 | 2808.217 | 5.63 | 0.627 |

+1 charge

```
		      B   	       Y
  1	 L 	   114.09	        -  	 26
  2	 A 	   185.13	  2693.46	 25
  3	 Q 	   313.19	  2622.42	 24
  4	 K 	   441.28	  2494.37	 23
  5	 V 	   540.35	  2366.27	 22
  6	 P 	   637.40	  2267.20	 21
  7	 T 	   738.45	  2170.15	 20
  8	 A 	   809.49	  2069.10	 19
  9	 D 	   924.51	  1998.06	 18
 10	 L 	  1037.60	  1883.04	 17
 11	 E 	  1166.64	  1769.95	 16
 12	 D 	  1281.67	  1640.91	 15
 13	 V 	  1380.74	  1525.88	 14
 14	 L 	  1493.82	  1426.82	 13
 15	 P 	  1590.87	  1313.73	 12
 16	 L 	  1703.96	  1216.68	 11
 17	 A 	  1774.99	  1103.59	 10
 18	 E 	  1904.04	  1032.56	  9
 19	 D 	  2019.06	   903.51	  8
 20	 I 	  2132.15	   788.49	  7
 21	 T 	  2233.20	   675.40	  6
 22	 N 	  2347.24	   574.36	  5
 23	 I 	  2460.32	   460.31	  4
 24	 L 	  2573.41	   347.23	  3
 25	 S 	  2660.44	   234.14	  2
 26	 K 	        -  	   147.11	  1  _


+2 charge
		      B   	       Y
  1	 L 	    57.55	        -  	 26
  2	 A 	    93.07	  1347.23	 25
  3	 Q 	   157.10	  1311.72	 24
  4	 K 	   221.14	  1247.69	 23
  5	 V 	   270.68	  1183.64	 22
  6	 P 	   319.21	  1134.10	 21
  7	 T 	   369.73	  1085.58	 20
  8	 A 	   405.25	  1035.05	 19
  9	 D 	   462.76	   999.54	 18
 10	 L 	   519.30	   942.02	 17
 11	 E 	   583.82	   885.48	 16
 12	 D 	   641.34	   820.96	 15
 13	 V 	   690.87	   763.45	 14
 14	 L 	   747.41	   713.91	 13
 15	 P 	   795.94	   657.37	 12
 16	 L 	   852.48	   608.84	 11
 17	 A 	   888.00	   552.30	 10
 18	 E 	   952.52	   516.78	  9
 19	 D 	  1010.04	   452.26	  8
 20	 I 	  1066.58	   394.75	  7
 21	 T 	  1117.10	   338.21	  6
 22	 N 	  1174.12	   287.68	  5
 23	 I 	  1230.67	   230.66	  4
 24	 L 	  1287.21	   174.12	  3
 25	 S 	  1330.72	   117.58	  2
 26	 K 	        -  	    74.06	  1  _


+3 charge
		      B   	       Y
  1	 L 	    38.70	        -  	 26
  2	 A 	    62.38	   898.49	 25
  3	 Q 	   105.07	   874.81	 24
  4	 K 	   147.77	   832.13	 23
  5	 V 	   180.79	   789.43	 22
  6	 P 	   213.14	   756.41	 21
  7	 T 	   246.82	   724.05	 20
  8	 A 	   270.50	   690.37	 19
  9	 D 	   308.84	   666.69	 18
 10	 L 	   346.54	   628.35	 17
 11	 E 	   389.55	   590.66	 16
 12	 D 	   427.89	   547.64	 15
 13	 V 	   460.92	   509.30	 14
 14	 L 	   498.61	   476.28	 13
 15	 P 	   530.96	   438.58	 12
 16	 L 	   568.66	   406.23	 11
 17	 A 	   592.34	   368.54	 10
 18	 E 	   635.35	   344.86	  9
 19	 D 	   673.69	   301.84	  8
 20	 I 	   711.39	   263.50	  7
 21	 T 	   745.07	   225.81	  6
 22	 N 	   783.08	   192.12	  5
 23	 I 	   820.78	   154.11	  4
 24	 L 	   858.47	   116.41	  3
 25	 S 	   887.48	    78.72	  2
 26	 K 	        -  	    49.71	  1  _
```

**52. IPI00013890 Isoform 1 of 14-3-3 protein sigma**

| Sequence | Charge | M+H+ | XCorr | DeltaCn |
| --- | --- | --- | --- | --- |
| R.VLSSIEQKSNEEGSEEKGPEVR.E | 3 | 2432.584 | 3.364 | 0.456 |

+1 charge

```
		      B   	       Y
  1	 V 	   100.08	        -  	 22
  2	 L 	   213.16	  2332.13	 21
  3	 S 	   300.19	  2219.04	 20
  4	 S 	   387.22	  2132.01	 19
  5	 I 	   500.31	  2044.98	 18
  6	 E 	   629.35	  1931.89	 17
  7	 Q 	   757.41	  1802.85	 16
  8	 K 	   885.50	  1674.79	 15
  9	 S 	   972.54	  1546.70	 14
 10	 N 	  1086.58	  1459.67	 13
 11	 E 	  1215.62	  1345.62	 12
 12	 E 	  1344.66	  1216.58	 11
 13	 G 	  1401.69	  1087.54	 10
 14	 S 	  1488.72	  1030.52	  9
 15	 E 	  1617.76	   943.48	  8
 16	 E 	  1746.80	   814.44	  7
 17	 K 	  1874.90	   685.40	  6
 18	 G 	  1931.92	   557.30	  5
 19	 P 	  2028.97	   500.28	  4
 20	 E 	  2158.01	   403.23	  3
 21	 V 	  2257.08	   274.19	  2
 22	 R 	        -  	   175.12	  1  _


+2 charge
		      B   	       Y
  1	 V 	    50.54	        -  	 22
  2	 L 	   107.08	  1166.57	 21
  3	 S 	   150.60	  1110.02	 20
  4	 S 	   194.12	  1066.51	 19
  5	 I 	   250.66	  1022.99	 18
  6	 E 	   315.18	   966.45	 17
  7	 Q 	   379.21	   901.93	 16
  8	 K 	   443.26	   837.90	 15
  9	 S 	   486.77	   773.85	 14
 10	 N 	   543.79	   730.34	 13
 11	 E 	   608.31	   673.32	 12
 12	 E 	   672.84	   608.79	 11
 13	 G 	   701.35	   544.27	 10
 14	 S 	   744.86	   515.76	  9
 15	 E 	   809.38	   472.25	  8
 16	 E 	   873.91	   407.72	  7
 17	 K 	   937.95	   343.20	  6
 18	 G 	   966.46	   279.16	  5
 19	 P 	  1014.99	   250.64	  4
 20	 E 	  1079.51	   202.12	  3
 21	 V 	  1129.05	   137.60	  2
 22	 R 	        -  	    88.06	  1  _
+3 charge
		      B   	       Y
  1	 V 	    34.03	        -  	 22
  2	 L 	    71.72	   778.05	 21
  3	 S 	   100.74	   740.35	 20
  4	 S 	   129.75	   711.34	 19
  5	 I 	   167.44	   682.33	 18
  6	 E 	   210.46	   644.64	 17
  7	 Q 	   253.14	   601.62	 16
  8	 K 	   295.84	   558.94	 15
  9	 S 	   324.85	   516.24	 14
 10	 N 	   362.86	   487.23	 13
 11	 E 	   405.88	   449.21	 12
 12	 E 	   448.89	   406.20	 11
 13	 G 	   467.90	   363.18	 10
 14	 S 	   496.91	   344.18	  9
 15	 E 	   539.92	   315.17	  8
 16	 E 	   582.94	   272.15	  7
 17	 K 	   625.64	   229.14	  6
 18	 G 	   644.64	   186.44	  5
 19	 P 	   677.00	   167.43	  4
 20	 E 	   720.01	   135.08	  3
 21	 V 	   753.03	    92.07	  2
 22	 R 	        -  	    59.04	  1  _
```

**53. IPI00027166 Metalloproteinase inhibitor 2**

| Sequence | Charge | M+H+ | XCorr | DeltaCn |
| --- | --- | --- | --- | --- |
| K.AVSEKEVDSGNDIYGNPIKR.I | 3 | 2192.372 | 5.113 | 0.308 |

+1 charge

```
		      B   	       Y
  1	 A 	    72.04	        -  	 20
  2	 V 	   171.11	  2120.06	 19
  3	 S 	   258.14	  2020.99	 18
  4	 E 	   387.19	  1933.96	 17
  5	 K 	   515.28	  1804.92	 16
  6	 E 	   644.32	  1676.82	 15
  7	 V 	   743.39	  1547.78	 14
  8	 D 	   858.42	  1448.71	 13
  9	 S 	   945.45	  1333.69	 12
 10	 G 	  1002.47	  1246.65	 11
 11	 N 	  1116.52	  1189.63	 10
 12	 D 	  1231.54	  1075.59	  9
 13	 I 	  1344.63	   960.56	  8
 14	 Y 	  1507.69	   847.48	  7
 15	 G 	  1564.71	   684.42	  6
 16	 N 	  1678.76	   627.39	  5
 17	 P 	  1775.81	   513.35	  4
 18	 I 	  1888.89	   416.30	  3
 19	 K 	  2016.99	   303.21	  2
 20	 R 	        -  	   175.12	  1  _
+2 charge
		      B   	       Y
  1	 A 	    36.53	        -  	 20
  2	 V 	    86.06	  1060.53	 19
  3	 S 	   129.58	  1011.00	 18
  4	 E 	   194.10	   967.48	 17
  5	 K 	   258.14	   902.96	 16
  6	 E 	   322.67	   838.92	 15
  7	 V 	   372.20	   774.39	 14
  8	 D 	   429.71	   724.86	 13
  9	 S 	   473.23	   667.35	 12
 10	 G 	   501.74	   623.83	 11
 11	 N 	   558.76	   595.32	 10
 12	 D 	   616.28	   538.30	  9
 13	 I 	   672.82	   480.78	  8
 14	 Y 	   754.35	   424.24	  7
 15	 G 	   782.86	   342.71	  6
 16	 N 	   839.88	   314.20	  5
 17	 P 	   888.41	   257.18	  4
 18	 I 	   944.95	   208.65	  3
 19	 K 	  1009.00	   152.11	  2
 20	 R 	        -  	    88.06	  1  _

+3 charge
		      B   	       Y
  1	 A 	    24.69	        -  	 20
  2	 V 	    57.71	   707.36	 19
  3	 S 	    86.72	   674.34	 18
  4	 E 	   129.73	   645.33	 17
  5	 K 	   172.43	   602.31	 16
  6	 E 	   215.45	   559.61	 15
  7	 V 	   248.47	   516.60	 14
  8	 D 	   286.81	   483.58	 13
  9	 S 	   315.82	   445.23	 12
 10	 G 	   334.83	   416.22	 11
 11	 N 	   372.84	   397.22	 10
 12	 D 	   411.19	   359.20	  9
 13	 I 	   448.88	   320.86	  8
 14	 Y 	   503.24	   283.16	  7
 15	 G 	   522.24	   228.81	  6
 16	 N 	   560.26	   209.80	  5
 17	 P 	   592.61	   171.79	  4
 18	 I 	   630.30	   139.44	  3
 19	 K 	   673.00	   101.74	  2
 20	 R 	        -  	    59.04	  1  _
```

**54. IPI00061116 Mucin-like protein 1**

| Sequence | Charge | M+H+ | XCorr | DeltaCn |
| --- | --- | --- | --- | --- |
| K.WVGDLPNGRVCP.- | 2 | 1370.532 | 3.68 | 0.271 |

+1 charge

```
		      B   	       Y
  1	 W 	   187.09	        -  	 12
  2	 V 	   286.16	  1183.72	 11
  3	 G 	   343.18	  1084.65	 10
  4	 D 	   458.20	  1027.63	  9
  5	 L 	   571.29	   912.61	  8
  6	 P 	   668.34	   799.52	  7
  7	 N 	   782.38	   702.47	  6
  8	 G 	   839.40	   588.43	  5
  9	 R 	   995.51	   531.41	  4
 10	 V 	  1094.57	   375.30	  3
 11	 C 	  1254.74	   276.24	  2
 12	 P 	        -  	   116.07	  1  _
+2 charge
		      B   	       Y
  1	 W 	    94.05	        -  	 12
  2	 V 	   143.58	   592.37	 11
  3	 G 	   172.09	   542.83	 10
  4	 D 	   229.61	   514.32	  9
  5	 L 	   286.15	   456.81	  8
  6	 P 	   334.67	   400.26	  7
  7	 N 	   391.70	   351.74	  6
  8	 G 	   420.21	   294.72	  5
  9	 R 	   498.26	   266.21	  4
 10	 V 	   547.79	   188.16	  3
 11	 C 	   627.87	   138.62	  2
 12	 P 	        -  	    58.54	  1  _
```

**55. IPI00784119 V-type proton ATPase subunit S1**

| Sequence | Charge | M+H+ | XCorr | DeltaCn |
| --- | --- | --- | --- | --- |
| K.LGASPLHVDLATLR.E | 2 | 1463.707 | 3.501 | 0.434 |

+1 charge

```
		      B   	       Y
  1	 L 	   114.09	        -  	 14
  2	 G 	   171.11	  1349.75	 13
  3	 A 	   242.15	  1292.73	 12
  4	 S 	   329.18	  1221.69	 11
  5	 P 	   426.23	  1134.66	 10
  6	 L 	   539.32	  1037.61	  9
  7	 H 	   676.38	   924.53	  8
  8	 V 	   775.45	   787.47	  7
  9	 D 	   890.47	   688.40	  6
 10	 L 	  1003.56	   573.37	  5
 11	 A 	  1074.59	   460.29	  4
 12	 T 	  1175.64	   389.25	  3
 13	 L 	  1288.73	   288.20	  2
 14	 R 	        -  	   175.12	  1  _
+2 charge
		      B   	       Y
  1	 L 	    57.55	        -  	 14
  2	 G 	    86.06	   675.38	 13
  3	 A 	   121.58	   646.87	 12
  4	 S 	   165.09	   611.35	 11
  5	 P 	   213.62	   567.84	 10
  6	 L 	   270.16	   519.31	  9
  7	 H 	   338.69	   462.77	  8
  8	 V 	   388.23	   394.24	  7
  9	 D 	   445.74	   344.70	  6
 10	 L 	   502.28	   287.19	  5
 11	 A 	   537.80	   230.65	  4
 12	 T 	   588.32	   195.13	  3
 13	 L 	   644.87	   144.61	  2
 14	 R 	        -  	    88.06	  1  _
```

**56. IPI00746352 SH3 domain-binding glutamic acid-rich-like protein 3**

| Sequence | Charge | M+H+ | XCorr | DeltaCn |
| --- | --- | --- | --- | --- |
| K.RIQYQLVDISQDNALRDEMR.A | 3 | 2464.744 | 3.33 | 0.421 |

+1 charge

```
		      B   	       Y
  1	 R 	   157.11	        -  	 20
  2	 I 	   270.19	  2307.14	 19
  3	 Q 	   398.25	  2194.06	 18
  4	 Y 	   561.31	  2066.00	 17
  5	 Q 	   689.37	  1902.93	 16
  6	 L 	   802.46	  1774.88	 15
  7	 V 	   901.53	  1661.79	 14
  8	 D 	  1016.55	  1562.72	 13
  9	 I 	  1129.64	  1447.70	 12
 10	 S 	  1216.67	  1334.61	 11
 11	 Q 	  1344.73	  1247.58	 10
 12	 D 	  1459.75	  1119.52	  9
 13	 N 	  1573.80	  1004.49	  8
 14	 A 	  1644.83	   890.45	  7
 15	 L 	  1757.92	   819.41	  6
 16	 R 	  1914.02	   706.33	  5
 17	 D 	  2029.05	   550.23	  4
 18	 E 	  2158.09	   435.20	  3
 19	 M 	  2289.13	   306.16	  2
 20	 R 	        -  	   175.12	  1  _
+2 charge
		      B   	       Y
  1	 R 	    79.06	        -  	 20
  2	 I 	   135.60	  1154.07	 19
  3	 Q 	   199.63	  1097.53	 18
  4	 Y 	   281.16	  1033.50	 17
  5	 Q 	   345.19	   951.97	 16
  6	 L 	   401.73	   887.94	 15
  7	 V 	   451.27	   831.40	 14
  8	 D 	   508.78	   781.87	 13
  9	 I 	   565.32	   724.35	 12
 10	 S 	   608.84	   667.81	 11
 11	 Q 	   672.87	   624.29	 10
 12	 D 	   730.38	   560.26	  9
 13	 N 	   787.40	   502.75	  8
 14	 A 	   822.92	   445.73	  7
 15	 L 	   879.46	   410.21	  6
 16	 R 	   957.51	   353.67	  5
 17	 D 	  1015.03	   275.62	  4
 18	 E 	  1079.55	   218.10	  3
 19	 M 	  1145.07	   153.58	  2
 20	 R 	        -  	    88.06	  1  _

+3 charge
		      B   	       Y
  1	 R 	    53.04	        -  	 20
  2	 I 	    90.74	   769.72	 19
  3	 Q 	   133.42	   732.02	 18
  4	 Y 	   187.78	   689.34	 17
  5	 Q 	   230.46	   634.98	 16
  6	 L 	   268.16	   592.30	 15
  7	 V 	   301.18	   554.60	 14
  8	 D 	   339.52	   521.58	 13
  9	 I 	   377.22	   483.24	 12
 10	 S 	   406.23	   445.54	 11
 11	 Q 	   448.91	   416.53	 10
 12	 D 	   487.26	   373.85	  9
 13	 N 	   525.27	   335.50	  8
 14	 A 	   548.95	   297.49	  7
 15	 L 	   586.64	   273.81	  6
 16	 R 	   638.68	   236.11	  5
 17	 D 	   677.02	   184.08	  4
 18	 E 	   720.03	   145.74	  3
 19	 M 	   763.71	   102.72	  2
 20	 R 	        -  	    59.04	  1  _
```

**57. IPI00382478 Ig heavy chain V-III region TIL**

| Sequence | Charge | M+H+ | XCorr | DeltaCn |
| --- | --- | --- | --- | --- |
| - .EVQLLESGGGLVQPGGSLR.L | 2 | 1897.122 | 2.887 | 0.305 |

+1 charge

```
		      B   	       Y
  1	 E 	   130.05	        -  	 19
  2	 V 	   229.12	  1766.98	 18
  3	 Q 	   357.18	  1667.91	 17
  4	 L 	   470.26	  1539.85	 16
  5	 L 	   583.34	  1426.76	 15
  6	 E 	   712.39	  1313.68	 14
  7	 S 	   799.42	  1184.64	 13
  8	 G 	   856.44	  1097.61	 12
  9	 G 	   913.46	  1040.58	 11
 10	 G 	   970.48	   983.56	 10
 11	 L 	  1083.57	   926.54	  9
 12	 V 	  1182.64	   813.46	  8
 13	 Q 	  1310.70	   714.39	  7
 14	 P 	  1407.75	   586.33	  6
 15	 G 	  1464.77	   489.28	  5
 16	 G 	  1521.79	   432.26	  4
 17	 S 	  1608.82	   375.24	  3
 18	 L 	  1721.91	   288.20	  2
 19	 R 	        -  	   175.12	  1  _
+2 charge
		      B   	       Y
  1	 E 	    65.53	        -  	 19
  2	 V 	   115.06	   883.99	 18
  3	 Q 	   179.09	   834.46	 17
  4	 L 	   235.63	   770.43	 16
  5	 L 	   292.18	   713.89	 15
  6	 E 	   356.70	   657.34	 14
  7	 S 	   400.21	   592.82	 13
  8	 G 	   428.72	   549.31	 12
  9	 G 	   457.23	   520.80	 11
 10	 G 	   485.75	   492.29	 10
 11	 L 	   542.29	   463.77	  9
 12	 V 	   591.82	   407.23	  8
 13	 Q 	   655.85	   357.70	  7
 14	 P 	   704.38	   293.67	  6
 15	 G 	   732.89	   245.14	  5
 16	 G 	   761.40	   216.63	  4
 17	 S 	   804.92	   188.12	  3
 18	 L 	   861.46	   144.61	  2
 19	 R 	        -  	    88.06	  1  _
```

**58. IPI00470361 Isoform 2 of Kin of IRRE-like protein 1**

| Sequence | Charge | M+H+ | XCorr | DeltaCn |
| --- | --- | --- | --- | --- |
| K.DGLALGMGQGLKAWPR.Y | 2 | 1670.961 | 3.688 | 0.471 |

+1 charge

```
		      B   	       Y
  1	 D 	   116.03	        -  	 16
  2	 G 	   173.06	  1554.86	 15
  3	 L 	   286.14	  1497.84	 14
  4	 A 	   357.18	  1384.75	 13
  5	 L 	   470.26	  1313.71	 12
  6	 G 	   527.28	  1200.63	 11
  7	 M 	   658.32	  1143.61	 10
  8	 G 	   715.34	  1012.57	  9
  9	 Q 	   843.40	   955.55	  8
 10	 G 	   900.42	   827.49	  7
 11	 L 	  1013.51	   770.47	  6
 12	 K 	  1141.60	   657.38	  5
 13	 A 	  1212.64	   529.29	  4
 14	 W 	  1398.72	   458.25	  3
 15	 P 	  1495.77	   272.17	  2
 16	 R 	        -  	   175.12	  1  _
+2 charge
		      B   	       Y
  1	 D 	    58.52	        -  	 16
  2	 G 	    87.03	   777.93	 15
  3	 L 	   143.57	   749.42	 14
  4	 A 	   179.09	   692.88	 13
  5	 L 	   235.63	   657.36	 12
  6	 G 	   264.14	   600.82	 11
  7	 M 	   329.67	   572.31	 10
  8	 G 	   358.18	   506.79	  9
  9	 Q 	   422.21	   478.28	  8
 10	 G 	   450.72	   414.25	  7
 11	 L 	   507.26	   385.74	  6
 12	 K 	   571.31	   329.20	  5
 13	 A 	   606.82	   265.15	  4
 14	 W 	   699.86	   229.63	  3
 15	 P 	   748.39	   136.59	  2
 16	 R 	        -  	    88.06	  1  _
```

**59. IPI00072044 Isoform 1 of Ester hydrolase C11orf54**

| Sequence | Charge | M+H+ | XCorr | DeltaCn |
| --- | --- | --- | --- | --- |
| R.IAEVGGVPYLLPLVNQK.K | 2 | 1811.157 | 4.17 | 0.456 |

+1 charge

```
		      B   	       Y
  1	 I 	   114.09	        -  	 17
  2	 A 	   185.13	  1696.96	 16
  3	 E 	   314.17	  1625.93	 15
  4	 V 	   413.24	  1496.88	 14
  5	 G 	   470.26	  1397.82	 13
  6	 G 	   527.28	  1340.79	 12
  7	 V 	   626.35	  1283.77	 11
  8	 P 	   723.40	  1184.70	 10
  9	 Y 	   886.47	  1087.65	  9
 10	 L 	   999.55	   924.59	  8
 11	 L 	  1112.64	   811.50	  7
 12	 P 	  1209.69	   698.42	  6
 13	 L 	  1322.77	   601.37	  5
 14	 V 	  1421.84	   488.28	  4
 15	 N 	  1535.88	   389.21	  3
 16	 Q 	  1663.94	   275.17	  2
 17	 K 	        -  	   147.11	  1  _
+2 charge
		      B   	       Y
  1	 I 	    57.55	        -  	 17
  2	 A 	    93.07	   848.99	 16
  3	 E 	   157.59	   813.47	 15
  4	 V 	   207.12	   748.95	 14
  5	 G 	   235.63	   699.41	 13
  6	 G 	   264.14	   670.90	 12
  7	 V 	   313.68	   642.39	 11
  8	 P 	   362.21	   592.86	 10
  9	 Y 	   443.74	   544.33	  9
 10	 L 	   500.28	   462.80	  8
 11	 L 	   556.82	   406.26	  7
 12	 P 	   605.35	   349.71	  6
 13	 L 	   661.89	   301.19	  5
 14	 V 	   711.42	   244.64	  4
 15	 N 	   768.45	   195.11	  3
 16	 Q 	   832.47	   138.09	  2
 17	 K 	        -  	    74.06	  1  _
```

**60. IPI00007797 Fatty acid-binding protein, epidermal**

| Sequence | Charge | M+H+ | XCorr | DeltaCn |
| --- | --- | --- | --- | --- |
| K.GFDEYMKELGVGIALR.K | 2 | 1799.084 | 3.557 | 0.47 |

+1 charge

```
		      B   	       Y
  1	 G 	    58.03	        -  	 16
  2	 F 	   205.10	  1740.90	 15
  3	 D 	   320.12	  1593.83	 14
  4	 E 	   449.17	  1478.80	 13
  5	 Y 	   612.23	  1349.76	 12
  6	 M 	   743.27	  1186.70	 11
  7	 K 	   871.37	  1055.66	 10
  8	 E 	  1000.41	   927.56	  9
  9	 L 	  1113.49	   798.52	  8
 10	 G 	  1170.51	   685.44	  7
 11	 V 	  1269.58	   628.41	  6
 12	 G 	  1326.60	   529.35	  5
 13	 I 	  1439.69	   472.32	  4
 14	 A 	  1510.72	   359.24	  3
 15	 L 	  1623.81	   288.20	  2
 16	 R 	        -  	   175.12	  1  _
+2 charge
		      B   	       Y
  1	 G 	    29.52	        -  	 16
  2	 F 	   103.05	   870.95	 15
  3	 D 	   160.57	   797.42	 14
  4	 E 	   225.09	   739.91	 13
  5	 Y 	   306.62	   675.38	 12
  6	 M 	   372.14	   593.85	 11
  7	 K 	   436.19	   528.33	 10
  8	 E 	   500.71	   464.28	  9
  9	 L 	   557.25	   399.76	  8
 10	 G 	   585.76	   343.22	  7
 11	 V 	   635.29	   314.71	  6
 12	 G 	   663.81	   265.18	  5
 13	 I 	   720.35	   236.67	  4
 14	 A 	   755.87	   180.12	  3
 15	 L 	   812.41	   144.61	  2
 16	 R 	        -  	    88.06	  1  _
```

**61. IPI00219037 Histone H2A.x**

| Sequence | Charge | M+H+ | XCorr | DeltaCn |
| --- | --- | --- | --- | --- |
| R.VTIAQGGVLPNIQAVLLPK.K | 2 | 1932.339 | 3.947 | 0.457 |

+1 charge

```
		      B   	       Y
  1	 V 	   100.08	        -  	 19
  2	 T 	   201.12	  1832.10	 18
  3	 I 	   314.21	  1731.05	 17
  4	 A 	   385.24	  1617.97	 16
  5	 Q 	   513.30	  1546.93	 15
  6	 G 	   570.32	  1418.87	 14
  7	 G 	   627.35	  1361.85	 13
  8	 V 	   726.41	  1304.83	 12
  9	 L 	   839.50	  1205.76	 11
 10	 P 	   936.55	  1092.68	 10
 11	 N 	  1050.59	   995.62	  9
 12	 I 	  1163.68	   881.58	  8
 13	 Q 	  1291.74	   768.50	  7
 14	 A 	  1362.77	   640.44	  6
 15	 V 	  1461.84	   569.40	  5
 16	 L 	  1574.93	   470.33	  4
 17	 L 	  1688.01	   357.25	  3
 18	 P 	  1785.06	   244.17	  2
 19	 K 	        -  	   147.11	  1  _
+2 charge
		      B   	       Y
  1	 V 	    50.54	        -  	 19
  2	 T 	   101.07	   916.55	 18
  3	 I 	   157.61	   866.03	 17
  4	 A 	   193.13	   809.49	 16
  5	 Q 	   257.16	   773.97	 15
  6	 G 	   285.67	   709.94	 14
  7	 G 	   314.18	   681.43	 13
  8	 V 	   363.71	   652.92	 12
  9	 L 	   420.25	   603.38	 11
 10	 P 	   468.78	   546.84	 10
 11	 N 	   525.80	   498.32	  9
 12	 I 	   582.34	   441.29	  8
 13	 Q 	   646.37	   384.75	  7
 14	 A 	   681.89	   320.72	  6
 15	 V 	   731.42	   285.20	  5
 16	 L 	   787.97	   235.67	  4
 17	 L 	   844.51	   179.13	  3
 18	 P 	   893.04	   122.59	  2
 19	 K 	        -  	    74.06	  1  _
```

**62. IPI00873924 Isoform A of Interleukin-18-binding protein**

| Sequence | Charge | M+H+ | XCorr | DeltaCn |  |
| --- | --- | --- | --- | --- | --- |
| K.ALVLEQLTPALHSTNFSCVLVDPEQVVQR.H | 3 | 3265.699 | 5.328 | 0.57 |  |

+1 charge

```
		      B   	       Y
  1	 A 	    72.04	        -  	 29
  2	 L 	   185.13	  3192.81	 28
  3	 V 	   284.20	  3079.72	 27
  4	 L 	   397.28	  2980.65	 26
  5	 E 	   526.32	  2867.57	 25
  6	 Q 	   654.38	  2738.53	 24
  7	 L 	   767.47	  2610.47	 23
  8	 T 	   868.51	  2497.38	 22
  9	 P 	   965.57	  2396.34	 21
 10	 A 	  1036.60	  2299.28	 20
 11	 L 	  1149.69	  2228.25	 19
 12	 H 	  1286.75	  2115.16	 18
 13	 S 	  1373.78	  1978.10	 17
 14	 T 	  1474.83	  1891.07	 16
 15	 N 	  1588.87	  1790.02	 15
 16	 F 	  1735.94	  1675.98	 14
 17	 S 	  1822.97	  1528.91	 13
 18	 C 	  1983.14	  1441.88	 12
 19	 V 	  2082.20	  1281.72	 11
 20	 L 	  2195.29	  1182.65	 10
 21	 V 	  2294.36	  1069.56	  9
 22	 D 	  2409.38	   970.50	  8
 23	 P 	  2506.44	   855.47	  7
 24	 E 	  2635.48	   758.42	  6
 25	 Q 	  2763.54	   629.37	  5
 26	 V 	  2862.61	   501.31	  4
 27	 V 	  2961.67	   402.25	  3
 28	 Q 	  3089.73	   303.18	  2
 29	 R 	        -  	   175.12	  1  _


+2 charge
		      B   	       Y
  1	 A 	    36.53	        -  	 29
  2	 L 	    93.07	  1596.91	 28
  3	 V 	   142.60	  1540.36	 27
  4	 L 	   199.14	  1490.83	 26
  5	 E 	   263.67	  1434.29	 25
  6	 Q 	   327.69	  1369.77	 24
  7	 L 	   384.24	  1305.74	 23
  8	 T 	   434.76	  1249.20	 22
  9	 P 	   483.29	  1198.67	 21
 10	 A 	   518.81	  1150.15	 20
 11	 L 	   575.35	  1114.63	 19
 12	 H 	   643.88	  1058.09	 18
 13	 S 	   687.39	   989.56	 17
 14	 T 	   737.92	   946.04	 16
 15	 N 	   794.94	   895.52	 15
 16	 F 	   868.47	   838.49	 14
 17	 S 	   911.99	   764.96	 13
 18	 C 	   992.07	   721.44	 12
 19	 V 	  1041.61	   641.36	 11
 20	 L 	  1098.15	   591.83	 10
 21	 V 	  1147.68	   535.29	  9
 22	 D 	  1205.20	   485.75	  8
 23	 P 	  1253.72	   428.24	  7
 24	 E 	  1318.24	   379.71	  6
 25	 Q 	  1382.27	   315.19	  5
 26	 V 	  1431.81	   251.16	  4
 27	 V 	  1481.34	   201.63	  3
 28	 Q 	  1545.37	   152.09	  2
 29	 R 	        -  	    88.06	  1  _


+3 charge
		      B   	       Y
  1	 A 	    24.69	        -  	 29
  2	 L 	    62.38	  1064.94	 28
  3	 V 	    95.40	  1027.25	 27
  4	 L 	   133.10	   994.22	 26
  5	 E 	   176.11	   956.53	 25
  6	 Q 	   218.80	   913.51	 24
  7	 L 	   256.49	   870.83	 23
  8	 T 	   290.18	   833.13	 22
  9	 P 	   322.53	   799.45	 21
 10	 A 	   346.21	   767.10	 20
 11	 L 	   383.90	   743.42	 19
 12	 H 	   429.59	   705.73	 18
 13	 S 	   458.60	   660.04	 17
 14	 T 	   492.28	   631.03	 16
 15	 N 	   530.29	   597.35	 15
 16	 F 	   579.32	   559.33	 14
 17	 S 	   608.33	   510.31	 13
 18	 C 	   661.72	   481.30	 12
 19	 V 	   694.74	   427.91	 11
 20	 L 	   732.43	   394.89	 10
 21	 V 	   765.46	   357.19	  9
 22	 D 	   803.80	   324.17	  8
 23	 P 	   836.15	   285.83	  7
 24	 E 	   879.16	   253.48	  6
 25	 Q 	   921.85	   210.46	  5
 26	 V 	   954.87	   167.78	  4
 27	 V 	   987.90	   134.75	  3
 28	 Q 	  1030.58	   101.73	  2
 29	 R 	        -  	    59.04	  1  _
```

**63. IPI00303161 Endothelial cell-selective adhesion molecule**

| Sequence | Charge | M+H+ | XCorr | DeltaCn |
| --- | --- | --- | --- | --- |
| R.QLPSFQTFFAPALDVIR.G | 2 | 1951.257 | 4.12 | 0.603 |

+1 charge

```
		      B   	       Y
  1	 Q 	   129.07	        -  	 17
  2	 L 	   242.15	  1821.99	 16
  3	 P 	   339.20	  1708.91	 15
  4	 S 	   426.23	  1611.85	 14
  5	 F 	   573.30	  1524.82	 13
  6	 Q 	   701.36	  1377.75	 12
  7	 T 	   802.41	  1249.69	 11
  8	 F 	   949.48	  1148.65	 10
  9	 F 	  1096.55	  1001.58	  9
 10	 A 	  1167.58	   854.51	  8
 11	 P 	  1264.64	   783.47	  7
 12	 A 	  1335.67	   686.42	  6
 13	 L 	  1448.76	   615.38	  5
 14	 D 	  1563.78	   502.30	  4
 15	 V 	  1662.85	   387.27	  3
 16	 I 	  1775.94	   288.20	  2
 17	 R 	        -  	   175.12	  1  _
+2 charge
		      B   	       Y
  1	 Q 	    65.04	        -  	 17
  2	 L 	   121.58	   911.50	 16
  3	 P 	   170.10	   854.96	 15
  4	 S 	   213.62	   806.43	 14
  5	 F 	   287.16	   762.91	 13
  6	 Q 	   351.18	   689.38	 12
  7	 T 	   401.71	   625.35	 11
  8	 F 	   475.24	   574.83	 10
  9	 F 	   548.78	   501.29	  9
 10	 A 	   584.30	   427.76	  8
 11	 P 	   632.82	   392.24	  7
 12	 A 	   668.34	   343.71	  6
 13	 L 	   724.88	   308.19	  5
 14	 D 	   782.40	   251.65	  4
 15	 V 	   831.93	   194.14	  3
 16	 I 	   888.47	   144.61	  2
 17	 R 	        -  	    88.06	  1  _
```

**64. IPI00006988 Resistin**

| Sequence | Charge | M+H+ | XCorr | DeltaCn |
| --- | --- | --- | --- | --- |
| K.TLCSMEEAINERIQEVAGSLIFR.A | 3 | 2668.014 | 4.647 | 0.543 |

+1 charge

```
		      B   	       Y
  1	 T 	   102.05	        -  	 23
  2	 L 	   215.14	  2565.41	 22
  3	 C 	   375.30	  2452.33	 21
  4	 S 	   462.34	  2292.17	 20
  5	 M 	   593.38	  2205.13	 19
  6	 E 	   722.42	  2074.09	 18
  7	 E 	   851.46	  1945.05	 17
  8	 A 	   922.50	  1816.01	 16
  9	 I 	  1035.58	  1744.97	 15
 10	 N 	  1149.63	  1631.89	 14
 11	 E 	  1278.67	  1517.84	 13
 12	 R 	  1434.77	  1388.80	 12
 13	 I 	  1547.85	  1232.70	 11
 14	 Q 	  1675.91	  1119.62	 10
 15	 E 	  1804.95	   991.56	  9
 16	 V 	  1904.02	   862.51	  8
 17	 A 	  1975.06	   763.45	  7
 18	 G 	  2032.08	   692.41	  6
 19	 S 	  2119.11	   635.39	  5
 20	 L 	  2232.20	   548.36	  4
 21	 I 	  2345.28	   435.27	  3
 22	 F 	  2492.35	   322.19	  2
 23	 R 	        -  	   175.12	  1  _


+2 charge
		      B   	       Y
  1	 T 	    51.53	        -  	 23
  2	 L 	   108.07	  1283.21	 22
  3	 C 	   188.16	  1226.67	 21
  4	 S 	   231.67	  1146.59	 20
  5	 M 	   297.19	  1103.07	 19
  6	 E 	   361.71	  1037.55	 18
  7	 E 	   426.23	   973.03	 17
  8	 A 	   461.75	   908.51	 16
  9	 I 	   518.30	   872.99	 15
 10	 N 	   575.32	   816.45	 14
 11	 E 	   639.84	   759.43	 13
 12	 R 	   717.89	   694.90	 12
 13	 I 	   774.43	   616.85	 11
 14	 Q 	   838.46	   560.31	 10
 15	 E 	   902.98	   496.28	  9
 16	 V 	   952.52	   431.76	  8
 17	 A 	   988.03	   382.23	  7
 18	 G 	  1016.54	   346.71	  6
 19	 S 	  1060.06	   318.20	  5
 20	 L 	  1116.60	   274.68	  4
 21	 I 	  1173.14	   218.14	  3
 22	 F 	  1246.68	   161.60	  2
 23	 R 	        -  	    88.06	  1  _


+3 charge
		      B   	       Y
  1	 T 	    34.69	        -  	 23
  2	 L 	    72.38	   855.81	 22
  3	 C 	   125.77	   818.12	 21
  4	 S 	   154.78	   764.73	 20
  5	 M 	   198.46	   735.72	 19
  6	 E 	   241.48	   692.04	 18
  7	 E 	   284.49	   649.02	 17
  8	 A 	   308.17	   606.01	 16
  9	 I 	   345.87	   582.33	 15
 10	 N 	   383.88	   544.63	 14
 11	 E 	   426.89	   506.62	 13
 12	 R 	   478.93	   463.61	 12
 13	 I 	   516.62	   411.57	 11
 14	 Q 	   559.31	   373.88	 10
 15	 E 	   602.32	   331.19	  9
 16	 V 	   635.35	   288.18	  8
 17	 A 	   659.03	   255.15	  7
 18	 G 	   678.03	   231.47	  6
 19	 S 	   707.04	   212.47	  5
 20	 L 	   744.74	   183.46	  4
 21	 I 	   782.43	   145.76	  3
 22	 F 	   831.46	   108.07	  2
 23	 R 	        -  	    59.04	  1  _
```

**65. IPI00028347 Isoform 1 of F-box only protein 31**

| Sequence | Charge | M+H+ | XCorr | DeltaCn |
| --- | --- | --- | --- | --- |
| R.IYLPPSRPDDLIKPGLFK.G | 2 | 2070.462 | 3.024 | 0.239 |

+1 charge

```
		      B   	       Y
  1	 I 	   114.09	        -  	 18
  2	 Y 	   277.15	  1956.10	 17
  3	 L 	   390.24	  1793.03	 16
  4	 P 	   487.29	  1679.95	 15
  5	 P 	   584.34	  1582.90	 14
  6	 S 	   671.38	  1485.84	 13
  7	 R 	   827.48	  1398.81	 12
  8	 P 	   924.53	  1242.71	 11
  9	 D 	  1039.56	  1145.66	 10
 10	 D 	  1154.58	  1030.63	  9
 11	 L 	  1267.67	   915.60	  8
 12	 I 	  1380.75	   802.52	  7
 13	 K 	  1508.85	   689.43	  6
 14	 P 	  1605.90	   561.34	  5
 15	 G 	  1662.92	   464.29	  4
 16	 L 	  1776.01	   407.27	  3
 17	 F 	  1923.07	   294.18	  2
 18	 K 	        -  	   147.11	  1  _
+2 charge
		      B   	       Y
  1	 I 	    57.55	        -  	 18
  2	 Y 	   139.08	   978.55	 17
  3	 L 	   195.62	   897.02	 16
  4	 P 	   244.15	   840.48	 15
  5	 P 	   292.68	   791.95	 14
  6	 S 	   336.19	   743.42	 13
  7	 R 	   414.24	   699.91	 12
  8	 P 	   462.77	   621.86	 11
  9	 D 	   520.28	   573.33	 10
 10	 D 	   577.80	   515.82	  9
 11	 L 	   634.34	   458.30	  8
 12	 I 	   690.88	   401.76	  7
 13	 K 	   754.93	   345.22	  6
 14	 P 	   803.45	   281.17	  5
 15	 G 	   831.96	   232.65	  4
 16	 L 	   888.51	   204.14	  3
 17	 F 	   962.04	   147.59	  2
 18	 K 	        -  	    74.06	  1  _
```

**66. IPI00007221 Plasma serine protease inhibitor**

| Sequence | Charge | M+H+ | XCorr | DeltaCn |
| --- | --- | --- | --- | --- |
| R.AAAATGTIFTFR.S | 2 | 1227.394 | 2.894 | 0.38 |

+1 charge

```
		      B   	       Y
  1	 A 	    72.04	        -  	 12
  2	 A 	   143.08	  1155.62	 11
  3	 A 	   214.12	  1084.58	 10
  4	 A 	   285.16	  1013.54	  9
  5	 T 	   386.20	   942.50	  8
  6	 G 	   443.22	   841.46	  7
  7	 T 	   544.27	   784.44	  6
  8	 I 	   657.36	   683.39	  5
  9	 F 	   804.43	   570.30	  4
 10	 T 	   905.47	   423.24	  3
 11	 F 	  1052.54	   322.19	  2
 12	 R 	        -  	   175.12	  1  _
+2 charge
		      B   	       Y
  1	 A 	    36.53	        -  	 12
  2	 A 	    72.04	   578.31	 11
  3	 A 	   107.56	   542.79	 10
  4	 A 	   143.08	   507.27	  9
  5	 T 	   193.61	   471.76	  8
  6	 G 	   222.12	   421.23	  7
  7	 T 	   272.64	   392.72	  6
  8	 I 	   329.18	   342.20	  5
  9	 F 	   402.72	   285.66	  4
 10	 T 	   453.24	   212.12	  3
 11	 F 	   526.77	   161.60	  2
 12	 R 	        -  	    88.06	  1  _
```

**67. IPI00013446 Prostate stem cell antigen**

| Sequence | Charge | M+H+ | XCorr | DeltaCn |
| --- | --- | --- | --- | --- |
| R.AVGLLTVISK.G | 2 | 1001.245 | 2.837 | 0.279 |

+1 charge

```
		      B   	       Y
  1	 A 	    72.04	        -  	 10
  2	 V 	   171.11	   929.60	  9
  3	 G 	   228.13	   830.53	  8
  4	 L 	   341.22	   773.51	  7
  5	 L 	   454.30	   660.43	  6
  6	 T 	   555.35	   547.34	  5
  7	 V 	   654.42	   446.30	  4
  8	 I 	   767.50	   347.23	  3
  9	 S 	   854.53	   234.14	  2
 10	 K 	        -  	   147.11	  1  _
+2 charge
		      B   	       Y
  1	 A 	    36.53	        -  	 10
  2	 V 	    86.06	   465.31	  9
  3	 G 	   114.57	   415.77	  8
  4	 L 	   171.11	   387.26	  7
  5	 L 	   227.65	   330.72	  6
  6	 T 	   278.18	   274.18	  5
  7	 V 	   327.71	   223.65	  4
  8	 I 	   384.25	   174.12	  3
  9	 S 	   427.77	   117.58	  2
 10	 K 	        -  	    74.06	  1  _
```

**68. IPI00297420 Frizzled-4 precursor**

| Sequence | Charge | M+H+ | XCorr | DeltaCn |
| --- | --- | --- | --- | --- |
| K.INIPIGPCGGMCLSVKR.R | 2 | 1873.241 | 2.914 | 0.429 |

+1 charge

```
		      B   	       Y
  1	 I 	   114.09	        -  	 17
  2	 N 	   228.13	  1759.15	 16
  3	 I 	   341.22	  1645.11	 15
  4	 P 	   438.27	  1532.02	 14
  5	 I 	   551.36	  1434.97	 13
  6	 G 	   608.38	  1321.89	 12
  7	 P 	   705.43	  1264.87	 11
  8	 C 	   865.59	  1167.81	 10
  9	 G 	   922.62	  1007.65	  9
 10	 G 	   979.64	   950.63	  8
 11	 M 	  1110.68	   893.60	  7
 12	 C 	  1270.84	   762.56	  6
 13	 L 	  1383.93	   602.40	  5
 14	 S 	  1470.96	   489.31	  4
 15	 V 	  1570.03	   402.28	  3
 16	 K 	  1698.12	   303.21	  2
 17	 R 	        -  	   175.12	  1  _
+2 charge
		      B   	       Y
  1	 I 	    57.55	        -  	 17
  2	 N 	   114.57	   880.08	 16
  3	 I 	   171.11	   823.06	 15
  4	 P 	   219.64	   766.52	 14
  5	 I 	   276.18	   717.99	 13
  6	 G 	   304.69	   661.45	 12
  7	 P 	   353.22	   632.94	 11
  8	 C 	   433.30	   584.41	 10
  9	 G 	   461.81	   504.33	  9
 10	 G 	   490.32	   475.82	  8
 11	 M 	   555.84	   447.31	  7
 12	 C 	   635.93	   381.79	  6
 13	 L 	   692.47	   301.70	  5
 14	 S 	   735.98	   245.16	  4
 15	 V 	   785.52	   201.64	  3
 16	 K 	   849.57	   152.11	  2
 17	 R 	        -  	    88.06	  1  _
```

**69. IPI00001754 Junctional adhesion molecule A**

| Sequence | Charge | M+H+ | XCorr | DeltaCn |
| --- | --- | --- | --- | --- |
| K.LIVLVPPSKPTVNIPSSATIGNR.A | 3 | 2374.808 | 3.42 | 0.43 |

+1 charge

```
		      B   	       Y
  1	 L 	   114.09	        -  	 23
  2	 I 	   227.18	  2260.30	 22
  3	 V 	   326.24	  2147.22	 21
  4	 L 	   439.33	  2048.15	 20
  5	 V 	   538.40	  1935.07	 19
  6	 P 	   635.45	  1836.00	 18
  7	 P 	   732.50	  1738.94	 17
  8	 S 	   819.53	  1641.89	 16
  9	 K 	   947.63	  1554.86	 15
 10	 P 	  1044.68	  1426.76	 14
 11	 T 	  1145.73	  1329.71	 13
 12	 V 	  1244.80	  1228.66	 12
 13	 N 	  1358.84	  1129.60	 11
 14	 I 	  1471.92	  1015.55	 10
 15	 P 	  1568.98	   902.47	  9
 16	 S 	  1656.01	   805.42	  8
 17	 S 	  1743.04	   718.38	  7
 18	 A 	  1814.08	   631.35	  6
 19	 T 	  1915.13	   560.32	  5
 20	 I 	  2028.21	   459.27	  4
 21	 G 	  2085.23	   346.18	  3
 22	 N 	  2199.27	   289.16	  2
 23	 R 	        -  	   175.12	  1  _


+2 charge
		      B   	       Y
  1	 L 	    57.55	        -  	 23
  2	 I 	   114.09	  1130.65	 22
  3	 V 	   163.63	  1074.11	 21
  4	 L 	   220.17	  1024.58	 20
  5	 V 	   269.70	   968.04	 19
  6	 P 	   318.23	   918.50	 18
  7	 P 	   366.75	   869.98	 17
  8	 S 	   410.27	   821.45	 16
  9	 K 	   474.32	   777.93	 15
 10	 P 	   522.84	   713.89	 14
 11	 T 	   573.37	   665.36	 13
 12	 V 	   622.90	   614.84	 12
 13	 N 	   679.92	   565.30	 11
 14	 I 	   736.47	   508.28	 10
 15	 P 	   784.99	   451.74	  9
 16	 S 	   828.51	   403.21	  8
 17	 S 	   872.02	   359.70	  7
 18	 A 	   907.54	   316.18	  6
 19	 T 	   958.07	   280.66	  5
 20	 I 	  1014.61	   230.14	  4
 21	 G 	  1043.12	   173.60	  3
 22	 N 	  1100.14	   145.08	  2
 23	 R 	        -  	    88.06	  1  _
+3 charge
		      B   	       Y
  1	 L 	    38.70	        -  	 23
  2	 I 	    76.40	   754.11	 22
  3	 V 	   109.42	   716.41	 21
  4	 L 	   147.11	   683.39	 20
  5	 V 	   180.14	   645.69	 19
  6	 P 	   212.49	   612.67	 18
  7	 P 	   244.84	   580.32	 17
  8	 S 	   273.85	   547.97	 16
  9	 K 	   316.55	   518.96	 15
 10	 P 	   348.90	   476.26	 14
 11	 T 	   382.58	   443.91	 13
 12	 V 	   415.60	   410.23	 12
 13	 N 	   453.62	   377.20	 11
 14	 I 	   491.31	   339.19	 10
 15	 P 	   523.66	   301.49	  9
 16	 S 	   552.67	   269.14	  8
 17	 S 	   581.69	   240.13	  7
 18	 A 	   605.36	   211.12	  6
 19	 T 	   639.05	   187.44	  5
 20	 I 	   676.74	   153.76	  4
 21	 G 	   695.75	   116.07	  3
 22	 N 	   733.76	    97.06	  2
 23	 R 	        -  	    59.04	  1  _
```

**70. IPI00025861 Cadherin-1**

| Sequence | Charge | M+H+ | XCorr | DeltaCn |
| --- | --- | --- | --- | --- |
| K.MALEVGDYKINLK.L | 2 | 1494.781 | 3.809 | 0.359 |

+1 charge

```
		      B   	       Y
  1	 M 	   132.05	        -  	 13
  2	 A 	   203.08	  1362.76	 12
  3	 L 	   316.17	  1291.73	 11
  4	 E 	   445.21	  1178.64	 10
  5	 V 	   544.28	  1049.60	  9
  6	 G 	   601.30	   950.53	  8
  7	 D 	   716.33	   893.51	  7
  8	 Y 	   879.39	   778.48	  6
  9	 K 	  1007.49	   615.42	  5
 10	 I 	  1120.57	   487.32	  4
 11	 N 	  1234.61	   374.24	  3
 12	 L 	  1347.70	   260.20	  2
 13	 K 	        -  	   147.11	  1  _
+2 charge
		      B   	       Y
  1	 M 	    66.53	        -  	 13
  2	 A 	   102.05	   681.89	 12
  3	 L 	   158.59	   646.37	 11
  4	 E 	   223.11	   589.82	 10
  5	 V 	   272.64	   525.30	  9
  6	 G 	   301.15	   475.77	  8
  7	 D 	   358.67	   447.26	  7
  8	 Y 	   440.20	   389.74	  6
  9	 K 	   504.25	   308.21	  5
 10	 I 	   560.79	   244.17	  4
 11	 N 	   617.81	   187.62	  3
 12	 L 	   674.35	   130.60	  2
 13	 K 	        -  	    74.06	  1  _
```

**71. IPI00216065 Isoform 2 of Vitamin K-dependent protein Z**

| Sequence | Charge | M+H+ | XCorr | DeltaCn |
| --- | --- | --- | --- | --- |
| R.APDLQDLPWQVK.L | 2 | 1410.599 | 3.048 | 0.389 |

+1 charge

```
		      B   	       Y
  1	 A 	    72.04	        -  	 12
  2	 P 	   169.10	  1338.71	 11
  3	 D 	   284.12	  1241.65	 10
  4	 L 	   397.21	  1126.63	  9
  5	 Q 	   525.27	  1013.54	  8
  6	 D 	   640.29	   885.48	  7
  7	 L 	   753.38	   770.46	  6
  8	 P 	   850.43	   657.37	  5
  9	 W 	  1036.51	   560.32	  4
 10	 Q 	  1164.57	   374.24	  3
 11	 V 	  1263.64	   246.18	  2
 12	 K 	        -  	   147.11	  1  _
+2 charge
		      B   	       Y
  1	 A 	    36.53	        -  	 12
  2	 P 	    85.05	   669.86	 11
  3	 D 	   142.57	   621.33	 10
  4	 L 	   199.11	   563.82	  9
  5	 Q 	   263.14	   507.27	  8
  6	 D 	   320.65	   443.25	  7
  7	 L 	   377.19	   385.73	  6
  8	 P 	   425.72	   329.19	  5
  9	 W 	   518.76	   280.66	  4
 10	 Q 	   582.79	   187.62	  3
 11	 V 	   632.32	   123.59	  2
 12	 K 	        -  	    74.06	  1  _
```

**72. IPI00022620 Secreted Ly-6/uPAR-related protein 1**

| Sequence | Charge | M+H+ | XCorr | DeltaCn |
| --- | --- | --- | --- | --- |
| R.SCSSSCVATDPDSIGAAHLIFCCFR.D | 3 | 2820.036 | 5.166 | 0.502 |

+1 charge

```
		      B   	       Y
  1	 S 	    88.04	        -  	 25
  2	 C 	   248.20	  2731.71	 24
  3	 S 	   335.24	  2571.55	 23
  4	 S 	   422.27	  2484.51	 22
  5	 S 	   509.30	  2397.48	 21
  6	 C 	   669.47	  2310.45	 20
  7	 V 	   768.53	  2150.28	 19
  8	 A 	   839.57	  2051.22	 18
  9	 T 	   940.62	  1980.18	 17
 10	 D 	  1055.65	  1879.13	 16
 11	 P 	  1152.70	  1764.10	 15
 12	 D 	  1267.73	  1667.05	 14
 13	 S 	  1354.76	  1552.03	 13
 14	 I 	  1467.84	  1464.99	 12
 15	 G 	  1524.86	  1351.91	 11
 16	 A 	  1595.90	  1294.89	 10
 17	 A 	  1666.94	  1223.85	  9
 18	 H 	  1804.00	  1152.81	  8
 19	 L 	  1917.08	  1015.75	  7
 20	 I 	  2030.16	   902.67	  6
 21	 F 	  2177.23	   789.59	  5
 22	 C 	  2337.40	   642.52	  4
 23	 C 	  2497.56	   482.35	  3
 24	 F 	  2644.63	   322.19	  2
 25	 R 	        -  	   175.12	  1  _


+2 charge
		      B   	       Y
  1	 S 	    44.52	        -  	 25
  2	 C 	   124.61	  1366.36	 24
  3	 S 	   168.12	  1286.28	 23
  4	 S 	   211.64	  1242.76	 22
  5	 S 	   255.15	  1199.24	 21
  6	 C 	   335.24	  1155.73	 20
  7	 V 	   384.77	  1075.65	 19
  8	 A 	   420.29	  1026.11	 18
  9	 T 	   470.81	   990.59	 17
 10	 D 	   528.33	   940.07	 16
 11	 P 	   576.85	   882.56	 15
 12	 D 	   634.37	   834.03	 14
 13	 S 	   677.88	   776.52	 13
 14	 I 	   734.42	   733.00	 12
 15	 G 	   762.94	   676.46	 11
 16	 A 	   798.45	   647.95	 10
 17	 A 	   833.97	   612.43	  9
 18	 H 	   902.50	   576.91	  8
 19	 L 	   959.04	   508.38	  7
 20	 I 	  1015.59	   451.84	  6
 21	 F 	  1089.12	   395.30	  5
 22	 C 	  1169.20	   321.76	  4
 23	 C 	  1249.29	   241.68	  3
 24	 F 	  1322.82	   161.60	  2
 25	 R 	        -  	    88.06	  1  _


+3 charge
		      B   	       Y
  1	 S 	    30.02	        -  	 25
  2	 C 	    83.41	   911.24	 24
  3	 S 	   112.42	   857.85	 23
  4	 S 	   141.43	   828.84	 22
  5	 S 	   170.44	   799.83	 21
  6	 C 	   223.83	   770.82	 20
  7	 V 	   256.85	   717.43	 19
  8	 A 	   280.53	   684.41	 18
  9	 T 	   314.21	   660.73	 17
 10	 D 	   352.55	   627.05	 16
 11	 P 	   384.90	   588.71	 15
 12	 D 	   423.25	   556.36	 14
 13	 S 	   452.26	   518.01	 13
 14	 I 	   489.95	   489.00	 12
 15	 G 	   508.96	   451.31	 11
 16	 A 	   532.64	   432.30	 10
 17	 A 	   556.32	   408.62	  9
 18	 H 	   602.00	   384.94	  8
 19	 L 	   639.70	   339.26	  7
 20	 I 	   677.39	   301.56	  6
 21	 F 	   726.42	   263.87	  5
 22	 C 	   779.80	   214.84	  4
 23	 C 	   833.19	   161.46	  3
 24	 F 	   882.22	   108.07	  2
 25	 R 	        -  	    59.04	  1  _
```

**73. IPI00219788 Isoform A of Protein FAM3B**

| Sequence | Charge | M+H+ | XCorr | DeltaCn |
| --- | --- | --- | --- | --- |
| K.ICFEDNLLMGEQLGNVAR.G | 2 | 2080.345 | 3.6 | 0.456 |

+1 charge

```
		      B   	       Y
  1	 I 	   114.09	        -  	 18
  2	 C 	   274.26	  1966.05	 17
  3	 F 	   421.32	  1805.89	 16
  4	 E 	   550.37	  1658.82	 15
  5	 D 	   665.39	  1529.77	 14
  6	 N 	   779.44	  1414.75	 13
  7	 L 	   892.52	  1300.70	 12
  8	 L 	  1005.61	  1187.62	 11
  9	 M 	  1136.65	  1074.54	 10
 10	 G 	  1193.67	   943.50	  9
 11	 E 	  1322.71	   886.47	  8
 12	 Q 	  1450.77	   757.43	  7
 13	 L 	  1563.85	   629.37	  6
 14	 G 	  1620.87	   516.29	  5
 15	 N 	  1734.92	   459.27	  4
 16	 V 	  1833.99	   345.22	  3
 17	 A 	  1905.02	   246.16	  2
 18	 R 	        -  	   175.12	  1  _
+2 charge
		      B   	       Y
  1	 I 	    57.55	        -  	 18
  2	 C 	   137.63	   983.53	 17
  3	 F 	   211.17	   903.45	 16
  4	 E 	   275.69	   829.91	 15
  5	 D 	   333.20	   765.39	 14
  6	 N 	   390.22	   707.88	 13
  7	 L 	   446.76	   650.86	 12
  8	 L 	   503.31	   594.31	 11
  9	 M 	   568.83	   537.77	 10
 10	 G 	   597.34	   472.25	  9
 11	 E 	   661.86	   443.74	  8
 12	 Q 	   725.89	   379.22	  7
 13	 L 	   782.43	   315.19	  6
 14	 G 	   810.94	   258.65	  5
 15	 N 	   867.96	   230.14	  4
 16	 V 	   917.50	   173.12	  3
 17	 A 	   953.01	   123.58	  2
 18	 R 	        -  	    88.06	  1  _
```

**74. IPI00009276 Endothelial protein C receptor precursor**

| Sequence | Charge | M+H+ | XCorr | DeltaCn |
| --- | --- | --- | --- | --- |
| R.TQSGLQSYLLQFHGLVR.L | 2 | 1948.214 | 3.146 | 0.273 |

+1 charge

```
		      B   	       Y
  1	 T 	   102.05	        -  	 17
  2	 Q 	   230.11	  1846.00	 16
  3	 S 	   317.15	  1717.94	 15
  4	 G 	   374.17	  1630.91	 14
  5	 L 	   487.25	  1573.88	 13
  6	 Q 	   615.31	  1460.80	 12
  7	 S 	   702.34	  1332.74	 11
  8	 Y 	   865.41	  1245.71	 10
  9	 L 	   978.49	  1082.65	  9
 10	 L 	  1091.57	   969.56	  8
 11	 Q 	  1219.63	   856.48	  7
 12	 F 	  1366.70	   728.42	  6
 13	 H 	  1503.76	   581.35	  5
 14	 G 	  1560.78	   444.29	  4
 15	 L 	  1673.86	   387.27	  3
 16	 V 	  1772.93	   274.19	  2
 17	 R 	        -  	   175.12	  1  _
+2 charge
		      B   	       Y
  1	 T 	    51.53	        -  	 17
  2	 Q 	   115.56	   923.50	 16
  3	 S 	   159.08	   859.47	 15
  4	 G 	   187.59	   815.96	 14
  5	 L 	   244.13	   787.45	 13
  6	 Q 	   308.16	   730.90	 12
  7	 S 	   351.67	   666.87	 11
  8	 Y 	   433.21	   623.36	 10
  9	 L 	   489.75	   541.83	  9
 10	 L 	   546.29	   485.29	  8
 11	 Q 	   610.32	   428.74	  7
 12	 F 	   683.85	   364.71	  6
 13	 H 	   752.38	   291.18	  5
 14	 G 	   780.89	   222.65	  4
 15	 L 	   837.44	   194.14	  3
 16	 V 	   886.97	   137.60	  2
 17	 R 	        -  	    88.06	  1  _
```

**75. IPI00028074 Isoform PlGF-3 of Placenta growth factor**

| Sequence | Charge | M+H+ | XCorr | DeltaCn |
| --- | --- | --- | --- | --- |
| R.LVDVVSEYPSEVEHMFSPSCVSLLR.C | 3 | 2881.244 | 3.999 | 0.382 |

+1 charge

```
		      B   	       Y
  1	 L 	   114.09	        -  	 25
  2	 V 	   213.16	  2766.45	 24
  3	 D 	   328.19	  2667.38	 23
  4	 V 	   427.26	  2552.35	 22
  5	 V 	   526.32	  2453.28	 21
  6	 S 	   613.36	  2354.21	 20
  7	 E 	   742.40	  2267.18	 19
  8	 Y 	   905.46	  2138.14	 18
  9	 P 	  1002.51	  1975.08	 17
 10	 S 	  1089.55	  1878.02	 16
 11	 E 	  1218.59	  1790.99	 15
 12	 V 	  1317.66	  1661.95	 14
 13	 E 	  1446.70	  1562.88	 13
 14	 H 	  1583.76	  1433.84	 12
 15	 M 	  1714.80	  1296.78	 11
 16	 F 	  1861.87	  1165.74	 10
 17	 S 	  1948.90	  1018.67	  9
 18	 P 	  2045.95	   931.64	  8
 19	 S 	  2132.98	   834.58	  7
 20	 C 	  2293.15	   747.55	  6
 21	 V 	  2392.22	   587.39	  5
 22	 S 	  2479.25	   488.32	  4
 23	 L 	  2592.33	   401.29	  3
 24	 L 	  2705.42	   288.20	  2
 25	 R 	        -  	   175.12	  1  _


+2 charge
		      B   	       Y
  1	 L 	    57.55	        -  	 25
  2	 V 	   107.08	  1383.73	 24
  3	 D 	   164.60	  1334.19	 23
  4	 V 	   214.13	  1276.68	 22
  5	 V 	   263.67	  1227.14	 21
  6	 S 	   307.18	  1177.61	 20
  7	 E 	   371.70	  1134.09	 19
  8	 Y 	   453.23	  1069.57	 18
  9	 P 	   501.76	   988.04	 17
 10	 S 	   545.28	   939.52	 16
 11	 E 	   609.80	   896.00	 15
 12	 V 	   659.33	   831.48	 14
 13	 E 	   723.85	   781.94	 13
 14	 H 	   792.38	   717.42	 12
 15	 M 	   857.90	   648.89	 11
 16	 F 	   931.44	   583.37	 10
 17	 S 	   974.95	   509.84	  9
 18	 P 	  1023.48	   466.32	  8
 19	 S 	  1067.00	   417.80	  7
 20	 C 	  1147.08	   374.28	  6
 21	 V 	  1196.61	   294.20	  5
 22	 S 	  1240.13	   244.66	  4
 23	 L 	  1296.67	   201.15	  3
 24	 L 	  1353.21	   144.61	  2
 25	 R 	        -  	    88.06	  1  _


+3 charge
		      B   	       Y
  1	 L 	    38.70	        -  	 25
  2	 V 	    71.72	   922.82	 24
  3	 D 	   110.07	   889.80	 23
  4	 V 	   143.09	   851.46	 22
  5	 V 	   176.11	   818.43	 21
  6	 S 	   205.12	   785.41	 20
  7	 E 	   248.14	   756.40	 19
  8	 Y 	   302.49	   713.38	 18
  9	 P 	   334.84	   659.03	 17
 10	 S 	   363.85	   626.68	 16
 11	 E 	   406.87	   597.67	 15
 12	 V 	   439.89	   554.65	 14
 13	 E 	   482.90	   521.63	 13
 14	 H 	   528.59	   478.62	 12
 15	 M 	   572.27	   432.93	 11
 16	 F 	   621.29	   389.25	 10
 17	 S 	   650.30	   340.23	  9
 18	 P 	   682.66	   311.22	  8
 19	 S 	   711.67	   278.87	  7
 20	 C 	   765.05	   249.86	  6
 21	 V 	   798.08	   196.47	  5
 22	 S 	   827.09	   163.44	  4
 23	 L 	   864.78	   134.43	  3
 24	 L 	   902.48	    96.74	  2
 25	 R 	        -  	    59.04	  1  _
```

**76. IPI00008857 Protein amnionless**

| Sequence | Charge | M+H+ | XCorr | DeltaCn |
| --- | --- | --- | --- | --- |
| R.ALLADVAENGEALGVLEATMR.E | 2 | 2144.435 | 5.107 | 0.665 |

+1 charge

```
		      B   	       Y
  1	 A 	    72.04	        -  	 21
  2	 L 	   185.13	  2072.07	 20
  3	 L 	   298.21	  1958.99	 19
  4	 A 	   369.25	  1845.90	 18
  5	 D 	   484.28	  1774.86	 17
  6	 V 	   583.34	  1659.84	 16
  7	 A 	   654.38	  1560.77	 15
  8	 E 	   783.42	  1489.73	 14
  9	 N 	   897.47	  1360.69	 13
 10	 G 	   954.49	  1246.65	 12
 11	 E 	  1083.53	  1189.62	 11
 12	 A 	  1154.57	  1060.58	 10
 13	 L 	  1267.65	   989.54	  9
 14	 G 	  1324.67	   876.46	  8
 15	 V 	  1423.74	   819.44	  7
 16	 L 	  1536.83	   720.37	  6
 17	 E 	  1665.87	   607.29	  5
 18	 A 	  1736.91	   478.24	  4
 19	 T 	  1837.95	   407.21	  3
 20	 M 	  1968.99	   306.16	  2
 21	 R 	        -  	   175.12	  1  _
+2 charge
		      B   	       Y
  1	 A 	    36.53	        -  	 21
  2	 L 	    93.07	  1036.54	 20
  3	 L 	   149.61	   980.00	 19
  4	 A 	   185.13	   923.45	 18
  5	 D 	   242.64	   887.94	 17
  6	 V 	   292.18	   830.42	 16
  7	 A 	   327.69	   780.89	 15
  8	 E 	   392.22	   745.37	 14
  9	 N 	   449.24	   680.85	 13
 10	 G 	   477.75	   623.83	 12
 11	 E 	   542.27	   595.32	 11
 12	 A 	   577.79	   530.79	 10
 13	 L 	   634.33	   495.28	  9
 14	 G 	   662.84	   438.73	  8
 15	 V 	   712.38	   410.22	  7
 16	 L 	   768.92	   360.69	  6
 17	 E 	   833.44	   304.15	  5
 18	 A 	   868.96	   239.63	  4
 19	 T 	   919.48	   204.11	  3
 20	 M 	   985.00	   153.58	  2
 21	 R 	        -  	    88.06	  1  _
```

**77. IPI00009865 Keratin, type I cytoskeletal 10**

| Sequence | Charge | M+H+ | XCorr | DeltaCn |
| --- | --- | --- | --- | --- |
| K.TIDDLKNQILNLTTDNANILLQIDNAR.L | 3 | 3054.402 | 3.542 | 0.337 |

+1 charge

```
		      B   	       Y
  1	 T 	   102.05	        -  	 27
  2	 I 	   215.14	  2951.58	 26
  3	 D 	   330.17	  2838.50	 25
  4	 D 	   445.19	  2723.47	 24
  5	 L 	   558.28	  2608.44	 23
  6	 K 	   686.37	  2495.36	 22
  7	 N 	   800.41	  2367.26	 21
  8	 Q 	   928.47	  2253.22	 20
  9	 I 	  1041.56	  2125.16	 19
 10	 L 	  1154.64	  2012.08	 18
 11	 N 	  1268.68	  1898.99	 17
 12	 L 	  1381.77	  1784.95	 16
 13	 T 	  1482.82	  1671.87	 15
 14	 T 	  1583.86	  1570.82	 14
 15	 D 	  1698.89	  1469.77	 13
 16	 N 	  1812.93	  1354.74	 12
 17	 A 	  1883.97	  1240.70	 11
 18	 N 	  1998.01	  1169.66	 10
 19	 I 	  2111.10	  1055.62	  9
 20	 L 	  2224.18	   942.54	  8
 21	 L 	  2337.27	   829.45	  7
 22	 Q 	  2465.32	   716.37	  6
 23	 I 	  2578.41	   588.31	  5
 24	 D 	  2693.44	   475.23	  4
 25	 N 	  2807.48	   360.20	  3
 26	 A 	  2878.52	   246.16	  2
 27	 R 	        -  	   175.12	  1  _


+2 charge
		      B   	       Y
  1	 T 	    51.53	        -  	 27
  2	 I 	   108.07	  1476.29	 26
  3	 D 	   165.59	  1419.75	 25
  4	 D 	   223.10	  1362.24	 24
  5	 L 	   279.64	  1304.72	 23
  6	 K 	   343.69	  1248.18	 22
  7	 N 	   400.71	  1184.13	 21
  8	 Q 	   464.74	  1127.11	 20
  9	 I 	   521.28	  1063.08	 19
 10	 L 	   577.82	  1006.54	 18
 11	 N 	   634.85	   950.00	 17
 12	 L 	   691.39	   892.98	 16
 13	 T 	   741.91	   836.44	 15
 14	 T 	   792.44	   785.91	 14
 15	 D 	   849.95	   735.39	 13
 16	 N 	   906.97	   677.88	 12
 17	 A 	   942.49	   620.85	 11
 18	 N 	   999.51	   585.34	 10
 19	 I 	  1056.05	   528.31	  9
 20	 L 	  1112.59	   471.77	  8
 21	 L 	  1169.14	   415.23	  7
 22	 Q 	  1233.17	   358.69	  6
 23	 I 	  1289.71	   294.66	  5
 24	 D 	  1347.22	   238.12	  4
 25	 N 	  1404.24	   180.60	  3
 26	 A 	  1439.76	   123.58	  2
 27	 R 	        -  	    88.06	  1  _


+3 charge
		      B   	       Y
  1	 T 	    34.69	        -  	 27
  2	 I 	    72.38	   984.53	 26
  3	 D 	   110.73	   946.84	 25
  4	 D 	   149.07	   908.49	 24
  5	 L 	   186.76	   870.15	 23
  6	 K 	   229.46	   832.46	 22
  7	 N 	   267.48	   789.76	 21
  8	 Q 	   310.16	   751.74	 20
  9	 I 	   347.86	   709.06	 19
 10	 L 	   385.55	   671.36	 18
 11	 N 	   423.57	   633.67	 17
 12	 L 	   461.26	   595.65	 16
 13	 T 	   494.94	   557.96	 15
 14	 T 	   528.63	   524.28	 14
 15	 D 	   566.97	   490.60	 13
 16	 N 	   604.98	   452.25	 12
 17	 A 	   628.66	   414.24	 11
 18	 N 	   666.68	   390.56	 10
 19	 I 	   704.37	   352.55	  9
 20	 L 	   742.07	   314.85	  8
 21	 L 	   779.76	   277.16	  7
 22	 Q 	   822.45	   239.46	  6
 23	 I 	   860.14	   196.77	  5
 24	 D 	   898.48	   159.08	  4
 25	 N 	   936.50	   120.74	  3
 26	 A 	   960.18	    82.72	  2
 27	 R 	        -  	    59.04	  1  _
```

**78. IPI00005563 Isoform 1 of Tubulointerstitial nephritis antigen-like**

| Sequence | Charge | M+H+ | XCorr | DeltaCn |
| --- | --- | --- | --- | --- |
| R.DAGGRYCQEQDLCCR.G | 3 | 1888.954 | 4.131 | 0.369 |

+1 charge

```
		      B   	       Y
  1	 D 	   116.03	        -  	 15
  2	 A 	   187.07	  1773.13	 14
  3	 G 	   244.09	  1702.09	 13
  4	 G 	   301.11	  1645.07	 12
  5	 R 	   457.22	  1588.05	 11
  6	 Y 	   620.28	  1431.95	 10
  7	 C 	   780.44	  1268.89	  9
  8	 Q 	   908.50	  1108.72	  8
  9	 E 	  1037.55	   980.66	  7
 10	 Q 	  1165.60	   851.62	  6
 11	 D 	  1280.63	   723.56	  5
 12	 L 	  1393.71	   608.53	  4
 13	 C 	  1553.88	   495.45	  3
 14	 C 	  1714.05	   335.28	  2
 15	 R 	        -  	   175.12	  1  _
+2 charge
		      B   	       Y
  1	 D 	    58.52	        -  	 15
  2	 A 	    94.04	   887.07	 14
  3	 G 	   122.55	   851.55	 13
  4	 G 	   151.06	   823.04	 12
  5	 R 	   229.11	   794.53	 11
  6	 Y 	   310.64	   716.48	 10
  7	 C 	   390.73	   634.95	  9
  8	 Q 	   454.75	   554.86	  8
  9	 E 	   519.28	   490.83	  7
 10	 Q 	   583.31	   426.31	  6
 11	 D 	   640.82	   362.28	  5
 12	 L 	   697.36	   304.77	  4
 13	 C 	   777.44	   248.23	  3
 14	 C 	   857.53	   168.15	  2
 15	 R 	        -  	    88.06	  1  _


+3 charge
		      B   	       Y
  1	 D 	    39.35	        -  	 15
  2	 A 	    63.03	   591.71	 14
  3	 G 	    82.04	   568.04	 13
  4	 G 	   101.04	   549.03	 12
  5	 R 	   153.08	   530.02	 11
  6	 Y 	   207.43	   477.99	 10
  7	 C 	   260.82	   423.63	  9
  8	 Q 	   303.51	   370.24	  8
  9	 E 	   346.52	   327.56	  7
 10	 Q 	   389.21	   284.54	  6
 11	 D 	   427.55	   241.86	  5
 12	 L 	   465.24	   203.52	  4
 13	 C 	   518.63	   165.82	  3
 14	 C 	   572.02	   112.43	  2
 15	 R 	        -  	    59.04	  1  _
```

**79. IPI00896380 Isoform 2 of Ig mu chain C region**

| Sequence | Charge | M+H+ | XCorr | DeltaCn |
| --- | --- | --- | --- | --- |
| K.LICQATGFSPR.Q | 2 | 1250.423 | 3.232 | 0.374 |

+1 charge

```
		      B   	       Y
  1	 L 	   114.09	        -  	 11
  2	 I 	   227.18	  1136.69	 10
  3	 C 	   387.34	  1023.60	  9
  4	 Q 	   515.40	   863.44	  8
  5	 A 	   586.44	   735.38	  7
  6	 T 	   687.48	   664.34	  6
  7	 G 	   744.51	   563.29	  5
  8	 F 	   891.57	   506.27	  4
  9	 S 	   978.61	   359.20	  3
 10	 P 	  1075.66	   272.17	  2
 11	 R 	        -  	   175.12	  1  _
+2 charge
		      B   	       Y
  1	 L 	    57.55	        -  	 11
  2	 I 	   114.09	   568.85	 10
  3	 C 	   194.17	   512.30	  9
  4	 Q 	   258.20	   432.22	  8
  5	 A 	   293.72	   368.19	  7
  6	 T 	   344.25	   332.67	  6
  7	 G 	   372.76	   282.15	  5
  8	 F 	   446.29	   253.64	  4
  9	 S 	   489.81	   180.11	  3
 10	 P 	   538.33	   136.59	  2
 11	 R 	        -  	    88.06	  1  _
```

**80. IPI00160130 Cubilin**

| Sequence | Charge | M+H+ | XCorr | DeltaCn |
| --- | --- | --- | --- | --- |
| R.FVTDGSVTASGFR.L | 2 | 1344.455 | 3.524 | 0.563 |

+1 charge

```
		      B   	       Y
  1	 F 	   148.08	        -  	 13
  2	 V 	   247.14	  1196.59	 12
  3	 T 	   348.19	  1097.52	 11
  4	 D 	   463.22	   996.47	 10
  5	 G 	   520.24	   881.45	  9
  6	 S 	   607.27	   824.43	  8
  7	 V 	   706.34	   737.39	  7
  8	 T 	   807.39	   638.33	  6
  9	 A 	   878.43	   537.28	  5
 10	 S 	   965.46	   466.24	  4
 11	 G 	  1022.48	   379.21	  3
 12	 F 	  1169.55	   322.19	  2
 13	 R 	        -  	   175.12	  1  _
+2 charge
		      B   	       Y
  1	 F 	    74.54	        -  	 13
  2	 V 	   124.08	   598.80	 12
  3	 T 	   174.60	   549.26	 11
  4	 D 	   232.11	   498.74	 10
  5	 G 	   260.62	   441.23	  9
  6	 S 	   304.14	   412.72	  8
  7	 V 	   353.67	   369.20	  7
  8	 T 	   404.20	   319.67	  6
  9	 A 	   439.72	   269.14	  5
 10	 S 	   483.23	   233.62	  4
 11	 G 	   511.74	   190.11	  3
 12	 F 	   585.28	   161.60	  2
 13	 R 	        -  	    88.06	  1  _
```

**81. IPI00303832 Paf1/RNA polymerase II complex component**

| Sequence | Charge | M+H+ | XCorr | DeltaCn |
| --- | --- | --- | --- | --- |
| K.DKDLNSKSASDLSEDLFK.V | 2 | 2013.149 | 3.015 | 0.25 |

+1 charge

```
		      B   	       Y
  1	 D 	   116.03	        -  	 18
  2	 K 	   244.13	  1896.95	 17
  3	 D 	   359.16	  1768.86	 16
  4	 L 	   472.24	  1653.83	 15
  5	 N 	   586.28	  1540.75	 14
  6	 S 	   673.32	  1426.71	 13
  7	 K 	   801.41	  1339.67	 12
  8	 S 	   888.44	  1211.58	 11
  9	 A 	   959.48	  1124.55	 10
 10	 S 	  1046.51	  1053.51	  9
 11	 D 	  1161.54	   966.48	  8
 12	 L 	  1274.62	   851.45	  7
 13	 S 	  1361.65	   738.37	  6
 14	 E 	  1490.70	   651.33	  5
 15	 D 	  1605.72	   522.29	  4
 16	 L 	  1718.81	   407.27	  3
 17	 F 	  1865.88	   294.18	  2
 18	 K 	        -  	   147.11	  1  _
+2 charge
		      B   	       Y
  1	 D 	    58.52	        -  	 18
  2	 K 	   122.57	   948.98	 17
  3	 D 	   180.08	   884.93	 16
  4	 L 	   236.62	   827.42	 15
  5	 N 	   293.65	   770.88	 14
  6	 S 	   337.16	   713.86	 13
  7	 K 	   401.21	   670.34	 12
  8	 S 	   444.72	   606.29	 11
  9	 A 	   480.24	   562.78	 10
 10	 S 	   523.76	   527.26	  9
 11	 D 	   581.27	   483.74	  8
 12	 L 	   637.81	   426.23	  7
 13	 S 	   681.33	   369.69	  6
 14	 E 	   745.85	   326.17	  5
 15	 D 	   803.37	   261.65	  4
 16	 L 	   859.91	   204.14	  3
 17	 F 	   933.44	   147.59	  2
 18	 K 	        -  	    74.06	  1  _
```

**82. IPI00102300 Isoform 3 of Platelet glycoprotein VI**

| Sequence | Charge | M+H+ | XCorr | DeltaCn |
| --- | --- | --- | --- | --- |
| R.ASFPIITVTAAHSGTYR.C | 2 | 1793.016 | 4.178 | 0.592 |

+1 charge

```
		      B   	       Y
  1	 A 	    72.04	        -  	 17
  2	 S 	   159.08	  1720.90	 16
  3	 F 	   306.14	  1633.87	 15
  4	 P 	   403.20	  1486.80	 14
  5	 I 	   516.28	  1389.75	 13
  6	 I 	   629.37	  1276.66	 12
  7	 T 	   730.41	  1163.58	 11
  8	 V 	   829.48	  1062.53	 10
  9	 T 	   930.53	   963.46	  9
 10	 A 	  1001.57	   862.42	  8
 11	 A 	  1072.60	   791.38	  7
 12	 H 	  1209.66	   720.34	  6
 13	 S 	  1296.69	   583.28	  5
 14	 G 	  1353.72	   496.25	  4
 15	 T 	  1454.76	   439.23	  3
 16	 Y 	  1617.83	   338.18	  2
 17	 R 	        -  	   175.12	  1  _
+2 charge
		      B   	       Y
  1	 A 	    36.53	        -  	 17
  2	 S 	    80.04	   860.95	 16
  3	 F 	   153.58	   817.44	 15
  4	 P 	   202.10	   743.90	 14
  5	 I 	   258.64	   695.38	 13
  6	 I 	   315.19	   638.84	 12
  7	 T 	   365.71	   582.29	 11
  8	 V 	   415.24	   531.77	 10
  9	 T 	   465.77	   482.24	  9
 10	 A 	   501.29	   431.71	  8
 11	 A 	   536.81	   396.19	  7
 12	 H 	   605.33	   360.67	  6
 13	 S 	   648.85	   292.15	  5
 14	 G 	   677.36	   248.63	  4
 15	 T 	   727.89	   220.12	  3
 16	 Y 	   809.42	   169.59	  2
 17	 R 	        -  	    88.06	  1  _
```

**83. IPI00299738 Procollagen C-endopeptidase enhancer 1**

| Sequence | Charge | M+H+ | XCorr | DeltaCn |
| --- | --- | --- | --- | --- |
| R.FCGTFRPAPLVAPGNQVTLR.M | 3 | 2202.538 | 4.11 | 0.439 |

+1 charge

```
		      B   	       Y
  1	 F 	   148.08	        -  	 20
  2	 C 	   308.24	  2054.23	 19
  3	 G 	   365.26	  1894.07	 18
  4	 T 	   466.31	  1837.04	 17
  5	 F 	   613.38	  1736.00	 16
  6	 R 	   769.48	  1588.93	 15
  7	 P 	   866.53	  1432.83	 14
  8	 A 	   937.57	  1335.77	 13
  9	 P 	  1034.62	  1264.74	 12
 10	 L 	  1147.71	  1167.68	 11
 11	 V 	  1246.77	  1054.60	 10
 12	 A 	  1317.81	   955.53	  9
 13	 P 	  1414.86	   884.49	  8
 14	 G 	  1471.89	   787.44	  7
 15	 N 	  1585.93	   730.42	  6
 16	 Q 	  1713.99	   616.38	  5
 17	 V 	  1813.06	   488.32	  4
 18	 T 	  1914.10	   389.25	  3
 19	 L 	  2027.19	   288.20	  2
 20	 R 	        -  	   175.12	  1  _
+2 charge
		      B   	       Y
  1	 F 	    74.54	        -  	 20
  2	 C 	   154.62	  1027.62	 19
  3	 G 	   183.13	   947.54	 18
  4	 T 	   233.66	   919.03	 17
  5	 F 	   307.19	   868.50	 16
  6	 R 	   385.24	   794.97	 15
  7	 P 	   433.77	   716.92	 14
  8	 A 	   469.29	   668.39	 13
  9	 P 	   517.81	   632.87	 12
 10	 L 	   574.36	   584.35	 11
 11	 V 	   623.89	   527.80	 10
 12	 A 	   659.41	   478.27	  9
 13	 P 	   707.94	   442.75	  8
 14	 G 	   736.45	   394.22	  7
 15	 N 	   793.47	   365.71	  6
 16	 Q 	   857.50	   308.69	  5
 17	 V 	   907.03	   244.66	  4
 18	 T 	   957.56	   195.13	  3
 19	 L 	  1014.10	   144.61	  2
 20	 R 	        -  	    88.06	  1  _

+3 charge
		      B   	       Y
  1	 F 	    50.03	        -  	 20
  2	 C 	   103.42	   685.42	 19
  3	 G 	   122.43	   632.03	 18
  4	 T 	   156.11	   613.02	 17
  5	 F 	   205.13	   579.34	 16
  6	 R 	   257.16	   530.31	 15
  7	 P 	   289.52	   478.28	 14
  8	 A 	   313.19	   445.93	 13
  9	 P 	   345.55	   422.25	 12
 10	 L 	   383.24	   389.90	 11
 11	 V 	   416.26	   352.20	 10
 12	 A 	   439.94	   319.18	  9
 13	 P 	   472.29	   295.50	  8
 14	 G 	   491.30	   263.15	  7
 15	 N 	   529.31	   244.15	  6
 16	 Q 	   572.00	   206.13	  5
 17	 V 	   605.02	   163.44	  4
 18	 T 	   638.71	   130.42	  3
 19	 L 	   676.40	    96.74	  2
 20	 R 	        -  	    59.04	  1  _
```

**84. IPI00000760 N(G),N(G)-dimethylarginine dimethylaminohydrolase 2**

| Sequence | Charge | M+H+ | XCorr | DeltaCn |
| --- | --- | --- | --- | --- |
| R.GVPESLASGEGAGAGLPALDLAK.A | 2 | 2081.313 | 3.15 | 0.359 |

+1 charge

```
		      B   	       Y
  1	 G 	    58.03	        -  	 23
  2	 V 	   157.10	  2023.07	 22
  3	 P 	   254.15	  1924.00	 21
  4	 E 	   383.19	  1826.95	 20
  5	 S 	   470.22	  1697.91	 19
  6	 L 	   583.31	  1610.87	 18
  7	 A 	   654.35	  1497.79	 17
  8	 S 	   741.38	  1426.75	 16
  9	 G 	   798.40	  1339.72	 15
 10	 E 	   927.44	  1282.70	 14
 11	 G 	   984.46	  1153.66	 13
 12	 A 	  1055.50	  1096.64	 12
 13	 G 	  1112.52	  1025.60	 11
 14	 A 	  1183.56	   968.58	 10
 15	 G 	  1240.58	   897.54	  9
 16	 L 	  1353.66	   840.52	  8
 17	 P 	  1450.72	   727.43	  7
 18	 A 	  1521.75	   630.38	  6
 19	 L 	  1634.84	   559.34	  5
 20	 D 	  1749.87	   446.26	  4
 21	 L 	  1862.95	   331.23	  3
 22	 A 	  1933.99	   218.15	  2
 23	 K 	        -  	   147.11	  1  _


+2 charge
		      B   	       Y
  1	 G 	    29.52	        -  	 23
  2	 V 	    79.05	  1012.04	 22
  3	 P 	   127.58	   962.50	 21
  4	 E 	   192.10	   913.98	 20
  5	 S 	   235.62	   849.46	 19
  6	 L 	   292.16	   805.94	 18
  7	 A 	   327.68	   749.40	 17
  8	 S 	   371.19	   713.88	 16
  9	 G 	   399.70	   670.36	 15
 10	 E 	   464.22	   641.85	 14
 11	 G 	   492.74	   577.33	 13
 12	 A 	   528.25	   548.82	 12
 13	 G 	   556.76	   513.30	 11
 14	 A 	   592.28	   484.79	 10
 15	 G 	   620.79	   449.27	  9
 16	 L 	   677.34	   420.76	  8
 17	 P 	   725.86	   364.22	  7
 18	 A 	   761.38	   315.69	  6
 19	 L 	   817.92	   280.18	  5
 20	 D 	   875.44	   223.63	  4
 21	 L 	   931.98	   166.12	  3
 22	 A 	   967.50	   109.58	  2
 23	 K 	        -  	    74.06	  1  _
```

**85. IPI00736885 Ig kappa chain V-II region TEW**

| Sequence | Charge | M+H+ | XCorr | DeltaCn |
| --- | --- | --- | --- | --- |
| - .DIVMTQSPLSLPVTPGEPASISCR.S | 3 | 2556.911 | 4.868 | 0.329 |

+1 charge

```
		      B   	       Y
  1	 D 	   116.03	        -  	 24
  2	 I 	   229.12	  2440.39	 23
  3	 V 	   328.19	  2327.31	 22
  4	 M 	   459.23	  2228.24	 21
  5	 T 	   560.27	  2097.20	 20
  6	 Q 	   688.33	  1996.15	 19
  7	 S 	   775.37	  1868.09	 18
  8	 P 	   872.42	  1781.06	 17
  9	 L 	   985.50	  1684.01	 16
 10	 S 	  1072.53	  1570.92	 15
 11	 L 	  1185.62	  1483.89	 14
 12	 P 	  1282.67	  1370.81	 13
 13	 V 	  1381.74	  1273.76	 12
 14	 T 	  1482.79	  1174.69	 11
 15	 P 	  1579.84	  1073.64	 10
 16	 G 	  1636.86	   976.59	  9
 17	 E 	  1765.90	   919.56	  8
 18	 P 	  1862.96	   790.52	  7
 19	 A 	  1933.99	   693.47	  6
 20	 S 	  2021.03	   622.43	  5
 21	 I 	  2134.11	   535.40	  4
 22	 S 	  2221.14	   422.32	  3
 23	 C 	  2381.31	   335.28	  2
 24	 R 	        -  	   175.12	  1  _


+2 charge
		      B   	       Y
  1	 D 	    58.52	        -  	 24
  2	 I 	   115.06	  1220.70	 23
  3	 V 	   164.60	  1164.16	 22
  4	 M 	   230.12	  1114.62	 21
  5	 T 	   280.64	  1049.10	 20
  6	 Q 	   344.67	   998.58	 19
  7	 S 	   388.19	   934.55	 18
  8	 P 	   436.71	   891.03	 17
  9	 L 	   493.25	   842.51	 16
 10	 S 	   536.77	   785.97	 15
 11	 L 	   593.31	   742.45	 14
 12	 P 	   641.84	   685.91	 13
 13	 V 	   691.37	   637.38	 12
 14	 T 	   741.90	   587.85	 11
 15	 P 	   790.42	   537.32	 10
 16	 G 	   818.93	   488.80	  9
 17	 E 	   883.46	   460.29	  8
 18	 P 	   931.98	   395.76	  7
 19	 A 	   967.50	   347.24	  6
 20	 S 	  1011.02	   311.72	  5
 21	 I 	  1067.56	   268.20	  4
 22	 S 	  1111.07	   211.66	  3
 23	 C 	  1191.16	   168.15	  2
 24	 R 	        -  	    88.06	  1  _


+3 charge
		      B   	       Y
  1	 D 	    39.35	        -  	 24
  2	 I 	    77.04	   814.14	 23
  3	 V 	   110.07	   776.44	 22
  4	 M 	   153.75	   743.42	 21
  5	 T 	   187.43	   699.74	 20
  6	 Q 	   230.12	   666.06	 19
  7	 S 	   259.13	   623.37	 18
  8	 P 	   291.48	   594.36	 17
  9	 L 	   329.17	   562.01	 16
 10	 S 	   358.18	   524.31	 15
 11	 L 	   395.88	   495.30	 14
 12	 P 	   428.23	   457.61	 13
 13	 V 	   461.25	   425.26	 12
 14	 T 	   494.93	   392.23	 11
 15	 P 	   527.28	   358.55	 10
 16	 G 	   546.29	   326.20	  9
 17	 E 	   589.31	   307.19	  8
 18	 P 	   621.66	   264.18	  7
 19	 A 	   645.34	   231.83	  6
 20	 S 	   674.35	   208.15	  5
 21	 I 	   712.04	   179.14	  4
 22	 S 	   741.05	   141.44	  3
 23	 C 	   794.44	   112.43	  2
 24	 R 	        -  	    59.04	  1  _
```

**86. IPI00022331 Phosphatidylcholine-sterol acyltransferase**

| Sequence | Charge | M+H+ | XCorr | DeltaCn |
| --- | --- | --- | --- | --- |
| R.TYIYDHGFPYTDPVGVLYEDGDDTVATR.S | 3 | 3181.367 | 3.434 | 0.242 |

+1 charge

```
		      B   	       Y
  1	 T 	   102.05	        -  	 28
  2	 Y 	   265.12	  3078.40	 27
  3	 I 	   378.20	  2915.34	 26
  4	 Y 	   541.27	  2802.25	 25
  5	 D 	   656.29	  2639.19	 24
  6	 H 	   793.35	  2524.16	 23
  7	 G 	   850.37	  2387.10	 22
  8	 F 	   997.44	  2330.08	 21
  9	 P 	  1094.49	  2183.01	 20
 10	 Y 	  1257.56	  2085.96	 19
 11	 T 	  1358.61	  1922.90	 18
 12	 D 	  1473.63	  1821.85	 17
 13	 P 	  1570.68	  1706.82	 16
 14	 V 	  1669.75	  1609.77	 15
 15	 G 	  1726.77	  1510.70	 14
 16	 V 	  1825.84	  1453.68	 13
 17	 L 	  1938.93	  1354.61	 12
 18	 Y 	  2101.99	  1241.53	 11
 19	 E 	  2231.03	  1078.46	 10
 20	 D 	  2346.06	   949.42	  9
 21	 G 	  2403.08	   834.40	  8
 22	 D 	  2518.11	   777.37	  7
 23	 D 	  2633.14	   662.35	  6
 24	 T 	  2734.18	   547.32	  5
 25	 V 	  2833.25	   446.27	  4
 26	 A 	  2904.29	   347.20	  3
 27	 T 	  3005.34	   276.17	  2
 28	 R 	        -  	   175.12	  1  _


+2 charge
		      B   	       Y
  1	 T 	    51.53	        -  	 28
  2	 Y 	   133.06	  1539.70	 27
  3	 I 	   189.60	  1458.17	 26
  4	 Y 	   271.14	  1401.63	 25
  5	 D 	   328.65	  1320.10	 24
  6	 H 	   397.18	  1262.58	 23
  7	 G 	   425.69	  1194.06	 22
  8	 F 	   499.22	  1165.54	 21
  9	 P 	   547.75	  1092.01	 20
 10	 Y 	   629.28	  1043.48	 19
 11	 T 	   679.81	   961.95	 18
 12	 D 	   737.32	   911.43	 17
 13	 P 	   785.85	   853.92	 16
 14	 V 	   835.38	   805.39	 15
 15	 G 	   863.89	   755.85	 14
 16	 V 	   913.43	   727.34	 13
 17	 L 	   969.97	   677.81	 12
 18	 Y 	  1051.50	   621.27	 11
 19	 E 	  1116.02	   539.74	 10
 20	 D 	  1173.53	   475.21	  9
 21	 G 	  1202.04	   417.70	  8
 22	 D 	  1259.56	   389.19	  7
 23	 D 	  1317.07	   331.68	  6
 24	 T 	  1367.60	   274.16	  5
 25	 V 	  1417.13	   223.64	  4
 26	 A 	  1452.65	   174.11	  3
 27	 T 	  1503.17	   138.59	  2
 28	 R 	        -  	    88.06	  1  _


+3 charge
		      B   	       Y
  1	 T 	    34.69	        -  	 28
  2	 Y 	    89.04	  1026.80	 27
  3	 I 	   126.74	   972.45	 26
  4	 Y 	   181.09	   934.76	 25
  5	 D 	   219.44	   880.40	 24
  6	 H 	   265.12	   842.06	 23
  7	 G 	   284.13	   796.37	 22
  8	 F 	   333.15	   777.37	 21
  9	 P 	   365.50	   728.34	 20
 10	 Y 	   419.86	   695.99	 19
 11	 T 	   453.54	   641.64	 18
 12	 D 	   491.88	   607.95	 17
 13	 P 	   524.23	   569.61	 16
 14	 V 	   557.26	   537.26	 15
 15	 G 	   576.26	   504.24	 14
 16	 V 	   609.29	   485.23	 13
 17	 L 	   646.98	   452.21	 12
 18	 Y 	   701.34	   414.51	 11
 19	 E 	   744.35	   360.16	 10
 20	 D 	   782.69	   317.15	  9
 21	 G 	   801.70	   278.80	  8
 22	 D 	   840.04	   259.80	  7
 23	 D 	   878.38	   221.45	  6
 24	 T 	   912.07	   183.11	  5
 25	 V 	   945.09	   149.43	  4
 26	 A 	   968.77	   116.41	  3
 27	 T 	  1002.45	    92.73	  2
 28	 R 	        -  	    59.04	  1  _
```

**87. IPI00240345 C-type lectin domain family 14 member A**

| Sequence | Charge | M+H+ | XCorr | DeltaCn |
| --- | --- | --- | --- | --- |
| R.GQLPISVTCIADEIGAR.W | 2 | 1801.028 | 3.36 | 0.408 |

+1 charge

```
		      B   	       Y
  1	 G 	    58.03	        -  	 17
  2	 Q 	   186.09	  1743.05	 16
  3	 L 	   299.17	  1614.99	 15
  4	 P 	   396.22	  1501.90	 14
  5	 I 	   509.31	  1404.85	 13
  6	 S 	   596.34	  1291.77	 12
  7	 V 	   695.41	  1204.73	 11
  8	 T 	   796.46	  1105.67	 10
  9	 C 	   956.62	  1004.62	  9
 10	 I 	  1069.71	   844.45	  8
 11	 A 	  1140.74	   731.37	  7
 12	 D 	  1255.77	   660.33	  6
 13	 E 	  1384.81	   545.30	  5
 14	 I 	  1497.90	   416.26	  4
 15	 G 	  1554.92	   303.18	  3
 16	 A 	  1625.95	   246.16	  2
 17	 R 	        -  	   175.12	  1  _
+2 charge
		      B   	       Y
  1	 G 	    29.52	        -  	 17
  2	 Q 	    93.55	   872.03	 16
  3	 L 	   150.09	   808.00	 15
  4	 P 	   198.62	   751.45	 14
  5	 I 	   255.16	   702.93	 13
  6	 S 	   298.67	   646.39	 12
  7	 V 	   348.21	   602.87	 11
  8	 T 	   398.73	   553.34	 10
  9	 C 	   478.81	   502.81	  9
 10	 I 	   535.36	   422.73	  8
 11	 A 	   570.88	   366.19	  7
 12	 D 	   628.39	   330.67	  6
 13	 E 	   692.91	   273.16	  5
 14	 I 	   749.45	   208.63	  4
 15	 G 	   777.96	   152.09	  3
 16	 A 	   813.48	   123.58	  2
 17	 R 	        -  	    88.06	  1  _
```

**88. IPI00003590 Isoform 1 of Sulfhydryl oxidase 1**

| Sequence | Charge | M+H+ | XCorr | DeltaCn |
| --- | --- | --- | --- | --- |
| R.SALYSPSDPLTLLQADTVR.G | 2 | 2048.282 | 3.076 | 0.5 |

+1 charge

```
		      B   	       Y
  1	 S 	    88.04	        -  	 19
  2	 A 	   159.08	  1960.04	 18
  3	 L 	   272.16	  1889.00	 17
  4	 Y 	   435.22	  1775.92	 16
  5	 S 	   522.26	  1612.85	 15
  6	 P 	   619.31	  1525.82	 14
  7	 S 	   706.34	  1428.77	 13
  8	 D 	   821.37	  1341.74	 12
  9	 P 	   918.42	  1226.71	 11
 10	 L 	  1031.50	  1129.66	 10
 11	 T 	  1132.55	  1016.57	  9
 12	 L 	  1245.64	   915.53	  8
 13	 L 	  1358.72	   802.44	  7
 14	 Q 	  1486.78	   689.36	  6
 15	 A 	  1557.82	   561.30	  5
 16	 D 	  1672.84	   490.26	  4
 17	 T 	  1773.89	   375.24	  3
 18	 V 	  1872.96	   274.19	  2
 19	 R 	        -  	   175.12	  1  _
+2 charge
		      B   	       Y
  1	 S 	    44.52	        -  	 19
  2	 A 	    80.04	   980.52	 18
  3	 L 	   136.58	   945.00	 17
  4	 Y 	   218.12	   888.46	 16
  5	 S 	   261.63	   806.93	 15
  6	 P 	   310.16	   763.41	 14
  7	 S 	   353.67	   714.89	 13
  8	 D 	   411.19	   671.37	 12
  9	 P 	   459.71	   613.86	 11
 10	 L 	   516.26	   565.33	 10
 11	 T 	   566.78	   508.79	  9
 12	 L 	   623.32	   458.27	  8
 13	 L 	   679.86	   401.72	  7
 14	 Q 	   743.89	   345.18	  6
 15	 A 	   779.41	   281.15	  5
 16	 D 	   836.93	   245.63	  4
 17	 T 	   887.45	   188.12	  3
 18	 V 	   936.98	   137.60	  2
 19	 R 	        -  	    88.06	  1  _
```

**89. IPI00045512 Hemicentin 1**

| Sequence | Charge | M+H+ | XCorr | DeltaCn |
| --- | --- | --- | --- | --- |
| K.LVSLPFGIATNQDLIR.L | 2 | 1758.055 | 4.999 | 0.519 |

+1 charge

```
		      B   	       Y
  1	 L 	   114.09	        -  	 16
  2	 V 	   213.16	  1643.91	 15
  3	 S 	   300.19	  1544.84	 14
  4	 L 	   413.28	  1457.81	 13
  5	 P 	   510.33	  1344.73	 12
  6	 F 	   657.40	  1247.67	 11
  7	 G 	   714.42	  1100.61	 10
  8	 I 	   827.50	  1043.58	  9
  9	 A 	   898.54	   930.50	  8
 10	 T 	   999.59	   859.46	  7
 11	 N 	  1113.63	   758.42	  6
 12	 Q 	  1241.69	   644.37	  5
 13	 D 	  1356.72	   516.31	  4
 14	 L 	  1469.80	   401.29	  3
 15	 I 	  1582.88	   288.20	  2
 16	 R 	        -  	   175.12	  1  _


+2 charge
		      B   	       Y
  1	 L 	    57.55	        -  	 16
  2	 V 	   107.08	   822.46	 15
  3	 S 	   150.60	   772.93	 14
  4	 L 	   207.14	   729.41	 13
  5	 P 	   255.67	   672.87	 12
  6	 F 	   329.20	   624.34	 11
  7	 G 	   357.71	   550.81	 10
  8	 I 	   414.25	   522.30	  9
  9	 A 	   449.77	   465.75	  8
 10	 T 	   500.30	   430.24	  7
 11	 N 	   557.32	   379.71	  6
 12	 Q 	   621.35	   322.69	  5
 13	 D 	   678.86	   258.66	  4
 14	 L 	   735.40	   201.15	  3
 15	 I 	   791.95	   144.61	  2
 16	 R 	        -  	    88.06	  1  _
```

**90. IPI00021263 14-3-3 protein zeta/delta**

| Sequence | Charge | M+H+ | XCorr | DeltaCn |
| --- | --- | --- | --- | --- |
| K.SVTEQGAELSNEERNLLSVAYKNVVGAR.R | 3 | 3035.316 | 4.801 | 0.558 |

+1 charge

```
		      B   	       Y
  1	 S 	    88.04	        -  	 28
  2	 V 	   187.11	  2946.53	 27
  3	 T 	   288.16	  2847.46	 26
  4	 E 	   417.20	  2746.41	 25
  5	 Q 	   545.26	  2617.37	 24
  6	 G 	   602.28	  2489.31	 23
  7	 A 	   673.32	  2432.29	 22
  8	 E 	   802.36	  2361.25	 21
  9	 L 	   915.44	  2232.21	 20
 10	 S 	  1002.47	  2119.13	 19
 11	 N 	  1116.52	  2032.09	 18
 12	 E 	  1245.56	  1918.05	 17
 13	 E 	  1374.60	  1789.01	 16
 14	 R 	  1530.70	  1659.97	 15
 15	 N 	  1644.75	  1503.86	 14
 16	 L 	  1757.83	  1389.82	 13
 17	 L 	  1870.91	  1276.74	 12
 18	 S 	  1957.95	  1163.65	 11
 19	 V 	  2057.01	  1076.62	 10
 20	 A 	  2128.05	   977.55	  9
 21	 Y 	  2291.11	   906.52	  8
 22	 K 	  2419.21	   743.45	  7
 23	 N 	  2533.25	   615.36	  6
 24	 V 	  2632.32	   501.31	  5
 25	 V 	  2731.39	   402.25	  4
 26	 G 	  2788.41	   303.18	  3
 27	 A 	  2859.45	   246.16	  2
 28	 R 	        -  	   175.12	  1  _


+2 charge
		      B   	       Y
  1	 S 	    44.52	        -  	 28
  2	 V 	    94.06	  1473.77	 27
  3	 T 	   144.58	  1424.23	 26
  4	 E 	   209.10	  1373.71	 25
  5	 Q 	   273.13	  1309.19	 24
  6	 G 	   301.64	  1245.16	 23
  7	 A 	   337.16	  1216.65	 22
  8	 E 	   401.68	  1181.13	 21
  9	 L 	   458.22	  1116.61	 20
 10	 S 	   501.74	  1060.07	 19
 11	 N 	   558.76	  1016.55	 18
 12	 E 	   623.28	   959.53	 17
 13	 E 	   687.80	   895.01	 16
 14	 R 	   765.86	   830.49	 15
 15	 N 	   822.88	   752.44	 14
 16	 L 	   879.42	   695.41	 13
 17	 L 	   935.96	   638.87	 12
 18	 S 	   979.48	   582.33	 11
 19	 V 	  1029.01	   538.81	 10
 20	 A 	  1064.53	   489.28	  9
 21	 Y 	  1146.06	   453.76	  8
 22	 K 	  1210.11	   372.23	  7
 23	 N 	  1267.13	   308.18	  6
 24	 V 	  1316.66	   251.16	  5
 25	 V 	  1366.20	   201.63	  4
 26	 G 	  1394.71	   152.09	  3
 27	 A 	  1430.23	   123.58	  2
 28	 R 	        -  	    88.06	  1  _


+3 charge
		      B   	       Y
  1	 S 	    30.02	        -  	 28
  2	 V 	    63.04	   982.85	 27
  3	 T 	    96.72	   949.82	 26
  4	 E 	   139.74	   916.14	 25
  5	 Q 	   182.42	   873.13	 24
  6	 G 	   201.43	   830.44	 23
  7	 A 	   225.11	   811.43	 22
  8	 E 	   268.12	   787.76	 21
  9	 L 	   305.82	   744.74	 20
 10	 S 	   334.83	   707.05	 19
 11	 N 	   372.84	   678.04	 18
 12	 E 	   415.86	   640.02	 17
 13	 E 	   458.87	   597.01	 16
 14	 R 	   510.91	   553.99	 15
 15	 N 	   548.92	   501.96	 14
 16	 L 	   586.61	   463.95	 13
 17	 L 	   624.31	   426.25	 12
 18	 S 	   653.32	   388.56	 11
 19	 V 	   686.34	   359.55	 10
 20	 A 	   710.02	   326.52	  9
 21	 Y 	   764.38	   302.84	  8
 22	 K 	   807.07	   248.49	  7
 23	 N 	   845.09	   205.79	  6
 24	 V 	   878.11	   167.78	  5
 25	 V 	   911.13	   134.75	  4
 26	 G 	   930.14	   101.73	  3
 27	 A 	   953.82	    82.72	  2
 28	 R 	        -  	    59.04	  1  _
```

**91. IPI00219217 L-lactate dehydrogenase B chain**

| Sequence | Charge | M+H+ | XCorr | DeltaCn |
| --- | --- | --- | --- | --- |
| K.SADTLWDIQKDLKDL.- | 2 | 1761.954 | 3.636 | 0.423 |

+1 charge

```
		      B   	       Y
  1	 S 	    88.04	        -  	 15
  2	 A 	   159.08	  1673.87	 14
  3	 D 	   274.10	  1602.84	 13
  4	 T 	   375.15	  1487.81	 12
  5	 L 	   488.24	  1386.76	 11
  6	 W 	   674.31	  1273.68	 10
  7	 D 	   789.34	  1087.60	  9
  8	 I 	   902.43	   972.57	  8
  9	 Q 	  1030.48	   859.49	  7
 10	 K 	  1158.58	   731.43	  6
 11	 D 	  1273.61	   603.33	  5
 12	 L 	  1386.69	   488.31	  4
 13	 K 	  1514.78	   375.22	  3
 14	 D 	  1629.81	   247.13	  2
 15	 L 	        -  	   132.10	  1  _
+2 charge
		      B   	       Y
  1	 S 	    44.52	        -  	 15
  2	 A 	    80.04	   837.44	 14
  3	 D 	   137.56	   801.92	 13
  4	 T 	   188.08	   744.41	 12
  5	 L 	   244.62	   693.89	 11
  6	 W 	   337.66	   637.34	 10
  7	 D 	   395.17	   544.30	  9
  8	 I 	   451.72	   486.79	  8
  9	 Q 	   515.75	   430.25	  7
 10	 K 	   579.79	   366.22	  6
 11	 D 	   637.31	   302.17	  5
 12	 L 	   693.85	   244.66	  4
 13	 K 	   757.90	   188.12	  3
 14	 D 	   815.41	   124.07	  2
 15	 L 	        -  	    66.55	  1  _
```

**92. IPI00852735 Isoform 1 of Protocadherin Fat 4**

| Sequence | Charge | M+H+ | XCorr | DeltaCn |
| --- | --- | --- | --- | --- |
| R.ESLPVIIVANEPLQPFLCK.C | 2 | 2168.555 | 3.206 | 0.439 |

+1 charge

```
		      B   	       Y
  1	 E 	   130.05	        -  	 19
  2	 S 	   217.08	  2038.28	 18
  3	 L 	   330.17	  1951.24	 17
  4	 P 	   427.22	  1838.16	 16
  5	 V 	   526.29	  1741.11	 15
  6	 I 	   639.37	  1642.04	 14
  7	 I 	   752.46	  1528.95	 13
  8	 V 	   851.52	  1415.87	 12
  9	 A 	   922.56	  1316.80	 11
 10	 N 	  1036.60	  1245.76	 10
 11	 E 	  1165.65	  1131.72	  9
 12	 P 	  1262.70	  1002.68	  8
 13	 L 	  1375.78	   905.63	  7
 14	 Q 	  1503.84	   792.54	  6
 15	 P 	  1600.89	   664.48	  5
 16	 F 	  1747.96	   567.43	  4
 17	 L 	  1861.05	   420.36	  3
 18	 C 	  2021.21	   307.28	  2
 19	 K 	        -  	   147.11	  1  _
+2 charge
		      B   	       Y
  1	 E 	    65.53	        -  	 19
  2	 S 	   109.04	  1019.64	 18
  3	 L 	   165.59	   976.13	 17
  4	 P 	   214.11	   919.58	 16
  5	 V 	   263.65	   871.06	 15
  6	 I 	   320.19	   821.52	 14
  7	 I 	   376.73	   764.98	 13
  8	 V 	   426.27	   708.44	 12
  9	 A 	   461.78	   658.90	 11
 10	 N 	   518.81	   623.39	 10
 11	 E 	   583.33	   566.36	  9
 12	 P 	   631.85	   501.84	  8
 13	 L 	   688.40	   453.32	  7
 14	 Q 	   752.42	   396.77	  6
 15	 P 	   800.95	   332.75	  5
 16	 F 	   874.49	   284.22	  4
 17	 L 	   931.03	   210.68	  3
 18	 C 	  1011.11	   154.14	  2
 19	 K 	        -  	    74.06	  1  _
```

**93. IPI00012503 Isoform Sap-mu-0 of Proactivator polypeptide**

| Sequence | Charge | M+H+ | XCorr | DeltaCn |
| --- | --- | --- | --- | --- |
| K.NYISQYSEIAIQMMMHMQPK.E | 3 | 2444.903 | 3.973 | 0.488 |

+1 charge

```
		      B   	       Y
  1	 N 	   115.05	        -  	 20
  2	 Y 	   278.11	  2329.08	 19
  3	 I 	   391.20	  2166.02	 18
  4	 S 	   478.23	  2052.94	 17
  5	 Q 	   606.29	  1965.91	 16
  6	 Y 	   769.35	  1837.85	 15
  7	 S 	   856.38	  1674.78	 14
  8	 E 	   985.43	  1587.75	 13
  9	 I 	  1098.51	  1458.71	 12
 10	 A 	  1169.55	  1345.62	 11
 11	 I 	  1282.63	  1274.59	 10
 12	 Q 	  1410.69	  1161.50	  9
 13	 M 	  1541.73	  1033.44	  8
 14	 M 	  1672.77	   902.40	  7
 15	 M 	  1803.81	   771.36	  6
 16	 H 	  1940.87	   640.32	  5
 17	 M 	  2071.91	   503.26	  4
 18	 Q 	  2199.97	   372.22	  3
 19	 P 	  2297.02	   244.17	  2
 20	 K 	        -  	   147.11	  1  _
+2 charge
		      B   	       Y
  1	 N 	    58.03	        -  	 20
  2	 Y 	   139.56	  1165.05	 19
  3	 I 	   196.10	  1083.51	 18
  4	 S 	   239.62	  1026.97	 17
  5	 Q 	   303.65	   983.46	 16
  6	 Y 	   385.18	   919.43	 15
  7	 S 	   428.70	   837.90	 14
  8	 E 	   493.22	   794.38	 13
  9	 I 	   549.76	   729.86	 12
 10	 A 	   585.28	   673.32	 11
 11	 I 	   641.82	   637.80	 10
 12	 Q 	   705.85	   581.26	  9
 13	 M 	   771.37	   517.23	  8
 14	 M 	   836.89	   451.71	  7
 15	 M 	   902.41	   386.19	  6
 16	 H 	   970.94	   320.67	  5
 17	 M 	  1036.46	   252.14	  4
 18	 Q 	  1100.49	   186.62	  3
 19	 P 	  1149.01	   122.59	  2
 20	 K 	        -  	    74.06	  1  _
+3 charge
		      B   	       Y
  1	 N 	    39.02	        -  	 20
  2	 Y 	    93.38	   777.03	 19
  3	 I 	   131.07	   722.68	 18
  4	 S 	   160.08	   684.98	 17
  5	 Q 	   202.77	   655.97	 16
  6	 Y 	   257.12	   613.29	 15
  7	 S 	   286.13	   558.93	 14
  8	 E 	   329.15	   529.92	 13
  9	 I 	   366.84	   486.91	 12
 10	 A 	   390.52	   449.21	 11
 11	 I 	   428.22	   425.53	 10
 12	 Q 	   470.90	   387.84	  9
 13	 M 	   514.58	   345.15	  8
 14	 M 	   558.26	   301.47	  7
 15	 M 	   601.94	   257.79	  6
 16	 H 	   647.63	   214.11	  5
 17	 M 	   691.31	   168.43	  4
 18	 Q 	   733.99	   124.75	  3
 19	 P 	   766.35	    82.06	  2
 20	 K 	        -  	    49.71	  1  _
```

**94. IPI00011662 Kunitz-type protease inhibitor 2**

| Sequence | Charge | M+H+ | XCorr | DeltaCn |
| --- | --- | --- | --- | --- |
| R.SIHDFCLVSK.V | 2 | 1206.367 | 3.026 | 0.327 |

+1 charge

```
		      B   	       Y
  1	 S 	    88.04	        -  	 10
  2	 I 	   201.12	  1118.70	  9
  3	 H 	   338.18	  1005.62	  8
  4	 D 	   453.21	   868.56	  7
  5	 F 	   600.28	   753.53	  6
  6	 C 	   760.44	   606.46	  5
  7	 L 	   873.53	   446.30	  4
  8	 V 	   972.60	   333.21	  3
  9	 S 	  1059.63	   234.14	  2
 10	 K 	        -  	   147.11	  1  _
+2 charge
		      B   	       Y
  1	 S 	    44.52	        -  	 10
  2	 I 	   101.07	   559.85	  9
  3	 H 	   169.59	   503.31	  8
  4	 D 	   227.11	   434.78	  7
  5	 F 	   300.64	   377.27	  6
  6	 C 	   380.73	   303.73	  5
  7	 L 	   437.27	   223.65	  4
  8	 V 	   486.80	   167.11	  3
  9	 S 	   530.32	   117.58	  2
 10	 K 	        -  	    74.06	  1  _
```

**95. IPI00010676 Isoform 1 of Urokinase plasminogen activator surface receptor**

| Sequence | Charge | M+H+ | XCorr | DeltaCn |
| --- | --- | --- | --- | --- |
| K.TNGDCRVEECALGQDLCR.T | 3 | 2154.262 | 3.495 | 0.365 |

+1 charge

```
		      B   	       Y
  1	 T 	   102.05	        -  	 18
  2	 N 	   216.10	  2052.27	 17
  3	 G 	   273.12	  1938.23	 16
  4	 D 	   388.15	  1881.21	 15
  5	 C 	   548.31	  1766.18	 14
  6	 R 	   704.41	  1606.02	 13
  7	 V 	   803.48	  1449.92	 12
  8	 E 	   932.52	  1350.85	 11
  9	 E 	  1061.57	  1221.80	 10
 10	 C 	  1221.73	  1092.76	  9
 11	 A 	  1292.77	   932.60	  8
 12	 L 	  1405.85	   861.56	  7
 13	 G 	  1462.87	   748.48	  6
 14	 Q 	  1590.93	   691.45	  5
 15	 D 	  1705.96	   563.40	  4
 16	 L 	  1819.04	   448.37	  3
 17	 C 	  1979.21	   335.28	  2
 18	 R 	        -  	   175.12	  1  _
+2 charge
		      B   	       Y
  1	 T 	    51.53	        -  	 18
  2	 N 	   108.55	  1026.64	 17
  3	 G 	   137.06	   969.62	 16
  4	 D 	   194.58	   941.11	 15
  5	 C 	   274.66	   883.59	 14
  6	 R 	   352.71	   803.51	 13
  7	 V 	   402.24	   725.46	 12
  8	 E 	   466.77	   675.93	 11
  9	 E 	   531.29	   611.41	 10
 10	 C 	   611.37	   546.88	  9
 11	 A 	   646.89	   466.80	  8
 12	 L 	   703.43	   431.28	  7
 13	 G 	   731.94	   374.74	  6
 14	 Q 	   795.97	   346.23	  5
 15	 D 	   853.48	   282.20	  4
 16	 L 	   910.03	   224.69	  3
 17	 C 	   990.11	   168.15	  2
 18	 R 	        -  	    88.06	  1  _
+3 charge
		      B   	       Y
  1	 T 	    34.69	        -  	 18
  2	 N 	    72.70	   684.76	 17
  3	 G 	    91.71	   646.75	 16
  4	 D 	   130.05	   627.74	 15
  5	 C 	   183.44	   589.40	 14
  6	 R 	   235.48	   536.01	 13
  7	 V 	   268.50	   483.98	 12
  8	 E 	   311.51	   450.95	 11
  9	 E 	   354.53	   407.94	 10
 10	 C 	   407.92	   364.93	  9
 11	 A 	   431.59	   311.54	  8
 12	 L 	   469.29	   287.86	  7
 13	 G 	   488.30	   250.16	  6
 14	 Q 	   530.98	   231.16	  5
 15	 D 	   569.32	   188.47	  4
 16	 L 	   607.02	   150.13	  3
 17	 C 	   660.41	   112.43	  2
 18	 R 	        -  	    59.04	  1  _
```

**96. IPI00236556 Isoform H7 of Myeloperoxidase**

| Sequence | Charge | M+H+ | XCorr | DeltaCn |
| --- | --- | --- | --- | --- |
| R.AVSNEIVRFPTDQLTPDQER.S | 3 | 2316.513 | 4.642 | 0.493 |

+1 charge

```
		      B   	       Y
  1	 A 	    72.04	        -  	 20
  2	 V 	   171.11	  2244.13	 19
  3	 S 	   258.14	  2145.06	 18
  4	 N 	   372.19	  2058.03	 17
  5	 E 	   501.23	  1943.98	 16
  6	 I 	   614.31	  1814.94	 15
  7	 V 	   713.38	  1701.86	 14
  8	 R 	   869.48	  1602.79	 13
  9	 F 	  1016.55	  1446.69	 12
 10	 P 	  1113.61	  1299.62	 11
 11	 T 	  1214.65	  1202.56	 10
 12	 D 	  1329.68	  1101.52	  9
 13	 Q 	  1457.74	   986.49	  8
 14	 L 	  1570.82	   858.43	  7
 15	 T 	  1671.87	   745.35	  6
 16	 P 	  1768.92	   644.30	  5
 17	 D 	  1883.95	   547.25	  4
 18	 Q 	  2012.01	   432.22	  3
 19	 E 	  2141.05	   304.16	  2
 20	 R 	        -  	   175.12	  1  _
+2 charge
		      B   	       Y
  1	 A 	    36.53	        -  	 20
  2	 V 	    86.06	  1122.57	 19
  3	 S 	   129.58	  1073.03	 18
  4	 N 	   186.60	  1029.52	 17
  5	 E 	   251.12	   972.49	 16
  6	 I 	   307.66	   907.97	 15
  7	 V 	   357.20	   851.43	 14
  8	 R 	   435.25	   801.90	 13
  9	 F 	   508.78	   723.85	 12
 10	 P 	   557.31	   650.31	 11
 11	 T 	   607.83	   601.79	 10
 12	 D 	   665.34	   551.26	  9
 13	 Q 	   729.37	   493.75	  8
 14	 L 	   785.91	   429.72	  7
 15	 T 	   836.44	   373.18	  6
 16	 P 	   884.97	   322.65	  5
 17	 D 	   942.48	   274.13	  4
 18	 Q 	  1006.51	   216.61	  3
 19	 E 	  1071.03	   152.58	  2
 20	 R 	        -  	    88.06	  1  _

+3 charge
		      B   	       Y
  1	 A 	    24.69	        -  	 20
  2	 V 	    57.71	   748.71	 19
  3	 S 	    86.72	   715.69	 18
  4	 N 	   124.73	   686.68	 17
  5	 E 	   167.75	   648.67	 16
  6	 I 	   205.44	   605.65	 15
  7	 V 	   238.47	   567.96	 14
  8	 R 	   290.50	   534.93	 13
  9	 F 	   339.52	   482.90	 12
 10	 P 	   371.87	   433.88	 11
 11	 T 	   405.56	   401.53	 10
 12	 D 	   443.90	   367.84	  9
 13	 Q 	   486.58	   329.50	  8
 14	 L 	   524.28	   286.82	  7
 15	 T 	   557.96	   249.12	  6
 16	 P 	   590.31	   215.44	  5
 17	 D 	   628.65	   183.09	  4
 18	 Q 	   671.34	   144.74	  3
 19	 E 	   714.36	   102.06	  2
 20	 R 	        -  	    59.04	  1  _
```

**97. IPI00296058 EGF-containing fibulin-like extracellular matrix protein 2**

| Sequence | Charge | M+H+ | XCorr | DeltaCn |
| --- | --- | --- | --- | --- |
| K.CINHYGGYLCLPR.S | 2 | 1623.824 | 4.42 | 0.545 |

+1 charge

```
		      B   	       Y
  1	 C 	   161.17	        -  	 13
  2	 I 	   274.26	  1462.86	 12
  3	 N 	   388.30	  1349.78	 11
  4	 H 	   525.36	  1235.73	 10
  5	 Y 	   688.42	  1098.67	  9
  6	 G 	   745.44	   935.61	  8
  7	 G 	   802.46	   878.59	  7
  8	 Y 	   965.53	   821.57	  6
  9	 L 	  1078.61	   658.51	  5
 10	 C 	  1238.78	   545.42	  4
 11	 L 	  1351.86	   385.26	  3
 12	 P 	  1448.91	   272.17	  2
 13	 R 	        -  	   175.12	  1  _
+2 charge
		      B   	       Y
  1	 C 	    81.09	        -  	 13
  2	 I 	   137.63	   731.93	 12
  3	 N 	   194.65	   675.39	 11
  4	 H 	   263.18	   618.37	 10
  5	 Y 	   344.71	   549.84	  9
  6	 G 	   373.23	   468.31	  8
  7	 G 	   401.74	   439.80	  7
  8	 Y 	   483.27	   411.29	  6
  9	 L 	   539.81	   329.76	  5
 10	 C 	   619.89	   273.21	  4
 11	 L 	   676.43	   193.13	  3
 12	 P 	   724.96	   136.59	  2
 13	 R 	        -  	    88.06	  1  _
```

**98. IPI00009477 Intercellular adhesion molecule 2**

| Sequence | Charge | M+H+ | XCorr | DeltaCn |
| --- | --- | --- | --- | --- |
| R.QVILTLQPTLVAVGK.S | 2 | 1580.937 | 3.201 | 0.49 |

+1 charge

```
		      B   	       Y
  1	 Q 	   129.07	        -  	 15
  2	 V 	   228.13	  1451.92	 14
  3	 I 	   341.22	  1352.85	 13
  4	 L 	   454.30	  1239.77	 12
  5	 T 	   555.35	  1126.68	 11
  6	 L 	   668.43	  1025.64	 10
  7	 Q 	   796.49	   912.55	  9
  8	 P 	   893.55	   784.49	  8
  9	 T 	   994.59	   687.44	  7
 10	 L 	  1107.68	   586.39	  6
 11	 V 	  1206.75	   473.31	  5
 12	 A 	  1277.78	   374.24	  4
 13	 V 	  1376.85	   303.20	  3
 14	 G 	  1433.87	   204.13	  2
 15	 K 	        -  	   147.11	  1  _
+2 charge
		      B   	       Y
  1	 Q 	    65.04	        -  	 15
  2	 V 	   114.57	   726.46	 14
  3	 I 	   171.11	   676.93	 13
  4	 L 	   227.65	   620.39	 12
  5	 T 	   278.18	   563.85	 11
  6	 L 	   334.72	   513.32	 10
  7	 Q 	   398.75	   456.78	  9
  8	 P 	   447.28	   392.75	  8
  9	 T 	   497.80	   344.22	  7
 10	 L 	   554.34	   293.70	  6
 11	 V 	   603.88	   237.16	  5
 12	 A 	   639.40	   187.62	  4
 13	 V 	   688.93	   152.10	  3
 14	 G 	   717.44	   102.57	  2
 15	 K 	        -  	    74.06	  1  _
```

**99. IPI00409566 Isoform 1 of Polycystin-1**

| Sequence | Charge | M+H+ | XCorr | DeltaCn |
| --- | --- | --- | --- | --- |
| R.TLGPALRIPADATALDVSHNLLR.A | 3 | 2415.777 | 5.925 | 0.522 |

+1 charge

```
		      B   	       Y
  1	 T 	   102.05	        -  	 23
  2	 L 	   215.14	  2313.30	 22
  3	 G 	   272.16	  2200.22	 21
  4	 P 	   369.21	  2143.20	 20
  5	 A 	   440.25	  2046.15	 19
  6	 L 	   553.33	  1975.11	 18
  7	 R 	   709.44	  1862.02	 17
  8	 I 	   822.52	  1705.92	 16
  9	 P 	   919.57	  1592.84	 15
 10	 A 	   990.61	  1495.79	 14
 11	 D 	  1105.64	  1424.75	 13
 12	 A 	  1176.67	  1309.72	 12
 13	 T 	  1277.72	  1238.69	 11
 14	 A 	  1348.76	  1137.64	 10
 15	 L 	  1461.84	  1066.60	  9
 16	 D 	  1576.87	   953.52	  8
 17	 V 	  1675.94	   838.49	  7
 18	 S 	  1762.97	   739.42	  6
 19	 H 	  1900.03	   652.39	  5
 20	 N 	  2014.07	   515.33	  4
 21	 L 	  2127.16	   401.29	  3
 22	 L 	  2240.24	   288.20	  2
 23	 R 	        -  	   175.12	  1  _


+2 charge
		      B   	       Y
  1	 T 	    51.53	        -  	 23
  2	 L 	   108.07	  1157.16	 22
  3	 G 	   136.58	  1100.61	 21
  4	 P 	   185.11	  1072.10	 20
  5	 A 	   220.63	  1023.58	 19
  6	 L 	   277.17	   988.06	 18
  7	 R 	   355.22	   931.52	 17
  8	 I 	   411.76	   853.47	 16
  9	 P 	   460.29	   796.92	 15
 10	 A 	   495.81	   748.40	 14
 11	 D 	   553.32	   712.88	 13
 12	 A 	   588.84	   655.36	 12
 13	 T 	   639.36	   619.85	 11
 14	 A 	   674.88	   569.32	 10
 15	 L 	   731.42	   533.80	  9
 16	 D 	   788.94	   477.26	  8
 17	 V 	   838.47	   419.75	  7
 18	 S 	   881.99	   370.21	  6
 19	 H 	   950.52	   326.70	  5
 20	 N 	  1007.54	   258.17	  4
 21	 L 	  1064.08	   201.15	  3
 22	 L 	  1120.62	   144.61	  2
 23	 R 	        -  	    88.06	  1  _
+3 charge
		      B   	       Y
  1	 T 	    34.69	        -  	 23
  2	 L 	    72.38	   771.77	 22
  3	 G 	    91.39	   734.08	 21
  4	 P 	   123.74	   715.07	 20
  5	 A 	   147.42	   682.72	 19
  6	 L 	   185.12	   659.04	 18
  7	 R 	   237.15	   621.35	 17
  8	 I 	   274.84	   569.31	 16
  9	 P 	   307.20	   531.62	 15
 10	 A 	   330.87	   499.27	 14
 11	 D 	   369.22	   475.59	 13
 12	 A 	   392.90	   437.25	 12
 13	 T 	   426.58	   413.57	 11
 14	 A 	   450.26	   379.88	 10
 15	 L 	   487.95	   356.20	  9
 16	 D 	   526.29	   318.51	  8
 17	 V 	   559.32	   280.17	  7
 18	 S 	   588.33	   247.15	  6
 19	 H 	   634.01	   218.13	  5
 20	 N 	   672.03	   172.45	  4
 21	 L 	   709.72	   134.43	  3
 22	 L 	   747.42	    96.74	  2
 23	 R 	        -  	    59.04	  1  _
```

**100. IPI00921523 Isoform 1 of Complement factor B (Fragment)**

| Sequence | Charge | M+H+ | XCorr | DeltaCn |
| --- | --- | --- | --- | --- |
| R.LLQEGQALEYVCPSGFYPYPVQTR.T | 3 | 2817.138 | 3.602 | 0.25 |

+1 charge

```
		      B   	       Y
  1	 L 	   114.09	        -  	 24
  2	 L 	   227.18	  2702.43	 23
  3	 Q 	   355.23	  2589.34	 22
  4	 E 	   484.28	  2461.28	 21
  5	 G 	   541.30	  2332.24	 20
  6	 Q 	   669.36	  2275.22	 19
  7	 A 	   740.39	  2147.16	 18
  8	 L 	   853.48	  2076.12	 17
  9	 E 	   982.52	  1963.04	 16
 10	 Y 	  1145.58	  1834.00	 15
 11	 V 	  1244.65	  1670.93	 14
 12	 C 	  1404.82	  1571.87	 13
 13	 P 	  1501.87	  1411.70	 12
 14	 S 	  1588.90	  1314.65	 11
 15	 G 	  1645.92	  1227.62	 10
 16	 F 	  1792.99	  1170.59	  9
 17	 Y 	  1956.06	  1023.53	  8
 18	 P 	  2053.11	   860.46	  7
 19	 Y 	  2216.17	   763.41	  6
 20	 P 	  2313.22	   600.35	  5
 21	 V 	  2412.29	   503.29	  4
 22	 Q 	  2540.35	   404.23	  3
 23	 T 	  2641.40	   276.17	  2
 24	 R 	        -  	   175.12	  1  _


+2 charge
		      B   	       Y
  1	 L 	    57.55	        -  	 24
  2	 L 	   114.09	  1351.72	 23
  3	 Q 	   178.12	  1295.17	 22
  4	 E 	   242.64	  1231.15	 21
  5	 G 	   271.15	  1166.62	 20
  6	 Q 	   335.18	  1138.11	 19
  7	 A 	   370.70	  1074.08	 18
  8	 L 	   427.24	  1038.57	 17
  9	 E 	   491.76	   982.02	 16
 10	 Y 	   573.30	   917.50	 15
 11	 V 	   622.83	   835.97	 14
 12	 C 	   702.91	   786.44	 13
 13	 P 	   751.44	   706.35	 12
 14	 S 	   794.95	   657.83	 11
 15	 G 	   823.47	   614.31	 10
 16	 F 	   897.00	   585.80	  9
 17	 Y 	   978.53	   512.27	  8
 18	 P 	  1027.06	   430.73	  7
 19	 Y 	  1108.59	   382.21	  6
 20	 P 	  1157.12	   300.68	  5
 21	 V 	  1206.65	   252.15	  4
 22	 Q 	  1270.68	   202.62	  3
 23	 T 	  1321.20	   138.59	  2
 24	 R 	        -  	    88.06	  1  _


+3 charge
		      B   	       Y
  1	 L 	    38.70	        -  	 24
  2	 L 	    76.40	   901.48	 23
  3	 Q 	   119.08	   863.79	 22
  4	 E 	   162.10	   821.10	 21
  5	 G 	   181.10	   778.09	 20
  6	 Q 	   223.79	   759.08	 19
  7	 A 	   247.47	   716.39	 18
  8	 L 	   285.16	   692.71	 17
  9	 E 	   328.18	   655.02	 16
 10	 Y 	   382.53	   612.00	 15
 11	 V 	   415.56	   557.65	 14
 12	 C 	   468.94	   524.63	 13
 13	 P 	   501.29	   471.24	 12
 14	 S 	   530.31	   438.89	 11
 15	 G 	   549.31	   409.88	 10
 16	 F 	   598.34	   390.87	  9
 17	 Y 	   652.69	   341.85	  8
 18	 P 	   685.04	   287.49	  7
 19	 Y 	   739.40	   255.14	  6
 20	 P 	   771.75	   200.79	  5
 21	 V 	   804.77	   168.44	  4
 22	 Q 	   847.46	   135.41	  3
 23	 T 	   881.14	    92.73	  2
 24	 R 	        -  	    59.04	  1  _
```

**101. IPI00027462 Protein S100-A9**

| Sequence | Charge | M+H+ | XCorr | DeltaCn |
| --- | --- | --- | --- | --- |
| R.NIETIINTFHQYSVK.L | 2 | 1808.027 | 4.697 | 0.53 |

+1 charge

```
		      B   	       Y
  1	 N 	   115.05	        -  	 15
  2	 I 	   228.13	  1692.90	 14
  3	 E 	   357.18	  1579.81	 13
  4	 T 	   458.22	  1450.77	 12
  5	 I 	   571.31	  1349.72	 11
  6	 I 	   684.39	  1236.64	 10
  7	 N 	   798.44	  1123.55	  9
  8	 T 	   899.48	  1009.51	  8
  9	 F 	  1046.55	   908.46	  7
 10	 H 	  1183.61	   761.39	  6
 11	 Q 	  1311.67	   624.34	  5
 12	 Y 	  1474.73	   496.28	  4
 13	 S 	  1561.76	   333.21	  3
 14	 V 	  1660.83	   246.18	  2
 15	 K 	        -  	   147.11	  1  _
+2 charge
		      B   	       Y
  1	 N 	    58.03	        -  	 15
  2	 I 	   114.57	   846.95	 14
  3	 E 	   179.09	   790.41	 13
  4	 T 	   229.62	   725.89	 12
  5	 I 	   286.16	   675.36	 11
  6	 I 	   342.70	   618.82	 10
  7	 N 	   399.72	   562.28	  9
  8	 T 	   450.25	   505.26	  8
  9	 F 	   523.78	   454.73	  7
 10	 H 	   592.31	   381.20	  6
 11	 Q 	   656.34	   312.67	  5
 12	 Y 	   737.87	   248.64	  4
 13	 S 	   781.39	   167.11	  3
 14	 V 	   830.92	   123.59	  2
 15	 K 	        -  	    74.06	  1  _
```

**102.IPI00019954 Cystatin-M**

| Sequence | Charge | M+H+ | XCorr | DeltaCn |
| --- | --- | --- | --- | --- |
| R.DLSPDDPQVQKAAQAAVASYNMGSNSIYYFR.D | 3 | 3408.699 | 4.917 | 0.714 |

+1 charge

```
		      B   	       Y
  1	 D 	   116.03	        -  	 31
  2	 L 	   229.12	  3291.57	 30
  3	 S 	   316.15	  3178.49	 29
  4	 P 	   413.20	  3091.46	 28
  5	 D 	   528.23	  2994.41	 27
  6	 D 	   643.26	  2879.38	 26
  7	 P 	   740.31	  2764.35	 25
  8	 Q 	   868.37	  2667.30	 24
  9	 V 	   967.44	  2539.24	 23
 10	 Q 	  1095.50	  2440.17	 22
 11	 K 	  1223.59	  2312.11	 21
 12	 A 	  1294.63	  2184.02	 20
 13	 A 	  1365.66	  2112.98	 19
 14	 Q 	  1493.72	  2041.94	 18
 15	 A 	  1564.76	  1913.89	 17
 16	 A 	  1635.80	  1842.85	 16
 17	 V 	  1734.87	  1771.81	 15
 18	 A 	  1805.90	  1672.74	 14
 19	 S 	  1892.93	  1601.71	 13
 20	 Y 	  2056.00	  1514.67	 12
 21	 N 	  2170.04	  1351.61	 11
 22	 M 	  2301.08	  1237.57	 10
 23	 G 	  2358.10	  1106.53	  9
 24	 S 	  2445.14	  1049.51	  8
 25	 N 	  2559.18	   962.47	  7
 26	 S 	  2646.21	   848.43	  6
 27	 I 	  2759.29	   761.40	  5
 28	 Y 	  2922.36	   648.31	  4
 29	 Y 	  3085.42	   485.25	  3
 30	 F 	  3232.49	   322.19	  2
 31	 R 	        -  	   175.12	  1  _


+2 charge
		      B   	       Y
  1	 D 	    58.52	        -  	 31
  2	 L 	   115.06	  1646.29	 30
  3	 S 	   158.58	  1589.75	 29
  4	 P 	   207.11	  1546.23	 28
  5	 D 	   264.62	  1497.71	 27
  6	 D 	   322.13	  1440.19	 26
  7	 P 	   370.66	  1382.68	 25
  8	 Q 	   434.69	  1334.15	 24
  9	 V 	   484.22	  1270.12	 23
 10	 Q 	   548.25	  1220.59	 22
 11	 K 	   612.30	  1156.56	 21
 12	 A 	   647.82	  1092.51	 20
 13	 A 	   683.34	  1056.99	 19
 14	 Q 	   747.37	  1021.48	 18
 15	 A 	   782.88	   957.45	 17
 16	 A 	   818.40	   921.93	 16
 17	 V 	   867.94	   886.41	 15
 18	 A 	   903.46	   836.87	 14
 19	 S 	   946.97	   801.36	 13
 20	 Y 	  1028.50	   757.84	 12
 21	 N 	  1085.52	   676.31	 11
 22	 M 	  1151.04	   619.29	 10
 23	 G 	  1179.56	   553.77	  9
 24	 S 	  1223.07	   525.26	  8
 25	 N 	  1280.09	   481.74	  7
 26	 S 	  1323.61	   424.72	  6
 27	 I 	  1380.15	   381.20	  5
 28	 Y 	  1461.68	   324.66	  4
 29	 Y 	  1543.21	   243.13	  3
 30	 F 	  1616.75	   161.60	  2
 31	 R 	        -  	    88.06	  1  _


+3 charge
		      B   	       Y
  1	 D 	    39.35	        -  	 31
  2	 L 	    77.04	  1097.86	 30
  3	 S 	   106.05	  1060.17	 29
  4	 P 	   138.41	  1031.16	 28
  5	 D 	   176.75	   998.81	 27
  6	 D 	   215.09	   960.46	 26
  7	 P 	   247.44	   922.12	 25
  8	 Q 	   290.13	   889.77	 24
  9	 V 	   323.15	   847.08	 23
 10	 Q 	   365.84	   814.06	 22
 11	 K 	   408.53	   771.38	 21
 12	 A 	   432.21	   728.68	 20
 13	 A 	   455.89	   705.00	 19
 14	 Q 	   498.58	   681.32	 18
 15	 A 	   522.26	   638.63	 17
 16	 A 	   545.94	   614.95	 16
 17	 V 	   578.96	   591.28	 15
 18	 A 	   602.64	   558.25	 14
 19	 S 	   631.65	   534.57	 13
 20	 Y 	   686.00	   505.56	 12
 21	 N 	   724.02	   451.21	 11
 22	 M 	   767.70	   413.19	 10
 23	 G 	   786.71	   369.51	  9
 24	 S 	   815.72	   350.51	  8
 25	 N 	   853.73	   321.50	  7
 26	 S 	   882.74	   283.48	  6
 27	 I 	   920.44	   254.47	  5
 28	 Y 	   974.79	   216.78	  4
 29	 Y 	  1029.15	   162.42	  3
 30	 F 	  1078.17	   108.07	  2
 31	 R 	        -  	    59.04	  1  _
```

**103. IPI00019502 Isoform 1 of Myosin-9**

| Sequence | Charge | M+H+ | XCorr | DeltaCn |
| --- | --- | --- | --- | --- |
| K.SMEAEMIQLQEELAAAER.A | 2 | 2050.301 | 3.121 | 0.414 |

+1 charge

```
		      B   	       Y
  1	 S 	    88.04	        -  	 18
  2	 M 	   219.08	  1961.93	 17
  3	 E 	   348.12	  1830.89	 16
  4	 A 	   419.16	  1701.85	 15
  5	 E 	   548.20	  1630.81	 14
  6	 M 	   679.24	  1501.77	 13
  7	 I 	   792.33	  1370.73	 12
  8	 Q 	   920.39	  1257.64	 11
  9	 L 	  1033.47	  1129.58	 10
 10	 Q 	  1161.53	  1016.50	  9
 11	 E 	  1290.57	   888.44	  8
 12	 E 	  1419.61	   759.40	  7
 13	 L 	  1532.70	   630.36	  6
 14	 A 	  1603.73	   517.27	  5
 15	 A 	  1674.77	   446.24	  4
 16	 A 	  1745.81	   375.20	  3
 17	 E 	  1874.85	   304.16	  2
 18	 R 	        -  	   175.12	  1  _
+2 charge
		      B   	       Y
  1	 S 	    44.52	        -  	 18
  2	 M 	   110.04	   981.47	 17
  3	 E 	   174.56	   915.95	 16
  4	 A 	   210.08	   851.43	 15
  5	 E 	   274.60	   815.91	 14
  6	 M 	   340.12	   751.39	 13
  7	 I 	   396.67	   685.87	 12
  8	 Q 	   460.70	   629.33	 11
  9	 L 	   517.24	   565.30	 10
 10	 Q 	   581.27	   508.75	  9
 11	 E 	   645.79	   444.72	  8
 12	 E 	   710.31	   380.20	  7
 13	 L 	   766.85	   315.68	  6
 14	 A 	   802.37	   259.14	  5
 15	 A 	   837.89	   223.62	  4
 16	 A 	   873.41	   188.10	  3
 17	 E 	   937.93	   152.58	  2
 18	 R 	        -  	    88.06	  1  _
```

**104. IPI00011229 Cathepsin D**

| Sequence | Charge | M+H+ | XCorr | DeltaCn |
| --- | --- | --- | --- | --- |
| R.ISVNNVLPVFDNLMQQK.L | 2 | 1960.287 | 2.832 | 0.318 |

+1 charge

```
		      B   	       Y
  1	 I 	   114.09	        -  	 17
  2	 S 	   201.12	  1845.95	 16
  3	 V 	   300.19	  1758.92	 15
  4	 N 	   414.23	  1659.85	 14
  5	 N 	   528.28	  1545.81	 13
  6	 V 	   627.35	  1431.77	 12
  7	 L 	   740.43	  1332.70	 11
  8	 P 	   837.48	  1219.61	 10
  9	 V 	   936.55	  1122.56	  9
 10	 F 	  1083.62	  1023.49	  8
 11	 D 	  1198.65	   876.42	  7
 12	 N 	  1312.69	   761.40	  6
 13	 L 	  1425.77	   647.35	  5
 14	 M 	  1556.81	   534.27	  4
 15	 Q 	  1684.87	   403.23	  3
 16	 Q 	  1812.93	   275.17	  2
 17	 K 	        -  	   147.11	  1  _
+2 charge
		      B   	       Y
  1	 I 	    57.55	        -  	 17
  2	 S 	   101.07	   923.48	 16
  3	 V 	   150.60	   879.96	 15
  4	 N 	   207.62	   830.43	 14
  5	 N 	   264.64	   773.41	 13
  6	 V 	   314.18	   716.39	 12
  7	 L 	   370.72	   666.85	 11
  8	 P 	   419.25	   610.31	 10
  9	 V 	   468.78	   561.78	  9
 10	 F 	   542.31	   512.25	  8
 11	 D 	   599.83	   438.72	  7
 12	 N 	   656.85	   381.20	  6
 13	 L 	   713.39	   324.18	  5
 14	 M 	   778.91	   267.64	  4
 15	 Q 	   842.94	   202.12	  3
 16	 Q 	   906.97	   138.09	  2
 17	 K 	        -  	    74.06	  1  _
```

**105. IPI00027436 Tumor necrosis factor receptor superfamily member 16**

| Sequence | Charge | M+H+ | XCorr | DeltaCn |
| --- | --- | --- | --- | --- |
| R.WADAECEEIPGR.W | 2 | 1433.499 | 3.416 | 0.372 |

+1 charge

```
		      B   	       Y
  1	 W 	   187.09	        -  	 12
  2	 A 	   258.12	  1246.67	 11
  3	 D 	   373.15	  1175.63	 10
  4	 A 	   444.19	  1060.61	  9
  5	 E 	   573.23	   989.57	  8
  6	 C 	   733.40	   860.53	  7
  7	 E 	   862.44	   700.36	  6
  8	 E 	   991.48	   571.32	  5
  9	 I 	  1104.56	   442.28	  4
 10	 P 	  1201.62	   329.19	  3
 11	 G 	  1258.64	   232.14	  2
 12	 R 	        -  	   175.12	  1  _
+2 charge
		      B   	       Y
  1	 W 	    94.05	        -  	 12
  2	 A 	   129.57	   623.84	 11
  3	 D 	   187.08	   588.32	 10
  4	 A 	   222.60	   530.81	  9
  5	 E 	   287.12	   495.29	  8
  6	 C 	   367.20	   430.77	  7
  7	 E 	   431.72	   350.68	  6
  8	 E 	   496.24	   286.16	  5
  9	 I 	   552.79	   221.64	  4
 10	 P 	   601.31	   165.10	  3
 11	 G 	   629.82	   116.57	  2
 12	 R 	        -  	    88.06	  1  _
```

**106. IPI00298388 Isoform 1 of Phosphoinositide-3-kinase-interacting protein 1**

| Sequence | Charge | M+H+ | XCorr | DeltaCn |
| --- | --- | --- | --- | --- |
| R.SEAAAVQPVIGISQR.V | 2 | 1526.72 | 4.833 | 0.516 |

+1 charge

```
		      B   	       Y
  1	 S 	    88.04	        -  	 15
  2	 E 	   217.08	  1438.80	 14
  3	 A 	   288.12	  1309.76	 13
  4	 A 	   359.16	  1238.72	 12
  5	 A 	   430.19	  1167.68	 11
  6	 V 	   529.26	  1096.65	 10
  7	 Q 	   657.32	   997.58	  9
  8	 P 	   754.37	   869.52	  8
  9	 V 	   853.44	   772.47	  7
 10	 I 	   966.53	   673.40	  6
 11	 G 	  1023.55	   560.32	  5
 12	 I 	  1136.63	   503.29	  4
 13	 S 	  1223.66	   390.21	  3
 14	 Q 	  1351.72	   303.18	  2
 15	 R 	        -  	   175.12	  1  _
+2 charge
		      B   	       Y
  1	 S 	    44.52	        -  	 15
  2	 E 	   109.04	   719.90	 14
  3	 A 	   144.56	   655.38	 13
  4	 A 	   180.08	   619.86	 12
  5	 A 	   215.60	   584.35	 11
  6	 V 	   265.13	   548.83	 10
  7	 Q 	   329.16	   499.29	  9
  8	 P 	   377.69	   435.26	  8
  9	 V 	   427.22	   386.74	  7
 10	 I 	   483.77	   337.20	  6
 11	 G 	   512.28	   280.66	  5
 12	 I 	   568.82	   252.15	  4
 13	 S 	   612.34	   195.61	  3
 14	 Q 	   676.36	   152.09	  2
 15	 R 	        -  	    88.06	  1  _
```

**107. IPI00329688 Protein YIPF3**

| Sequence | Charge | M+H+ | XCorr | DeltaCn |
| --- | --- | --- | --- | --- |
| R.DIPAMLPAAR.L | 1 | 1055.276 | 2.41 | 0.398 |

+1 charge

```
		      B   	       Y
  1	 D 	   116.03	        -  	 10
  2	 I 	   229.12	   939.54	  9
  3	 P 	   326.17	   826.46	  8
  4	 A 	   397.21	   729.41	  7
  5	 M 	   528.25	   658.37	  6
  6	 L 	   641.33	   527.33	  5
  7	 P 	   738.39	   414.25	  4
  8	 A 	   809.42	   317.19	  3
  9	 A 	   880.46	   246.16	  2
 10	 R 	        -  	   175.12	  1  _
```

**108. IPI00009901 Nuclear transport factor 2**

| Sequence | Charge | M+H+ | XCorr | DeltaCn |
| --- | --- | --- | --- | --- |
| K.AAIVEKLSSLPFQK.I | 2 | 1531.821 | 3.667 | 0.497 |

+1 charge

```
		      B   	       Y
  1	 A 	    72.04	        -  	 14
  2	 A 	   143.08	  1459.85	 13
  3	 I 	   256.17	  1388.81	 12
  4	 V 	   355.23	  1275.73	 11
  5	 E 	   484.28	  1176.66	 10
  6	 K 	   612.37	  1047.62	  9
  7	 L 	   725.46	   919.52	  8
  8	 S 	   812.49	   806.44	  7
  9	 S 	   899.52	   719.41	  6
 10	 L 	  1012.60	   632.38	  5
 11	 P 	  1109.66	   519.29	  4
 12	 F 	  1256.72	   422.24	  3
 13	 Q 	  1384.78	   275.17	  2
 14	 K 	        -  	   147.11	  1  _
+2 charge
		      B   	       Y
  1	 A 	    36.53	        -  	 14
  2	 A 	    72.04	   730.43	 13
  3	 I 	   128.59	   694.91	 12
  4	 V 	   178.12	   638.37	 11
  5	 E 	   242.64	   588.83	 10
  6	 K 	   306.69	   524.31	  9
  7	 L 	   363.23	   460.27	  8
  8	 S 	   406.75	   403.72	  7
  9	 S 	   450.26	   360.21	  6
 10	 L 	   506.81	   316.69	  5
 11	 P 	   555.33	   260.15	  4
 12	 F 	   628.87	   211.62	  3
 13	 Q 	   692.90	   138.09	  2
 14	 K 	        -  	    74.06	  1  _
```

**109. IPI00006608 Isoform APP770 of Amyloid beta A4 protein (Fragment)**

| Sequence | Charge | M+H+ | XCorr | DeltaCn |
| --- | --- | --- | --- | --- |
| R.WYFDVTEGK.C | 2 | 1145.245 | 2.881 | 0.418 |

+1 charge

```
		      B   	       Y
  1	 W 	   187.09	        -  	  9
  2	 Y 	   350.15	   958.45	  8
  3	 F 	   497.22	   795.39	  7
  4	 D 	   612.25	   648.32	  6
  5	 V 	   711.31	   533.29	  5
  6	 T 	   812.36	   434.22	  4
  7	 E 	   941.40	   333.18	  3
  8	 G 	   998.43	   204.13	  2
  9	 K 	        -  	   147.11	  1  _
+2 charge
		      B   	       Y
  1	 W 	    94.05	        -  	  9
  2	 Y 	   175.58	   479.73	  8
  3	 F 	   249.11	   398.20	  7
  4	 D 	   306.63	   324.66	  6
  5	 V 	   356.16	   267.15	  5
  6	 T 	   406.68	   217.62	  4
  7	 E 	   471.21	   167.09	  3
  8	 G 	   499.72	   102.57	  2
  9	 K 	        -  	    74.06	  1  _
```

**110. IPI00015199 T-cell antigen CD7**

| Sequence | Charge | M+H+ | XCorr | DeltaCn |
| --- | --- | --- | --- | --- |
| R.QLGPQPQDIIYYEDGVVPTTDRR.F | 3 | 2661.908 | 3.316 | 0.416 |

+1 charge

```
		      B   	       Y
  1	 Q 	   129.07	        -  	 23
  2	 L 	   242.15	  2532.27	 22
  3	 G 	   299.17	  2419.19	 21
  4	 P 	   396.22	  2362.17	 20
  5	 Q 	   524.28	  2265.11	 19
  6	 P 	   621.34	  2137.06	 18
  7	 Q 	   749.39	  2040.00	 17
  8	 D 	   864.42	  1911.94	 16
  9	 I 	   977.51	  1796.92	 15
 10	 I 	  1090.59	  1683.83	 14
 11	 Y 	  1253.65	  1570.75	 13
 12	 Y 	  1416.72	  1407.69	 12
 13	 E 	  1545.76	  1244.62	 11
 14	 D 	  1660.79	  1115.58	 10
 15	 G 	  1717.81	  1000.55	  9
 16	 V 	  1816.88	   943.53	  8
 17	 V 	  1915.94	   844.46	  7
 18	 P 	  2013.00	   745.40	  6
 19	 T 	  2114.04	   648.34	  5
 20	 T 	  2215.09	   547.29	  4
 21	 D 	  2330.12	   446.25	  3
 22	 R 	  2486.22	   331.22	  2
 23	 R 	        -  	   175.12	  1  _


+2 charge
		      B   	       Y
  1	 Q 	    65.04	        -  	 23
  2	 L 	   121.58	  1266.64	 22
  3	 G 	   150.09	  1210.10	 21
  4	 P 	   198.62	  1181.59	 20
  5	 Q 	   262.64	  1133.06	 19
  6	 P 	   311.17	  1069.03	 18
  7	 Q 	   375.20	  1020.51	 17
  8	 D 	   432.71	   956.48	 16
  9	 I 	   489.26	   898.96	 15
 10	 I 	   545.80	   842.42	 14
 11	 Y 	   627.33	   785.88	 13
 12	 Y 	   708.86	   704.35	 12
 13	 E 	   773.38	   622.82	 11
 14	 D 	   830.90	   558.29	 10
 15	 G 	   859.41	   500.78	  9
 16	 V 	   908.94	   472.27	  8
 17	 V 	   958.48	   422.74	  7
 18	 P 	  1007.00	   373.20	  6
 19	 T 	  1057.53	   324.67	  5
 20	 T 	  1108.05	   274.15	  4
 21	 D 	  1165.56	   223.63	  3
 22	 R 	  1243.61	   166.11	  2
 23	 R 	        -  	    88.06	  1  _
+3 charge
		      B   	       Y
  1	 Q 	    43.69	        -  	 23
  2	 L 	    81.39	   844.76	 22
  3	 G 	   100.40	   807.07	 21
  4	 P 	   132.75	   788.06	 20
  5	 Q 	   175.43	   755.71	 19
  6	 P 	   207.78	   713.02	 18
  7	 Q 	   250.47	   680.67	 17
  8	 D 	   288.81	   637.99	 16
  9	 I 	   326.51	   599.64	 15
 10	 I 	   364.20	   561.95	 14
 11	 Y 	   418.56	   524.25	 13
 12	 Y 	   472.91	   469.90	 12
 13	 E 	   515.92	   415.55	 11
 14	 D 	   554.27	   372.53	 10
 15	 G 	   573.27	   334.19	  9
 16	 V 	   606.30	   315.18	  8
 17	 V 	   639.32	   282.16	  7
 18	 P 	   671.67	   249.14	  6
 19	 T 	   705.35	   216.79	  5
 20	 T 	   739.04	   183.10	  4
 21	 D 	   777.38	   149.42	  3
 22	 R 	   829.41	   111.08	  2
 23	 R 	        -  	    59.04	  1  _
```

**111. IPI00029235 Insulin-like growth factor-binding protein 6**

| Sequence | Charge | M+H+ | XCorr | DeltaCn |
| --- | --- | --- | --- | --- |
| R.HLDSVLQQLQTEVYR.G | 2 | 1830.035 | 3.749 | 0.466 |

+1 charge

```
		      B   	       Y
  1	 H 	   138.07	        -  	 15
  2	 L 	   251.15	  1691.90	 14
  3	 D 	   366.18	  1578.81	 13
  4	 S 	   453.21	  1463.79	 12
  5	 V 	   552.28	  1376.75	 11
  6	 L 	   665.36	  1277.68	 10
  7	 Q 	   793.42	  1164.60	  9
  8	 Q 	   921.48	  1036.54	  8
  9	 L 	  1034.56	   908.48	  7
 10	 Q 	  1162.62	   795.40	  6
 11	 T 	  1263.67	   667.34	  5
 12	 E 	  1392.71	   566.29	  4
 13	 V 	  1491.78	   437.25	  3
 14	 Y 	  1654.84	   338.18	  2
 15	 R 	        -  	   175.12	  1  _
+2 charge
		      B   	       Y
  1	 H 	    69.54	        -  	 15
  2	 L 	   126.08	   846.45	 14
  3	 D 	   183.59	   789.91	 13
  4	 S 	   227.11	   732.40	 12
  5	 V 	   276.64	   688.88	 11
  6	 L 	   333.18	   639.35	 10
  7	 Q 	   397.21	   582.80	  9
  8	 Q 	   461.24	   518.77	  8
  9	 L 	   517.79	   454.75	  7
 10	 Q 	   581.81	   398.20	  6
 11	 T 	   632.34	   334.17	  5
 12	 E 	   696.86	   283.65	  4
 13	 V 	   746.39	   219.13	  3
 14	 Y 	   827.93	   169.59	  2
 15	 R 	        -  	    88.06	  1  _
```

**112. IPI00298860 cDNA FLJ78440, highly similar to Human lactoferrin**

| Sequence | Charge | M+H+ | XCorr | DeltaCn |
| --- | --- | --- | --- | --- |
| R.SVNGKEDAIWNLLR.Q | 2 | 1615.815 | 2.938 | 0.345 |

+1 charge

```
		      B   	       Y
  1	 S 	    88.04	        -  	 14
  2	 V 	   187.11	  1527.83	 13
  3	 N 	   301.15	  1428.76	 12
  4	 G 	   358.17	  1314.72	 11
  5	 K 	   486.27	  1257.69	 10
  6	 E 	   615.31	  1129.60	  9
  7	 D 	   730.34	  1000.56	  8
  8	 A 	   801.37	   885.53	  7
  9	 I 	   914.46	   814.49	  6
 10	 W 	  1100.54	   701.41	  5
 11	 N 	  1214.58	   515.33	  4
 12	 L 	  1327.66	   401.29	  3
 13	 L 	  1440.75	   288.20	  2
 14	 R 	        -  	   175.12	  1  _
+2 charge
		      B   	       Y
  1	 S 	    44.52	        -  	 14
  2	 V 	    94.06	   764.42	 13
  3	 N 	   151.08	   714.88	 12
  4	 G 	   179.59	   657.86	 11
  5	 K 	   243.64	   629.35	 10
  6	 E 	   308.16	   565.30	  9
  7	 D 	   365.67	   500.78	  8
  8	 A 	   401.19	   443.27	  7
  9	 I 	   457.73	   407.75	  6
 10	 W 	   550.77	   351.21	  5
 11	 N 	   607.79	   258.17	  4
 12	 L 	   664.34	   201.15	  3
 13	 L 	   720.88	   144.61	  2
 14	 R 	        -  	    88.06	  1  _
```

**113. IPI00220644 Isoform M1 of Pyruvate kinase isozymes M1/M2**

| Sequence | Charge | M+H+ | XCorr | DeltaCn |
| --- | --- | --- | --- | --- |
| R.TATESFASDPILYRPVAVALDTKGPEIR.T | 3 | 3019.398 | 3.584 | 0.409 |

+1 charge

```
		      B   	       Y
  1	 T 	   102.05	        -  	 28
  2	 A 	   173.09	  2916.55	 27
  3	 T 	   274.14	  2845.51	 26
  4	 E 	   403.18	  2744.46	 25
  5	 S 	   490.21	  2615.42	 24
  6	 F 	   637.28	  2528.39	 23
  7	 A 	   708.32	  2381.32	 22
  8	 S 	   795.35	  2310.28	 21
  9	 D 	   910.38	  2223.25	 20
 10	 P 	  1007.43	  2108.22	 19
 11	 I 	  1120.52	  2011.17	 18
 12	 L 	  1233.60	  1898.09	 17
 13	 Y 	  1396.66	  1785.00	 16
 14	 R 	  1552.76	  1621.94	 15
 15	 P 	  1649.82	  1465.84	 14
 16	 V 	  1748.89	  1368.78	 13
 17	 A 	  1819.92	  1269.72	 12
 18	 V 	  1918.99	  1198.68	 11
 19	 A 	  1990.03	  1099.61	 10
 20	 L 	  2103.11	  1028.57	  9
 21	 D 	  2218.14	   915.49	  8
 22	 T 	  2319.19	   800.46	  7
 23	 K 	  2447.28	   699.41	  6
 24	 G 	  2504.30	   571.32	  5
 25	 P 	  2601.36	   514.30	  4
 26	 E 	  2730.40	   417.25	  3
 27	 I 	  2843.48	   288.20	  2
 28	 R 	        -  	   175.12	  1  _


+2 charge
		      B   	       Y
  1	 T 	    51.53	        -  	 28
  2	 A 	    87.05	  1458.78	 27
  3	 T 	   137.57	  1423.26	 26
  4	 E 	   202.09	  1372.73	 25
  5	 S 	   245.61	  1308.21	 24
  6	 F 	   319.15	  1264.70	 23
  7	 A 	   354.66	  1191.16	 22
  8	 S 	   398.18	  1155.64	 21
  9	 D 	   455.69	  1112.13	 20
 10	 P 	   504.22	  1054.61	 19
 11	 I 	   560.76	  1006.09	 18
 12	 L 	   617.30	   949.55	 17
 13	 Y 	   698.84	   893.00	 16
 14	 R 	   776.89	   811.47	 15
 15	 P 	   825.41	   733.42	 14
 16	 V 	   874.95	   684.90	 13
 17	 A 	   910.46	   635.36	 12
 18	 V 	   960.00	   599.84	 11
 19	 A 	   995.52	   550.31	 10
 20	 L 	  1052.06	   514.79	  9
 21	 D 	  1109.57	   458.25	  8
 22	 T 	  1160.10	   400.73	  7
 23	 K 	  1224.14	   350.21	  6
 24	 G 	  1252.66	   286.16	  5
 25	 P 	  1301.18	   257.65	  4
 26	 E 	  1365.70	   209.13	  3
 27	 I 	  1422.24	   144.61	  2
 28	 R 	        -  	    88.06	  1  _


+3 charge
		      B   	       Y
  1	 T 	    34.69	        -  	 28
  2	 A 	    58.37	   972.85	 27
  3	 T 	    92.05	   949.17	 26
  4	 E 	   135.07	   915.49	 25
  5	 S 	   164.08	   872.48	 24
  6	 F 	   213.10	   843.47	 23
  7	 A 	   236.78	   794.44	 22
  8	 S 	   265.79	   770.77	 21
  9	 D 	   304.13	   741.75	 20
 10	 P 	   336.48	   703.41	 19
 11	 I 	   374.18	   671.06	 18
 12	 L 	   411.87	   633.37	 17
 13	 Y 	   466.23	   595.67	 16
 14	 R 	   518.26	   541.32	 15
 15	 P 	   550.61	   489.28	 14
 16	 V 	   583.63	   456.93	 13
 17	 A 	   607.31	   423.91	 12
 18	 V 	   640.34	   400.23	 11
 19	 A 	   664.01	   367.21	 10
 20	 L 	   701.71	   343.53	  9
 21	 D 	   740.05	   305.83	  8
 22	 T 	   773.73	   267.49	  7
 23	 K 	   816.43	   233.81	  6
 24	 G 	   835.44	   191.11	  5
 25	 P 	   867.79	   172.10	  4
 26	 E 	   910.80	   139.75	  3
 27	 I 	   948.50	    96.74	  2
 28	 R 	        -  	    59.04	  1  _
```

**114. IPI00026314 Isoform 1 of Gelsolin**

| Sequence | Charge | M+H+ | XCorr | DeltaCn |
| --- | --- | --- | --- | --- |
| R.AQPVQVAEGSEPDGFWEALGGK.A | 2 | 2273.443 | 3.509 | 0.347 |

+1 charge

```
		      B   	       Y
  1	 A 	    72.04	        -  	 22
  2	 Q 	   200.10	  2201.05	 21
  3	 P 	   297.16	  2072.99	 20
  4	 V 	   396.22	  1975.94	 19
  5	 Q 	   524.28	  1876.87	 18
  6	 V 	   623.35	  1748.81	 17
  7	 A 	   694.39	  1649.74	 16
  8	 E 	   823.43	  1578.71	 15
  9	 G 	   880.45	  1449.66	 14
 10	 S 	   967.48	  1392.64	 13
 11	 E 	  1096.53	  1305.61	 12
 12	 P 	  1193.58	  1176.57	 11
 13	 D 	  1308.61	  1079.52	 10
 14	 G 	  1365.63	   964.49	  9
 15	 F 	  1512.70	   907.47	  8
 16	 W 	  1698.78	   760.40	  7
 17	 E 	  1827.82	   574.32	  6
 18	 A 	  1898.86	   445.28	  5
 19	 L 	  2011.94	   374.24	  4
 20	 G 	  2068.96	   261.16	  3
 21	 G 	  2125.98	   204.13	  2
 22	 K 	        -  	   147.11	  1  _


+2 charge
		      B   	       Y
  1	 A 	    36.53	        -  	 22
  2	 Q 	   100.56	  1101.03	 21
  3	 P 	   149.08	  1037.00	 20
  4	 V 	   198.62	   988.47	 19
  5	 Q 	   262.64	   938.94	 18
  6	 V 	   312.18	   874.91	 17
  7	 A 	   347.70	   825.38	 16
  8	 E 	   412.22	   789.86	 15
  9	 G 	   440.73	   725.34	 14
 10	 S 	   484.25	   696.83	 13
 11	 E 	   548.77	   653.31	 12
 12	 P 	   597.29	   588.79	 11
 13	 D 	   654.81	   540.26	 10
 14	 G 	   683.32	   482.75	  9
 15	 F 	   756.85	   454.24	  8
 16	 W 	   849.89	   380.70	  7
 17	 E 	   914.41	   287.66	  6
 18	 A 	   949.93	   223.14	  5
 19	 L 	  1006.47	   187.62	  4
 20	 G 	  1034.98	   131.08	  3
 21	 G 	  1063.49	   102.57	  2
 22	 K 	        -  	    74.06	  1  _
```

**115. IPI00296777 SPARC-like protein 1**

| Sequence | Charge | M+H+ | XCorr | DeltaCn |
| --- | --- | --- | --- | --- |
| K.SIPTCTDFEVIQFPLR.M | 2 | 1924.18 | 3.169 | 0.385 |

+1 charge

```
		      B   	       Y
  1	 S 	    88.04	        -  	 16
  2	 I 	   201.12	  1836.07	 15
  3	 P 	   298.18	  1722.99	 14
  4	 T 	   399.22	  1625.93	 13
  5	 C 	   559.39	  1524.89	 12
  6	 T 	   660.44	  1364.72	 11
  7	 D 	   775.46	  1263.67	 10
  8	 F 	   922.53	  1148.65	  9
  9	 E 	  1051.57	  1001.58	  8
 10	 V 	  1150.64	   872.54	  7
 11	 I 	  1263.73	   773.47	  6
 12	 Q 	  1391.79	   660.38	  5
 13	 F 	  1538.85	   532.32	  4
 14	 P 	  1635.91	   385.26	  3
 15	 L 	  1748.99	   288.20	  2
 16	 R 	        -  	   175.12	  1  _
+2 charge
		      B   	       Y
  1	 S 	    44.52	        -  	 16
  2	 I 	   101.07	   918.54	 15
  3	 P 	   149.59	   862.00	 14
  4	 T 	   200.12	   813.47	 13
  5	 C 	   280.20	   762.95	 12
  6	 T 	   330.72	   682.86	 11
  7	 D 	   388.24	   632.34	 10
  8	 F 	   461.77	   574.83	  9
  9	 E 	   526.29	   501.29	  8
 10	 V 	   575.83	   436.77	  7
 11	 I 	   632.37	   387.24	  6
 12	 Q 	   696.40	   330.70	  5
 13	 F 	   769.93	   266.67	  4
 14	 P 	   818.46	   193.13	  3
 15	 L 	   875.00	   144.61	  2
 16	 R 	        -  	    88.06	  1  _
```

**116. IPI00306543 Growth/differentiation factor 15**

| Sequence | Charge | M+H+ | XCorr | DeltaCn |
| --- | --- | --- | --- | --- |
| R.ASLEDLGWADWVLSPR.E | 2 | 1816.006 | 4.12 | 0.549 |

+1 charge

```
		      B   	       Y
  1	 A 	    72.04	        -  	 16
  2	 S 	   159.08	  1743.87	 15
  3	 L 	   272.16	  1656.84	 14
  4	 E 	   401.20	  1543.75	 13
  5	 D 	   516.23	  1414.71	 12
  6	 L 	   629.31	  1299.68	 11
  7	 G 	   686.34	  1186.60	 10
  8	 W 	   872.41	  1129.58	  9
  9	 A 	   943.45	   943.50	  8
 10	 D 	  1058.48	   872.46	  7
 11	 W 	  1244.56	   757.44	  6
 12	 V 	  1343.63	   571.36	  5
 13	 L 	  1456.71	   472.29	  4
 14	 S 	  1543.74	   359.20	  3
 15	 P 	  1640.80	   272.17	  2
 16	 R 	        -  	   175.12	  1  _
+2 charge
		      B   	       Y
  1	 A 	    36.53	        -  	 16
  2	 S 	    80.04	   872.44	 15
  3	 L 	   136.58	   828.92	 14
  4	 E 	   201.11	   772.38	 13
  5	 D 	   258.62	   707.86	 12
  6	 L 	   315.16	   650.35	 11
  7	 G 	   343.67	   593.80	 10
  8	 W 	   436.71	   565.29	  9
  9	 A 	   472.23	   472.25	  8
 10	 D 	   529.74	   436.73	  7
 11	 W 	   622.78	   379.22	  6
 12	 V 	   672.32	   286.18	  5
 13	 L 	   728.86	   236.65	  4
 14	 S 	   772.38	   180.11	  3
 15	 P 	   820.90	   136.59	  2
 16	 R 	        -  	    88.06	  1  _
```

**117. IPI00030786 Isoform 1 of Matrilin-4**

| Sequence | Charge | M+H+ | XCorr | DeltaCn |
| --- | --- | --- | --- | --- |
| R.TLGALESLTLNLAQLTAR.L | 2 | 1886.183 | 4.902 | 0.563 |

+1 charge

```
		      B   	       Y
  1	 T 	   102.05	        -  	 18
  2	 L 	   215.14	  1784.03	 17
  3	 G 	   272.16	  1670.94	 16
  4	 A 	   343.20	  1613.92	 15
  5	 L 	   456.28	  1542.88	 14
  6	 E 	   585.32	  1429.80	 13
  7	 S 	   672.36	  1300.76	 12
  8	 L 	   785.44	  1213.73	 11
  9	 T 	   886.49	  1100.64	 10
 10	 L 	   999.57	   999.59	  9
 11	 N 	  1113.62	   886.51	  8
 12	 L 	  1226.70	   772.47	  7
 13	 A 	  1297.74	   659.38	  6
 14	 Q 	  1425.79	   588.35	  5
 15	 L 	  1538.88	   460.29	  4
 16	 T 	  1639.93	   347.20	  3
 17	 A 	  1710.96	   246.16	  2
 18	 R 	        -  	   175.12	  1  _
+2 charge
		      B   	       Y
  1	 T 	    51.53	        -  	 18
  2	 L 	   108.07	   892.52	 17
  3	 G 	   136.58	   835.98	 16
  4	 A 	   172.10	   807.46	 15
  5	 L 	   228.64	   771.95	 14
  6	 E 	   293.17	   715.40	 13
  7	 S 	   336.68	   650.88	 12
  8	 L 	   393.22	   607.37	 11
  9	 T 	   443.75	   550.82	 10
 10	 L 	   500.29	   500.30	  9
 11	 N 	   557.31	   443.76	  8
 12	 L 	   613.85	   386.74	  7
 13	 A 	   649.37	   330.20	  6
 14	 Q 	   713.40	   294.68	  5
 15	 L 	   769.94	   230.65	  4
 16	 T 	   820.47	   174.11	  3
 17	 A 	   855.99	   123.58	  2
 18	 R 	        -  	    88.06	  1  _
```

**118. IPI0005630 Liver-expressed antimicrobial peptide 2**

| Sequence | Charge | M+H+ | XCorr | DeltaCn |
| --- | --- | --- | --- | --- |
| R.GVSLRPIGASCRDDSECITR.L | 3 | 2250.443 | 4.116 | 0.359 |

+1 charge

```
		      B   	       Y
  1	 G 	    58.03	        -  	 20
  2	 V 	   157.10	  2192.32	 19
  3	 S 	   244.13	  2093.26	 18
  4	 L 	   357.21	  2006.22	 17
  5	 R 	   513.31	  1893.14	 16
  6	 P 	   610.37	  1737.04	 15
  7	 I 	   723.45	  1639.99	 14
  8	 G 	   780.47	  1526.90	 13
  9	 A 	   851.51	  1469.88	 12
 10	 S 	   938.54	  1398.84	 11
 11	 C 	  1098.71	  1311.81	 10
 12	 R 	  1254.81	  1151.65	  9
 13	 D 	  1369.84	   995.54	  8
 14	 D 	  1484.86	   880.52	  7
 15	 S 	  1571.89	   765.49	  6
 16	 E 	  1700.94	   678.46	  5
 17	 C 	  1861.10	   549.42	  4
 18	 I 	  1974.19	   389.25	  3
 19	 T 	  2075.23	   276.17	  2
 20	 R 	        -  	   175.12	  1  _
+2 charge
		      B   	       Y
  1	 G 	    29.52	        -  	 20
  2	 V 	    79.05	  1096.67	 19
  3	 S 	   122.57	  1047.13	 18
  4	 L 	   179.11	  1003.62	 17
  5	 R 	   257.16	   947.07	 16
  6	 P 	   305.69	   869.02	 15
  7	 I 	   362.23	   820.50	 14
  8	 G 	   390.74	   763.95	 13
  9	 A 	   426.26	   735.44	 12
 10	 S 	   469.77	   699.93	 11
 11	 C 	   549.86	   656.41	 10
 12	 R 	   627.91	   576.33	  9
 13	 D 	   685.42	   498.28	  8
 14	 D 	   742.93	   440.76	  7
 15	 S 	   786.45	   383.25	  6
 16	 E 	   850.97	   339.73	  5
 17	 C 	   931.05	   275.21	  4
 18	 I 	   987.60	   195.13	  3
 19	 T 	  1038.12	   138.59	  2
 20	 R 	        -  	    88.06	  1  _


+3 charge
		      B   	       Y
  1	 G 	    20.01	        -  	 20
  2	 V 	    53.04	   731.45	 19
  3	 S 	    82.05	   698.42	 18
  4	 L 	   119.74	   669.41	 17
  5	 R 	   171.78	   631.72	 16
  6	 P 	   204.13	   579.68	 15
  7	 I 	   241.82	   547.33	 14
  8	 G 	   260.83	   509.64	 13
  9	 A 	   284.51	   490.63	 12
 10	 S 	   313.52	   466.95	 11
 11	 C 	   366.91	   437.94	 10
 12	 R 	   418.94	   384.55	  9
 13	 D 	   457.28	   332.52	  8
 14	 D 	   495.63	   294.18	  7
 15	 S 	   524.64	   255.84	  6
 16	 E 	   567.65	   226.82	  5
 17	 C 	   621.04	   183.81	  4
 18	 I 	   658.73	   130.42	  3
 19	 T 	   692.42	    92.73	  2
 20	 R 	        -  	    59.04	  1  _
```

**119. IPI00289931 MADCAM1 protein**

| Sequence | Charge | M+H+ | XCorr | DeltaCn |
| --- | --- | --- | --- | --- |
| R.GLDTSLGAVQSDTGR.S | 2 | 1477.559 | 4.635 | 0.607 |

+1 charge

```
		      B   	       Y
  1	 G 	    58.03	        -  	 15
  2	 L 	   171.11	  1419.71	 14
  3	 D 	   286.14	  1306.62	 13
  4	 T 	   387.19	  1191.60	 12
  5	 S 	   474.22	  1090.55	 11
  6	 L 	   587.30	  1003.52	 10
  7	 G 	   644.32	   890.43	  9
  8	 A 	   715.36	   833.41	  8
  9	 V 	   814.43	   762.37	  7
 10	 Q 	   942.49	   663.31	  6
 11	 S 	  1029.52	   535.25	  5
 12	 D 	  1144.55	   448.22	  4
 13	 T 	  1245.60	   333.19	  3
 14	 G 	  1302.62	   232.14	  2
 15	 R 	        -  	   175.12	  1  _
+2 charge
		      B   	       Y
  1	 G 	    29.52	        -  	 15
  2	 L 	    86.06	   710.36	 14
  3	 D 	   143.57	   653.82	 13
  4	 T 	   194.10	   596.30	 12
  5	 S 	   237.61	   545.78	 11
  6	 L 	   294.16	   502.26	 10
  7	 G 	   322.67	   445.72	  9
  8	 A 	   358.18	   417.21	  8
  9	 V 	   407.72	   381.69	  7
 10	 Q 	   471.75	   332.16	  6
 11	 S 	   515.26	   268.13	  5
 12	 D 	   572.78	   224.61	  4
 13	 T 	   623.30	   167.10	  3
 14	 G 	   651.81	   116.57	  2
 15	 R 	        -  	    88.06	  1  _
```

**120. IPI00854644 similar to hCG1686089**

| Sequence | Charge | M+H+ | XCorr | DeltaCn |
| --- | --- | --- | --- | --- |
| R.LLIYDASSR.A | 2 | 1038.179 | 3.123 | 0.336 |

+1 charge

```
		      B   	       Y
  1	 L 	   114.09	        -  	  9
  2	 L 	   227.18	   924.48	  8
  3	 I 	   340.26	   811.39	  7
  4	 Y 	   503.32	   698.31	  6
  5	 D 	   618.35	   535.25	  5
  6	 A 	   689.39	   420.22	  4
  7	 S 	   776.42	   349.18	  3
  8	 S 	   863.45	   262.15	  2
  9	 R 	        -  	   175.12	  1  _
+2 charge
		      B   	       Y
  1	 L 	    57.55	        -  	  9
  2	 L 	   114.09	   462.74	  8
  3	 I 	   170.63	   406.20	  7
  4	 Y 	   252.17	   349.66	  6
  5	 D 	   309.68	   268.13	  5
  6	 A 	   345.20	   210.61	  4
  7	 S 	   388.71	   175.10	  3
  8	 S 	   432.23	   131.58	  2
  9	 R 	        -  	    88.06	  1  _
```

**121. IPI00176222 Isoform 1 of Zinc finger protein 549**

| Sequence | Charge | M+H+ | XCorr | DeltaCn |
| --- | --- | --- | --- | --- |
| K.WFSFGSNLQQHQNQDSGEKHIRK.E | 3 | 2772.975 | 3.317 | 0.207 |

+1 charge

```
		      B   	       Y
  1	 W 	   187.09	        -  	 23
  2	 F 	   334.16	  2585.26	 22
  3	 S 	   421.19	  2438.19	 21
  4	 F 	   568.26	  2351.16	 20
  5	 G 	   625.28	  2204.09	 19
  6	 S 	   712.31	  2147.07	 18
  7	 N 	   826.35	  2060.04	 17
  8	 L 	   939.44	  1946.00	 16
  9	 Q 	  1067.49	  1832.91	 15
 10	 Q 	  1195.55	  1704.85	 14
 11	 H 	  1332.61	  1576.79	 13
 12	 Q 	  1460.67	  1439.73	 12
 13	 N 	  1574.71	  1311.68	 11
 14	 Q 	  1702.77	  1197.63	 10
 15	 D 	  1817.80	  1069.57	  9
 16	 S 	  1904.83	   954.55	  8
 17	 G 	  1961.85	   867.52	  7
 18	 E 	  2090.90	   810.49	  6
 19	 K 	  2218.99	   681.45	  5
 20	 H 	  2356.05	   553.36	  4
 21	 I 	  2469.13	   416.30	  3
 22	 R 	  2625.23	   303.21	  2
 23	 K 	        -  	   147.11	  1  _


+2 charge
		      B   	       Y
  1	 W 	    94.05	        -  	 23
  2	 F 	   167.58	  1293.13	 22
  3	 S 	   211.10	  1219.60	 21
  4	 F 	   284.63	  1176.08	 20
  5	 G 	   313.14	  1102.55	 19
  6	 S 	   356.66	  1074.04	 18
  7	 N 	   413.68	  1030.52	 17
  8	 L 	   470.22	   973.50	 16
  9	 Q 	   534.25	   916.96	 15
 10	 Q 	   598.28	   852.93	 14
 11	 H 	   666.81	   788.90	 13
 12	 Q 	   730.84	   720.37	 12
 13	 N 	   787.86	   656.34	 11
 14	 Q 	   851.89	   599.32	 10
 15	 D 	   909.40	   535.29	  9
 16	 S 	   952.92	   477.78	  8
 17	 G 	   981.43	   434.26	  7
 18	 E 	  1045.95	   405.75	  6
 19	 K 	  1110.00	   341.23	  5
 20	 H 	  1178.53	   277.18	  4
 21	 I 	  1235.07	   208.65	  3
 22	 R 	  1313.12	   152.11	  2
 23	 K 	        -  	    74.06	  1  _
+3 charge
		      B   	       Y
  1	 W 	    63.03	        -  	 23
  2	 F 	   112.06	   862.42	 22
  3	 S 	   141.07	   813.40	 21
  4	 F 	   190.09	   784.39	 20
  5	 G 	   209.10	   735.37	 19
  6	 S 	   238.11	   716.36	 18
  7	 N 	   276.12	   687.35	 17
  8	 L 	   313.82	   649.34	 16
  9	 Q 	   356.50	   611.64	 15
 10	 Q 	   399.19	   568.96	 14
 11	 H 	   444.88	   526.27	 13
 12	 Q 	   487.56	   480.58	 12
 13	 N 	   525.58	   437.90	 11
 14	 Q 	   568.26	   399.88	 10
 15	 D 	   606.60	   357.20	  9
 16	 S 	   635.62	   318.85	  8
 17	 G 	   654.62	   289.84	  7
 18	 E 	   697.64	   270.84	  6
 19	 K 	   740.33	   227.82	  5
 20	 H 	   786.02	   185.12	  4
 21	 I 	   823.72	   139.44	  3
 22	 R 	   875.75	   101.74	  2
 23	 K 	        -  	    49.71	  1  _
```

**122. IPI00025846 Isoform 2A of Desmocollin-2**

| Sequence | Charge | M+H+ | XCorr | DeltaCn |
| --- | --- | --- | --- | --- |
| R.LSYQNDPPFGSYVVPITVR.D | 2 | 2153.422 | 4.79 | 0.502 |

+1 charge

```
		      B   	       Y
  1	 L 	   114.09	        -  	 19
  2	 S 	   201.12	  2039.02	 18
  3	 Y 	   364.19	  1951.99	 17
  4	 Q 	   492.25	  1788.93	 16
  5	 N 	   606.29	  1660.87	 15
  6	 D 	   721.32	  1546.83	 14
  7	 P 	   818.37	  1431.80	 13
  8	 P 	   915.42	  1334.75	 12
  9	 F 	  1062.49	  1237.69	 11
 10	 G 	  1119.51	  1090.63	 10
 11	 S 	  1206.54	  1033.60	  9
 12	 Y 	  1369.61	   946.57	  8
 13	 V 	  1468.67	   783.51	  7
 14	 V 	  1567.74	   684.44	  6
 15	 P 	  1664.80	   585.37	  5
 16	 I 	  1777.88	   488.32	  4
 17	 T 	  1878.93	   375.24	  3
 18	 V 	  1978.00	   274.19	  2
 19	 R 	        -  	   175.12	  1  _
+2 charge
		      B   	       Y
  1	 L 	    57.55	        -  	 19
  2	 S 	   101.07	  1020.02	 18
  3	 Y 	   182.60	   976.50	 17
  4	 Q 	   246.63	   894.97	 16
  5	 N 	   303.65	   830.94	 15
  6	 D 	   361.16	   773.92	 14
  7	 P 	   409.69	   716.40	 13
  8	 P 	   458.21	   667.88	 12
  9	 F 	   531.75	   619.35	 11
 10	 G 	   560.26	   545.82	 10
 11	 S 	   603.77	   517.31	  9
 12	 Y 	   685.31	   473.79	  8
 13	 V 	   734.84	   392.26	  7
 14	 V 	   784.38	   342.72	  6
 15	 P 	   832.90	   293.19	  5
 16	 I 	   889.44	   244.66	  4
 17	 T 	   939.97	   188.12	  3
 18	 V 	   989.50	   137.60	  2
 19	 R 	        -  	    88.06	  1  _
```

**123. IPI00940158 trefoil factor 3 precursor**

| Sequence | Charge | M+H+ | XCorr | DeltaCn |
| --- | --- | --- | --- | --- |
| R.IPGVPWCFKPLQEAECTF.- | 2 | 2180.474 | 3.454 | 0.588 |

+1 charge

```
		      B   	       Y
  1	 I 	   114.09	        -  	 18
  2	 P 	   211.14	  2066.22	 17
  3	 G 	   268.17	  1969.17	 16
  4	 V 	   367.23	  1912.15	 15
  5	 P 	   464.29	  1813.08	 14
  6	 W 	   650.37	  1716.02	 13
  7	 C 	   810.53	  1529.95	 12
  8	 F 	   957.60	  1369.78	 11
  9	 K 	  1085.69	  1222.71	 10
 10	 P 	  1182.75	  1094.62	  9
 11	 L 	  1295.83	   997.56	  8
 12	 Q 	  1423.89	   884.48	  7
 13	 E 	  1552.93	   756.42	  6
 14	 A 	  1623.97	   627.38	  5
 15	 E 	  1753.01	   556.34	  4
 16	 C 	  1913.18	   427.30	  3
 17	 T 	  2014.23	   267.13	  2
 18	 F 	        -  	   166.09	  1  _
+2 charge
		      B   	       Y
  1	 I 	    57.55	        -  	 18
  2	 P 	   106.08	  1033.61	 17
  3	 G 	   134.59	   985.09	 16
  4	 V 	   184.12	   956.58	 15
  5	 P 	   232.65	   907.04	 14
  6	 W 	   325.69	   858.52	 13
  7	 C 	   405.77	   765.48	 12
  8	 F 	   479.30	   685.39	 11
  9	 K 	   543.35	   611.86	 10
 10	 P 	   591.88	   547.81	  9
 11	 L 	   648.42	   499.29	  8
 12	 Q 	   712.45	   442.74	  7
 13	 E 	   776.97	   378.71	  6
 14	 A 	   812.49	   314.19	  5
 15	 E 	   877.01	   278.67	  4
 16	 C 	   957.09	   214.15	  3
 17	 T 	  1007.62	   134.07	  2
 18	 F 	        -  	    83.55	  1  _
```

**124. IPI00855785 Isoform 15 of Fibronectin**

| Sequence | Charge | M+H+ | XCorr | DeltaCn |
| --- | --- | --- | --- | --- |
| R.VTWAPPPSIDLTNFLVR.Y | 2 | 1927.235 | 2.895 | 0.39 |

+1 charge

```
		      B   	       Y
  1	 V 	   100.08	        -  	 17
  2	 T 	   201.12	  1826.98	 16
  3	 W 	   387.20	  1725.93	 15
  4	 A 	   458.24	  1539.85	 14
  5	 P 	   555.29	  1468.82	 13
  6	 P 	   652.35	  1371.76	 12
  7	 P 	   749.40	  1274.71	 11
  8	 S 	   836.43	  1177.66	 10
  9	 I 	   949.51	  1090.63	  9
 10	 D 	  1064.54	   977.54	  8
 11	 L 	  1177.63	   862.51	  7
 12	 T 	  1278.67	   749.43	  6
 13	 N 	  1392.72	   648.38	  5
 14	 F 	  1539.78	   534.34	  4
 15	 L 	  1652.87	   387.27	  3
 16	 V 	  1751.94	   274.19	  2
 17	 R 	        -  	   175.12	  1  _
+2 charge
		      B   	       Y
  1	 V 	    50.54	        -  	 17
  2	 T 	   101.07	   913.99	 16
  3	 W 	   194.10	   863.47	 15
  4	 A 	   229.62	   770.43	 14
  5	 P 	   278.15	   734.91	 13
  6	 P 	   326.68	   686.39	 12
  7	 P 	   375.20	   637.86	 11
  8	 S 	   418.72	   589.33	 10
  9	 I 	   475.26	   545.82	  9
 10	 D 	   532.77	   489.27	  8
 11	 L 	   589.32	   431.76	  7
 12	 T 	   639.84	   375.22	  6
 13	 N 	   696.86	   324.70	  5
 14	 F 	   770.40	   267.67	  4
 15	 L 	   826.94	   194.14	  3
 16	 V 	   876.47	   137.60	  2
 17	 R 	        -  	    88.06	  1  _
```

**125. IPI00021885 Isoform 1 of Fibrinogen alpha chain**

| Sequence | Charge | M+H+ | XCorr | DeltaCn |
| --- | --- | --- | --- | --- |
| R.GFGSLNDEGEGEFWLGNDYLHLLTQR.G | 3 | 2969.17 | 5.448 | 0.518 |

+1 charge

```
		      B   	       Y
  1	 G 	    58.03	        -  	 26
  2	 F 	   205.10	  2910.37	 25
  3	 G 	   262.12	  2763.30	 24
  4	 S 	   349.15	  2706.28	 23
  5	 L 	   462.23	  2619.25	 22
  6	 N 	   576.28	  2506.16	 21
  7	 D 	   691.30	  2392.12	 20
  8	 E 	   820.35	  2277.09	 19
  9	 G 	   877.37	  2148.05	 18
 10	 E 	  1006.41	  2091.03	 17
 11	 G 	  1063.43	  1961.99	 16
 12	 E 	  1192.48	  1904.97	 15
 13	 F 	  1339.54	  1775.92	 14
 14	 W 	  1525.62	  1628.85	 13
 15	 L 	  1638.71	  1442.78	 12
 16	 G 	  1695.73	  1329.69	 11
 17	 N 	  1809.77	  1272.67	 10
 18	 D 	  1924.80	  1158.63	  9
 19	 Y 	  2087.86	  1043.60	  8
 20	 L 	  2200.95	   880.54	  7
 21	 H 	  2338.00	   767.45	  6
 22	 L 	  2451.09	   630.39	  5
 23	 L 	  2564.17	   517.31	  4
 24	 T 	  2665.22	   404.23	  3
 25	 Q 	  2793.28	   303.18	  2
 26	 R 	        -  	   175.12	  1  _


+2 charge
		      B   	       Y
  1	 G 	    29.52	        -  	 26
  2	 F 	   103.05	  1455.69	 25
  3	 G 	   131.56	  1382.15	 24
  4	 S 	   175.08	  1353.64	 23
  5	 L 	   231.62	  1310.13	 22
  6	 N 	   288.64	  1253.59	 21
  7	 D 	   346.16	  1196.56	 20
  8	 E 	   410.68	  1139.05	 19
  9	 G 	   439.19	  1074.53	 18
 10	 E 	   503.71	  1046.02	 17
 11	 G 	   532.22	   981.50	 16
 12	 E 	   596.74	   952.99	 15
 13	 F 	   670.28	   888.47	 14
 14	 W 	   763.32	   814.93	 13
 15	 L 	   819.86	   721.89	 12
 16	 G 	   848.37	   665.35	 11
 17	 N 	   905.39	   636.84	 10
 18	 D 	   962.90	   579.82	  9
 19	 Y 	  1044.43	   522.30	  8
 20	 L 	  1100.98	   440.77	  7
 21	 H 	  1169.51	   384.23	  6
 22	 L 	  1226.05	   315.70	  5
 23	 L 	  1282.59	   259.16	  4
 24	 T 	  1333.11	   202.62	  3
 25	 Q 	  1397.14	   152.09	  2
 26	 R 	        -  	    88.06	  1  _


+3 charge
		      B   	       Y
  1	 G 	    20.01	        -  	 26
  2	 F 	    69.04	   970.79	 25
  3	 G 	    88.04	   921.77	 24
  4	 S 	   117.06	   902.76	 23
  5	 L 	   154.75	   873.75	 22
  6	 N 	   192.76	   836.06	 21
  7	 D 	   231.11	   798.04	 20
  8	 E 	   274.12	   759.70	 19
  9	 G 	   293.13	   716.69	 18
 10	 E 	   336.14	   697.68	 17
 11	 G 	   355.15	   654.67	 16
 12	 E 	   398.16	   635.66	 15
 13	 F 	   447.19	   592.65	 14
 14	 W 	   509.21	   543.62	 13
 15	 L 	   546.91	   481.60	 12
 16	 G 	   565.91	   443.90	 11
 17	 N 	   603.93	   424.89	 10
 18	 D 	   642.27	   386.88	  9
 19	 Y 	   696.63	   348.54	  8
 20	 L 	   734.32	   294.18	  7
 21	 H 	   780.01	   256.49	  6
 22	 L 	   817.70	   210.80	  5
 23	 L 	   855.40	   173.11	  4
 24	 T 	   889.08	   135.41	  3
 25	 Q 	   931.76	   101.73	  2
 26	 R 	        -  	    59.04	  1  _
```

**Female 5**

**1. IPI00183487 Xylosyltransferase 1**

| Sequence | Charge | M+H+ | XCorr | DeltaCn |
| --- | --- | --- | --- | --- |
| R.GGAAVGGGEQPPPAPAPR.R | 2 | 1586.734 | 4.651 | 0.637 |

+1 charge

```
		      B   	       Y
  1	 G 	    58.03	        -  	 18
  2	 G 	   115.05	  1528.79	 17
  3	 A 	   186.09	  1471.77	 16
  4	 A 	   257.12	  1400.73	 15
  5	 V 	   356.19	  1329.69	 14
  6	 G 	   413.21	  1230.62	 13
  7	 G 	   470.24	  1173.60	 12
  8	 G 	   527.26	  1116.58	 11
  9	 E 	   656.30	  1059.56	 10
 10	 Q 	   784.36	   930.52	  9
 11	 P 	   881.41	   802.46	  8
 12	 P 	   978.46	   705.40	  7
 13	 P 	  1075.52	   608.35	  6
 14	 A 	  1146.55	   511.30	  5
 15	 P 	  1243.61	   440.26	  4
 16	 A 	  1314.64	   343.21	  3
 17	 P 	  1411.70	   272.17	  2
 18	 R 	        -  	   175.12	  1  _
+2 charge
		      B   	       Y
  1	 G 	    29.52	        -  	 18
  2	 G 	    58.03	   764.90	 17
  3	 A 	    93.55	   736.39	 16
  4	 A 	   129.07	   700.87	 15
  5	 V 	   178.60	   665.35	 14
  6	 G 	   207.11	   615.81	 13
  7	 G 	   235.62	   587.30	 12
  8	 G 	   264.13	   558.79	 11
  9	 E 	   328.65	   530.28	 10
 10	 Q 	   392.68	   465.76	  9
 11	 P 	   441.21	   401.73	  8
 12	 P 	   489.74	   353.21	  7
 13	 P 	   538.26	   304.68	  6
 14	 A 	   573.78	   256.15	  5
 15	 P 	   622.31	   220.63	  4
 16	 A 	   657.83	   172.11	  3
 17	 P 	   706.35	   136.59	  2
 18	 R 	        -  	    88.06	  1  _
```

**2. IPI00005721 Neutrophil defensin 1**

| Sequence | Charge | M+H+ | XCorr | DeltaCn |
| --- | --- | --- | --- | --- |
| R.YGTCIYQGR.L | 2 | 1118.218 | 3.127 | 0.559 |

+1 charge

```
		      B   	       Y
  1	 Y 	   164.07	        -  	  9
  2	 G 	   221.09	   954.58	  8
  3	 T 	   322.14	   897.56	  7
  4	 C 	   482.30	   796.51	  6
  5	 I 	   595.39	   636.35	  5
  6	 Y 	   758.45	   523.26	  4
  7	 Q 	   886.51	   360.20	  3
  8	 G 	   943.53	   232.14	  2
  9	 R 	        -  	   175.12	  1  _
+2 charge
		      B   	       Y
  1	 Y 	    82.54	        -  	  9
  2	 G 	   111.05	   477.79	  8
  3	 T 	   161.57	   449.28	  7
  4	 C 	   241.66	   398.76	  6
  5	 I 	   298.20	   318.68	  5
  6	 Y 	   379.73	   262.13	  4
  7	 Q 	   443.76	   180.60	  3
  8	 G 	   472.27	   116.57	  2
  9	 R 	        -  	    88.06	  1  _
```

**3. IPI00153049 Isoform 2 of Matrix-remodeling-associated protein 8**

| Sequence | Charge | M+H+ | XCorr | DeltaCn |
| --- | --- | --- | --- | --- |
| R.GAPALLTCVNR.G | 2 | 1172.353 | 2.816 | 0.497 |

+1 charge

```
		      B   	       Y
  1	 G 	    58.03	        -  	 11
  2	 A 	   129.07	  1114.74	 10
  3	 P 	   226.12	  1043.70	  9
  4	 A 	   297.16	   946.65	  8
  5	 L 	   410.24	   875.61	  7
  6	 L 	   523.32	   762.53	  6
  7	 T 	   624.37	   649.44	  5
  8	 C 	   784.54	   548.40	  4
  9	 V 	   883.61	   388.23	  3
 10	 N 	   997.65	   289.16	  2
 11	 R 	        -  	   175.12	  1  _
+2 charge
		      B   	       Y
  1	 G 	    29.52	        -  	 11
  2	 A 	    65.04	   557.87	 10
  3	 P 	   113.56	   522.35	  9
  4	 A 	   149.08	   473.83	  8
  5	 L 	   205.62	   438.31	  7
  6	 L 	   262.17	   381.77	  6
  7	 T 	   312.69	   325.23	  5
  8	 C 	   392.77	   274.70	  4
  9	 V 	   442.31	   194.62	  3
 10	 N 	   499.33	   145.08	  2
 11	 R 	        -  	    88.06	  1  _
```

**4. IPI00453473 Histone H4**

| Sequence | Charge | M+H+ | XCorr | DeltaCn |
| --- | --- | --- | --- | --- |
| R.ISGLIYEETR.G | 2 | 1181.32 | 3.469 | 0.482 |

+1 charge

```
		      B   	       Y
  1	 I 	   114.09	        -  	 10
  2	 S 	   201.12	  1067.54	  9
  3	 G 	   258.14	   980.50	  8
  4	 L 	   371.23	   923.48	  7
  5	 I 	   484.31	   810.40	  6
  6	 Y 	   647.38	   697.32	  5
  7	 E 	   776.42	   534.25	  4
  8	 E 	   905.46	   405.21	  3
  9	 T 	  1006.51	   276.17	  2
 10	 R 	        -  	   175.12	  1  _
+2 charge
		      B   	       Y
  1	 I 	    57.55	        -  	 10
  2	 S 	   101.07	   534.27	  9
  3	 G 	   129.58	   490.76	  8
  4	 L 	   186.12	   462.25	  7
  5	 I 	   242.66	   405.70	  6
  6	 Y 	   324.19	   349.16	  5
  7	 E 	   388.71	   267.63	  4
  8	 E 	   453.23	   203.11	  3
  9	 T 	   503.76	   138.59	  2
 10	 R 	        -  	    88.06	  1  _
```

**5. IPI00219827 Secreted Ly-6/uPAR-related protein 2**

| Sequence | Charge | M+H+ | XCorr | DeltaCn |
| --- | --- | --- | --- | --- |
| R.VLSNTEDLPLVTK.M | 2 | 1429.641 | 4.641 | 0.536 |

+1 charge

```
		      B   	       Y
  1	 V 	   100.08	        -  	 13
  2	 L 	   213.16	  1329.73	 12
  3	 S 	   300.19	  1216.64	 11
  4	 N 	   414.23	  1129.61	 10
  5	 T 	   515.28	  1015.57	  9
  6	 E 	   644.32	   914.52	  8
  7	 D 	   759.35	   785.48	  7
  8	 L 	   872.44	   670.45	  6
  9	 P 	   969.49	   557.37	  5
 10	 L 	  1082.57	   460.31	  4
 11	 V 	  1181.64	   347.23	  3
 12	 T 	  1282.69	   248.16	  2
 13	 K 	        -  	   147.11	  1  _
+2 charge
		      B   	       Y
  1	 V 	    50.54	        -  	 13
  2	 L 	   107.08	   665.37	 12
  3	 S 	   150.60	   608.82	 11
  4	 N 	   207.62	   565.31	 10
  5	 T 	   258.14	   508.29	  9
  6	 E 	   322.67	   457.76	  8
  7	 D 	   380.18	   393.24	  7
  8	 L 	   436.72	   335.73	  6
  9	 P 	   485.25	   279.19	  5
 10	 L 	   541.79	   230.66	  4
 11	 V 	   591.32	   174.12	  3
 12	 T 	   641.85	   124.58	  2
 13	 K 	        -  	    74.06	  1  _
```

**6. IPI00553215 V1-5 protein**

| Sequence | Charge | M+H+ | XCorr | DeltaCn |
| --- | --- | --- | --- | --- |
| K.LMIYEVSNRPSGVPDRFSGSK.S | 3 | 2340.644 | 3.741 | 0.36 |

+1 charge

```
		      B   	       Y
  1	 L 	   114.09	        -  	 21
  2	 M 	   245.13	  2226.10	 20
  3	 I 	   358.22	  2095.06	 19
  4	 Y 	   521.28	  1981.97	 18
  5	 E 	   650.32	  1818.91	 17
  6	 V 	   749.39	  1689.87	 16
  7	 S 	   836.42	  1590.80	 15
  8	 N 	   950.47	  1503.77	 14
  9	 R 	  1106.57	  1389.72	 13
 10	 P 	  1203.62	  1233.62	 12
 11	 S 	  1290.65	  1136.57	 11
 12	 G 	  1347.67	  1049.54	 10
 13	 V 	  1446.74	   992.52	  9
 14	 P 	  1543.79	   893.45	  8
 15	 D 	  1658.82	   796.39	  7
 16	 R 	  1814.92	   681.37	  6
 17	 F 	  1961.99	   525.27	  5
 18	 S 	  2049.02	   378.20	  4
 19	 G 	  2106.04	   291.17	  3
 20	 S 	  2193.08	   234.14	  2
 21	 K 	        -  	   147.11	  1  _
+2 charge
		      B   	       Y
  1	 L 	    57.55	        -  	 21
  2	 M 	   123.07	  1113.55	 20
  3	 I 	   179.61	  1048.03	 19
  4	 Y 	   261.14	   991.49	 18
  5	 E 	   325.66	   909.96	 17
  6	 V 	   375.20	   845.44	 16
  7	 S 	   418.71	   795.90	 15
  8	 N 	   475.74	   752.39	 14
  9	 R 	   553.79	   695.37	 13
 10	 P 	   602.31	   617.31	 12
 11	 S 	   645.83	   568.79	 11
 12	 G 	   674.34	   525.27	 10
 13	 V 	   723.87	   496.76	  9
 14	 P 	   772.40	   447.23	  8
 15	 D 	   829.91	   398.70	  7
 16	 R 	   907.96	   341.19	  6
 17	 F 	   981.50	   263.14	  5
 18	 S 	  1025.01	   189.60	  4
 19	 G 	  1053.53	   146.09	  3
 20	 S 	  1097.04	   117.58	  2
 21	 K 	        -  	    74.06	  1  _
+3 charge
		      B   	       Y
  1	 L 	    38.70	        -  	 21
  2	 M 	    82.38	   742.70	 20
  3	 I 	   120.08	   699.02	 19
  4	 Y 	   174.43	   661.33	 18
  5	 E 	   217.45	   606.97	 17
  6	 V 	   250.47	   563.96	 16
  7	 S 	   279.48	   530.94	 15
  8	 N 	   317.49	   501.93	 14
  9	 R 	   369.53	   463.91	 13
 10	 P 	   401.88	   411.88	 12
 11	 S 	   430.89	   379.53	 11
 12	 G 	   449.90	   350.52	 10
 13	 V 	   482.92	   331.51	  9
 14	 P 	   515.27	   298.49	  8
 15	 D 	   553.61	   266.14	  7
 16	 R 	   605.65	   227.79	  6
 17	 F 	   654.67	   175.76	  5
 18	 S 	   683.68	   126.74	  4
 19	 G 	   702.69	    97.73	  3
 20	 S 	   731.70	    78.72	  2
 21	 K 	        -  	    49.71	  1  _
```

**7. IPI00301143 Isoform 1 of Peptidase inhibitor 16**

| Sequence | Charge | M+H+ | XCorr | DeltaCn |
| --- | --- | --- | --- | --- |
| R.LMVELHNLYR.A | 2 | 1288.544 | 3.128 | 0.244 |

+1 charge

```
		      B   	       Y
  1	 L 	   114.09	        -  	 10
  2	 M 	   245.13	  1174.60	  9
  3	 V 	   344.20	  1043.56	  8
  4	 E 	   473.24	   944.49	  7
  5	 L 	   586.33	   815.45	  6
  6	 H 	   723.39	   702.37	  5
  7	 N 	   837.43	   565.31	  4
  8	 L 	   950.51	   451.27	  3
  9	 Y 	  1113.58	   338.18	  2
 10	 R 	        -  	   175.12	  1  _
+2 charge
		      B   	       Y
  1	 L 	    57.55	        -  	 10
  2	 M 	   123.07	   587.81	  9
  3	 V 	   172.60	   522.29	  8
  4	 E 	   237.13	   472.75	  7
  5	 L 	   293.67	   408.23	  6
  6	 H 	   362.20	   351.69	  5
  7	 N 	   419.22	   283.16	  4
  8	 L 	   475.76	   226.14	  3
  9	 Y 	   557.29	   169.59	  2
 10	 R 	        -  	    88.06	  1  _
```

**8. IPI00022290 Beta-defensin 1**

| Sequence | Charge | M+H+ | XCorr | DeltaCn |
| --- | --- | --- | --- | --- |
| R.SDHYNCVSSGGQCLYSACPIFTK.I | 3 | 2652.835 | 4.703 | 0.675 |

+1 charge

```
		      B   	       Y
  1	 S 	    88.04	        -  	 23
  2	 D 	   203.07	  2564.50	 22
  3	 H 	   340.13	  2449.48	 21
  4	 Y 	   503.19	  2312.42	 20
  5	 N 	   617.23	  2149.35	 19
  6	 C 	   777.40	  2035.31	 18
  7	 V 	   876.47	  1875.15	 17
  8	 S 	   963.50	  1776.08	 16
  9	 S 	  1050.53	  1689.05	 15
 10	 G 	  1107.55	  1602.01	 14
 11	 G 	  1164.57	  1544.99	 13
 12	 Q 	  1292.63	  1487.97	 12
 13	 C 	  1452.80	  1359.91	 11
 14	 L 	  1565.88	  1199.75	 10
 15	 Y 	  1728.94	  1086.66	  9
 16	 S 	  1815.98	   923.60	  8
 17	 A 	  1887.01	   836.57	  7
 18	 C 	  2047.18	   765.53	  6
 19	 P 	  2144.23	   605.37	  5
 20	 I 	  2257.31	   508.31	  4
 21	 F 	  2404.38	   395.23	  3
 22	 T 	  2505.43	   248.16	  2
 23	 K 	        -  	   147.11	  1  _


+2 charge
		      B   	       Y
  1	 S 	    44.52	        -  	 23
  2	 D 	   102.04	  1282.76	 22
  3	 H 	   170.57	  1225.24	 21
  4	 Y 	   252.10	  1156.71	 20
  5	 N 	   309.12	  1075.18	 19
  6	 C 	   389.20	  1018.16	 18
  7	 V 	   438.74	   938.08	 17
  8	 S 	   482.25	   888.54	 16
  9	 S 	   525.77	   845.03	 15
 10	 G 	   554.28	   801.51	 14
 11	 G 	   582.79	   773.00	 13
 12	 Q 	   646.82	   744.49	 12
 13	 C 	   726.90	   680.46	 11
 14	 L 	   783.44	   600.38	 10
 15	 Y 	   864.98	   543.84	  9
 16	 S 	   908.49	   462.30	  8
 17	 A 	   944.01	   418.79	  7
 18	 C 	  1024.09	   383.27	  6
 19	 P 	  1072.62	   303.19	  5
 20	 I 	  1129.16	   254.66	  4
 21	 F 	  1202.70	   198.12	  3
 22	 T 	  1253.22	   124.58	  2
 23	 K 	        -  	    74.06	  1  _
+3 charge
		      B   	       Y
  1	 S 	    30.02	        -  	 23
  2	 D 	    68.36	   855.51	 22
  3	 H 	   114.05	   817.16	 21
  4	 Y 	   168.40	   771.48	 20
  5	 N 	   206.42	   717.12	 19
  6	 C 	   259.80	   679.11	 18
  7	 V 	   292.83	   625.72	 17
  8	 S 	   321.84	   592.70	 16
  9	 S 	   350.85	   563.69	 15
 10	 G 	   369.86	   534.68	 14
 11	 G 	   388.86	   515.67	 13
 12	 Q 	   431.55	   496.66	 12
 13	 C 	   484.94	   453.98	 11
 14	 L 	   522.63	   400.59	 10
 15	 Y 	   576.99	   362.89	  9
 16	 S 	   606.00	   308.54	  8
 17	 A 	   629.68	   279.53	  7
 18	 C 	   683.06	   255.85	  6
 19	 P 	   715.41	   202.46	  5
 20	 I 	   753.11	   170.11	  4
 21	 F 	   802.13	   132.41	  3
 22	 T 	   835.82	    83.39	  2
 23	 K 	        -  	    49.71	  1  _
```

**9. IPI00023648 Immunoglobulin superfamily containing leucine-rich repeat protein**

| Sequence | Charge | M+H+ | XCorr | DeltaCn |
| --- | --- | --- | --- | --- |
| R.TVAAGALASLSHLK.S | 2 | 1339.566 | 3.778 | 0.455 |

+1 charge

```
		      B   	       Y
  1	 T 	   102.05	        -  	 14
  2	 V 	   201.12	  1237.73	 13
  3	 A 	   272.16	  1138.66	 12
  4	 A 	   343.20	  1067.62	 11
  5	 G 	   400.22	   996.58	 10
  6	 A 	   471.26	   939.56	  9
  7	 L 	   584.34	   868.53	  8
  8	 A 	   655.38	   755.44	  7
  9	 S 	   742.41	   684.40	  6
 10	 L 	   855.49	   597.37	  5
 11	 S 	   942.53	   484.29	  4
 12	 H 	  1079.58	   397.26	  3
 13	 L 	  1192.67	   260.20	  2
 14	 K 	        -  	   147.11	  1  _
+2 charge
		      B   	       Y
  1	 T 	    51.53	        -  	 14
  2	 V 	   101.07	   619.37	 13
  3	 A 	   136.58	   569.83	 12
  4	 A 	   172.10	   534.31	 11
  5	 G 	   200.61	   498.80	 10
  6	 A 	   236.13	   470.28	  9
  7	 L 	   292.67	   434.77	  8
  8	 A 	   328.19	   378.22	  7
  9	 S 	   371.71	   342.71	  6
 10	 L 	   428.25	   299.19	  5
 11	 S 	   471.77	   242.65	  4
 12	 H 	   540.30	   199.13	  3
 13	 L 	   596.84	   130.60	  2
 14	 K 	        -  	    74.06	  1  _
```

**10. IPI00003111 Ig kappa chain V-I region AU**

| Sequence | Charge | M+H+ | XCorr | DeltaCn |
| --- | --- | --- | --- | --- |
| K.LLIYDASNLETGVPSR.F | 2 | 1748.958 | 3.247 | 0.204 |

+1 charge

```
		      B   	       Y
  1	 L 	   114.09	        -  	 16
  2	 L 	   227.18	  1634.84	 15
  3	 I 	   340.26	  1521.75	 14
  4	 Y 	   503.32	  1408.67	 13
  5	 D 	   618.35	  1245.61	 12
  6	 A 	   689.39	  1130.58	 11
  7	 S 	   776.42	  1059.54	 10
  8	 N 	   890.46	   972.51	  9
  9	 L 	  1003.55	   858.47	  8
 10	 E 	  1132.59	   745.38	  7
 11	 T 	  1233.64	   616.34	  6
 12	 G 	  1290.66	   515.29	  5
 13	 V 	  1389.73	   458.27	  4
 14	 P 	  1486.78	   359.20	  3
 15	 S 	  1573.81	   262.15	  2
 16	 R 	        -  	   175.12	  1  _
+2 charge
		      B   	       Y
  1	 L 	    57.55	        -  	 16
  2	 L 	   114.09	   817.92	 15
  3	 I 	   170.63	   761.38	 14
  4	 Y 	   252.17	   704.84	 13
  5	 D 	   309.68	   623.31	 12
  6	 A 	   345.20	   565.79	 11
  7	 S 	   388.71	   530.28	 10
  8	 N 	   445.73	   486.76	  9
  9	 L 	   502.28	   429.74	  8
 10	 E 	   566.80	   373.20	  7
 11	 T 	   617.32	   308.67	  6
 12	 G 	   645.83	   258.15	  5
 13	 V 	   695.37	   229.64	  4
 14	 P 	   743.89	   180.11	  3
 15	 S 	   787.41	   131.58	  2
 16	 R 	        -  	    88.06	  1  _
```

**11. IPI00025861 Cadherin-1**

| Sequence | Charge | M+H+ | XCorr | DeltaCn |
| --- | --- | --- | --- | --- |
| K.MALEVGDYKINLK.L | 2 | 1494.781 | 3.868 | 0.461 |

+1 charge

```
		      B   	       Y
  1	 M 	   132.05	        -  	 13
  2	 A 	   203.08	  1362.76	 12
  3	 L 	   316.17	  1291.73	 11
  4	 E 	   445.21	  1178.64	 10
  5	 V 	   544.28	  1049.60	  9
  6	 G 	   601.30	   950.53	  8
  7	 D 	   716.33	   893.51	  7
  8	 Y 	   879.39	   778.48	  6
  9	 K 	  1007.49	   615.42	  5
 10	 I 	  1120.57	   487.32	  4
 11	 N 	  1234.61	   374.24	  3
 12	 L 	  1347.70	   260.20	  2
 13	 K 	        -  	   147.11	  1  _
+2 charge
		      B   	       Y
  1	 M 	    66.53	        -  	 13
  2	 A 	   102.05	   681.89	 12
  3	 L 	   158.59	   646.37	 11
  4	 E 	   223.11	   589.82	 10
  5	 V 	   272.64	   525.30	  9
  6	 G 	   301.15	   475.77	  8
  7	 D 	   358.67	   447.26	  7
  8	 Y 	   440.20	   389.74	  6
  9	 K 	   504.25	   308.21	  5
 10	 I 	   560.79	   244.17	  4
 11	 N 	   617.81	   187.62	  3
 12	 L 	   674.35	   130.60	  2
 13	 K 	        -  	    74.06	  1  _
```

**12. IPI00009477 Intercellular adhesion molecule 2**

| Sequence | Charge | M+H+ | XCorr | DeltaCn |
| --- | --- | --- | --- | --- |
| R.QVILTLQPTLVAVGK.S | 2 | 1580.937 | 3.134 | 0.47 |

+1 charge

```
		      B   	       Y
  1	 Q 	   129.07	        -  	 15
  2	 V 	   228.13	  1451.92	 14
  3	 I 	   341.22	  1352.85	 13
  4	 L 	   454.30	  1239.77	 12
  5	 T 	   555.35	  1126.68	 11
  6	 L 	   668.43	  1025.64	 10
  7	 Q 	   796.49	   912.55	  9
  8	 P 	   893.55	   784.49	  8
  9	 T 	   994.59	   687.44	  7
 10	 L 	  1107.68	   586.39	  6
 11	 V 	  1206.75	   473.31	  5
 12	 A 	  1277.78	   374.24	  4
 13	 V 	  1376.85	   303.20	  3
 14	 G 	  1433.87	   204.13	  2
 15	 K 	        -  	   147.11	  1  _
+2 charge
		      B   	       Y
  1	 Q 	    65.04	        -  	 15
  2	 V 	   114.57	   726.46	 14
  3	 I 	   171.11	   676.93	 13
  4	 L 	   227.65	   620.39	 12
  5	 T 	   278.18	   563.85	 11
  6	 L 	   334.72	   513.32	 10
  7	 Q 	   398.75	   456.78	  9
  8	 P 	   447.28	   392.75	  8
  9	 T 	   497.80	   344.22	  7
 10	 L 	   554.34	   293.70	  6
 11	 V 	   603.88	   237.16	  5
 12	 A 	   639.40	   187.62	  4
 13	 V 	   688.93	   152.10	  3
 14	 G 	   717.44	   102.57	  2
 15	 K 	        -  	    74.06	  1  _
```

**13. IPI00297420 Frizzled-4 precursor**

| Sequence | Charge | M+H+ | XCorr | DeltaCn |
| --- | --- | --- | --- | --- |
| K.INIPIGPCGGMCLSVK.R | 2 | 1717.054 | 3.313 | 0.461 |

+1 charge

```
		      B   	       Y
  1	 I 	   114.09	        -  	 16
  2	 N 	   228.13	  1603.05	 15
  3	 I 	   341.22	  1489.01	 14
  4	 P 	   438.27	  1375.92	 13
  5	 I 	   551.36	  1278.87	 12
  6	 G 	   608.38	  1165.79	 11
  7	 P 	   705.43	  1108.76	 10
  8	 C 	   865.59	  1011.71	  9
  9	 G 	   922.62	   851.55	  8
 10	 G 	   979.64	   794.52	  7
 11	 M 	  1110.68	   737.50	  6
 12	 C 	  1270.84	   606.46	  5
 13	 L 	  1383.93	   446.30	  4
 14	 S 	  1470.96	   333.21	  3
 15	 V 	  1570.03	   246.18	  2
 16	 K 	        -  	   147.11	  1  _
+2 charge
		      B   	       Y
  1	 I 	    57.55	        -  	 16
  2	 N 	   114.57	   802.03	 15
  3	 I 	   171.11	   745.01	 14
  4	 P 	   219.64	   688.46	 13
  5	 I 	   276.18	   639.94	 12
  6	 G 	   304.69	   583.40	 11
  7	 P 	   353.22	   554.89	 10
  8	 C 	   433.30	   506.36	  9
  9	 G 	   461.81	   426.28	  8
 10	 G 	   490.32	   397.77	  7
 11	 M 	   555.84	   369.26	  6
 12	 C 	   635.93	   303.73	  5
 13	 L 	   692.47	   223.65	  4
 14	 S 	   735.98	   167.11	  3
 15	 V 	   785.52	   123.59	  2
 16	 K 	        -  	    74.06	  1  _
```

**14. IPI00236556 Isoform H7 of Myeloperoxidase**

| Sequence | Charge | M+H+ | XCorr | DeltaCn |
| --- | --- | --- | --- | --- |
| R.VGPLLACIIGTQFR.K | 2 | 1545.845 | 3.229 | 0.456 |

+1 charge

```
		      B   	       Y
  1	 V 	   100.08	        -  	 14
  2	 G 	   157.10	  1445.93	 13
  3	 P 	   254.15	  1388.91	 12
  4	 L 	   367.23	  1291.85	 11
  5	 L 	   480.32	  1178.77	 10
  6	 A 	   551.36	  1065.69	  9
  7	 C 	   711.52	   994.65	  8
  8	 I 	   824.60	   834.48	  7
  9	 I 	   937.69	   721.40	  6
 10	 G 	   994.71	   608.32	  5
 11	 T 	  1095.76	   551.29	  4
 12	 Q 	  1223.82	   450.25	  3
 13	 F 	  1370.88	   322.19	  2
 14	 R 	        -  	   175.12	  1  _
+2 charge
		      B   	       Y
  1	 V 	    50.54	        -  	 14
  2	 G 	    79.05	   723.47	 13
  3	 P 	   127.58	   694.96	 12
  4	 L 	   184.12	   646.43	 11
  5	 L 	   240.66	   589.89	 10
  6	 A 	   276.18	   533.35	  9
  7	 C 	   356.26	   497.83	  8
  8	 I 	   412.81	   417.75	  7
  9	 I 	   469.35	   361.20	  6
 10	 G 	   497.86	   304.66	  5
 11	 T 	   548.38	   276.15	  4
 12	 Q 	   612.41	   225.63	  3
 13	 F 	   685.95	   161.60	  2
 14	 R 	        -  	    88.06	  1  _
```

**15. IPI00744184 Arylsulfatase A**

| Sequence | Charge | M+H+ | XCorr | DeltaCn |
| --- | --- | --- | --- | --- |
| R.GGLPLEEVTVAEVLAAR.G | 2 | 1724.98 | 3.752 | 0.44 |

+1 charge

```
		      B   	       Y
  1	 G 	    58.03	        -  	 17
  2	 G 	   115.05	  1666.94	 16
  3	 L 	   228.13	  1609.92	 15
  4	 P 	   325.19	  1496.83	 14
  5	 L 	   438.27	  1399.78	 13
  6	 E 	   567.31	  1286.70	 12
  7	 E 	   696.36	  1157.65	 11
  8	 V 	   795.42	  1028.61	 10
  9	 T 	   896.47	   929.54	  9
 10	 V 	   995.54	   828.49	  8
 11	 A 	  1066.58	   729.43	  7
 12	 E 	  1195.62	   658.39	  6
 13	 V 	  1294.69	   529.35	  5
 14	 L 	  1407.77	   430.28	  4
 15	 A 	  1478.81	   317.19	  3
 16	 A 	  1549.85	   246.16	  2
 17	 R 	        -  	   175.12	  1  _
+2 charge
		      B   	       Y
  1	 G 	    29.52	        -  	 17
  2	 G 	    58.03	   833.97	 16
  3	 L 	   114.57	   805.46	 15
  4	 P 	   163.10	   748.92	 14
  5	 L 	   219.64	   700.39	 13
  6	 E 	   284.16	   643.85	 12
  7	 E 	   348.68	   579.33	 11
  8	 V 	   398.22	   514.81	 10
  9	 T 	   448.74	   465.27	  9
 10	 V 	   498.27	   414.75	  8
 11	 A 	   533.79	   365.22	  7
 12	 E 	   598.31	   329.70	  6
 13	 V 	   647.85	   265.18	  5
 14	 L 	   704.39	   215.64	  4
 15	 A 	   739.91	   159.10	  3
 16	 A 	   775.43	   123.58	  2
 17	 R 	        -  	    88.06	  1  _
```

**16. IPI00056309 Liver-expressed antimicrobial peptide 2**

| Sequence | Charge | M+H+ | XCorr | DeltaCn |
| --- | --- | --- | --- | --- |
| R.GVSLRPIGASCRDDSECITR.L | 3 | 2250.443 | 4.655 | 0.396 |

+1 charge

```
		      B   	       Y
  1	 G 	    58.03	        -  	 20
  2	 V 	   157.10	  2192.32	 19
  3	 S 	   244.13	  2093.26	 18
  4	 L 	   357.21	  2006.22	 17
  5	 R 	   513.31	  1893.14	 16
  6	 P 	   610.37	  1737.04	 15
  7	 I 	   723.45	  1639.99	 14
  8	 G 	   780.47	  1526.90	 13
  9	 A 	   851.51	  1469.88	 12
 10	 S 	   938.54	  1398.84	 11
 11	 C 	  1098.71	  1311.81	 10
 12	 R 	  1254.81	  1151.65	  9
 13	 D 	  1369.84	   995.54	  8
 14	 D 	  1484.86	   880.52	  7
 15	 S 	  1571.89	   765.49	  6
 16	 E 	  1700.94	   678.46	  5
 17	 C 	  1861.10	   549.42	  4
 18	 I 	  1974.19	   389.25	  3
 19	 T 	  2075.23	   276.17	  2
 20	 R 	        -  	   175.12	  1  _
+2 charge
		      B   	       Y
  1	 G 	    29.52	        -  	 20
  2	 V 	    79.05	  1096.67	 19
  3	 S 	   122.57	  1047.13	 18
  4	 L 	   179.11	  1003.62	 17
  5	 R 	   257.16	   947.07	 16
  6	 P 	   305.69	   869.02	 15
  7	 I 	   362.23	   820.50	 14
  8	 G 	   390.74	   763.95	 13
  9	 A 	   426.26	   735.44	 12
 10	 S 	   469.77	   699.93	 11
 11	 C 	   549.86	   656.41	 10
 12	 R 	   627.91	   576.33	  9
 13	 D 	   685.42	   498.28	  8
 14	 D 	   742.93	   440.76	  7
 15	 S 	   786.45	   383.25	  6
 16	 E 	   850.97	   339.73	  5
 17	 C 	   931.05	   275.21	  4
 18	 I 	   987.60	   195.13	  3
 19	 T 	  1038.12	   138.59	  2
 20	 R 	        -  	    88.06	  1  _


+3 charge
		      B   	       Y
  1	 G 	    20.01	        -  	 20
  2	 V 	    53.04	   731.45	 19
  3	 S 	    82.05	   698.42	 18
  4	 L 	   119.74	   669.41	 17
  5	 R 	   171.78	   631.72	 16
  6	 P 	   204.13	   579.68	 15
  7	 I 	   241.82	   547.33	 14
  8	 G 	   260.83	   509.64	 13
  9	 A 	   284.51	   490.63	 12
 10	 S 	   313.52	   466.95	 11
 11	 C 	   366.91	   437.94	 10
 12	 R 	   418.94	   384.55	  9
 13	 D 	   457.28	   332.52	  8
 14	 D 	   495.63	   294.18	  7
 15	 S 	   524.64	   255.84	  6
 16	 E 	   567.65	   226.82	  5
 17	 C 	   621.04	   183.81	  4
 18	 I 	   658.73	   130.42	  3
 19	 T 	   692.42	    92.73	  2
 20	 R 	        -  	    59.04	  1  _
```

**17. IPI00374563 Agrin**

| Sequence | Charge | M+H+ | XCorr | DeltaCn |
| --- | --- | --- | --- | --- |
| R.ALGPAGCEADASAPATCAEMR.C | 2 | 2107.276 | 3.659 | 0.568 |

+1 charge

```
		      B   	       Y
  1	 A 	    72.04	        -  	 21
  2	 L 	   185.13	  2035.14	 20
  3	 G 	   242.15	  1922.05	 19
  4	 P 	   339.20	  1865.03	 18
  5	 A 	   410.24	  1767.98	 17
  6	 G 	   467.26	  1696.94	 16
  7	 C 	   627.43	  1639.92	 15
  8	 E 	   756.47	  1479.75	 14
  9	 A 	   827.51	  1350.71	 13
 10	 D 	   942.53	  1279.68	 12
 11	 A 	  1013.57	  1164.65	 11
 12	 S 	  1100.60	  1093.61	 10
 13	 A 	  1171.64	  1006.58	  9
 14	 P 	  1268.69	   935.54	  8
 15	 A 	  1339.73	   838.49	  7
 16	 T 	  1440.78	   767.45	  6
 17	 C 	  1600.94	   666.40	  5
 18	 A 	  1671.98	   506.24	  4
 19	 E 	  1801.02	   435.20	  3
 20	 M 	  1932.06	   306.16	  2
 21	 R 	        -  	   175.12	  1  _
+2 charge
		      B   	       Y
  1	 A 	    36.53	        -  	 21
  2	 L 	    93.07	  1018.07	 20
  3	 G 	   121.58	   961.53	 19
  4	 P 	   170.10	   933.02	 18
  5	 A 	   205.62	   884.49	 17
  6	 G 	   234.13	   848.97	 16
  7	 C 	   314.22	   820.46	 15
  8	 E 	   378.74	   740.38	 14
  9	 A 	   414.26	   675.86	 13
 10	 D 	   471.77	   640.34	 12
 11	 A 	   507.29	   582.83	 11
 12	 S 	   550.80	   547.31	 10
 13	 A 	   586.32	   503.79	  9
 14	 P 	   634.85	   468.27	  8
 15	 A 	   670.37	   419.75	  7
 16	 T 	   720.89	   384.23	  6
 17	 C 	   800.97	   333.71	  5
 18	 A 	   836.49	   253.62	  4
 19	 E 	   901.01	   218.10	  3
 20	 M 	   966.53	   153.58	  2
 21	 R 	        -  	    88.06	  1  _
```

**18. IPI00220642 14-3-3 protein gamma**

| Sequence | Charge | M+H+ | XCorr | DeltaCn |
| --- | --- | --- | --- | --- |
| K.LAEQAERYDDMAAAMK.A | 2 | 1814.034 | 2.977 | 0.421 |

+1 charge

```
		      B   	       Y
  1	 L 	   114.09	        -  	 16
  2	 A 	   185.13	  1699.74	 15
  3	 E 	   314.17	  1628.70	 14
  4	 Q 	   442.23	  1499.66	 13
  5	 A 	   513.27	  1371.60	 12
  6	 E 	   642.31	  1300.57	 11
  7	 R 	   798.41	  1171.52	 10
  8	 Y 	   961.47	  1015.42	  9
  9	 D 	  1076.50	   852.36	  8
 10	 D 	  1191.53	   737.33	  7
 11	 M 	  1322.57	   622.31	  6
 12	 A 	  1393.61	   491.26	  5
 13	 A 	  1464.64	   420.23	  4
 14	 A 	  1535.68	   349.19	  3
 15	 M 	  1666.72	   278.15	  2
 16	 K 	        -  	   147.11	  1  _
+2 charge
		      B   	       Y
  1	 L 	    57.55	        -  	 16
  2	 A 	    93.07	   850.37	 15
  3	 E 	   157.59	   814.86	 14
  4	 Q 	   221.62	   750.33	 13
  5	 A 	   257.14	   686.31	 12
  6	 E 	   321.66	   650.79	 11
  7	 R 	   399.71	   586.27	 10
  8	 Y 	   481.24	   508.21	  9
  9	 D 	   538.75	   426.68	  8
 10	 D 	   596.27	   369.17	  7
 11	 M 	   661.79	   311.66	  6
 12	 A 	   697.31	   246.14	  5
 13	 A 	   732.82	   210.62	  4
 14	 A 	   768.34	   175.10	  3
 15	 M 	   833.86	   139.58	  2
 16	 K 	        -  	    74.06	  1  _
```

**19. IPI00011694 Trypsin-1**

| Sequence | Charge | M+H+ | XCorr | DeltaCn |
| --- | --- | --- | --- | --- |
| K.TLNNDIMLIK.L | 2 | 1175.424 | 3.402 | 0.318 |

+1 charge

```
		      B   	       Y
  1	 T 	   102.05	        -  	 10
  2	 L 	   215.14	  1073.60	  9
  3	 N 	   329.18	   960.52	  8
  4	 N 	   443.22	   846.48	  7
  5	 D 	   558.25	   732.43	  6
  6	 I 	   671.34	   617.41	  5
  7	 M 	   802.38	   504.32	  4
  8	 L 	   915.46	   373.28	  3
  9	 I 	  1028.54	   260.20	  2
 10	 K 	        -  	   147.11	  1  _
+2 charge
		      B   	       Y
  1	 T 	    51.53	        -  	 10
  2	 L 	   108.07	   537.30	  9
  3	 N 	   165.09	   480.76	  8
  4	 N 	   222.12	   423.74	  7
  5	 D 	   279.63	   366.72	  6
  6	 I 	   336.17	   309.21	  5
  7	 M 	   401.69	   252.66	  4
  8	 L 	   458.23	   187.14	  3
  9	 I 	   514.78	   130.60	  2
 10	 K 	        -  	    74.06	  1  _
```

**20. IPI00651692 Stromal cell-derived factor 1 theta**

| Sequence | Charge | M+H+ | XCorr | DeltaCn |
| --- | --- | --- | --- | --- |
| K.ILNTPNCALQIVAR.L | 2 | 1583.851 | 4.133 | 0.482 |

+1 charge

```
		      B   	       Y
  1	 I 	   114.09	        -  	 14
  2	 L 	   227.18	  1469.92	 13
  3	 N 	   341.22	  1356.84	 12
  4	 T 	   442.27	  1242.80	 11
  5	 P 	   539.32	  1141.75	 10
  6	 N 	   653.36	  1044.70	  9
  7	 C 	   813.53	   930.65	  8
  8	 A 	   884.56	   770.49	  7
  9	 L 	   997.65	   699.45	  6
 10	 Q 	  1125.71	   586.37	  5
 11	 I 	  1238.79	   458.31	  4
 12	 V 	  1337.86	   345.22	  3
 13	 A 	  1408.90	   246.16	  2
 14	 R 	        -  	   175.12	  1  _
+2 charge
		      B   	       Y
  1	 I 	    57.55	        -  	 14
  2	 L 	   114.09	   735.47	 13
  3	 N 	   171.11	   678.92	 12
  4	 T 	   221.64	   621.90	 11
  5	 P 	   270.16	   571.38	 10
  6	 N 	   327.18	   522.85	  9
  7	 C 	   407.27	   465.83	  8
  8	 A 	   442.79	   385.75	  7
  9	 L 	   499.33	   350.23	  6
 10	 Q 	   563.36	   293.69	  5
 11	 I 	   619.90	   229.66	  4
 12	 V 	   669.43	   173.12	  3
 13	 A 	   704.95	   123.58	  2
 14	 R 	        -  	    88.06	  1  _
```

**21. IPI00025992 Hepcidin**

| Sequence | Charge | M+H+ | XCorr | DeltaCn |
| --- | --- | --- | --- | --- |
| R.DTHFPICIFCCGCCHR.S | 2 | 2141.34 | 3.529 | 0.524 |

+1 charge

```
		      B   	       Y
  1	 D 	   116.03	        -  	 16
  2	 T 	   217.08	  2025.49	 15
  3	 H 	   354.14	  1924.44	 14
  4	 F 	   501.21	  1787.38	 13
  5	 P 	   598.26	  1640.31	 12
  6	 I 	   711.35	  1543.26	 11
  7	 C 	   871.51	  1430.18	 10
  8	 I 	   984.60	  1270.01	  9
  9	 F 	  1131.66	  1156.93	  8
 10	 C 	  1291.83	  1009.86	  7
 11	 C 	  1451.99	   849.70	  6
 12	 G 	  1509.02	   689.53	  5
 13	 C 	  1669.18	   632.51	  4
 14	 C 	  1829.35	   472.34	  3
 15	 H 	  1966.41	   312.18	  2
 16	 R 	        -  	   175.12	  1  _
+2 charge
		      B   	       Y
  1	 D 	    58.52	        -  	 16
  2	 T 	   109.04	  1013.25	 15
  3	 H 	   177.57	   962.72	 14
  4	 F 	   251.11	   894.20	 13
  5	 P 	   299.63	   820.66	 12
  6	 I 	   356.18	   772.13	 11
  7	 C 	   436.26	   715.59	 10
  8	 I 	   492.80	   635.51	  9
  9	 F 	   566.34	   578.97	  8
 10	 C 	   646.42	   505.43	  7
 11	 C 	   726.50	   425.35	  6
 12	 G 	   755.01	   345.27	  5
 13	 C 	   835.09	   316.76	  4
 14	 C 	   915.18	   236.68	  3
 15	 H 	   983.71	   156.59	  2
 16	 R 	        -  	    88.06	  1  _
```

**22. IPI00031065 Deoxyribonuclease-1**

| Sequence | Charge | M+H+ | XCorr | DeltaCn |
| --- | --- | --- | --- | --- |
| K.LLDNLNQDAPDTYHYVVSEPLGR.N | 3 | 2630.851 | 4.228 | 0.478 |

+1 charge

```
		      B   	       Y
  1	 L 	   114.09	        -  	 23
  2	 L 	   227.18	  2516.21	 22
  3	 D 	   342.20	  2403.12	 21
  4	 N 	   456.25	  2288.09	 20
  5	 L 	   569.33	  2174.05	 19
  6	 N 	   683.37	  2060.97	 18
  7	 Q 	   811.43	  1946.92	 17
  8	 D 	   926.46	  1818.87	 16
  9	 A 	   997.49	  1703.84	 15
 10	 P 	  1094.55	  1632.80	 14
 11	 D 	  1209.57	  1535.75	 13
 12	 T 	  1310.62	  1420.72	 12
 13	 Y 	  1473.69	  1319.67	 11
 14	 H 	  1610.74	  1156.61	 10
 15	 Y 	  1773.81	  1019.55	  9
 16	 V 	  1872.88	   856.49	  8
 17	 V 	  1971.94	   757.42	  7
 18	 S 	  2058.98	   658.35	  6
 19	 E 	  2188.02	   571.32	  5
 20	 P 	  2285.07	   442.28	  4
 21	 L 	  2398.16	   345.22	  3
 22	 G 	  2455.18	   232.14	  2
 23	 R 	        -  	   175.12	  1  _


+2 charge
		      B   	       Y
  1	 L 	    57.55	        -  	 23
  2	 L 	   114.09	  1258.61	 22
  3	 D 	   171.60	  1202.06	 21
  4	 N 	   228.63	  1144.55	 20
  5	 L 	   285.17	  1087.53	 19
  6	 N 	   342.19	  1030.99	 18
  7	 Q 	   406.22	   973.97	 17
  8	 D 	   463.73	   909.94	 16
  9	 A 	   499.25	   852.42	 15
 10	 P 	   547.78	   816.90	 14
 11	 D 	   605.29	   768.38	 13
 12	 T 	   655.81	   710.86	 12
 13	 Y 	   737.35	   660.34	 11
 14	 H 	   805.88	   578.81	 10
 15	 Y 	   887.41	   510.28	  9
 16	 V 	   936.94	   428.75	  8
 17	 V 	   986.48	   379.21	  7
 18	 S 	  1029.99	   329.68	  6
 19	 E 	  1094.51	   286.16	  5
 20	 P 	  1143.04	   221.64	  4
 21	 L 	  1199.58	   173.12	  3
 22	 G 	  1228.09	   116.57	  2
 23	 R 	        -  	    88.06	  1  _


+3 charge
		      B   	       Y
  1	 L 	    38.70	        -  	 23
  2	 L 	    76.40	   839.41	 22
  3	 D 	   114.74	   801.71	 21
  4	 N 	   152.75	   763.37	 20
  5	 L 	   190.45	   725.36	 19
  6	 N 	   228.46	   687.66	 18
  7	 Q 	   271.15	   649.65	 17
  8	 D 	   309.49	   606.96	 16
  9	 A 	   333.17	   568.62	 15
 10	 P 	   365.52	   544.94	 14
 11	 D 	   403.86	   512.59	 13
 12	 T 	   437.55	   474.25	 12
 13	 Y 	   491.90	   440.56	 11
 14	 H 	   537.59	   386.21	 10
 15	 Y 	   591.94	   340.52	  9
 16	 V 	   624.96	   286.17	  8
 17	 V 	   657.99	   253.14	  7
 18	 S 	   687.00	   220.12	  6
 19	 E 	   730.01	   191.11	  5
 20	 P 	   762.36	   148.10	  4
 21	 L 	   800.06	   115.75	  3
 22	 G 	   819.06	    78.05	  2
 23	 R 	        -  	    59.04	  1  _
```

**23. IPI00884105 Lysosome-associated membrane glycoprotein 1**

| Sequence | Charge | M+H+ | XCorr | DeltaCn |
| --- | --- | --- | --- | --- |
| R.FFLQGIQLNTILPDARDPAFK.A | 3 | 2405.779 | 3.423 | 0.437 |

+1 charge

```
		      B   	       Y
  1	 F 	   148.08	        -  	 21
  2	 F 	   295.14	  2257.23	 20
  3	 L 	   408.23	  2110.17	 19
  4	 Q 	   536.29	  1997.08	 18
  5	 G 	   593.31	  1869.02	 17
  6	 I 	   706.39	  1812.00	 16
  7	 Q 	   834.45	  1698.92	 15
  8	 L 	   947.53	  1570.86	 14
  9	 N 	  1061.58	  1457.77	 13
 10	 T 	  1162.63	  1343.73	 12
 11	 I 	  1275.71	  1242.68	 11
 12	 L 	  1388.79	  1129.60	 10
 13	 P 	  1485.85	  1016.52	  9
 14	 D 	  1600.87	   919.46	  8
 15	 A 	  1671.91	   804.44	  7
 16	 R 	  1828.01	   733.40	  6
 17	 D 	  1943.04	   577.30	  5
 18	 P 	  2040.09	   462.27	  4
 19	 A 	  2111.13	   365.22	  3
 20	 F 	  2258.20	   294.18	  2
 21	 K 	        -  	   147.11	  1  _


+2 charge
		      B   	       Y
  1	 F 	    74.54	        -  	 21
  2	 F 	   148.08	  1129.12	 20
  3	 L 	   204.62	  1055.59	 19
  4	 Q 	   268.65	   999.04	 18
  5	 G 	   297.16	   935.02	 17
  6	 I 	   353.70	   906.50	 16
  7	 Q 	   417.73	   849.96	 15
  8	 L 	   474.27	   785.93	 14
  9	 N 	   531.29	   729.39	 13
 10	 T 	   581.82	   672.37	 12
 11	 I 	   638.36	   621.85	 11
 12	 L 	   694.90	   565.30	 10
 13	 P 	   743.43	   508.76	  9
 14	 D 	   800.94	   460.24	  8
 15	 A 	   836.46	   402.72	  7
 16	 R 	   914.51	   367.20	  6
 17	 D 	   972.02	   289.15	  5
 18	 P 	  1020.55	   231.64	  4
 19	 A 	  1056.07	   183.11	  3
 20	 F 	  1129.60	   147.59	  2
 21	 K 	        -  	    74.06	  1  _
+3 charge
		      B   	       Y
  1	 F 	    50.03	        -  	 21
  2	 F 	    99.05	   753.08	 20
  3	 L 	   136.75	   704.06	 19
  4	 Q 	   179.43	   666.37	 18
  5	 G 	   198.44	   623.68	 17
  6	 I 	   236.14	   604.67	 16
  7	 Q 	   278.82	   566.98	 15
  8	 L 	   316.52	   524.29	 14
  9	 N 	   354.53	   486.60	 13
 10	 T 	   388.21	   448.58	 12
 11	 I 	   425.91	   414.90	 11
 12	 L 	   463.60	   377.20	 10
 13	 P 	   495.95	   339.51	  9
 14	 D 	   534.30	   307.16	  8
 15	 A 	   557.98	   268.82	  7
 16	 R 	   610.01	   245.14	  6
 17	 D 	   648.35	   193.10	  5
 18	 P 	   680.70	   154.76	  4
 19	 A 	   704.38	   122.41	  3
 20	 F 	   753.40	    98.73	  2
 21	 K 	        -  	    49.71	  1  _
```

**24. IPI00005573 Isoform 1 of 5'(3')-deoxyribonucleotidase, cytosolic type**

| Sequence | Charge | M+H+ | XCorr | DeltaCn |
| --- | --- | --- | --- | --- |
| R.VLVDMDGVLADFEAGLLR.G | 2 | 1934.246 | 5.08 | 0.57 |

+1 charge

```
		      B   	       Y
  1	 V 	   100.08	        -  	 18
  2	 L 	   213.16	  1833.94	 17
  3	 V 	   312.23	  1720.86	 16
  4	 D 	   427.26	  1621.79	 15
  5	 M 	   558.30	  1506.76	 14
  6	 D 	   673.32	  1375.72	 13
  7	 G 	   730.34	  1260.69	 12
  8	 V 	   829.41	  1203.67	 11
  9	 L 	   942.50	  1104.60	 10
 10	 A 	  1013.53	   991.52	  9
 11	 D 	  1128.56	   920.48	  8
 12	 F 	  1275.63	   805.46	  7
 13	 E 	  1404.67	   658.39	  6
 14	 A 	  1475.71	   529.35	  5
 15	 G 	  1532.73	   458.31	  4
 16	 L 	  1645.81	   401.29	  3
 17	 L 	  1758.90	   288.20	  2
 18	 R 	        -  	   175.12	  1  _
+2 charge
		      B   	       Y
  1	 V 	    50.54	        -  	 18
  2	 L 	   107.08	   917.47	 17
  3	 V 	   156.62	   860.93	 16
  4	 D 	   214.13	   811.40	 15
  5	 M 	   279.65	   753.88	 14
  6	 D 	   337.16	   688.36	 13
  7	 G 	   365.68	   630.85	 12
  8	 V 	   415.21	   602.34	 11
  9	 L 	   471.75	   552.81	 10
 10	 A 	   507.27	   496.26	  9
 11	 D 	   564.78	   460.75	  8
 12	 F 	   638.32	   403.23	  7
 13	 E 	   702.84	   329.70	  6
 14	 A 	   738.36	   265.18	  5
 15	 G 	   766.87	   229.66	  4
 16	 L 	   823.41	   201.15	  3
 17	 L 	   879.95	   144.61	  2
 18	 R 	        -  	    88.06	  1  _
```

**25. IPI00022283 Trefoil factor 1**

| Sequence | Charge | M+H+ | XCorr | DeltaCn |
| --- | --- | --- | --- | --- |
| R.ERQNCGFPGVTPSQCANK.G | 2 | 2051.194 | 3.574 | 0.464 |

+1 charge

```
		      B   	       Y
  1	 E 	   130.05	        -  	 18
  2	 R 	   286.15	  1921.15	 17
  3	 Q 	   414.21	  1765.05	 16
  4	 N 	   528.25	  1636.99	 15
  5	 C 	   688.42	  1522.95	 14
  6	 G 	   745.44	  1362.78	 13
  7	 F 	   892.51	  1305.76	 12
  8	 P 	   989.56	  1158.69	 11
  9	 G 	  1046.58	  1061.64	 10
 10	 V 	  1145.65	  1004.62	  9
 11	 T 	  1246.70	   905.55	  8
 12	 P 	  1343.75	   804.50	  7
 13	 S 	  1430.78	   707.45	  6
 14	 Q 	  1558.84	   620.42	  5
 15	 C 	  1719.01	   492.36	  4
 16	 A 	  1790.04	   332.19	  3
 17	 N 	  1904.09	   261.16	  2
 18	 K 	        -  	   147.11	  1  _
+2 charge
		      B   	       Y
  1	 E 	    65.53	        -  	 18
  2	 R 	   143.58	   961.08	 17
  3	 Q 	   207.61	   883.03	 16
  4	 N 	   264.63	   819.00	 15
  5	 C 	   344.71	   761.98	 14
  6	 G 	   373.22	   681.89	 13
  7	 F 	   446.76	   653.38	 12
  8	 P 	   495.28	   579.85	 11
  9	 G 	   523.79	   531.32	 10
 10	 V 	   573.33	   502.81	  9
 11	 T 	   623.85	   453.28	  8
 12	 P 	   672.38	   402.75	  7
 13	 S 	   715.89	   354.23	  6
 14	 Q 	   779.92	   310.71	  5
 15	 C 	   860.01	   246.68	  4
 16	 A 	   895.53	   166.60	  3
 17	 N 	   952.55	   131.08	  2
 18	 K 	        -  	    74.06	  1  _
```

**26. IPI00102300 Isoform 3 of Platelet glycoprotein VI**

| Sequence | Charge | M+H+ | XCorr | DeltaCn |
| --- | --- | --- | --- | --- |
| R.ASFPIITVTAAHSGTYR.C | 2 | 1793.016 | 3.647 | 0.526 |

+1 charge

```
		      B   	       Y
  1	 A 	    72.04	        -  	 17
  2	 S 	   159.08	  1720.90	 16
  3	 F 	   306.14	  1633.87	 15
  4	 P 	   403.20	  1486.80	 14
  5	 I 	   516.28	  1389.75	 13
  6	 I 	   629.37	  1276.66	 12
  7	 T 	   730.41	  1163.58	 11
  8	 V 	   829.48	  1062.53	 10
  9	 T 	   930.53	   963.46	  9
 10	 A 	  1001.57	   862.42	  8
 11	 A 	  1072.60	   791.38	  7
 12	 H 	  1209.66	   720.34	  6
 13	 S 	  1296.69	   583.28	  5
 14	 G 	  1353.72	   496.25	  4
 15	 T 	  1454.76	   439.23	  3
 16	 Y 	  1617.83	   338.18	  2
 17	 R 	        -  	   175.12	  1  _
+2 charge
		      B   	       Y
  1	 A 	    36.53	        -  	 17
  2	 S 	    80.04	   860.95	 16
  3	 F 	   153.58	   817.44	 15
  4	 P 	   202.10	   743.90	 14
  5	 I 	   258.64	   695.38	 13
  6	 I 	   315.19	   638.84	 12
  7	 T 	   365.71	   582.29	 11
  8	 V 	   415.24	   531.77	 10
  9	 T 	   465.77	   482.24	  9
 10	 A 	   501.29	   431.71	  8
 11	 A 	   536.81	   396.19	  7
 12	 H 	   605.33	   360.67	  6
 13	 S 	   648.85	   292.15	  5
 14	 G 	   677.36	   248.63	  4
 15	 T 	   727.89	   220.12	  3
 16	 Y 	   809.42	   169.59	  2
 17	 R 	        -  	    88.06	  1  _
```

**27. IPI00011899 BMP and activin membrane-bound inhibitor homolog**

| Sequence | Charge | M+H+ | XCorr | DeltaCn |
| --- | --- | --- | --- | --- |
| R.LLDPQNSNSPLTHGCLDSLASTTDICQAK.Q | 3 | 3158.408 | 3.841 | 0.472 |

+1 charge

```
		      B   	       Y
  1	 L 	   114.09	        -  	 29
  2	 L 	   227.18	  3043.68	 28
  3	 D 	   342.20	  2930.59	 27
  4	 P 	   439.26	  2815.57	 26
  5	 Q 	   567.31	  2718.51	 25
  6	 N 	   681.36	  2590.46	 24
  7	 S 	   768.39	  2476.41	 23
  8	 N 	   882.43	  2389.38	 22
  9	 S 	   969.46	  2275.34	 21
 10	 P 	  1066.52	  2188.31	 20
 11	 L 	  1179.60	  2091.25	 19
 12	 T 	  1280.65	  1978.17	 18
 13	 H 	  1417.71	  1877.12	 17
 14	 G 	  1474.73	  1740.06	 16
 15	 C 	  1634.89	  1683.04	 15
 16	 L 	  1747.98	  1522.88	 14
 17	 D 	  1863.00	  1409.79	 13
 18	 S 	  1950.04	  1294.77	 12
 19	 L 	  2063.12	  1207.73	 11
 20	 A 	  2134.16	  1094.65	 10
 21	 S 	  2221.19	  1023.61	  9
 22	 T 	  2322.24	   936.58	  8
 23	 T 	  2423.29	   835.53	  7
 24	 D 	  2538.31	   734.48	  6
 25	 I 	  2651.40	   619.46	  5
 26	 C 	  2811.56	   506.37	  4
 27	 Q 	  2939.62	   346.21	  3
 28	 A 	  3010.66	   218.15	  2
 29	 K 	        -  	   147.11	  1  _


+2 charge
		      B   	       Y
  1	 L 	    57.55	        -  	 29
  2	 L 	   114.09	  1522.34	 28
  3	 D 	   171.60	  1465.80	 27
  4	 P 	   220.13	  1408.29	 26
  5	 Q 	   284.16	  1359.76	 25
  6	 N 	   341.18	  1295.73	 24
  7	 S 	   384.70	  1238.71	 23
  8	 N 	   441.72	  1195.19	 22
  9	 S 	   485.24	  1138.17	 21
 10	 P 	   533.76	  1094.66	 20
 11	 L 	   590.30	  1046.13	 19
 12	 T 	   640.83	   989.59	 18
 13	 H 	   709.36	   939.06	 17
 14	 G 	   737.87	   870.54	 16
 15	 C 	   817.95	   842.02	 15
 16	 L 	   874.49	   761.94	 14
 17	 D 	   932.01	   705.40	 13
 18	 S 	   975.52	   647.89	 12
 19	 L 	  1032.06	   604.37	 11
 20	 A 	  1067.58	   547.83	 10
 21	 S 	  1111.10	   512.31	  9
 22	 T 	  1161.62	   468.79	  8
 23	 T 	  1212.15	   418.27	  7
 24	 D 	  1269.66	   367.75	  6
 25	 I 	  1326.20	   310.23	  5
 26	 C 	  1406.28	   253.69	  4
 27	 Q 	  1470.31	   173.61	  3
 28	 A 	  1505.83	   109.58	  2
 29	 K 	        -  	    74.06	  1  _


+3 charge
		      B   	       Y
  1	 L 	    38.70	        -  	 29
  2	 L 	    76.40	  1015.23	 28
  3	 D 	   114.74	   977.54	 27
  4	 P 	   147.09	   939.19	 26
  5	 Q 	   189.78	   906.84	 25
  6	 N 	   227.79	   864.16	 24
  7	 S 	   256.80	   826.14	 23
  8	 N 	   294.82	   797.13	 22
  9	 S 	   323.83	   759.12	 21
 10	 P 	   356.18	   730.11	 20
 11	 L 	   393.87	   697.76	 19
 12	 T 	   427.55	   660.06	 18
 13	 H 	   473.24	   626.38	 17
 14	 G 	   492.25	   580.69	 16
 15	 C 	   545.64	   561.69	 15
 16	 L 	   583.33	   508.30	 14
 17	 D 	   621.67	   470.60	 13
 18	 S 	   650.68	   432.26	 12
 19	 L 	   688.38	   403.25	 11
 20	 A 	   712.06	   365.55	 10
 21	 S 	   741.07	   341.88	  9
 22	 T 	   774.75	   312.86	  8
 23	 T 	   808.43	   279.18	  7
 24	 D 	   846.78	   245.50	  6
 25	 I 	   884.47	   207.16	  5
 26	 C 	   937.86	   169.46	  4
 27	 Q 	   980.54	   116.07	  3
 28	 A 	  1004.22	    73.39	  2
 29	 K 	        -  	    49.71	  1  _
```

**28. IPI00004656 Beta-2-microglobulin**

| Sequence | Charge | M+H+ | XCorr | DeltaCn |
| --- | --- | --- | --- | --- |
| K.SNFLNCYVSGFHPSDIEVDLLKNGER.I | 3 | 3012.272 | 3.637 | 0.541 |

+1 charge

```
		      B   	       Y
  1	 S 	    88.04	        -  	 26
  2	 N 	   202.08	  2923.54	 25
  3	 F 	   349.15	  2809.50	 24
  4	 L 	   462.23	  2662.43	 23
  5	 N 	   576.28	  2549.34	 22
  6	 C 	   736.44	  2435.30	 21
  7	 Y 	   899.51	  2275.14	 20
  8	 V 	   998.57	  2112.07	 19
  9	 S 	  1085.61	  2013.00	 18
 10	 G 	  1142.63	  1925.97	 17
 11	 F 	  1289.70	  1868.95	 16
 12	 H 	  1426.76	  1721.88	 15
 13	 P 	  1523.81	  1584.82	 14
 14	 S 	  1610.84	  1487.77	 13
 15	 D 	  1725.87	  1400.74	 12
 16	 I 	  1838.95	  1285.71	 11
 17	 E 	  1967.99	  1172.63	 10
 18	 V 	  2067.06	  1043.58	  9
 19	 D 	  2182.09	   944.52	  8
 20	 L 	  2295.17	   829.49	  7
 21	 L 	  2408.26	   716.40	  6
 22	 K 	  2536.35	   603.32	  5
 23	 N 	  2650.40	   475.23	  4
 24	 G 	  2707.42	   361.18	  3
 25	 E 	  2836.46	   304.16	  2
 26	 R 	        -  	   175.12	  1  _


+2 charge
		      B   	       Y
  1	 S 	    44.52	        -  	 26
  2	 N 	   101.54	  1462.27	 25
  3	 F 	   175.08	  1405.25	 24
  4	 L 	   231.62	  1331.72	 23
  5	 N 	   288.64	  1275.18	 22
  6	 C 	   368.73	  1218.15	 21
  7	 Y 	   450.26	  1138.07	 20
  8	 V 	   499.79	  1056.54	 19
  9	 S 	   543.31	  1007.01	 18
 10	 G 	   571.82	   963.49	 17
 11	 F 	   645.35	   934.98	 16
 12	 H 	   713.88	   861.44	 15
 13	 P 	   762.41	   792.92	 14
 14	 S 	   805.92	   744.39	 13
 15	 D 	   863.44	   700.87	 12
 16	 I 	   919.98	   643.36	 11
 17	 E 	   984.50	   586.82	 10
 18	 V 	  1034.03	   522.30	  9
 19	 D 	  1091.55	   472.76	  8
 20	 L 	  1148.09	   415.25	  7
 21	 L 	  1204.63	   358.71	  6
 22	 K 	  1268.68	   302.16	  5
 23	 N 	  1325.70	   238.12	  4
 24	 G 	  1354.21	   181.10	  3
 25	 E 	  1418.73	   152.58	  2
 26	 R 	        -  	    88.06	  1  _


+3 charge
		      B   	       Y
  1	 S 	    30.02	        -  	 26
  2	 N 	    68.03	   975.18	 25
  3	 F 	   117.06	   937.17	 24
  4	 L 	   154.75	   888.15	 23
  5	 N 	   192.76	   850.45	 22
  6	 C 	   246.15	   812.44	 21
  7	 Y 	   300.51	   759.05	 20
  8	 V 	   333.53	   704.70	 19
  9	 S 	   362.54	   671.67	 18
 10	 G 	   381.55	   642.66	 17
 11	 F 	   430.57	   623.65	 16
 12	 H 	   476.26	   574.63	 15
 13	 P 	   508.61	   528.95	 14
 14	 S 	   537.62	   496.59	 13
 15	 D 	   575.96	   467.58	 12
 16	 I 	   613.66	   429.24	 11
 17	 E 	   656.67	   391.55	 10
 18	 V 	   689.69	   348.53	  9
 19	 D 	   728.03	   315.51	  8
 20	 L 	   765.73	   277.17	  7
 21	 L 	   803.42	   239.47	  6
 22	 K 	   846.12	   201.78	  5
 23	 N 	   884.14	   159.08	  4
 24	 G 	   903.14	   121.07	  3
 25	 E 	   946.16	   102.06	  2
 26	 R 	        -  	    59.04	  1  _
```

**29. IPI00073772 Fructose-1,6-bisphosphatase 1**

| Sequence | Charge | M+H+ | XCorr | DeltaCn |
| --- | --- | --- | --- | --- |
| R.APVILGSPDDVLEFLK.V | 2 | 1713.995 | 4.014 | 0.611 |

+1 charge

```
		      B   	       Y
  1	 A 	    72.04	        -  	 16
  2	 P 	   169.10	  1641.91	 15
  3	 V 	   268.17	  1544.86	 14
  4	 I 	   381.25	  1445.79	 13
  5	 L 	   494.33	  1332.70	 12
  6	 G 	   551.36	  1219.62	 11
  7	 S 	   638.39	  1162.60	 10
  8	 P 	   735.44	  1075.57	  9
  9	 D 	   850.47	   978.51	  8
 10	 D 	   965.49	   863.49	  7
 11	 V 	  1064.56	   748.46	  6
 12	 L 	  1177.65	   649.39	  5
 13	 E 	  1306.69	   536.31	  4
 14	 F 	  1453.76	   407.27	  3
 15	 L 	  1566.84	   260.20	  2
 16	 K 	        -  	   147.11	  1  _
+2 charge
		      B   	       Y
  1	 A 	    36.53	        -  	 16
  2	 P 	    85.05	   821.46	 15
  3	 V 	   134.59	   772.93	 14
  4	 I 	   191.13	   723.40	 13
  5	 L 	   247.67	   666.86	 12
  6	 G 	   276.18	   610.31	 11
  7	 S 	   319.70	   581.80	 10
  8	 P 	   368.22	   538.29	  9
  9	 D 	   425.74	   489.76	  8
 10	 D 	   483.25	   432.25	  7
 11	 V 	   532.78	   374.73	  6
 12	 L 	   589.33	   325.20	  5
 13	 E 	   653.85	   268.66	  4
 14	 F 	   727.38	   204.14	  3
 15	 L 	   783.92	   130.60	  2
 16	 K 	        -  	    74.06	  1  _
```

**30. IPI00470823 Isoform 1 of Pancreatic secretory granule membrane major glycoprotein GP2**

| Sequence | Charge | M+H+ | XCorr | DeltaCn |
| --- | --- | --- | --- | --- |
| R.VLDLGPITR.R | 1 | 984.1747 | 1.83 | 0.279 |

+1 charge

```
		      B   	       Y
  1	 V 	   100.08	        -  	  9
  2	 L 	   213.16	   884.52	  8
  3	 D 	   328.19	   771.44	  7
  4	 L 	   441.27	   656.41	  6
  5	 G 	   498.29	   543.32	  5
  6	 P 	   595.34	   486.30	  4
  7	 I 	   708.43	   389.25	  3
  8	 T 	   809.48	   276.17	  2
  9	 R 	        -  	   175.12	  1  _
```

**31. IPI00021439 Actin, cytoplasmic 1**

| Sequence | Charge | M+H+ | XCorr | DeltaCn |
| --- | --- | --- | --- | --- |
| R.VAPEEHPVLLTEAPLNPK.A | 2 | 1955.244 | 3.223 | 0.484 |

+1 charge

```
		      B   	       Y
  1	 V 	   100.08	        -  	 18
  2	 A 	   171.11	  1855.00	 17
  3	 P 	   268.17	  1783.96	 16
  4	 E 	   397.21	  1686.91	 15
  5	 E 	   526.25	  1557.86	 14
  6	 H 	   663.31	  1428.82	 13
  7	 P 	   760.36	  1291.76	 12
  8	 V 	   859.43	  1194.71	 11
  9	 L 	   972.51	  1095.64	 10
 10	 L 	  1085.60	   982.56	  9
 11	 T 	  1186.65	   869.47	  8
 12	 E 	  1315.69	   768.43	  7
 13	 A 	  1386.73	   639.38	  6
 14	 P 	  1483.78	   568.35	  5
 15	 L 	  1596.86	   471.29	  4
 16	 N 	  1710.91	   358.21	  3
 17	 P 	  1807.96	   244.17	  2
 18	 K 	        -  	   147.11	  1  _
+2 charge
		      B   	       Y
  1	 V 	    50.54	        -  	 18
  2	 A 	    86.06	   928.00	 17
  3	 P 	   134.59	   892.48	 16
  4	 E 	   199.11	   843.96	 15
  5	 E 	   263.63	   779.44	 14
  6	 H 	   332.16	   714.91	 13
  7	 P 	   380.68	   646.38	 12
  8	 V 	   430.22	   597.86	 11
  9	 L 	   486.76	   548.32	 10
 10	 L 	   543.30	   491.78	  9
 11	 T 	   593.83	   435.24	  8
 12	 E 	   658.35	   384.72	  7
 13	 A 	   693.87	   320.19	  6
 14	 P 	   742.39	   284.68	  5
 15	 L 	   798.94	   236.15	  4
 16	 N 	   855.96	   179.61	  3
 17	 P 	   904.48	   122.59	  2
 18	 K 	        -  	    74.06	  1  _
```

**32. IPI00179330 ubiquitin and ribosomal protein S27a precursor**

| Sequence | Charge | M+H+ | XCorr | DeltaCn |
| --- | --- | --- | --- | --- |
| K.TITLEVEPSDTIENVK.A | 2 | 1788.974 | 2.927 | 0.296 |

+1 charge

```
		      B   	       Y
  1	 T 	   102.05	        -  	 16
  2	 I 	   215.14	  1686.88	 15
  3	 T 	   316.19	  1573.80	 14
  4	 L 	   429.27	  1472.75	 13
  5	 E 	   558.31	  1359.66	 12
  6	 V 	   657.38	  1230.62	 11
  7	 E 	   786.42	  1131.55	 10
  8	 P 	   883.48	  1002.51	  9
  9	 S 	   970.51	   905.46	  8
 10	 D 	  1085.54	   818.43	  7
 11	 T 	  1186.58	   703.40	  6
 12	 I 	  1299.67	   602.35	  5
 13	 E 	  1428.71	   489.27	  4
 14	 N 	  1542.75	   360.22	  3
 15	 V 	  1641.82	   246.18	  2
 16	 K 	        -  	   147.11	  1  _
+2 charge
		      B   	       Y
  1	 T 	    51.53	        -  	 16
  2	 I 	   108.07	   843.94	 15
  3	 T 	   158.60	   787.40	 14
  4	 L 	   215.14	   736.88	 13
  5	 E 	   279.66	   680.34	 12
  6	 V 	   329.19	   615.81	 11
  7	 E 	   393.72	   566.28	 10
  8	 P 	   442.24	   501.76	  9
  9	 S 	   485.76	   453.23	  8
 10	 D 	   543.27	   409.72	  7
 11	 T 	   593.80	   352.20	  6
 12	 I 	   650.34	   301.68	  5
 13	 E 	   714.86	   245.14	  4
 14	 N 	   771.88	   180.62	  3
 15	 V 	   821.41	   123.59	  2
 16	 K 	        -  	    74.06	  1  _
```

**33. IPI00896419 Isoform 1 of Inter-alpha-trypsin inhibitor heavy chain H4**

| Sequence | Charge | M+H+ | XCorr | DeltaCn |
| --- | --- | --- | --- | --- |
| R.QGPVNLLSDPEQGVEVTGQYEREK.A | 3 | 2673.874 | 3.958 | 0.455 |

+1 charge

```
		      B   	       Y
  1	 Q 	   129.07	        -  	 24
  2	 G 	   186.09	  2544.26	 23
  3	 P 	   283.14	  2487.24	 22
  4	 V 	   382.21	  2390.18	 21
  5	 N 	   496.25	  2291.11	 20
  6	 L 	   609.34	  2177.07	 19
  7	 L 	   722.42	  2063.99	 18
  8	 S 	   809.45	  1950.90	 17
  9	 D 	   924.48	  1863.87	 16
 10	 P 	  1021.53	  1748.84	 15
 11	 E 	  1150.57	  1651.79	 14
 12	 Q 	  1278.63	  1522.75	 13
 13	 G 	  1335.65	  1394.69	 12
 14	 V 	  1434.72	  1337.67	 11
 15	 E 	  1563.76	  1238.60	 10
 16	 V 	  1662.83	  1109.56	  9
 17	 T 	  1763.88	  1010.49	  8
 18	 G 	  1820.90	   909.44	  7
 19	 Q 	  1948.96	   852.42	  6
 20	 Y 	  2112.02	   724.36	  5
 21	 E 	  2241.07	   561.30	  4
 22	 R 	  2397.17	   432.26	  3
 23	 E 	  2526.21	   276.16	  2
 24	 K 	        -  	   147.11	  1  _


+2 charge
		      B   	       Y
  1	 Q 	    65.04	        -  	 24
  2	 G 	    93.55	  1272.63	 23
  3	 P 	   142.07	  1244.12	 22
  4	 V 	   191.61	  1195.60	 21
  5	 N 	   248.63	  1146.06	 20
  6	 L 	   305.17	  1089.04	 19
  7	 L 	   361.71	  1032.50	 18
  8	 S 	   405.23	   975.96	 17
  9	 D 	   462.74	   932.44	 16
 10	 P 	   511.27	   874.93	 15
 11	 E 	   575.79	   826.40	 14
 12	 Q 	   639.82	   761.88	 13
 13	 G 	   668.33	   697.85	 12
 14	 V 	   717.86	   669.34	 11
 15	 E 	   782.39	   619.80	 10
 16	 V 	   831.92	   555.28	  9
 17	 T 	   882.44	   505.75	  8
 18	 G 	   910.95	   455.22	  7
 19	 Q 	   974.98	   426.71	  6
 20	 Y 	  1056.52	   362.68	  5
 21	 E 	  1121.04	   281.15	  4
 22	 R 	  1199.09	   216.63	  3
 23	 E 	  1263.61	   138.58	  2
 24	 K 	        -  	    74.06	  1  _


+3 charge
		      B   	       Y
  1	 Q 	    43.69	        -  	 24
  2	 G 	    62.70	   848.76	 23
  3	 P 	    95.05	   829.75	 22
  4	 V 	   128.07	   797.40	 21
  5	 N 	   166.09	   764.38	 20
  6	 L 	   203.78	   726.36	 19
  7	 L 	   241.48	   688.67	 18
  8	 S 	   270.49	   650.97	 17
  9	 D 	   308.83	   621.96	 16
 10	 P 	   341.18	   583.62	 15
 11	 E 	   384.20	   551.27	 14
 12	 Q 	   426.88	   508.25	 13
 13	 G 	   445.89	   465.57	 12
 14	 V 	   478.91	   446.56	 11
 15	 E 	   521.93	   413.54	 10
 16	 V 	   554.95	   370.52	  9
 17	 T 	   588.63	   337.50	  8
 18	 G 	   607.64	   303.82	  7
 19	 Q 	   650.33	   284.81	  6
 20	 Y 	   704.68	   242.13	  5
 21	 E 	   747.69	   187.77	  4
 22	 R 	   799.73	   144.76	  3
 23	 E 	   842.74	    92.72	  2
 24	 K 	        -  	    49.71	  1  _
```

**34. IPI00299162 V(D)J recombination-activating protein 2**

| Sequence | Charge | M+H+ | XCorr | DeltaCn |
| --- | --- | --- | --- | --- |
| K.RMICNIISLEDNKIEIR.E | 2 | 2118.481 | 2.968 | 0.237 |

+1 charge

```
		      B   	       Y
  1	 R 	   157.11	        -  	 17
  2	 M 	   288.15	  1961.15	 16
  3	 I 	   401.23	  1830.11	 15
  4	 C 	   561.40	  1717.03	 14
  5	 N 	   675.44	  1556.86	 13
  6	 I 	   788.53	  1442.82	 12
  7	 I 	   901.61	  1329.74	 11
  8	 S 	   988.64	  1216.65	 10
  9	 L 	  1101.73	  1129.62	  9
 10	 E 	  1230.77	  1016.54	  8
 11	 D 	  1345.79	   887.49	  7
 12	 N 	  1459.84	   772.47	  6
 13	 K 	  1587.93	   658.42	  5
 14	 I 	  1701.02	   530.33	  4
 15	 E 	  1830.06	   417.25	  3
 16	 I 	  1943.14	   288.20	  2
 17	 R 	        -  	   175.12	  1  _
+2 charge
		      B   	       Y
  1	 R 	    79.06	        -  	 17
  2	 M 	   144.58	   981.08	 16
  3	 I 	   201.12	   915.56	 15
  4	 C 	   281.20	   859.02	 14
  5	 N 	   338.22	   778.94	 13
  6	 I 	   394.77	   721.91	 12
  7	 I 	   451.31	   665.37	 11
  8	 S 	   494.82	   608.83	 10
  9	 L 	   551.37	   565.31	  9
 10	 E 	   615.89	   508.77	  8
 11	 D 	   673.40	   444.25	  7
 12	 N 	   730.42	   386.74	  6
 13	 K 	   794.47	   329.72	  5
 14	 I 	   851.01	   265.67	  4
 15	 E 	   915.53	   209.13	  3
 16	 I 	   972.08	   144.61	  2
 17	 R 	        -  	    88.06	  1  _
```

**35. IPI00176221 Neuronal growth regulator 1**

| Sequence | Charge | M+H+ | XCorr | DeltaCn |
| --- | --- | --- | --- | --- |
| K.VVVNFAPTIQEIK.S | 2 | 1458.727 | 3.78 | 0.382 |

+1 charge

```
		      B   	       Y
  1	 V 	   100.08	        -  	 13
  2	 V 	   199.14	  1358.77	 12
  3	 V 	   298.21	  1259.70	 11
  4	 N 	   412.26	  1160.63	 10
  5	 F 	   559.32	  1046.59	  9
  6	 A 	   630.36	   899.52	  8
  7	 P 	   727.41	   828.48	  7
  8	 T 	   828.46	   731.43	  6
  9	 I 	   941.55	   630.38	  5
 10	 Q 	  1069.60	   517.30	  4
 11	 E 	  1198.65	   389.24	  3
 12	 I 	  1311.73	   260.20	  2
 13	 K 	        -  	   147.11	  1  _
+2 charge
		      B   	       Y
  1	 V 	    50.54	        -  	 13
  2	 V 	   100.08	   679.89	 12
  3	 V 	   149.61	   630.35	 11
  4	 N 	   206.63	   580.82	 10
  5	 F 	   280.17	   523.80	  9
  6	 A 	   315.68	   450.26	  8
  7	 P 	   364.21	   414.74	  7
  8	 T 	   414.73	   366.22	  6
  9	 I 	   471.28	   315.69	  5
 10	 Q 	   535.31	   259.15	  4
 11	 E 	   599.83	   195.12	  3
 12	 I 	   656.37	   130.60	  2
 13	 K 	        -  	    74.06	  1  _
```

**36. IPI00395337 Isoform 1 of Pre-mRNA 3'-end-processing factor FIP1**

| Sequence | Charge | M+H+ | XCorr | DeltaCn |
| --- | --- | --- | --- | --- |
| K.GVDLDAPGSINGVPLLEVDLDSFEDK.P | 3 | 2715.947 | 4.06 | 0.219 |

+1 charge

```
		      B   	       Y
  1	 G 	    58.03	        -  	 26
  2	 V 	   157.10	  2657.32	 25
  3	 D 	   272.12	  2558.25	 24
  4	 L 	   385.21	  2443.22	 23
  5	 D 	   500.24	  2330.14	 22
  6	 A 	   571.27	  2215.11	 21
  7	 P 	   668.32	  2144.08	 20
  8	 G 	   725.35	  2047.02	 19
  9	 S 	   812.38	  1990.00	 18
 10	 I 	   925.46	  1902.97	 17
 11	 N 	  1039.51	  1789.89	 16
 12	 G 	  1096.53	  1675.84	 15
 13	 V 	  1195.60	  1618.82	 14
 14	 P 	  1292.65	  1519.75	 13
 15	 L 	  1405.73	  1422.70	 12
 16	 L 	  1518.82	  1309.62	 11
 17	 E 	  1647.86	  1196.53	 10
 18	 V 	  1746.93	  1067.49	  9
 19	 D 	  1861.95	   968.42	  8
 20	 L 	  1975.04	   853.39	  7
 21	 D 	  2090.07	   740.31	  6
 22	 S 	  2177.10	   625.28	  5
 23	 F 	  2324.17	   538.25	  4
 24	 E 	  2453.21	   391.18	  3
 25	 D 	  2568.24	   262.14	  2
 26	 K 	        -  	   147.11	  1  _


+2 charge
		      B   	       Y
  1	 G 	    29.52	        -  	 26
  2	 V 	    79.05	  1329.16	 25
  3	 D 	   136.57	  1279.63	 24
  4	 L 	   193.11	  1222.12	 23
  5	 D 	   250.62	  1165.57	 22
  6	 A 	   286.14	  1108.06	 21
  7	 P 	   334.67	  1072.54	 20
  8	 G 	   363.18	  1024.02	 19
  9	 S 	   406.69	   995.50	 18
 10	 I 	   463.23	   951.99	 17
 11	 N 	   520.26	   895.45	 16
 12	 G 	   548.77	   838.42	 15
 13	 V 	   598.30	   809.91	 14
 14	 P 	   646.83	   760.38	 13
 15	 L 	   703.37	   711.85	 12
 16	 L 	   759.91	   655.31	 11
 17	 E 	   824.43	   598.77	 10
 18	 V 	   873.97	   534.25	  9
 19	 D 	   931.48	   484.71	  8
 20	 L 	   988.02	   427.20	  7
 21	 D 	  1045.54	   370.66	  6
 22	 S 	  1089.05	   313.15	  5
 23	 F 	  1162.59	   269.63	  4
 24	 E 	  1227.11	   196.09	  3
 25	 D 	  1284.62	   131.57	  2
 26	 K 	        -  	    74.06	  1  _


+3 charge
		      B   	       Y
  1	 G 	    20.01	        -  	 26
  2	 V 	    53.04	   886.44	 25
  3	 D 	    91.38	   853.42	 24
  4	 L 	   129.07	   815.08	 23
  5	 D 	   167.42	   777.38	 22
  6	 A 	   191.10	   739.04	 21
  7	 P 	   223.45	   715.36	 20
  8	 G 	   242.45	   683.01	 19
  9	 S 	   271.46	   664.01	 18
 10	 I 	   309.16	   634.99	 17
 11	 N 	   347.17	   597.30	 16
 12	 G 	   366.18	   559.29	 15
 13	 V 	   399.20	   540.28	 14
 14	 P 	   431.55	   507.26	 13
 15	 L 	   469.25	   474.90	 12
 16	 L 	   506.94	   437.21	 11
 17	 E 	   549.96	   399.52	 10
 18	 V 	   582.98	   356.50	  9
 19	 D 	   621.32	   323.48	  8
 20	 L 	   659.02	   285.14	  7
 21	 D 	   697.36	   247.44	  6
 22	 S 	   726.37	   209.10	  5
 23	 F 	   775.39	   180.09	  4
 24	 E 	   818.41	   131.07	  3
 25	 D 	   856.75	    88.05	  2
 26	 K 	        -  	    49.71	  1  _
```

**37. IPI00064377 Tumor necrosis factor receptor superfamily member 19L**

| Sequence | Charge | M+H+ | XCorr | DeltaCn |
| --- | --- | --- | --- | --- |
| R.RLEAQVGMATR.D | 2 | 1232.439 | 3.561 | 0.314 |

+1 charge

```
		      B   	       Y
  1	 R 	   157.11	        -  	 11
  2	 L 	   270.19	  1075.56	 10
  3	 E 	   399.24	   962.47	  9
  4	 A 	   470.27	   833.43	  8
  5	 Q 	   598.33	   762.39	  7
  6	 V 	   697.40	   634.33	  6
  7	 G 	   754.42	   535.27	  5
  8	 M 	   885.46	   478.24	  4
  9	 A 	   956.50	   347.20	  3
 10	 T 	  1057.55	   276.17	  2
 11	 R 	        -  	   175.12	  1  _
+2 charge
		      B   	       Y
  1	 R 	    79.06	        -  	 11
  2	 L 	   135.60	   538.28	 10
  3	 E 	   200.12	   481.74	  9
  4	 A 	   235.64	   417.22	  8
  5	 Q 	   299.67	   381.70	  7
  6	 V 	   349.20	   317.67	  6
  7	 G 	   377.71	   268.14	  5
  8	 M 	   443.23	   239.63	  4
  9	 A 	   478.75	   174.11	  3
 10	 T 	   529.28	   138.59	  2
 11	 R 	        -  	    88.06	  1  _
```

**38. IPI00008239 cDNA FLJ55176, highly similar to G-protein coupled receptor family C group 5 member B**

| Sequence | Charge | M+H+ | XCorr | DeltaCn |
| --- | --- | --- | --- | --- |
| K.AFSMDEHNAALR.T | 2 | 1362.497 | 3.172 | 0.4 |

+1 charge

```
		      B   	       Y
  1	 A 	    72.04	        -  	 12
  2	 F 	   219.11	  1290.59	 11
  3	 S 	   306.14	  1143.52	 10
  4	 M 	   437.19	  1056.49	  9
  5	 D 	   552.21	   925.45	  8
  6	 E 	   681.25	   810.42	  7
  7	 H 	   818.31	   681.38	  6
  8	 N 	   932.36	   544.32	  5
  9	 A 	  1003.39	   430.28	  4
 10	 A 	  1074.43	   359.24	  3
 11	 L 	  1187.51	   288.20	  2
 12	 R 	        -  	   175.12	  1  _
+2 charge
		      B   	       Y
  1	 A 	    36.53	        -  	 12
  2	 F 	   110.06	   645.80	 11
  3	 S 	   153.58	   572.26	 10
  4	 M 	   219.10	   528.75	  9
  5	 D 	   276.61	   463.23	  8
  6	 E 	   341.13	   405.71	  7
  7	 H 	   409.66	   341.19	  6
  8	 N 	   466.68	   272.66	  5
  9	 A 	   502.20	   215.64	  4
 10	 A 	   537.72	   180.12	  3
 11	 L 	   594.26	   144.61	  2
 12	 R 	        -  	    88.06	  1  _
```

**39. IPI00018769 Thrombospondin-2**

| Sequence | Charge | M+H+ | XCorr | DeltaCn |
| --- | --- | --- | --- | --- |
| R.SCDVTSNTCLGPSIQTR.A | 2 | 1897.021 | 2.816 | 0.343 |

+1 charge

```
		      B   	       Y
  1	 S 	    88.04	        -  	 17
  2	 C 	   248.20	  1809.10	 16
  3	 D 	   363.23	  1648.93	 15
  4	 V 	   462.30	  1533.90	 14
  5	 T 	   563.35	  1434.84	 13
  6	 S 	   650.38	  1333.79	 12
  7	 N 	   764.42	  1246.76	 11
  8	 T 	   865.47	  1132.71	 10
  9	 C 	  1025.64	  1031.66	  9
 10	 L 	  1138.72	   871.50	  8
 11	 G 	  1195.74	   758.42	  7
 12	 P 	  1292.79	   701.39	  6
 13	 S 	  1379.83	   604.34	  5
 14	 I 	  1492.91	   517.31	  4
 15	 Q 	  1620.97	   404.23	  3
 16	 T 	  1722.02	   276.17	  2
 17	 R 	        -  	   175.12	  1  _
+2 charge
		      B   	       Y
  1	 S 	    44.52	        -  	 17
  2	 C 	   124.61	   905.05	 16
  3	 D 	   182.12	   824.97	 15
  4	 V 	   231.65	   767.46	 14
  5	 T 	   282.18	   717.92	 13
  6	 S 	   325.69	   667.40	 12
  7	 N 	   382.71	   623.88	 11
  8	 T 	   433.24	   566.86	 10
  9	 C 	   513.32	   516.34	  9
 10	 L 	   569.86	   436.25	  8
 11	 G 	   598.37	   379.71	  7
 12	 P 	   646.90	   351.20	  6
 13	 S 	   690.42	   302.67	  5
 14	 I 	   746.96	   259.16	  4
 15	 Q 	   810.99	   202.62	  3
 16	 T 	   861.51	   138.59	  2
 17	 R 	        -  	    88.06	  1  _
```

**40. IPI00219018 Glyceraldehyde-3-phosphate dehydrogenase**

| Sequence | Charge | M+H+ | XCorr | DeltaCn |
| --- | --- | --- | --- | --- |
| K.IISNASCTTNCLAPLAK.V | 2 | 1835.079 | 3.67 | 0.477 |

+1 charge

```
		      B   	       Y
  1	 I 	   114.09	        -  	 17
  2	 I 	   227.18	  1721.10	 16
  3	 S 	   314.21	  1608.02	 15
  4	 N 	   428.25	  1520.99	 14
  5	 A 	   499.29	  1406.95	 13
  6	 S 	   586.32	  1335.91	 12
  7	 C 	   746.48	  1248.88	 11
  8	 T 	   847.53	  1088.71	 10
  9	 T 	   948.58	   987.66	  9
 10	 N 	  1062.62	   886.62	  8
 11	 C 	  1222.79	   772.57	  7
 12	 L 	  1335.87	   612.41	  6
 13	 A 	  1406.91	   499.32	  5
 14	 P 	  1503.96	   428.29	  4
 15	 L 	  1617.05	   331.23	  3
 16	 A 	  1688.08	   218.15	  2
 17	 K 	        -  	   147.11	  1  _
+2 charge
		      B   	       Y
  1	 I 	    57.55	        -  	 17
  2	 I 	   114.09	   861.06	 16
  3	 S 	   157.61	   804.51	 15
  4	 N 	   214.63	   761.00	 14
  5	 A 	   250.15	   703.98	 13
  6	 S 	   293.66	   668.46	 12
  7	 C 	   373.75	   624.94	 11
  8	 T 	   424.27	   544.86	 10
  9	 T 	   474.79	   494.34	  9
 10	 N 	   531.82	   443.81	  8
 11	 C 	   611.90	   386.79	  7
 12	 L 	   668.44	   306.71	  6
 13	 A 	   703.96	   250.17	  5
 14	 P 	   752.48	   214.65	  4
 15	 L 	   809.03	   166.12	  3
 16	 A 	   844.55	   109.58	  2
 17	 K 	        -  	    74.06	  1  _
```

**41. IPI00099670 carboxyl ester lipase precursor**

| Sequence | Charge | M+H+ | XCorr | DeltaCn |
| --- | --- | --- | --- | --- |
| K.TYAYLFSHPSR.M | 2 | 1342.484 | 3.136 | 0.476 |

+1 charge

```
		      B   	       Y
  1	 T 	   102.05	        -  	 11
  2	 Y 	   265.12	  1240.61	 10
  3	 A 	   336.16	  1077.55	  9
  4	 Y 	   499.22	  1006.51	  8
  5	 L 	   612.30	   843.45	  7
  6	 F 	   759.37	   730.36	  6
  7	 S 	   846.40	   583.29	  5
  8	 H 	   983.46	   496.26	  4
  9	 P 	  1080.51	   359.20	  3
 10	 S 	  1167.55	   262.15	  2
 11	 R 	        -  	   175.12	  1  _
+2 charge
		      B   	       Y
  1	 T 	    51.53	        -  	 11
  2	 Y 	   133.06	   620.81	 10
  3	 A 	   168.58	   539.28	  9
  4	 Y 	   250.11	   503.76	  8
  5	 L 	   306.66	   422.23	  7
  6	 F 	   380.19	   365.69	  6
  7	 S 	   423.71	   292.15	  5
  8	 H 	   492.23	   248.63	  4
  9	 P 	   540.76	   180.11	  3
 10	 S 	   584.28	   131.58	  2
 11	 R 	        -  	    88.06	  1  _
```

**42. IPI00289831 Isoform PTPS of Receptor-type tyrosine-protein phosphatase S**

| Sequence | Charge | M+H+ | XCorr | DeltaCn |
| --- | --- | --- | --- | --- |
| R.SPQGLGAFTPVVR.Q | 2 | 1329.53 | 2.866 | 0.332 |

+1 charge

```
		      B   	       Y
  1	 S 	    88.04	        -  	 13
  2	 P 	   185.09	  1241.70	 12
  3	 Q 	   313.15	  1144.65	 11
  4	 G 	   370.17	  1016.59	 10
  5	 L 	   483.26	   959.57	  9
  6	 G 	   540.28	   846.48	  8
  7	 A 	   611.31	   789.46	  7
  8	 F 	   758.38	   718.42	  6
  9	 T 	   859.43	   571.36	  5
 10	 P 	   956.48	   470.31	  4
 11	 V 	  1055.55	   373.26	  3
 12	 V 	  1154.62	   274.19	  2
 13	 R 	        -  	   175.12	  1  _
+2 charge
		      B   	       Y
  1	 S 	    44.52	        -  	 13
  2	 P 	    93.05	   621.35	 12
  3	 Q 	   157.08	   572.83	 11
  4	 G 	   185.59	   508.80	 10
  5	 L 	   242.13	   480.29	  9
  6	 G 	   270.64	   423.75	  8
  7	 A 	   306.16	   395.23	  7
  8	 F 	   379.70	   359.72	  6
  9	 T 	   430.22	   286.18	  5
 10	 P 	   478.75	   235.66	  4
 11	 V 	   528.28	   187.13	  3
 12	 V 	   577.81	   137.60	  2
 13	 R 	        -  	    88.06	  1  _
```

**43. IPI00382488 Ig heavy chain V-III region HIL**

| Sequence | Charge | M+H+ | XCorr | DeltaCn |
| --- | --- | --- | --- | --- |
| - .QVKLVQAGGGVVQPGR.S | 2 | 1593.856 | 3.035 | 0.319 |

+1 charge

```
		      B   	       Y
  1	 Q 	   129.07	        -  	 16
  2	 V 	   228.13	  1464.86	 15
  3	 K 	   356.23	  1365.80	 14
  4	 L 	   469.31	  1237.70	 13
  5	 V 	   568.38	  1124.62	 12
  6	 Q 	   696.44	  1025.55	 11
  7	 A 	   767.48	   897.49	 10
  8	 G 	   824.50	   826.45	  9
  9	 G 	   881.52	   769.43	  8
 10	 G 	   938.54	   712.41	  7
 11	 V 	  1037.61	   655.39	  6
 12	 V 	  1136.68	   556.32	  5
 13	 Q 	  1264.74	   457.25	  4
 14	 P 	  1361.79	   329.19	  3
 15	 G 	  1418.81	   232.14	  2
 16	 R 	        -  	   175.12	  1  _
+2 charge
		      B   	       Y
  1	 Q 	    65.04	        -  	 16
  2	 V 	   114.57	   732.94	 15
  3	 K 	   178.62	   683.40	 14
  4	 L 	   235.16	   619.35	 13
  5	 V 	   284.69	   562.81	 12
  6	 Q 	   348.72	   513.28	 11
  7	 A 	   384.24	   449.25	 10
  8	 G 	   412.75	   413.73	  9
  9	 G 	   441.26	   385.22	  8
 10	 G 	   469.77	   356.71	  7
 11	 V 	   519.31	   328.20	  6
 12	 V 	   568.84	   278.66	  5
 13	 Q 	   632.87	   229.13	  4
 14	 P 	   681.40	   165.10	  3
 15	 G 	   709.91	   116.57	  2
 16	 R 	        -  	    88.06	  1  _
```

**44. IPI00385252 Ig kappa chain V-III region GOL**

| Sequence | Charge | M+H+ | XCorr | DeltaCn |
| --- | --- | --- | --- | --- |
| - .EIVLTQSPGTLSLSPGER.A | 2 | 1885.108 | 4.942 | 0.517 |

+1 charge

```
		      B   	       Y
  1	 E 	   130.05	        -  	 18
  2	 I 	   243.13	  1754.96	 17
  3	 V 	   342.20	  1641.88	 16
  4	 L 	   455.29	  1542.81	 15
  5	 T 	   556.33	  1429.73	 14
  6	 Q 	   684.39	  1328.68	 13
  7	 S 	   771.42	  1200.62	 12
  8	 P 	   868.48	  1113.59	 11
  9	 G 	   925.50	  1016.54	 10
 10	 T 	  1026.55	   959.52	  9
 11	 L 	  1139.63	   858.47	  8
 12	 S 	  1226.66	   745.38	  7
 13	 L 	  1339.75	   658.35	  6
 14	 S 	  1426.78	   545.27	  5
 15	 P 	  1523.83	   458.24	  4
 16	 G 	  1580.85	   361.18	  3
 17	 E 	  1709.90	   304.16	  2
 18	 R 	        -  	   175.12	  1  _
+2 charge
		      B   	       Y
  1	 E 	    65.53	        -  	 18
  2	 I 	   122.07	   877.99	 17
  3	 V 	   171.60	   821.44	 16
  4	 L 	   228.15	   771.91	 15
  5	 T 	   278.67	   715.37	 14
  6	 Q 	   342.70	   664.84	 13
  7	 S 	   386.22	   600.81	 12
  8	 P 	   434.74	   557.30	 11
  9	 G 	   463.25	   508.77	 10
 10	 T 	   513.78	   480.26	  9
 11	 L 	   570.32	   429.74	  8
 12	 S 	   613.83	   373.20	  7
 13	 L 	   670.38	   329.68	  6
 14	 S 	   713.89	   273.14	  5
 15	 P 	   762.42	   229.62	  4
 16	 G 	   790.93	   181.10	  3
 17	 E 	   855.45	   152.58	  2
 18	 R 	        -  	    88.06	  1  _
```

**45. IPI00382436 Ig lambda chain V-III region SH**

| Sequence | Charge | M+H+ | XCorr | DeltaCn |
| --- | --- | --- | --- | --- |
| - .SELTQDPAVSVALGQTVR.I | 2 | 1872.07 | 4.333 | 0.408 |

+1 charge

```
		      B   	       Y
  1	 S 	    88.04	        -  	 18
  2	 E 	   217.08	  1783.95	 17
  3	 L 	   330.17	  1654.91	 16
  4	 T 	   431.21	  1541.83	 15
  5	 Q 	   559.27	  1440.78	 14
  6	 D 	   674.30	  1312.72	 13
  7	 P 	   771.35	  1197.69	 12
  8	 A 	   842.39	  1100.64	 11
  9	 V 	   941.46	  1029.61	 10
 10	 S 	  1028.49	   930.54	  9
 11	 V 	  1127.56	   843.50	  8
 12	 A 	  1198.60	   744.44	  7
 13	 L 	  1311.68	   673.40	  6
 14	 G 	  1368.70	   560.32	  5
 15	 Q 	  1496.76	   503.29	  4
 16	 T 	  1597.81	   375.24	  3
 17	 V 	  1696.88	   274.19	  2
 18	 R 	        -  	   175.12	  1  _
+2 charge
		      B   	       Y
  1	 S 	    44.52	        -  	 18
  2	 E 	   109.04	   892.48	 17
  3	 L 	   165.59	   827.96	 16
  4	 T 	   216.11	   771.42	 15
  5	 Q 	   280.14	   720.89	 14
  6	 D 	   337.65	   656.86	 13
  7	 P 	   386.18	   599.35	 12
  8	 A 	   421.70	   550.82	 11
  9	 V 	   471.23	   515.31	 10
 10	 S 	   514.75	   465.77	  9
 11	 V 	   564.28	   422.26	  8
 12	 A 	   599.80	   372.72	  7
 13	 L 	   656.34	   337.20	  6
 14	 G 	   684.85	   280.66	  5
 15	 Q 	   748.88	   252.15	  4
 16	 T 	   799.41	   188.12	  3
 17	 V 	   848.94	   137.60	  2
 18	 R 	        -  	    88.06	  1  _
```

**46. IPI00879999 ATP-dependent RNA helicase DDX54**

| Sequence | Charge | M+H+ | XCorr | DeltaCn |
| --- | --- | --- | --- | --- |
| R.DQEFYIPYRPKDFDSER.G | 2 | 2206.355 | 3.46 | 0.296 |

+1 charge

```
		      B   	       Y
  1	 D 	   116.03	        -  	 17
  2	 Q 	   244.09	  2090.00	 16
  3	 E 	   373.14	  1961.94	 15
  4	 F 	   520.20	  1832.90	 14
  5	 Y 	   683.27	  1685.83	 13
  6	 I 	   796.35	  1522.76	 12
  7	 P 	   893.40	  1409.68	 11
  8	 Y 	  1056.47	  1312.63	 10
  9	 R 	  1212.57	  1149.56	  9
 10	 P 	  1309.62	   993.46	  8
 11	 K 	  1437.72	   896.41	  7
 12	 D 	  1552.74	   768.32	  6
 13	 F 	  1699.81	   653.29	  5
 14	 D 	  1814.84	   506.22	  4
 15	 S 	  1901.87	   391.19	  3
 16	 E 	  2030.91	   304.16	  2
 17	 R 	        -  	   175.12	  1  _
+2 charge
		      B   	       Y
  1	 D 	    58.52	        -  	 17
  2	 Q 	   122.55	  1045.50	 16
  3	 E 	   187.07	   981.47	 15
  4	 F 	   260.61	   916.95	 14
  5	 Y 	   342.14	   843.42	 13
  6	 I 	   398.68	   761.89	 12
  7	 P 	   447.21	   705.34	 11
  8	 Y 	   528.74	   656.82	 10
  9	 R 	   606.79	   575.29	  9
 10	 P 	   655.31	   497.24	  8
 11	 K 	   719.36	   448.71	  7
 12	 D 	   776.88	   384.66	  6
 13	 F 	   850.41	   327.15	  5
 14	 D 	   907.92	   253.61	  4
 15	 S 	   951.44	   196.10	  3
 16	 E 	  1015.96	   152.58	  2
 17	 R 	        -  	    88.06	  1  _
```

**47. IPI00000760 N(G),N(G)-dimethylarginine dimethylaminohydrolase 2**

| Sequence | Charge | M+H+ | XCorr | DeltaCn |
| --- | --- | --- | --- | --- |
| R.GVPESLASGEGAGAGLPALDLAK.A | 2 | 2081.313 | 4.07 | 0.579 |

+1 charge

```
		      B   	       Y
  1	 G 	    58.03	        -  	 23
  2	 V 	   157.10	  2023.07	 22
  3	 P 	   254.15	  1924.00	 21
  4	 E 	   383.19	  1826.95	 20
  5	 S 	   470.22	  1697.91	 19
  6	 L 	   583.31	  1610.87	 18
  7	 A 	   654.35	  1497.79	 17
  8	 S 	   741.38	  1426.75	 16
  9	 G 	   798.40	  1339.72	 15
 10	 E 	   927.44	  1282.70	 14
 11	 G 	   984.46	  1153.66	 13
 12	 A 	  1055.50	  1096.64	 12
 13	 G 	  1112.52	  1025.60	 11
 14	 A 	  1183.56	   968.58	 10
 15	 G 	  1240.58	   897.54	  9
 16	 L 	  1353.66	   840.52	  8
 17	 P 	  1450.72	   727.43	  7
 18	 A 	  1521.75	   630.38	  6
 19	 L 	  1634.84	   559.34	  5
 20	 D 	  1749.87	   446.26	  4
 21	 L 	  1862.95	   331.23	  3
 22	 A 	  1933.99	   218.15	  2
 23	 K 	        -  	   147.11	  1  _


+2 charge
		      B   	       Y
  1	 G 	    29.52	        -  	 23
  2	 V 	    79.05	  1012.04	 22
  3	 P 	   127.58	   962.50	 21
  4	 E 	   192.10	   913.98	 20
  5	 S 	   235.62	   849.46	 19
  6	 L 	   292.16	   805.94	 18
  7	 A 	   327.68	   749.40	 17
  8	 S 	   371.19	   713.88	 16
  9	 G 	   399.70	   670.36	 15
 10	 E 	   464.22	   641.85	 14
 11	 G 	   492.74	   577.33	 13
 12	 A 	   528.25	   548.82	 12
 13	 G 	   556.76	   513.30	 11
 14	 A 	   592.28	   484.79	 10
 15	 G 	   620.79	   449.27	  9
 16	 L 	   677.34	   420.76	  8
 17	 P 	   725.86	   364.22	  7
 18	 A 	   761.38	   315.69	  6
 19	 L 	   817.92	   280.18	  5
 20	 D 	   875.44	   223.63	  4
 21	 L 	   931.98	   166.12	  3
 22	 A 	   967.50	   109.58	  2
 23	 K 	        -  	    74.06	  1  _
```

**48. IPI00296777 SPARC-like protein 1**

| Sequence | Charge | M+H+ | XCorr | DeltaCn |
| --- | --- | --- | --- | --- |
| K.SIPTCTDFEVIQFPLR.M | 2 | 1924.18 | 3.03 | 0.439 |

+1 charge

```
		      B   	       Y
  1	 S 	    88.04	        -  	 16
  2	 I 	   201.12	  1836.07	 15
  3	 P 	   298.18	  1722.99	 14
  4	 T 	   399.22	  1625.93	 13
  5	 C 	   559.39	  1524.89	 12
  6	 T 	   660.44	  1364.72	 11
  7	 D 	   775.46	  1263.67	 10
  8	 F 	   922.53	  1148.65	  9
  9	 E 	  1051.57	  1001.58	  8
 10	 V 	  1150.64	   872.54	  7
 11	 I 	  1263.73	   773.47	  6
 12	 Q 	  1391.79	   660.38	  5
 13	 F 	  1538.85	   532.32	  4
 14	 P 	  1635.91	   385.26	  3
 15	 L 	  1748.99	   288.20	  2
 16	 R 	        -  	   175.12	  1  _
+2 charge
		      B   	       Y
  1	 S 	    44.52	        -  	 16
  2	 I 	   101.07	   918.54	 15
  3	 P 	   149.59	   862.00	 14
  4	 T 	   200.12	   813.47	 13
  5	 C 	   280.20	   762.95	 12
  6	 T 	   330.72	   682.86	 11
  7	 D 	   388.24	   632.34	 10
  8	 F 	   461.77	   574.83	  9
  9	 E 	   526.29	   501.29	  8
 10	 V 	   575.83	   436.77	  7
 11	 I 	   632.37	   387.24	  6
 12	 Q 	   696.40	   330.70	  5
 13	 F 	   769.93	   266.67	  4
 14	 P 	   818.46	   193.13	  3
 15	 L 	   875.00	   144.61	  2
 16	 R 	        -  	    88.06	  1  _
```

**49. IPI00794461 Histone H2B type 1-N**

| Sequence | Charge | M+H+ | XCorr | DeltaCn |
| --- | --- | --- | --- | --- |
| K.AMGIMNSFVNDIFER.I | 2 | 1745.017 | 4.956 | 0.621 |

+1 charge

```
		      B   	       Y
  1	 A 	    72.04	        -  	 15
  2	 M 	   203.08	  1672.78	 14
  3	 G 	   260.11	  1541.74	 13
  4	 I 	   373.19	  1484.72	 12
  5	 M 	   504.23	  1371.64	 11
  6	 N 	   618.27	  1240.60	 10
  7	 S 	   705.31	  1126.55	  9
  8	 F 	   852.37	  1039.52	  8
  9	 V 	   951.44	   892.45	  7
 10	 N 	  1065.49	   793.38	  6
 11	 D 	  1180.51	   679.34	  5
 12	 I 	  1293.60	   564.31	  4
 13	 F 	  1440.67	   451.23	  3
 14	 E 	  1569.71	   304.16	  2
 15	 R 	        -  	   175.12	  1  _
+2 charge
		      B   	       Y
  1	 A 	    36.53	        -  	 15
  2	 M 	   102.05	   836.89	 14
  3	 G 	   130.56	   771.37	 13
  4	 I 	   187.10	   742.86	 12
  5	 M 	   252.62	   686.32	 11
  6	 N 	   309.64	   620.80	 10
  7	 S 	   353.16	   563.78	  9
  8	 F 	   426.69	   520.26	  8
  9	 V 	   476.22	   446.73	  7
 10	 N 	   533.25	   397.20	  6
 11	 D 	   590.76	   340.17	  5
 12	 I 	   647.30	   282.66	  4
 13	 F 	   720.84	   226.12	  3
 14	 E 	   785.36	   152.58	  2
 15	 R 	        -  	    88.06	  1  _
```

**50. IPI00386747 Isoform 2 of Codanin-1**

| Sequence | Charge | M+H+ | XCorr | DeltaCn |
| --- | --- | --- | --- | --- |
| R.STQGSEDNAGEAAALSSLRALRK.E | 3 | 2333.502 | 3.454 | 0.402 |

+1 charge

```
		      B   	       Y
  1	 S 	    88.04	        -  	 23
  2	 T 	   189.09	  2245.15	 22
  3	 Q 	   317.15	  2144.11	 21
  4	 G 	   374.17	  2016.05	 20
  5	 S 	   461.20	  1959.03	 19
  6	 E 	   590.24	  1871.99	 18
  7	 D 	   705.27	  1742.95	 17
  8	 N 	   819.31	  1627.92	 16
  9	 A 	   890.35	  1513.88	 15
 10	 G 	   947.37	  1442.84	 14
 11	 E 	  1076.41	  1385.82	 13
 12	 A 	  1147.45	  1256.78	 12
 13	 A 	  1218.49	  1185.74	 11
 14	 A 	  1289.52	  1114.71	 10
 15	 L 	  1402.61	  1043.67	  9
 16	 S 	  1489.64	   930.58	  8
 17	 S 	  1576.67	   843.55	  7
 18	 L 	  1689.76	   756.52	  6
 19	 R 	  1845.86	   643.44	  5
 20	 A 	  1916.89	   487.34	  4
 21	 L 	  2029.98	   416.30	  3
 22	 R 	  2186.08	   303.21	  2
 23	 K 	        -  	   147.11	  1  _


+2 charge
		      B   	       Y
  1	 S 	    44.52	        -  	 23
  2	 T 	    95.05	  1123.08	 22
  3	 Q 	   159.08	  1072.56	 21
  4	 G 	   187.59	  1008.53	 20
  5	 S 	   231.10	   980.02	 19
  6	 E 	   295.62	   936.50	 18
  7	 D 	   353.14	   871.98	 17
  8	 N 	   410.16	   814.47	 16
  9	 A 	   445.68	   757.44	 15
 10	 G 	   474.19	   721.93	 14
 11	 E 	   538.71	   693.41	 13
 12	 A 	   574.23	   628.89	 12
 13	 A 	   609.75	   593.37	 11
 14	 A 	   645.27	   557.86	 10
 15	 L 	   701.81	   522.34	  9
 16	 S 	   745.32	   465.80	  8
 17	 S 	   788.84	   422.28	  7
 18	 L 	   845.38	   378.76	  6
 19	 R 	   923.43	   322.22	  5
 20	 A 	   958.95	   244.17	  4
 21	 L 	  1015.49	   208.65	  3
 22	 R 	  1093.54	   152.11	  2
 23	 K 	        -  	    74.06	  1  _


+3 charge
		      B   	       Y
  1	 S 	    30.02	        -  	 23
  2	 T 	    63.70	   749.06	 22
  3	 Q 	   106.39	   715.37	 21
  4	 G 	   125.39	   672.69	 20
  5	 S 	   154.40	   653.68	 19
  6	 E 	   197.42	   624.67	 18
  7	 D 	   235.76	   581.66	 17
  8	 N 	   273.78	   543.31	 16
  9	 A 	   297.45	   505.30	 15
 10	 G 	   316.46	   481.62	 14
 11	 E 	   359.48	   462.61	 13
 12	 A 	   383.15	   419.60	 12
 13	 A 	   406.83	   395.92	 11
 14	 A 	   430.51	   372.24	 10
 15	 L 	   468.21	   348.56	  9
 16	 S 	   497.22	   310.87	  8
 17	 S 	   526.23	   281.86	  7
 18	 L 	   563.92	   252.84	  6
 19	 R 	   615.96	   215.15	  5
 20	 A 	   639.64	   163.12	  4
 21	 L 	   677.33	   139.44	  3
 22	 R 	   729.36	   101.74	  2
 23	 K 	        -  	    49.71	  1  _
```

**51. IPI00297910 Tumor-associated calcium signal transducer 2**

| Sequence | Charge | M+H+ | XCorr | DeltaCn |
| --- | --- | --- | --- | --- |
| K.MTVCSPDGPGGR.C | 2 | 1234.359 | 3.008 | 0.444 |

+1 charge

```
		      B   	       Y
  1	 M 	   132.05	        -  	 12
  2	 T 	   233.10	  1102.63	 11
  3	 V 	   332.16	  1001.58	 10
  4	 C 	   492.33	   902.51	  9
  5	 S 	   579.36	   742.35	  8
  6	 P 	   676.41	   655.32	  7
  7	 D 	   791.44	   558.26	  6
  8	 G 	   848.46	   443.24	  5
  9	 P 	   945.52	   386.21	  4
 10	 G 	  1002.54	   289.16	  3
 11	 G 	  1059.56	   232.14	  2
 12	 R 	        -  	   175.12	  1  _
+2 charge
		      B   	       Y
  1	 M 	    66.53	        -  	 12
  2	 T 	   117.05	   551.82	 11
  3	 V 	   166.59	   501.29	 10
  4	 C 	   246.67	   451.76	  9
  5	 S 	   290.18	   371.68	  8
  6	 P 	   338.71	   328.16	  7
  7	 D 	   396.22	   279.64	  6
  8	 G 	   424.73	   222.12	  5
  9	 P 	   473.26	   193.61	  4
 10	 G 	   501.77	   145.08	  3
 11	 G 	   530.28	   116.57	  2
 12	 R 	        -  	    88.06	  1  _
```

**52. IPI00023501 Isoform 1 of Tumor necrosis factor receptor superfamily member 1B**

| Sequence | Charge | M+H+ | XCorr | DeltaCn |
| --- | --- | --- | --- | --- |
| R.LREYYDQTAQMCCSK.C | 2 | 1954.138 | 2.895 | 0.306 |

+1 charge

```
		      B   	       Y
  1	 L 	   114.09	        -  	 15
  2	 R 	   270.19	  1840.02	 14
  3	 E 	   399.24	  1683.91	 13
  4	 Y 	   562.30	  1554.87	 12
  5	 Y 	   725.36	  1391.81	 11
  6	 D 	   840.39	  1228.74	 10
  7	 Q 	   968.45	  1113.72	  9
  8	 T 	  1069.49	   985.66	  8
  9	 A 	  1140.53	   884.61	  7
 10	 Q 	  1268.59	   813.57	  6
 11	 M 	  1399.63	   685.52	  5
 12	 C 	  1559.80	   554.48	  4
 13	 C 	  1719.96	   394.31	  3
 14	 S 	  1806.99	   234.14	  2
 15	 K 	        -  	   147.11	  1  _
+2 charge
		      B   	       Y
  1	 L 	    57.55	        -  	 15
  2	 R 	   135.60	   920.51	 14
  3	 E 	   200.12	   842.46	 13
  4	 Y 	   281.65	   777.94	 12
  5	 Y 	   363.18	   696.41	 11
  6	 D 	   420.70	   614.88	 10
  7	 Q 	   484.73	   557.36	  9
  8	 T 	   535.25	   493.33	  8
  9	 A 	   570.77	   442.81	  7
 10	 Q 	   634.80	   407.29	  6
 11	 M 	   700.32	   343.26	  5
 12	 C 	   780.40	   277.74	  4
 13	 C 	   860.48	   197.66	  3
 14	 S 	   904.00	   117.58	  2
 15	 K 	        -  	    74.06	  1  _
```

**53. IPI00386839 Amyloid lambda 6 light chain variable region SAR (Fragment)**

| Sequence | Charge | M+H+ | XCorr | DeltaCn |
| --- | --- | --- | --- | --- |
| - .NFMLTQPHSVSESPGK.T | 2 | 1759.965 | 2.834 | 0.397 |

+1 charge

```
		      B   	       Y
  1	 N 	   115.05	        -  	 16
  2	 F 	   262.12	  1644.81	 15
  3	 M 	   393.16	  1497.74	 14
  4	 L 	   506.24	  1366.70	 13
  5	 T 	   607.29	  1253.61	 12
  6	 Q 	   735.35	  1152.56	 11
  7	 P 	   832.40	  1024.51	 10
  8	 H 	   969.46	   927.45	  9
  9	 S 	  1056.49	   790.39	  8
 10	 V 	  1155.56	   703.36	  7
 11	 S 	  1242.59	   604.29	  6
 12	 E 	  1371.64	   517.26	  5
 13	 S 	  1458.67	   388.22	  4
 14	 P 	  1555.72	   301.19	  3
 15	 G 	  1612.74	   204.13	  2
 16	 K 	        -  	   147.11	  1  _
+2 charge
		      B   	       Y
  1	 N 	    58.03	        -  	 16
  2	 F 	   131.56	   822.91	 15
  3	 M 	   197.08	   749.37	 14
  4	 L 	   253.63	   683.85	 13
  5	 T 	   304.15	   627.31	 12
  6	 Q 	   368.18	   576.79	 11
  7	 P 	   416.70	   512.76	 10
  8	 H 	   485.23	   464.23	  9
  9	 S 	   528.75	   395.70	  8
 10	 V 	   578.28	   352.18	  7
 11	 S 	   621.80	   302.65	  6
 12	 E 	   686.32	   259.13	  5
 13	 S 	   729.84	   194.61	  4
 14	 P 	   778.36	   151.10	  3
 15	 G 	   806.87	   102.57	  2
 16	 K 	        -  	    74.06	  1  _
```

**54. IPI00939667 Isoform 1 of Tripeptidyl-peptidase 1**

| Sequence | Charge | M+H+ | XCorr | DeltaCn |
| --- | --- | --- | --- | --- |
| R.LFGGNFAHQASVAR.V | 2 | 1475.636 | 4.553 | 0.522 |

+1 charge

```
		      B   	       Y
  1	 L 	   114.09	        -  	 14
  2	 F 	   261.16	  1361.67	 13
  3	 G 	   318.18	  1214.60	 12
  4	 G 	   375.20	  1157.58	 11
  5	 N 	   489.25	  1100.56	 10
  6	 F 	   636.31	   986.52	  9
  7	 A 	   707.35	   839.45	  8
  8	 H 	   844.41	   768.41	  7
  9	 Q 	   972.47	   631.35	  6
 10	 A 	  1043.51	   503.29	  5
 11	 S 	  1130.54	   432.26	  4
 12	 V 	  1229.61	   345.22	  3
 13	 A 	  1300.64	   246.16	  2
 14	 R 	        -  	   175.12	  1  _
+2 charge
		      B   	       Y
  1	 L 	    57.55	        -  	 14
  2	 F 	   131.08	   681.34	 13
  3	 G 	   159.59	   607.80	 12
  4	 G 	   188.10	   579.29	 11
  5	 N 	   245.13	   550.78	 10
  6	 F 	   318.66	   493.76	  9
  7	 A 	   354.18	   420.23	  8
  8	 H 	   422.71	   384.71	  7
  9	 Q 	   486.74	   316.18	  6
 10	 A 	   522.26	   252.15	  5
 11	 S 	   565.77	   216.63	  4
 12	 V 	   615.31	   173.12	  3
 13	 A 	   650.83	   123.58	  2
 14	 R 	        -  	    88.06	  1  _
```

**55. IPI00816555 similar to Ig lambda chain**

| Sequence | Charge | M+H+ | XCorr | DeltaCn |
| --- | --- | --- | --- | --- |
| K.LMIYDVSNRPSGVSNR.F | 2 | 1809.04 | 3.15 | 0.251 |

+1 charge

```
		      B   	       Y
  1	 L 	   114.09	        -  	 16
  2	 M 	   245.13	  1694.83	 15
  3	 I 	   358.22	  1563.79	 14
  4	 Y 	   521.28	  1450.70	 13
  5	 D 	   636.31	  1287.64	 12
  6	 V 	   735.37	  1172.61	 11
  7	 S 	   822.41	  1073.54	 10
  8	 N 	   936.45	   986.51	  9
  9	 R 	  1092.55	   872.47	  8
 10	 P 	  1189.60	   716.37	  7
 11	 S 	  1276.64	   619.32	  6
 12	 G 	  1333.66	   532.28	  5
 13	 V 	  1432.73	   475.26	  4
 14	 S 	  1519.76	   376.19	  3
 15	 N 	  1633.80	   289.16	  2
 16	 R 	        -  	   175.12	  1  _
+2 charge
		      B   	       Y
  1	 L 	    57.55	        -  	 16
  2	 M 	   123.07	   847.92	 15
  3	 I 	   179.61	   782.40	 14
  4	 Y 	   261.14	   725.86	 13
  5	 D 	   318.66	   644.32	 12
  6	 V 	   368.19	   586.81	 11
  7	 S 	   411.71	   537.28	 10
  8	 N 	   468.73	   493.76	  9
  9	 R 	   546.78	   436.74	  8
 10	 P 	   595.31	   358.69	  7
 11	 S 	   638.82	   310.16	  6
 12	 G 	   667.33	   266.65	  5
 13	 V 	   716.87	   238.13	  4
 14	 S 	   760.38	   188.60	  3
 15	 N 	   817.40	   145.08	  2
 16	 R 	        -  	    88.06	  1  _
```

**56. IPI00294713 Isoform 1 of Mannan-binding lectin serine protease 2**

| Sequence | Charge | M+H+ | XCorr | DeltaCn |
| --- | --- | --- | --- | --- |
| R.LASPGFPGEYANDQER.R | 2 | 1751.834 | 3.794 | 0.53 |

+1 charge

```
		      B   	       Y
  1	 L 	   114.09	        -  	 16
  2	 A 	   185.13	  1637.72	 15
  3	 S 	   272.16	  1566.68	 14
  4	 P 	   369.21	  1479.65	 13
  5	 G 	   426.23	  1382.60	 12
  6	 F 	   573.30	  1325.58	 11
  7	 P 	   670.36	  1178.51	 10
  8	 G 	   727.38	  1081.45	  9
  9	 E 	   856.42	  1024.43	  8
 10	 Y 	  1019.48	   895.39	  7
 11	 A 	  1090.52	   732.33	  6
 12	 N 	  1204.56	   661.29	  5
 13	 D 	  1319.59	   547.25	  4
 14	 Q 	  1447.65	   432.22	  3
 15	 E 	  1576.69	   304.16	  2
 16	 R 	        -  	   175.12	  1  _
+2 charge
		      B   	       Y
  1	 L 	    57.55	        -  	 16
  2	 A 	    93.07	   819.36	 15
  3	 S 	   136.58	   783.84	 14
  4	 P 	   185.11	   740.33	 13
  5	 G 	   213.62	   691.80	 12
  6	 F 	   287.16	   663.29	 11
  7	 P 	   335.68	   589.76	 10
  8	 G 	   364.19	   541.23	  9
  9	 E 	   428.71	   512.72	  8
 10	 Y 	   510.25	   448.20	  7
 11	 A 	   545.76	   366.67	  6
 12	 N 	   602.79	   331.15	  5
 13	 D 	   660.30	   274.13	  4
 14	 Q 	   724.33	   216.61	  3
 15	 E 	   788.85	   152.58	  2
 16	 R 	        -  	    88.06	  1  _
```

**57. IPI00001952 Endonuclease domain-containing 1 protein**

| Sequence | Charge | M+H+ | XCorr | DeltaCn |
| --- | --- | --- | --- | --- |
| K.ILEVVNQIQDEER.M | 2 | 1585.741 | 4.399 | 0.472 |

+1 charge

```
		      B   	       Y
  1	 I 	   114.09	        -  	 13
  2	 L 	   227.18	  1471.74	 12
  3	 E 	   356.22	  1358.65	 11
  4	 V 	   455.29	  1229.61	 10
  5	 V 	   554.35	  1130.54	  9
  6	 N 	   668.40	  1031.48	  8
  7	 Q 	   796.46	   917.43	  7
  8	 I 	   909.54	   789.37	  6
  9	 Q 	  1037.60	   676.29	  5
 10	 D 	  1152.63	   548.23	  4
 11	 E 	  1281.67	   433.20	  3
 12	 E 	  1410.71	   304.16	  2
 13	 R 	        -  	   175.12	  1  _
+2 charge
		      B   	       Y
  1	 I 	    57.55	        -  	 13
  2	 L 	   114.09	   736.37	 12
  3	 E 	   178.61	   679.83	 11
  4	 V 	   228.15	   615.31	 10
  5	 V 	   277.68	   565.78	  9
  6	 N 	   334.70	   516.24	  8
  7	 Q 	   398.73	   459.22	  7
  8	 I 	   455.27	   395.19	  6
  9	 Q 	   519.30	   338.65	  5
 10	 D 	   576.82	   274.62	  4
 11	 E 	   641.34	   217.11	  3
 12	 E 	   705.86	   152.58	  2
 13	 R 	        -  	    88.06	  1  _
```

**58. IPI00829834 Ig kappa chain V-III region VH (Fragment)**

| Sequence | Charge | M+H+ | XCorr | DeltaCn |
| --- | --- | --- | --- | --- |
| R.EIVMTQSPPTLSLSPGER.V | 2 | 1943.211 | 3.963 | 0.381 |

+1 charge

```
		      B   	       Y
  1	 E 	   130.05	        -  	 18
  2	 I 	   243.13	  1812.95	 17
  3	 V 	   342.20	  1699.87	 16
  4	 M 	   473.24	  1600.80	 15
  5	 T 	   574.29	  1469.76	 14
  6	 Q 	   702.35	  1368.71	 13
  7	 S 	   789.38	  1240.65	 12
  8	 P 	   886.43	  1153.62	 11
  9	 P 	   983.49	  1056.57	 10
 10	 T 	  1084.53	   959.52	  9
 11	 L 	  1197.62	   858.47	  8
 12	 S 	  1284.65	   745.38	  7
 13	 L 	  1397.73	   658.35	  6
 14	 S 	  1484.77	   545.27	  5
 15	 P 	  1581.82	   458.24	  4
 16	 G 	  1638.84	   361.18	  3
 17	 E 	  1767.88	   304.16	  2
 18	 R 	        -  	   175.12	  1  _
+2 charge
		      B   	       Y
  1	 E 	    65.53	        -  	 18
  2	 I 	   122.07	   906.98	 17
  3	 V 	   171.60	   850.44	 16
  4	 M 	   237.13	   800.90	 15
  5	 T 	   287.65	   735.38	 14
  6	 Q 	   351.68	   684.86	 13
  7	 S 	   395.19	   620.83	 12
  8	 P 	   443.72	   577.31	 11
  9	 P 	   492.25	   528.79	 10
 10	 T 	   542.77	   480.26	  9
 11	 L 	   599.31	   429.74	  8
 12	 S 	   642.83	   373.20	  7
 13	 L 	   699.37	   329.68	  6
 14	 S 	   742.89	   273.14	  5
 15	 P 	   791.41	   229.62	  4
 16	 G 	   819.92	   181.10	  3
 17	 E 	   884.45	   152.58	  2
 18	 R 	        -  	    88.06	  1  _
```

**59. IPI00336112 Isoform 1 of Mucin and cadherin-like protein**

| Sequence | Charge | M+H+ | XCorr | DeltaCn |
| --- | --- | --- | --- | --- |
| R.IQAQDPEFSDLNSAITYR.I | 2 | 2069.217 | 2.877 | 0.313 |

+1 charge

```
		      B   	       Y
  1	 I 	   114.09	        -  	 18
  2	 Q 	   242.15	  1954.91	 17
  3	 A 	   313.19	  1826.86	 16
  4	 Q 	   441.25	  1755.82	 15
  5	 D 	   556.27	  1627.76	 14
  6	 P 	   653.33	  1512.73	 13
  7	 E 	   782.37	  1415.68	 12
  8	 F 	   929.44	  1286.64	 11
  9	 S 	  1016.47	  1139.57	 10
 10	 D 	  1131.50	  1052.54	  9
 11	 L 	  1244.58	   937.51	  8
 12	 N 	  1358.62	   824.43	  7
 13	 S 	  1445.65	   710.38	  6
 14	 A 	  1516.69	   623.35	  5
 15	 I 	  1629.78	   552.31	  4
 16	 T 	  1730.82	   439.23	  3
 17	 Y 	  1893.89	   338.18	  2
 18	 R 	        -  	   175.12	  1  _
+2 charge
		      B   	       Y
  1	 I 	    57.55	        -  	 18
  2	 Q 	   121.58	   977.96	 17
  3	 A 	   157.10	   913.93	 16
  4	 Q 	   221.13	   878.41	 15
  5	 D 	   278.64	   814.38	 14
  6	 P 	   327.17	   756.87	 13
  7	 E 	   391.69	   708.34	 12
  8	 F 	   465.22	   643.82	 11
  9	 S 	   508.74	   570.29	 10
 10	 D 	   566.25	   526.77	  9
 11	 L 	   622.79	   469.26	  8
 12	 N 	   679.81	   412.72	  7
 13	 S 	   723.33	   355.70	  6
 14	 A 	   758.85	   312.18	  5
 15	 I 	   815.39	   276.66	  4
 16	 T 	   865.92	   220.12	  3
 17	 Y 	   947.45	   169.59	  2
 18	 R 	        -  	    88.06	  1  _
```

**60. IPI00013438 Immunoglobulin lambda-like polypeptide 1**

| Sequence | Charge | M+H+ | XCorr | DeltaCn |
| --- | --- | --- | --- | --- |
| R.SYSCQVMHEGSTVEK.T | 2 | 1742.883 | 4.03 | 0.417 |

+1 charge

```
		      B   	       Y
  1	 S 	    88.04	        -  	 15
  2	 Y 	   251.10	  1654.85	 14
  3	 S 	   338.13	  1491.79	 13
  4	 C 	   498.30	  1404.76	 12
  5	 Q 	   626.36	  1244.59	 11
  6	 V 	   725.43	  1116.54	 10
  7	 M 	   856.47	  1017.47	  9
  8	 H 	   993.53	   886.43	  8
  9	 E 	  1122.57	   749.37	  7
 10	 G 	  1179.59	   620.32	  6
 11	 S 	  1266.62	   563.30	  5
 12	 T 	  1367.67	   476.27	  4
 13	 V 	  1466.74	   375.22	  3
 14	 E 	  1595.78	   276.16	  2
 15	 K 	        -  	   147.11	  1  _
+2 charge
		      B   	       Y
  1	 S 	    44.52	        -  	 15
  2	 Y 	   126.05	   827.93	 14
  3	 S 	   169.57	   746.40	 13
  4	 C 	   249.65	   702.88	 12
  5	 Q 	   313.68	   622.80	 11
  6	 V 	   363.22	   558.77	 10
  7	 M 	   428.74	   509.24	  9
  8	 H 	   497.27	   443.72	  8
  9	 E 	   561.79	   375.19	  7
 10	 G 	   590.30	   310.67	  6
 11	 S 	   633.81	   282.16	  5
 12	 T 	   684.34	   238.64	  4
 13	 V 	   733.87	   188.12	  3
 14	 E 	   798.39	   138.58	  2
 15	 K 	        -  	    74.06	  1  _
```

**61. IPI00433028 Isoform 1 of Leukocyte-associated immunoglobulin-like receptor 1**

| Sequence | Charge | M+H+ | XCorr | DeltaCn |
| --- | --- | --- | --- | --- |
| R.FRIDSVSEGNAGPYR.C | 2 | 1668.792 | 4.448 | 0.432 |

+1 charge

```
		      B   	       Y
  1	 F 	   148.08	        -  	 15
  2	 R 	   304.18	  1520.75	 14
  3	 I 	   417.26	  1364.64	 13
  4	 D 	   532.29	  1251.56	 12
  5	 S 	   619.32	  1136.53	 11
  6	 V 	   718.39	  1049.50	 10
  7	 S 	   805.42	   950.43	  9
  8	 E 	   934.46	   863.40	  8
  9	 G 	   991.48	   734.36	  7
 10	 N 	  1105.53	   677.34	  6
 11	 A 	  1176.56	   563.29	  5
 12	 G 	  1233.59	   492.26	  4
 13	 P 	  1330.64	   435.24	  3
 14	 Y 	  1493.70	   338.18	  2
 15	 R 	        -  	   175.12	  1  _
+2 charge
		      B   	       Y
  1	 F 	    74.54	        -  	 15
  2	 R 	   152.59	   760.88	 14
  3	 I 	   209.13	   682.83	 13
  4	 D 	   266.65	   626.28	 12
  5	 S 	   310.16	   568.77	 11
  6	 V 	   359.70	   525.25	 10
  7	 S 	   403.21	   475.72	  9
  8	 E 	   467.74	   432.20	  8
  9	 G 	   496.25	   367.68	  7
 10	 N 	   553.27	   339.17	  6
 11	 A 	   588.79	   282.15	  5
 12	 G 	   617.30	   246.63	  4
 13	 P 	   665.82	   218.12	  3
 14	 Y 	   747.35	   169.59	  2
 15	 R 	        -  	    88.06	  1  _
```

**62. IPI00018279 Collagen alpha-3(V) chain**

| Sequence | Charge | M+H+ | XCorr | DeltaCn |
| --- | --- | --- | --- | --- |
| K.ALGVQGGQAGVPEGPGFCPQR.T | 2 | 2083.287 | 2.942 | 0.504 |

+1 charge

```
		      B   	       Y
  1	 A 	    72.04	        -  	 21
  2	 L 	   185.13	  2011.12	 20
  3	 G 	   242.15	  1898.03	 19
  4	 V 	   341.22	  1841.01	 18
  5	 Q 	   469.28	  1741.94	 17
  6	 G 	   526.30	  1613.88	 16
  7	 G 	   583.32	  1556.86	 15
  8	 Q 	   711.38	  1499.84	 14
  9	 A 	   782.42	  1371.78	 13
 10	 G 	   839.44	  1300.74	 12
 11	 V 	   938.51	  1243.72	 11
 12	 P 	  1035.56	  1144.65	 10
 13	 E 	  1164.60	  1047.60	  9
 14	 G 	  1221.62	   918.56	  8
 15	 P 	  1318.67	   861.54	  7
 16	 G 	  1375.70	   764.49	  6
 17	 F 	  1522.76	   707.46	  5
 18	 C 	  1682.93	   560.40	  4
 19	 P 	  1779.98	   400.23	  3
 20	 Q 	  1908.04	   303.18	  2
 21	 R 	        -  	   175.12	  1  _


+2 charge
		      B   	       Y
  1	 A 	    36.53	        -  	 21
  2	 L 	    93.07	  1006.06	 20
  3	 G 	   121.58	   949.52	 19
  4	 V 	   171.11	   921.01	 18
  5	 Q 	   235.14	   871.47	 17
  6	 G 	   263.65	   807.45	 16
  7	 G 	   292.16	   778.93	 15
  8	 Q 	   356.19	   750.42	 14
  9	 A 	   391.71	   686.39	 13
 10	 G 	   420.22	   650.88	 12
 11	 V 	   469.76	   622.37	 11
 12	 P 	   518.28	   572.83	 10
 13	 E 	   582.80	   524.30	  9
 14	 G 	   611.31	   459.78	  8
 15	 P 	   659.84	   431.27	  7
 16	 G 	   688.35	   382.75	  6
 17	 F 	   761.89	   354.24	  5
 18	 C 	   841.97	   280.70	  4
 19	 P 	   890.50	   200.62	  3
 20	 Q 	   954.52	   152.09	  2
 21	 R 	        -  	    88.06	  1  _
```

**63. IPI00387113 Ig kappa chain V-III region B6**

| Sequence | Charge | M+H+ | XCorr | DeltaCn |
| --- | --- | --- | --- | --- |
| - .ZIVLTZSPGTLSLSPGZR.A | 2 | 1884.616 | 3.952 | 0.611 |

+1 charge

```
		      B   	       Y
  1	 Z 	   129.56	        -  	 18
  2	 I 	   242.64	  1754.96	 17
  3	 V 	   341.71	  1641.88	 16
  4	 L 	   454.79	  1542.81	 15
  5	 T 	   555.84	  1429.73	 14
  6	 Z 	   684.39	  1328.68	 13
  7	 S 	   771.42	  1200.13	 12
  8	 P 	   868.48	  1113.10	 11
  9	 G 	   925.50	  1016.05	 10
 10	 T 	  1026.55	   959.02	  9
 11	 L 	  1139.63	   857.98	  8
 12	 S 	  1226.66	   744.89	  7
 13	 L 	  1339.75	   657.86	  6
 14	 S 	  1426.78	   544.78	  5
 15	 P 	  1523.83	   457.74	  4
 16	 G 	  1580.85	   360.69	  3
 17	 Z 	  1709.40	   303.67	  2
 18	 R 	        -  	   175.12	  1  _
+2 charge
		      B   	       Y
  1	 Z 	    65.28	        -  	 18
  2	 I 	   121.82	   877.99	 17
  3	 V 	   171.36	   821.44	 16
  4	 L 	   227.90	   771.91	 15
  5	 T 	   278.42	   715.37	 14
  6	 Z 	   342.70	   664.84	 13
  7	 S 	   386.22	   600.57	 12
  8	 P 	   434.74	   557.05	 11
  9	 G 	   463.25	   508.53	 10
 10	 T 	   513.78	   480.02	  9
 11	 L 	   570.32	   429.49	  8
 12	 S 	   613.83	   372.95	  7
 13	 L 	   670.38	   329.43	  6
 14	 S 	   713.89	   272.89	  5
 15	 P 	   762.42	   229.38	  4
 16	 G 	   790.93	   180.85	  3
 17	 Z 	   855.21	   152.34	  2
 18	 R 	        -  	    88.06	  1  _
```

**64. IPI00006957 Isoform 1 of Dehydrogenase/reductase SDR family member 7**

| Sequence | Charge | M+H+ | XCorr | DeltaCn |
| --- | --- | --- | --- | --- |
| K.LIELNYLGTVSLTKCVLPHMIERK.Q | 3 | 2829.388 | 3.508 | 0.214 |

+1 charge

```
		      B   	       Y
  1	 L 	   114.09	        -  	 24
  2	 I 	   227.18	  2714.61	 23
  3	 E 	   356.22	  2601.52	 22
  4	 L 	   469.30	  2472.48	 21
  5	 N 	   583.34	  2359.40	 20
  6	 Y 	   746.41	  2245.35	 19
  7	 L 	   859.49	  2082.29	 18
  8	 G 	   916.51	  1969.21	 17
  9	 T 	  1017.56	  1912.19	 16
 10	 V 	  1116.63	  1811.14	 15
 11	 S 	  1203.66	  1712.07	 14
 12	 L 	  1316.75	  1625.04	 13
 13	 T 	  1417.79	  1511.95	 12
 14	 K 	  1545.89	  1410.91	 11
 15	 C 	  1706.05	  1282.81	 10
 16	 V 	  1805.12	  1122.65	  9
 17	 L 	  1918.21	  1023.58	  8
 18	 P 	  2015.26	   910.49	  7
 19	 H 	  2152.32	   813.44	  6
 20	 M 	  2283.36	   676.38	  5
 21	 I 	  2396.44	   545.34	  4
 22	 E 	  2525.49	   432.26	  3
 23	 R 	  2681.59	   303.21	  2
 24	 K 	        -  	   147.11	  1  _


+2 charge
		      B   	       Y
  1	 L 	    57.55	        -  	 24
  2	 I 	   114.09	  1357.81	 23
  3	 E 	   178.61	  1301.27	 22
  4	 L 	   235.15	  1236.74	 21
  5	 N 	   292.18	  1180.20	 20
  6	 Y 	   373.71	  1123.18	 19
  7	 L 	   430.25	  1041.65	 18
  8	 G 	   458.76	   985.11	 17
  9	 T 	   509.28	   956.60	 16
 10	 V 	   558.82	   906.07	 15
 11	 S 	   602.33	   856.54	 14
 12	 L 	   658.88	   813.02	 13
 13	 T 	   709.40	   756.48	 12
 14	 K 	   773.45	   705.96	 11
 15	 C 	   853.53	   641.91	 10
 16	 V 	   903.06	   561.83	  9
 17	 L 	   959.61	   512.29	  8
 18	 P 	  1008.13	   455.75	  7
 19	 H 	  1076.66	   407.22	  6
 20	 M 	  1142.18	   338.69	  5
 21	 I 	  1198.72	   273.17	  4
 22	 E 	  1263.25	   216.63	  3
 23	 R 	  1341.30	   152.11	  2
 24	 K 	        -  	    74.06	  1  _


+3 charge
		      B   	       Y
  1	 L 	    38.70	        -  	 24
  2	 I 	    76.40	   905.54	 23
  3	 E 	   119.41	   867.85	 22
  4	 L 	   157.11	   824.83	 21
  5	 N 	   195.12	   787.14	 20
  6	 Y 	   249.47	   749.12	 19
  7	 L 	   287.17	   694.77	 18
  8	 G 	   306.18	   657.07	 17
  9	 T 	   339.86	   638.07	 16
 10	 V 	   372.88	   604.38	 15
 11	 S 	   401.89	   571.36	 14
 12	 L 	   439.59	   542.35	 13
 13	 T 	   473.27	   504.66	 12
 14	 K 	   515.97	   470.97	 11
 15	 C 	   569.36	   428.27	 10
 16	 V 	   602.38	   374.89	  9
 17	 L 	   640.07	   341.86	  8
 18	 P 	   672.42	   304.17	  7
 19	 H 	   718.11	   271.82	  6
 20	 M 	   761.79	   226.13	  5
 21	 I 	   799.49	   182.45	  4
 22	 E 	   842.50	   144.76	  3
 23	 R 	   894.53	   101.74	  2
 24	 K 	        -  	    49.71	  1  _
```

**65. IPI00290856 Lymphatic vessel endothelial hyaluronic acid receptor 1**

| Sequence | Charge | M+H+ | XCorr | DeltaCn |
| --- | --- | --- | --- | --- |
| R.LLGLSLAGKDQVETALK.A | 2 | 1757.065 | 4.219 | 0.535 |

+1 charge

```
		      B   	       Y
  1	 L 	   114.09	        -  	 17
  2	 L 	   227.18	  1642.94	 16
  3	 G 	   284.20	  1529.85	 15
  4	 L 	   397.28	  1472.83	 14
  5	 S 	   484.31	  1359.75	 13
  6	 L 	   597.40	  1272.72	 12
  7	 A 	   668.43	  1159.63	 11
  8	 G 	   725.46	  1088.59	 10
  9	 K 	   853.55	  1031.57	  9
 10	 D 	   968.58	   903.48	  8
 11	 Q 	  1096.64	   788.45	  7
 12	 V 	  1195.70	   660.39	  6
 13	 E 	  1324.75	   561.32	  5
 14	 T 	  1425.79	   432.28	  4
 15	 A 	  1496.83	   331.23	  3
 16	 L 	  1609.92	   260.20	  2
 17	 K 	        -  	   147.11	  1  _
+2 charge
		      B   	       Y
  1	 L 	    57.55	        -  	 17
  2	 L 	   114.09	   821.97	 16
  3	 G 	   142.60	   765.43	 15
  4	 L 	   199.14	   736.92	 14
  5	 S 	   242.66	   680.38	 13
  6	 L 	   299.20	   636.86	 12
  7	 A 	   334.72	   580.32	 11
  8	 G 	   363.23	   544.80	 10
  9	 K 	   427.28	   516.29	  9
 10	 D 	   484.79	   452.24	  8
 11	 Q 	   548.82	   394.73	  7
 12	 V 	   598.36	   330.70	  6
 13	 E 	   662.88	   281.17	  5
 14	 T 	   713.40	   216.64	  4
 15	 A 	   748.92	   166.12	  3
 16	 L 	   805.46	   130.60	  2
 17	 K 	        -  	    74.06	  1  _
```

**66. IPI00176222 Isoform 1 of Zinc finger protein 549**

| Sequence | Charge | M+H+ | XCorr | DeltaCn |
| --- | --- | --- | --- | --- |
| K.WFSFGSNLQQHQNQDSGEKHIRK.E | 3 | 2772.975 | 3.355 | 0.215 |

+1 charge

```
		      B   	       Y
  1	 W 	   187.09	        -  	 23
  2	 F 	   334.16	  2585.26	 22
  3	 S 	   421.19	  2438.19	 21
  4	 F 	   568.26	  2351.16	 20
  5	 G 	   625.28	  2204.09	 19
  6	 S 	   712.31	  2147.07	 18
  7	 N 	   826.35	  2060.04	 17
  8	 L 	   939.44	  1946.00	 16
  9	 Q 	  1067.49	  1832.91	 15
 10	 Q 	  1195.55	  1704.85	 14
 11	 H 	  1332.61	  1576.79	 13
 12	 Q 	  1460.67	  1439.73	 12
 13	 N 	  1574.71	  1311.68	 11
 14	 Q 	  1702.77	  1197.63	 10
 15	 D 	  1817.80	  1069.57	  9
 16	 S 	  1904.83	   954.55	  8
 17	 G 	  1961.85	   867.52	  7
 18	 E 	  2090.90	   810.49	  6
 19	 K 	  2218.99	   681.45	  5
 20	 H 	  2356.05	   553.36	  4
 21	 I 	  2469.13	   416.30	  3
 22	 R 	  2625.23	   303.21	  2
 23	 K 	        -  	   147.11	  1  _


+2 charge
		      B   	       Y
  1	 W 	    94.05	        -  	 23
  2	 F 	   167.58	  1293.13	 22
  3	 S 	   211.10	  1219.60	 21
  4	 F 	   284.63	  1176.08	 20
  5	 G 	   313.14	  1102.55	 19
  6	 S 	   356.66	  1074.04	 18
  7	 N 	   413.68	  1030.52	 17
  8	 L 	   470.22	   973.50	 16
  9	 Q 	   534.25	   916.96	 15
 10	 Q 	   598.28	   852.93	 14
 11	 H 	   666.81	   788.90	 13
 12	 Q 	   730.84	   720.37	 12
 13	 N 	   787.86	   656.34	 11
 14	 Q 	   851.89	   599.32	 10
 15	 D 	   909.40	   535.29	  9
 16	 S 	   952.92	   477.78	  8
 17	 G 	   981.43	   434.26	  7
 18	 E 	  1045.95	   405.75	  6
 19	 K 	  1110.00	   341.23	  5
 20	 H 	  1178.53	   277.18	  4
 21	 I 	  1235.07	   208.65	  3
 22	 R 	  1313.12	   152.11	  2
 23	 K 	        -  	    74.06	  1  _


+3 charge
		      B   	       Y
  1	 W 	    63.03	        -  	 23
  2	 F 	   112.06	   862.42	 22
  3	 S 	   141.07	   813.40	 21
  4	 F 	   190.09	   784.39	 20
  5	 G 	   209.10	   735.37	 19
  6	 S 	   238.11	   716.36	 18
  7	 N 	   276.12	   687.35	 17
  8	 L 	   313.82	   649.34	 16
  9	 Q 	   356.50	   611.64	 15
 10	 Q 	   399.19	   568.96	 14
 11	 H 	   444.88	   526.27	 13
 12	 Q 	   487.56	   480.58	 12
 13	 N 	   525.58	   437.90	 11
 14	 Q 	   568.26	   399.88	 10
 15	 D 	   606.60	   357.20	  9
 16	 S 	   635.62	   318.85	  8
 17	 G 	   654.62	   289.84	  7
 18	 E 	   697.64	   270.84	  6
 19	 K 	   740.33	   227.82	  5
 20	 H 	   786.02	   185.12	  4
 21	 I 	   823.72	   139.44	  3
 22	 R 	   875.75	   101.74	  2
 23	 K 	        -  	    49.71	  1  _
```

**67. IPI00004416 Charged multivesicular body protein 2a**

| Sequence | Charge | M+H+ | XCorr | DeltaCn |
| --- | --- | --- | --- | --- |
| K.AEAAASALADADADLEERLK.N | 2 | 2031.167 | 3.617 | 0.479 |

+1 charge

```
		      B   	       Y
  1	 A 	    72.04	        -  	 20
  2	 E 	   201.09	  1958.97	 19
  3	 A 	   272.12	  1829.92	 18
  4	 A 	   343.16	  1758.89	 17
  5	 A 	   414.20	  1687.85	 16
  6	 S 	   501.23	  1616.81	 15
  7	 A 	   572.27	  1529.78	 14
  8	 L 	   685.35	  1458.74	 13
  9	 A 	   756.39	  1345.66	 12
 10	 D 	   871.42	  1274.62	 11
 11	 A 	   942.45	  1159.60	 10
 12	 D 	  1057.48	  1088.56	  9
 13	 A 	  1128.52	   973.53	  8
 14	 D 	  1243.54	   902.49	  7
 15	 L 	  1356.63	   787.47	  6
 16	 E 	  1485.67	   674.38	  5
 17	 E 	  1614.71	   545.34	  4
 18	 R 	  1770.81	   416.30	  3
 19	 L 	  1883.90	   260.20	  2
 20	 K 	        -  	   147.11	  1  _
+2 charge
		      B   	       Y
  1	 A 	    36.53	        -  	 20
  2	 E 	   101.05	   979.99	 19
  3	 A 	   136.57	   915.47	 18
  4	 A 	   172.08	   879.95	 17
  5	 A 	   207.60	   844.43	 16
  6	 S 	   251.12	   808.91	 15
  7	 A 	   286.64	   765.39	 14
  8	 L 	   343.18	   729.88	 13
  9	 A 	   378.70	   673.33	 12
 10	 D 	   436.21	   637.81	 11
 11	 A 	   471.73	   580.30	 10
 12	 D 	   529.24	   544.78	  9
 13	 A 	   564.76	   487.27	  8
 14	 D 	   622.28	   451.75	  7
 15	 L 	   678.82	   394.24	  6
 16	 E 	   743.34	   337.70	  5
 17	 E 	   807.86	   273.17	  4
 18	 R 	   885.91	   208.65	  3
 19	 L 	   942.45	   130.60	  2
 20	 K 	        -  	    74.06	  1  _
```

**68. IPI00382844 Aconitase (Fragment)**

| Sequence | Charge | M+H+ | XCorr | DeltaCn |
| --- | --- | --- | --- | --- |
| R.DGYAQILRDVGGIVLANACGPCIGQR.D | 3 | 2775.101 | 3.522 | 0.264 |

+1 charge

```
		      B   	       Y
  1	 D 	   116.03	        -  	 26
  2	 G 	   173.06	  2658.63	 25
  3	 Y 	   336.12	  2601.61	 24
  4	 A 	   407.16	  2438.54	 23
  5	 Q 	   535.21	  2367.51	 22
  6	 I 	   648.30	  2239.45	 21
  7	 L 	   761.38	  2126.36	 20
  8	 R 	   917.48	  2013.28	 19
  9	 D 	  1032.51	  1857.18	 18
 10	 V 	  1131.58	  1742.15	 17
 11	 G 	  1188.60	  1643.08	 16
 12	 G 	  1245.62	  1586.06	 15
 13	 I 	  1358.71	  1529.04	 14
 14	 V 	  1457.77	  1415.96	 13
 15	 L 	  1570.86	  1316.89	 12
 16	 A 	  1641.90	  1203.80	 11
 17	 N 	  1755.94	  1132.77	 10
 18	 A 	  1826.98	  1018.72	  9
 19	 C 	  1987.14	   947.69	  8
 20	 G 	  2044.16	   787.52	  7
 21	 P 	  2141.22	   730.50	  6
 22	 C 	  2301.38	   633.45	  5
 23	 I 	  2414.46	   473.28	  4
 24	 G 	  2471.49	   360.20	  3
 25	 Q 	  2599.54	   303.18	  2
 26	 R 	        -  	   175.12	  1  _


+2 charge
		      B   	       Y
  1	 D 	    58.52	        -  	 26
  2	 G 	    87.03	  1329.82	 25
  3	 Y 	   168.56	  1301.31	 24
  4	 A 	   204.08	  1219.78	 23
  5	 Q 	   268.11	  1184.26	 22
  6	 I 	   324.65	  1120.23	 21
  7	 L 	   381.20	  1063.69	 20
  8	 R 	   459.25	  1007.14	 19
  9	 D 	   516.76	   929.09	 18
 10	 V 	   566.29	   871.58	 17
 11	 G 	   594.80	   822.05	 16
 12	 G 	   623.31	   793.54	 15
 13	 I 	   679.86	   765.02	 14
 14	 V 	   729.39	   708.48	 13
 15	 L 	   785.93	   658.95	 12
 16	 A 	   821.45	   602.41	 11
 17	 N 	   878.47	   566.89	 10
 18	 A 	   913.99	   509.87	  9
 19	 C 	   994.07	   474.35	  8
 20	 G 	  1022.58	   394.26	  7
 21	 P 	  1071.11	   365.75	  6
 22	 C 	  1151.19	   317.23	  5
 23	 I 	  1207.74	   237.15	  4
 24	 G 	  1236.25	   180.60	  3
 25	 Q 	  1300.28	   152.09	  2
 26	 R 	        -  	    88.06	  1  _


+3 charge
		      B   	       Y
  1	 D 	    39.35	        -  	 26
  2	 G 	    58.36	   886.88	 25
  3	 Y 	   112.71	   867.87	 24
  4	 A 	   136.39	   813.52	 23
  5	 Q 	   179.08	   789.84	 22
  6	 I 	   216.77	   747.15	 21
  7	 L 	   254.47	   709.46	 20
  8	 R 	   306.50	   671.77	 19
  9	 D 	   344.84	   619.73	 18
 10	 V 	   377.86	   581.39	 17
 11	 G 	   396.87	   548.37	 16
 12	 G 	   415.88	   529.36	 15
 13	 I 	   453.57	   510.35	 14
 14	 V 	   486.60	   472.66	 13
 15	 L 	   524.29	   439.63	 12
 16	 A 	   547.97	   401.94	 11
 17	 N 	   585.98	   378.26	 10
 18	 A 	   609.66	   340.25	  9
 19	 C 	   663.05	   316.57	  8
 20	 G 	   682.06	   263.18	  7
 21	 P 	   714.41	   244.17	  6
 22	 C 	   767.80	   211.82	  5
 23	 I 	   805.49	   158.43	  4
 24	 G 	   824.50	   120.74	  3
 25	 Q 	   867.19	   101.73	  2
 26	 R 	        -  	    59.04	  1  _
```

**69. IPI00003919 Isoform 1 of Glutaminyl-peptide cyclotransferase**

| Sequence | Charge | M+H+ | XCorr | DeltaCn |
| --- | --- | --- | --- | --- |
| R.VFVGATDSAVPCAMMLELAR.A | 2 | 2139.519 | 4.662 | 0.535 |

+1 charge

```
		      B   	       Y
  1	 V 	   100.08	        -  	 20
  2	 F 	   247.14	  2039.11	 19
  3	 V 	   346.21	  1892.04	 18
  4	 G 	   403.23	  1792.97	 17
  5	 A 	   474.27	  1735.95	 16
  6	 T 	   575.32	  1664.92	 15
  7	 D 	   690.35	  1563.87	 14
  8	 S 	   777.38	  1448.84	 13
  9	 A 	   848.41	  1361.81	 12
 10	 V 	   947.48	  1290.77	 11
 11	 P 	  1044.54	  1191.70	 10
 12	 C 	  1204.70	  1094.65	  9
 13	 A 	  1275.74	   934.48	  8
 14	 M 	  1406.78	   863.45	  7
 15	 M 	  1537.82	   732.41	  6
 16	 L 	  1650.90	   601.37	  5
 17	 E 	  1779.95	   488.28	  4
 18	 L 	  1893.03	   359.24	  3
 19	 A 	  1964.07	   246.16	  2
 20	 R 	        -  	   175.12	  1  _
+2 charge
		      B   	       Y
  1	 V 	    50.54	        -  	 20
  2	 F 	   124.08	  1020.06	 19
  3	 V 	   173.61	   946.52	 18
  4	 G 	   202.12	   896.99	 17
  5	 A 	   237.64	   868.48	 16
  6	 T 	   288.16	   832.96	 15
  7	 D 	   345.68	   782.44	 14
  8	 S 	   389.19	   724.92	 13
  9	 A 	   424.71	   681.41	 12
 10	 V 	   474.25	   645.89	 11
 11	 P 	   522.77	   596.36	 10
 12	 C 	   602.85	   547.83	  9
 13	 A 	   638.37	   467.75	  8
 14	 M 	   703.89	   432.23	  7
 15	 M 	   769.41	   366.71	  6
 16	 L 	   825.96	   301.19	  5
 17	 E 	   890.48	   244.64	  4
 18	 L 	   947.02	   180.12	  3
 19	 A 	   982.54	   123.58	  2
 20	 R 	        -  	    88.06	  1  _
```

**70. IPI00410714 Hemoglobin subunit alpha**

| Sequence | Charge | M+H+ | XCorr | DeltaCn |
| --- | --- | --- | --- | --- |
| K.VGAHAGEYGAEALER.M | 2 | 1530.624 | 4.187 | 0.563 |

+1 charge

```
		      B   	       Y
  1	 V 	   100.08	        -  	 15
  2	 G 	   157.10	  1430.67	 14
  3	 A 	   228.13	  1373.64	 13
  4	 H 	   365.19	  1302.61	 12
  5	 A 	   436.23	  1165.55	 11
  6	 G 	   493.25	  1094.51	 10
  7	 E 	   622.29	  1037.49	  9
  8	 Y 	   785.36	   908.45	  8
  9	 G 	   842.38	   745.38	  7
 10	 A 	   913.42	   688.36	  6
 11	 E 	  1042.46	   617.33	  5
 12	 A 	  1113.50	   488.28	  4
 13	 L 	  1226.58	   417.25	  3
 14	 E 	  1355.62	   304.16	  2
 15	 R 	        -  	   175.12	  1  _
+2 charge
		      B   	       Y
  1	 V 	    50.54	        -  	 15
  2	 G 	    79.05	   715.84	 14
  3	 A 	   114.57	   687.33	 13
  4	 H 	   183.10	   651.81	 12
  5	 A 	   218.62	   583.28	 11
  6	 G 	   247.13	   547.76	 10
  7	 E 	   311.65	   519.25	  9
  8	 Y 	   393.18	   454.73	  8
  9	 G 	   421.69	   373.20	  7
 10	 A 	   457.21	   344.68	  6
 11	 E 	   521.73	   309.17	  5
 12	 A 	   557.25	   244.64	  4
 13	 L 	   613.79	   209.13	  3
 14	 E 	   678.31	   152.58	  2
 15	 R 	        -  	    88.06	  1  _
```

**71. IPI00442297 Isoform 2 of Neurotrimin**

| Sequence | Charge | M+H+ | XCorr | DeltaCn |
| --- | --- | --- | --- | --- |
| R.EQSGDYECSASNDVAAPVVR.R | 2 | 2155.216 | 4.203 | 0.364 |

```
+1 charge
		      B   	       Y
  1	 E 	   130.05	        -  	 20
  2	 Q 	   258.11	  2025.03	 19
  3	 S 	   345.14	  1896.97	 18
  4	 G 	   402.16	  1809.94	 17
  5	 D 	   517.19	  1752.92	 16
  6	 Y 	   680.25	  1637.89	 15
  7	 E 	   809.29	  1474.83	 14
  8	 C 	   969.46	  1345.79	 13
  9	 S 	  1056.49	  1185.62	 12
 10	 A 	  1127.53	  1098.59	 11
 11	 S 	  1214.56	  1027.55	 10
 12	 N 	  1328.60	   940.52	  9
 13	 D 	  1443.63	   826.48	  8
 14	 V 	  1542.70	   711.45	  7
 15	 A 	  1613.74	   612.38	  6
 16	 A 	  1684.77	   541.35	  5
 17	 P 	  1781.83	   470.31	  4
 18	 V 	  1880.89	   373.26	  3
 19	 V 	  1979.96	   274.19	  2
 20	 R 	        -  	   175.12	  1  _


+2 charge
		      B   	       Y
  1	 E 	    65.53	        -  	 20
  2	 Q 	   129.56	  1013.02	 19
  3	 S 	   173.07	   948.99	 18
  4	 G 	   201.58	   905.47	 17
  5	 D 	   259.10	   876.96	 16
  6	 Y 	   340.63	   819.45	 15
  7	 E 	   405.15	   737.92	 14
  8	 C 	   485.23	   673.40	 13
  9	 S 	   528.75	   593.31	 12
 10	 A 	   564.27	   549.80	 11
 11	 S 	   607.78	   514.28	 10
 12	 N 	   664.81	   470.76	  9
 13	 D 	   722.32	   413.74	  8
 14	 V 	   771.85	   356.23	  7
 15	 A 	   807.37	   306.70	  6
 16	 A 	   842.89	   271.18	  5
 17	 P 	   891.42	   235.66	  4
 18	 V 	   940.95	   187.13	  3
 19	 V 	   990.49	   137.60	  2
 20	 R 	        -  	    88.06	  1  _
```

**72. IPI00916111 Malate dehydrogenase**

| Sequence | Charge | M+H+ | XCorr | DeltaCn |
| --- | --- | --- | --- | --- |
| K.VIVVGNPANTNCLTASK.S | 2 | 1758.991 | 4.032 | 0.455 |

+1 charge

```
		      B   	       Y
  1	 V 	   100.08	        -  	 17
  2	 I 	   213.16	  1658.99	 16
  3	 V 	   312.23	  1545.90	 15
  4	 V 	   411.30	  1446.84	 14
  5	 G 	   468.32	  1347.77	 13
  6	 N 	   582.36	  1290.75	 12
  7	 P 	   679.41	  1176.70	 11
  8	 A 	   750.45	  1079.65	 10
  9	 N 	   864.49	  1008.61	  9
 10	 T 	   965.54	   894.57	  8
 11	 N 	  1079.58	   793.52	  7
 12	 C 	  1239.75	   679.48	  6
 13	 L 	  1352.83	   519.31	  5
 14	 T 	  1453.88	   406.23	  4
 15	 A 	  1524.92	   305.18	  3
 16	 S 	  1611.95	   234.14	  2
 17	 K 	        -  	   147.11	  1  _
+2 charge
		      B   	       Y
  1	 V 	    50.54	        -  	 17
  2	 I 	   107.08	   830.00	 16
  3	 V 	   156.62	   773.46	 15
  4	 V 	   206.15	   723.92	 14
  5	 G 	   234.66	   674.39	 13
  6	 N 	   291.68	   645.88	 12
  7	 P 	   340.21	   588.85	 11
  8	 A 	   375.73	   540.33	 10
  9	 N 	   432.75	   504.81	  9
 10	 T 	   483.27	   447.79	  8
 11	 N 	   540.30	   397.26	  7
 12	 C 	   620.38	   340.24	  6
 13	 L 	   676.92	   260.16	  5
 14	 T 	   727.44	   203.62	  4
 15	 A 	   762.96	   153.09	  3
 16	 S 	   806.48	   117.58	  2
 17	 K 	        -  	    74.06	  1  _
```

**73. IPI00298388 Isoform 1 of Phosphoinositide-3-kinase-interacting protein 1**

| Sequence | Charge | M+H+ | XCorr | DeltaCn |
| --- | --- | --- | --- | --- |
| R.SEAAAVQPVIGISQR.V | 2 | 1526.72 | 4.354 | 0.517 |

+1 charge

```
		      B   	       Y
  1	 S 	    88.04	        -  	 15
  2	 E 	   217.08	  1438.80	 14
  3	 A 	   288.12	  1309.76	 13
  4	 A 	   359.16	  1238.72	 12
  5	 A 	   430.19	  1167.68	 11
  6	 V 	   529.26	  1096.65	 10
  7	 Q 	   657.32	   997.58	  9
  8	 P 	   754.37	   869.52	  8
  9	 V 	   853.44	   772.47	  7
 10	 I 	   966.53	   673.40	  6
 11	 G 	  1023.55	   560.32	  5
 12	 I 	  1136.63	   503.29	  4
 13	 S 	  1223.66	   390.21	  3
 14	 Q 	  1351.72	   303.18	  2
 15	 R 	        -  	   175.12	  1  _
+2 charge
		      B   	       Y
  1	 S 	    44.52	        -  	 15
  2	 E 	   109.04	   719.90	 14
  3	 A 	   144.56	   655.38	 13
  4	 A 	   180.08	   619.86	 12
  5	 A 	   215.60	   584.35	 11
  6	 V 	   265.13	   548.83	 10
  7	 Q 	   329.16	   499.29	  9
  8	 P 	   377.69	   435.26	  8
  9	 V 	   427.22	   386.74	  7
 10	 I 	   483.77	   337.20	  6
 11	 G 	   512.28	   280.66	  5
 12	 I 	   568.82	   252.15	  4
 13	 S 	   612.34	   195.61	  3
 14	 Q 	   676.36	   152.09	  2
 15	 R 	        -  	    88.06	  1  _
```

**74. IPI00246058 Programmed cell death 6-interacting protein**

| Sequence | Charge | M+H+ | XCorr | DeltaCn |
| --- | --- | --- | --- | --- |
| K.MVPVSVQQSLAAYNQR.K | 2 | 1792.053 | 3.159 | 0.367 |

+1 charge

```
		      B   	       Y
  1	 M 	   132.05	        -  	 16
  2	 V 	   231.12	  1659.88	 15
  3	 P 	   328.17	  1560.81	 14
  4	 V 	   427.24	  1463.76	 13
  5	 S 	   514.27	  1364.69	 12
  6	 V 	   613.34	  1277.66	 11
  7	 Q 	   741.40	  1178.59	 10
  8	 Q 	   869.45	  1050.53	  9
  9	 S 	   956.49	   922.47	  8
 10	 L 	  1069.57	   835.44	  7
 11	 A 	  1140.61	   722.36	  6
 12	 A 	  1211.65	   651.32	  5
 13	 Y 	  1374.71	   580.28	  4
 14	 N 	  1488.75	   417.22	  3
 15	 Q 	  1616.81	   303.18	  2
 16	 R 	        -  	   175.12	  1  _
+2 charge
		      B   	       Y
  1	 M 	    66.53	        -  	 16
  2	 V 	   116.06	   830.44	 15
  3	 P 	   164.59	   780.91	 14
  4	 V 	   214.12	   732.38	 13
  5	 S 	   257.64	   682.85	 12
  6	 V 	   307.17	   639.33	 11
  7	 Q 	   371.20	   589.80	 10
  8	 Q 	   435.23	   525.77	  9
  9	 S 	   478.75	   461.74	  8
 10	 L 	   535.29	   418.22	  7
 11	 A 	   570.81	   361.68	  6
 12	 A 	   606.33	   326.16	  5
 13	 Y 	   687.86	   290.65	  4
 14	 N 	   744.88	   209.11	  3
 15	 Q 	   808.91	   152.09	  2
 16	 R 	        -  	    88.06	  1  _
```

**75. IPI00030786 Isoform 1 of Matrilin-4**

| Sequence | Charge | M+H+ | XCorr | DeltaCn |
| --- | --- | --- | --- | --- |
| R.TLGALESLTLNLAQLTAR.L | 2 | 1886.183 | 4.669 | 0.61 |

+1 charge

```
		      B   	       Y
  1	 T 	   102.05	        -  	 18
  2	 L 	   215.14	  1784.03	 17
  3	 G 	   272.16	  1670.94	 16
  4	 A 	   343.20	  1613.92	 15
  5	 L 	   456.28	  1542.88	 14
  6	 E 	   585.32	  1429.80	 13
  7	 S 	   672.36	  1300.76	 12
  8	 L 	   785.44	  1213.73	 11
  9	 T 	   886.49	  1100.64	 10
 10	 L 	   999.57	   999.59	  9
 11	 N 	  1113.62	   886.51	  8
 12	 L 	  1226.70	   772.47	  7
 13	 A 	  1297.74	   659.38	  6
 14	 Q 	  1425.79	   588.35	  5
 15	 L 	  1538.88	   460.29	  4
 16	 T 	  1639.93	   347.20	  3
 17	 A 	  1710.96	   246.16	  2
 18	 R 	        -  	   175.12	  1  _
+2 charge
		      B   	       Y
  1	 T 	    51.53	        -  	 18
  2	 L 	   108.07	   892.52	 17
  3	 G 	   136.58	   835.98	 16
  4	 A 	   172.10	   807.46	 15
  5	 L 	   228.64	   771.95	 14
  6	 E 	   293.17	   715.40	 13
  7	 S 	   336.68	   650.88	 12
  8	 L 	   393.22	   607.37	 11
  9	 T 	   443.75	   550.82	 10
 10	 L 	   500.29	   500.30	  9
 11	 N 	   557.31	   443.76	  8
 12	 L 	   613.85	   386.74	  7
 13	 A 	   649.37	   330.20	  6
 14	 Q 	   713.40	   294.68	  5
 15	 L 	   769.94	   230.65	  4
 16	 T 	   820.47	   174.11	  3
 17	 A 	   855.99	   123.58	  2
 18	 R 	        -  	    88.06	  1  _
```

**76. IPI00873924 Isoform A of Interleukin-18-binding protein**

| Sequence | Charge | M+H+ | XCorr | DeltaCn |
| --- | --- | --- | --- | --- |
| R.STKDPCPSQPPVFPAAK.Q | 2 | 1828.052 | 3.67 | 0.421 |

+1 charge

```
		      B   	       Y
  1	 S 	    88.04	        -  	 17
  2	 T 	   189.09	  1740.01	 16
  3	 K 	   317.18	  1638.97	 15
  4	 D 	   432.21	  1510.87	 14
  5	 P 	   529.26	  1395.84	 13
  6	 C 	   689.43	  1298.79	 12
  7	 P 	   786.48	  1138.63	 11
  8	 S 	   873.51	  1041.57	 10
  9	 Q 	  1001.57	   954.54	  9
 10	 P 	  1098.62	   826.48	  8
 11	 P 	  1195.68	   729.43	  7
 12	 V 	  1294.74	   632.38	  6
 13	 F 	  1441.81	   533.31	  5
 14	 P 	  1538.87	   386.24	  4
 15	 A 	  1609.90	   289.19	  3
 16	 A 	  1680.94	   218.15	  2
 17	 K 	        -  	   147.11	  1  _
+2 charge
		      B   	       Y
  1	 S 	    44.52	        -  	 17
  2	 T 	    95.05	   870.51	 16
  3	 K 	   159.09	   819.99	 15
  4	 D 	   216.61	   755.94	 14
  5	 P 	   265.13	   698.43	 13
  6	 C 	   345.22	   649.90	 12
  7	 P 	   393.74	   569.82	 11
  8	 S 	   437.26	   521.29	 10
  9	 Q 	   501.29	   477.77	  9
 10	 P 	   549.82	   413.74	  8
 11	 P 	   598.34	   365.22	  7
 12	 V 	   647.88	   316.69	  6
 13	 F 	   721.41	   267.16	  5
 14	 P 	   769.94	   193.62	  4
 15	 A 	   805.45	   145.10	  3
 16	 A 	   840.97	   109.58	  2
 17	 K 	        -  	    74.06	  1  _
```

**77. IPI00384404 Rheumatoid factor RF-ET9 (Fragment)**

| Sequence | Charge | M+H+ | XCorr | DeltaCn |
| --- | --- | --- | --- | --- |
| - .LVESGGGLVKPGGSLR.L | 2 | 1526.763 | 4.996 | 0.54 |

+1 charge

```
		      B   	       Y
  1	 L 	   114.09	        -  	 16
  2	 V 	   213.16	  1412.79	 15
  3	 E 	   342.20	  1313.72	 14
  4	 S 	   429.23	  1184.67	 13
  5	 G 	   486.26	  1097.64	 12
  6	 G 	   543.28	  1040.62	 11
  7	 G 	   600.30	   983.60	 10
  8	 L 	   713.38	   926.58	  9
  9	 V 	   812.45	   813.49	  8
 10	 K 	   940.55	   714.43	  7
 11	 P 	  1037.60	   586.33	  6
 12	 G 	  1094.62	   489.28	  5
 13	 G 	  1151.64	   432.26	  4
 14	 S 	  1238.67	   375.24	  3
 15	 L 	  1351.76	   288.20	  2
 16	 R 	        -  	   175.12	  1  _
+2 charge
		      B   	       Y
  1	 L 	    57.55	        -  	 16
  2	 V 	   107.08	   706.90	 15
  3	 E 	   171.60	   657.36	 14
  4	 S 	   215.12	   592.84	 13
  5	 G 	   243.63	   549.32	 12
  6	 G 	   272.14	   520.81	 11
  7	 G 	   300.65	   492.30	 10
  8	 L 	   357.20	   463.79	  9
  9	 V 	   406.73	   407.25	  8
 10	 K 	   470.78	   357.72	  7
 11	 P 	   519.30	   293.67	  6
 12	 G 	   547.81	   245.14	  5
 13	 G 	   576.32	   216.63	  4
 14	 S 	   619.84	   188.12	  3
 15	 L 	   676.38	   144.61	  2
 16	 R 	        -  	    88.06	  1  _
```

**78. IPI00012102 N-acetylglucosamine-6-sulfatase**

| Sequence | Charge | M+H+ | XCorr | DeltaCn |
| --- | --- | --- | --- | --- |
| R.LMMLQSCSGPTCR.T | 2 | 1541.807 | 3.029 | 0.357 |

+1 charge

```
		      B   	       Y
  1	 L 	   114.09	        -  	 13
  2	 M 	   245.13	  1427.86	 12
  3	 M 	   376.17	  1296.82	 11
  4	 L 	   489.26	  1165.78	 10
  5	 Q 	   617.31	  1052.69	  9
  6	 S 	   704.35	   924.64	  8
  7	 C 	   864.51	   837.60	  7
  8	 S 	   951.54	   677.44	  6
  9	 G 	  1008.57	   590.41	  5
 10	 P 	  1105.62	   533.38	  4
 11	 T 	  1206.67	   436.33	  3
 12	 C 	  1366.83	   335.28	  2
 13	 R 	        -  	   175.12	  1  _
+2 charge
		      B   	       Y
  1	 L 	    57.55	        -  	 13
  2	 M 	   123.07	   714.43	 12
  3	 M 	   188.59	   648.91	 11
  4	 L 	   245.13	   583.39	 10
  5	 Q 	   309.16	   526.85	  9
  6	 S 	   352.68	   462.82	  8
  7	 C 	   432.76	   419.31	  7
  8	 S 	   476.28	   339.22	  6
  9	 G 	   504.79	   295.71	  5
 10	 P 	   553.31	   267.20	  4
 11	 T 	   603.84	   218.67	  3
 12	 C 	   683.92	   168.15	  2
 13	 R 	        -  	    88.06	  1  _
```

**79. IPI00384401 Myosin-reactive immunoglobulin kappa chain variable region (Fragment)**

| Sequence | Charge | M+H+ | XCorr | DeltaCn |
| --- | --- | --- | --- | --- |
| - .EIVMTQSPATLSVSPGER.A | 2 | 1903.147 | 4.559 | 0.503 |

+1 charge

```
		      B   	       Y
  1	 E 	   130.05	        -  	 18
  2	 I 	   243.13	  1772.92	 17
  3	 V 	   342.20	  1659.84	 16
  4	 M 	   473.24	  1560.77	 15
  5	 T 	   574.29	  1429.73	 14
  6	 Q 	   702.35	  1328.68	 13
  7	 S 	   789.38	  1200.62	 12
  8	 P 	   886.43	  1113.59	 11
  9	 A 	   957.47	  1016.54	 10
 10	 T 	  1058.52	   945.50	  9
 11	 L 	  1171.60	   844.45	  8
 12	 S 	  1258.63	   731.37	  7
 13	 V 	  1357.70	   644.34	  6
 14	 S 	  1444.74	   545.27	  5
 15	 P 	  1541.79	   458.24	  4
 16	 G 	  1598.81	   361.18	  3
 17	 E 	  1727.85	   304.16	  2
 18	 R 	        -  	   175.12	  1  _
+2 charge
		      B   	       Y
  1	 E 	    65.53	        -  	 18
  2	 I 	   122.07	   886.96	 17
  3	 V 	   171.60	   830.42	 16
  4	 M 	   237.13	   780.89	 15
  5	 T 	   287.65	   715.37	 14
  6	 Q 	   351.68	   664.84	 13
  7	 S 	   395.19	   600.81	 12
  8	 P 	   443.72	   557.30	 11
  9	 A 	   479.24	   508.77	 10
 10	 T 	   529.76	   473.25	  9
 11	 L 	   586.31	   422.73	  8
 12	 S 	   629.82	   366.19	  7
 13	 V 	   679.36	   322.67	  6
 14	 S 	   722.87	   273.14	  5
 15	 P 	   771.40	   229.62	  4
 16	 G 	   799.91	   181.10	  3
 17	 E 	   864.43	   152.58	  2
 18	 R 	        -  	    88.06	  1  _
```

**80. IPI00018901 Isoform 1 of Gamma-glutamyltranspeptidase 1**

| Sequence | Charge | M+H+ | XCorr | DeltaCn |
| --- | --- | --- | --- | --- |
| R.TVIEQQPVLCEVFCR.D | 2 | 1879.134 | 3.706 | 0.348 |

+1 charge

```
		      B   	       Y
  1	 T 	   102.05	        -  	 15
  2	 V 	   201.12	  1777.15	 14
  3	 I 	   314.21	  1678.08	 13
  4	 E 	   443.25	  1564.99	 12
  5	 Q 	   571.31	  1435.95	 11
  6	 Q 	   699.37	  1307.89	 10
  7	 P 	   796.42	  1179.83	  9
  8	 V 	   895.49	  1082.78	  8
  9	 L 	  1008.57	   983.71	  7
 10	 C 	  1168.74	   870.63	  6
 11	 E 	  1297.78	   710.46	  5
 12	 V 	  1396.85	   581.42	  4
 13	 F 	  1543.92	   482.35	  3
 14	 C 	  1704.08	   335.28	  2
 15	 R 	        -  	   175.12	  1  _
+2 charge
		      B   	       Y
  1	 T 	    51.53	        -  	 15
  2	 V 	   101.07	   889.08	 14
  3	 I 	   157.61	   839.54	 13
  4	 E 	   222.13	   783.00	 12
  5	 Q 	   286.16	   718.48	 11
  6	 Q 	   350.19	   654.45	 10
  7	 P 	   398.71	   590.42	  9
  8	 V 	   448.25	   541.89	  8
  9	 L 	   504.79	   492.36	  7
 10	 C 	   584.87	   435.82	  6
 11	 E 	   649.39	   355.74	  5
 12	 V 	   698.93	   291.21	  4
 13	 F 	   772.46	   241.68	  3
 14	 C 	   852.54	   168.15	  2
 15	 R 	        -  	    88.06	  1  _
```

**81. IPI00022620 Secreted Ly-6/uPAR-related protein 1**

| Sequence | Charge | M+H+ | XCorr | DeltaCn |
| --- | --- | --- | --- | --- |
| R.SCSSSCVATDPDSIGAAHLIFCCFR.D | 3 | 2820.036 | 4.264 | 0.324 |

+1 charge

```
		      B   	       Y
  1	 S 	    88.04	        -  	 25
  2	 C 	   248.20	  2731.71	 24
  3	 S 	   335.24	  2571.55	 23
  4	 S 	   422.27	  2484.51	 22
  5	 S 	   509.30	  2397.48	 21
  6	 C 	   669.47	  2310.45	 20
  7	 V 	   768.53	  2150.28	 19
  8	 A 	   839.57	  2051.22	 18
  9	 T 	   940.62	  1980.18	 17
 10	 D 	  1055.65	  1879.13	 16
 11	 P 	  1152.70	  1764.10	 15
 12	 D 	  1267.73	  1667.05	 14
 13	 S 	  1354.76	  1552.03	 13
 14	 I 	  1467.84	  1464.99	 12
 15	 G 	  1524.86	  1351.91	 11
 16	 A 	  1595.90	  1294.89	 10
 17	 A 	  1666.94	  1223.85	  9
 18	 H 	  1804.00	  1152.81	  8
 19	 L 	  1917.08	  1015.75	  7
 20	 I 	  2030.16	   902.67	  6
 21	 F 	  2177.23	   789.59	  5
 22	 C 	  2337.40	   642.52	  4
 23	 C 	  2497.56	   482.35	  3
 24	 F 	  2644.63	   322.19	  2
 25	 R 	        -  	   175.12	  1  _


+2 charge
		      B   	       Y
  1	 S 	    44.52	        -  	 25
  2	 C 	   124.61	  1366.36	 24
  3	 S 	   168.12	  1286.28	 23
  4	 S 	   211.64	  1242.76	 22
  5	 S 	   255.15	  1199.24	 21
  6	 C 	   335.24	  1155.73	 20
  7	 V 	   384.77	  1075.65	 19
  8	 A 	   420.29	  1026.11	 18
  9	 T 	   470.81	   990.59	 17
 10	 D 	   528.33	   940.07	 16
 11	 P 	   576.85	   882.56	 15
 12	 D 	   634.37	   834.03	 14
 13	 S 	   677.88	   776.52	 13
 14	 I 	   734.42	   733.00	 12
 15	 G 	   762.94	   676.46	 11
 16	 A 	   798.45	   647.95	 10
 17	 A 	   833.97	   612.43	  9
 18	 H 	   902.50	   576.91	  8
 19	 L 	   959.04	   508.38	  7
 20	 I 	  1015.59	   451.84	  6
 21	 F 	  1089.12	   395.30	  5
 22	 C 	  1169.20	   321.76	  4
 23	 C 	  1249.29	   241.68	  3
 24	 F 	  1322.82	   161.60	  2
 25	 R 	        -  	    88.06	  1  _


+3 charge
		      B   	       Y
  1	 S 	    30.02	        -  	 25
  2	 C 	    83.41	   911.24	 24
  3	 S 	   112.42	   857.85	 23
  4	 S 	   141.43	   828.84	 22
  5	 S 	   170.44	   799.83	 21
  6	 C 	   223.83	   770.82	 20
  7	 V 	   256.85	   717.43	 19
  8	 A 	   280.53	   684.41	 18
  9	 T 	   314.21	   660.73	 17
 10	 D 	   352.55	   627.05	 16
 11	 P 	   384.90	   588.71	 15
 12	 D 	   423.25	   556.36	 14
 13	 S 	   452.26	   518.01	 13
 14	 I 	   489.95	   489.00	 12
 15	 G 	   508.96	   451.31	 11
 16	 A 	   532.64	   432.30	 10
 17	 A 	   556.32	   408.62	  9
 18	 H 	   602.00	   384.94	  8
 19	 L 	   639.70	   339.26	  7
 20	 I 	   677.39	   301.56	  6
 21	 F 	   726.42	   263.87	  5
 22	 C 	   779.80	   214.84	  4
 23	 C 	   833.19	   161.46	  3
 24	 F 	   882.22	   108.07	  2
 25	 R 	        -  	    59.04	  1  _
```

**82. IPI00016786 Isoform 2 of Cell division control protein 42 homolog**

| Sequence | Charge | M+H+ | XCorr | DeltaCn |
| --- | --- | --- | --- | --- |
| K.GLKNVFDEAILAALEPPEPK.K | 2 | 2152.476 | 3.022 | 0.439 |

+1 charge

```
		      B   	       Y
  1	 G 	    58.03	        -  	 20
  2	 L 	   171.11	  2094.15	 19
  3	 K 	   299.21	  1981.06	 18
  4	 N 	   413.25	  1852.97	 17
  5	 V 	   512.32	  1738.93	 16
  6	 F 	   659.39	  1639.86	 15
  7	 D 	   774.41	  1492.79	 14
  8	 E 	   903.46	  1377.76	 13
  9	 A 	   974.49	  1248.72	 12
 10	 I 	  1087.58	  1177.68	 11
 11	 L 	  1200.66	  1064.60	 10
 12	 A 	  1271.70	   951.51	  9
 13	 A 	  1342.74	   880.48	  8
 14	 L 	  1455.82	   809.44	  7
 15	 E 	  1584.86	   696.36	  6
 16	 P 	  1681.92	   567.31	  5
 17	 P 	  1778.97	   470.26	  4
 18	 E 	  1908.01	   373.21	  3
 19	 P 	  2005.06	   244.17	  2
 20	 K 	        -  	   147.11	  1  _
+2 charge
		      B   	       Y
  1	 G 	    29.52	        -  	 20
  2	 L 	    86.06	  1047.58	 19
  3	 K 	   150.11	   991.04	 18
  4	 N 	   207.13	   926.99	 17
  5	 V 	   256.66	   869.97	 16
  6	 F 	   330.20	   820.43	 15
  7	 D 	   387.71	   746.90	 14
  8	 E 	   452.23	   689.38	 13
  9	 A 	   487.75	   624.86	 12
 10	 I 	   544.29	   589.34	 11
 11	 L 	   600.83	   532.80	 10
 12	 A 	   636.35	   476.26	  9
 13	 A 	   671.87	   440.74	  8
 14	 L 	   728.41	   405.22	  7
 15	 E 	   792.94	   348.68	  6
 16	 P 	   841.46	   284.16	  5
 17	 P 	   889.99	   235.63	  4
 18	 E 	   954.51	   187.11	  3
 19	 P 	  1003.04	   122.59	  2
 20	 K 	        -  	    74.06	  1  _
```

**83. IPI00018236 Ganglioside GM2 activator**

| Sequence | Charge | M+H+ | XCorr | DeltaCn |
| --- | --- | --- | --- | --- |
| K.SEFVVPDLELPSWLTTGNYR.I | 2 | 2324.573 | 3.274 | 0.53 |

+1 charge

```
		      B   	       Y
  1	 S 	    88.04	        -  	 20
  2	 E 	   217.08	  2236.13	 19
  3	 F 	   364.15	  2107.09	 18
  4	 V 	   463.22	  1960.02	 17
  5	 V 	   562.29	  1860.95	 16
  6	 P 	   659.34	  1761.88	 15
  7	 D 	   774.37	  1664.83	 14
  8	 L 	   887.45	  1549.80	 13
  9	 E 	  1016.49	  1436.72	 12
 10	 L 	  1129.58	  1307.67	 11
 11	 P 	  1226.63	  1194.59	 10
 12	 S 	  1313.66	  1097.54	  9
 13	 W 	  1499.74	  1010.51	  8
 14	 L 	  1612.83	   824.43	  7
 15	 T 	  1713.87	   711.34	  6
 16	 T 	  1814.92	   610.29	  5
 17	 G 	  1871.94	   509.25	  4
 18	 N 	  1985.99	   452.23	  3
 19	 Y 	  2149.05	   338.18	  2
 20	 R 	        -  	   175.12	  1  _
+2 charge
		      B   	       Y
  1	 S 	    44.52	        -  	 20
  2	 E 	   109.04	  1118.57	 19
  3	 F 	   182.58	  1054.05	 18
  4	 V 	   232.11	   980.51	 17
  5	 V 	   281.65	   930.98	 16
  6	 P 	   330.17	   881.44	 15
  7	 D 	   387.69	   832.92	 14
  8	 L 	   444.23	   775.40	 13
  9	 E 	   508.75	   718.86	 12
 10	 L 	   565.29	   654.34	 11
 11	 P 	   613.82	   597.80	 10
 12	 S 	   657.33	   549.27	  9
 13	 W 	   750.37	   505.76	  8
 14	 L 	   806.92	   412.72	  7
 15	 T 	   857.44	   356.17	  6
 16	 T 	   907.96	   305.65	  5
 17	 G 	   936.47	   255.13	  4
 18	 N 	   993.50	   226.62	  3
 19	 Y 	  1075.03	   169.59	  2
 20	 R 	        -  	    88.06	  1  _
```

**84. IPI00005563 Isoform 1 of Tubulointerstitial nephritis antigen-like**

| Sequence | Charge | M+H+ | XCorr | DeltaCn |
| --- | --- | --- | --- | --- |
| R.YCQEQDLCCR.G | 2 | 1432.499 | 2.908 | 0.215 |

+1 charge

```
		      B   	       Y
  1	 Y 	   164.07	        -  	 10
  2	 C 	   324.24	  1268.89	  9
  3	 Q 	   452.29	  1108.72	  8
  4	 E 	   581.34	   980.66	  7
  5	 Q 	   709.40	   851.62	  6
  6	 D 	   824.42	   723.56	  5
  7	 L 	   937.51	   608.53	  4
  8	 C 	  1097.67	   495.45	  3
  9	 C 	  1257.84	   335.28	  2
 10	 R 	        -  	   175.12	  1  _
+2 charge
		      B   	       Y
  1	 Y 	    82.54	        -  	 10
  2	 C 	   162.62	   634.95	  9
  3	 Q 	   226.65	   554.86	  8
  4	 E 	   291.17	   490.83	  7
  5	 Q 	   355.20	   426.31	  6
  6	 D 	   412.71	   362.28	  5
  7	 L 	   469.26	   304.77	  4
  8	 C 	   549.34	   248.23	  3
  9	 C 	   629.42	   168.15	  2
 10	 R 	        -  	    88.06	  1  _
```

**85. IPI00451132 cGMP-dependent 3',5'-cyclic phosphodiesterase**

| Sequence | Charge | M+H+ | XCorr | DeltaCn |
| --- | --- | --- | --- | --- |
| K.EFFSQGDLEKAMGNRPMEMMDR.E | 3 | 2620.991 | 3.303 | 0.274 |

+1 charge

```
		      B   	       Y
  1	 E 	   130.05	        -  	 22
  2	 F 	   277.12	  2490.10	 21
  3	 F 	   424.19	  2343.03	 20
  4	 S 	   511.22	  2195.97	 19
  5	 Q 	   639.28	  2108.93	 18
  6	 G 	   696.30	  1980.88	 17
  7	 D 	   811.33	  1923.85	 16
  8	 L 	   924.41	  1808.83	 15
  9	 E 	  1053.45	  1695.74	 14
 10	 K 	  1181.55	  1566.70	 13
 11	 A 	  1252.58	  1438.61	 12
 12	 M 	  1383.62	  1367.57	 11
 13	 G 	  1440.65	  1236.53	 10
 14	 N 	  1554.69	  1179.51	  9
 15	 R 	  1710.79	  1065.46	  8
 16	 P 	  1807.84	   909.36	  7
 17	 M 	  1938.88	   812.31	  6
 18	 E 	  2067.93	   681.27	  5
 19	 M 	  2198.97	   552.23	  4
 20	 M 	  2330.01	   421.19	  3
 21	 D 	  2445.03	   290.15	  2
 22	 R 	        -  	   175.12	  1  _


+2 charge
		      B   	       Y
  1	 E 	    65.53	        -  	 22
  2	 F 	   139.06	  1245.56	 21
  3	 F 	   212.60	  1172.02	 20
  4	 S 	   256.11	  1098.49	 19
  5	 Q 	   320.14	  1054.97	 18
  6	 G 	   348.65	   990.94	 17
  7	 D 	   406.17	   962.43	 16
  8	 L 	   462.71	   904.92	 15
  9	 E 	   527.23	   848.38	 14
 10	 K 	   591.28	   783.85	 13
 11	 A 	   626.80	   719.81	 12
 12	 M 	   692.32	   684.29	 11
 13	 G 	   720.83	   618.77	 10
 14	 N 	   777.85	   590.26	  9
 15	 R 	   855.90	   533.24	  8
 16	 P 	   904.43	   455.18	  7
 17	 M 	   969.95	   406.66	  6
 18	 E 	  1034.47	   341.14	  5
 19	 M 	  1099.99	   276.62	  4
 20	 M 	  1165.51	   211.10	  3
 21	 D 	  1223.02	   145.58	  2
 22	 R 	        -  	    88.06	  1  _
+3 charge
		      B   	       Y
  1	 E 	    44.02	        -  	 22
  2	 F 	    93.04	   830.71	 21
  3	 F 	   142.07	   781.68	 20
  4	 S 	   171.08	   732.66	 19
  5	 Q 	   213.76	   703.65	 18
  6	 G 	   232.77	   660.96	 17
  7	 D 	   271.11	   641.96	 16
  8	 L 	   308.81	   603.61	 15
  9	 E 	   351.82	   565.92	 14
 10	 K 	   394.52	   522.91	 13
 11	 A 	   418.20	   480.21	 12
 12	 M 	   461.88	   456.53	 11
 13	 G 	   480.89	   412.85	 10
 14	 N 	   518.90	   393.84	  9
 15	 R 	   570.93	   355.83	  8
 16	 P 	   603.29	   303.79	  7
 17	 M 	   646.97	   271.44	  6
 18	 E 	   689.98	   227.76	  5
 19	 M 	   733.66	   184.75	  4
 20	 M 	   777.34	   141.07	  3
 21	 D 	   815.68	    97.39	  2
 22	 R 	        -  	    59.04	  1  _
```

**86. IPI00289931 MADCAM1 protein**

| Sequence | Charge | M+H+ | XCorr | DeltaCn |
| --- | --- | --- | --- | --- |
| R.GLDTSLGAVQSDTGR.S | 2 | 1477.559 | 4.551 | 0.509 |

+1 charge

```
		      B   	       Y
  1	 G 	    58.03	        -  	 15
  2	 L 	   171.11	  1419.71	 14
  3	 D 	   286.14	  1306.62	 13
  4	 T 	   387.19	  1191.60	 12
  5	 S 	   474.22	  1090.55	 11
  6	 L 	   587.30	  1003.52	 10
  7	 G 	   644.32	   890.43	  9
  8	 A 	   715.36	   833.41	  8
  9	 V 	   814.43	   762.37	  7
 10	 Q 	   942.49	   663.31	  6
 11	 S 	  1029.52	   535.25	  5
 12	 D 	  1144.55	   448.22	  4
 13	 T 	  1245.60	   333.19	  3
 14	 G 	  1302.62	   232.14	  2
 15	 R 	        -  	   175.12	  1  _
+2 charge
		      B   	       Y
  1	 G 	    29.52	        -  	 15
  2	 L 	    86.06	   710.36	 14
  3	 D 	   143.57	   653.82	 13
  4	 T 	   194.10	   596.30	 12
  5	 S 	   237.61	   545.78	 11
  6	 L 	   294.16	   502.26	 10
  7	 G 	   322.67	   445.72	  9
  8	 A 	   358.18	   417.21	  8
  9	 V 	   407.72	   381.69	  7
 10	 Q 	   471.75	   332.16	  6
 11	 S 	   515.26	   268.13	  5
 12	 D 	   572.78	   224.61	  4
 13	 T 	   623.30	   167.10	  3
 14	 G 	   651.81	   116.57	  2
 15	 R 	        -  	    88.06	  1  _
```

**87. IPI00011140 Protein NOV homolog**

| Sequence | Charge | M+H+ | XCorr | DeltaCn |
| --- | --- | --- | --- | --- |
| R.AVLDGCSCCLVCAR.Q | 2 | 1641.823 | 4.304 | 0.603 |

+1 charge

```
		      B   	       Y
  1	 A 	    72.04	        -  	 14
  2	 V 	   171.11	  1570.20	 13
  3	 L 	   284.20	  1471.13	 12
  4	 D 	   399.22	  1358.05	 11
  5	 G 	   456.25	  1243.02	 10
  6	 C 	   616.41	  1186.00	  9
  7	 S 	   703.44	  1025.84	  8
  8	 C 	   863.61	   938.80	  7
  9	 C 	  1023.77	   778.64	  6
 10	 L 	  1136.86	   618.47	  5
 11	 V 	  1235.93	   505.39	  4
 12	 C 	  1396.09	   406.32	  3
 13	 A 	  1467.13	   246.16	  2
 14	 R 	        -  	   175.12	  1  _
+2 charge
		      B   	       Y
  1	 A 	    36.53	        -  	 14
  2	 V 	    86.06	   785.60	 13
  3	 L 	   142.60	   736.07	 12
  4	 D 	   200.12	   679.53	 11
  5	 G 	   228.63	   622.02	 10
  6	 C 	   308.71	   593.50	  9
  7	 S 	   352.22	   513.42	  8
  8	 C 	   432.31	   469.91	  7
  9	 C 	   512.39	   389.82	  6
 10	 L 	   568.93	   309.74	  5
 11	 V 	   618.47	   253.20	  4
 12	 C 	   698.55	   203.66	  3
 13	 A 	   734.07	   123.58	  2
 14	 R 	        -  	    88.06	  1  _
```

**88. IPI00385985 Ig lambda chain V-III region LOI**

| Sequence | Charge | M+H+ | XCorr | DeltaCn |
| --- | --- | --- | --- | --- |
| - .YVLTQPPSVSVAPGETAR.L | 2 | 1873.099 | 3.872 | 0.501 |

+1 charge

```
		      B   	       Y
  1	 Y 	   164.07	        -  	 18
  2	 V 	   263.14	  1708.92	 17
  3	 L 	   376.22	  1609.85	 16
  4	 T 	   477.27	  1496.77	 15
  5	 Q 	   605.33	  1395.72	 14
  6	 P 	   702.38	  1267.66	 13
  7	 P 	   799.43	  1170.61	 12
  8	 S 	   886.47	  1073.56	 11
  9	 V 	   985.54	   986.53	 10
 10	 S 	  1072.57	   887.46	  9
 11	 V 	  1171.64	   800.43	  8
 12	 A 	  1242.67	   701.36	  7
 13	 P 	  1339.73	   630.32	  6
 14	 G 	  1396.75	   533.27	  5
 15	 E 	  1525.79	   476.25	  4
 16	 T 	  1626.84	   347.20	  3
 17	 A 	  1697.87	   246.16	  2
 18	 R 	        -  	   175.12	  1  _
+2 charge
		      B   	       Y
  1	 Y 	    82.54	        -  	 18
  2	 V 	   132.07	   854.97	 17
  3	 L 	   188.62	   805.43	 16
  4	 T 	   239.14	   748.89	 15
  5	 Q 	   303.17	   698.36	 14
  6	 P 	   351.69	   634.34	 13
  7	 P 	   400.22	   585.81	 12
  8	 S 	   443.74	   537.28	 11
  9	 V 	   493.27	   493.77	 10
 10	 S 	   536.79	   444.23	  9
 11	 V 	   586.32	   400.72	  8
 12	 A 	   621.84	   351.18	  7
 13	 P 	   670.37	   315.66	  6
 14	 G 	   698.88	   267.14	  5
 15	 E 	   763.40	   238.63	  4
 16	 T 	   813.92	   174.11	  3
 17	 A 	   849.44	   123.58	  2
 18	 R 	        -  	    88.06	  1  _
```

**89. IPI00953221 Isoform 6 of Extended synaptotagmin-2**

| Sequence | Charge | M+H+ | XCorr | DeltaCn |
| --- | --- | --- | --- | --- |
| R.APSPPRPGGPENPGGVLSVELPGLLAQLAR.S | 3 | 2948.369 | 3.399 | 0.286 |

+1 charge

```
		      B   	       Y
  1	 A 	    72.04	        -  	 30
  2	 P 	   169.10	  2875.58	 29
  3	 S 	   256.13	  2778.53	 28
  4	 P 	   353.18	  2691.49	 27
  5	 P 	   450.23	  2594.44	 26
  6	 R 	   606.34	  2497.39	 25
  7	 P 	   703.39	  2341.29	 24
  8	 G 	   760.41	  2244.23	 23
  9	 G 	   817.43	  2187.21	 22
 10	 P 	   914.48	  2130.19	 21
 11	 E 	  1043.53	  2033.14	 20
 12	 N 	  1157.57	  1904.10	 19
 13	 P 	  1254.62	  1790.05	 18
 14	 G 	  1311.64	  1693.00	 17
 15	 G 	  1368.67	  1635.98	 16
 16	 V 	  1467.73	  1578.96	 15
 17	 L 	  1580.82	  1479.89	 14
 18	 S 	  1667.85	  1366.81	 13
 19	 V 	  1766.92	  1279.77	 12
 20	 E 	  1895.96	  1180.70	 11
 21	 L 	  2009.05	  1051.66	 10
 22	 P 	  2106.10	   938.58	  9
 23	 G 	  2163.12	   841.53	  8
 24	 L 	  2276.20	   784.50	  7
 25	 L 	  2389.29	   671.42	  6
 26	 A 	  2460.32	   558.34	  5
 27	 Q 	  2588.38	   487.30	  4
 28	 L 	  2701.47	   359.24	  3
 29	 A 	  2772.50	   246.16	  2
 30	 R 	        -  	   175.12	  1  _


+2 charge
		      B   	       Y
  1	 A 	    36.53	        -  	 30
  2	 P 	    85.05	  1438.29	 29
  3	 S 	   128.57	  1389.77	 28
  4	 P 	   177.09	  1346.25	 27
  5	 P 	   225.62	  1297.72	 26
  6	 R 	   303.67	  1249.20	 25
  7	 P 	   352.20	  1171.15	 24
  8	 G 	   380.71	  1122.62	 23
  9	 G 	   409.22	  1094.11	 22
 10	 P 	   457.75	  1065.60	 21
 11	 E 	   522.27	  1017.07	 20
 12	 N 	   579.29	   952.55	 19
 13	 P 	   627.81	   895.53	 18
 14	 G 	   656.33	   847.00	 17
 15	 G 	   684.84	   818.49	 16
 16	 V 	   734.37	   789.98	 15
 17	 L 	   790.91	   740.45	 14
 18	 S 	   834.43	   683.91	 13
 19	 V 	   883.96	   640.39	 12
 20	 E 	   948.48	   590.86	 11
 21	 L 	  1005.03	   526.33	 10
 22	 P 	  1053.55	   469.79	  9
 23	 G 	  1082.06	   421.27	  8
 24	 L 	  1138.61	   392.76	  7
 25	 L 	  1195.15	   336.21	  6
 26	 A 	  1230.67	   279.67	  5
 27	 Q 	  1294.70	   244.15	  4
 28	 L 	  1351.24	   180.12	  3
 29	 A 	  1386.76	   123.58	  2
 30	 R 	        -  	    88.06	  1  _


+3 charge
		      B   	       Y
  1	 A 	    24.69	        -  	 30
  2	 P 	    57.04	   959.20	 29
  3	 S 	    86.05	   926.85	 28
  4	 P 	   118.40	   897.84	 27
  5	 P 	   150.75	   865.49	 26
  6	 R 	   202.78	   833.13	 25
  7	 P 	   235.13	   781.10	 24
  8	 G 	   254.14	   748.75	 23
  9	 G 	   273.15	   729.74	 22
 10	 P 	   305.50	   710.74	 21
 11	 E 	   348.51	   678.38	 20
 12	 N 	   386.53	   635.37	 19
 13	 P 	   418.88	   597.36	 18
 14	 G 	   437.89	   565.01	 17
 15	 G 	   456.89	   546.00	 16
 16	 V 	   489.92	   526.99	 15
 17	 L 	   527.61	   493.97	 14
 18	 S 	   556.62	   456.27	 13
 19	 V 	   589.64	   427.26	 12
 20	 E 	   632.66	   394.24	 11
 21	 L 	   670.35	   351.23	 10
 22	 P 	   702.70	   313.53	  9
 23	 G 	   721.71	   281.18	  8
 24	 L 	   759.41	   262.17	  7
 25	 L 	   797.10	   224.48	  6
 26	 A 	   820.78	   186.78	  5
 27	 Q 	   863.47	   163.10	  4
 28	 L 	   901.16	   120.42	  3
 29	 A 	   924.84	    82.72	  2
 30	 R 	        -  	    59.04	  1  _
```

**90. IPI00387119 Ig kappa chain V-III region POM**

| Sequence | Charge | M+H+ | XCorr | DeltaCn |
| --- | --- | --- | --- | --- |
| - .EIVMTQSPVTLSVSPGER.A | 2 | 1931.201 | 3.497 | 0.513 |

+1 charge

```
		      B   	       Y
  1	 E 	   130.05	        -  	 18
  2	 I 	   243.13	  1800.95	 17
  3	 V 	   342.20	  1687.87	 16
  4	 M 	   473.24	  1588.80	 15
  5	 T 	   574.29	  1457.76	 14
  6	 Q 	   702.35	  1356.71	 13
  7	 S 	   789.38	  1228.65	 12
  8	 P 	   886.43	  1141.62	 11
  9	 V 	   985.50	  1044.57	 10
 10	 T 	  1086.55	   945.50	  9
 11	 L 	  1199.63	   844.45	  8
 12	 S 	  1286.67	   731.37	  7
 13	 V 	  1385.73	   644.34	  6
 14	 S 	  1472.77	   545.27	  5
 15	 P 	  1569.82	   458.24	  4
 16	 G 	  1626.84	   361.18	  3
 17	 E 	  1755.88	   304.16	  2
 18	 R 	        -  	   175.12	  1  _
+2 charge
		      B   	       Y
  1	 E 	    65.53	        -  	 18
  2	 I 	   122.07	   900.98	 17
  3	 V 	   171.60	   844.44	 16
  4	 M 	   237.13	   794.90	 15
  5	 T 	   287.65	   729.38	 14
  6	 Q 	   351.68	   678.86	 13
  7	 S 	   395.19	   614.83	 12
  8	 P 	   443.72	   571.31	 11
  9	 V 	   493.25	   522.79	 10
 10	 T 	   543.78	   473.25	  9
 11	 L 	   600.32	   422.73	  8
 12	 S 	   643.84	   366.19	  7
 13	 V 	   693.37	   322.67	  6
 14	 S 	   736.89	   273.14	  5
 15	 P 	   785.41	   229.62	  4
 16	 G 	   813.92	   181.10	  3
 17	 E 	   878.45	   152.58	  2
 18	 R 	        -  	    88.06	  1  _
```

**91. IPI00292530 Inter-alpha-trypsin inhibitor heavy chain H1**

| Sequence | Charge | M+H+ | XCorr | DeltaCn |
| --- | --- | --- | --- | --- |
| R.GFSLDEATNLNGGLLR.G | 2 | 1677.84 | 2.942 | 0.383 |

+1 charge

```
		      B   	       Y
  1	 G 	    58.03	        -  	 16
  2	 F 	   205.10	  1619.84	 15
  3	 S 	   292.13	  1472.77	 14
  4	 L 	   405.21	  1385.74	 13
  5	 D 	   520.24	  1272.65	 12
  6	 E 	   649.28	  1157.63	 11
  7	 A 	   720.32	  1028.58	 10
  8	 T 	   821.37	   957.55	  9
  9	 N 	   935.41	   856.50	  8
 10	 L 	  1048.49	   742.46	  7
 11	 N 	  1162.54	   629.37	  6
 12	 G 	  1219.56	   515.33	  5
 13	 G 	  1276.58	   458.31	  4
 14	 L 	  1389.66	   401.29	  3
 15	 L 	  1502.75	   288.20	  2
 16	 R 	        -  	   175.12	  1  _
+2 charge
		      B   	       Y
  1	 G 	    29.52	        -  	 16
  2	 F 	   103.05	   810.42	 15
  3	 S 	   146.57	   736.89	 14
  4	 L 	   203.11	   693.37	 13
  5	 D 	   260.62	   636.83	 12
  6	 E 	   325.15	   579.32	 11
  7	 A 	   360.66	   514.80	 10
  8	 T 	   411.19	   479.28	  9
  9	 N 	   468.21	   428.75	  8
 10	 L 	   524.75	   371.73	  7
 11	 N 	   581.77	   315.19	  6
 12	 G 	   610.28	   258.17	  5
 13	 G 	   638.79	   229.66	  4
 14	 L 	   695.34	   201.15	  3
 15	 L 	   751.88	   144.61	  2
 16	 R 	        -  	    88.06	  1  _
```

**92. IPI00017987 Cornifin-A**

| Sequence | Charge | M+H+ | XCorr | DeltaCn |
| --- | --- | --- | --- | --- |
| K.VPEPCPSTVTPAPAQQK.T | 2 | 1808.019 | 4.679 | 0.581 |

+1 charge

```
		      B   	       Y
  1	 V 	   100.08	        -  	 17
  2	 P 	   197.13	  1707.97	 16
  3	 E 	   326.17	  1610.92	 15
  4	 P 	   423.22	  1481.88	 14
  5	 C 	   583.39	  1384.82	 13
  6	 P 	   680.44	  1224.66	 12
  7	 S 	   767.47	  1127.61	 11
  8	 T 	   868.52	  1040.57	 10
  9	 V 	   967.59	   939.53	  9
 10	 T 	  1068.64	   840.46	  8
 11	 P 	  1165.69	   739.41	  7
 12	 A 	  1236.73	   642.36	  6
 13	 P 	  1333.78	   571.32	  5
 14	 A 	  1404.82	   474.27	  4
 15	 Q 	  1532.88	   403.23	  3
 16	 Q 	  1660.93	   275.17	  2
 17	 K 	        -  	   147.11	  1  _
+2 charge
		      B   	       Y
  1	 V 	    50.54	        -  	 17
  2	 P 	    99.07	   854.49	 16
  3	 E 	   163.59	   805.96	 15
  4	 P 	   212.12	   741.44	 14
  5	 C 	   292.20	   692.92	 13
  6	 P 	   340.72	   612.83	 12
  7	 S 	   384.24	   564.31	 11
  8	 T 	   434.76	   520.79	 10
  9	 V 	   484.30	   470.27	  9
 10	 T 	   534.82	   420.73	  8
 11	 P 	   583.35	   370.21	  7
 12	 A 	   618.87	   321.68	  6
 13	 P 	   667.39	   286.16	  5
 14	 A 	   702.91	   237.64	  4
 15	 Q 	   766.94	   202.12	  3
 16	 Q 	   830.97	   138.09	  2
 17	 K 	        -  	    74.06	  1  _
```

**93. IPI00297252 Isoform 1 of Extracellular sulfatase Sulf-2**

| Sequence | Charge | M+H+ | XCorr | DeltaCn |
| --- | --- | --- | --- | --- |
| R.VYHVGLGDAAQPR.N | 2 | 1383.537 | 3.874 | 0.384 |

+1 charge

```
		      B   	       Y
  1	 V 	   100.08	        -  	 13
  2	 Y 	   263.14	  1283.65	 12
  3	 H 	   400.20	  1120.59	 11
  4	 V 	   499.27	   983.53	 10
  5	 G 	   556.29	   884.46	  9
  6	 L 	   669.37	   827.44	  8
  7	 G 	   726.39	   714.35	  7
  8	 D 	   841.42	   657.33	  6
  9	 A 	   912.46	   542.30	  5
 10	 A 	   983.49	   471.27	  4
 11	 Q 	  1111.55	   400.23	  3
 12	 P 	  1208.61	   272.17	  2
 13	 R 	        -  	   175.12	  1  _
+2 charge
		      B   	       Y
  1	 V 	    50.54	        -  	 13
  2	 Y 	   132.07	   642.33	 12
  3	 H 	   200.60	   560.80	 11
  4	 V 	   250.14	   492.27	 10
  5	 G 	   278.65	   442.73	  9
  6	 L 	   335.19	   414.22	  8
  7	 G 	   363.70	   357.68	  7
  8	 D 	   421.21	   329.17	  6
  9	 A 	   456.73	   271.66	  5
 10	 A 	   492.25	   236.14	  4
 11	 Q 	   556.28	   200.62	  3
 12	 P 	   604.81	   136.59	  2
 13	 R 	        -  	    88.06	  1  _
```

**94. IPI00028030 Cartilage oligomeric matrix protein**

| Sequence | Charge | M+H+ | XCorr | DeltaCn |
| --- | --- | --- | --- | --- |
| R.ELQETNAALQDVR.E | 2 | 1487.597 | 4.253 | 0.463 |

+1 charge

```
		      B   	       Y
  1	 E 	   130.05	        -  	 13
  2	 L 	   243.13	  1357.71	 12
  3	 Q 	   371.19	  1244.62	 11
  4	 E 	   500.24	  1116.56	 10
  5	 T 	   601.28	   987.52	  9
  6	 N 	   715.33	   886.47	  8
  7	 A 	   786.36	   772.43	  7
  8	 A 	   857.40	   701.39	  6
  9	 L 	   970.48	   630.36	  5
 10	 Q 	  1098.54	   517.27	  4
 11	 D 	  1213.57	   389.21	  3
 12	 V 	  1312.64	   274.19	  2
 13	 R 	        -  	   175.12	  1  _
+2 charge
		      B   	       Y
  1	 E 	    65.53	        -  	 13
  2	 L 	   122.07	   679.36	 12
  3	 Q 	   186.10	   622.82	 11
  4	 E 	   250.62	   558.79	 10
  5	 T 	   301.15	   494.26	  9
  6	 N 	   358.17	   443.74	  8
  7	 A 	   393.69	   386.72	  7
  8	 A 	   429.20	   351.20	  6
  9	 L 	   485.75	   315.68	  5
 10	 Q 	   549.77	   259.14	  4
 11	 D 	   607.29	   195.11	  3
 12	 V 	   656.82	   137.60	  2
 13	 R 	        -  	    88.06	  1  _
```

**95. IPI00329688 Protein YIPF3**

| Sequence | Charge | M+H+ | XCorr | DeltaCn |
| --- | --- | --- | --- | --- |
| R.DIPAMLPAAR.L | 1 | 1055.276 | 2.9 | 0.271 |

+1 charge

```
		      B   	       Y
  1	 D 	   116.03	        -  	 10
  2	 I 	   229.12	   939.54	  9
  3	 P 	   326.17	   826.46	  8
  4	 A 	   397.21	   729.41	  7
  5	 M 	   528.25	   658.37	  6
  6	 L 	   641.33	   527.33	  5
  7	 P 	   738.39	   414.25	  4
  8	 A 	   809.42	   317.19	  3
  9	 A 	   880.46	   246.16	  2
 10	 R 	        -  	   175.12	  1  _
```

**96. IPI00013972 Carcinoembryonic antigen-related cell adhesion molecule 8**

| Sequence | Charge | M+H+ | XCorr | DeltaCn |
| --- | --- | --- | --- | --- |
| R.IIGYVISNQQITPGPAYSNR.E | 2 | 2192.46 | 3.208 | 0.593 |

+1 charge

```
		      B   	       Y
  1	 I 	   114.09	        -  	 20
  2	 I 	   227.18	  2078.07	 19
  3	 G 	   284.20	  1964.98	 18
  4	 Y 	   447.26	  1907.96	 17
  5	 V 	   546.33	  1744.90	 16
  6	 I 	   659.41	  1645.83	 15
  7	 S 	   746.44	  1532.75	 14
  8	 N 	   860.49	  1445.71	 13
  9	 Q 	   988.55	  1331.67	 12
 10	 Q 	  1116.60	  1203.61	 11
 11	 I 	  1229.69	  1075.55	 10
 12	 T 	  1330.74	   962.47	  9
 13	 P 	  1427.79	   861.42	  8
 14	 G 	  1484.81	   764.37	  7
 15	 P 	  1581.86	   707.35	  6
 16	 A 	  1652.90	   610.29	  5
 17	 Y 	  1815.96	   539.26	  4
 18	 S 	  1903.00	   376.19	  3
 19	 N 	  2017.04	   289.16	  2
 20	 R 	        -  	   175.12	  1  _
+2 charge
		      B   	       Y
  1	 I 	    57.55	        -  	 20
  2	 I 	   114.09	  1039.54	 19
  3	 G 	   142.60	   982.99	 18
  4	 Y 	   224.13	   954.48	 17
  5	 V 	   273.67	   872.95	 16
  6	 I 	   330.21	   823.42	 15
  7	 S 	   373.73	   766.88	 14
  8	 N 	   430.75	   723.36	 13
  9	 Q 	   494.78	   666.34	 12
 10	 Q 	   558.81	   602.31	 11
 11	 I 	   615.35	   538.28	 10
 12	 T 	   665.87	   481.74	  9
 13	 P 	   714.40	   431.21	  8
 14	 G 	   742.91	   382.69	  7
 15	 P 	   791.44	   354.18	  6
 16	 A 	   826.95	   305.65	  5
 17	 Y 	   908.49	   270.13	  4
 18	 S 	   952.00	   188.60	  3
 19	 N 	  1009.02	   145.08	  2
 20	 R 	        -  	    88.06	  1  _
```

**97. IPI00419585 Peptidyl-prolyl cis-trans isomerase A**

| Sequence | Charge | M+H+ | XCorr | DeltaCn |
| --- | --- | --- | --- | --- |
| R.VSFELFADKVPK.T | 2 | 1380.613 | 3.2 | 0.464 |

+1 charge

```
		      B   	       Y
  1	 V 	   100.08	        -  	 12
  2	 S 	   187.11	  1280.69	 11
  3	 F 	   334.18	  1193.66	 10
  4	 E 	   463.22	  1046.59	  9
  5	 L 	   576.30	   917.55	  8
  6	 F 	   723.37	   804.46	  7
  7	 A 	   794.41	   657.39	  6
  8	 D 	   909.44	   586.36	  5
  9	 K 	  1037.53	   471.33	  4
 10	 V 	  1136.60	   343.23	  3
 11	 P 	  1233.65	   244.17	  2
 12	 K 	        -  	   147.11	  1  _
+2 charge
		      B   	       Y
  1	 V 	    50.54	        -  	 12
  2	 S 	    94.06	   640.85	 11
  3	 F 	   167.59	   597.33	 10
  4	 E 	   232.11	   523.80	  9
  5	 L 	   288.66	   459.28	  8
  6	 F 	   362.19	   402.73	  7
  7	 A 	   397.71	   329.20	  6
  8	 D 	   455.22	   293.68	  5
  9	 K 	   519.27	   236.17	  4
 10	 V 	   568.80	   172.12	  3
 11	 P 	   617.33	   122.59	  2
 12	 K 	        -  	    74.06	  1  _
```

**98. IPI00873840 Conserved hypothetical protein**

| Sequence | Charge | M+H+ | XCorr | DeltaCn |
| --- | --- | --- | --- | --- |
| K.ACLYSSKVKPQFHDQPCSPQSSSYGGR.P | 3 | 3073.309 | 3.57 | 0.202 |

+1 charge

```
		      B   	       Y
  1	 A 	    72.04	        -  	 27
  2	 C 	   232.21	  3000.64	 26
  3	 L 	   345.29	  2840.48	 25
  4	 Y 	   508.36	  2727.39	 24
  5	 S 	   595.39	  2564.33	 23
  6	 S 	   682.42	  2477.30	 22
  7	 K 	   810.52	  2390.27	 21
  8	 V 	   909.58	  2262.17	 20
  9	 K 	  1037.68	  2163.10	 19
 10	 P 	  1134.73	  2035.01	 18
 11	 Q 	  1262.79	  1937.95	 17
 12	 F 	  1409.86	  1809.90	 16
 13	 H 	  1546.92	  1662.83	 15
 14	 D 	  1661.95	  1525.77	 14
 15	 Q 	  1790.00	  1410.74	 13
 16	 P 	  1887.06	  1282.68	 12
 17	 C 	  2047.22	  1185.63	 11
 18	 S 	  2134.25	  1025.46	 10
 19	 P 	  2231.31	   938.43	  9
 20	 Q 	  2359.36	   841.38	  8
 21	 S 	  2446.40	   713.32	  7
 22	 S 	  2533.43	   626.29	  6
 23	 S 	  2620.46	   539.26	  5
 24	 Y 	  2783.52	   452.23	  4
 25	 G 	  2840.55	   289.16	  3
 26	 G 	  2897.57	   232.14	  2
 27	 R 	        -  	   175.12	  1  _


+2 charge
		      B   	       Y
  1	 A 	    36.53	        -  	 27
  2	 C 	   116.61	  1500.82	 26
  3	 L 	   173.15	  1420.74	 25
  4	 Y 	   254.68	  1364.20	 24
  5	 S 	   298.20	  1282.67	 23
  6	 S 	   341.71	  1239.15	 22
  7	 K 	   405.76	  1195.64	 21
  8	 V 	   455.30	  1131.59	 20
  9	 K 	   519.34	  1082.05	 19
 10	 P 	   567.87	  1018.01	 18
 11	 Q 	   631.90	   969.48	 17
 12	 F 	   705.43	   905.45	 16
 13	 H 	   773.96	   831.92	 15
 14	 D 	   831.48	   763.39	 14
 15	 Q 	   895.51	   705.87	 13
 16	 P 	   944.03	   641.84	 12
 17	 C 	  1024.11	   593.32	 11
 18	 S 	  1067.63	   513.24	 10
 19	 P 	  1116.16	   469.72	  9
 20	 Q 	  1180.19	   421.19	  8
 21	 S 	  1223.70	   357.16	  7
 22	 S 	  1267.22	   313.65	  6
 23	 S 	  1310.73	   270.13	  5
 24	 Y 	  1392.27	   226.62	  4
 25	 G 	  1420.78	   145.08	  3
 26	 G 	  1449.29	   116.57	  2
 27	 R 	        -  	    88.06	  1  _


+3 charge
		      B   	       Y
  1	 A 	    24.69	        -  	 27
  2	 C 	    78.07	  1000.89	 26
  3	 L 	   115.77	   947.50	 25
  4	 Y 	   170.12	   909.80	 24
  5	 S 	   199.13	   855.45	 23
  6	 S 	   228.15	   826.44	 22
  7	 K 	   270.84	   797.43	 21
  8	 V 	   303.87	   754.73	 20
  9	 K 	   346.56	   721.71	 19
 10	 P 	   378.92	   679.01	 18
 11	 Q 	   421.60	   646.66	 17
 12	 F 	   470.62	   603.97	 16
 13	 H 	   516.31	   554.95	 15
 14	 D 	   554.65	   509.26	 14
 15	 Q 	   597.34	   470.92	 13
 16	 P 	   629.69	   428.23	 12
 17	 C 	   683.08	   395.88	 11
 18	 S 	   712.09	   342.49	 10
 19	 P 	   744.44	   313.48	  9
 20	 Q 	   787.13	   281.13	  8
 21	 S 	   816.14	   238.45	  7
 22	 S 	   845.15	   209.43	  6
 23	 S 	   874.16	   180.42	  5
 24	 Y 	   928.51	   151.41	  4
 25	 G 	   947.52	    97.06	  3
 26	 G 	   966.53	    78.05	  2
 27	 R 	        -  	    59.04	  1  _
```

**99. IPI00022420 Retinol-binding protein 4**

| Sequence | Charge | M+H+ | XCorr | DeltaCn |
| --- | --- | --- | --- | --- |
| R.LLNLDGTCADSYSFVFSRDPNGLPPEAQK.I | 3 | 3213.493 | 3.465 | 0.515 |

+1 charge

```
		      B   	       Y
  1	 L 	   114.09	        -  	 29
  2	 L 	   227.18	  3098.59	 28
  3	 N 	   341.22	  2985.50	 27
  4	 L 	   454.30	  2871.46	 26
  5	 D 	   569.33	  2758.38	 25
  6	 G 	   626.35	  2643.35	 24
  7	 T 	   727.40	  2586.33	 23
  8	 C 	   887.56	  2485.28	 22
  9	 A 	   958.60	  2325.11	 21
 10	 D 	  1073.63	  2254.08	 20
 11	 S 	  1160.66	  2139.05	 19
 12	 Y 	  1323.72	  2052.02	 18
 13	 S 	  1410.76	  1888.96	 17
 14	 F 	  1557.82	  1801.92	 16
 15	 V 	  1656.89	  1654.85	 15
 16	 F 	  1803.96	  1555.79	 14
 17	 S 	  1890.99	  1408.72	 13
 18	 R 	  2047.09	  1321.69	 12
 19	 D 	  2162.12	  1165.58	 11
 20	 P 	  2259.17	  1050.56	 10
 21	 N 	  2373.22	   953.51	  9
 22	 G 	  2430.24	   839.46	  8
 23	 L 	  2543.32	   782.44	  7
 24	 P 	  2640.37	   669.36	  6
 25	 P 	  2737.43	   572.30	  5
 26	 E 	  2866.47	   475.25	  4
 27	 A 	  2937.51	   346.21	  3
 28	 Q 	  3065.57	   275.17	  2
 29	 K 	        -  	   147.11	  1  _


+2 charge
		      B   	       Y
  1	 L 	    57.55	        -  	 29
  2	 L 	   114.09	  1549.80	 28
  3	 N 	   171.11	  1493.26	 27
  4	 L 	   227.65	  1436.23	 26
  5	 D 	   285.17	  1379.69	 25
  6	 G 	   313.68	  1322.18	 24
  7	 T 	   364.20	  1293.67	 23
  8	 C 	   444.29	  1243.14	 22
  9	 A 	   479.80	  1163.06	 21
 10	 D 	   537.32	  1127.54	 20
 11	 S 	   580.83	  1070.03	 19
 12	 Y 	   662.37	  1026.51	 18
 13	 S 	   705.88	   944.98	 17
 14	 F 	   779.42	   901.47	 16
 15	 V 	   828.95	   827.93	 15
 16	 F 	   902.48	   778.40	 14
 17	 S 	   946.00	   704.86	 13
 18	 R 	  1024.05	   661.35	 12
 19	 D 	  1081.56	   583.30	 11
 20	 P 	  1130.09	   525.78	 10
 21	 N 	  1187.11	   477.26	  9
 22	 G 	  1215.62	   420.23	  8
 23	 L 	  1272.16	   391.72	  7
 24	 P 	  1320.69	   335.18	  6
 25	 P 	  1369.22	   286.66	  5
 26	 E 	  1433.74	   238.13	  4
 27	 A 	  1469.26	   173.61	  3
 28	 Q 	  1533.29	   138.09	  2
 29	 K 	        -  	    74.06	  1  _


+3 charge
		      B   	       Y
  1	 L 	    38.70	        -  	 29
  2	 L 	    76.40	  1033.53	 28
  3	 N 	   114.41	   995.84	 27
  4	 L 	   152.11	   957.82	 26
  5	 D 	   190.45	   920.13	 25
  6	 G 	   209.46	   881.79	 24
  7	 T 	   243.14	   862.78	 23
  8	 C 	   296.53	   829.10	 22
  9	 A 	   320.21	   775.71	 21
 10	 D 	   358.55	   752.03	 20
 11	 S 	   387.56	   713.69	 19
 12	 Y 	   441.91	   684.68	 18
 13	 S 	   470.92	   630.32	 17
 14	 F 	   519.95	   601.31	 16
 15	 V 	   552.97	   552.29	 15
 16	 F 	   601.99	   519.27	 14
 17	 S 	   631.00	   470.24	 13
 18	 R 	   683.04	   441.23	 12
 19	 D 	   721.38	   389.20	 11
 20	 P 	   753.73	   350.86	 10
 21	 N 	   791.74	   318.51	  9
 22	 G 	   810.75	   280.49	  8
 23	 L 	   848.45	   261.49	  7
 24	 P 	   880.80	   223.79	  6
 25	 P 	   913.15	   191.44	  5
 26	 E 	   956.16	   159.09	  4
 27	 A 	   979.84	   116.07	  3
 28	 Q 	  1022.53	    92.40	  2
 29	 K 	        -  	    49.71	  1  _
```

**100. IPI00000828 Proenkephalin-A**

| Sequence | Charge | M+H+ | XCorr | DeltaCn |
| --- | --- | --- | --- | --- |
| K.DAEEDDSLANSSDLLKELLETGDNRER.S | 3 | 3036.123 | 4.669 | 0.549 |

+1 charge

```
		      B   	       Y
  1	 D 	   116.03	        -  	 27
  2	 A 	   187.07	  2919.38	 26
  3	 E 	   316.11	  2848.34	 25
  4	 E 	   445.16	  2719.30	 24
  5	 D 	   560.18	  2590.26	 23
  6	 D 	   675.21	  2475.23	 22
  7	 S 	   762.24	  2360.21	 21
  8	 L 	   875.33	  2273.17	 20
  9	 A 	   946.36	  2160.09	 19
 10	 N 	  1060.41	  2089.05	 18
 11	 S 	  1147.44	  1975.01	 17
 12	 S 	  1234.47	  1887.98	 16
 13	 D 	  1349.50	  1800.95	 15
 14	 L 	  1462.58	  1685.92	 14
 15	 L 	  1575.67	  1572.83	 13
 16	 K 	  1703.76	  1459.75	 12
 17	 E 	  1832.80	  1331.65	 11
 18	 L 	  1945.89	  1202.61	 10
 19	 L 	  2058.97	  1089.53	  9
 20	 E 	  2188.01	   976.44	  8
 21	 T 	  2289.06	   847.40	  7
 22	 G 	  2346.08	   746.35	  6
 23	 D 	  2461.11	   689.33	  5
 24	 N 	  2575.15	   574.31	  4
 25	 R 	  2731.25	   460.26	  3
 26	 E 	  2860.30	   304.16	  2
 27	 R 	        -  	   175.12	  1  _


+2 charge
		      B   	       Y
  1	 D 	    58.52	        -  	 27
  2	 A 	    94.04	  1460.19	 26
  3	 E 	   158.56	  1424.68	 25
  4	 E 	   223.08	  1360.15	 24
  5	 D 	   280.60	  1295.63	 23
  6	 D 	   338.11	  1238.12	 22
  7	 S 	   381.62	  1180.61	 21
  8	 L 	   438.17	  1137.09	 20
  9	 A 	   473.69	  1080.55	 19
 10	 N 	   530.71	  1045.03	 18
 11	 S 	   574.22	   988.01	 17
 12	 S 	   617.74	   944.49	 16
 13	 D 	   675.25	   900.98	 15
 14	 L 	   731.79	   843.46	 14
 15	 L 	   788.34	   786.92	 13
 16	 K 	   852.38	   730.38	 12
 17	 E 	   916.91	   666.33	 11
 18	 L 	   973.45	   601.81	 10
 19	 L 	  1029.99	   545.27	  9
 20	 E 	  1094.51	   488.73	  8
 21	 T 	  1145.03	   424.20	  7
 22	 G 	  1173.55	   373.68	  6
 23	 D 	  1231.06	   345.17	  5
 24	 N 	  1288.08	   287.66	  4
 25	 R 	  1366.13	   230.63	  3
 26	 E 	  1430.65	   152.58	  2
 27	 R 	        -  	    88.06	  1  _


+3 charge
		      B   	       Y
  1	 D 	    39.35	        -  	 27
  2	 A 	    63.03	   973.80	 26
  3	 E 	   106.04	   950.12	 25
  4	 E 	   149.06	   907.11	 24
  5	 D 	   187.40	   864.09	 23
  6	 D 	   225.74	   825.75	 22
  7	 S 	   254.75	   787.41	 21
  8	 L 	   292.45	   758.40	 20
  9	 A 	   316.13	   720.70	 19
 10	 N 	   354.14	   697.02	 18
 11	 S 	   383.15	   659.01	 17
 12	 S 	   412.16	   630.00	 16
 13	 D 	   450.50	   600.99	 15
 14	 L 	   488.20	   562.64	 14
 15	 L 	   525.89	   524.95	 13
 16	 K 	   568.59	   487.25	 12
 17	 E 	   611.61	   444.56	 11
 18	 L 	   649.30	   401.54	 10
 19	 L 	   687.00	   363.85	  9
 20	 E 	   730.01	   326.15	  8
 21	 T 	   763.69	   283.14	  7
 22	 G 	   782.70	   249.46	  6
 23	 D 	   821.04	   230.45	  5
 24	 N 	   859.06	   192.11	  4
 25	 R 	   911.09	   154.09	  3
 26	 E 	   954.10	   102.06	  2
 27	 R 	        -  	    59.04	  1  _
```

**101. IPI00022331 Phosphatidylcholine-sterol acyltransferase**

| Sequence | Charge | M+H+ | XCorr | DeltaCn |
| --- | --- | --- | --- | --- |
| K.LAGYLHTLVQNLVNNGYVRDETVR.A | 3 | 2746.074 | 3.333 | 0.205 |

+1 charge

```
		      B   	       Y
  1	 L 	   114.09	        -  	 24
  2	 A 	   185.13	  2631.36	 23
  3	 G 	   242.15	  2560.33	 22
  4	 Y 	   405.21	  2503.31	 21
  5	 L 	   518.30	  2340.24	 20
  6	 H 	   655.36	  2227.16	 19
  7	 T 	   756.40	  2090.10	 18
  8	 L 	   869.49	  1989.05	 17
  9	 V 	   968.56	  1875.97	 16
 10	 Q 	  1096.61	  1776.90	 15
 11	 N 	  1210.66	  1648.84	 14
 12	 L 	  1323.74	  1534.80	 13
 13	 V 	  1422.81	  1421.71	 12
 14	 N 	  1536.85	  1322.64	 11
 15	 N 	  1650.90	  1208.60	 10
 16	 G 	  1707.92	  1094.56	  9
 17	 Y 	  1870.98	  1037.54	  8
 18	 V 	  1970.05	   874.47	  7
 19	 R 	  2126.15	   775.41	  6
 20	 D 	  2241.18	   619.30	  5
 21	 E 	  2370.22	   504.28	  4
 22	 T 	  2471.27	   375.24	  3
 23	 V 	  2570.34	   274.19	  2
 24	 R 	        -  	   175.12	  1  _


+2 charge
		      B   	       Y
  1	 L 	    57.55	        -  	 24
  2	 A 	    93.07	  1316.19	 23
  3	 G 	   121.58	  1280.67	 22
  4	 Y 	   203.11	  1252.16	 21
  5	 L 	   259.65	  1170.62	 20
  6	 H 	   328.18	  1114.08	 19
  7	 T 	   378.71	  1045.55	 18
  8	 L 	   435.25	   995.03	 17
  9	 V 	   484.78	   938.49	 16
 10	 Q 	   548.81	   888.95	 15
 11	 N 	   605.83	   824.92	 14
 12	 L 	   662.37	   767.90	 13
 13	 V 	   711.91	   711.36	 12
 14	 N 	   768.93	   661.83	 11
 15	 N 	   825.95	   604.80	 10
 16	 G 	   854.46	   547.78	  9
 17	 Y 	   935.99	   519.27	  8
 18	 V 	   985.53	   437.74	  7
 19	 R 	  1063.58	   388.21	  6
 20	 D 	  1121.09	   310.16	  5
 21	 E 	  1185.61	   252.64	  4
 22	 T 	  1236.14	   188.12	  3
 23	 V 	  1285.67	   137.60	  2
 24	 R 	        -  	    88.06	  1  _


+3 charge
		      B   	       Y
  1	 L 	    38.70	        -  	 24
  2	 A 	    62.38	   877.79	 23
  3	 G 	    81.39	   854.11	 22
  4	 Y 	   135.74	   835.11	 21
  5	 L 	   173.44	   780.75	 20
  6	 H 	   219.12	   743.06	 19
  7	 T 	   252.81	   697.37	 18
  8	 L 	   290.50	   663.69	 17
  9	 V 	   323.52	   625.99	 16
 10	 Q 	   366.21	   592.97	 15
 11	 N 	   404.22	   550.28	 14
 12	 L 	   441.92	   512.27	 13
 13	 V 	   474.94	   474.58	 12
 14	 N 	   512.96	   441.55	 11
 15	 N 	   550.97	   403.54	 10
 16	 G 	   569.98	   365.52	  9
 17	 Y 	   624.33	   346.52	  8
 18	 V 	   657.35	   292.16	  7
 19	 R 	   709.39	   259.14	  6
 20	 D 	   747.73	   207.11	  5
 21	 E 	   790.74	   168.76	  4
 22	 T 	   824.43	   125.75	  3
 23	 V 	   857.45	    92.07	  2
 24	 R 	        -  	    59.04	  1  _
```

**102. IPI00003590 Isoform 1 of Sulfhydryl oxidase 1**

| Sequence | Charge | M+H+ | XCorr | DeltaCn |
| --- | --- | --- | --- | --- |
| R.AAPGQEPPEHMAELQR.N | 2 | 1761.941 | 3.475 | 0.562 |

+1 charge

```
		      B   	       Y
  1	 A 	    72.04	        -  	 16
  2	 A 	   143.08	  1689.80	 15
  3	 P 	   240.13	  1618.76	 14
  4	 G 	   297.16	  1521.71	 13
  5	 Q 	   425.21	  1464.69	 12
  6	 E 	   554.26	  1336.63	 11
  7	 P 	   651.31	  1207.59	 10
  8	 P 	   748.36	  1110.54	  9
  9	 E 	   877.41	  1013.48	  8
 10	 H 	  1014.46	   884.44	  7
 11	 M 	  1145.50	   747.38	  6
 12	 A 	  1216.54	   616.34	  5
 13	 E 	  1345.58	   545.30	  4
 14	 L 	  1458.67	   416.26	  3
 15	 Q 	  1586.73	   303.18	  2
 16	 R 	        -  	   175.12	  1  _
+2 charge
		      B   	       Y
  1	 A 	    36.53	        -  	 16
  2	 A 	    72.04	   845.40	 15
  3	 P 	   120.57	   809.89	 14
  4	 G 	   149.08	   761.36	 13
  5	 Q 	   213.11	   732.85	 12
  6	 E 	   277.63	   668.82	 11
  7	 P 	   326.16	   604.30	 10
  8	 P 	   374.68	   555.77	  9
  9	 E 	   439.21	   507.25	  8
 10	 H 	   507.74	   442.72	  7
 11	 M 	   573.26	   374.19	  6
 12	 A 	   608.77	   308.67	  5
 13	 E 	   673.30	   273.16	  4
 14	 L 	   729.84	   208.63	  3
 15	 Q 	   793.87	   152.09	  2
 16	 R 	        -  	    88.06	  1  _
```

**103. IPI00022284 Major prion protein**

| Sequence | Charge | M+H+ | XCorr | DeltaCn |
| --- | --- | --- | --- | --- |
| R.VVEQMCITQYER.E | 2 | 1556.76 | 2.883 | 0.349 |

+1 charge

```
		      B   	       Y
  1	 V 	   100.08	        -  	 12
  2	 V 	   199.14	  1456.79	 11
  3	 E 	   328.19	  1357.72	 10
  4	 Q 	   456.25	  1228.68	  9
  5	 M 	   587.29	  1100.62	  8
  6	 C 	   747.45	   969.58	  7
  7	 I 	   860.54	   809.42	  6
  8	 T 	   961.58	   696.33	  5
  9	 Q 	  1089.64	   595.28	  4
 10	 Y 	  1252.70	   467.22	  3
 11	 E 	  1381.75	   304.16	  2
 12	 R 	        -  	   175.12	  1  _
+2 charge
		      B   	       Y
  1	 V 	    50.54	        -  	 12
  2	 V 	   100.08	   728.90	 11
  3	 E 	   164.60	   679.36	 10
  4	 Q 	   228.63	   614.84	  9
  5	 M 	   294.15	   550.81	  8
  6	 C 	   374.23	   485.29	  7
  7	 I 	   430.77	   405.21	  6
  8	 T 	   481.30	   348.67	  5
  9	 Q 	   545.32	   298.15	  4
 10	 Y 	   626.86	   234.12	  3
 11	 E 	   691.38	   152.58	  2
 12	 R 	        -  	    88.06	  1  _
```

**104. IPI00477714 V3-4 protein**

| Sequence | Charge | M+H+ | XCorr | DeltaCn |
| --- | --- | --- | --- | --- |
| R.SSGVPDRFSGSILGNK.A | 2 | 1621.776 | 3.361 | 0.277 |

+1 charge

```
		      B   	       Y
  1	 S 	    88.04	        -  	 16
  2	 S 	   175.07	  1533.80	 15
  3	 G 	   232.09	  1446.77	 14
  4	 V 	   331.16	  1389.75	 13
  5	 P 	   428.21	  1290.68	 12
  6	 D 	   543.24	  1193.63	 11
  7	 R 	   699.34	  1078.60	 10
  8	 F 	   846.41	   922.50	  9
  9	 S 	   933.44	   775.43	  8
 10	 G 	   990.46	   688.40	  7
 11	 S 	  1077.50	   631.38	  6
 12	 I 	  1190.58	   544.35	  5
 13	 L 	  1303.66	   431.26	  4
 14	 G 	  1360.69	   318.18	  3
 15	 N 	  1474.73	   261.16	  2
 16	 K 	        -  	   147.11	  1  _
+2 charge
		      B   	       Y
  1	 S 	    44.52	        -  	 16
  2	 S 	    88.04	   767.40	 15
  3	 G 	   116.55	   723.89	 14
  4	 V 	   166.08	   695.38	 13
  5	 P 	   214.61	   645.84	 12
  6	 D 	   272.12	   597.32	 11
  7	 R 	   350.17	   539.80	 10
  8	 F 	   423.71	   461.75	  9
  9	 S 	   467.22	   388.22	  8
 10	 G 	   495.74	   344.70	  7
 11	 S 	   539.25	   316.19	  6
 12	 I 	   595.79	   272.68	  5
 13	 L 	   652.34	   216.13	  4
 14	 G 	   680.85	   159.59	  3
 15	 N 	   737.87	   131.08	  2
 16	 K 	        -  	    74.06	  1  _
```

**105. IPI00382440 Ig lambda chain V-IV region Hil**

| Sequence | Charge | M+H+ | XCorr | DeltaCn |
| --- | --- | --- | --- | --- |
| - .SYELTQPPSVSVSPGQTAR.I | 2 | 2005.175 | 4.851 | 0.546 |

+1 charge

```
		      B   	       Y
  1	 S 	    88.04	        -  	 19
  2	 Y 	   251.10	  1916.97	 18
  3	 E 	   380.15	  1753.91	 17
  4	 L 	   493.23	  1624.87	 16
  5	 T 	   594.28	  1511.78	 15
  6	 Q 	   722.34	  1410.73	 14
  7	 P 	   819.39	  1282.67	 13
  8	 P 	   916.44	  1185.62	 12
  9	 S 	  1003.47	  1088.57	 11
 10	 V 	  1102.54	  1001.54	 10
 11	 S 	  1189.57	   902.47	  9
 12	 V 	  1288.64	   815.44	  8
 13	 S 	  1375.67	   716.37	  7
 14	 P 	  1472.73	   629.34	  6
 15	 G 	  1529.75	   532.28	  5
 16	 Q 	  1657.81	   475.26	  4
 17	 T 	  1758.85	   347.20	  3
 18	 A 	  1829.89	   246.16	  2
 19	 R 	        -  	   175.12	  1  _
+2 charge
		      B   	       Y
  1	 S 	    44.52	        -  	 19
  2	 Y 	   126.05	   958.99	 18
  3	 E 	   190.58	   877.46	 17
  4	 L 	   247.12	   812.94	 16
  5	 T 	   297.64	   756.39	 15
  6	 Q 	   361.67	   705.87	 14
  7	 P 	   410.20	   641.84	 13
  8	 P 	   458.72	   593.31	 12
  9	 S 	   502.24	   544.79	 11
 10	 V 	   551.77	   501.27	 10
 11	 S 	   595.29	   451.74	  9
 12	 V 	   644.82	   408.22	  8
 13	 S 	   688.34	   358.69	  7
 14	 P 	   736.87	   315.17	  6
 15	 G 	   765.38	   266.65	  5
 16	 Q 	   829.41	   238.13	  4
 17	 T 	   879.93	   174.11	  3
 18	 A 	   915.45	   123.58	  2
 19	 R 	        -  	    88.06	  1  _
```

**106. IPI00178926 Immunoglobulin J chain**

| Sequence | Charge | M+H+ | XCorr | DeltaCn |
| --- | --- | --- | --- | --- |
| R.FVYHLSDLCK.K | 2 | 1282.463 | 3.401 | 0.449 |

+1 charge

```
		      B   	       Y
  1	 F 	   148.08	        -  	 10
  2	 V 	   247.14	  1134.70	  9
  3	 Y 	   410.21	  1035.63	  8
  4	 H 	   547.27	   872.56	  7
  5	 L 	   660.35	   735.51	  6
  6	 S 	   747.38	   622.42	  5
  7	 D 	   862.41	   535.39	  4
  8	 L 	   975.49	   420.36	  3
  9	 C 	  1135.66	   307.28	  2
 10	 K 	        -  	   147.11	  1  _
+2 charge
		      B   	       Y
  1	 F 	    74.54	        -  	 10
  2	 V 	   124.08	   567.85	  9
  3	 Y 	   205.61	   518.32	  8
  4	 H 	   274.14	   436.79	  7
  5	 L 	   330.68	   368.26	  6
  6	 S 	   374.19	   311.71	  5
  7	 D 	   431.71	   268.20	  4
  8	 L 	   488.25	   210.68	  3
  9	 C 	   568.33	   154.14	  2
 10	 K 	        -  	    74.06	  1  _
```

**107. IPI00387120 Ig kappa chain V-IV region Len**

| Sequence | Charge | M+H+ | XCorr | DeltaCn |
| --- | --- | --- | --- | --- |
| - .DIVMTQSPDSLAVSLGER.A | 2 | 1919.147 | 5.66 | 0.536 |

+1 charge

```
		      B   	       Y
  1	 D 	   116.03	        -  	 18
  2	 I 	   229.12	  1802.93	 17
  3	 V 	   328.19	  1689.85	 16
  4	 M 	   459.23	  1590.78	 15
  5	 T 	   560.27	  1459.74	 14
  6	 Q 	   688.33	  1358.69	 13
  7	 S 	   775.37	  1230.63	 12
  8	 P 	   872.42	  1143.60	 11
  9	 D 	   987.45	  1046.55	 10
 10	 S 	  1074.48	   931.52	  9
 11	 L 	  1187.56	   844.49	  8
 12	 A 	  1258.60	   731.40	  7
 13	 V 	  1357.67	   660.37	  6
 14	 S 	  1444.70	   561.30	  5
 15	 L 	  1557.78	   474.27	  4
 16	 G 	  1614.80	   361.18	  3
 17	 E 	  1743.85	   304.16	  2
 18	 R 	        -  	   175.12	  1  _
+2 charge
		      B   	       Y
  1	 D 	    58.52	        -  	 18
  2	 I 	   115.06	   901.97	 17
  3	 V 	   164.60	   845.43	 16
  4	 M 	   230.12	   795.89	 15
  5	 T 	   280.64	   730.37	 14
  6	 Q 	   344.67	   679.85	 13
  7	 S 	   388.19	   615.82	 12
  8	 P 	   436.71	   572.30	 11
  9	 D 	   494.23	   523.78	 10
 10	 S 	   537.74	   466.26	  9
 11	 L 	   594.28	   422.75	  8
 12	 A 	   629.80	   366.21	  7
 13	 V 	   679.34	   330.69	  6
 14	 S 	   722.85	   281.15	  5
 15	 L 	   779.40	   237.64	  4
 16	 G 	   807.91	   181.10	  3
 17	 E 	   872.43	   152.58	  2
 18	 R 	        -  	    88.06	  1  _
```

**108. IPI00409698 Isoform 1 of Transportin-2**

| Sequence | Charge | M+H+ | XCorr | DeltaCn |
| --- | --- | --- | --- | --- |
| K.QECLNNIGDASSLIR.A | 2 | 1690.831 | 3.572 | 0.254 |

+1 charge

```
		      B   	       Y
  1	 Q 	   129.07	        -  	 15
  2	 E 	   258.11	  1561.90	 14
  3	 C 	   418.27	  1432.86	 13
  4	 L 	   531.36	  1272.69	 12
  5	 N 	   645.40	  1159.61	 11
  6	 N 	   759.44	  1045.56	 10
  7	 I 	   872.53	   931.52	  9
  8	 G 	   929.55	   818.44	  8
  9	 D 	  1044.58	   761.42	  7
 10	 A 	  1115.61	   646.39	  6
 11	 S 	  1202.65	   575.35	  5
 12	 S 	  1289.68	   488.32	  4
 13	 L 	  1402.76	   401.29	  3
 14	 I 	  1515.85	   288.20	  2
 15	 R 	        -  	   175.12	  1  _
+2 charge
		      B   	       Y
  1	 Q 	    65.04	        -  	 15
  2	 E 	   129.56	   781.45	 14
  3	 C 	   209.64	   716.93	 13
  4	 L 	   266.18	   636.85	 12
  5	 N 	   323.20	   580.31	 11
  6	 N 	   380.23	   523.29	 10
  7	 I 	   436.77	   466.26	  9
  8	 G 	   465.28	   409.72	  8
  9	 D 	   522.79	   381.21	  7
 10	 A 	   558.31	   323.70	  6
 11	 S 	   601.83	   288.18	  5
 12	 S 	   645.34	   244.66	  4
 13	 L 	   701.88	   201.15	  3
 14	 I 	   758.43	   144.61	  2
 15	 R 	        -  	    88.06	  1  _
```

**109. IPI00385255 Ig lambda chain V-II region NIG-84**

| Sequence | Charge | M+H+ | XCorr | DeltaCn |
| --- | --- | --- | --- | --- |
| K.LLIYDVNSRPSGISNR.F | 2 | 1805.028 | 2.961 | 0.314 |

+1 charge

```
		      B   	       Y
  1	 L 	   114.09	        -  	 16
  2	 L 	   227.18	  1690.89	 15
  3	 I 	   340.26	  1577.80	 14
  4	 Y 	   503.32	  1464.72	 13
  5	 D 	   618.35	  1301.66	 12
  6	 V 	   717.42	  1186.63	 11
  7	 N 	   831.46	  1087.56	 10
  8	 S 	   918.49	   973.52	  9
  9	 R 	  1074.59	   886.49	  8
 10	 P 	  1171.65	   730.38	  7
 11	 S 	  1258.68	   633.33	  6
 12	 G 	  1315.70	   546.30	  5
 13	 I 	  1428.78	   489.28	  4
 14	 S 	  1515.82	   376.19	  3
 15	 N 	  1629.86	   289.16	  2
 16	 R 	        -  	   175.12	  1  _
+2 charge
		      B   	       Y
  1	 L 	    57.55	        -  	 16
  2	 L 	   114.09	   845.95	 15
  3	 I 	   170.63	   789.41	 14
  4	 Y 	   252.17	   732.86	 13
  5	 D 	   309.68	   651.33	 12
  6	 V 	   359.21	   593.82	 11
  7	 N 	   416.23	   544.28	 10
  8	 S 	   459.75	   487.26	  9
  9	 R 	   537.80	   443.75	  8
 10	 P 	   586.33	   365.70	  7
 11	 S 	   629.84	   317.17	  6
 12	 G 	   658.35	   273.65	  5
 13	 I 	   714.90	   245.14	  4
 14	 S 	   758.41	   188.60	  3
 15	 N 	   815.43	   145.08	  2
 16	 R 	        -  	    88.06	  1  _
```

**110. IPI00016070 Glycerol-3-phosphate acyltransferase 4**

| Sequence | Charge | M+H+ | XCorr | DeltaCn |
| --- | --- | --- | --- | --- |
| K.SAIARQGGLVDLLWDGGLK.R | 2 | 1970.261 | 3.004 | 0.251 |

+1 charge

```
		      B   	       Y
  1	 S 	    88.04	        -  	 19
  2	 A 	   159.08	  1882.05	 18
  3	 I 	   272.16	  1811.02	 17
  4	 A 	   343.20	  1697.93	 16
  5	 R 	   499.30	  1626.90	 15
  6	 Q 	   627.36	  1470.80	 14
  7	 G 	   684.38	  1342.74	 13
  8	 G 	   741.40	  1285.72	 12
  9	 L 	   854.48	  1228.69	 11
 10	 V 	   953.55	  1115.61	 10
 11	 D 	  1068.58	  1016.54	  9
 12	 L 	  1181.66	   901.51	  8
 13	 L 	  1294.75	   788.43	  7
 14	 W 	  1480.83	   675.35	  6
 15	 D 	  1595.85	   489.27	  5
 16	 G 	  1652.88	   374.24	  4
 17	 G 	  1709.90	   317.22	  3
 18	 L 	  1822.98	   260.20	  2
 19	 K 	        -  	   147.11	  1  _
+2 charge
		      B   	       Y
  1	 S 	    44.52	        -  	 19
  2	 A 	    80.04	   941.53	 18
  3	 I 	   136.58	   906.01	 17
  4	 A 	   172.10	   849.47	 16
  5	 R 	   250.15	   813.95	 15
  6	 Q 	   314.18	   735.90	 14
  7	 G 	   342.69	   671.87	 13
  8	 G 	   371.20	   643.36	 12
  9	 L 	   427.75	   614.85	 11
 10	 V 	   477.28	   558.31	 10
 11	 D 	   534.79	   508.77	  9
 12	 L 	   591.34	   451.26	  8
 13	 L 	   647.88	   394.72	  7
 14	 W 	   740.92	   338.18	  6
 15	 D 	   798.43	   245.14	  5
 16	 G 	   826.94	   187.62	  4
 17	 G 	   855.45	   159.11	  3
 18	 L 	   911.99	   130.60	  2
 19	 K 	        -  	    74.06	  1  _
```

**111. IPI00480159 Isoform 1 of Phosphatidylinositide phosphatase SAC2**

| Sequence | Charge | M+H+ | XCorr | DeltaCn |
| --- | --- | --- | --- | --- |
| R.IYQIMWANNGDSISR.Q | 2 | 1768.975 | 3.413 | 0.283 |

+1 charge

```
		      B   	       Y
  1	 I 	   114.09	        -  	 15
  2	 Y 	   277.15	  1654.76	 14
  3	 Q 	   405.21	  1491.70	 13
  4	 I 	   518.30	  1363.64	 12
  5	 M 	   649.34	  1250.56	 11
  6	 W 	   835.42	  1119.52	 10
  7	 A 	   906.45	   933.44	  9
  8	 N 	  1020.50	   862.40	  8
  9	 N 	  1134.54	   748.36	  7
 10	 G 	  1191.56	   634.32	  6
 11	 D 	  1306.59	   577.29	  5
 12	 S 	  1393.62	   462.27	  4
 13	 I 	  1506.70	   375.24	  3
 14	 S 	  1593.74	   262.15	  2
 15	 R 	        -  	   175.12	  1  _
+2 charge
		      B   	       Y
  1	 I 	    57.55	        -  	 15
  2	 Y 	   139.08	   827.89	 14
  3	 Q 	   203.11	   746.35	 13
  4	 I 	   259.65	   682.32	 12
  5	 M 	   325.17	   625.78	 11
  6	 W 	   418.21	   560.26	 10
  7	 A 	   453.73	   467.22	  9
  8	 N 	   510.75	   431.70	  8
  9	 N 	   567.77	   374.68	  7
 10	 G 	   596.28	   317.66	  6
 11	 D 	   653.80	   289.15	  5
 12	 S 	   697.31	   231.64	  4
 13	 I 	   753.86	   188.12	  3
 14	 S 	   797.37	   131.58	  2
 15	 R 	        -  	    88.06	  1  _
```

**112. IPI00303161 Endothelial cell-selective adhesion molecule**

| Sequence | Charge | M+H+ | XCorr | DeltaCn |
| --- | --- | --- | --- | --- |
| R.QLPSFQTFFAPALDVIR.G | 2 | 1951.257 | 4.515 | 0.513 |

+1 charge

```
		      B   	       Y
  1	 Q 	   129.07	        -  	 17
  2	 L 	   242.15	  1821.99	 16
  3	 P 	   339.20	  1708.91	 15
  4	 S 	   426.23	  1611.85	 14
  5	 F 	   573.30	  1524.82	 13
  6	 Q 	   701.36	  1377.75	 12
  7	 T 	   802.41	  1249.69	 11
  8	 F 	   949.48	  1148.65	 10
  9	 F 	  1096.55	  1001.58	  9
 10	 A 	  1167.58	   854.51	  8
 11	 P 	  1264.64	   783.47	  7
 12	 A 	  1335.67	   686.42	  6
 13	 L 	  1448.76	   615.38	  5
 14	 D 	  1563.78	   502.30	  4
 15	 V 	  1662.85	   387.27	  3
 16	 I 	  1775.94	   288.20	  2
 17	 R 	        -  	   175.12	  1  _
+2 charge
		      B   	       Y
  1	 Q 	    65.04	        -  	 17
  2	 L 	   121.58	   911.50	 16
  3	 P 	   170.10	   854.96	 15
  4	 S 	   213.62	   806.43	 14
  5	 F 	   287.16	   762.91	 13
  6	 Q 	   351.18	   689.38	 12
  7	 T 	   401.71	   625.35	 11
  8	 F 	   475.24	   574.83	 10
  9	 F 	   548.78	   501.29	  9
 10	 A 	   584.30	   427.76	  8
 11	 P 	   632.82	   392.24	  7
 12	 A 	   668.34	   343.71	  6
 13	 L 	   724.88	   308.19	  5
 14	 D 	   782.40	   251.65	  4
 15	 V 	   831.93	   194.14	  3
 16	 I 	   888.47	   144.61	  2
 17	 R 	        -  	    88.06	  1  _
```

**113. IPI00029751 Isoform 1 of Contactin-1**

| Sequence | Charge | M+H+ | XCorr | DeltaCn |
| --- | --- | --- | --- | --- |
| R.IKTDGAAPNVAPSDVGGGGGR.N | 2 | 1897.039 | 3.538 | 0.368 |

+1 charge

```
		      B   	       Y
  1	 I 	   114.09	        -  	 21
  2	 K 	   242.19	  1782.87	 20
  3	 T 	   343.23	  1654.78	 19
  4	 D 	   458.26	  1553.73	 18
  5	 G 	   515.28	  1438.70	 17
  6	 A 	   586.32	  1381.68	 16
  7	 A 	   657.36	  1310.64	 15
  8	 P 	   754.41	  1239.61	 14
  9	 N 	   868.45	  1142.55	 13
 10	 V 	   967.52	  1028.51	 12
 11	 A 	  1038.56	   929.44	 11
 12	 P 	  1135.61	   858.41	 10
 13	 S 	  1222.64	   761.35	  9
 14	 D 	  1337.67	   674.32	  8
 15	 V 	  1436.74	   559.29	  7
 16	 G 	  1493.76	   460.23	  6
 17	 G 	  1550.78	   403.20	  5
 18	 G 	  1607.80	   346.18	  4
 19	 G 	  1664.82	   289.16	  3
 20	 G 	  1721.85	   232.14	  2
 21	 R 	        -  	   175.12	  1  _


+2 charge
		      B   	       Y
  1	 I 	    57.55	        -  	 21
  2	 K 	   121.60	   891.94	 20
  3	 T 	   172.12	   827.89	 19
  4	 D 	   229.63	   777.37	 18
  5	 G 	   258.14	   719.86	 17
  6	 A 	   293.66	   691.34	 16
  7	 A 	   329.18	   655.83	 15
  8	 P 	   377.71	   620.31	 14
  9	 N 	   434.73	   571.78	 13
 10	 V 	   484.26	   514.76	 12
 11	 A 	   519.78	   465.23	 11
 12	 P 	   568.31	   429.71	 10
 13	 S 	   611.82	   381.18	  9
 14	 D 	   669.34	   337.66	  8
 15	 V 	   718.87	   280.15	  7
 16	 G 	   747.38	   230.62	  6
 17	 G 	   775.89	   202.11	  5
 18	 G 	   804.40	   173.60	  4
 19	 G 	   832.92	   145.08	  3
 20	 G 	   861.43	   116.57	  2
 21	 R 	        -  	    88.06	  1  _
```

**114. IPI00017601 Ceruloplasmin**

| Sequence | Charge | M+H+ | XCorr | DeltaCn |
| --- | --- | --- | --- | --- |
| K.EVGPTNADPVCLAK.M | 2 | 1471.631 | 2.992 | 0.45 |

+1 charge

```
		      B   	       Y
  1	 E 	   130.05	        -  	 14
  2	 V 	   229.12	  1341.82	 13
  3	 G 	   286.14	  1242.75	 12
  4	 P 	   383.19	  1185.73	 11
  5	 T 	   484.24	  1088.68	 10
  6	 N 	   598.28	   987.63	  9
  7	 A 	   669.32	   873.58	  8
  8	 D 	   784.35	   802.55	  7
  9	 P 	   881.40	   687.52	  6
 10	 V 	   980.47	   590.47	  5
 11	 C 	  1140.63	   491.40	  4
 12	 L 	  1253.72	   331.23	  3
 13	 A 	  1324.75	   218.15	  2
 14	 K 	        -  	   147.11	  1  _
+2 charge
		      B   	       Y
  1	 E 	    65.53	        -  	 14
  2	 V 	   115.06	   671.41	 13
  3	 G 	   143.57	   621.88	 12
  4	 P 	   192.10	   593.37	 11
  5	 T 	   242.62	   544.84	 10
  6	 N 	   299.65	   494.32	  9
  7	 A 	   335.16	   437.30	  8
  8	 D 	   392.68	   401.78	  7
  9	 P 	   441.20	   344.26	  6
 10	 V 	   490.74	   295.74	  5
 11	 C 	   570.82	   246.20	  4
 12	 L 	   627.36	   166.12	  3
 13	 A 	   662.88	   109.58	  2
 14	 K 	        -  	    74.06	  1  _
```

**115. IPI00304903 Cornifin-B**

| Sequence | Charge | M+H+ | XCorr | DeltaCn |
| --- | --- | --- | --- | --- |
| K.VPEPCPSIVTPAPAQQK.T | 2 | 1820.073 | 4.177 | 0.563 |

+1 charge

```
		      B   	       Y
  1	 V 	   100.08	        -  	 17
  2	 P 	   197.13	  1720.01	 16
  3	 E 	   326.17	  1622.96	 15
  4	 P 	   423.22	  1493.91	 14
  5	 C 	   583.39	  1396.86	 13
  6	 P 	   680.44	  1236.69	 12
  7	 S 	   767.47	  1139.64	 11
  8	 I 	   880.56	  1052.61	 10
  9	 V 	   979.63	   939.53	  9
 10	 T 	  1080.67	   840.46	  8
 11	 P 	  1177.73	   739.41	  7
 12	 A 	  1248.76	   642.36	  6
 13	 P 	  1345.82	   571.32	  5
 14	 A 	  1416.85	   474.27	  4
 15	 Q 	  1544.91	   403.23	  3
 16	 Q 	  1672.97	   275.17	  2
 17	 K 	        -  	   147.11	  1  _
+2 charge
		      B   	       Y
  1	 V 	    50.54	        -  	 17
  2	 P 	    99.07	   860.51	 16
  3	 E 	   163.59	   811.98	 15
  4	 P 	   212.12	   747.46	 14
  5	 C 	   292.20	   698.93	 13
  6	 P 	   340.72	   618.85	 12
  7	 S 	   384.24	   570.32	 11
  8	 I 	   440.78	   526.81	 10
  9	 V 	   490.32	   470.27	  9
 10	 T 	   540.84	   420.73	  8
 11	 P 	   589.37	   370.21	  7
 12	 A 	   624.89	   321.68	  6
 13	 P 	   673.41	   286.16	  5
 14	 A 	   708.93	   237.64	  4
 15	 Q 	   772.96	   202.12	  3
 16	 Q 	   836.99	   138.09	  2
 17	 K 	        -  	    74.06	  1  _
```

**116. IPI00024331 Tumor necrosis factor receptor superfamily member 14**

| Sequence | Charge | M+H+ | XCorr | DeltaCn |
| --- | --- | --- | --- | --- |
| R.TENAVCGCSPGHFCIVQDGDHCAACR.A | 3 | 2980.121 | 4.383 | 0.536 |

+1 charge

```
		      B   	       Y
  1	 T 	   102.05	        -  	 26
  2	 E 	   231.10	  2877.81	 25
  3	 N 	   345.14	  2748.77	 24
  4	 A 	   416.18	  2634.73	 23
  5	 V 	   515.25	  2563.69	 22
  6	 C 	   675.41	  2464.62	 21
  7	 G 	   732.43	  2304.45	 20
  8	 C 	   892.60	  2247.43	 19
  9	 S 	   979.63	  2087.27	 18
 10	 P 	  1076.68	  2000.24	 17
 11	 G 	  1133.70	  1903.18	 16
 12	 H 	  1270.76	  1846.16	 15
 13	 F 	  1417.83	  1709.10	 14
 14	 C 	  1578.00	  1562.03	 13
 15	 I 	  1691.08	  1401.87	 12
 16	 V 	  1790.15	  1288.78	 11
 17	 Q 	  1918.21	  1189.72	 10
 18	 D 	  2033.23	  1061.66	  9
 19	 G 	  2090.26	   946.63	  8
 20	 D 	  2205.28	   889.61	  7
 21	 H 	  2342.34	   774.58	  6
 22	 C 	  2502.51	   637.52	  5
 23	 A 	  2573.54	   477.36	  4
 24	 A 	  2644.58	   406.32	  3
 25	 C 	  2804.75	   335.28	  2
 26	 R 	        -  	   175.12	  1  _


+2 charge
		      B   	       Y
  1	 T 	    51.53	        -  	 26
  2	 E 	   116.05	  1439.41	 25
  3	 N 	   173.07	  1374.89	 24
  4	 A 	   208.59	  1317.87	 23
  5	 V 	   258.13	  1282.35	 22
  6	 C 	   338.21	  1232.81	 21
  7	 G 	   366.72	  1152.73	 20
  8	 C 	   446.80	  1124.22	 19
  9	 S 	   490.32	  1044.14	 18
 10	 P 	   538.84	  1000.62	 17
 11	 G 	   567.36	   952.10	 16
 12	 H 	   635.89	   923.58	 15
 13	 F 	   709.42	   855.05	 14
 14	 C 	   789.50	   781.52	 13
 15	 I 	   846.04	   701.44	 12
 16	 V 	   895.58	   644.90	 11
 17	 Q 	   959.61	   595.36	 10
 18	 D 	  1017.12	   531.33	  9
 19	 G 	  1045.63	   473.82	  8
 20	 D 	  1103.15	   445.31	  7
 21	 H 	  1171.67	   387.79	  6
 22	 C 	  1251.76	   319.27	  5
 23	 A 	  1287.28	   239.18	  4
 24	 A 	  1322.79	   203.66	  3
 25	 C 	  1402.88	   168.15	  2
 26	 R 	        -  	    88.06	  1  _


+3 charge
		      B   	       Y
  1	 T 	    34.69	        -  	 26
  2	 E 	    77.70	   959.94	 25
  3	 N 	   115.72	   916.93	 24
  4	 A 	   139.40	   878.91	 23
  5	 V 	   172.42	   855.23	 22
  6	 C 	   225.81	   822.21	 21
  7	 G 	   244.82	   768.82	 20
  8	 C 	   298.20	   749.82	 19
  9	 S 	   327.21	   696.43	 18
 10	 P 	   359.57	   667.42	 17
 11	 G 	   378.57	   635.07	 16
 12	 H 	   424.26	   616.06	 15
 13	 F 	   473.28	   570.37	 14
 14	 C 	   526.67	   521.35	 13
 15	 I 	   564.37	   467.96	 12
 16	 V 	   597.39	   430.27	 11
 17	 Q 	   640.07	   397.24	 10
 18	 D 	   678.42	   354.56	  9
 19	 G 	   697.42	   316.22	  8
 20	 D 	   735.77	   297.21	  7
 21	 H 	   781.45	   258.87	  6
 22	 C 	   834.84	   213.18	  5
 23	 A 	   858.52	   159.79	  4
 24	 A 	   882.20	   136.11	  3
 25	 C 	   935.59	   112.43	  2
 26	 R 	        -  	    59.04	  1  _
```

**117. IPI00006971 Isoform 1 of Endosialin**

| Sequence | Charge | M+H+ | XCorr | DeltaCn |
| --- | --- | --- | --- | --- |
| R.TPEEAQRVDSLVGAGPASR.L | 2 | 1941.092 | 3.17 | 0.447 |

+1 charge

```
		      B   	       Y
  1	 T 	   102.05	        -  	 19
  2	 P 	   199.11	  1838.94	 18
  3	 E 	   328.15	  1741.88	 17
  4	 E 	   457.19	  1612.84	 16
  5	 A 	   528.23	  1483.80	 15
  6	 Q 	   656.29	  1412.76	 14
  7	 R 	   812.39	  1284.70	 13
  8	 V 	   911.46	  1128.60	 12
  9	 D 	  1026.49	  1029.53	 11
 10	 S 	  1113.52	   914.51	 10
 11	 L 	  1226.60	   827.47	  9
 12	 V 	  1325.67	   714.39	  8
 13	 G 	  1382.69	   615.32	  7
 14	 A 	  1453.73	   558.30	  6
 15	 G 	  1510.75	   487.26	  5
 16	 P 	  1607.80	   430.24	  4
 17	 A 	  1678.84	   333.19	  3
 18	 S 	  1765.87	   262.15	  2
 19	 R 	        -  	   175.12	  1  _
+2 charge
		      B   	       Y
  1	 T 	    51.53	        -  	 19
  2	 P 	   100.06	   919.97	 18
  3	 E 	   164.58	   871.45	 17
  4	 E 	   229.10	   806.92	 16
  5	 A 	   264.62	   742.40	 15
  6	 Q 	   328.65	   706.88	 14
  7	 R 	   406.70	   642.85	 13
  8	 V 	   456.23	   564.80	 12
  9	 D 	   513.75	   515.27	 11
 10	 S 	   557.26	   457.76	 10
 11	 L 	   613.80	   414.24	  9
 12	 V 	   663.34	   357.70	  8
 13	 G 	   691.85	   308.16	  7
 14	 A 	   727.37	   279.65	  6
 15	 G 	   755.88	   244.13	  5
 16	 P 	   804.40	   215.62	  4
 17	 A 	   839.92	   167.10	  3
 18	 S 	   883.44	   131.58	  2
 19	 R 	        -  	    88.06	  1  _
```

**118. IPI00021828 Cystatin-B**

| Sequence | Charge | M+H+ | XCorr | DeltaCn |
| --- | --- | --- | --- | --- |
| K.VHVGDEDFVHLR.V | 3 | 1423.558 | 3.645 | 0.446 |

+1 charge

```
		      B   	       Y
  1	 V 	   100.08	        -  	 12
  2	 H 	   237.13	  1323.64	 11
  3	 V 	   336.20	  1186.59	 10
  4	 G 	   393.22	  1087.52	  9
  5	 D 	   508.25	  1030.50	  8
  6	 E 	   637.29	   915.47	  7
  7	 D 	   752.32	   786.43	  6
  8	 F 	   899.39	   671.40	  5
  9	 V 	   998.46	   524.33	  4
 10	 H 	  1135.52	   425.26	  3
 11	 L 	  1248.60	   288.20	  2
 12	 R 	        -  	   175.12	  1  _
+2 charge
		      B   	       Y
  1	 V 	    50.54	        -  	 12
  2	 H 	   119.07	   662.33	 11
  3	 V 	   168.61	   593.80	 10
  4	 G 	   197.12	   544.26	  9
  5	 D 	   254.63	   515.75	  8
  6	 E 	   319.15	   458.24	  7
  7	 D 	   376.66	   393.72	  6
  8	 F 	   450.20	   336.20	  5
  9	 V 	   499.73	   262.67	  4
 10	 H 	   568.26	   213.13	  3
 11	 L 	   624.80	   144.61	  2
 12	 R 	        -  	    88.06	  1  _


+3 charge
		      B   	       Y
  1	 V 	    34.03	        -  	 12
  2	 H 	    79.72	   441.89	 11
  3	 V 	   112.74	   396.20	 10
  4	 G 	   131.75	   363.18	  9
  5	 D 	   170.09	   344.17	  8
  6	 E 	   213.10	   305.83	  7
  7	 D 	   251.45	   262.81	  6
  8	 F 	   300.47	   224.47	  5
  9	 V 	   333.49	   175.45	  4
 10	 H 	   379.18	   142.43	  3
 11	 L 	   416.87	    96.74	  2
 12	 R 	        -  	    59.04	  1  _
```

**119. IPI00218407 Fructose-bisphosphate aldolase B**

| Sequence | Charge | M+H+ | XCorr | DeltaCn |
| --- | --- | --- | --- | --- |
| R.IADQCPSSLAIQENANALAR.Y | 2 | 2143.337 | 6.056 | 0.497 |

+1 charge

```
		      B   	       Y
  1	 I 	   114.09	        -  	 20
  2	 A 	   185.13	  2029.11	 19
  3	 D 	   300.16	  1958.07	 18
  4	 Q 	   428.21	  1843.05	 17
  5	 C 	   588.38	  1714.99	 16
  6	 P 	   685.43	  1554.82	 15
  7	 S 	   772.46	  1457.77	 14
  8	 S 	   859.50	  1370.74	 13
  9	 L 	   972.58	  1283.71	 12
 10	 A 	  1043.62	  1170.62	 11
 11	 I 	  1156.70	  1099.59	 10
 12	 Q 	  1284.76	   986.50	  9
 13	 E 	  1413.80	   858.44	  8
 14	 N 	  1527.85	   729.40	  7
 15	 A 	  1598.88	   615.36	  6
 16	 N 	  1712.93	   544.32	  5
 17	 A 	  1783.96	   430.28	  4
 18	 L 	  1897.05	   359.24	  3
 19	 A 	  1968.08	   246.16	  2
 20	 R 	        -  	   175.12	  1  _
+2 charge
		      B   	       Y
  1	 I 	    57.55	        -  	 20
  2	 A 	    93.07	  1015.06	 19
  3	 D 	   150.58	   979.54	 18
  4	 Q 	   214.61	   922.03	 17
  5	 C 	   294.69	   858.00	 16
  6	 P 	   343.22	   777.92	 15
  7	 S 	   386.74	   729.39	 14
  8	 S 	   430.25	   685.87	 13
  9	 L 	   486.79	   642.36	 12
 10	 A 	   522.31	   585.81	 11
 11	 I 	   578.85	   550.30	 10
 12	 Q 	   642.88	   493.75	  9
 13	 E 	   707.40	   429.73	  8
 14	 N 	   764.43	   365.20	  7
 15	 A 	   799.94	   308.18	  6
 16	 N 	   856.97	   272.66	  5
 17	 A 	   892.48	   215.64	  4
 18	 L 	   949.03	   180.12	  3
 19	 A 	   984.55	   123.58	  2
 20	 R 	        -  	    88.06	  1  _
```

**120. IPI00008787 Alpha-N-acetylglucosaminidase**

| Sequence | Charge | M+H+ | XCorr | DeltaCn |
| --- | --- | --- | --- | --- |
| R.LLGPGPAADFSVSVER.A | 2 | 1615.812 | 4.312 | 0.528 |

+1 charge

```
		      B   	       Y
  1	 L 	   114.09	        -  	 16
  2	 L 	   227.18	  1501.76	 15
  3	 G 	   284.20	  1388.68	 14
  4	 P 	   381.25	  1331.66	 13
  5	 G 	   438.27	  1234.61	 12
  6	 P 	   535.32	  1177.58	 11
  7	 A 	   606.36	  1080.53	 10
  8	 A 	   677.40	  1009.49	  9
  9	 D 	   792.43	   938.46	  8
 10	 F 	   939.49	   823.43	  7
 11	 S 	  1026.53	   676.36	  6
 12	 V 	  1125.59	   589.33	  5
 13	 S 	  1212.63	   490.26	  4
 14	 V 	  1311.69	   403.23	  3
 15	 E 	  1440.74	   304.16	  2
 16	 R 	        -  	   175.12	  1  _
+2 charge
		      B   	       Y
  1	 L 	    57.55	        -  	 16
  2	 L 	   114.09	   751.39	 15
  3	 G 	   142.60	   694.84	 14
  4	 P 	   191.13	   666.33	 13
  5	 G 	   219.64	   617.81	 12
  6	 P 	   268.17	   589.30	 11
  7	 A 	   303.68	   540.77	 10
  8	 A 	   339.20	   505.25	  9
  9	 D 	   396.72	   469.73	  8
 10	 F 	   470.25	   412.22	  7
 11	 S 	   513.77	   338.68	  6
 12	 V 	   563.30	   295.17	  5
 13	 S 	   606.82	   245.63	  4
 14	 V 	   656.35	   202.12	  3
 15	 E 	   720.87	   152.58	  2
 16	 R 	        -  	    88.06	  1  _
```

**121. IPI00003865 Isoform 1 of Heat shock cognate 71 kDa protein**

| Sequence | Charge | M+H+ | XCorr | DeltaCn |
| --- | --- | --- | --- | --- |
| R.ARFEELNADLFR.G | 2 | 1481.638 | 2.802 | 0.315 |

+1 charge

```
		      B   	       Y
  1	 A 	    72.04	        -  	 12
  2	 R 	   228.15	  1409.72	 11
  3	 F 	   375.21	  1253.62	 10
  4	 E 	   504.26	  1106.55	  9
  5	 E 	   633.30	   977.51	  8
  6	 L 	   746.38	   848.46	  7
  7	 N 	   860.43	   735.38	  6
  8	 A 	   931.46	   621.34	  5
  9	 D 	  1046.49	   550.30	  4
 10	 L 	  1159.57	   435.27	  3
 11	 F 	  1306.64	   322.19	  2
 12	 R 	        -  	   175.12	  1  _
+2 charge
		      B   	       Y
  1	 A 	    36.53	        -  	 12
  2	 R 	   114.58	   705.36	 11
  3	 F 	   188.11	   627.31	 10
  4	 E 	   252.63	   553.78	  9
  5	 E 	   317.15	   489.26	  8
  6	 L 	   373.70	   424.73	  7
  7	 N 	   430.72	   368.19	  6
  8	 A 	   466.24	   311.17	  5
  9	 D 	   523.75	   275.65	  4
 10	 L 	   580.29	   218.14	  3
 11	 F 	   653.82	   161.60	  2
 12	 R 	        -  	    88.06	  1  _
```

**122. IPI00170635 Secreted and transmembrane protein 1**

| Sequence | Charge | M+H+ | XCorr | DeltaCn |
| --- | --- | --- | --- | --- |
| R.AHGQESAIFNEVAPGYFSR.D | 2 | 2081.233 | 4.78 | 0.617 |

+1 charge

```
		      B   	       Y
  1	 A 	    72.04	        -  	 19
  2	 H 	   209.10	  2008.95	 18
  3	 G 	   266.12	  1871.89	 17
  4	 Q 	   394.18	  1814.87	 16
  5	 E 	   523.23	  1686.81	 15
  6	 S 	   610.26	  1557.77	 14
  7	 A 	   681.30	  1470.74	 13
  8	 I 	   794.38	  1399.70	 12
  9	 F 	   941.45	  1286.62	 11
 10	 N 	  1055.49	  1139.55	 10
 11	 E 	  1184.53	  1025.51	  9
 12	 V 	  1283.60	   896.46	  8
 13	 A 	  1354.64	   797.39	  7
 14	 P 	  1451.69	   726.36	  6
 15	 G 	  1508.71	   629.30	  5
 16	 Y 	  1671.78	   572.28	  4
 17	 F 	  1818.84	   409.22	  3
 18	 S 	  1905.88	   262.15	  2
 19	 R 	        -  	   175.12	  1  _
+2 charge
		      B   	       Y
  1	 A 	    36.53	        -  	 19
  2	 H 	   105.06	  1004.98	 18
  3	 G 	   133.57	   936.45	 17
  4	 Q 	   197.60	   907.94	 16
  5	 E 	   262.12	   843.91	 15
  6	 S 	   305.63	   779.39	 14
  7	 A 	   341.15	   735.87	 13
  8	 I 	   397.69	   700.35	 12
  9	 F 	   471.23	   643.81	 11
 10	 N 	   528.25	   570.28	 10
 11	 E 	   592.77	   513.26	  9
 12	 V 	   642.30	   448.73	  8
 13	 A 	   677.82	   399.20	  7
 14	 P 	   726.35	   363.68	  6
 15	 G 	   754.86	   315.16	  5
 16	 Y 	   836.39	   286.64	  4
 17	 F 	   909.93	   205.11	  3
 18	 S 	   953.44	   131.58	  2
 19	 R 	        -  	    88.06	  1  _
```

**123. IPI00005774 Isoform 1 of Low-density lipoprotein receptor-related protein 8**

| Sequence | Charge | M+H+ | XCorr | DeltaCn |
| --- | --- | --- | --- | --- |
| R.CYRAPQSTSTTTLASTMTR.T | 2 | 2134.351 | 3.183 | 0.216 |

+1 charge

```
		      B   	       Y
  1	 C 	   161.17	        -  	 19
  2	 Y 	   324.24	  1972.98	 18
  3	 R 	   480.34	  1809.91	 17
  4	 A 	   551.37	  1653.81	 16
  5	 P 	   648.43	  1582.77	 15
  6	 Q 	   776.49	  1485.72	 14
  7	 S 	   863.52	  1357.66	 13
  8	 T 	   964.57	  1270.63	 12
  9	 S 	  1051.60	  1169.58	 11
 10	 T 	  1152.64	  1082.55	 10
 11	 T 	  1253.69	   981.50	  9
 12	 T 	  1354.74	   880.46	  8
 13	 L 	  1467.82	   779.41	  7
 14	 A 	  1538.86	   666.32	  6
 15	 S 	  1625.89	   595.29	  5
 16	 T 	  1726.94	   508.25	  4
 17	 M 	  1857.98	   407.21	  3
 18	 T 	  1959.03	   276.17	  2
 19	 R 	        -  	   175.12	  1  _
+2 charge
		      B   	       Y
  1	 C 	    81.09	        -  	 19
  2	 Y 	   162.62	   986.99	 18
  3	 R 	   240.67	   905.46	 17
  4	 A 	   276.19	   827.41	 16
  5	 P 	   324.72	   791.89	 15
  6	 Q 	   388.75	   743.36	 14
  7	 S 	   432.26	   679.34	 13
  8	 T 	   482.79	   635.82	 12
  9	 S 	   526.30	   585.30	 11
 10	 T 	   576.83	   541.78	 10
 11	 T 	   627.35	   491.26	  9
 12	 T 	   677.87	   440.73	  8
 13	 L 	   734.42	   390.21	  7
 14	 A 	   769.93	   333.67	  6
 15	 S 	   813.45	   298.15	  5
 16	 T 	   863.97	   254.63	  4
 17	 M 	   929.49	   204.11	  3
 18	 T 	   980.02	   138.59	  2
 19	 R 	        -  	    88.06	  1  _
```

**124. IPI00291866 Plasma protease C1 inhibitor**

| Sequence | Charge | M+H+ | XCorr | DeltaCn |
| --- | --- | --- | --- | --- |
| K.GVTSVSQIFHSPDLAIR.D | 2 | 1828.062 | 4.028 | 0.47 |

+1 charge

```
		      B   	       Y
  1	 G 	    58.03	        -  	 17
  2	 V 	   157.10	  1769.95	 16
  3	 T 	   258.14	  1670.89	 15
  4	 S 	   345.18	  1569.84	 14
  5	 V 	   444.25	  1482.81	 13
  6	 S 	   531.28	  1383.74	 12
  7	 Q 	   659.34	  1296.71	 11
  8	 I 	   772.42	  1168.65	 10
  9	 F 	   919.49	  1055.56	  9
 10	 H 	  1056.55	   908.49	  8
 11	 S 	  1143.58	   771.44	  7
 12	 P 	  1240.63	   684.40	  6
 13	 D 	  1355.66	   587.35	  5
 14	 L 	  1468.74	   472.32	  4
 15	 A 	  1539.78	   359.24	  3
 16	 I 	  1652.86	   288.20	  2
 17	 R 	        -  	   175.12	  1  _
+2 charge
		      B   	       Y
  1	 G 	    29.52	        -  	 17
  2	 V 	    79.05	   885.48	 16
  3	 T 	   129.58	   835.95	 15
  4	 S 	   173.09	   785.42	 14
  5	 V 	   222.63	   741.91	 13
  6	 S 	   266.14	   692.37	 12
  7	 Q 	   330.17	   648.86	 11
  8	 I 	   386.71	   584.83	 10
  9	 F 	   460.25	   528.29	  9
 10	 H 	   528.78	   454.75	  8
 11	 S 	   572.29	   386.22	  7
 12	 P 	   620.82	   342.71	  6
 13	 D 	   678.33	   294.18	  5
 14	 L 	   734.88	   236.67	  4
 15	 A 	   770.39	   180.12	  3
 16	 I 	   826.94	   144.61	  2
 17	 R 	        -  	    88.06	  1  _
```

**125. IPI00003091 Isoform 1 of Protein phosphatase 1 regulatory subunit 3A**

| Sequence | Charge | M+H+ | XCorr | DeltaCn |
| --- | --- | --- | --- | --- |
| K.EQEPEPVKPWKEVPNR.Q | 2 | 1963.183 | 3.125 | 0.243 |

+1 charge

```
		      B   	       Y
  1	 E 	   130.05	        -  	 16
  2	 Q 	   258.11	  1832.97	 15
  3	 E 	   387.15	  1704.91	 14
  4	 P 	   484.20	  1575.86	 13
  5	 E 	   613.25	  1478.81	 12
  6	 P 	   710.30	  1349.77	 11
  7	 V 	   809.37	  1252.72	 10
  8	 K 	   937.46	  1153.65	  9
  9	 P 	  1034.52	  1025.55	  8
 10	 W 	  1220.59	   928.50	  7
 11	 K 	  1348.69	   742.42	  6
 12	 E 	  1477.73	   614.33	  5
 13	 V 	  1576.80	   485.28	  4
 14	 P 	  1673.85	   386.21	  3
 15	 N 	  1787.90	   289.16	  2
 16	 R 	        -  	   175.12	  1  _
+2 charge
		      B   	       Y
  1	 E 	    65.53	        -  	 16
  2	 Q 	   129.56	   916.99	 15
  3	 E 	   194.08	   852.96	 14
  4	 P 	   242.61	   788.44	 13
  5	 E 	   307.13	   739.91	 12
  6	 P 	   355.65	   675.39	 11
  7	 V 	   405.19	   626.86	 10
  8	 K 	   469.23	   577.33	  9
  9	 P 	   517.76	   513.28	  8
 10	 W 	   610.80	   464.75	  7
 11	 K 	   674.85	   371.71	  6
 12	 E 	   739.37	   307.67	  5
 13	 V 	   788.90	   243.15	  4
 14	 P 	   837.43	   193.61	  3
 15	 N 	   894.45	   145.08	  2
 16	 R 	        -  	    88.06	  1  _
```

**126. IPI00005732 Isoform 1 of Activin receptor type-1B**

| Sequence | Charge | M+H+ | XCorr | DeltaCn |
| --- | --- | --- | --- | --- |
| R.TCIPKVELVPAGKPFYCLSSEDLR.N | 3 | 2781.184 | 4.339 | 0.583 |

+1 charge

```
		      B   	       Y
  1	 T 	   102.05	        -  	 24
  2	 C 	   262.22	  2678.64	 23
  3	 I 	   375.30	  2518.47	 22
  4	 P 	   472.36	  2405.39	 21
  5	 K 	   600.45	  2308.34	 20
  6	 V 	   699.52	  2180.24	 19
  7	 E 	   828.56	  2081.17	 18
  8	 L 	   941.65	  1952.13	 17
  9	 V 	  1040.72	  1839.05	 16
 10	 P 	  1137.77	  1739.98	 15
 11	 A 	  1208.81	  1642.92	 14
 12	 G 	  1265.83	  1571.89	 13
 13	 K 	  1393.92	  1514.87	 12
 14	 P 	  1490.97	  1386.77	 11
 15	 F 	  1638.04	  1289.72	 10
 16	 Y 	  1801.11	  1142.65	  9
 17	 C 	  1961.27	   979.59	  8
 18	 L 	  2074.36	   819.42	  7
 19	 S 	  2161.39	   706.34	  6
 20	 S 	  2248.42	   619.30	  5
 21	 E 	  2377.46	   532.27	  4
 22	 D 	  2492.49	   403.23	  3
 23	 L 	  2605.57	   288.20	  2
 24	 R 	        -  	   175.12	  1  _


+2 charge
		      B   	       Y
  1	 T 	    51.53	        -  	 24
  2	 C 	   131.61	  1339.82	 23
  3	 I 	   188.16	  1259.74	 22
  4	 P 	   236.68	  1203.20	 21
  5	 K 	   300.73	  1154.67	 20
  6	 V 	   350.26	  1090.62	 19
  7	 E 	   414.79	  1041.09	 18
  8	 L 	   471.33	   976.57	 17
  9	 V 	   520.86	   920.03	 16
 10	 P 	   569.39	   870.49	 15
 11	 A 	   604.91	   821.97	 14
 12	 G 	   633.42	   786.45	 13
 13	 K 	   697.46	   757.94	 12
 14	 P 	   745.99	   693.89	 11
 15	 F 	   819.53	   645.36	 10
 16	 Y 	   901.06	   571.83	  9
 17	 C 	   981.14	   490.30	  8
 18	 L 	  1037.68	   410.21	  7
 19	 S 	  1081.20	   353.67	  6
 20	 S 	  1124.71	   310.16	  5
 21	 E 	  1189.23	   266.64	  4
 22	 D 	  1246.75	   202.12	  3
 23	 L 	  1303.29	   144.61	  2
 24	 R 	        -  	    88.06	  1  _


+3 charge
		      B   	       Y
  1	 T 	    34.69	        -  	 24
  2	 C 	    88.08	   893.55	 23
  3	 I 	   125.77	   840.16	 22
  4	 P 	   158.12	   802.47	 21
  5	 K 	   200.82	   770.12	 20
  6	 V 	   233.84	   727.42	 19
  7	 E 	   276.86	   694.40	 18
  8	 L 	   314.55	   651.38	 17
  9	 V 	   347.58	   613.69	 16
 10	 P 	   379.93	   580.66	 15
 11	 A 	   403.61	   548.31	 14
 12	 G 	   422.61	   524.63	 13
 13	 K 	   465.31	   505.63	 12
 14	 P 	   497.66	   462.93	 11
 15	 F 	   546.69	   430.58	 10
 16	 Y 	   601.04	   381.55	  9
 17	 C 	   654.43	   327.20	  8
 18	 L 	   692.12	   273.81	  7
 19	 S 	   721.13	   236.12	  6
 20	 S 	   750.14	   207.11	  5
 21	 E 	   793.16	   178.10	  4
 22	 D 	   831.50	   135.08	  3
 23	 L 	   869.20	    96.74	  2
 24	 R 	        -  	    59.04	  1  _
```

**127. IPI00025846 Isoform 2A of Desmocollin-2**

| Sequence | Charge | M+H+ | XCorr | DeltaCn |
| --- | --- | --- | --- | --- |
| R.LSYQNDPPFGSYVVPITVR.D | 2 | 2153.422 | 3.948 | 0.559 |

+1 charge

```
		      B   	       Y
  1	 L 	   114.09	        -  	 19
  2	 S 	   201.12	  2039.02	 18
  3	 Y 	   364.19	  1951.99	 17
  4	 Q 	   492.25	  1788.93	 16
  5	 N 	   606.29	  1660.87	 15
  6	 D 	   721.32	  1546.83	 14
  7	 P 	   818.37	  1431.80	 13
  8	 P 	   915.42	  1334.75	 12
  9	 F 	  1062.49	  1237.69	 11
 10	 G 	  1119.51	  1090.63	 10
 11	 S 	  1206.54	  1033.60	  9
 12	 Y 	  1369.61	   946.57	  8
 13	 V 	  1468.67	   783.51	  7
 14	 V 	  1567.74	   684.44	  6
 15	 P 	  1664.80	   585.37	  5
 16	 I 	  1777.88	   488.32	  4
 17	 T 	  1878.93	   375.24	  3
 18	 V 	  1978.00	   274.19	  2
 19	 R 	        -  	   175.12	  1  _
+2 charge
		      B   	       Y
  1	 L 	    57.55	        -  	 19
  2	 S 	   101.07	  1020.02	 18
  3	 Y 	   182.60	   976.50	 17
  4	 Q 	   246.63	   894.97	 16
  5	 N 	   303.65	   830.94	 15
  6	 D 	   361.16	   773.92	 14
  7	 P 	   409.69	   716.40	 13
  8	 P 	   458.21	   667.88	 12
  9	 F 	   531.75	   619.35	 11
 10	 G 	   560.26	   545.82	 10
 11	 S 	   603.77	   517.31	  9
 12	 Y 	   685.31	   473.79	  8
 13	 V 	   734.84	   392.26	  7
 14	 V 	   784.38	   342.72	  6
 15	 P 	   832.90	   293.19	  5
 16	 I 	   889.44	   244.66	  4
 17	 T 	   939.97	   188.12	  3
 18	 V 	   989.50	   137.60	  2
 19	 R 	        -  	    88.06	  1  _
```

**128. IPI00240345 C-type lectin domain family 14 member A**

| Sequence | Charge | M+H+ | XCorr | DeltaCn |
| --- | --- | --- | --- | --- |
| R.GQLPISVTCIADEIGAR.W | 2 | 1801.028 | 4.386 | 0.544 |

+1 charge

```
		      B   	       Y
  1	 G 	    58.03	        -  	 17
  2	 Q 	   186.09	  1743.05	 16
  3	 L 	   299.17	  1614.99	 15
  4	 P 	   396.22	  1501.90	 14
  5	 I 	   509.31	  1404.85	 13
  6	 S 	   596.34	  1291.77	 12
  7	 V 	   695.41	  1204.73	 11
  8	 T 	   796.46	  1105.67	 10
  9	 C 	   956.62	  1004.62	  9
 10	 I 	  1069.71	   844.45	  8
 11	 A 	  1140.74	   731.37	  7
 12	 D 	  1255.77	   660.33	  6
 13	 E 	  1384.81	   545.30	  5
 14	 I 	  1497.90	   416.26	  4
 15	 G 	  1554.92	   303.18	  3
 16	 A 	  1625.95	   246.16	  2
 17	 R 	        -  	   175.12	  1  _
+2 charge
		      B   	       Y
  1	 G 	    29.52	        -  	 17
  2	 Q 	    93.55	   872.03	 16
  3	 L 	   150.09	   808.00	 15
  4	 P 	   198.62	   751.45	 14
  5	 I 	   255.16	   702.93	 13
  6	 S 	   298.67	   646.39	 12
  7	 V 	   348.21	   602.87	 11
  8	 T 	   398.73	   553.34	 10
  9	 C 	   478.81	   502.81	  9
 10	 I 	   535.36	   422.73	  8
 11	 A 	   570.88	   366.19	  7
 12	 D 	   628.39	   330.67	  6
 13	 E 	   692.91	   273.16	  5
 14	 I 	   749.45	   208.63	  4
 15	 G 	   777.96	   152.09	  3
 16	 A 	   813.48	   123.58	  2
 17	 R 	        -  	    88.06	  1  _
```

**129. IPI00020091 Alpha-1-acid glycoprotein 2**

| Sequence | Charge | M+H+ | XCorr | DeltaCn |
| --- | --- | --- | --- | --- |
| K.EQLGEFYEALDCLCIPR.S | 2 | 2114.328 | 3.326 | 0.536 |

+1 charge

```
		      B   	       Y
  1	 E 	   130.05	        -  	 17
  2	 Q 	   258.11	  1984.20	 16
  3	 L 	   371.19	  1856.14	 15
  4	 G 	   428.21	  1743.06	 14
  5	 E 	   557.26	  1686.04	 13
  6	 F 	   704.32	  1556.99	 12
  7	 Y 	   867.39	  1409.92	 11
  8	 E 	   996.43	  1246.86	 10
  9	 A 	  1067.47	  1117.82	  9
 10	 L 	  1180.55	  1046.78	  8
 11	 D 	  1295.58	   933.70	  7
 12	 C 	  1455.74	   818.67	  6
 13	 L 	  1568.83	   658.51	  5
 14	 C 	  1728.99	   545.42	  4
 15	 I 	  1842.08	   385.26	  3
 16	 P 	  1939.13	   272.17	  2
 17	 R 	        -  	   175.12	  1  _
+2 charge
		      B   	       Y
  1	 E 	    65.53	        -  	 17
  2	 Q 	   129.56	   992.60	 16
  3	 L 	   186.10	   928.57	 15
  4	 G 	   214.61	   872.03	 14
  5	 E 	   279.13	   843.52	 13
  6	 F 	   352.67	   779.00	 12
  7	 Y 	   434.20	   705.47	 11
  8	 E 	   498.72	   623.93	 10
  9	 A 	   534.24	   559.41	  9
 10	 L 	   590.78	   523.89	  8
 11	 D 	   648.29	   467.35	  7
 12	 C 	   728.38	   409.84	  6
 13	 L 	   784.92	   329.76	  5
 14	 C 	   865.00	   273.21	  4
 15	 I 	   921.54	   193.13	  3
 16	 P 	   970.07	   136.59	  2
 17	 R 	        -  	    88.06	  1  _
```

**130. IPI00335168 Isoform Non-muscle of Myosin light polypeptide 6**

| Sequence | Charge | M+H+ | XCorr | DeltaCn |
| --- | --- | --- | --- | --- |
| K.VLDFEHFLPMLQTVAK.N | 2 | 1889.25 | 3.252 | 0.471 |

+1 charge

```
		      B   	       Y
  1	 V 	   100.08	        -  	 16
  2	 L 	   213.16	  1788.94	 15
  3	 D 	   328.19	  1675.85	 14
  4	 F 	   475.26	  1560.82	 13
  5	 E 	   604.30	  1413.76	 12
  6	 H 	   741.36	  1284.71	 11
  7	 F 	   888.43	  1147.65	 10
  8	 L 	  1001.51	  1000.59	  9
  9	 P 	  1098.56	   887.50	  8
 10	 M 	  1229.60	   790.45	  7
 11	 L 	  1342.69	   659.41	  6
 12	 Q 	  1470.74	   546.32	  5
 13	 T 	  1571.79	   418.27	  4
 14	 V 	  1670.86	   317.22	  3
 15	 A 	  1741.90	   218.15	  2
 16	 K 	        -  	   147.11	  1  _
+2 charge
		      B   	       Y
  1	 V 	    50.54	        -  	 16
  2	 L 	   107.08	   894.97	 15
  3	 D 	   164.60	   838.43	 14
  4	 F 	   238.13	   780.92	 13
  5	 E 	   302.65	   707.38	 12
  6	 H 	   371.18	   642.86	 11
  7	 F 	   444.72	   574.33	 10
  8	 L 	   501.26	   500.80	  9
  9	 P 	   549.78	   444.25	  8
 10	 M 	   615.30	   395.73	  7
 11	 L 	   671.85	   330.21	  6
 12	 Q 	   735.88	   273.67	  5
 13	 T 	   786.40	   209.64	  4
 14	 V 	   835.93	   159.11	  3
 15	 A 	   871.45	   109.58	  2
 16	 K 	        -  	    74.06	  1  _
```

**131. IPI00001872 Isoform 1 of Protocadherin gamma-C3**

| Sequence | Charge | M+H+ | XCorr | DeltaCn |
| --- | --- | --- | --- | --- |
| K.GFAVGNVVANLGLDLGSLSAR.R | 2 | 2031.302 | 3.228 | 0.483 |

+1 charge

```
		      B   	       Y
  1	 G 	    58.03	        -  	 21
  2	 F 	   205.10	  1973.08	 20
  3	 A 	   276.13	  1826.01	 19
  4	 V 	   375.20	  1754.98	 18
  5	 G 	   432.22	  1655.91	 17
  6	 N 	   546.27	  1598.89	 16
  7	 V 	   645.34	  1484.84	 15
  8	 V 	   744.40	  1385.77	 14
  9	 A 	   815.44	  1286.71	 13
 10	 N 	   929.48	  1215.67	 12
 11	 L 	  1042.57	  1101.63	 11
 12	 G 	  1099.59	   988.54	 10
 13	 L 	  1212.67	   931.52	  9
 14	 D 	  1327.70	   818.44	  8
 15	 L 	  1440.78	   703.41	  7
 16	 G 	  1497.81	   590.33	  6
 17	 S 	  1584.84	   533.30	  5
 18	 L 	  1697.92	   446.27	  4
 19	 S 	  1784.95	   333.19	  3
 20	 A 	  1855.99	   246.16	  2
 21	 R 	        -  	   175.12	  1  _


+2 charge
		      B   	       Y
  1	 G 	    29.52	        -  	 21
  2	 F 	   103.05	   987.04	 20
  3	 A 	   138.57	   913.51	 19
  4	 V 	   188.10	   877.99	 18
  5	 G 	   216.62	   828.46	 17
  6	 N 	   273.64	   799.95	 16
  7	 V 	   323.17	   742.93	 15
  8	 V 	   372.71	   693.39	 14
  9	 A 	   408.22	   643.86	 13
 10	 N 	   465.25	   608.34	 12
 11	 L 	   521.79	   551.32	 11
 12	 G 	   550.30	   494.77	 10
 13	 L 	   606.84	   466.26	  9
 14	 D 	   664.35	   409.72	  8
 15	 L 	   720.90	   352.21	  7
 16	 G 	   749.41	   295.67	  6
 17	 S 	   792.92	   267.16	  5
 18	 L 	   849.46	   223.64	  4
 19	 S 	   892.98	   167.10	  3
 20	 A 	   928.50	   123.58	  2
 21	 R 	        -  	    88.06	  1  _
```

**132. IPI00001662 OPCML protein**

| Sequence | Charge | M+H+ | XCorr | DeltaCn |
| --- | --- | --- | --- | --- |
| R.DQSGEYECSALNDVAAPDVR.K | 2 | 2197.252 | 3.034 | 0.53 |

+1 charge

```
		      B   	       Y
  1	 D 	   116.03	        -  	 20
  2	 Q 	   244.09	  2081.06	 19
  3	 S 	   331.12	  1953.00	 18
  4	 G 	   388.15	  1865.97	 17
  5	 E 	   517.19	  1808.95	 16
  6	 Y 	   680.25	  1679.90	 15
  7	 E 	   809.29	  1516.84	 14
  8	 C 	   969.46	  1387.80	 13
  9	 S 	  1056.49	  1227.63	 12
 10	 A 	  1127.53	  1140.60	 11
 11	 L 	  1240.61	  1069.56	 10
 12	 N 	  1354.66	   956.48	  9
 13	 D 	  1469.68	   842.44	  8
 14	 V 	  1568.75	   727.41	  7
 15	 A 	  1639.79	   628.34	  6
 16	 A 	  1710.83	   557.30	  5
 17	 P 	  1807.88	   486.27	  4
 18	 D 	  1922.91	   389.21	  3
 19	 V 	  2021.97	   274.19	  2
 20	 R 	        -  	   175.12	  1  _
+2 charge
		      B   	       Y
  1	 D 	    58.52	        -  	 20
  2	 Q 	   122.55	  1041.03	 19
  3	 S 	   166.07	   977.00	 18
  4	 G 	   194.58	   933.49	 17
  5	 E 	   259.10	   904.98	 16
  6	 Y 	   340.63	   840.46	 15
  7	 E 	   405.15	   758.92	 14
  8	 C 	   485.23	   694.40	 13
  9	 S 	   528.75	   614.32	 12
 10	 A 	   564.27	   570.80	 11
 11	 L 	   620.81	   535.29	 10
 12	 N 	   677.83	   478.74	  9
 13	 D 	   735.35	   421.72	  8
 14	 V 	   784.88	   364.21	  7
 15	 A 	   820.40	   314.67	  6
 16	 A 	   855.92	   279.16	  5
 17	 P 	   904.44	   243.64	  4
 18	 D 	   961.96	   195.11	  3
 19	 V 	  1011.49	   137.60	  2
 20	 R 	        -  	    88.06	  1  _
```

**133. IPI00002435 CD27 antigen**

| Sequence | Charge | M+H+ | XCorr | DeltaCn |
| --- | --- | --- | --- | --- |
| K.LCCQMCEPGTFLVK.D | 2 | 1744.027 | 4.965 | 0.515 |

+1 charge

```
		      B   	       Y
  1	 L 	   114.09	        -  	 14
  2	 C 	   274.26	  1630.09	 13
  3	 C 	   434.42	  1469.93	 12
  4	 Q 	   562.48	  1309.76	 11
  5	 M 	   693.52	  1181.70	 10
  6	 C 	   853.69	  1050.66	  9
  7	 E 	   982.73	   890.50	  8
  8	 P 	  1079.78	   761.46	  7
  9	 G 	  1136.80	   664.40	  6
 10	 T 	  1237.85	   607.38	  5
 11	 F 	  1384.92	   506.33	  4
 12	 L 	  1498.00	   359.27	  3
 13	 V 	  1597.07	   246.18	  2
 14	 K 	        -  	   147.11	  1  _
+2 charge
		      B   	       Y
  1	 L 	    57.55	        -  	 14
  2	 C 	   137.63	   815.55	 13
  3	 C 	   217.71	   735.47	 12
  4	 Q 	   281.74	   655.38	 11
  5	 M 	   347.26	   591.36	 10
  6	 C 	   427.35	   525.84	  9
  7	 E 	   491.87	   445.75	  8
  8	 P 	   540.39	   381.23	  7
  9	 G 	   568.91	   332.71	  6
 10	 T 	   619.43	   304.19	  5
 11	 F 	   692.96	   253.67	  4
 12	 L 	   749.51	   180.14	  3
 13	 V 	   799.04	   123.59	  2
 14	 K 	        -  	    74.06	  1  _
```

**134. IPI00018402 Tubulin-specific chaperone E**

| Sequence | Charge | M+H+ | XCorr | DeltaCn |
| --- | --- | --- | --- | --- |
| R.PNKVNFGTDFLTAIKNR.Y | 2 | 1936.203 | 3.156 | 0.263 |

+1 charge

```
		      B   	       Y
  1	 P 	    98.06	        -  	 17
  2	 N 	   212.10	  1837.99	 16
  3	 K 	   340.20	  1723.95	 15
  4	 V 	   439.27	  1595.85	 14
  5	 N 	   553.31	  1496.79	 13
  6	 F 	   700.38	  1382.74	 12
  7	 G 	   757.40	  1235.67	 11
  8	 T 	   858.45	  1178.65	 10
  9	 D 	   973.47	  1077.61	  9
 10	 F 	  1120.54	   962.58	  8
 11	 L 	  1233.63	   815.51	  7
 12	 T 	  1334.67	   702.43	  6
 13	 A 	  1405.71	   601.38	  5
 14	 I 	  1518.80	   530.34	  4
 15	 K 	  1646.89	   417.26	  3
 16	 N 	  1760.93	   289.16	  2
 17	 R 	        -  	   175.12	  1  _
+2 charge
		      B   	       Y
  1	 P 	    49.53	        -  	 17
  2	 N 	   106.56	   919.50	 16
  3	 K 	   170.60	   862.48	 15
  4	 V 	   220.14	   798.43	 14
  5	 N 	   277.16	   748.90	 13
  6	 F 	   350.69	   691.87	 12
  7	 G 	   379.20	   618.34	 11
  8	 T 	   429.73	   589.83	 10
  9	 D 	   487.24	   539.31	  9
 10	 F 	   560.77	   481.79	  8
 11	 L 	   617.32	   408.26	  7
 12	 T 	   667.84	   351.72	  6
 13	 A 	   703.36	   301.19	  5
 14	 I 	   759.90	   265.67	  4
 15	 K 	   823.95	   209.13	  3
 16	 N 	   880.97	   145.08	  2
 17	 R 	        -  	    88.06	  1  _
```

**135. IPI00409566 Isoform 1 of Polycystin-1**

| Sequence | Charge | M+H+ | XCorr | DeltaCn |
| --- | --- | --- | --- | --- |
| R.TLGPALRIPADATALDVSHNLLR.A | 3 | 2415.777 | 3.829 | 0.4 |

+1 charge

```
		      B   	       Y
  1	 T 	   102.05	        -  	 23
  2	 L 	   215.14	  2313.30	 22
  3	 G 	   272.16	  2200.22	 21
  4	 P 	   369.21	  2143.20	 20
  5	 A 	   440.25	  2046.15	 19
  6	 L 	   553.33	  1975.11	 18
  7	 R 	   709.44	  1862.02	 17
  8	 I 	   822.52	  1705.92	 16
  9	 P 	   919.57	  1592.84	 15
 10	 A 	   990.61	  1495.79	 14
 11	 D 	  1105.64	  1424.75	 13
 12	 A 	  1176.67	  1309.72	 12
 13	 T 	  1277.72	  1238.69	 11
 14	 A 	  1348.76	  1137.64	 10
 15	 L 	  1461.84	  1066.60	  9
 16	 D 	  1576.87	   953.52	  8
 17	 V 	  1675.94	   838.49	  7
 18	 S 	  1762.97	   739.42	  6
 19	 H 	  1900.03	   652.39	  5
 20	 N 	  2014.07	   515.33	  4
 21	 L 	  2127.16	   401.29	  3
 22	 L 	  2240.24	   288.20	  2
 23	 R 	        -  	   175.12	  1  _


+2 charge
		      B   	       Y
  1	 T 	    51.53	        -  	 23
  2	 L 	   108.07	  1157.16	 22
  3	 G 	   136.58	  1100.61	 21
  4	 P 	   185.11	  1072.10	 20
  5	 A 	   220.63	  1023.58	 19
  6	 L 	   277.17	   988.06	 18
  7	 R 	   355.22	   931.52	 17
  8	 I 	   411.76	   853.47	 16
  9	 P 	   460.29	   796.92	 15
 10	 A 	   495.81	   748.40	 14
 11	 D 	   553.32	   712.88	 13
 12	 A 	   588.84	   655.36	 12
 13	 T 	   639.36	   619.85	 11
 14	 A 	   674.88	   569.32	 10
 15	 L 	   731.42	   533.80	  9
 16	 D 	   788.94	   477.26	  8
 17	 V 	   838.47	   419.75	  7
 18	 S 	   881.99	   370.21	  6
 19	 H 	   950.52	   326.70	  5
 20	 N 	  1007.54	   258.17	  4
 21	 L 	  1064.08	   201.15	  3
 22	 L 	  1120.62	   144.61	  2
 23	 R 	        -  	    88.06	  1  _


+3 charge
		      B   	       Y
  1	 T 	    34.69	        -  	 23
  2	 L 	    72.38	   771.77	 22
  3	 G 	    91.39	   734.08	 21
  4	 P 	   123.74	   715.07	 20
  5	 A 	   147.42	   682.72	 19
  6	 L 	   185.12	   659.04	 18
  7	 R 	   237.15	   621.35	 17
  8	 I 	   274.84	   569.31	 16
  9	 P 	   307.20	   531.62	 15
 10	 A 	   330.87	   499.27	 14
 11	 D 	   369.22	   475.59	 13
 12	 A 	   392.90	   437.25	 12
 13	 T 	   426.58	   413.57	 11
 14	 A 	   450.26	   379.88	 10
 15	 L 	   487.95	   356.20	  9
 16	 D 	   526.29	   318.51	  8
 17	 V 	   559.32	   280.17	  7
 18	 S 	   588.33	   247.15	  6
 19	 H 	   634.01	   218.13	  5
 20	 N 	   672.03	   172.45	  4
 21	 L 	   709.72	   134.43	  3
 22	 L 	   747.42	    96.74	  2
 23	 R 	        -  	    59.04	  1  _
```

**136. IPI00297124 Isoform 1 of Interleukin-6 receptor subunit beta**

| Sequence | Charge | M+H+ | XCorr | DeltaCn |
| --- | --- | --- | --- | --- |
| R.SSFTVQDLKPFTEYVFR.I | 2 | 2065.313 | 3.381 | 0.543 |

+1 charge

```
		      B   	       Y
  1	 S 	    88.04	        -  	 17
  2	 S 	   175.07	  1977.01	 16
  3	 F 	   322.14	  1889.98	 15
  4	 T 	   423.19	  1742.91	 14
  5	 V 	   522.26	  1641.86	 13
  6	 Q 	   650.31	  1542.80	 12
  7	 D 	   765.34	  1414.74	 11
  8	 L 	   878.43	  1299.71	 10
  9	 K 	  1006.52	  1186.63	  9
 10	 P 	  1103.57	  1058.53	  8
 11	 F 	  1250.64	   961.48	  7
 12	 T 	  1351.69	   814.41	  6
 13	 E 	  1480.73	   713.36	  5
 14	 Y 	  1643.80	   584.32	  4
 15	 V 	  1742.86	   421.26	  3
 16	 F 	  1889.93	   322.19	  2
 17	 R 	        -  	   175.12	  1  _
+2 charge
		      B   	       Y
  1	 S 	    44.52	        -  	 17
  2	 S 	    88.04	   989.01	 16
  3	 F 	   161.57	   945.49	 15
  4	 T 	   212.10	   871.96	 14
  5	 V 	   261.63	   821.44	 13
  6	 Q 	   325.66	   771.90	 12
  7	 D 	   383.17	   707.87	 11
  8	 L 	   439.72	   650.36	 10
  9	 K 	   503.76	   593.82	  9
 10	 P 	   552.29	   529.77	  8
 11	 F 	   625.82	   481.24	  7
 12	 T 	   676.35	   407.71	  6
 13	 E 	   740.87	   357.18	  5
 14	 Y 	   822.40	   292.66	  4
 15	 V 	   871.94	   211.13	  3
 16	 F 	   945.47	   161.60	  2
 17	 R 	        -  	    88.06	  1  _
```

**137. IPI00032288 MANSC domain-containing protein 1**

| Sequence | Charge | M+H+ | XCorr | DeltaCn |
| --- | --- | --- | --- | --- |
| K.SLEDVVIDIQSSLSK.G | 2 | 1633.822 | 3.366 | 0.34 |

+1 charge

```
		      B   	       Y
  1	 S 	    88.04	        -  	 15
  2	 L 	   201.12	  1545.84	 14
  3	 E 	   330.17	  1432.75	 13
  4	 D 	   445.19	  1303.71	 12
  5	 V 	   544.26	  1188.68	 11
  6	 V 	   643.33	  1089.62	 10
  7	 I 	   756.41	   990.55	  9
  8	 D 	   871.44	   877.46	  8
  9	 I 	   984.52	   762.44	  7
 10	 Q 	  1112.58	   649.35	  6
 11	 S 	  1199.62	   521.29	  5
 12	 S 	  1286.65	   434.26	  4
 13	 L 	  1399.73	   347.23	  3
 14	 S 	  1486.76	   234.14	  2
 15	 K 	        -  	   147.11	  1  _
+2 charge
		      B   	       Y
  1	 S 	    44.52	        -  	 15
  2	 L 	   101.07	   773.42	 14
  3	 E 	   165.59	   716.88	 13
  4	 D 	   223.10	   652.36	 12
  5	 V 	   272.63	   594.85	 11
  6	 V 	   322.17	   545.31	 10
  7	 I 	   378.71	   495.78	  9
  8	 D 	   436.22	   439.23	  8
  9	 I 	   492.77	   381.72	  7
 10	 Q 	   556.80	   325.18	  6
 11	 S 	   600.31	   261.15	  5
 12	 S 	   643.83	   217.63	  4
 13	 L 	   700.37	   174.12	  3
 14	 S 	   743.89	   117.58	  2
 15	 K 	        -  	    74.06	  1  _
```

**138. IPI00176125 Isoform 1 of Collagen alpha-5(VI) chain**

| Sequence | Charge | M+H+ | XCorr | DeltaCn |
| --- | --- | --- | --- | --- |
| K.GDIGNPGIPGGPGPKGFR.G | 2 | 1693.888 | 3.315 | 0.344 |

+1 charge

```
		      B   	       Y
  1	 G 	    58.03	        -  	 18
  2	 D 	   173.06	  1635.86	 17
  3	 I 	   286.14	  1520.83	 16
  4	 G 	   343.16	  1407.75	 15
  5	 N 	   457.20	  1350.73	 14
  6	 P 	   554.26	  1236.68	 13
  7	 G 	   611.28	  1139.63	 12
  8	 I 	   724.36	  1082.61	 11
  9	 P 	   821.42	   969.53	 10
 10	 G 	   878.44	   872.47	  9
 11	 G 	   935.46	   815.45	  8
 12	 P 	  1032.51	   758.43	  7
 13	 G 	  1089.53	   661.38	  6
 14	 P 	  1186.59	   604.36	  5
 15	 K 	  1314.68	   507.30	  4
 16	 G 	  1371.70	   379.21	  3
 17	 F 	  1518.77	   322.19	  2
 18	 R 	        -  	   175.12	  1  _
+2 charge
		      B   	       Y
  1	 G 	    29.52	        -  	 18
  2	 D 	    87.03	   818.43	 17
  3	 I 	   143.57	   760.92	 16
  4	 G 	   172.08	   704.38	 15
  5	 N 	   229.11	   675.87	 14
  6	 P 	   277.63	   618.85	 13
  7	 G 	   306.14	   570.32	 12
  8	 I 	   362.68	   541.81	 11
  9	 P 	   411.21	   485.27	 10
 10	 G 	   439.72	   436.74	  9
 11	 G 	   468.23	   408.23	  8
 12	 P 	   516.76	   379.72	  7
 13	 G 	   545.27	   331.19	  6
 14	 P 	   593.80	   302.68	  5
 15	 K 	   657.84	   254.16	  4
 16	 G 	   686.35	   190.11	  3
 17	 F 	   759.89	   161.60	  2
 18	 R 	        -  	    88.06	  1  _
```

**139. IPI00029275 Isoform 1 of Melanotransferrin**

| Sequence | Charge | M+H+ | XCorr | DeltaCn |
| --- | --- | --- | --- | --- |
| R.LCRGDSSGEGVCDKSPLER.Y | 3 | 2123.255 | 4.189 | 0.513 |

+1 charge

```
		      B   	       Y
  1	 L 	   114.09	        -  	 19
  2	 C 	   274.26	  2009.15	 18
  3	 R 	   430.36	  1848.99	 17
  4	 G 	   487.38	  1692.88	 16
  5	 D 	   602.41	  1635.86	 15
  6	 S 	   689.44	  1520.84	 14
  7	 S 	   776.47	  1433.80	 13
  8	 G 	   833.49	  1346.77	 12
  9	 E 	   962.53	  1289.75	 11
 10	 G 	  1019.56	  1160.71	 10
 11	 V 	  1118.62	  1103.69	  9
 12	 C 	  1278.79	  1004.62	  8
 13	 D 	  1393.82	   844.45	  7
 14	 K 	  1521.91	   729.43	  6
 15	 S 	  1608.94	   601.33	  5
 16	 P 	  1706.00	   514.30	  4
 17	 L 	  1819.08	   417.25	  3
 18	 E 	  1948.12	   304.16	  2
 19	 R 	        -  	   175.12	  1  _
+2 charge
		      B   	       Y
  1	 L 	    57.55	        -  	 19
  2	 C 	   137.63	  1005.08	 18
  3	 R 	   215.68	   925.00	 17
  4	 G 	   244.19	   846.95	 16
  5	 D 	   301.71	   818.43	 15
  6	 S 	   345.22	   760.92	 14
  7	 S 	   388.74	   717.41	 13
  8	 G 	   417.25	   673.89	 12
  9	 E 	   481.77	   645.38	 11
 10	 G 	   510.28	   580.86	 10
 11	 V 	   559.82	   552.35	  9
 12	 C 	   639.90	   502.81	  8
 13	 D 	   697.41	   422.73	  7
 14	 K 	   761.46	   365.22	  6
 15	 S 	   804.98	   301.17	  5
 16	 P 	   853.50	   257.65	  4
 17	 L 	   910.04	   209.13	  3
 18	 E 	   974.56	   152.58	  2
 19	 R 	        -  	    88.06	  1  _


+3 charge
		      B   	       Y
  1	 L 	    38.70	        -  	 19
  2	 C 	    92.09	   670.39	 18
  3	 R 	   144.12	   617.00	 17
  4	 G 	   163.13	   564.97	 16
  5	 D 	   201.47	   545.96	 15
  6	 S 	   230.48	   507.62	 14
  7	 S 	   259.49	   478.61	 13
  8	 G 	   278.50	   449.60	 12
  9	 E 	   321.52	   430.59	 11
 10	 G 	   340.52	   387.57	 10
 11	 V 	   373.55	   368.57	  9
 12	 C 	   426.93	   335.54	  8
 13	 D 	   465.28	   282.16	  7
 14	 K 	   507.98	   243.81	  6
 15	 S 	   536.99	   201.11	  5
 16	 P 	   569.34	   172.10	  4
 17	 L 	   607.03	   139.75	  3
 18	 E 	   650.05	   102.06	  2
 19	 R 	        -  	    59.04	  1  _
```

**140. IPI00028347 Isoform 1 of F-box only protein 31**

| Sequence | Charge | M+H+ | XCorr | DeltaCn |
| --- | --- | --- | --- | --- |
| R.IYLPPSRPDDLIKPGLFK.G | 2 | 2070.462 | 3.173 | 0.281 |

+1 charge

```
		      B   	       Y
  1	 I 	   114.09	        -  	 18
  2	 Y 	   277.15	  1956.10	 17
  3	 L 	   390.24	  1793.03	 16
  4	 P 	   487.29	  1679.95	 15
  5	 P 	   584.34	  1582.90	 14
  6	 S 	   671.38	  1485.84	 13
  7	 R 	   827.48	  1398.81	 12
  8	 P 	   924.53	  1242.71	 11
  9	 D 	  1039.56	  1145.66	 10
 10	 D 	  1154.58	  1030.63	  9
 11	 L 	  1267.67	   915.60	  8
 12	 I 	  1380.75	   802.52	  7
 13	 K 	  1508.85	   689.43	  6
 14	 P 	  1605.90	   561.34	  5
 15	 G 	  1662.92	   464.29	  4
 16	 L 	  1776.01	   407.27	  3
 17	 F 	  1923.07	   294.18	  2
 18	 K 	        -  	   147.11	  1  _
+2 charge
		      B   	       Y
  1	 I 	    57.55	        -  	 18
  2	 Y 	   139.08	   978.55	 17
  3	 L 	   195.62	   897.02	 16
  4	 P 	   244.15	   840.48	 15
  5	 P 	   292.68	   791.95	 14
  6	 S 	   336.19	   743.42	 13
  7	 R 	   414.24	   699.91	 12
  8	 P 	   462.77	   621.86	 11
  9	 D 	   520.28	   573.33	 10
 10	 D 	   577.80	   515.82	  9
 11	 L 	   634.34	   458.30	  8
 12	 I 	   690.88	   401.76	  7
 13	 K 	   754.93	   345.22	  6
 14	 P 	   803.45	   281.17	  5
 15	 G 	   831.96	   232.65	  4
 16	 L 	   888.51	   204.14	  3
 17	 F 	   962.04	   147.59	  2
 18	 K 	        -  	    74.06	  1  _
```

**141. IPI00100796 Charged multivesicular body protein 5**

| Sequence | Charge | M+H+ | XCorr | DeltaCn |
| --- | --- | --- | --- | --- |
| K.NKDGVLVDEFGLPQIPAS.- | 2 | 1900.122 | 2.966 | 0.451 |

+1 charge

```
		      B   	       Y
  1	 N 	   115.05	        -  	 18
  2	 K 	   243.15	  1784.94	 17
  3	 D 	   358.17	  1656.85	 16
  4	 G 	   415.19	  1541.82	 15
  5	 V 	   514.26	  1484.80	 14
  6	 L 	   627.35	  1385.73	 13
  7	 V 	   726.41	  1272.65	 12
  8	 D 	   841.44	  1173.58	 11
  9	 E 	   970.48	  1058.55	 10
 10	 F 	  1117.55	   929.51	  9
 11	 G 	  1174.57	   782.44	  8
 12	 L 	  1287.66	   725.42	  7
 13	 P 	  1384.71	   612.34	  6
 14	 Q 	  1512.77	   515.28	  5
 15	 I 	  1625.85	   387.22	  4
 16	 P 	  1722.91	   274.14	  3
 17	 A 	  1793.94	   177.09	  2
 18	 S 	        -  	   106.05	  1  _
+2 charge
		      B   	       Y
  1	 N 	    58.03	        -  	 18
  2	 K 	   122.08	   892.98	 17
  3	 D 	   179.59	   828.93	 16
  4	 G 	   208.10	   771.41	 15
  5	 V 	   257.63	   742.90	 14
  6	 L 	   314.18	   693.37	 13
  7	 V 	   363.71	   636.83	 12
  8	 D 	   421.22	   587.29	 11
  9	 E 	   485.75	   529.78	 10
 10	 F 	   559.28	   465.26	  9
 11	 G 	   587.79	   391.72	  8
 12	 L 	   644.33	   363.21	  7
 13	 P 	   692.86	   306.67	  6
 14	 Q 	   756.89	   258.14	  5
 15	 I 	   813.43	   194.12	  4
 16	 P 	   861.96	   137.57	  3
 17	 A 	   897.48	    89.05	  2
 18	 S 	        -  	    53.53	  1  _
```

**142. IPI00304273 Apolipoprotein A-IV**

| Sequence | Charge | M+H+ | XCorr | DeltaCn |
| --- | --- | --- | --- | --- |
| K.SELTQQLNALFQDKLGEVNTYAGDLQK.K | 3 | 3025.316 | 4.715 | 0.585 |

+1 charge

```
		      B   	       Y
  1	 S 	    88.04	        -  	 27
  2	 E 	   217.08	  2936.50	 26
  3	 L 	   330.17	  2807.46	 25
  4	 T 	   431.21	  2694.37	 24
  5	 Q 	   559.27	  2593.33	 23
  6	 Q 	   687.33	  2465.27	 22
  7	 L 	   800.41	  2337.21	 21
  8	 N 	   914.46	  2224.12	 20
  9	 A 	   985.49	  2110.08	 19
 10	 L 	  1098.58	  2039.04	 18
 11	 F 	  1245.65	  1925.96	 17
 12	 Q 	  1373.71	  1778.89	 16
 13	 D 	  1488.73	  1650.83	 15
 14	 K 	  1616.83	  1535.81	 14
 15	 L 	  1729.91	  1407.71	 13
 16	 G 	  1786.93	  1294.63	 12
 17	 E 	  1915.98	  1237.61	 11
 18	 V 	  2015.04	  1108.56	 10
 19	 N 	  2129.09	  1009.49	  9
 20	 T 	  2230.14	   895.45	  8
 21	 Y 	  2393.20	   794.40	  7
 22	 A 	  2464.24	   631.34	  6
 23	 G 	  2521.26	   560.30	  5
 24	 D 	  2636.28	   503.28	  4
 25	 L 	  2749.37	   388.26	  3
 26	 Q 	  2877.43	   275.17	  2
 27	 K 	        -  	   147.11	  1  _


+2 charge
		      B   	       Y
  1	 S 	    44.52	        -  	 27
  2	 E 	   109.04	  1468.75	 26
  3	 L 	   165.59	  1404.23	 25
  4	 T 	   216.11	  1347.69	 24
  5	 Q 	   280.14	  1297.17	 23
  6	 Q 	   344.17	  1233.14	 22
  7	 L 	   400.71	  1169.11	 21
  8	 N 	   457.73	  1112.57	 20
  9	 A 	   493.25	  1055.54	 19
 10	 L 	   549.79	  1020.03	 18
 11	 F 	   623.33	   963.48	 17
 12	 Q 	   687.36	   889.95	 16
 13	 D 	   744.87	   825.92	 15
 14	 K 	   808.92	   768.41	 14
 15	 L 	   865.46	   704.36	 13
 16	 G 	   893.97	   647.82	 12
 17	 E 	   958.49	   619.31	 11
 18	 V 	  1008.03	   554.79	 10
 19	 N 	  1065.05	   505.25	  9
 20	 T 	  1115.57	   448.23	  8
 21	 Y 	  1197.10	   397.71	  7
 22	 A 	  1232.62	   316.17	  6
 23	 G 	  1261.13	   280.66	  5
 24	 D 	  1318.65	   252.14	  4
 25	 L 	  1375.19	   194.63	  3
 26	 Q 	  1439.22	   138.09	  2
 27	 K 	        -  	    74.06	  1  _


+3 charge
		      B   	       Y
  1	 S 	    30.02	        -  	 27
  2	 E 	    73.03	   979.50	 26
  3	 L 	   110.73	   936.49	 25
  4	 T 	   144.41	   898.80	 24
  5	 Q 	   187.10	   865.11	 23
  6	 Q 	   229.78	   822.43	 22
  7	 L 	   267.48	   779.74	 21
  8	 N 	   305.49	   742.05	 20
  9	 A 	   329.17	   704.03	 19
 10	 L 	   366.86	   680.35	 18
 11	 F 	   415.89	   642.66	 17
 12	 Q 	   458.57	   593.64	 16
 13	 D 	   496.92	   550.95	 15
 14	 K 	   539.61	   512.61	 14
 15	 L 	   577.31	   469.91	 13
 16	 G 	   596.32	   432.21	 12
 17	 E 	   639.33	   413.21	 11
 18	 V 	   672.35	   370.19	 10
 19	 N 	   710.37	   337.17	  9
 20	 T 	   744.05	   299.16	  8
 21	 Y 	   798.40	   265.47	  7
 22	 A 	   822.08	   211.12	  6
 23	 G 	   841.09	   187.44	  5
 24	 D 	   879.43	   168.43	  4
 25	 L 	   917.13	   130.09	  3
 26	 Q 	   959.81	    92.40	  2
 27	 K 	        -  	    49.71	  1  _
```

**143. IPI00021885 Isoform 1 of Fibrinogen alpha chain**

| Sequence | Charge | M+H+ | XCorr | DeltaCn |
| --- | --- | --- | --- | --- |
| R.GFGSLNDEGEGEFWLGNDYLHLLTQR.G | 3 | 2969.17 | 5.491 | 0.579 |

+1 charge

```
		      B   	       Y
  1	 G 	    58.03	        -  	 26
  2	 F 	   205.10	  2910.37	 25
  3	 G 	   262.12	  2763.30	 24
  4	 S 	   349.15	  2706.28	 23
  5	 L 	   462.23	  2619.25	 22
  6	 N 	   576.28	  2506.16	 21
  7	 D 	   691.30	  2392.12	 20
  8	 E 	   820.35	  2277.09	 19
  9	 G 	   877.37	  2148.05	 18
 10	 E 	  1006.41	  2091.03	 17
 11	 G 	  1063.43	  1961.99	 16
 12	 E 	  1192.48	  1904.97	 15
 13	 F 	  1339.54	  1775.92	 14
 14	 W 	  1525.62	  1628.85	 13
 15	 L 	  1638.71	  1442.78	 12
 16	 G 	  1695.73	  1329.69	 11
 17	 N 	  1809.77	  1272.67	 10
 18	 D 	  1924.80	  1158.63	  9
 19	 Y 	  2087.86	  1043.60	  8
 20	 L 	  2200.95	   880.54	  7
 21	 H 	  2338.00	   767.45	  6
 22	 L 	  2451.09	   630.39	  5
 23	 L 	  2564.17	   517.31	  4
 24	 T 	  2665.22	   404.23	  3
 25	 Q 	  2793.28	   303.18	  2
 26	 R 	        -  	   175.12	  1  _


+2 charge
		      B   	       Y
  1	 G 	    29.52	        -  	 26
  2	 F 	   103.05	  1455.69	 25
  3	 G 	   131.56	  1382.15	 24
  4	 S 	   175.08	  1353.64	 23
  5	 L 	   231.62	  1310.13	 22
  6	 N 	   288.64	  1253.59	 21
  7	 D 	   346.16	  1196.56	 20
  8	 E 	   410.68	  1139.05	 19
  9	 G 	   439.19	  1074.53	 18
 10	 E 	   503.71	  1046.02	 17
 11	 G 	   532.22	   981.50	 16
 12	 E 	   596.74	   952.99	 15
 13	 F 	   670.28	   888.47	 14
 14	 W 	   763.32	   814.93	 13
 15	 L 	   819.86	   721.89	 12
 16	 G 	   848.37	   665.35	 11
 17	 N 	   905.39	   636.84	 10
 18	 D 	   962.90	   579.82	  9
 19	 Y 	  1044.43	   522.30	  8
 20	 L 	  1100.98	   440.77	  7
 21	 H 	  1169.51	   384.23	  6
 22	 L 	  1226.05	   315.70	  5
 23	 L 	  1282.59	   259.16	  4
 24	 T 	  1333.11	   202.62	  3
 25	 Q 	  1397.14	   152.09	  2
 26	 R 	        -  	    88.06	  1  _


+3 charge
		      B   	       Y
  1	 G 	    20.01	        -  	 26
  2	 F 	    69.04	   970.79	 25
  3	 G 	    88.04	   921.77	 24
  4	 S 	   117.06	   902.76	 23
  5	 L 	   154.75	   873.75	 22
  6	 N 	   192.76	   836.06	 21
  7	 D 	   231.11	   798.04	 20
  8	 E 	   274.12	   759.70	 19
  9	 G 	   293.13	   716.69	 18
 10	 E 	   336.14	   697.68	 17
 11	 G 	   355.15	   654.67	 16
 12	 E 	   398.16	   635.66	 15
 13	 F 	   447.19	   592.65	 14
 14	 W 	   509.21	   543.62	 13
 15	 L 	   546.91	   481.60	 12
 16	 G 	   565.91	   443.90	 11
 17	 N 	   603.93	   424.89	 10
 18	 D 	   642.27	   386.88	  9
 19	 Y 	   696.63	   348.54	  8
 20	 L 	   734.32	   294.18	  7
 21	 H 	   780.01	   256.49	  6
 22	 L 	   817.70	   210.80	  5
 23	 L 	   855.40	   173.11	  4
 24	 T 	   889.08	   135.41	  3
 25	 Q 	   931.76	   101.73	  2
 26	 R 	        -  	    59.04	  1  _
```

**Female 6.**

**1. IPI00028030 Cartilage oligomeric matrix protein**

| Sequence | Charge | M+H+ | XCorr | DeltaCn |
| --- | --- | --- | --- | --- |
| R.ELQETNAALQDVR.E | 2 | 1487.597 | 4.453 | 0.381 |

+1 charge

```
		      B   	       Y
  1	 E 	   130.05	        -  	 13
  2	 L 	   243.13	  1357.71	 12
  3	 Q 	   371.19	  1244.62	 11
  4	 E 	   500.24	  1116.56	 10
  5	 T 	   601.28	   987.52	  9
  6	 N 	   715.33	   886.47	  8
  7	 A 	   786.36	   772.43	  7
  8	 A 	   857.40	   701.39	  6
  9	 L 	   970.48	   630.36	  5
 10	 Q 	  1098.54	   517.27	  4
 11	 D 	  1213.57	   389.21	  3
 12	 V 	  1312.64	   274.19	  2
 13	 R 	        -  	   175.12	  1  _
+2 charge
		      B   	       Y
  1	 E 	    65.53	        -  	 13
  2	 L 	   122.07	   679.36	 12
  3	 Q 	   186.10	   622.82	 11
  4	 E 	   250.62	   558.79	 10
  5	 T 	   301.15	   494.26	  9
  6	 N 	   358.17	   443.74	  8
  7	 A 	   393.69	   386.72	  7
  8	 A 	   429.20	   351.20	  6
  9	 L 	   485.75	   315.68	  5
 10	 Q 	   549.77	   259.14	  4
 11	 D 	   607.29	   195.11	  3
 12	 V 	   656.82	   137.60	  2
 13	 R 	        -  	    88.06	  1  _
```

**2. IPI00217236 Tubulin-specific chaperone A**

| Sequence | Charge | M+H+ | XCorr | DeltaCn |
| --- | --- | --- | --- | --- |
| R.ILENEKDLEEAEEYKEAR.L | 3 | 2209.353 | 4.101 | 0.408 |

+1 charge

```
		      B   	       Y
  1	 I 	   114.09	        -  	 18
  2	 L 	   227.18	  2094.98	 17
  3	 E 	   356.22	  1981.90	 16
  4	 N 	   470.26	  1852.86	 15
  5	 E 	   599.30	  1738.81	 14
  6	 K 	   727.40	  1609.77	 13
  7	 D 	   842.43	  1481.68	 12
  8	 L 	   955.51	  1366.65	 11
  9	 E 	  1084.55	  1253.56	 10
 10	 E 	  1213.59	  1124.52	  9
 11	 A 	  1284.63	   995.48	  8
 12	 E 	  1413.67	   924.44	  7
 13	 E 	  1542.72	   795.40	  6
 14	 Y 	  1705.78	   666.36	  5
 15	 K 	  1833.88	   503.29	  4
 16	 E 	  1962.92	   375.20	  3
 17	 A 	  2033.95	   246.16	  2
 18	 R 	        -  	   175.12	  1  _
+2 charge
		      B   	       Y
  1	 I 	    57.55	        -  	 18
  2	 L 	   114.09	  1047.99	 17
  3	 E 	   178.61	   991.45	 16
  4	 N 	   235.63	   926.93	 15
  5	 E 	   300.16	   869.91	 14
  6	 K 	   364.20	   805.39	 13
  7	 D 	   421.72	   741.34	 12
  8	 L 	   478.26	   683.83	 11
  9	 E 	   542.78	   627.29	 10
 10	 E 	   607.30	   562.76	  9
 11	 A 	   642.82	   498.24	  8
 12	 E 	   707.34	   462.72	  7
 13	 E 	   771.86	   398.20	  6
 14	 Y 	   853.39	   333.68	  5
 15	 K 	   917.44	   252.15	  4
 16	 E 	   981.96	   188.10	  3
 17	 A 	  1017.48	   123.58	  2
 18	 R 	        -  	    88.06	  1  _
+3 charge
		      B   	       Y
  1	 I 	    38.70	        -  	 18
  2	 L 	    76.40	   699.00	 17
  3	 E 	   119.41	   661.30	 16
  4	 N 	   157.43	   618.29	 15
  5	 E 	   200.44	   580.28	 14
  6	 K 	   243.14	   537.26	 13
  7	 D 	   281.48	   494.56	 12
  8	 L 	   319.17	   456.22	 11
  9	 E 	   362.19	   418.53	 10
 10	 E 	   405.20	   375.51	  9
 11	 A 	   428.88	   332.50	  8
 12	 E 	   471.90	   308.82	  7
 13	 E 	   514.91	   265.80	  6
 14	 Y 	   569.26	   222.79	  5
 15	 K 	   611.96	   168.44	  4
 16	 E 	   654.98	   125.74	  3
 17	 A 	   678.66	    82.72	  2
 18	 R 	        -  	    59.04	  1  _
```

**3. IPI00297420 Frizzled-4 precursor**

| Sequence | Charge | M+H+ | XCorr | DeltaCn |
| --- | --- | --- | --- | --- |
| K.INIPIGPCGGMCLSVK.R | 2 | 1717.054 | 3.292 | 0.418 |

+1 charge

```
		      B   	       Y
  1	 I 	   114.09	        -  	 16
  2	 N 	   228.13	  1603.05	 15
  3	 I 	   341.22	  1489.01	 14
  4	 P 	   438.27	  1375.92	 13
  5	 I 	   551.36	  1278.87	 12
  6	 G 	   608.38	  1165.79	 11
  7	 P 	   705.43	  1108.76	 10
  8	 C 	   865.59	  1011.71	  9
  9	 G 	   922.62	   851.55	  8
 10	 G 	   979.64	   794.52	  7
 11	 M 	  1110.68	   737.50	  6
 12	 C 	  1270.84	   606.46	  5
 13	 L 	  1383.93	   446.30	  4
 14	 S 	  1470.96	   333.21	  3
 15	 V 	  1570.03	   246.18	  2
 16	 K 	        -  	   147.11	  1  _
+2 charge
		      B   	       Y
  1	 I 	    57.55	        -  	 16
  2	 N 	   114.57	   802.03	 15
  3	 I 	   171.11	   745.01	 14
  4	 P 	   219.64	   688.46	 13
  5	 I 	   276.18	   639.94	 12
  6	 G 	   304.69	   583.40	 11
  7	 P 	   353.22	   554.89	 10
  8	 C 	   433.30	   506.36	  9
  9	 G 	   461.81	   426.28	  8
 10	 G 	   490.32	   397.77	  7
 11	 M 	   555.84	   369.26	  6
 12	 C 	   635.93	   303.73	  5
 13	 L 	   692.47	   223.65	  4
 14	 S 	   735.98	   167.11	  3
 15	 V 	   785.52	   123.59	  2
 16	 K 	        -  	    74.06	  1  _
```

**4. IPI00290085 Cadherin-2**

| Sequence | Charge | M+H+ | XCorr | DeltaCn |
| --- | --- | --- | --- | --- |
| R.FAIQTDPNSNDGLVTVVKPIDFETNR.M | 3 | 2892.17 | 3.545 | 0.373 |

+1 charge

```
		      B   	       Y
  1	 F 	   148.08	        -  	 26
  2	 A 	   219.11	  2743.39	 25
  3	 I 	   332.20	  2672.35	 24
  4	 Q 	   460.26	  2559.27	 23
  5	 T 	   561.30	  2431.21	 22
  6	 D 	   676.33	  2330.16	 21
  7	 P 	   773.38	  2215.14	 20
  8	 N 	   887.43	  2118.08	 19
  9	 S 	   974.46	  2004.04	 18
 10	 N 	  1088.50	  1917.01	 17
 11	 D 	  1203.53	  1802.96	 16
 12	 G 	  1260.55	  1687.94	 15
 13	 L 	  1373.63	  1630.92	 14
 14	 V 	  1472.70	  1517.83	 13
 15	 T 	  1573.75	  1418.76	 12
 16	 V 	  1672.82	  1317.72	 11
 17	 V 	  1771.89	  1218.65	 10
 18	 K 	  1899.98	  1119.58	  9
 19	 P 	  1997.03	   991.48	  8
 20	 I 	  2110.12	   894.43	  7
 21	 D 	  2225.14	   781.35	  6
 22	 F 	  2372.21	   666.32	  5
 23	 E 	  2501.26	   519.25	  4
 24	 T 	  2602.30	   390.21	  3
 25	 N 	  2716.35	   289.16	  2
 26	 R 	        -  	   175.12	  1  _


+2 charge
		      B   	       Y
  1	 F 	    74.54	        -  	 26
  2	 A 	   110.06	  1372.20	 25
  3	 I 	   166.60	  1336.68	 24
  4	 Q 	   230.63	  1280.14	 23
  5	 T 	   281.16	  1216.11	 22
  6	 D 	   338.67	  1165.58	 21
  7	 P 	   387.20	  1108.07	 20
  8	 N 	   444.22	  1059.54	 19
  9	 S 	   487.73	  1002.52	 18
 10	 N 	   544.75	   959.01	 17
 11	 D 	   602.27	   901.99	 16
 12	 G 	   630.78	   844.47	 15
 13	 L 	   687.32	   815.96	 14
 14	 V 	   736.85	   759.42	 13
 15	 T 	   787.38	   709.89	 12
 16	 V 	   836.91	   659.36	 11
 17	 V 	   886.45	   609.83	 10
 18	 K 	   950.49	   560.29	  9
 19	 P 	   999.02	   496.25	  8
 20	 I 	  1055.56	   447.72	  7
 21	 D 	  1113.08	   391.18	  6
 22	 F 	  1186.61	   333.66	  5
 23	 E 	  1251.13	   260.13	  4
 24	 T 	  1301.66	   195.61	  3
 25	 N 	  1358.68	   145.08	  2
 26	 R 	        -  	    88.06	  1  _


+3 charge
		      B   	       Y
  1	 F 	    50.03	        -  	 26
  2	 A 	    73.71	   915.13	 25
  3	 I 	   111.40	   891.46	 24
  4	 Q 	   154.09	   853.76	 23
  5	 T 	   187.77	   811.07	 22
  6	 D 	   226.11	   777.39	 21
  7	 P 	   258.47	   739.05	 20
  8	 N 	   296.48	   706.70	 19
  9	 S 	   325.49	   668.68	 18
 10	 N 	   363.51	   639.67	 17
 11	 D 	   401.85	   601.66	 16
 12	 G 	   420.85	   563.32	 15
 13	 L 	   458.55	   544.31	 14
 14	 V 	   491.57	   506.62	 13
 15	 T 	   525.25	   473.59	 12
 16	 V 	   558.28	   439.91	 11
 17	 V 	   591.30	   406.89	 10
 18	 K 	   634.00	   373.86	  9
 19	 P 	   666.35	   331.17	  8
 20	 I 	   704.04	   298.82	  7
 21	 D 	   742.39	   261.12	  6
 22	 F 	   791.41	   222.78	  5
 23	 E 	   834.42	   173.76	  4
 24	 T 	   868.11	   130.74	  3
 25	 N 	   906.12	    97.06	  2
 26	 R 	        -  	    59.04	  1  _
```

**5. IPI00023673 Galectin-3-binding protein**

| Sequence | Charge | M+H+ | XCorr | DeltaCn |
| --- | --- | --- | --- | --- |
| R.ELSEALGQIFDSQR.G | 2 | 1593.72 | 4.173 | 0.53 |

+1 charge

```
		      B   	       Y
  1	 E 	   130.05	        -  	 14
  2	 L 	   243.13	  1463.75	 13
  3	 S 	   330.17	  1350.66	 12
  4	 E 	   459.21	  1263.63	 11
  5	 A 	   530.25	  1134.59	 10
  6	 L 	   643.33	  1063.55	  9
  7	 G 	   700.35	   950.47	  8
  8	 Q 	   828.41	   893.45	  7
  9	 I 	   941.49	   765.39	  6
 10	 F 	  1088.56	   652.30	  5
 11	 D 	  1203.59	   505.24	  4
 12	 S 	  1290.62	   390.21	  3
 13	 Q 	  1418.68	   303.18	  2
 14	 R 	        -  	   175.12	  1  _
+2 charge
		      B   	       Y
  1	 E 	    65.53	        -  	 14
  2	 L 	   122.07	   732.38	 13
  3	 S 	   165.59	   675.84	 12
  4	 E 	   230.11	   632.32	 11
  5	 A 	   265.63	   567.80	 10
  6	 L 	   322.17	   532.28	  9
  7	 G 	   350.68	   475.74	  8
  8	 Q 	   414.71	   447.23	  7
  9	 I 	   471.25	   383.20	  6
 10	 F 	   544.78	   326.66	  5
 11	 D 	   602.30	   253.12	  4
 12	 S 	   645.81	   195.61	  3
 13	 Q 	   709.84	   152.09	  2
 14	 R 	        -  	    88.06	  1  _
```

**6. IPI00029260 Monocyte differentiation antigen CD14**

| Sequence | Charge | M+H+ | XCorr | DeltaCn |
| --- | --- | --- | --- | --- |
| R.AFPALTSLDLSDNPGLGER.G | 2 | 1974.161 | 4.674 | 0.54 |

+1 charge

```
		      B   	       Y
  1	 A 	    72.04	        -  	 19
  2	 F 	   219.11	  1901.96	 18
  3	 P 	   316.17	  1754.89	 17
  4	 A 	   387.20	  1657.84	 16
  5	 L 	   500.29	  1586.80	 15
  6	 T 	   601.33	  1473.72	 14
  7	 S 	   688.37	  1372.67	 13
  8	 L 	   801.45	  1285.64	 12
  9	 D 	   916.48	  1172.55	 11
 10	 L 	  1029.56	  1057.53	 10
 11	 S 	  1116.59	   944.44	  9
 12	 D 	  1231.62	   857.41	  8
 13	 N 	  1345.66	   742.38	  7
 14	 P 	  1442.72	   628.34	  6
 15	 G 	  1499.74	   531.29	  5
 16	 L 	  1612.82	   474.27	  4
 17	 G 	  1669.84	   361.18	  3
 18	 E 	  1798.89	   304.16	  2
 19	 R 	        -  	   175.12	  1  _
+2 charge
		      B   	       Y
  1	 A 	    36.53	        -  	 19
  2	 F 	   110.06	   951.48	 18
  3	 P 	   158.59	   877.95	 17
  4	 A 	   194.10	   829.42	 16
  5	 L 	   250.65	   793.90	 15
  6	 T 	   301.17	   737.36	 14
  7	 S 	   344.69	   686.84	 13
  8	 L 	   401.23	   643.32	 12
  9	 D 	   458.74	   586.78	 11
 10	 L 	   515.28	   529.27	 10
 11	 S 	   558.80	   472.73	  9
 12	 D 	   616.31	   429.21	  8
 13	 N 	   673.34	   371.70	  7
 14	 P 	   721.86	   314.67	  6
 15	 G 	   750.37	   266.15	  5
 16	 L 	   806.91	   237.64	  4
 17	 G 	   835.43	   181.10	  3
 18	 E 	   899.95	   152.58	  2
 19	 R 	        -  	    88.06	  1  _
```

**7. IPI00005707 C-type mannose receptor 2**

| Sequence | Charge | M+H+ | XCorr | DeltaCn |
| --- | --- | --- | --- | --- |
| R.TLGDQLSLLLGAR.T | 2 | 1357.581 | 3.688 | 0.404 |

+1 charge

```
		      B   	       Y
  1	 T 	   102.05	        -  	 13
  2	 L 	   215.14	  1255.74	 12
  3	 G 	   272.16	  1142.65	 11
  4	 D 	   387.19	  1085.63	 10
  5	 Q 	   515.25	   970.60	  9
  6	 L 	   628.33	   842.55	  8
  7	 S 	   715.36	   729.46	  7
  8	 L 	   828.45	   642.43	  6
  9	 L 	   941.53	   529.35	  5
 10	 L 	  1054.61	   416.26	  4
 11	 G 	  1111.64	   303.18	  3
 12	 A 	  1182.67	   246.16	  2
 13	 R 	        -  	   175.12	  1  _
+2 charge
		      B   	       Y
  1	 T 	    51.53	        -  	 13
  2	 L 	   108.07	   628.37	 12
  3	 G 	   136.58	   571.83	 11
  4	 D 	   194.10	   543.32	 10
  5	 Q 	   258.13	   485.81	  9
  6	 L 	   314.67	   421.78	  8
  7	 S 	   358.18	   365.23	  7
  8	 L 	   414.73	   321.72	  6
  9	 L 	   471.27	   265.18	  5
 10	 L 	   527.81	   208.63	  4
 11	 G 	   556.32	   152.09	  3
 12	 A 	   591.84	   123.58	  2
 13	 R 	        -  	    88.06	  1  _
```

**8. IPI00410323 Isoform 1 of Thioredoxin domain-containing protein 2**

| Sequence | Charge | M+H+ | XCorr | DeltaCn |
| --- | --- | --- | --- | --- |
| K.EGDIPKSPEEAIQPKEGDIPK.S | 3 | 2278.501 | 3.535 | 0.36 |

+1 charge

```
		      B   	       Y
  1	 E 	   130.05	        -  	 21
  2	 G 	   187.07	  2148.12	 20
  3	 D 	   302.10	  2091.10	 19
  4	 I 	   415.18	  1976.07	 18
  5	 P 	   512.24	  1862.99	 17
  6	 K 	   640.33	  1765.93	 16
  7	 S 	   727.36	  1637.84	 15
  8	 P 	   824.41	  1550.81	 14
  9	 E 	   953.46	  1453.75	 13
 10	 E 	  1082.50	  1324.71	 12
 11	 A 	  1153.54	  1195.67	 11
 12	 I 	  1266.62	  1124.63	 10
 13	 Q 	  1394.68	  1011.55	  9
 14	 P 	  1491.73	   883.49	  8
 15	 K 	  1619.83	   786.44	  7
 16	 E 	  1748.87	   658.34	  6
 17	 G 	  1805.89	   529.30	  5
 18	 D 	  1920.92	   472.28	  4
 19	 I 	  2034.00	   357.25	  3
 20	 P 	  2131.06	   244.17	  2
 21	 K 	        -  	   147.11	  1  _


+2 charge
		      B   	       Y
  1	 E 	    65.53	        -  	 21
  2	 G 	    94.04	  1074.56	 20
  3	 D 	   151.55	  1046.05	 19
  4	 I 	   208.09	   988.54	 18
  5	 P 	   256.62	   932.00	 17
  6	 K 	   320.67	   883.47	 16
  7	 S 	   364.18	   819.42	 15
  8	 P 	   412.71	   775.91	 14
  9	 E 	   477.23	   727.38	 13
 10	 E 	   541.75	   662.86	 12
 11	 A 	   577.27	   598.34	 11
 12	 I 	   633.81	   562.82	 10
 13	 Q 	   697.84	   506.28	  9
 14	 P 	   746.37	   442.25	  8
 15	 K 	   810.42	   393.72	  7
 16	 E 	   874.94	   329.67	  6
 17	 G 	   903.45	   265.15	  5
 18	 D 	   960.96	   236.64	  4
 19	 I 	  1017.50	   179.13	  3
 20	 P 	  1066.03	   122.59	  2
 21	 K 	        -  	    74.06	  1  _
+3 charge
		      B   	       Y
  1	 E 	    44.02	        -  	 21
  2	 G 	    63.03	   716.71	 20
  3	 D 	   101.37	   697.70	 19
  4	 I 	   139.07	   659.36	 18
  5	 P 	   171.42	   621.67	 17
  6	 K 	   214.11	   589.32	 16
  7	 S 	   243.13	   546.62	 15
  8	 P 	   275.48	   517.61	 14
  9	 E 	   318.49	   485.26	 13
 10	 E 	   361.50	   442.24	 12
 11	 A 	   385.18	   399.23	 11
 12	 I 	   422.88	   375.55	 10
 13	 Q 	   465.56	   337.85	  9
 14	 P 	   497.92	   295.17	  8
 15	 K 	   540.61	   262.82	  7
 16	 E 	   583.63	   220.12	  6
 17	 G 	   602.64	   177.10	  5
 18	 D 	   640.98	   158.10	  4
 19	 I 	   678.67	   119.75	  3
 20	 P 	   711.02	    82.06	  2
 21	 K 	        -  	    49.71	  1  _
```

**9. IPI00936772 Isoform 1 of Golgi membrane protein 1**

| Sequence | Charge | M+H+ | XCorr | DeltaCn |
| --- | --- | --- | --- | --- |
| R.DLSENNDQRQQLQALSEPQPR.L | 3 | 2467.595 | 3.543 | 0.51 |

+1 charge

```
		      B   	       Y
  1	 D 	   116.03	        -  	 21
  2	 L 	   229.12	  2351.17	 20
  3	 S 	   316.15	  2238.09	 19
  4	 E 	   445.19	  2151.05	 18
  5	 N 	   559.24	  2022.01	 17
  6	 N 	   673.28	  1907.97	 16
  7	 D 	   788.31	  1793.93	 15
  8	 Q 	   916.36	  1678.90	 14
  9	 R 	  1072.47	  1550.84	 13
 10	 Q 	  1200.52	  1394.74	 12
 11	 Q 	  1328.58	  1266.68	 11
 12	 L 	  1441.67	  1138.62	 10
 13	 Q 	  1569.73	  1025.54	  9
 14	 A 	  1640.76	   897.48	  8
 15	 L 	  1753.85	   826.44	  7
 16	 S 	  1840.88	   713.36	  6
 17	 E 	  1969.92	   626.33	  5
 18	 P 	  2066.97	   497.28	  4
 19	 Q 	  2195.03	   400.23	  3
 20	 P 	  2292.09	   272.17	  2
 21	 R 	        -  	   175.12	  1  _


+2 charge
		      B   	       Y
  1	 D 	    58.52	        -  	 21
  2	 L 	   115.06	  1176.09	 20
  3	 S 	   158.58	  1119.55	 19
  4	 E 	   223.10	  1076.03	 18
  5	 N 	   280.12	  1011.51	 17
  6	 N 	   337.14	   954.49	 16
  7	 D 	   394.66	   897.47	 15
  8	 Q 	   458.69	   839.95	 14
  9	 R 	   536.74	   775.92	 13
 10	 Q 	   600.77	   697.87	 12
 11	 Q 	   664.79	   633.84	 11
 12	 L 	   721.34	   569.81	 10
 13	 Q 	   785.37	   513.27	  9
 14	 A 	   820.88	   449.24	  8
 15	 L 	   877.43	   413.72	  7
 16	 S 	   920.94	   357.18	  6
 17	 E 	   985.46	   313.67	  5
 18	 P 	  1033.99	   249.15	  4
 19	 Q 	  1098.02	   200.62	  3
 20	 P 	  1146.55	   136.59	  2
 21	 R 	        -  	    88.06	  1  _
+3 charge
		      B   	       Y
  1	 D 	    39.35	        -  	 21
  2	 L 	    77.04	   784.39	 20
  3	 S 	   106.05	   746.70	 19
  4	 E 	   149.07	   717.69	 18
  5	 N 	   187.08	   674.68	 17
  6	 N 	   225.10	   636.66	 16
  7	 D 	   263.44	   598.65	 15
  8	 Q 	   306.13	   560.30	 14
  9	 R 	   358.16	   517.62	 13
 10	 Q 	   400.85	   465.58	 12
 11	 Q 	   443.53	   422.90	 11
 12	 L 	   481.23	   380.21	 10
 13	 Q 	   523.91	   342.52	  9
 14	 A 	   547.59	   299.83	  8
 15	 L 	   585.29	   276.15	  7
 16	 S 	   614.30	   238.46	  6
 17	 E 	   657.31	   209.45	  5
 18	 P 	   689.66	   166.43	  4
 19	 Q 	   732.35	   134.08	  3
 20	 P 	   764.70	    91.40	  2
 21	 R 	        -  	    59.04	  1  _
```

**10. IPI00953695 Ig kappa chain V-II region GM607 (Fragment)**

| Sequence | Charge | M+H+ | XCorr | DeltaCn |
| --- | --- | --- | --- | --- |
| R.ASGVPDRFSGSGSGTDFTLK.I | 2 | 1987.116 | 3.881 | 0.519 |

+1 charge

```
		      B   	       Y
  1	 A 	    72.04	        -  	 20
  2	 S 	   159.08	  1914.92	 19
  3	 G 	   216.10	  1827.89	 18
  4	 V 	   315.17	  1770.87	 17
  5	 P 	   412.22	  1671.80	 16
  6	 D 	   527.25	  1574.74	 15
  7	 R 	   683.35	  1459.72	 14
  8	 F 	   830.42	  1303.62	 13
  9	 S 	   917.45	  1156.55	 12
 10	 G 	   974.47	  1069.52	 11
 11	 S 	  1061.50	  1012.49	 10
 12	 G 	  1118.52	   925.46	  9
 13	 S 	  1205.55	   868.44	  8
 14	 G 	  1262.58	   781.41	  7
 15	 T 	  1363.62	   724.39	  6
 16	 D 	  1478.65	   623.34	  5
 17	 F 	  1625.72	   508.31	  4
 18	 T 	  1726.77	   361.24	  3
 19	 L 	  1839.85	   260.20	  2
 20	 K 	        -  	   147.11	  1  _
+2 charge
		      B   	       Y
  1	 A 	    36.53	        -  	 20
  2	 S 	    80.04	   957.96	 19
  3	 G 	   108.55	   914.45	 18
  4	 V 	   158.09	   885.94	 17
  5	 P 	   206.61	   836.40	 16
  6	 D 	   264.13	   787.88	 15
  7	 R 	   342.18	   730.36	 14
  8	 F 	   415.71	   652.31	 13
  9	 S 	   459.23	   578.78	 12
 10	 G 	   487.74	   535.26	 11
 11	 S 	   531.25	   506.75	 10
 12	 G 	   559.76	   463.23	  9
 13	 S 	   603.28	   434.72	  8
 14	 G 	   631.79	   391.21	  7
 15	 T 	   682.32	   362.70	  6
 16	 D 	   739.83	   312.17	  5
 17	 F 	   813.36	   254.66	  4
 18	 T 	   863.89	   181.13	  3
 19	 L 	   920.43	   130.60	  2
 20	 K 	        -  	    74.06	  1  _
```

**11. IPI00043992 Poliovirus receptor-related protein 4**

| Sequence | Charge | M+H+ | XCorr | DeltaCn |
| --- | --- | --- | --- | --- |
| R.VDAGEGAQELALLHSK.Y | 2 | 1638.804 | 3.13 | 0.489 |

+1 charge

```
		      B   	       Y
  1	 V 	   100.08	        -  	 16
  2	 D 	   215.10	  1538.78	 15
  3	 A 	   286.14	  1423.75	 14
  4	 G 	   343.16	  1352.72	 13
  5	 E 	   472.20	  1295.70	 12
  6	 G 	   529.23	  1166.65	 11
  7	 A 	   600.26	  1109.63	 10
  8	 Q 	   728.32	  1038.59	  9
  9	 E 	   857.36	   910.54	  8
 10	 L 	   970.45	   781.49	  7
 11	 A 	  1041.48	   668.41	  6
 12	 L 	  1154.57	   597.37	  5
 13	 L 	  1267.65	   484.29	  4
 14	 H 	  1404.71	   371.20	  3
 15	 S 	  1491.74	   234.14	  2
 16	 K 	        -  	   147.11	  1  _
+2 charge
		      B   	       Y
  1	 V 	    50.54	        -  	 16
  2	 D 	   108.05	   769.89	 15
  3	 A 	   143.57	   712.38	 14
  4	 G 	   172.08	   676.86	 13
  5	 E 	   236.61	   648.35	 12
  6	 G 	   265.12	   583.83	 11
  7	 A 	   300.63	   555.32	 10
  8	 Q 	   364.66	   519.80	  9
  9	 E 	   429.19	   455.77	  8
 10	 L 	   485.73	   391.25	  7
 11	 A 	   521.25	   334.71	  6
 12	 L 	   577.79	   299.19	  5
 13	 L 	   634.33	   242.65	  4
 14	 H 	   702.86	   186.11	  3
 15	 S 	   746.38	   117.58	  2
 16	 K 	        -  	    74.06	  1  _
```

**12. IPI00384195 Isoform 3 of Plexin-B1**

| Sequence | Charge | M+H+ | XCorr | DeltaCn |
| --- | --- | --- | --- | --- |
| R.PASTTSMTPPGSGSWKRR.P | 2 | 1905.128 | 3.383 | 0.258 |

+1 charge

```
		      B   	       Y
  1	 P 	    98.06	        -  	 18
  2	 A 	   169.10	  1806.89	 17
  3	 S 	   256.13	  1735.85	 16
  4	 T 	   357.18	  1648.82	 15
  5	 T 	   458.22	  1547.77	 14
  6	 S 	   545.26	  1446.73	 13
  7	 M 	   676.30	  1359.70	 12
  8	 T 	   777.34	  1228.65	 11
  9	 P 	   874.40	  1127.61	 10
 10	 P 	   971.45	  1030.55	  9
 11	 G 	  1028.47	   933.50	  8
 12	 S 	  1115.50	   876.48	  7
 13	 G 	  1172.53	   789.45	  6
 14	 S 	  1259.56	   732.43	  5
 15	 W 	  1445.64	   645.39	  4
 16	 K 	  1573.73	   459.32	  3
 17	 R 	  1729.83	   331.22	  2
 18	 R 	        -  	   175.12	  1  _
+2 charge
		      B   	       Y
  1	 P 	    49.53	        -  	 18
  2	 A 	    85.05	   903.95	 17
  3	 S 	   128.57	   868.43	 16
  4	 T 	   179.09	   824.91	 15
  5	 T 	   229.62	   774.39	 14
  6	 S 	   273.13	   723.87	 13
  7	 M 	   338.65	   680.35	 12
  8	 T 	   389.18	   614.83	 11
  9	 P 	   437.70	   564.31	 10
 10	 P 	   486.23	   515.78	  9
 11	 G 	   514.74	   467.25	  8
 12	 S 	   558.26	   438.74	  7
 13	 G 	   586.77	   395.23	  6
 14	 S 	   630.28	   366.72	  5
 15	 W 	   723.32	   323.20	  4
 16	 K 	   787.37	   230.16	  3
 17	 R 	   865.42	   166.11	  2
 18	 R 	        -  	    88.06	  1  _
```

**13 IPI00384401 Myosin-reactive immunoglobulin kappa chain variable region (Fragment)**

| Sequence | Charge | M+H+ | XCorr | DeltaCn |
| --- | --- | --- | --- | --- |
| - .EIVMTQSPATLSVSPGER.A | 2 | 1903.147 | 4.952 | 0.526 |

+1 charge

```
		      B   	       Y
  1	 E 	   130.05	        -  	 18
  2	 I 	   243.13	  1772.92	 17
  3	 V 	   342.20	  1659.84	 16
  4	 M 	   473.24	  1560.77	 15
  5	 T 	   574.29	  1429.73	 14
  6	 Q 	   702.35	  1328.68	 13
  7	 S 	   789.38	  1200.62	 12
  8	 P 	   886.43	  1113.59	 11
  9	 A 	   957.47	  1016.54	 10
 10	 T 	  1058.52	   945.50	  9
 11	 L 	  1171.60	   844.45	  8
 12	 S 	  1258.63	   731.37	  7
 13	 V 	  1357.70	   644.34	  6
 14	 S 	  1444.74	   545.27	  5
 15	 P 	  1541.79	   458.24	  4
 16	 G 	  1598.81	   361.18	  3
 17	 E 	  1727.85	   304.16	  2
 18	 R 	        -  	   175.12	  1  _
+2 charge
		      B   	       Y
  1	 E 	    65.53	        -  	 18
  2	 I 	   122.07	   886.96	 17
  3	 V 	   171.60	   830.42	 16
  4	 M 	   237.13	   780.89	 15
  5	 T 	   287.65	   715.37	 14
  6	 Q 	   351.68	   664.84	 13
  7	 S 	   395.19	   600.81	 12
  8	 P 	   443.72	   557.30	 11
  9	 A 	   479.24	   508.77	 10
 10	 T 	   529.76	   473.25	  9
 11	 L 	   586.31	   422.73	  8
 12	 S 	   629.82	   366.19	  7
 13	 V 	   679.36	   322.67	  6
 14	 S 	   722.87	   273.14	  5
 15	 P 	   771.40	   229.62	  4
 16	 G 	   799.91	   181.10	  3
 17	 E 	   864.43	   152.58	  2
 18	 R 	        -  	    88.06	  1  _
```

**14. IPI00410487 Isoform 1 of Twisted gastrulation protein homolog 1**

| Sequence | Charge | M+H+ | XCorr | DeltaCn |
| --- | --- | --- | --- | --- |
| K.STVEELHEPIPSLFR.A | 2 | 1754.964 | 4.36 | 0.519 |

+1 charge

```
		      B   	       Y
  1	 S 	    88.04	        -  	 15
  2	 T 	   189.09	  1666.88	 14
  3	 V 	   288.16	  1565.83	 13
  4	 E 	   417.20	  1466.76	 12
  5	 E 	   546.24	  1337.72	 11
  6	 L 	   659.32	  1208.68	 10
  7	 H 	   796.38	  1095.59	  9
  8	 E 	   925.43	   958.54	  8
  9	 P 	  1022.48	   829.49	  7
 10	 I 	  1135.56	   732.44	  6
 11	 P 	  1232.62	   619.36	  5
 12	 S 	  1319.65	   522.30	  4
 13	 L 	  1432.73	   435.27	  3
 14	 F 	  1579.80	   322.19	  2
 15	 R 	        -  	   175.12	  1  _
+2 charge
		      B   	       Y
  1	 S 	    44.52	        -  	 15
  2	 T 	    95.05	   833.94	 14
  3	 V 	   144.58	   783.42	 13
  4	 E 	   209.10	   733.89	 12
  5	 E 	   273.62	   669.36	 11
  6	 L 	   330.17	   604.84	 10
  7	 H 	   398.70	   548.30	  9
  8	 E 	   463.22	   479.77	  8
  9	 P 	   511.74	   415.25	  7
 10	 I 	   568.29	   366.72	  6
 11	 P 	   616.81	   310.18	  5
 12	 S 	   660.33	   261.66	  4
 13	 L 	   716.87	   218.14	  3
 14	 F 	   790.40	   161.60	  2
 15	 R 	        -  	    88.06	  1  _
```

**15. IPI00289931 MADCAM1 protein**

| Sequence | Charge | M+H+ | XCorr | DeltaCn |
| --- | --- | --- | --- | --- |
| R.GLDTSLGAVQSDTGR.S | 2 | 1477.559 | 5.385 | 0.569 |

+1 charge

```
		      B   	       Y
  1	 G 	    58.03	        -  	 15
  2	 L 	   171.11	  1419.71	 14
  3	 D 	   286.14	  1306.62	 13
  4	 T 	   387.19	  1191.60	 12
  5	 S 	   474.22	  1090.55	 11
  6	 L 	   587.30	  1003.52	 10
  7	 G 	   644.32	   890.43	  9
  8	 A 	   715.36	   833.41	  8
  9	 V 	   814.43	   762.37	  7
 10	 Q 	   942.49	   663.31	  6
 11	 S 	  1029.52	   535.25	  5
 12	 D 	  1144.55	   448.22	  4
 13	 T 	  1245.60	   333.19	  3
 14	 G 	  1302.62	   232.14	  2
 15	 R 	        -  	   175.12	  1  _
+2 charge
		      B   	       Y
  1	 G 	    29.52	        -  	 15
  2	 L 	    86.06	   710.36	 14
  3	 D 	   143.57	   653.82	 13
  4	 T 	   194.10	   596.30	 12
  5	 S 	   237.61	   545.78	 11
  6	 L 	   294.16	   502.26	 10
  7	 G 	   322.67	   445.72	  9
  8	 A 	   358.18	   417.21	  8
  9	 V 	   407.72	   381.69	  7
 10	 Q 	   471.75	   332.16	  6
 11	 S 	   515.26	   268.13	  5
 12	 D 	   572.78	   224.61	  4
 13	 T 	   623.30	   167.10	  3
 14	 G 	   651.81	   116.57	  2
 15	 R 	        -  	    88.06	  1  _
```

**16. IPI00296058 EGF-containing fibulin-like extracellular matrix protein 2**

| Sequence | Charge | M+H+ | XCorr | DeltaCn |
| --- | --- | --- | --- | --- |
| R.DVNECLTIPEACKGEMK.C | 2 | 1995.228 | 3.155 | 0.412 |

+1 charge

```
		      B   	       Y
  1	 D 	   116.03	        -  	 17
  2	 V 	   215.10	  1879.14	 16
  3	 N 	   329.15	  1780.08	 15
  4	 E 	   458.19	  1666.03	 14
  5	 C 	   618.35	  1536.99	 13
  6	 L 	   731.44	  1376.83	 12
  7	 T 	   832.49	  1263.74	 11
  8	 I 	   945.57	  1162.69	 10
  9	 P 	  1042.62	  1049.61	  9
 10	 E 	  1171.66	   952.56	  8
 11	 A 	  1242.70	   823.51	  7
 12	 C 	  1402.87	   752.48	  6
 13	 K 	  1530.96	   592.31	  5
 14	 G 	  1587.98	   464.22	  4
 15	 E 	  1717.03	   407.20	  3
 16	 M 	  1848.07	   278.15	  2
 17	 K 	        -  	   147.11	  1  _
+2 charge
		      B   	       Y
  1	 D 	    58.52	        -  	 17
  2	 V 	   108.05	   940.08	 16
  3	 N 	   165.08	   890.54	 15
  4	 E 	   229.60	   833.52	 14
  5	 C 	   309.68	   769.00	 13
  6	 L 	   366.22	   688.92	 12
  7	 T 	   416.75	   632.37	 11
  8	 I 	   473.29	   581.85	 10
  9	 P 	   521.81	   525.31	  9
 10	 E 	   586.34	   476.78	  8
 11	 A 	   621.85	   412.26	  7
 12	 C 	   701.94	   376.74	  6
 13	 K 	   765.98	   296.66	  5
 14	 G 	   794.50	   232.61	  4
 15	 E 	   859.02	   204.10	  3
 16	 M 	   924.54	   139.58	  2
 17	 K 	        -  	    74.06	  1  _
```

**17. IPI00651692 Stromal cell-derived factor 1 theta**

| Sequence | Charge | M+H+ | XCorr | DeltaCn |
| --- | --- | --- | --- | --- |
| K.ILNTPNCALQIVAR.L | 2 | 1583.851 | 4.242 | 0.348 |

+1 charge

```
		      B   	       Y
  1	 I 	   114.09	        -  	 14
  2	 L 	   227.18	  1469.92	 13
  3	 N 	   341.22	  1356.84	 12
  4	 T 	   442.27	  1242.80	 11
  5	 P 	   539.32	  1141.75	 10
  6	 N 	   653.36	  1044.70	  9
  7	 C 	   813.53	   930.65	  8
  8	 A 	   884.56	   770.49	  7
  9	 L 	   997.65	   699.45	  6
 10	 Q 	  1125.71	   586.37	  5
 11	 I 	  1238.79	   458.31	  4
 12	 V 	  1337.86	   345.22	  3
 13	 A 	  1408.90	   246.16	  2
 14	 R 	        -  	   175.12	  1  _
+2 charge
		      B   	       Y
  1	 I 	    57.55	        -  	 14
  2	 L 	   114.09	   735.47	 13
  3	 N 	   171.11	   678.92	 12
  4	 T 	   221.64	   621.90	 11
  5	 P 	   270.16	   571.38	 10
  6	 N 	   327.18	   522.85	  9
  7	 C 	   407.27	   465.83	  8
  8	 A 	   442.79	   385.75	  7
  9	 L 	   499.33	   350.23	  6
 10	 Q 	   563.36	   293.69	  5
 11	 I 	   619.90	   229.66	  4
 12	 V 	   669.43	   173.12	  3
 13	 A 	   704.95	   123.58	  2
 14	 R 	        -  	    88.06	  1  _
```

**18. IPI00006034 Cysteine-rich protein 2**

| Sequence | Charge | M+H+ | XCorr | DeltaCn |
| --- | --- | --- | --- | --- |
| K.GVNIGGAGSYIYEKPLAEGPQVTGPIEVPAAR.A | 3 | 3212.601 | 3.452 | 0.481 |

+1 charge

```
		      B   	       Y
  1	 G 	    58.03	        -  	 32
  2	 V 	   157.10	  3153.66	 31
  3	 N 	   271.14	  3054.59	 30
  4	 I 	   384.22	  2940.55	 29
  5	 G 	   441.25	  2827.46	 28
  6	 G 	   498.27	  2770.44	 27
  7	 A 	   569.30	  2713.42	 26
  8	 G 	   626.33	  2642.38	 25
  9	 S 	   713.36	  2585.36	 24
 10	 Y 	   876.42	  2498.33	 23
 11	 I 	   989.51	  2335.27	 22
 12	 Y 	  1152.57	  2222.18	 21
 13	 E 	  1281.61	  2059.12	 20
 14	 K 	  1409.71	  1930.08	 19
 15	 P 	  1506.76	  1801.98	 18
 16	 L 	  1619.84	  1704.93	 17
 17	 A 	  1690.88	  1591.84	 16
 18	 E 	  1819.92	  1520.81	 15
 19	 G 	  1876.94	  1391.76	 14
 20	 P 	  1974.00	  1334.74	 13
 21	 Q 	  2102.06	  1237.69	 12
 22	 V 	  2201.12	  1109.63	 11
 23	 T 	  2302.17	  1010.56	 10
 24	 G 	  2359.19	   909.52	  9
 25	 P 	  2456.25	   852.49	  8
 26	 I 	  2569.33	   755.44	  7
 27	 E 	  2698.37	   642.36	  6
 28	 V 	  2797.44	   513.31	  5
 29	 P 	  2894.49	   414.25	  4
 30	 A 	  2965.53	   317.19	  3
 31	 A 	  3036.57	   246.16	  2
 32	 R 	        -  	   175.12	  1  _


+2 charge
		      B   	       Y
  1	 G 	    29.52	        -  	 32
  2	 V 	    79.05	  1577.33	 31
  3	 N 	   136.07	  1527.80	 30
  4	 I 	   192.62	  1470.78	 29
  5	 G 	   221.13	  1414.23	 28
  6	 G 	   249.64	  1385.72	 27
  7	 A 	   285.16	  1357.21	 26
  8	 G 	   313.67	  1321.69	 25
  9	 S 	   357.18	  1293.18	 24
 10	 Y 	   438.71	  1249.67	 23
 11	 I 	   495.26	  1168.14	 22
 12	 Y 	   576.79	  1111.59	 21
 13	 E 	   641.31	  1030.06	 20
 14	 K 	   705.36	   965.54	 19
 15	 P 	   753.88	   901.49	 18
 16	 L 	   810.43	   852.97	 17
 17	 A 	   845.94	   796.43	 16
 18	 E 	   910.46	   760.91	 15
 19	 G 	   938.98	   696.39	 14
 20	 P 	   987.50	   667.87	 13
 21	 Q 	  1051.53	   619.35	 12
 22	 V 	  1101.07	   555.32	 11
 23	 T 	  1151.59	   505.79	 10
 24	 G 	  1180.10	   455.26	  9
 25	 P 	  1228.63	   426.75	  8
 26	 I 	  1285.17	   378.22	  7
 27	 E 	  1349.69	   321.68	  6
 28	 V 	  1399.22	   257.16	  5
 29	 P 	  1447.75	   207.63	  4
 30	 A 	  1483.27	   159.10	  3
 31	 A 	  1518.79	   123.58	  2
 32	 R 	        -  	    88.06	  1  _


+3 charge
		      B   	       Y
  1	 G 	    20.01	        -  	 32
  2	 V 	    53.04	  1051.89	 31
  3	 N 	    91.05	  1018.87	 30
  4	 I 	   128.75	   980.85	 29
  5	 G 	   147.75	   943.16	 28
  6	 G 	   166.76	   924.15	 27
  7	 A 	   190.44	   905.14	 26
  8	 G 	   209.45	   881.47	 25
  9	 S 	   238.46	   862.46	 24
 10	 Y 	   292.81	   833.45	 23
 11	 I 	   330.51	   779.09	 22
 12	 Y 	   384.86	   741.40	 21
 13	 E 	   427.88	   687.04	 20
 14	 K 	   470.57	   644.03	 19
 15	 P 	   502.92	   601.33	 18
 16	 L 	   540.62	   568.98	 17
 17	 A 	   564.30	   531.29	 16
 18	 E 	   607.31	   507.61	 15
 19	 G 	   626.32	   464.59	 14
 20	 P 	   658.67	   445.59	 13
 21	 Q 	   701.36	   413.23	 12
 22	 V 	   734.38	   370.55	 11
 23	 T 	   768.06	   337.53	 10
 24	 G 	   787.07	   303.84	  9
 25	 P 	   819.42	   284.84	  8
 26	 I 	   857.11	   252.49	  7
 27	 E 	   900.13	   214.79	  6
 28	 V 	   933.15	   171.78	  5
 29	 P 	   965.50	   138.75	  4
 30	 A 	   989.18	   106.40	  3
 31	 A 	  1012.86	    82.72	  2
 32	 R 	        -  	    59.04	  1  _
```

**19. IPI00409698 Isoform 1 of Transportin-2**

| Sequence | Charge | M+H+ | XCorr | DeltaCn |
| --- | --- | --- | --- | --- |
| K.QECLNNIGDASSLIR.A | 2 | 1690.831 | 3.193 | 0.225 |

+1 charge

```
		      B   	       Y
  1	 Q 	   129.07	        -  	 15
  2	 E 	   258.11	  1561.90	 14
  3	 C 	   418.27	  1432.86	 13
  4	 L 	   531.36	  1272.69	 12
  5	 N 	   645.40	  1159.61	 11
  6	 N 	   759.44	  1045.56	 10
  7	 I 	   872.53	   931.52	  9
  8	 G 	   929.55	   818.44	  8
  9	 D 	  1044.58	   761.42	  7
 10	 A 	  1115.61	   646.39	  6
 11	 S 	  1202.65	   575.35	  5
 12	 S 	  1289.68	   488.32	  4
 13	 L 	  1402.76	   401.29	  3
 14	 I 	  1515.85	   288.20	  2
 15	 R 	        -  	   175.12	  1  _
+2 charge
		      B   	       Y
  1	 Q 	    65.04	        -  	 15
  2	 E 	   129.56	   781.45	 14
  3	 C 	   209.64	   716.93	 13
  4	 L 	   266.18	   636.85	 12
  5	 N 	   323.20	   580.31	 11
  6	 N 	   380.23	   523.29	 10
  7	 I 	   436.77	   466.26	  9
  8	 G 	   465.28	   409.72	  8
  9	 D 	   522.79	   381.21	  7
 10	 A 	   558.31	   323.70	  6
 11	 S 	   601.83	   288.18	  5
 12	 S 	   645.34	   244.66	  4
 13	 L 	   701.88	   201.15	  3
 14	 I 	   758.43	   144.61	  2
 15	 R 	        -  	    88.06	  1  _
```

**20. IPI00304903 Cornifin-B**

| Sequence | Charge | M+H+ | XCorr | DeltaCn |
| --- | --- | --- | --- | --- |
| K.VPEPCPSIVTPAPAQQK.T | 2 | 1820.073 | 3.435 | 0.412 |

+1 charge

```
		      B   	       Y
  1	 V 	   100.08	        -  	 17
  2	 P 	   197.13	  1720.01	 16
  3	 E 	   326.17	  1622.96	 15
  4	 P 	   423.22	  1493.91	 14
  5	 C 	   583.39	  1396.86	 13
  6	 P 	   680.44	  1236.69	 12
  7	 S 	   767.47	  1139.64	 11
  8	 I 	   880.56	  1052.61	 10
  9	 V 	   979.63	   939.53	  9
 10	 T 	  1080.67	   840.46	  8
 11	 P 	  1177.73	   739.41	  7
 12	 A 	  1248.76	   642.36	  6
 13	 P 	  1345.82	   571.32	  5
 14	 A 	  1416.85	   474.27	  4
 15	 Q 	  1544.91	   403.23	  3
 16	 Q 	  1672.97	   275.17	  2
 17	 K 	        -  	   147.11	  1  _
+2 charge
		      B   	       Y
  1	 V 	    50.54	        -  	 17
  2	 P 	    99.07	   860.51	 16
  3	 E 	   163.59	   811.98	 15
  4	 P 	   212.12	   747.46	 14
  5	 C 	   292.20	   698.93	 13
  6	 P 	   340.72	   618.85	 12
  7	 S 	   384.24	   570.32	 11
  8	 I 	   440.78	   526.81	 10
  9	 V 	   490.32	   470.27	  9
 10	 T 	   540.84	   420.73	  8
 11	 P 	   589.37	   370.21	  7
 12	 A 	   624.89	   321.68	  6
 13	 P 	   673.41	   286.16	  5
 14	 A 	   708.93	   237.64	  4
 15	 Q 	   772.96	   202.12	  3
 16	 Q 	   836.99	   138.09	  2
 17	 K 	        -  	    74.06	  1  _
```

**21. IPI00027436 Tumor necrosis factor receptor superfamily member 16**

| Sequence | Charge | M+H+ | XCorr | DeltaCn |
| --- | --- | --- | --- | --- |
| R.WADAECEEIPGR.W | 2 | 1433.499 | 3.688 | 0.364 |

+1 charge

```
		      B   	       Y
  1	 W 	   187.09	        -  	 12
  2	 A 	   258.12	  1246.67	 11
  3	 D 	   373.15	  1175.63	 10
  4	 A 	   444.19	  1060.61	  9
  5	 E 	   573.23	   989.57	  8
  6	 C 	   733.40	   860.53	  7
  7	 E 	   862.44	   700.36	  6
  8	 E 	   991.48	   571.32	  5
  9	 I 	  1104.56	   442.28	  4
 10	 P 	  1201.62	   329.19	  3
 11	 G 	  1258.64	   232.14	  2
 12	 R 	        -  	   175.12	  1  _
+2 charge
		      B   	       Y
  1	 W 	    94.05	        -  	 12
  2	 A 	   129.57	   623.84	 11
  3	 D 	   187.08	   588.32	 10
  4	 A 	   222.60	   530.81	  9
  5	 E 	   287.12	   495.29	  8
  6	 C 	   367.20	   430.77	  7
  7	 E 	   431.72	   350.68	  6
  8	 E 	   496.24	   286.16	  5
  9	 I 	   552.79	   221.64	  4
 10	 P 	   601.31	   165.10	  3
 11	 G 	   629.82	   116.57	  2
 12	 R 	        -  	    88.06	  1  _
```

**22. IPI00011899 BMP and activin membrane-bound inhibitor homolog**

| Sequence | Charge | M+H+ | XCorr | DeltaCn |
| --- | --- | --- | --- | --- |
| R.LLDPQNSNSPLTHGCLDSLASTTDICQAK.Q | 3 | 3158.408 | 4.22 | 0.475 |

+1 charge

```
		      B   	       Y
  1	 L 	   114.09	        -  	 29
  2	 L 	   227.18	  3043.68	 28
  3	 D 	   342.20	  2930.59	 27
  4	 P 	   439.26	  2815.57	 26
  5	 Q 	   567.31	  2718.51	 25
  6	 N 	   681.36	  2590.46	 24
  7	 S 	   768.39	  2476.41	 23
  8	 N 	   882.43	  2389.38	 22
  9	 S 	   969.46	  2275.34	 21
 10	 P 	  1066.52	  2188.31	 20
 11	 L 	  1179.60	  2091.25	 19
 12	 T 	  1280.65	  1978.17	 18
 13	 H 	  1417.71	  1877.12	 17
 14	 G 	  1474.73	  1740.06	 16
 15	 C 	  1634.89	  1683.04	 15
 16	 L 	  1747.98	  1522.88	 14
 17	 D 	  1863.00	  1409.79	 13
 18	 S 	  1950.04	  1294.77	 12
 19	 L 	  2063.12	  1207.73	 11
 20	 A 	  2134.16	  1094.65	 10
 21	 S 	  2221.19	  1023.61	  9
 22	 T 	  2322.24	   936.58	  8
 23	 T 	  2423.29	   835.53	  7
 24	 D 	  2538.31	   734.48	  6
 25	 I 	  2651.40	   619.46	  5
 26	 C 	  2811.56	   506.37	  4
 27	 Q 	  2939.62	   346.21	  3
 28	 A 	  3010.66	   218.15	  2
 29	 K 	        -  	   147.11	  1  _


+2 charge
		      B   	       Y
  1	 L 	    57.55	        -  	 29
  2	 L 	   114.09	  1522.34	 28
  3	 D 	   171.60	  1465.80	 27
  4	 P 	   220.13	  1408.29	 26
  5	 Q 	   284.16	  1359.76	 25
  6	 N 	   341.18	  1295.73	 24
  7	 S 	   384.70	  1238.71	 23
  8	 N 	   441.72	  1195.19	 22
  9	 S 	   485.24	  1138.17	 21
 10	 P 	   533.76	  1094.66	 20
 11	 L 	   590.30	  1046.13	 19
 12	 T 	   640.83	   989.59	 18
 13	 H 	   709.36	   939.06	 17
 14	 G 	   737.87	   870.54	 16
 15	 C 	   817.95	   842.02	 15
 16	 L 	   874.49	   761.94	 14
 17	 D 	   932.01	   705.40	 13
 18	 S 	   975.52	   647.89	 12
 19	 L 	  1032.06	   604.37	 11
 20	 A 	  1067.58	   547.83	 10
 21	 S 	  1111.10	   512.31	  9
 22	 T 	  1161.62	   468.79	  8
 23	 T 	  1212.15	   418.27	  7
 24	 D 	  1269.66	   367.75	  6
 25	 I 	  1326.20	   310.23	  5
 26	 C 	  1406.28	   253.69	  4
 27	 Q 	  1470.31	   173.61	  3
 28	 A 	  1505.83	   109.58	  2
 29	 K 	        -  	    74.06	  1  _


+3 charge
		      B   	       Y
  1	 L 	    38.70	        -  	 29
  2	 L 	    76.40	  1015.23	 28
  3	 D 	   114.74	   977.54	 27
  4	 P 	   147.09	   939.19	 26
  5	 Q 	   189.78	   906.84	 25
  6	 N 	   227.79	   864.16	 24
  7	 S 	   256.80	   826.14	 23
  8	 N 	   294.82	   797.13	 22
  9	 S 	   323.83	   759.12	 21
 10	 P 	   356.18	   730.11	 20
 11	 L 	   393.87	   697.76	 19
 12	 T 	   427.55	   660.06	 18
 13	 H 	   473.24	   626.38	 17
 14	 G 	   492.25	   580.69	 16
 15	 C 	   545.64	   561.69	 15
 16	 L 	   583.33	   508.30	 14
 17	 D 	   621.67	   470.60	 13
 18	 S 	   650.68	   432.26	 12
 19	 L 	   688.38	   403.25	 11
 20	 A 	   712.06	   365.55	 10
 21	 S 	   741.07	   341.88	  9
 22	 T 	   774.75	   312.86	  8
 23	 T 	   808.43	   279.18	  7
 24	 D 	   846.78	   245.50	  6
 25	 I 	   884.47	   207.16	  5
 26	 C 	   937.86	   169.46	  4
 27	 Q 	   980.54	   116.07	  3
 28	 A 	  1004.22	    73.39	  2
 29	 K 	        -  	    49.71	  1  _
```

**23. IPI00018769 Thrombospondin-2**

| Sequence | Charge | M+H+ | XCorr | DeltaCn |
| --- | --- | --- | --- | --- |
| R.SCDVTSNTCLGPSIQTR.A | 2 | 1897.021 | 3.559 | 0.436 |

+1 charge

```
		      B   	       Y
  1	 S 	    88.04	        -  	 17
  2	 C 	   248.20	  1809.10	 16
  3	 D 	   363.23	  1648.93	 15
  4	 V 	   462.30	  1533.90	 14
  5	 T 	   563.35	  1434.84	 13
  6	 S 	   650.38	  1333.79	 12
  7	 N 	   764.42	  1246.76	 11
  8	 T 	   865.47	  1132.71	 10
  9	 C 	  1025.64	  1031.66	  9
 10	 L 	  1138.72	   871.50	  8
 11	 G 	  1195.74	   758.42	  7
 12	 P 	  1292.79	   701.39	  6
 13	 S 	  1379.83	   604.34	  5
 14	 I 	  1492.91	   517.31	  4
 15	 Q 	  1620.97	   404.23	  3
 16	 T 	  1722.02	   276.17	  2
 17	 R 	        -  	   175.12	  1  _
+2 charge
		      B   	       Y
  1	 S 	    44.52	        -  	 17
  2	 C 	   124.61	   905.05	 16
  3	 D 	   182.12	   824.97	 15
  4	 V 	   231.65	   767.46	 14
  5	 T 	   282.18	   717.92	 13
  6	 S 	   325.69	   667.40	 12
  7	 N 	   382.71	   623.88	 11
  8	 T 	   433.24	   566.86	 10
  9	 C 	   513.32	   516.34	  9
 10	 L 	   569.86	   436.25	  8
 11	 G 	   598.37	   379.71	  7
 12	 P 	   646.90	   351.20	  6
 13	 S 	   690.42	   302.67	  5
 14	 I 	   746.96	   259.16	  4
 15	 Q 	   810.99	   202.62	  3
 16	 T 	   861.51	   138.59	  2
 17	 R 	        -  	    88.06	  1  _
```

**24. IPI00555877 NADH dehydrogenase (Ubiquinone) 1 alpha subcomplex, 10, 42kDa variant (Fragment)**

| Sequence | Charge | M+H+ | XCorr | DeltaCn |
| --- | --- | --- | --- | --- |
| K.GDVMQFSIKGQASLF.- | 2 | 1628.874 | 2.87 | 0.314 |

+1 charge

```
		      B   	       Y
  1	 G 	    58.03	        -  	 15
  2	 D 	   173.06	  1570.79	 14
  3	 V 	   272.12	  1455.77	 13
  4	 M 	   403.16	  1356.70	 12
  5	 Q 	   531.22	  1225.66	 11
  6	 F 	   678.29	  1097.60	 10
  7	 S 	   765.32	   950.53	  9
  8	 I 	   878.41	   863.50	  8
  9	 K 	  1006.50	   750.41	  7
 10	 G 	  1063.52	   622.32	  6
 11	 Q 	  1191.58	   565.30	  5
 12	 A 	  1262.62	   437.24	  4
 13	 S 	  1349.65	   366.20	  3
 14	 L 	  1462.74	   279.17	  2
 15	 F 	        -  	   166.09	  1  _
+2 charge
		      B   	       Y
  1	 G 	    29.52	        -  	 15
  2	 D 	    87.03	   785.90	 14
  3	 V 	   136.57	   728.39	 13
  4	 M 	   202.09	   678.85	 12
  5	 Q 	   266.12	   613.33	 11
  6	 F 	   339.65	   549.30	 10
  7	 S 	   383.17	   475.77	  9
  8	 I 	   439.71	   432.25	  8
  9	 K 	   503.75	   375.71	  7
 10	 G 	   532.27	   311.66	  6
 11	 Q 	   596.29	   283.15	  5
 12	 A 	   631.81	   219.12	  4
 13	 S 	   675.33	   183.60	  3
 14	 L 	   731.87	   140.09	  2
 15	 F 	        -  	    83.55	  1  _
```

**25. IPI00939667 Isoform 1 of Tripeptidyl-peptidase 1**

| Sequence | Charge | M+H+ | XCorr | DeltaCn |
| --- | --- | --- | --- | --- |
| R.LFGGNFAHQASVAR.V | 2 | 1475.636 | 3.003 | 0.353 |

+1 charge

```
		      B   	       Y
  1	 L 	   114.09	        -  	 14
  2	 F 	   261.16	  1361.67	 13
  3	 G 	   318.18	  1214.60	 12
  4	 G 	   375.20	  1157.58	 11
  5	 N 	   489.25	  1100.56	 10
  6	 F 	   636.31	   986.52	  9
  7	 A 	   707.35	   839.45	  8
  8	 H 	   844.41	   768.41	  7
  9	 Q 	   972.47	   631.35	  6
 10	 A 	  1043.51	   503.29	  5
 11	 S 	  1130.54	   432.26	  4
 12	 V 	  1229.61	   345.22	  3
 13	 A 	  1300.64	   246.16	  2
 14	 R 	        -  	   175.12	  1  _
+2 charge
		      B   	       Y
  1	 L 	    57.55	        -  	 14
  2	 F 	   131.08	   681.34	 13
  3	 G 	   159.59	   607.80	 12
  4	 G 	   188.10	   579.29	 11
  5	 N 	   245.13	   550.78	 10
  6	 F 	   318.66	   493.76	  9
  7	 A 	   354.18	   420.23	  8
  8	 H 	   422.71	   384.71	  7
  9	 Q 	   486.74	   316.18	  6
 10	 A 	   522.26	   252.15	  5
 11	 S 	   565.77	   216.63	  4
 12	 V 	   615.31	   173.12	  3
 13	 A 	   650.83	   123.58	  2
 14	 R 	        -  	    88.06	  1  _
```

**26. IPI00008787 Alpha-N-acetylglucosaminidase**

| Sequence | Charge | M+H+ | XCorr | DeltaCn |
| --- | --- | --- | --- | --- |
| R.LLGPGPAADFSVSVER.A | 2 | 1615.812 | 4.516 | 0.518 |

+1 charge

```
		      B   	       Y
  1	 L 	   114.09	        -  	 16
  2	 L 	   227.18	  1501.76	 15
  3	 G 	   284.20	  1388.68	 14
  4	 P 	   381.25	  1331.66	 13
  5	 G 	   438.27	  1234.61	 12
  6	 P 	   535.32	  1177.58	 11
  7	 A 	   606.36	  1080.53	 10
  8	 A 	   677.40	  1009.49	  9
  9	 D 	   792.43	   938.46	  8
 10	 F 	   939.49	   823.43	  7
 11	 S 	  1026.53	   676.36	  6
 12	 V 	  1125.59	   589.33	  5
 13	 S 	  1212.63	   490.26	  4
 14	 V 	  1311.69	   403.23	  3
 15	 E 	  1440.74	   304.16	  2
 16	 R 	        -  	   175.12	  1  _
+2 charge
		      B   	       Y
  1	 L 	    57.55	        -  	 16
  2	 L 	   114.09	   751.39	 15
  3	 G 	   142.60	   694.84	 14
  4	 P 	   191.13	   666.33	 13
  5	 G 	   219.64	   617.81	 12
  6	 P 	   268.17	   589.30	 11
  7	 A 	   303.68	   540.77	 10
  8	 A 	   339.20	   505.25	  9
  9	 D 	   396.72	   469.73	  8
 10	 F 	   470.25	   412.22	  7
 11	 S 	   513.77	   338.68	  6
 12	 V 	   563.30	   295.17	  5
 13	 S 	   606.82	   245.63	  4
 14	 V 	   656.35	   202.12	  3
 15	 E 	   720.87	   152.58	  2
 16	 R 	        -  	    88.06	  1  _
```

**27. IPI00024331 Tumor necrosis factor receptor superfamily member 14**

| Sequence | Charge | M+H+ | XCorr | DeltaCn |
| --- | --- | --- | --- | --- |
| K.EACGELTGTVCEPCPPGTYIAHLNGLSK.C | 3 | 3033.317 | 4.018 | 0.458 |

+1 charge

```
		      B   	       Y
  1	 E 	   130.05	        -  	 28
  2	 A 	   201.09	  2902.76	 27
  3	 C 	   361.25	  2831.72	 26
  4	 G 	   418.27	  2671.55	 25
  5	 E 	   547.32	  2614.53	 24
  6	 L 	   660.40	  2485.49	 23
  7	 T 	   761.45	  2372.41	 22
  8	 G 	   818.47	  2271.36	 21
  9	 T 	   919.52	  2214.34	 20
 10	 V 	  1018.59	  2113.29	 19
 11	 C 	  1178.75	  2014.22	 18
 12	 E 	  1307.79	  1854.06	 17
 13	 P 	  1404.85	  1725.01	 16
 14	 C 	  1565.01	  1627.96	 15
 15	 P 	  1662.06	  1467.80	 14
 16	 P 	  1759.12	  1370.74	 13
 17	 G 	  1816.14	  1273.69	 12
 18	 T 	  1917.19	  1216.67	 11
 19	 Y 	  2080.25	  1115.62	 10
 20	 I 	  2193.33	   952.56	  9
 21	 A 	  2264.37	   839.47	  8
 22	 H 	  2401.43	   768.44	  7
 23	 L 	  2514.51	   631.38	  6
 24	 N 	  2628.56	   518.29	  5
 25	 G 	  2685.58	   404.25	  4
 26	 L 	  2798.66	   347.23	  3
 27	 S 	  2885.69	   234.14	  2
 28	 K 	        -  	   147.11	  1  _


+2 charge
		      B   	       Y
  1	 E 	    65.53	        -  	 28
  2	 A 	   101.05	  1451.88	 27
  3	 C 	   181.13	  1416.36	 26
  4	 G 	   209.64	  1336.28	 25
  5	 E 	   274.16	  1307.77	 24
  6	 L 	   330.70	  1243.25	 23
  7	 T 	   381.23	  1186.71	 22
  8	 G 	   409.74	  1136.18	 21
  9	 T 	   460.26	  1107.67	 20
 10	 V 	   509.80	  1057.15	 19
 11	 C 	   589.88	  1007.61	 18
 12	 E 	   654.40	   927.53	 17
 13	 P 	   702.93	   863.01	 16
 14	 C 	   783.01	   814.48	 15
 15	 P 	   831.54	   734.40	 14
 16	 P 	   880.06	   685.87	 13
 17	 G 	   908.57	   637.35	 12
 18	 T 	   959.10	   608.84	 11
 19	 Y 	  1040.63	   558.31	 10
 20	 I 	  1097.17	   476.78	  9
 21	 A 	  1132.69	   420.24	  8
 22	 H 	  1201.22	   384.72	  7
 23	 L 	  1257.76	   316.19	  6
 24	 N 	  1314.78	   259.65	  5
 25	 G 	  1343.29	   202.63	  4
 26	 L 	  1399.83	   174.12	  3
 27	 S 	  1443.35	   117.58	  2
 28	 K 	        -  	    74.06	  1  _


+3 charge
		      B   	       Y
  1	 E 	    44.02	        -  	 28
  2	 A 	    67.70	   968.26	 27
  3	 C 	   121.09	   944.58	 26
  4	 G 	   140.10	   891.19	 25
  5	 E 	   183.11	   872.18	 24
  6	 L 	   220.80	   829.17	 23
  7	 T 	   254.49	   791.47	 22
  8	 G 	   273.49	   757.79	 21
  9	 T 	   307.18	   738.78	 20
 10	 V 	   340.20	   705.10	 19
 11	 C 	   393.59	   672.08	 18
 12	 E 	   436.60	   618.69	 17
 13	 P 	   468.95	   575.68	 16
 14	 C 	   522.34	   543.33	 15
 15	 P 	   554.69	   489.94	 14
 16	 P 	   587.04	   457.59	 13
 17	 G 	   606.05	   425.23	 12
 18	 T 	   639.73	   406.23	 11
 19	 Y 	   694.09	   372.55	 10
 20	 I 	   731.78	   318.19	  9
 21	 A 	   755.46	   280.50	  8
 22	 H 	   801.15	   256.82	  7
 23	 L 	   838.84	   211.13	  6
 24	 N 	   876.86	   173.44	  5
 25	 G 	   895.86	   135.42	  4
 26	 L 	   933.56	   116.41	  3
 27	 S 	   962.57	    78.72	  2
 28	 K 	        -  	    49.71	  1  _
```

**28. IPI00186554 Serine/arginine repetitive matrix protein 4**

| Sequence | Charge | M+H+ | XCorr | DeltaCn |
| --- | --- | --- | --- | --- |
| R.SQEYDSGNDTSSPPSTQTSSARSRGQEK.G | 3 | 2988.987 | 3.632 | 0.264 |

```
+1 charge
		      B   	       Y
  1	 S 	    88.04	        -  	 28
  2	 Q 	   216.10	  2900.29	 27
  3	 E 	   345.14	  2772.23	 26
  4	 Y 	   508.20	  2643.19	 25
  5	 D 	   623.23	  2480.12	 24
  6	 S 	   710.26	  2365.10	 23
  7	 G 	   767.28	  2278.07	 22
  8	 N 	   881.33	  2221.04	 21
  9	 D 	   996.35	  2107.00	 20
 10	 T 	  1097.40	  1991.97	 19
 11	 S 	  1184.43	  1890.93	 18
 12	 S 	  1271.47	  1803.89	 17
 13	 P 	  1368.52	  1716.86	 16
 14	 P 	  1465.57	  1619.81	 15
 15	 S 	  1552.60	  1522.76	 14
 16	 T 	  1653.65	  1435.72	 13
 17	 Q 	  1781.71	  1334.68	 12
 18	 T 	  1882.76	  1206.62	 11
 19	 S 	  1969.79	  1105.57	 10
 20	 S 	  2056.82	  1018.54	  9
 21	 A 	  2127.86	   931.51	  8
 22	 R 	  2283.96	   860.47	  7
 23	 S 	  2370.99	   704.37	  6
 24	 R 	  2527.09	   617.34	  5
 25	 G 	  2584.11	   461.24	  4
 26	 Q 	  2712.17	   404.21	  3
 27	 E 	  2841.22	   276.16	  2
 28	 K 	        -  	   147.11	  1  _


+2 charge
		      B   	       Y
  1	 S 	    44.52	        -  	 28
  2	 Q 	   108.55	  1450.65	 27
  3	 E 	   173.07	  1386.62	 26
  4	 Y 	   254.61	  1322.10	 25
  5	 D 	   312.12	  1240.57	 24
  6	 S 	   355.64	  1183.05	 23
  7	 G 	   384.15	  1139.54	 22
  8	 N 	   441.17	  1111.03	 21
  9	 D 	   498.68	  1054.00	 20
 10	 T 	   549.20	   996.49	 19
 11	 S 	   592.72	   945.97	 18
 12	 S 	   636.24	   902.45	 17
 13	 P 	   684.76	   858.93	 16
 14	 P 	   733.29	   810.41	 15
 15	 S 	   776.81	   761.88	 14
 16	 T 	   827.33	   718.37	 13
 17	 Q 	   891.36	   667.84	 12
 18	 T 	   941.88	   603.81	 11
 19	 S 	   985.40	   553.29	 10
 20	 S 	  1028.91	   509.77	  9
 21	 A 	  1064.43	   466.26	  8
 22	 R 	  1142.48	   430.74	  7
 23	 S 	  1186.00	   352.69	  6
 24	 R 	  1264.05	   309.17	  5
 25	 G 	  1292.56	   231.12	  4
 26	 Q 	  1356.59	   202.61	  3
 27	 E 	  1421.11	   138.58	  2
 28	 K 	        -  	    74.06	  1  _


+3 charge
		      B   	       Y
  1	 S 	    30.02	        -  	 28
  2	 Q 	    72.70	   967.43	 27
  3	 E 	   115.72	   924.75	 26
  4	 Y 	   170.07	   881.73	 25
  5	 D 	   208.42	   827.38	 24
  6	 S 	   237.43	   789.04	 23
  7	 G 	   256.43	   760.03	 22
  8	 N 	   294.45	   741.02	 21
  9	 D 	   332.79	   703.01	 20
 10	 T 	   366.47	   664.66	 19
 11	 S 	   395.48	   630.98	 18
 12	 S 	   424.49	   601.97	 17
 13	 P 	   456.84	   572.96	 16
 14	 P 	   489.20	   540.61	 15
 15	 S 	   518.21	   508.26	 14
 16	 T 	   551.89	   479.25	 13
 17	 Q 	   594.57	   445.56	 12
 18	 T 	   628.26	   402.88	 11
 19	 S 	   657.27	   369.20	 10
 20	 S 	   686.28	   340.18	  9
 21	 A 	   709.96	   311.17	  8
 22	 R 	   761.99	   287.49	  7
 23	 S 	   791.00	   235.46	  6
 24	 R 	   843.04	   206.45	  5
 25	 G 	   862.04	   154.42	  4
 26	 Q 	   904.73	   135.41	  3
 27	 E 	   947.74	    92.72	  2
 28	 K 	        -  	    49.71	  1  _
```

**29. IPI00152018 Isoform 1 of CMRF35-like molecule 9**

| Sequence | Charge | M+H+ | XCorr | DeltaCn |
| --- | --- | --- | --- | --- |
| R.KGGILFSR.C | 2 | 878.0538 | 3.076 | 0.337 |

+1 charge

```
		      B   	       Y
  1	 K 	   129.10	        -  	  8
  2	 G 	   186.12	   749.43	  7
  3	 G 	   243.15	   692.41	  6
  4	 I 	   356.23	   635.39	  5
  5	 L 	   469.31	   522.30	  4
  6	 F 	   616.38	   409.22	  3
  7	 S 	   703.41	   262.15	  2
  8	 R 	        -  	   175.12	  1  _
+2 charge
		      B   	       Y
  1	 K 	    65.05	        -  	  8
  2	 G 	    93.57	   375.22	  7
  3	 G 	   122.08	   346.71	  6
  4	 I 	   178.62	   318.20	  5
  5	 L 	   235.16	   261.66	  4
  6	 F 	   308.69	   205.11	  3
  7	 S 	   352.21	   131.58	  2
  8	 R 	        -  	    88.06	  1  _
```

**30. IPI00441498 Folate receptor alpha**

| Sequence | Charge | M+H+ | XCorr | DeltaCn |
| --- | --- | --- | --- | --- |
| R.TELLNVCMNAK.H | 2 | 1293.509 | 3.112 | 0.417 |

+1 charge

```
		      B   	       Y
  1	 T 	   102.05	        -  	 11
  2	 E 	   231.10	  1191.72	 10
  3	 L 	   344.18	  1062.68	  9
  4	 L 	   457.27	   949.59	  8
  5	 N 	   571.31	   836.51	  7
  6	 V 	   670.38	   722.47	  6
  7	 C 	   830.54	   623.40	  5
  8	 M 	   961.58	   463.23	  4
  9	 N 	  1075.63	   332.19	  3
 10	 A 	  1146.66	   218.15	  2
 11	 K 	        -  	   147.11	  1  _
+2 charge
		      B   	       Y
  1	 T 	    51.53	        -  	 11
  2	 E 	   116.05	   596.36	 10
  3	 L 	   172.59	   531.84	  9
  4	 L 	   229.14	   475.30	  8
  5	 N 	   286.16	   418.76	  7
  6	 V 	   335.69	   361.74	  6
  7	 C 	   415.77	   312.20	  5
  8	 M 	   481.30	   232.12	  4
  9	 N 	   538.32	   166.60	  3
 10	 A 	   573.84	   109.58	  2
 11	 K 	        -  	    74.06	  1  _
```

**31. IPI00289831 Isoform PTPS of Receptor-type tyrosine-protein phosphatase S**

| Sequence | Charge | M+H+ | XCorr | DeltaCn |
| --- | --- | --- | --- | --- |
| R.SPQGLGAFTPVVR.Q | 2 | 1329.53 | 2.937 | 0.491 |

+1 charge

```
		      B   	       Y
  1	 S 	    88.04	        -  	 13
  2	 P 	   185.09	  1241.70	 12
  3	 Q 	   313.15	  1144.65	 11
  4	 G 	   370.17	  1016.59	 10
  5	 L 	   483.26	   959.57	  9
  6	 G 	   540.28	   846.48	  8
  7	 A 	   611.31	   789.46	  7
  8	 F 	   758.38	   718.42	  6
  9	 T 	   859.43	   571.36	  5
 10	 P 	   956.48	   470.31	  4
 11	 V 	  1055.55	   373.26	  3
 12	 V 	  1154.62	   274.19	  2
 13	 R 	        -  	   175.12	  1  _
+2 charge
		      B   	       Y
  1	 S 	    44.52	        -  	 13
  2	 P 	    93.05	   621.35	 12
  3	 Q 	   157.08	   572.83	 11
  4	 G 	   185.59	   508.80	 10
  5	 L 	   242.13	   480.29	  9
  6	 G 	   270.64	   423.75	  8
  7	 A 	   306.16	   395.23	  7
  8	 F 	   379.70	   359.72	  6
  9	 T 	   430.22	   286.18	  5
 10	 P 	   478.75	   235.66	  4
 11	 V 	   528.28	   187.13	  3
 12	 V 	   577.81	   137.60	  2
 13	 R 	        -  	    88.06	  1  _
```

**32. IPI00395337 Isoform 1 of Pre-mRNA 3'-end-processing factor FIP1**

| Sequence | Charge | M+H+ | XCorr | DeltaCn |
| --- | --- | --- | --- | --- |
| K.GVDLDAPGSINGVPLLEVDLDSFEDK.P | 3 | 2715.947 | 5.138 | 0.223 |

+1 charge

```
		      B   	       Y
  1	 G 	    58.03	        -  	 26
  2	 V 	   157.10	  2657.32	 25
  3	 D 	   272.12	  2558.25	 24
  4	 L 	   385.21	  2443.22	 23
  5	 D 	   500.24	  2330.14	 22
  6	 A 	   571.27	  2215.11	 21
  7	 P 	   668.32	  2144.08	 20
  8	 G 	   725.35	  2047.02	 19
  9	 S 	   812.38	  1990.00	 18
 10	 I 	   925.46	  1902.97	 17
 11	 N 	  1039.51	  1789.89	 16
 12	 G 	  1096.53	  1675.84	 15
 13	 V 	  1195.60	  1618.82	 14
 14	 P 	  1292.65	  1519.75	 13
 15	 L 	  1405.73	  1422.70	 12
 16	 L 	  1518.82	  1309.62	 11
 17	 E 	  1647.86	  1196.53	 10
 18	 V 	  1746.93	  1067.49	  9
 19	 D 	  1861.95	   968.42	  8
 20	 L 	  1975.04	   853.39	  7
 21	 D 	  2090.07	   740.31	  6
 22	 S 	  2177.10	   625.28	  5
 23	 F 	  2324.17	   538.25	  4
 24	 E 	  2453.21	   391.18	  3
 25	 D 	  2568.24	   262.14	  2
 26	 K 	        -  	   147.11	  1  _


+2 charge
		      B   	       Y
  1	 G 	    29.52	        -  	 26
  2	 V 	    79.05	  1329.16	 25
  3	 D 	   136.57	  1279.63	 24
  4	 L 	   193.11	  1222.12	 23
  5	 D 	   250.62	  1165.57	 22
  6	 A 	   286.14	  1108.06	 21
  7	 P 	   334.67	  1072.54	 20
  8	 G 	   363.18	  1024.02	 19
  9	 S 	   406.69	   995.50	 18
 10	 I 	   463.23	   951.99	 17
 11	 N 	   520.26	   895.45	 16
 12	 G 	   548.77	   838.42	 15
 13	 V 	   598.30	   809.91	 14
 14	 P 	   646.83	   760.38	 13
 15	 L 	   703.37	   711.85	 12
 16	 L 	   759.91	   655.31	 11
 17	 E 	   824.43	   598.77	 10
 18	 V 	   873.97	   534.25	  9
 19	 D 	   931.48	   484.71	  8
 20	 L 	   988.02	   427.20	  7
 21	 D 	  1045.54	   370.66	  6
 22	 S 	  1089.05	   313.15	  5
 23	 F 	  1162.59	   269.63	  4
 24	 E 	  1227.11	   196.09	  3
 25	 D 	  1284.62	   131.57	  2
 26	 K 	        -  	    74.06	  1  _


+3 charge
		      B   	       Y
  1	 G 	    20.01	        -  	 26
  2	 V 	    53.04	   886.44	 25
  3	 D 	    91.38	   853.42	 24
  4	 L 	   129.07	   815.08	 23
  5	 D 	   167.42	   777.38	 22
  6	 A 	   191.10	   739.04	 21
  7	 P 	   223.45	   715.36	 20
  8	 G 	   242.45	   683.01	 19
  9	 S 	   271.46	   664.01	 18
 10	 I 	   309.16	   634.99	 17
 11	 N 	   347.17	   597.30	 16
 12	 G 	   366.18	   559.29	 15
 13	 V 	   399.20	   540.28	 14
 14	 P 	   431.55	   507.26	 13
 15	 L 	   469.25	   474.90	 12
 16	 L 	   506.94	   437.21	 11
 17	 E 	   549.96	   399.52	 10
 18	 V 	   582.98	   356.50	  9
 19	 D 	   621.32	   323.48	  8
 20	 L 	   659.02	   285.14	  7
 21	 D 	   697.36	   247.44	  6
 22	 S 	   726.37	   209.10	  5
 23	 F 	   775.39	   180.09	  4
 24	 E 	   818.41	   131.07	  3
 25	 D 	   856.75	    88.05	  2
 26	 K 	        -  	    49.71	  1  _
```

**33. IPI00072044 Isoform 1 of Ester hydrolase C11orf54**

| Sequence | Charge | M+H+ | XCorr | DeltaCn |
| --- | --- | --- | --- | --- |
| R.IAEVGGVPYLLPLVNQK.K | 2 | 1811.157 | 3.147 | 0.393 |

+1 charge

```
		      B   	       Y
  1	 I 	   114.09	        -  	 17
  2	 A 	   185.13	  1696.96	 16
  3	 E 	   314.17	  1625.93	 15
  4	 V 	   413.24	  1496.88	 14
  5	 G 	   470.26	  1397.82	 13
  6	 G 	   527.28	  1340.79	 12
  7	 V 	   626.35	  1283.77	 11
  8	 P 	   723.40	  1184.70	 10
  9	 Y 	   886.47	  1087.65	  9
 10	 L 	   999.55	   924.59	  8
 11	 L 	  1112.64	   811.50	  7
 12	 P 	  1209.69	   698.42	  6
 13	 L 	  1322.77	   601.37	  5
 14	 V 	  1421.84	   488.28	  4
 15	 N 	  1535.88	   389.21	  3
 16	 Q 	  1663.94	   275.17	  2
 17	 K 	        -  	   147.11	  1  _
+2 charge
		      B   	       Y
  1	 I 	    57.55	        -  	 17
  2	 A 	    93.07	   848.99	 16
  3	 E 	   157.59	   813.47	 15
  4	 V 	   207.12	   748.95	 14
  5	 G 	   235.63	   699.41	 13
  6	 G 	   264.14	   670.90	 12
  7	 V 	   313.68	   642.39	 11
  8	 P 	   362.21	   592.86	 10
  9	 Y 	   443.74	   544.33	  9
 10	 L 	   500.28	   462.80	  8
 11	 L 	   556.82	   406.26	  7
 12	 P 	   605.35	   349.71	  6
 13	 L 	   661.89	   301.19	  5
 14	 V 	   711.42	   244.64	  4
 15	 N 	   768.45	   195.11	  3
 16	 Q 	   832.47	   138.09	  2
 17	 K 	        -  	    74.06	  1  _
```

**34. IPI00045512 Hemicentin 1**

| Sequence | Charge | M+H+ | XCorr | DeltaCn |
| --- | --- | --- | --- | --- |
| K.LVSLPFGIATNQDLIR.L | 2 | 1758.055 | 4.01 | 0.525 |

+1 charge

```
		      B   	       Y
  1	 L 	   114.09	        -  	 16
  2	 V 	   213.16	  1643.91	 15
  3	 S 	   300.19	  1544.84	 14
  4	 L 	   413.28	  1457.81	 13
  5	 P 	   510.33	  1344.73	 12
  6	 F 	   657.40	  1247.67	 11
  7	 G 	   714.42	  1100.61	 10
  8	 I 	   827.50	  1043.58	  9
  9	 A 	   898.54	   930.50	  8
 10	 T 	   999.59	   859.46	  7
 11	 N 	  1113.63	   758.42	  6
 12	 Q 	  1241.69	   644.37	  5
 13	 D 	  1356.72	   516.31	  4
 14	 L 	  1469.80	   401.29	  3
 15	 I 	  1582.88	   288.20	  2
 16	 R 	        -  	   175.12	  1  _
+2 charge
		      B   	       Y
  1	 L 	    57.55	        -  	 16
  2	 V 	   107.08	   822.46	 15
  3	 S 	   150.60	   772.93	 14
  4	 L 	   207.14	   729.41	 13
  5	 P 	   255.67	   672.87	 12
  6	 F 	   329.20	   624.34	 11
  7	 G 	   357.71	   550.81	 10
  8	 I 	   414.25	   522.30	  9
  9	 A 	   449.77	   465.75	  8
 10	 T 	   500.30	   430.24	  7
 11	 N 	   557.32	   379.71	  6
 12	 Q 	   621.35	   322.69	  5
 13	 D 	   678.86	   258.66	  4
 14	 L 	   735.40	   201.15	  3
 15	 I 	   791.95	   144.61	  2
 16	 R 	        -  	    88.06	  1  _
```

**35. IPI00003590 Isoform 1 of Sulfhydryl oxidase 1**

| Sequence | Charge | M+H+ | XCorr | DeltaCn |
| --- | --- | --- | --- | --- |
| R.AAPGQEPPEHMAELQR.N | 2 | 1761.941 | 3.6 | 0.555 |

+1 charge

```
		      B   	       Y
  1	 A 	    72.04	        -  	 16
  2	 A 	   143.08	  1689.80	 15
  3	 P 	   240.13	  1618.76	 14
  4	 G 	   297.16	  1521.71	 13
  5	 Q 	   425.21	  1464.69	 12
  6	 E 	   554.26	  1336.63	 11
  7	 P 	   651.31	  1207.59	 10
  8	 P 	   748.36	  1110.54	  9
  9	 E 	   877.41	  1013.48	  8
 10	 H 	  1014.46	   884.44	  7
 11	 M 	  1145.50	   747.38	  6
 12	 A 	  1216.54	   616.34	  5
 13	 E 	  1345.58	   545.30	  4
 14	 L 	  1458.67	   416.26	  3
 15	 Q 	  1586.73	   303.18	  2
 16	 R 	        -  	   175.12	  1  _
+2 charge
		      B   	       Y
  1	 A 	    36.53	        -  	 16
  2	 A 	    72.04	   845.40	 15
  3	 P 	   120.57	   809.89	 14
  4	 G 	   149.08	   761.36	 13
  5	 Q 	   213.11	   732.85	 12
  6	 E 	   277.63	   668.82	 11
  7	 P 	   326.16	   604.30	 10
  8	 P 	   374.68	   555.77	  9
  9	 E 	   439.21	   507.25	  8
 10	 H 	   507.74	   442.72	  7
 11	 M 	   573.26	   374.19	  6
 12	 A 	   608.77	   308.67	  5
 13	 E 	   673.30	   273.16	  4
 14	 L 	   729.84	   208.63	  3
 15	 Q 	   793.87	   152.09	  2
 16	 R 	        -  	    88.06	  1  _
```

**36. IPI00291867 Complement factor I**

| Sequence | Charge | M+H+ | XCorr | DeltaCn |
| --- | --- | --- | --- | --- |
| K.LVDQDKTMFICK.S | 2 | 1498.763 | 3.353 | 0.513 |

+1 charge

```
		      B   	       Y
  1	 L 	   114.09	        -  	 12
  2	 V 	   213.16	  1384.79	 11
  3	 D 	   328.19	  1285.73	 10
  4	 Q 	   456.25	  1170.70	  9
  5	 D 	   571.27	  1042.64	  8
  6	 K 	   699.37	   927.61	  7
  7	 T 	   800.41	   799.52	  6
  8	 M 	   931.46	   698.47	  5
  9	 F 	  1078.52	   567.43	  4
 10	 I 	  1191.61	   420.36	  3
 11	 C 	  1351.77	   307.28	  2
 12	 K 	        -  	   147.11	  1  _
+2 charge
		      B   	       Y
  1	 L 	    57.55	        -  	 12
  2	 V 	   107.08	   692.90	 11
  3	 D 	   164.60	   643.37	 10
  4	 Q 	   228.63	   585.85	  9
  5	 D 	   286.14	   521.82	  8
  6	 K 	   350.19	   464.31	  7
  7	 T 	   400.71	   400.26	  6
  8	 M 	   466.23	   349.74	  5
  9	 F 	   539.77	   284.22	  4
 10	 I 	   596.31	   210.68	  3
 11	 C 	   676.39	   154.14	  2
 12	 K 	        -  	    74.06	  1  _
```

**37. IPI00009950 Vesicular integral-membrane protein VIP36**

| Sequence | Charge | M+H+ | XCorr | DeltaCn |
| --- | --- | --- | --- | --- |
| R.WTELAGCTADFR.N | 2 | 1427.537 | 3.056 | 0.328 |

+1 charge

```
		      B   	       Y
  1	 W 	   187.09	        -  	 12
  2	 T 	   288.13	  1240.70	 11
  3	 E 	   417.18	  1139.65	 10
  4	 L 	   530.26	  1010.61	  9
  5	 A 	   601.30	   897.52	  8
  6	 G 	   658.32	   826.49	  7
  7	 C 	   818.48	   769.46	  6
  8	 T 	   919.53	   609.30	  5
  9	 A 	   990.57	   508.25	  4
 10	 D 	  1105.60	   437.21	  3
 11	 F 	  1252.66	   322.19	  2
 12	 R 	        -  	   175.12	  1  _
+2 charge
		      B   	       Y
  1	 W 	    94.05	        -  	 12
  2	 T 	   144.57	   620.85	 11
  3	 E 	   209.09	   570.33	 10
  4	 L 	   265.63	   505.81	  9
  5	 A 	   301.15	   449.27	  8
  6	 G 	   329.66	   413.75	  7
  7	 C 	   409.75	   385.24	  6
  8	 T 	   460.27	   305.15	  5
  9	 A 	   495.79	   254.63	  4
 10	 D 	   553.30	   219.11	  3
 11	 F 	   626.84	   161.60	  2
 12	 R 	        -  	    88.06	  1  _
```

**38. IPI00387113 Ig kappa chain V-III region B6**

| Sequence | Charge | M+H+ | XCorr | DeltaCn |
| --- | --- | --- | --- | --- |
| - .ZIVLTZSPGTLSLSPGZR.A | 2 | 1884.616 | 4.083 | 0.638 |

+1 charge

```
		      B   	       Y
  1	 Z 	   129.56	        -  	 18
  2	 I 	   242.64	  1754.96	 17
  3	 V 	   341.71	  1641.88	 16
  4	 L 	   454.79	  1542.81	 15
  5	 T 	   555.84	  1429.73	 14
  6	 Z 	   684.39	  1328.68	 13
  7	 S 	   771.42	  1200.13	 12
  8	 P 	   868.48	  1113.10	 11
  9	 G 	   925.50	  1016.05	 10
 10	 T 	  1026.55	   959.02	  9
 11	 L 	  1139.63	   857.98	  8
 12	 S 	  1226.66	   744.89	  7
 13	 L 	  1339.75	   657.86	  6
 14	 S 	  1426.78	   544.78	  5
 15	 P 	  1523.83	   457.74	  4
 16	 G 	  1580.85	   360.69	  3
 17	 Z 	  1709.40	   303.67	  2
 18	 R 	        -  	   175.12	  1  _
+2 charge
		      B   	       Y
  1	 Z 	    65.28	        -  	 18
  2	 I 	   121.82	   877.99	 17
  3	 V 	   171.36	   821.44	 16
  4	 L 	   227.90	   771.91	 15
  5	 T 	   278.42	   715.37	 14
  6	 Z 	   342.70	   664.84	 13
  7	 S 	   386.22	   600.57	 12
  8	 P 	   434.74	   557.05	 11
  9	 G 	   463.25	   508.53	 10
 10	 T 	   513.78	   480.02	  9
 11	 L 	   570.32	   429.49	  8
 12	 S 	   613.83	   372.95	  7
 13	 L 	   670.38	   329.43	  6
 14	 S 	   713.89	   272.89	  5
 15	 P 	   762.42	   229.38	  4
 16	 G 	   790.93	   180.85	  3
 17	 Z 	   855.21	   152.34	  2
 18	 R 	        -  	    88.06	  1  _
```

**39. IPI00022620 Secreted Ly-6/uPAR-related protein 1**

| Sequence | Charge | M+H+ | XCorr | DeltaCn |
| --- | --- | --- | --- | --- |
| R.SCSSSCVATDPDSIGAAHLIFCCFR.D | 3 | 2820.036 | 3.751 | 0.34 |

+1 charge

```
		      B   	       Y
  1	 S 	    88.04	        -  	 25
  2	 C 	   248.20	  2731.71	 24
  3	 S 	   335.24	  2571.55	 23
  4	 S 	   422.27	  2484.51	 22
  5	 S 	   509.30	  2397.48	 21
  6	 C 	   669.47	  2310.45	 20
  7	 V 	   768.53	  2150.28	 19
  8	 A 	   839.57	  2051.22	 18
  9	 T 	   940.62	  1980.18	 17
 10	 D 	  1055.65	  1879.13	 16
 11	 P 	  1152.70	  1764.10	 15
 12	 D 	  1267.73	  1667.05	 14
 13	 S 	  1354.76	  1552.03	 13
 14	 I 	  1467.84	  1464.99	 12
 15	 G 	  1524.86	  1351.91	 11
 16	 A 	  1595.90	  1294.89	 10
 17	 A 	  1666.94	  1223.85	  9
 18	 H 	  1804.00	  1152.81	  8
 19	 L 	  1917.08	  1015.75	  7
 20	 I 	  2030.16	   902.67	  6
 21	 F 	  2177.23	   789.59	  5
 22	 C 	  2337.40	   642.52	  4
 23	 C 	  2497.56	   482.35	  3
 24	 F 	  2644.63	   322.19	  2
 25	 R 	        -  	   175.12	  1  _


+2 charge
		      B   	       Y
  1	 S 	    44.52	        -  	 25
  2	 C 	   124.61	  1366.36	 24
  3	 S 	   168.12	  1286.28	 23
  4	 S 	   211.64	  1242.76	 22
  5	 S 	   255.15	  1199.24	 21
  6	 C 	   335.24	  1155.73	 20
  7	 V 	   384.77	  1075.65	 19
  8	 A 	   420.29	  1026.11	 18
  9	 T 	   470.81	   990.59	 17
 10	 D 	   528.33	   940.07	 16
 11	 P 	   576.85	   882.56	 15
 12	 D 	   634.37	   834.03	 14
 13	 S 	   677.88	   776.52	 13
 14	 I 	   734.42	   733.00	 12
 15	 G 	   762.94	   676.46	 11
 16	 A 	   798.45	   647.95	 10
 17	 A 	   833.97	   612.43	  9
 18	 H 	   902.50	   576.91	  8
 19	 L 	   959.04	   508.38	  7
 20	 I 	  1015.59	   451.84	  6
 21	 F 	  1089.12	   395.30	  5
 22	 C 	  1169.20	   321.76	  4
 23	 C 	  1249.29	   241.68	  3
 24	 F 	  1322.82	   161.60	  2
 25	 R 	        -  	    88.06	  1  _


+3 charge
		      B   	       Y
  1	 S 	    30.02	        -  	 25
  2	 C 	    83.41	   911.24	 24
  3	 S 	   112.42	   857.85	 23
  4	 S 	   141.43	   828.84	 22
  5	 S 	   170.44	   799.83	 21
  6	 C 	   223.83	   770.82	 20
  7	 V 	   256.85	   717.43	 19
  8	 A 	   280.53	   684.41	 18
  9	 T 	   314.21	   660.73	 17
 10	 D 	   352.55	   627.05	 16
 11	 P 	   384.90	   588.71	 15
 12	 D 	   423.25	   556.36	 14
 13	 S 	   452.26	   518.01	 13
 14	 I 	   489.95	   489.00	 12
 15	 G 	   508.96	   451.31	 11
 16	 A 	   532.64	   432.30	 10
 17	 A 	   556.32	   408.62	  9
 18	 H 	   602.00	   384.94	  8
 19	 L 	   639.70	   339.26	  7
 20	 I 	   677.39	   301.56	  6
 21	 F 	   726.42	   263.87	  5
 22	 C 	   779.80	   214.84	  4
 23	 C 	   833.19	   161.46	  3
 24	 F 	   882.22	   108.07	  2
 25	 R 	        -  	    59.04	  1  _
```

**40. IPI00030786 Isoform 1 of Matrilin-4**

| Sequence | Charge | M+H+ | XCorr | DeltaCn |
| --- | --- | --- | --- | --- |
| R.TLGALESLTLNLAQLTAR.L | 2 | 1886.183 | 3.557 | 0.579 |

+1 charge

```
		      B   	       Y
  1	 T 	   102.05	        -  	 18
  2	 L 	   215.14	  1784.03	 17
  3	 G 	   272.16	  1670.94	 16
  4	 A 	   343.20	  1613.92	 15
  5	 L 	   456.28	  1542.88	 14
  6	 E 	   585.32	  1429.80	 13
  7	 S 	   672.36	  1300.76	 12
  8	 L 	   785.44	  1213.73	 11
  9	 T 	   886.49	  1100.64	 10
 10	 L 	   999.57	   999.59	  9
 11	 N 	  1113.62	   886.51	  8
 12	 L 	  1226.70	   772.47	  7
 13	 A 	  1297.74	   659.38	  6
 14	 Q 	  1425.79	   588.35	  5
 15	 L 	  1538.88	   460.29	  4
 16	 T 	  1639.93	   347.20	  3
 17	 A 	  1710.96	   246.16	  2
 18	 R 	        -  	   175.12	  1  _
+2 charge
		      B   	       Y
  1	 T 	    51.53	        -  	 18
  2	 L 	   108.07	   892.52	 17
  3	 G 	   136.58	   835.98	 16
  4	 A 	   172.10	   807.46	 15
  5	 L 	   228.64	   771.95	 14
  6	 E 	   293.17	   715.40	 13
  7	 S 	   336.68	   650.88	 12
  8	 L 	   393.22	   607.37	 11
  9	 T 	   443.75	   550.82	 10
 10	 L 	   500.29	   500.30	  9
 11	 N 	   557.31	   443.76	  8
 12	 L 	   613.85	   386.74	  7
 13	 A 	   649.37	   330.20	  6
 14	 Q 	   713.40	   294.68	  5
 15	 L 	   769.94	   230.65	  4
 16	 T 	   820.47	   174.11	  3
 17	 A 	   855.99	   123.58	  2
 18	 R 	        -  	    88.06	  1  _
```

**41. IPI00293887 Isoform 2 of StAR-related lipid transfer protein 8**

| Sequence | Charge | M+H+ | XCorr | DeltaCn |
| --- | --- | --- | --- | --- |
| K.YTVPHKQGWVWSMPKFMR.R | 2 | 2279.717 | 2.81 | 0.269 |

+1 charge

```
		      B   	       Y
  1	 Y 	   164.07	        -  	 18
  2	 T 	   265.12	  2115.08	 17
  3	 V 	   364.19	  2014.03	 16
  4	 P 	   461.24	  1914.96	 15
  5	 H 	   598.30	  1817.91	 14
  6	 K 	   726.39	  1680.85	 13
  7	 Q 	   854.45	  1552.76	 12
  8	 G 	   911.47	  1424.70	 11
  9	 W 	  1097.55	  1367.68	 10
 10	 V 	  1196.62	  1181.60	  9
 11	 W 	  1382.70	  1082.53	  8
 12	 S 	  1469.73	   896.45	  7
 13	 M 	  1600.77	   809.42	  6
 14	 P 	  1697.83	   678.38	  5
 15	 K 	  1825.92	   581.32	  4
 16	 F 	  1972.99	   453.23	  3
 17	 M 	  2104.03	   306.16	  2
 18	 R 	        -  	   175.12	  1  _
+2 charge
		      B   	       Y
  1	 Y 	    82.54	        -  	 18
  2	 T 	   133.06	  1058.04	 17
  3	 V 	   182.60	  1007.52	 16
  4	 P 	   231.12	   957.98	 15
  5	 H 	   299.65	   909.46	 14
  6	 K 	   363.70	   840.93	 13
  7	 Q 	   427.73	   776.88	 12
  8	 G 	   456.24	   712.85	 11
  9	 W 	   549.28	   684.34	 10
 10	 V 	   598.81	   591.30	  9
 11	 W 	   691.85	   541.77	  8
 12	 S 	   735.37	   448.73	  7
 13	 M 	   800.89	   405.21	  6
 14	 P 	   849.42	   339.69	  5
 15	 K 	   913.46	   291.17	  4
 16	 F 	   987.00	   227.12	  3
 17	 M 	  1052.52	   153.58	  2
 18	 R 	        -  	    88.06	  1  _
```

**42. IPI00011564 Syndecan-4**

| Sequence | Charge | M+H+ | XCorr | DeltaCn |
| --- | --- | --- | --- | --- |
| K.KLEENEVIPK.R | 2 | 1199.379 | 3.053 | 0.27 |

+1 charge

```
		      B   	       Y
  1	 K 	   129.10	        -  	 10
  2	 L 	   242.19	  1070.57	  9
  3	 E 	   371.23	   957.49	  8
  4	 E 	   500.27	   828.45	  7
  5	 N 	   614.31	   699.40	  6
  6	 E 	   743.36	   585.36	  5
  7	 V 	   842.43	   456.32	  4
  8	 I 	   955.51	   357.25	  3
  9	 P 	  1052.56	   244.17	  2
 10	 K 	        -  	   147.11	  1  _
+2 charge
		      B   	       Y
  1	 K 	    65.05	        -  	 10
  2	 L 	   121.60	   535.79	  9
  3	 E 	   186.12	   479.25	  8
  4	 E 	   250.64	   414.73	  7
  5	 N 	   307.66	   350.21	  6
  6	 E 	   372.18	   293.18	  5
  7	 V 	   421.72	   228.66	  4
  8	 I 	   478.26	   179.13	  3
  9	 P 	   526.78	   122.59	  2
 10	 K 	        -  	    74.06	  1  _
```

**43. IPI00783987 Complement C3 (Fragment)**

| Sequence | Charge | M+H+ | XCorr | DeltaCn |
| --- | --- | --- | --- | --- |
| R.EGVQKEDIPPADLSDQVPDTESETR.I | 3 | 2756.871 | 3.604 | 0.356 |

+1 charge

```
		      B   	       Y
  1	 E 	   130.05	        -  	 25
  2	 G 	   187.07	  2626.25	 24
  3	 V 	   286.14	  2569.23	 23
  4	 Q 	   414.20	  2470.16	 22
  5	 K 	   542.29	  2342.10	 21
  6	 E 	   671.34	  2214.00	 20
  7	 D 	   786.36	  2084.96	 19
  8	 I 	   899.45	  1969.93	 18
  9	 P 	   996.50	  1856.85	 17
 10	 P 	  1093.55	  1759.80	 16
 11	 A 	  1164.59	  1662.75	 15
 12	 D 	  1279.62	  1591.71	 14
 13	 L 	  1392.70	  1476.68	 13
 14	 S 	  1479.73	  1363.60	 12
 15	 D 	  1594.76	  1276.57	 11
 16	 Q 	  1722.82	  1161.54	 10
 17	 V 	  1821.89	  1033.48	  9
 18	 P 	  1918.94	   934.41	  8
 19	 D 	  2033.97	   837.36	  7
 20	 T 	  2135.01	   722.33	  6
 21	 E 	  2264.06	   621.28	  5
 22	 S 	  2351.09	   492.24	  4
 23	 E 	  2480.13	   405.21	  3
 24	 T 	  2581.18	   276.17	  2
 25	 R 	        -  	   175.12	  1  _


+2 charge
		      B   	       Y
  1	 E 	    65.53	        -  	 25
  2	 G 	    94.04	  1313.63	 24
  3	 V 	   143.57	  1285.12	 23
  4	 Q 	   207.60	  1235.58	 22
  5	 K 	   271.65	  1171.55	 21
  6	 E 	   336.17	  1107.51	 20
  7	 D 	   393.69	  1042.98	 19
  8	 I 	   450.23	   985.47	 18
  9	 P 	   498.75	   928.93	 17
 10	 P 	   547.28	   880.40	 16
 11	 A 	   582.80	   831.88	 15
 12	 D 	   640.31	   796.36	 14
 13	 L 	   696.85	   738.84	 13
 14	 S 	   740.37	   682.30	 12
 15	 D 	   797.88	   638.79	 11
 16	 Q 	   861.91	   581.27	 10
 17	 V 	   911.45	   517.24	  9
 18	 P 	   959.97	   467.71	  8
 19	 D 	  1017.49	   419.18	  7
 20	 T 	  1068.01	   361.67	  6
 21	 E 	  1132.53	   311.15	  5
 22	 S 	  1176.05	   246.62	  4
 23	 E 	  1240.57	   203.11	  3
 24	 T 	  1291.09	   138.59	  2
 25	 R 	        -  	    88.06	  1  _


+3 charge
		      B   	       Y
  1	 E 	    44.02	        -  	 25
  2	 G 	    63.03	   876.09	 24
  3	 V 	    96.05	   857.08	 23
  4	 Q 	   138.74	   824.06	 22
  5	 K 	   181.44	   781.37	 21
  6	 E 	   224.45	   738.67	 20
  7	 D 	   262.79	   695.66	 19
  8	 I 	   300.49	   657.32	 18
  9	 P 	   332.84	   619.62	 17
 10	 P 	   365.19	   587.27	 16
 11	 A 	   388.87	   554.92	 15
 12	 D 	   427.21	   531.24	 14
 13	 L 	   464.91	   492.90	 13
 14	 S 	   493.92	   455.20	 12
 15	 D 	   532.26	   426.19	 11
 16	 Q 	   574.94	   387.85	 10
 17	 V 	   607.97	   345.16	  9
 18	 P 	   640.32	   312.14	  8
 19	 D 	   678.66	   279.79	  7
 20	 T 	   712.34	   241.45	  6
 21	 E 	   755.36	   207.77	  5
 22	 S 	   784.37	   164.75	  4
 23	 E 	   827.38	   135.74	  3
 24	 T 	   861.06	    92.73	  2
 25	 R 	        -  	    59.04	  1  _
```

**44. IPI00382440 Ig lambda chain V-IV region Hil**

| Sequence | Charge | M+H+ | XCorr | DeltaCn |
| --- | --- | --- | --- | --- |
| - .SYELTQPPSVSVSPGQTAR.I | 2 | 2005.175 | 3.532 | 0.461 |

+1 charge

```
		      B   	       Y
  1	 S 	    88.04	        -  	 19
  2	 Y 	   251.10	  1916.97	 18
  3	 E 	   380.15	  1753.91	 17
  4	 L 	   493.23	  1624.87	 16
  5	 T 	   594.28	  1511.78	 15
  6	 Q 	   722.34	  1410.73	 14
  7	 P 	   819.39	  1282.67	 13
  8	 P 	   916.44	  1185.62	 12
  9	 S 	  1003.47	  1088.57	 11
 10	 V 	  1102.54	  1001.54	 10
 11	 S 	  1189.57	   902.47	  9
 12	 V 	  1288.64	   815.44	  8
 13	 S 	  1375.67	   716.37	  7
 14	 P 	  1472.73	   629.34	  6
 15	 G 	  1529.75	   532.28	  5
 16	 Q 	  1657.81	   475.26	  4
 17	 T 	  1758.85	   347.20	  3
 18	 A 	  1829.89	   246.16	  2
 19	 R 	        -  	   175.12	  1  _
+2 charge
		      B   	       Y
  1	 S 	    44.52	        -  	 19
  2	 Y 	   126.05	   958.99	 18
  3	 E 	   190.58	   877.46	 17
  4	 L 	   247.12	   812.94	 16
  5	 T 	   297.64	   756.39	 15
  6	 Q 	   361.67	   705.87	 14
  7	 P 	   410.20	   641.84	 13
  8	 P 	   458.72	   593.31	 12
  9	 S 	   502.24	   544.79	 11
 10	 V 	   551.77	   501.27	 10
 11	 S 	   595.29	   451.74	  9
 12	 V 	   644.82	   408.22	  8
 13	 S 	   688.34	   358.69	  7
 14	 P 	   736.87	   315.17	  6
 15	 G 	   765.38	   266.65	  5
 16	 Q 	   829.41	   238.13	  4
 17	 T 	   879.93	   174.11	  3
 18	 A 	   915.45	   123.58	  2
 19	 R 	        -  	    88.06	  1  _
```

**45. IPI00292150 Latent-transforming growth factor beta-binding protein 2**

| Sequence | Charge | M+H+ | XCorr | DeltaCn |
| --- | --- | --- | --- | --- |
| R.SLGPGTCTLPLAQR.I | 2 | 1471.678 | 3.573 | 0.454 |

+1 charge

```
		      B   	       Y
  1	 S 	    88.04	        -  	 14
  2	 L 	   201.12	  1383.88	 13
  3	 G 	   258.14	  1270.79	 12
  4	 P 	   355.20	  1213.77	 11
  5	 G 	   412.22	  1116.72	 10
  6	 T 	   513.27	  1059.70	  9
  7	 C 	   673.43	   958.65	  8
  8	 T 	   774.48	   798.48	  7
  9	 L 	   887.56	   697.44	  6
 10	 P 	   984.62	   584.35	  5
 11	 L 	  1097.70	   487.30	  4
 12	 A 	  1168.74	   374.21	  3
 13	 Q 	  1296.80	   303.18	  2
 14	 R 	        -  	   175.12	  1  _
+2 charge
		      B   	       Y
  1	 S 	    44.52	        -  	 14
  2	 L 	   101.07	   692.44	 13
  3	 G 	   129.58	   635.90	 12
  4	 P 	   178.10	   607.39	 11
  5	 G 	   206.61	   558.86	 10
  6	 T 	   257.14	   530.35	  9
  7	 C 	   337.22	   479.83	  8
  8	 T 	   387.74	   399.75	  7
  9	 L 	   444.29	   349.22	  6
 10	 P 	   492.81	   292.68	  5
 11	 L 	   549.35	   244.15	  4
 12	 A 	   584.87	   187.61	  3
 13	 Q 	   648.90	   152.09	  2
 14	 R 	        -  	    88.06	  1  _
```

**46. IPI00019954 Cystatin-M**

| Sequence | Charge | M+H+ | XCorr | DeltaCn |
| --- | --- | --- | --- | --- |
| R.VTGDHVDLTTCPLAAGAQQEKLR.C | 3 | 2481.741 | 5.493 | 0.515 |

+1 charge

```
		      B   	       Y
  1	 V 	   100.08	        -  	 23
  2	 T 	   201.12	  2381.32	 22
  3	 G 	   258.14	  2280.27	 21
  4	 D 	   373.17	  2223.25	 20
  5	 H 	   510.23	  2108.23	 19
  6	 V 	   609.30	  1971.17	 18
  7	 D 	   724.33	  1872.10	 17
  8	 L 	   837.41	  1757.07	 16
  9	 T 	   938.46	  1643.99	 15
 10	 T 	  1039.51	  1542.94	 14
 11	 C 	  1199.67	  1441.89	 13
 12	 P 	  1296.72	  1281.73	 12
 13	 L 	  1409.81	  1184.67	 11
 14	 A 	  1480.84	  1071.59	 10
 15	 A 	  1551.88	  1000.55	  9
 16	 G 	  1608.90	   929.52	  8
 17	 A 	  1679.94	   872.49	  7
 18	 Q 	  1808.00	   801.46	  6
 19	 Q 	  1936.06	   673.40	  5
 20	 E 	  2065.10	   545.34	  4
 21	 K 	  2193.20	   416.30	  3
 22	 L 	  2306.28	   288.20	  2
 23	 R 	        -  	   175.12	  1  _


+2 charge
		      B   	       Y
  1	 V 	    50.54	        -  	 23
  2	 T 	   101.07	  1191.16	 22
  3	 G 	   129.58	  1140.64	 21
  4	 D 	   187.09	  1112.13	 20
  5	 H 	   255.62	  1054.62	 19
  6	 V 	   305.15	   986.09	 18
  7	 D 	   362.67	   936.55	 17
  8	 L 	   419.21	   879.04	 16
  9	 T 	   469.73	   822.50	 15
 10	 T 	   520.26	   771.97	 14
 11	 C 	   600.34	   721.45	 13
 12	 P 	   648.87	   641.37	 12
 13	 L 	   705.41	   592.84	 11
 14	 A 	   740.93	   536.30	 10
 15	 A 	   776.44	   500.78	  9
 16	 G 	   804.96	   465.26	  8
 17	 A 	   840.47	   436.75	  7
 18	 Q 	   904.50	   401.23	  6
 19	 Q 	   968.53	   337.20	  5
 20	 E 	  1033.05	   273.17	  4
 21	 K 	  1097.10	   208.65	  3
 22	 L 	  1153.64	   144.61	  2
 23	 R 	        -  	    88.06	  1  _


+3 charge
		      B   	       Y
  1	 V 	    34.03	        -  	 23
  2	 T 	    67.71	   794.45	 22
  3	 G 	    86.72	   760.76	 21
  4	 D 	   125.06	   741.76	 20
  5	 H 	   170.75	   703.41	 19
  6	 V 	   203.77	   657.73	 18
  7	 D 	   242.11	   624.70	 17
  8	 L 	   279.81	   586.36	 16
  9	 T 	   313.49	   548.67	 15
 10	 T 	   347.17	   514.98	 14
 11	 C 	   400.56	   481.30	 13
 12	 P 	   432.91	   427.91	 12
 13	 L 	   470.61	   395.56	 11
 14	 A 	   494.29	   357.87	 10
 15	 A 	   517.97	   334.19	  9
 16	 G 	   536.97	   310.51	  8
 17	 A 	   560.65	   291.50	  7
 18	 Q 	   603.34	   267.82	  6
 19	 Q 	   646.02	   225.14	  5
 20	 E 	   689.04	   182.45	  4
 21	 K 	   731.74	   139.44	  3
 22	 L 	   769.43	    96.74	  2
 23	 R 	        -  	    59.04	  1  _
```

**47. IPI00219827 Secreted Ly-6/uPAR-related protein 2**

| Sequence | Charge | M+H+ | XCorr | DeltaCn |
| --- | --- | --- | --- | --- |
| R.VLSNTEDLPLVTK.M | 2 | 1429.641 | 4.578 | 0.571 |

+1 charge

```
		      B   	       Y
  1	 V 	   100.08	        -  	 13
  2	 L 	   213.16	  1329.73	 12
  3	 S 	   300.19	  1216.64	 11
  4	 N 	   414.23	  1129.61	 10
  5	 T 	   515.28	  1015.57	  9
  6	 E 	   644.32	   914.52	  8
  7	 D 	   759.35	   785.48	  7
  8	 L 	   872.44	   670.45	  6
  9	 P 	   969.49	   557.37	  5
 10	 L 	  1082.57	   460.31	  4
 11	 V 	  1181.64	   347.23	  3
 12	 T 	  1282.69	   248.16	  2
 13	 K 	        -  	   147.11	  1  _
+2 charge
		      B   	       Y
  1	 V 	    50.54	        -  	 13
  2	 L 	   107.08	   665.37	 12
  3	 S 	   150.60	   608.82	 11
  4	 N 	   207.62	   565.31	 10
  5	 T 	   258.14	   508.29	  9
  6	 E 	   322.67	   457.76	  8
  7	 D 	   380.18	   393.24	  7
  8	 L 	   436.72	   335.73	  6
  9	 P 	   485.25	   279.19	  5
 10	 L 	   541.79	   230.66	  4
 11	 V 	   591.32	   174.12	  3
 12	 T 	   641.85	   124.58	  2
 13	 K 	        -  	    74.06	  1  _
```

**48. IPI00015881 Isoform 1 of Macrophage colony-stimulating factor 1**

| Sequence | Charge | M+H+ | XCorr | DeltaCn |
| --- | --- | --- | --- | --- |
| R.SHSSGSVLPLGELEGR.R | 2 | 1625.765 | 4.307 | 0.563 |

+1 charge

```
		      B   	       Y
  1	 S 	    88.04	        -  	 16
  2	 H 	   225.10	  1537.80	 15
  3	 S 	   312.13	  1400.74	 14
  4	 S 	   399.16	  1313.71	 13
  5	 G 	   456.18	  1226.67	 12
  6	 S 	   543.22	  1169.65	 11
  7	 V 	   642.28	  1082.62	 10
  8	 L 	   755.37	   983.55	  9
  9	 P 	   852.42	   870.47	  8
 10	 L 	   965.51	   773.42	  7
 11	 G 	  1022.53	   660.33	  6
 12	 E 	  1151.57	   603.31	  5
 13	 L 	  1264.65	   474.27	  4
 14	 E 	  1393.70	   361.18	  3
 15	 G 	  1450.72	   232.14	  2
 16	 R 	        -  	   175.12	  1  _
+2 charge
		      B   	       Y
  1	 S 	    44.52	        -  	 16
  2	 H 	   113.05	   769.40	 15
  3	 S 	   156.57	   700.87	 14
  4	 S 	   200.08	   657.36	 13
  5	 G 	   228.60	   613.84	 12
  6	 S 	   272.11	   585.33	 11
  7	 V 	   321.65	   541.81	 10
  8	 L 	   378.19	   492.28	  9
  9	 P 	   426.71	   435.74	  8
 10	 L 	   483.26	   387.21	  7
 11	 G 	   511.77	   330.67	  6
 12	 E 	   576.29	   302.16	  5
 13	 L 	   632.83	   237.64	  4
 14	 E 	   697.35	   181.10	  3
 15	 G 	   725.86	   116.57	  2
 16	 R 	        -  	    88.06	  1  _
```

**49．IPI00024046 cDNA FLJ52398, highly similar to Cadherin-13**

| Sequence | Charge | M+H+ | XCorr | DeltaCn |
| --- | --- | --- | --- | --- |
| R.MTAFDADDPATDNALLR.Y | 2 | 1837.989 | 4.643 | 0.517 |

+1 charge

```
		      B   	       Y
  1	 M 	   132.05	        -  	 17
  2	 T 	   233.10	  1705.80	 16
  3	 A 	   304.13	  1604.76	 15
  4	 F 	   451.20	  1533.72	 14
  5	 D 	   566.23	  1386.65	 13
  6	 A 	   637.27	  1271.62	 12
  7	 D 	   752.29	  1200.59	 11
  8	 D 	   867.32	  1085.56	 10
  9	 P 	   964.37	   970.53	  9
 10	 A 	  1035.41	   873.48	  8
 11	 T 	  1136.46	   802.44	  7
 12	 D 	  1251.48	   701.39	  6
 13	 N 	  1365.53	   586.37	  5
 14	 A 	  1436.56	   472.32	  4
 15	 L 	  1549.65	   401.29	  3
 16	 L 	  1662.73	   288.20	  2
 17	 R 	        -  	   175.12	  1  _
+2 charge
		      B   	       Y
  1	 M 	    66.53	        -  	 17
  2	 T 	   117.05	   853.41	 16
  3	 A 	   152.57	   802.88	 15
  4	 F 	   226.10	   767.36	 14
  5	 D 	   283.62	   693.83	 13
  6	 A 	   319.14	   636.31	 12
  7	 D 	   376.65	   600.80	 11
  8	 D 	   434.16	   543.28	 10
  9	 P 	   482.69	   485.77	  9
 10	 A 	   518.21	   437.24	  8
 11	 T 	   568.73	   401.72	  7
 12	 D 	   626.25	   351.20	  6
 13	 N 	   683.27	   293.69	  5
 14	 A 	   718.79	   236.67	  4
 15	 L 	   775.33	   201.15	  3
 16	 L 	   831.87	   144.61	  2
 17	 R 	        -  	    88.06	  1  _
```

**50. IPI00880178 Isoform 3 of UPF0510 protein INM02**

| Sequence | Charge | M+H+ | XCorr | DeltaCn |
| --- | --- | --- | --- | --- |
| R.GSLLWNQQDGTLSLSQR.Q | 2 | 1904.074 | 3.019 | 0.539 |

+1 charge

```
		      B   	       Y
  1	 G 	    58.03	        -  	 17
  2	 S 	   145.06	  1845.95	 16
  3	 L 	   258.14	  1758.91	 15
  4	 L 	   371.23	  1645.83	 14
  5	 W 	   557.31	  1532.75	 13
  6	 N 	   671.35	  1346.67	 12
  7	 Q 	   799.41	  1232.62	 11
  8	 Q 	   927.47	  1104.56	 10
  9	 D 	  1042.50	   976.51	  9
 10	 G 	  1099.52	   861.48	  8
 11	 T 	  1200.56	   804.46	  7
 12	 L 	  1313.65	   703.41	  6
 13	 S 	  1400.68	   590.33	  5
 14	 L 	  1513.76	   503.29	  4
 15	 S 	  1600.80	   390.21	  3
 16	 Q 	  1728.86	   303.18	  2
 17	 R 	        -  	   175.12	  1  _
+2 charge
		      B   	       Y
  1	 G 	    29.52	        -  	 17
  2	 S 	    73.03	   923.48	 16
  3	 L 	   129.58	   879.96	 15
  4	 L 	   186.12	   823.42	 14
  5	 W 	   279.16	   766.88	 13
  6	 N 	   336.18	   673.84	 12
  7	 Q 	   400.21	   616.82	 11
  8	 Q 	   464.24	   552.79	 10
  9	 D 	   521.75	   488.76	  9
 10	 G 	   550.26	   431.24	  8
 11	 T 	   600.79	   402.73	  7
 12	 L 	   657.33	   352.21	  6
 13	 S 	   700.84	   295.67	  5
 14	 L 	   757.39	   252.15	  4
 15	 S 	   800.90	   195.61	  3
 16	 Q 	   864.93	   152.09	  2
 17	 R 	        -  	    88.06	  1  _
```

**51. IPI00003091 Isoform 1 of Protein phosphatase 1 regulatory subunit 3A**

| Sequence | Charge | M+H+ | XCorr | DeltaCn |
| --- | --- | --- | --- | --- |
| K.EQEPEPVKPWKEVPNR.Q | 2 | 1963.183 | 3.166 | 0.214 |

+1 charge

```
		      B   	       Y
  1	 E 	   130.05	        -  	 16
  2	 Q 	   258.11	  1832.97	 15
  3	 E 	   387.15	  1704.91	 14
  4	 P 	   484.20	  1575.86	 13
  5	 E 	   613.25	  1478.81	 12
  6	 P 	   710.30	  1349.77	 11
  7	 V 	   809.37	  1252.72	 10
  8	 K 	   937.46	  1153.65	  9
  9	 P 	  1034.52	  1025.55	  8
 10	 W 	  1220.59	   928.50	  7
 11	 K 	  1348.69	   742.42	  6
 12	 E 	  1477.73	   614.33	  5
 13	 V 	  1576.80	   485.28	  4
 14	 P 	  1673.85	   386.21	  3
 15	 N 	  1787.90	   289.16	  2
 16	 R 	        -  	   175.12	  1  _
+2 charge
		      B   	       Y
  1	 E 	    65.53	        -  	 16
  2	 Q 	   129.56	   916.99	 15
  3	 E 	   194.08	   852.96	 14
  4	 P 	   242.61	   788.44	 13
  5	 E 	   307.13	   739.91	 12
  6	 P 	   355.65	   675.39	 11
  7	 V 	   405.19	   626.86	 10
  8	 K 	   469.23	   577.33	  9
  9	 P 	   517.76	   513.28	  8
 10	 W 	   610.80	   464.75	  7
 11	 K 	   674.85	   371.71	  6
 12	 E 	   739.37	   307.67	  5
 13	 V 	   788.90	   243.15	  4
 14	 P 	   837.43	   193.61	  3
 15	 N 	   894.45	   145.08	  2
 16	 R 	        -  	    88.06	  1  _
```

**52. IPI00176222 Isoform 1 of Zinc finger protein 549**

| Sequence | Charge | M+H+ | XCorr | DeltaCn |
| --- | --- | --- | --- | --- |
| K.WFSFGSNLQQHQNQDSGEKHIRK.E | 3 | 2772.975 | 3.823 | 0.237 |

+1 charge

```
		      B   	       Y
  1	 W 	   187.09	        -  	 23
  2	 F 	   334.16	  2585.26	 22
  3	 S 	   421.19	  2438.19	 21
  4	 F 	   568.26	  2351.16	 20
  5	 G 	   625.28	  2204.09	 19
  6	 S 	   712.31	  2147.07	 18
  7	 N 	   826.35	  2060.04	 17
  8	 L 	   939.44	  1946.00	 16
  9	 Q 	  1067.49	  1832.91	 15
 10	 Q 	  1195.55	  1704.85	 14
 11	 H 	  1332.61	  1576.79	 13
 12	 Q 	  1460.67	  1439.73	 12
 13	 N 	  1574.71	  1311.68	 11
 14	 Q 	  1702.77	  1197.63	 10
 15	 D 	  1817.80	  1069.57	  9
 16	 S 	  1904.83	   954.55	  8
 17	 G 	  1961.85	   867.52	  7
 18	 E 	  2090.90	   810.49	  6
 19	 K 	  2218.99	   681.45	  5
 20	 H 	  2356.05	   553.36	  4
 21	 I 	  2469.13	   416.30	  3
 22	 R 	  2625.23	   303.21	  2
 23	 K 	        -  	   147.11	  1  _


+2 charge
		      B   	       Y
  1	 W 	    94.05	        -  	 23
  2	 F 	   167.58	  1293.13	 22
  3	 S 	   211.10	  1219.60	 21
  4	 F 	   284.63	  1176.08	 20
  5	 G 	   313.14	  1102.55	 19
  6	 S 	   356.66	  1074.04	 18
  7	 N 	   413.68	  1030.52	 17
  8	 L 	   470.22	   973.50	 16
  9	 Q 	   534.25	   916.96	 15
 10	 Q 	   598.28	   852.93	 14
 11	 H 	   666.81	   788.90	 13
 12	 Q 	   730.84	   720.37	 12
 13	 N 	   787.86	   656.34	 11
 14	 Q 	   851.89	   599.32	 10
 15	 D 	   909.40	   535.29	  9
 16	 S 	   952.92	   477.78	  8
 17	 G 	   981.43	   434.26	  7
 18	 E 	  1045.95	   405.75	  6
 19	 K 	  1110.00	   341.23	  5
 20	 H 	  1178.53	   277.18	  4
 21	 I 	  1235.07	   208.65	  3
 22	 R 	  1313.12	   152.11	  2
 23	 K 	        -  	    74.06	  1  _


+3 charge
		      B   	       Y
  1	 W 	    63.03	        -  	 23
  2	 F 	   112.06	   862.42	 22
  3	 S 	   141.07	   813.40	 21
  4	 F 	   190.09	   784.39	 20
  5	 G 	   209.10	   735.37	 19
  6	 S 	   238.11	   716.36	 18
  7	 N 	   276.12	   687.35	 17
  8	 L 	   313.82	   649.34	 16
  9	 Q 	   356.50	   611.64	 15
 10	 Q 	   399.19	   568.96	 14
 11	 H 	   444.88	   526.27	 13
 12	 Q 	   487.56	   480.58	 12
 13	 N 	   525.58	   437.90	 11
 14	 Q 	   568.26	   399.88	 10
 15	 D 	   606.60	   357.20	  9
 16	 S 	   635.62	   318.85	  8
 17	 G 	   654.62	   289.84	  7
 18	 E 	   697.64	   270.84	  6
 19	 K 	   740.33	   227.82	  5
 20	 H 	   786.02	   185.12	  4
 21	 I 	   823.72	   139.44	  3
 22	 R 	   875.75	   101.74	  2
 23	 K 	        -  	    49.71	  1  _
```

**53. IPI00000828 Proenkephalin-A**

| Sequence | Charge | M+H+ | XCorr | DeltaCn |
| --- | --- | --- | --- | --- |
| K.DAEEDDSLANSSDLLKELLETGDNRER.S | 3 | 3036.123 | 4.833 | 0.549 |

+1 charge

```
		      B   	       Y
  1	 D 	   116.03	        -  	 27
  2	 A 	   187.07	  2919.38	 26
  3	 E 	   316.11	  2848.34	 25
  4	 E 	   445.16	  2719.30	 24
  5	 D 	   560.18	  2590.26	 23
  6	 D 	   675.21	  2475.23	 22
  7	 S 	   762.24	  2360.21	 21
  8	 L 	   875.33	  2273.17	 20
  9	 A 	   946.36	  2160.09	 19
 10	 N 	  1060.41	  2089.05	 18
 11	 S 	  1147.44	  1975.01	 17
 12	 S 	  1234.47	  1887.98	 16
 13	 D 	  1349.50	  1800.95	 15
 14	 L 	  1462.58	  1685.92	 14
 15	 L 	  1575.67	  1572.83	 13
 16	 K 	  1703.76	  1459.75	 12
 17	 E 	  1832.80	  1331.65	 11
 18	 L 	  1945.89	  1202.61	 10
 19	 L 	  2058.97	  1089.53	  9
 20	 E 	  2188.01	   976.44	  8
 21	 T 	  2289.06	   847.40	  7
 22	 G 	  2346.08	   746.35	  6
 23	 D 	  2461.11	   689.33	  5
 24	 N 	  2575.15	   574.31	  4
 25	 R 	  2731.25	   460.26	  3
 26	 E 	  2860.30	   304.16	  2
 27	 R 	        -  	   175.12	  1  _


+2 charge
		      B   	       Y
  1	 D 	    58.52	        -  	 27
  2	 A 	    94.04	  1460.19	 26
  3	 E 	   158.56	  1424.68	 25
  4	 E 	   223.08	  1360.15	 24
  5	 D 	   280.60	  1295.63	 23
  6	 D 	   338.11	  1238.12	 22
  7	 S 	   381.62	  1180.61	 21
  8	 L 	   438.17	  1137.09	 20
  9	 A 	   473.69	  1080.55	 19
 10	 N 	   530.71	  1045.03	 18
 11	 S 	   574.22	   988.01	 17
 12	 S 	   617.74	   944.49	 16
 13	 D 	   675.25	   900.98	 15
 14	 L 	   731.79	   843.46	 14
 15	 L 	   788.34	   786.92	 13
 16	 K 	   852.38	   730.38	 12
 17	 E 	   916.91	   666.33	 11
 18	 L 	   973.45	   601.81	 10
 19	 L 	  1029.99	   545.27	  9
 20	 E 	  1094.51	   488.73	  8
 21	 T 	  1145.03	   424.20	  7
 22	 G 	  1173.55	   373.68	  6
 23	 D 	  1231.06	   345.17	  5
 24	 N 	  1288.08	   287.66	  4
 25	 R 	  1366.13	   230.63	  3
 26	 E 	  1430.65	   152.58	  2
 27	 R 	        -  	    88.06	  1  _


+3 charge
		      B   	       Y
  1	 D 	    39.35	        -  	 27
  2	 A 	    63.03	   973.80	 26
  3	 E 	   106.04	   950.12	 25
  4	 E 	   149.06	   907.11	 24
  5	 D 	   187.40	   864.09	 23
  6	 D 	   225.74	   825.75	 22
  7	 S 	   254.75	   787.41	 21
  8	 L 	   292.45	   758.40	 20
  9	 A 	   316.13	   720.70	 19
 10	 N 	   354.14	   697.02	 18
 11	 S 	   383.15	   659.01	 17
 12	 S 	   412.16	   630.00	 16
 13	 D 	   450.50	   600.99	 15
 14	 L 	   488.20	   562.64	 14
 15	 L 	   525.89	   524.95	 13
 16	 K 	   568.59	   487.25	 12
 17	 E 	   611.61	   444.56	 11
 18	 L 	   649.30	   401.54	 10
 19	 L 	   687.00	   363.85	  9
 20	 E 	   730.01	   326.15	  8
 21	 T 	   763.69	   283.14	  7
 22	 G 	   782.70	   249.46	  6
 23	 D 	   821.04	   230.45	  5
 24	 N 	   859.06	   192.11	  4
 25	 R 	   911.09	   154.09	  3
 26	 E 	   954.10	   102.06	  2
 27	 R 	        -  	    59.04	  1  _
```

**54. IPI00028347 Isoform 1 of F-box only protein 31**

| Sequence | Charge | M+H+ | XCorr | DeltaCn |
| --- | --- | --- | --- | --- |
| R.IYLPPSRPDDLIKPGLFK.G | 2 | 2070.462 | 3.009 | 0.259 |

+1 charge

```
		      B   	       Y
  1	 I 	   114.09	        -  	 18
  2	 Y 	   277.15	  1956.10	 17
  3	 L 	   390.24	  1793.03	 16
  4	 P 	   487.29	  1679.95	 15
  5	 P 	   584.34	  1582.90	 14
  6	 S 	   671.38	  1485.84	 13
  7	 R 	   827.48	  1398.81	 12
  8	 P 	   924.53	  1242.71	 11
  9	 D 	  1039.56	  1145.66	 10
 10	 D 	  1154.58	  1030.63	  9
 11	 L 	  1267.67	   915.60	  8
 12	 I 	  1380.75	   802.52	  7
 13	 K 	  1508.85	   689.43	  6
 14	 P 	  1605.90	   561.34	  5
 15	 G 	  1662.92	   464.29	  4
 16	 L 	  1776.01	   407.27	  3
 17	 F 	  1923.07	   294.18	  2
 18	 K 	        -  	   147.11	  1  _
+2 charge
		      B   	       Y
  1	 I 	    57.55	        -  	 18
  2	 Y 	   139.08	   978.55	 17
  3	 L 	   195.62	   897.02	 16
  4	 P 	   244.15	   840.48	 15
  5	 P 	   292.68	   791.95	 14
  6	 S 	   336.19	   743.42	 13
  7	 R 	   414.24	   699.91	 12
  8	 P 	   462.77	   621.86	 11
  9	 D 	   520.28	   573.33	 10
 10	 D 	   577.80	   515.82	  9
 11	 L 	   634.34	   458.30	  8
 12	 I 	   690.88	   401.76	  7
 13	 K 	   754.93	   345.22	  6
 14	 P 	   803.45	   281.17	  5
 15	 G 	   831.96	   232.65	  4
 16	 L 	   888.51	   204.14	  3
 17	 F 	   962.04	   147.59	  2
 18	 K 	        -  	    74.06	  1  _
```

**55. IPI00001952 Endonuclease domain-containing 1 protein**

| Sequence | Charge | M+H+ | XCorr | DeltaCn |
| --- | --- | --- | --- | --- |
| K.ILEVVNQIQDEER.M | 2 | 1585.741 | 4.1 | 0.396 |

+1 charge

```
		      B   	       Y
  1	 I 	   114.09	        -  	 13
  2	 L 	   227.18	  1471.74	 12
  3	 E 	   356.22	  1358.65	 11
  4	 V 	   455.29	  1229.61	 10
  5	 V 	   554.35	  1130.54	  9
  6	 N 	   668.40	  1031.48	  8
  7	 Q 	   796.46	   917.43	  7
  8	 I 	   909.54	   789.37	  6
  9	 Q 	  1037.60	   676.29	  5
 10	 D 	  1152.63	   548.23	  4
 11	 E 	  1281.67	   433.20	  3
 12	 E 	  1410.71	   304.16	  2
 13	 R 	        -  	   175.12	  1  _
+2 charge
		      B   	       Y
  1	 I 	    57.55	        -  	 13
  2	 L 	   114.09	   736.37	 12
  3	 E 	   178.61	   679.83	 11
  4	 V 	   228.15	   615.31	 10
  5	 V 	   277.68	   565.78	  9
  6	 N 	   334.70	   516.24	  8
  7	 Q 	   398.73	   459.22	  7
  8	 I 	   455.27	   395.19	  6
  9	 Q 	   519.30	   338.65	  5
 10	 D 	   576.82	   274.62	  4
 11	 E 	   641.34	   217.11	  3
 12	 E 	   705.86	   152.58	  2
 13	 R 	        -  	    88.06	  1  _
```

**56. IPI00011694 Trypsin-1**

| Sequence | Charge | M+H+ | XCorr | DeltaCn |
| --- | --- | --- | --- | --- |
| K.TLNNDIMLIK.L | 2 | 1175.424 | 3.501 | 0.426 |

+1 charge

```
		      B   	       Y
  1	 T 	   102.05	        -  	 10
  2	 L 	   215.14	  1073.60	  9
  3	 N 	   329.18	   960.52	  8
  4	 N 	   443.22	   846.48	  7
  5	 D 	   558.25	   732.43	  6
  6	 I 	   671.34	   617.41	  5
  7	 M 	   802.38	   504.32	  4
  8	 L 	   915.46	   373.28	  3
  9	 I 	  1028.54	   260.20	  2
 10	 K 	        -  	   147.11	  1  _
+2 charge
		      B   	       Y
  1	 T 	    51.53	        -  	 10
  2	 L 	   108.07	   537.30	  9
  3	 N 	   165.09	   480.76	  8
  4	 N 	   222.12	   423.74	  7
  5	 D 	   279.63	   366.72	  6
  6	 I 	   336.17	   309.21	  5
  7	 M 	   401.69	   252.66	  4
  8	 L 	   458.23	   187.14	  3
  9	 I 	   514.78	   130.60	  2
 10	 K 	        -  	    74.06	  1  _
```

**57. IPI00216780 Cartilage intermediate layer protein 2 precursor**

| Sequence | Charge | M+H+ | XCorr | DeltaCn |
| --- | --- | --- | --- | --- |
| R.LLESPATALGDIR.R | 2 | 1356.55 | 3.217 | 0.453 |

+1 charge

```
		      B   	       Y
  1	 L 	   114.09	        -  	 13
  2	 L 	   227.18	  1242.67	 12
  3	 E 	   356.22	  1129.58	 11
  4	 S 	   443.25	  1000.54	 10
  5	 P 	   540.30	   913.51	  9
  6	 A 	   611.34	   816.46	  8
  7	 T 	   712.39	   745.42	  7
  8	 A 	   783.42	   644.37	  6
  9	 L 	   896.51	   573.34	  5
 10	 G 	   953.53	   460.25	  4
 11	 D 	  1068.56	   403.23	  3
 12	 I 	  1181.64	   288.20	  2
 13	 R 	        -  	   175.12	  1  _
+2 charge
		      B   	       Y
  1	 L 	    57.55	        -  	 13
  2	 L 	   114.09	   621.84	 12
  3	 E 	   178.61	   565.30	 11
  4	 S 	   222.13	   500.77	 10
  5	 P 	   270.66	   457.26	  9
  6	 A 	   306.17	   408.73	  8
  7	 T 	   356.70	   373.21	  7
  8	 A 	   392.22	   322.69	  6
  9	 L 	   448.76	   287.17	  5
 10	 G 	   477.27	   230.63	  4
 11	 D 	   534.78	   202.12	  3
 12	 I 	   591.32	   144.61	  2
 13	 R 	        -  	    88.06	  1  _
```

**58. IPI00008239 cDNA FLJ55176, highly similar to G-protein coupled receptor family C group 5 member B**

| Sequence | Charge | M+H+ | XCorr | DeltaCn |
| --- | --- | --- | --- | --- |
| R.SNVYQPTEMAVVLNGGTIPTAPPSHTGR.H | 3 | 2896.228 | 3.731 | 0.52 |

+1 charge

```
		      B   	       Y
  1	 S 	    88.04	        -  	 28
  2	 N 	   202.08	  2807.41	 27
  3	 V 	   301.15	  2693.37	 26
  4	 Y 	   464.21	  2594.30	 25
  5	 Q 	   592.27	  2431.24	 24
  6	 P 	   689.33	  2303.18	 23
  7	 T 	   790.37	  2206.13	 22
  8	 E 	   919.42	  2105.08	 21
  9	 M 	  1050.46	  1976.04	 20
 10	 A 	  1121.49	  1845.00	 19
 11	 V 	  1220.56	  1773.96	 18
 12	 V 	  1319.63	  1674.89	 17
 13	 L 	  1432.71	  1575.82	 16
 14	 N 	  1546.76	  1462.74	 15
 15	 G 	  1603.78	  1348.70	 14
 16	 G 	  1660.80	  1291.68	 13
 17	 T 	  1761.85	  1234.65	 12
 18	 I 	  1874.93	  1133.61	 11
 19	 P 	  1971.98	  1020.52	 10
 20	 T 	  2073.03	   923.47	  9
 21	 A 	  2144.07	   822.42	  8
 22	 P 	  2241.12	   751.38	  7
 23	 P 	  2338.17	   654.33	  6
 24	 S 	  2425.21	   557.28	  5
 25	 H 	  2562.27	   470.25	  4
 26	 T 	  2663.31	   333.19	  3
 27	 G 	  2720.33	   232.14	  2
 28	 R 	        -  	   175.12	  1  _


+2 charge
		      B   	       Y
  1	 S 	    44.52	        -  	 28
  2	 N 	   101.54	  1404.21	 27
  3	 V 	   151.08	  1347.19	 26
  4	 Y 	   232.61	  1297.66	 25
  5	 Q 	   296.64	  1216.12	 24
  6	 P 	   345.17	  1152.09	 23
  7	 T 	   395.69	  1103.57	 22
  8	 E 	   460.21	  1053.04	 21
  9	 M 	   525.73	   988.52	 20
 10	 A 	   561.25	   923.00	 19
 11	 V 	   610.78	   887.48	 18
 12	 V 	   660.32	   837.95	 17
 13	 L 	   716.86	   788.42	 16
 14	 N 	   773.88	   731.87	 15
 15	 G 	   802.39	   674.85	 14
 16	 G 	   830.90	   646.34	 13
 17	 T 	   881.43	   617.83	 12
 18	 I 	   937.97	   567.31	 11
 19	 P 	   986.50	   510.76	 10
 20	 T 	  1037.02	   462.24	  9
 21	 A 	  1072.54	   411.71	  8
 22	 P 	  1121.06	   376.20	  7
 23	 P 	  1169.59	   327.67	  6
 24	 S 	  1213.11	   279.14	  5
 25	 H 	  1281.64	   235.63	  4
 26	 T 	  1332.16	   167.10	  3
 27	 G 	  1360.67	   116.57	  2
 28	 R 	        -  	    88.06	  1  _


+3 charge
		      B   	       Y
  1	 S 	    30.02	        -  	 28
  2	 N 	    68.03	   936.48	 27
  3	 V 	   101.06	   898.46	 26
  4	 Y 	   155.41	   865.44	 25
  5	 Q 	   198.10	   811.08	 24
  6	 P 	   230.45	   768.40	 23
  7	 T 	   264.13	   736.05	 22
  8	 E 	   307.14	   702.37	 21
  9	 M 	   350.82	   659.35	 20
 10	 A 	   374.50	   615.67	 19
 11	 V 	   407.53	   591.99	 18
 12	 V 	   440.55	   558.97	 17
 13	 L 	   478.24	   525.95	 16
 14	 N 	   516.26	   488.25	 15
 15	 G 	   535.26	   450.24	 14
 16	 G 	   554.27	   431.23	 13
 17	 T 	   587.95	   412.22	 12
 18	 I 	   625.65	   378.54	 11
 19	 P 	   658.00	   340.85	 10
 20	 T 	   691.68	   308.49	  9
 21	 A 	   715.36	   274.81	  8
 22	 P 	   747.71	   251.13	  7
 23	 P 	   780.06	   218.78	  6
 24	 S 	   809.07	   186.43	  5
 25	 H 	   854.76	   157.42	  4
 26	 T 	   888.44	   111.73	  3
 27	 G 	   907.45	    78.05	  2
 28	 R 	        -  	    59.04	  1  _
```

**59. IPI00215767 Isoform Long of Beta-1,4-galactosyltransferase 1**

| Sequence | Charge | M+H+ | XCorr | DeltaCn |
| --- | --- | --- | --- | --- |
| R.DLSRLPQLVGVSTPLQGGSNSAAAIGQSSGELR.T | 3 | 3267.597 | 4.182 | 0.59 |

+1 charge

```
		      B   	       Y
  1	 D 	   116.03	        -  	 33
  2	 L 	   229.12	  3150.69	 32
  3	 S 	   316.15	  3037.60	 31
  4	 R 	   472.25	  2950.57	 30
  5	 L 	   585.34	  2794.47	 29
  6	 P 	   682.39	  2681.39	 28
  7	 Q 	   810.45	  2584.33	 27
  8	 L 	   923.53	  2456.27	 26
  9	 V 	  1022.60	  2343.19	 25
 10	 G 	  1079.62	  2244.12	 24
 11	 V 	  1178.69	  2187.10	 23
 12	 S 	  1265.72	  2088.03	 22
 13	 T 	  1366.77	  2001.00	 21
 14	 P 	  1463.82	  1899.95	 20
 15	 L 	  1576.91	  1802.90	 19
 16	 Q 	  1704.96	  1689.82	 18
 17	 G 	  1761.99	  1561.76	 17
 18	 G 	  1819.01	  1504.74	 16
 19	 S 	  1906.04	  1447.71	 15
 20	 N 	  2020.08	  1360.68	 14
 21	 S 	  2107.11	  1246.64	 13
 22	 A 	  2178.15	  1159.61	 12
 23	 A 	  2249.19	  1088.57	 11
 24	 A 	  2320.23	  1017.53	 10
 25	 I 	  2433.31	   946.50	  9
 26	 G 	  2490.33	   833.41	  8
 27	 Q 	  2618.39	   776.39	  7
 28	 S 	  2705.42	   648.33	  6
 29	 S 	  2792.45	   561.30	  5
 30	 G 	  2849.48	   474.27	  4
 31	 E 	  2978.52	   417.25	  3
 32	 L 	  3091.60	   288.20	  2
 33	 R 	        -  	   175.12	  1  _


+2 charge
		      B   	       Y
  1	 D 	    58.52	        -  	 33
  2	 L 	   115.06	  1575.85	 32
  3	 S 	   158.58	  1519.30	 31
  4	 R 	   236.63	  1475.79	 30
  5	 L 	   293.17	  1397.74	 29
  6	 P 	   341.70	  1341.20	 28
  7	 Q 	   405.73	  1292.67	 27
  8	 L 	   462.27	  1228.64	 26
  9	 V 	   511.80	  1172.10	 25
 10	 G 	   540.31	  1122.56	 24
 11	 V 	   589.85	  1094.05	 23
 12	 S 	   633.36	  1044.52	 22
 13	 T 	   683.89	  1001.00	 21
 14	 P 	   732.41	   950.48	 20
 15	 L 	   788.96	   901.95	 19
 16	 Q 	   852.99	   845.41	 18
 17	 G 	   881.50	   781.38	 17
 18	 G 	   910.01	   752.87	 16
 19	 S 	   953.52	   724.36	 15
 20	 N 	  1010.54	   680.84	 14
 21	 S 	  1054.06	   623.82	 13
 22	 A 	  1089.58	   580.31	 12
 23	 A 	  1125.10	   544.79	 11
 24	 A 	  1160.62	   509.27	 10
 25	 I 	  1217.16	   473.75	  9
 26	 G 	  1245.67	   417.21	  8
 27	 Q 	  1309.70	   388.70	  7
 28	 S 	  1353.21	   324.67	  6
 29	 S 	  1396.73	   281.15	  5
 30	 G 	  1425.24	   237.64	  4
 31	 E 	  1489.76	   209.13	  3
 32	 L 	  1546.30	   144.61	  2
 33	 R 	        -  	    88.06	  1  _


+3 charge
		      B   	       Y
  1	 D 	    39.35	        -  	 33
  2	 L 	    77.04	  1050.90	 32
  3	 S 	   106.05	  1013.21	 31
  4	 R 	   158.09	   984.20	 30
  5	 L 	   195.78	   932.16	 29
  6	 P 	   228.13	   894.47	 28
  7	 Q 	   270.82	   862.12	 27
  8	 L 	   308.52	   819.43	 26
  9	 V 	   341.54	   781.73	 25
 10	 G 	   360.55	   748.71	 24
 11	 V 	   393.57	   729.70	 23
 12	 S 	   422.58	   696.68	 22
 13	 T 	   456.26	   667.67	 21
 14	 P 	   488.61	   633.99	 20
 15	 L 	   526.31	   601.64	 19
 16	 Q 	   568.99	   563.94	 18
 17	 G 	   588.00	   521.26	 17
 18	 G 	   607.01	   502.25	 16
 19	 S 	   636.02	   483.24	 15
 20	 N 	   674.03	   454.23	 14
 21	 S 	   703.04	   416.22	 13
 22	 A 	   726.72	   387.21	 12
 23	 A 	   750.40	   363.53	 11
 24	 A 	   774.08	   339.85	 10
 25	 I 	   811.77	   316.17	  9
 26	 G 	   830.78	   278.48	  8
 27	 Q 	   873.47	   259.47	  7
 28	 S 	   902.48	   216.78	  6
 29	 S 	   931.49	   187.77	  5
 30	 G 	   950.50	   158.76	  4
 31	 E 	   993.51	   139.75	  3
 32	 L 	  1031.21	    96.74	  2
 33	 R 	        -  	    59.04	  1  _
```

**60. IPI00296777 SPARC-like protein 1**

| Sequence | Charge | M+H+ | XCorr | DeltaCn |
| --- | --- | --- | --- | --- |
| K.SIPTCTDFEVIQFPLR.M | 2 | 1924.18 | 2.95 | 0.445 |

+1 charge

```
		      B   	       Y
  1	 S 	    88.04	        -  	 16
  2	 I 	   201.12	  1836.07	 15
  3	 P 	   298.18	  1722.99	 14
  4	 T 	   399.22	  1625.93	 13
  5	 C 	   559.39	  1524.89	 12
  6	 T 	   660.44	  1364.72	 11
  7	 D 	   775.46	  1263.67	 10
  8	 F 	   922.53	  1148.65	  9
  9	 E 	  1051.57	  1001.58	  8
 10	 V 	  1150.64	   872.54	  7
 11	 I 	  1263.73	   773.47	  6
 12	 Q 	  1391.79	   660.38	  5
 13	 F 	  1538.85	   532.32	  4
 14	 P 	  1635.91	   385.26	  3
 15	 L 	  1748.99	   288.20	  2
 16	 R 	        -  	   175.12	  1  _
+2 charge
		      B   	       Y
  1	 S 	    44.52	        -  	 16
  2	 I 	   101.07	   918.54	 15
  3	 P 	   149.59	   862.00	 14
  4	 T 	   200.12	   813.47	 13
  5	 C 	   280.20	   762.95	 12
  6	 T 	   330.72	   682.86	 11
  7	 D 	   388.24	   632.34	 10
  8	 F 	   461.77	   574.83	  9
  9	 E 	   526.29	   501.29	  8
 10	 V 	   575.83	   436.77	  7
 11	 I 	   632.37	   387.24	  6
 12	 Q 	   696.40	   330.70	  5
 13	 F 	   769.93	   266.67	  4
 14	 P 	   818.46	   193.13	  3
 15	 L 	   875.00	   144.61	  2
 16	 R 	        -  	    88.06	  1  _
```

**61. IPI00297263 Isoform 1 of Protein HEG homolog 1**

| Sequence | Charge | M+H+ | XCorr | DeltaCn |
| --- | --- | --- | --- | --- |
| R.ALSLAPLAGAGLELQLER.R | 2 | 1823.127 | 5.164 | 0.514 |

+1 charge

```
		      B   	       Y
  1	 A 	    72.04	        -  	 18
  2	 L 	   185.13	  1751.01	 17
  3	 S 	   272.16	  1637.92	 16
  4	 L 	   385.24	  1550.89	 15
  5	 A 	   456.28	  1437.81	 14
  6	 P 	   553.33	  1366.77	 13
  7	 L 	   666.42	  1269.72	 12
  8	 A 	   737.46	  1156.63	 11
  9	 G 	   794.48	  1085.59	 10
 10	 A 	   865.51	  1028.57	  9
 11	 G 	   922.54	   957.54	  8
 12	 L 	  1035.62	   900.51	  7
 13	 E 	  1164.66	   787.43	  6
 14	 L 	  1277.75	   658.39	  5
 15	 Q 	  1405.80	   545.30	  4
 16	 L 	  1518.89	   417.25	  3
 17	 E 	  1647.93	   304.16	  2
 18	 R 	        -  	   175.12	  1  _
+2 charge
		      B   	       Y
  1	 A 	    36.53	        -  	 18
  2	 L 	    93.07	   876.01	 17
  3	 S 	   136.58	   819.46	 16
  4	 L 	   193.13	   775.95	 15
  5	 A 	   228.64	   719.41	 14
  6	 P 	   277.17	   683.89	 13
  7	 L 	   333.71	   635.36	 12
  8	 A 	   369.23	   578.82	 11
  9	 G 	   397.74	   543.30	 10
 10	 A 	   433.26	   514.79	  9
 11	 G 	   461.77	   479.27	  8
 12	 L 	   518.31	   450.76	  7
 13	 E 	   582.83	   394.22	  6
 14	 L 	   639.38	   329.70	  5
 15	 Q 	   703.41	   273.16	  4
 16	 L 	   759.95	   209.13	  3
 17	 E 	   824.47	   152.58	  2
 18	 R 	        -  	    88.06	  1  _
```

**62. IPI00184546 UPF0444 transmembrane protein C12orf23**

| Sequence | Charge | M+H+ | XCorr | DeltaCn |
| --- | --- | --- | --- | --- |
| K.GGVSAVAGGVTAVGSAVVNKVPLTGK.K | 2 | 2296.653 | 3.259 | 0.303 |

+1 charge

```
		      B   	       Y
  1	 G 	    58.03	        -  	 26
  2	 G 	   115.05	  2238.28	 25
  3	 V 	   214.12	  2181.26	 24
  4	 S 	   301.15	  2082.19	 23
  5	 A 	   372.19	  1995.16	 22
  6	 V 	   471.26	  1924.12	 21
  7	 A 	   542.29	  1825.05	 20
  8	 G 	   599.31	  1754.02	 19
  9	 G 	   656.34	  1697.00	 18
 10	 V 	   755.40	  1639.97	 17
 11	 T 	   856.45	  1540.91	 16
 12	 A 	   927.49	  1439.86	 15
 13	 V 	  1026.56	  1368.82	 14
 14	 G 	  1083.58	  1269.75	 13
 15	 S 	  1170.61	  1212.73	 12
 16	 A 	  1241.65	  1125.70	 11
 17	 V 	  1340.72	  1054.66	 10
 18	 V 	  1439.79	   955.59	  9
 19	 N 	  1553.83	   856.53	  8
 20	 K 	  1681.92	   742.48	  7
 21	 V 	  1780.99	   614.39	  6
 22	 P 	  1878.04	   515.32	  5
 23	 L 	  1991.13	   418.27	  4
 24	 T 	  2092.18	   305.18	  3
 25	 G 	  2149.20	   204.13	  2
 26	 K 	        -  	   147.11	  1  _


+2 charge
		      B   	       Y
  1	 G 	    29.52	        -  	 26
  2	 G 	    58.03	  1119.64	 25
  3	 V 	   107.56	  1091.13	 24
  4	 S 	   151.08	  1041.60	 23
  5	 A 	   186.60	   998.08	 22
  6	 V 	   236.13	   962.56	 21
  7	 A 	   271.65	   913.03	 20
  8	 G 	   300.16	   877.51	 19
  9	 G 	   328.67	   849.00	 18
 10	 V 	   378.21	   820.49	 17
 11	 T 	   428.73	   770.96	 16
 12	 A 	   464.25	   720.43	 15
 13	 V 	   513.78	   684.91	 14
 14	 G 	   542.29	   635.38	 13
 15	 S 	   585.81	   606.87	 12
 16	 A 	   621.33	   563.35	 11
 17	 V 	   670.86	   527.83	 10
 18	 V 	   720.40	   478.30	  9
 19	 N 	   777.42	   428.77	  8
 20	 K 	   841.47	   371.74	  7
 21	 V 	   891.00	   307.70	  6
 22	 P 	   939.53	   258.16	  5
 23	 L 	   996.07	   209.64	  4
 24	 T 	  1046.59	   153.09	  3
 25	 G 	  1075.10	   102.57	  2
 26	 K 	        -  	    74.06	  1  _
```

**63. IPI00009027 Lithostathine-1-alpha**

| Sequence | Charge | M+H+ | XCorr | DeltaCn |
| --- | --- | --- | --- | --- |
| R.ISCPEGTNAYR.S | 2 | 1268.352 | 3.387 | 0.532 |

+1 charge

```
		      B   	       Y
  1	 I 	   114.09	        -  	 11
  2	 S 	   201.12	  1154.62	 10
  3	 C 	   361.29	  1067.59	  9
  4	 P 	   458.34	   907.43	  8
  5	 E 	   587.38	   810.37	  7
  6	 G 	   644.41	   681.33	  6
  7	 T 	   745.45	   624.31	  5
  8	 N 	   859.50	   523.26	  4
  9	 A 	   930.53	   409.22	  3
 10	 Y 	  1093.60	   338.18	  2
 11	 R 	        -  	   175.12	  1  _
+2 charge
		      B   	       Y
  1	 I 	    57.55	        -  	 11
  2	 S 	   101.07	   577.82	 10
  3	 C 	   181.15	   534.30	  9
  4	 P 	   229.67	   454.22	  8
  5	 E 	   294.20	   405.69	  7
  6	 G 	   322.71	   341.17	  6
  7	 T 	   373.23	   312.66	  5
  8	 N 	   430.25	   262.13	  4
  9	 A 	   465.77	   205.11	  3
 10	 Y 	   547.30	   169.59	  2
 11	 R 	        -  	    88.06	  1  _
```

**64. IPI00299145 Keratin, type II cytoskeletal 6C**

| Sequence | Charge | M+H+ | XCorr | DeltaCn |
| --- | --- | --- | --- | --- |
| R.AIGGGLSSVGGGSSTIK.Y | 2 | 1448.604 | 4.308 | 0.517 |

+1 charge

```
		      B   	       Y
  1	 A 	    72.04	        -  	 17
  2	 I 	   185.13	  1376.74	 16
  3	 G 	   242.15	  1263.65	 15
  4	 G 	   299.17	  1206.63	 14
  5	 G 	   356.19	  1149.61	 13
  6	 L 	   469.28	  1092.59	 12
  7	 S 	   556.31	   979.51	 11
  8	 S 	   643.34	   892.47	 10
  9	 V 	   742.41	   805.44	  9
 10	 G 	   799.43	   706.37	  8
 11	 G 	   856.45	   649.35	  7
 12	 G 	   913.47	   592.33	  6
 13	 S 	  1000.51	   535.31	  5
 14	 S 	  1087.54	   448.28	  4
 15	 T 	  1188.59	   361.24	  3
 16	 I 	  1301.67	   260.20	  2
 17	 K 	        -  	   147.11	  1  _
+2 charge
		      B   	       Y
  1	 A 	    36.53	        -  	 17
  2	 I 	    93.07	   688.87	 16
  3	 G 	   121.58	   632.33	 15
  4	 G 	   150.09	   603.82	 14
  5	 G 	   178.60	   575.31	 13
  6	 L 	   235.14	   546.80	 12
  7	 S 	   278.66	   490.26	 11
  8	 S 	   322.17	   446.74	 10
  9	 V 	   371.71	   403.22	  9
 10	 G 	   400.22	   353.69	  8
 11	 G 	   428.73	   325.18	  7
 12	 G 	   457.24	   296.67	  6
 13	 S 	   500.76	   268.16	  5
 14	 S 	   544.27	   224.64	  4
 15	 T 	   594.80	   181.13	  3
 16	 I 	   651.34	   130.60	  2
 17	 K 	        -  	    74.06	  1  _
```

**65. IPI00384404 Rheumatoid factor RF-ET9 (Fragment)**

| Sequence | Charge | M+H+ | XCorr | DeltaCn |
| --- | --- | --- | --- | --- |
| - .LVESGGGLVKPGGSLR.L | 2 | 1526.763 | 4.079 | 0.372 |

+1 charge

```
		      B   	       Y
  1	 L 	   114.09	        -  	 16
  2	 V 	   213.16	  1412.79	 15
  3	 E 	   342.20	  1313.72	 14
  4	 S 	   429.23	  1184.67	 13
  5	 G 	   486.26	  1097.64	 12
  6	 G 	   543.28	  1040.62	 11
  7	 G 	   600.30	   983.60	 10
  8	 L 	   713.38	   926.58	  9
  9	 V 	   812.45	   813.49	  8
 10	 K 	   940.55	   714.43	  7
 11	 P 	  1037.60	   586.33	  6
 12	 G 	  1094.62	   489.28	  5
 13	 G 	  1151.64	   432.26	  4
 14	 S 	  1238.67	   375.24	  3
 15	 L 	  1351.76	   288.20	  2
 16	 R 	        -  	   175.12	  1  _
+2 charge
		      B   	       Y
  1	 L 	    57.55	        -  	 16
  2	 V 	   107.08	   706.90	 15
  3	 E 	   171.60	   657.36	 14
  4	 S 	   215.12	   592.84	 13
  5	 G 	   243.63	   549.32	 12
  6	 G 	   272.14	   520.81	 11
  7	 G 	   300.65	   492.30	 10
  8	 L 	   357.20	   463.79	  9
  9	 V 	   406.73	   407.25	  8
 10	 K 	   470.78	   357.72	  7
 11	 P 	   519.30	   293.67	  6
 12	 G 	   547.81	   245.14	  5
 13	 G 	   576.32	   216.63	  4
 14	 S 	   619.84	   188.12	  3
 15	 L 	   676.38	   144.61	  2
 16	 R 	        -  	    88.06	  1  _
```

**66. IPI00746352 SH3 domain-binding glutamic acid-rich-like protein 3**

| Sequence | Charge | M+H+ | XCorr | DeltaCn |
| --- | --- | --- | --- | --- |
| R.IQYQLVDISQDNALRDEMR.A | 3 | 2308.558 | 3.377 | 0.378 |

+1 charge

```
		      B   	       Y
  1	 I 	   114.09	        -  	 19
  2	 Q 	   242.15	  2194.06	 18
  3	 Y 	   405.21	  2066.00	 17
  4	 Q 	   533.27	  1902.93	 16
  5	 L 	   646.36	  1774.88	 15
  6	 V 	   745.42	  1661.79	 14
  7	 D 	   860.45	  1562.72	 13
  8	 I 	   973.54	  1447.70	 12
  9	 S 	  1060.57	  1334.61	 11
 10	 Q 	  1188.63	  1247.58	 10
 11	 D 	  1303.65	  1119.52	  9
 12	 N 	  1417.70	  1004.49	  8
 13	 A 	  1488.73	   890.45	  7
 14	 L 	  1601.82	   819.41	  6
 15	 R 	  1757.92	   706.33	  5
 16	 D 	  1872.95	   550.23	  4
 17	 E 	  2001.99	   435.20	  3
 18	 M 	  2133.03	   306.16	  2
 19	 R 	        -  	   175.12	  1  _
+2 charge
		      B   	       Y
  1	 I 	    57.55	        -  	 19
  2	 Q 	   121.58	  1097.53	 18
  3	 Y 	   203.11	  1033.50	 17
  4	 Q 	   267.14	   951.97	 16
  5	 L 	   323.68	   887.94	 15
  6	 V 	   373.22	   831.40	 14
  7	 D 	   430.73	   781.87	 13
  8	 I 	   487.27	   724.35	 12
  9	 S 	   530.79	   667.81	 11
 10	 Q 	   594.82	   624.29	 10
 11	 D 	   652.33	   560.26	  9
 12	 N 	   709.35	   502.75	  8
 13	 A 	   744.87	   445.73	  7
 14	 L 	   801.41	   410.21	  6
 15	 R 	   879.46	   353.67	  5
 16	 D 	   936.98	   275.62	  4
 17	 E 	  1001.50	   218.10	  3
 18	 M 	  1067.02	   153.58	  2
 19	 R 	        -  	    88.06	  1  _


+3 charge
		      B   	       Y
  1	 I 	    38.70	        -  	 19
  2	 Q 	    81.39	   732.02	 18
  3	 Y 	   135.74	   689.34	 17
  4	 Q 	   178.43	   634.98	 16
  5	 L 	   216.12	   592.30	 15
  6	 V 	   249.15	   554.60	 14
  7	 D 	   287.49	   521.58	 13
  8	 I 	   325.18	   483.24	 12
  9	 S 	   354.19	   445.54	 11
 10	 Q 	   396.88	   416.53	 10
 11	 D 	   435.22	   373.85	  9
 12	 N 	   473.24	   335.50	  8
 13	 A 	   496.92	   297.49	  7
 14	 L 	   534.61	   273.81	  6
 15	 R 	   586.64	   236.11	  5
 16	 D 	   624.99	   184.08	  4
 17	 E 	   668.00	   145.74	  3
 18	 M 	   711.68	   102.72	  2
 19	 R 	        -  	    59.04	  1  _
```

**67. IPI00000854 Lutropin subunit beta**

| Sequence | Charge | M+H+ | XCorr | DeltaCn |
| --- | --- | --- | --- | --- |
| R.GVDPVVSFPVALSCR.C | 2 | 1603.838 | 2.885 | 0.424 |

+1 charge

```
		      B   	       Y
  1	 G 	    58.03	        -  	 15
  2	 V 	   157.10	  1545.94	 14
  3	 D 	   272.12	  1446.88	 13
  4	 P 	   369.18	  1331.85	 12
  5	 V 	   468.25	  1234.80	 11
  6	 V 	   567.31	  1135.73	 10
  7	 S 	   654.35	  1036.66	  9
  8	 F 	   801.41	   949.63	  8
  9	 P 	   898.47	   802.56	  7
 10	 V 	   997.54	   705.51	  6
 11	 A 	  1068.57	   606.44	  5
 12	 L 	  1181.66	   535.40	  4
 13	 S 	  1268.69	   422.32	  3
 14	 C 	  1428.85	   335.28	  2
 15	 R 	        -  	   175.12	  1  _
+2 charge
		      B   	       Y
  1	 G 	    29.52	        -  	 15
  2	 V 	    79.05	   773.48	 14
  3	 D 	   136.57	   723.94	 13
  4	 P 	   185.09	   666.43	 12
  5	 V 	   234.63	   617.90	 11
  6	 V 	   284.16	   568.37	 10
  7	 S 	   327.68	   518.83	  9
  8	 F 	   401.21	   475.32	  8
  9	 P 	   449.74	   401.78	  7
 10	 V 	   499.27	   353.26	  6
 11	 A 	   534.79	   303.72	  5
 12	 L 	   591.33	   268.20	  4
 13	 S 	   634.85	   211.66	  3
 14	 C 	   714.93	   168.15	  2
 15	 R 	        -  	    88.06	  1  _
```

**68. IPI00873924 Isoform A of Interleukin-18-binding protein**

| Sequence | Charge | M+H+ | XCorr | DeltaCn |
| --- | --- | --- | --- | --- |
| K.ALVLEQLTPALHSTNFSCVLVDPEQVVQR.H | 3 | 3265.699 | 4.885 | 0.565 |

+1 charge

```
		      B   	       Y
  1	 A 	    72.04	        -  	 29
  2	 L 	   185.13	  3192.81	 28
  3	 V 	   284.20	  3079.72	 27
  4	 L 	   397.28	  2980.65	 26
  5	 E 	   526.32	  2867.57	 25
  6	 Q 	   654.38	  2738.53	 24
  7	 L 	   767.47	  2610.47	 23
  8	 T 	   868.51	  2497.38	 22
  9	 P 	   965.57	  2396.34	 21
 10	 A 	  1036.60	  2299.28	 20
 11	 L 	  1149.69	  2228.25	 19
 12	 H 	  1286.75	  2115.16	 18
 13	 S 	  1373.78	  1978.10	 17
 14	 T 	  1474.83	  1891.07	 16
 15	 N 	  1588.87	  1790.02	 15
 16	 F 	  1735.94	  1675.98	 14
 17	 S 	  1822.97	  1528.91	 13
 18	 C 	  1983.14	  1441.88	 12
 19	 V 	  2082.20	  1281.72	 11
 20	 L 	  2195.29	  1182.65	 10
 21	 V 	  2294.36	  1069.56	  9
 22	 D 	  2409.38	   970.50	  8
 23	 P 	  2506.44	   855.47	  7
 24	 E 	  2635.48	   758.42	  6
 25	 Q 	  2763.54	   629.37	  5
 26	 V 	  2862.61	   501.31	  4
 27	 V 	  2961.67	   402.25	  3
 28	 Q 	  3089.73	   303.18	  2
 29	 R 	        -  	   175.12	  1  _


+2 charge
		      B   	       Y
  1	 A 	    36.53	        -  	 29
  2	 L 	    93.07	  1596.91	 28
  3	 V 	   142.60	  1540.36	 27
  4	 L 	   199.14	  1490.83	 26
  5	 E 	   263.67	  1434.29	 25
  6	 Q 	   327.69	  1369.77	 24
  7	 L 	   384.24	  1305.74	 23
  8	 T 	   434.76	  1249.20	 22
  9	 P 	   483.29	  1198.67	 21
 10	 A 	   518.81	  1150.15	 20
 11	 L 	   575.35	  1114.63	 19
 12	 H 	   643.88	  1058.09	 18
 13	 S 	   687.39	   989.56	 17
 14	 T 	   737.92	   946.04	 16
 15	 N 	   794.94	   895.52	 15
 16	 F 	   868.47	   838.49	 14
 17	 S 	   911.99	   764.96	 13
 18	 C 	   992.07	   721.44	 12
 19	 V 	  1041.61	   641.36	 11
 20	 L 	  1098.15	   591.83	 10
 21	 V 	  1147.68	   535.29	  9
 22	 D 	  1205.20	   485.75	  8
 23	 P 	  1253.72	   428.24	  7
 24	 E 	  1318.24	   379.71	  6
 25	 Q 	  1382.27	   315.19	  5
 26	 V 	  1431.81	   251.16	  4
 27	 V 	  1481.34	   201.63	  3
 28	 Q 	  1545.37	   152.09	  2
 29	 R 	        -  	    88.06	  1  _


+3 charge
		      B   	       Y
  1	 A 	    24.69	        -  	 29
  2	 L 	    62.38	  1064.94	 28
  3	 V 	    95.40	  1027.25	 27
  4	 L 	   133.10	   994.22	 26
  5	 E 	   176.11	   956.53	 25
  6	 Q 	   218.80	   913.51	 24
  7	 L 	   256.49	   870.83	 23
  8	 T 	   290.18	   833.13	 22
  9	 P 	   322.53	   799.45	 21
 10	 A 	   346.21	   767.10	 20
 11	 L 	   383.90	   743.42	 19
 12	 H 	   429.59	   705.73	 18
 13	 S 	   458.60	   660.04	 17
 14	 T 	   492.28	   631.03	 16
 15	 N 	   530.29	   597.35	 15
 16	 F 	   579.32	   559.33	 14
 17	 S 	   608.33	   510.31	 13
 18	 C 	   661.72	   481.30	 12
 19	 V 	   694.74	   427.91	 11
 20	 L 	   732.43	   394.89	 10
 21	 V 	   765.46	   357.19	  9
 22	 D 	   803.80	   324.17	  8
 23	 P 	   836.15	   285.83	  7
 24	 E 	   879.16	   253.48	  6
 25	 Q 	   921.85	   210.46	  5
 26	 V 	   954.87	   167.78	  4
 27	 V 	   987.90	   134.75	  3
 28	 Q 	  1030.58	   101.73	  2
 29	 R 	        -  	    59.04	  1  _
```

**69. IPI00789902 Isoform 2 of G-protein coupled receptor family C group 5 member C**

| Sequence | Charge | M+H+ | XCorr | DeltaCn |
| --- | --- | --- | --- | --- |
| R.ATANSQVMGSANSTLR.A | 2 | 1608.76 | 2.913 | 0.428 |

**+1 charge**

```
		      B   	       Y
  1	 A 	    72.04	        -  	 16
  2	 T 	   173.09	  1536.74	 15
  3	 A 	   244.13	  1435.70	 14
  4	 N 	   358.17	  1364.66	 13
  5	 S 	   445.20	  1250.62	 12
  6	 Q 	   573.26	  1163.58	 11
  7	 V 	   672.33	  1035.53	 10
  8	 M 	   803.37	   936.46	  9
  9	 G 	   860.39	   805.42	  8
 10	 S 	   947.43	   748.39	  7
 11	 A 	  1018.46	   661.36	  6
 12	 N 	  1132.51	   590.33	  5
 13	 S 	  1219.54	   476.28	  4
 14	 T 	  1320.58	   389.25	  3
 15	 L 	  1433.67	   288.20	  2
 16	 R 	        -  	   175.12	  1  _
+2 charge
		      B   	       Y
  1	 A 	    36.53	        -  	 16
  2	 T 	    87.05	   768.88	 15
  3	 A 	   122.57	   718.35	 14
  4	 N 	   179.59	   682.83	 13
  5	 S 	   223.11	   625.81	 12
  6	 Q 	   287.13	   582.30	 11
  7	 V 	   336.67	   518.27	 10
  8	 M 	   402.19	   468.73	  9
  9	 G 	   430.70	   403.21	  8
 10	 S 	   474.22	   374.70	  7
 11	 A 	   509.73	   331.19	  6
 12	 N 	   566.76	   295.67	  5
 13	 S 	   610.27	   238.64	  4
 14	 T 	   660.80	   195.13	  3
 15	 L 	   717.34	   144.61	  2
 16	 R 	        -  	    88.06	  1  _
```

**70. IPI00006091 Isoform 4 of Dystrophin**

| Sequence | Charge | M+H+ | XCorr | DeltaCn |
| --- | --- | --- | --- | --- |
| K.ETLVEDKLSLLNSNWIAVTSR.A | 3 | 2389.69 | 3.476 | 0.279 |

+1 charge

```
		      B   	       Y
  1	 E 	   130.05	        -  	 21
  2	 T 	   231.10	  2259.23	 20
  3	 L 	   344.18	  2158.19	 19
  4	 V 	   443.25	  2045.10	 18
  5	 E 	   572.29	  1946.03	 17
  6	 D 	   687.32	  1816.99	 16
  7	 K 	   815.41	  1701.96	 15
  8	 L 	   928.50	  1573.87	 14
  9	 S 	  1015.53	  1460.79	 13
 10	 L 	  1128.61	  1373.75	 12
 11	 L 	  1241.70	  1260.67	 11
 12	 N 	  1355.74	  1147.59	 10
 13	 S 	  1442.77	  1033.54	  9
 14	 N 	  1556.82	   946.51	  8
 15	 W 	  1742.90	   832.47	  7
 16	 I 	  1855.98	   646.39	  6
 17	 A 	  1927.02	   533.30	  5
 18	 V 	  2026.09	   462.27	  4
 19	 T 	  2127.13	   363.20	  3
 20	 S 	  2214.17	   262.15	  2
 21	 R 	        -  	   175.12	  1  _
+2 charge
		      B   	       Y
  1	 E 	    65.53	        -  	 21
  2	 T 	   116.05	  1130.12	 20
  3	 L 	   172.59	  1079.60	 19
  4	 V 	   222.13	  1023.05	 18
  5	 E 	   286.65	   973.52	 17
  6	 D 	   344.16	   909.00	 16
  7	 K 	   408.21	   851.49	 15
  8	 L 	   464.75	   787.44	 14
  9	 S 	   508.27	   730.90	 13
 10	 L 	   564.81	   687.38	 12
 11	 L 	   621.35	   630.84	 11
 12	 N 	   678.37	   574.30	 10
 13	 S 	   721.89	   517.27	  9
 14	 N 	   778.91	   473.76	  8
 15	 W 	   871.95	   416.74	  7
 16	 I 	   928.49	   323.70	  6
 17	 A 	   964.01	   267.16	  5
 18	 V 	  1013.55	   231.64	  4
 19	 T 	  1064.07	   182.10	  3
 20	 S 	  1107.59	   131.58	  2
 21	 R 	        -  	    88.06	  1  _
+3 charge
		      B   	       Y
  1	 E 	    44.02	        -  	 21
  2	 T 	    77.70	   753.75	 20
  3	 L 	   115.40	   720.07	 19
  4	 V 	   148.42	   682.37	 18
  5	 E 	   191.44	   649.35	 17
  6	 D 	   229.78	   606.34	 16
  7	 K 	   272.48	   567.99	 15
  8	 L 	   310.17	   525.29	 14
  9	 S 	   339.18	   487.60	 13
 10	 L 	   376.88	   458.59	 12
 11	 L 	   414.57	   420.89	 11
 12	 N 	   452.59	   383.20	 10
 13	 S 	   481.60	   345.19	  9
 14	 N 	   519.61	   316.18	  8
 15	 W 	   581.64	   278.16	  7
 16	 I 	   619.33	   216.13	  6
 17	 A 	   643.01	   178.44	  5
 18	 V 	   676.03	   154.76	  4
 19	 T 	   709.72	   121.74	  3
 20	 S 	   738.73	    88.06	  2
 21	 R 	        -  	    59.04	  1  _
```

**71. IPI00183445 Isoform 1 of Latrophilin-1**

| Sequence | Charge | M+H+ | XCorr | DeltaCn |
| --- | --- | --- | --- | --- |
| R.ELACEGYPIELR.C | 2 | 1450.612 | 3.04 | 0.229 |

+1 charge

```
		      B   	       Y
  1	 E 	   130.05	        -  	 12
  2	 L 	   243.13	  1320.80	 11
  3	 A 	   314.17	  1207.71	 10
  4	 C 	   474.34	  1136.68	  9
  5	 E 	   603.38	   976.51	  8
  6	 G 	   660.40	   847.47	  7
  7	 Y 	   823.46	   790.45	  6
  8	 P 	   920.52	   627.38	  5
  9	 I 	  1033.60	   530.33	  4
 10	 E 	  1162.64	   417.25	  3
 11	 L 	  1275.73	   288.20	  2
 12	 R 	        -  	   175.12	  1  _
+2 charge
		      B   	       Y
  1	 E 	    65.53	        -  	 12
  2	 L 	   122.07	   660.90	 11
  3	 A 	   157.59	   604.36	 10
  4	 C 	   237.67	   568.84	  9
  5	 E 	   302.19	   488.76	  8
  6	 G 	   330.70	   424.24	  7
  7	 Y 	   412.24	   395.73	  6
  8	 P 	   460.76	   314.19	  5
  9	 I 	   517.30	   265.67	  4
 10	 E 	   581.83	   209.13	  3
 11	 L 	   638.37	   144.61	  2
 12	 R 	        -  	    88.06	  1  _
```

**72. IPI00004416 Charged multivesicular body protein 2a**

| Sequence | Charge | M+H+ | XCorr | DeltaCn |
| --- | --- | --- | --- | --- |
| K.AEAAASALADADADLEERLK.N | 2 | 2031.167 | 3.518 | 0.404 |

+1 charge

```
		      B   	       Y
  1	 A 	    72.04	        -  	 20
  2	 E 	   201.09	  1958.97	 19
  3	 A 	   272.12	  1829.92	 18
  4	 A 	   343.16	  1758.89	 17
  5	 A 	   414.20	  1687.85	 16
  6	 S 	   501.23	  1616.81	 15
  7	 A 	   572.27	  1529.78	 14
  8	 L 	   685.35	  1458.74	 13
  9	 A 	   756.39	  1345.66	 12
 10	 D 	   871.42	  1274.62	 11
 11	 A 	   942.45	  1159.60	 10
 12	 D 	  1057.48	  1088.56	  9
 13	 A 	  1128.52	   973.53	  8
 14	 D 	  1243.54	   902.49	  7
 15	 L 	  1356.63	   787.47	  6
 16	 E 	  1485.67	   674.38	  5
 17	 E 	  1614.71	   545.34	  4
 18	 R 	  1770.81	   416.30	  3
 19	 L 	  1883.90	   260.20	  2
 20	 K 	        -  	   147.11	  1  _
+2 charge
		      B   	       Y
  1	 A 	    36.53	        -  	 20
  2	 E 	   101.05	   979.99	 19
  3	 A 	   136.57	   915.47	 18
  4	 A 	   172.08	   879.95	 17
  5	 A 	   207.60	   844.43	 16
  6	 S 	   251.12	   808.91	 15
  7	 A 	   286.64	   765.39	 14
  8	 L 	   343.18	   729.88	 13
  9	 A 	   378.70	   673.33	 12
 10	 D 	   436.21	   637.81	 11
 11	 A 	   471.73	   580.30	 10
 12	 D 	   529.24	   544.78	  9
 13	 A 	   564.76	   487.27	  8
 14	 D 	   622.28	   451.75	  7
 15	 L 	   678.82	   394.24	  6
 16	 E 	   743.34	   337.70	  5
 17	 E 	   807.86	   273.17	  4
 18	 R 	   885.91	   208.65	  3
 19	 L 	   942.45	   130.60	  2
 20	 K 	        -  	    74.06	  1  _
```

**73. IPI00166392 Similar to Cell adhesion molecule 1 precursor**

| Sequence | Charge | M+H+ | XCorr | DeltaCn |
| --- | --- | --- | --- | --- |
| R.YLEVQYKPQVHIQMTYPLQGLTR.E | 3 | 2807.262 | 3.379 | 0.326 |

+1 charge

```
		      B   	       Y
  1	 Y 	   164.07	        -  	 23
  2	 L 	   277.15	  2642.41	 22
  3	 E 	   406.20	  2529.33	 21
  4	 V 	   505.27	  2400.29	 20
  5	 Q 	   633.32	  2301.22	 19
  6	 Y 	   796.39	  2173.16	 18
  7	 K 	   924.48	  2010.10	 17
  8	 P 	  1021.54	  1882.00	 16
  9	 Q 	  1149.59	  1784.95	 15
 10	 V 	  1248.66	  1656.89	 14
 11	 H 	  1385.72	  1557.82	 13
 12	 I 	  1498.81	  1420.76	 12
 13	 Q 	  1626.86	  1307.68	 11
 14	 M 	  1757.90	  1179.62	 10
 15	 T 	  1858.95	  1048.58	  9
 16	 Y 	  2022.02	   947.53	  8
 17	 P 	  2119.07	   784.47	  7
 18	 L 	  2232.15	   687.41	  6
 19	 Q 	  2360.21	   574.33	  5
 20	 G 	  2417.23	   446.27	  4
 21	 L 	  2530.32	   389.25	  3
 22	 T 	  2631.36	   276.17	  2
 23	 R 	        -  	   175.12	  1  _


+2 charge
		      B   	       Y
  1	 Y 	    82.54	        -  	 23
  2	 L 	   139.08	  1321.71	 22
  3	 E 	   203.60	  1265.17	 21
  4	 V 	   253.14	  1200.65	 20
  5	 Q 	   317.17	  1151.11	 19
  6	 Y 	   398.70	  1087.08	 18
  7	 K 	   462.74	  1005.55	 17
  8	 P 	   511.27	   941.50	 16
  9	 Q 	   575.30	   892.98	 15
 10	 V 	   624.83	   828.95	 14
 11	 H 	   693.36	   779.41	 13
 12	 I 	   749.91	   710.88	 12
 13	 Q 	   813.94	   654.34	 11
 14	 M 	   879.46	   590.31	 10
 15	 T 	   929.98	   524.79	  9
 16	 Y 	  1011.51	   474.27	  8
 17	 P 	  1060.04	   392.74	  7
 18	 L 	  1116.58	   344.21	  6
 19	 Q 	  1180.61	   287.67	  5
 20	 G 	  1209.12	   223.64	  4
 21	 L 	  1265.66	   195.13	  3
 22	 T 	  1316.19	   138.59	  2
 23	 R 	        -  	    88.06	  1  _
+3 charge
		      B   	       Y
  1	 Y 	    55.36	        -  	 23
  2	 L 	    93.06	   881.48	 22
  3	 E 	   136.07	   843.78	 21
  4	 V 	   169.09	   800.77	 20
  5	 Q 	   211.78	   767.74	 19
  6	 Y 	   266.13	   725.06	 18
  7	 K 	   308.83	   670.70	 17
  8	 P 	   341.18	   628.00	 16
  9	 Q 	   383.87	   595.65	 15
 10	 V 	   416.89	   552.97	 14
 11	 H 	   462.58	   519.95	 13
 12	 I 	   500.27	   474.26	 12
 13	 Q 	   542.96	   436.56	 11
 14	 M 	   586.64	   393.88	 10
 15	 T 	   620.32	   350.20	  9
 16	 Y 	   674.68	   316.52	  8
 17	 P 	   707.03	   262.16	  7
 18	 L 	   744.72	   229.81	  6
 19	 Q 	   787.41	   192.12	  5
 20	 G 	   806.42	   149.43	  4
 21	 L 	   844.11	   130.42	  3
 22	 T 	   877.79	    92.73	  2
 23	 R 	        -  	    59.04	  1  _
```

**74. IPI00056309 Liver-expressed antimicrobial peptide 2**

| Sequence | Charge | M+H+ | XCorr | DeltaCn |
| --- | --- | --- | --- | --- |
| R.GVSLRPIGASCRDDSECITR.L | 3 | 2250.443 | 4.732 | 0.414 |

+1 charge

```
		      B   	       Y
  1	 G 	    58.03	        -  	 20
  2	 V 	   157.10	  2192.32	 19
  3	 S 	   244.13	  2093.26	 18
  4	 L 	   357.21	  2006.22	 17
  5	 R 	   513.31	  1893.14	 16
  6	 P 	   610.37	  1737.04	 15
  7	 I 	   723.45	  1639.99	 14
  8	 G 	   780.47	  1526.90	 13
  9	 A 	   851.51	  1469.88	 12
 10	 S 	   938.54	  1398.84	 11
 11	 C 	  1098.71	  1311.81	 10
 12	 R 	  1254.81	  1151.65	  9
 13	 D 	  1369.84	   995.54	  8
 14	 D 	  1484.86	   880.52	  7
 15	 S 	  1571.89	   765.49	  6
 16	 E 	  1700.94	   678.46	  5
 17	 C 	  1861.10	   549.42	  4
 18	 I 	  1974.19	   389.25	  3
 19	 T 	  2075.23	   276.17	  2
 20	 R 	        -  	   175.12	  1  _
+2 charge
		      B   	       Y
  1	 G 	    29.52	        -  	 20
  2	 V 	    79.05	  1096.67	 19
  3	 S 	   122.57	  1047.13	 18
  4	 L 	   179.11	  1003.62	 17
  5	 R 	   257.16	   947.07	 16
  6	 P 	   305.69	   869.02	 15
  7	 I 	   362.23	   820.50	 14
  8	 G 	   390.74	   763.95	 13
  9	 A 	   426.26	   735.44	 12
 10	 S 	   469.77	   699.93	 11
 11	 C 	   549.86	   656.41	 10
 12	 R 	   627.91	   576.33	  9
 13	 D 	   685.42	   498.28	  8
 14	 D 	   742.93	   440.76	  7
 15	 S 	   786.45	   383.25	  6
 16	 E 	   850.97	   339.73	  5
 17	 C 	   931.05	   275.21	  4
 18	 I 	   987.60	   195.13	  3
 19	 T 	  1038.12	   138.59	  2
 20	 R 	        -  	    88.06	  1  _


+3 charge
		      B   	       Y
  1	 G 	    20.01	        -  	 20
  2	 V 	    53.04	   731.45	 19
  3	 S 	    82.05	   698.42	 18
  4	 L 	   119.74	   669.41	 17
  5	 R 	   171.78	   631.72	 16
  6	 P 	   204.13	   579.68	 15
  7	 I 	   241.82	   547.33	 14
  8	 G 	   260.83	   509.64	 13
  9	 A 	   284.51	   490.63	 12
 10	 S 	   313.52	   466.95	 11
 11	 C 	   366.91	   437.94	 10
 12	 R 	   418.94	   384.55	  9
 13	 D 	   457.28	   332.52	  8
 14	 D 	   495.63	   294.18	  7
 15	 S 	   524.64	   255.84	  6
 16	 E 	   567.65	   226.82	  5
 17	 C 	   621.04	   183.81	  4
 18	 I 	   658.73	   130.42	  3
 19	 T 	   692.42	    92.73	  2
 20	 R 	        -  	    59.04	  1  _
```

**75. IPI00031464 Butyrophilin subfamily 2 member A3**

| Sequence | Charge | M+H+ | XCorr | DeltaCn |
| --- | --- | --- | --- | --- |
| R.SQFSPAVFVYKGGR.E | 2 | 1543.751 | 3.681 | 0.526 |

+1 charge

```
		      B   	       Y
  1	 S 	    88.04	        -  	 14
  2	 Q 	   216.10	  1455.77	 13
  3	 F 	   363.17	  1327.72	 12
  4	 S 	   450.20	  1180.65	 11
  5	 P 	   547.25	  1093.62	 10
  6	 A 	   618.29	   996.56	  9
  7	 V 	   717.36	   925.53	  8
  8	 F 	   864.43	   826.46	  7
  9	 V 	   963.49	   679.39	  6
 10	 Y 	  1126.56	   580.32	  5
 11	 K 	  1254.65	   417.26	  4
 12	 G 	  1311.67	   289.16	  3
 13	 G 	  1368.69	   232.14	  2
 14	 R 	        -  	   175.12	  1  _
+2 charge
		      B   	       Y
  1	 S 	    44.52	        -  	 14
  2	 Q 	   108.55	   728.39	 13
  3	 F 	   182.09	   664.36	 12
  4	 S 	   225.60	   590.83	 11
  5	 P 	   274.13	   547.31	 10
  6	 A 	   309.65	   498.78	  9
  7	 V 	   359.18	   463.27	  8
  8	 F 	   432.72	   413.73	  7
  9	 V 	   482.25	   340.20	  6
 10	 Y 	   563.78	   290.66	  5
 11	 K 	   627.83	   209.13	  4
 12	 G 	   656.34	   145.08	  3
 13	 G 	   684.85	   116.57	  2
 14	 R 	        -  	    88.06	  1  _
```

**76. IPI00414542 Isoform 1 of Programmed cell death 1 ligand 2**

| Sequence | Charge | M+H+ | XCorr | DeltaCn |
| --- | --- | --- | --- | --- |
| R.ATLLEEQLPLGK.A | 2 | 1312.537 | 2.872 | 0.421 |

+1 charge

```
		      B   	       Y
  1	 A 	    72.04	        -  	 12
  2	 T 	   173.09	  1240.71	 11
  3	 L 	   286.18	  1139.67	 10
  4	 L 	   399.26	  1026.58	  9
  5	 E 	   528.30	   913.50	  8
  6	 E 	   657.35	   784.46	  7
  7	 Q 	   785.40	   655.41	  6
  8	 L 	   898.49	   527.36	  5
  9	 P 	   995.54	   414.27	  4
 10	 L 	  1108.62	   317.22	  3
 11	 G 	  1165.65	   204.13	  2
 12	 K 	        -  	   147.11	  1  _
+2 charge
		      B   	       Y
  1	 A 	    36.53	        -  	 12
  2	 T 	    87.05	   620.86	 11
  3	 L 	   143.59	   570.34	 10
  4	 L 	   200.13	   513.80	  9
  5	 E 	   264.66	   457.25	  8
  6	 E 	   329.18	   392.73	  7
  7	 Q 	   393.21	   328.21	  6
  8	 L 	   449.75	   264.18	  5
  9	 P 	   498.27	   207.64	  4
 10	 L 	   554.82	   159.11	  3
 11	 G 	   583.33	   102.57	  2
 12	 K 	        -  	    74.06	  1  _
```

**77. IPI00940353 Isoform 1 of Gamma-glutamyltransferase 6**

| Sequence | Charge | M+H+ | XCorr | DeltaCn |
| --- | --- | --- | --- | --- |
| R.LLVGPTTLAQEGFLVDTPLAR.A | 2 | 2212.574 | 3.566 | 0.552 |

+1 charge

```
		      B   	       Y
  1	 L 	   114.09	        -  	 21
  2	 L 	   227.18	  2098.15	 20
  3	 V 	   326.24	  1985.07	 19
  4	 G 	   383.27	  1886.00	 18
  5	 P 	   480.32	  1828.98	 17
  6	 T 	   581.37	  1731.93	 16
  7	 T 	   682.41	  1630.88	 15
  8	 L 	   795.50	  1529.83	 14
  9	 A 	   866.53	  1416.75	 13
 10	 Q 	   994.59	  1345.71	 12
 11	 E 	  1123.64	  1217.65	 11
 12	 G 	  1180.66	  1088.61	 10
 13	 F 	  1327.73	  1031.59	  9
 14	 L 	  1440.81	   884.52	  8
 15	 V 	  1539.88	   771.44	  7
 16	 D 	  1654.91	   672.37	  6
 17	 T 	  1755.95	   557.34	  5
 18	 P 	  1853.01	   456.29	  4
 19	 L 	  1966.09	   359.24	  3
 20	 A 	  2037.13	   246.16	  2
 21	 R 	        -  	   175.12	  1  _
+2 charge
		      B   	       Y
  1	 L 	    57.55	        -  	 21
  2	 L 	   114.09	  1049.58	 20
  3	 V 	   163.63	   993.04	 19
  4	 G 	   192.14	   943.50	 18
  5	 P 	   240.66	   914.99	 17
  6	 T 	   291.19	   866.47	 16
  7	 T 	   341.71	   815.94	 15
  8	 L 	   398.25	   765.42	 14
  9	 A 	   433.77	   708.88	 13
 10	 Q 	   497.80	   673.36	 12
 11	 E 	   562.32	   609.33	 11
 12	 G 	   590.83	   544.81	 10
 13	 F 	   664.37	   516.30	  9
 14	 L 	   720.91	   442.76	  8
 15	 V 	   770.44	   386.22	  7
 16	 D 	   827.96	   336.69	  6
 17	 T 	   878.48	   279.17	  5
 18	 P 	   927.01	   228.65	  4
 19	 L 	   983.55	   180.12	  3
 20	 A 	  1019.07	   123.58	  2
 21	 R 	        -  	    88.06	  1  _
```

**78. IPI00026926 Guanylin**

| Sequence | Charge | M+H+ | XCorr | DeltaCn |
| --- | --- | --- | --- | --- |
| K.LKDLQEPQEPR.V | 2 | 1353.506 | 2.984 | 0.351 |

+1 charge

```
		      B   	       Y
  1	 L 	   114.09	        -  	 11
  2	 K 	   242.19	  1239.63	 10
  3	 D 	   357.21	  1111.54	  9
  4	 L 	   470.30	   996.51	  8
  5	 Q 	   598.36	   883.43	  7
  6	 E 	   727.40	   755.37	  6
  7	 P 	   824.45	   626.33	  5
  8	 Q 	   952.51	   529.27	  4
  9	 E 	  1081.55	   401.21	  3
 10	 P 	  1178.61	   272.17	  2
 11	 R 	        -  	   175.12	  1  _
+2 charge
		      B   	       Y
  1	 L 	    57.55	        -  	 11
  2	 K 	   121.60	   620.32	 10
  3	 D 	   179.11	   556.27	  9
  4	 L 	   235.65	   498.76	  8
  5	 Q 	   299.68	   442.22	  7
  6	 E 	   364.20	   378.19	  6
  7	 P 	   412.73	   313.67	  5
  8	 Q 	   476.76	   265.14	  4
  9	 E 	   541.28	   201.11	  3
 10	 P 	   589.81	   136.59	  2
 11	 R 	        -  	    88.06	  1  _
```

**79. IPI00045957 Isoform 1 of Cyclic AMP-responsive element-binding protein 3-like protein 3**

| Sequence | Charge | M+H+ | XCorr | DeltaCn |
| --- | --- | --- | --- | --- |
| R.AGLEAAGDEL.- | 1 | 945.9938 | 2.313 | 0.322 |

+1 charge

```
		      B   	       Y
  1	 A 	    72.04	        -  	 10
  2	 G 	   129.07	   874.42	  9
  3	 L 	   242.15	   817.39	  8
  4	 E 	   371.19	   704.31	  7
  5	 A 	   442.23	   575.27	  6
  6	 A 	   513.27	   504.23	  5
  7	 G 	   570.29	   433.19	  4
  8	 D 	   685.32	   376.17	  3
  9	 E 	   814.36	   261.14	  2
 10	 L 	        -  	   132.10	  1  _
```

**80. IPI00553177 Isoform 1 of Alpha-1-antitrypsin**

| Sequence | Charge | M+H+ | XCorr | DeltaCn |
| --- | --- | --- | --- | --- |
| K.SPLFMGKVVNPTQK.- | 2 | 1546.859 | 4.057 | 0.366 |

+1 charge

```
		      B   	       Y
  1	 S 	    88.04	        -  	 14
  2	 P 	   185.09	  1458.81	 13
  3	 L 	   298.18	  1361.76	 12
  4	 F 	   445.24	  1248.68	 11
  5	 M 	   576.29	  1101.61	 10
  6	 G 	   633.31	   970.57	  9
  7	 K 	   761.40	   913.55	  8
  8	 V 	   860.47	   785.45	  7
  9	 V 	   959.54	   686.38	  6
 10	 N 	  1073.58	   587.31	  5
 11	 P 	  1170.63	   473.27	  4
 12	 T 	  1271.68	   376.22	  3
 13	 Q 	  1399.74	   275.17	  2
 14	 K 	        -  	   147.11	  1  _
+2 charge
		      B   	       Y
  1	 S 	    44.52	        -  	 14
  2	 P 	    93.05	   729.91	 13
  3	 L 	   149.59	   681.38	 12
  4	 F 	   223.13	   624.84	 11
  5	 M 	   288.65	   551.31	 10
  6	 G 	   317.16	   485.79	  9
  7	 K 	   381.20	   457.28	  8
  8	 V 	   430.74	   393.23	  7
  9	 V 	   480.27	   343.70	  6
 10	 N 	   537.29	   294.16	  5
 11	 P 	   585.82	   237.14	  4
 12	 T 	   636.34	   188.61	  3
 13	 Q 	   700.37	   138.09	  2
 14	 K 	        -  	    74.06	  1  _
```

**81. IPI00013446 Prostate stem cell antigen**

| Sequence | Charge | M+H+ | XCorr | DeltaCn |
| --- | --- | --- | --- | --- |
| R.AVGLLTVISK.G | 2 | 1001.245 | 3.637 | 0.203 |

+1 charge

```
		      B   	       Y
  1	 A 	    72.04	        -  	 10
  2	 V 	   171.11	   929.60	  9
  3	 G 	   228.13	   830.53	  8
  4	 L 	   341.22	   773.51	  7
  5	 L 	   454.30	   660.43	  6
  6	 T 	   555.35	   547.34	  5
  7	 V 	   654.42	   446.30	  4
  8	 I 	   767.50	   347.23	  3
  9	 S 	   854.53	   234.14	  2
 10	 K 	        -  	   147.11	  1  _
+2 charge
		      B   	       Y
  1	 A 	    36.53	        -  	 10
  2	 V 	    86.06	   465.31	  9
  3	 G 	   114.57	   415.77	  8
  4	 L 	   171.11	   387.26	  7
  5	 L 	   227.65	   330.72	  6
  6	 T 	   278.18	   274.18	  5
  7	 V 	   327.71	   223.65	  4
  8	 I 	   384.25	   174.12	  3
  9	 S 	   427.77	   117.58	  2
 10	 K 	        -  	    74.06	  1  _
```

**82. IPI00219788 Isoform A of Protein FAM3B**

| Sequence | Charge | M+H+ | XCorr | DeltaCn |
| --- | --- | --- | --- | --- |
| K.ICFEDNLLMGEQLGNVAR.G | 2 | 2080.345 | 3.083 | 0.396 |

+1 charge

```
		      B   	       Y
  1	 I 	   114.09	        -  	 18
  2	 C 	   274.26	  1966.05	 17
  3	 F 	   421.32	  1805.89	 16
  4	 E 	   550.37	  1658.82	 15
  5	 D 	   665.39	  1529.77	 14
  6	 N 	   779.44	  1414.75	 13
  7	 L 	   892.52	  1300.70	 12
  8	 L 	  1005.61	  1187.62	 11
  9	 M 	  1136.65	  1074.54	 10
 10	 G 	  1193.67	   943.50	  9
 11	 E 	  1322.71	   886.47	  8
 12	 Q 	  1450.77	   757.43	  7
 13	 L 	  1563.85	   629.37	  6
 14	 G 	  1620.87	   516.29	  5
 15	 N 	  1734.92	   459.27	  4
 16	 V 	  1833.99	   345.22	  3
 17	 A 	  1905.02	   246.16	  2
 18	 R 	        -  	   175.12	  1  _
+2 charge
		      B   	       Y
  1	 I 	    57.55	        -  	 18
  2	 C 	   137.63	   983.53	 17
  3	 F 	   211.17	   903.45	 16
  4	 E 	   275.69	   829.91	 15
  5	 D 	   333.20	   765.39	 14
  6	 N 	   390.22	   707.88	 13
  7	 L 	   446.76	   650.86	 12
  8	 L 	   503.31	   594.31	 11
  9	 M 	   568.83	   537.77	 10
 10	 G 	   597.34	   472.25	  9
 11	 E 	   661.86	   443.74	  8
 12	 Q 	   725.89	   379.22	  7
 13	 L 	   782.43	   315.19	  6
 14	 G 	   810.94	   258.65	  5
 15	 N 	   867.96	   230.14	  4
 16	 V 	   917.50	   173.12	  3
 17	 A 	   953.01	   123.58	  2
 18	 R 	        -  	    88.06	  1  _
```

**83. IPI00794461 Histone H2B type 1-N**

| Sequence | Charge | M+H+ | XCorr | DeltaCn |
| --- | --- | --- | --- | --- |
| K.AMGIMNSFVNDIFER.I | 2 | 1745.017 | 4.153 | 0.566 |

+1 charge

```
		      B   	       Y
  1	 A 	    72.04	        -  	 15
  2	 M 	   203.08	  1672.78	 14
  3	 G 	   260.11	  1541.74	 13
  4	 I 	   373.19	  1484.72	 12
  5	 M 	   504.23	  1371.64	 11
  6	 N 	   618.27	  1240.60	 10
  7	 S 	   705.31	  1126.55	  9
  8	 F 	   852.37	  1039.52	  8
  9	 V 	   951.44	   892.45	  7
 10	 N 	  1065.49	   793.38	  6
 11	 D 	  1180.51	   679.34	  5
 12	 I 	  1293.60	   564.31	  4
 13	 F 	  1440.67	   451.23	  3
 14	 E 	  1569.71	   304.16	  2
 15	 R 	        -  	   175.12	  1  _
+2 charge
		      B   	       Y
  1	 A 	    36.53	        -  	 15
  2	 M 	   102.05	   836.89	 14
  3	 G 	   130.56	   771.37	 13
  4	 I 	   187.10	   742.86	 12
  5	 M 	   252.62	   686.32	 11
  6	 N 	   309.64	   620.80	 10
  7	 S 	   353.16	   563.78	  9
  8	 F 	   426.69	   520.26	  8
  9	 V 	   476.22	   446.73	  7
 10	 N 	   533.25	   397.20	  6
 11	 D 	   590.76	   340.17	  5
 12	 I 	   647.30	   282.66	  4
 13	 F 	   720.84	   226.12	  3
 14	 E 	   785.36	   152.58	  2
 15	 R 	        -  	    88.06	  1  _
```

**84. IPI00027235 Isoform 1 of Attractin**

| Sequence | Charge | M+H+ | XCorr | DeltaCn |
| --- | --- | --- | --- | --- |
| R.SCALDQNCQWEPR.N | 2 | 1664.745 | 2.852 | 0.259 |

+1 charge

```
		      B   	       Y
  1	 S 	    88.04	        -  	 13
  2	 C 	   248.20	  1576.93	 12
  3	 A 	   319.24	  1416.77	 11
  4	 L 	   432.33	  1345.73	 10
  5	 D 	   547.35	  1232.65	  9
  6	 Q 	   675.41	  1117.62	  8
  7	 N 	   789.45	   989.56	  7
  8	 C 	   949.62	   875.52	  6
  9	 Q 	  1077.68	   715.35	  5
 10	 W 	  1263.76	   587.29	  4
 11	 E 	  1392.80	   401.21	  3
 12	 P 	  1489.85	   272.17	  2
 13	 R 	        -  	   175.12	  1  _
+2 charge
		      B   	       Y
  1	 S 	    44.52	        -  	 13
  2	 C 	   124.61	   788.97	 12
  3	 A 	   160.12	   708.89	 11
  4	 L 	   216.67	   673.37	 10
  5	 D 	   274.18	   616.83	  9
  6	 Q 	   338.21	   559.31	  8
  7	 N 	   395.23	   495.28	  7
  8	 C 	   475.31	   438.26	  6
  9	 Q 	   539.34	   358.18	  5
 10	 W 	   632.38	   294.15	  4
 11	 E 	   696.90	   201.11	  3
 12	 P 	   745.43	   136.59	  2
 13	 R 	        -  	    88.06	  1  _
```

**85. IPI00022284 Major prion protein**

| Sequence | Charge | M+H+ | XCorr | DeltaCn |
| --- | --- | --- | --- | --- |
| R.VVEQMCITQYER.E | 2 | 1556.76 | 4.064 | 0.401 |

+1 charge

```
		      B   	       Y
  1	 V 	   100.08	        -  	 12
  2	 V 	   199.14	  1456.79	 11
  3	 E 	   328.19	  1357.72	 10
  4	 Q 	   456.25	  1228.68	  9
  5	 M 	   587.29	  1100.62	  8
  6	 C 	   747.45	   969.58	  7
  7	 I 	   860.54	   809.42	  6
  8	 T 	   961.58	   696.33	  5
  9	 Q 	  1089.64	   595.28	  4
 10	 Y 	  1252.70	   467.22	  3
 11	 E 	  1381.75	   304.16	  2
 12	 R 	        -  	   175.12	  1  _
+2 charge
		      B   	       Y
  1	 V 	    50.54	        -  	 12
  2	 V 	   100.08	   728.90	 11
  3	 E 	   164.60	   679.36	 10
  4	 Q 	   228.63	   614.84	  9
  5	 M 	   294.15	   550.81	  8
  6	 C 	   374.23	   485.29	  7
  7	 I 	   430.77	   405.21	  6
  8	 T 	   481.30	   348.67	  5
  9	 Q 	   545.32	   298.15	  4
 10	 Y 	   626.86	   234.12	  3
 11	 E 	   691.38	   152.58	  2
 12	 R 	        -  	    88.06	  1  _
```

**86. IPI00299158 Isoform Alpha of Poliovirus receptor**

| Sequence | Charge | M+H+ | XCorr | DeltaCn |
| --- | --- | --- | --- | --- |
| R.VLAKPQNTAEVQKVQLTGEPVPMAR.C | 3 | 2706.158 | 5.393 | 0.568 |

+1 charge

```
		      B   	       Y
  1	 V 	   100.08	        -  	 25
  2	 L 	   213.16	  2605.41	 24
  3	 A 	   284.20	  2492.33	 23
  4	 K 	   412.29	  2421.29	 22
  5	 P 	   509.34	  2293.20	 21
  6	 Q 	   637.40	  2196.14	 20
  7	 N 	   751.45	  2068.09	 19
  8	 T 	   852.49	  1954.04	 18
  9	 A 	   923.53	  1852.99	 17
 10	 E 	  1052.57	  1781.96	 16
 11	 V 	  1151.64	  1652.92	 15
 12	 Q 	  1279.70	  1553.85	 14
 13	 K 	  1407.80	  1425.79	 13
 14	 V 	  1506.86	  1297.69	 12
 15	 Q 	  1634.92	  1198.62	 11
 16	 L 	  1748.01	  1070.57	 10
 17	 T 	  1849.05	   957.48	  9
 18	 G 	  1906.08	   856.43	  8
 19	 E 	  2035.12	   799.41	  7
 20	 P 	  2132.17	   670.37	  6
 21	 V 	  2231.24	   573.32	  5
 22	 P 	  2328.29	   474.25	  4
 23	 M 	  2459.33	   377.20	  3
 24	 A 	  2530.37	   246.16	  2
 25	 R 	        -  	   175.12	  1  _


+2 charge
		      B   	       Y
  1	 V 	    50.54	        -  	 25
  2	 L 	   107.08	  1303.21	 24
  3	 A 	   142.60	  1246.67	 23
  4	 K 	   206.65	  1211.15	 22
  5	 P 	   255.18	  1147.10	 21
  6	 Q 	   319.21	  1098.58	 20
  7	 N 	   376.23	  1034.55	 19
  8	 T 	   426.75	   977.52	 18
  9	 A 	   462.27	   927.00	 17
 10	 E 	   526.79	   891.48	 16
 11	 V 	   576.32	   826.96	 15
 12	 Q 	   640.35	   777.43	 14
 13	 K 	   704.40	   713.40	 13
 14	 V 	   753.94	   649.35	 12
 15	 Q 	   817.96	   599.82	 11
 16	 L 	   874.51	   535.79	 10
 17	 T 	   925.03	   479.24	  9
 18	 G 	   953.54	   428.72	  8
 19	 E 	  1018.06	   400.21	  7
 20	 P 	  1066.59	   335.69	  6
 21	 V 	  1116.12	   287.16	  5
 22	 P 	  1164.65	   237.63	  4
 23	 M 	  1230.17	   189.10	  3
 24	 A 	  1265.69	   123.58	  2
 25	 R 	        -  	    88.06	  1  _


+3 charge
		      B   	       Y
  1	 V 	    34.03	        -  	 25
  2	 L 	    71.72	   869.14	 24
  3	 A 	    95.40	   831.45	 23
  4	 K 	   138.10	   807.77	 22
  5	 P 	   170.45	   765.07	 21
  6	 Q 	   213.14	   732.72	 20
  7	 N 	   251.15	   690.03	 19
  8	 T 	   284.84	   652.02	 18
  9	 A 	   308.52	   618.34	 17
 10	 E 	   351.53	   594.66	 16
 11	 V 	   384.55	   551.64	 15
 12	 Q 	   427.24	   518.62	 14
 13	 K 	   469.94	   475.93	 13
 14	 V 	   502.96	   433.24	 12
 15	 Q 	   545.65	   400.21	 11
 16	 L 	   583.34	   357.53	 10
 17	 T 	   617.02	   319.83	  9
 18	 G 	   636.03	   286.15	  8
 19	 E 	   679.04	   267.14	  7
 20	 P 	   711.40	   224.13	  6
 21	 V 	   744.42	   191.78	  5
 22	 P 	   776.77	   158.75	  4
 23	 M 	   820.45	   126.40	  3
 24	 A 	   844.13	    82.72	  2
 25	 R 	        -  	    59.04	  1  _
```

**87. IPI00329688 Protein YIPF3**

| Sequence | Charge | M+H+ | XCorr | DeltaCn |
| --- | --- | --- | --- | --- |
| R.DIPAMLPAAR.L | 1 | 1055.276 | 2.548 | 0.31 |

+1 charge

```
		      B   	       Y
  1	 D 	   116.03	        -  	 10
  2	 I 	   229.12	   939.54	  9
  3	 P 	   326.17	   826.46	  8
  4	 A 	   397.21	   729.41	  7
  5	 M 	   528.25	   658.37	  6
  6	 L 	   641.33	   527.33	  5
  7	 P 	   738.39	   414.25	  4
  8	 A 	   809.42	   317.19	  3
  9	 A 	   880.46	   246.16	  2
 10	 R 	        -  	   175.12	  1  _
```

**88. IPI00002435 CD27 antigen**

| Sequence | Charge | M+H+ | XCorr | DeltaCn |
| --- | --- | --- | --- | --- |
| K.LCCQMCEPGTFLVK.D | 2 | 1744.027 | 4.488 | 0.551 |

+1 charge

```
		      B   	       Y
  1	 L 	   114.09	        -  	 14
  2	 C 	   274.26	  1630.09	 13
  3	 C 	   434.42	  1469.93	 12
  4	 Q 	   562.48	  1309.76	 11
  5	 M 	   693.52	  1181.70	 10
  6	 C 	   853.69	  1050.66	  9
  7	 E 	   982.73	   890.50	  8
  8	 P 	  1079.78	   761.46	  7
  9	 G 	  1136.80	   664.40	  6
 10	 T 	  1237.85	   607.38	  5
 11	 F 	  1384.92	   506.33	  4
 12	 L 	  1498.00	   359.27	  3
 13	 V 	  1597.07	   246.18	  2
 14	 K 	        -  	   147.11	  1  _
+2 charge
		      B   	       Y
  1	 L 	    57.55	        -  	 14
  2	 C 	   137.63	   815.55	 13
  3	 C 	   217.71	   735.47	 12
  4	 Q 	   281.74	   655.38	 11
  5	 M 	   347.26	   591.36	 10
  6	 C 	   427.35	   525.84	  9
  7	 E 	   491.87	   445.75	  8
  8	 P 	   540.39	   381.23	  7
  9	 G 	   568.91	   332.71	  6
 10	 T 	   619.43	   304.19	  5
 11	 F 	   692.96	   253.67	  4
 12	 L 	   749.51	   180.14	  3
 13	 V 	   799.04	   123.59	  2
 14	 K 	        -  	    74.06	  1  _
```

**89. IPI00784119 V-type proton ATPase subunit S1**

| Sequence | Charge | M+H+ | XCorr | DeltaCn |
| --- | --- | --- | --- | --- |
| K.LGASPLHVDLATLR.E | 3 | 1463.707 | 3.437 | 0.429 |

+1 charge

```
		      B   	       Y
  1	 L 	   114.09	        -  	 14
  2	 G 	   171.11	  1349.75	 13
  3	 A 	   242.15	  1292.73	 12
  4	 S 	   329.18	  1221.69	 11
  5	 P 	   426.23	  1134.66	 10
  6	 L 	   539.32	  1037.61	  9
  7	 H 	   676.38	   924.53	  8
  8	 V 	   775.45	   787.47	  7
  9	 D 	   890.47	   688.40	  6
 10	 L 	  1003.56	   573.37	  5
 11	 A 	  1074.59	   460.29	  4
 12	 T 	  1175.64	   389.25	  3
 13	 L 	  1288.73	   288.20	  2
 14	 R 	        -  	   175.12	  1  _
+2 charge
		      B   	       Y
  1	 L 	    57.55	        -  	 14
  2	 G 	    86.06	   675.38	 13
  3	 A 	   121.58	   646.87	 12
  4	 S 	   165.09	   611.35	 11
  5	 P 	   213.62	   567.84	 10
  6	 L 	   270.16	   519.31	  9
  7	 H 	   338.69	   462.77	  8
  8	 V 	   388.23	   394.24	  7
  9	 D 	   445.74	   344.70	  6
 10	 L 	   502.28	   287.19	  5
 11	 A 	   537.80	   230.65	  4
 12	 T 	   588.32	   195.13	  3
 13	 L 	   644.87	   144.61	  2
 14	 R 	        -  	    88.06	  1  _


+3 charge
		      B   	       Y
  1	 L 	    38.70	        -  	 14
  2	 G 	    57.71	   450.59	 13
  3	 A 	    81.39	   431.58	 12
  4	 S 	   110.40	   407.90	 11
  5	 P 	   142.75	   378.89	 10
  6	 L 	   180.44	   346.54	  9
  7	 H 	   226.13	   308.85	  8
  8	 V 	   259.15	   263.16	  7
  9	 D 	   297.50	   230.14	  6
 10	 L 	   335.19	   191.80	  5
 11	 A 	   358.87	   154.10	  4
 12	 T 	   392.55	   130.42	  3
 13	 L 	   430.25	    96.74	  2
 14	 R 	        -  	    59.04	  1  _
```

**90. IPI00387120 Ig kappa chain V-IV region Len**

| Sequence | Charge | M+H+ | XCorr | DeltaCn |
| --- | --- | --- | --- | --- |
| - .DIVMTQSPDSLAVSLGER.A | 2 | 1919.147 | 5.607 | 0.511 |

+1 charge

```
		      B   	       Y
  1	 D 	   116.03	        -  	 18
  2	 I 	   229.12	  1802.93	 17
  3	 V 	   328.19	  1689.85	 16
  4	 M 	   459.23	  1590.78	 15
  5	 T 	   560.27	  1459.74	 14
  6	 Q 	   688.33	  1358.69	 13
  7	 S 	   775.37	  1230.63	 12
  8	 P 	   872.42	  1143.60	 11
  9	 D 	   987.45	  1046.55	 10
 10	 S 	  1074.48	   931.52	  9
 11	 L 	  1187.56	   844.49	  8
 12	 A 	  1258.60	   731.40	  7
 13	 V 	  1357.67	   660.37	  6
 14	 S 	  1444.70	   561.30	  5
 15	 L 	  1557.78	   474.27	  4
 16	 G 	  1614.80	   361.18	  3
 17	 E 	  1743.85	   304.16	  2
 18	 R 	        -  	   175.12	  1  _
+2 charge
		      B   	       Y
  1	 D 	    58.52	        -  	 18
  2	 I 	   115.06	   901.97	 17
  3	 V 	   164.60	   845.43	 16
  4	 M 	   230.12	   795.89	 15
  5	 T 	   280.64	   730.37	 14
  6	 Q 	   344.67	   679.85	 13
  7	 S 	   388.19	   615.82	 12
  8	 P 	   436.71	   572.30	 11
  9	 D 	   494.23	   523.78	 10
 10	 S 	   537.74	   466.26	  9
 11	 L 	   594.28	   422.75	  8
 12	 A 	   629.80	   366.21	  7
 13	 V 	   679.34	   330.69	  6
 14	 S 	   722.85	   281.15	  5
 15	 L 	   779.40	   237.64	  4
 16	 G 	   807.91	   181.10	  3
 17	 E 	   872.43	   152.58	  2
 18	 R 	        -  	    88.06	  1  _
```

**91. IPI00736885 Ig kappa chain V-II region TEW**

| Sequence | Charge | M+H+ | XCorr | DeltaCn |
| --- | --- | --- | --- | --- |
| - .DIVMTQSPLSLPVTPGEPASISCR.S | 3 | 2556.911 | 4.986 | 0.47 |

+1 charge

```
		      B   	       Y
  1	 D 	   116.03	        -  	 24
  2	 I 	   229.12	  2440.39	 23
  3	 V 	   328.19	  2327.31	 22
  4	 M 	   459.23	  2228.24	 21
  5	 T 	   560.27	  2097.20	 20
  6	 Q 	   688.33	  1996.15	 19
  7	 S 	   775.37	  1868.09	 18
  8	 P 	   872.42	  1781.06	 17
  9	 L 	   985.50	  1684.01	 16
 10	 S 	  1072.53	  1570.92	 15
 11	 L 	  1185.62	  1483.89	 14
 12	 P 	  1282.67	  1370.81	 13
 13	 V 	  1381.74	  1273.76	 12
 14	 T 	  1482.79	  1174.69	 11
 15	 P 	  1579.84	  1073.64	 10
 16	 G 	  1636.86	   976.59	  9
 17	 E 	  1765.90	   919.56	  8
 18	 P 	  1862.96	   790.52	  7
 19	 A 	  1933.99	   693.47	  6
 20	 S 	  2021.03	   622.43	  5
 21	 I 	  2134.11	   535.40	  4
 22	 S 	  2221.14	   422.32	  3
 23	 C 	  2381.31	   335.28	  2
 24	 R 	        -  	   175.12	  1  _


+2 charge
		      B   	       Y
  1	 D 	    58.52	        -  	 24
  2	 I 	   115.06	  1220.70	 23
  3	 V 	   164.60	  1164.16	 22
  4	 M 	   230.12	  1114.62	 21
  5	 T 	   280.64	  1049.10	 20
  6	 Q 	   344.67	   998.58	 19
  7	 S 	   388.19	   934.55	 18
  8	 P 	   436.71	   891.03	 17
  9	 L 	   493.25	   842.51	 16
 10	 S 	   536.77	   785.97	 15
 11	 L 	   593.31	   742.45	 14
 12	 P 	   641.84	   685.91	 13
 13	 V 	   691.37	   637.38	 12
 14	 T 	   741.90	   587.85	 11
 15	 P 	   790.42	   537.32	 10
 16	 G 	   818.93	   488.80	  9
 17	 E 	   883.46	   460.29	  8
 18	 P 	   931.98	   395.76	  7
 19	 A 	   967.50	   347.24	  6
 20	 S 	  1011.02	   311.72	  5
 21	 I 	  1067.56	   268.20	  4
 22	 S 	  1111.07	   211.66	  3
 23	 C 	  1191.16	   168.15	  2
 24	 R 	        -  	    88.06	  1  _


+3 charge
		      B   	       Y
  1	 D 	    39.35	        -  	 24
  2	 I 	    77.04	   814.14	 23
  3	 V 	   110.07	   776.44	 22
  4	 M 	   153.75	   743.42	 21
  5	 T 	   187.43	   699.74	 20
  6	 Q 	   230.12	   666.06	 19
  7	 S 	   259.13	   623.37	 18
  8	 P 	   291.48	   594.36	 17
  9	 L 	   329.17	   562.01	 16
 10	 S 	   358.18	   524.31	 15
 11	 L 	   395.88	   495.30	 14
 12	 P 	   428.23	   457.61	 13
 13	 V 	   461.25	   425.26	 12
 14	 T 	   494.93	   392.23	 11
 15	 P 	   527.28	   358.55	 10
 16	 G 	   546.29	   326.20	  9
 17	 E 	   589.31	   307.19	  8
 18	 P 	   621.66	   264.18	  7
 19	 A 	   645.34	   231.83	  6
 20	 S 	   674.35	   208.15	  5
 21	 I 	   712.04	   179.14	  4
 22	 S 	   741.05	   141.44	  3
 23	 C 	   794.44	   112.43	  2
 24	 R 	        -  	    59.04	  1  _
```

**92. IPI00930263 cDNA FLJ61099, highly similar to ADP-ribosylation factor 1**

| Sequence | Charge | M+H+ | XCorr | DeltaCn |
| --- | --- | --- | --- | --- |
| K.QDLPNAMNAAGITDKLGLHSLR.H | 3 | 2336.657 | 3.33 | 0.31 |

+1 charge

```
		      B   	       Y
  1	 Q 	   129.07	        -  	 22
  2	 D 	   244.09	  2207.16	 21
  3	 L 	   357.18	  2092.13	 20
  4	 P 	   454.23	  1979.05	 19
  5	 N 	   568.27	  1882.00	 18
  6	 A 	   639.31	  1767.95	 17
  7	 M 	   770.35	  1696.92	 16
  8	 N 	   884.39	  1565.88	 15
  9	 A 	   955.43	  1451.83	 14
 10	 A 	  1026.47	  1380.80	 13
 11	 G 	  1083.49	  1309.76	 12
 12	 I 	  1196.57	  1252.74	 11
 13	 T 	  1297.62	  1139.65	 10
 14	 D 	  1412.65	  1038.61	  9
 15	 K 	  1540.74	   923.58	  8
 16	 L 	  1653.83	   795.48	  7
 17	 G 	  1710.85	   682.40	  6
 18	 L 	  1823.93	   625.38	  5
 19	 H 	  1960.99	   512.29	  4
 20	 S 	  2048.02	   375.24	  3
 21	 L 	  2161.11	   288.20	  2
 22	 R 	        -  	   175.12	  1  _


+2 charge
		      B   	       Y
  1	 Q 	    65.04	        -  	 22
  2	 D 	   122.55	  1104.08	 21
  3	 L 	   179.09	  1046.57	 20
  4	 P 	   227.62	   990.03	 19
  5	 N 	   284.64	   941.50	 18
  6	 A 	   320.16	   884.48	 17
  7	 M 	   385.68	   848.96	 16
  8	 N 	   442.70	   783.44	 15
  9	 A 	   478.22	   726.42	 14
 10	 A 	   513.74	   690.90	 13
 11	 G 	   542.25	   655.38	 12
 12	 I 	   598.79	   626.87	 11
 13	 T 	   649.31	   570.33	 10
 14	 D 	   706.83	   519.81	  9
 15	 K 	   770.87	   462.29	  8
 16	 L 	   827.42	   398.25	  7
 17	 G 	   855.93	   341.70	  6
 18	 L 	   912.47	   313.19	  5
 19	 H 	   981.00	   256.65	  4
 20	 S 	  1024.52	   188.12	  3
 21	 L 	  1081.06	   144.61	  2
 22	 R 	        -  	    88.06	  1  _
+3 charge
		      B   	       Y
  1	 Q 	    43.69	        -  	 22
  2	 D 	    82.04	   736.39	 21
  3	 L 	   119.73	   698.05	 20
  4	 P 	   152.08	   660.35	 19
  5	 N 	   190.10	   628.00	 18
  6	 A 	   213.77	   589.99	 17
  7	 M 	   257.45	   566.31	 16
  8	 N 	   295.47	   522.63	 15
  9	 A 	   319.15	   484.62	 14
 10	 A 	   342.83	   460.94	 13
 11	 G 	   361.83	   437.26	 12
 12	 I 	   399.53	   418.25	 11
 13	 T 	   433.21	   380.56	 10
 14	 D 	   471.55	   346.87	  9
 15	 K 	   514.25	   308.53	  8
 16	 L 	   551.95	   265.83	  7
 17	 G 	   570.95	   228.14	  6
 18	 L 	   608.65	   209.13	  5
 19	 H 	   654.34	   171.44	  4
 20	 S 	   683.35	   125.75	  3
 21	 L 	   721.04	    96.74	  2
 22	 R 	        -  	    59.04	  1  _
```

**93. IPI00066620 Isoform 1 of Uncharacterized protein C16orf46**

| Sequence | Charge | M+H+ | XCorr | DeltaCn |
| --- | --- | --- | --- | --- |
| K.DWAIPGTNRGKASGNPSGGAHR.G | 3 | 2207.355 | 3.316 | 0.337 |

+1 charge

```
		      B   	       Y
  1	 D 	   116.03	        -  	 22
  2	 W 	   302.11	  2091.06	 21
  3	 A 	   373.15	  1904.98	 20
  4	 I 	   486.23	  1833.94	 19
  5	 P 	   583.29	  1720.86	 18
  6	 G 	   640.31	  1623.81	 17
  7	 T 	   741.36	  1566.78	 16
  8	 N 	   855.40	  1465.74	 15
  9	 R 	  1011.50	  1351.69	 14
 10	 G 	  1068.52	  1195.59	 13
 11	 K 	  1196.62	  1138.57	 12
 12	 A 	  1267.65	  1010.48	 11
 13	 S 	  1354.69	   939.44	 10
 14	 G 	  1411.71	   852.41	  9
 15	 N 	  1525.75	   795.39	  8
 16	 P 	  1622.80	   681.34	  7
 17	 S 	  1709.84	   584.29	  6
 18	 G 	  1766.86	   497.26	  5
 19	 G 	  1823.88	   440.24	  4
 20	 A 	  1894.92	   383.21	  3
 21	 H 	  2031.97	   312.18	  2
 22	 R 	        -  	   175.12	  1  _


+2 charge
		      B   	       Y
  1	 D 	    58.52	        -  	 22
  2	 W 	   151.56	  1046.03	 21
  3	 A 	   187.08	   952.99	 20
  4	 I 	   243.62	   917.47	 19
  5	 P 	   292.15	   860.93	 18
  6	 G 	   320.66	   812.41	 17
  7	 T 	   371.18	   783.90	 16
  8	 N 	   428.20	   733.37	 15
  9	 R 	   506.25	   676.35	 14
 10	 G 	   534.76	   598.30	 13
 11	 K 	   598.81	   569.79	 12
 12	 A 	   634.33	   505.74	 11
 13	 S 	   677.85	   470.22	 10
 14	 G 	   706.36	   426.71	  9
 15	 N 	   763.38	   398.20	  8
 16	 P 	   811.91	   341.17	  7
 17	 S 	   855.42	   292.65	  6
 18	 G 	   883.93	   249.13	  5
 19	 G 	   912.44	   220.62	  4
 20	 A 	   947.96	   192.11	  3
 21	 H 	  1016.49	   156.59	  2
 22	 R 	        -  	    88.06	  1  _
+3 charge
		      B   	       Y
  1	 D 	    39.35	        -  	 22
  2	 W 	   101.38	   697.69	 21
  3	 A 	   125.06	   635.66	 20
  4	 I 	   162.75	   611.99	 19
  5	 P 	   195.10	   574.29	 18
  6	 G 	   214.11	   541.94	 17
  7	 T 	   247.79	   522.93	 16
  8	 N 	   285.80	   489.25	 15
  9	 R 	   337.84	   451.24	 14
 10	 G 	   356.85	   399.20	 13
 11	 K 	   399.54	   380.20	 12
 12	 A 	   423.22	   337.50	 11
 13	 S 	   452.23	   313.82	 10
 14	 G 	   471.24	   284.81	  9
 15	 N 	   509.26	   265.80	  8
 16	 P 	   541.61	   227.79	  7
 17	 S 	   570.62	   195.43	  6
 18	 G 	   589.62	   166.42	  5
 19	 G 	   608.63	   147.42	  4
 20	 A 	   632.31	   128.41	  3
 21	 H 	   678.00	   104.73	  2
 22	 R 	        -  	    59.04	  1  _
```

**94. IPI00297252 Isoform 1 of Extracellular sulfatase Sulf-2**

| Sequence | Charge | M+H+ | XCorr | DeltaCn |
| --- | --- | --- | --- | --- |
| R.VYHVGLGDAAQPR.N | 3 | 1383.537 | 4.51 | 0.318 |

+1 charge

```
		      B   	       Y
  1	 V 	   100.08	        -  	 13
  2	 Y 	   263.14	  1283.65	 12
  3	 H 	   400.20	  1120.59	 11
  4	 V 	   499.27	   983.53	 10
  5	 G 	   556.29	   884.46	  9
  6	 L 	   669.37	   827.44	  8
  7	 G 	   726.39	   714.35	  7
  8	 D 	   841.42	   657.33	  6
  9	 A 	   912.46	   542.30	  5
 10	 A 	   983.49	   471.27	  4
 11	 Q 	  1111.55	   400.23	  3
 12	 P 	  1208.61	   272.17	  2
 13	 R 	        -  	   175.12	  1  _
+2 charge
		      B   	       Y
  1	 V 	    50.54	        -  	 13
  2	 Y 	   132.07	   642.33	 12
  3	 H 	   200.60	   560.80	 11
  4	 V 	   250.14	   492.27	 10
  5	 G 	   278.65	   442.73	  9
  6	 L 	   335.19	   414.22	  8
  7	 G 	   363.70	   357.68	  7
  8	 D 	   421.21	   329.17	  6
  9	 A 	   456.73	   271.66	  5
 10	 A 	   492.25	   236.14	  4
 11	 Q 	   556.28	   200.62	  3
 12	 P 	   604.81	   136.59	  2
 13	 R 	        -  	    88.06	  1  _
+3 charge
		      B   	       Y
  1	 V 	    34.03	        -  	 13
  2	 Y 	    88.38	   428.55	 12
  3	 H 	   134.07	   374.20	 11
  4	 V 	   167.09	   328.51	 10
  5	 G 	   186.10	   295.49	  9
  6	 L 	   223.80	   276.48	  8
  7	 G 	   242.80	   238.79	  7
  8	 D 	   281.14	   219.78	  6
  9	 A 	   304.82	   181.44	  5
 10	 A 	   328.50	   157.76	  4
 11	 Q 	   371.19	   134.08	  3
 12	 P 	   403.54	    91.40	  2
 13	 R 	        -  	    59.04	  1  _
```

**95. IPI00009793 Complement C1r subcomponent-like protein**

| Sequence | Charge | M+H+ | XCorr | DeltaCn |
| --- | --- | --- | --- | --- |
| K.GQESSTDIKAPEGFAVR.L | 2 | 1792.928 | 3.062 | 0.45 |

+1 charge

```
		      B   	       Y
  1	 G 	    58.03	        -  	 17
  2	 Q 	   186.09	  1734.87	 16
  3	 E 	   315.13	  1606.81	 15
  4	 S 	   402.16	  1477.76	 14
  5	 S 	   489.19	  1390.73	 13
  6	 T 	   590.24	  1303.70	 12
  7	 D 	   705.27	  1202.65	 11
  8	 I 	   818.35	  1087.63	 10
  9	 K 	   946.45	   974.54	  9
 10	 A 	  1017.48	   846.45	  8
 11	 P 	  1114.54	   775.41	  7
 12	 E 	  1243.58	   678.36	  6
 13	 G 	  1300.60	   549.31	  5
 14	 F 	  1447.67	   492.29	  4
 15	 A 	  1518.71	   345.22	  3
 16	 V 	  1617.78	   274.19	  2
 17	 R 	        -  	   175.12	  1  _
+2 charge
		      B   	       Y
  1	 G 	    29.52	        -  	 17
  2	 Q 	    93.55	   867.94	 16
  3	 E 	   158.07	   803.91	 15
  4	 S 	   201.58	   739.39	 14
  5	 S 	   245.10	   695.87	 13
  6	 T 	   295.62	   652.35	 12
  7	 D 	   353.14	   601.83	 11
  8	 I 	   409.68	   544.32	 10
  9	 K 	   473.73	   487.77	  9
 10	 A 	   509.25	   423.73	  8
 11	 P 	   557.77	   388.21	  7
 12	 E 	   622.29	   339.68	  6
 13	 G 	   650.80	   275.16	  5
 14	 F 	   724.34	   246.65	  4
 15	 A 	   759.86	   173.12	  3
 16	 V 	   809.39	   137.60	  2
 17	 R 	        -  	    88.06	  1  _
```

**96. IPI00011140 Protein NOV homolog**

| Sequence | Charge | M+H+ | XCorr | DeltaCn |
| --- | --- | --- | --- | --- |
| R.AVLDGCSCCLVCAR.Q | 2 | 1641.823 | 3.894 | 0.582 |

+1 charge

```
		      B   	       Y
  1	 A 	    72.04	        -  	 14
  2	 V 	   171.11	  1570.20	 13
  3	 L 	   284.20	  1471.13	 12
  4	 D 	   399.22	  1358.05	 11
  5	 G 	   456.25	  1243.02	 10
  6	 C 	   616.41	  1186.00	  9
  7	 S 	   703.44	  1025.84	  8
  8	 C 	   863.61	   938.80	  7
  9	 C 	  1023.77	   778.64	  6
 10	 L 	  1136.86	   618.47	  5
 11	 V 	  1235.93	   505.39	  4
 12	 C 	  1396.09	   406.32	  3
 13	 A 	  1467.13	   246.16	  2
 14	 R 	        -  	   175.12	  1  _
+2 charge
		      B   	       Y
  1	 A 	    36.53	        -  	 14
  2	 V 	    86.06	   785.60	 13
  3	 L 	   142.60	   736.07	 12
  4	 D 	   200.12	   679.53	 11
  5	 G 	   228.63	   622.02	 10
  6	 C 	   308.71	   593.50	  9
  7	 S 	   352.22	   513.42	  8
  8	 C 	   432.31	   469.91	  7
  9	 C 	   512.39	   389.82	  6
 10	 L 	   568.93	   309.74	  5
 11	 V 	   618.47	   253.20	  4
 12	 C 	   698.55	   203.66	  3
 13	 A 	   734.07	   123.58	  2
 14	 R 	        -  	    88.06	  1  _
```

**97. IPI00026944 Isoform 1 of Nidogen-1**

| Sequence | Charge | M+H+ | XCorr | DeltaCn |
| --- | --- | --- | --- | --- |
| R.RGGADTYSVPSVLSPR.R | 2 | 1662.829 | 3.185 | 0.348 |

+1 charge

```
		      B   	       Y
  1	 R 	   157.11	        -  	 16
  2	 G 	   214.13	  1505.76	 15
  3	 G 	   271.15	  1448.74	 14
  4	 A 	   342.19	  1391.72	 13
  5	 D 	   457.22	  1320.68	 12
  6	 T 	   558.26	  1205.65	 11
  7	 Y 	   721.33	  1104.60	 10
  8	 S 	   808.36	   941.54	  9
  9	 V 	   907.43	   854.51	  8
 10	 P 	  1004.48	   755.44	  7
 11	 S 	  1091.51	   658.39	  6
 12	 V 	  1190.58	   571.36	  5
 13	 L 	  1303.66	   472.29	  4
 14	 S 	  1390.70	   359.20	  3
 15	 P 	  1487.75	   272.17	  2
 16	 R 	        -  	   175.12	  1  _
+2 charge
		      B   	       Y
  1	 R 	    79.06	        -  	 16
  2	 G 	   107.57	   753.38	 15
  3	 G 	   136.08	   724.87	 14
  4	 A 	   171.60	   696.36	 13
  5	 D 	   229.11	   660.84	 12
  6	 T 	   279.64	   603.33	 11
  7	 Y 	   361.17	   552.81	 10
  8	 S 	   404.68	   471.27	  9
  9	 V 	   454.22	   427.76	  8
 10	 P 	   502.74	   378.22	  7
 11	 S 	   546.26	   329.70	  6
 12	 V 	   595.79	   286.18	  5
 13	 L 	   652.34	   236.65	  4
 14	 S 	   695.85	   180.11	  3
 15	 P 	   744.38	   136.59	  2
 16	 R 	        -  	    88.06	  1  _
```

**98. IPI00178926 Immunoglobulin J chain**

| Sequence | Charge | M+H+ | XCorr | DeltaCn |
| --- | --- | --- | --- | --- |
| R.FVYHLSDLCK.K | 2 | 1282.463 | 3.149 | 0.463 |

+1 charge

```
		      B   	       Y
  1	 F 	   148.08	        -  	 10
  2	 V 	   247.14	  1134.70	  9
  3	 Y 	   410.21	  1035.63	  8
  4	 H 	   547.27	   872.56	  7
  5	 L 	   660.35	   735.51	  6
  6	 S 	   747.38	   622.42	  5
  7	 D 	   862.41	   535.39	  4
  8	 L 	   975.49	   420.36	  3
  9	 C 	  1135.66	   307.28	  2
 10	 K 	        -  	   147.11	  1  _
+2 charge
		      B   	       Y
  1	 F 	    74.54	        -  	 10
  2	 V 	   124.08	   567.85	  9
  3	 Y 	   205.61	   518.32	  8
  4	 H 	   274.14	   436.79	  7
  5	 L 	   330.68	   368.26	  6
  6	 S 	   374.19	   311.71	  5
  7	 D 	   431.71	   268.20	  4
  8	 L 	   488.25	   210.68	  3
  9	 C 	   568.33	   154.14	  2
 10	 K 	        -  	    74.06	  1  _
```

**99. IPI00218407 Fructose-bisphosphate aldolase B**

| Sequence | Charge | M+H+ | XCorr | DeltaCn |
| --- | --- | --- | --- | --- |
| R.IADQCPSSLAIQENANALAR.Y | 2 | 2143.337 | 5.413 | 0.428 |

+1 charge

```
		      B   	       Y
  1	 I 	   114.09	        -  	 20
  2	 A 	   185.13	  2029.11	 19
  3	 D 	   300.16	  1958.07	 18
  4	 Q 	   428.21	  1843.05	 17
  5	 C 	   588.38	  1714.99	 16
  6	 P 	   685.43	  1554.82	 15
  7	 S 	   772.46	  1457.77	 14
  8	 S 	   859.50	  1370.74	 13
  9	 L 	   972.58	  1283.71	 12
 10	 A 	  1043.62	  1170.62	 11
 11	 I 	  1156.70	  1099.59	 10
 12	 Q 	  1284.76	   986.50	  9
 13	 E 	  1413.80	   858.44	  8
 14	 N 	  1527.85	   729.40	  7
 15	 A 	  1598.88	   615.36	  6
 16	 N 	  1712.93	   544.32	  5
 17	 A 	  1783.96	   430.28	  4
 18	 L 	  1897.05	   359.24	  3
 19	 A 	  1968.08	   246.16	  2
 20	 R 	        -  	   175.12	  1  _
+2 charge
		      B   	       Y
  1	 I 	    57.55	        -  	 20
  2	 A 	    93.07	  1015.06	 19
  3	 D 	   150.58	   979.54	 18
  4	 Q 	   214.61	   922.03	 17
  5	 C 	   294.69	   858.00	 16
  6	 P 	   343.22	   777.92	 15
  7	 S 	   386.74	   729.39	 14
  8	 S 	   430.25	   685.87	 13
  9	 L 	   486.79	   642.36	 12
 10	 A 	   522.31	   585.81	 11
 11	 I 	   578.85	   550.30	 10
 12	 Q 	   642.88	   493.75	  9
 13	 E 	   707.40	   429.73	  8
 14	 N 	   764.43	   365.20	  7
 15	 A 	   799.94	   308.18	  6
 16	 N 	   856.97	   272.66	  5
 17	 A 	   892.48	   215.64	  4
 18	 L 	   949.03	   180.12	  3
 19	 A 	   984.55	   123.58	  2
 20	 R 	        -  	    88.06	  1  _
```

**100. IPI00018901 Isoform 1 of Gamma-glutamyltranspeptidase 1**

| Sequence | Charge | M+H+ | XCorr | DeltaCn |
| --- | --- | --- | --- | --- |
| R.LFQPSIQLAR.Q | 2 | 1173.39 | 2.811 | 0.251 |

+1 charge

```
		      B   	       Y
  1	 L 	   114.09	        -  	 10
  2	 F 	   261.16	  1059.59	  9
  3	 Q 	   389.22	   912.53	  8
  4	 P 	   486.27	   784.47	  7
  5	 S 	   573.30	   687.41	  6
  6	 I 	   686.39	   600.38	  5
  7	 Q 	   814.45	   487.30	  4
  8	 L 	   927.53	   359.24	  3
  9	 A 	   998.57	   246.16	  2
 10	 R 	        -  	   175.12	  1  _
+2 charge
		      B   	       Y
  1	 L 	    57.55	        -  	 10
  2	 F 	   131.08	   530.30	  9
  3	 Q 	   195.11	   456.77	  8
  4	 P 	   243.64	   392.74	  7
  5	 S 	   287.16	   344.21	  6
  6	 I 	   343.70	   300.70	  5
  7	 Q 	   407.73	   244.15	  4
  8	 L 	   464.27	   180.12	  3
  9	 A 	   499.79	   123.58	  2
 10	 R 	        -  	    88.06	  1  _
```

**101. IPI00293088 Lysosomal alpha-glucosidase**

| Sequence | Charge | M+H+ | XCorr | DeltaCn |
| --- | --- | --- | --- | --- |
| R.AGYIIPLQGPGLTTTESR.Q | 2 | 1875.115 | 4.671 | 0.595 |

+1 charge

```
		      B   	       Y
  1	 A 	    72.04	        -  	 18
  2	 G 	   129.07	  1802.96	 17
  3	 Y 	   292.13	  1745.94	 16
  4	 I 	   405.21	  1582.88	 15
  5	 I 	   518.30	  1469.80	 14
  6	 P 	   615.35	  1356.71	 13
  7	 L 	   728.43	  1259.66	 12
  8	 Q 	   856.49	  1146.57	 11
  9	 G 	   913.51	  1018.52	 10
 10	 P 	  1010.57	   961.49	  9
 11	 G 	  1067.59	   864.44	  8
 12	 L 	  1180.67	   807.42	  7
 13	 T 	  1281.72	   694.34	  6
 14	 T 	  1382.77	   593.29	  5
 15	 T 	  1483.82	   492.24	  4
 16	 E 	  1612.86	   391.19	  3
 17	 S 	  1699.89	   262.15	  2
 18	 R 	        -  	   175.12	  1  _
+2 charge
		      B   	       Y
  1	 A 	    36.53	        -  	 18
  2	 G 	    65.04	   901.99	 17
  3	 Y 	   146.57	   873.48	 16
  4	 I 	   203.11	   791.94	 15
  5	 I 	   259.65	   735.40	 14
  6	 P 	   308.18	   678.86	 13
  7	 L 	   364.72	   630.33	 12
  8	 Q 	   428.75	   573.79	 11
  9	 G 	   457.26	   509.76	 10
 10	 P 	   505.79	   481.25	  9
 11	 G 	   534.30	   432.72	  8
 12	 L 	   590.84	   404.21	  7
 13	 T 	   641.36	   347.67	  6
 14	 T 	   691.89	   297.15	  5
 15	 T 	   742.41	   246.62	  4
 16	 E 	   806.93	   196.10	  3
 17	 S 	   850.45	   131.58	  2
 18	 R 	        -  	    88.06	  1  _
```

**102. IPI00025846 Isoform 2A of Desmocollin-2**

| Sequence | Charge | M+H+ | XCorr | DeltaCn |
| --- | --- | --- | --- | --- |
| R.LSYQNDPPFGSYVVPITVR.D | 2 | 2153.422 | 3.246 | 0.391 |

+1 charge

```
		      B   	       Y
  1	 L 	   114.09	        -  	 19
  2	 S 	   201.12	  2039.02	 18
  3	 Y 	   364.19	  1951.99	 17
  4	 Q 	   492.25	  1788.93	 16
  5	 N 	   606.29	  1660.87	 15
  6	 D 	   721.32	  1546.83	 14
  7	 P 	   818.37	  1431.80	 13
  8	 P 	   915.42	  1334.75	 12
  9	 F 	  1062.49	  1237.69	 11
 10	 G 	  1119.51	  1090.63	 10
 11	 S 	  1206.54	  1033.60	  9
 12	 Y 	  1369.61	   946.57	  8
 13	 V 	  1468.67	   783.51	  7
 14	 V 	  1567.74	   684.44	  6
 15	 P 	  1664.80	   585.37	  5
 16	 I 	  1777.88	   488.32	  4
 17	 T 	  1878.93	   375.24	  3
 18	 V 	  1978.00	   274.19	  2
 19	 R 	        -  	   175.12	  1  _
+2 charge
		      B   	       Y
  1	 L 	    57.55	        -  	 19
  2	 S 	   101.07	  1020.02	 18
  3	 Y 	   182.60	   976.50	 17
  4	 Q 	   246.63	   894.97	 16
  5	 N 	   303.65	   830.94	 15
  6	 D 	   361.16	   773.92	 14
  7	 P 	   409.69	   716.40	 13
  8	 P 	   458.21	   667.88	 12
  9	 F 	   531.75	   619.35	 11
 10	 G 	   560.26	   545.82	 10
 11	 S 	   603.77	   517.31	  9
 12	 Y 	   685.31	   473.79	  8
 13	 V 	   734.84	   392.26	  7
 14	 V 	   784.38	   342.72	  6
 15	 P 	   832.90	   293.19	  5
 16	 I 	   889.44	   244.66	  4
 17	 T 	   939.97	   188.12	  3
 18	 V 	   989.50	   137.60	  2
 19	 R 	        -  	    88.06	  1  _
```

**103. IPI00304808 Isoform 1 of Kallikrein-1**

| Sequence | Charge | M+H+ | XCorr | DeltaCn |
| --- | --- | --- | --- | --- |
| R.LTEPADTITDAVK.V | 2 | 1374.519 | 3.712 | 0.495 |

+1 charge

```
		      B   	       Y
  1	 L 	   114.09	        -  	 13
  2	 T 	   215.14	  1260.63	 12
  3	 E 	   344.18	  1159.58	 11
  4	 P 	   441.23	  1030.54	 10
  5	 A 	   512.27	   933.49	  9
  6	 D 	   627.30	   862.45	  8
  7	 T 	   728.35	   747.42	  7
  8	 I 	   841.43	   646.38	  6
  9	 T 	   942.48	   533.29	  5
 10	 D 	  1057.50	   432.25	  4
 11	 A 	  1128.54	   317.22	  3
 12	 V 	  1227.61	   246.18	  2
 13	 K 	        -  	   147.11	  1  _
+2 charge
		      B   	       Y
  1	 L 	    57.55	        -  	 13
  2	 T 	   108.07	   630.82	 12
  3	 E 	   172.59	   580.30	 11
  4	 P 	   221.12	   515.77	 10
  5	 A 	   256.64	   467.25	  9
  6	 D 	   314.15	   431.73	  8
  7	 T 	   364.68	   374.22	  7
  8	 I 	   421.22	   323.69	  6
  9	 T 	   471.74	   267.15	  5
 10	 D 	   529.26	   216.63	  4
 11	 A 	   564.77	   159.11	  3
 12	 V 	   614.31	   123.59	  2
 13	 K 	        -  	    74.06	  1  _
```

**104. IPI00003111 Ig kappa chain V-I region AU**

| Sequence | Charge | M+H+ | XCorr | DeltaCn |
| --- | --- | --- | --- | --- |
| K.LLIYDASNLETGVPSR.F | 2 | 1748.958 | 3.057 | 0.222 |

+1 charge

```
		      B   	       Y
  1	 L 	   114.09	        -  	 16
  2	 L 	   227.18	  1634.84	 15
  3	 I 	   340.26	  1521.75	 14
  4	 Y 	   503.32	  1408.67	 13
  5	 D 	   618.35	  1245.61	 12
  6	 A 	   689.39	  1130.58	 11
  7	 S 	   776.42	  1059.54	 10
  8	 N 	   890.46	   972.51	  9
  9	 L 	  1003.55	   858.47	  8
 10	 E 	  1132.59	   745.38	  7
 11	 T 	  1233.64	   616.34	  6
 12	 G 	  1290.66	   515.29	  5
 13	 V 	  1389.73	   458.27	  4
 14	 P 	  1486.78	   359.20	  3
 15	 S 	  1573.81	   262.15	  2
 16	 R 	        -  	   175.12	  1  _
+2 charge
		      B   	       Y
  1	 L 	    57.55	        -  	 16
  2	 L 	   114.09	   817.92	 15
  3	 I 	   170.63	   761.38	 14
  4	 Y 	   252.17	   704.84	 13
  5	 D 	   309.68	   623.31	 12
  6	 A 	   345.20	   565.79	 11
  7	 S 	   388.71	   530.28	 10
  8	 N 	   445.73	   486.76	  9
  9	 L 	   502.28	   429.74	  8
 10	 E 	   566.80	   373.20	  7
 11	 T 	   617.32	   308.67	  6
 12	 G 	   645.83	   258.15	  5
 13	 V 	   695.37	   229.64	  4
 14	 P 	   743.89	   180.11	  3
 15	 S 	   787.41	   131.58	  2
 16	 R 	        -  	    88.06	  1  _
```

**105. IPI00043740 Isoform 1 of Sodium channel and clathrin linker 1**

| Sequence | Charge | M+H+ | XCorr | DeltaCn |
| --- | --- | --- | --- | --- |
| K.LHTQELLSQLEMANEK.V | 2 | 1885.132 | 3.551 | 0.209 |

+1 charge

```
		      B   	       Y
  1	 L 	   114.09	        -  	 16
  2	 H 	   251.15	  1770.87	 15
  3	 T 	   352.20	  1633.81	 14
  4	 Q 	   480.26	  1532.76	 13
  5	 E 	   609.30	  1404.70	 12
  6	 L 	   722.38	  1275.66	 11
  7	 L 	   835.47	  1162.58	 10
  8	 S 	   922.50	  1049.49	  9
  9	 Q 	  1050.56	   962.46	  8
 10	 L 	  1163.64	   834.40	  7
 11	 E 	  1292.68	   721.32	  6
 12	 M 	  1423.72	   592.28	  5
 13	 A 	  1494.76	   461.24	  4
 14	 N 	  1608.81	   390.20	  3
 15	 E 	  1737.85	   276.16	  2
 16	 K 	        -  	   147.11	  1  _
+2 charge
		      B   	       Y
  1	 L 	    57.55	        -  	 16
  2	 H 	   126.08	   885.94	 15
  3	 T 	   176.60	   817.41	 14
  4	 Q 	   240.63	   766.88	 13
  5	 E 	   305.15	   702.86	 12
  6	 L 	   361.70	   638.33	 11
  7	 L 	   418.24	   581.79	 10
  8	 S 	   461.75	   525.25	  9
  9	 Q 	   525.78	   481.73	  8
 10	 L 	   582.32	   417.70	  7
 11	 E 	   646.85	   361.16	  6
 12	 M 	   712.37	   296.64	  5
 13	 A 	   747.88	   231.12	  4
 14	 N 	   804.91	   195.60	  3
 15	 E 	   869.43	   138.58	  2
 16	 K 	        -  	    74.06	  1  _
```

**106. IPI00015199 T-cell antigen CD7**

| Sequence | Charge | M+H+ | XCorr | DeltaCn |
| --- | --- | --- | --- | --- |
| R.QLGPQPQDIIYYEDGVVPTTDRR.F | 3 | 2661.908 | 3.37 | 0.459 |

+1 charge

```
		      B   	       Y
  1	 Q 	   129.07	        -  	 23
  2	 L 	   242.15	  2532.27	 22
  3	 G 	   299.17	  2419.19	 21
  4	 P 	   396.22	  2362.17	 20
  5	 Q 	   524.28	  2265.11	 19
  6	 P 	   621.34	  2137.06	 18
  7	 Q 	   749.39	  2040.00	 17
  8	 D 	   864.42	  1911.94	 16
  9	 I 	   977.51	  1796.92	 15
 10	 I 	  1090.59	  1683.83	 14
 11	 Y 	  1253.65	  1570.75	 13
 12	 Y 	  1416.72	  1407.69	 12
 13	 E 	  1545.76	  1244.62	 11
 14	 D 	  1660.79	  1115.58	 10
 15	 G 	  1717.81	  1000.55	  9
 16	 V 	  1816.88	   943.53	  8
 17	 V 	  1915.94	   844.46	  7
 18	 P 	  2013.00	   745.40	  6
 19	 T 	  2114.04	   648.34	  5
 20	 T 	  2215.09	   547.29	  4
 21	 D 	  2330.12	   446.25	  3
 22	 R 	  2486.22	   331.22	  2
 23	 R 	        -  	   175.12	  1  _


+2 charge
		      B   	       Y
  1	 Q 	    65.04	        -  	 23
  2	 L 	   121.58	  1266.64	 22
  3	 G 	   150.09	  1210.10	 21
  4	 P 	   198.62	  1181.59	 20
  5	 Q 	   262.64	  1133.06	 19
  6	 P 	   311.17	  1069.03	 18
  7	 Q 	   375.20	  1020.51	 17
  8	 D 	   432.71	   956.48	 16
  9	 I 	   489.26	   898.96	 15
 10	 I 	   545.80	   842.42	 14
 11	 Y 	   627.33	   785.88	 13
 12	 Y 	   708.86	   704.35	 12
 13	 E 	   773.38	   622.82	 11
 14	 D 	   830.90	   558.29	 10
 15	 G 	   859.41	   500.78	  9
 16	 V 	   908.94	   472.27	  8
 17	 V 	   958.48	   422.74	  7
 18	 P 	  1007.00	   373.20	  6
 19	 T 	  1057.53	   324.67	  5
 20	 T 	  1108.05	   274.15	  4
 21	 D 	  1165.56	   223.63	  3
 22	 R 	  1243.61	   166.11	  2
 23	 R 	        -  	    88.06	  1  _
+3 charge
		      B   	       Y
  1	 Q 	    43.69	        -  	 23
  2	 L 	    81.39	   844.76	 22
  3	 G 	   100.40	   807.07	 21
  4	 P 	   132.75	   788.06	 20
  5	 Q 	   175.43	   755.71	 19
  6	 P 	   207.78	   713.02	 18
  7	 Q 	   250.47	   680.67	 17
  8	 D 	   288.81	   637.99	 16
  9	 I 	   326.51	   599.64	 15
 10	 I 	   364.20	   561.95	 14
 11	 Y 	   418.56	   524.25	 13
 12	 Y 	   472.91	   469.90	 12
 13	 E 	   515.92	   415.55	 11
 14	 D 	   554.27	   372.53	 10
 15	 G 	   573.27	   334.19	  9
 16	 V 	   606.30	   315.18	  8
 17	 V 	   639.32	   282.16	  7
 18	 P 	   671.67	   249.14	  6
 19	 T 	   705.35	   216.79	  5
 20	 T 	   739.04	   183.10	  4
 21	 D 	   777.38	   149.42	  3
 22	 R 	   829.41	   111.08	  2
 23	 R 	        -  	    59.04	  1  _
```

**107. IPI00291866 Plasma protease C1 inhibitor**

| Sequence | Charge | M+H+ | XCorr | DeltaCn |
| --- | --- | --- | --- | --- |
| K.GVTSVSQIFHSPDLAIR.D | 2 | 1828.062 | 3.463 | 0.456 |

+1 charge

```
		      B   	       Y
  1	 G 	    58.03	        -  	 17
  2	 V 	   157.10	  1769.95	 16
  3	 T 	   258.14	  1670.89	 15
  4	 S 	   345.18	  1569.84	 14
  5	 V 	   444.25	  1482.81	 13
  6	 S 	   531.28	  1383.74	 12
  7	 Q 	   659.34	  1296.71	 11
  8	 I 	   772.42	  1168.65	 10
  9	 F 	   919.49	  1055.56	  9
 10	 H 	  1056.55	   908.49	  8
 11	 S 	  1143.58	   771.44	  7
 12	 P 	  1240.63	   684.40	  6
 13	 D 	  1355.66	   587.35	  5
 14	 L 	  1468.74	   472.32	  4
 15	 A 	  1539.78	   359.24	  3
 16	 I 	  1652.86	   288.20	  2
 17	 R 	        -  	   175.12	  1  _
+2 charge
		      B   	       Y
  1	 G 	    29.52	        -  	 17
  2	 V 	    79.05	   885.48	 16
  3	 T 	   129.58	   835.95	 15
  4	 S 	   173.09	   785.42	 14
  5	 V 	   222.63	   741.91	 13
  6	 S 	   266.14	   692.37	 12
  7	 Q 	   330.17	   648.86	 11
  8	 I 	   386.71	   584.83	 10
  9	 F 	   460.25	   528.29	  9
 10	 H 	   528.78	   454.75	  8
 11	 S 	   572.29	   386.22	  7
 12	 P 	   620.82	   342.71	  6
 13	 D 	   678.33	   294.18	  5
 14	 L 	   734.88	   236.67	  4
 15	 A 	   770.39	   180.12	  3
 16	 I 	   826.94	   144.61	  2
 17	 R 	        -  	    88.06	  1  _
```

**108. IPI00414888 Isoform 2 of ICOS ligand**

| Sequence | Charge | M+H+ | XCorr | DeltaCn |
| --- | --- | --- | --- | --- |
| R.GLYDVVSVLR.I | 2 | 1121.311 | 3.791 | 0.368 |

+1 charge

```
		      B   	       Y
  1	 G 	    58.03	        -  	 10
  2	 L 	   171.11	  1063.61	  9
  3	 Y 	   334.18	   950.53	  8
  4	 D 	   449.20	   787.47	  7
  5	 V 	   548.27	   672.44	  6
  6	 V 	   647.34	   573.37	  5
  7	 S 	   734.37	   474.30	  4
  8	 V 	   833.44	   387.27	  3
  9	 L 	   946.52	   288.20	  2
 10	 R 	        -  	   175.12	  1  _
+2 charge
		      B   	       Y
  1	 G 	    29.52	        -  	 10
  2	 L 	    86.06	   532.31	  9
  3	 Y 	   167.59	   475.77	  8
  4	 D 	   225.11	   394.24	  7
  5	 V 	   274.64	   336.72	  6
  6	 V 	   324.17	   287.19	  5
  7	 S 	   367.69	   237.66	  4
  8	 V 	   417.22	   194.14	  3
  9	 L 	   473.77	   144.61	  2
 10	 R 	        -  	    88.06	  1  _
```

**Female 7.**

**1. IPI00639924 97 kDa protein**

| Sequence | Charge | M+H+ | XCorr | DeltaCn |
| --- | --- | --- | --- | --- |
| R.GLEDIVLVQLGDTRVTGMENGDGGGAR.S | 3 | 2730.993 | 3.312 | 0.261 |

+1 charge

```
		      B   	     Y
  1	 G 	    58.03	        -  	 27
  2	 L 	   171.11	  2672.33	 26
  3	 E 	   300.16	  2559.25	 25
  4	 D 	   415.18	  2430.20	 24
  5	 I 	   528.27	  2315.18	 23
  6	 V 	   627.33	  2202.09	 22
  7	 L 	   740.42	  2103.02	 21
  8	 V 	   839.49	  1989.94	 20
  9	 Q 	   967.55	  1890.87	 19
 10	 L 	  1080.63	  1762.81	 18
 11	 G 	  1137.65	  1649.73	 17
 12	 D 	  1252.68	  1592.71	 16
 13	 T 	  1353.73	  1477.68	 15
 14	 R 	  1509.83	  1376.63	 14
 15	 V 	  1608.90	  1220.53	 13
 16	 T 	  1709.94	  1121.46	 12
 17	 G 	  1766.96	  1020.42	 11
 18	 M 	  1898.01	   963.39	 10
 19	 E 	  2027.05	   832.35	  9
 20	 N 	  2141.09	   703.31	  8
 21	 G 	  2198.11	   589.27	  7
 22	 D 	  2313.14	   532.25	  6
 23	 G 	  2370.16	   417.22	  5
 24	 G 	  2427.18	   360.20	  4
 25	 G 	  2484.20	   303.18	  3
 26	 A 	  2555.24	   246.16	  2
 27	 R 	        -  	   175.12	  1  _


+2 charge
		      B   	     Y
  1	 G 	    29.52	        -  	 27
  2	 L 	    86.06	  1336.67	 26
  3	 E 	   150.58	  1280.13	 25
  4	 D 	   208.09	  1215.61	 24
  5	 I 	   264.64	  1158.09	 23
  6	 V 	   314.17	  1101.55	 22
  7	 L 	   370.71	  1052.02	 21
  8	 V 	   420.25	   995.47	 20
  9	 Q 	   484.28	   945.94	 19
 10	 L 	   540.82	   881.91	 18
 11	 G 	   569.33	   825.37	 17
 12	 D 	   626.84	   796.86	 16
 13	 T 	   677.37	   739.34	 15
 14	 R 	   755.42	   688.82	 14
 15	 V 	   804.95	   610.77	 13
 16	 T 	   855.48	   561.24	 12
 17	 G 	   883.99	   510.71	 11
 18	 M 	   949.51	   482.20	 10
 19	 E 	  1014.03	   416.68	  9
 20	 N 	  1071.05	   352.16	  8
 21	 G 	  1099.56	   295.14	  7
 22	 D 	  1157.07	   266.63	  6
 23	 G 	  1185.58	   209.11	  5
 24	 G 	  1214.09	   180.60	  4
 25	 G 	  1242.61	   152.09	  3
 26	 A 	  1278.12	   123.58	  2
 27	 R 	        -  	    88.06	  1  _


+3 charge
		      B   	     Y
  1	 G 	    20.01	        -  	 27
  2	 L 	    57.71	   891.45	 26
  3	 E 	   100.72	   853.75	 25
  4	 D 	   139.07	   810.74	 24
  5	 I 	   176.76	   772.40	 23
  6	 V 	   209.78	   734.70	 22
  7	 L 	   247.48	   701.68	 21
  8	 V 	   280.50	   663.99	 20
  9	 Q 	   323.19	   630.96	 19
 10	 L 	   360.88	   588.28	 18
 11	 G 	   379.89	   550.58	 17
 12	 D 	   418.23	   531.57	 16
 13	 T 	   451.91	   493.23	 15
 14	 R 	   503.95	   459.55	 14
 15	 V 	   536.97	   407.52	 13
 16	 T 	   570.65	   374.49	 12
 17	 G 	   589.66	   340.81	 11
 18	 M 	   633.34	   321.80	 10
 19	 E 	   676.35	   278.12	  9
 20	 N 	   714.37	   235.11	  8
 21	 G 	   733.38	   197.09	  7
 22	 D 	   771.72	   178.09	  6
 23	 G 	   790.73	   139.75	  5
 24	 G 	   809.73	   120.74	  4
 25	 G 	   828.74	   101.73	  3
 26	 A 	   852.42	    82.72	  2
 27	 R 	        -  	    59.04	  1  _
```

**2. IPI00297252 Isoform 1 of Extracellular sulfatase Sulf-2**

| Sequence | Charge | M+H+ | XCorr | DeltaCn |
| --- | --- | --- | --- | --- |
| R.VYHVGLGDAAQPR.N | 3 | 1383.537 | 3.974 | 0.391 |

+1 charge

```
		      B   	     Y
  1	 V 	   100.08	        -  	 13
  2	 Y 	   263.14	  1283.65	 12
  3	 H 	   400.20	  1120.59	 11
  4	 V 	   499.27	   983.53	 10
  5	 G 	   556.29	   884.46	  9
  6	 L 	   669.37	   827.44	  8
  7	 G 	   726.39	   714.35	  7
  8	 D 	   841.42	   657.33	  6
  9	 A 	   912.46	   542.30	  5
 10	 A 	   983.49	   471.27	  4
 11	 Q 	  1111.55	   400.23	  3
 12	 P 	  1208.61	   272.17	  2
 13	 R 	        -  	   175.12	  1  _
+2 charge
		      B   	     Y
  1	 V 	    50.54	        -  	 13
  2	 Y 	   132.07	   642.33	 12
  3	 H 	   200.60	   560.80	 11
  4	 V 	   250.14	   492.27	 10
  5	 G 	   278.65	   442.73	  9
  6	 L 	   335.19	   414.22	  8
  7	 G 	   363.70	   357.68	  7
  8	 D 	   421.21	   329.17	  6
  9	 A 	   456.73	   271.66	  5
 10	 A 	   492.25	   236.14	  4
 11	 Q 	   556.28	   200.62	  3
 12	 P 	   604.81	   136.59	  2
 13	 R 	        -  	    88.06	  1  _
+3 charge
		      B   	     Y
  1	 V 	    34.03	        -  	 13
  2	 Y 	    88.38	   428.55	 12
  3	 H 	   134.07	   374.20	 11
  4	 V 	   167.09	   328.51	 10
  5	 G 	   186.10	   295.49	  9
  6	 L 	   223.80	   276.48	  8
  7	 G 	   242.80	   238.79	  7
  8	 D 	   281.14	   219.78	  6
  9	 A 	   304.82	   181.44	  5
 10	 A 	   328.50	   157.76	  4
 11	 Q 	   371.19	   134.08	  3
 12	 P 	   403.54	    91.40	  2
 13	 R 	        -  	    59.04	  1  _
```

**3. IPI00290410 Deoxynucleotidyltransferase terminal-interacting protein 2**

| Sequence | Charge | M+H+ | XCorr | DeltaCn |
| --- | --- | --- | --- | --- |
| R.KRTIVEELLADSEFR.S | 2 | 1807.041 | 2.896 | 0.24 |

+1 charge

```
		      B   	     Y
  1	 K 	   129.10	        -  	 15
  2	 R 	   285.20	  1677.88	 14
  3	 T 	   386.25	  1521.78	 13
  4	 I 	   499.34	  1420.73	 12
  5	 V 	   598.40	  1307.65	 11
  6	 E 	   727.45	  1208.58	 10
  7	 E 	   856.49	  1079.54	  9
  8	 L 	   969.57	   950.49	  8
  9	 L 	  1082.66	   837.41	  7
 10	 A 	  1153.69	   724.33	  6
 11	 D 	  1268.72	   653.29	  5
 12	 S 	  1355.75	   538.26	  4
 13	 E 	  1484.80	   451.23	  3
 14	 F 	  1631.86	   322.19	  2
 15	 R 	        -  	   175.12	  1  _
+2 charge
		      B   	     Y
  1	 K 	    65.05	        -  	 15
  2	 R 	   143.11	   839.44	 14
  3	 T 	   193.63	   761.39	 13
  4	 I 	   250.17	   710.87	 12
  5	 V 	   299.71	   654.33	 11
  6	 E 	   364.23	   604.79	 10
  7	 E 	   428.75	   540.27	  9
  8	 L 	   485.29	   475.75	  8
  9	 L 	   541.83	   419.21	  7
 10	 A 	   577.35	   362.67	  6
 11	 D 	   634.86	   327.15	  5
 12	 S 	   678.38	   269.63	  4
 13	 E 	   742.90	   226.12	  3
 14	 F 	   816.44	   161.60	  2
 15	 R 	        -  	    88.06	  1  _
```

**4. IPI00030075 Fibroleukin**

| Sequence | Charge | M+H+ | XCorr | DeltaCn |
| --- | --- | --- | --- | --- |
| R.ELESEVNKLSSELK.N | 2 | 1605.769 | 3.05 | 0.261 |

+1 charge

```
		      B   	     Y
  1	 E 	   130.05	        -  	 14
  2	 L 	   243.13	  1475.80	 13
  3	 E 	   372.18	  1362.71	 12
  4	 S 	   459.21	  1233.67	 11
  5	 E 	   588.25	  1146.64	 10
  6	 V 	   687.32	  1017.59	  9
  7	 N 	   801.36	   918.53	  8
  8	 K 	   929.46	   804.48	  7
  9	 L 	  1042.54	   676.39	  6
 10	 S 	  1129.57	   563.30	  5
 11	 S 	  1216.61	   476.27	  4
 12	 E 	  1345.65	   389.24	  3
 13	 L 	  1458.73	   260.20	  2
 14	 K 	        -  	   147.11	  1  _
+2 charge
		      B   	     Y
  1	 E 	    65.53	        -  	 14
  2	 L 	   122.07	   738.40	 13
  3	 E 	   186.59	   681.86	 12
  4	 S 	   230.11	   617.34	 11
  5	 E 	   294.63	   573.82	 10
  6	 V 	   344.16	   509.30	  9
  7	 N 	   401.18	   459.77	  8
  8	 K 	   465.23	   402.74	  7
  9	 L 	   521.77	   338.70	  6
 10	 S 	   565.29	   282.16	  5
 11	 S 	   608.81	   238.64	  4
 12	 E 	   673.33	   195.12	  3
 13	 L 	   729.87	   130.60	  2
 14	 K 	        -  	    74.06	  1  _
```

**5. IPI00021842 Apolipoprotein E**

| Sequence | Charge | M+H+ | XCorr | DeltaCn |
| --- | --- | --- | --- | --- |
| R.GEVQAMLGQSTEELR.V | 2 | 1648.821 | 3.035 | 0.478 |

+1 charge

```
		      B   	     Y
  1	 G 	    58.03	        -  	 15
  2	 E 	   187.07	  1590.78	 14
  3	 V 	   286.14	  1461.74	 13
  4	 Q 	   414.20	  1362.67	 12
  5	 A 	   485.24	  1234.61	 11
  6	 M 	   616.28	  1163.57	 10
  7	 L 	   729.36	  1032.53	  9
  8	 G 	   786.38	   919.45	  8
  9	 Q 	   914.44	   862.43	  7
 10	 S 	  1001.47	   734.37	  6
 11	 T 	  1102.52	   647.34	  5
 12	 E 	  1231.56	   546.29	  4
 13	 E 	  1360.60	   417.25	  3
 14	 L 	  1473.69	   288.20	  2
 15	 R 	        -  	   175.12	  1  _
+2 charge
		      B   	     Y
  1	 G 	    29.52	        -  	 15
  2	 E 	    94.04	   795.89	 14
  3	 V 	   143.57	   731.37	 13
  4	 Q 	   207.60	   681.84	 12
  5	 A 	   243.12	   617.81	 11
  6	 M 	   308.64	   582.29	 10
  7	 L 	   365.18	   516.77	  9
  8	 G 	   393.69	   460.23	  8
  9	 Q 	   457.72	   431.72	  7
 10	 S 	   501.24	   367.69	  6
 11	 T 	   551.76	   324.17	  5
 12	 E 	   616.28	   273.65	  4
 13	 E 	   680.81	   209.13	  3
 14	 L 	   737.35	   144.61	  2
 15	 R 	        -  	    88.06	  1  _
```

**6. IPI00031065 Deoxyribonuclease-1**

| Sequence | Charge | M+H+ | XCorr | DeltaCn |
| --- | --- | --- | --- | --- |
| K.LLDNLNQDAPDTYHYVVSEPLGR.N | 3 | 2630.851 | 4.56 | 0.439 |

+1 charge

```
		      B   	     Y
  1	 L 	   114.09	        -  	 23
  2	 L 	   227.18	  2516.21	 22
  3	 D 	   342.20	  2403.12	 21
  4	 N 	   456.25	  2288.09	 20
  5	 L 	   569.33	  2174.05	 19
  6	 N 	   683.37	  2060.97	 18
  7	 Q 	   811.43	  1946.92	 17
  8	 D 	   926.46	  1818.87	 16
  9	 A 	   997.49	  1703.84	 15
 10	 P 	  1094.55	  1632.80	 14
 11	 D 	  1209.57	  1535.75	 13
 12	 T 	  1310.62	  1420.72	 12
 13	 Y 	  1473.69	  1319.67	 11
 14	 H 	  1610.74	  1156.61	 10
 15	 Y 	  1773.81	  1019.55	  9
 16	 V 	  1872.88	   856.49	  8
 17	 V 	  1971.94	   757.42	  7
 18	 S 	  2058.98	   658.35	  6
 19	 E 	  2188.02	   571.32	  5
 20	 P 	  2285.07	   442.28	  4
 21	 L 	  2398.16	   345.22	  3
 22	 G 	  2455.18	   232.14	  2
 23	 R 	        -  	   175.12	  1  _


+2 charge
		      B   	     Y
  1	 L 	    57.55	        -  	 23
  2	 L 	   114.09	  1258.61	 22
  3	 D 	   171.60	  1202.06	 21
  4	 N 	   228.63	  1144.55	 20
  5	 L 	   285.17	  1087.53	 19
  6	 N 	   342.19	  1030.99	 18
  7	 Q 	   406.22	   973.97	 17
  8	 D 	   463.73	   909.94	 16
  9	 A 	   499.25	   852.42	 15
 10	 P 	   547.78	   816.90	 14
 11	 D 	   605.29	   768.38	 13
 12	 T 	   655.81	   710.86	 12
 13	 Y 	   737.35	   660.34	 11
 14	 H 	   805.88	   578.81	 10
 15	 Y 	   887.41	   510.28	  9
 16	 V 	   936.94	   428.75	  8
 17	 V 	   986.48	   379.21	  7
 18	 S 	  1029.99	   329.68	  6
 19	 E 	  1094.51	   286.16	  5
 20	 P 	  1143.04	   221.64	  4
 21	 L 	  1199.58	   173.12	  3
 22	 G 	  1228.09	   116.57	  2
 23	 R 	        -  	    88.06	  1  _
+3 charge
		      B   	     Y
  1	 L 	    38.70	        -  	 23
  2	 L 	    76.40	   839.41	 22
  3	 D 	   114.74	   801.71	 21
  4	 N 	   152.75	   763.37	 20
  5	 L 	   190.45	   725.36	 19
  6	 N 	   228.46	   687.66	 18
  7	 Q 	   271.15	   649.65	 17
  8	 D 	   309.49	   606.96	 16
  9	 A 	   333.17	   568.62	 15
 10	 P 	   365.52	   544.94	 14
 11	 D 	   403.86	   512.59	 13
 12	 T 	   437.55	   474.25	 12
 13	 Y 	   491.90	   440.56	 11
 14	 H 	   537.59	   386.21	 10
 15	 Y 	   591.94	   340.52	  9
 16	 V 	   624.96	   286.17	  8
 17	 V 	   657.99	   253.14	  7
 18	 S 	   687.00	   220.12	  6
 19	 E 	   730.01	   191.11	  5
 20	 P 	   762.36	   148.10	  4
 21	 L 	   800.06	   115.75	  3
 22	 G 	   819.06	    78.05	  2
 23	 R 	        -  	    59.04	  1  _
```

**7. IPI00409698 Isoform 1 of Transportin-2**

| Sequence | Charge | M+H+ | XCorr | DeltaCn |
| --- | --- | --- | --- | --- |
| K.QECLNNIGDASSLIR.A | 2 | 1690.831 | 3.538 | 0.307 |

+1 charge

```
		      B   	     Y
  1	 Q 	   129.07	        -  	 15
  2	 E 	   258.11	  1561.90	 14
  3	 C 	   418.27	  1432.86	 13
  4	 L 	   531.36	  1272.69	 12
  5	 N 	   645.40	  1159.61	 11
  6	 N 	   759.44	  1045.56	 10
  7	 I 	   872.53	   931.52	  9
  8	 G 	   929.55	   818.44	  8
  9	 D 	  1044.58	   761.42	  7
 10	 A 	  1115.61	   646.39	  6
 11	 S 	  1202.65	   575.35	  5
 12	 S 	  1289.68	   488.32	  4
 13	 L 	  1402.76	   401.29	  3
 14	 I 	  1515.85	   288.20	  2
 15	 R 	        -  	   175.12	  1  _
+2 charge
		      B   	     Y
  1	 Q 	    65.04	        -  	 15
  2	 E 	   129.56	   781.45	 14
  3	 C 	   209.64	   716.93	 13
  4	 L 	   266.18	   636.85	 12
  5	 N 	   323.20	   580.31	 11
  6	 N 	   380.23	   523.29	 10
  7	 I 	   436.77	   466.26	  9
  8	 G 	   465.28	   409.72	  8
  9	 D 	   522.79	   381.21	  7
 10	 A 	   558.31	   323.70	  6
 11	 S 	   601.83	   288.18	  5
 12	 S 	   645.34	   244.66	  4
 13	 L 	   701.88	   201.15	  3
 14	 I 	   758.43	   144.61	  2
 15	 R 	        -  	    88.06	  1  _
```

**8. IPI00032826 Hsc70-interacting protein**

| Sequence | Charge | M+H+ | XCorr | DeltaCn |
| --- | --- | --- | --- | --- |
| K.AIDLFTDAIKLNPR.L | 2 | 1587.845 | 3.721 | 0.454 |

+1 charge

```
		      B   	     Y
  1	 A 	    72.04	        -  	 14
  2	 I 	   185.13	  1515.85	 13
  3	 D 	   300.16	  1402.77	 12
  4	 L 	   413.24	  1287.74	 11
  5	 F 	   560.31	  1174.66	 10
  6	 T 	   661.36	  1027.59	  9
  7	 D 	   776.38	   926.54	  8
  8	 A 	   847.42	   811.51	  7
  9	 I 	   960.50	   740.48	  6
 10	 K 	  1088.60	   627.39	  5
 11	 L 	  1201.68	   499.30	  4
 12	 N 	  1315.73	   386.21	  3
 13	 P 	  1412.78	   272.17	  2
 14	 R 	        -  	   175.12	  1  _
+2 charge
		      B   	     Y
  1	 A 	    36.53	        -  	 14
  2	 I 	    93.07	   758.43	 13
  3	 D 	   150.58	   701.89	 12
  4	 L 	   207.12	   644.37	 11
  5	 F 	   280.66	   587.83	 10
  6	 T 	   331.18	   514.30	  9
  7	 D 	   388.69	   463.77	  8
  8	 A 	   424.21	   406.26	  7
  9	 I 	   480.76	   370.74	  6
 10	 K 	   544.80	   314.20	  5
 11	 L 	   601.34	   250.15	  4
 12	 N 	   658.37	   193.61	  3
 13	 P 	   706.89	   136.59	  2
 14	 R 	        -  	    88.06	  1  _
```

**9. IPI00744702 Isoform 1 of Multivesicular body subunit 12A**

| Sequence | Charge | M+H+ | XCorr | DeltaCn |
| --- | --- | --- | --- | --- |
| K.LLPLGATDTAVFDVR.L | 2 | 1588.83 | 3.097 | 0.495 |

+1 charge

```
		      B   	     Y
  1	 L 	   114.09	        -  	 15
  2	 L 	   227.18	  1474.79	 14
  3	 P 	   324.23	  1361.71	 13
  4	 L 	   437.31	  1264.65	 12
  5	 G 	   494.33	  1151.57	 11
  6	 A 	   565.37	  1094.55	 10
  7	 T 	   666.42	  1023.51	  9
  8	 D 	   781.45	   922.46	  8
  9	 T 	   882.49	   807.44	  7
 10	 A 	   953.53	   706.39	  6
 11	 V 	  1052.60	   635.35	  5
 12	 F 	  1199.67	   536.28	  4
 13	 D 	  1314.69	   389.21	  3
 14	 V 	  1413.76	   274.19	  2
 15	 R 	        -  	   175.12	  1  _
+2 charge
		      B   	     Y
  1	 L 	    57.55	        -  	 15
  2	 L 	   114.09	   737.90	 14
  3	 P 	   162.62	   681.36	 13
  4	 L 	   219.16	   632.83	 12
  5	 G 	   247.67	   576.29	 11
  6	 A 	   283.19	   547.78	 10
  7	 T 	   333.71	   512.26	  9
  8	 D 	   391.23	   461.74	  8
  9	 T 	   441.75	   404.22	  7
 10	 A 	   477.27	   353.70	  6
 11	 V 	   526.80	   318.18	  5
 12	 F 	   600.34	   268.64	  4
 13	 D 	   657.85	   195.11	  3
 14	 V 	   707.38	   137.60	  2
 15	 R 	        -  	    88.06	  1  _
```

**10. IPI00909850 cDNA FLJ58287, highly similar to Galactoside 2-alpha-L-fucosyltransferase 2**

| Sequence | Charge | M+H+ | XCorr | DeltaCn |
| --- | --- | --- | --- | --- |
| - .MMKLCKPK.N | 2 | 1036.375 | 2.838 | 0.369 |

+1 charge

```
		      B   	     Y
  1	 M 	   132.05	        -  	  8
  2	 M 	   263.09	   904.65	  7
  3	 K 	   391.18	   773.60	  6
  4	 L 	   504.27	   645.51	  5
  5	 C 	   664.43	   532.43	  4
  6	 K 	   792.53	   372.26	  3
  7	 P 	   889.58	   244.17	  2
  8	 K 	        -  	   147.11	  1  _
+2 charge
		      B   	     Y
  1	 M 	    66.53	        -  	  8
  2	 M 	   132.05	   452.83	  7
  3	 K 	   196.10	   387.31	  6
  4	 L 	   252.64	   323.26	  5
  5	 C 	   332.72	   266.72	  4
  6	 K 	   396.77	   186.63	  3
  7	 P 	   445.29	   122.59	  2
  8	 K 	        -  	    74.06	  1  _
```

**11. IPI00216088 Cellular retinoic acid-binding protein 2**

| Sequence | Charge | M+H+ | XCorr | DeltaCn |
| --- | --- | --- | --- | --- |
| K.VGEEFEEQTVDGRPCK.S | 2 | 1880.984 | 3.319 | 0.25 |

+1 charge

```
		      B   	     Y
  1	 V 	   100.08	        -  	 16
  2	 G 	   157.10	  1780.92	 15
  3	 E 	   286.14	  1723.89	 14
  4	 E 	   415.18	  1594.85	 13
  5	 F 	   562.25	  1465.81	 12
  6	 E 	   691.29	  1318.74	 11
  7	 E 	   820.34	  1189.70	 10
  8	 Q 	   948.39	  1060.65	  9
  9	 T 	  1049.44	   932.60	  8
 10	 V 	  1148.51	   831.55	  7
 11	 D 	  1263.54	   732.48	  6
 12	 G 	  1320.56	   617.45	  5
 13	 R 	  1476.66	   560.43	  4
 14	 P 	  1573.71	   404.33	  3
 15	 C 	  1733.88	   307.28	  2
 16	 K 	        -  	   147.11	  1  _
+2 charge
		      B   	     Y
  1	 V 	    50.54	        -  	 16
  2	 G 	    79.05	   890.96	 15
  3	 E 	   143.57	   862.45	 14
  4	 E 	   208.09	   797.93	 13
  5	 F 	   281.63	   733.41	 12
  6	 E 	   346.15	   659.87	 11
  7	 E 	   410.67	   595.35	 10
  8	 Q 	   474.70	   530.83	  9
  9	 T 	   525.22	   466.80	  8
 10	 V 	   574.76	   416.28	  7
 11	 D 	   632.27	   366.74	  6
 12	 G 	   660.78	   309.23	  5
 13	 R 	   738.83	   280.72	  4
 14	 P 	   787.36	   202.67	  3
 15	 C 	   867.44	   154.14	  2
 16	 K 	        -  	    74.06	  1  _
```

**12. IPI00023858 Cellular retinoic acid-binding protein 2**

| Sequence | Charge | M+H+ | XCorr | DeltaCn |
| --- | --- | --- | --- | --- |
| K.ATLKDSGSYFCR.G | 2 | 1405.531 | 3.324 | 0.393 |

+1 charge

```
		      B   	     Y
  1	 A 	    72.04	        -  	 12
  2	 T 	   173.09	  1333.76	 11
  3	 L 	   286.18	  1232.71	 10
  4	 K 	   414.27	  1119.62	  9
  5	 D 	   529.30	   991.53	  8
  6	 S 	   616.33	   876.50	  7
  7	 G 	   673.35	   789.47	  6
  8	 S 	   760.38	   732.45	  5
  9	 Y 	   923.45	   645.42	  4
 10	 F 	  1070.52	   482.35	  3
 11	 C 	  1230.68	   335.28	  2
 12	 R 	        -  	   175.12	  1  _
+2 charge
		      B   	     Y
  1	 A 	    36.53	        -  	 12
  2	 T 	    87.05	   667.38	 11
  3	 L 	   143.59	   616.86	 10
  4	 K 	   207.64	   560.32	  9
  5	 D 	   265.15	   496.27	  8
  6	 S 	   308.67	   438.75	  7
  7	 G 	   337.18	   395.24	  6
  8	 S 	   380.70	   366.73	  5
  9	 Y 	   462.23	   323.21	  4
 10	 F 	   535.76	   241.68	  3
 11	 C 	   615.84	   168.15	  2
 12	 R 	        -  	    88.06	  1  _
```

**13. IPI00384404 Rheumatoid factor RF-ET9 (Fragment)**

| Sequence | Charge | M+H+ | XCorr | DeltaCn |
| --- | --- | --- | --- | --- |
| - .LVESGGGLVKPGGSLR.L | 2 | 1526.763 | 4.2 | 0.508 |

+1 charge

```
		      B   	     Y
  1	 L 	   114.09	        -  	 16
  2	 V 	   213.16	  1412.79	 15
  3	 E 	   342.20	  1313.72	 14
  4	 S 	   429.23	  1184.67	 13
  5	 G 	   486.26	  1097.64	 12
  6	 G 	   543.28	  1040.62	 11
  7	 G 	   600.30	   983.60	 10
  8	 L 	   713.38	   926.58	  9
  9	 V 	   812.45	   813.49	  8
 10	 K 	   940.55	   714.43	  7
 11	 P 	  1037.60	   586.33	  6
 12	 G 	  1094.62	   489.28	  5
 13	 G 	  1151.64	   432.26	  4
 14	 S 	  1238.67	   375.24	  3
 15	 L 	  1351.76	   288.20	  2
 16	 R 	        -  	   175.12	  1  _
+2 charge
		      B   	     Y
  1	 L 	    57.55	        -  	 16
  2	 V 	   107.08	   706.90	 15
  3	 E 	   171.60	   657.36	 14
  4	 S 	   215.12	   592.84	 13
  5	 G 	   243.63	   549.32	 12
  6	 G 	   272.14	   520.81	 11
  7	 G 	   300.65	   492.30	 10
  8	 L 	   357.20	   463.79	  9
  9	 V 	   406.73	   407.25	  8
 10	 K 	   470.78	   357.72	  7
 11	 P 	   519.30	   293.67	  6
 12	 G 	   547.81	   245.14	  5
 13	 G 	   576.32	   216.63	  4
 14	 S 	   619.84	   188.12	  3
 15	 L 	   676.38	   144.61	  2
 16	 R 	        -  	    88.06	  1  _
```

**14. IPI00219827 Secreted Ly-6/uPAR-related protein 2**

| Sequence | Charge | M+H+ | XCorr | DeltaCn |
| --- | --- | --- | --- | --- |
| R.VLSNTEDLPLVTK.M | 2 | 1429.641 | 4.461 | 0.552 |

+1 charge

```
		      B   	     Y
  1	 V 	   100.08	        -  	 13
  2	 L 	   213.16	  1329.73	 12
  3	 S 	   300.19	  1216.64	 11
  4	 N 	   414.23	  1129.61	 10
  5	 T 	   515.28	  1015.57	  9
  6	 E 	   644.32	   914.52	  8
  7	 D 	   759.35	   785.48	  7
  8	 L 	   872.44	   670.45	  6
  9	 P 	   969.49	   557.37	  5
 10	 L 	  1082.57	   460.31	  4
 11	 V 	  1181.64	   347.23	  3
 12	 T 	  1282.69	   248.16	  2
 13	 K 	        -  	   147.11	  1  _
+2 charge
		      B   	     Y
  1	 V 	    50.54	        -  	 13
  2	 L 	   107.08	   665.37	 12
  3	 S 	   150.60	   608.82	 11
  4	 N 	   207.62	   565.31	 10
  5	 T 	   258.14	   508.29	  9
  6	 E 	   322.67	   457.76	  8
  7	 D 	   380.18	   393.24	  7
  8	 L 	   436.72	   335.73	  6
  9	 P 	   485.25	   279.19	  5
 10	 L 	   541.79	   230.66	  4
 11	 V 	   591.32	   174.12	  3
 12	 T 	   641.85	   124.58	  2
 13	 K 	        -  	    74.06	  1  _
```

**15. IPI00005038 Ribonuclease UK114**

| Sequence | Charge | M+H+ | XCorr | DeltaCn |
| --- | --- | --- | --- | --- |
| K.APGAIGPYSQAVLVDR.T | 2 | 1614.827 | 3.427 | 0.485 |

+1 charge

```
		      B   	     Y
  1	 A 	    72.04	        -  	 16
  2	 P 	   169.10	  1542.83	 15
  3	 G 	   226.12	  1445.77	 14
  4	 A 	   297.16	  1388.75	 13
  5	 I 	   410.24	  1317.72	 12
  6	 G 	   467.26	  1204.63	 11
  7	 P 	   564.31	  1147.61	 10
  8	 Y 	   727.38	  1050.56	  9
  9	 S 	   814.41	   887.49	  8
 10	 Q 	   942.47	   800.46	  7
 11	 A 	  1013.51	   672.40	  6
 12	 V 	  1112.57	   601.37	  5
 13	 L 	  1225.66	   502.30	  4
 14	 V 	  1324.73	   389.21	  3
 15	 D 	  1439.75	   290.15	  2
 16	 R 	        -  	   175.12	  1  _
+2 charge
		      B   	     Y
  1	 A 	    36.53	        -  	 16
  2	 P 	    85.05	   771.92	 15
  3	 G 	   113.56	   723.39	 14
  4	 A 	   149.08	   694.88	 13
  5	 I 	   205.62	   659.36	 12
  6	 G 	   234.13	   602.82	 11
  7	 P 	   282.66	   574.31	 10
  8	 Y 	   364.19	   525.78	  9
  9	 S 	   407.71	   444.25	  8
 10	 Q 	   471.74	   400.73	  7
 11	 A 	   507.26	   336.71	  6
 12	 V 	   556.79	   301.19	  5
 13	 L 	   613.33	   251.65	  4
 14	 V 	   662.87	   195.11	  3
 15	 D 	   720.38	   145.58	  2
 16	 R 	        -  	    88.06	  1  _
```

**16. IPI00012540 Prominin-1**

| Sequence | Charge | M+H+ | XCorr | DeltaCn |
| --- | --- | --- | --- | --- |
| R.LSLSQLNSNPELR.Q | 2 | 1471.641 | 2.845 | 0.395 |

+1 charge

```
		      B   	     Y
  1	 L 	   114.09	        -  	 13
  2	 S 	   201.12	  1357.71	 12
  3	 L 	   314.21	  1270.67	 11
  4	 S 	   401.24	  1157.59	 10
  5	 Q 	   529.30	  1070.56	  9
  6	 L 	   642.38	   942.50	  8
  7	 N 	   756.43	   829.42	  7
  8	 S 	   843.46	   715.37	  6
  9	 N 	   957.50	   628.34	  5
 10	 P 	  1054.55	   514.30	  4
 11	 E 	  1183.60	   417.25	  3
 12	 L 	  1296.68	   288.20	  2
 13	 R 	        -  	   175.12	  1  _
+2 charge
		      B   	     Y
  1	 L 	    57.55	        -  	 13
  2	 S 	   101.07	   679.36	 12
  3	 L 	   157.61	   635.84	 11
  4	 S 	   201.12	   579.30	 10
  5	 Q 	   265.15	   535.78	  9
  6	 L 	   321.69	   471.75	  8
  7	 N 	   378.72	   415.21	  7
  8	 S 	   422.23	   358.19	  6
  9	 N 	   479.25	   314.67	  5
 10	 P 	   527.78	   257.65	  4
 11	 E 	   592.30	   209.13	  3
 12	 L 	   648.84	   144.61	  2
 13	 R 	        -  	    88.06	  1  _
```

**17. IPI00031547 Desmoglein-3**

| Sequence | Charge | M+H+ | XCorr | DeltaCn |
| --- | --- | --- | --- | --- |
| R.YTGPYTFALEDQPVK.L | 2 | 1729.91 | 3.698 | 0.471 |

+1 charge

```
		      B   	     Y
  1	 Y 	   164.07	        -  	 15
  2	 T 	   265.12	  1565.78	 14
  3	 G 	   322.14	  1464.74	 13
  4	 P 	   419.19	  1407.72	 12
  5	 Y 	   582.26	  1310.66	 11
  6	 T 	   683.30	  1147.60	 10
  7	 F 	   830.37	  1046.55	  9
  8	 A 	   901.41	   899.48	  8
  9	 L 	  1014.49	   828.45	  7
 10	 E 	  1143.54	   715.36	  6
 11	 D 	  1258.56	   586.32	  5
 12	 Q 	  1386.62	   471.29	  4
 13	 P 	  1483.67	   343.23	  3
 14	 V 	  1582.74	   246.18	  2
 15	 K 	        -  	   147.11	  1  _
+2 charge
		      B   	     Y
  1	 Y 	    82.54	        -  	 15
  2	 T 	   133.06	   783.40	 14
  3	 G 	   161.57	   732.87	 13
  4	 P 	   210.10	   704.36	 12
  5	 Y 	   291.63	   655.83	 11
  6	 T 	   342.16	   574.30	 10
  7	 F 	   415.69	   523.78	  9
  8	 A 	   451.21	   450.25	  8
  9	 L 	   507.75	   414.73	  7
 10	 E 	   572.27	   358.18	  6
 11	 D 	   629.78	   293.66	  5
 12	 Q 	   693.81	   236.15	  4
 13	 P 	   742.34	   172.12	  3
 14	 V 	   791.87	   123.59	  2
 15	 K 	        -  	    74.06	  1  _
```

**18. IPI00879999 ATP-dependent RNA helicase DDX54**

| Sequence | Charge | M+H+ | XCorr | DeltaCn |
| --- | --- | --- | --- | --- |
| R.DQEFYIPYRPKDFDSER.G | 2 | 2206.355 | 3.522 | 0.291 |

+1 charge

```
		      B   	     Y
  1	 D 	   116.03	        -  	 17
  2	 Q 	   244.09	  2090.00	 16
  3	 E 	   373.14	  1961.94	 15
  4	 F 	   520.20	  1832.90	 14
  5	 Y 	   683.27	  1685.83	 13
  6	 I 	   796.35	  1522.76	 12
  7	 P 	   893.40	  1409.68	 11
  8	 Y 	  1056.47	  1312.63	 10
  9	 R 	  1212.57	  1149.56	  9
 10	 P 	  1309.62	   993.46	  8
 11	 K 	  1437.72	   896.41	  7
 12	 D 	  1552.74	   768.32	  6
 13	 F 	  1699.81	   653.29	  5
 14	 D 	  1814.84	   506.22	  4
 15	 S 	  1901.87	   391.19	  3
 16	 E 	  2030.91	   304.16	  2
 17	 R 	        -  	   175.12	  1  _
+2 charge
		      B   	     Y
  1	 D 	    58.52	        -  	 17
  2	 Q 	   122.55	  1045.50	 16
  3	 E 	   187.07	   981.47	 15
  4	 F 	   260.61	   916.95	 14
  5	 Y 	   342.14	   843.42	 13
  6	 I 	   398.68	   761.89	 12
  7	 P 	   447.21	   705.34	 11
  8	 Y 	   528.74	   656.82	 10
  9	 R 	   606.79	   575.29	  9
 10	 P 	   655.31	   497.24	  8
 11	 K 	   719.36	   448.71	  7
 12	 D 	   776.88	   384.66	  6
 13	 F 	   850.41	   327.15	  5
 14	 D 	   907.92	   253.61	  4
 15	 S 	   951.44	   196.10	  3
 16	 E 	  1015.96	   152.58	  2
 17	 R 	        -  	    88.06	  1  _
```

**19. IPI00063827 Isoform 1 of Abhydrolase domain-containing protein 14B**

| Sequence | Charge | M+H+ | XCorr | DeltaCn |
| --- | --- | --- | --- | --- |
| K.TPALIVYGDQDPMGQTSFEHLK.Q | 3 | 2448.736 | 3.981 | 0.46 |

+1 charge

```
		      B   	     Y
  1	 T 	   102.05	        -  	 22
  2	 P 	   199.11	  2346.14	 21
  3	 A 	   270.14	  2249.09	 20
  4	 L 	   383.23	  2178.05	 19
  5	 I 	   496.31	  2064.97	 18
  6	 V 	   595.38	  1951.89	 17
  7	 Y 	   758.44	  1852.82	 16
  8	 G 	   815.47	  1689.75	 15
  9	 D 	   930.49	  1632.73	 14
 10	 Q 	  1058.55	  1517.71	 13
 11	 D 	  1173.58	  1389.65	 12
 12	 P 	  1270.63	  1274.62	 11
 13	 M 	  1401.67	  1177.57	 10
 14	 G 	  1458.69	  1046.53	  9
 15	 Q 	  1586.75	   989.51	  8
 16	 T 	  1687.80	   861.45	  7
 17	 S 	  1774.83	   760.40	  6
 18	 F 	  1921.90	   673.37	  5
 19	 E 	  2050.94	   526.30	  4
 20	 H 	  2188.00	   397.26	  3
 21	 L 	  2301.09	   260.20	  2
 22	 K 	        -  	   147.11	  1  _


+2 charge
		      B   	     Y
  1	 T 	    51.53	        -  	 22
  2	 P 	   100.06	  1173.58	 21
  3	 A 	   135.58	  1125.05	 20
  4	 L 	   192.12	  1089.53	 19
  5	 I 	   248.66	  1032.99	 18
  6	 V 	   298.19	   976.45	 17
  7	 Y 	   379.73	   926.91	 16
  8	 G 	   408.24	   845.38	 15
  9	 D 	   465.75	   816.87	 14
 10	 Q 	   529.78	   759.36	 13
 11	 D 	   587.29	   695.33	 12
 12	 P 	   635.82	   637.81	 11
 13	 M 	   701.34	   589.29	 10
 14	 G 	   729.85	   523.77	  9
 15	 Q 	   793.88	   495.26	  8
 16	 T 	   844.40	   431.23	  7
 17	 S 	   887.92	   380.70	  6
 18	 F 	   961.45	   337.19	  5
 19	 E 	  1025.97	   263.65	  4
 20	 H 	  1094.50	   199.13	  3
 21	 L 	  1151.05	   130.60	  2
 22	 K 	        -  	    74.06	  1  _
+3 charge
		      B   	     Y
  1	 T 	    34.69	        -  	 22
  2	 P 	    67.04	   782.72	 21
  3	 A 	    90.72	   750.37	 20
  4	 L 	   128.41	   726.69	 19
  5	 I 	   166.11	   688.99	 18
  6	 V 	   199.13	   651.30	 17
  7	 Y 	   253.49	   618.28	 16
  8	 G 	   272.49	   563.92	 15
  9	 D 	   310.84	   544.92	 14
 10	 Q 	   353.52	   506.57	 13
 11	 D 	   391.86	   463.89	 12
 12	 P 	   424.22	   425.54	 11
 13	 M 	   467.90	   393.19	 10
 14	 G 	   486.90	   349.51	  9
 15	 Q 	   529.59	   330.51	  8
 16	 T 	   563.27	   287.82	  7
 17	 S 	   592.28	   254.14	  6
 18	 F 	   641.30	   225.13	  5
 19	 E 	   684.32	   176.10	  4
 20	 H 	   730.01	   133.09	  3
 21	 L 	   767.70	    87.40	  2
 22	 K 	        -  	    49.71	  1  _
```

**20. IPI00000760 N(G),N(G)-dimethylarginine dimethylaminohydrolase 2**

| Sequence | Charge | M+H+ | XCorr | DeltaCn |
| --- | --- | --- | --- | --- |
| R.GVPESLASGEGAGAGLPALDLAK.A | 2 | 2081.313 | 5.259 | 0.619 |

+1 charge

```
		      B   	     Y
  1	 G 	    58.03	        -  	 23
  2	 V 	   157.10	  2023.07	 22
  3	 P 	   254.15	  1924.00	 21
  4	 E 	   383.19	  1826.95	 20
  5	 S 	   470.22	  1697.91	 19
  6	 L 	   583.31	  1610.87	 18
  7	 A 	   654.35	  1497.79	 17
  8	 S 	   741.38	  1426.75	 16
  9	 G 	   798.40	  1339.72	 15
 10	 E 	   927.44	  1282.70	 14
 11	 G 	   984.46	  1153.66	 13
 12	 A 	  1055.50	  1096.64	 12
 13	 G 	  1112.52	  1025.60	 11
 14	 A 	  1183.56	   968.58	 10
 15	 G 	  1240.58	   897.54	  9
 16	 L 	  1353.66	   840.52	  8
 17	 P 	  1450.72	   727.43	  7
 18	 A 	  1521.75	   630.38	  6
 19	 L 	  1634.84	   559.34	  5
 20	 D 	  1749.87	   446.26	  4
 21	 L 	  1862.95	   331.23	  3
 22	 A 	  1933.99	   218.15	  2
 23	 K 	        -  	   147.11	  1  _


+2 charge
		      B   	     Y
  1	 G 	    29.52	        -  	 23
  2	 V 	    79.05	  1012.04	 22
  3	 P 	   127.58	   962.50	 21
  4	 E 	   192.10	   913.98	 20
  5	 S 	   235.62	   849.46	 19
  6	 L 	   292.16	   805.94	 18
  7	 A 	   327.68	   749.40	 17
  8	 S 	   371.19	   713.88	 16
  9	 G 	   399.70	   670.36	 15
 10	 E 	   464.22	   641.85	 14
 11	 G 	   492.74	   577.33	 13
 12	 A 	   528.25	   548.82	 12
 13	 G 	   556.76	   513.30	 11
 14	 A 	   592.28	   484.79	 10
 15	 G 	   620.79	   449.27	  9
 16	 L 	   677.34	   420.76	  8
 17	 P 	   725.86	   364.22	  7
 18	 A 	   761.38	   315.69	  6
 19	 L 	   817.92	   280.18	  5
 20	 D 	   875.44	   223.63	  4
 21	 L 	   931.98	   166.12	  3
 22	 A 	   967.50	   109.58	  2
 23	 K 	        -  	    74.06	  1  _
```

**21. IPI00032294 Cystatin-S**

| Sequence | Charge | M+H+ | XCorr | DeltaCn |
| --- | --- | --- | --- | --- |
| R.IIPGGIYDADLNDEWVQR.A | 2 | 2075.267 | 3.491 | 0.532 |

+1 charge

```
		      B   	     Y
  1	 I 	   114.09	        -  	 18
  2	 I 	   227.18	  1960.94	 17
  3	 P 	   324.23	  1847.86	 16
  4	 G 	   381.25	  1750.80	 15
  5	 G 	   438.27	  1693.78	 14
  6	 I 	   551.36	  1636.76	 13
  7	 Y 	   714.42	  1523.68	 12
  8	 D 	   829.45	  1360.61	 11
  9	 A 	   900.48	  1245.59	 10
 10	 D 	  1015.51	  1174.55	  9
 11	 L 	  1128.59	  1059.52	  8
 12	 N 	  1242.64	   946.44	  7
 13	 D 	  1357.66	   832.39	  6
 14	 E 	  1486.71	   717.37	  5
 15	 W 	  1672.79	   588.33	  4
 16	 V 	  1771.85	   402.25	  3
 17	 Q 	  1899.91	   303.18	  2
 18	 R 	        -  	   175.12	  1  _
+2 charge
		      B   	     Y
  1	 I 	    57.55	        -  	 18
  2	 I 	   114.09	   980.97	 17
  3	 P 	   162.62	   924.43	 16
  4	 G 	   191.13	   875.91	 15
  5	 G 	   219.64	   847.39	 14
  6	 I 	   276.18	   818.88	 13
  7	 Y 	   357.71	   762.34	 12
  8	 D 	   415.23	   680.81	 11
  9	 A 	   450.74	   623.30	 10
 10	 D 	   508.26	   587.78	  9
 11	 L 	   564.80	   530.26	  8
 12	 N 	   621.82	   473.72	  7
 13	 D 	   679.34	   416.70	  6
 14	 E 	   743.86	   359.19	  5
 15	 W 	   836.90	   294.67	  4
 16	 V 	   886.43	   201.63	  3
 17	 Q 	   950.46	   152.09	  2
 18	 R 	        -  	    88.06	  1  _
```

**22. IPI00009276 Endothelial protein C receptor precursor**

| Sequence | Charge | M+H+ | XCorr | DeltaCn |
| --- | --- | --- | --- | --- |
| R.TQSGLQSYLLQFHGLVR.L | 2 | 1948.214 | 3.433 | 0.404 |

+1 charge

```
		      B   	     Y
  1	 T 	   102.05	        -  	 17
  2	 Q 	   230.11	  1846.00	 16
  3	 S 	   317.15	  1717.94	 15
  4	 G 	   374.17	  1630.91	 14
  5	 L 	   487.25	  1573.88	 13
  6	 Q 	   615.31	  1460.80	 12
  7	 S 	   702.34	  1332.74	 11
  8	 Y 	   865.41	  1245.71	 10
  9	 L 	   978.49	  1082.65	  9
 10	 L 	  1091.57	   969.56	  8
 11	 Q 	  1219.63	   856.48	  7
 12	 F 	  1366.70	   728.42	  6
 13	 H 	  1503.76	   581.35	  5
 14	 G 	  1560.78	   444.29	  4
 15	 L 	  1673.86	   387.27	  3
 16	 V 	  1772.93	   274.19	  2
 17	 R 	        -  	   175.12	  1  _
+2 charge
		      B   	     Y
  1	 T 	    51.53	        -  	 17
  2	 Q 	   115.56	   923.50	 16
  3	 S 	   159.08	   859.47	 15
  4	 G 	   187.59	   815.96	 14
  5	 L 	   244.13	   787.45	 13
  6	 Q 	   308.16	   730.90	 12
  7	 S 	   351.67	   666.87	 11
  8	 Y 	   433.21	   623.36	 10
  9	 L 	   489.75	   541.83	  9
 10	 L 	   546.29	   485.29	  8
 11	 Q 	   610.32	   428.74	  7
 12	 F 	   683.85	   364.71	  6
 13	 H 	   752.38	   291.18	  5
 14	 G 	   780.89	   222.65	  4
 15	 L 	   837.44	   194.14	  3
 16	 V 	   886.97	   137.60	  2
 17	 R 	        -  	    88.06	  1  _
```

**23. IPI00942979 Transketolase**

| Sequence | Charge | M+H+ | XCorr | DeltaCn |
| --- | --- | --- | --- | --- |
| K.NMAEQIIQEIYSQIQSK.K | 3 | 2024.285 | 3.324 | 0.318 |

+1 charge

```
		      B   	     Y
  1	 N 	   115.05	        -  	 17
  2	 M 	   246.09	  1908.97	 16
  3	 A 	   317.13	  1777.93	 15
  4	 E 	   446.17	  1706.90	 14
  5	 Q 	   574.23	  1577.85	 13
  6	 I 	   687.31	  1449.79	 12
  7	 I 	   800.40	  1336.71	 11
  8	 Q 	   928.46	  1223.63	 10
  9	 E 	  1057.50	  1095.57	  9
 10	 I 	  1170.58	   966.53	  8
 11	 Y 	  1333.65	   853.44	  7
 12	 S 	  1420.68	   690.38	  6
 13	 Q 	  1548.74	   603.35	  5
 14	 I 	  1661.82	   475.29	  4
 15	 Q 	  1789.88	   362.20	  3
 16	 S 	  1876.91	   234.14	  2
 17	 K 	        -  	   147.11	  1  _
+2 charge
		      B   	     Y
  1	 N 	    58.03	        -  	 17
  2	 M 	   123.55	   954.99	 16
  3	 A 	   159.07	   889.47	 15
  4	 E 	   223.59	   853.95	 14
  5	 Q 	   287.62	   789.43	 13
  6	 I 	   344.16	   725.40	 12
  7	 I 	   400.70	   668.86	 11
  8	 Q 	   464.73	   612.32	 10
  9	 E 	   529.25	   548.29	  9
 10	 I 	   585.79	   483.77	  8
 11	 Y 	   667.33	   427.22	  7
 12	 S 	   710.84	   345.69	  6
 13	 Q 	   774.87	   302.18	  5
 14	 I 	   831.41	   238.15	  4
 15	 Q 	   895.44	   181.61	  3
 16	 S 	   938.96	   117.58	  2
 17	 K 	        -  	    74.06	  1  _


+3 charge
		      B   	     Y
  1	 N 	    39.02	        -  	 17
  2	 M 	    82.70	   637.00	 16
  3	 A 	   106.38	   593.32	 15
  4	 E 	   149.39	   569.64	 14
  5	 Q 	   192.08	   526.62	 13
  6	 I 	   229.78	   483.94	 12
  7	 I 	   267.47	   446.24	 11
  8	 Q 	   310.16	   408.55	 10
  9	 E 	   353.17	   365.86	  9
 10	 I 	   390.87	   322.85	  8
 11	 Y 	   445.22	   285.15	  7
 12	 S 	   474.23	   230.80	  6
 13	 Q 	   516.92	   201.79	  5
 14	 I 	   554.61	   159.10	  4
 15	 Q 	   597.30	   121.41	  3
 16	 S 	   626.31	    78.72	  2
 17	 K 	        -  	    49.71	  1  _
```

**24. IPI00329538 Prostasin**

| Sequence | Charge | M+H+ | XCorr | DeltaCn |
| --- | --- | --- | --- | --- |
| K.LGAHQLDSYSEDAK.V | 2 | 1534.609 | 2.891 | 0.471 |

+1 charge

```
		      B   	     Y
  1	 L 	   114.09	        -  	 14
  2	 G 	   171.11	  1420.63	 13
  3	 A 	   242.15	  1363.61	 12
  4	 H 	   379.21	  1292.58	 11
  5	 Q 	   507.27	  1155.52	 10
  6	 L 	   620.35	  1027.46	  9
  7	 D 	   735.38	   914.37	  8
  8	 S 	   822.41	   799.35	  7
  9	 Y 	   985.47	   712.31	  6
 10	 S 	  1072.51	   549.25	  5
 11	 E 	  1201.55	   462.22	  4
 12	 D 	  1316.58	   333.18	  3
 13	 A 	  1387.61	   218.15	  2
 14	 K 	        -  	   147.11	  1  _
+2 charge
		      B   	     Y
  1	 L 	   114.09	        -  	 14
  2	 G 	   171.11	  1420.63	 13
  3	 A 	   242.15	  1363.61	 12
  4	 H 	   379.21	  1292.58	 11
  5	 Q 	   507.27	  1155.52	 10
  6	 L 	   620.35	  1027.46	  9
  7	 D 	   735.38	   914.37	  8
  8	 S 	   822.41	   799.35	  7
  9	 Y 	   985.47	   712.31	  6
 10	 S 	  1072.51	   549.25	  5
 11	 E 	  1201.55	   462.22	  4
 12	 D 	  1316.58	   333.18	  3
 13	 A 	  1387.61	   218.15	  2
 14	 K 	        -  	   147.11	  1  _
```

**25. IPI00021302 Sushi domain-containing protein 2**

| Sequence | Charge | M+H+ | XCorr | DeltaCn |
| --- | --- | --- | --- | --- |
| K.MSSPTDASVICR.F | 2 | 1324.48 | 3.031 | 0.596 |

+1 charge

```
		      B   	     Y
  1	 M 	   132.05	        -  	 12
  2	 S 	   219.08	  1192.70	 11
  3	 S 	   306.11	  1105.67	 10
  4	 P 	   403.16	  1018.63	  9
  5	 T 	   504.21	   921.58	  8
  6	 D 	   619.24	   820.53	  7
  7	 A 	   690.28	   705.51	  6
  8	 S 	   777.31	   634.47	  5
  9	 V 	   876.38	   547.44	  4
 10	 I 	   989.46	   448.37	  3
 11	 C 	  1149.63	   335.28	  2
 12	 R 	        -  	   175.12	  1  _
+2 charge
		      B   	     Y
  1	 M 	    66.53	        -  	 12
  2	 S 	   110.04	   596.85	 11
  3	 S 	   153.56	   553.34	 10
  4	 P 	   202.09	   509.82	  9
  5	 T 	   252.61	   461.29	  8
  6	 D 	   310.12	   410.77	  7
  7	 A 	   345.64	   353.26	  6
  8	 S 	   389.16	   317.74	  5
  9	 V 	   438.69	   274.22	  4
 10	 I 	   495.23	   224.69	  3
 11	 C 	   575.32	   168.15	  2
 12	 R 	        -  	    88.06	  1  _
```

**26. IPI00006971 Isoform 1 of Endosialin**

| Sequence | Charge | M+H+ | XCorr | DeltaCn |
| --- | --- | --- | --- | --- |
| R.TPEEAQRVDSLVGAGPASR.L | 3 | 1941.092 | 4.149 | 0.481 |

+1 charge

```
		      B   	     Y
  1	 T 	   102.05	        -  	 19
  2	 P 	   199.11	  1838.94	 18
  3	 E 	   328.15	  1741.88	 17
  4	 E 	   457.19	  1612.84	 16
  5	 A 	   528.23	  1483.80	 15
  6	 Q 	   656.29	  1412.76	 14
  7	 R 	   812.39	  1284.70	 13
  8	 V 	   911.46	  1128.60	 12
  9	 D 	  1026.49	  1029.53	 11
 10	 S 	  1113.52	   914.51	 10
 11	 L 	  1226.60	   827.47	  9
 12	 V 	  1325.67	   714.39	  8
 13	 G 	  1382.69	   615.32	  7
 14	 A 	  1453.73	   558.30	  6
 15	 G 	  1510.75	   487.26	  5
 16	 P 	  1607.80	   430.24	  4
 17	 A 	  1678.84	   333.19	  3
 18	 S 	  1765.87	   262.15	  2
 19	 R 	        -  	   175.12	  1  _
+2 charge
		      B   	     Y
  1	 T 	    51.53	        -  	 19
  2	 P 	   100.06	   919.97	 18
  3	 E 	   164.58	   871.45	 17
  4	 E 	   229.10	   806.92	 16
  5	 A 	   264.62	   742.40	 15
  6	 Q 	   328.65	   706.88	 14
  7	 R 	   406.70	   642.85	 13
  8	 V 	   456.23	   564.80	 12
  9	 D 	   513.75	   515.27	 11
 10	 S 	   557.26	   457.76	 10
 11	 L 	   613.80	   414.24	  9
 12	 V 	   663.34	   357.70	  8
 13	 G 	   691.85	   308.16	  7
 14	 A 	   727.37	   279.65	  6
 15	 G 	   755.88	   244.13	  5
 16	 P 	   804.40	   215.62	  4
 17	 A 	   839.92	   167.10	  3
 18	 S 	   883.44	   131.58	  2
 19	 R 	        -  	    88.06	  1  _


+3 charge
		      B   	     Y
  1	 T 	    34.69	        -  	 19
  2	 P 	    67.04	   613.65	 18
  3	 E 	   110.05	   581.30	 17
  4	 E 	   153.07	   538.28	 16
  5	 A 	   176.75	   495.27	 15
  6	 Q 	   219.43	   471.59	 14
  7	 R 	   271.47	   428.91	 13
  8	 V 	   304.49	   376.87	 12
  9	 D 	   342.83	   343.85	 11
 10	 S 	   371.84	   305.51	 10
 11	 L 	   409.54	   276.50	  9
 12	 V 	   442.56	   238.80	  8
 13	 G 	   461.57	   205.78	  7
 14	 A 	   485.25	   186.77	  6
 15	 G 	   504.25	   163.09	  5
 16	 P 	   536.61	   144.09	  4
 17	 A 	   560.28	   111.73	  3
 18	 S 	   589.30	    88.06	  2
 19	 R 	        -  	    59.04	  1  _
```

**27. IPI00295777 Glycerol-3-phosphate dehydrogenase [NAD+], cytoplasmic**

| Sequence | Charge | M+H+ | XCorr | DeltaCn |
| --- | --- | --- | --- | --- |
| K.IVGGNAAQLAQFDPR.V | 2 | 1557.736 | 3.161 | 0.411 |

+1 charge

```
		      B   	     Y
  1	 I 	   114.09	        -  	 15
  2	 V 	   213.16	  1443.73	 14
  3	 G 	   270.18	  1344.67	 13
  4	 G 	   327.20	  1287.64	 12
  5	 N 	   441.25	  1230.62	 11
  6	 A 	   512.28	  1116.58	 10
  7	 A 	   583.32	  1045.54	  9
  8	 Q 	   711.38	   974.51	  8
  9	 L 	   824.46	   846.45	  7
 10	 A 	   895.50	   733.36	  6
 11	 Q 	  1023.56	   662.33	  5
 12	 F 	  1170.63	   534.27	  4
 13	 D 	  1285.65	   387.20	  3
 14	 P 	  1382.71	   272.17	  2
 15	 R 	        -  	   175.12	  1  _
+2 charge
		      B   	     Y
  1	 I 	    57.55	        -  	 15
  2	 V 	   107.08	   722.37	 14
  3	 G 	   135.59	   672.84	 13
  4	 G 	   164.10	   644.33	 12
  5	 N 	   221.13	   615.81	 11
  6	 A 	   256.64	   558.79	 10
  7	 A 	   292.16	   523.27	  9
  8	 Q 	   356.19	   487.76	  8
  9	 L 	   412.73	   423.73	  7
 10	 A 	   448.25	   367.19	  6
 11	 Q 	   512.28	   331.67	  5
 12	 F 	   585.82	   267.64	  4
 13	 D 	   643.33	   194.10	  3
 14	 P 	   691.86	   136.59	  2
 15	 R 	        -  	    88.06	  1  _
```

**28. IPI00414888 Isoform 2 of ICOS ligand**

| Sequence | Charge | M+H+ | XCorr | DeltaCn |
| --- | --- | --- | --- | --- |
| R.ALMSPAGMLR.G | 2 | 1047.321 | 2.895 | 0.293 |

+1 charge

```
		      B   	     Y
  1	 A 	    72.04	        -  	 10
  2	 L 	   185.13	   975.51	  9
  3	 M 	   316.17	   862.43	  8
  4	 S 	   403.20	   731.39	  7
  5	 P 	   500.25	   644.35	  6
  6	 A 	   571.29	   547.30	  5
  7	 G 	   628.31	   476.26	  4
  8	 M 	   759.35	   419.24	  3
  9	 L 	   872.44	   288.20	  2
 10	 R 	        -  	   175.12	  1  _
+2 charge
		      B   	     Y
  1	 A 	    36.53	        -  	 10
  2	 L 	    93.07	   488.26	  9
  3	 M 	   158.59	   431.72	  8
  4	 S 	   202.10	   366.20	  7
  5	 P 	   250.63	   322.68	  6
  6	 A 	   286.15	   274.15	  5
  7	 G 	   314.66	   238.64	  4
  8	 M 	   380.18	   210.13	  3
  9	 L 	   436.72	   144.61	  2
 10	 R 	        -  	    88.06	  1  _
```

**29. IPI00382488 Ig heavy chain V-III region HIL**

| Sequence | Charge | M+H+ | XCorr | DeltaCn |
| --- | --- | --- | --- | --- |
| - .QVKLVQAGGGVVQPGR.S | 2 | 1593.856 | 3.346 | 0.379 |

+1 charge

```
		      B   	     Y
  1	 Q 	   129.07	        -  	 16
  2	 V 	   228.13	  1464.86	 15
  3	 K 	   356.23	  1365.80	 14
  4	 L 	   469.31	  1237.70	 13
  5	 V 	   568.38	  1124.62	 12
  6	 Q 	   696.44	  1025.55	 11
  7	 A 	   767.48	   897.49	 10
  8	 G 	   824.50	   826.45	  9
  9	 G 	   881.52	   769.43	  8
 10	 G 	   938.54	   712.41	  7
 11	 V 	  1037.61	   655.39	  6
 12	 V 	  1136.68	   556.32	  5
 13	 Q 	  1264.74	   457.25	  4
 14	 P 	  1361.79	   329.19	  3
 15	 G 	  1418.81	   232.14	  2
 16	 R 	        -  	   175.12	  1  _
+2 charge
		      B   	     Y
  1	 Q 	    65.04	        -  	 16
  2	 V 	   114.57	   732.94	 15
  3	 K 	   178.62	   683.40	 14
  4	 L 	   235.16	   619.35	 13
  5	 V 	   284.69	   562.81	 12
  6	 Q 	   348.72	   513.28	 11
  7	 A 	   384.24	   449.25	 10
  8	 G 	   412.75	   413.73	  9
  9	 G 	   441.26	   385.22	  8
 10	 G 	   469.77	   356.71	  7
 11	 V 	   519.31	   328.20	  6
 12	 V 	   568.84	   278.66	  5
 13	 Q 	   632.87	   229.13	  4
 14	 P 	   681.40	   165.10	  3
 15	 G 	   709.91	   116.57	  2
 16	 R 	        -  	    88.06	  1  _
```

**30. IPI00002435 CD27 antigen**

| Sequence | Charge | M+H+ | XCorr | DeltaCn |
| --- | --- | --- | --- | --- |
| K.LCCQMCEPGTFLVK.D | 2 | 1744.027 | 4.772 | 0.573 |

+1 charge

```
		      B   	     Y
  1	 L 	   114.09	        -  	 14
  2	 C 	   274.26	  1630.09	 13
  3	 C 	   434.42	  1469.93	 12
  4	 Q 	   562.48	  1309.76	 11
  5	 M 	   693.52	  1181.70	 10
  6	 C 	   853.69	  1050.66	  9
  7	 E 	   982.73	   890.50	  8
  8	 P 	  1079.78	   761.46	  7
  9	 G 	  1136.80	   664.40	  6
 10	 T 	  1237.85	   607.38	  5
 11	 F 	  1384.92	   506.33	  4
 12	 L 	  1498.00	   359.27	  3
 13	 V 	  1597.07	   246.18	  2
 14	 K 	        -  	   147.11	  1  _
+2 charge
		      B   	     Y
  1	 L 	    57.55	        -  	 14
  2	 C 	   137.63	   815.55	 13
  3	 C 	   217.71	   735.47	 12
  4	 Q 	   281.74	   655.38	 11
  5	 M 	   347.26	   591.36	 10
  6	 C 	   427.35	   525.84	  9
  7	 E 	   491.87	   445.75	  8
  8	 P 	   540.39	   381.23	  7
  9	 G 	   568.91	   332.71	  6
 10	 T 	   619.43	   304.19	  5
 11	 F 	   692.96	   253.67	  4
 12	 L 	   749.51	   180.14	  3
 13	 V 	   799.04	   123.59	  2
 14	 K 	        -  	    74.06	  1  _
```

**31. IPI00385252 Ig kappa chain V-III region GOL**

| Sequence | Charge | M+H+ | XCorr | DeltaCn |
| --- | --- | --- | --- | --- |
| - .EIVLTQSPGTLSLSPGER.A | 2 | 1885.108 | 5.608 | 0.573 |

+1 charge

```
		      B   	     Y
  1	 E 	   130.05	        -  	 18
  2	 I 	   243.13	  1754.96	 17
  3	 V 	   342.20	  1641.88	 16
  4	 L 	   455.29	  1542.81	 15
  5	 T 	   556.33	  1429.73	 14
  6	 Q 	   684.39	  1328.68	 13
  7	 S 	   771.42	  1200.62	 12
  8	 P 	   868.48	  1113.59	 11
  9	 G 	   925.50	  1016.54	 10
 10	 T 	  1026.55	   959.52	  9
 11	 L 	  1139.63	   858.47	  8
 12	 S 	  1226.66	   745.38	  7
 13	 L 	  1339.75	   658.35	  6
 14	 S 	  1426.78	   545.27	  5
 15	 P 	  1523.83	   458.24	  4
 16	 G 	  1580.85	   361.18	  3
 17	 E 	  1709.90	   304.16	  2
 18	 R 	        -  	   175.12	  1  _
+2 charge
		      B   	     Y
  1	 E 	    65.53	        -  	 18
  2	 I 	   122.07	   877.99	 17
  3	 V 	   171.60	   821.44	 16
  4	 L 	   228.15	   771.91	 15
  5	 T 	   278.67	   715.37	 14
  6	 Q 	   342.70	   664.84	 13
  7	 S 	   386.22	   600.81	 12
  8	 P 	   434.74	   557.30	 11
  9	 G 	   463.25	   508.77	 10
 10	 T 	   513.78	   480.26	  9
 11	 L 	   570.32	   429.74	  8
 12	 S 	   613.83	   373.20	  7
 13	 L 	   670.38	   329.68	  6
 14	 S 	   713.89	   273.14	  5
 15	 P 	   762.42	   229.62	  4
 16	 G 	   790.93	   181.10	  3
 17	 E 	   855.45	   152.58	  2
 18	 R 	        -  	    88.06	  1  _
```

**32. IPI00001754 Junctional adhesion molecule A**

| Sequence | Charge | M+H+ | XCorr | DeltaCn |
| --- | --- | --- | --- | --- |
| K.LIVLVPPSKPTVNIPSSATIGNR.A | 3 | 2374.808 | 3.401 | 0.393 |

+1 charge

```
		      B   	     Y
  1	 L 	   114.09	        -  	 23
  2	 I 	   227.18	  2260.30	 22
  3	 V 	   326.24	  2147.22	 21
  4	 L 	   439.33	  2048.15	 20
  5	 V 	   538.40	  1935.07	 19
  6	 P 	   635.45	  1836.00	 18
  7	 P 	   732.50	  1738.94	 17
  8	 S 	   819.53	  1641.89	 16
  9	 K 	   947.63	  1554.86	 15
 10	 P 	  1044.68	  1426.76	 14
 11	 T 	  1145.73	  1329.71	 13
 12	 V 	  1244.80	  1228.66	 12
 13	 N 	  1358.84	  1129.60	 11
 14	 I 	  1471.92	  1015.55	 10
 15	 P 	  1568.98	   902.47	  9
 16	 S 	  1656.01	   805.42	  8
 17	 S 	  1743.04	   718.38	  7
 18	 A 	  1814.08	   631.35	  6
 19	 T 	  1915.13	   560.32	  5
 20	 I 	  2028.21	   459.27	  4
 21	 G 	  2085.23	   346.18	  3
 22	 N 	  2199.27	   289.16	  2
 23	 R 	        -  	   175.12	  1  _


+2 charge
		      B   	     Y
  1	 L 	    57.55	        -  	 23
  2	 I 	   114.09	  1130.65	 22
  3	 V 	   163.63	  1074.11	 21
  4	 L 	   220.17	  1024.58	 20
  5	 V 	   269.70	   968.04	 19
  6	 P 	   318.23	   918.50	 18
  7	 P 	   366.75	   869.98	 17
  8	 S 	   410.27	   821.45	 16
  9	 K 	   474.32	   777.93	 15
 10	 P 	   522.84	   713.89	 14
 11	 T 	   573.37	   665.36	 13
 12	 V 	   622.90	   614.84	 12
 13	 N 	   679.92	   565.30	 11
 14	 I 	   736.47	   508.28	 10
 15	 P 	   784.99	   451.74	  9
 16	 S 	   828.51	   403.21	  8
 17	 S 	   872.02	   359.70	  7
 18	 A 	   907.54	   316.18	  6
 19	 T 	   958.07	   280.66	  5
 20	 I 	  1014.61	   230.14	  4
 21	 G 	  1043.12	   173.60	  3
 22	 N 	  1100.14	   145.08	  2
 23	 R 	        -  	    88.06	  1  _


+3 charge
		      B   	     Y
  1	 L 	    38.70	        -  	 23
  2	 I 	    76.40	   754.11	 22
  3	 V 	   109.42	   716.41	 21
  4	 L 	   147.11	   683.39	 20
  5	 V 	   180.14	   645.69	 19
  6	 P 	   212.49	   612.67	 18
  7	 P 	   244.84	   580.32	 17
  8	 S 	   273.85	   547.97	 16
  9	 K 	   316.55	   518.96	 15
 10	 P 	   348.90	   476.26	 14
 11	 T 	   382.58	   443.91	 13
 12	 V 	   415.60	   410.23	 12
 13	 N 	   453.62	   377.20	 11
 14	 I 	   491.31	   339.19	 10
 15	 P 	   523.66	   301.49	  9
 16	 S 	   552.67	   269.14	  8
 17	 S 	   581.69	   240.13	  7
 18	 A 	   605.36	   211.12	  6
 19	 T 	   639.05	   187.44	  5
 20	 I 	   676.74	   153.76	  4
 21	 G 	   695.75	   116.07	  3
 22	 N 	   733.76	    97.06	  2
 23	 R 	        -  	    59.04	  1  _
```

**33. IPI00029168 Apolipoprotein(a)**

| Sequence | Charge | M+H+ | XCorr | DeltaCn |
| --- | --- | --- | --- | --- |
| R.TPEYYPNAGLIMNYCR.N | 2 | 1963.196 | 3.548 | 0.601 |

+1 charge

```
		      B   	     Y
  1	 T 	   102.05	        -  	 16
  2	 P 	   199.11	  1860.98	 15
  3	 E 	   328.15	  1763.92	 14
  4	 Y 	   491.21	  1634.88	 13
  5	 Y 	   654.28	  1471.82	 12
  6	 P 	   751.33	  1308.75	 11
  7	 N 	   865.37	  1211.70	 10
  8	 A 	   936.41	  1097.66	  9
  9	 G 	   993.43	  1026.62	  8
 10	 L 	  1106.52	   969.60	  7
 11	 I 	  1219.60	   856.51	  6
 12	 M 	  1350.64	   743.43	  5
 13	 N 	  1464.68	   612.39	  4
 14	 Y 	  1627.75	   498.35	  3
 15	 C 	  1787.91	   335.28	  2
 16	 R 	        -  	   175.12	  1  _
+2 charge
		      B   	     Y
  1	 T 	    51.53	        -  	 16
  2	 P 	   100.06	   930.99	 15
  3	 E 	   164.58	   882.46	 14
  4	 Y 	   246.11	   817.94	 13
  5	 Y 	   327.64	   736.41	 12
  6	 P 	   376.17	   654.88	 11
  7	 N 	   433.19	   606.35	 10
  8	 A 	   468.71	   549.33	  9
  9	 G 	   497.22	   513.81	  8
 10	 L 	   553.76	   485.30	  7
 11	 I 	   610.30	   428.76	  6
 12	 M 	   675.82	   372.22	  5
 13	 N 	   732.85	   306.70	  4
 14	 Y 	   814.38	   249.68	  3
 15	 C 	   894.46	   168.15	  2
 16	 R 	        -  	    88.06	  1  _
```

**34. IPI00065450 Isoform 1 of Putative adenylate kinase-like protein C9orf98**

| Sequence | Charge | M+H+ | XCorr | DeltaCn |
| --- | --- | --- | --- | --- |
| R.KTVPSALLVQLIQERLAEEDCIK.Q | 3 | 2655.078 | 3.629 | 0.218 |

+1 charge

```
		      B   	     Y
  1	 K 	   129.10	        -  	 23
  2	 T 	   230.15	  2525.50	 22
  3	 V 	   329.22	  2424.45	 21
  4	 P 	   426.27	  2325.38	 20
  5	 S 	   513.30	  2228.33	 19
  6	 A 	   584.34	  2141.30	 18
  7	 L 	   697.42	  2070.26	 17
  8	 L 	   810.51	  1957.18	 16
  9	 V 	   909.58	  1844.09	 15
 10	 Q 	  1037.64	  1745.02	 14
 11	 L 	  1150.72	  1616.97	 13
 12	 I 	  1263.80	  1503.88	 12
 13	 Q 	  1391.86	  1390.80	 11
 14	 E 	  1520.90	  1262.74	 10
 15	 R 	  1677.01	  1133.70	  9
 16	 L 	  1790.09	   977.60	  8
 17	 A 	  1861.13	   864.51	  7
 18	 E 	  1990.17	   793.47	  6
 19	 E 	  2119.21	   664.43	  5
 20	 D 	  2234.24	   535.39	  4
 21	 C 	  2394.40	   420.36	  3
 22	 I 	  2507.49	   260.20	  2
 23	 K 	        -  	   147.11	  1  _


+2 charge
		      B   	     Y
  1	 K 	    65.05	        -  	 23
  2	 T 	   115.58	  1263.25	 22
  3	 V 	   165.11	  1212.73	 21
  4	 P 	   213.64	  1163.20	 20
  5	 S 	   257.16	  1114.67	 19
  6	 A 	   292.67	  1071.15	 18
  7	 L 	   349.22	  1035.63	 17
  8	 L 	   405.76	   979.09	 16
  9	 V 	   455.29	   922.55	 15
 10	 Q 	   519.32	   873.02	 14
 11	 L 	   575.86	   808.99	 13
 12	 I 	   632.41	   752.44	 12
 13	 Q 	   696.43	   695.90	 11
 14	 E 	   760.96	   631.87	 10
 15	 R 	   839.01	   567.35	  9
 16	 L 	   895.55	   489.30	  8
 17	 A 	   931.07	   432.76	  7
 18	 E 	   995.59	   397.24	  6
 19	 E 	  1060.11	   332.72	  5
 20	 D 	  1117.62	   268.20	  4
 21	 C 	  1197.71	   210.68	  3
 22	 I 	  1254.25	   130.60	  2
 23	 K 	        -  	    74.06	  1  _


+3 charge
		      B   	     Y
  1	 K 	    43.71	        -  	 23
  2	 T 	    77.39	   842.50	 22
  3	 V 	   110.41	   808.82	 21
  4	 P 	   142.76	   775.80	 20
  5	 S 	   171.77	   743.45	 19
  6	 A 	   195.45	   714.44	 18
  7	 L 	   233.15	   690.76	 17
  8	 L 	   270.84	   653.06	 16
  9	 V 	   303.86	   615.37	 15
 10	 Q 	   346.55	   582.35	 14
 11	 L 	   384.24	   539.66	 13
 12	 I 	   421.94	   501.97	 12
 13	 Q 	   464.63	   464.27	 11
 14	 E 	   507.64	   421.58	 10
 15	 R 	   559.67	   378.57	  9
 16	 L 	   597.37	   326.54	  8
 17	 A 	   621.05	   288.84	  7
 18	 E 	   664.06	   265.16	  6
 19	 E 	   707.08	   222.15	  5
 20	 D 	   745.42	   179.13	  4
 21	 C 	   798.81	   140.79	  3
 22	 I 	   836.50	    87.40	  2
 23	 K 	        -  	    49.71	  1  _
```

**35. IPI00166392 Similar to Cell adhesion molecule 1 precursor**

| Sequence | Charge | M+H+ | XCorr | DeltaCn |
| --- | --- | --- | --- | --- |
| R.YLEVQYKPQVHIQMTYPLQGLTR.E | 3 | 2807.262 | 3.663 | 0.439 |

+1 charge

```
		      B   	     Y
  1	 Y 	   164.07	        -  	 23
  2	 L 	   277.15	  2642.41	 22
  3	 E 	   406.20	  2529.33	 21
  4	 V 	   505.27	  2400.29	 20
  5	 Q 	   633.32	  2301.22	 19
  6	 Y 	   796.39	  2173.16	 18
  7	 K 	   924.48	  2010.10	 17
  8	 P 	  1021.54	  1882.00	 16
  9	 Q 	  1149.59	  1784.95	 15
 10	 V 	  1248.66	  1656.89	 14
 11	 H 	  1385.72	  1557.82	 13
 12	 I 	  1498.81	  1420.76	 12
 13	 Q 	  1626.86	  1307.68	 11
 14	 M 	  1757.90	  1179.62	 10
 15	 T 	  1858.95	  1048.58	  9
 16	 Y 	  2022.02	   947.53	  8
 17	 P 	  2119.07	   784.47	  7
 18	 L 	  2232.15	   687.41	  6
 19	 Q 	  2360.21	   574.33	  5
 20	 G 	  2417.23	   446.27	  4
 21	 L 	  2530.32	   389.25	  3
 22	 T 	  2631.36	   276.17	  2
 23	 R 	        -  	   175.12	  1  _


+2 charge
		      B   	     Y
  1	 Y 	    82.54	        -  	 23
  2	 L 	   139.08	  1321.71	 22
  3	 E 	   203.60	  1265.17	 21
  4	 V 	   253.14	  1200.65	 20
  5	 Q 	   317.17	  1151.11	 19
  6	 Y 	   398.70	  1087.08	 18
  7	 K 	   462.74	  1005.55	 17
  8	 P 	   511.27	   941.50	 16
  9	 Q 	   575.30	   892.98	 15
 10	 V 	   624.83	   828.95	 14
 11	 H 	   693.36	   779.41	 13
 12	 I 	   749.91	   710.88	 12
 13	 Q 	   813.94	   654.34	 11
 14	 M 	   879.46	   590.31	 10
 15	 T 	   929.98	   524.79	  9
 16	 Y 	  1011.51	   474.27	  8
 17	 P 	  1060.04	   392.74	  7
 18	 L 	  1116.58	   344.21	  6
 19	 Q 	  1180.61	   287.67	  5
 20	 G 	  1209.12	   223.64	  4
 21	 L 	  1265.66	   195.13	  3
 22	 T 	  1316.19	   138.59	  2
 23	 R 	        -  	    88.06	  1  _


+3 charge
		      B   	     Y
  1	 Y 	    55.36	        -  	 23
  2	 L 	    93.06	   881.48	 22
  3	 E 	   136.07	   843.78	 21
  4	 V 	   169.09	   800.77	 20
  5	 Q 	   211.78	   767.74	 19
  6	 Y 	   266.13	   725.06	 18
  7	 K 	   308.83	   670.70	 17
  8	 P 	   341.18	   628.00	 16
  9	 Q 	   383.87	   595.65	 15
 10	 V 	   416.89	   552.97	 14
 11	 H 	   462.58	   519.95	 13
 12	 I 	   500.27	   474.26	 12
 13	 Q 	   542.96	   436.56	 11
 14	 M 	   586.64	   393.88	 10
 15	 T 	   620.32	   350.20	  9
 16	 Y 	   674.68	   316.52	  8
 17	 P 	   707.03	   262.16	  7
 18	 L 	   744.72	   229.81	  6
 19	 Q 	   787.41	   192.12	  5
 20	 G 	   806.42	   149.43	  4
 21	 L 	   844.11	   130.42	  3
 22	 T 	   877.79	    92.73	  2
 23	 R 	        -  	    59.04	  1  _
```

**36. IPI00745814 hypothetical protein XP_002343109**

| Sequence | Charge | M+H+ | XCorr | DeltaCn |
| --- | --- | --- | --- | --- |
| R.MDVNLCSGILTSAEAGAASR.Q | 2 | 2024.236 | 3.19 | 0.228 |

+1 charge

```
		      B   	     Y
  1	 M 	   132.05	        -  	 20
  2	 D 	   247.07	  1892.05	 19
  3	 V 	   346.14	  1777.03	 18
  4	 N 	   460.19	  1677.96	 17
  5	 L 	   573.27	  1563.91	 16
  6	 C 	   733.44	  1450.83	 15
  7	 S 	   820.47	  1290.66	 14
  8	 G 	   877.49	  1203.63	 13
  9	 I 	   990.57	  1146.61	 12
 10	 L 	  1103.66	  1033.53	 11
 11	 T 	  1204.70	   920.44	 10
 12	 S 	  1291.74	   819.40	  9
 13	 A 	  1362.77	   732.36	  8
 14	 E 	  1491.82	   661.33	  7
 15	 A 	  1562.85	   532.28	  6
 16	 G 	  1619.87	   461.25	  5
 17	 A 	  1690.91	   404.23	  4
 18	 A 	  1761.95	   333.19	  3
 19	 S 	  1848.98	   262.15	  2
 20	 R 	        -  	   175.12	  1  _
+2 charge
		      B   	     Y
  1	 M 	    66.53	        -  	 20
  2	 D 	   124.04	   946.53	 19
  3	 V 	   173.58	   889.02	 18
  4	 N 	   230.60	   839.48	 17
  5	 L 	   287.14	   782.46	 16
  6	 C 	   367.22	   725.92	 15
  7	 S 	   410.74	   645.84	 14
  8	 G 	   439.25	   602.32	 13
  9	 I 	   495.79	   573.81	 12
 10	 L 	   552.33	   517.27	 11
 11	 T 	   602.86	   460.73	 10
 12	 S 	   646.37	   410.20	  9
 13	 A 	   681.89	   366.69	  8
 14	 E 	   746.41	   331.17	  7
 15	 A 	   781.93	   266.65	  6
 16	 G 	   810.44	   231.13	  5
 17	 A 	   845.96	   202.62	  4
 18	 A 	   881.48	   167.10	  3
 19	 S 	   924.99	   131.58	  2
 20	 R 	        -  	    88.06	  1  _
```

**37. IPI00335168 Isoform Non-muscle of Myosin light polypeptide 6**

| Sequence | Charge | M+H+ | XCorr | DeltaCn |
| --- | --- | --- | --- | --- |
| K.VLDFEHFLPMLQTVAK.N | 2 | 1889.25 | 3.337 | 0.439 |

+1 charge

```
		      B   	     Y
  1	 V 	   100.08	        -  	 16
  2	 L 	   213.16	  1788.94	 15
  3	 D 	   328.19	  1675.85	 14
  4	 F 	   475.26	  1560.82	 13
  5	 E 	   604.30	  1413.76	 12
  6	 H 	   741.36	  1284.71	 11
  7	 F 	   888.43	  1147.65	 10
  8	 L 	  1001.51	  1000.59	  9
  9	 P 	  1098.56	   887.50	  8
 10	 M 	  1229.60	   790.45	  7
 11	 L 	  1342.69	   659.41	  6
 12	 Q 	  1470.74	   546.32	  5
 13	 T 	  1571.79	   418.27	  4
 14	 V 	  1670.86	   317.22	  3
 15	 A 	  1741.90	   218.15	  2
 16	 K 	        -  	   147.11	  1  _
+2 charge
		      B   	     Y
  1	 V 	    50.54	        -  	 16
  2	 L 	   107.08	   894.97	 15
  3	 D 	   164.60	   838.43	 14
  4	 F 	   238.13	   780.92	 13
  5	 E 	   302.65	   707.38	 12
  6	 H 	   371.18	   642.86	 11
  7	 F 	   444.72	   574.33	 10
  8	 L 	   501.26	   500.80	  9
  9	 P 	   549.78	   444.25	  8
 10	 M 	   615.30	   395.73	  7
 11	 L 	   671.85	   330.21	  6
 12	 Q 	   735.88	   273.67	  5
 13	 T 	   786.40	   209.64	  4
 14	 V 	   835.93	   159.11	  3
 15	 A 	   871.45	   109.58	  2
 16	 K 	        -  	    74.06	  1  _
```

**38. IPI00885179 Isoform 1 of Mucin-20**

| Sequence | Charge | M+H+ | XCorr | DeltaCn |
| --- | --- | --- | --- | --- |
| R.ASESSASSDGPHPVITPSR.A | 2 | 1882.966 | 2.964 | 0.455 |

+1 charge

```
		      B   	     Y
  1	 A 	    72.04	        -  	 19
  2	 S 	   159.08	  1810.86	 18
  3	 E 	   288.12	  1723.82	 17
  4	 S 	   375.15	  1594.78	 16
  5	 S 	   462.18	  1507.75	 15
  6	 A 	   533.22	  1420.72	 14
  7	 S 	   620.25	  1349.68	 13
  8	 S 	   707.28	  1262.65	 12
  9	 D 	   822.31	  1175.62	 11
 10	 G 	   879.33	  1060.59	 10
 11	 P 	   976.39	  1003.57	  9
 12	 H 	  1113.44	   906.52	  8
 13	 P 	  1210.50	   769.46	  7
 14	 V 	  1309.57	   672.40	  6
 15	 I 	  1422.65	   573.34	  5
 16	 T 	  1523.70	   460.25	  4
 17	 P 	  1620.75	   359.20	  3
 18	 S 	  1707.78	   262.15	  2
 19	 R 	        -  	   175.12	  1  _
+2 charge
		      B   	     Y
  1	 A 	    36.53	        -  	 19
  2	 S 	    80.04	   905.93	 18
  3	 E 	   144.56	   862.42	 17
  4	 S 	   188.08	   797.89	 16
  5	 S 	   231.60	   754.38	 15
  6	 A 	   267.11	   710.86	 14
  7	 S 	   310.63	   675.34	 13
  8	 S 	   354.15	   631.83	 12
  9	 D 	   411.66	   588.31	 11
 10	 G 	   440.17	   530.80	 10
 11	 P 	   488.70	   502.29	  9
 12	 H 	   557.23	   453.76	  8
 13	 P 	   605.75	   385.23	  7
 14	 V 	   655.29	   336.71	  6
 15	 I 	   711.83	   287.17	  5
 16	 T 	   762.35	   230.63	  4
 17	 P 	   810.88	   180.11	  3
 18	 S 	   854.39	   131.58	  2
 19	 R 	        -  	    88.06	  1  _
```

**39. IPI00064262 Protocadherin-16**

| Sequence | Charge | M+H+ | XCorr | DeltaCn |
| --- | --- | --- | --- | --- |
| R.VADINDHAPAFPQAR.A | 2 | 1622.766 | 3.16 | 0.324 |

+1 charge

```
		      B   	     Y
  1	 V 	   100.08	        -  	 15
  2	 A 	   171.11	  1522.74	 14
  3	 D 	   286.14	  1451.70	 13
  4	 I 	   399.22	  1336.68	 12
  5	 N 	   513.27	  1223.59	 11
  6	 D 	   628.29	  1109.55	 10
  7	 H 	   765.35	   994.52	  9
  8	 A 	   836.39	   857.46	  8
  9	 P 	   933.44	   786.43	  7
 10	 A 	  1004.48	   689.37	  6
 11	 F 	  1151.55	   618.34	  5
 12	 P 	  1248.60	   471.27	  4
 13	 Q 	  1376.66	   374.21	  3
 14	 A 	  1447.70	   246.16	  2
 15	 R 	        -  	   175.12	  1  _
+2 charge
		      B   	     Y
  1	 V 	    50.54	        -  	 15
  2	 A 	    86.06	   761.87	 14
  3	 D 	   143.57	   726.35	 13
  4	 I 	   200.12	   668.84	 12
  5	 N 	   257.14	   612.30	 11
  6	 D 	   314.65	   555.28	 10
  7	 H 	   383.18	   497.76	  9
  8	 A 	   418.70	   429.24	  8
  9	 P 	   467.22	   393.72	  7
 10	 A 	   502.74	   345.19	  6
 11	 F 	   576.28	   309.67	  5
 12	 P 	   624.80	   236.14	  4
 13	 Q 	   688.83	   187.61	  3
 14	 A 	   724.35	   123.58	  2
 15	 R 	        -  	    88.06	  1  _
```

**40. IPI00477714 V3-4 protein**

| Sequence | Charge | M+H+ | XCorr | DeltaCn |
| --- | --- | --- | --- | --- |
| R.SSGVPDRFSGSILGNK.A | 2 | 1621.776 | 3.333 | 0.257 |

+1 charge

```
		      B   	     Y
  1	 S 	    88.04	        -  	 16
  2	 S 	   175.07	  1533.80	 15
  3	 G 	   232.09	  1446.77	 14
  4	 V 	   331.16	  1389.75	 13
  5	 P 	   428.21	  1290.68	 12
  6	 D 	   543.24	  1193.63	 11
  7	 R 	   699.34	  1078.60	 10
  8	 F 	   846.41	   922.50	  9
  9	 S 	   933.44	   775.43	  8
 10	 G 	   990.46	   688.40	  7
 11	 S 	  1077.50	   631.38	  6
 12	 I 	  1190.58	   544.35	  5
 13	 L 	  1303.66	   431.26	  4
 14	 G 	  1360.69	   318.18	  3
 15	 N 	  1474.73	   261.16	  2
 16	 K 	        -  	   147.11	  1  _
+2 charge
		      B   	     Y
  1	 S 	    44.52	        -  	 16
  2	 S 	    88.04	   767.40	 15
  3	 G 	   116.55	   723.89	 14
  4	 V 	   166.08	   695.38	 13
  5	 P 	   214.61	   645.84	 12
  6	 D 	   272.12	   597.32	 11
  7	 R 	   350.17	   539.80	 10
  8	 F 	   423.71	   461.75	  9
  9	 S 	   467.22	   388.22	  8
 10	 G 	   495.74	   344.70	  7
 11	 S 	   539.25	   316.19	  6
 12	 I 	   595.79	   272.68	  5
 13	 L 	   652.34	   216.13	  4
 14	 G 	   680.85	   159.59	  3
 15	 N 	   737.87	   131.08	  2
 16	 K 	        -  	    74.06	  1  _
```

**41. IPI00001662 OPCML protein**

| Sequence | Charge | M+H+ | XCorr | DeltaCn |
| --- | --- | --- | --- | --- |
| K.ITVNYPPYISK.A | 2 | 1295.508 | 3.171 | 0.348 |

+1 charge

```
		      B   	     Y
  1	 I 	   114.09	        -  	 11
  2	 T 	   215.14	  1181.62	 10
  3	 V 	   314.21	  1080.57	  9
  4	 N 	   428.25	   981.50	  8
  5	 Y 	   591.31	   867.46	  7
  6	 P 	   688.37	   704.40	  6
  7	 P 	   785.42	   607.34	  5
  8	 Y 	   948.48	   510.29	  4
  9	 I 	  1061.57	   347.23	  3
 10	 S 	  1148.60	   234.14	  2
 11	 K 	        -  	   147.11	  1  _
+2 charge
		      B   	     Y
  1	 I 	    57.55	        -  	 11
  2	 T 	   108.07	   591.31	 10
  3	 V 	   157.61	   540.79	  9
  4	 N 	   214.63	   491.26	  8
  5	 Y 	   296.16	   434.23	  7
  6	 P 	   344.69	   352.70	  6
  7	 P 	   393.21	   304.18	  5
  8	 Y 	   474.74	   255.65	  4
  9	 I 	   531.29	   174.12	  3
 10	 S 	   574.80	   117.58	  2
 11	 K 	        -  	    74.06	  1  _
```

**42. IPI00183445 Isoform 1 of Latrophilin-1**

| Sequence | Charge | M+H+ | XCorr | DeltaCn |
| --- | --- | --- | --- | --- |
| R.ELACEGYPIELR.C | 2 | 1450.612 | 3.346 | 0.462 |

+1 charge

```
		      B   	     Y
  1	 E 	   130.05	        -  	 12
  2	 L 	   243.13	  1320.80	 11
  3	 A 	   314.17	  1207.71	 10
  4	 C 	   474.34	  1136.68	  9
  5	 E 	   603.38	   976.51	  8
  6	 G 	   660.40	   847.47	  7
  7	 Y 	   823.46	   790.45	  6
  8	 P 	   920.52	   627.38	  5
  9	 I 	  1033.60	   530.33	  4
 10	 E 	  1162.64	   417.25	  3
 11	 L 	  1275.73	   288.20	  2
 12	 R 	        -  	   175.12	  1  _
+2 charge
		      B   	     Y
  1	 E 	    65.53	        -  	 12
  2	 L 	   122.07	   660.90	 11
  3	 A 	   157.59	   604.36	 10
  4	 C 	   237.67	   568.84	  9
  5	 E 	   302.19	   488.76	  8
  6	 G 	   330.70	   424.24	  7
  7	 Y 	   412.24	   395.73	  6
  8	 P 	   460.76	   314.19	  5
  9	 I 	   517.30	   265.67	  4
 10	 E 	   581.83	   209.13	  3
 11	 L 	   638.37	   144.61	  2
 12	 R 	        -  	    88.06	  1  _
```

**43. IPI00003865 Isoform 1 of Heat shock cognate 71 kDa protein**

| Sequence | Charge | M+H+ | XCorr | DeltaCn |
| --- | --- | --- | --- | --- |
| R.ARFEELNADLFR.G | 2 | 1481.638 | 3.092 | 0.396 |

+1 charge

```
		      B   	     Y
  1	 A 	    72.04	        -  	 12
  2	 R 	   228.15	  1409.72	 11
  3	 F 	   375.21	  1253.62	 10
  4	 E 	   504.26	  1106.55	  9
  5	 E 	   633.30	   977.51	  8
  6	 L 	   746.38	   848.46	  7
  7	 N 	   860.43	   735.38	  6
  8	 A 	   931.46	   621.34	  5
  9	 D 	  1046.49	   550.30	  4
 10	 L 	  1159.57	   435.27	  3
 11	 F 	  1306.64	   322.19	  2
 12	 R 	        -  	   175.12	  1  _
+2 charge
		      B   	     Y
  1	 A 	    36.53	        -  	 12
  2	 R 	   114.58	   705.36	 11
  3	 F 	   188.11	   627.31	 10
  4	 E 	   252.63	   553.78	  9
  5	 E 	   317.15	   489.26	  8
  6	 L 	   373.70	   424.73	  7
  7	 N 	   430.72	   368.19	  6
  8	 A 	   466.24	   311.17	  5
  9	 D 	   523.75	   275.65	  4
 10	 L 	   580.29	   218.14	  3
 11	 F 	   653.82	   161.60	  2
 12	 R 	        -  	    88.06	  1  _
```

**44. IPI00015199 T-cell antigen CD7**

| Sequence | Charge | M+H+ | XCorr | DeltaCn |
| --- | --- | --- | --- | --- |
| R.QLGPQPQDIIYYEDGVVPTTDRR.F | 3 | 2661.908 | 3.664 | 0.408 |

+1 charge

```
		      B   	     Y
  1	 Q 	   129.07	        -  	 23
  2	 L 	   242.15	  2532.27	 22
  3	 G 	   299.17	  2419.19	 21
  4	 P 	   396.22	  2362.17	 20
  5	 Q 	   524.28	  2265.11	 19
  6	 P 	   621.34	  2137.06	 18
  7	 Q 	   749.39	  2040.00	 17
  8	 D 	   864.42	  1911.94	 16
  9	 I 	   977.51	  1796.92	 15
 10	 I 	  1090.59	  1683.83	 14
 11	 Y 	  1253.65	  1570.75	 13
 12	 Y 	  1416.72	  1407.69	 12
 13	 E 	  1545.76	  1244.62	 11
 14	 D 	  1660.79	  1115.58	 10
 15	 G 	  1717.81	  1000.55	  9
 16	 V 	  1816.88	   943.53	  8
 17	 V 	  1915.94	   844.46	  7
 18	 P 	  2013.00	   745.40	  6
 19	 T 	  2114.04	   648.34	  5
 20	 T 	  2215.09	   547.29	  4
 21	 D 	  2330.12	   446.25	  3
 22	 R 	  2486.22	   331.22	  2
 23	 R 	        -  	   175.12	  1  _


+2 charge
		      B   	     Y
  1	 Q 	    65.04	        -  	 23
  2	 L 	   121.58	  1266.64	 22
  3	 G 	   150.09	  1210.10	 21
  4	 P 	   198.62	  1181.59	 20
  5	 Q 	   262.64	  1133.06	 19
  6	 P 	   311.17	  1069.03	 18
  7	 Q 	   375.20	  1020.51	 17
  8	 D 	   432.71	   956.48	 16
  9	 I 	   489.26	   898.96	 15
 10	 I 	   545.80	   842.42	 14
 11	 Y 	   627.33	   785.88	 13
 12	 Y 	   708.86	   704.35	 12
 13	 E 	   773.38	   622.82	 11
 14	 D 	   830.90	   558.29	 10
 15	 G 	   859.41	   500.78	  9
 16	 V 	   908.94	   472.27	  8
 17	 V 	   958.48	   422.74	  7
 18	 P 	  1007.00	   373.20	  6
 19	 T 	  1057.53	   324.67	  5
 20	 T 	  1108.05	   274.15	  4
 21	 D 	  1165.56	   223.63	  3
 22	 R 	  1243.61	   166.11	  2
 23	 R 	        -  	    88.06	  1  _
+3 charge
		      B   	     Y
  1	 Q 	    43.69	        -  	 23
  2	 L 	    81.39	   844.76	 22
  3	 G 	   100.40	   807.07	 21
  4	 P 	   132.75	   788.06	 20
  5	 Q 	   175.43	   755.71	 19
  6	 P 	   207.78	   713.02	 18
  7	 Q 	   250.47	   680.67	 17
  8	 D 	   288.81	   637.99	 16
  9	 I 	   326.51	   599.64	 15
 10	 I 	   364.20	   561.95	 14
 11	 Y 	   418.56	   524.25	 13
 12	 Y 	   472.91	   469.90	 12
 13	 E 	   515.92	   415.55	 11
 14	 D 	   554.27	   372.53	 10
 15	 G 	   573.27	   334.19	  9
 16	 V 	   606.30	   315.18	  8
 17	 V 	   639.32	   282.16	  7
 18	 P 	   671.67	   249.14	  6
 19	 T 	   705.35	   216.79	  5
 20	 T 	   739.04	   183.10	  4
 21	 D 	   777.38	   149.42	  3
 22	 R 	   829.41	   111.08	  2
 23	 R 	        -  	    59.04	  1  _
```

**45. IPI00297420 Frizzled-4 precursor**

| Sequence | Charge | M+H+ | XCorr | DeltaCn |
| --- | --- | --- | --- | --- |
| K.INIPIGPCGGMCLSVK.R | 2 | 1717.054 | 3.61 | 0.475 |

+1 charge

```
		      B   	     Y
  1	 I 	   114.09	        -  	 16
  2	 N 	   228.13	  1603.05	 15
  3	 I 	   341.22	  1489.01	 14
  4	 P 	   438.27	  1375.92	 13
  5	 I 	   551.36	  1278.87	 12
  6	 G 	   608.38	  1165.79	 11
  7	 P 	   705.43	  1108.76	 10
  8	 C 	   865.59	  1011.71	  9
  9	 G 	   922.62	   851.55	  8
 10	 G 	   979.64	   794.52	  7
 11	 M 	  1110.68	   737.50	  6
 12	 C 	  1270.84	   606.46	  5
 13	 L 	  1383.93	   446.30	  4
 14	 S 	  1470.96	   333.21	  3
 15	 V 	  1570.03	   246.18	  2
 16	 K 	        -  	   147.11	  1  _
+2 charge
		      B   	     Y
  1	 I 	    57.55	        -  	 16
  2	 N 	   114.57	   802.03	 15
  3	 I 	   171.11	   745.01	 14
  4	 P 	   219.64	   688.46	 13
  5	 I 	   276.18	   639.94	 12
  6	 G 	   304.69	   583.40	 11
  7	 P 	   353.22	   554.89	 10
  8	 C 	   433.30	   506.36	  9
  9	 G 	   461.81	   426.28	  8
 10	 G 	   490.32	   397.77	  7
 11	 M 	   555.84	   369.26	  6
 12	 C 	   635.93	   303.73	  5
 13	 L 	   692.47	   223.65	  4
 14	 S 	   735.98	   167.11	  3
 15	 V 	   785.52	   123.59	  2
 16	 K 	        -  	    74.06	  1  _
```

**46. IPI00218163 Isoform 2 of Mucin-1**

| Sequence | Charge | M+H+ | XCorr | DeltaCn |
| --- | --- | --- | --- | --- |
| R.DISEMFLQIYK.Q | 2 | 1387.626 | 3.365 | 0.545 |

+1 charge

```
		      B   	     Y
  1	 D 	   116.03	        -  	 11
  2	 I 	   229.12	  1271.67	 10
  3	 S 	   316.15	  1158.59	  9
  4	 E 	   445.19	  1071.55	  8
  5	 M 	   576.23	   942.51	  7
  6	 F 	   723.30	   811.47	  6
  7	 L 	   836.39	   664.40	  5
  8	 Q 	   964.44	   551.32	  4
  9	 I 	  1077.53	   423.26	  3
 10	 Y 	  1240.59	   310.18	  2
 11	 K 	        -  	   147.11	  1  _
+2 charge
		      B   	     Y
  1	 D 	    58.52	        -  	 11
  2	 I 	   115.06	   636.34	 10
  3	 S 	   158.58	   579.80	  9
  4	 E 	   223.10	   536.28	  8
  5	 M 	   288.62	   471.76	  7
  6	 F 	   362.15	   406.24	  6
  7	 L 	   418.70	   332.71	  5
  8	 Q 	   482.73	   276.16	  4
  9	 I 	   539.27	   212.13	  3
 10	 Y 	   620.80	   155.59	  2
 11	 K 	        -  	    74.06	  1  _
```

**47. IPI00016513 Ras-related protein Rab-10**

| Sequence | Charge | M+H+ | XCorr | DeltaCn |
| --- | --- | --- | --- | --- |
| K.AFLTLAEDILR.K | 2 | 1262.48 | 3.025 | 0.46 |

+1 charge

```
		      B   	     Y
  1	 A 	    72.04	        -  	 11
  2	 F 	   219.11	  1190.68	 10
  3	 L 	   332.20	  1043.61	  9
  4	 T 	   433.24	   930.53	  8
  5	 L 	   546.33	   829.48	  7
  6	 A 	   617.37	   716.39	  6
  7	 E 	   746.41	   645.36	  5
  8	 D 	   861.44	   516.31	  4
  9	 I 	   974.52	   401.29	  3
 10	 L 	  1087.60	   288.20	  2
 11	 R 	        -  	   175.12	  1  _
+2 charge
		      B   	     Y
  1	 A 	    36.53	        -  	 11
  2	 F 	   110.06	   595.84	 10
  3	 L 	   166.60	   522.31	  9
  4	 T 	   217.13	   465.77	  8
  5	 L 	   273.67	   415.24	  7
  6	 A 	   309.19	   358.70	  6
  7	 E 	   373.71	   323.18	  5
  8	 D 	   431.22	   258.66	  4
  9	 I 	   487.76	   201.15	  3
 10	 L 	   544.31	   144.61	  2
 11	 R 	        -  	    88.06	  1  _
```

**48. IPI00292858 Thymidine phosphorylase**

| Sequence | Charge | M+H+ | XCorr | DeltaCn |
| --- | --- | --- | --- | --- |
| R.ALPLALVLHELGAGR.S | 2 | 1530.84 | 3.656 | 0.532 |

+1 charge

```
		      B   	     Y
  1	 A 	    72.04	        -  	 15
  2	 L 	   185.13	  1458.88	 14
  3	 P 	   282.18	  1345.80	 13
  4	 L 	   395.27	  1248.74	 12
  5	 A 	   466.30	  1135.66	 11
  6	 L 	   579.39	  1064.62	 10
  7	 V 	   678.45	   951.54	  9
  8	 L 	   791.54	   852.47	  8
  9	 H 	   928.60	   739.38	  7
 10	 E 	  1057.64	   602.33	  6
 11	 L 	  1170.72	   473.28	  5
 12	 G 	  1227.75	   360.20	  4
 13	 A 	  1298.78	   303.18	  3
 14	 G 	  1355.80	   232.14	  2
 15	 R 	        -  	   175.12	  1  _
+2 charge
		      B   	     Y
  1	 A 	    36.53	        -  	 15
  2	 L 	    93.07	   729.94	 14
  3	 P 	   141.59	   673.40	 13
  4	 L 	   198.14	   624.87	 12
  5	 A 	   233.65	   568.33	 11
  6	 L 	   290.20	   532.81	 10
  7	 V 	   339.73	   476.27	  9
  8	 L 	   396.27	   426.74	  8
  9	 H 	   464.80	   370.20	  7
 10	 E 	   529.32	   301.67	  6
 11	 L 	   585.87	   237.15	  5
 12	 G 	   614.38	   180.60	  4
 13	 A 	   649.90	   152.09	  3
 14	 G 	   678.41	   116.57	  2
 15	 R 	        -  	    88.06	  1  _
```

**49. IPI00658073 Coiled-coil domain-containing protein 126**

| Sequence | Charge | M+H+ | XCorr | DeltaCn |
| --- | --- | --- | --- | --- |
| R.TIAVLLDDILQR.L | 2 | 1370.62 | 3.07 | 0.293 |

+1 charge

```
		      B   	     Y
  1	 T 	   102.05	        -  	 12
  2	 I 	   215.14	  1268.76	 11
  3	 A 	   286.18	  1155.67	 10
  4	 V 	   385.24	  1084.64	  9
  5	 L 	   498.33	   985.57	  8
  6	 L 	   611.41	   872.48	  7
  7	 D 	   726.44	   759.40	  6
  8	 D 	   841.47	   644.37	  5
  9	 I 	   954.55	   529.35	  4
 10	 L 	  1067.63	   416.26	  3
 11	 Q 	  1195.69	   303.18	  2
 12	 R 	        -  	   175.12	  1  _
+2 charge
		      B   	     Y
  1	 T 	    51.53	        -  	 12
  2	 I 	   108.07	   634.88	 11
  3	 A 	   143.59	   578.34	 10
  4	 V 	   193.13	   542.82	  9
  5	 L 	   249.67	   493.29	  8
  6	 L 	   306.21	   436.75	  7
  7	 D 	   363.72	   380.20	  6
  8	 D 	   421.24	   322.69	  5
  9	 I 	   477.78	   265.18	  4
 10	 L 	   534.32	   208.63	  3
 11	 Q 	   598.35	   152.09	  2
 12	 R 	        -  	    88.06	  1  _
```

**50. IPI00018236 Ganglioside GM2 activator**

| Sequence | Charge | M+H+ | XCorr | DeltaCn |
| --- | --- | --- | --- | --- |
| K.SEFVVPDLELPSWLTTGNYR.I | 2 | 2324.573 | 3.209 | 0.58 |

+1 charge

```
		      B   	     Y
  1	 S 	    88.04	        -  	 20
  2	 E 	   217.08	  2236.13	 19
  3	 F 	   364.15	  2107.09	 18
  4	 V 	   463.22	  1960.02	 17
  5	 V 	   562.29	  1860.95	 16
  6	 P 	   659.34	  1761.88	 15
  7	 D 	   774.37	  1664.83	 14
  8	 L 	   887.45	  1549.80	 13
  9	 E 	  1016.49	  1436.72	 12
 10	 L 	  1129.58	  1307.67	 11
 11	 P 	  1226.63	  1194.59	 10
 12	 S 	  1313.66	  1097.54	  9
 13	 W 	  1499.74	  1010.51	  8
 14	 L 	  1612.83	   824.43	  7
 15	 T 	  1713.87	   711.34	  6
 16	 T 	  1814.92	   610.29	  5
 17	 G 	  1871.94	   509.25	  4
 18	 N 	  1985.99	   452.23	  3
 19	 Y 	  2149.05	   338.18	  2
 20	 R 	        -  	   175.12	  1  _
+2 charge
		      B   	     Y
  1	 S 	    44.52	        -  	 20
  2	 E 	   109.04	  1118.57	 19
  3	 F 	   182.58	  1054.05	 18
  4	 V 	   232.11	   980.51	 17
  5	 V 	   281.65	   930.98	 16
  6	 P 	   330.17	   881.44	 15
  7	 D 	   387.69	   832.92	 14
  8	 L 	   444.23	   775.40	 13
  9	 E 	   508.75	   718.86	 12
 10	 L 	   565.29	   654.34	 11
 11	 P 	   613.82	   597.80	 10
 12	 S 	   657.33	   549.27	  9
 13	 W 	   750.37	   505.76	  8
 14	 L 	   806.92	   412.72	  7
 15	 T 	   857.44	   356.17	  6
 16	 T 	   907.96	   305.65	  5
 17	 G 	   936.47	   255.13	  4
 18	 N 	   993.50	   226.62	  3
 19	 Y 	  1075.03	   169.59	  2
 20	 R 	        -  	    88.06	  1  _
```

**51. IPI00451132 cGMP-dependent 3',5'-cyclic phosphodiesterase**

| Sequence | Charge | M+H+ | XCorr | DeltaCn |
| --- | --- | --- | --- | --- |
| K.EFFSQGDLEKAMGNRPMEMMDR.E | 3 | 2620.991 | 3.518 | 0.3 |

+1 charge

```
		      B   	     Y
  1	 E 	   130.05	        -  	 22
  2	 F 	   277.12	  2490.10	 21
  3	 F 	   424.19	  2343.03	 20
  4	 S 	   511.22	  2195.97	 19
  5	 Q 	   639.28	  2108.93	 18
  6	 G 	   696.30	  1980.88	 17
  7	 D 	   811.33	  1923.85	 16
  8	 L 	   924.41	  1808.83	 15
  9	 E 	  1053.45	  1695.74	 14
 10	 K 	  1181.55	  1566.70	 13
 11	 A 	  1252.58	  1438.61	 12
 12	 M 	  1383.62	  1367.57	 11
 13	 G 	  1440.65	  1236.53	 10
 14	 N 	  1554.69	  1179.51	  9
 15	 R 	  1710.79	  1065.46	  8
 16	 P 	  1807.84	   909.36	  7
 17	 M 	  1938.88	   812.31	  6
 18	 E 	  2067.93	   681.27	  5
 19	 M 	  2198.97	   552.23	  4
 20	 M 	  2330.01	   421.19	  3
 21	 D 	  2445.03	   290.15	  2
 22	 R 	        -  	   175.12	  1  _


+2 charge
		      B   	     Y
  1	 E 	    65.53	        -  	 22
  2	 F 	   139.06	  1245.56	 21
  3	 F 	   212.60	  1172.02	 20
  4	 S 	   256.11	  1098.49	 19
  5	 Q 	   320.14	  1054.97	 18
  6	 G 	   348.65	   990.94	 17
  7	 D 	   406.17	   962.43	 16
  8	 L 	   462.71	   904.92	 15
  9	 E 	   527.23	   848.38	 14
 10	 K 	   591.28	   783.85	 13
 11	 A 	   626.80	   719.81	 12
 12	 M 	   692.32	   684.29	 11
 13	 G 	   720.83	   618.77	 10
 14	 N 	   777.85	   590.26	  9
 15	 R 	   855.90	   533.24	  8
 16	 P 	   904.43	   455.18	  7
 17	 M 	   969.95	   406.66	  6
 18	 E 	  1034.47	   341.14	  5
 19	 M 	  1099.99	   276.62	  4
 20	 M 	  1165.51	   211.10	  3
 21	 D 	  1223.02	   145.58	  2
 22	 R 	        -  	    88.06	  1  _
+3 charge
		      B   	     Y
  1	 E 	    44.02	        -  	 22
  2	 F 	    93.04	   830.71	 21
  3	 F 	   142.07	   781.68	 20
  4	 S 	   171.08	   732.66	 19
  5	 Q 	   213.76	   703.65	 18
  6	 G 	   232.77	   660.96	 17
  7	 D 	   271.11	   641.96	 16
  8	 L 	   308.81	   603.61	 15
  9	 E 	   351.82	   565.92	 14
 10	 K 	   394.52	   522.91	 13
 11	 A 	   418.20	   480.21	 12
 12	 M 	   461.88	   456.53	 11
 13	 G 	   480.89	   412.85	 10
 14	 N 	   518.90	   393.84	  9
 15	 R 	   570.93	   355.83	  8
 16	 P 	   603.29	   303.79	  7
 17	 M 	   646.97	   271.44	  6
 18	 E 	   689.98	   227.76	  5
 19	 M 	   733.66	   184.75	  4
 20	 M 	   777.34	   141.07	  3
 21	 D 	   815.68	    97.39	  2
 22	 R 	        -  	    59.04	  1  _
```

**52. IPI00019580 Plasminogen**

| Sequence | Charge | M+H+ | XCorr | DeltaCn |
| --- | --- | --- | --- | --- |
| R.HSIFTPETNPR.A | 2 | 1299.417 | 2.918 | 0.479 |

+1 charge

```
		      B   	     Y
  1	 H 	   138.07	        -  	 11
  2	 S 	   225.10	  1161.59	 10
  3	 I 	   338.18	  1074.56	  9
  4	 F 	   485.25	   961.47	  8
  5	 T 	   586.30	   814.41	  7
  6	 P 	   683.35	   713.36	  6
  7	 E 	   812.39	   616.30	  5
  8	 T 	   913.44	   487.26	  4
  9	 N 	  1027.48	   386.21	  3
 10	 P 	  1124.54	   272.17	  2
 11	 R 	        -  	   175.12	  1  _
+2 charge
		      B   	     Y
  1	 H 	    69.54	        -  	 11
  2	 S 	   113.05	   581.30	 10
  3	 I 	   169.59	   537.78	  9
  4	 F 	   243.13	   481.24	  8
  5	 T 	   293.65	   407.71	  7
  6	 P 	   342.18	   357.18	  6
  7	 E 	   406.70	   308.66	  5
  8	 T 	   457.22	   244.13	  4
  9	 N 	   514.25	   193.61	  3
 10	 P 	   562.77	   136.59	  2
 11	 R 	        -  	    88.06	  1  _
```

**53. IPI00011140 Protein NOV homolog**

| Sequence | Charge | M+H+ | XCorr | DeltaCn |
| --- | --- | --- | --- | --- |
| R.AVLDGCSCCLVCAR.Q | 2 | 1641.823 | 3.216 | 0.413 |

+1 charge

```
		      B   	     Y
  1	 A 	    72.04	        -  	 14
  2	 V 	   171.11	  1570.20	 13
  3	 L 	   284.20	  1471.13	 12
  4	 D 	   399.22	  1358.05	 11
  5	 G 	   456.25	  1243.02	 10
  6	 C 	   616.41	  1186.00	  9
  7	 S 	   703.44	  1025.84	  8
  8	 C 	   863.61	   938.80	  7
  9	 C 	  1023.77	   778.64	  6
 10	 L 	  1136.86	   618.47	  5
 11	 V 	  1235.93	   505.39	  4
 12	 C 	  1396.09	   406.32	  3
 13	 A 	  1467.13	   246.16	  2
 14	 R 	        -  	   175.12	  1  _
+2 charge
		      B   	     Y
  1	 A 	    36.53	        -  	 14
  2	 V 	    86.06	   785.60	 13
  3	 L 	   142.60	   736.07	 12
  4	 D 	   200.12	   679.53	 11
  5	 G 	   228.63	   622.02	 10
  6	 C 	   308.71	   593.50	  9
  7	 S 	   352.22	   513.42	  8
  8	 C 	   432.31	   469.91	  7
  9	 C 	   512.39	   389.82	  6
 10	 L 	   568.93	   309.74	  5
 11	 V 	   618.47	   253.20	  4
 12	 C 	   698.55	   203.66	  3
 13	 A 	   734.07	   123.58	  2
 14	 R 	        -  	    88.06	  1  _
```

**54. IPI00009027 Lithostathine-1-alpha**

| Sequence | Charge | M+H+ | XCorr | DeltaCn |
| --- | --- | --- | --- | --- |
| K.DVPCEDKFSFVCK.F | 2 | 1631.795 | 2.883 | 0.389 |

+1 charge

```
		      B   	     Y
  1	 D 	   116.03	        -  	 13
  2	 V 	   215.10	  1515.97	 12
  3	 P 	   312.16	  1416.90	 11
  4	 C 	   472.32	  1319.85	 10
  5	 E 	   601.36	  1159.68	  9
  6	 D 	   716.39	  1030.64	  8
  7	 K 	   844.49	   915.61	  7
  8	 F 	   991.55	   787.52	  6
  9	 S 	  1078.59	   640.45	  5
 10	 F 	  1225.65	   553.41	  4
 11	 V 	  1324.72	   406.35	  3
 12	 C 	  1484.89	   307.28	  2
 13	 K 	        -  	   147.11	  1  _
+2 charge
		      B   	     Y
  1	 D 	    58.52	        -  	 13
  2	 V 	   108.05	   758.49	 12
  3	 P 	   156.58	   708.95	 11
  4	 C 	   236.66	   660.43	 10
  5	 E 	   301.19	   580.34	  9
  6	 D 	   358.70	   515.82	  8
  7	 K 	   422.75	   458.31	  7
  8	 F 	   496.28	   394.26	  6
  9	 S 	   539.80	   320.73	  5
 10	 F 	   613.33	   277.21	  4
 11	 V 	   662.86	   203.68	  3
 12	 C 	   742.95	   154.14	  2
 13	 K 	        -  	    74.06	  1  _
```

**55. IPI00419722 cDNA FLJ61340, highly similar to Homo sapiens seizure related 6 homolog-like 2 (SEZ6L2), transcript variant 2, mRNA**

| Sequence | Charge | M+H+ | XCorr | DeltaCn |
| --- | --- | --- | --- | --- |
| R.EGDMLTLFDGDGPSAR.V | 2 | 1681.806 | 2.929 | 0.307 |

+1 charge

```
		      B   	     Y
  1	 E 	   130.05	        -  	 16
  2	 G 	   187.07	  1551.71	 15
  3	 D 	   302.10	  1494.69	 14
  4	 M 	   433.14	  1379.66	 13
  5	 L 	   546.22	  1248.62	 12
  6	 T 	   647.27	  1135.54	 11
  7	 L 	   760.35	  1034.49	 10
  8	 F 	   907.42	   921.41	  9
  9	 D 	  1022.45	   774.34	  8
 10	 G 	  1079.47	   659.31	  7
 11	 D 	  1194.50	   602.29	  6
 12	 G 	  1251.52	   487.26	  5
 13	 P 	  1348.57	   430.24	  4
 14	 S 	  1435.60	   333.19	  3
 15	 A 	  1506.64	   246.16	  2
 16	 R 	        -  	   175.12	  1  _
+2 charge
		      B   	     Y
  1	 E 	    65.53	        -  	 16
  2	 G 	    94.04	   776.36	 15
  3	 D 	   151.55	   747.85	 14
  4	 M 	   217.07	   690.33	 13
  5	 L 	   273.62	   624.81	 12
  6	 T 	   324.14	   568.27	 11
  7	 L 	   380.68	   517.75	 10
  8	 F 	   454.22	   461.21	  9
  9	 D 	   511.73	   387.67	  8
 10	 G 	   540.24	   330.16	  7
 11	 D 	   597.75	   301.65	  6
 12	 G 	   626.26	   244.13	  5
 13	 P 	   674.79	   215.62	  4
 14	 S 	   718.31	   167.10	  3
 15	 A 	   753.82	   123.58	  2
 16	 R 	        -  	    88.06	  1  _
```

**56. IPI00000828 Proenkephalin-A**

| Sequence | Charge | M+H+ | XCorr | DeltaCn |
| --- | --- | --- | --- | --- |
| K.DAEEDDSLANSSDLLKELLETGDNRER.S | 3 | 3036.123 | 5.452 | 0.544 |

+1 charge

```
		      B   	     Y
  1	 D 	   116.03	        -  	 27
  2	 A 	   187.07	  2919.38	 26
  3	 E 	   316.11	  2848.34	 25
  4	 E 	   445.16	  2719.30	 24
  5	 D 	   560.18	  2590.26	 23
  6	 D 	   675.21	  2475.23	 22
  7	 S 	   762.24	  2360.21	 21
  8	 L 	   875.33	  2273.17	 20
  9	 A 	   946.36	  2160.09	 19
 10	 N 	  1060.41	  2089.05	 18
 11	 S 	  1147.44	  1975.01	 17
 12	 S 	  1234.47	  1887.98	 16
 13	 D 	  1349.50	  1800.95	 15
 14	 L 	  1462.58	  1685.92	 14
 15	 L 	  1575.67	  1572.83	 13
 16	 K 	  1703.76	  1459.75	 12
 17	 E 	  1832.80	  1331.65	 11
 18	 L 	  1945.89	  1202.61	 10
 19	 L 	  2058.97	  1089.53	  9
 20	 E 	  2188.01	   976.44	  8
 21	 T 	  2289.06	   847.40	  7
 22	 G 	  2346.08	   746.35	  6
 23	 D 	  2461.11	   689.33	  5
 24	 N 	  2575.15	   574.31	  4
 25	 R 	  2731.25	   460.26	  3
 26	 E 	  2860.30	   304.16	  2
 27	 R 	        -  	   175.12	  1  _


+2 charge
		      B   	     Y
  1	 D 	    58.52	        -  	 27
  2	 A 	    94.04	  1460.19	 26
  3	 E 	   158.56	  1424.68	 25
  4	 E 	   223.08	  1360.15	 24
  5	 D 	   280.60	  1295.63	 23
  6	 D 	   338.11	  1238.12	 22
  7	 S 	   381.62	  1180.61	 21
  8	 L 	   438.17	  1137.09	 20
  9	 A 	   473.69	  1080.55	 19
 10	 N 	   530.71	  1045.03	 18
 11	 S 	   574.22	   988.01	 17
 12	 S 	   617.74	   944.49	 16
 13	 D 	   675.25	   900.98	 15
 14	 L 	   731.79	   843.46	 14
 15	 L 	   788.34	   786.92	 13
 16	 K 	   852.38	   730.38	 12
 17	 E 	   916.91	   666.33	 11
 18	 L 	   973.45	   601.81	 10
 19	 L 	  1029.99	   545.27	  9
 20	 E 	  1094.51	   488.73	  8
 21	 T 	  1145.03	   424.20	  7
 22	 G 	  1173.55	   373.68	  6
 23	 D 	  1231.06	   345.17	  5
 24	 N 	  1288.08	   287.66	  4
 25	 R 	  1366.13	   230.63	  3
 26	 E 	  1430.65	   152.58	  2
 27	 R 	        -  	    88.06	  1  _


+3 charge
		      B   	     Y
  1	 D 	    39.35	        -  	 27
  2	 A 	    63.03	   973.80	 26
  3	 E 	   106.04	   950.12	 25
  4	 E 	   149.06	   907.11	 24
  5	 D 	   187.40	   864.09	 23
  6	 D 	   225.74	   825.75	 22
  7	 S 	   254.75	   787.41	 21
  8	 L 	   292.45	   758.40	 20
  9	 A 	   316.13	   720.70	 19
 10	 N 	   354.14	   697.02	 18
 11	 S 	   383.15	   659.01	 17
 12	 S 	   412.16	   630.00	 16
 13	 D 	   450.50	   600.99	 15
 14	 L 	   488.20	   562.64	 14
 15	 L 	   525.89	   524.95	 13
 16	 K 	   568.59	   487.25	 12
 17	 E 	   611.61	   444.56	 11
 18	 L 	   649.30	   401.54	 10
 19	 L 	   687.00	   363.85	  9
 20	 E 	   730.01	   326.15	  8
 21	 T 	   763.69	   283.14	  7
 22	 G 	   782.70	   249.46	  6
 23	 D 	   821.04	   230.45	  5
 24	 N 	   859.06	   192.11	  4
 25	 R 	   911.09	   154.09	  3
 26	 E 	   954.10	   102.06	  2
 27	 R 	        -  	    59.04	  1  _
```

**57. IPI00009866 Isoform 1 of Keratin, type I cytoskeletal 13**

| Sequence | Charge | M+H+ | XCorr | DeltaCn |
| --- | --- | --- | --- | --- |
| R.VLDELTLSKTDLEMQIESLNEELAYMKK.N | 3 | 3285.772 | 4.231 | 0.447 |

+1 charge

```
		      B   	     Y
  1	 V 	   100.08	        -  	 28
  2	 L 	   213.16	  3184.60	 27
  3	 D 	   328.19	  3071.52	 26
  4	 E 	   457.23	  2956.49	 25
  5	 L 	   570.31	  2827.45	 24
  6	 T 	   671.36	  2714.36	 23
  7	 L 	   784.45	  2613.32	 22
  8	 S 	   871.48	  2500.23	 21
  9	 K 	   999.57	  2413.20	 20
 10	 T 	  1100.62	  2285.10	 19
 11	 D 	  1215.65	  2184.06	 18
 12	 L 	  1328.73	  2069.03	 17
 13	 E 	  1457.77	  1955.95	 16
 14	 M 	  1588.81	  1826.90	 15
 15	 Q 	  1716.87	  1695.86	 14
 16	 I 	  1829.96	  1567.80	 13
 17	 E 	  1959.00	  1454.72	 12
 18	 S 	  2046.03	  1325.68	 11
 19	 L 	  2159.12	  1238.64	 10
 20	 N 	  2273.16	  1125.56	  9
 21	 E 	  2402.20	  1011.52	  8
 22	 E 	  2531.24	   882.48	  7
 23	 L 	  2644.33	   753.43	  6
 24	 A 	  2715.36	   640.35	  5
 25	 Y 	  2878.43	   569.31	  4
 26	 M 	  3009.47	   406.25	  3
 27	 K 	  3137.56	   275.21	  2
 28	 K 	        -  	   147.11	  1  _


+2 charge
		      B   	     Y
  1	 V 	    50.54	        -  	 28
  2	 L 	   107.08	  1592.80	 27
  3	 D 	   164.60	  1536.26	 26
  4	 E 	   229.12	  1478.75	 25
  5	 L 	   285.66	  1414.23	 24
  6	 T 	   336.18	  1357.68	 23
  7	 L 	   392.73	  1307.16	 22
  8	 S 	   436.24	  1250.62	 21
  9	 K 	   500.29	  1207.10	 20
 10	 T 	   550.81	  1143.06	 19
 11	 D 	   608.33	  1092.53	 18
 12	 L 	   664.87	  1035.02	 17
 13	 E 	   729.39	   978.48	 16
 14	 M 	   794.91	   913.95	 15
 15	 Q 	   858.94	   848.43	 14
 16	 I 	   915.48	   784.41	 13
 17	 E 	   980.00	   727.86	 12
 18	 S 	  1023.52	   663.34	 11
 19	 L 	  1080.06	   619.83	 10
 20	 N 	  1137.08	   563.28	  9
 21	 E 	  1201.60	   506.26	  8
 22	 E 	  1266.13	   441.74	  7
 23	 L 	  1322.67	   377.22	  6
 24	 A 	  1358.19	   320.68	  5
 25	 Y 	  1439.72	   285.16	  4
 26	 M 	  1505.24	   203.63	  3
 27	 K 	  1569.29	   138.11	  2
 28	 K 	        -  	    74.06	  1  _


+3 charge
		      B   	     Y
  1	 V 	    34.03	        -  	 28
  2	 L 	    71.72	  1062.20	 27
  3	 D 	   110.07	  1024.51	 26
  4	 E 	   153.08	   986.17	 25
  5	 L 	   190.78	   943.15	 24
  6	 T 	   224.46	   905.46	 23
  7	 L 	   262.15	   871.78	 22
  8	 S 	   291.16	   834.08	 21
  9	 K 	   333.86	   805.07	 20
 10	 T 	   367.54	   762.37	 19
 11	 D 	   405.89	   728.69	 18
 12	 L 	   443.58	   690.35	 17
 13	 E 	   486.60	   652.65	 16
 14	 M 	   530.28	   609.64	 15
 15	 Q 	   572.96	   565.96	 14
 16	 I 	   610.66	   523.27	 13
 17	 E 	   653.67	   485.58	 12
 18	 S 	   682.68	   442.56	 11
 19	 L 	   720.38	   413.55	 10
 20	 N 	   758.39	   375.86	  9
 21	 E 	   801.41	   337.84	  8
 22	 E 	   844.42	   294.83	  7
 23	 L 	   882.11	   251.82	  6
 24	 A 	   905.79	   214.12	  5
 25	 Y 	   960.15	   190.44	  4
 26	 M 	  1003.83	   136.09	  3
 27	 K 	  1046.53	    92.41	  2
 28	 K 	        -  	    49.71	  1  _
```

**58. IPI00004310 Ly6/PLAUR domain-containing protein 3**

| Sequence | Charge | M+H+ | XCorr | DeltaCn |
| --- | --- | --- | --- | --- |
| R.GLDLHGLLAFIQLQQCAQDR.C | 3 | 2297.593 | 6.105 | 0.361 |

+1 charge

```
		      B   	     Y
  1	 G 	    58.03	        -  	 20
  2	 L 	   171.11	  2239.30	 19
  3	 D 	   286.14	  2126.22	 18
  4	 L 	   399.22	  2011.19	 17
  5	 H 	   536.28	  1898.10	 16
  6	 G 	   593.30	  1761.05	 15
  7	 L 	   706.39	  1704.02	 14
  8	 L 	   819.47	  1590.94	 13
  9	 A 	   890.51	  1477.86	 12
 10	 F 	  1037.58	  1406.82	 11
 11	 I 	  1150.66	  1259.75	 10
 12	 Q 	  1278.72	  1146.67	  9
 13	 L 	  1391.80	  1018.61	  8
 14	 Q 	  1519.86	   905.52	  7
 15	 Q 	  1647.92	   777.47	  6
 16	 C 	  1808.09	   649.41	  5
 17	 A 	  1879.12	   489.24	  4
 18	 Q 	  2007.18	   418.20	  3
 19	 D 	  2122.21	   290.15	  2
 20	 R 	        -  	   175.12	  1  _
+2 charge
		      B   	     Y
  1	 G 	    29.52	        -  	 20
  2	 L 	    86.06	  1120.15	 19
  3	 D 	   143.57	  1063.61	 18
  4	 L 	   200.12	  1006.10	 17
  5	 H 	   268.64	   949.56	 16
  6	 G 	   297.16	   881.03	 15
  7	 L 	   353.70	   852.52	 14
  8	 L 	   410.24	   795.97	 13
  9	 A 	   445.76	   739.43	 12
 10	 F 	   519.29	   703.91	 11
 11	 I 	   575.83	   630.38	 10
 12	 Q 	   639.86	   573.84	  9
 13	 L 	   696.41	   509.81	  8
 14	 Q 	   760.44	   453.27	  7
 15	 Q 	   824.46	   389.24	  6
 16	 C 	   904.55	   325.21	  5
 17	 A 	   940.07	   245.12	  4
 18	 Q 	  1004.09	   209.61	  3
 19	 D 	  1061.61	   145.58	  2
 20	 R 	        -  	    88.06	  1  _

+3 charge
		      B   	     Y
  1	 G 	    20.01	        -  	 20
  2	 L 	    57.71	   747.10	 19
  3	 D 	    96.05	   709.41	 18
  4	 L 	   133.75	   671.07	 17
  5	 H 	   179.43	   633.37	 16
  6	 G 	   198.44	   587.69	 15
  7	 L 	   236.13	   568.68	 14
  8	 L 	   273.83	   530.98	 13
  9	 A 	   297.51	   493.29	 12
 10	 F 	   346.53	   469.61	 11
 11	 I 	   384.23	   420.59	 10
 12	 Q 	   426.91	   382.89	  9
 13	 L 	   464.61	   340.21	  8
 14	 Q 	   507.29	   302.51	  7
 15	 Q 	   549.98	   259.83	  6
 16	 C 	   603.37	   217.14	  5
 17	 A 	   627.05	   163.75	  4
 18	 Q 	   669.73	   140.07	  3
 19	 D 	   708.07	    97.39	  2
 20	 R 	        -  	    59.04	  1  _
```

**59. IPI00010949 Isoform 1 of Sialate O-acetylesterase**

| Sequence | Charge | M+H+ | XCorr | DeltaCn |
| --- | --- | --- | --- | --- |
| R.FFPFGLVQLSSDLSK.K | 2 | 1685.944 | 3.078 | 0.434 |

+1 charge

```
		      B   	     Y
  1	 F 	   148.08	        -  	 15
  2	 F 	   295.14	  1537.83	 14
  3	 P 	   392.20	  1390.76	 13
  4	 F 	   539.27	  1293.70	 12
  5	 G 	   596.29	  1146.64	 11
  6	 L 	   709.37	  1089.62	 10
  7	 V 	   808.44	   976.53	  9
  8	 Q 	   936.50	   877.46	  8
  9	 L 	  1049.58	   749.40	  7
 10	 S 	  1136.61	   636.32	  6
 11	 S 	  1223.65	   549.29	  5
 12	 D 	  1338.67	   462.26	  4
 13	 L 	  1451.76	   347.23	  3
 14	 S 	  1538.79	   234.14	  2
 15	 K 	        -  	   147.11	  1  _
+2 charge
		      B   	     Y
  1	 F 	    74.54	        -  	 15
  2	 F 	   148.08	   769.42	 14
  3	 P 	   196.60	   695.88	 13
  4	 F 	   270.14	   647.36	 12
  5	 G 	   298.65	   573.82	 11
  6	 L 	   355.19	   545.31	 10
  7	 V 	   404.72	   488.77	  9
  8	 Q 	   468.75	   439.23	  8
  9	 L 	   525.29	   375.21	  7
 10	 S 	   568.81	   318.66	  6
 11	 S 	   612.33	   275.15	  5
 12	 D 	   669.84	   231.63	  4
 13	 L 	   726.38	   174.12	  3
 14	 S 	   769.90	   117.58	  2
 15	 K 	        -  	    74.06	  1  _
```

**60. IPI00855790 zinc finger protein 251**

| Sequence | Charge | M+H+ | XCorr | DeltaCn |
| --- | --- | --- | --- | --- |
| K.ELWVLNLLGAEEPDILK.S | 2 | 1953.268 | 2.981 | 0.313 |

+1 charge

```
		      B   	     Y
  1	 E 	   130.05	        -  	 17
  2	 L 	   243.13	  1823.03	 16
  3	 W 	   429.21	  1709.95	 15
  4	 V 	   528.28	  1523.87	 14
  5	 L 	   641.37	  1424.80	 13
  6	 N 	   755.41	  1311.72	 12
  7	 L 	   868.49	  1197.67	 11
  8	 L 	   981.58	  1084.59	 10
  9	 G 	  1038.60	   971.50	  9
 10	 A 	  1109.64	   914.48	  8
 11	 E 	  1238.68	   843.45	  7
 12	 E 	  1367.72	   714.40	  6
 13	 P 	  1464.77	   585.36	  5
 14	 D 	  1579.80	   488.31	  4
 15	 I 	  1692.88	   373.28	  3
 16	 L 	  1805.97	   260.20	  2
 17	 K 	        -  	   147.11	  1  _
+2 charge
		      B   	     Y
  1	 E 	    65.53	        -  	 17
  2	 L 	   122.07	   912.02	 16
  3	 W 	   215.11	   855.48	 15
  4	 V 	   264.64	   762.44	 14
  5	 L 	   321.19	   712.90	 13
  6	 N 	   378.21	   656.36	 12
  7	 L 	   434.75	   599.34	 11
  8	 L 	   491.29	   542.80	 10
  9	 G 	   519.80	   486.26	  9
 10	 A 	   555.32	   457.75	  8
 11	 E 	   619.84	   422.23	  7
 12	 E 	   684.36	   357.71	  6
 13	 P 	   732.89	   293.18	  5
 14	 D 	   790.40	   244.66	  4
 15	 I 	   846.95	   187.14	  3
 16	 L 	   903.49	   130.60	  2
 17	 K 	        -  	    74.06	  1  _
```

**61. IPI00027436 Tumor necrosis factor receptor superfamily member 16**

| Sequence | Charge | M+H+ | XCorr | DeltaCn |
| --- | --- | --- | --- | --- |
| R.WADAECEEIPGR.W | 2 | 1433.499 | 4.437 | 0.475 |

+1 charge

```
		      B   	     Y
  1	 W 	   187.09	        -  	 12
  2	 A 	   258.12	  1246.67	 11
  3	 D 	   373.15	  1175.63	 10
  4	 A 	   444.19	  1060.61	  9
  5	 E 	   573.23	   989.57	  8
  6	 C 	   733.40	   860.53	  7
  7	 E 	   862.44	   700.36	  6
  8	 E 	   991.48	   571.32	  5
  9	 I 	  1104.56	   442.28	  4
 10	 P 	  1201.62	   329.19	  3
 11	 G 	  1258.64	   232.14	  2
 12	 R 	        -  	   175.12	  1  _
+2 charge
		      B   	     Y
  1	 W 	    94.05	        -  	 12
  2	 A 	   129.57	   623.84	 11
  3	 D 	   187.08	   588.32	 10
  4	 A 	   222.60	   530.81	  9
  5	 E 	   287.12	   495.29	  8
  6	 C 	   367.20	   430.77	  7
  7	 E 	   431.72	   350.68	  6
  8	 E 	   496.24	   286.16	  5
  9	 I 	   552.79	   221.64	  4
 10	 P 	   601.31	   165.10	  3
 11	 G 	   629.82	   116.57	  2
 12	 R 	        -  	    88.06	  1  _
```

**62. IPI00953695 Ig kappa chain V-II region GM607 (Fragment)**

| Sequence | Charge | M+H+ | XCorr | DeltaCn |
| --- | --- | --- | --- | --- |
| R.ASGVPDRFSGSGSGTDFTLK.I | 3 | 1987.116 | 4.42 | 0.573 |

+1 charge

```
		      B   	     Y
  1	 A 	    72.04	        -  	 20
  2	 S 	   159.08	  1914.92	 19
  3	 G 	   216.10	  1827.89	 18
  4	 V 	   315.17	  1770.87	 17
  5	 P 	   412.22	  1671.80	 16
  6	 D 	   527.25	  1574.74	 15
  7	 R 	   683.35	  1459.72	 14
  8	 F 	   830.42	  1303.62	 13
  9	 S 	   917.45	  1156.55	 12
 10	 G 	   974.47	  1069.52	 11
 11	 S 	  1061.50	  1012.49	 10
 12	 G 	  1118.52	   925.46	  9
 13	 S 	  1205.55	   868.44	  8
 14	 G 	  1262.58	   781.41	  7
 15	 T 	  1363.62	   724.39	  6
 16	 D 	  1478.65	   623.34	  5
 17	 F 	  1625.72	   508.31	  4
 18	 T 	  1726.77	   361.24	  3
 19	 L 	  1839.85	   260.20	  2
 20	 K 	        -  	   147.11	  1  _
+2 charge
		      B   	     Y
  1	 A 	    36.53	        -  	 20
  2	 S 	    80.04	   957.96	 19
  3	 G 	   108.55	   914.45	 18
  4	 V 	   158.09	   885.94	 17
  5	 P 	   206.61	   836.40	 16
  6	 D 	   264.13	   787.88	 15
  7	 R 	   342.18	   730.36	 14
  8	 F 	   415.71	   652.31	 13
  9	 S 	   459.23	   578.78	 12
 10	 G 	   487.74	   535.26	 11
 11	 S 	   531.25	   506.75	 10
 12	 G 	   559.76	   463.23	  9
 13	 S 	   603.28	   434.72	  8
 14	 G 	   631.79	   391.21	  7
 15	 T 	   682.32	   362.70	  6
 16	 D 	   739.83	   312.17	  5
 17	 F 	   813.36	   254.66	  4
 18	 T 	   863.89	   181.13	  3
 19	 L 	   920.43	   130.60	  2
 20	 K 	        -  	    74.06	  1  _

+3 charge
		      B   	     Y
  1	 A 	    24.69	        -  	 20
  2	 S 	    53.70	   638.98	 19
  3	 G 	    72.70	   609.97	 18
  4	 V 	   105.73	   590.96	 17
  5	 P 	   138.08	   557.94	 16
  6	 D 	   176.42	   525.59	 15
  7	 R 	   228.45	   487.24	 14
  8	 F 	   277.48	   435.21	 13
  9	 S 	   306.49	   386.19	 12
 10	 G 	   325.49	   357.18	 11
 11	 S 	   354.51	   338.17	 10
 12	 G 	   373.51	   309.16	  9
 13	 S 	   402.52	   290.15	  8
 14	 G 	   421.53	   261.14	  7
 15	 T 	   455.21	   242.13	  6
 16	 D 	   493.56	   208.45	  5
 17	 F 	   542.58	   170.11	  4
 18	 T 	   576.26	   121.09	  3
 19	 L 	   613.96	    87.40	  2
 20	 K 	        -  	    49.71	  1  _
```

**63. IPI00304273 Apolipoprotein A-IV**

| Sequence | Charge | M+H+ | XCorr | DeltaCn |
| --- | --- | --- | --- | --- |
| K.ALVQQMEQLR.Q | 2 | 1216.436 | 3.188 | 0.374 |

+1 charge

```
		      B   	     Y
  1	 A 	    72.04	        -  	 10
  2	 L 	   185.13	  1144.61	  9
  3	 V 	   284.20	  1031.53	  8
  4	 Q 	   412.26	   932.46	  7
  5	 Q 	   540.31	   804.40	  6
  6	 M 	   671.35	   676.34	  5
  7	 E 	   800.40	   545.30	  4
  8	 Q 	   928.46	   416.26	  3
  9	 L 	  1041.54	   288.20	  2
 10	 R 	        -  	   175.12	  1  _
+2 charge
		      B   	     Y
  1	 A 	    36.53	        -  	 10
  2	 L 	    93.07	   572.81	  9
  3	 V 	   142.60	   516.27	  8
  4	 Q 	   206.63	   466.73	  7
  5	 Q 	   270.66	   402.71	  6
  6	 M 	   336.18	   338.68	  5
  7	 E 	   400.70	   273.16	  4
  8	 Q 	   464.73	   208.63	  3
  9	 L 	   521.27	   144.61	  2
 10	 R 	        -  	    88.06	  1  _
```

**64. IPI00003807 Lysosomal acid phosphatase**

| Sequence | Charge | M+H+ | XCorr | DeltaCn |
| --- | --- | --- | --- | --- |
| R.LLKFPLGPCPR.Y | 2 | 1298.595 | 2.978 | 0.475 |

```
+1 charge
		      B   	     Y
  1	 L 	   114.09	        -  	 11
  2	 L 	   227.18	  1184.80	 10
  3	 K 	   355.27	  1071.71	  9
  4	 F 	   502.34	   943.62	  8
  5	 P 	   599.39	   796.55	  7
  6	 L 	   712.48	   699.50	  6
  7	 G 	   769.50	   586.41	  5
  8	 P 	   866.55	   529.39	  4
  9	 C 	  1026.72	   432.34	  3
 10	 P 	  1123.77	   272.17	  2
 11	 R 	        -  	   175.12	  1  _
+2 charge
		      B   	     Y
  1	 L 	    57.55	        -  	 11
  2	 L 	   114.09	   592.90	 10
  3	 K 	   178.14	   536.36	  9
  4	 F 	   251.67	   472.31	  8
  5	 P 	   300.20	   398.78	  7
  6	 L 	   356.74	   350.25	  6
  7	 G 	   385.25	   293.71	  5
  8	 P 	   433.78	   265.20	  4
  9	 C 	   513.86	   216.67	  3
 10	 P 	   562.39	   136.59	  2
 11	 R 	        -  	    88.06	  1  _
```

**65. IPI00843975 Ezrin**

| Sequence | Charge | M+H+ | XCorr | DeltaCn |
| --- | --- | --- | --- | --- |
| K.SQEQLAAELAEYTAK.I | 2 | 1652.784 | 3.208 | 0.489 |

+1 charge

```
		      B   	     Y
  1	 S 	    88.04	        -  	 15
  2	 Q 	   216.10	  1564.79	 14
  3	 E 	   345.14	  1436.73	 13
  4	 Q 	   473.20	  1307.68	 12
  5	 L 	   586.28	  1179.63	 11
  6	 A 	   657.32	  1066.54	 10
  7	 A 	   728.36	   995.50	  9
  8	 E 	   857.40	   924.47	  8
  9	 L 	   970.48	   795.42	  7
 10	 A 	  1041.52	   682.34	  6
 11	 E 	  1170.56	   611.30	  5
 12	 Y 	  1333.63	   482.26	  4
 13	 T 	  1434.67	   319.20	  3
 14	 A 	  1505.71	   218.15	  2
 15	 K 	        -  	   147.11	  1  _
+2 charge
		      B   	     Y
  1	 S 	    44.52	        -  	 15
  2	 Q 	   108.55	   782.90	 14
  3	 E 	   173.07	   718.87	 13
  4	 Q 	   237.10	   654.35	 12
  5	 L 	   293.65	   590.32	 11
  6	 A 	   329.16	   533.77	 10
  7	 A 	   364.68	   498.26	  9
  8	 E 	   429.20	   462.74	  8
  9	 L 	   485.75	   398.22	  7
 10	 A 	   521.26	   341.67	  6
 11	 E 	   585.79	   306.16	  5
 12	 Y 	   667.32	   241.63	  4
 13	 T 	   717.84	   160.10	  3
 14	 A 	   753.36	   109.58	  2
 15	 K 	        -  	    74.06	  1  _
```

**66. IPI00940464 Aminoacylase-1**

| Sequence | Charge | M+H+ | XCorr | DeltaCn |
| --- | --- | --- | --- | --- |
| R.AVGVPALGFSPMNR.T | 2 | 1416.674 | 3.414 | 0.626 |

+1 charge

```
		      B   	     Y
  1	 A 	    72.04	        -  	 14
  2	 V 	   171.11	  1344.71	 13
  3	 G 	   228.13	  1245.64	 12
  4	 V 	   327.20	  1188.62	 11
  5	 P 	   424.26	  1089.55	 10
  6	 A 	   495.29	   992.50	  9
  7	 L 	   608.38	   921.46	  8
  8	 G 	   665.40	   808.38	  7
  9	 F 	   812.47	   751.36	  6
 10	 S 	   899.50	   604.29	  5
 11	 P 	   996.55	   517.26	  4
 12	 M 	  1127.59	   420.20	  3
 13	 N 	  1241.63	   289.16	  2
 14	 R 	        -  	   175.12	  1  _
+2 charge
		      B   	     Y
  1	 A 	    36.53	        -  	 14
  2	 V 	    86.06	   672.86	 13
  3	 G 	   114.57	   623.32	 12
  4	 V 	   164.10	   594.81	 11
  5	 P 	   212.63	   545.28	 10
  6	 A 	   248.15	   496.75	  9
  7	 L 	   304.69	   461.23	  8
  8	 G 	   333.20	   404.69	  7
  9	 F 	   406.74	   376.18	  6
 10	 S 	   450.25	   302.65	  5
 11	 P 	   498.78	   259.13	  4
 12	 M 	   564.30	   210.60	  3
 13	 N 	   621.32	   145.08	  2
 14	 R 	        -  	    88.06	  1  _
```

**67. IPI00298057 Periplakin**

| Sequence | Charge | M+H+ | XCorr | DeltaCn |
| --- | --- | --- | --- | --- |
| K.SLLGEVEQNLQAAK.Q | 2 | 1500.679 | 3.426 | 0.529 |

+1 charge

```
		      B   	     Y
  1	 S 	    88.04	        -  	 14
  2	 L 	   201.12	  1412.77	 13
  3	 L 	   314.21	  1299.69	 12
  4	 G 	   371.23	  1186.61	 11
  5	 E 	   500.27	  1129.58	 10
  6	 V 	   599.34	  1000.54	  9
  7	 E 	   728.38	   901.47	  8
  8	 Q 	   856.44	   772.43	  7
  9	 N 	   970.48	   644.37	  6
 10	 L 	  1083.57	   530.33	  5
 11	 Q 	  1211.63	   417.25	  4
 12	 A 	  1282.66	   289.19	  3
 13	 A 	  1353.70	   218.15	  2
 14	 K 	        -  	   147.11	  1  _
+2 charge
		      B   	     Y
  1	 S 	    44.52	        -  	 14
  2	 L 	   101.07	   706.89	 13
  3	 L 	   157.61	   650.35	 12
  4	 G 	   186.12	   593.81	 11
  5	 E 	   250.64	   565.30	 10
  6	 V 	   300.17	   500.77	  9
  7	 E 	   364.69	   451.24	  8
  8	 Q 	   428.72	   386.72	  7
  9	 N 	   485.75	   322.69	  6
 10	 L 	   542.29	   265.67	  5
 11	 Q 	   606.32	   209.13	  4
 12	 A 	   641.84	   145.10	  3
 13	 A 	   677.35	   109.58	  2
 14	 K 	        -  	    74.06	  1  _
```

**68. IPI00410259 Isoform 1 of Pleckstrin homology-like domain family B member 2**

| Sequence | Charge | M+H+ | XCorr | DeltaCn |
| --- | --- | --- | --- | --- |
| R.LDEEKENLTQQLLR.E | 2 | 1729.913 | 2.866 | 0.223 |

+1 charge

```
		      B   	     Y
  1	 L 	   114.09	        -  	 14
  2	 D 	   229.12	  1615.83	 13
  3	 E 	   358.16	  1500.80	 12
  4	 E 	   487.20	  1371.76	 11
  5	 K 	   615.30	  1242.72	 10
  6	 E 	   744.34	  1114.62	  9
  7	 N 	   858.38	   985.58	  8
  8	 L 	   971.47	   871.54	  7
  9	 T 	  1072.52	   758.45	  6
 10	 Q 	  1200.57	   657.40	  5
 11	 Q 	  1328.63	   529.35	  4
 12	 L 	  1441.72	   401.29	  3
 13	 L 	  1554.80	   288.20	  2
 14	 R 	        -  	   175.12	  1  _
+2 charge
		      B   	     Y
  1	 L 	    57.55	        -  	 14
  2	 D 	   115.06	   808.42	 13
  3	 E 	   179.58	   750.90	 12
  4	 E 	   244.11	   686.38	 11
  5	 K 	   308.15	   621.86	 10
  6	 E 	   372.67	   557.81	  9
  7	 N 	   429.70	   493.29	  8
  8	 L 	   486.24	   436.27	  7
  9	 T 	   536.76	   379.73	  6
 10	 Q 	   600.79	   329.21	  5
 11	 Q 	   664.82	   265.18	  4
 12	 L 	   721.36	   201.15	  3
 13	 L 	   777.90	   144.61	  2
 14	 R 	        -  	    88.06	  1  _
```

**69. IPI00872780 Annexin A4**

| Sequence | Charge | M+H+ | XCorr | DeltaCn |
| --- | --- | --- | --- | --- |
| K.GLGTDEDAIISVLAYR.N | 2 | 1693.879 | 3.242 | 0.428 |

+1 charge

```
		      B   	     Y
  1	 G 	    58.03	        -  	 16
  2	 L 	   171.11	  1635.86	 15
  3	 G 	   228.13	  1522.77	 14
  4	 T 	   329.18	  1465.75	 13
  5	 D 	   444.21	  1364.71	 12
  6	 E 	   573.25	  1249.68	 11
  7	 D 	   688.28	  1120.64	 10
  8	 A 	   759.32	  1005.61	  9
  9	 I 	   872.40	   934.57	  8
 10	 I 	   985.48	   821.49	  7
 11	 S 	  1072.52	   708.40	  6
 12	 V 	  1171.58	   621.37	  5
 13	 L 	  1284.67	   522.30	  4
 14	 A 	  1355.71	   409.22	  3
 15	 Y 	  1518.77	   338.18	  2
 16	 R 	        -  	   175.12	  1  _
+2 charge
		      B   	     Y
  1	 G 	    29.52	        -  	 16
  2	 L 	    86.06	   818.43	 15
  3	 G 	   114.57	   761.89	 14
  4	 T 	   165.09	   733.38	 13
  5	 D 	   222.61	   682.86	 12
  6	 E 	   287.13	   625.34	 11
  7	 D 	   344.64	   560.82	 10
  8	 A 	   380.16	   503.31	  9
  9	 I 	   436.70	   467.79	  8
 10	 I 	   493.25	   411.25	  7
 11	 S 	   536.76	   354.71	  6
 12	 V 	   586.30	   311.19	  5
 13	 L 	   642.84	   261.66	  4
 14	 A 	   678.36	   205.11	  3
 15	 Y 	   759.89	   169.59	  2
 16	 R 	        -  	    88.06	  1  _
```

**70. IPI00013808 Alpha-actinin-4**

| Sequence | Charge | M+H+ | XCorr | DeltaCn |
| --- | --- | --- | --- | --- |
| R.VGWEQLLTTIAR.T | 2 | 1387.609 | 3.127 | 0.466 |

+1 charge

```
		      B   	     Y
  1	 V 	   100.08	        -  	 12
  2	 G 	   157.10	  1287.71	 11
  3	 W 	   343.18	  1230.68	 10
  4	 E 	   472.22	  1044.60	  9
  5	 Q 	   600.28	   915.56	  8
  6	 L 	   713.36	   787.50	  7
  7	 L 	   826.45	   674.42	  6
  8	 T 	   927.49	   561.34	  5
  9	 T 	  1028.54	   460.29	  4
 10	 I 	  1141.63	   359.24	  3
 11	 A 	  1212.66	   246.16	  2
 12	 R 	        -  	   175.12	  1  _
+2 charge
		      B   	     Y
  1	 V 	    50.54	        -  	 12
  2	 G 	    79.05	   644.36	 11
  3	 W 	   172.09	   615.85	 10
  4	 E 	   236.61	   522.81	  9
  5	 Q 	   300.64	   458.28	  8
  6	 L 	   357.18	   394.26	  7
  7	 L 	   413.73	   337.71	  6
  8	 T 	   464.25	   281.17	  5
  9	 T 	   514.77	   230.65	  4
 10	 I 	   571.32	   180.12	  3
 11	 A 	   606.83	   123.58	  2
 12	 R 	        -  	    88.06	  1  _
```

**71. IPI00855785 Isoform 15 of Fibronectin**

| Sequence | Charge | M+H+ | XCorr | DeltaCn |
| --- | --- | --- | --- | --- |
| R.VTWAPPPSIDLTNFLVR.Y | 2 | 1927.235 | 2.815 | 0.471 |

+1 charge

```
		      B   	     Y
  1	 V 	   100.08	        -  	 17
  2	 T 	   201.12	  1826.98	 16
  3	 W 	   387.20	  1725.93	 15
  4	 A 	   458.24	  1539.85	 14
  5	 P 	   555.29	  1468.82	 13
  6	 P 	   652.35	  1371.76	 12
  7	 P 	   749.40	  1274.71	 11
  8	 S 	   836.43	  1177.66	 10
  9	 I 	   949.51	  1090.63	  9
 10	 D 	  1064.54	   977.54	  8
 11	 L 	  1177.63	   862.51	  7
 12	 T 	  1278.67	   749.43	  6
 13	 N 	  1392.72	   648.38	  5
 14	 F 	  1539.78	   534.34	  4
 15	 L 	  1652.87	   387.27	  3
 16	 V 	  1751.94	   274.19	  2
 17	 R 	        -  	   175.12	  1  _
+2 charge
		      B   	     Y
  1	 V 	    50.54	        -  	 17
  2	 T 	   101.07	   913.99	 16
  3	 W 	   194.10	   863.47	 15
  4	 A 	   229.62	   770.43	 14
  5	 P 	   278.15	   734.91	 13
  6	 P 	   326.68	   686.39	 12
  7	 P 	   375.20	   637.86	 11
  8	 S 	   418.72	   589.33	 10
  9	 I 	   475.26	   545.82	  9
 10	 D 	   532.77	   489.27	  8
 11	 L 	   589.32	   431.76	  7
 12	 T 	   639.84	   375.22	  6
 13	 N 	   696.86	   324.70	  5
 14	 F 	   770.40	   267.67	  4
 15	 L 	   826.94	   194.14	  3
 16	 V 	   876.47	   137.60	  2
 17	 R 	        -  	    88.06	  1  _
```

**72. IPI00026270 Carboxypeptidase M**

| Sequence | Charge | M+H+ | XCorr | DeltaCn |
| --- | --- | --- | --- | --- |
| K.DILLPFQGQLDSIPVSNPSCPMIPLYR.N | 3 | 3072.561 | 3.6 | 0.502 |

+1 charge

```
		      B   	     Y
  1	 D 	   116.03	        -  	 27
  2	 I 	   229.12	  2955.68	 26
  3	 L 	   342.20	  2842.60	 25
  4	 L 	   455.29	  2729.51	 24
  5	 P 	   552.34	  2616.43	 23
  6	 F 	   699.41	  2519.38	 22
  7	 Q 	   827.47	  2372.31	 21
  8	 G 	   884.49	  2244.25	 20
  9	 Q 	  1012.55	  2187.23	 19
 10	 L 	  1125.63	  2059.17	 18
 11	 D 	  1240.66	  1946.09	 17
 12	 S 	  1327.69	  1831.06	 16
 13	 I 	  1440.77	  1744.03	 15
 14	 P 	  1537.83	  1630.94	 14
 15	 V 	  1636.89	  1533.89	 13
 16	 S 	  1723.93	  1434.82	 12
 17	 N 	  1837.97	  1347.79	 11
 18	 P 	  1935.02	  1233.75	 10
 19	 S 	  2022.05	  1136.69	  9
 20	 C 	  2182.22	  1049.66	  8
 21	 P 	  2279.27	   889.50	  7
 22	 M 	  2410.31	   792.44	  6
 23	 I 	  2523.40	   661.40	  5
 24	 P 	  2620.45	   548.32	  4
 25	 L 	  2733.53	   451.27	  3
 26	 Y 	  2896.60	   338.18	  2
 27	 R 	        -  	   175.12	  1  _


+2 charge
		      B   	     Y
  1	 D 	    58.52	        -  	 27
  2	 I 	   115.06	  1478.34	 26
  3	 L 	   171.60	  1421.80	 25
  4	 L 	   228.15	  1365.26	 24
  5	 P 	   276.67	  1308.72	 23
  6	 F 	   350.21	  1260.19	 22
  7	 Q 	   414.24	  1186.66	 21
  8	 G 	   442.75	  1122.63	 20
  9	 Q 	   506.78	  1094.12	 19
 10	 L 	   563.32	  1030.09	 18
 11	 D 	   620.83	   973.55	 17
 12	 S 	   664.35	   916.03	 16
 13	 I 	   720.89	   872.52	 15
 14	 P 	   769.42	   815.97	 14
 15	 V 	   818.95	   767.45	 13
 16	 S 	   862.47	   717.91	 12
 17	 N 	   919.49	   674.40	 11
 18	 P 	   968.01	   617.38	 10
 19	 S 	  1011.53	   568.85	  9
 20	 C 	  1091.61	   525.33	  8
 21	 P 	  1140.14	   445.25	  7
 22	 M 	  1205.66	   396.73	  6
 23	 I 	  1262.20	   331.21	  5
 24	 P 	  1310.73	   274.66	  4
 25	 L 	  1367.27	   226.14	  3
 26	 Y 	  1448.80	   169.59	  2
 27	 R 	        -  	    88.06	  1  _


+3 charge
		      B   	     Y
  1	 D 	    39.35	        -  	 27
  2	 I 	    77.04	   985.90	 26
  3	 L 	   114.74	   948.20	 25
  4	 L 	   152.43	   910.51	 24
  5	 P 	   184.78	   872.81	 23
  6	 F 	   233.81	   840.46	 22
  7	 Q 	   276.49	   791.44	 21
  8	 G 	   295.50	   748.75	 20
  9	 Q 	   338.19	   729.75	 19
 10	 L 	   375.88	   687.06	 18
 11	 D 	   414.22	   649.37	 17
 12	 S 	   443.23	   611.02	 16
 13	 I 	   480.93	   582.01	 15
 14	 P 	   513.28	   544.32	 14
 15	 V 	   546.30	   511.97	 13
 16	 S 	   575.31	   478.95	 12
 17	 N 	   613.33	   449.93	 11
 18	 P 	   645.68	   411.92	 10
 19	 S 	   674.69	   379.57	  9
 20	 C 	   728.08	   350.56	  8
 21	 P 	   760.43	   297.17	  7
 22	 M 	   804.11	   264.82	  6
 23	 I 	   841.80	   221.14	  5
 24	 P 	   874.15	   183.44	  4
 25	 L 	   911.85	   151.09	  3
 26	 Y 	   966.20	   113.40	  2
 27	 R 	        -  	    59.04	  1  _
```

**73. IPI00479116 Carboxypeptidase N subunit 2**

| Sequence | Charge | M+H+ | XCorr | DeltaCn |
| --- | --- | --- | --- | --- |
| K.TLNLAQNLLAQLPEELFHPLTSLQTLK.L | 3 | 3047.537 | 5.437 | 0.63 |

+1 charge

```
		      B   	     Y
  1	 T 	   102.05	        -  	 27
  2	 L 	   215.14	  2944.65	 26
  3	 N 	   329.18	  2831.57	 25
  4	 L 	   442.27	  2717.52	 24
  5	 A 	   513.30	  2604.44	 23
  6	 Q 	   641.36	  2533.40	 22
  7	 N 	   755.40	  2405.34	 21
  8	 L 	   868.49	  2291.30	 20
  9	 L 	   981.57	  2178.22	 19
 10	 A 	  1052.61	  2065.13	 18
 11	 Q 	  1180.67	  1994.10	 17
 12	 L 	  1293.75	  1866.04	 16
 13	 P 	  1390.81	  1752.95	 15
 14	 E 	  1519.85	  1655.90	 14
 15	 E 	  1648.89	  1526.86	 13
 16	 L 	  1761.97	  1397.82	 12
 17	 F 	  1909.04	  1284.73	 11
 18	 H 	  2046.10	  1137.66	 10
 19	 P 	  2143.15	  1000.60	  9
 20	 L 	  2256.24	   903.55	  8
 21	 T 	  2357.29	   790.47	  7
 22	 S 	  2444.32	   689.42	  6
 23	 L 	  2557.40	   602.39	  5
 24	 Q 	  2685.46	   489.30	  4
 25	 T 	  2786.51	   361.24	  3
 26	 L 	  2899.59	   260.20	  2
 27	 K 	        -  	   147.11	  1  _


+2 charge
		      B   	     Y
  1	 T 	    51.53	        -  	 27
  2	 L 	   108.07	  1472.83	 26
  3	 N 	   165.09	  1416.29	 25
  4	 L 	   221.64	  1359.27	 24
  5	 A 	   257.16	  1302.72	 23
  6	 Q 	   321.18	  1267.20	 22
  7	 N 	   378.21	  1203.18	 21
  8	 L 	   434.75	  1146.15	 20
  9	 L 	   491.29	  1089.61	 19
 10	 A 	   526.81	  1033.07	 18
 11	 Q 	   590.84	   997.55	 17
 12	 L 	   647.38	   933.52	 16
 13	 P 	   695.91	   876.98	 15
 14	 E 	   760.43	   828.45	 14
 15	 E 	   824.95	   763.93	 13
 16	 L 	   881.49	   699.41	 12
 17	 F 	   955.03	   642.87	 11
 18	 H 	  1023.55	   569.33	 10
 19	 P 	  1072.08	   500.81	  9
 20	 L 	  1128.62	   452.28	  8
 21	 T 	  1179.15	   395.74	  7
 22	 S 	  1222.66	   345.21	  6
 23	 L 	  1279.20	   301.70	  5
 24	 Q 	  1343.23	   245.16	  4
 25	 T 	  1393.76	   181.13	  3
 26	 L 	  1450.30	   130.60	  2
 27	 K 	        -  	    74.06	  1  _


+3 charge
		      B   	     Y
  1	 T 	    34.69	        -  	 27
  2	 L 	    72.38	   982.22	 26
  3	 N 	   110.40	   944.53	 25
  4	 L 	   148.09	   906.51	 24
  5	 A 	   171.77	   868.82	 23
  6	 Q 	   214.46	   845.14	 22
  7	 N 	   252.47	   802.45	 21
  8	 L 	   290.17	   764.44	 20
  9	 L 	   327.86	   726.74	 19
 10	 A 	   351.54	   689.05	 18
 11	 Q 	   394.23	   665.37	 17
 12	 L 	   431.92	   622.68	 16
 13	 P 	   464.27	   584.99	 15
 14	 E 	   507.29	   552.64	 14
 15	 E 	   550.30	   509.62	 13
 16	 L 	   588.00	   466.61	 12
 17	 F 	   637.02	   428.92	 11
 18	 H 	   682.71	   379.89	 10
 19	 P 	   715.06	   334.21	  9
 20	 L 	   752.75	   301.86	  8
 21	 T 	   786.43	   264.16	  7
 22	 S 	   815.44	   230.48	  6
 23	 L 	   853.14	   201.47	  5
 24	 Q 	   895.83	   163.77	  4
 25	 T 	   929.51	   121.09	  3
 26	 L 	   967.20	    87.40	  2
 27	 K 	        -  	    49.71	  1  _
```

**74. IPI00152018 Isoform 1 of CMRF35-like molecule 9**

| Sequence | Charge | M+H+ | XCorr | DeltaCn |
| --- | --- | --- | --- | --- |
| R.KGGILFSR.C | 2 | 878.0538 | 3.039 | 0.397 |

+1 charge

```
		      B   	     Y
  1	 K 	   129.10	        -  	  8
  2	 G 	   186.12	   749.43	  7
  3	 G 	   243.15	   692.41	  6
  4	 I 	   356.23	   635.39	  5
  5	 L 	   469.31	   522.30	  4
  6	 F 	   616.38	   409.22	  3
  7	 S 	   703.41	   262.15	  2
  8	 R 	        -  	   175.12	  1  _
+2 charge
		      B   	     Y
  1	 K 	    65.05	        -  	  8
  2	 G 	    93.57	   375.22	  7
  3	 G 	   122.08	   346.71	  6
  4	 I 	   178.62	   318.20	  5
  5	 L 	   235.16	   261.66	  4
  6	 F 	   308.69	   205.11	  3
  7	 S 	   352.21	   131.58	  2
  8	 R 	        -  	    88.06	  1  _
```

**75. IPI00027166 Metalloproteinase inhibitor 2**

| Sequence | Charge | M+H+ | XCorr | DeltaCn |
| --- | --- | --- | --- | --- |
| K.AVSEKEVDSGNDIYGNPIKR.I | 3 | 2192.372 | 5.303 | 0.545 |

+1 charge

```
		      B   	     Y
  1	 A 	    72.04	        -  	 20
  2	 V 	   171.11	  2120.06	 19
  3	 S 	   258.14	  2020.99	 18
  4	 E 	   387.19	  1933.96	 17
  5	 K 	   515.28	  1804.92	 16
  6	 E 	   644.32	  1676.82	 15
  7	 V 	   743.39	  1547.78	 14
  8	 D 	   858.42	  1448.71	 13
  9	 S 	   945.45	  1333.69	 12
 10	 G 	  1002.47	  1246.65	 11
 11	 N 	  1116.52	  1189.63	 10
 12	 D 	  1231.54	  1075.59	  9
 13	 I 	  1344.63	   960.56	  8
 14	 Y 	  1507.69	   847.48	  7
 15	 G 	  1564.71	   684.42	  6
 16	 N 	  1678.76	   627.39	  5
 17	 P 	  1775.81	   513.35	  4
 18	 I 	  1888.89	   416.30	  3
 19	 K 	  2016.99	   303.21	  2
 20	 R 	        -  	   175.12	  1  _
+2 charge
		      B   	     Y
  1	 A 	    36.53	        -  	 20
  2	 V 	    86.06	  1060.53	 19
  3	 S 	   129.58	  1011.00	 18
  4	 E 	   194.10	   967.48	 17
  5	 K 	   258.14	   902.96	 16
  6	 E 	   322.67	   838.92	 15
  7	 V 	   372.20	   774.39	 14
  8	 D 	   429.71	   724.86	 13
  9	 S 	   473.23	   667.35	 12
 10	 G 	   501.74	   623.83	 11
 11	 N 	   558.76	   595.32	 10
 12	 D 	   616.28	   538.30	  9
 13	 I 	   672.82	   480.78	  8
 14	 Y 	   754.35	   424.24	  7
 15	 G 	   782.86	   342.71	  6
 16	 N 	   839.88	   314.20	  5
 17	 P 	   888.41	   257.18	  4
 18	 I 	   944.95	   208.65	  3
 19	 K 	  1009.00	   152.11	  2
 20	 R 	        -  	    88.06	  1  _

+3 charge
		      B   	     Y
  1	 A 	    24.69	        -  	 20
  2	 V 	    57.71	   707.36	 19
  3	 S 	    86.72	   674.34	 18
  4	 E 	   129.73	   645.33	 17
  5	 K 	   172.43	   602.31	 16
  6	 E 	   215.45	   559.61	 15
  7	 V 	   248.47	   516.60	 14
  8	 D 	   286.81	   483.58	 13
  9	 S 	   315.82	   445.23	 12
 10	 G 	   334.83	   416.22	 11
 11	 N 	   372.84	   397.22	 10
 12	 D 	   411.19	   359.20	  9
 13	 I 	   448.88	   320.86	  8
 14	 Y 	   503.24	   283.16	  7
 15	 G 	   522.24	   228.81	  6
 16	 N 	   560.26	   209.80	  5
 17	 P 	   592.61	   171.79	  4
 18	 I 	   630.30	   139.44	  3
 19	 K 	   673.00	   101.74	  2
 20	 R 	        -  	    59.04	  1  _
```

**76. IPI00061116 Mucin-like protein 1**

| Sequence | Charge | M+H+ | XCorr | DeltaCn |
| --- | --- | --- | --- | --- |
| K.WVGDLPNGR.V | 2 | 1014.119 | 2.903 | 0.29 |

+1 charge

```
		      B   	     Y
  1	 W 	   187.09	        -  	  9
  2	 V 	   286.16	   827.44	  8
  3	 G 	   343.18	   728.37	  7
  4	 D 	   458.20	   671.35	  6
  5	 L 	   571.29	   556.32	  5
  6	 P 	   668.34	   443.24	  4
  7	 N 	   782.38	   346.18	  3
  8	 G 	   839.40	   232.14	  2
  9	 R 	        -  	   175.12	  1  _
+2 charge
		      B   	     Y
  1	 W 	    94.05	        -  	  9
  2	 V 	   143.58	   414.22	  8
  3	 G 	   172.09	   364.69	  7
  4	 D 	   229.61	   336.18	  6
  5	 L 	   286.15	   278.66	  5
  6	 P 	   334.67	   222.12	  4
  7	 N 	   391.70	   173.60	  3
  8	 G 	   420.21	   116.57	  2
  9	 R 	        -  	    88.06	  1  _
```

**77. IPI00414717 Isoform 2 of Golgi apparatus protein 1**

| Sequence | Charge | M+H+ | XCorr | DeltaCn |
| --- | --- | --- | --- | --- |
| R.VAELSSDDFHLDR.H | 2 | 1504.583 | 3.073 | 0.425 |

+1 charge

```
		      B   	     Y
  1	 V 	   100.08	        -  	 13
  2	 A 	   171.11	  1404.64	 12
  3	 E 	   300.16	  1333.60	 11
  4	 L 	   413.24	  1204.56	 10
  5	 S 	   500.27	  1091.48	  9
  6	 S 	   587.30	  1004.44	  8
  7	 D 	   702.33	   917.41	  7
  8	 D 	   817.36	   802.38	  6
  9	 F 	   964.43	   687.36	  5
 10	 H 	  1101.48	   540.29	  4
 11	 L 	  1214.57	   403.23	  3
 12	 D 	  1329.60	   290.15	  2
 13	 R 	        -  	   175.12	  1  _
+2 charge
		      B   	     Y
  1	 V 	    50.54	        -  	 13
  2	 A 	    86.06	   702.82	 12
  3	 E 	   150.58	   667.30	 11
  4	 L 	   207.12	   602.78	 10
  5	 S 	   250.64	   546.24	  9
  6	 S 	   294.16	   502.73	  8
  7	 D 	   351.67	   459.21	  7
  8	 D 	   409.18	   401.70	  6
  9	 F 	   482.72	   344.18	  5
 10	 H 	   551.25	   270.65	  4
 11	 L 	   607.79	   202.12	  3
 12	 D 	   665.30	   145.58	  2
 13	 R 	        -  	    88.06	  1  _
```

**78. IPI00028347 Isoform 1 of F-box only protein 31**

| Sequence | Charge | M+H+ | XCorr | DeltaCn |
| --- | --- | --- | --- | --- |
| R.IYLPPSRPDDLIKPGLFK.G | 2 | 2070.462 | 3.167 | 0.292 |

+1 charge

```
		      B   	     Y
  1	 I 	   114.09	        -  	 18
  2	 Y 	   277.15	  1956.10	 17
  3	 L 	   390.24	  1793.03	 16
  4	 P 	   487.29	  1679.95	 15
  5	 P 	   584.34	  1582.90	 14
  6	 S 	   671.38	  1485.84	 13
  7	 R 	   827.48	  1398.81	 12
  8	 P 	   924.53	  1242.71	 11
  9	 D 	  1039.56	  1145.66	 10
 10	 D 	  1154.58	  1030.63	  9
 11	 L 	  1267.67	   915.60	  8
 12	 I 	  1380.75	   802.52	  7
 13	 K 	  1508.85	   689.43	  6
 14	 P 	  1605.90	   561.34	  5
 15	 G 	  1662.92	   464.29	  4
 16	 L 	  1776.01	   407.27	  3
 17	 F 	  1923.07	   294.18	  2
 18	 K 	        -  	   147.11	  1  _
+2 charge
		      B   	     Y
  1	 I 	    57.55	        -  	 18
  2	 Y 	   139.08	   978.55	 17
  3	 L 	   195.62	   897.02	 16
  4	 P 	   244.15	   840.48	 15
  5	 P 	   292.68	   791.95	 14
  6	 S 	   336.19	   743.42	 13
  7	 R 	   414.24	   699.91	 12
  8	 P 	   462.77	   621.86	 11
  9	 D 	   520.28	   573.33	 10
 10	 D 	   577.80	   515.82	  9
 11	 L 	   634.34	   458.30	  8
 12	 I 	   690.88	   401.76	  7
 13	 K 	   754.93	   345.22	  6
 14	 P 	   803.45	   281.17	  5
 15	 G 	   831.96	   232.65	  4
 16	 L 	   888.51	   204.14	  3
 17	 F 	   962.04	   147.59	  2
 18	 K 	        -  	    74.06	  1  _
```

**79. IPI00012007 Adenosylhomocysteinase**

| Sequence | Charge | M+H+ | XCorr | DeltaCn |
| --- | --- | --- | --- | --- |
| R.KALDIAENEMPGLMR.M | 2 | 1688.995 | 3.603 | 0.308 |

+1 charge

```
		      B   	     Y
  1	 K 	   129.10	        -  	 15
  2	 A 	   200.14	  1559.76	 14
  3	 L 	   313.22	  1488.72	 13
  4	 D 	   428.25	  1375.63	 12
  5	 I 	   541.33	  1260.61	 11
  6	 A 	   612.37	  1147.52	 10
  7	 E 	   741.41	  1076.49	  9
  8	 N 	   855.46	   947.44	  8
  9	 E 	   984.50	   833.40	  7
 10	 M 	  1115.54	   704.36	  6
 11	 P 	  1212.59	   573.32	  5
 12	 G 	  1269.61	   476.26	  4
 13	 L 	  1382.70	   419.24	  3
 14	 M 	  1513.74	   306.16	  2
 15	 R 	        -  	   175.12	  1  _
+2 charge
		      B   	     Y
  1	 K 	    65.05	        -  	 15
  2	 A 	   100.57	   780.38	 14
  3	 L 	   157.12	   744.86	 13
  4	 D 	   214.63	   688.32	 12
  5	 I 	   271.17	   630.81	 11
  6	 A 	   306.69	   574.27	 10
  7	 E 	   371.21	   538.75	  9
  8	 N 	   428.23	   474.23	  8
  9	 E 	   492.75	   417.20	  7
 10	 M 	   558.27	   352.68	  6
 11	 P 	   606.80	   287.16	  5
 12	 G 	   635.31	   238.64	  4
 13	 L 	   691.85	   210.13	  3
 14	 M 	   757.37	   153.58	  2
 15	 R 	        -  	    88.06	  1  _
```

**80. IPI00183968 tropomyosin 3 isoform 1**

| Sequence | Charge | M+H+ | XCorr | DeltaCn |
| --- | --- | --- | --- | --- |
| R.LATALQKLEEAEKAADESER.G | 3 | 2203.393 | 4.15 | 0.474 |

+1 charge

```
		      B   	     Y
  1	 L 	   114.09	        -  	 20
  2	 A 	   185.13	  2089.04	 19
  3	 T 	   286.18	  2018.00	 18
  4	 A 	   357.21	  1916.96	 17
  5	 L 	   470.30	  1845.92	 16
  6	 Q 	   598.36	  1732.83	 15
  7	 K 	   726.45	  1604.78	 14
  8	 L 	   839.53	  1476.68	 13
  9	 E 	   968.58	  1363.60	 12
 10	 E 	  1097.62	  1234.55	 11
 11	 A 	  1168.66	  1105.51	 10
 12	 E 	  1297.70	  1034.47	  9
 13	 K 	  1425.79	   905.43	  8
 14	 A 	  1496.83	   777.34	  7
 15	 A 	  1567.87	   706.30	  6
 16	 D 	  1682.90	   635.26	  5
 17	 E 	  1811.94	   520.24	  4
 18	 S 	  1898.97	   391.19	  3
 19	 E 	  2028.01	   304.16	  2
 20	 R 	        -  	   175.12	  1  _
+2 charge
		      B   	     Y
  1	 L 	    57.55	        -  	 20
  2	 A 	    93.07	  1045.02	 19
  3	 T 	   143.59	  1009.51	 18
  4	 A 	   179.11	   958.98	 17
  5	 L 	   235.65	   923.46	 16
  6	 Q 	   299.68	   866.92	 15
  7	 K 	   363.73	   802.89	 14
  8	 L 	   420.27	   738.84	 13
  9	 E 	   484.79	   682.30	 12
 10	 E 	   549.31	   617.78	 11
 11	 A 	   584.83	   553.26	 10
 12	 E 	   649.35	   517.74	  9
 13	 K 	   713.40	   453.22	  8
 14	 A 	   748.92	   389.17	  7
 15	 A 	   784.44	   353.65	  6
 16	 D 	   841.95	   318.14	  5
 17	 E 	   906.47	   260.62	  4
 18	 S 	   949.99	   196.10	  3
 19	 E 	  1014.51	   152.58	  2
 20	 R 	        -  	    88.06	  1  _

+3 charge
		      B   	     Y
  1	 L 	    38.70	        -  	 20
  2	 A 	    62.38	   697.02	 19
  3	 T 	    96.06	   673.34	 18
  4	 A 	   119.74	   639.66	 17
  5	 L 	   157.44	   615.98	 16
  6	 Q 	   200.12	   578.28	 15
  7	 K 	   242.82	   535.60	 14
  8	 L 	   280.52	   492.90	 13
  9	 E 	   323.53	   455.20	 12
 10	 E 	   366.54	   412.19	 11
 11	 A 	   390.22	   369.18	 10
 12	 E 	   433.24	   345.50	  9
 13	 K 	   475.94	   302.48	  8
 14	 A 	   499.62	   259.78	  7
 15	 A 	   523.29	   236.10	  6
 16	 D 	   561.64	   212.43	  5
 17	 E 	   604.65	   174.08	  4
 18	 S 	   633.66	   131.07	  3
 19	 E 	   676.68	   102.06	  2
 20	 R 	        -  	    59.04	  1  _
```

**81. IPI00025753 Desmoglein-1**

| Sequence | Charge | M+H+ | XCorr | DeltaCn |
| --- | --- | --- | --- | --- |
| R.YVMGNNPADLLAVDSR.T | 2 | 1735.943 | 3.458 | 0.477 |

+1 charge

```
		      B   	     Y
  1	 Y 	   164.07	        -  	 16
  2	 V 	   263.14	  1571.78	 15
  3	 M 	   394.18	  1472.72	 14
  4	 G 	   451.20	  1341.68	 13
  5	 N 	   565.24	  1284.65	 12
  6	 N 	   679.29	  1170.61	 11
  7	 P 	   776.34	  1056.57	 10
  8	 A 	   847.38	   959.52	  9
  9	 D 	   962.40	   888.48	  8
 10	 L 	  1075.49	   773.45	  7
 11	 L 	  1188.57	   660.37	  6
 12	 A 	  1259.61	   547.28	  5
 13	 V 	  1358.68	   476.25	  4
 14	 D 	  1473.70	   377.18	  3
 15	 S 	  1560.74	   262.15	  2
 16	 R 	        -  	   175.12	  1  _
+2 charge
		      B   	     Y
  1	 Y 	    82.54	        -  	 16
  2	 V 	   132.07	   786.40	 15
  3	 M 	   197.59	   736.86	 14
  4	 G 	   226.10	   671.34	 13
  5	 N 	   283.13	   642.83	 12
  6	 N 	   340.15	   585.81	 11
  7	 P 	   388.67	   528.79	 10
  8	 A 	   424.19	   480.26	  9
  9	 D 	   481.71	   444.74	  8
 10	 L 	   538.25	   387.23	  7
 11	 L 	   594.79	   330.69	  6
 12	 A 	   630.31	   274.15	  5
 13	 V 	   679.84	   238.63	  4
 14	 D 	   737.36	   189.09	  3
 15	 S 	   780.87	   131.58	  2
 16	 R 	        -  	    88.06	  1  _
```

**82. IPI00470361 Isoform 2 of Kin of IRRE-like protein 1**

| Sequence | Charge | M+H+ | XCorr | DeltaCn |
| --- | --- | --- | --- | --- |
| K.DGLALGMGQGLKAWPR.Y | 2 | 1670.961 | 3.493 | 0.458 |

+1 charge

```
		      B   	     Y
  1	 D 	   116.03	        -  	 16
  2	 G 	   173.06	  1554.86	 15
  3	 L 	   286.14	  1497.84	 14
  4	 A 	   357.18	  1384.75	 13
  5	 L 	   470.26	  1313.71	 12
  6	 G 	   527.28	  1200.63	 11
  7	 M 	   658.32	  1143.61	 10
  8	 G 	   715.34	  1012.57	  9
  9	 Q 	   843.40	   955.55	  8
 10	 G 	   900.42	   827.49	  7
 11	 L 	  1013.51	   770.47	  6
 12	 K 	  1141.60	   657.38	  5
 13	 A 	  1212.64	   529.29	  4
 14	 W 	  1398.72	   458.25	  3
 15	 P 	  1495.77	   272.17	  2
 16	 R 	        -  	   175.12	  1  _
+2 charge
		      B   	     Y
  1	 D 	    58.52	        -  	 16
  2	 G 	    87.03	   777.93	 15
  3	 L 	   143.57	   749.42	 14
  4	 A 	   179.09	   692.88	 13
  5	 L 	   235.63	   657.36	 12
  6	 G 	   264.14	   600.82	 11
  7	 M 	   329.67	   572.31	 10
  8	 G 	   358.18	   506.79	  9
  9	 Q 	   422.21	   478.28	  8
 10	 G 	   450.72	   414.25	  7
 11	 L 	   507.26	   385.74	  6
 12	 K 	   571.31	   329.20	  5
 13	 A 	   606.82	   265.15	  4
 14	 W 	   699.86	   229.63	  3
 15	 P 	   748.39	   136.59	  2
 16	 R 	        -  	    88.06	  1  _
```

**83. IPI00009477 Intercellular adhesion molecule 2**

| Sequence | Charge | M+H+ | XCorr | DeltaCn |
| --- | --- | --- | --- | --- |
| R.QVILTLQPTLVAVGK.S | 2 | 1580.937 | 3.239 | 0.315 |

+1 charge

```
		      B   	     Y
  1	 Q 	   129.07	        -  	 15
  2	 V 	   228.13	  1451.92	 14
  3	 I 	   341.22	  1352.85	 13
  4	 L 	   454.30	  1239.77	 12
  5	 T 	   555.35	  1126.68	 11
  6	 L 	   668.43	  1025.64	 10
  7	 Q 	   796.49	   912.55	  9
  8	 P 	   893.55	   784.49	  8
  9	 T 	   994.59	   687.44	  7
 10	 L 	  1107.68	   586.39	  6
 11	 V 	  1206.75	   473.31	  5
 12	 A 	  1277.78	   374.24	  4
 13	 V 	  1376.85	   303.20	  3
 14	 G 	  1433.87	   204.13	  2
 15	 K 	        -  	   147.11	  1  _
+2 charge
		      B   	     Y
  1	 Q 	    65.04	        -  	 15
  2	 V 	   114.57	   726.46	 14
  3	 I 	   171.11	   676.93	 13
  4	 L 	   227.65	   620.39	 12
  5	 T 	   278.18	   563.85	 11
  6	 L 	   334.72	   513.32	 10
  7	 Q 	   398.75	   456.78	  9
  8	 P 	   447.28	   392.75	  8
  9	 T 	   497.80	   344.22	  7
 10	 L 	   554.34	   293.70	  6
 11	 V 	   603.88	   237.16	  5
 12	 A 	   639.40	   187.62	  4
 13	 V 	   688.93	   152.10	  3
 14	 G 	   717.44	   102.57	  2
 15	 K 	        -  	    74.06	  1  _
```

**84. IPI00002460 Isoform 1 of Annexin A7**

| Sequence | Charge | M+H+ | XCorr | DeltaCn |
| --- | --- | --- | --- | --- |
| K.GFGTDEQAIVDVVANR.S | 2 | 1691.824 | 3.046 | 0.371 |

+1 charge

```
		      B   	     Y
  1	 G 	    58.03	        -  	 16
  2	 F 	   205.10	  1633.82	 15
  3	 G 	   262.12	  1486.75	 14
  4	 T 	   363.17	  1429.73	 13
  5	 D 	   478.19	  1328.68	 12
  6	 E 	   607.24	  1213.65	 11
  7	 Q 	   735.29	  1084.61	 10
  8	 A 	   806.33	   956.55	  9
  9	 I 	   919.42	   885.52	  8
 10	 V 	  1018.48	   772.43	  7
 11	 D 	  1133.51	   673.36	  6
 12	 V 	  1232.58	   558.34	  5
 13	 V 	  1331.65	   459.27	  4
 14	 A 	  1402.68	   360.20	  3
 15	 N 	  1516.73	   289.16	  2
 16	 R 	        -  	   175.12	  1  _
+2 charge
		      B   	     Y
  1	 G 	    29.52	        -  	 16
  2	 F 	   103.05	   817.41	 15
  3	 G 	   131.56	   743.88	 14
  4	 T 	   182.09	   715.37	 13
  5	 D 	   239.60	   664.84	 12
  6	 E 	   304.12	   607.33	 11
  7	 Q 	   368.15	   542.81	 10
  8	 A 	   403.67	   478.78	  9
  9	 I 	   460.21	   443.26	  8
 10	 V 	   509.75	   386.72	  7
 11	 D 	   567.26	   337.19	  6
 12	 V 	   616.79	   279.67	  5
 13	 V 	   666.33	   230.14	  4
 14	 A 	   701.85	   180.60	  3
 15	 N 	   758.87	   145.08	  2
 16	 R 	        -  	    88.06	  1  _
```

**85. IPI00290085 Cadherin-2**

| Sequence | Charge | M+H+ | XCorr | DeltaCn |
| --- | --- | --- | --- | --- |
| R.FAIQTDPNSNDGLVTVVKPIDFETNR.M | 3 | 2892.17 | 4.744 | 0.548 |

+1 charge

```
		      B   	     Y
  1	 F 	   148.08	        -  	 26
  2	 A 	   219.11	  2743.39	 25
  3	 I 	   332.20	  2672.35	 24
  4	 Q 	   460.26	  2559.27	 23
  5	 T 	   561.30	  2431.21	 22
  6	 D 	   676.33	  2330.16	 21
  7	 P 	   773.38	  2215.14	 20
  8	 N 	   887.43	  2118.08	 19
  9	 S 	   974.46	  2004.04	 18
 10	 N 	  1088.50	  1917.01	 17
 11	 D 	  1203.53	  1802.96	 16
 12	 G 	  1260.55	  1687.94	 15
 13	 L 	  1373.63	  1630.92	 14
 14	 V 	  1472.70	  1517.83	 13
 15	 T 	  1573.75	  1418.76	 12
 16	 V 	  1672.82	  1317.72	 11
 17	 V 	  1771.89	  1218.65	 10
 18	 K 	  1899.98	  1119.58	  9
 19	 P 	  1997.03	   991.48	  8
 20	 I 	  2110.12	   894.43	  7
 21	 D 	  2225.14	   781.35	  6
 22	 F 	  2372.21	   666.32	  5
 23	 E 	  2501.26	   519.25	  4
 24	 T 	  2602.30	   390.21	  3
 25	 N 	  2716.35	   289.16	  2
 26	 R 	        -  	   175.12	  1  _


+2 charge
		      B   	     Y
  1	 F 	    74.54	        -  	 26
  2	 A 	   110.06	  1372.20	 25
  3	 I 	   166.60	  1336.68	 24
  4	 Q 	   230.63	  1280.14	 23
  5	 T 	   281.16	  1216.11	 22
  6	 D 	   338.67	  1165.58	 21
  7	 P 	   387.20	  1108.07	 20
  8	 N 	   444.22	  1059.54	 19
  9	 S 	   487.73	  1002.52	 18
 10	 N 	   544.75	   959.01	 17
 11	 D 	   602.27	   901.99	 16
 12	 G 	   630.78	   844.47	 15
 13	 L 	   687.32	   815.96	 14
 14	 V 	   736.85	   759.42	 13
 15	 T 	   787.38	   709.89	 12
 16	 V 	   836.91	   659.36	 11
 17	 V 	   886.45	   609.83	 10
 18	 K 	   950.49	   560.29	  9
 19	 P 	   999.02	   496.25	  8
 20	 I 	  1055.56	   447.72	  7
 21	 D 	  1113.08	   391.18	  6
 22	 F 	  1186.61	   333.66	  5
 23	 E 	  1251.13	   260.13	  4
 24	 T 	  1301.66	   195.61	  3
 25	 N 	  1358.68	   145.08	  2
 26	 R 	        -  	    88.06	  1  _


+3 charge
		      B   	     Y
  1	 F 	    50.03	        -  	 26
  2	 A 	    73.71	   915.13	 25
  3	 I 	   111.40	   891.46	 24
  4	 Q 	   154.09	   853.76	 23
  5	 T 	   187.77	   811.07	 22
  6	 D 	   226.11	   777.39	 21
  7	 P 	   258.47	   739.05	 20
  8	 N 	   296.48	   706.70	 19
  9	 S 	   325.49	   668.68	 18
 10	 N 	   363.51	   639.67	 17
 11	 D 	   401.85	   601.66	 16
 12	 G 	   420.85	   563.32	 15
 13	 L 	   458.55	   544.31	 14
 14	 V 	   491.57	   506.62	 13
 15	 T 	   525.25	   473.59	 12
 16	 V 	   558.28	   439.91	 11
 17	 V 	   591.30	   406.89	 10
 18	 K 	   634.00	   373.86	  9
 19	 P 	   666.35	   331.17	  8
 20	 I 	   704.04	   298.82	  7
 21	 D 	   742.39	   261.12	  6
 22	 F 	   791.41	   222.78	  5
 23	 E 	   834.42	   173.76	  4
 24	 T 	   868.11	   130.74	  3
 25	 N 	   906.12	    97.06	  2
 26	 R 	        -  	    59.04	  1  _
```

**86. IPI00736885 Ig kappa chain V-II region TEW**

| Sequence | Charge | M+H+ | XCorr | DeltaCn |
| --- | --- | --- | --- | --- |
| - .DIVMTQSPLSLPVTPGEPASISCR.S | 3 | 2556.911 | 3.709 | 0.47 |

+1 charge

```
		      B   	     Y
  1	 D 	   116.03	        -  	 24
  2	 I 	   229.12	  2440.39	 23
  3	 V 	   328.19	  2327.31	 22
  4	 M 	   459.23	  2228.24	 21
  5	 T 	   560.27	  2097.20	 20
  6	 Q 	   688.33	  1996.15	 19
  7	 S 	   775.37	  1868.09	 18
  8	 P 	   872.42	  1781.06	 17
  9	 L 	   985.50	  1684.01	 16
 10	 S 	  1072.53	  1570.92	 15
 11	 L 	  1185.62	  1483.89	 14
 12	 P 	  1282.67	  1370.81	 13
 13	 V 	  1381.74	  1273.76	 12
 14	 T 	  1482.79	  1174.69	 11
 15	 P 	  1579.84	  1073.64	 10
 16	 G 	  1636.86	   976.59	  9
 17	 E 	  1765.90	   919.56	  8
 18	 P 	  1862.96	   790.52	  7
 19	 A 	  1933.99	   693.47	  6
 20	 S 	  2021.03	   622.43	  5
 21	 I 	  2134.11	   535.40	  4
 22	 S 	  2221.14	   422.32	  3
 23	 C 	  2381.31	   335.28	  2
 24	 R 	        -  	   175.12	  1  _


+2 charge
		      B   	     Y
  1	 D 	    58.52	        -  	 24
  2	 I 	   115.06	  1220.70	 23
  3	 V 	   164.60	  1164.16	 22
  4	 M 	   230.12	  1114.62	 21
  5	 T 	   280.64	  1049.10	 20
  6	 Q 	   344.67	   998.58	 19
  7	 S 	   388.19	   934.55	 18
  8	 P 	   436.71	   891.03	 17
  9	 L 	   493.25	   842.51	 16
 10	 S 	   536.77	   785.97	 15
 11	 L 	   593.31	   742.45	 14
 12	 P 	   641.84	   685.91	 13
 13	 V 	   691.37	   637.38	 12
 14	 T 	   741.90	   587.85	 11
 15	 P 	   790.42	   537.32	 10
 16	 G 	   818.93	   488.80	  9
 17	 E 	   883.46	   460.29	  8
 18	 P 	   931.98	   395.76	  7
 19	 A 	   967.50	   347.24	  6
 20	 S 	  1011.02	   311.72	  5
 21	 I 	  1067.56	   268.20	  4
 22	 S 	  1111.07	   211.66	  3
 23	 C 	  1191.16	   168.15	  2
 24	 R 	        -  	    88.06	  1  _


+3 charge
		      B   	     Y
  1	 D 	    39.35	        -  	 24
  2	 I 	    77.04	   814.14	 23
  3	 V 	   110.07	   776.44	 22
  4	 M 	   153.75	   743.42	 21
  5	 T 	   187.43	   699.74	 20
  6	 Q 	   230.12	   666.06	 19
  7	 S 	   259.13	   623.37	 18
  8	 P 	   291.48	   594.36	 17
  9	 L 	   329.17	   562.01	 16
 10	 S 	   358.18	   524.31	 15
 11	 L 	   395.88	   495.30	 14
 12	 P 	   428.23	   457.61	 13
 13	 V 	   461.25	   425.26	 12
 14	 T 	   494.93	   392.23	 11
 15	 P 	   527.28	   358.55	 10
 16	 G 	   546.29	   326.20	  9
 17	 E 	   589.31	   307.19	  8
 18	 P 	   621.66	   264.18	  7
 19	 A 	   645.34	   231.83	  6
 20	 S 	   674.35	   208.15	  5
 21	 I 	   712.04	   179.14	  4
 22	 S 	   741.05	   141.44	  3
 23	 C 	   794.44	   112.43	  2
 24	 R 	        -  	    59.04	  1  _
```

**87. IPI00554648 Keratin, type II cytoskeletal 8**

| Sequence | Charge | M+H+ | XCorr | DeltaCn |
| --- | --- | --- | --- | --- |
| R.ASLEAAIADAEQRGELAIKDANAK.L | 3 | 2456.695 | 3.968 | 0.375 |

+1 charge

```
		      B   	     Y
  1	 A 	    72.04	        -  	 24
  2	 S 	   159.08	  2384.24	 23
  3	 L 	   272.16	  2297.21	 22
  4	 E 	   401.20	  2184.13	 21
  5	 A 	   472.24	  2055.08	 20
  6	 A 	   543.28	  1984.05	 19
  7	 I 	   656.36	  1913.01	 18
  8	 A 	   727.40	  1799.92	 17
  9	 D 	   842.43	  1728.89	 16
 10	 A 	   913.46	  1613.86	 15
 11	 E 	  1042.51	  1542.82	 14
 12	 Q 	  1170.56	  1413.78	 13
 13	 R 	  1326.66	  1285.72	 12
 14	 G 	  1383.69	  1129.62	 11
 15	 E 	  1512.73	  1072.60	 10
 16	 L 	  1625.81	   943.56	  9
 17	 A 	  1696.85	   830.47	  8
 18	 I 	  1809.93	   759.44	  7
 19	 K 	  1938.03	   646.35	  6
 20	 D 	  2053.06	   518.26	  5
 21	 A 	  2124.09	   403.23	  4
 22	 N 	  2238.14	   332.19	  3
 23	 A 	  2309.17	   218.15	  2
 24	 K 	        -  	   147.11	  1  _


+2 charge
		      B   	     Y
  1	 A 	    36.53	        -  	 24
  2	 S 	    80.04	  1192.62	 23
  3	 L 	   136.58	  1149.11	 22
  4	 E 	   201.11	  1092.57	 21
  5	 A 	   236.62	  1028.05	 20
  6	 A 	   272.14	   992.53	 19
  7	 I 	   328.68	   957.01	 18
  8	 A 	   364.20	   900.47	 17
  9	 D 	   421.72	   864.95	 16
 10	 A 	   457.23	   807.43	 15
 11	 E 	   521.76	   771.92	 14
 12	 Q 	   585.79	   707.39	 13
 13	 R 	   663.84	   643.36	 12
 14	 G 	   692.35	   565.31	 11
 15	 E 	   756.87	   536.80	 10
 16	 L 	   813.41	   472.28	  9
 17	 A 	   848.93	   415.74	  8
 18	 I 	   905.47	   380.22	  7
 19	 K 	   969.52	   323.68	  6
 20	 D 	  1027.03	   259.63	  5
 21	 A 	  1062.55	   202.12	  4
 22	 N 	  1119.57	   166.60	  3
 23	 A 	  1155.09	   109.58	  2
 24	 K 	        -  	    74.06	  1  _


+3 charge
		      B   	     Y
  1	 A 	    24.69	        -  	 24
  2	 S 	    53.70	   795.42	 23
  3	 L 	    91.39	   766.41	 22
  4	 E 	   134.41	   728.71	 21
  5	 A 	   158.08	   685.70	 20
  6	 A 	   181.76	   662.02	 19
  7	 I 	   219.46	   638.34	 18
  8	 A 	   243.14	   600.65	 17
  9	 D 	   281.48	   576.97	 16
 10	 A 	   305.16	   538.63	 15
 11	 E 	   348.17	   514.95	 14
 12	 Q 	   390.86	   471.93	 13
 13	 R 	   442.89	   429.25	 12
 14	 G 	   461.90	   377.21	 11
 15	 E 	   504.91	   358.20	 10
 16	 L 	   542.61	   315.19	  9
 17	 A 	   566.29	   277.50	  8
 18	 I 	   603.98	   253.82	  7
 19	 K 	   646.68	   216.12	  6
 20	 D 	   685.02	   173.42	  5
 21	 A 	   708.70	   135.08	  4
 22	 N 	   746.72	   111.40	  3
 23	 A 	   770.40	    73.39	  2
 24	 K 	        -  	    49.71	  1  _
```

**88. IPI00218414 Carbonic anhydrase 2**

| Sequence | Charge | M+H+ | XCorr | DeltaCn |
| --- | --- | --- | --- | --- |
| K.AVQQPDGLAVLGIFLK.V | 2 | 1669.989 | 3.569 | 0.611 |

+1 charge

```
		      B   	     Y
  1	 A 	    72.04	        -  	 16
  2	 V 	   171.11	  1597.93	 15
  3	 Q 	   299.17	  1498.86	 14
  4	 Q 	   427.23	  1370.80	 13
  5	 P 	   524.28	  1242.75	 12
  6	 D 	   639.31	  1145.69	 11
  7	 G 	   696.33	  1030.67	 10
  8	 L 	   809.42	   973.64	  9
  9	 A 	   880.45	   860.56	  8
 10	 V 	   979.52	   789.52	  7
 11	 L 	  1092.60	   690.45	  6
 12	 G 	  1149.63	   577.37	  5
 13	 I 	  1262.71	   520.35	  4
 14	 F 	  1409.78	   407.27	  3
 15	 L 	  1522.86	   260.20	  2
 16	 K 	        -  	   147.11	  1  _
+2 charge
		      B   	     Y
  1	 A 	    36.53	        -  	 16
  2	 V 	    86.06	   799.47	 15
  3	 Q 	   150.09	   749.94	 14
  4	 Q 	   214.12	   685.91	 13
  5	 P 	   262.64	   621.88	 12
  6	 D 	   320.16	   573.35	 11
  7	 G 	   348.67	   515.84	 10
  8	 L 	   405.21	   487.33	  9
  9	 A 	   440.73	   430.78	  8
 10	 V 	   490.26	   395.27	  7
 11	 L 	   546.81	   345.73	  6
 12	 G 	   575.32	   289.19	  5
 13	 I 	   631.86	   260.68	  4
 14	 F 	   705.39	   204.14	  3
 15	 L 	   761.94	   130.60	  2
 16	 K 	        -  	    74.06	  1  _
```

**89. IPI00005573 Isoform 1 of 5'(3')-deoxyribonucleotidase, cytosolic type**

| Sequence | Charge | M+H+ | XCorr | DeltaCn |
| --- | --- | --- | --- | --- |
| R.VLVDMDGVLADFEAGLLR.G | 2 | 1934.246 | 5.827 | 0.599 |

+1 charge

```
		      B   	     Y
  1	 V 	   100.08	        -  	 18
  2	 L 	   213.16	  1833.94	 17
  3	 V 	   312.23	  1720.86	 16
  4	 D 	   427.26	  1621.79	 15
  5	 M 	   558.30	  1506.76	 14
  6	 D 	   673.32	  1375.72	 13
  7	 G 	   730.34	  1260.69	 12
  8	 V 	   829.41	  1203.67	 11
  9	 L 	   942.50	  1104.60	 10
 10	 A 	  1013.53	   991.52	  9
 11	 D 	  1128.56	   920.48	  8
 12	 F 	  1275.63	   805.46	  7
 13	 E 	  1404.67	   658.39	  6
 14	 A 	  1475.71	   529.35	  5
 15	 G 	  1532.73	   458.31	  4
 16	 L 	  1645.81	   401.29	  3
 17	 L 	  1758.90	   288.20	  2
 18	 R 	        -  	   175.12	  1  _
+2 charge
		      B   	     Y
  1	 V 	    50.54	        -  	 18
  2	 L 	   107.08	   917.47	 17
  3	 V 	   156.62	   860.93	 16
  4	 D 	   214.13	   811.40	 15
  5	 M 	   279.65	   753.88	 14
  6	 D 	   337.16	   688.36	 13
  7	 G 	   365.68	   630.85	 12
  8	 V 	   415.21	   602.34	 11
  9	 L 	   471.75	   552.81	 10
 10	 A 	   507.27	   496.26	  9
 11	 D 	   564.78	   460.75	  8
 12	 F 	   638.32	   403.23	  7
 13	 E 	   702.84	   329.70	  6
 14	 A 	   738.36	   265.18	  5
 15	 G 	   766.87	   229.66	  4
 16	 L 	   823.41	   201.15	  3
 17	 L 	   879.95	   144.61	  2
 18	 R 	        -  	    88.06	  1  _
```

**90. IPI00942117 Cysteine-rich secretory protein 3**

| Sequence | Charge | M+H+ | XCorr | DeltaCn |
| --- | --- | --- | --- | --- |
| K.MEWNKEAAANAQK.W | 2 | 1491.654 | 3.823 | 0.334 |

+1 charge

```
		      B   	     Y
  1	 M 	   132.05	        -  	 13
  2	 E 	   261.09	  1359.67	 12
  3	 W 	   447.17	  1230.62	 11
  4	 N 	   561.21	  1044.54	 10
  5	 K 	   689.31	   930.50	  9
  6	 E 	   818.35	   802.41	  8
  7	 A 	   889.39	   673.36	  7
  8	 A 	   960.42	   602.33	  6
  9	 A 	  1031.46	   531.29	  5
 10	 N 	  1145.50	   460.25	  4
 11	 A 	  1216.54	   346.21	  3
 12	 Q 	  1344.60	   275.17	  2
 13	 K 	        -  	   147.11	  1  _
+2 charge
		      B   	     Y
  1	 M 	    66.53	        -  	 13
  2	 E 	   131.05	   680.34	 12
  3	 W 	   224.09	   615.81	 11
  4	 N 	   281.11	   522.78	 10
  5	 K 	   345.16	   465.75	  9
  6	 E 	   409.68	   401.71	  8
  7	 A 	   445.20	   337.19	  7
  8	 A 	   480.72	   301.67	  6
  9	 A 	   516.23	   266.15	  5
 10	 N 	   573.26	   230.63	  4
 11	 A 	   608.77	   173.61	  3
 12	 Q 	   672.80	   138.09	  2
 13	 K 	        -  	    74.06	  1  _
```

**91. IPI00939709 Isoform 4 of IST1 homolog**

| Sequence | Charge | M+H+ | XCorr | DeltaCn |
| --- | --- | --- | --- | --- |
| K.EIADYLAAGKDER.A | 2 | 1451.563 | 3.096 | 0.362 |

+1 charge

```
		      B   	     Y
  1	 E 	   130.05	        -  	 13
  2	 I 	   243.13	  1321.67	 12
  3	 A 	   314.17	  1208.59	 11
  4	 D 	   429.20	  1137.55	 10
  5	 Y 	   592.26	  1022.53	  9
  6	 L 	   705.35	   859.46	  8
  7	 A 	   776.38	   746.38	  7
  8	 A 	   847.42	   675.34	  6
  9	 G 	   904.44	   604.30	  5
 10	 K 	  1032.54	   547.28	  4
 11	 D 	  1147.56	   419.19	  3
 12	 E 	  1276.61	   304.16	  2
 13	 R 	        -  	   175.12	  1  _
+2 charge
		      B   	     Y
  1	 E 	    65.53	        -  	 13
  2	 I 	   122.07	   661.34	 12
  3	 A 	   157.59	   604.80	 11
  4	 D 	   215.10	   569.28	 10
  5	 Y 	   296.63	   511.77	  9
  6	 L 	   353.18	   430.24	  8
  7	 A 	   388.69	   373.69	  7
  8	 A 	   424.21	   338.17	  6
  9	 G 	   452.72	   302.66	  5
 10	 K 	   516.77	   274.15	  4
 11	 D 	   574.29	   210.10	  3
 12	 E 	   638.81	   152.58	  2
 13	 R 	        -  	    88.06	  1  _
```

**92. IPI00651768 Isoform 1 of Hepatitis A virus cellular receptor 2**

| Sequence | Charge | M+H+ | XCorr | DeltaCn |
| --- | --- | --- | --- | --- |
| K.GACPVFECGNVVLR.T | 2 | 1578.782 | 4.149 | 0.341 |

+1 charge

```
		      B   	     Y
  1	 G 	    58.03	        -  	 14
  2	 A 	   129.07	  1521.00	 13
  3	 C 	   289.23	  1449.97	 12
  4	 P 	   386.28	  1289.80	 11
  5	 V 	   485.35	  1192.75	 10
  6	 F 	   632.42	  1093.68	  9
  7	 E 	   761.46	   946.61	  8
  8	 C 	   921.63	   817.57	  7
  9	 G 	   978.65	   657.40	  6
 10	 N 	  1092.69	   600.38	  5
 11	 V 	  1191.76	   486.34	  4
 12	 V 	  1290.83	   387.27	  3
 13	 L 	  1403.91	   288.20	  2
 14	 R 	        -  	   175.12	  1  _
+2 charge
		      B   	     Y
  1	 G 	    29.52	        -  	 14
  2	 A 	    65.04	   761.01	 13
  3	 C 	   145.12	   725.49	 12
  4	 P 	   193.65	   645.40	 11
  5	 V 	   243.18	   596.88	 10
  6	 F 	   316.71	   547.34	  9
  7	 E 	   381.24	   473.81	  8
  8	 C 	   461.32	   409.29	  7
  9	 G 	   489.83	   329.21	  6
 10	 N 	   546.85	   300.70	  5
 11	 V 	   596.38	   243.67	  4
 12	 V 	   645.92	   194.14	  3
 13	 L 	   702.46	   144.61	  2
 14	 R 	        -  	    88.06	  1  _
```

**93. IPI00829834 Ig kappa chain V-III region VH (Fragment)**

| Sequence | Charge | M+H+ | XCorr | DeltaCn |
| --- | --- | --- | --- | --- |
| R.EIVMTQSPPTLSLSPGER.V | 2 | 1943.211 | 3.874 | 0.389 |

+1 charge

```
		      B   	     Y
  1	 E 	   130.05	        -  	 18
  2	 I 	   243.13	  1812.95	 17
  3	 V 	   342.20	  1699.87	 16
  4	 M 	   473.24	  1600.80	 15
  5	 T 	   574.29	  1469.76	 14
  6	 Q 	   702.35	  1368.71	 13
  7	 S 	   789.38	  1240.65	 12
  8	 P 	   886.43	  1153.62	 11
  9	 P 	   983.49	  1056.57	 10
 10	 T 	  1084.53	   959.52	  9
 11	 L 	  1197.62	   858.47	  8
 12	 S 	  1284.65	   745.38	  7
 13	 L 	  1397.73	   658.35	  6
 14	 S 	  1484.77	   545.27	  5
 15	 P 	  1581.82	   458.24	  4
 16	 G 	  1638.84	   361.18	  3
 17	 E 	  1767.88	   304.16	  2
 18	 R 	        -  	   175.12	  1  _
+2 charge
		      B   	     Y
  1	 E 	    65.53	        -  	 18
  2	 I 	   122.07	   906.98	 17
  3	 V 	   171.60	   850.44	 16
  4	 M 	   237.13	   800.90	 15
  5	 T 	   287.65	   735.38	 14
  6	 Q 	   351.68	   684.86	 13
  7	 S 	   395.19	   620.83	 12
  8	 P 	   443.72	   577.31	 11
  9	 P 	   492.25	   528.79	 10
 10	 T 	   542.77	   480.26	  9
 11	 L 	   599.31	   429.74	  8
 12	 S 	   642.83	   373.20	  7
 13	 L 	   699.37	   329.68	  6
 14	 S 	   742.89	   273.14	  5
 15	 P 	   791.41	   229.62	  4
 16	 G 	   819.92	   181.10	  3
 17	 E 	   884.45	   152.58	  2
 18	 R 	        -  	    88.06	  1  _
```

**94. IPI00784119 V-type proton ATPase subunit S1**

| Sequence | Charge | M+H+ | XCorr | DeltaCn |
| --- | --- | --- | --- | --- |
| K.LGASPLHVDLATLR.E | 2 | 1463.707 | 4.12 | 0.581 |

+1 charge

```
		      B   	     Y
  1	 L 	   114.09	        -  	 14
  2	 G 	   171.11	  1349.75	 13
  3	 A 	   242.15	  1292.73	 12
  4	 S 	   329.18	  1221.69	 11
  5	 P 	   426.23	  1134.66	 10
  6	 L 	   539.32	  1037.61	  9
  7	 H 	   676.38	   924.53	  8
  8	 V 	   775.45	   787.47	  7
  9	 D 	   890.47	   688.40	  6
 10	 L 	  1003.56	   573.37	  5
 11	 A 	  1074.59	   460.29	  4
 12	 T 	  1175.64	   389.25	  3
 13	 L 	  1288.73	   288.20	  2
 14	 R 	        -  	   175.12	  1  _
+2 charge
		      B   	     Y
  1	 L 	    57.55	        -  	 14
  2	 G 	    86.06	   675.38	 13
  3	 A 	   121.58	   646.87	 12
  4	 S 	   165.09	   611.35	 11
  5	 P 	   213.62	   567.84	 10
  6	 L 	   270.16	   519.31	  9
  7	 H 	   338.69	   462.77	  8
  8	 V 	   388.23	   394.24	  7
  9	 D 	   445.74	   344.70	  6
 10	 L 	   502.28	   287.19	  5
 11	 A 	   537.80	   230.65	  4
 12	 T 	   588.32	   195.13	  3
 13	 L 	   644.87	   144.61	  2
 14	 R 	        -  	    88.06	  1  _
```

**95. IPI00219446 Phosphatidylethanolamine-binding protein 1**

| Sequence | Charge | M+H+ | XCorr | DeltaCn |
| --- | --- | --- | --- | --- |
| R.APVAGTCYQAEWDDYVPK.L | 2 | 2071.225 | 2.849 | 0.572 |

+1 charge

```
		      B   	     Y
  1	 A 	    72.04	        -  	 18
  2	 P 	   169.10	  1999.02	 17
  3	 V 	   268.17	  1901.97	 16
  4	 A 	   339.20	  1802.90	 15
  5	 G 	   396.22	  1731.87	 14
  6	 T 	   497.27	  1674.85	 13
  7	 C 	   657.44	  1573.80	 12
  8	 Y 	   820.50	  1413.63	 11
  9	 Q 	   948.56	  1250.57	 10
 10	 A 	  1019.60	  1122.51	  9
 11	 E 	  1148.64	  1051.47	  8
 12	 W 	  1334.72	   922.43	  7
 13	 D 	  1449.74	   736.35	  6
 14	 D 	  1564.77	   621.32	  5
 15	 Y 	  1727.84	   506.30	  4
 16	 V 	  1826.90	   343.23	  3
 17	 P 	  1923.96	   244.17	  2
 18	 K 	        -  	   147.11	  1  _
+2 charge
		      B   	     Y
  1	 A 	    36.53	        -  	 18
  2	 P 	    85.05	  1000.02	 17
  3	 V 	   134.59	   951.49	 16
  4	 A 	   170.10	   901.96	 15
  5	 G 	   198.62	   866.44	 14
  6	 T 	   249.14	   837.93	 13
  7	 C 	   329.22	   787.40	 12
  8	 Y 	   410.75	   707.32	 11
  9	 Q 	   474.78	   625.79	 10
 10	 A 	   510.30	   561.76	  9
 11	 E 	   574.82	   526.24	  8
 12	 W 	   667.86	   461.72	  7
 13	 D 	   725.38	   368.68	  6
 14	 D 	   782.89	   311.17	  5
 15	 Y 	   864.42	   253.65	  4
 16	 V 	   913.96	   172.12	  3
 17	 P 	   962.48	   122.59	  2
 18	 K 	        -  	    74.06	  1  _
```

**96. IPI00021841 Apolipoprotein A-I**

| Sequence | Charge | M+H+ | XCorr | DeltaCn |
| --- | --- | --- | --- | --- |
| K.LLDNWDSVTSTFSK.L | 2 | 1613.75 | 3.147 | 0.459 |

+1 charge

```
		      B   	     Y
  1	 L 	   114.09	        -  	 14
  2	 L 	   227.18	  1499.70	 13
  3	 D 	   342.20	  1386.62	 12
  4	 N 	   456.25	  1271.59	 11
  5	 W 	   642.32	  1157.55	 10
  6	 D 	   757.35	   971.47	  9
  7	 S 	   844.38	   856.44	  8
  8	 V 	   943.45	   769.41	  7
  9	 T 	  1044.50	   670.34	  6
 10	 S 	  1131.53	   569.29	  5
 11	 T 	  1232.58	   482.26	  4
 12	 F 	  1379.65	   381.21	  3
 13	 S 	  1466.68	   234.14	  2
 14	 K 	        -  	   147.11	  1  _
+2 charge
		      B   	     Y
  1	 L 	    57.55	        -  	 14
  2	 L 	   114.09	   750.35	 13
  3	 D 	   171.60	   693.81	 12
  4	 N 	   228.63	   636.30	 11
  5	 W 	   321.67	   579.28	 10
  6	 D 	   379.18	   486.24	  9
  7	 S 	   422.70	   428.72	  8
  8	 V 	   472.23	   385.21	  7
  9	 T 	   522.75	   335.67	  6
 10	 S 	   566.27	   285.15	  5
 11	 T 	   616.79	   241.63	  4
 12	 F 	   690.33	   191.11	  3
 13	 S 	   733.84	   117.58	  2
 14	 K 	        -  	    74.06	  1  _
```

**97. IPI00005707 C-type mannose receptor 2**

| Sequence | Charge | M+H+ | XCorr | DeltaCn |
| --- | --- | --- | --- | --- |
| R.TLGDQLSLLLGAR.T | 2 | 1357.581 | 3.866 | 0.378 |

+1 charge

```
		      B   	     Y
  1	 T 	   102.05	        -  	 13
  2	 L 	   215.14	  1255.74	 12
  3	 G 	   272.16	  1142.65	 11
  4	 D 	   387.19	  1085.63	 10
  5	 Q 	   515.25	   970.60	  9
  6	 L 	   628.33	   842.55	  8
  7	 S 	   715.36	   729.46	  7
  8	 L 	   828.45	   642.43	  6
  9	 L 	   941.53	   529.35	  5
 10	 L 	  1054.61	   416.26	  4
 11	 G 	  1111.64	   303.18	  3
 12	 A 	  1182.67	   246.16	  2
 13	 R 	        -  	   175.12	  1  _
+2 charge
		      B   	     Y
  1	 T 	    51.53	        -  	 13
  2	 L 	   108.07	   628.37	 12
  3	 G 	   136.58	   571.83	 11
  4	 D 	   194.10	   543.32	 10
  5	 Q 	   258.13	   485.81	  9
  6	 L 	   314.67	   421.78	  8
  7	 S 	   358.18	   365.23	  7
  8	 L 	   414.73	   321.72	  6
  9	 L 	   471.27	   265.18	  5
 10	 L 	   527.81	   208.63	  4
 11	 G 	   556.32	   152.09	  3
 12	 A 	   591.84	   123.58	  2
 13	 R 	        -  	    88.06	  1  _
```

**98. IPI00010790 Biglycan**

| Sequence | Charge | M+H+ | XCorr | DeltaCn |
| --- | --- | --- | --- | --- |
| K.DLPETLNELHLDHNKIQAIELEDLLR.Y | 3 | 3083.442 | 5.456 | 0.584 |

+1 charge

```
		      B   	     Y
  1	 D 	   116.03	        -  	 26
  2	 L 	   229.12	  2966.59	 25
  3	 P 	   326.17	  2853.51	 24
  4	 E 	   455.21	  2756.46	 23
  5	 T 	   556.26	  2627.42	 22
  6	 L 	   669.35	  2526.37	 21
  7	 N 	   783.39	  2413.28	 20
  8	 E 	   912.43	  2299.24	 19
  9	 L 	  1025.51	  2170.20	 18
 10	 H 	  1162.57	  2057.11	 17
 11	 L 	  1275.66	  1920.05	 16
 12	 D 	  1390.68	  1806.97	 15
 13	 H 	  1527.74	  1691.94	 14
 14	 N 	  1641.79	  1554.88	 13
 15	 K 	  1769.88	  1440.84	 12
 16	 I 	  1882.97	  1312.75	 11
 17	 Q 	  2011.02	  1199.66	 10
 18	 A 	  2082.06	  1071.60	  9
 19	 I 	  2195.15	  1000.57	  8
 20	 E 	  2324.19	   887.48	  7
 21	 L 	  2437.27	   758.44	  6
 22	 E 	  2566.31	   645.36	  5
 23	 D 	  2681.34	   516.31	  4
 24	 L 	  2794.43	   401.29	  3
 25	 L 	  2907.51	   288.20	  2
 26	 R 	        -  	   175.12	  1  _


+2 charge
		      B   	     Y
  1	 D 	    58.52	        -  	 26
  2	 L 	   115.06	  1483.80	 25
  3	 P 	   163.59	  1427.26	 24
  4	 E 	   228.11	  1378.73	 23
  5	 T 	   278.63	  1314.21	 22
  6	 L 	   335.18	  1263.69	 21
  7	 N 	   392.20	  1207.15	 20
  8	 E 	   456.72	  1150.12	 19
  9	 L 	   513.26	  1085.60	 18
 10	 H 	   581.79	  1029.06	 17
 11	 L 	   638.33	   960.53	 16
 12	 D 	   695.85	   903.99	 15
 13	 H 	   764.38	   846.48	 14
 14	 N 	   821.40	   777.95	 13
 15	 K 	   885.44	   720.92	 12
 16	 I 	   941.99	   656.88	 11
 17	 Q 	  1006.02	   600.34	 10
 18	 A 	  1041.53	   536.31	  9
 19	 I 	  1098.08	   500.79	  8
 20	 E 	  1162.60	   444.25	  7
 21	 L 	  1219.14	   379.72	  6
 22	 E 	  1283.66	   323.18	  5
 23	 D 	  1341.17	   258.66	  4
 24	 L 	  1397.72	   201.15	  3
 25	 L 	  1454.26	   144.61	  2
 26	 R 	        -  	    88.06	  1  _


+3 charge
		      B   	     Y
  1	 D 	    39.35	        -  	 26
  2	 L 	    77.04	   989.54	 25
  3	 P 	   109.40	   951.84	 24
  4	 E 	   152.41	   919.49	 23
  5	 T 	   186.09	   876.48	 22
  6	 L 	   223.79	   842.79	 21
  7	 N 	   261.80	   805.10	 20
  8	 E 	   304.82	   767.09	 19
  9	 L 	   342.51	   724.07	 18
 10	 H 	   388.20	   686.38	 17
 11	 L 	   425.89	   640.69	 16
 12	 D 	   464.23	   603.00	 15
 13	 H 	   509.92	   564.65	 14
 14	 N 	   547.93	   518.97	 13
 15	 K 	   590.63	   480.95	 12
 16	 I 	   628.33	   438.25	 11
 17	 Q 	   671.01	   400.56	 10
 18	 A 	   694.69	   357.87	  9
 19	 I 	   732.39	   334.19	  8
 20	 E 	   775.40	   296.50	  7
 21	 L 	   813.10	   253.49	  6
 22	 E 	   856.11	   215.79	  5
 23	 D 	   894.45	   172.78	  4
 24	 L 	   932.15	   134.43	  3
 25	 L 	   969.84	    96.74	  2
 26	 R 	        -  	    59.04	  1  _
```

**99. IPI00020407 Alpha-1,6-mannosylglycoprotein 6-beta-N-acetylglucosaminyltransferase A**

| Sequence | Charge | M+H+ | XCorr | DeltaCn |
| --- | --- | --- | --- | --- |
| K.TLAVLLDNILQR.I | 2 | 1369.635 | 3.539 | 0.234 |

+1 charge

```
		      B   	     Y
  1	 T 	   102.05	        -  	 12
  2	 L 	   215.14	  1267.77	 11
  3	 A 	   286.18	  1154.69	 10
  4	 V 	   385.24	  1083.65	  9
  5	 L 	   498.33	   984.58	  8
  6	 L 	   611.41	   871.50	  7
  7	 D 	   726.44	   758.42	  6
  8	 N 	   840.48	   643.39	  5
  9	 I 	   953.57	   529.35	  4
 10	 L 	  1066.65	   416.26	  3
 11	 Q 	  1194.71	   303.18	  2
 12	 R 	        -  	   175.12	  1  _
+2 charge
		      B   	     Y
  1	 T 	    51.53	        -  	 12
  2	 L 	   108.07	   634.39	 11
  3	 A 	   143.59	   577.85	 10
  4	 V 	   193.13	   542.33	  9
  5	 L 	   249.67	   492.80	  8
  6	 L 	   306.21	   436.25	  7
  7	 D 	   363.72	   379.71	  6
  8	 N 	   420.74	   322.20	  5
  9	 I 	   477.29	   265.18	  4
 10	 L 	   533.83	   208.63	  3
 11	 Q 	   597.86	   152.09	  2
 12	 R 	        -  	    88.06	  1  _
```

**100. IPI00027235 Isoform 1 of Attractin**

| Sequence | Charge | M+H+ | XCorr | DeltaCn |
| --- | --- | --- | --- | --- |
| R.SCALDQNCQWEPR.N | 2 | 1664.745 | 4.077 | 0.413 |

+1 charge

```
		      B   	     Y
  1	 S 	    88.04	        -  	 13
  2	 C 	   248.20	  1576.93	 12
  3	 A 	   319.24	  1416.77	 11
  4	 L 	   432.33	  1345.73	 10
  5	 D 	   547.35	  1232.65	  9
  6	 Q 	   675.41	  1117.62	  8
  7	 N 	   789.45	   989.56	  7
  8	 C 	   949.62	   875.52	  6
  9	 Q 	  1077.68	   715.35	  5
 10	 W 	  1263.76	   587.29	  4
 11	 E 	  1392.80	   401.21	  3
 12	 P 	  1489.85	   272.17	  2
 13	 R 	        -  	   175.12	  1  _
+2 charge
		      B   	     Y
  1	 S 	    44.52	        -  	 13
  2	 C 	   124.61	   788.97	 12
  3	 A 	   160.12	   708.89	 11
  4	 L 	   216.67	   673.37	 10
  5	 D 	   274.18	   616.83	  9
  6	 Q 	   338.21	   559.31	  8
  7	 N 	   395.23	   495.28	  7
  8	 C 	   475.31	   438.26	  6
  9	 Q 	   539.34	   358.18	  5
 10	 W 	   632.38	   294.15	  4
 11	 E 	   696.90	   201.11	  3
 12	 P 	   745.43	   136.59	  2
 13	 R 	        -  	    88.06	  1  _
```

**101. IPI00022284 Major prion protein**

| Sequence | Charge | M+H+ | XCorr | DeltaCn |
| --- | --- | --- | --- | --- |
| R.VVEQMCITQYER.E | 2 | 1556.76 | 4.121 | 0.568 |

+1 charge

```
		      B   	     Y
  1	 V 	   100.08	        -  	 12
  2	 V 	   199.14	  1456.79	 11
  3	 E 	   328.19	  1357.72	 10
  4	 Q 	   456.25	  1228.68	  9
  5	 M 	   587.29	  1100.62	  8
  6	 C 	   747.45	   969.58	  7
  7	 I 	   860.54	   809.42	  6
  8	 T 	   961.58	   696.33	  5
  9	 Q 	  1089.64	   595.28	  4
 10	 Y 	  1252.70	   467.22	  3
 11	 E 	  1381.75	   304.16	  2
 12	 R 	        -  	   175.12	  1  _
+2 charge
		      B   	     Y
  1	 V 	    50.54	        -  	 12
  2	 V 	   100.08	   728.90	 11
  3	 E 	   164.60	   679.36	 10
  4	 Q 	   228.63	   614.84	  9
  5	 M 	   294.15	   550.81	  8
  6	 C 	   374.23	   485.29	  7
  7	 I 	   430.77	   405.21	  6
  8	 T 	   481.30	   348.67	  5
  9	 Q 	   545.32	   298.15	  4
 10	 Y 	   626.86	   234.12	  3
 11	 E 	   691.38	   152.58	  2
 12	 R 	        -  	    88.06	  1  _
```

**102. IPI00178926 Immunoglobulin J chain**

| Sequence | Charge | M+H+ | XCorr | DeltaCn |
| --- | --- | --- | --- | --- |
| R.FVYHLSDLCK.K | 2 | 1282.463 | 3.492 | 0.502 |

+1 charge

```
		      B   	     Y
  1	 F 	   148.08	        -  	 10
  2	 V 	   247.14	  1134.70	  9
  3	 Y 	   410.21	  1035.63	  8
  4	 H 	   547.27	   872.56	  7
  5	 L 	   660.35	   735.51	  6
  6	 S 	   747.38	   622.42	  5
  7	 D 	   862.41	   535.39	  4
  8	 L 	   975.49	   420.36	  3
  9	 C 	  1135.66	   307.28	  2
 10	 K 	        -  	   147.11	  1  _
+2 charge
		      B   	     Y
  1	 F 	    74.54	        -  	 10
  2	 V 	   124.08	   567.85	  9
  3	 Y 	   205.61	   518.32	  8
  4	 H 	   274.14	   436.79	  7
  5	 L 	   330.68	   368.26	  6
  6	 S 	   374.19	   311.71	  5
  7	 D 	   431.71	   268.20	  4
  8	 L 	   488.25	   210.68	  3
  9	 C 	   568.33	   154.14	  2
 10	 K 	        -  	    74.06	  1  _
```

**103. IPI00411356 Vacuolar protein sorting-associated protein 4A**

| Sequence | Charge | M+H+ | XCorr | DeltaCn |
| --- | --- | --- | --- | --- |
| R.SLATTRPTVNADDLLK.V | 2 | 1715.93 | 2.919 | 0.247 |

+1 charge

```
		      B   	     Y
  1	 S 	    88.04	        -  	 16
  2	 L 	   201.12	  1627.90	 15
  3	 A 	   272.16	  1514.82	 14
  4	 T 	   373.21	  1443.78	 13
  5	 T 	   474.26	  1342.73	 12
  6	 R 	   630.36	  1241.68	 11
  7	 P 	   727.41	  1085.58	 10
  8	 T 	   828.46	   988.53	  9
  9	 V 	   927.53	   887.48	  8
 10	 N 	  1041.57	   788.41	  7
 11	 A 	  1112.61	   674.37	  6
 12	 D 	  1227.63	   603.33	  5
 13	 D 	  1342.66	   488.31	  4
 14	 L 	  1455.74	   373.28	  3
 15	 L 	  1568.83	   260.20	  2
 16	 K 	        -  	   147.11	  1  _
+2 charge
		      B   	     Y
  1	 S 	    44.52	        -  	 16
  2	 L 	   101.07	   814.45	 15
  3	 A 	   136.58	   757.91	 14
  4	 T 	   187.11	   722.39	 13
  5	 T 	   237.63	   671.87	 12
  6	 R 	   315.68	   621.35	 11
  7	 P 	   364.21	   543.30	 10
  8	 T 	   414.73	   494.77	  9
  9	 V 	   464.27	   444.25	  8
 10	 N 	   521.29	   394.71	  7
 11	 A 	   556.81	   337.69	  6
 12	 D 	   614.32	   302.17	  5
 13	 D 	   671.83	   244.66	  4
 14	 L 	   728.38	   187.14	  3
 15	 L 	   784.92	   130.60	  2
 16	 K 	        -  	    74.06	  1  _
```

**104. IPI00001952 Endonuclease domain-containing 1 protein**

| Sequence | Charge | M+H+ | XCorr | DeltaCn |
| --- | --- | --- | --- | --- |
| K.ILEVVNQIQDEER.M | 2 | 1585.741 | 3.75 | 0.494 |

+1 charge

```
		      B   	     Y
  1	 I 	   114.09	        -  	 13
  2	 L 	   227.18	  1471.74	 12
  3	 E 	   356.22	  1358.65	 11
  4	 V 	   455.29	  1229.61	 10
  5	 V 	   554.35	  1130.54	  9
  6	 N 	   668.40	  1031.48	  8
  7	 Q 	   796.46	   917.43	  7
  8	 I 	   909.54	   789.37	  6
  9	 Q 	  1037.60	   676.29	  5
 10	 D 	  1152.63	   548.23	  4
 11	 E 	  1281.67	   433.20	  3
 12	 E 	  1410.71	   304.16	  2
 13	 R 	        -  	   175.12	  1  _
+2 charge
		      B   	     Y
  1	 I 	    57.55	        -  	 13
  2	 L 	   114.09	   736.37	 12
  3	 E 	   178.61	   679.83	 11
  4	 V 	   228.15	   615.31	 10
  5	 V 	   277.68	   565.78	  9
  6	 N 	   334.70	   516.24	  8
  7	 Q 	   398.73	   459.22	  7
  8	 I 	   455.27	   395.19	  6
  9	 Q 	   519.30	   338.65	  5
 10	 D 	   576.82	   274.62	  4
 11	 E 	   641.34	   217.11	  3
 12	 E 	   705.86	   152.58	  2
 13	 R 	        -  	    88.06	  1  _
```

**105. IPI00011694 Trypsin-1**

| Sequence | Charge | M+H+ | XCorr | DeltaCn |
| --- | --- | --- | --- | --- |
| K.TLNNDIMLIK.L | 2 | 1175.424 | 3.731 | 0.238 |

+1 charge

```
		      B   	     Y
  1	 T 	   102.05	        -  	 10
  2	 L 	   215.14	  1073.60	  9
  3	 N 	   329.18	   960.52	  8
  4	 N 	   443.22	   846.48	  7
  5	 D 	   558.25	   732.43	  6
  6	 I 	   671.34	   617.41	  5
  7	 M 	   802.38	   504.32	  4
  8	 L 	   915.46	   373.28	  3
  9	 I 	  1028.54	   260.20	  2
 10	 K 	        -  	   147.11	  1  _
+2 charge
		      B   	     Y
  1	 T 	    51.53	        -  	 10
  2	 L 	   108.07	   537.30	  9
  3	 N 	   165.09	   480.76	  8
  4	 N 	   222.12	   423.74	  7
  5	 D 	   279.63	   366.72	  6
  6	 I 	   336.17	   309.21	  5
  7	 M 	   401.69	   252.66	  4
  8	 L 	   458.23	   187.14	  3
  9	 I 	   514.78	   130.60	  2
 10	 K 	        -  	    74.06	  1  _
```

**106. IPI00299738 Procollagen C-endopeptidase enhancer 1**

| Sequence | Charge | M+H+ | XCorr | DeltaCn |
| --- | --- | --- | --- | --- |
| R.FCGTFRPAPLVAPGNQVTLR.M | 3 | 2202.538 | 3.752 | 0.416 |

+1 charge

```
		      B   	     Y
  1	 F 	   148.08	        -  	 20
  2	 C 	   308.24	  2054.23	 19
  3	 G 	   365.26	  1894.07	 18
  4	 T 	   466.31	  1837.04	 17
  5	 F 	   613.38	  1736.00	 16
  6	 R 	   769.48	  1588.93	 15
  7	 P 	   866.53	  1432.83	 14
  8	 A 	   937.57	  1335.77	 13
  9	 P 	  1034.62	  1264.74	 12
 10	 L 	  1147.71	  1167.68	 11
 11	 V 	  1246.77	  1054.60	 10
 12	 A 	  1317.81	   955.53	  9
 13	 P 	  1414.86	   884.49	  8
 14	 G 	  1471.89	   787.44	  7
 15	 N 	  1585.93	   730.42	  6
 16	 Q 	  1713.99	   616.38	  5
 17	 V 	  1813.06	   488.32	  4
 18	 T 	  1914.10	   389.25	  3
 19	 L 	  2027.19	   288.20	  2
 20	 R 	        -  	   175.12	  1  _
+2 charge
		      B   	     Y
  1	 F 	    74.54	        -  	 20
  2	 C 	   154.62	  1027.62	 19
  3	 G 	   183.13	   947.54	 18
  4	 T 	   233.66	   919.03	 17
  5	 F 	   307.19	   868.50	 16
  6	 R 	   385.24	   794.97	 15
  7	 P 	   433.77	   716.92	 14
  8	 A 	   469.29	   668.39	 13
  9	 P 	   517.81	   632.87	 12
 10	 L 	   574.36	   584.35	 11
 11	 V 	   623.89	   527.80	 10
 12	 A 	   659.41	   478.27	  9
 13	 P 	   707.94	   442.75	  8
 14	 G 	   736.45	   394.22	  7
 15	 N 	   793.47	   365.71	  6
 16	 Q 	   857.50	   308.69	  5
 17	 V 	   907.03	   244.66	  4
 18	 T 	   957.56	   195.13	  3
 19	 L 	  1014.10	   144.61	  2
 20	 R 	        -  	    88.06	  1  _

\+3 charge
		      B   	     Y
  1	 F 	    50.03	        -  	 20
  2	 C 	   103.42	   685.42	 19
  3	 G 	   122.43	   632.03	 18
  4	 T 	   156.11	   613.02	 17
  5	 F 	   205.13	   579.34	 16
  6	 R 	   257.16	   530.31	 15
  7	 P 	   289.52	   478.28	 14
  8	 A 	   313.19	   445.93	 13
  9	 P 	   345.55	   422.25	 12
 10	 L 	   383.24	   389.90	 11
 11	 V 	   416.26	   352.20	 10
 12	 A 	   439.94	   319.18	  9
 13	 P 	   472.29	   295.50	  8
 14	 G 	   491.30	   263.15	  7
 15	 N 	   529.31	   244.15	  6
 16	 Q 	   572.00	   206.13	  5
 17	 V 	   605.02	   163.44	  4
 18	 T 	   638.71	   130.42	  3
 19	 L 	   676.40	    96.74	  2
 20	 R 	        -  	    59.04	  1  _
```

**107. IPI00024046 cDNA FLJ52398, highly similar to Cadherin-13**

| Sequence | Charge | M+H+ | XCorr | DeltaCn |
| --- | --- | --- | --- | --- |
| R.MTAFDADDPATDNALLR.Y | 2 | 1837.989 | 4.491 | 0.554 |

+1 charge

```
		      B   	     Y
  1	 M 	   132.05	        -  	 17
  2	 T 	   233.10	  1705.80	 16
  3	 A 	   304.13	  1604.76	 15
  4	 F 	   451.20	  1533.72	 14
  5	 D 	   566.23	  1386.65	 13
  6	 A 	   637.27	  1271.62	 12
  7	 D 	   752.29	  1200.59	 11
  8	 D 	   867.32	  1085.56	 10
  9	 P 	   964.37	   970.53	  9
 10	 A 	  1035.41	   873.48	  8
 11	 T 	  1136.46	   802.44	  7
 12	 D 	  1251.48	   701.39	  6
 13	 N 	  1365.53	   586.37	  5
 14	 A 	  1436.56	   472.32	  4
 15	 L 	  1549.65	   401.29	  3
 16	 L 	  1662.73	   288.20	  2
 17	 R 	        -  	   175.12	  1  _
+2 charge
		      B   	     Y
  1	 M 	    66.53	        -  	 17
  2	 T 	   117.05	   853.41	 16
  3	 A 	   152.57	   802.88	 15
  4	 F 	   226.10	   767.36	 14
  5	 D 	   283.62	   693.83	 13
  6	 A 	   319.14	   636.31	 12
  7	 D 	   376.65	   600.80	 11
  8	 D 	   434.16	   543.28	 10
  9	 P 	   482.69	   485.77	  9
 10	 A 	   518.21	   437.24	  8
 11	 T 	   568.73	   401.72	  7
 12	 D 	   626.25	   351.20	  6
 13	 N 	   683.27	   293.69	  5
 14	 A 	   718.79	   236.67	  4
 15	 L 	   775.33	   201.15	  3
 16	 L 	   831.87	   144.61	  2
 17	 R 	        -  	    88.06	  1  _
```

**108. IPI00031121 Carboxypeptidase E precursor**

| Sequence | Charge | M+H+ | XCorr | DeltaCn |
| --- | --- | --- | --- | --- |
| K.AASQPGELKDWFVGR.S | 2 | 1661.842 | 2.88 | 0.41 |

+1 charge

```
		      B   	     Y
  1	 A 	    72.04	        -  	 15
  2	 A 	   143.08	  1589.81	 14
  3	 S 	   230.11	  1518.77	 13
  4	 Q 	   358.17	  1431.74	 12
  5	 P 	   455.22	  1303.68	 11
  6	 G 	   512.25	  1206.63	 10
  7	 E 	   641.29	  1149.61	  9
  8	 L 	   754.37	  1020.56	  8
  9	 K 	   882.47	   907.48	  7
 10	 D 	   997.49	   779.38	  6
 11	 W 	  1183.57	   664.36	  5
 12	 F 	  1330.64	   478.28	  4
 13	 V 	  1429.71	   331.21	  3
 14	 G 	  1486.73	   232.14	  2
 15	 R 	        -  	   175.12	  1  _
+2 charge
		      B   	     Y
  1	 A 	    36.53	        -  	 15
  2	 A 	    72.04	   795.41	 14
  3	 S 	   115.56	   759.89	 13
  4	 Q 	   179.59	   716.37	 12
  5	 P 	   228.12	   652.34	 11
  6	 G 	   256.63	   603.82	 10
  7	 E 	   321.15	   575.31	  9
  8	 L 	   377.69	   510.78	  8
  9	 K 	   441.74	   454.24	  7
 10	 D 	   499.25	   390.20	  6
 11	 W 	   592.29	   332.68	  5
 12	 F 	   665.82	   239.64	  4
 13	 V 	   715.36	   166.11	  3
 14	 G 	   743.87	   116.57	  2
 15	 R 	        -  	    88.06	  1  _
```

**109. IPI00739464 similar to actin, gamma 1**

| Sequence | Charge | M+H+ | XCorr | DeltaCn |
| --- | --- | --- | --- | --- |
| K.SYKLPDGQVITIGNER.F | 2 | 1790.998 | 2.809 | 0.428 |

**+1 charge**

```
		      B   	     Y
  1	 S 	    88.04	        -  	 16
  2	 Y 	   251.10	  1702.91	 15
  3	 K 	   379.20	  1539.85	 14
  4	 L 	   492.28	  1411.75	 13
  5	 P 	   589.33	  1298.67	 12
  6	 D 	   704.36	  1201.62	 11
  7	 G 	   761.38	  1086.59	 10
  8	 Q 	   889.44	  1029.57	  9
  9	 V 	   988.51	   901.51	  8
 10	 I 	  1101.59	   802.44	  7
 11	 T 	  1202.64	   689.36	  6
 12	 I 	  1315.73	   588.31	  5
 13	 G 	  1372.75	   475.23	  4
 14	 N 	  1486.79	   418.20	  3
 15	 E 	  1615.83	   304.16	  2
 16	 R 	        -  	   175.12	  1  _
+2 charge
		      B   	     Y
  1	 S 	    44.52	        -  	 16
  2	 Y 	   126.05	   851.96	 15
  3	 K 	   190.10	   770.43	 14
  4	 L 	   246.64	   706.38	 13
  5	 P 	   295.17	   649.84	 12
  6	 D 	   352.68	   601.31	 11
  7	 G 	   381.20	   543.80	 10
  8	 Q 	   445.22	   515.29	  9
  9	 V 	   494.76	   451.26	  8
 10	 I 	   551.30	   401.72	  7
 11	 T 	   601.82	   345.18	  6
 12	 I 	   658.37	   294.66	  5
 13	 G 	   686.88	   238.12	  4
 14	 N 	   743.90	   209.61	  3
 15	 E 	   808.42	   152.58	  2
 16	 R 	        -  	    88.06	  1  _
```

**110. IPI00018136 Isoform 1 of Vascular cell adhesion protein 1**

| Sequence | Charge | M+H+ | XCorr | DeltaCn |
| --- | --- | --- | --- | --- |
| K.SQEFLEDADRKSLETK.S | 2 | 1897.033 | 3.507 | 0.216 |

+1 charge

```
		      B   	     Y
  1	 S 	    88.04	        -  	 16
  2	 Q 	   216.10	  1808.90	 15
  3	 E 	   345.14	  1680.84	 14
  4	 F 	   492.21	  1551.80	 13
  5	 L 	   605.29	  1404.73	 12
  6	 E 	   734.34	  1291.65	 11
  7	 D 	   849.36	  1162.61	 10
  8	 A 	   920.40	  1047.58	  9
  9	 D 	  1035.43	   976.54	  8
 10	 R 	  1191.53	   861.52	  7
 11	 K 	  1319.62	   705.41	  6
 12	 S 	  1406.65	   577.32	  5
 13	 L 	  1519.74	   490.29	  4
 14	 E 	  1648.78	   377.20	  3
 15	 T 	  1749.83	   248.16	  2
 16	 K 	        -  	   147.11	  1  _
+2 charge
		      B   	     Y
  1	 S 	    44.52	        -  	 16
  2	 Q 	   108.55	   904.95	 15
  3	 E 	   173.07	   840.93	 14
  4	 F 	   246.61	   776.40	 13
  5	 L 	   303.15	   702.87	 12
  6	 E 	   367.67	   646.33	 11
  7	 D 	   425.18	   581.81	 10
  8	 A 	   460.70	   524.29	  9
  9	 D 	   518.22	   488.77	  8
 10	 R 	   596.27	   431.26	  7
 11	 K 	   660.31	   353.21	  6
 12	 S 	   703.83	   289.16	  5
 13	 L 	   760.37	   245.65	  4
 14	 E 	   824.89	   189.11	  3
 15	 T 	   875.42	   124.58	  2
 16	 K 	        -  	    74.06	  1  _
```

**111. IPI00329801 Annexin A5**

| Sequence | Charge | M+H+ | XCorr | DeltaCn |
| --- | --- | --- | --- | --- |
| K.GLGTDEESILTLLTSR.S | 2 | 1705.889 | 3.563 | 0.424 |

+1 charge

```
		      B   	     Y
  1	 G 	    58.03	        -  	 16
  2	 L 	   171.11	  1647.88	 15
  3	 G 	   228.13	  1534.80	 14
  4	 T 	   329.18	  1477.77	 13
  5	 D 	   444.21	  1376.73	 12
  6	 E 	   573.25	  1261.70	 11
  7	 E 	   702.29	  1132.66	 10
  8	 S 	   789.33	  1003.61	  9
  9	 I 	   902.41	   916.58	  8
 10	 L 	  1015.49	   803.50	  7
 11	 T 	  1116.54	   690.41	  6
 12	 L 	  1229.63	   589.37	  5
 13	 L 	  1342.71	   476.28	  4
 14	 T 	  1443.76	   363.20	  3
 15	 S 	  1530.79	   262.15	  2
 16	 R 	        -  	   175.12	  1  _
+2 charge
		      B   	     Y
  1	 G 	    29.52	        -  	 16
  2	 L 	    86.06	   824.44	 15
  3	 G 	   114.57	   767.90	 14
  4	 T 	   165.09	   739.39	 13
  5	 D 	   222.61	   688.87	 12
  6	 E 	   287.13	   631.35	 11
  7	 E 	   351.65	   566.83	 10
  8	 S 	   395.17	   502.31	  9
  9	 I 	   451.71	   458.79	  8
 10	 L 	   508.25	   402.25	  7
 11	 T 	   558.77	   345.71	  6
 12	 L 	   615.32	   295.19	  5
 13	 L 	   671.86	   238.64	  4
 14	 T 	   722.38	   182.10	  3
 15	 S 	   765.90	   131.58	  2
 16	 R 	        -  	    88.06	  1  _
```

**112. IPI00015902 Beta-type platelet-derived growth factor receptor**

| Sequence | Charge | M+H+ | XCorr | DeltaCn |
| --- | --- | --- | --- | --- |
| R.LVEPVTDFLLDMPYHIR.S | 2 | 2059.417 | 3.927 | 0.508 |

+1 charge

```
		      B   	     Y
  1	 L 	   114.09	        -  	 17
  2	 V 	   213.16	  1944.99	 16
  3	 E 	   342.20	  1845.92	 15
  4	 P 	   439.26	  1716.88	 14
  5	 V 	   538.32	  1619.83	 13
  6	 T 	   639.37	  1520.76	 12
  7	 D 	   754.40	  1419.71	 11
  8	 F 	   901.47	  1304.68	 10
  9	 L 	  1014.55	  1157.61	  9
 10	 L 	  1127.63	  1044.53	  8
 11	 D 	  1242.66	   931.45	  7
 12	 M 	  1373.70	   816.42	  6
 13	 P 	  1470.75	   685.38	  5
 14	 Y 	  1633.82	   588.33	  4
 15	 H 	  1770.88	   425.26	  3
 16	 I 	  1883.96	   288.20	  2
 17	 R 	        -  	   175.12	  1  _
+2 charge
		      B   	     Y
  1	 L 	    57.55	        -  	 17
  2	 V 	   107.08	   973.00	 16
  3	 E 	   171.60	   923.46	 15
  4	 P 	   220.13	   858.94	 14
  5	 V 	   269.67	   810.42	 13
  6	 T 	   320.19	   760.88	 12
  7	 D 	   377.70	   710.36	 11
  8	 F 	   451.24	   652.84	 10
  9	 L 	   507.78	   579.31	  9
 10	 L 	   564.32	   522.77	  8
 11	 D 	   621.83	   466.23	  7
 12	 M 	   687.35	   408.71	  6
 13	 P 	   735.88	   343.19	  5
 14	 Y 	   817.41	   294.67	  4
 15	 H 	   885.94	   213.13	  3
 16	 I 	   942.48	   144.61	  2
 17	 R 	        -  	    88.06	  1  _
```

**113. IPI00020546 Probable histone-lysine N-methyltransferase ASH1L**

| Sequence | Charge | M+H+ | XCorr | DeltaCn |
| --- | --- | --- | --- | --- |
| R.SELFKNVSCSSLSNSNSEPAKFMK.N | 3 | 2692.977 | 3.745 | 0.306 |

+1 charge

```
		      B   	     Y
  1	 S 	    88.04	        -  	 24
  2	 E 	   217.08	  2604.38	 23
  3	 L 	   330.17	  2475.34	 22
  4	 F 	   477.23	  2362.25	 21
  5	 K 	   605.33	  2215.18	 20
  6	 N 	   719.37	  2087.09	 19
  7	 V 	   818.44	  1973.04	 18
  8	 S 	   905.47	  1873.98	 17
  9	 C 	  1065.64	  1786.94	 16
 10	 S 	  1152.67	  1626.78	 15
 11	 S 	  1239.70	  1539.75	 14
 12	 L 	  1352.79	  1452.72	 13
 13	 S 	  1439.82	  1339.63	 12
 14	 N 	  1553.86	  1252.60	 11
 15	 S 	  1640.89	  1138.56	 10
 16	 N 	  1754.94	  1051.52	  9
 17	 S 	  1841.97	   937.48	  8
 18	 E 	  1971.01	   850.45	  7
 19	 P 	  2068.06	   721.41	  6
 20	 A 	  2139.10	   624.35	  5
 21	 K 	  2267.20	   553.32	  4
 22	 F 	  2414.26	   425.22	  3
 23	 M 	  2545.30	   278.15	  2
 24	 K 	        -  	   147.11	  1  _


+2 charge
		      B   	     Y
  1	 S 	    44.52	        -  	 24
  2	 E 	   109.04	  1302.69	 23
  3	 L 	   165.59	  1238.17	 22
  4	 F 	   239.12	  1181.63	 21
  5	 K 	   303.17	  1108.10	 20
  6	 N 	   360.19	  1044.05	 19
  7	 V 	   409.72	   987.03	 18
  8	 S 	   453.24	   937.49	 17
  9	 C 	   533.32	   893.98	 16
 10	 S 	   576.84	   813.89	 15
 11	 S 	   620.35	   770.38	 14
 12	 L 	   676.90	   726.86	 13
 13	 S 	   720.41	   670.32	 12
 14	 N 	   777.43	   626.80	 11
 15	 S 	   820.95	   569.78	 10
 16	 N 	   877.97	   526.27	  9
 17	 S 	   921.49	   469.24	  8
 18	 E 	   986.01	   425.73	  7
 19	 P 	  1034.54	   361.21	  6
 20	 A 	  1070.05	   312.68	  5
 21	 K 	  1134.10	   277.16	  4
 22	 F 	  1207.64	   213.11	  3
 23	 M 	  1273.16	   139.58	  2
 24	 K 	        -  	    74.06	  1  _


+3 charge
		      B   	     Y
  1	 S 	    30.02	        -  	 24
  2	 E 	    73.03	   868.80	 23
  3	 L 	   110.73	   825.78	 22
  4	 F 	   159.75	   788.09	 21
  5	 K 	   202.45	   739.07	 20
  6	 N 	   240.46	   696.37	 19
  7	 V 	   273.49	   658.35	 18
  8	 S 	   302.50	   625.33	 17
  9	 C 	   355.88	   596.32	 16
 10	 S 	   384.89	   542.93	 15
 11	 S 	   413.91	   513.92	 14
 12	 L 	   451.60	   484.91	 13
 13	 S 	   480.61	   447.22	 12
 14	 N 	   518.63	   418.20	 11
 15	 S 	   547.64	   380.19	 10
 16	 N 	   585.65	   351.18	  9
 17	 S 	   614.66	   313.17	  8
 18	 E 	   657.68	   284.15	  7
 19	 P 	   690.03	   241.14	  6
 20	 A 	   713.71	   208.79	  5
 21	 K 	   756.40	   185.11	  4
 22	 F 	   805.43	   142.41	  3
 23	 M 	   849.11	    93.39	  2
 24	 K 	        -  	    49.71	  1  _
```

**114. IPI00100933 Isoform 1 of Phosphotriesterase-related protein**

| Sequence | Charge | M+H+ | XCorr | DeltaCn |
| --- | --- | --- | --- | --- |
| R.GITENVLDKILIENPKQWLTFK.- | 3 | 2601.037 | 3.623 | 0.441 |

+1 charge

```
		      B   	     Y
  1	 G 	    58.03	        -  	 22
  2	 I 	   171.11	  2542.43	 21
  3	 T 	   272.16	  2429.34	 20
  4	 E 	   401.20	  2328.30	 19
  5	 N 	   515.25	  2199.25	 18
  6	 V 	   614.31	  2085.21	 17
  7	 L 	   727.40	  1986.14	 16
  8	 D 	   842.43	  1873.06	 15
  9	 K 	   970.52	  1758.03	 14
 10	 I 	  1083.60	  1629.94	 13
 11	 L 	  1196.69	  1516.85	 12
 12	 I 	  1309.77	  1403.77	 11
 13	 E 	  1438.82	  1290.68	 10
 14	 N 	  1552.86	  1161.64	  9
 15	 P 	  1649.91	  1047.60	  8
 16	 K 	  1778.01	   950.55	  7
 17	 Q 	  1906.06	   822.45	  6
 18	 W 	  2092.14	   694.39	  5
 19	 L 	  2205.23	   508.31	  4
 20	 T 	  2306.28	   395.23	  3
 21	 F 	  2453.34	   294.18	  2
 22	 K 	        -  	   147.11	  1  _


+2 charge
		      B   	     Y
  1	 G 	    29.52	        -  	 22
  2	 I 	    86.06	  1271.72	 21
  3	 T 	   136.58	  1215.18	 20
  4	 E 	   201.11	  1164.65	 19
  5	 N 	   258.13	  1100.13	 18
  6	 V 	   307.66	  1043.11	 17
  7	 L 	   364.20	   993.57	 16
  8	 D 	   421.72	   937.03	 15
  9	 K 	   485.76	   879.52	 14
 10	 I 	   542.31	   815.47	 13
 11	 L 	   598.85	   758.93	 12
 12	 I 	   655.39	   702.39	 11
 13	 E 	   719.91	   645.85	 10
 14	 N 	   776.93	   581.32	  9
 15	 P 	   825.46	   524.30	  8
 16	 K 	   889.51	   475.78	  7
 17	 Q 	   953.54	   411.73	  6
 18	 W 	  1046.58	   347.70	  5
 19	 L 	  1103.12	   254.66	  4
 20	 T 	  1153.64	   198.12	  3
 21	 F 	  1227.18	   147.59	  2
 22	 K 	        -  	    74.06	  1  _
+3 charge
		      B   	     Y
  1	 G 	    20.01	        -  	 22
  2	 I 	    57.71	   848.15	 21
  3	 T 	    91.39	   810.45	 20
  4	 E 	   134.41	   776.77	 19
  5	 N 	   172.42	   733.76	 18
  6	 V 	   205.44	   695.74	 17
  7	 L 	   243.14	   662.72	 16
  8	 D 	   281.48	   625.02	 15
  9	 K 	   324.18	   586.68	 14
 10	 I 	   361.87	   543.98	 13
 11	 L 	   399.57	   506.29	 12
 12	 I 	   437.26	   468.59	 11
 13	 E 	   480.28	   430.90	 10
 14	 N 	   518.29	   387.89	  9
 15	 P 	   550.64	   349.87	  8
 16	 K 	   593.34	   317.52	  7
 17	 Q 	   636.03	   274.82	  6
 18	 W 	   698.05	   232.14	  5
 19	 L 	   735.75	   170.11	  4
 20	 T 	   769.43	   132.41	  3
 21	 F 	   818.45	    98.73	  2
 22	 K 	        -  	    49.71	  1  _
```

**115. IPI00247063 Neprilysin**

| Sequence | Charge | M+H+ | XCorr | DeltaCn |
| --- | --- | --- | --- | --- |
| R.YGNFDILRDELEVVLKDVLQEPK.T | 3 | 2734.097 | 4.707 | 0.274 |

+1 charge

```
		      B   	     Y
  1	 Y 	   164.07	        -  	 23
  2	 G 	   221.09	  2569.39	 22
  3	 N 	   335.13	  2512.37	 21
  4	 F 	   482.20	  2398.32	 20
  5	 D 	   597.23	  2251.25	 19
  6	 I 	   710.31	  2136.23	 18
  7	 L 	   823.40	  2023.14	 17
  8	 R 	   979.50	  1910.06	 16
  9	 D 	  1094.53	  1753.96	 15
 10	 E 	  1223.57	  1638.93	 14
 11	 L 	  1336.65	  1509.89	 13
 12	 E 	  1465.70	  1396.80	 12
 13	 V 	  1564.76	  1267.76	 11
 14	 V 	  1663.83	  1168.69	 10
 15	 L 	  1776.92	  1069.63	  9
 16	 K 	  1905.01	   956.54	  8
 17	 D 	  2020.04	   828.45	  7
 18	 V 	  2119.11	   713.42	  6
 19	 L 	  2232.19	   614.35	  5
 20	 Q 	  2360.25	   501.27	  4
 21	 E 	  2489.29	   373.21	  3
 22	 P 	  2586.34	   244.17	  2
 23	 K 	        -  	   147.11	  1  _


+2 charge
		      B   	     Y
  1	 Y 	    82.54	        -  	 23
  2	 G 	   111.05	  1285.20	 22
  3	 N 	   168.07	  1256.69	 21
  4	 F 	   241.61	  1199.67	 20
  5	 D 	   299.12	  1126.13	 19
  6	 I 	   355.66	  1068.62	 18
  7	 L 	   412.20	  1012.08	 17
  8	 R 	   490.25	   955.53	 16
  9	 D 	   547.77	   877.48	 15
 10	 E 	   612.29	   819.97	 14
 11	 L 	   668.83	   755.45	 13
 12	 E 	   733.35	   698.91	 12
 13	 V 	   782.89	   634.38	 11
 14	 V 	   832.42	   584.85	 10
 15	 L 	   888.96	   535.32	  9
 16	 K 	   953.01	   478.77	  8
 17	 D 	  1010.52	   414.73	  7
 18	 V 	  1060.06	   357.21	  6
 19	 L 	  1116.60	   307.68	  5
 20	 Q 	  1180.63	   251.14	  4
 21	 E 	  1245.15	   187.11	  3
 22	 P 	  1293.68	   122.59	  2
 23	 K 	        -  	    74.06	  1  _
+3 charge
		      B   	     Y
  1	 Y 	    55.36	        -  	 23
  2	 G 	    74.37	   857.13	 22
  3	 N 	   112.38	   838.13	 21
  4	 F 	   161.41	   800.11	 20
  5	 D 	   199.75	   751.09	 19
  6	 I 	   237.44	   712.75	 18
  7	 L 	   275.14	   675.05	 17
  8	 R 	   327.17	   637.36	 16
  9	 D 	   365.51	   585.32	 15
 10	 E 	   408.53	   546.98	 14
 11	 L 	   446.22	   503.97	 13
 12	 E 	   489.24	   466.27	 12
 13	 V 	   522.26	   423.26	 11
 14	 V 	   555.28	   390.24	 10
 15	 L 	   592.98	   357.21	  9
 16	 K 	   635.68	   319.52	  8
 17	 D 	   674.02	   276.82	  7
 18	 V 	   707.04	   238.48	  6
 19	 L 	   744.74	   205.46	  5
 20	 Q 	   787.42	   167.76	  4
 21	 E 	   830.44	   125.07	  3
 22	 P 	   862.79	    82.06	  2
 23	 K 	        -  	    49.71	  1  _
```

**116. IPI00029235 Insulin-like growth factor-binding protein 6**

| Sequence | Charge | M+H+ | XCorr | DeltaCn |
| --- | --- | --- | --- | --- |
| R.GAQTLYVPNCDHR.G | 2 | 1531.649 | 3.133 | 0.47 |

+1 charge

```
		      B   	     Y
  1	 G 	    58.03	        -  	 13
  2	 A 	   129.07	  1473.82	 12
  3	 Q 	   257.12	  1402.79	 11
  4	 T 	   358.17	  1274.73	 10
  5	 L 	   471.26	  1173.68	  9
  6	 Y 	   634.32	  1060.60	  8
  7	 V 	   733.39	   897.53	  7
  8	 P 	   830.44	   798.47	  6
  9	 N 	   944.48	   701.41	  5
 10	 C 	  1104.65	   587.37	  4
 11	 D 	  1219.68	   427.20	  3
 12	 H 	  1356.73	   312.18	  2
 13	 R 	        -  	   175.12	  1  _
+2 charge
		      B   	     Y
  1	 G 	    29.52	        -  	 13
  2	 A 	    65.04	   737.42	 12
  3	 Q 	   129.07	   701.90	 11
  4	 T 	   179.59	   637.87	 10
  5	 L 	   236.13	   587.34	  9
  6	 Y 	   317.66	   530.80	  8
  7	 V 	   367.20	   449.27	  7
  8	 P 	   415.72	   399.74	  6
  9	 N 	   472.75	   351.21	  5
 10	 C 	   552.83	   294.19	  4
 11	 D 	   610.34	   214.11	  3
 12	 H 	   678.87	   156.59	  2
 13	 R 	        -  	    88.06	  1  _
```

**117. IPI00005563 Isoform 1 of Tubulointerstitial nephritis antigen-like**

| Sequence | Charge | M+H+ | XCorr | DeltaCn |
| --- | --- | --- | --- | --- |
| K.ITGWGEETLPDGR.T | 2 | 1431.532 | 2.825 | 0.306 |

+1 charge

```
		      B   	     Y
  1	 I 	   114.09	        -  	 13
  2	 T 	   215.14	  1317.61	 12
  3	 G 	   272.16	  1216.56	 11
  4	 W 	   458.24	  1159.54	 10
  5	 G 	   515.26	   973.46	  9
  6	 E 	   644.30	   916.44	  8
  7	 E 	   773.35	   787.39	  7
  8	 T 	   874.39	   658.35	  6
  9	 L 	   987.48	   557.30	  5
 10	 P 	  1084.53	   444.22	  4
 11	 D 	  1199.56	   347.17	  3
 12	 G 	  1256.58	   232.14	  2
 13	 R 	        -  	   175.12	  1  _
+2 charge
		      B   	     Y
  1	 I 	    57.55	        -  	 13
  2	 T 	   108.07	   659.31	 12
  3	 G 	   136.58	   608.78	 11
  4	 W 	   229.62	   580.27	 10
  5	 G 	   258.13	   487.23	  9
  6	 E 	   322.66	   458.72	  8
  7	 E 	   387.18	   394.20	  7
  8	 T 	   437.70	   329.68	  6
  9	 L 	   494.24	   279.16	  5
 10	 P 	   542.77	   222.61	  4
 11	 D 	   600.28	   174.09	  3
 12	 G 	   628.79	   116.57	  2
 13	 R 	        -  	    88.06	  1  _
```

**118. IPI00011692 Involucrin**

| Sequence | Charge | M+H+ | XCorr | DeltaCn |
| --- | --- | --- | --- | --- |
| K.HLEQQEGQLEQLEEQEGQLK.H | 3 | 2394.537 | 3.322 | 0.325 |

+1 charge

```
		      B   	     Y
  1	 H 	   138.07	        -  	 20
  2	 L 	   251.15	  2256.10	 19
  3	 E 	   380.19	  2143.01	 18
  4	 Q 	   508.25	  2013.97	 17
  5	 Q 	   636.31	  1885.91	 16
  6	 E 	   765.35	  1757.86	 15
  7	 G 	   822.37	  1628.81	 14
  8	 Q 	   950.43	  1571.79	 13
  9	 L 	  1063.52	  1443.73	 12
 10	 E 	  1192.56	  1330.65	 11
 11	 Q 	  1320.62	  1201.61	 10
 12	 L 	  1433.70	  1073.55	  9
 13	 E 	  1562.74	   960.46	  8
 14	 E 	  1691.79	   831.42	  7
 15	 Q 	  1819.85	   702.38	  6
 16	 E 	  1948.89	   574.32	  5
 17	 G 	  2005.91	   445.28	  4
 18	 Q 	  2133.97	   388.26	  3
 19	 L 	  2247.05	   260.20	  2
 20	 K 	        -  	   147.11	  1  _
+2 charge
		      B   	     Y
  1	 H 	    69.54	        -  	 20
  2	 L 	   126.08	  1128.55	 19
  3	 E 	   190.60	  1072.01	 18
  4	 Q 	   254.63	  1007.49	 17
  5	 Q 	   318.66	   943.46	 16
  6	 E 	   383.18	   879.43	 15
  7	 G 	   411.69	   814.91	 14
  8	 Q 	   475.72	   786.40	 13
  9	 L 	   532.26	   722.37	 12
 10	 E 	   596.78	   665.83	 11
 11	 Q 	   660.81	   601.31	 10
 12	 L 	   717.35	   537.28	  9
 13	 E 	   781.88	   480.74	  8
 14	 E 	   846.40	   416.21	  7
 15	 Q 	   910.43	   351.69	  6
 16	 E 	   974.95	   287.66	  5
 17	 G 	  1003.46	   223.14	  4
 18	 Q 	  1067.49	   194.63	  3
 19	 L 	  1124.03	   130.60	  2
 20	 K 	        -  	    74.06	  1  _

+3 charge
		      B   	     Y
  1	 H 	    46.69	        -  	 20
  2	 L 	    84.39	   752.70	 19
  3	 E 	   127.40	   715.01	 18
  4	 Q 	   170.09	   672.00	 17
  5	 Q 	   212.77	   629.31	 16
  6	 E 	   255.79	   586.62	 15
  7	 G 	   274.80	   543.61	 14
  8	 Q 	   317.48	   524.60	 13
  9	 L 	   355.18	   481.92	 12
 10	 E 	   398.19	   444.22	 11
 11	 Q 	   440.88	   401.21	 10
 12	 L 	   478.57	   358.52	  9
 13	 E 	   521.59	   320.83	  8
 14	 E 	   564.60	   277.81	  7
 15	 Q 	   607.29	   234.80	  6
 16	 E 	   650.30	   192.11	  5
 17	 G 	   669.31	   149.10	  4
 18	 Q 	   711.99	   130.09	  3
 19	 L 	   749.69	    87.40	  2
 20	 K 	        -  	    49.71	  1  _
```

**119. IPI00059476 Dipeptidase 1**

| Sequence | Charge | M+H+ | XCorr | DeltaCn |
| --- | --- | --- | --- | --- |
| R.AVGFGGDFDGVPR.V | 2 | 1294.397 | 3.117 | 0.528 |

+1 charge

```
		      B   	     Y
  1	 A 	    72.04	        -  	 13
  2	 V 	   171.11	  1222.59	 12
  3	 G 	   228.13	  1123.52	 11
  4	 F 	   375.20	  1066.50	 10
  5	 G 	   432.22	   919.43	  9
  6	 G 	   489.25	   862.41	  8
  7	 D 	   604.27	   805.38	  7
  8	 F 	   751.34	   690.36	  6
  9	 D 	   866.37	   543.29	  5
 10	 G 	   923.39	   428.26	  4
 11	 V 	  1022.46	   371.24	  3
 12	 P 	  1119.51	   272.17	  2
 13	 R 	        -  	   175.12	  1  _
+2 charge
		      B   	     Y
  1	 A 	    36.53	        -  	 13
  2	 V 	    86.06	   611.80	 12
  3	 G 	   114.57	   562.26	 11
  4	 F 	   188.10	   533.75	 10
  5	 G 	   216.62	   460.22	  9
  6	 G 	   245.13	   431.71	  8
  7	 D 	   302.64	   403.20	  7
  8	 F 	   376.17	   345.68	  6
  9	 D 	   433.69	   272.15	  5
 10	 G 	   462.20	   214.63	  4
 11	 V 	   511.73	   186.12	  3
 12	 P 	   560.26	   136.59	  2
 13	 R 	        -  	    88.06	  1  _
```

**120. IPI00009901 Nuclear transport factor 2**

| Sequence | Charge | M+H+ | XCorr | DeltaCn |
| --- | --- | --- | --- | --- |
| K.AAIVEKLSSLPFQK.I | 2 | 1531.821 | 3.553 | 0.397 |

+1 charge

```
		      B   	     Y
  1	 A 	    72.04	        -  	 14
  2	 A 	   143.08	  1459.85	 13
  3	 I 	   256.17	  1388.81	 12
  4	 V 	   355.23	  1275.73	 11
  5	 E 	   484.28	  1176.66	 10
  6	 K 	   612.37	  1047.62	  9
  7	 L 	   725.46	   919.52	  8
  8	 S 	   812.49	   806.44	  7
  9	 S 	   899.52	   719.41	  6
 10	 L 	  1012.60	   632.38	  5
 11	 P 	  1109.66	   519.29	  4
 12	 F 	  1256.72	   422.24	  3
 13	 Q 	  1384.78	   275.17	  2
 14	 K 	        -  	   147.11	  1  _
+2 charge
		      B   	     Y
  1	 A 	    36.53	        -  	 14
  2	 A 	    72.04	   730.43	 13
  3	 I 	   128.59	   694.91	 12
  4	 V 	   178.12	   638.37	 11
  5	 E 	   242.64	   588.83	 10
  6	 K 	   306.69	   524.31	  9
  7	 L 	   363.23	   460.27	  8
  8	 S 	   406.75	   403.72	  7
  9	 S 	   450.26	   360.21	  6
 10	 L 	   506.81	   316.69	  5
 11	 P 	   555.33	   260.15	  4
 12	 F 	   628.87	   211.62	  3
 13	 Q 	   692.90	   138.09	  2
 14	 K 	        -  	    74.06	  1  _
```

**121. IPI00418471 Vimentin**

| Sequence | Charge | M+H+ | XCorr | DeltaCn |
| --- | --- | --- | --- | --- |
| K.ILLAELEQLKGQGK.S | 2 | 1540.83 | 3.024 | 0.352 |

+1 charge

```
		      B   	     Y
  1	 I 	   114.09	        -  	 14
  2	 L 	   227.18	  1426.83	 13
  3	 L 	   340.26	  1313.74	 12
  4	 A 	   411.30	  1200.66	 11
  5	 E 	   540.34	  1129.62	 10
  6	 L 	   653.42	  1000.58	  9
  7	 E 	   782.47	   887.49	  8
  8	 Q 	   910.52	   758.45	  7
  9	 L 	  1023.61	   630.39	  6
 10	 K 	  1151.70	   517.31	  5
 11	 G 	  1208.72	   389.21	  4
 12	 Q 	  1336.78	   332.19	  3
 13	 G 	  1393.80	   204.13	  2
 14	 K 	        -  	   147.11	  1  _
+2 charge
		      B   	     Y
  1	 I 	    57.55	        -  	 14
  2	 L 	   114.09	   713.92	 13
  3	 L 	   170.63	   657.37	 12
  4	 A 	   206.15	   600.83	 11
  5	 E 	   270.67	   565.31	 10
  6	 L 	   327.22	   500.79	  9
  7	 E 	   391.74	   444.25	  8
  8	 Q 	   455.77	   379.73	  7
  9	 L 	   512.31	   315.70	  6
 10	 K 	   576.36	   259.16	  5
 11	 G 	   604.87	   195.11	  4
 12	 Q 	   668.90	   166.60	  3
 13	 G 	   697.41	   102.57	  2
 14	 K 	        -  	    74.06	  1  _
```

**122. IPI00220143 Maltase-glucoamylase, intestinal**

| Sequence | Charge | M+H+ | XCorr | DeltaCn |
| --- | --- | --- | --- | --- |
| R.EIEELYNNPQNPER.S | 2 | 1745.828 | 3.247 | 0.349 |

+1 charge

```
		      B   	     Y
  1	 E 	   130.05	        -  	 14
  2	 I 	   243.13	  1615.77	 13
  3	 E 	   372.18	  1502.69	 12
  4	 E 	   501.22	  1373.64	 11
  5	 L 	   614.30	  1244.60	 10
  6	 Y 	   777.37	  1131.52	  9
  7	 N 	   891.41	   968.45	  8
  8	 N 	  1005.45	   854.41	  7
  9	 P 	  1102.51	   740.37	  6
 10	 Q 	  1230.56	   643.32	  5
 11	 N 	  1344.61	   515.26	  4
 12	 P 	  1441.66	   401.21	  3
 13	 E 	  1570.70	   304.16	  2
 14	 R 	        -  	   175.12	  1  _
+2 charge
		      B   	     Y
  1	 E 	    65.53	        -  	 14
  2	 I 	   122.07	   808.39	 13
  3	 E 	   186.59	   751.85	 12
  4	 E 	   251.11	   687.33	 11
  5	 L 	   307.66	   622.80	 10
  6	 Y 	   389.19	   566.26	  9
  7	 N 	   446.21	   484.73	  8
  8	 N 	   503.23	   427.71	  7
  9	 P 	   551.76	   370.69	  6
 10	 Q 	   615.79	   322.16	  5
 11	 N 	   672.81	   258.13	  4
 12	 P 	   721.33	   201.11	  3
 13	 E 	   785.85	   152.58	  2
 14	 R 	        -  	    88.06	  1  _
```

**123. IPI00384401 Myosin-reactive immunoglobulin kappa chain variable region (Fragment)**

| Sequence | Charge | M+H+ | XCorr | DeltaCn |
| --- | --- | --- | --- | --- |
| - .EIVMTQSPATLSVSPGER.A | 2 | 1903.147 | 4.62 | 0.549 |

+1 charge

```
		      B   	     Y
  1	 E 	   130.05	        -  	 18
  2	 I 	   243.13	  1772.92	 17
  3	 V 	   342.20	  1659.84	 16
  4	 M 	   473.24	  1560.77	 15
  5	 T 	   574.29	  1429.73	 14
  6	 Q 	   702.35	  1328.68	 13
  7	 S 	   789.38	  1200.62	 12
  8	 P 	   886.43	  1113.59	 11
  9	 A 	   957.47	  1016.54	 10
 10	 T 	  1058.52	   945.50	  9
 11	 L 	  1171.60	   844.45	  8
 12	 S 	  1258.63	   731.37	  7
 13	 V 	  1357.70	   644.34	  6
 14	 S 	  1444.74	   545.27	  5
 15	 P 	  1541.79	   458.24	  4
 16	 G 	  1598.81	   361.18	  3
 17	 E 	  1727.85	   304.16	  2
 18	 R 	        -  	   175.12	  1  _
+2 charge
		      B   	     Y
  1	 E 	    65.53	        -  	 18
  2	 I 	   122.07	   886.96	 17
  3	 V 	   171.60	   830.42	 16
  4	 M 	   237.13	   780.89	 15
  5	 T 	   287.65	   715.37	 14
  6	 Q 	   351.68	   664.84	 13
  7	 S 	   395.19	   600.81	 12
  8	 P 	   443.72	   557.30	 11
  9	 A 	   479.24	   508.77	 10
 10	 T 	   529.76	   473.25	  9
 11	 L 	   586.31	   422.73	  8
 12	 S 	   629.82	   366.19	  7
 13	 V 	   679.36	   322.67	  6
 14	 S 	   722.87	   273.14	  5
 15	 P 	   771.40	   229.62	  4
 16	 G 	   799.91	   181.10	  3
 17	 E 	   864.43	   152.58	  2
 18	 R 	        -  	    88.06	  1  _
```

**124. IPI00216780 Cartilage intermediate layer protein 2 precursor**

| Sequence | Charge | M+H+ | XCorr | DeltaCn |
| --- | --- | --- | --- | --- |
| R.INLAKPRPGDPAEANGPVYPWR.S | 3 | 2419.726 | 4.649 | 0.423 |

+1 charge

```
		      B   	     Y
  1	 I 	   114.09	        -  	 22
  2	 N 	   228.13	  2305.18	 21
  3	 L 	   341.22	  2191.14	 20
  4	 A 	   412.26	  2078.06	 19
  5	 K 	   540.35	  2007.02	 18
  6	 P 	   637.40	  1878.92	 17
  7	 R 	   793.50	  1781.87	 16
  8	 P 	   890.56	  1625.77	 15
  9	 G 	   947.58	  1528.72	 14
 10	 D 	  1062.61	  1471.70	 13
 11	 P 	  1159.66	  1356.67	 12
 12	 A 	  1230.70	  1259.62	 11
 13	 E 	  1359.74	  1188.58	 10
 14	 A 	  1430.78	  1059.54	  9
 15	 N 	  1544.82	   988.50	  8
 16	 G 	  1601.84	   874.46	  7
 17	 P 	  1698.89	   817.44	  6
 18	 V 	  1797.96	   720.38	  5
 19	 Y 	  1961.02	   621.31	  4
 20	 P 	  2058.08	   458.25	  3
 21	 W 	  2244.16	   361.20	  2
 22	 R 	        -  	   175.12	  1  _


+2 charge
		      B   	     Y
  1	 I 	    57.55	        -  	 22
  2	 N 	   114.57	  1153.10	 21
  3	 L 	   171.11	  1096.07	 20
  4	 A 	   206.63	  1039.53	 19
  5	 K 	   270.68	  1004.01	 18
  6	 P 	   319.21	   939.97	 17
  7	 R 	   397.26	   891.44	 16
  8	 P 	   445.78	   813.39	 15
  9	 G 	   474.29	   764.86	 14
 10	 D 	   531.81	   736.35	 13
 11	 P 	   580.33	   678.84	 12
 12	 A 	   615.85	   630.31	 11
 13	 E 	   680.37	   594.79	 10
 14	 A 	   715.89	   530.27	  9
 15	 N 	   772.91	   494.75	  8
 16	 G 	   801.42	   437.73	  7
 17	 P 	   849.95	   409.22	  6
 18	 V 	   899.48	   360.70	  5
 19	 Y 	   981.02	   311.16	  4
 20	 P 	  1029.54	   229.63	  3
 21	 W 	  1122.58	   181.10	  2
 22	 R 	        -  	    88.06	  1  _
+3 charge
		      B   	     Y
  1	 I 	    38.70	        -  	 22
  2	 N 	    76.72	   769.07	 21
  3	 L 	   114.41	   731.05	 20
  4	 A 	   138.09	   693.36	 19
  5	 K 	   180.79	   669.68	 18
  6	 P 	   213.14	   626.98	 17
  7	 R 	   265.17	   594.63	 16
  8	 P 	   297.52	   542.60	 15
  9	 G 	   316.53	   510.24	 14
 10	 D 	   354.87	   491.24	 13
 11	 P 	   387.22	   452.89	 12
 12	 A 	   410.90	   420.54	 11
 13	 E 	   453.92	   396.86	 10
 14	 A 	   477.60	   353.85	  9
 15	 N 	   515.61	   330.17	  8
 16	 G 	   534.62	   292.16	  7
 17	 P 	   566.97	   273.15	  6
 18	 V 	   599.99	   240.80	  5
 19	 Y 	   654.35	   207.78	  4
 20	 P 	   686.70	   153.42	  3
 21	 W 	   748.72	   121.07	  2
 22	 R 	        -  	    59.04	  1  _
```

**125. IPI00219217 L-lactate dehydrogenase B chain**

| Sequence | Charge | M+H+ | XCorr | DeltaCn |
| --- | --- | --- | --- | --- |
| K.SADTLWDIQKDLK.D | 2 | 1533.708 | 3.682 | 0.215 |

+1 charge

```
		      B   	     Y
  1	 S 	    88.04	        -  	 13
  2	 A 	   159.08	  1445.76	 12
  3	 D 	   274.10	  1374.73	 11
  4	 T 	   375.15	  1259.70	 10
  5	 L 	   488.24	  1158.65	  9
  6	 W 	   674.31	  1045.57	  8
  7	 D 	   789.34	   859.49	  7
  8	 I 	   902.43	   744.46	  6
  9	 Q 	  1030.48	   631.38	  5
 10	 K 	  1158.58	   503.32	  4
 11	 D 	  1273.61	   375.22	  3
 12	 L 	  1386.69	   260.20	  2
 13	 K 	        -  	   147.11	  1  _
+2 charge
		      B   	     Y
  1	 S 	    44.52	        -  	 13
  2	 A 	    80.04	   723.39	 12
  3	 D 	   137.56	   687.87	 11
  4	 T 	   188.08	   630.35	 10
  5	 L 	   244.62	   579.83	  9
  6	 W 	   337.66	   523.29	  8
  7	 D 	   395.17	   430.25	  7
  8	 I 	   451.72	   372.73	  6
  9	 Q 	   515.75	   316.19	  5
 10	 K 	   579.79	   252.16	  4
 11	 D 	   637.31	   188.12	  3
 12	 L 	   693.85	   130.60	  2
 13	 K 	        -  	    74.06	  1  _
```

**126. IPI00940065 Isoform 2 of Insulin-like growth factor II**

| Sequence | Charge | M+H+ | XCorr | DeltaCn |
| --- | --- | --- | --- | --- |
| R.PLIALPTQDPAHGGAPPEMASNR.K | 3 | 2341.632 | 3.427 | 0.214 |

+1 charge

```
		      B   	     Y
  1	 P 	    98.06	        -  	 23
  2	 L 	   211.14	  2243.12	 22
  3	 I 	   324.23	  2130.04	 21
  4	 A 	   395.27	  2016.96	 20
  5	 L 	   508.35	  1945.92	 19
  6	 P 	   605.40	  1832.83	 18
  7	 T 	   706.45	  1735.78	 17
  8	 Q 	   834.51	  1634.73	 16
  9	 D 	   949.54	  1506.68	 15
 10	 P 	  1046.59	  1391.65	 14
 11	 A 	  1117.63	  1294.60	 13
 12	 H 	  1254.68	  1223.56	 12
 13	 G 	  1311.71	  1086.50	 11
 14	 G 	  1368.73	  1029.48	 10
 15	 A 	  1439.76	   972.46	  9
 16	 P 	  1536.82	   901.42	  8
 17	 P 	  1633.87	   804.37	  7
 18	 E 	  1762.91	   707.31	  6
 19	 M 	  1893.95	   578.27	  5
 20	 A 	  1964.99	   447.23	  4
 21	 S 	  2052.02	   376.19	  3
 22	 N 	  2166.06	   289.16	  2
 23	 R 	        -  	   175.12	  1  _


+2 charge
		      B   	     Y
  1	 P 	    49.53	        -  	 23
  2	 L 	   106.08	  1122.07	 22
  3	 I 	   162.62	  1065.52	 21
  4	 A 	   198.14	  1008.98	 20
  5	 L 	   254.68	   973.46	 19
  6	 P 	   303.20	   916.92	 18
  7	 T 	   353.73	   868.39	 17
  8	 Q 	   417.76	   817.87	 16
  9	 D 	   475.27	   753.84	 15
 10	 P 	   523.80	   696.33	 14
 11	 A 	   559.32	   647.80	 13
 12	 H 	   627.85	   612.28	 12
 13	 G 	   656.36	   543.75	 11
 14	 G 	   684.87	   515.24	 10
 15	 A 	   720.39	   486.73	  9
 16	 P 	   768.91	   451.21	  8
 17	 P 	   817.44	   402.69	  7
 18	 E 	   881.96	   354.16	  6
 19	 M 	   947.48	   289.64	  5
 20	 A 	   983.00	   224.12	  4
 21	 S 	  1026.51	   188.60	  3
 22	 N 	  1083.54	   145.08	  2
 23	 R 	        -  	    88.06	  1  _
+3 charge
		      B   	     Y
  1	 P 	    33.36	        -  	 23
  2	 L 	    71.05	   748.38	 22
  3	 I 	   108.75	   710.68	 21
  4	 A 	   132.43	   672.99	 20
  5	 L 	   170.12	   649.31	 19
  6	 P 	   202.47	   611.62	 18
  7	 T 	   236.15	   579.27	 17
  8	 Q 	   278.84	   545.58	 16
  9	 D 	   317.18	   502.90	 15
 10	 P 	   349.53	   464.55	 14
 11	 A 	   373.21	   432.20	 13
 12	 H 	   418.90	   408.52	 12
 13	 G 	   437.91	   362.84	 11
 14	 G 	   456.91	   343.83	 10
 15	 A 	   480.59	   324.82	  9
 16	 P 	   512.94	   301.14	  8
 17	 P 	   545.29	   268.79	  7
 18	 E 	   588.31	   236.44	  6
 19	 M 	   631.99	   193.43	  5
 20	 A 	   655.67	   149.75	  4
 21	 S 	   684.68	   126.07	  3
 22	 N 	   722.69	    97.06	  2
 23	 R 	        -  	    59.04	  1  _
```

**127. IPI00005526 PRO0419**

| Sequence | Charge | M+H+ | XCorr | DeltaCn |
| --- | --- | --- | --- | --- |
| R.RSELFILPQDDLDPPNFPCSR.P | 3 | 2517.772 | 4.005 | 0.265 |

+1 charge

```
		      B   	     Y
  1	 R 	   157.11	        -  	 21
  2	 S 	   244.14	  2360.26	 20
  3	 E 	   373.18	  2273.23	 19
  4	 L 	   486.27	  2144.18	 18
  5	 F 	   633.34	  2031.10	 17
  6	 I 	   746.42	  1884.03	 16
  7	 L 	   859.50	  1770.95	 15
  8	 P 	   956.56	  1657.86	 14
  9	 Q 	  1084.61	  1560.81	 13
 10	 D 	  1199.64	  1432.75	 12
 11	 D 	  1314.67	  1317.72	 11
 12	 L 	  1427.75	  1202.70	 10
 13	 D 	  1542.78	  1089.61	  9
 14	 P 	  1639.83	   974.59	  8
 15	 P 	  1736.89	   877.53	  7
 16	 N 	  1850.93	   780.48	  6
 17	 F 	  1998.00	   666.44	  5
 18	 P 	  2095.05	   519.37	  4
 19	 C 	  2255.21	   422.32	  3
 20	 S 	  2342.25	   262.15	  2
 21	 R 	        -  	   175.12	  1  _


+2 charge
		      B   	     Y
  1	 R 	    79.06	        -  	 21
  2	 S 	   122.57	  1180.63	 20
  3	 E 	   187.10	  1137.12	 19
  4	 L 	   243.64	  1072.59	 18
  5	 F 	   317.17	  1016.05	 17
  6	 I 	   373.71	   942.52	 16
  7	 L 	   430.26	   885.98	 15
  8	 P 	   478.78	   829.43	 14
  9	 Q 	   542.81	   780.91	 13
 10	 D 	   600.32	   716.88	 12
 11	 D 	   657.84	   659.37	 11
 12	 L 	   714.38	   601.85	 10
 13	 D 	   771.89	   545.31	  9
 14	 P 	   820.42	   487.80	  8
 15	 P 	   868.95	   439.27	  7
 16	 N 	   925.97	   390.74	  6
 17	 F 	   999.50	   333.72	  5
 18	 P 	  1048.03	   260.19	  4
 19	 C 	  1128.11	   211.66	  3
 20	 S 	  1171.63	   131.58	  2
 21	 R 	        -  	    88.06	  1  _
+3 charge
		      B   	     Y
  1	 R 	    53.04	        -  	 21
  2	 S 	    82.05	   787.42	 20
  3	 E 	   125.07	   758.41	 19
  4	 L 	   162.76	   715.40	 18
  5	 F 	   211.78	   677.70	 17
  6	 I 	   249.48	   628.68	 16
  7	 L 	   287.17	   590.99	 15
  8	 P 	   319.52	   553.29	 14
  9	 Q 	   362.21	   520.94	 13
 10	 D 	   400.55	   478.26	 12
 11	 D 	   438.89	   439.91	 11
 12	 L 	   476.59	   401.57	 10
 13	 D 	   514.93	   363.88	  9
 14	 P 	   547.28	   325.53	  8
 15	 P 	   579.63	   293.18	  7
 16	 N 	   617.65	   260.83	  6
 17	 F 	   666.67	   222.82	  5
 18	 P 	   699.02	   173.79	  4
 19	 C 	   752.41	   141.44	  3
 20	 S 	   781.42	    88.06	  2
 21	 R 	        -  	    59.04	  1  _
```

**128. IPI00306322 Collagen alpha-2(IV) chain**

| Sequence | Charge | M+H+ | XCorr | DeltaCn |
| --- | --- | --- | --- | --- |
| K.IAVQPGTVGPQGR.R | 2 | 1280.459 | 3.35 | 0.465 |

+1 charge

```
		      B   	     Y
  1	 I 	   114.09	        -  	 13
  2	 A 	   185.13	  1166.63	 12
  3	 V 	   284.20	  1095.59	 11
  4	 Q 	   412.26	   996.52	 10
  5	 P 	   509.31	   868.46	  9
  6	 G 	   566.33	   771.41	  8
  7	 T 	   667.38	   714.39	  7
  8	 V 	   766.45	   613.34	  6
  9	 G 	   823.47	   514.27	  5
 10	 P 	   920.52	   457.25	  4
 11	 Q 	  1048.58	   360.20	  3
 12	 G 	  1105.60	   232.14	  2
 13	 R 	        -  	   175.12	  1  _
+2 charge
		      B   	     Y
  1	 I 	    57.55	        -  	 13
  2	 A 	    93.07	   583.82	 12
  3	 V 	   142.60	   548.30	 11
  4	 Q 	   206.63	   498.76	 10
  5	 P 	   255.16	   434.74	  9
  6	 G 	   283.67	   386.21	  8
  7	 T 	   334.19	   357.70	  7
  8	 V 	   383.73	   307.17	  6
  9	 G 	   412.24	   257.64	  5
 10	 P 	   460.76	   229.13	  4
 11	 Q 	   524.79	   180.60	  3
 12	 G 	   553.30	   116.57	  2
 13	 R 	        -  	    88.06	  1  _
```

**129. IPI00789902 Isoform 2 of G-protein coupled receptor family C group 5 member C**

| Sequence | Charge | M+H+ | XCorr | DeltaCn |
| --- | --- | --- | --- | --- |
| R.ATANSQVMGSANSTLR.A | 2 | 1608.76 | 3.978 | 0.439 |

+1 charge

```
		      B   	     Y
  1	 A 	    72.04	        -  	 16
  2	 T 	   173.09	  1536.74	 15
  3	 A 	   244.13	  1435.70	 14
  4	 N 	   358.17	  1364.66	 13
  5	 S 	   445.20	  1250.62	 12
  6	 Q 	   573.26	  1163.58	 11
  7	 V 	   672.33	  1035.53	 10
  8	 M 	   803.37	   936.46	  9
  9	 G 	   860.39	   805.42	  8
 10	 S 	   947.43	   748.39	  7
 11	 A 	  1018.46	   661.36	  6
 12	 N 	  1132.51	   590.33	  5
 13	 S 	  1219.54	   476.28	  4
 14	 T 	  1320.58	   389.25	  3
 15	 L 	  1433.67	   288.20	  2
 16	 R 	        -  	   175.12	  1  _
+2 charge
		      B   	     Y
  1	 A 	    36.53	        -  	 16
  2	 T 	    87.05	   768.88	 15
  3	 A 	   122.57	   718.35	 14
  4	 N 	   179.59	   682.83	 13
  5	 S 	   223.11	   625.81	 12
  6	 Q 	   287.13	   582.30	 11
  7	 V 	   336.67	   518.27	 10
  8	 M 	   402.19	   468.73	  9
  9	 G 	   430.70	   403.21	  8
 10	 S 	   474.22	   374.70	  7
 11	 A 	   509.73	   331.19	  6
 12	 N 	   566.76	   295.67	  5
 13	 S 	   610.27	   238.64	  4
 14	 T 	   660.80	   195.13	  3
 15	 L 	   717.34	   144.61	  2
 16	 R 	        -  	    88.06	  1  _
```

**130. IPI00604773 PODXL protein**

| Sequence | Charge | M+H+ | XCorr | DeltaCn |
| --- | --- | --- | --- | --- |
| R.LASVPGSQTVVVK.E | 2 | 1285.515 | 3.014 | 0.468 |

+1 charge

```
		      B   	     Y
  1	 L 	   114.09	        -  	 13
  2	 A 	   185.13	  1171.67	 12
  3	 S 	   272.16	  1100.63	 11
  4	 V 	   371.23	  1013.60	 10
  5	 P 	   468.28	   914.53	  9
  6	 G 	   525.30	   817.48	  8
  7	 S 	   612.34	   760.46	  7
  8	 Q 	   740.39	   673.42	  6
  9	 T 	   841.44	   545.37	  5
 10	 V 	   940.51	   444.32	  4
 11	 V 	  1039.58	   345.25	  3
 12	 V 	  1138.65	   246.18	  2
 13	 K 	        -  	   147.11	  1  _
+2 charge
		      B   	     Y
  1	 L 	    57.55	        -  	 13
  2	 A 	    93.07	   586.34	 12
  3	 S 	   136.58	   550.82	 11
  4	 V 	   186.12	   507.30	 10
  5	 P 	   234.64	   457.77	  9
  6	 G 	   263.16	   409.24	  8
  7	 S 	   306.67	   380.73	  7
  8	 Q 	   370.70	   337.22	  6
  9	 T 	   421.22	   273.19	  5
 10	 V 	   470.76	   222.66	  4
 11	 V 	   520.29	   173.13	  3
 12	 V 	   569.83	   123.59	  2
 13	 K 	        -  	    74.06	  1  _
```

**131. IPI00056309 Liver-expressed antimicrobial peptide 2**

| Sequence | Charge | M+H+ | XCorr | DeltaCn |
| --- | --- | --- | --- | --- |
| R.GVSLRPIGASCRDDSECITR.L | 3 | 2250.443 | 4.143 | 0.387 |

+1 charge

```
		      B   	     Y
  1	 G 	    58.03	        -  	 20
  2	 V 	   157.10	  2192.32	 19
  3	 S 	   244.13	  2093.26	 18
  4	 L 	   357.21	  2006.22	 17
  5	 R 	   513.31	  1893.14	 16
  6	 P 	   610.37	  1737.04	 15
  7	 I 	   723.45	  1639.99	 14
  8	 G 	   780.47	  1526.90	 13
  9	 A 	   851.51	  1469.88	 12
 10	 S 	   938.54	  1398.84	 11
 11	 C 	  1098.71	  1311.81	 10
 12	 R 	  1254.81	  1151.65	  9
 13	 D 	  1369.84	   995.54	  8
 14	 D 	  1484.86	   880.52	  7
 15	 S 	  1571.89	   765.49	  6
 16	 E 	  1700.94	   678.46	  5
 17	 C 	  1861.10	   549.42	  4
 18	 I 	  1974.19	   389.25	  3
 19	 T 	  2075.23	   276.17	  2
 20	 R 	        -  	   175.12	  1  _
+2 charge
		      B   	     Y
  1	 G 	    29.52	        -  	 20
  2	 V 	    79.05	  1096.67	 19
  3	 S 	   122.57	  1047.13	 18
  4	 L 	   179.11	  1003.62	 17
  5	 R 	   257.16	   947.07	 16
  6	 P 	   305.69	   869.02	 15
  7	 I 	   362.23	   820.50	 14
  8	 G 	   390.74	   763.95	 13
  9	 A 	   426.26	   735.44	 12
 10	 S 	   469.77	   699.93	 11
 11	 C 	   549.86	   656.41	 10
 12	 R 	   627.91	   576.33	  9
 13	 D 	   685.42	   498.28	  8
 14	 D 	   742.93	   440.76	  7
 15	 S 	   786.45	   383.25	  6
 16	 E 	   850.97	   339.73	  5
 17	 C 	   931.05	   275.21	  4
 18	 I 	   987.60	   195.13	  3
 19	 T 	  1038.12	   138.59	  2
 20	 R 	        -  	    88.06	  1  _

+3 charge
		      B   	     Y
  1	 G 	    20.01	        -  	 20
  2	 V 	    53.04	   731.45	 19
  3	 S 	    82.05	   698.42	 18
  4	 L 	   119.74	   669.41	 17
  5	 R 	   171.78	   631.72	 16
  6	 P 	   204.13	   579.68	 15
  7	 I 	   241.82	   547.33	 14
  8	 G 	   260.83	   509.64	 13
  9	 A 	   284.51	   490.63	 12
 10	 S 	   313.52	   466.95	 11
 11	 C 	   366.91	   437.94	 10
 12	 R 	   418.94	   384.55	  9
 13	 D 	   457.28	   332.52	  8
 14	 D 	   495.63	   294.18	  7
 15	 S 	   524.64	   255.84	  6
 16	 E 	   567.65	   226.82	  5
 17	 C 	   621.04	   183.81	  4
 18	 I 	   658.73	   130.42	  3
 19	 T 	   692.42	    92.73	  2
 20	 R 	        -  	    59.04	  1  _
```

**132. IPI00414784 Isoform 1 of CMRF35-like molecule 8**

| Sequence | Charge | M+H+ | XCorr | DeltaCn |
| --- | --- | --- | --- | --- |
| R.TVAGPVGGSLSVQCPYEK.E | 2 | 1850.056 | 2.817 | 0.563 |

+1 charge

```
		      B   	     Y
  1	 T 	   102.05	        -  	 18
  2	 V 	   201.12	  1748.00	 17
  3	 A 	   272.16	  1648.93	 16
  4	 G 	   329.18	  1577.90	 15
  5	 P 	   426.23	  1520.88	 14
  6	 V 	   525.30	  1423.82	 13
  7	 G 	   582.32	  1324.75	 12
  8	 G 	   639.35	  1267.73	 11
  9	 S 	   726.38	  1210.71	 10
 10	 L 	   839.46	  1123.68	  9
 11	 S 	   926.49	  1010.60	  8
 12	 V 	  1025.56	   923.56	  7
 13	 Q 	  1153.62	   824.50	  6
 14	 C 	  1313.79	   696.44	  5
 15	 P 	  1410.84	   536.27	  4
 16	 Y 	  1573.90	   439.22	  3
 17	 E 	  1702.95	   276.16	  2
 18	 K 	        -  	   147.11	  1  _
+2 charge
		      B   	     Y
  1	 T 	    51.53	        -  	 18
  2	 V 	   101.07	   874.51	 17
  3	 A 	   136.58	   824.97	 16
  4	 G 	   165.09	   789.45	 15
  5	 P 	   213.62	   760.94	 14
  6	 V 	   263.16	   712.42	 13
  7	 G 	   291.67	   662.88	 12
  8	 G 	   320.18	   634.37	 11
  9	 S 	   363.69	   605.86	 10
 10	 L 	   420.23	   562.34	  9
 11	 S 	   463.75	   505.80	  8
 12	 V 	   513.28	   462.29	  7
 13	 Q 	   577.31	   412.75	  6
 14	 C 	   657.40	   348.72	  5
 15	 P 	   705.92	   268.64	  4
 16	 Y 	   787.45	   220.11	  3
 17	 E 	   851.98	   138.58	  2
 18	 K 	        -  	    74.06	  1  _
```

**133. IPI00298281 Laminin subunit gamma-1**

| Sequence | Charge | M+H+ | XCorr | DeltaCn |
| --- | --- | --- | --- | --- |
| R.SQECYFDPELYR.S | 2 | 1607.695 | 2.917 | 0.477 |

+1 charge

```
		      B   	     Y
  1	 S 	    88.04	        -  	 12
  2	 Q 	   216.10	  1519.79	 11
  3	 E 	   345.14	  1391.73	 10
  4	 C 	   505.31	  1262.69	  9
  5	 Y 	   668.37	  1102.52	  8
  6	 F 	   815.44	   939.46	  7
  7	 D 	   930.46	   792.39	  6
  8	 P 	  1027.52	   677.36	  5
  9	 E 	  1156.56	   580.31	  4
 10	 L 	  1269.64	   451.27	  3
 11	 Y 	  1432.71	   338.18	  2
 12	 R 	        -  	   175.12	  1  _
+2 charge
		      B   	     Y
  1	 S 	    44.52	        -  	 12
  2	 Q 	   108.55	   760.40	 11
  3	 E 	   173.07	   696.37	 10
  4	 C 	   253.16	   631.85	  9
  5	 Y 	   334.69	   551.76	  8
  6	 F 	   408.22	   470.23	  7
  7	 D 	   465.74	   396.70	  6
  8	 P 	   514.26	   339.18	  5
  9	 E 	   578.78	   290.66	  4
 10	 L 	   635.33	   226.14	  3
 11	 Y 	   716.86	   169.59	  2
 12	 R 	        -  	    88.06	  1  _
```

**134. IPI00022488 Hemopexin**

| Sequence | Charge | M+H+ | XCorr | DeltaCn |
| --- | --- | --- | --- | --- |
| R.YYCFQGNQFLR.F | 2 | 1496.644 | 3.2 | 0.505 |

+1 charge

```
		      B   	     Y
  1	 Y 	   164.07	        -  	 11
  2	 Y 	   327.13	  1332.75	 10
  3	 C 	   487.30	  1169.69	  9
  4	 F 	   634.37	  1009.52	  8
  5	 Q 	   762.43	   862.45	  7
  6	 G 	   819.45	   734.39	  6
  7	 N 	   933.49	   677.37	  5
  8	 Q 	  1061.55	   563.33	  4
  9	 F 	  1208.62	   435.27	  3
 10	 L 	  1321.70	   288.20	  2
 11	 R 	        -  	   175.12	  1  _
+2 charge
		      B   	     Y
  1	 Y 	    82.54	        -  	 11
  2	 Y 	   164.07	   666.88	 10
  3	 C 	   244.15	   585.35	  9
  4	 F 	   317.69	   505.26	  8
  5	 Q 	   381.72	   431.73	  7
  6	 G 	   410.23	   367.70	  6
  7	 N 	   467.25	   339.19	  5
  8	 Q 	   531.28	   282.17	  4
  9	 F 	   604.81	   218.14	  3
 10	 L 	   661.35	   144.61	  2
 11	 R 	        -  	    88.06	  1  _
```

**135. IPI00410487 Isoform 1 of Twisted gastrulation protein homolog 1**

| Sequence | Charge | M+H+ | XCorr | DeltaCn |
| --- | --- | --- | --- | --- |
| K.STVEELHEPIPSLFR.A | 2 | 1754.964 | 4.547 | 0.573 |

+1 charge

```
		      B   	     Y
  1	 S 	    88.04	        -  	 15
  2	 T 	   189.09	  1666.88	 14
  3	 V 	   288.16	  1565.83	 13
  4	 E 	   417.20	  1466.76	 12
  5	 E 	   546.24	  1337.72	 11
  6	 L 	   659.32	  1208.68	 10
  7	 H 	   796.38	  1095.59	  9
  8	 E 	   925.43	   958.54	  8
  9	 P 	  1022.48	   829.49	  7
 10	 I 	  1135.56	   732.44	  6
 11	 P 	  1232.62	   619.36	  5
 12	 S 	  1319.65	   522.30	  4
 13	 L 	  1432.73	   435.27	  3
 14	 F 	  1579.80	   322.19	  2
 15	 R 	        -  	   175.12	  1  _
+2 charge
		      B   	     Y
  1	 S 	    44.52	        -  	 15
  2	 T 	    95.05	   833.94	 14
  3	 V 	   144.58	   783.42	 13
  4	 E 	   209.10	   733.89	 12
  5	 E 	   273.62	   669.36	 11
  6	 L 	   330.17	   604.84	 10
  7	 H 	   398.70	   548.30	  9
  8	 E 	   463.22	   479.77	  8
  9	 P 	   511.74	   415.25	  7
 10	 I 	   568.29	   366.72	  6
 11	 P 	   616.81	   310.18	  5
 12	 S 	   660.33	   261.66	  4
 13	 L 	   716.87	   218.14	  3
 14	 F 	   790.40	   161.60	  2
 15	 R 	        -  	    88.06	  1  _
```

**136. IPI00102300 Isoform 3 of Platelet glycoprotein VI**

| Sequence | Charge | M+H+ | XCorr | DeltaCn |
| --- | --- | --- | --- | --- |
| R.YQDQAVLFIPAMK.R | 2 | 1524.809 | 3.139 | 0.401 |

+1 charge

```
		      B   	     Y
  1	 Y 	   164.07	        -  	 13
  2	 Q 	   292.13	  1360.73	 12
  3	 D 	   407.16	  1232.67	 11
  4	 Q 	   535.21	  1117.64	 10
  5	 A 	   606.25	   989.59	  9
  6	 V 	   705.32	   918.55	  8
  7	 L 	   818.40	   819.48	  7
  8	 F 	   965.47	   706.40	  6
  9	 I 	  1078.56	   559.33	  5
 10	 P 	  1175.61	   446.24	  4
 11	 A 	  1246.65	   349.19	  3
 12	 M 	  1377.69	   278.15	  2
 13	 K 	        -  	   147.11	  1  _
+2 charge
		      B   	     Y
  1	 Y 	    82.54	        -  	 13
  2	 Q 	   146.57	   680.87	 12
  3	 D 	   204.08	   616.84	 11
  4	 Q 	   268.11	   559.33	 10
  5	 A 	   303.63	   495.30	  9
  6	 V 	   353.16	   459.78	  8
  7	 L 	   409.71	   410.24	  7
  8	 F 	   483.24	   353.70	  6
  9	 I 	   539.78	   280.17	  5
 10	 P 	   588.31	   223.63	  4
 11	 A 	   623.83	   175.10	  3
 12	 M 	   689.35	   139.58	  2
 13	 K 	        -  	    74.06	  1  _
```

**137. IPI00021885 Isoform 1 of Fibrinogen alpha chain**

| Sequence | Charge | M+H+ | XCorr | DeltaCn |
| --- | --- | --- | --- | --- |
| R.GFGSLNDEGEGEFWLGNDYLHLLTQR.G | 3 | 2969.17 | 5.933 | 0.687 |

+1 charge

```
		      B   	     Y
  1	 G 	    58.03	        -  	 26
  2	 F 	   205.10	  2910.37	 25
  3	 G 	   262.12	  2763.30	 24
  4	 S 	   349.15	  2706.28	 23
  5	 L 	   462.23	  2619.25	 22
  6	 N 	   576.28	  2506.16	 21
  7	 D 	   691.30	  2392.12	 20
  8	 E 	   820.35	  2277.09	 19
  9	 G 	   877.37	  2148.05	 18
 10	 E 	  1006.41	  2091.03	 17
 11	 G 	  1063.43	  1961.99	 16
 12	 E 	  1192.48	  1904.97	 15
 13	 F 	  1339.54	  1775.92	 14
 14	 W 	  1525.62	  1628.85	 13
 15	 L 	  1638.71	  1442.78	 12
 16	 G 	  1695.73	  1329.69	 11
 17	 N 	  1809.77	  1272.67	 10
 18	 D 	  1924.80	  1158.63	  9
 19	 Y 	  2087.86	  1043.60	  8
 20	 L 	  2200.95	   880.54	  7
 21	 H 	  2338.00	   767.45	  6
 22	 L 	  2451.09	   630.39	  5
 23	 L 	  2564.17	   517.31	  4
 24	 T 	  2665.22	   404.23	  3
 25	 Q 	  2793.28	   303.18	  2
 26	 R 	        -  	   175.12	  1  _


+2 charge
		      B   	     Y
  1	 G 	    29.52	        -  	 26
  2	 F 	   103.05	  1455.69	 25
  3	 G 	   131.56	  1382.15	 24
  4	 S 	   175.08	  1353.64	 23
  5	 L 	   231.62	  1310.13	 22
  6	 N 	   288.64	  1253.59	 21
  7	 D 	   346.16	  1196.56	 20
  8	 E 	   410.68	  1139.05	 19
  9	 G 	   439.19	  1074.53	 18
 10	 E 	   503.71	  1046.02	 17
 11	 G 	   532.22	   981.50	 16
 12	 E 	   596.74	   952.99	 15
 13	 F 	   670.28	   888.47	 14
 14	 W 	   763.32	   814.93	 13
 15	 L 	   819.86	   721.89	 12
 16	 G 	   848.37	   665.35	 11
 17	 N 	   905.39	   636.84	 10
 18	 D 	   962.90	   579.82	  9
 19	 Y 	  1044.43	   522.30	  8
 20	 L 	  1100.98	   440.77	  7
 21	 H 	  1169.51	   384.23	  6
 22	 L 	  1226.05	   315.70	  5
 23	 L 	  1282.59	   259.16	  4
 24	 T 	  1333.11	   202.62	  3
 25	 Q 	  1397.14	   152.09	  2
 26	 R 	        -  	    88.06	  1  _


+3 charge
		      B   	     Y
  1	 G 	    20.01	        -  	 26
  2	 F 	    69.04	   970.79	 25
  3	 G 	    88.04	   921.77	 24
  4	 S 	   117.06	   902.76	 23
  5	 L 	   154.75	   873.75	 22
  6	 N 	   192.76	   836.06	 21
  7	 D 	   231.11	   798.04	 20
  8	 E 	   274.12	   759.70	 19
  9	 G 	   293.13	   716.69	 18
 10	 E 	   336.14	   697.68	 17
 11	 G 	   355.15	   654.67	 16
 12	 E 	   398.16	   635.66	 15
 13	 F 	   447.19	   592.65	 14
 14	 W 	   509.21	   543.62	 13
 15	 L 	   546.91	   481.60	 12
 16	 G 	   565.91	   443.90	 11
 17	 N 	   603.93	   424.89	 10
 18	 D 	   642.27	   386.88	  9
 19	 Y 	   696.63	   348.54	  8
 20	 L 	   734.32	   294.18	  7
 21	 H 	   780.01	   256.49	  6
 22	 L 	   817.70	   210.80	  5
 23	 L 	   855.40	   173.11	  4
 24	 T 	   889.08	   135.41	  3
 25	 Q 	   931.76	   101.73	  2
 26	 R 	        -  	    59.04	  1  _
```

**138. IPI00009865 Keratin, type I cytoskeletal 10**

| Sequence | Charge | M+H+ | XCorr | DeltaCn |
| --- | --- | --- | --- | --- |
| K.TIDDLKNQILNLTTDNANILLQIDNAR.L | 3 | 3054.402 | 3.516 | 0.421 |

+1 charge

```
		      B   	     Y
  1	 T 	   102.05	        -  	 27
  2	 I 	   215.14	  2951.58	 26
  3	 D 	   330.17	  2838.50	 25
  4	 D 	   445.19	  2723.47	 24
  5	 L 	   558.28	  2608.44	 23
  6	 K 	   686.37	  2495.36	 22
  7	 N 	   800.41	  2367.26	 21
  8	 Q 	   928.47	  2253.22	 20
  9	 I 	  1041.56	  2125.16	 19
 10	 L 	  1154.64	  2012.08	 18
 11	 N 	  1268.68	  1898.99	 17
 12	 L 	  1381.77	  1784.95	 16
 13	 T 	  1482.82	  1671.87	 15
 14	 T 	  1583.86	  1570.82	 14
 15	 D 	  1698.89	  1469.77	 13
 16	 N 	  1812.93	  1354.74	 12
 17	 A 	  1883.97	  1240.70	 11
 18	 N 	  1998.01	  1169.66	 10
 19	 I 	  2111.10	  1055.62	  9
 20	 L 	  2224.18	   942.54	  8
 21	 L 	  2337.27	   829.45	  7
 22	 Q 	  2465.32	   716.37	  6
 23	 I 	  2578.41	   588.31	  5
 24	 D 	  2693.44	   475.23	  4
 25	 N 	  2807.48	   360.20	  3
 26	 A 	  2878.52	   246.16	  2
 27	 R 	        -  	   175.12	  1  _


+2 charge
		      B   	     Y
  1	 T 	    51.53	        -  	 27
  2	 I 	   108.07	  1476.29	 26
  3	 D 	   165.59	  1419.75	 25
  4	 D 	   223.10	  1362.24	 24
  5	 L 	   279.64	  1304.72	 23
  6	 K 	   343.69	  1248.18	 22
  7	 N 	   400.71	  1184.13	 21
  8	 Q 	   464.74	  1127.11	 20
  9	 I 	   521.28	  1063.08	 19
 10	 L 	   577.82	  1006.54	 18
 11	 N 	   634.85	   950.00	 17
 12	 L 	   691.39	   892.98	 16
 13	 T 	   741.91	   836.44	 15
 14	 T 	   792.44	   785.91	 14
 15	 D 	   849.95	   735.39	 13
 16	 N 	   906.97	   677.88	 12
 17	 A 	   942.49	   620.85	 11
 18	 N 	   999.51	   585.34	 10
 19	 I 	  1056.05	   528.31	  9
 20	 L 	  1112.59	   471.77	  8
 21	 L 	  1169.14	   415.23	  7
 22	 Q 	  1233.17	   358.69	  6
 23	 I 	  1289.71	   294.66	  5
 24	 D 	  1347.22	   238.12	  4
 25	 N 	  1404.24	   180.60	  3
 26	 A 	  1439.76	   123.58	  2
 27	 R 	        -  	    88.06	  1  _


+3 charge
		      B   	     Y
  1	 T 	    34.69	        -  	 27
  2	 I 	    72.38	   984.53	 26
  3	 D 	   110.73	   946.84	 25
  4	 D 	   149.07	   908.49	 24
  5	 L 	   186.76	   870.15	 23
  6	 K 	   229.46	   832.46	 22
  7	 N 	   267.48	   789.76	 21
  8	 Q 	   310.16	   751.74	 20
  9	 I 	   347.86	   709.06	 19
 10	 L 	   385.55	   671.36	 18
 11	 N 	   423.57	   633.67	 17
 12	 L 	   461.26	   595.65	 16
 13	 T 	   494.94	   557.96	 15
 14	 T 	   528.63	   524.28	 14
 15	 D 	   566.97	   490.60	 13
 16	 N 	   604.98	   452.25	 12
 17	 A 	   628.66	   414.24	 11
 18	 N 	   666.68	   390.56	 10
 19	 I 	   704.37	   352.55	  9
 20	 L 	   742.07	   314.85	  8
 21	 L 	   779.76	   277.16	  7
 22	 Q 	   822.45	   239.46	  6
 23	 I 	   860.14	   196.77	  5
 24	 D 	   898.48	   159.08	  4
 25	 N 	   936.50	   120.74	  3
 26	 A 	   960.18	    82.72	  2
 27	 R 	        -  	    59.04	  1  _
```

**139. IPI00916111 Malate dehydrogenase**

| Sequence | Charge | M+H+ | XCorr | DeltaCn |
| --- | --- | --- | --- | --- |
| K.SVKVIVVGNPANTNCLTASK.S | 2 | 2073.373 | 2.85 | 0.27 |

+1 charge

```
		      B   	     Y
  1	 S 	    88.04	        -  	 20
  2	 V 	   187.11	  1985.22	 19
  3	 K 	   315.20	  1886.15	 18
  4	 V 	   414.27	  1758.06	 17
  5	 I 	   527.36	  1658.99	 16
  6	 V 	   626.42	  1545.90	 15
  7	 V 	   725.49	  1446.84	 14
  8	 G 	   782.51	  1347.77	 13
  9	 N 	   896.56	  1290.75	 12
 10	 P 	   993.61	  1176.70	 11
 11	 A 	  1064.65	  1079.65	 10
 12	 N 	  1178.69	  1008.61	  9
 13	 T 	  1279.74	   894.57	  8
 14	 N 	  1393.78	   793.52	  7
 15	 C 	  1553.95	   679.48	  6
 16	 L 	  1667.03	   519.31	  5
 17	 T 	  1768.08	   406.23	  4
 18	 A 	  1839.11	   305.18	  3
 19	 S 	  1926.15	   234.14	  2
 20	 K 	        -  	   147.11	  1  _
+2 charge
		      B   	     Y
  1	 S 	    44.52	        -  	 20
  2	 V 	    94.06	   993.11	 19
  3	 K 	   158.10	   943.58	 18
  4	 V 	   207.64	   879.53	 17
  5	 I 	   264.18	   830.00	 16
  6	 V 	   313.72	   773.46	 15
  7	 V 	   363.25	   723.92	 14
  8	 G 	   391.76	   674.39	 13
  9	 N 	   448.78	   645.88	 12
 10	 P 	   497.31	   588.85	 11
 11	 A 	   532.83	   540.33	 10
 12	 N 	   589.85	   504.81	  9
 13	 T 	   640.37	   447.79	  8
 14	 N 	   697.39	   397.26	  7
 15	 C 	   777.48	   340.24	  6
 16	 L 	   834.02	   260.16	  5
 17	 T 	   884.54	   203.62	  4
 18	 A 	   920.06	   153.09	  3
 19	 S 	   963.58	   117.58	  2
 20	 K 	        -  	    74.06	  1  _
```

**140. IPI00873924 Isoform A of Interleukin-18-binding protein**

| Sequence | Charge | M+H+ | XCorr | DeltaCn |
| --- | --- | --- | --- | --- |
| R.STKDPCPSQPPVFPAAK.Q | 2 | 1828.052 | 3.683 | 0.468 |

+1 charge

```
		      B   	     Y
  1	 S 	    88.04	        -  	 17
  2	 T 	   189.09	  1740.01	 16
  3	 K 	   317.18	  1638.97	 15
  4	 D 	   432.21	  1510.87	 14
  5	 P 	   529.26	  1395.84	 13
  6	 C 	   689.43	  1298.79	 12
  7	 P 	   786.48	  1138.63	 11
  8	 S 	   873.51	  1041.57	 10
  9	 Q 	  1001.57	   954.54	  9
 10	 P 	  1098.62	   826.48	  8
 11	 P 	  1195.68	   729.43	  7
 12	 V 	  1294.74	   632.38	  6
 13	 F 	  1441.81	   533.31	  5
 14	 P 	  1538.87	   386.24	  4
 15	 A 	  1609.90	   289.19	  3
 16	 A 	  1680.94	   218.15	  2
 17	 K 	        -  	   147.11	  1  _
+2 charge
		      B   	     Y
  1	 S 	    44.52	        -  	 17
  2	 T 	    95.05	   870.51	 16
  3	 K 	   159.09	   819.99	 15
  4	 D 	   216.61	   755.94	 14
  5	 P 	   265.13	   698.43	 13
  6	 C 	   345.22	   649.90	 12
  7	 P 	   393.74	   569.82	 11
  8	 S 	   437.26	   521.29	 10
  9	 Q 	   501.29	   477.77	  9
 10	 P 	   549.82	   413.74	  8
 11	 P 	   598.34	   365.22	  7
 12	 V 	   647.88	   316.69	  6
 13	 F 	   721.41	   267.16	  5
 14	 P 	   769.94	   193.62	  4
 15	 A 	   805.45	   145.10	  3
 16	 A 	   840.97	   109.58	  2
 17	 K 	        -  	    74.06	  1  _
```

**141. IPI00441498 Folate receptor alpha**

| Sequence | Charge | M+H+ | XCorr | DeltaCn |
| --- | --- | --- | --- | --- |
| R.FNWNHCGEMAPACK.R | 2 | 1722.893 | 3.055 | 0.406 |

+1 charge

```
		      B   	     Y
  1	 F 	   148.08	        -  	 14
  2	 N 	   262.12	  1574.90	 13
  3	 W 	   448.20	  1460.86	 12
  4	 N 	   562.24	  1274.78	 11
  5	 H 	   699.30	  1160.73	 10
  6	 C 	   859.47	  1023.67	  9
  7	 G 	   916.49	   863.51	  8
  8	 E 	  1045.53	   806.49	  7
  9	 M 	  1176.57	   677.45	  6
 10	 A 	  1247.61	   546.41	  5
 11	 P 	  1344.66	   475.37	  4
 12	 A 	  1415.70	   378.32	  3
 13	 C 	  1575.86	   307.28	  2
 14	 K 	        -  	   147.11	  1  _
+2 charge
		      B   	     Y
  1	 F 	    74.54	        -  	 14
  2	 N 	   131.56	   787.95	 13
  3	 W 	   224.60	   730.93	 12
  4	 N 	   281.62	   637.89	 11
  5	 H 	   350.15	   580.87	 10
  6	 C 	   430.24	   512.34	  9
  7	 G 	   458.75	   432.26	  8
  8	 E 	   523.27	   403.75	  7
  9	 M 	   588.79	   339.23	  6
 10	 A 	   624.31	   273.71	  5
 11	 P 	   672.83	   238.19	  4
 12	 A 	   708.35	   189.66	  3
 13	 C 	   788.43	   154.14	  2
 14	 K 	        -  	    74.06	  1  _
```

**142. IPI00171611 Histone H3.2**

| Sequence | Charge | M+H+ | XCorr | DeltaCn |
| --- | --- | --- | --- | --- |
| R.EIAQDFKTDLR.F | 2 | 1336.476 | 3.374 | 0.387 |

+1 charge

```
		      B   	     Y
  1	 E 	   130.05	        -  	 11
  2	 I 	   243.13	  1206.65	 10
  3	 A 	   314.17	  1093.56	  9
  4	 Q 	   442.23	  1022.53	  8
  5	 D 	   557.26	   894.47	  7
  6	 F 	   704.32	   779.44	  6
  7	 K 	   832.42	   632.37	  5
  8	 T 	   933.47	   504.28	  4
  9	 D 	  1048.49	   403.23	  3
 10	 L 	  1161.58	   288.20	  2
 11	 R 	        -  	   175.12	  1  _
+2 charge
		      B   	     Y
  1	 E 	    65.53	        -  	 11
  2	 I 	   122.07	   603.83	 10
  3	 A 	   157.59	   547.29	  9
  4	 Q 	   221.62	   511.77	  8
  5	 D 	   279.13	   447.74	  7
  6	 F 	   352.67	   390.22	  6
  7	 K 	   416.71	   316.69	  5
  8	 T 	   467.24	   252.64	  4
  9	 D 	   524.75	   202.12	  3
 10	 L 	   581.29	   144.61	  2
 11	 R 	        -  	    88.06	  1  _
```

**143. IPI00017529 Isoform 1 of Lymphocyte function-associated antigen 3**

| Sequence | Charge | M+H+ | XCorr | DeltaCn |
| --- | --- | --- | --- | --- |
| K.DKVAELENSEFR.A | 2 | 1437.537 | 2.819 | 0.244 |

+1 charge

```
		      B   	     Y
  1	 D 	   116.03	        -  	 12
  2	 K 	   244.13	  1321.67	 11
  3	 V 	   343.20	  1193.58	 10
  4	 A 	   414.23	  1094.51	  9
  5	 E 	   543.28	  1023.47	  8
  6	 L 	   656.36	   894.43	  7
  7	 E 	   785.40	   781.35	  6
  8	 N 	   899.45	   652.30	  5
  9	 S 	   986.48	   538.26	  4
 10	 E 	  1115.52	   451.23	  3
 11	 F 	  1262.59	   322.19	  2
 12	 R 	        -  	   175.12	  1  _
+2 charge
		      B   	     Y
  1	 D 	    58.52	        -  	 12
  2	 K 	   122.57	   661.34	 11
  3	 V 	   172.10	   597.29	 10
  4	 A 	   207.62	   547.76	  9
  5	 E 	   272.14	   512.24	  8
  6	 L 	   328.68	   447.72	  7
  7	 E 	   393.21	   391.18	  6
  8	 N 	   450.23	   326.66	  5
  9	 S 	   493.74	   269.63	  4
 10	 E 	   558.26	   226.12	  3
 11	 F 	   631.80	   161.60	  2
 12	 R 	        -  	    88.06	  1  _
```

**144. IPI00289931 MADCAM1 protein**

| Sequence | Charge | M+H+ | XCorr | DeltaCn |
| --- | --- | --- | --- | --- |
| R.GLDTSLGAVQSDTGR.S | 2 | 1477.559 | 4.893 | 0.479 |

+1 charge

```
		      B   	     Y
  1	 G 	    58.03	        -  	 15
  2	 L 	   171.11	  1419.71	 14
  3	 D 	   286.14	  1306.62	 13
  4	 T 	   387.19	  1191.60	 12
  5	 S 	   474.22	  1090.55	 11
  6	 L 	   587.30	  1003.52	 10
  7	 G 	   644.32	   890.43	  9
  8	 A 	   715.36	   833.41	  8
  9	 V 	   814.43	   762.37	  7
 10	 Q 	   942.49	   663.31	  6
 11	 S 	  1029.52	   535.25	  5
 12	 D 	  1144.55	   448.22	  4
 13	 T 	  1245.60	   333.19	  3
 14	 G 	  1302.62	   232.14	  2
 15	 R 	        -  	   175.12	  1  _
+2 charge
		      B   	     Y
  1	 G 	    29.52	        -  	 15
  2	 L 	    86.06	   710.36	 14
  3	 D 	   143.57	   653.82	 13
  4	 T 	   194.10	   596.30	 12
  5	 S 	   237.61	   545.78	 11
  6	 L 	   294.16	   502.26	 10
  7	 G 	   322.67	   445.72	  9
  8	 A 	   358.18	   417.21	  8
  9	 V 	   407.72	   381.69	  7
 10	 Q 	   471.75	   332.16	  6
 11	 S 	   515.26	   268.13	  5
 12	 D 	   572.78	   224.61	  4
 13	 T 	   623.30	   167.10	  3
 14	 G 	   651.81	   116.57	  2
 15	 R 	        -  	    88.06	  1  _
```

**145. IPI00295414 Collagen alpha-1(XV) chain**

| Sequence | Charge | M+H+ | XCorr | DeltaCn |
| --- | --- | --- | --- | --- |
| R.FTGSLQQLTVHPDPR.T | 2 | 1696.888 | 2.876 | 0.496 |

+1 charge

```
		      B   	     Y
  1	 F 	   148.08	        -  	 15
  2	 T 	   249.12	  1548.81	 14
  3	 G 	   306.14	  1447.77	 13
  4	 S 	   393.18	  1390.74	 12
  5	 L 	   506.26	  1303.71	 11
  6	 Q 	   634.32	  1190.63	 10
  7	 Q 	   762.38	  1062.57	  9
  8	 L 	   875.46	   934.51	  8
  9	 T 	   976.51	   821.43	  7
 10	 V 	  1075.58	   720.38	  6
 11	 H 	  1212.64	   621.31	  5
 12	 P 	  1309.69	   484.25	  4
 13	 D 	  1424.72	   387.20	  3
 14	 P 	  1521.77	   272.17	  2
 15	 R 	        -  	   175.12	  1  _
+2 charge
		      B   	     Y
  1	 F 	    74.54	        -  	 15
  2	 T 	   125.07	   774.91	 14
  3	 G 	   153.58	   724.39	 13
  4	 S 	   197.09	   695.88	 12
  5	 L 	   253.63	   652.36	 11
  6	 Q 	   317.66	   595.82	 10
  7	 Q 	   381.69	   531.79	  9
  8	 L 	   438.23	   467.76	  8
  9	 T 	   488.76	   411.22	  7
 10	 V 	   538.29	   360.69	  6
 11	 H 	   606.82	   311.16	  5
 12	 P 	   655.35	   242.63	  4
 13	 D 	   712.86	   194.10	  3
 14	 P 	   761.39	   136.59	  2
 15	 R 	        -  	    88.06	  1  _
```

**146. IPI00910790 cDNA FLJ54587, highly similar to Splicing factor, arginine/serine-rich 16**

| Sequence | Charge | M+H+ | XCorr | DeltaCn |
| --- | --- | --- | --- | --- |
| K.ASIGYTYEDSTVAEVEK.A | 2 | 1862.969 | 3.169 | 0.262 |

+1 charge

```
		      B   	     Y
  1	 A 	    72.04	        -  	 17
  2	 S 	   159.08	  1790.83	 16
  3	 I 	   272.16	  1703.80	 15
  4	 G 	   329.18	  1590.72	 14
  5	 Y 	   492.25	  1533.70	 13
  6	 T 	   593.29	  1370.63	 12
  7	 Y 	   756.36	  1269.58	 11
  8	 E 	   885.40	  1106.52	 10
  9	 D 	  1000.43	   977.48	  9
 10	 S 	  1087.46	   862.45	  8
 11	 T 	  1188.51	   775.42	  7
 12	 V 	  1287.57	   674.37	  6
 13	 A 	  1358.61	   575.30	  5
 14	 E 	  1487.65	   504.27	  4
 15	 V 	  1586.72	   375.22	  3
 16	 E 	  1715.76	   276.16	  2
 17	 K 	        -  	   147.11	  1  _
+2 charge
		      B   	     Y
  1	 A 	    36.53	        -  	 17
  2	 S 	    80.04	   895.92	 16
  3	 I 	   136.58	   852.40	 15
  4	 G 	   165.09	   795.86	 14
  5	 Y 	   246.63	   767.35	 13
  6	 T 	   297.15	   685.82	 12
  7	 Y 	   378.68	   635.30	 11
  8	 E 	   443.20	   553.76	 10
  9	 D 	   500.72	   489.24	  9
 10	 S 	   544.23	   431.73	  8
 11	 T 	   594.76	   388.21	  7
 12	 V 	   644.29	   337.69	  6
 13	 A 	   679.81	   288.16	  5
 14	 E 	   744.33	   252.64	  4
 15	 V 	   793.86	   188.12	  3
 16	 E 	   858.39	   138.58	  2
 17	 K 	        -  	    74.06	  1  _
```

**147. IPI00029723 Follistatin-related protein 1**

| Sequence | Charge | M+H+ | XCorr | DeltaCn |
| --- | --- | --- | --- | --- |
| K.NFDNGDSRLDSSEFLK.F | 2 | 1844.917 | 2.989 | 0.502 |

+1 charge

```
		      B   	     Y
  1	 N 	   115.05	        -  	 16
  2	 F 	   262.12	  1729.80	 15
  3	 D 	   377.15	  1582.73	 14
  4	 N 	   491.19	  1467.71	 13
  5	 G 	   548.21	  1353.66	 12
  6	 D 	   663.24	  1296.64	 11
  7	 S 	   750.27	  1181.62	 10
  8	 R 	   906.37	  1094.58	  9
  9	 L 	  1019.45	   938.48	  8
 10	 D 	  1134.48	   825.40	  7
 11	 S 	  1221.51	   710.37	  6
 12	 S 	  1308.55	   623.34	  5
 13	 E 	  1437.59	   536.31	  4
 14	 F 	  1584.66	   407.27	  3
 15	 L 	  1697.74	   260.20	  2
 16	 K 	        -  	   147.11	  1  _
+2 charge
		      B   	     Y
  1	 N 	    58.03	        -  	 16
  2	 F 	   131.56	   865.41	 15
  3	 D 	   189.08	   791.87	 14
  4	 N 	   246.10	   734.36	 13
  5	 G 	   274.61	   677.34	 12
  6	 D 	   332.12	   648.83	 11
  7	 S 	   375.64	   591.31	 10
  8	 R 	   453.69	   547.80	  9
  9	 L 	   510.23	   469.75	  8
 10	 D 	   567.74	   413.20	  7
 11	 S 	   611.26	   355.69	  6
 12	 S 	   654.78	   312.17	  5
 13	 E 	   719.30	   268.66	  4
 14	 F 	   792.83	   204.14	  3
 15	 L 	   849.37	   130.60	  2
 16	 K 	        -  	    74.06	  1  _
```

**148. IPI00169383 Phosphoglycerate kinase 1**

| Sequence | Charge | M+H+ | XCorr | DeltaCn |
| --- | --- | --- | --- | --- |
| K.AAVPSIKFCLDNGAK.S | 2 | 1591.827 | 2.989 | 0.296 |

+1 charge

```
		      B   	     Y
  1	 A 	    72.04	        -  	 15
  2	 A 	   143.08	  1519.93	 14
  3	 V 	   242.15	  1448.89	 13
  4	 P 	   339.20	  1349.82	 12
  5	 S 	   426.23	  1252.77	 11
  6	 I 	   539.32	  1165.74	 10
  7	 K 	   667.41	  1052.65	  9
  8	 F 	   814.48	   924.56	  8
  9	 C 	   974.65	   777.49	  7
 10	 L 	  1087.73	   617.33	  6
 11	 D 	  1202.76	   504.24	  5
 12	 N 	  1316.80	   389.21	  4
 13	 G 	  1373.82	   275.17	  3
 14	 A 	  1444.86	   218.15	  2
 15	 K 	        -  	   147.11	  1  _
+2 charge
		      B   	     Y
  1	 A 	    36.53	        -  	 15
  2	 A 	    72.04	   760.47	 14
  3	 V 	   121.58	   724.95	 13
  4	 P 	   170.10	   675.42	 12
  5	 S 	   213.62	   626.89	 11
  6	 I 	   270.16	   583.37	 10
  7	 K 	   334.21	   526.83	  9
  8	 F 	   407.74	   462.78	  8
  9	 C 	   487.83	   389.25	  7
 10	 L 	   544.37	   309.17	  6
 11	 D 	   601.88	   252.62	  5
 12	 N 	   658.90	   195.11	  4
 13	 G 	   687.42	   138.09	  3
 14	 A 	   722.93	   109.58	  2
 15	 K 	        -  	    74.06	  1  _
```

**149. IPI00783156 Bone morphogenetic protein receptor type-2**

| Sequence | Charge | M+H+ | XCorr | DeltaCn |
| --- | --- | --- | --- | --- |
| R.LCAFKDPYQQDLGIGESR.I | 2 | 2098.295 | 3.075 | 0.514 |

+1 charge

```
		      B   	     Y
  1	 L 	   114.09	        -  	 18
  2	 C 	   274.26	  1984.06	 17
  3	 A 	   345.29	  1823.89	 16
  4	 F 	   492.36	  1752.86	 15
  5	 K 	   620.46	  1605.79	 14
  6	 D 	   735.48	  1477.69	 13
  7	 P 	   832.54	  1362.66	 12
  8	 Y 	   995.60	  1265.61	 11
  9	 Q 	  1123.66	  1102.55	 10
 10	 Q 	  1251.72	   974.49	  9
 11	 D 	  1366.74	   846.43	  8
 12	 L 	  1479.83	   731.40	  7
 13	 G 	  1536.85	   618.32	  6
 14	 I 	  1649.93	   561.30	  5
 15	 G 	  1706.96	   448.22	  4
 16	 E 	  1836.00	   391.19	  3
 17	 S 	  1923.03	   262.15	  2
 18	 R 	        -  	   175.12	  1  _
+2 charge
		      B   	     Y
  1	 L 	    57.55	        -  	 18
  2	 C 	   137.63	   992.53	 17
  3	 A 	   173.15	   912.45	 16
  4	 F 	   246.68	   876.93	 15
  5	 K 	   310.73	   803.40	 14
  6	 D 	   368.25	   739.35	 13
  7	 P 	   416.77	   681.84	 12
  8	 Y 	   498.30	   633.31	 11
  9	 Q 	   562.33	   551.78	 10
 10	 Q 	   626.36	   487.75	  9
 11	 D 	   683.88	   423.72	  8
 12	 L 	   740.42	   366.21	  7
 13	 G 	   768.93	   309.66	  6
 14	 I 	   825.47	   281.15	  5
 15	 G 	   853.98	   224.61	  4
 16	 E 	   918.50	   196.10	  3
 17	 S 	   962.02	   131.58	  2
 18	 R 	        -  	    88.06	  1  _
```

**150. IPI00022417 Leucine-rich alpha-2-glycoprotein**

| Sequence | Charge | M+H+ | XCorr | DeltaCn |
| --- | --- | --- | --- | --- |
| K.LQELHLSSNGLESLSPEFLRPVPQLR.V | 3 | 2961.365 | 4.442 | 0.535 |

+1 charge

```
		      B   	     Y
  1	 L 	   114.09	        -  	 26
  2	 Q 	   242.15	  2846.52	 25
  3	 E 	   371.19	  2718.46	 24
  4	 L 	   484.28	  2589.41	 23
  5	 H 	   621.34	  2476.33	 22
  6	 L 	   734.42	  2339.27	 21
  7	 S 	   821.45	  2226.19	 20
  8	 S 	   908.48	  2139.16	 19
  9	 N 	  1022.53	  2052.12	 18
 10	 G 	  1079.55	  1938.08	 17
 11	 L 	  1192.63	  1881.06	 16
 12	 E 	  1321.67	  1767.98	 15
 13	 S 	  1408.71	  1638.93	 14
 14	 L 	  1521.79	  1551.90	 13
 15	 S 	  1608.82	  1438.82	 12
 16	 P 	  1705.88	  1351.78	 11
 17	 E 	  1834.92	  1254.73	 10
 18	 F 	  1981.99	  1125.69	  9
 19	 L 	  2095.07	   978.62	  8
 20	 R 	  2251.17	   865.54	  7
 21	 P 	  2348.22	   709.44	  6
 22	 V 	  2447.29	   612.38	  5
 23	 P 	  2544.35	   513.31	  4
 24	 Q 	  2672.40	   416.26	  3
 25	 L 	  2785.49	   288.20	  2
 26	 R 	        -  	   175.12	  1  _


+2 charge
		      B   	     Y
  1	 L 	    57.55	        -  	 26
  2	 Q 	   121.58	  1423.76	 25
  3	 E 	   186.10	  1359.73	 24
  4	 L 	   242.64	  1295.21	 23
  5	 H 	   311.17	  1238.67	 22
  6	 L 	   367.71	  1170.14	 21
  7	 S 	   411.23	  1113.60	 20
  8	 S 	   454.75	  1070.08	 19
  9	 N 	   511.77	  1026.57	 18
 10	 G 	   540.28	   969.54	 17
 11	 L 	   596.82	   941.03	 16
 12	 E 	   661.34	   884.49	 15
 13	 S 	   704.86	   819.97	 14
 14	 L 	   761.40	   776.45	 13
 15	 S 	   804.92	   719.91	 12
 16	 P 	   853.44	   676.40	 11
 17	 E 	   917.96	   627.87	 10
 18	 F 	   991.50	   563.35	  9
 19	 L 	  1048.04	   489.81	  8
 20	 R 	  1126.09	   433.27	  7
 21	 P 	  1174.62	   355.22	  6
 22	 V 	  1224.15	   306.70	  5
 23	 P 	  1272.68	   257.16	  4
 24	 Q 	  1336.71	   208.63	  3
 25	 L 	  1393.25	   144.61	  2
 26	 R 	        -  	    88.06	  1  _


+3 charge
		      B   	     Y
  1	 L 	    38.70	        -  	 26
  2	 Q 	    81.39	   949.51	 25
  3	 E 	   124.40	   906.82	 24
  4	 L 	   162.10	   863.81	 23
  5	 H 	   207.78	   826.12	 22
  6	 L 	   245.48	   780.43	 21
  7	 S 	   274.49	   742.73	 20
  8	 S 	   303.50	   713.72	 19
  9	 N 	   341.51	   684.71	 18
 10	 G 	   360.52	   646.70	 17
 11	 L 	   398.22	   627.69	 16
 12	 E 	   441.23	   590.00	 15
 13	 S 	   470.24	   546.98	 14
 14	 L 	   507.94	   517.97	 13
 15	 S 	   536.95	   480.28	 12
 16	 P 	   569.30	   451.27	 11
 17	 E 	   612.31	   418.92	 10
 18	 F 	   661.33	   375.90	  9
 19	 L 	   699.03	   326.88	  8
 20	 R 	   751.06	   289.18	  7
 21	 P 	   783.41	   237.15	  6
 22	 V 	   816.44	   204.80	  5
 23	 P 	   848.79	   171.78	  4
 24	 Q 	   891.47	   139.43	  3
 25	 L 	   929.17	    96.74	  2
 26	 R 	        -  	    59.04	  1  _
```

**151. IPI00940353 Isoform 1 of Gamma-glutamyltransferase 6**

| Sequence | Charge | M+H+ | XCorr | DeltaCn |
| --- | --- | --- | --- | --- |
| R.LLVGPTTLAQEGFLVDTPLAR.A | 2 | 2212.574 | 4.559 | 0.54 |

+1 charge

```
		      B   	     Y
  1	 L 	   114.09	        -  	 21
  2	 L 	   227.18	  2098.15	 20
  3	 V 	   326.24	  1985.07	 19
  4	 G 	   383.27	  1886.00	 18
  5	 P 	   480.32	  1828.98	 17
  6	 T 	   581.37	  1731.93	 16
  7	 T 	   682.41	  1630.88	 15
  8	 L 	   795.50	  1529.83	 14
  9	 A 	   866.53	  1416.75	 13
 10	 Q 	   994.59	  1345.71	 12
 11	 E 	  1123.64	  1217.65	 11
 12	 G 	  1180.66	  1088.61	 10
 13	 F 	  1327.73	  1031.59	  9
 14	 L 	  1440.81	   884.52	  8
 15	 V 	  1539.88	   771.44	  7
 16	 D 	  1654.91	   672.37	  6
 17	 T 	  1755.95	   557.34	  5
 18	 P 	  1853.01	   456.29	  4
 19	 L 	  1966.09	   359.24	  3
 20	 A 	  2037.13	   246.16	  2
 21	 R 	        -  	   175.12	  1  _
+2 charge
		      B   	     Y
  1	 L 	    57.55	        -  	 21
  2	 L 	   114.09	  1049.58	 20
  3	 V 	   163.63	   993.04	 19
  4	 G 	   192.14	   943.50	 18
  5	 P 	   240.66	   914.99	 17
  6	 T 	   291.19	   866.47	 16
  7	 T 	   341.71	   815.94	 15
  8	 L 	   398.25	   765.42	 14
  9	 A 	   433.77	   708.88	 13
 10	 Q 	   497.80	   673.36	 12
 11	 E 	   562.32	   609.33	 11
 12	 G 	   590.83	   544.81	 10
 13	 F 	   664.37	   516.30	  9
 14	 L 	   720.91	   442.76	  8
 15	 V 	   770.44	   386.22	  7
 16	 D 	   827.96	   336.69	  6
 17	 T 	   878.48	   279.17	  5
 18	 P 	   927.01	   228.65	  4
 19	 L 	   983.55	   180.12	  3
 20	 A 	  1019.07	   123.58	  2
 21	 R 	        -  	    88.06	  1  _
```

**152. IPI00176125 Isoform 1 of Collagen alpha-5(VI) chain**

| Sequence | Charge | M+H+ | XCorr | DeltaCn |
| --- | --- | --- | --- | --- |
| K.GDIGNPGIPGGPGPKGFR.G | 2 | 1693.888 | 2.938 | 0.342 |

+1 charge

```
		      B   	     Y
  1	 G 	    58.03	        -  	 18
  2	 D 	   173.06	  1635.86	 17
  3	 I 	   286.14	  1520.83	 16
  4	 G 	   343.16	  1407.75	 15
  5	 N 	   457.20	  1350.73	 14
  6	 P 	   554.26	  1236.68	 13
  7	 G 	   611.28	  1139.63	 12
  8	 I 	   724.36	  1082.61	 11
  9	 P 	   821.42	   969.53	 10
 10	 G 	   878.44	   872.47	  9
 11	 G 	   935.46	   815.45	  8
 12	 P 	  1032.51	   758.43	  7
 13	 G 	  1089.53	   661.38	  6
 14	 P 	  1186.59	   604.36	  5
 15	 K 	  1314.68	   507.30	  4
 16	 G 	  1371.70	   379.21	  3
 17	 F 	  1518.77	   322.19	  2
 18	 R 	        -  	   175.12	  1  _
+2 charge
		      B   	     Y
  1	 G 	    29.52	        -  	 18
  2	 D 	    87.03	   818.43	 17
  3	 I 	   143.57	   760.92	 16
  4	 G 	   172.08	   704.38	 15
  5	 N 	   229.11	   675.87	 14
  6	 P 	   277.63	   618.85	 13
  7	 G 	   306.14	   570.32	 12
  8	 I 	   362.68	   541.81	 11
  9	 P 	   411.21	   485.27	 10
 10	 G 	   439.72	   436.74	  9
 11	 G 	   468.23	   408.23	  8
 12	 P 	   516.76	   379.72	  7
 13	 G 	   545.27	   331.19	  6
 14	 P 	   593.80	   302.68	  5
 15	 K 	   657.84	   254.16	  4
 16	 G 	   686.35	   190.11	  3
 17	 F 	   759.89	   161.60	  2
 18	 R 	        -  	    88.06	  1  _
```

**153. IPI00056478 Isoform 1 of Immunoglobulin superfamily member 8**

| Sequence | Charge | M+H+ | XCorr | DeltaCn |
| --- | --- | --- | --- | --- |
| R.LQAQDAGIYECHTPSTDTR.Y | 3 | 2164.269 | 3.616 | 0.414 |

+1 charge

```
		      B   	     Y
  1	 L 	   114.09	        -  	 19
  2	 Q 	   242.15	  2050.03	 18
  3	 A 	   313.19	  1921.97	 17
  4	 Q 	   441.25	  1850.93	 16
  5	 D 	   556.27	  1722.87	 15
  6	 A 	   627.31	  1607.85	 14
  7	 G 	   684.33	  1536.81	 13
  8	 I 	   797.42	  1479.79	 12
  9	 Y 	   960.48	  1366.70	 11
 10	 E 	  1089.52	  1203.64	 10
 11	 C 	  1249.69	  1074.60	  9
 12	 H 	  1386.75	   914.43	  8
 13	 T 	  1487.79	   777.37	  7
 14	 P 	  1584.85	   676.33	  6
 15	 S 	  1671.88	   579.27	  5
 16	 T 	  1772.93	   492.24	  4
 17	 D 	  1887.95	   391.19	  3
 18	 T 	  1989.00	   276.17	  2
 19	 R 	        -  	   175.12	  1  _
+2 charge
		      B   	     Y
  1	 L 	    57.55	        -  	 19
  2	 Q 	   121.58	  1025.52	 18
  3	 A 	   157.10	   961.49	 17
  4	 Q 	   221.13	   925.97	 16
  5	 D 	   278.64	   861.94	 15
  6	 A 	   314.16	   804.43	 14
  7	 G 	   342.67	   768.91	 13
  8	 I 	   399.21	   740.40	 12
  9	 Y 	   480.74	   683.86	 11
 10	 E 	   545.26	   602.32	 10
 11	 C 	   625.35	   537.80	  9
 12	 H 	   693.88	   457.72	  8
 13	 T 	   744.40	   389.19	  7
 14	 P 	   792.93	   338.67	  6
 15	 S 	   836.44	   290.14	  5
 16	 T 	   886.97	   246.62	  4
 17	 D 	   944.48	   196.10	  3
 18	 T 	   995.00	   138.59	  2
 19	 R 	        -  	    88.06	  1  _


+3 charge
		      B   	     Y
  1	 L 	    38.70	        -  	 19
  2	 Q 	    81.39	   684.01	 18
  3	 A 	   105.07	   641.33	 17
  4	 Q 	   147.75	   617.65	 16
  5	 D 	   186.10	   574.96	 15
  6	 A 	   209.77	   536.62	 14
  7	 G 	   228.78	   512.94	 13
  8	 I 	   266.48	   493.93	 12
  9	 Y 	   320.83	   456.24	 11
 10	 E 	   363.85	   401.89	 10
 11	 C 	   417.23	   358.87	  9
 12	 H 	   462.92	   305.48	  8
 13	 T 	   496.60	   259.80	  7
 14	 P 	   528.95	   226.11	  6
 15	 S 	   557.96	   193.76	  5
 16	 T 	   591.65	   164.75	  4
 17	 D 	   629.99	   131.07	  3
 18	 T 	   663.67	    92.73	  2
 19	 R 	        -  	    59.04	  1  _
```

**154. IPI00010676 Isoform 1 of Urokinase plasminogen activator surface receptor**

| Sequence | Charge | M+H+ | XCorr | DeltaCn |
| --- | --- | --- | --- | --- |
| R.VEECALGQDLCR.T | 2 | 1450.564 | 3.059 | 0.445 |

+1 charge

```
		      B   	     Y
  1	 V 	   100.08	        -  	 12
  2	 E 	   229.12	  1350.85	 11
  3	 E 	   358.16	  1221.80	 10
  4	 C 	   518.33	  1092.76	  9
  5	 A 	   589.36	   932.60	  8
  6	 L 	   702.45	   861.56	  7
  7	 G 	   759.47	   748.48	  6
  8	 Q 	   887.53	   691.45	  5
  9	 D 	  1002.55	   563.40	  4
 10	 L 	  1115.64	   448.37	  3
 11	 C 	  1275.80	   335.28	  2
 12	 R 	        -  	   175.12	  1  _
+2 charge
		      B   	     Y
  1	 V 	    50.54	        -  	 12
  2	 E 	   115.06	   675.93	 11
  3	 E 	   179.58	   611.41	 10
  4	 C 	   259.67	   546.88	  9
  5	 A 	   295.19	   466.80	  8
  6	 L 	   351.73	   431.28	  7
  7	 G 	   380.24	   374.74	  6
  8	 Q 	   444.27	   346.23	  5
  9	 D 	   501.78	   282.20	  4
 10	 L 	   558.32	   224.69	  3
 11	 C 	   638.41	   168.15	  2
 12	 R 	        -  	    88.06	  1  _
```

**155. IPI00298388 Isoform 1 of Phosphoinositide-3-kinase-interacting protein 1**

| Sequence | Charge | M+H+ | XCorr | DeltaCn |
| --- | --- | --- | --- | --- |
| R.SEAAAVQPVIGISQR.V | 2 | 1526.72 | 4.669 | 0.568 |

+1 charge

```
		      B   	     Y
  1	 S 	    88.04	        -  	 15
  2	 E 	   217.08	  1438.80	 14
  3	 A 	   288.12	  1309.76	 13
  4	 A 	   359.16	  1238.72	 12
  5	 A 	   430.19	  1167.68	 11
  6	 V 	   529.26	  1096.65	 10
  7	 Q 	   657.32	   997.58	  9
  8	 P 	   754.37	   869.52	  8
  9	 V 	   853.44	   772.47	  7
 10	 I 	   966.53	   673.40	  6
 11	 G 	  1023.55	   560.32	  5
 12	 I 	  1136.63	   503.29	  4
 13	 S 	  1223.66	   390.21	  3
 14	 Q 	  1351.72	   303.18	  2
 15	 R 	        -  	   175.12	  1  _
+2 charge
		      B   	     Y
  1	 S 	    44.52	        -  	 15
  2	 E 	   109.04	   719.90	 14
  3	 A 	   144.56	   655.38	 13
  4	 A 	   180.08	   619.86	 12
  5	 A 	   215.60	   584.35	 11
  6	 V 	   265.13	   548.83	 10
  7	 Q 	   329.16	   499.29	  9
  8	 P 	   377.69	   435.26	  8
  9	 V 	   427.22	   386.74	  7
 10	 I 	   483.77	   337.20	  6
 11	 G 	   512.28	   280.66	  5
 12	 I 	   568.82	   252.15	  4
 13	 S 	   612.34	   195.61	  3
 14	 Q 	   676.36	   152.09	  2
 15	 R 	        -  	    88.06	  1  _
```

**156. IPI00102165 Isoform 2 of Histone H2A.J**

| Sequence | Charge | M+H+ | XCorr | DeltaCn |
| --- | --- | --- | --- | --- |
| R.AGLQFPVGR.I | 2 | 945.1002 | 3.209 | 0.263 |

+1 charge

```
		      B   	     Y
  1	 A 	    72.04	        -  	  9
  2	 G 	   129.07	   873.49	  8
  3	 L 	   242.15	   816.47	  7
  4	 Q 	   370.21	   703.39	  6
  5	 F 	   517.28	   575.33	  5
  6	 P 	   614.33	   428.26	  4
  7	 V 	   713.40	   331.21	  3
  8	 G 	   770.42	   232.14	  2
  9	 R 	        -  	   175.12	  1  _
+2 charge
		      B   	     Y
  1	 A 	    36.53	        -  	  9
  2	 G 	    65.04	   437.25	  8
  3	 L 	   121.58	   408.74	  7
  4	 Q 	   185.61	   352.20	  6
  5	 F 	   259.14	   288.17	  5
  6	 P 	   307.67	   214.63	  4
  7	 V 	   357.20	   166.11	  3
  8	 G 	   385.71	   116.57	  2
  9	 R 	        -  	    88.06	  1  _
```

**157. IPI00329688 Protein YIPF3**

| Sequence | Charge | M+H+ | XCorr | DeltaCn |
| --- | --- | --- | --- | --- |
| R.DIPAMLPAAR.L | 1 | 1055.276 | 2.399 | 0.435 |

+1 charge

```
		      B   	     Y
  1	 D 	   116.03	        -  	 10
  2	 I 	   229.12	   939.54	  9
  3	 P 	   326.17	   826.46	  8
  4	 A 	   397.21	   729.41	  7
  5	 M 	   528.25	   658.37	  6
  6	 L 	   641.33	   527.33	  5
  7	 P 	   738.39	   414.25	  4
  8	 A 	   809.42	   317.19	  3
  9	 A 	   880.46	   246.16	  2
 10	 R 	        -  	   175.12	  1  _
```

**158. IPI00387113 Ig kappa chain V-III region B6**

| Sequence | Charge | M+H+ | XCorr | DeltaCn |
| --- | --- | --- | --- | --- |
| - .ZIVLTZSPGTLSLSPGZR.A | 2 | 1884.616 | 4.472 | 0.587 |

+1 charge

```
		      B   	     Y
  1	 Z 	   129.56	        -  	 18
  2	 I 	   242.64	  1754.96	 17
  3	 V 	   341.71	  1641.88	 16
  4	 L 	   454.79	  1542.81	 15
  5	 T 	   555.84	  1429.73	 14
  6	 Z 	   684.39	  1328.68	 13
  7	 S 	   771.42	  1200.13	 12
  8	 P 	   868.48	  1113.10	 11
  9	 G 	   925.50	  1016.05	 10
 10	 T 	  1026.55	   959.02	  9
 11	 L 	  1139.63	   857.98	  8
 12	 S 	  1226.66	   744.89	  7
 13	 L 	  1339.75	   657.86	  6
 14	 S 	  1426.78	   544.78	  5
 15	 P 	  1523.83	   457.74	  4
 16	 G 	  1580.85	   360.69	  3
 17	 Z 	  1709.40	   303.67	  2
 18	 R 	        -  	   175.12	  1  _
+2 charge
		      B   	     Y
  1	 Z 	    65.28	        -  	 18
  2	 I 	   121.82	   877.99	 17
  3	 V 	   171.36	   821.44	 16
  4	 L 	   227.90	   771.91	 15
  5	 T 	   278.42	   715.37	 14
  6	 Z 	   342.70	   664.84	 13
  7	 S 	   386.22	   600.57	 12
  8	 P 	   434.74	   557.05	 11
  9	 G 	   463.25	   508.53	 10
 10	 T 	   513.78	   480.02	  9
 11	 L 	   570.32	   429.49	  8
 12	 S 	   613.83	   372.95	  7
 13	 L 	   670.38	   329.43	  6
 14	 S 	   713.89	   272.89	  5
 15	 P 	   762.42	   229.38	  4
 16	 G 	   790.93	   180.85	  3
 17	 Z 	   855.21	   152.34	  2
 18	 R 	        -  	    88.06	  1  _
```

**159. IPI00019641 Pepsin A**

| Sequence | Charge | M+H+ | XCorr | DeltaCn |
| --- | --- | --- | --- | --- |
| R.GLLKDFLK.K | 2 | 934.1573 | 2.954 | 0.246 |

+1 charge

```
		      B   	     Y
  1	 G 	    58.03	        -  	  8
  2	 L 	   171.11	   876.56	  7
  3	 L 	   284.20	   763.47	  6
  4	 K 	   412.29	   650.39	  5
  5	 D 	   527.32	   522.29	  4
  6	 F 	   674.39	   407.27	  3
  7	 L 	   787.47	   260.20	  2
  8	 K 	        -  	   147.11	  1  _
+2 charge
		      B   	     Y
  1	 G 	    29.52	        -  	  8
  2	 L 	    86.06	   438.78	  7
  3	 L 	   142.60	   382.24	  6
  4	 K 	   206.65	   325.70	  5
  5	 D 	   264.16	   261.65	  4
  6	 F 	   337.70	   204.14	  3
  7	 L 	   394.24	   130.60	  2
  8	 K 	        -  	    74.06	  1  _
```

**160. IPI00011899 BMP and activin membrane-bound inhibitor homolog**

| Sequence | Charge | M+H+ | XCorr | DeltaCn |
| --- | --- | --- | --- | --- |
| R.LLDPQNSNSPLTHGCLDSLASTTDICQAK.Q | 3 | 3158.408 | 3.304 | 0.337 |

+1 charge

```
		      B   	     Y
  1	 L 	   114.09	        -  	 29
  2	 L 	   227.18	  3043.68	 28
  3	 D 	   342.20	  2930.59	 27
  4	 P 	   439.26	  2815.57	 26
  5	 Q 	   567.31	  2718.51	 25
  6	 N 	   681.36	  2590.46	 24
  7	 S 	   768.39	  2476.41	 23
  8	 N 	   882.43	  2389.38	 22
  9	 S 	   969.46	  2275.34	 21
 10	 P 	  1066.52	  2188.31	 20
 11	 L 	  1179.60	  2091.25	 19
 12	 T 	  1280.65	  1978.17	 18
 13	 H 	  1417.71	  1877.12	 17
 14	 G 	  1474.73	  1740.06	 16
 15	 C 	  1634.89	  1683.04	 15
 16	 L 	  1747.98	  1522.88	 14
 17	 D 	  1863.00	  1409.79	 13
 18	 S 	  1950.04	  1294.77	 12
 19	 L 	  2063.12	  1207.73	 11
 20	 A 	  2134.16	  1094.65	 10
 21	 S 	  2221.19	  1023.61	  9
 22	 T 	  2322.24	   936.58	  8
 23	 T 	  2423.29	   835.53	  7
 24	 D 	  2538.31	   734.48	  6
 25	 I 	  2651.40	   619.46	  5
 26	 C 	  2811.56	   506.37	  4
 27	 Q 	  2939.62	   346.21	  3
 28	 A 	  3010.66	   218.15	  2
 29	 K 	        -  	   147.11	  1  _


+2 charge
		      B   	     Y
  1	 L 	    57.55	        -  	 29
  2	 L 	   114.09	  1522.34	 28
  3	 D 	   171.60	  1465.80	 27
  4	 P 	   220.13	  1408.29	 26
  5	 Q 	   284.16	  1359.76	 25
  6	 N 	   341.18	  1295.73	 24
  7	 S 	   384.70	  1238.71	 23
  8	 N 	   441.72	  1195.19	 22
  9	 S 	   485.24	  1138.17	 21
 10	 P 	   533.76	  1094.66	 20
 11	 L 	   590.30	  1046.13	 19
 12	 T 	   640.83	   989.59	 18
 13	 H 	   709.36	   939.06	 17
 14	 G 	   737.87	   870.54	 16
 15	 C 	   817.95	   842.02	 15
 16	 L 	   874.49	   761.94	 14
 17	 D 	   932.01	   705.40	 13
 18	 S 	   975.52	   647.89	 12
 19	 L 	  1032.06	   604.37	 11
 20	 A 	  1067.58	   547.83	 10
 21	 S 	  1111.10	   512.31	  9
 22	 T 	  1161.62	   468.79	  8
 23	 T 	  1212.15	   418.27	  7
 24	 D 	  1269.66	   367.75	  6
 25	 I 	  1326.20	   310.23	  5
 26	 C 	  1406.28	   253.69	  4
 27	 Q 	  1470.31	   173.61	  3
 28	 A 	  1505.83	   109.58	  2
 29	 K 	        -  	    74.06	  1  _


+3 charge
		      B   	     Y
  1	 L 	    38.70	        -  	 29
  2	 L 	    76.40	  1015.23	 28
  3	 D 	   114.74	   977.54	 27
  4	 P 	   147.09	   939.19	 26
  5	 Q 	   189.78	   906.84	 25
  6	 N 	   227.79	   864.16	 24
  7	 S 	   256.80	   826.14	 23
  8	 N 	   294.82	   797.13	 22
  9	 S 	   323.83	   759.12	 21
 10	 P 	   356.18	   730.11	 20
 11	 L 	   393.87	   697.76	 19
 12	 T 	   427.55	   660.06	 18
 13	 H 	   473.24	   626.38	 17
 14	 G 	   492.25	   580.69	 16
 15	 C 	   545.64	   561.69	 15
 16	 L 	   583.33	   508.30	 14
 17	 D 	   621.67	   470.60	 13
 18	 S 	   650.68	   432.26	 12
 19	 L 	   688.38	   403.25	 11
 20	 A 	   712.06	   365.55	 10
 21	 S 	   741.07	   341.88	  9
 22	 T 	   774.75	   312.86	  8
 23	 T 	   808.43	   279.18	  7
 24	 D 	   846.78	   245.50	  6
 25	 I 	   884.47	   207.16	  5
 26	 C 	   937.86	   169.46	  4
 27	 Q 	   980.54	   116.07	  3
 28	 A 	  1004.22	    73.39	  2
 29	 K 	        -  	    49.71	  1  _
```

**161. IPI00395826 Serine protease hepsin**

| Sequence | Charge | M+H+ | XCorr | DeltaCn |
| --- | --- | --- | --- | --- |
| R.VAGLSCEEMGFLR.A | 2 | 1469.682 | 3.83 | 0.517 |

+1 charge

```
		      B   	     Y
  1	 V 	   100.08	        -  	 13
  2	 A 	   171.11	  1369.76	 12
  3	 G 	   228.13	  1298.72	 11
  4	 L 	   341.22	  1241.70	 10
  5	 S 	   428.25	  1128.62	  9
  6	 C 	   588.42	  1041.58	  8
  7	 E 	   717.46	   881.42	  7
  8	 E 	   846.50	   752.38	  6
  9	 M 	   977.54	   623.33	  5
 10	 G 	  1034.56	   492.29	  4
 11	 F 	  1181.63	   435.27	  3
 12	 L 	  1294.72	   288.20	  2
 13	 R 	        -  	   175.12	  1  _
+2 charge
		      B   	     Y
  1	 V 	    50.54	        -  	 13
  2	 A 	    86.06	   685.38	 12
  3	 G 	   114.57	   649.86	 11
  4	 L 	   171.11	   621.35	 10
  5	 S 	   214.63	   564.81	  9
  6	 C 	   294.71	   521.30	  8
  7	 E 	   359.23	   441.21	  7
  8	 E 	   423.75	   376.69	  6
  9	 M 	   489.27	   312.17	  5
 10	 G 	   517.79	   246.65	  4
 11	 F 	   591.32	   218.14	  3
 12	 L 	   647.86	   144.61	  2
 13	 R 	        -  	    88.06	  1  _
```

**162. IPI00387120 Ig kappa chain V-IV region Len**

| Sequence | Charge | M+H+ | XCorr | DeltaCn |
| --- | --- | --- | --- | --- |
| - .DIVMTQSPDSLAVSLGER.A | 2 | 1919.147 | 5.405 | 0.547 |

+1 charge

```
		      B   	     Y
  1	 D 	   116.03	        -  	 18
  2	 I 	   229.12	  1802.93	 17
  3	 V 	   328.19	  1689.85	 16
  4	 M 	   459.23	  1590.78	 15
  5	 T 	   560.27	  1459.74	 14
  6	 Q 	   688.33	  1358.69	 13
  7	 S 	   775.37	  1230.63	 12
  8	 P 	   872.42	  1143.60	 11
  9	 D 	   987.45	  1046.55	 10
 10	 S 	  1074.48	   931.52	  9
 11	 L 	  1187.56	   844.49	  8
 12	 A 	  1258.60	   731.40	  7
 13	 V 	  1357.67	   660.37	  6
 14	 S 	  1444.70	   561.30	  5
 15	 L 	  1557.78	   474.27	  4
 16	 G 	  1614.80	   361.18	  3
 17	 E 	  1743.85	   304.16	  2
 18	 R 	        -  	   175.12	  1  _
+2 charge
		      B   	     Y
  1	 D 	    58.52	        -  	 18
  2	 I 	   115.06	   901.97	 17
  3	 V 	   164.60	   845.43	 16
  4	 M 	   230.12	   795.89	 15
  5	 T 	   280.64	   730.37	 14
  6	 Q 	   344.67	   679.85	 13
  7	 S 	   388.19	   615.82	 12
  8	 P 	   436.71	   572.30	 11
  9	 D 	   494.23	   523.78	 10
 10	 S 	   537.74	   466.26	  9
 11	 L 	   594.28	   422.75	  8
 12	 A 	   629.80	   366.21	  7
 13	 V 	   679.34	   330.69	  6
 14	 S 	   722.85	   281.15	  5
 15	 L 	   779.40	   237.64	  4
 16	 G 	   807.91	   181.10	  3
 17	 E 	   872.43	   152.58	  2
 18	 R 	        -  	    88.06	  1  _
```

**163. IPI00217466 Histone H1.3**

| Sequence | Charge | M+H+ | XCorr | DeltaCn |
| --- | --- | --- | --- | --- |
| R.KASGPPVSELITK.A | 2 | 1327.552 | 3.911 | 0.342 |

+1 charge

```
		      B   	     Y
  1	 K 	   129.10	        -  	 13
  2	 A 	   200.14	  1198.67	 12
  3	 S 	   287.17	  1127.63	 11
  4	 G 	   344.19	  1040.60	 10
  5	 P 	   441.25	   983.58	  9
  6	 P 	   538.30	   886.52	  8
  7	 V 	   637.37	   789.47	  7
  8	 S 	   724.40	   690.40	  6
  9	 E 	   853.44	   603.37	  5
 10	 L 	   966.53	   474.33	  4
 11	 I 	  1079.61	   361.24	  3
 12	 T 	  1180.66	   248.16	  2
 13	 K 	        -  	   147.11	  1  _
+2 charge
		      B   	     Y
  1	 K 	    65.05	        -  	 13
  2	 A 	   100.57	   599.84	 12
  3	 S 	   144.09	   564.32	 11
  4	 G 	   172.60	   520.80	 10
  5	 P 	   221.13	   492.29	  9
  6	 P 	   269.65	   443.77	  8
  7	 V 	   319.19	   395.24	  7
  8	 S 	   362.70	   345.71	  6
  9	 E 	   427.22	   302.19	  5
 10	 L 	   483.77	   237.67	  4
 11	 I 	   540.31	   181.13	  3
 12	 T 	   590.83	   124.58	  2
 13	 K 	        -  	    74.06	  1  _
```

**164. IPI00021405 Isoform A of Lamin-A/C**

| Sequence | Charge | M+H+ | XCorr | DeltaCn |
| --- | --- | --- | --- | --- |
| R.VAVEEVDEEGKFVR.L | 2 | 1606.759 | 3.305 | 0.525 |

+1 charge

```
		      B   	     Y
  1	 V 	   100.08	        -  	 14
  2	 A 	   171.11	  1506.74	 13
  3	 V 	   270.18	  1435.71	 12
  4	 E 	   399.22	  1336.64	 11
  5	 E 	   528.27	  1207.60	 10
  6	 V 	   627.33	  1078.55	  9
  7	 D 	   742.36	   979.48	  8
  8	 E 	   871.40	   864.46	  7
  9	 E 	  1000.45	   735.41	  6
 10	 G 	  1057.47	   606.37	  5
 11	 K 	  1185.56	   549.35	  4
 12	 F 	  1332.63	   421.26	  3
 13	 V 	  1431.70	   274.19	  2
 14	 R 	        -  	   175.12	  1  _
+2 charge
		      B   	     Y
  1	 V 	    50.54	        -  	 14
  2	 A 	    86.06	   753.88	 13
  3	 V 	   135.59	   718.36	 12
  4	 E 	   200.12	   668.82	 11
  5	 E 	   264.64	   604.30	 10
  6	 V 	   314.17	   539.78	  9
  7	 D 	   371.68	   490.25	  8
  8	 E 	   436.21	   432.73	  7
  9	 E 	   500.73	   368.21	  6
 10	 G 	   529.24	   303.69	  5
 11	 K 	   593.29	   275.18	  4
 12	 F 	   666.82	   211.13	  3
 13	 V 	   716.35	   137.60	  2
 14	 R 	        -  	    88.06	  1  _
```

**165. IPI00066856 Ly6/PLAUR domain-containing protein 2**

| Sequence | Charge | M+H+ | XCorr | DeltaCn |
| --- | --- | --- | --- | --- |
| R.EIVYPFQGDSTVTK.S | 2 | 1584.751 | 3.288 | 0.485 |

+1 charge

```
		      B   	     Y
  1	 E 	   130.05	        -  	 14
  2	 I 	   243.13	  1454.75	 13
  3	 V 	   342.20	  1341.67	 12
  4	 Y 	   505.27	  1242.60	 11
  5	 P 	   602.32	  1079.54	 10
  6	 F 	   749.39	   982.48	  9
  7	 Q 	   877.45	   835.42	  8
  8	 G 	   934.47	   707.36	  7
  9	 D 	  1049.49	   650.34	  6
 10	 S 	  1136.53	   535.31	  5
 11	 T 	  1237.57	   448.28	  4
 12	 V 	  1336.64	   347.23	  3
 13	 T 	  1437.69	   248.16	  2
 14	 K 	        -  	   147.11	  1  _
+2 charge
		      B   	     Y
  1	 E 	    65.53	        -  	 14
  2	 I 	   122.07	   727.88	 13
  3	 V 	   171.60	   671.34	 12
  4	 Y 	   253.14	   621.80	 11
  5	 P 	   301.66	   540.27	 10
  6	 F 	   375.20	   491.75	  9
  7	 Q 	   439.23	   418.21	  8
  8	 G 	   467.74	   354.18	  7
  9	 D 	   525.25	   325.67	  6
 10	 S 	   568.77	   268.16	  5
 11	 T 	   619.29	   224.64	  4
 12	 V 	   668.82	   174.12	  3
 13	 T 	   719.35	   124.58	  2
 14	 K 	        -  	    74.06	  1  _
```

**166. IPI00031008 Isoform 1 of Tenascin**

| Sequence | Charge | M+H+ | XCorr | DeltaCn |
| --- | --- | --- | --- | --- |
| R.PSNFRNLEGR.R | 2 | 1190.294 | 2.916 | 0.236 |

+1 charge

```
		      B   	     Y
  1	 P 	    98.06	        -  	 10
  2	 S 	   185.09	  1092.55	  9
  3	 N 	   299.13	  1005.52	  8
  4	 F 	   446.20	   891.48	  7
  5	 R 	   602.30	   744.41	  6
  6	 N 	   716.35	   588.31	  5
  7	 L 	   829.43	   474.27	  4
  8	 E 	   958.47	   361.18	  3
  9	 G 	  1015.50	   232.14	  2
 10	 R 	        -  	   175.12	  1  _
+2 charge
		      B   	     Y
  1	 P 	    49.53	        -  	 10
  2	 S 	    93.05	   546.78	  9
  3	 N 	   150.07	   503.26	  8
  4	 F 	   223.61	   446.24	  7
  5	 R 	   301.66	   372.71	  6
  6	 N 	   358.68	   294.66	  5
  7	 L 	   415.22	   237.64	  4
  8	 E 	   479.74	   181.10	  3
  9	 G 	   508.25	   116.57	  2
 10	 R 	        -  	    88.06	  1  _
```

**167. IPI00023648 Immunoglobulin superfamily containing leucine-rich repeat protein**

| Sequence | Charge | M+H+ | XCorr | DeltaCn |
| --- | --- | --- | --- | --- |
| R.TVAAGALASLSHLK.S | 2 | 1339.566 | 3.663 | 0.43 |

+1 charge

```
		      B   	     Y
  1	 T 	   102.05	        -  	 14
  2	 V 	   201.12	  1237.73	 13
  3	 A 	   272.16	  1138.66	 12
  4	 A 	   343.20	  1067.62	 11
  5	 G 	   400.22	   996.58	 10
  6	 A 	   471.26	   939.56	  9
  7	 L 	   584.34	   868.53	  8
  8	 A 	   655.38	   755.44	  7
  9	 S 	   742.41	   684.40	  6
 10	 L 	   855.49	   597.37	  5
 11	 S 	   942.53	   484.29	  4
 12	 H 	  1079.58	   397.26	  3
 13	 L 	  1192.67	   260.20	  2
 14	 K 	        -  	   147.11	  1  _
+2 charge
		      B   	     Y
  1	 T 	    51.53	        -  	 14
  2	 V 	   101.07	   619.37	 13
  3	 A 	   136.58	   569.83	 12
  4	 A 	   172.10	   534.31	 11
  5	 G 	   200.61	   498.80	 10
  6	 A 	   236.13	   470.28	  9
  7	 L 	   292.67	   434.77	  8
  8	 A 	   328.19	   378.22	  7
  9	 S 	   371.71	   342.71	  6
 10	 L 	   428.25	   299.19	  5
 11	 S 	   471.77	   242.65	  4
 12	 H 	   540.30	   199.13	  3
 13	 L 	   596.84	   130.60	  2
 14	 K 	        -  	    74.06	  1  _
```

**168. IPI00013446 Prostate stem cell antigen**

| Sequence | Charge | M+H+ | XCorr | DeltaCn |
| --- | --- | --- | --- | --- |
| R.AVGLLTVISK.G | 2 | 1001.245 | 3.509 | 0.255 |

+1 charge

```
		      B   	     Y
  1	 A 	    72.04	        -  	 10
  2	 V 	   171.11	   929.60	  9
  3	 G 	   228.13	   830.53	  8
  4	 L 	   341.22	   773.51	  7
  5	 L 	   454.30	   660.43	  6
  6	 T 	   555.35	   547.34	  5
  7	 V 	   654.42	   446.30	  4
  8	 I 	   767.50	   347.23	  3
  9	 S 	   854.53	   234.14	  2
 10	 K 	        -  	   147.11	  1  _
+2 charge
		      B   	     Y
  1	 A 	    36.53	        -  	 10
  2	 V 	    86.06	   465.31	  9
  3	 G 	   114.57	   415.77	  8
  4	 L 	   171.11	   387.26	  7
  5	 L 	   227.65	   330.72	  6
  6	 T 	   278.18	   274.18	  5
  7	 V 	   327.71	   223.65	  4
  8	 I 	   384.25	   174.12	  3
  9	 S 	   427.77	   117.58	  2
 10	 K 	        -  	    74.06	  1  _
```

**169. IPI00296058 EGF-containing fibulin-like extracellular matrix protein 2**

| Sequence | Charge | M+H+ | XCorr | DeltaCn |
| --- | --- | --- | --- | --- |
| K.CINHYGGYLCLPR.S | 2 | 1623.824 | 4.121 | 0.494 |

+1 charge

```
		      B   	     Y
  1	 C 	   161.17	        -  	 13
  2	 I 	   274.26	  1462.86	 12
  3	 N 	   388.30	  1349.78	 11
  4	 H 	   525.36	  1235.73	 10
  5	 Y 	   688.42	  1098.67	  9
  6	 G 	   745.44	   935.61	  8
  7	 G 	   802.46	   878.59	  7
  8	 Y 	   965.53	   821.57	  6
  9	 L 	  1078.61	   658.51	  5
 10	 C 	  1238.78	   545.42	  4
 11	 L 	  1351.86	   385.26	  3
 12	 P 	  1448.91	   272.17	  2
 13	 R 	        -  	   175.12	  1  _
+2 charge
		      B   	     Y
  1	 C 	    81.09	        -  	 13
  2	 I 	   137.63	   731.93	 12
  3	 N 	   194.65	   675.39	 11
  4	 H 	   263.18	   618.37	 10
  5	 Y 	   344.71	   549.84	  9
  6	 G 	   373.23	   468.31	  8
  7	 G 	   401.74	   439.80	  7
  8	 Y 	   483.27	   411.29	  6
  9	 L 	   539.81	   329.76	  5
 10	 C 	   619.89	   273.21	  4
 11	 L 	   676.43	   193.13	  3
 12	 P 	   724.96	   136.59	  2
 13	 R 	        -  	    88.06	  1  _
```

**170. IPI00220644 Isoform M1 of Pyruvate kinase isozymes M1/M2**

| Sequence | Charge | M+H+ | XCorr | DeltaCn |
| --- | --- | --- | --- | --- |
| K.KGVNLPGAAVDLPAVSEKDIQDLK.F | 3 | 2478.827 | 4.333 | 0.499 |

+1 charge

```
		      B   	     Y
  1	 K 	   129.10	        -  	 24
  2	 G 	   186.12	  2349.27	 23
  3	 V 	   285.19	  2292.24	 22
  4	 N 	   399.24	  2193.18	 21
  5	 L 	   512.32	  2079.13	 20
  6	 P 	   609.37	  1966.05	 19
  7	 G 	   666.39	  1869.00	 18
  8	 A 	   737.43	  1811.97	 17
  9	 A 	   808.47	  1740.94	 16
 10	 V 	   907.54	  1669.90	 15
 11	 D 	  1022.56	  1570.83	 14
 12	 L 	  1135.65	  1455.81	 13
 13	 P 	  1232.70	  1342.72	 12
 14	 A 	  1303.74	  1245.67	 11
 15	 V 	  1402.81	  1174.63	 10
 16	 S 	  1489.84	  1075.56	  9
 17	 E 	  1618.88	   988.53	  8
 18	 K 	  1746.97	   859.49	  7
 19	 D 	  1862.00	   731.39	  6
 20	 I 	  1975.09	   616.37	  5
 21	 Q 	  2103.14	   503.28	  4
 22	 D 	  2218.17	   375.22	  3
 23	 L 	  2331.26	   260.20	  2
 24	 K 	        -  	   147.11	  1  _


+2 charge
		      B   	     Y
  1	 K 	    65.05	        -  	 24
  2	 G 	    93.57	  1175.14	 23
  3	 V 	   143.10	  1146.63	 22
  4	 N 	   200.12	  1097.09	 21
  5	 L 	   256.66	  1040.07	 20
  6	 P 	   305.19	   983.53	 19
  7	 G 	   333.70	   935.00	 18
  8	 A 	   369.22	   906.49	 17
  9	 A 	   404.74	   870.97	 16
 10	 V 	   454.27	   835.45	 15
 11	 D 	   511.79	   785.92	 14
 12	 L 	   568.33	   728.41	 13
 13	 P 	   616.85	   671.86	 12
 14	 A 	   652.37	   623.34	 11
 15	 V 	   701.91	   587.82	 10
 16	 S 	   745.42	   538.29	  9
 17	 E 	   809.94	   494.77	  8
 18	 K 	   873.99	   430.25	  7
 19	 D 	   931.50	   366.20	  6
 20	 I 	   988.05	   308.69	  5
 21	 Q 	  1052.08	   252.14	  4
 22	 D 	  1109.59	   188.12	  3
 23	 L 	  1166.13	   130.60	  2
 24	 K 	        -  	    74.06	  1  _


+3 charge
		      B   	     Y
  1	 K 	    43.71	        -  	 24
  2	 G 	    62.71	   783.76	 23
  3	 V 	    95.74	   764.75	 22
  4	 N 	   133.75	   731.73	 21
  5	 L 	   171.44	   693.72	 20
  6	 P 	   203.80	   656.02	 19
  7	 G 	   222.80	   623.67	 18
  8	 A 	   246.48	   604.66	 17
  9	 A 	   270.16	   580.98	 16
 10	 V 	   303.18	   557.31	 15
 11	 D 	   341.53	   524.28	 14
 12	 L 	   379.22	   485.94	 13
 13	 P 	   411.57	   448.25	 12
 14	 A 	   435.25	   415.89	 11
 15	 V 	   468.27	   392.22	 10
 16	 S 	   497.28	   359.19	  9
 17	 E 	   540.30	   330.18	  8
 18	 K 	   583.00	   287.17	  7
 19	 D 	   621.34	   244.47	  6
 20	 I 	   659.03	   206.13	  5
 21	 Q 	   701.72	   168.43	  4
 22	 D 	   740.06	   125.75	  3
 23	 L 	   777.76	    87.40	  2
 24	 K 	        -  	    49.71	  1  _
```

**171. IPI00479018 Syntenin isoform 3**

| Sequence | Charge | M+H+ | XCorr | DeltaCn |
| --- | --- | --- | --- | --- |
| R.LKSIDNGIFVQLVQANSPASLVGLR.F | 3 | 2641.063 | 4.087 | 0.291 |

+1 charge

```
		      B   	     Y
  1	 L 	   114.09	        -  	 25
  2	 K 	   242.19	  2526.40	 24
  3	 S 	   329.22	  2398.31	 23
  4	 I 	   442.30	  2311.28	 22
  5	 D 	   557.33	  2198.19	 21
  6	 N 	   671.37	  2083.17	 20
  7	 G 	   728.39	  1969.12	 19
  8	 I 	   841.48	  1912.10	 18
  9	 F 	   988.55	  1799.02	 17
 10	 V 	  1087.61	  1651.95	 16
 11	 Q 	  1215.67	  1552.88	 15
 12	 L 	  1328.76	  1424.82	 14
 13	 V 	  1427.83	  1311.74	 13
 14	 Q 	  1555.88	  1212.67	 12
 15	 A 	  1626.92	  1084.61	 11
 16	 N 	  1740.96	  1013.57	 10
 17	 S 	  1828.00	   899.53	  9
 18	 P 	  1925.05	   812.50	  8
 19	 A 	  1996.09	   715.45	  7
 20	 S 	  2083.12	   644.41	  6
 21	 L 	  2196.20	   557.38	  5
 22	 V 	  2295.27	   444.29	  4
 23	 G 	  2352.29	   345.22	  3
 24	 L 	  2465.38	   288.20	  2
 25	 R 	        -  	   175.12	  1  _


+2 charge
		      B   	     Y
  1	 L 	    57.55	        -  	 25
  2	 K 	   121.60	  1263.71	 24
  3	 S 	   165.11	  1199.66	 23
  4	 I 	   221.65	  1156.14	 22
  5	 D 	   279.17	  1099.60	 21
  6	 N 	   336.19	  1042.09	 20
  7	 G 	   364.70	   985.07	 19
  8	 I 	   421.24	   956.55	 18
  9	 F 	   494.78	   900.01	 17
 10	 V 	   544.31	   826.48	 16
 11	 Q 	   608.34	   776.94	 15
 12	 L 	   664.88	   712.91	 14
 13	 V 	   714.42	   656.37	 13
 14	 Q 	   778.45	   606.84	 12
 15	 A 	   813.96	   542.81	 11
 16	 N 	   870.99	   507.29	 10
 17	 S 	   914.50	   450.27	  9
 18	 P 	   963.03	   406.75	  8
 19	 A 	   998.55	   358.23	  7
 20	 S 	  1042.06	   322.71	  6
 21	 L 	  1098.60	   279.19	  5
 22	 V 	  1148.14	   222.65	  4
 23	 G 	  1176.65	   173.12	  3
 24	 L 	  1233.19	   144.61	  2
 25	 R 	        -  	    88.06	  1  _


+3 charge
		      B   	     Y
  1	 L 	    38.70	        -  	 25
  2	 K 	    81.40	   842.81	 24
  3	 S 	   110.41	   800.11	 23
  4	 I 	   148.11	   771.10	 22
  5	 D 	   186.45	   733.40	 21
  6	 N 	   224.46	   695.06	 20
  7	 G 	   243.47	   657.05	 19
  8	 I 	   281.16	   638.04	 18
  9	 F 	   330.19	   600.34	 17
 10	 V 	   363.21	   551.32	 16
 11	 Q 	   405.90	   518.30	 15
 12	 L 	   443.59	   475.61	 14
 13	 V 	   476.61	   437.92	 13
 14	 Q 	   519.30	   404.89	 12
 15	 A 	   542.98	   362.21	 11
 16	 N 	   580.99	   338.53	 10
 17	 S 	   610.00	   300.52	  9
 18	 P 	   642.35	   271.50	  8
 19	 A 	   666.03	   239.15	  7
 20	 S 	   695.04	   215.47	  6
 21	 L 	   732.74	   186.46	  5
 22	 V 	   765.76	   148.77	  4
 23	 G 	   784.77	   115.75	  3
 24	 L 	   822.46	    96.74	  2
 25	 R 	        -  	    59.04	  1  _
```

**172. IPI00029275 Isoform 1 of Melanotransferrin**

| Sequence | Charge | M+H+ | XCorr | DeltaCn |
| --- | --- | --- | --- | --- |
| R.LCRGDSSGEGVCDKSPLER.Y | 3 | 2123.255 | 4.319 | 0.338 |

+1 charge

```
		      B   	     Y
  1	 L 	   114.09	        -  	 19
  2	 C 	   274.26	  2009.15	 18
  3	 R 	   430.36	  1848.99	 17
  4	 G 	   487.38	  1692.88	 16
  5	 D 	   602.41	  1635.86	 15
  6	 S 	   689.44	  1520.84	 14
  7	 S 	   776.47	  1433.80	 13
  8	 G 	   833.49	  1346.77	 12
  9	 E 	   962.53	  1289.75	 11
 10	 G 	  1019.56	  1160.71	 10
 11	 V 	  1118.62	  1103.69	  9
 12	 C 	  1278.79	  1004.62	  8
 13	 D 	  1393.82	   844.45	  7
 14	 K 	  1521.91	   729.43	  6
 15	 S 	  1608.94	   601.33	  5
 16	 P 	  1706.00	   514.30	  4
 17	 L 	  1819.08	   417.25	  3
 18	 E 	  1948.12	   304.16	  2
 19	 R 	        -  	   175.12	  1  _
+2 charge
		      B   	     Y
  1	 L 	    57.55	        -  	 19
  2	 C 	   137.63	  1005.08	 18
  3	 R 	   215.68	   925.00	 17
  4	 G 	   244.19	   846.95	 16
  5	 D 	   301.71	   818.43	 15
  6	 S 	   345.22	   760.92	 14
  7	 S 	   388.74	   717.41	 13
  8	 G 	   417.25	   673.89	 12
  9	 E 	   481.77	   645.38	 11
 10	 G 	   510.28	   580.86	 10
 11	 V 	   559.82	   552.35	  9
 12	 C 	   639.90	   502.81	  8
 13	 D 	   697.41	   422.73	  7
 14	 K 	   761.46	   365.22	  6
 15	 S 	   804.98	   301.17	  5
 16	 P 	   853.50	   257.65	  4
 17	 L 	   910.04	   209.13	  3
 18	 E 	   974.56	   152.58	  2
 19	 R 	        -  	    88.06	  1  _

+3 charge
		      B   	     Y
  1	 L 	    38.70	        -  	 19
  2	 C 	    92.09	   670.39	 18
  3	 R 	   144.12	   617.00	 17
  4	 G 	   163.13	   564.97	 16
  5	 D 	   201.47	   545.96	 15
  6	 S 	   230.48	   507.62	 14
  7	 S 	   259.49	   478.61	 13
  8	 G 	   278.50	   449.60	 12
  9	 E 	   321.52	   430.59	 11
 10	 G 	   340.52	   387.57	 10
 11	 V 	   373.55	   368.57	  9
 12	 C 	   426.93	   335.54	  8
 13	 D 	   465.28	   282.16	  7
 14	 K 	   507.98	   243.81	  6
 15	 S 	   536.99	   201.11	  5
 16	 P 	   569.34	   172.10	  4
 17	 L 	   607.03	   139.75	  3
 18	 E 	   650.05	   102.06	  2
 19	 R 	        -  	    59.04	  1  _
```

**173. IPI00001759 Oxidized low-density lipoprotein receptor 1**

| Sequence | Charge | M+H+ | XCorr | DeltaCn |
| --- | --- | --- | --- | --- |
| R.GAVSQTYPSGTCAYIQR.G | 2 | 1860.011 | 3.413 | 0.443 |

+1 charge

```
		      B   	     Y
  1	 G 	    58.03	        -  	 17
  2	 A 	   129.07	  1801.99	 16
  3	 V 	   228.13	  1730.95	 15
  4	 S 	   315.17	  1631.88	 14
  5	 Q 	   443.22	  1544.85	 13
  6	 T 	   544.27	  1416.79	 12
  7	 Y 	   707.34	  1315.74	 11
  8	 P 	   804.39	  1152.68	 10
  9	 S 	   891.42	  1055.63	  9
 10	 G 	   948.44	   968.60	  8
 11	 T 	  1049.49	   911.57	  7
 12	 C 	  1209.66	   810.53	  6
 13	 A 	  1280.69	   650.36	  5
 14	 Y 	  1443.76	   579.32	  4
 15	 I 	  1556.84	   416.26	  3
 16	 Q 	  1684.90	   303.18	  2
 17	 R 	        -  	   175.12	  1  _
+2 charge
		      B   	     Y
  1	 G 	    29.52	        -  	 17
  2	 A 	    65.04	   901.50	 16
  3	 V 	   114.57	   865.98	 15
  4	 S 	   158.09	   816.45	 14
  5	 Q 	   222.12	   772.93	 13
  6	 T 	   272.64	   708.90	 12
  7	 Y 	   354.17	   658.38	 11
  8	 P 	   402.70	   576.84	 10
  9	 S 	   446.21	   528.32	  9
 10	 G 	   474.72	   484.80	  8
 11	 T 	   525.25	   456.29	  7
 12	 C 	   605.33	   405.77	  6
 13	 A 	   640.85	   325.68	  5
 14	 Y 	   722.38	   290.17	  4
 15	 I 	   778.92	   208.63	  3
 16	 Q 	   842.95	   152.09	  2
 17	 R 	        -  	    88.06	  1  _
```

**174. IPI00386839 Amyloid lambda 6 light chain variable region SAR (Fragment)**

| Sequence | Charge | M+H+ | XCorr | DeltaCn |
| --- | --- | --- | --- | --- |
| - .NFMLTQPHSVSESPGK.T | 2 | 1759.965 | 3.14 | 0.516 |

+1 charge

```
		      B   	     Y
  1	 N 	   115.05	        -  	 16
  2	 F 	   262.12	  1644.81	 15
  3	 M 	   393.16	  1497.74	 14
  4	 L 	   506.24	  1366.70	 13
  5	 T 	   607.29	  1253.61	 12
  6	 Q 	   735.35	  1152.56	 11
  7	 P 	   832.40	  1024.51	 10
  8	 H 	   969.46	   927.45	  9
  9	 S 	  1056.49	   790.39	  8
 10	 V 	  1155.56	   703.36	  7
 11	 S 	  1242.59	   604.29	  6
 12	 E 	  1371.64	   517.26	  5
 13	 S 	  1458.67	   388.22	  4
 14	 P 	  1555.72	   301.19	  3
 15	 G 	  1612.74	   204.13	  2
 16	 K 	        -  	   147.11	  1  _
+2 charge
		      B   	     Y
  1	 N 	    58.03	        -  	 16
  2	 F 	   131.56	   822.91	 15
  3	 M 	   197.08	   749.37	 14
  4	 L 	   253.63	   683.85	 13
  5	 T 	   304.15	   627.31	 12
  6	 Q 	   368.18	   576.79	 11
  7	 P 	   416.70	   512.76	 10
  8	 H 	   485.23	   464.23	  9
  9	 S 	   528.75	   395.70	  8
 10	 V 	   578.28	   352.18	  7
 11	 S 	   621.80	   302.65	  6
 12	 E 	   686.32	   259.13	  5
 13	 S 	   729.84	   194.61	  4
 14	 P 	   778.36	   151.10	  3
 15	 G 	   806.87	   102.57	  2
 16	 K 	        -  	    74.06	  1  _
```

**175. IPI00012102 N-acetylglucosamine-6-sulfatase**

| Sequence | Charge | M+H+ | XCorr | DeltaCn |
| --- | --- | --- | --- | --- |
| R.LMMLQSCSGPTCR.T | 2 | 1541.807 | 3.754 | 0.478 |

+1 charge

```
		      B   	     Y
  1	 L 	   114.09	        -  	 13
  2	 M 	   245.13	  1427.86	 12
  3	 M 	   376.17	  1296.82	 11
  4	 L 	   489.26	  1165.78	 10
  5	 Q 	   617.31	  1052.69	  9
  6	 S 	   704.35	   924.64	  8
  7	 C 	   864.51	   837.60	  7
  8	 S 	   951.54	   677.44	  6
  9	 G 	  1008.57	   590.41	  5
 10	 P 	  1105.62	   533.38	  4
 11	 T 	  1206.67	   436.33	  3
 12	 C 	  1366.83	   335.28	  2
 13	 R 	        -  	   175.12	  1  _
+2 charge
		      B   	     Y
  1	 L 	    57.55	        -  	 13
  2	 M 	   123.07	   714.43	 12
  3	 M 	   188.59	   648.91	 11
  4	 L 	   245.13	   583.39	 10
  5	 Q 	   309.16	   526.85	  9
  6	 S 	   352.68	   462.82	  8
  7	 C 	   432.76	   419.31	  7
  8	 S 	   476.28	   339.22	  6
  9	 G 	   504.79	   295.71	  5
 10	 P 	   553.31	   267.20	  4
 11	 T 	   603.84	   218.67	  3
 12	 C 	   683.92	   168.15	  2
 13	 R 	        -  	    88.06	  1  _
```

**176. IPI00790530 Nuclear pore complex protein Nup85**

| Sequence | Charge | M+H+ | XCorr | DeltaCn |
| --- | --- | --- | --- | --- |
| K.IMLGDEAALLEQK.E | 2 | 1431.68 | 3.083 | 0.248 |

+1 charge

B	Y

1	I	114.09	-	13

2	M	245.13	1317.67	12

3	L	358.22	1186.63	11

4	G	415.24	1073.55	10

5	D	530.26	1016.53	9

6	E	659.31	901.50	8

7	A	730.34	772.46	7

8	A	801.38	701.42	6

9	L	914.47	630.38	5

10	L	1027.55	517.30	4

11	E	1156.59	404.21	3

12	Q	1284.65	275.17	2

13	K	-	147.11	1

+2 charge

B	Y

1	I	57.55	-	13

2	M	123.07	659.34	12

3	L	179.61	593.82	11

4	G	208.12	537.28	10

5	D	265.64	508.77	9

6	E	330.16	451.25	8

7	A	365.68	386.73	7

8	A	401.19	351.21	6

9	L	457.74	315.69	5

10	L	514.28	259.15	4

11	E	578.80	202.61	3

12	Q	642.83	138.09	2

13	K	-	74.06	1

**177. IPI00005222 ephrin receptor EphB6 precursor**

| Sequence | Charge | M+H+ | XCorr | DeltaCn |
| --- | --- | --- | --- | --- |
| R.SHAPNPAAPVCPCLEGFYR.A | 2 | 2144.362 | 2.928 | 0.457 |

+1 charge

```
		      B   	     Y
  1	 S 	    88.04	        -  	 19
  2	 H 	   225.10	  2056.22	 18
  3	 A 	   296.14	  1919.16	 17
  4	 P 	   393.19	  1848.13	 16
  5	 N 	   507.23	  1751.07	 15
  6	 P 	   604.28	  1637.03	 14
  7	 A 	   675.32	  1539.98	 13
  8	 A 	   746.36	  1468.94	 12
  9	 P 	   843.41	  1397.90	 11
 10	 V 	   942.48	  1300.85	 10
 11	 C 	  1102.64	  1201.78	  9
 12	 P 	  1199.70	  1041.62	  8
 13	 C 	  1359.86	   944.56	  7
 14	 L 	  1472.95	   784.40	  6
 15	 E 	  1601.99	   671.31	  5
 16	 G 	  1659.01	   542.27	  4
 17	 F 	  1806.08	   485.25	  3
 18	 Y 	  1969.14	   338.18	  2
 19	 R 	        -  	   175.12	  1  _
+2 charge
		      B   	     Y
  1	 S 	    44.52	        -  	 19
  2	 H 	   113.05	  1028.61	 18
  3	 A 	   148.57	   960.09	 17
  4	 P 	   197.10	   924.57	 16
  5	 N 	   254.12	   876.04	 15
  6	 P 	   302.65	   819.02	 14
  7	 A 	   338.16	   770.49	 13
  8	 A 	   373.68	   734.97	 12
  9	 P 	   422.21	   699.46	 11
 10	 V 	   471.74	   650.93	 10
 11	 C 	   551.83	   601.39	  9
 12	 P 	   600.35	   521.31	  8
 13	 C 	   680.43	   472.79	  7
 14	 L 	   736.98	   392.70	  6
 15	 E 	   801.50	   336.16	  5
 16	 G 	   830.01	   271.64	  4
 17	 F 	   903.54	   243.13	  3
 18	 Y 	   985.07	   169.59	  2
 19	 R 	        -  	    88.06	  1  _
```

**178. IPI00018953 Dipeptidyl peptidase 4**

| Sequence | Charge | M+H+ | XCorr | DeltaCn |
| --- | --- | --- | --- | --- |
| R.LGTFEVEDQIEAAR.Q | 2 | 1578.705 | 3.695 | 0.445 |

+1 charge

```
		      B   	     Y
  1	 L 	   114.09	        -  	 14
  2	 G 	   171.11	  1464.70	 13
  3	 T 	   272.16	  1407.68	 12
  4	 F 	   419.23	  1306.63	 11
  5	 E 	   548.27	  1159.56	 10
  6	 V 	   647.34	  1030.52	  9
  7	 E 	   776.38	   931.45	  8
  8	 D 	   891.41	   802.41	  7
  9	 Q 	  1019.47	   687.38	  6
 10	 I 	  1132.55	   559.32	  5
 11	 E 	  1261.59	   446.24	  4
 12	 A 	  1332.63	   317.19	  3
 13	 A 	  1403.67	   246.16	  2
 14	 R 	        -  	   175.12	  1  _
+2 charge
		      B   	     Y
  1	 L 	    57.55	        -  	 14
  2	 G 	    86.06	   732.85	 13
  3	 T 	   136.58	   704.34	 12
  4	 F 	   210.12	   653.82	 11
  5	 E 	   274.64	   580.28	 10
  6	 V 	   324.17	   515.76	  9
  7	 E 	   388.69	   466.23	  8
  8	 D 	   446.21	   401.71	  7
  9	 Q 	   510.24	   344.19	  6
 10	 I 	   566.78	   280.16	  5
 11	 E 	   631.30	   223.62	  4
 12	 A 	   666.82	   159.10	  3
 13	 A 	   702.34	   123.58	  2
 14	 R 	        -  	    88.06	  1  _
```

**179. IPI00065500 BRO1 domain-containing protein BROX**

| Sequence | Charge | M+H+ | XCorr | DeltaCn |
| --- | --- | --- | --- | --- |
| K.ATAPVSFNYYGVVTGPSASK.I | 2 | 2017.227 | 3.165 | 0.367 |

+1 charge

```
		      B   	     Y
  1	 A 	    72.04	        -  	 20
  2	 T 	   173.09	  1944.97	 19
  3	 A 	   244.13	  1843.92	 18
  4	 P 	   341.18	  1772.89	 17
  5	 V 	   440.25	  1675.83	 16
  6	 S 	   527.28	  1576.76	 15
  7	 F 	   674.35	  1489.73	 14
  8	 N 	   788.39	  1342.66	 13
  9	 Y 	   951.46	  1228.62	 12
 10	 Y 	  1114.52	  1065.56	 11
 11	 G 	  1171.54	   902.49	 10
 12	 V 	  1270.61	   845.47	  9
 13	 V 	  1369.68	   746.40	  8
 14	 T 	  1470.73	   647.34	  7
 15	 G 	  1527.75	   546.29	  6
 16	 P 	  1624.80	   489.27	  5
 17	 S 	  1711.83	   392.21	  4
 18	 A 	  1782.87	   305.18	  3
 19	 S 	  1869.90	   234.14	  2
 20	 K 	        -  	   147.11	  1  _
+2 charge
		      B   	     Y
  1	 A 	    36.53	        -  	 20
  2	 T 	    87.05	   972.99	 19
  3	 A 	   122.57	   922.46	 18
  4	 P 	   171.09	   886.95	 17
  5	 V 	   220.63	   838.42	 16
  6	 S 	   264.14	   788.89	 15
  7	 F 	   337.68	   745.37	 14
  8	 N 	   394.70	   671.84	 13
  9	 Y 	   476.23	   614.81	 12
 10	 Y 	   557.76	   533.28	 11
 11	 G 	   586.27	   451.75	 10
 12	 V 	   635.81	   423.24	  9
 13	 V 	   685.34	   373.71	  8
 14	 T 	   735.87	   324.17	  7
 15	 G 	   764.38	   273.65	  6
 16	 P 	   812.90	   245.14	  5
 17	 S 	   856.42	   196.61	  4
 18	 A 	   891.94	   153.09	  3
 19	 S 	   935.45	   117.58	  2
 20	 K 	        -  	    74.06	  1  _
```

**180. IPI00382478 Ig heavy chain V-III region TIL**

| Sequence | Charge | M+H+ | XCorr | DeltaCn |
| --- | --- | --- | --- | --- |
| - .EVQLLESGGGLVQPGGSLR.L | 2 | 1897.122 | 3.089 | 0.269 |

+1 charge

```
		      B   	     Y
  1	 E 	   130.05	        -  	 19
  2	 V 	   229.12	  1766.98	 18
  3	 Q 	   357.18	  1667.91	 17
  4	 L 	   470.26	  1539.85	 16
  5	 L 	   583.34	  1426.76	 15
  6	 E 	   712.39	  1313.68	 14
  7	 S 	   799.42	  1184.64	 13
  8	 G 	   856.44	  1097.61	 12
  9	 G 	   913.46	  1040.58	 11
 10	 G 	   970.48	   983.56	 10
 11	 L 	  1083.57	   926.54	  9
 12	 V 	  1182.64	   813.46	  8
 13	 Q 	  1310.70	   714.39	  7
 14	 P 	  1407.75	   586.33	  6
 15	 G 	  1464.77	   489.28	  5
 16	 G 	  1521.79	   432.26	  4
 17	 S 	  1608.82	   375.24	  3
 18	 L 	  1721.91	   288.20	  2
 19	 R 	        -  	   175.12	  1  _
+2 charge
		      B   	     Y
  1	 E 	    65.53	        -  	 19
  2	 V 	   115.06	   883.99	 18
  3	 Q 	   179.09	   834.46	 17
  4	 L 	   235.63	   770.43	 16
  5	 L 	   292.18	   713.89	 15
  6	 E 	   356.70	   657.34	 14
  7	 S 	   400.21	   592.82	 13
  8	 G 	   428.72	   549.31	 12
  9	 G 	   457.23	   520.80	 11
 10	 G 	   485.75	   492.29	 10
 11	 L 	   542.29	   463.77	  9
 12	 V 	   591.82	   407.23	  8
 13	 Q 	   655.85	   357.70	  7
 14	 P 	   704.38	   293.67	  6
 15	 G 	   732.89	   245.14	  5
 16	 G 	   761.40	   216.63	  4
 17	 S 	   804.92	   188.12	  3
 18	 L 	   861.46	   144.61	  2
 19	 R 	        -  	    88.06	  1  _
```

**181. IPI00005732 Isoform 1 of Activin receptor type-1B**

| Sequence | Charge | M+H+ | XCorr | DeltaCn |
| --- | --- | --- | --- | --- |
| R.TCIPKVELVPAGKPFYCLSSEDLR.N | 3 | 2781.184 | 3.934 | 0.487 |

+1 charge

```
		      B   	     Y
  1	 T 	   102.05	        -  	 24
  2	 C 	   262.22	  2678.64	 23
  3	 I 	   375.30	  2518.47	 22
  4	 P 	   472.36	  2405.39	 21
  5	 K 	   600.45	  2308.34	 20
  6	 V 	   699.52	  2180.24	 19
  7	 E 	   828.56	  2081.17	 18
  8	 L 	   941.65	  1952.13	 17
  9	 V 	  1040.72	  1839.05	 16
 10	 P 	  1137.77	  1739.98	 15
 11	 A 	  1208.81	  1642.92	 14
 12	 G 	  1265.83	  1571.89	 13
 13	 K 	  1393.92	  1514.87	 12
 14	 P 	  1490.97	  1386.77	 11
 15	 F 	  1638.04	  1289.72	 10
 16	 Y 	  1801.11	  1142.65	  9
 17	 C 	  1961.27	   979.59	  8
 18	 L 	  2074.36	   819.42	  7
 19	 S 	  2161.39	   706.34	  6
 20	 S 	  2248.42	   619.30	  5
 21	 E 	  2377.46	   532.27	  4
 22	 D 	  2492.49	   403.23	  3
 23	 L 	  2605.57	   288.20	  2
 24	 R 	        -  	   175.12	  1  _


+2 charge
		      B   	     Y
  1	 T 	    51.53	        -  	 24
  2	 C 	   131.61	  1339.82	 23
  3	 I 	   188.16	  1259.74	 22
  4	 P 	   236.68	  1203.20	 21
  5	 K 	   300.73	  1154.67	 20
  6	 V 	   350.26	  1090.62	 19
  7	 E 	   414.79	  1041.09	 18
  8	 L 	   471.33	   976.57	 17
  9	 V 	   520.86	   920.03	 16
 10	 P 	   569.39	   870.49	 15
 11	 A 	   604.91	   821.97	 14
 12	 G 	   633.42	   786.45	 13
 13	 K 	   697.46	   757.94	 12
 14	 P 	   745.99	   693.89	 11
 15	 F 	   819.53	   645.36	 10
 16	 Y 	   901.06	   571.83	  9
 17	 C 	   981.14	   490.30	  8
 18	 L 	  1037.68	   410.21	  7
 19	 S 	  1081.20	   353.67	  6
 20	 S 	  1124.71	   310.16	  5
 21	 E 	  1189.23	   266.64	  4
 22	 D 	  1246.75	   202.12	  3
 23	 L 	  1303.29	   144.61	  2
 24	 R 	        -  	    88.06	  1  _


+3 charge
		      B   	     Y
  1	 T 	    34.69	        -  	 24
  2	 C 	    88.08	   893.55	 23
  3	 I 	   125.77	   840.16	 22
  4	 P 	   158.12	   802.47	 21
  5	 K 	   200.82	   770.12	 20
  6	 V 	   233.84	   727.42	 19
  7	 E 	   276.86	   694.40	 18
  8	 L 	   314.55	   651.38	 17
  9	 V 	   347.58	   613.69	 16
 10	 P 	   379.93	   580.66	 15
 11	 A 	   403.61	   548.31	 14
 12	 G 	   422.61	   524.63	 13
 13	 K 	   465.31	   505.63	 12
 14	 P 	   497.66	   462.93	 11
 15	 F 	   546.69	   430.58	 10
 16	 Y 	   601.04	   381.55	  9
 17	 C 	   654.43	   327.20	  8
 18	 L 	   692.12	   273.81	  7
 19	 S 	   721.13	   236.12	  6
 20	 S 	   750.14	   207.11	  5
 21	 E 	   793.16	   178.10	  4
 22	 D 	   831.50	   135.08	  3
 23	 L 	   869.20	    96.74	  2
 24	 R 	        -  	    59.04	  1  _
```

**182. IPI00409566 Isoform 1 of Polycystin-1**

| Sequence | Charge | M+H+ | XCorr | DeltaCn |
| --- | --- | --- | --- | --- |
| R.TLGPALRIPADATALDVSHNLLR.A | 3 | 2415.777 | 3.743 | 0.375 |

+1 charge

```
		      B   	     Y
  1	 T 	   102.05	        -  	 23
  2	 L 	   215.14	  2313.30	 22
  3	 G 	   272.16	  2200.22	 21
  4	 P 	   369.21	  2143.20	 20
  5	 A 	   440.25	  2046.15	 19
  6	 L 	   553.33	  1975.11	 18
  7	 R 	   709.44	  1862.02	 17
  8	 I 	   822.52	  1705.92	 16
  9	 P 	   919.57	  1592.84	 15
 10	 A 	   990.61	  1495.79	 14
 11	 D 	  1105.64	  1424.75	 13
 12	 A 	  1176.67	  1309.72	 12
 13	 T 	  1277.72	  1238.69	 11
 14	 A 	  1348.76	  1137.64	 10
 15	 L 	  1461.84	  1066.60	  9
 16	 D 	  1576.87	   953.52	  8
 17	 V 	  1675.94	   838.49	  7
 18	 S 	  1762.97	   739.42	  6
 19	 H 	  1900.03	   652.39	  5
 20	 N 	  2014.07	   515.33	  4
 21	 L 	  2127.16	   401.29	  3
 22	 L 	  2240.24	   288.20	  2
 23	 R 	        -  	   175.12	  1  _


+2 charge
		      B   	     Y
  1	 T 	    51.53	        -  	 23
  2	 L 	   108.07	  1157.16	 22
  3	 G 	   136.58	  1100.61	 21
  4	 P 	   185.11	  1072.10	 20
  5	 A 	   220.63	  1023.58	 19
  6	 L 	   277.17	   988.06	 18
  7	 R 	   355.22	   931.52	 17
  8	 I 	   411.76	   853.47	 16
  9	 P 	   460.29	   796.92	 15
 10	 A 	   495.81	   748.40	 14
 11	 D 	   553.32	   712.88	 13
 12	 A 	   588.84	   655.36	 12
 13	 T 	   639.36	   619.85	 11
 14	 A 	   674.88	   569.32	 10
 15	 L 	   731.42	   533.80	  9
 16	 D 	   788.94	   477.26	  8
 17	 V 	   838.47	   419.75	  7
 18	 S 	   881.99	   370.21	  6
 19	 H 	   950.52	   326.70	  5
 20	 N 	  1007.54	   258.17	  4
 21	 L 	  1064.08	   201.15	  3
 22	 L 	  1120.62	   144.61	  2
 23	 R 	        -  	    88.06	  1  _


+3 charge
		      B   	     Y
  1	 T 	    34.69	        -  	 23
  2	 L 	    72.38	   771.77	 22
  3	 G 	    91.39	   734.08	 21
  4	 P 	   123.74	   715.07	 20
  5	 A 	   147.42	   682.72	 19
  6	 L 	   185.12	   659.04	 18
  7	 R 	   237.15	   621.35	 17
  8	 I 	   274.84	   569.31	 16
  9	 P 	   307.20	   531.62	 15
 10	 A 	   330.87	   499.27	 14
 11	 D 	   369.22	   475.59	 13
 12	 A 	   392.90	   437.25	 12
 13	 T 	   426.58	   413.57	 11
 14	 A 	   450.26	   379.88	 10
 15	 L 	   487.95	   356.20	  9
 16	 D 	   526.29	   318.51	  8
 17	 V 	   559.32	   280.17	  7
 18	 S 	   588.33	   247.15	  6
 19	 H 	   634.01	   218.13	  5
 20	 N 	   672.03	   172.45	  4
 21	 L 	   709.72	   134.43	  3
 22	 L 	   747.42	    96.74	  2
 23	 R 	        -  	    59.04	  1  _
```

**183. IPI00027350 Peroxiredoxin-2**

| Sequence | Charge | M+H+ | XCorr | DeltaCn |
| --- | --- | --- | --- | --- |
| R.KEGGLGPLNIPLLADVTR.R | 2 | 1864.179 | 3.024 | 0.417 |

+1 charge

```
		      B   	     Y
  1	 K 	   129.10	        -  	 18
  2	 E 	   258.14	  1734.97	 17
  3	 G 	   315.17	  1605.93	 16
  4	 G 	   372.19	  1548.91	 15
  5	 L 	   485.27	  1491.89	 14
  6	 G 	   542.29	  1378.81	 13
  7	 P 	   639.35	  1321.78	 12
  8	 L 	   752.43	  1224.73	 11
  9	 N 	   866.47	  1111.65	 10
 10	 I 	   979.56	   997.60	  9
 11	 P 	  1076.61	   884.52	  8
 12	 L 	  1189.69	   787.47	  7
 13	 L 	  1302.78	   674.38	  6
 14	 A 	  1373.82	   561.30	  5
 15	 D 	  1488.84	   490.26	  4
 16	 V 	  1587.91	   375.24	  3
 17	 T 	  1688.96	   276.17	  2
 18	 R 	        -  	   175.12	  1  _
+2 charge
		      B   	     Y
  1	 K 	    65.05	        -  	 18
  2	 E 	   129.58	   867.99	 17
  3	 G 	   158.09	   803.47	 16
  4	 G 	   186.60	   774.96	 15
  5	 L 	   243.14	   746.45	 14
  6	 G 	   271.65	   689.91	 13
  7	 P 	   320.18	   661.40	 12
  8	 L 	   376.72	   612.87	 11
  9	 N 	   433.74	   556.33	 10
 10	 I 	   490.28	   499.31	  9
 11	 P 	   538.81	   442.76	  8
 12	 L 	   595.35	   394.24	  7
 13	 L 	   651.89	   337.70	  6
 14	 A 	   687.41	   281.15	  5
 15	 D 	   744.92	   245.63	  4
 16	 V 	   794.46	   188.12	  3
 17	 T 	   844.98	   138.59	  2
 18	 R 	        -  	    88.06	  1  _
```

**184. IPI00021891 Isoform Gamma-B of Fibrinogen gamma chain**

| Sequence | Charge | M+H+ | XCorr | DeltaCn |
| --- | --- | --- | --- | --- |
| K.MLEEIMKYEASILTHDSSIR.Y | 3 | 2367.728 | 3.714 | 0.489 |

+1 charge

```
		      B   	     Y
  1	 M 	   132.05	        -  	 20
  2	 L 	   245.13	  2235.13	 19
  3	 E 	   374.17	  2122.05	 18
  4	 E 	   503.22	  1993.01	 17
  5	 I 	   616.30	  1863.96	 16
  6	 M 	   747.34	  1750.88	 15
  7	 K 	   875.44	  1619.84	 14
  8	 Y 	  1038.50	  1491.74	 13
  9	 E 	  1167.54	  1328.68	 12
 10	 A 	  1238.58	  1199.64	 11
 11	 S 	  1325.61	  1128.60	 10
 12	 I 	  1438.70	  1041.57	  9
 13	 L 	  1551.78	   928.48	  8
 14	 T 	  1652.83	   815.40	  7
 15	 H 	  1789.89	   714.35	  6
 16	 D 	  1904.91	   577.29	  5
 17	 S 	  1991.95	   462.27	  4
 18	 S 	  2078.98	   375.24	  3
 19	 I 	  2192.06	   288.20	  2
 20	 R 	        -  	   175.12	  1  _
+2 charge
		      B   	     Y
  1	 M 	    66.53	        -  	 20
  2	 L 	   123.07	  1118.07	 19
  3	 E 	   187.59	  1061.53	 18
  4	 E 	   252.11	   997.01	 17
  5	 I 	   308.65	   932.49	 16
  6	 M 	   374.17	   875.94	 15
  7	 K 	   438.22	   810.42	 14
  8	 Y 	   519.75	   746.38	 13
  9	 E 	   584.27	   664.84	 12
 10	 A 	   619.79	   600.32	 11
 11	 S 	   663.31	   564.80	 10
 12	 I 	   719.85	   521.29	  9
 13	 L 	   776.39	   464.75	  8
 14	 T 	   826.92	   408.20	  7
 15	 H 	   895.45	   357.68	  6
 16	 D 	   952.96	   289.15	  5
 17	 S 	   996.48	   231.64	  4
 18	 S 	  1039.99	   188.12	  3
 19	 I 	  1096.53	   144.61	  2
 20	 R 	        -  	    88.06	  1  _

+3 charge
		      B   	     Y
  1	 M 	    44.69	        -  	 20
  2	 L 	    82.38	   745.72	 19
  3	 E 	   125.40	   708.02	 18
  4	 E 	   168.41	   665.01	 17
  5	 I 	   206.11	   621.99	 16
  6	 M 	   249.79	   584.30	 15
  7	 K 	   292.48	   540.62	 14
  8	 Y 	   346.84	   497.92	 13
  9	 E 	   389.85	   443.57	 12
 10	 A 	   413.53	   400.55	 11
 11	 S 	   442.54	   376.87	 10
 12	 I 	   480.24	   347.86	  9
 13	 L 	   517.93	   310.17	  8
 14	 T 	   551.61	   272.47	  7
 15	 H 	   597.30	   238.79	  6
 16	 D 	   635.64	   193.10	  5
 17	 S 	   664.65	   154.76	  4
 18	 S 	   693.66	   125.75	  3
 19	 I 	   731.36	    96.74	  2
 20	 R 	        -  	    59.04	  1  _
```

**185. IPI00794461 Histone H2B type 1-N**

| Sequence | Charge | M+H+ | XCorr | DeltaCn |
| --- | --- | --- | --- | --- |
| K.AMGIMNSFVNDIFER.I | 2 | 1745.017 | 5.497 | 0.561 |

+1 charge

```
		      B   	     Y
  1	 A 	    72.04	        -  	 15
  2	 M 	   203.08	  1672.78	 14
  3	 G 	   260.11	  1541.74	 13
  4	 I 	   373.19	  1484.72	 12
  5	 M 	   504.23	  1371.64	 11
  6	 N 	   618.27	  1240.60	 10
  7	 S 	   705.31	  1126.55	  9
  8	 F 	   852.37	  1039.52	  8
  9	 V 	   951.44	   892.45	  7
 10	 N 	  1065.49	   793.38	  6
 11	 D 	  1180.51	   679.34	  5
 12	 I 	  1293.60	   564.31	  4
 13	 F 	  1440.67	   451.23	  3
 14	 E 	  1569.71	   304.16	  2
 15	 R 	        -  	   175.12	  1  _
+2 charge
		      B   	     Y
  1	 A 	    36.53	        -  	 15
  2	 M 	   102.05	   836.89	 14
  3	 G 	   130.56	   771.37	 13
  4	 I 	   187.10	   742.86	 12
  5	 M 	   252.62	   686.32	 11
  6	 N 	   309.64	   620.80	 10
  7	 S 	   353.16	   563.78	  9
  8	 F 	   426.69	   520.26	  8
  9	 V 	   476.22	   446.73	  7
 10	 N 	   533.25	   397.20	  6
 11	 D 	   590.76	   340.17	  5
 12	 I 	   647.30	   282.66	  4
 13	 F 	   720.84	   226.12	  3
 14	 E 	   785.36	   152.58	  2
 15	 R 	        -  	    88.06	  1  _
```

**Female 8.**

**1. IPI00292150 Latent-transforming growth factor beta-binding protein 2**

| Sequence | Charge | M+H+ | XCorr | DeltaCn |
| --- | --- | --- | --- | --- |
| R.CHCSPGYVAEAGPPHCTAKE.- | 3 | 2229.373 | 5.453 | 0.62 |

+1 charge

```
		      B   	     Y
  1	 C 	   161.17	        -  	 20
  2	 H 	   298.23	  2068.17	 19
  3	 C 	   458.40	  1931.11	 18
  4	 S 	   545.43	  1770.95	 17
  5	 P 	   642.48	  1683.91	 16
  6	 G 	   699.50	  1586.86	 15
  7	 Y 	   862.57	  1529.84	 14
  8	 V 	   961.63	  1366.78	 13
  9	 A 	  1032.67	  1267.71	 12
 10	 E 	  1161.71	  1196.67	 11
 11	 A 	  1232.75	  1067.63	 10
 12	 G 	  1289.77	   996.59	  9
 13	 P 	  1386.83	   939.57	  8
 14	 P 	  1483.88	   842.52	  7
 15	 H 	  1620.94	   745.46	  6
 16	 C 	  1781.10	   608.41	  5
 17	 T 	  1882.15	   448.24	  4
 18	 A 	  1953.19	   347.19	  3
 19	 K 	  2081.28	   276.16	  2
 20	 E 	        -  	   148.06	  1  _
+2 charge
		      B   	     Y
  1	 C 	    81.09	        -  	 20
  2	 H 	   149.62	  1034.59	 19
  3	 C 	   229.70	   966.06	 18
  4	 S 	   273.22	   885.98	 17
  5	 P 	   321.74	   842.46	 16
  6	 G 	   350.26	   793.93	 15
  7	 Y 	   431.79	   765.42	 14
  8	 V 	   481.32	   683.89	 13
  9	 A 	   516.84	   634.36	 12
 10	 E 	   581.36	   598.84	 11
 11	 A 	   616.88	   534.32	 10
 12	 G 	   645.39	   498.80	  9
 13	 P 	   693.92	   470.29	  8
 14	 P 	   742.44	   421.76	  7
 15	 H 	   810.97	   373.24	  6
 16	 C 	   891.05	   304.71	  5
 17	 T 	   941.58	   224.62	  4
 18	 A 	   977.10	   174.10	  3
 19	 K 	  1041.14	   138.58	  2
 20	 E 	        -  	    74.53	  1  _


+3 charge
		      B   	     Y
  1	 C 	    54.40	        -  	 20
  2	 H 	   100.08	   690.06	 19
  3	 C 	   153.47	   644.38	 18
  4	 S 	   182.48	   590.99	 17
  5	 P 	   214.83	   561.98	 16
  6	 G 	   233.84	   529.63	 15
  7	 Y 	   288.19	   510.62	 14
  8	 V 	   321.22	   456.26	 13
  9	 A 	   344.90	   423.24	 12
 10	 E 	   387.91	   399.56	 11
 11	 A 	   411.59	   356.55	 10
 12	 G 	   430.60	   332.87	  9
 13	 P 	   462.95	   313.86	  8
 14	 P 	   495.30	   281.51	  7
 15	 H 	   540.98	   249.16	  6
 16	 C 	   594.37	   203.47	  5
 17	 T 	   628.05	   150.08	  4
 18	 A 	   651.73	   116.40	  3
 19	 K 	   694.43	    92.72	  2
 20	 E 	        -  	    50.02	  1  _
```

**2. IPI00433028 Isoform 1 of Leukocyte-associated immunoglobulin-like receptor 1**

| Sequence | Charge | M+H+ | XCorr | DeltaCn |
| --- | --- | --- | --- | --- |
| R.FRIDSVSEGNAGPYR.C | 2 | 1668.792 | 4.686 | 0.385 |

+1 charge

```
		      B   	     Y
  1	 F 	   148.08	        -  	 15
  2	 R 	   304.18	  1520.75	 14
  3	 I 	   417.26	  1364.64	 13
  4	 D 	   532.29	  1251.56	 12
  5	 S 	   619.32	  1136.53	 11
  6	 V 	   718.39	  1049.50	 10
  7	 S 	   805.42	   950.43	  9
  8	 E 	   934.46	   863.40	  8
  9	 G 	   991.48	   734.36	  7
 10	 N 	  1105.53	   677.34	  6
 11	 A 	  1176.56	   563.29	  5
 12	 G 	  1233.59	   492.26	  4
 13	 P 	  1330.64	   435.24	  3
 14	 Y 	  1493.70	   338.18	  2
 15	 R 	        -  	   175.12	  1  _
+2 charge
		      B   	     Y
  1	 F 	    74.54	        -  	 15
  2	 R 	   152.59	   760.88	 14
  3	 I 	   209.13	   682.83	 13
  4	 D 	   266.65	   626.28	 12
  5	 S 	   310.16	   568.77	 11
  6	 V 	   359.70	   525.25	 10
  7	 S 	   403.21	   475.72	  9
  8	 E 	   467.74	   432.20	  8
  9	 G 	   496.25	   367.68	  7
 10	 N 	   553.27	   339.17	  6
 11	 A 	   588.79	   282.15	  5
 12	 G 	   617.30	   246.63	  4
 13	 P 	   665.82	   218.12	  3
 14	 Y 	   747.35	   169.59	  2
 15	 R 	        -  	    88.06	  1  _
```

**3. IPI00382440 Ig lambda chain V-IV region Hil**

| Sequence | Charge | M+H+ | XCorr | DeltaCn |
| --- | --- | --- | --- | --- |
| - .SYELTQPPSVSVSPGQTAR.I | 2 | 2005.175 | 3.477 | 0.602 |

+1 charge

```
		      B   	     Y
  1	 S 	    88.04	        -  	 19
  2	 Y 	   251.10	  1916.97	 18
  3	 E 	   380.15	  1753.91	 17
  4	 L 	   493.23	  1624.87	 16
  5	 T 	   594.28	  1511.78	 15
  6	 Q 	   722.34	  1410.73	 14
  7	 P 	   819.39	  1282.67	 13
  8	 P 	   916.44	  1185.62	 12
  9	 S 	  1003.47	  1088.57	 11
 10	 V 	  1102.54	  1001.54	 10
 11	 S 	  1189.57	   902.47	  9
 12	 V 	  1288.64	   815.44	  8
 13	 S 	  1375.67	   716.37	  7
 14	 P 	  1472.73	   629.34	  6
 15	 G 	  1529.75	   532.28	  5
 16	 Q 	  1657.81	   475.26	  4
 17	 T 	  1758.85	   347.20	  3
 18	 A 	  1829.89	   246.16	  2
 19	 R 	        -  	   175.12	  1  _
+2 charge
		      B   	     Y
  1	 S 	    44.52	        -  	 19
  2	 Y 	   126.05	   958.99	 18
  3	 E 	   190.58	   877.46	 17
  4	 L 	   247.12	   812.94	 16
  5	 T 	   297.64	   756.39	 15
  6	 Q 	   361.67	   705.87	 14
  7	 P 	   410.20	   641.84	 13
  8	 P 	   458.72	   593.31	 12
  9	 S 	   502.24	   544.79	 11
 10	 V 	   551.77	   501.27	 10
 11	 S 	   595.29	   451.74	  9
 12	 V 	   644.82	   408.22	  8
 13	 S 	   688.34	   358.69	  7
 14	 P 	   736.87	   315.17	  6
 15	 G 	   765.38	   266.65	  5
 16	 Q 	   829.41	   238.13	  4
 17	 T 	   879.93	   174.11	  3
 18	 A 	   915.45	   123.58	  2
 19	 R 	        -  	    88.06	  1  _
```

**4. IPI00009950 Vesicular integral-membrane protein VIP36**

| Sequence | Charge | M+H+ | XCorr | DeltaCn |
| --- | --- | --- | --- | --- |
| R.WTELAGCTADFR.N | 2 | 1427.537 | 4.474 | 0.445 |

+1 charge

```
		      B   	     Y
  1	 W 	   187.09	        -  	 12
  2	 T 	   288.13	  1240.70	 11
  3	 E 	   417.18	  1139.65	 10
  4	 L 	   530.26	  1010.61	  9
  5	 A 	   601.30	   897.52	  8
  6	 G 	   658.32	   826.49	  7
  7	 C 	   818.48	   769.46	  6
  8	 T 	   919.53	   609.30	  5
  9	 A 	   990.57	   508.25	  4
 10	 D 	  1105.60	   437.21	  3
 11	 F 	  1252.66	   322.19	  2
 12	 R 	        -  	   175.12	  1  _
+2 charge
		      B   	     Y
  1	 W 	    94.05	        -  	 12
  2	 T 	   144.57	   620.85	 11
  3	 E 	   209.09	   570.33	 10
  4	 L 	   265.63	   505.81	  9
  5	 A 	   301.15	   449.27	  8
  6	 G 	   329.66	   413.75	  7
  7	 C 	   409.75	   385.24	  6
  8	 T 	   460.27	   305.15	  5
  9	 A 	   495.79	   254.63	  4
 10	 D 	   553.30	   219.11	  3
 11	 F 	   626.84	   161.60	  2
 12	 R 	        -  	    88.06	  1  _
```

**5. IPI00298281 Laminin subunit gamma-1**

| Sequence | Charge | M+H+ | XCorr | DeltaCn |
| --- | --- | --- | --- | --- |
| R.SQECYFDPELYR.S | 2 | 1607.695 | 2.997 | 0.373 |

+1 charge

```
		      B   	     Y
  1	 S 	    88.04	        -  	 12
  2	 Q 	   216.10	  1519.79	 11
  3	 E 	   345.14	  1391.73	 10
  4	 C 	   505.31	  1262.69	  9
  5	 Y 	   668.37	  1102.52	  8
  6	 F 	   815.44	   939.46	  7
  7	 D 	   930.46	   792.39	  6
  8	 P 	  1027.52	   677.36	  5
  9	 E 	  1156.56	   580.31	  4
 10	 L 	  1269.64	   451.27	  3
 11	 Y 	  1432.71	   338.18	  2
 12	 R 	        -  	   175.12	  1  _
+2 charge
		      B   	     Y
  1	 S 	    44.52	        -  	 12
  2	 Q 	   108.55	   760.40	 11
  3	 E 	   173.07	   696.37	 10
  4	 C 	   253.16	   631.85	  9
  5	 Y 	   334.69	   551.76	  8
  6	 F 	   408.22	   470.23	  7
  7	 D 	   465.74	   396.70	  6
  8	 P 	   514.26	   339.18	  5
  9	 E 	   578.78	   290.66	  4
 10	 L 	   635.33	   226.14	  3
 11	 Y 	   716.86	   169.59	  2
 12	 R 	        -  	    88.06	  1  _
```

**6. IPI00019988 N-sulphoglucosamine sulphohydrolase**

| Sequence | Charge | M+H+ | XCorr | DeltaCn |
| --- | --- | --- | --- | --- |
| R.SLPLLLSQAGVR.T | 2 | 1254.504 | 3.031 | 0.407 |

+1 charge

```
		      B   	     Y
  1	 S 	    88.04	        -  	 12
  2	 L 	   201.12	  1166.73	 11
  3	 P 	   298.18	  1053.64	 10
  4	 L 	   411.26	   956.59	  9
  5	 L 	   524.34	   843.50	  8
  6	 L 	   637.43	   730.42	  7
  7	 S 	   724.46	   617.34	  6
  8	 Q 	   852.52	   530.30	  5
  9	 A 	   923.56	   402.25	  4
 10	 G 	   980.58	   331.21	  3
 11	 V 	  1079.65	   274.19	  2
 12	 R 	        -  	   175.12	  1  _
+2 charge
		      B   	     Y
  1	 S 	    44.52	        -  	 12
  2	 L 	   101.07	   583.87	 11
  3	 P 	   149.59	   527.32	 10
  4	 L 	   206.13	   478.80	  9
  5	 L 	   262.68	   422.26	  8
  6	 L 	   319.22	   365.71	  7
  7	 S 	   362.73	   309.17	  6
  8	 Q 	   426.76	   265.66	  5
  9	 A 	   462.28	   201.63	  4
 10	 G 	   490.79	   166.11	  3
 11	 V 	   540.33	   137.60	  2
 12	 R 	        -  	    88.06	  1  _
```

**7. IPI00291866 Plasma protease C1 inhibitor**

| Sequence | Charge | M+H+ | XCorr | DeltaCn |
| --- | --- | --- | --- | --- |
| K.GVTSVSQIFHSPDLAIR.D | 2 | 1828.062 | 3.338 | 0.374 |

+1 charge

```
		      B   	     Y
  1	 G 	    58.03	        -  	 17
  2	 V 	   157.10	  1769.95	 16
  3	 T 	   258.14	  1670.89	 15
  4	 S 	   345.18	  1569.84	 14
  5	 V 	   444.25	  1482.81	 13
  6	 S 	   531.28	  1383.74	 12
  7	 Q 	   659.34	  1296.71	 11
  8	 I 	   772.42	  1168.65	 10
  9	 F 	   919.49	  1055.56	  9
 10	 H 	  1056.55	   908.49	  8
 11	 S 	  1143.58	   771.44	  7
 12	 P 	  1240.63	   684.40	  6
 13	 D 	  1355.66	   587.35	  5
 14	 L 	  1468.74	   472.32	  4
 15	 A 	  1539.78	   359.24	  3
 16	 I 	  1652.86	   288.20	  2
 17	 R 	        -  	   175.12	  1  _
+2 charge
		      B   	     Y
  1	 G 	    29.52	        -  	 17
  2	 V 	    79.05	   885.48	 16
  3	 T 	   129.58	   835.95	 15
  4	 S 	   173.09	   785.42	 14
  5	 V 	   222.63	   741.91	 13
  6	 S 	   266.14	   692.37	 12
  7	 Q 	   330.17	   648.86	 11
  8	 I 	   386.71	   584.83	 10
  9	 F 	   460.25	   528.29	  9
 10	 H 	   528.78	   454.75	  8
 11	 S 	   572.29	   386.22	  7
 12	 P 	   620.82	   342.71	  6
 13	 D 	   678.33	   294.18	  5
 14	 L 	   734.88	   236.67	  4
 15	 A 	   770.39	   180.12	  3
 16	 I 	   826.94	   144.61	  2
 17	 R 	        -  	    88.06	  1  _
```

**8. IPI00005732 Isoform 1 of Activin receptor type-1B**

| Sequence | Charge | M+H+ | XCorr | DeltaCn |
| --- | --- | --- | --- | --- |
| R.TCIPKVELVPAGKPFYCLSSEDLR.N | 3 | 2781.184 | 4.794 | 0.583 |

+1 charge

```
		      B   	     Y
  1	 T 	   102.05	        -  	 24
  2	 C 	   262.22	  2678.64	 23
  3	 I 	   375.30	  2518.47	 22
  4	 P 	   472.36	  2405.39	 21
  5	 K 	   600.45	  2308.34	 20
  6	 V 	   699.52	  2180.24	 19
  7	 E 	   828.56	  2081.17	 18
  8	 L 	   941.65	  1952.13	 17
  9	 V 	  1040.72	  1839.05	 16
 10	 P 	  1137.77	  1739.98	 15
 11	 A 	  1208.81	  1642.92	 14
 12	 G 	  1265.83	  1571.89	 13
 13	 K 	  1393.92	  1514.87	 12
 14	 P 	  1490.97	  1386.77	 11
 15	 F 	  1638.04	  1289.72	 10
 16	 Y 	  1801.11	  1142.65	  9
 17	 C 	  1961.27	   979.59	  8
 18	 L 	  2074.36	   819.42	  7
 19	 S 	  2161.39	   706.34	  6
 20	 S 	  2248.42	   619.30	  5
 21	 E 	  2377.46	   532.27	  4
 22	 D 	  2492.49	   403.23	  3
 23	 L 	  2605.57	   288.20	  2
 24	 R 	        -  	   175.12	  1  _


+2 charge
		      B   	     Y
  1	 T 	    51.53	        -  	 24
  2	 C 	   131.61	  1339.82	 23
  3	 I 	   188.16	  1259.74	 22
  4	 P 	   236.68	  1203.20	 21
  5	 K 	   300.73	  1154.67	 20
  6	 V 	   350.26	  1090.62	 19
  7	 E 	   414.79	  1041.09	 18
  8	 L 	   471.33	   976.57	 17
  9	 V 	   520.86	   920.03	 16
 10	 P 	   569.39	   870.49	 15
 11	 A 	   604.91	   821.97	 14
 12	 G 	   633.42	   786.45	 13
 13	 K 	   697.46	   757.94	 12
 14	 P 	   745.99	   693.89	 11
 15	 F 	   819.53	   645.36	 10
 16	 Y 	   901.06	   571.83	  9
 17	 C 	   981.14	   490.30	  8
 18	 L 	  1037.68	   410.21	  7
 19	 S 	  1081.20	   353.67	  6
 20	 S 	  1124.71	   310.16	  5
 21	 E 	  1189.23	   266.64	  4
 22	 D 	  1246.75	   202.12	  3
 23	 L 	  1303.29	   144.61	  2
 24	 R 	        -  	    88.06	  1  _


+3 charge
		      B   	     Y
  1	 T 	    34.69	        -  	 24
  2	 C 	    88.08	   893.55	 23
  3	 I 	   125.77	   840.16	 22
  4	 P 	   158.12	   802.47	 21
  5	 K 	   200.82	   770.12	 20
  6	 V 	   233.84	   727.42	 19
  7	 E 	   276.86	   694.40	 18
  8	 L 	   314.55	   651.38	 17
  9	 V 	   347.58	   613.69	 16
 10	 P 	   379.93	   580.66	 15
 11	 A 	   403.61	   548.31	 14
 12	 G 	   422.61	   524.63	 13
 13	 K 	   465.31	   505.63	 12
 14	 P 	   497.66	   462.93	 11
 15	 F 	   546.69	   430.58	 10
 16	 Y 	   601.04	   381.55	  9
 17	 C 	   654.43	   327.20	  8
 18	 L 	   692.12	   273.81	  7
 19	 S 	   721.13	   236.12	  6
 20	 S 	   750.14	   207.11	  5
 21	 E 	   793.16	   178.10	  4
 22	 D 	   831.50	   135.08	  3
 23	 L 	   869.20	    96.74	  2
 24	 R 	        -  	    59.04	  1  _
```

**9. IPI00744702 Isoform 1 of Multivesicular body subunit 12A**

| Sequence | Charge | M+H+ | XCorr | DeltaCn |
| --- | --- | --- | --- | --- |
| K.LLPLGATDTAVFDVR.L | 2 | 1588.83 | 3.092 | 0.38 |

+1 charge

```
		      B   	     Y
  1	 L 	   114.09	        -  	 15
  2	 L 	   227.18	  1474.79	 14
  3	 P 	   324.23	  1361.71	 13
  4	 L 	   437.31	  1264.65	 12
  5	 G 	   494.33	  1151.57	 11
  6	 A 	   565.37	  1094.55	 10
  7	 T 	   666.42	  1023.51	  9
  8	 D 	   781.45	   922.46	  8
  9	 T 	   882.49	   807.44	  7
 10	 A 	   953.53	   706.39	  6
 11	 V 	  1052.60	   635.35	  5
 12	 F 	  1199.67	   536.28	  4
 13	 D 	  1314.69	   389.21	  3
 14	 V 	  1413.76	   274.19	  2
 15	 R 	        -  	   175.12	  1  _
+2 charge
		      B   	     Y
  1	 L 	    57.55	        -  	 15
  2	 L 	   114.09	   737.90	 14
  3	 P 	   162.62	   681.36	 13
  4	 L 	   219.16	   632.83	 12
  5	 G 	   247.67	   576.29	 11
  6	 A 	   283.19	   547.78	 10
  7	 T 	   333.71	   512.26	  9
  8	 D 	   391.23	   461.74	  8
  9	 T 	   441.75	   404.22	  7
 10	 A 	   477.27	   353.70	  6
 11	 V 	   526.80	   318.18	  5
 12	 F 	   600.34	   268.64	  4
 13	 D 	   657.85	   195.11	  3
 14	 V 	   707.38	   137.60	  2
 15	 R 	        -  	    88.06	  1  _
```

**10. IPI00306543 Growth/differentiation factor 15**

| Sequence | Charge | M+H+ | XCorr | DeltaCn |
| --- | --- | --- | --- | --- |
| R.ASLEDLGWADWVLSPR.E | 2 | 1816.006 | 4.429 | 0.489 |

+1 charge

```
		      B   	     Y
  1	 A 	    72.04	        -  	 16
  2	 S 	   159.08	  1743.87	 15
  3	 L 	   272.16	  1656.84	 14
  4	 E 	   401.20	  1543.75	 13
  5	 D 	   516.23	  1414.71	 12
  6	 L 	   629.31	  1299.68	 11
  7	 G 	   686.34	  1186.60	 10
  8	 W 	   872.41	  1129.58	  9
  9	 A 	   943.45	   943.50	  8
 10	 D 	  1058.48	   872.46	  7
 11	 W 	  1244.56	   757.44	  6
 12	 V 	  1343.63	   571.36	  5
 13	 L 	  1456.71	   472.29	  4
 14	 S 	  1543.74	   359.20	  3
 15	 P 	  1640.80	   272.17	  2
 16	 R 	        -  	   175.12	  1  _
+2 charge
		      B   	     Y
  1	 A 	    36.53	        -  	 16
  2	 S 	    80.04	   872.44	 15
  3	 L 	   136.58	   828.92	 14
  4	 E 	   201.11	   772.38	 13
  5	 D 	   258.62	   707.86	 12
  6	 L 	   315.16	   650.35	 11
  7	 G 	   343.67	   593.80	 10
  8	 W 	   436.71	   565.29	  9
  9	 A 	   472.23	   472.25	  8
 10	 D 	   529.74	   436.73	  7
 11	 W 	   622.78	   379.22	  6
 12	 V 	   672.32	   286.18	  5
 13	 L 	   728.86	   236.65	  4
 14	 S 	   772.38	   180.11	  3
 15	 P 	   820.90	   136.59	  2
 16	 R 	        -  	    88.06	  1  _
```

**11. IPI00010949 Isoform 1 of Sialate O-acetylesterase**

| Sequence | Charge | M+H+ | XCorr | DeltaCn |
| --- | --- | --- | --- | --- |
| R.FFPFGLVQLSSDLSK.K | 2 | 1685.944 | 3.261 | 0.365 |

+1 charge

```
		      B   	     Y
  1	 F 	   148.08	        -  	 15
  2	 F 	   295.14	  1537.83	 14
  3	 P 	   392.20	  1390.76	 13
  4	 F 	   539.27	  1293.70	 12
  5	 G 	   596.29	  1146.64	 11
  6	 L 	   709.37	  1089.62	 10
  7	 V 	   808.44	   976.53	  9
  8	 Q 	   936.50	   877.46	  8
  9	 L 	  1049.58	   749.40	  7
 10	 S 	  1136.61	   636.32	  6
 11	 S 	  1223.65	   549.29	  5
 12	 D 	  1338.67	   462.26	  4
 13	 L 	  1451.76	   347.23	  3
 14	 S 	  1538.79	   234.14	  2
 15	 K 	        -  	   147.11	  1  _
+2 charge
		      B   	     Y
  1	 F 	    74.54	        -  	 15
  2	 F 	   148.08	   769.42	 14
  3	 P 	   196.60	   695.88	 13
  4	 F 	   270.14	   647.36	 12
  5	 G 	   298.65	   573.82	 11
  6	 L 	   355.19	   545.31	 10
  7	 V 	   404.72	   488.77	  9
  8	 Q 	   468.75	   439.23	  8
  9	 L 	   525.29	   375.21	  7
 10	 S 	   568.81	   318.66	  6
 11	 S 	   612.33	   275.15	  5
 12	 D 	   669.84	   231.63	  4
 13	 L 	   726.38	   174.12	  3
 14	 S 	   769.90	   117.58	  2
 15	 K 	        -  	    74.06	  1  _
```

**12. IPI00017601 Ceruloplasmin**

| Sequence | Charge | M+H+ | XCorr | DeltaCn |
| --- | --- | --- | --- | --- |
| R.FNKNNEGTYYSPNYNPQSR.S | 3 | 2294.381 | 3.8 | 0.265 |

+1 charge

```
		      B   	     Y
  1	 F 	   148.08	        -  	 19
  2	 N 	   262.12	  2145.96	 18
  3	 K 	   390.21	  2031.92	 17
  4	 N 	   504.26	  1903.82	 16
  5	 N 	   618.30	  1789.78	 15
  6	 E 	   747.34	  1675.73	 14
  7	 G 	   804.36	  1546.69	 13
  8	 T 	   905.41	  1489.67	 12
  9	 Y 	  1068.47	  1388.62	 11
 10	 Y 	  1231.54	  1225.56	 10
 11	 S 	  1318.57	  1062.50	  9
 12	 P 	  1415.62	   975.46	  8
 13	 N 	  1529.67	   878.41	  7
 14	 Y 	  1692.73	   764.37	  6
 15	 N 	  1806.77	   601.31	  5
 16	 P 	  1903.82	   487.26	  4
 17	 Q 	  2031.88	   390.21	  3
 18	 S 	  2118.92	   262.15	  2
 19	 R 	        -  	   175.12	  1  _
+2 charge
		      B   	     Y
  1	 F 	    74.54	        -  	 19
  2	 N 	   131.56	  1073.48	 18
  3	 K 	   195.61	  1016.46	 17
  4	 N 	   252.63	   952.41	 16
  5	 N 	   309.65	   895.39	 15
  6	 E 	   374.17	   838.37	 14
  7	 G 	   402.69	   773.85	 13
  8	 T 	   453.21	   745.34	 12
  9	 Y 	   534.74	   694.82	 11
 10	 Y 	   616.27	   613.28	 10
 11	 S 	   659.79	   531.75	  9
 12	 P 	   708.31	   488.24	  8
 13	 N 	   765.34	   439.71	  7
 14	 Y 	   846.87	   382.69	  6
 15	 N 	   903.89	   301.16	  5
 16	 P 	   952.42	   244.13	  4
 17	 Q 	  1016.45	   195.61	  3
 18	 S 	  1059.96	   131.58	  2
 19	 R 	        -  	    88.06	  1  _


+3 charge
		      B   	     Y
  1	 F 	    50.03	        -  	 19
  2	 N 	    88.04	   715.99	 18
  3	 K 	   130.74	   677.98	 17
  4	 N 	   168.76	   635.28	 16
  5	 N 	   206.77	   597.26	 15
  6	 E 	   249.79	   559.25	 14
  7	 G 	   268.79	   516.24	 13
  8	 T 	   302.48	   497.23	 12
  9	 Y 	   356.83	   463.55	 11
 10	 Y 	   411.18	   409.19	 10
 11	 S 	   440.19	   354.84	  9
 12	 P 	   472.55	   325.83	  8
 13	 N 	   510.56	   293.48	  7
 14	 Y 	   564.91	   255.46	  6
 15	 N 	   602.93	   201.11	  5
 16	 P 	   635.28	   163.09	  4
 17	 Q 	   677.97	   130.74	  3
 18	 S 	   706.98	    88.06	  2
 19	 R 	        -  	    59.04	  1  _
```

**13. IPI00019954 Cystatin-M**

| Sequence | Charge | M+H+ | XCorr | DeltaCn |
| --- | --- | --- | --- | --- |
| R.VTGDHVDLTTCPLAAGAQQEKLR.C | 3 | 2481.741 | 4.842 | 0.546 |

+1 charge

```
		      B   	     Y
  1	 V 	   100.08	        -  	 23
  2	 T 	   201.12	  2381.32	 22
  3	 G 	   258.14	  2280.27	 21
  4	 D 	   373.17	  2223.25	 20
  5	 H 	   510.23	  2108.23	 19
  6	 V 	   609.30	  1971.17	 18
  7	 D 	   724.33	  1872.10	 17
  8	 L 	   837.41	  1757.07	 16
  9	 T 	   938.46	  1643.99	 15
 10	 T 	  1039.51	  1542.94	 14
 11	 C 	  1199.67	  1441.89	 13
 12	 P 	  1296.72	  1281.73	 12
 13	 L 	  1409.81	  1184.67	 11
 14	 A 	  1480.84	  1071.59	 10
 15	 A 	  1551.88	  1000.55	  9
 16	 G 	  1608.90	   929.52	  8
 17	 A 	  1679.94	   872.49	  7
 18	 Q 	  1808.00	   801.46	  6
 19	 Q 	  1936.06	   673.40	  5
 20	 E 	  2065.10	   545.34	  4
 21	 K 	  2193.20	   416.30	  3
 22	 L 	  2306.28	   288.20	  2
 23	 R 	        -  	   175.12	  1  _


+2 charge
		      B   	     Y
  1	 V 	    50.54	        -  	 23
  2	 T 	   101.07	  1191.16	 22
  3	 G 	   129.58	  1140.64	 21
  4	 D 	   187.09	  1112.13	 20
  5	 H 	   255.62	  1054.62	 19
  6	 V 	   305.15	   986.09	 18
  7	 D 	   362.67	   936.55	 17
  8	 L 	   419.21	   879.04	 16
  9	 T 	   469.73	   822.50	 15
 10	 T 	   520.26	   771.97	 14
 11	 C 	   600.34	   721.45	 13
 12	 P 	   648.87	   641.37	 12
 13	 L 	   705.41	   592.84	 11
 14	 A 	   740.93	   536.30	 10
 15	 A 	   776.44	   500.78	  9
 16	 G 	   804.96	   465.26	  8
 17	 A 	   840.47	   436.75	  7
 18	 Q 	   904.50	   401.23	  6
 19	 Q 	   968.53	   337.20	  5
 20	 E 	  1033.05	   273.17	  4
 21	 K 	  1097.10	   208.65	  3
 22	 L 	  1153.64	   144.61	  2
 23	 R 	        -  	    88.06	  1  _
+3 charge
		      B   	     Y
  1	 V 	    34.03	        -  	 23
  2	 T 	    67.71	   794.45	 22
  3	 G 	    86.72	   760.76	 21
  4	 D 	   125.06	   741.76	 20
  5	 H 	   170.75	   703.41	 19
  6	 V 	   203.77	   657.73	 18
  7	 D 	   242.11	   624.70	 17
  8	 L 	   279.81	   586.36	 16
  9	 T 	   313.49	   548.67	 15
 10	 T 	   347.17	   514.98	 14
 11	 C 	   400.56	   481.30	 13
 12	 P 	   432.91	   427.91	 12
 13	 L 	   470.61	   395.56	 11
 14	 A 	   494.29	   357.87	 10
 15	 A 	   517.97	   334.19	  9
 16	 G 	   536.97	   310.51	  8
 17	 A 	   560.65	   291.50	  7
 18	 Q 	   603.34	   267.82	  6
 19	 Q 	   646.02	   225.14	  5
 20	 E 	   689.04	   182.45	  4
 21	 K 	   731.74	   139.44	  3
 22	 L 	   769.43	    96.74	  2
 23	 R 	        -  	    59.04	  1  _
```

**14. IPI00872780 Annexin A4**

| Sequence | Charge | M+H+ | XCorr | DeltaCn |
| --- | --- | --- | --- | --- |
| K.AASGFNAMEDAQTLR.K | 2 | 1582.721 | 3.279 | 0.434 |

+1 charge

```
		      B   	     Y
  1	 A 	    72.04	        -  	 15
  2	 A 	   143.08	  1510.70	 14
  3	 S 	   230.11	  1439.66	 13
  4	 G 	   287.13	  1352.63	 12
  5	 F 	   434.20	  1295.60	 11
  6	 N 	   548.25	  1148.54	 10
  7	 A 	   619.28	  1034.49	  9
  8	 M 	   750.32	   963.46	  8
  9	 E 	   879.37	   832.42	  7
 10	 D 	   994.39	   703.37	  6
 11	 A 	  1065.43	   588.35	  5
 12	 Q 	  1193.49	   517.31	  4
 13	 T 	  1294.54	   389.25	  3
 14	 L 	  1407.62	   288.20	  2
 15	 R 	        -  	   175.12	  1  _
+2 charge
		      B   	     Y
  1	 A 	    36.53	        -  	 15
  2	 A 	    72.04	   755.85	 14
  3	 S 	   115.56	   720.33	 13
  4	 G 	   144.07	   676.82	 12
  5	 F 	   217.61	   648.31	 11
  6	 N 	   274.63	   574.77	 10
  7	 A 	   310.15	   517.75	  9
  8	 M 	   375.67	   482.23	  8
  9	 E 	   440.19	   416.71	  7
 10	 D 	   497.70	   352.19	  6
 11	 A 	   533.22	   294.68	  5
 12	 Q 	   597.25	   259.16	  4
 13	 T 	   647.77	   195.13	  3
 14	 L 	   704.31	   144.61	  2
 15	 R 	        -  	    88.06	  1  _
```

**15. IPI00384401 Myosin-reactive immunoglobulin kappa chain variable region (Fragment)**

| Sequence | Charge | M+H+ | XCorr | DeltaCn |
| --- | --- | --- | --- | --- |
| - .EIVMTQSPATLSVSPGER.A | 2 | 1903.147 | 4.604 | 0.466 |

+1 charge

```
		      B   	     Y
  1	 E 	   130.05	        -  	 18
  2	 I 	   243.13	  1772.92	 17
  3	 V 	   342.20	  1659.84	 16
  4	 M 	   473.24	  1560.77	 15
  5	 T 	   574.29	  1429.73	 14
  6	 Q 	   702.35	  1328.68	 13
  7	 S 	   789.38	  1200.62	 12
  8	 P 	   886.43	  1113.59	 11
  9	 A 	   957.47	  1016.54	 10
 10	 T 	  1058.52	   945.50	  9
 11	 L 	  1171.60	   844.45	  8
 12	 S 	  1258.63	   731.37	  7
 13	 V 	  1357.70	   644.34	  6
 14	 S 	  1444.74	   545.27	  5
 15	 P 	  1541.79	   458.24	  4
 16	 G 	  1598.81	   361.18	  3
 17	 E 	  1727.85	   304.16	  2
 18	 R 	        -  	   175.12	  1  _
+2 charge
		      B   	     Y
  1	 E 	    65.53	        -  	 18
  2	 I 	   122.07	   886.96	 17
  3	 V 	   171.60	   830.42	 16
  4	 M 	   237.13	   780.89	 15
  5	 T 	   287.65	   715.37	 14
  6	 Q 	   351.68	   664.84	 13
  7	 S 	   395.19	   600.81	 12
  8	 P 	   443.72	   557.30	 11
  9	 A 	   479.24	   508.77	 10
 10	 T 	   529.76	   473.25	  9
 11	 L 	   586.31	   422.73	  8
 12	 S 	   629.82	   366.19	  7
 13	 V 	   679.36	   322.67	  6
 14	 S 	   722.87	   273.14	  5
 15	 P 	   771.40	   229.62	  4
 16	 G 	   799.91	   181.10	  3
 17	 E 	   864.43	   152.58	  2
 18	 R 	        -  	    88.06	  1  _
```

**16. IPI00387113 Ig kappa chain V-III region B6**

| Sequence | Charge | M+H+ | XCorr | DeltaCn |
| --- | --- | --- | --- | --- |
| - .ZIVLTZSPGTLSLSPGZR.A | 2 | 1884.616 | 4.108 | 0.657 |

+1 charge

```
		      B   	     Y
  1	 Z 	   129.56	        -  	 18
  2	 I 	   242.64	  1754.96	 17
  3	 V 	   341.71	  1641.88	 16
  4	 L 	   454.79	  1542.81	 15
  5	 T 	   555.84	  1429.73	 14
  6	 Z 	   684.39	  1328.68	 13
  7	 S 	   771.42	  1200.13	 12
  8	 P 	   868.48	  1113.10	 11
  9	 G 	   925.50	  1016.05	 10
 10	 T 	  1026.55	   959.02	  9
 11	 L 	  1139.63	   857.98	  8
 12	 S 	  1226.66	   744.89	  7
 13	 L 	  1339.75	   657.86	  6
 14	 S 	  1426.78	   544.78	  5
 15	 P 	  1523.83	   457.74	  4
 16	 G 	  1580.85	   360.69	  3
 17	 Z 	  1709.40	   303.67	  2
 18	 R 	        -  	   175.12	  1  _
+2 charge
		      B   	     Y
  1	 Z 	    65.28	        -  	 18
  2	 I 	   121.82	   877.99	 17
  3	 V 	   171.36	   821.44	 16
  4	 L 	   227.90	   771.91	 15
  5	 T 	   278.42	   715.37	 14
  6	 Z 	   342.70	   664.84	 13
  7	 S 	   386.22	   600.57	 12
  8	 P 	   434.74	   557.05	 11
  9	 G 	   463.25	   508.53	 10
 10	 T 	   513.78	   480.02	  9
 11	 L 	   570.32	   429.49	  8
 12	 S 	   613.83	   372.95	  7
 13	 L 	   670.38	   329.43	  6
 14	 S 	   713.89	   272.89	  5
 15	 P 	   762.42	   229.38	  4
 16	 G 	   790.93	   180.85	  3
 17	 Z 	   855.21	   152.34	  2
 18	 R 	        -  	    88.06	  1  _
```

**17. IPI00879999 ATP-dependent RNA helicase DDX54**

| Sequence | Charge | M+H+ | XCorr | DeltaCn |
| --- | --- | --- | --- | --- |
| R.DQEFYIPYRPKDFDSER.G | 2 | 2206.355 | 3.947 | 0.322 |

+1 charge

```
		      B   	     Y
  1	 D 	   116.03	        -  	 17
  2	 Q 	   244.09	  2090.00	 16
  3	 E 	   373.14	  1961.94	 15
  4	 F 	   520.20	  1832.90	 14
  5	 Y 	   683.27	  1685.83	 13
  6	 I 	   796.35	  1522.76	 12
  7	 P 	   893.40	  1409.68	 11
  8	 Y 	  1056.47	  1312.63	 10
  9	 R 	  1212.57	  1149.56	  9
 10	 P 	  1309.62	   993.46	  8
 11	 K 	  1437.72	   896.41	  7
 12	 D 	  1552.74	   768.32	  6
 13	 F 	  1699.81	   653.29	  5
 14	 D 	  1814.84	   506.22	  4
 15	 S 	  1901.87	   391.19	  3
 16	 E 	  2030.91	   304.16	  2
 17	 R 	        -  	   175.12	  1  _
+2 charge
		      B   	     Y
  1	 D 	    58.52	        -  	 17
  2	 Q 	   122.55	  1045.50	 16
  3	 E 	   187.07	   981.47	 15
  4	 F 	   260.61	   916.95	 14
  5	 Y 	   342.14	   843.42	 13
  6	 I 	   398.68	   761.89	 12
  7	 P 	   447.21	   705.34	 11
  8	 Y 	   528.74	   656.82	 10
  9	 R 	   606.79	   575.29	  9
 10	 P 	   655.31	   497.24	  8
 11	 K 	   719.36	   448.71	  7
 12	 D 	   776.88	   384.66	  6
 13	 F 	   850.41	   327.15	  5
 14	 D 	   907.92	   253.61	  4
 15	 S 	   951.44	   196.10	  3
 16	 E 	  1015.96	   152.58	  2
 17	 R 	        -  	    88.06	  1  _
```

**18. IPI00410487 Isoform 1 of Twisted gastrulation protein homolog 1**

| Sequence | Charge | M+H+ | XCorr | DeltaCn |
| --- | --- | --- | --- | --- |
| K.STVEELHEPIPSLFR.A | 2 | 1754.964 | 4.157 | 0.574 |

+1 charge

```
		      B   	     Y
  1	 S 	    88.04	        -  	 15
  2	 T 	   189.09	  1666.88	 14
  3	 V 	   288.16	  1565.83	 13
  4	 E 	   417.20	  1466.76	 12
  5	 E 	   546.24	  1337.72	 11
  6	 L 	   659.32	  1208.68	 10
  7	 H 	   796.38	  1095.59	  9
  8	 E 	   925.43	   958.54	  8
  9	 P 	  1022.48	   829.49	  7
 10	 I 	  1135.56	   732.44	  6
 11	 P 	  1232.62	   619.36	  5
 12	 S 	  1319.65	   522.30	  4
 13	 L 	  1432.73	   435.27	  3
 14	 F 	  1579.80	   322.19	  2
 15	 R 	        -  	   175.12	  1  _
+2 charge
		      B   	     Y
  1	 S 	    44.52	        -  	 15
  2	 T 	    95.05	   833.94	 14
  3	 V 	   144.58	   783.42	 13
  4	 E 	   209.10	   733.89	 12
  5	 E 	   273.62	   669.36	 11
  6	 L 	   330.17	   604.84	 10
  7	 H 	   398.70	   548.30	  9
  8	 E 	   463.22	   479.77	  8
  9	 P 	   511.74	   415.25	  7
 10	 I 	   568.29	   366.72	  6
 11	 P 	   616.81	   310.18	  5
 12	 S 	   660.33	   261.66	  4
 13	 L 	   716.87	   218.14	  3
 14	 F 	   790.40	   161.60	  2
 15	 R 	        -  	    88.06	  1  _
```

**19. IPI00007664 Plasma glutamate carboxypeptidase**

| Sequence | Charge | M+H+ | XCorr | DeltaCn |
| --- | --- | --- | --- | --- |
| R.TQGAVEAAKVGALASLIR.S | 2 | 1756.04 | 3.556 | 0.467 |

+1 charge

```
		      B   	     Y
  1	 T 	   102.05	        -  	 18
  2	 Q 	   230.11	  1653.96	 17
  3	 G 	   287.13	  1525.91	 16
  4	 A 	   358.17	  1468.88	 15
  5	 V 	   457.24	  1397.85	 14
  6	 E 	   586.28	  1298.78	 13
  7	 A 	   657.32	  1169.74	 12
  8	 A 	   728.36	  1098.70	 11
  9	 K 	   856.45	  1027.66	 10
 10	 V 	   955.52	   899.57	  9
 11	 G 	  1012.54	   800.50	  8
 12	 A 	  1083.58	   743.48	  7
 13	 L 	  1196.66	   672.44	  6
 14	 A 	  1267.70	   559.36	  5
 15	 S 	  1354.73	   488.32	  4
 16	 L 	  1467.82	   401.29	  3
 17	 I 	  1580.90	   288.20	  2
 18	 R 	        -  	   175.12	  1  _
+2 charge
		      B   	     Y
  1	 T 	    51.53	        -  	 18
  2	 Q 	   115.56	   827.49	 17
  3	 G 	   144.07	   763.46	 16
  4	 A 	   179.59	   734.95	 15
  5	 V 	   229.12	   699.43	 14
  6	 E 	   293.65	   649.89	 13
  7	 A 	   329.16	   585.37	 12
  8	 A 	   364.68	   549.85	 11
  9	 K 	   428.73	   514.33	 10
 10	 V 	   478.26	   450.29	  9
 11	 G 	   506.77	   400.75	  8
 12	 A 	   542.29	   372.24	  7
 13	 L 	   598.84	   336.72	  6
 14	 A 	   634.35	   280.18	  5
 15	 S 	   677.87	   244.66	  4
 16	 L 	   734.41	   201.15	  3
 17	 I 	   790.95	   144.61	  2
 18	 R 	        -  	    88.06	  1  _
```

**20. IPI00032258 Complement C4-A**

| Sequence | Charge | M+H+ | XCorr | DeltaCn |
| --- | --- | --- | --- | --- |
| R.VTASDPLDTLGSEGALSPGGVASLLRLPR.G | 3 | 2851.205 | 4.418 | 0.603 |

+1 charge

```
		      B   	     Y
  1	 V 	   100.08	        -  	 29
  2	 T 	   201.12	  2750.47	 28
  3	 A 	   272.16	  2649.42	 27
  4	 S 	   359.19	  2578.38	 26
  5	 D 	   474.22	  2491.35	 25
  6	 P 	   571.27	  2376.32	 24
  7	 L 	   684.36	  2279.27	 23
  8	 D 	   799.38	  2166.19	 22
  9	 T 	   900.43	  2051.16	 21
 10	 L 	  1013.51	  1950.11	 20
 11	 G 	  1070.54	  1837.03	 19
 12	 S 	  1157.57	  1780.01	 18
 13	 E 	  1286.61	  1692.98	 17
 14	 G 	  1343.63	  1563.93	 16
 15	 A 	  1414.67	  1506.91	 15
 16	 L 	  1527.75	  1435.87	 14
 17	 S 	  1614.79	  1322.79	 13
 18	 P 	  1711.84	  1235.76	 12
 19	 G 	  1768.86	  1138.71	 11
 20	 G 	  1825.88	  1081.68	 10
 21	 V 	  1924.95	  1024.66	  9
 22	 A 	  1995.99	   925.59	  8
 23	 S 	  2083.02	   854.56	  7
 24	 L 	  2196.10	   767.53	  6
 25	 L 	  2309.19	   654.44	  5
 26	 R 	  2465.29	   541.36	  4
 27	 L 	  2578.37	   385.26	  3
 28	 P 	  2675.43	   272.17	  2
 29	 R 	        -  	   175.12	  1  _


+2 charge
		      B   	     Y
  1	 V 	    50.54	        -  	 29
  2	 T 	   101.07	  1375.74	 28
  3	 A 	   136.58	  1325.21	 27
  4	 S 	   180.10	  1289.70	 26
  5	 D 	   237.61	  1246.18	 25
  6	 P 	   286.14	  1188.67	 24
  7	 L 	   342.68	  1140.14	 23
  8	 D 	   400.20	  1083.60	 22
  9	 T 	   450.72	  1026.08	 21
 10	 L 	   507.26	   975.56	 20
 11	 G 	   535.77	   919.02	 19
 12	 S 	   579.29	   890.51	 18
 13	 E 	   643.81	   846.99	 17
 14	 G 	   672.32	   782.47	 16
 15	 A 	   707.84	   753.96	 15
 16	 L 	   764.38	   718.44	 14
 17	 S 	   807.90	   661.90	 13
 18	 P 	   856.42	   618.38	 12
 19	 G 	   884.93	   569.86	 11
 20	 G 	   913.44	   541.35	 10
 21	 V 	   962.98	   512.83	  9
 22	 A 	   998.50	   463.30	  8
 23	 S 	  1042.01	   427.78	  7
 24	 L 	  1098.56	   384.27	  6
 25	 L 	  1155.10	   327.72	  5
 26	 R 	  1233.15	   271.18	  4
 27	 L 	  1289.69	   193.13	  3
 28	 P 	  1338.22	   136.59	  2
 29	 R 	        -  	    88.06	  1  _


+3 charge
		      B   	     Y
  1	 V 	    34.03	        -  	 29
  2	 T 	    67.71	   917.49	 28
  3	 A 	    91.39	   883.81	 27
  4	 S 	   120.40	   860.13	 26
  5	 D 	   158.74	   831.12	 25
  6	 P 	   191.10	   792.78	 24
  7	 L 	   228.79	   760.43	 23
  8	 D 	   267.13	   722.73	 22
  9	 T 	   300.82	   684.39	 21
 10	 L 	   338.51	   650.71	 20
 11	 G 	   357.52	   613.01	 19
 12	 S 	   386.53	   594.01	 18
 13	 E 	   429.54	   565.00	 17
 14	 G 	   448.55	   521.98	 16
 15	 A 	   472.23	   502.98	 15
 16	 L 	   509.92	   479.30	 14
 17	 S 	   538.93	   441.60	 13
 18	 P 	   571.28	   412.59	 12
 19	 G 	   590.29	   380.24	 11
 20	 G 	   609.30	   361.23	 10
 21	 V 	   642.32	   342.23	  9
 22	 A 	   666.00	   309.20	  8
 23	 S 	   695.01	   285.52	  7
 24	 L 	   732.71	   256.51	  6
 25	 L 	   770.40	   218.82	  5
 26	 R 	   822.43	   181.12	  4
 27	 L 	   860.13	   129.09	  3
 28	 P 	   892.48	    91.40	  2
 29	 R 	        -  	    59.04	  1  _
```

**21. IPI00873924 Isoform A of Interleukin-18-binding protein**

| Sequence | Charge | M+H+ | XCorr | DeltaCn |
| --- | --- | --- | --- | --- |
| R.STKDPCPSQPPVFPAAK.Q | 2 | 1828.052 | 3.982 | 0.587 |

+1 charge

```
		      B   	     Y
  1	 S 	    88.04	        -  	 17
  2	 T 	   189.09	  1740.01	 16
  3	 K 	   317.18	  1638.97	 15
  4	 D 	   432.21	  1510.87	 14
  5	 P 	   529.26	  1395.84	 13
  6	 C 	   689.43	  1298.79	 12
  7	 P 	   786.48	  1138.63	 11
  8	 S 	   873.51	  1041.57	 10
  9	 Q 	  1001.57	   954.54	  9
 10	 P 	  1098.62	   826.48	  8
 11	 P 	  1195.68	   729.43	  7
 12	 V 	  1294.74	   632.38	  6
 13	 F 	  1441.81	   533.31	  5
 14	 P 	  1538.87	   386.24	  4
 15	 A 	  1609.90	   289.19	  3
 16	 A 	  1680.94	   218.15	  2
 17	 K 	        -  	   147.11	  1  _
+2 charge
		      B   	     Y
  1	 S 	    44.52	        -  	 17
  2	 T 	    95.05	   870.51	 16
  3	 K 	   159.09	   819.99	 15
  4	 D 	   216.61	   755.94	 14
  5	 P 	   265.13	   698.43	 13
  6	 C 	   345.22	   649.90	 12
  7	 P 	   393.74	   569.82	 11
  8	 S 	   437.26	   521.29	 10
  9	 Q 	   501.29	   477.77	  9
 10	 P 	   549.82	   413.74	  8
 11	 P 	   598.34	   365.22	  7
 12	 V 	   647.88	   316.69	  6
 13	 F 	   721.41	   267.16	  5
 14	 P 	   769.94	   193.62	  4
 15	 A 	   805.45	   145.10	  3
 16	 A 	   840.97	   109.58	  2
 17	 K 	        -  	    74.06	  1  _
```

**22. IPI00003362 HSPA5 protein**

| Sequence | Charge | M+H+ | XCorr | DeltaCn |
| --- | --- | --- | --- | --- |
| R.TWNDPSVQQDIK.F | 2 | 1431.532 | 2.899 | 0.326 |

+1 charge

```
		      B   	     Y
  1	 T 	   102.05	        -  	 12
  2	 W 	   288.13	  1329.64	 11
  3	 N 	   402.18	  1143.56	 10
  4	 D 	   517.20	  1029.52	  9
  5	 P 	   614.26	   914.49	  8
  6	 S 	   701.29	   817.44	  7
  7	 V 	   800.36	   730.41	  6
  8	 Q 	   928.42	   631.34	  5
  9	 Q 	  1056.47	   503.28	  4
 10	 D 	  1171.50	   375.22	  3
 11	 I 	  1284.59	   260.20	  2
 12	 K 	        -  	   147.11	  1  _
+2 charge
		      B   	     Y
  1	 T 	    51.53	        -  	 12
  2	 W 	   144.57	   665.33	 11
  3	 N 	   201.59	   572.29	 10
  4	 D 	   259.11	   515.26	  9
  5	 P 	   307.63	   457.75	  8
  6	 S 	   351.15	   409.22	  7
  7	 V 	   400.68	   365.71	  6
  8	 Q 	   464.71	   316.17	  5
  9	 Q 	   528.74	   252.14	  4
 10	 D 	   586.25	   188.12	  3
 11	 I 	   642.80	   130.60	  2
 12	 K 	        -  	    74.06	  1  _
```

**23. IPI00884105 Lysosome-associated membrane glycoprotein 1**

| Sequence | Charge | M+H+ | XCorr | DeltaCn |
| --- | --- | --- | --- | --- |
| K.TVESITDIRADIDKK.Y | 2 | 1704.904 | 3.096 | 0.313 |

+1 charge

```
		      B   	     Y
  1	 T 	   102.05	        -  	 15
  2	 V 	   201.12	  1602.87	 14
  3	 E 	   330.17	  1503.80	 13
  4	 S 	   417.20	  1374.76	 12
  5	 I 	   530.28	  1287.73	 11
  6	 T 	   631.33	  1174.64	 10
  7	 D 	   746.36	  1073.59	  9
  8	 I 	   859.44	   958.57	  8
  9	 R 	  1015.54	   845.48	  7
 10	 A 	  1086.58	   689.38	  6
 11	 D 	  1201.61	   618.35	  5
 12	 I 	  1314.69	   503.32	  4
 13	 D 	  1429.72	   390.23	  3
 14	 K 	  1557.81	   275.21	  2
 15	 K 	        -  	   147.11	  1  _
+2 charge
		      B   	     Y
  1	 T 	    51.53	        -  	 15
  2	 V 	   101.07	   801.94	 14
  3	 E 	   165.59	   752.40	 13
  4	 S 	   209.10	   687.88	 12
  5	 I 	   265.64	   644.37	 11
  6	 T 	   316.17	   587.82	 10
  7	 D 	   373.68	   537.30	  9
  8	 I 	   430.22	   479.79	  8
  9	 R 	   508.27	   423.25	  7
 10	 A 	   543.79	   345.20	  6
 11	 D 	   601.31	   309.68	  5
 12	 I 	   657.85	   252.16	  4
 13	 D 	   715.36	   195.62	  3
 14	 K 	   779.41	   138.11	  2
 15	 K 	        -  	    74.06	  1  _
```

**24. IPI00024331 Tumor necrosis factor receptor superfamily member 14**

| Sequence | Charge | M+H+ | XCorr | DeltaCn |
| --- | --- | --- | --- | --- |
| K.CLQCQMCDPAMGLR.A | 2 | 1741.008 | 4.598 | 0.551 |

+1 charge

```
		      B   	     Y
  1	 C 	   161.17	        -  	 14
  2	 L 	   274.26	  1579.95	 13
  3	 Q 	   402.32	  1466.87	 12
  4	 C 	   562.48	  1338.81	 11
  5	 Q 	   690.54	  1178.65	 10
  6	 M 	   821.58	  1050.59	  9
  7	 C 	   981.74	   919.55	  8
  8	 D 	  1096.77	   759.38	  7
  9	 P 	  1193.82	   644.35	  6
 10	 A 	  1264.86	   547.30	  5
 11	 M 	  1395.90	   476.26	  4
 12	 G 	  1452.92	   345.22	  3
 13	 L 	  1566.01	   288.20	  2
 14	 R 	        -  	   175.12	  1  _
+2 charge
		      B   	     Y
  1	 C 	    81.09	        -  	 14
  2	 L 	   137.63	   790.48	 13
  3	 Q 	   201.66	   733.94	 12
  4	 C 	   281.74	   669.91	 11
  5	 Q 	   345.77	   589.83	 10
  6	 M 	   411.29	   525.80	  9
  7	 C 	   491.38	   460.28	  8
  8	 D 	   548.89	   380.19	  7
  9	 P 	   597.42	   322.68	  6
 10	 A 	   632.93	   274.15	  5
 11	 M 	   698.45	   238.64	  4
 12	 G 	   726.97	   173.12	  3
 13	 L 	   783.51	   144.61	  2
 14	 R 	        -  	    88.06	  1  _
```

**25. IPI00783024 Myosin-reactive immunoglobulin heavy chain variable region (Fragment)**

| Sequence | Charge | M+H+ | XCorr | DeltaCn |
| --- | --- | --- | --- | --- |
| - .EVQLVESGGGLVKPGGSLR.L | 2 | 1883.139 | 3.508 | 0.218 |

+1 charge

```
		      B   	     Y
  1	 E 	   130.05	        -  	 19
  2	 V 	   229.12	  1753.00	 18
  3	 Q 	   357.18	  1653.93	 17
  4	 L 	   470.26	  1525.87	 16
  5	 V 	   569.33	  1412.79	 15
  6	 E 	   698.37	  1313.72	 14
  7	 S 	   785.40	  1184.67	 13
  8	 G 	   842.43	  1097.64	 12
  9	 G 	   899.45	  1040.62	 11
 10	 G 	   956.47	   983.60	 10
 11	 L 	  1069.55	   926.58	  9
 12	 V 	  1168.62	   813.49	  8
 13	 K 	  1296.72	   714.43	  7
 14	 P 	  1393.77	   586.33	  6
 15	 G 	  1450.79	   489.28	  5
 16	 G 	  1507.81	   432.26	  4
 17	 S 	  1594.84	   375.24	  3
 18	 L 	  1707.93	   288.20	  2
 19	 R 	        -  	   175.12	  1  _
+2 charge
		      B   	     Y
  1	 E 	    65.53	        -  	 19
  2	 V 	   115.06	   877.00	 18
  3	 Q 	   179.09	   827.47	 17
  4	 L 	   235.63	   763.44	 16
  5	 V 	   285.17	   706.90	 15
  6	 E 	   349.69	   657.36	 14
  7	 S 	   393.21	   592.84	 13
  8	 G 	   421.72	   549.32	 12
  9	 G 	   450.23	   520.81	 11
 10	 G 	   478.74	   492.30	 10
 11	 L 	   535.28	   463.79	  9
 12	 V 	   584.81	   407.25	  8
 13	 K 	   648.86	   357.72	  7
 14	 P 	   697.39	   293.67	  6
 15	 G 	   725.90	   245.14	  5
 16	 G 	   754.41	   216.63	  4
 17	 S 	   797.93	   188.12	  3
 18	 L 	   854.47	   144.61	  2
 19	 R 	        -  	    88.06	  1  _
```

**26. IPI00019157 Chondroitin sulfate proteoglycan 4**

| Sequence | Charge | M+H+ | XCorr | DeltaCn |
| --- | --- | --- | --- | --- |
| R.GSLLLGGLDAEASR.H | 2 | 1359.511 | 3.635 | 0.426 |

+1 charge

```
		      B   	     Y
  1	 G 	    58.03	        -  	 14
  2	 S 	   145.06	  1301.71	 13
  3	 L 	   258.14	  1214.67	 12
  4	 L 	   371.23	  1101.59	 11
  5	 L 	   484.31	   988.51	 10
  6	 G 	   541.33	   875.42	  9
  7	 G 	   598.36	   818.40	  8
  8	 L 	   711.44	   761.38	  7
  9	 D 	   826.47	   648.29	  6
 10	 A 	   897.50	   533.27	  5
 11	 E 	  1026.55	   462.23	  4
 12	 A 	  1097.58	   333.19	  3
 13	 S 	  1184.62	   262.15	  2
 14	 R 	        -  	   175.12	  1  _
+2 charge
		      B   	     Y
  1	 G 	    29.52	        -  	 14
  2	 S 	    73.03	   651.36	 13
  3	 L 	   129.58	   607.84	 12
  4	 L 	   186.12	   551.30	 11
  5	 L 	   242.66	   494.76	 10
  6	 G 	   271.17	   438.21	  9
  7	 G 	   299.68	   409.70	  8
  8	 L 	   356.22	   381.19	  7
  9	 D 	   413.74	   324.65	  6
 10	 A 	   449.26	   267.14	  5
 11	 E 	   513.78	   231.62	  4
 12	 A 	   549.30	   167.10	  3
 13	 S 	   592.81	   131.58	  2
 14	 R 	        -  	    88.06	  1  _
```

**27. IPI00465431 Galectin-3**

| Sequence | Charge | M+H+ | XCorr | DeltaCn |
| --- | --- | --- | --- | --- |
| R.MLITILGTVKPNANR.I | 2 | 1642.004 | 3.532 | 0.386 |

+1 charge

```
		      B   	     Y
  1	 M 	   132.05	        -  	 15
  2	 L 	   245.13	  1509.91	 14
  3	 I 	   358.22	  1396.83	 13
  4	 T 	   459.26	  1283.74	 12
  5	 I 	   572.35	  1182.70	 11
  6	 L 	   685.43	  1069.61	 10
  7	 G 	   742.45	   956.53	  9
  8	 T 	   843.50	   899.51	  8
  9	 V 	   942.57	   798.46	  7
 10	 K 	  1070.66	   699.39	  6
 11	 P 	  1167.72	   571.29	  5
 12	 N 	  1281.76	   474.24	  4
 13	 A 	  1352.80	   360.20	  3
 14	 N 	  1466.84	   289.16	  2
 15	 R 	        -  	   175.12	  1  _
+2 charge
		      B   	     Y
  1	 M 	    66.53	        -  	 15
  2	 L 	   123.07	   755.46	 14
  3	 I 	   179.61	   698.92	 13
  4	 T 	   230.14	   642.38	 12
  5	 I 	   286.68	   591.85	 11
  6	 L 	   343.22	   535.31	 10
  7	 G 	   371.73	   478.77	  9
  8	 T 	   422.25	   450.26	  8
  9	 V 	   471.79	   399.73	  7
 10	 K 	   535.84	   350.20	  6
 11	 P 	   584.36	   286.15	  5
 12	 N 	   641.38	   237.62	  4
 13	 A 	   676.90	   180.60	  3
 14	 N 	   733.92	   145.08	  2
 15	 R 	        -  	    88.06	  1  _
```

**28. IPI00470361 Isoform 2 of Kin of IRRE-like protein 1**

| Sequence | Charge | M+H+ | XCorr | DeltaCn |
| --- | --- | --- | --- | --- |
| K.DGLALGMGQGLKAWPR.Y | 2 | 1670.961 | 2.971 | 0.412 |

+1 charge

```
		      B   	     Y
  1	 D 	   116.03	        -  	 16
  2	 G 	   173.06	  1554.86	 15
  3	 L 	   286.14	  1497.84	 14
  4	 A 	   357.18	  1384.75	 13
  5	 L 	   470.26	  1313.71	 12
  6	 G 	   527.28	  1200.63	 11
  7	 M 	   658.32	  1143.61	 10
  8	 G 	   715.34	  1012.57	  9
  9	 Q 	   843.40	   955.55	  8
 10	 G 	   900.42	   827.49	  7
 11	 L 	  1013.51	   770.47	  6
 12	 K 	  1141.60	   657.38	  5
 13	 A 	  1212.64	   529.29	  4
 14	 W 	  1398.72	   458.25	  3
 15	 P 	  1495.77	   272.17	  2
 16	 R 	        -  	   175.12	  1  _
+2 charge
		      B   	     Y
  1	 D 	    58.52	        -  	 16
  2	 G 	    87.03	   777.93	 15
  3	 L 	   143.57	   749.42	 14
  4	 A 	   179.09	   692.88	 13
  5	 L 	   235.63	   657.36	 12
  6	 G 	   264.14	   600.82	 11
  7	 M 	   329.67	   572.31	 10
  8	 G 	   358.18	   506.79	  9
  9	 Q 	   422.21	   478.28	  8
 10	 G 	   450.72	   414.25	  7
 11	 L 	   507.26	   385.74	  6
 12	 K 	   571.31	   329.20	  5
 13	 A 	   606.82	   265.15	  4
 14	 W 	   699.86	   229.63	  3
 15	 P 	   748.39	   136.59	  2
 16	 R 	        -  	    88.06	  1  _
```

**29. IPI00005526 PRO0419**

| Sequence | Charge | M+H+ | XCorr | DeltaCn |
| --- | --- | --- | --- | --- |
| R.RSELFILPQDDLDPPNFPCSR.P | 3 | 2517.772 | 4.083 | 0.263 |

+1 charge

```
		      B   	     Y
  1	 R 	   157.11	        -  	 21
  2	 S 	   244.14	  2360.26	 20
  3	 E 	   373.18	  2273.23	 19
  4	 L 	   486.27	  2144.18	 18
  5	 F 	   633.34	  2031.10	 17
  6	 I 	   746.42	  1884.03	 16
  7	 L 	   859.50	  1770.95	 15
  8	 P 	   956.56	  1657.86	 14
  9	 Q 	  1084.61	  1560.81	 13
 10	 D 	  1199.64	  1432.75	 12
 11	 D 	  1314.67	  1317.72	 11
 12	 L 	  1427.75	  1202.70	 10
 13	 D 	  1542.78	  1089.61	  9
 14	 P 	  1639.83	   974.59	  8
 15	 P 	  1736.89	   877.53	  7
 16	 N 	  1850.93	   780.48	  6
 17	 F 	  1998.00	   666.44	  5
 18	 P 	  2095.05	   519.37	  4
 19	 C 	  2255.21	   422.32	  3
 20	 S 	  2342.25	   262.15	  2
 21	 R 	        -  	   175.12	  1  _


+2 charge
		      B   	     Y
  1	 R 	    79.06	        -  	 21
  2	 S 	   122.57	  1180.63	 20
  3	 E 	   187.10	  1137.12	 19
  4	 L 	   243.64	  1072.59	 18
  5	 F 	   317.17	  1016.05	 17
  6	 I 	   373.71	   942.52	 16
  7	 L 	   430.26	   885.98	 15
  8	 P 	   478.78	   829.43	 14
  9	 Q 	   542.81	   780.91	 13
 10	 D 	   600.32	   716.88	 12
 11	 D 	   657.84	   659.37	 11
 12	 L 	   714.38	   601.85	 10
 13	 D 	   771.89	   545.31	  9
 14	 P 	   820.42	   487.80	  8
 15	 P 	   868.95	   439.27	  7
 16	 N 	   925.97	   390.74	  6
 17	 F 	   999.50	   333.72	  5
 18	 P 	  1048.03	   260.19	  4
 19	 C 	  1128.11	   211.66	  3
 20	 S 	  1171.63	   131.58	  2
 21	 R 	        -  	    88.06	  1  _
+3 charge
		      B   	     Y
  1	 R 	    53.04	        -  	 21
  2	 S 	    82.05	   787.42	 20
  3	 E 	   125.07	   758.41	 19
  4	 L 	   162.76	   715.40	 18
  5	 F 	   211.78	   677.70	 17
  6	 I 	   249.48	   628.68	 16
  7	 L 	   287.17	   590.99	 15
  8	 P 	   319.52	   553.29	 14
  9	 Q 	   362.21	   520.94	 13
 10	 D 	   400.55	   478.26	 12
 11	 D 	   438.89	   439.91	 11
 12	 L 	   476.59	   401.57	 10
 13	 D 	   514.93	   363.88	  9
 14	 P 	   547.28	   325.53	  8
 15	 P 	   579.63	   293.18	  7
 16	 N 	   617.65	   260.83	  6
 17	 F 	   666.67	   222.82	  5
 18	 P 	   699.02	   173.79	  4
 19	 C 	   752.41	   141.44	  3
 20	 S 	   781.42	    88.06	  2
 21	 R 	        -  	    59.04	  1  _
```

**30. IPI00005707 C-type mannose receptor 2**

| Sequence | Charge | M+H+ | XCorr | DeltaCn |
| --- | --- | --- | --- | --- |
| R.TLGDQLSLLLGAR.T | 2 | 1357.581 | 3.506 | 0.338 |

+1 charge

```
		      B   	     Y
  1	 T 	   102.05	        -  	 13
  2	 L 	   215.14	  1255.74	 12
  3	 G 	   272.16	  1142.65	 11
  4	 D 	   387.19	  1085.63	 10
  5	 Q 	   515.25	   970.60	  9
  6	 L 	   628.33	   842.55	  8
  7	 S 	   715.36	   729.46	  7
  8	 L 	   828.45	   642.43	  6
  9	 L 	   941.53	   529.35	  5
 10	 L 	  1054.61	   416.26	  4
 11	 G 	  1111.64	   303.18	  3
 12	 A 	  1182.67	   246.16	  2
 13	 R 	        -  	   175.12	  1  _
+2 charge
		      B   	     Y
  1	 T 	    51.53	        -  	 13
  2	 L 	   108.07	   628.37	 12
  3	 G 	   136.58	   571.83	 11
  4	 D 	   194.10	   543.32	 10
  5	 Q 	   258.13	   485.81	  9
  6	 L 	   314.67	   421.78	  8
  7	 S 	   358.18	   365.23	  7
  8	 L 	   414.73	   321.72	  6
  9	 L 	   471.27	   265.18	  5
 10	 L 	   527.81	   208.63	  4
 11	 G 	   556.32	   152.09	  3
 12	 A 	   591.84	   123.58	  2
 13	 R 	        -  	    88.06	  1  _
```

**31. IPI00011229 Cathepsin D**

| Sequence | Charge | M+H+ | XCorr | DeltaCn |
| --- | --- | --- | --- | --- |
| K.AIGAVPLIQGEYMIPCEKVSTLPAITLK.L | 3 | 3014.606 | 3.378 | 0.545 |

+1 charge

```
		      B   	     Y
  1	 A 	    72.04	        -  	 28
  2	 I 	   185.13	  2941.75	 27
  3	 G 	   242.15	  2828.66	 26
  4	 A 	   313.19	  2771.64	 25
  5	 V 	   412.26	  2700.61	 24
  6	 P 	   509.31	  2601.54	 23
  7	 L 	   622.39	  2504.48	 22
  8	 I 	   735.48	  2391.40	 21
  9	 Q 	   863.53	  2278.32	 20
 10	 G 	   920.56	  2150.26	 19
 11	 E 	  1049.60	  2093.24	 18
 12	 Y 	  1212.66	  1964.19	 17
 13	 M 	  1343.70	  1801.13	 16
 14	 I 	  1456.79	  1670.09	 15
 15	 P 	  1553.84	  1557.01	 14
 16	 C 	  1714.00	  1459.95	 13
 17	 E 	  1843.05	  1299.79	 12
 18	 K 	  1971.14	  1170.75	 11
 19	 V 	  2070.21	  1042.65	 10
 20	 S 	  2157.24	   943.58	  9
 21	 T 	  2258.29	   856.55	  8
 22	 L 	  2371.37	   755.50	  7
 23	 P 	  2468.43	   642.42	  6
 24	 A 	  2539.46	   545.37	  5
 25	 I 	  2652.55	   474.33	  4
 26	 T 	  2753.60	   361.24	  3
 27	 L 	  2866.68	   260.20	  2
 28	 K 	        -  	   147.11	  1  _


+2 charge
		      B   	     Y
  1	 A 	    36.53	        -  	 28
  2	 I 	    93.07	  1471.38	 27
  3	 G 	   121.58	  1414.84	 26
  4	 A 	   157.10	  1386.33	 25
  5	 V 	   206.63	  1350.81	 24
  6	 P 	   255.16	  1301.27	 23
  7	 L 	   311.70	  1252.75	 22
  8	 I 	   368.24	  1196.20	 21
  9	 Q 	   432.27	  1139.66	 20
 10	 G 	   460.78	  1075.63	 19
 11	 E 	   525.30	  1047.12	 18
 12	 Y 	   606.83	   982.60	 17
 13	 M 	   672.36	   901.07	 16
 14	 I 	   728.90	   835.55	 15
 15	 P 	   777.42	   779.01	 14
 16	 C 	   857.51	   730.48	 13
 17	 E 	   922.03	   650.40	 12
 18	 K 	   986.07	   585.88	 11
 19	 V 	  1035.61	   521.83	 10
 20	 S 	  1079.13	   472.29	  9
 21	 T 	  1129.65	   428.78	  8
 22	 L 	  1186.19	   378.25	  7
 23	 P 	  1234.72	   321.71	  6
 24	 A 	  1270.24	   273.19	  5
 25	 I 	  1326.78	   237.67	  4
 26	 T 	  1377.30	   181.13	  3
 27	 L 	  1433.84	   130.60	  2
 28	 K 	        -  	    74.06	  1  _


+3 charge
		      B   	     Y
  1	 A 	    24.69	        -  	 28
  2	 I 	    62.38	   981.25	 27
  3	 G 	    81.39	   943.56	 26
  4	 A 	   105.07	   924.55	 25
  5	 V 	   138.09	   900.87	 24
  6	 P 	   170.44	   867.85	 23
  7	 L 	   208.14	   835.50	 22
  8	 I 	   245.83	   797.81	 21
  9	 Q 	   288.52	   760.11	 20
 10	 G 	   307.52	   717.42	 19
 11	 E 	   350.54	   698.42	 18
 12	 Y 	   404.89	   655.40	 17
 13	 M 	   448.57	   601.05	 16
 14	 I 	   486.27	   557.37	 15
 15	 P 	   518.62	   519.67	 14
 16	 C 	   572.01	   487.32	 13
 17	 E 	   615.02	   433.93	 12
 18	 K 	   657.72	   390.92	 11
 19	 V 	   690.74	   348.22	 10
 20	 S 	   719.75	   315.20	  9
 21	 T 	   753.44	   286.19	  8
 22	 L 	   791.13	   252.51	  7
 23	 P 	   823.48	   214.81	  6
 24	 A 	   847.16	   182.46	  5
 25	 I 	   884.85	   158.78	  4
 26	 T 	   918.54	   121.09	  3
 27	 L 	   956.23	    87.40	  2
 28	 K 	        -  	    49.71	  1  _
```

**32. IPI00027497 Glucose-6-phosphate isomerase**

| Sequence | Charge | M+H+ | XCorr | DeltaCn |
| --- | --- | --- | --- | --- |
| K.ILLANFLAQTEALMR.G | 2 | 1705.058 | 3.831 | 0.525 |

+1 charge

```
		      B   	     Y
  1	 I 	   114.09	        -  	 15
  2	 L 	   227.18	  1590.87	 14
  3	 L 	   340.26	  1477.78	 13
  4	 A 	   411.30	  1364.70	 12
  5	 N 	   525.34	  1293.66	 11
  6	 F 	   672.41	  1179.62	 10
  7	 L 	   785.49	  1032.55	  9
  8	 A 	   856.53	   919.47	  8
  9	 Q 	   984.59	   848.43	  7
 10	 T 	  1085.64	   720.37	  6
 11	 E 	  1214.68	   619.32	  5
 12	 A 	  1285.72	   490.28	  4
 13	 L 	  1398.80	   419.24	  3
 14	 M 	  1529.84	   306.16	  2
 15	 R 	        -  	   175.12	  1  _
+2 charge
		      B   	     Y
  1	 I 	    57.55	        -  	 15
  2	 L 	   114.09	   795.94	 14
  3	 L 	   170.63	   739.40	 13
  4	 A 	   206.15	   682.85	 12
  5	 N 	   263.17	   647.33	 11
  6	 F 	   336.71	   590.31	 10
  7	 L 	   393.25	   516.78	  9
  8	 A 	   428.77	   460.24	  8
  9	 Q 	   492.80	   424.72	  7
 10	 T 	   543.32	   360.69	  6
 11	 E 	   607.84	   310.17	  5
 12	 A 	   643.36	   245.64	  4
 13	 L 	   699.90	   210.13	  3
 14	 M 	   765.42	   153.58	  2
 15	 R 	        -  	    88.06	  1  _
```

**33. IPI00010737 Thrombomodulin**

| Sequence | Charge | M+H+ | XCorr | DeltaCn |
| --- | --- | --- | --- | --- |
| R.SSVAADVISLLLNGDGGVGR.R | 2 | 1901.111 | 3.283 | 0.487 |

+1 charge

```
		      B   	     Y
  1	 S 	    88.04	        -	     20
  2	 S 	   175.07	  1812.98	 19
  3	 V 	   274.14	  1725.95	 18
  4	 A 	   345.18	  1626.88	 17
  5	 A 	   416.21	  1555.84	 16
  6	 D 	   531.24	  1484.81	 15
  7	 V 	   630.31	  1369.78	 14
  8	 I 	   743.39	  1270.71	 13
  9	 S 	   830.43	  1157.63	 12
 10	 L 	   943.51	  1070.60	 11
 11	 L 	  1056.59	   957.51	 10
 12	 L 	  1169.68	   844.43	  9
 13	 N 	  1283.72	   731.34	  8
 14	 G 	  1340.74	   617.30	  7
 15	 D 	  1455.77	   560.28	  6
 16	 G 	  1512.79	   445.25	  5
 17	 G 	  1569.81	   388.23	  4
 18	 V 	  1668.88	   331.21	  3
 19	 G 	  1725.90	   232.14	  2
 20	 R 	        -	        175.12	  1  _
```

+2 charge

```
		    B   	   Y
  1	 S 	    44.52	        -	 20
  2	 S 	    88.04	   906.99	 19
  3	 V 	   137.57	   863.48	 18
  4	 A 	   173.09	   813.94	 17
  5	 A 	   208.61	   778.43	 16
  6	 D 	   266.12	   742.91	 15
  7	 V 	   315.66	   685.39	 14
  8	 I 	   372.20	   635.86	 13
  9	 S 	   415.72	   579.32	 12
 10	 L 	   472.26	   535.80	 11
 11	 L 	   528.80	   479.26	 10
 12	 L 	   585.34	   422.72	  9
 13	 N 	   642.36	   366.18	  8
 14	 G 	   670.87	   309.15	  7
 15	 D 	   728.39	   280.64	  6
 16	 G 	   756.90	   223.13	  5
 17	 G 	   785.41	   194.62	  4
 18	 V 	   834.94	   166.11	  3
 19	 G 	   863.45	   116.57	  2
 20	 R 	        -	    88.06	  1  _
```

**34. IPI00295414 Collagen alpha-1(XV) chain**

| Protein ID | Sequence | Charge | M+H+ | XCorr | DeltaCn |
| --- | --- | --- | --- | --- | --- |
| IPI00295414 | R.FTGSLQQLTVHPDPR.T | 2 | 1696.888 | 3.628 | 0.479 |

+1 charge

```
		      B   	     Y
  1	 F 	   148.08	        -  	 15
  2	 T 	   249.12	  1548.81	 14
  3	 G 	   306.14	  1447.77	 13
  4	 S 	   393.18	  1390.74	 12
  5	 L 	   506.26	  1303.71	 11
  6	 Q 	   634.32	  1190.63	 10
  7	 Q 	   762.38	  1062.57	  9
  8	 L 	   875.46	   934.51	  8
  9	 T 	   976.51	   821.43	  7
 10	 V 	  1075.58	   720.38	  6
 11	 H 	  1212.64	   621.31	  5
 12	 P 	  1309.69	   484.25	  4
 13	 D 	  1424.72	   387.20	  3
 14	 P 	  1521.77	   272.17	  2
 15	 R 	        -  	   175.12	  1  _
+2 charge
		      B   	     Y
  1	 F 	    74.54	        -  	 15
  2	 T 	   125.07	   774.91	 14
  3	 G 	   153.58	   724.39	 13
  4	 S 	   197.09	   695.88	 12
  5	 L 	   253.63	   652.36	 11
  6	 Q 	   317.66	   595.82	 10
  7	 Q 	   381.69	   531.79	  9
  8	 L 	   438.23	   467.76	  8
  9	 T 	   488.76	   411.22	  7
 10	 V 	   538.29	   360.69	  6
 11	 H 	   606.82	   311.16	  5
 12	 P 	   655.35	   242.63	  4
 13	 D 	   712.86	   194.10	  3
 14	 P 	   761.39	   136.59	  2
 15	 R 	        -  	    88.06	  1  _
```

**35. IPI00384195 Isoform 3 of Plexin-B1**

| Sequence | Charge | M+H+ | XCorr | DeltaCn |
| --- | --- | --- | --- | --- |
| R.PASTTSMTPPGSGSWKRR.P | 2 | 1905.128 | 3.337 | 0.252 |

+1 charge

```
		      B   	     Y
  1	 P 	    98.06	        -  	 18
  2	 A 	   169.10	  1806.89	 17
  3	 S 	   256.13	  1735.85	 16
  4	 T 	   357.18	  1648.82	 15
  5	 T 	   458.22	  1547.77	 14
  6	 S 	   545.26	  1446.73	 13
  7	 M 	   676.30	  1359.70	 12
  8	 T 	   777.34	  1228.65	 11
  9	 P 	   874.40	  1127.61	 10
 10	 P 	   971.45	  1030.55	  9
 11	 G 	  1028.47	   933.50	  8
 12	 S 	  1115.50	   876.48	  7
 13	 G 	  1172.53	   789.45	  6
 14	 S 	  1259.56	   732.43	  5
 15	 W 	  1445.64	   645.39	  4
 16	 K 	  1573.73	   459.32	  3
 17	 R 	  1729.83	   331.22	  2
 18	 R 	        -  	   175.12	  1  _
+2 charge
		      B   	     Y
  1	 P 	    49.53	        -  	 18
  2	 A 	    85.05	   903.95	 17
  3	 S 	   128.57	   868.43	 16
  4	 T 	   179.09	   824.91	 15
  5	 T 	   229.62	   774.39	 14
  6	 S 	   273.13	   723.87	 13
  7	 M 	   338.65	   680.35	 12
  8	 T 	   389.18	   614.83	 11
  9	 P 	   437.70	   564.31	 10
 10	 P 	   486.23	   515.78	  9
 11	 G 	   514.74	   467.25	  8
 12	 S 	   558.26	   438.74	  7
 13	 G 	   586.77	   395.23	  6
 14	 S 	   630.28	   366.72	  5
 15	 W 	   723.32	   323.20	  4
 16	 K 	   787.37	   230.16	  3
 17	 R 	   865.42	   166.11	  2
 18	 R 	        -  	    88.06	  1  _
```

**36. IPI00843975 Ezrin**

| Sequence | Charge | M+H+ | XCorr | DeltaCn |
| --- | --- | --- | --- | --- |
| R.GMLKDNAMLEYLK.I | 2 | 1526.847 | 2.968 | 0.425 |

+1 charge

```
		      B   	     Y
  1	 G 	    58.03	        -  	 13
  2	 M 	   189.07	  1468.75	 12
  3	 L 	   302.15	  1337.71	 11
  4	 K 	   430.25	  1224.63	 10
  5	 D 	   545.28	  1096.53	  9
  6	 N 	   659.32	   981.51	  8
  7	 A 	   730.36	   867.46	  7
  8	 M 	   861.40	   796.43	  6
  9	 L 	   974.48	   665.39	  5
 10	 E 	  1103.52	   552.30	  4
 11	 Y 	  1266.59	   423.26	  3
 12	 L 	  1379.67	   260.20	  2
 13	 K 	        -  	   147.11	  1  _
+2 charge
		      B   	     Y
  1	 G 	    29.52	        -  	 13
  2	 M 	    95.04	   734.88	 12
  3	 L 	   151.58	   669.36	 11
  4	 K 	   215.63	   612.82	 10
  5	 D 	   273.14	   548.77	  9
  6	 N 	   330.16	   491.26	  8
  7	 A 	   365.68	   434.24	  7
  8	 M 	   431.20	   398.72	  6
  9	 L 	   487.74	   333.20	  5
 10	 E 	   552.26	   276.66	  4
 11	 Y 	   633.80	   212.13	  3
 12	 L 	   690.34	   130.60	  2
 13	 K 	        -  	    74.06	  1  _
```

**37. IPI00216065 Isoform 2 of Vitamin K-dependent protein Z**

| Sequence | Charge | M+H+ | XCorr | DeltaCn |
| --- | --- | --- | --- | --- |
| R.APDLQDLPWQVK.L | 2 | 1410.599 | 4.249 | 0.489 |

+1 charge

```
		      B   	     Y
  1	 A 	    72.04	        -  	 12
  2	 P 	   169.10	  1338.71	 11
  3	 D 	   284.12	  1241.65	 10
  4	 L 	   397.21	  1126.63	  9
  5	 Q 	   525.27	  1013.54	  8
  6	 D 	   640.29	   885.48	  7
  7	 L 	   753.38	   770.46	  6
  8	 P 	   850.43	   657.37	  5
  9	 W 	  1036.51	   560.32	  4
 10	 Q 	  1164.57	   374.24	  3
 11	 V 	  1263.64	   246.18	  2
 12	 K 	        -  	   147.11	  1  _
+2 charge
		      B   	     Y
  1	 A 	    36.53	        -  	 12
  2	 P 	    85.05	   669.86	 11
  3	 D 	   142.57	   621.33	 10
  4	 L 	   199.11	   563.82	  9
  5	 Q 	   263.14	   507.27	  8
  6	 D 	   320.65	   443.25	  7
  7	 L 	   377.19	   385.73	  6
  8	 P 	   425.72	   329.19	  5
  9	 W 	   518.76	   280.66	  4
 10	 Q 	   582.79	   187.62	  3
 11	 V 	   632.32	   123.59	  2
 12	 K 	        -  	    74.06	  1  _
```

**38. IPI00030786 Isoform 1 of Matrilin-4**

| Sequence | Charge | M+H+ | XCorr | DeltaCn |
| --- | --- | --- | --- | --- |
| R.TLGALESLTLNLAQLTAR.L | 2 | 1886.183 | 5.199 | 0.609 |

+1 charge

```
		      B   	     Y
  1	 T 	   102.05	        -  	 18
  2	 L 	   215.14	  1784.03	 17
  3	 G 	   272.16	  1670.94	 16
  4	 A 	   343.20	  1613.92	 15
  5	 L 	   456.28	  1542.88	 14
  6	 E 	   585.32	  1429.80	 13
  7	 S 	   672.36	  1300.76	 12
  8	 L 	   785.44	  1213.73	 11
  9	 T 	   886.49	  1100.64	 10
 10	 L 	   999.57	   999.59	  9
 11	 N 	  1113.62	   886.51	  8
 12	 L 	  1226.70	   772.47	  7
 13	 A 	  1297.74	   659.38	  6
 14	 Q 	  1425.79	   588.35	  5
 15	 L 	  1538.88	   460.29	  4
 16	 T 	  1639.93	   347.20	  3
 17	 A 	  1710.96	   246.16	  2
 18	 R 	        -  	   175.12	  1  _
+2 charge
		      B   	     Y
  1	 T 	    51.53	        -  	 18
  2	 L 	   108.07	   892.52	 17
  3	 G 	   136.58	   835.98	 16
  4	 A 	   172.10	   807.46	 15
  5	 L 	   228.64	   771.95	 14
  6	 E 	   293.17	   715.40	 13
  7	 S 	   336.68	   650.88	 12
  8	 L 	   393.22	   607.37	 11
  9	 T 	   443.75	   550.82	 10
 10	 L 	   500.29	   500.30	  9
 11	 N 	   557.31	   443.76	  8
 12	 L 	   613.85	   386.74	  7
 13	 A 	   649.37	   330.20	  6
 14	 Q 	   713.40	   294.68	  5
 15	 L 	   769.94	   230.65	  4
 16	 T 	   820.47	   174.11	  3
 17	 A 	   855.99	   123.58	  2
 18	 R 	        -  	    88.06	  1  _
```

**39. IPI00000914 Isoform 1 of Calcitonin**

| Sequence | Charge | M+H+ | XCorr | DeltaCn |
| --- | --- | --- | --- | --- |
| R.LLLAALVQDYVQMK.A | 2 | 1605.967 | 3.385 | 0.264 |

+1 charge

```
		      B   	     Y
  1	 L 	   114.09	        -  	 14
  2	 L 	   227.18	  1491.82	 13
  3	 L 	   340.26	  1378.74	 12
  4	 A 	   411.30	  1265.66	 11
  5	 A 	   482.33	  1194.62	 10
  6	 L 	   595.42	  1123.58	  9
  7	 V 	   694.49	  1010.50	  8
  8	 Q 	   822.54	   911.43	  7
  9	 D 	   937.57	   783.37	  6
 10	 Y 	  1100.64	   668.34	  5
 11	 V 	  1199.70	   505.28	  4
 12	 Q 	  1327.76	   406.21	  3
 13	 M 	  1458.80	   278.15	  2
 14	 K 	        -  	   147.11	  1  _
+2 charge
		      B   	     Y
  1	 L 	    57.55	        -  	 14
  2	 L 	   114.09	   746.42	 13
  3	 L 	   170.63	   689.87	 12
  4	 A 	   206.15	   633.33	 11
  5	 A 	   241.67	   597.81	 10
  6	 L 	   298.21	   562.29	  9
  7	 V 	   347.75	   505.75	  8
  8	 Q 	   411.78	   456.22	  7
  9	 D 	   469.29	   392.19	  6
 10	 Y 	   550.82	   334.68	  5
 11	 V 	   600.36	   253.14	  4
 12	 Q 	   664.38	   203.61	  3
 13	 M 	   729.90	   139.58	  2
 14	 K 	        -  	    74.06	  1  _
```

**40. IPI00410714 Hemoglobin subunit alpha**

| Sequence | Charge | M+H+ | XCorr | DeltaCn |
| --- | --- | --- | --- | --- |
| K.VGAHAGEYGAEALER.M | 2 | 1530.624 | 3.039 | 0.391 |

+1 charge

```
		      B   	     Y
  1	 V 	   100.08	        -  	 15
  2	 G 	   157.10	  1430.67	 14
  3	 A 	   228.13	  1373.64	 13
  4	 H 	   365.19	  1302.61	 12
  5	 A 	   436.23	  1165.55	 11
  6	 G 	   493.25	  1094.51	 10
  7	 E 	   622.29	  1037.49	  9
  8	 Y 	   785.36	   908.45	  8
  9	 G 	   842.38	   745.38	  7
 10	 A 	   913.42	   688.36	  6
 11	 E 	  1042.46	   617.33	  5
 12	 A 	  1113.50	   488.28	  4
 13	 L 	  1226.58	   417.25	  3
 14	 E 	  1355.62	   304.16	  2
 15	 R 	        -  	   175.12	  1  _
+2 charge
		      B   	     Y
  1	 V 	    50.54	        -  	 15
  2	 G 	    79.05	   715.84	 14
  3	 A 	   114.57	   687.33	 13
  4	 H 	   183.10	   651.81	 12
  5	 A 	   218.62	   583.28	 11
  6	 G 	   247.13	   547.76	 10
  7	 E 	   311.65	   519.25	  9
  8	 Y 	   393.18	   454.73	  8
  9	 G 	   421.69	   373.20	  7
 10	 A 	   457.21	   344.68	  6
 11	 E 	   521.73	   309.17	  5
 12	 A 	   557.25	   244.64	  4
 13	 L 	   613.79	   209.13	  3
 14	 E 	   678.31	   152.58	  2
 15	 R 	        -  	    88.06	  1  _
```

**41. IPI00027166 Metalloproteinase inhibitor 2**

| Sequence | Charge | M+H+ | XCorr | DeltaCn |
| --- | --- | --- | --- | --- |
| K.AVSEKEVDSGNDIYGNPIKR.I | 3 | 2192.372 | 5.298 | 0.581 |

+1 charge

```
		      B   	     Y
  1	 A 	    72.04	        -  	 20
  2	 V 	   171.11	  2120.06	 19
  3	 S 	   258.14	  2020.99	 18
  4	 E 	   387.19	  1933.96	 17
  5	 K 	   515.28	  1804.92	 16
  6	 E 	   644.32	  1676.82	 15
  7	 V 	   743.39	  1547.78	 14
  8	 D 	   858.42	  1448.71	 13
  9	 S 	   945.45	  1333.69	 12
 10	 G 	  1002.47	  1246.65	 11
 11	 N 	  1116.52	  1189.63	 10
 12	 D 	  1231.54	  1075.59	  9
 13	 I 	  1344.63	   960.56	  8
 14	 Y 	  1507.69	   847.48	  7
 15	 G 	  1564.71	   684.42	  6
 16	 N 	  1678.76	   627.39	  5
 17	 P 	  1775.81	   513.35	  4
 18	 I 	  1888.89	   416.30	  3
 19	 K 	  2016.99	   303.21	  2
 20	 R 	        -  	   175.12	  1  _
+2 charge
		      B   	     Y
  1	 A 	    36.53	        -  	 20
  2	 V 	    86.06	  1060.53	 19
  3	 S 	   129.58	  1011.00	 18
  4	 E 	   194.10	   967.48	 17
  5	 K 	   258.14	   902.96	 16
  6	 E 	   322.67	   838.92	 15
  7	 V 	   372.20	   774.39	 14
  8	 D 	   429.71	   724.86	 13
  9	 S 	   473.23	   667.35	 12
 10	 G 	   501.74	   623.83	 11
 11	 N 	   558.76	   595.32	 10
 12	 D 	   616.28	   538.30	  9
 13	 I 	   672.82	   480.78	  8
 14	 Y 	   754.35	   424.24	  7
 15	 G 	   782.86	   342.71	  6
 16	 N 	   839.88	   314.20	  5
 17	 P 	   888.41	   257.18	  4
 18	 I 	   944.95	   208.65	  3
 19	 K 	  1009.00	   152.11	  2
 20	 R 	        -  	    88.06	  1  _

+3 charge
		      B   	     Y
  1	 A 	    24.69	        -  	 20
  2	 V 	    57.71	   707.36	 19
  3	 S 	    86.72	   674.34	 18
  4	 E 	   129.73	   645.33	 17
  5	 K 	   172.43	   602.31	 16
  6	 E 	   215.45	   559.61	 15
  7	 V 	   248.47	   516.60	 14
  8	 D 	   286.81	   483.58	 13
  9	 S 	   315.82	   445.23	 12
 10	 G 	   334.83	   416.22	 11
 11	 N 	   372.84	   397.22	 10
 12	 D 	   411.19	   359.20	  9
 13	 I 	   448.88	   320.86	  8
 14	 Y 	   503.24	   283.16	  7
 15	 G 	   522.24	   228.81	  6
 16	 N 	   560.26	   209.80	  5
 17	 P 	   592.61	   171.79	  4
 18	 I 	   630.30	   139.44	  3
 19	 K 	   673.00	   101.74	  2
 20	 R 	        -  	    59.04	  1  _
```

**42. IPI00896380 Isoform 2 of Ig mu chain C region**

| Sequence | Charge | M+H+ | XCorr | DeltaCn |
| --- | --- | --- | --- | --- |
| K.LICQATGFSPR.Q | 2 | 1250.423 | 3.119 | 0.383 |

+1 charge

```
		      B   	     Y
  1	 L 	   114.09	        -  	 11
  2	 I 	   227.18	  1136.69	 10
  3	 C 	   387.34	  1023.60	  9
  4	 Q 	   515.40	   863.44	  8
  5	 A 	   586.44	   735.38	  7
  6	 T 	   687.48	   664.34	  6
  7	 G 	   744.51	   563.29	  5
  8	 F 	   891.57	   506.27	  4
  9	 S 	   978.61	   359.20	  3
 10	 P 	  1075.66	   272.17	  2
 11	 R 	        -  	   175.12	  1  _
+2 charge
		      B   	     Y
  1	 L 	    57.55	        -  	 11
  2	 I 	   114.09	   568.85	 10
  3	 C 	   194.17	   512.30	  9
  4	 Q 	   258.20	   432.22	  8
  5	 A 	   293.72	   368.19	  7
  6	 T 	   344.25	   332.67	  6
  7	 G 	   372.76	   282.15	  5
  8	 F 	   446.29	   253.64	  4
  9	 S 	   489.81	   180.11	  3
 10	 P 	   538.33	   136.59	  2
 11	 R 	        -  	    88.06	  1  _
```

**43. IPI00877894 Protein FAM74A1/A2**

| Sequence | Charge | M+H+ | XCorr | DeltaCn |
| --- | --- | --- | --- | --- |
| R.GCPGGDVETAQRLSQRR.R | 2 | 1888.029 | 2.889 | 0.208 |

+1 charge

```
		      B   	     Y
  1	 G 	    58.03	        -  	 17
  2	 C 	   218.19	  1830.04	 16
  3	 P 	   315.25	  1669.87	 15
  4	 G 	   372.27	  1572.82	 14
  5	 G 	   429.29	  1515.80	 13
  6	 D 	   544.32	  1458.78	 12
  7	 V 	   643.39	  1343.75	 11
  8	 E 	   772.43	  1244.68	 10
  9	 T 	   873.48	  1115.64	  9
 10	 A 	   944.51	  1014.59	  8
 11	 Q 	  1072.57	   943.55	  7
 12	 R 	  1228.67	   815.50	  6
 13	 L 	  1341.76	   659.39	  5
 14	 S 	  1428.79	   546.31	  4
 15	 Q 	  1556.85	   459.28	  3
 16	 R 	  1712.95	   331.22	  2
 17	 R 	        -  	   175.12	  1  _
+2 charge
		      B   	     Y
  1	 G 	    29.52	        -  	 17
  2	 C 	   109.60	   915.52	 16
  3	 P 	   158.13	   835.44	 15
  4	 G 	   186.64	   786.91	 14
  5	 G 	   215.15	   758.40	 13
  6	 D 	   272.66	   729.89	 12
  7	 V 	   322.20	   672.38	 11
  8	 E 	   386.72	   622.84	 10
  9	 T 	   437.24	   558.32	  9
 10	 A 	   472.76	   507.80	  8
 11	 Q 	   536.79	   472.28	  7
 12	 R 	   614.84	   408.25	  6
 13	 L 	   671.38	   330.20	  5
 14	 S 	   714.90	   273.66	  4
 15	 Q 	   778.93	   230.14	  3
 16	 R 	   856.98	   166.11	  2
 17	 R 	        -  	    88.06	  1  _
```

**44. IPI00183968 tropomyosin 3 isoform 1**

| Sequence | Charge | M+H+ | XCorr | DeltaCn |
| --- | --- | --- | --- | --- |
| R.LATALQKLEEAEKAADESER.G | 3 | 2203.393 | 3.524 | 0.325 |

+1 charge

```
		      B   	     Y
  1	 L 	   114.09	        -  	 20
  2	 A 	   185.13	  2089.04	 19
  3	 T 	   286.18	  2018.00	 18
  4	 A 	   357.21	  1916.96	 17
  5	 L 	   470.30	  1845.92	 16
  6	 Q 	   598.36	  1732.83	 15
  7	 K 	   726.45	  1604.78	 14
  8	 L 	   839.53	  1476.68	 13
  9	 E 	   968.58	  1363.60	 12
 10	 E 	  1097.62	  1234.55	 11
 11	 A 	  1168.66	  1105.51	 10
 12	 E 	  1297.70	  1034.47	  9
 13	 K 	  1425.79	   905.43	  8
 14	 A 	  1496.83	   777.34	  7
 15	 A 	  1567.87	   706.30	  6
 16	 D 	  1682.90	   635.26	  5
 17	 E 	  1811.94	   520.24	  4
 18	 S 	  1898.97	   391.19	  3
 19	 E 	  2028.01	   304.16	  2
 20	 R 	        -  	   175.12	  1  _
+2 charge
		      B   	     Y
  1	 L 	    57.55	        -  	 20
  2	 A 	    93.07	  1045.02	 19
  3	 T 	   143.59	  1009.51	 18
  4	 A 	   179.11	   958.98	 17
  5	 L 	   235.65	   923.46	 16
  6	 Q 	   299.68	   866.92	 15
  7	 K 	   363.73	   802.89	 14
  8	 L 	   420.27	   738.84	 13
  9	 E 	   484.79	   682.30	 12
 10	 E 	   549.31	   617.78	 11
 11	 A 	   584.83	   553.26	 10
 12	 E 	   649.35	   517.74	  9
 13	 K 	   713.40	   453.22	  8
 14	 A 	   748.92	   389.17	  7
 15	 A 	   784.44	   353.65	  6
 16	 D 	   841.95	   318.14	  5
 17	 E 	   906.47	   260.62	  4
 18	 S 	   949.99	   196.10	  3
 19	 E 	  1014.51	   152.58	  2
 20	 R 	        -  	    88.06	  1  _

+3 charge
		      B   	     Y
  1	 L 	    38.70	        -  	 20
  2	 A 	    62.38	   697.02	 19
  3	 T 	    96.06	   673.34	 18
  4	 A 	   119.74	   639.66	 17
  5	 L 	   157.44	   615.98	 16
  6	 Q 	   200.12	   578.28	 15
  7	 K 	   242.82	   535.60	 14
  8	 L 	   280.52	   492.90	 13
  9	 E 	   323.53	   455.20	 12
 10	 E 	   366.54	   412.19	 11
 11	 A 	   390.22	   369.18	 10
 12	 E 	   433.24	   345.50	  9
 13	 K 	   475.94	   302.48	  8
 14	 A 	   499.62	   259.78	  7
 15	 A 	   523.29	   236.10	  6
 16	 D 	   561.64	   212.43	  5
 17	 E 	   604.65	   174.08	  4
 18	 S 	   633.66	   131.07	  3
 19	 E 	   676.68	   102.06	  2
 20	 R 	        -  	    59.04	  1  _
```

**45. IPI00013890 Isoform 1 of 14-3-3 protein sigma**

| Sequence | Charge | M+H+ | XCorr | DeltaCn |
| --- | --- | --- | --- | --- |
| K.LAEQAERYEDMAAFMK.G | 2 | 1904.157 | 3.259 | 0.536 |

+1 charge

```
		      B   	     Y
  1	 L 	   114.09	        -  	 16
  2	 A 	   185.13	  1789.79	 15
  3	 E 	   314.17	  1718.75	 14
  4	 Q 	   442.23	  1589.71	 13
  5	 A 	   513.27	  1461.65	 12
  6	 E 	   642.31	  1390.61	 11
  7	 R 	   798.41	  1261.57	 10
  8	 Y 	   961.47	  1105.47	  9
  9	 E 	  1090.52	   942.41	  8
 10	 D 	  1205.54	   813.36	  7
 11	 M 	  1336.58	   698.34	  6
 12	 A 	  1407.62	   567.30	  5
 13	 A 	  1478.66	   496.26	  4
 14	 F 	  1625.73	   425.22	  3
 15	 M 	  1756.77	   278.15	  2
 16	 K 	        -  	   147.11	  1  _
+2 charge
		      B   	     Y
  1	 L 	    57.55	        -  	 16
  2	 A 	    93.07	   895.40	 15
  3	 E 	   157.59	   859.88	 14
  4	 Q 	   221.62	   795.36	 13
  5	 A 	   257.14	   731.33	 12
  6	 E 	   321.66	   695.81	 11
  7	 R 	   399.71	   631.29	 10
  8	 Y 	   481.24	   553.24	  9
  9	 E 	   545.76	   471.71	  8
 10	 D 	   603.28	   407.19	  7
 11	 M 	   668.80	   349.67	  6
 12	 A 	   704.31	   284.15	  5
 13	 A 	   739.83	   248.63	  4
 14	 F 	   813.37	   213.11	  3
 15	 M 	   878.89	   139.58	  2
 16	 K 	        -  	    74.06	  1  _
```

**46. IPI00008239 cDNA FLJ55176, highly similar to G-protein coupled receptor family C group 5 member B**

| Sequence | Charge | M+H+ | XCorr | DeltaCn |
| --- | --- | --- | --- | --- |
| R.SNVYQPTEMAVVLNGGTIPTAPPSHTGR.H | 3 | 2896.228 | 4.424 | 0.566 |

+1 charge

```
		      B   	     Y
  1	 S 	    88.04	        -  	 28
  2	 N 	   202.08	  2807.41	 27
  3	 V 	   301.15	  2693.37	 26
  4	 Y 	   464.21	  2594.30	 25
  5	 Q 	   592.27	  2431.24	 24
  6	 P 	   689.33	  2303.18	 23
  7	 T 	   790.37	  2206.13	 22
  8	 E 	   919.42	  2105.08	 21
  9	 M 	  1050.46	  1976.04	 20
 10	 A 	  1121.49	  1845.00	 19
 11	 V 	  1220.56	  1773.96	 18
 12	 V 	  1319.63	  1674.89	 17
 13	 L 	  1432.71	  1575.82	 16
 14	 N 	  1546.76	  1462.74	 15
 15	 G 	  1603.78	  1348.70	 14
 16	 G 	  1660.80	  1291.68	 13
 17	 T 	  1761.85	  1234.65	 12
 18	 I 	  1874.93	  1133.61	 11
 19	 P 	  1971.98	  1020.52	 10
 20	 T 	  2073.03	   923.47	  9
 21	 A 	  2144.07	   822.42	  8
 22	 P 	  2241.12	   751.38	  7
 23	 P 	  2338.17	   654.33	  6
 24	 S 	  2425.21	   557.28	  5
 25	 H 	  2562.27	   470.25	  4
 26	 T 	  2663.31	   333.19	  3
 27	 G 	  2720.33	   232.14	  2
 28	 R 	        -  	   175.12	  1  _


+2 charge
		      B   	     Y
  1	 S 	    44.52	        -  	 28
  2	 N 	   101.54	  1404.21	 27
  3	 V 	   151.08	  1347.19	 26
  4	 Y 	   232.61	  1297.66	 25
  5	 Q 	   296.64	  1216.12	 24
  6	 P 	   345.17	  1152.09	 23
  7	 T 	   395.69	  1103.57	 22
  8	 E 	   460.21	  1053.04	 21
  9	 M 	   525.73	   988.52	 20
 10	 A 	   561.25	   923.00	 19
 11	 V 	   610.78	   887.48	 18
 12	 V 	   660.32	   837.95	 17
 13	 L 	   716.86	   788.42	 16
 14	 N 	   773.88	   731.87	 15
 15	 G 	   802.39	   674.85	 14
 16	 G 	   830.90	   646.34	 13
 17	 T 	   881.43	   617.83	 12
 18	 I 	   937.97	   567.31	 11
 19	 P 	   986.50	   510.76	 10
 20	 T 	  1037.02	   462.24	  9
 21	 A 	  1072.54	   411.71	  8
 22	 P 	  1121.06	   376.20	  7
 23	 P 	  1169.59	   327.67	  6
 24	 S 	  1213.11	   279.14	  5
 25	 H 	  1281.64	   235.63	  4
 26	 T 	  1332.16	   167.10	  3
 27	 G 	  1360.67	   116.57	  2
 28	 R 	        -  	    88.06	  1  _


+3 charge
		      B   	     Y
  1	 S 	    30.02	        -  	 28
  2	 N 	    68.03	   936.48	 27
  3	 V 	   101.06	   898.46	 26
  4	 Y 	   155.41	   865.44	 25
  5	 Q 	   198.10	   811.08	 24
  6	 P 	   230.45	   768.40	 23
  7	 T 	   264.13	   736.05	 22
  8	 E 	   307.14	   702.37	 21
  9	 M 	   350.82	   659.35	 20
 10	 A 	   374.50	   615.67	 19
 11	 V 	   407.53	   591.99	 18
 12	 V 	   440.55	   558.97	 17
 13	 L 	   478.24	   525.95	 16
 14	 N 	   516.26	   488.25	 15
 15	 G 	   535.26	   450.24	 14
 16	 G 	   554.27	   431.23	 13
 17	 T 	   587.95	   412.22	 12
 18	 I 	   625.65	   378.54	 11
 19	 P 	   658.00	   340.85	 10
 20	 T 	   691.68	   308.49	  9
 21	 A 	   715.36	   274.81	  8
 22	 P 	   747.71	   251.13	  7
 23	 P 	   780.06	   218.78	  6
 24	 S 	   809.07	   186.43	  5
 25	 H 	   854.76	   157.42	  4
 26	 T 	   888.44	   111.73	  3
 27	 G 	   907.45	    78.05	  2
 28	 R 	        -  	    59.04	  1  _
```

**47. IPI00387120 Ig kappa chain V-IV region Len**

| Sequence | Charge | M+H+ | XCorr | DeltaCn |
| --- | --- | --- | --- | --- |
| - .DIVMTQSPDSLAVSLGER.A | 2 | 1919.147 | 5.377 | 0.557 |

+1 charge

```
		      B   	     Y
  1	 D 	   116.03	        -  	 18
  2	 I 	   229.12	  1802.93	 17
  3	 V 	   328.19	  1689.85	 16
  4	 M 	   459.23	  1590.78	 15
  5	 T 	   560.27	  1459.74	 14
  6	 Q 	   688.33	  1358.69	 13
  7	 S 	   775.37	  1230.63	 12
  8	 P 	   872.42	  1143.60	 11
  9	 D 	   987.45	  1046.55	 10
 10	 S 	  1074.48	   931.52	  9
 11	 L 	  1187.56	   844.49	  8
 12	 A 	  1258.60	   731.40	  7
 13	 V 	  1357.67	   660.37	  6
 14	 S 	  1444.70	   561.30	  5
 15	 L 	  1557.78	   474.27	  4
 16	 G 	  1614.80	   361.18	  3
 17	 E 	  1743.85	   304.16	  2
 18	 R 	        -  	   175.12	  1  _
+2 charge
		      B   	     Y
  1	 D 	    58.52	        -  	 18
  2	 I 	   115.06	   901.97	 17
  3	 V 	   164.60	   845.43	 16
  4	 M 	   230.12	   795.89	 15
  5	 T 	   280.64	   730.37	 14
  6	 Q 	   344.67	   679.85	 13
  7	 S 	   388.19	   615.82	 12
  8	 P 	   436.71	   572.30	 11
  9	 D 	   494.23	   523.78	 10
 10	 S 	   537.74	   466.26	  9
 11	 L 	   594.28	   422.75	  8
 12	 A 	   629.80	   366.21	  7
 13	 V 	   679.34	   330.69	  6
 14	 S 	   722.85	   281.15	  5
 15	 L 	   779.40	   237.64	  4
 16	 G 	   807.91	   181.10	  3
 17	 E 	   872.43	   152.58	  2
 18	 R 	        -  	    88.06	  1  _
```

**48. IPI00065450 Isoform 1 of Putative adenylate kinase-like protein C9orf98**

| Sequence | Charge | M+H+ | XCorr | DeltaCn |
| --- | --- | --- | --- | --- |
| R.KTVPSALLVQLIQERLAEEDCIK.Q | 3 | 2655.078 | 3.859 | 0.338 |

+1 charge

```
		      B   	     Y
  1	 K 	   129.10	        -  	 23
  2	 T 	   230.15	  2525.50	 22
  3	 V 	   329.22	  2424.45	 21
  4	 P 	   426.27	  2325.38	 20
  5	 S 	   513.30	  2228.33	 19
  6	 A 	   584.34	  2141.30	 18
  7	 L 	   697.42	  2070.26	 17
  8	 L 	   810.51	  1957.18	 16
  9	 V 	   909.58	  1844.09	 15
 10	 Q 	  1037.64	  1745.02	 14
 11	 L 	  1150.72	  1616.97	 13
 12	 I 	  1263.80	  1503.88	 12
 13	 Q 	  1391.86	  1390.80	 11
 14	 E 	  1520.90	  1262.74	 10
 15	 R 	  1677.01	  1133.70	  9
 16	 L 	  1790.09	   977.60	  8
 17	 A 	  1861.13	   864.51	  7
 18	 E 	  1990.17	   793.47	  6
 19	 E 	  2119.21	   664.43	  5
 20	 D 	  2234.24	   535.39	  4
 21	 C 	  2394.40	   420.36	  3
 22	 I 	  2507.49	   260.20	  2
 23	 K 	        -  	   147.11	  1  _


+2 charge
		      B   	     Y
  1	 K 	    65.05	        -  	 23
  2	 T 	   115.58	  1263.25	 22
  3	 V 	   165.11	  1212.73	 21
  4	 P 	   213.64	  1163.20	 20
  5	 S 	   257.16	  1114.67	 19
  6	 A 	   292.67	  1071.15	 18
  7	 L 	   349.22	  1035.63	 17
  8	 L 	   405.76	   979.09	 16
  9	 V 	   455.29	   922.55	 15
 10	 Q 	   519.32	   873.02	 14
 11	 L 	   575.86	   808.99	 13
 12	 I 	   632.41	   752.44	 12
 13	 Q 	   696.43	   695.90	 11
 14	 E 	   760.96	   631.87	 10
 15	 R 	   839.01	   567.35	  9
 16	 L 	   895.55	   489.30	  8
 17	 A 	   931.07	   432.76	  7
 18	 E 	   995.59	   397.24	  6
 19	 E 	  1060.11	   332.72	  5
 20	 D 	  1117.62	   268.20	  4
 21	 C 	  1197.71	   210.68	  3
 22	 I 	  1254.25	   130.60	  2
 23	 K 	        -  	    74.06	  1  _


+3 charge
		      B   	     Y
  1	 K 	    43.71	        -  	 23
  2	 T 	    77.39	   842.50	 22
  3	 V 	   110.41	   808.82	 21
  4	 P 	   142.76	   775.80	 20
  5	 S 	   171.77	   743.45	 19
  6	 A 	   195.45	   714.44	 18
  7	 L 	   233.15	   690.76	 17
  8	 L 	   270.84	   653.06	 16
  9	 V 	   303.86	   615.37	 15
 10	 Q 	   346.55	   582.35	 14
 11	 L 	   384.24	   539.66	 13
 12	 I 	   421.94	   501.97	 12
 13	 Q 	   464.63	   464.27	 11
 14	 E 	   507.64	   421.58	 10
 15	 R 	   559.67	   378.57	  9
 16	 L 	   597.37	   326.54	  8
 17	 A 	   621.05	   288.84	  7
 18	 E 	   664.06	   265.16	  6
 19	 E 	   707.08	   222.15	  5
 20	 D 	   745.42	   179.13	  4
 21	 C 	   798.81	   140.79	  3
 22	 I 	   836.50	    87.40	  2
 23	 K 	        -  	    49.71	  1  _
```

**49. IPI00009793 Complement C1r subcomponent-like protein**

| Sequence | Charge | M+H+ | XCorr | DeltaCn |
| --- | --- | --- | --- | --- |
| R.AKLGNFPWQAFTSIHGR.G | 3 | 1931.189 | 4.027 | 0.476 |

+1 charge

```
		      B   	     Y
  1	 A 	    72.04	        -  	 17
  2	 K 	   200.14	  1858.97	 16
  3	 L 	   313.22	  1730.88	 15
  4	 G 	   370.24	  1617.79	 14
  5	 N 	   484.29	  1560.77	 13
  6	 F 	   631.36	  1446.73	 12
  7	 P 	   728.41	  1299.66	 11
  8	 W 	   914.49	  1202.61	 10
  9	 Q 	  1042.55	  1016.53	  9
 10	 A 	  1113.58	   888.47	  8
 11	 F 	  1260.65	   817.43	  7
 12	 T 	  1361.70	   670.36	  6
 13	 S 	  1448.73	   569.32	  5
 14	 I 	  1561.82	   482.28	  4
 15	 H 	  1698.88	   369.20	  3
 16	 G 	  1755.90	   232.14	  2
 17	 R 	        -  	   175.12	  1  _
+2 charge
		      B   	     Y
  1	 A 	    36.53	        -  	 17
  2	 K 	   100.57	   929.99	 16
  3	 L 	   157.12	   865.94	 15
  4	 G 	   185.63	   809.40	 14
  5	 N 	   242.65	   780.89	 13
  6	 F 	   316.18	   723.87	 12
  7	 P 	   364.71	   650.33	 11
  8	 W 	   457.75	   601.81	 10
  9	 Q 	   521.78	   508.77	  9
 10	 A 	   557.30	   444.74	  8
 11	 F 	   630.83	   409.22	  7
 12	 T 	   681.35	   335.69	  6
 13	 S 	   724.87	   285.16	  5
 14	 I 	   781.41	   241.65	  4
 15	 H 	   849.94	   185.10	  3
 16	 G 	   878.45	   116.57	  2
 17	 R 	        -  	    88.06	  1  _


+3 charge
		      B   	     Y
  1	 A 	    24.69	        -  	 17
  2	 K 	    67.38	   620.33	 16
  3	 L 	   105.08	   577.63	 15
  4	 G 	   124.09	   539.94	 14
  5	 N 	   162.10	   520.93	 13
  6	 F 	   211.12	   482.91	 12
  7	 P 	   243.47	   433.89	 11
  8	 W 	   305.50	   401.54	 10
  9	 Q 	   348.19	   339.51	  9
 10	 A 	   371.87	   296.83	  8
 11	 F 	   420.89	   273.15	  7
 12	 T 	   454.57	   224.13	  6
 13	 S 	   483.58	   190.44	  5
 14	 I 	   521.28	   161.43	  4
 15	 H 	   566.96	   123.74	  3
 16	 G 	   585.97	    78.05	  2
 17	 R 	        -  	    59.04	  1  _
```

**50. IPI00015902 Beta-type platelet-derived growth factor receptor**

| Sequence | Charge | M+H+ | XCorr | DeltaCn |
| --- | --- | --- | --- | --- |
| R.LVEPVTDFLLDMPYHIR.S | 2 | 2059.417 | 2.92 | 0.422 |

+1 charge

```
		      B   	     Y
  1	 L 	   114.09	        -  	 17
  2	 V 	   213.16	  1944.99	 16
  3	 E 	   342.20	  1845.92	 15
  4	 P 	   439.26	  1716.88	 14
  5	 V 	   538.32	  1619.83	 13
  6	 T 	   639.37	  1520.76	 12
  7	 D 	   754.40	  1419.71	 11
  8	 F 	   901.47	  1304.68	 10
  9	 L 	  1014.55	  1157.61	  9
 10	 L 	  1127.63	  1044.53	  8
 11	 D 	  1242.66	   931.45	  7
 12	 M 	  1373.70	   816.42	  6
 13	 P 	  1470.75	   685.38	  5
 14	 Y 	  1633.82	   588.33	  4
 15	 H 	  1770.88	   425.26	  3
 16	 I 	  1883.96	   288.20	  2
 17	 R 	        -  	   175.12	  1  _
+2 charge
		      B   	     Y
  1	 L 	    57.55	        -  	 17
  2	 V 	   107.08	   973.00	 16
  3	 E 	   171.60	   923.46	 15
  4	 P 	   220.13	   858.94	 14
  5	 V 	   269.67	   810.42	 13
  6	 T 	   320.19	   760.88	 12
  7	 D 	   377.70	   710.36	 11
  8	 F 	   451.24	   652.84	 10
  9	 L 	   507.78	   579.31	  9
 10	 L 	   564.32	   522.77	  8
 11	 D 	   621.83	   466.23	  7
 12	 M 	   687.35	   408.71	  6
 13	 P 	   735.88	   343.19	  5
 14	 Y 	   817.41	   294.67	  4
 15	 H 	   885.94	   213.13	  3
 16	 I 	   942.48	   144.61	  2
 17	 R 	        -  	    88.06	  1  _
```

**51. IPI00005573 Isoform 1 of 5'(3')-deoxyribonucleotidase, cytosolic type**

| Sequence | Charge | M+H+ | XCorr | DeltaCn |
| --- | --- | --- | --- | --- |
| R.VLVDMDGVLADFEAGLLR.G | 2 | 1934.246 | 4.466 | 0.583 |

+1 charge

```
		      B   	     Y
  1	 V 	   100.08	        -  	 18
  2	 L 	   213.16	  1833.94	 17
  3	 V 	   312.23	  1720.86	 16
  4	 D 	   427.26	  1621.79	 15
  5	 M 	   558.30	  1506.76	 14
  6	 D 	   673.32	  1375.72	 13
  7	 G 	   730.34	  1260.69	 12
  8	 V 	   829.41	  1203.67	 11
  9	 L 	   942.50	  1104.60	 10
 10	 A 	  1013.53	   991.52	  9
 11	 D 	  1128.56	   920.48	  8
 12	 F 	  1275.63	   805.46	  7
 13	 E 	  1404.67	   658.39	  6
 14	 A 	  1475.71	   529.35	  5
 15	 G 	  1532.73	   458.31	  4
 16	 L 	  1645.81	   401.29	  3
 17	 L 	  1758.90	   288.20	  2
 18	 R 	        -  	   175.12	  1  _
+2 charge
		      B   	     Y
  1	 V 	    50.54	        -  	 18
  2	 L 	   107.08	   917.47	 17
  3	 V 	   156.62	   860.93	 16
  4	 D 	   214.13	   811.40	 15
  5	 M 	   279.65	   753.88	 14
  6	 D 	   337.16	   688.36	 13
  7	 G 	   365.68	   630.85	 12
  8	 V 	   415.21	   602.34	 11
  9	 L 	   471.75	   552.81	 10
 10	 A 	   507.27	   496.26	  9
 11	 D 	   564.78	   460.75	  8
 12	 F 	   638.32	   403.23	  7
 13	 E 	   702.84	   329.70	  6
 14	 A 	   738.36	   265.18	  5
 15	 G 	   766.87	   229.66	  4
 16	 L 	   823.41	   201.15	  3
 17	 L 	   879.95	   144.61	  2
 18	 R 	        -  	    88.06	  1  _
```

**52. IPI00024292 Low-density lipoprotein receptor-related protein 2**

| Sequence | Charge | M+H+ | XCorr | DeltaCn |
| --- | --- | --- | --- | --- |
| R.HLCHCEEGYILER.G | 3 | 1716.863 | 3.811 | 0.41 |

+1 charge

```
		      B   	     Y
  1	 H 	   138.07	        -  	 13
  2	 L 	   251.15	  1578.97	 12
  3	 C 	   411.32	  1465.89	 11
  4	 H 	   548.37	  1305.72	 10
  5	 C 	   708.54	  1168.66	  9
  6	 E 	   837.58	  1008.50	  8
  7	 E 	   966.62	   879.46	  7
  8	 G 	  1023.65	   750.41	  6
  9	 Y 	  1186.71	   693.39	  5
 10	 I 	  1299.79	   530.33	  4
 11	 L 	  1412.88	   417.25	  3
 12	 E 	  1541.92	   304.16	  2
 13	 R 	        -  	   175.12	  1  _
+2 charge
		      B   	     Y
  1	 H 	    69.54	        -  	 13
  2	 L 	   126.08	   789.99	 12
  3	 C 	   206.16	   733.45	 11
  4	 H 	   274.69	   653.37	 10
  5	 C 	   354.77	   584.84	  9
  6	 E 	   419.29	   504.75	  8
  7	 E 	   483.82	   440.23	  7
  8	 G 	   512.33	   375.71	  6
  9	 Y 	   593.86	   347.20	  5
 10	 I 	   650.40	   265.67	  4
 11	 L 	   706.94	   209.13	  3
 12	 E 	   771.46	   152.58	  2
 13	 R 	        -  	    88.06	  1  _
+3 charge
		      B   	     Y
  1	 H 	    46.69	        -  	 13
  2	 L 	    84.39	   527.00	 12
  3	 C 	   137.78	   489.30	 11
  4	 H 	   183.46	   435.91	 10
  5	 C 	   236.85	   390.23	  9
  6	 E 	   279.87	   336.84	  8
  7	 E 	   322.88	   293.82	  7
  8	 G 	   341.89	   250.81	  6
  9	 Y 	   396.24	   231.80	  5
 10	 I 	   433.94	   177.45	  4
 11	 L 	   471.63	   139.75	  3
 12	 E 	   514.64	   102.06	  2
 13	 R 	        -  	    59.04	  1  _
```

**53. IPI00784430 Similar to Ig kappa chain V-III region VG precursor**

| Sequence | Charge | M+H+ | XCorr | DeltaCn |
| --- | --- | --- | --- | --- |
| R.LLIYDASNR.A | 2 | 1065.205 | 3.211 | 0.44 |

+1 charge

```
		      B   	     Y
  1	 L 	   114.09	        -  	  9
  2	 L 	   227.18	   951.49	  8
  3	 I 	   340.26	   838.41	  7
  4	 Y 	   503.32	   725.32	  6
  5	 D 	   618.35	   562.26	  5
  6	 A 	   689.39	   447.23	  4
  7	 S 	   776.42	   376.19	  3
  8	 N 	   890.46	   289.16	  2
  9	 R 	        -  	   175.12	  1  _
+2 charge
		      B   	     Y
  1	 L 	    57.55	        -  	  9
  2	 L 	   114.09	   476.25	  8
  3	 I 	   170.63	   419.71	  7
  4	 Y 	   252.17	   363.16	  6
  5	 D 	   309.68	   281.63	  5
  6	 A 	   345.20	   224.12	  4
  7	 S 	   388.71	   188.60	  3
  8	 N 	   445.73	   145.08	  2
  9	 R 	        -  	    88.06	  1  _
```

**54. IPI00009010 tRNA methyltransferase 112 homolog**

| Sequence | Charge | M+H+ | XCorr | DeltaCn |
| --- | --- | --- | --- | --- |
| K.LLTHNLLSSHVRGVGSRGFPLR.L | 3 | 2417.801 | 3.452 | 0.206 |

+1 charge

```
		      B   	     Y
  1	 L 	   114.09	        -  	 22
  2	 L 	   227.18	  2303.28	 21
  3	 T 	   328.22	  2190.20	 20
  4	 H 	   465.28	  2089.15	 19
  5	 N 	   579.32	  1952.09	 18
  6	 L 	   692.41	  1838.05	 17
  7	 L 	   805.49	  1724.97	 16
  8	 S 	   892.53	  1611.88	 15
  9	 S 	   979.56	  1524.85	 14
 10	 H 	  1116.62	  1437.82	 13
 11	 V 	  1215.68	  1300.76	 12
 12	 R 	  1371.79	  1201.69	 11
 13	 G 	  1428.81	  1045.59	 10
 14	 V 	  1527.88	   988.57	  9
 15	 G 	  1584.90	   889.50	  8
 16	 S 	  1671.93	   832.48	  7
 17	 R 	  1828.03	   745.45	  6
 18	 G 	  1885.05	   589.35	  5
 19	 F 	  2032.12	   532.32	  4
 20	 P 	  2129.17	   385.26	  3
 21	 L 	  2242.26	   288.20	  2
 22	 R 	        -  	   175.12	  1  _
+2 charge
		      B   	     Y
  1	 L 	    57.55	        -  	 22
  2	 L 	   114.09	  1152.15	 21
  3	 T 	   164.62	  1095.60	 20
  4	 H 	   233.14	  1045.08	 19
  5	 N 	   290.17	   976.55	 18
  6	 L 	   346.71	   919.53	 17
  7	 L 	   403.25	   862.99	 16
  8	 S 	   446.77	   806.44	 15
  9	 S 	   490.28	   762.93	 14
 10	 H 	   558.81	   719.41	 13
 11	 V 	   608.35	   650.88	 12
 12	 R 	   686.40	   601.35	 11
 13	 G 	   714.91	   523.30	 10
 14	 V 	   764.44	   494.79	  9
 15	 G 	   792.95	   445.25	  8
 16	 S 	   836.47	   416.74	  7
 17	 R 	   914.52	   373.23	  6
 18	 G 	   943.03	   295.18	  5
 19	 F 	  1016.56	   266.67	  4
 20	 P 	  1065.09	   193.13	  3
 21	 L 	  1121.63	   144.61	  2
 22	 R 	        -  	    88.06	  1  _
+3 charge
		      B   	     Y
  1	 L 	    38.70	        -  	 22
  2	 L 	    76.40	   768.43	 21
  3	 T 	   110.08	   730.74	 20
  4	 H 	   155.77	   697.06	 19
  5	 N 	   193.78	   651.37	 18
  6	 L 	   231.47	   613.36	 17
  7	 L 	   269.17	   575.66	 16
  8	 S 	   298.18	   537.97	 15
  9	 S 	   327.19	   508.96	 14
 10	 H 	   372.88	   479.94	 13
 11	 V 	   405.90	   434.26	 12
 12	 R 	   457.93	   401.24	 11
 13	 G 	   476.94	   349.20	 10
 14	 V 	   509.96	   330.19	  9
 15	 G 	   528.97	   297.17	  8
 16	 S 	   557.98	   278.16	  7
 17	 R 	   610.01	   249.15	  6
 18	 G 	   629.02	   197.12	  5
 19	 F 	   678.04	   178.11	  4
 20	 P 	   710.40	   129.09	  3
 21	 L 	   748.09	    96.74	  2
 22	 R 	        -  	    59.04	  1  _
```

**55. IPI00012540 Prominin-1**

| Sequence | Charge | M+H+ | XCorr | DeltaCn |
| --- | --- | --- | --- | --- |
| R.LSLSQLNSNPELR.Q | 2 | 1471.641 | 3.363 | 0.38 |

+1 charge

```
		      B   	     Y
  1	 L 	   114.09	        -  	 13
  2	 S 	   201.12	  1357.71	 12
  3	 L 	   314.21	  1270.67	 11
  4	 S 	   401.24	  1157.59	 10
  5	 Q 	   529.30	  1070.56	  9
  6	 L 	   642.38	   942.50	  8
  7	 N 	   756.43	   829.42	  7
  8	 S 	   843.46	   715.37	  6
  9	 N 	   957.50	   628.34	  5
 10	 P 	  1054.55	   514.30	  4
 11	 E 	  1183.60	   417.25	  3
 12	 L 	  1296.68	   288.20	  2
 13	 R 	        -  	   175.12	  1  _
+2 charge
		      B   	     Y
  1	 L 	    57.55	        -  	 13
  2	 S 	   101.07	   679.36	 12
  3	 L 	   157.61	   635.84	 11
  4	 S 	   201.12	   579.30	 10
  5	 Q 	   265.15	   535.78	  9
  6	 L 	   321.69	   471.75	  8
  7	 N 	   378.72	   415.21	  7
  8	 S 	   422.23	   358.19	  6
  9	 N 	   479.25	   314.67	  5
 10	 P 	   527.78	   257.65	  4
 11	 E 	   592.30	   209.13	  3
 12	 L 	   648.84	   144.61	  2
 13	 R 	        -  	    88.06	  1  _
```

**56. IPI00003091 Isoform 1 of Protein phosphatase 1 regulatory subunit 3A**

| Sequence | Charge | M+H+ | XCorr | DeltaCn |
| --- | --- | --- | --- | --- |
| K.EQEPEPVKPWKEVPNR.Q | 2 | 1963.183 | 3.168 | 0.204 |

+1 charge

```
		      B   	     Y
  1	 E 	   130.05	        -  	 16
  2	 Q 	   258.11	  1832.97	 15
  3	 E 	   387.15	  1704.91	 14
  4	 P 	   484.20	  1575.86	 13
  5	 E 	   613.25	  1478.81	 12
  6	 P 	   710.30	  1349.77	 11
  7	 V 	   809.37	  1252.72	 10
  8	 K 	   937.46	  1153.65	  9
  9	 P 	  1034.52	  1025.55	  8
 10	 W 	  1220.59	   928.50	  7
 11	 K 	  1348.69	   742.42	  6
 12	 E 	  1477.73	   614.33	  5
 13	 V 	  1576.80	   485.28	  4
 14	 P 	  1673.85	   386.21	  3
 15	 N 	  1787.90	   289.16	  2
 16	 R 	        -  	   175.12	  1  _
+2 charge
		      B   	     Y
  1	 E 	    65.53	        -  	 16
  2	 Q 	   129.56	   916.99	 15
  3	 E 	   194.08	   852.96	 14
  4	 P 	   242.61	   788.44	 13
  5	 E 	   307.13	   739.91	 12
  6	 P 	   355.65	   675.39	 11
  7	 V 	   405.19	   626.86	 10
  8	 K 	   469.23	   577.33	  9
  9	 P 	   517.76	   513.28	  8
 10	 W 	   610.80	   464.75	  7
 11	 K 	   674.85	   371.71	  6
 12	 E 	   739.37	   307.67	  5
 13	 V 	   788.90	   243.15	  4
 14	 P 	   837.43	   193.61	  3
 15	 N 	   894.45	   145.08	  2
 16	 R 	        -  	    88.06	  1  _
```

**57. IPI00921290 Conserved hypothetical protein**

| Sequence | Charge | M+H+ | XCorr | DeltaCn |
| --- | --- | --- | --- | --- |
| R.ISSQEAQGPPRCPMGR.E | 2 | 1771.974 | 2.879 | 0.327 |

+1 charge

```
		      B   	     Y
  1	 I 	   114.09	        -  	 16
  2	 S 	   201.12	  1657.89	 15
  3	 S 	   288.16	  1570.86	 14
  4	 Q 	   416.21	  1483.82	 13
  5	 E 	   545.26	  1355.77	 12
  6	 A 	   616.29	  1226.72	 11
  7	 Q 	   744.35	  1155.69	 10
  8	 G 	   801.37	  1027.63	  9
  9	 P 	   898.43	   970.61	  8
 10	 P 	   995.48	   873.55	  7
 11	 R 	  1151.58	   776.50	  6
 12	 C 	  1311.75	   620.40	  5
 13	 P 	  1408.80	   460.23	  4
 14	 M 	  1539.84	   363.18	  3
 15	 G 	  1596.86	   232.14	  2
 16	 R 	        -  	   175.12	  1  _
+2 charge
		      B   	     Y
  1	 I 	    57.55	        -  	 16
  2	 S 	   101.07	   829.45	 15
  3	 S 	   144.58	   785.93	 14
  4	 Q 	   208.61	   742.42	 13
  5	 E 	   273.13	   678.39	 12
  6	 A 	   308.65	   613.86	 11
  7	 Q 	   372.68	   578.35	 10
  8	 G 	   401.19	   514.32	  9
  9	 P 	   449.72	   485.81	  8
 10	 P 	   498.24	   437.28	  7
 11	 R 	   576.29	   388.75	  6
 12	 C 	   656.38	   310.70	  5
 13	 P 	   704.90	   230.62	  4
 14	 M 	   770.42	   182.09	  3
 15	 G 	   798.93	   116.57	  2
 16	 R 	        -  	    88.06	  1  _
```

**58. IPI00021842 Apolipoprotein E**

| Sequence | Charge | M+H+ | XCorr | DeltaCn |
| --- | --- | --- | --- | --- |
| R.LKSWFEPLVEDMQR.Q | 2 | 1779.053 | 3.282 | 0.545 |

+1 charge

```
		      B   	     Y
  1	 L 	   114.09	        -  	 14
  2	 K 	   242.19	  1664.81	 13
  3	 S 	   329.22	  1536.72	 12
  4	 W 	   515.30	  1449.68	 11
  5	 F 	   662.37	  1263.60	 10
  6	 E 	   791.41	  1116.54	  9
  7	 P 	   888.46	   987.49	  8
  8	 L 	  1001.55	   890.44	  7
  9	 V 	  1100.61	   777.36	  6
 10	 E 	  1229.66	   678.29	  5
 11	 D 	  1344.68	   549.24	  4
 12	 M 	  1475.72	   434.22	  3
 13	 Q 	  1603.78	   303.18	  2
 14	 R 	        -  	   175.12	  1  _
+2 charge
		      B   	     Y
  1	 L 	    57.55	        -  	 14
  2	 K 	   121.60	   832.91	 13
  3	 S 	   165.11	   768.86	 12
  4	 W 	   258.15	   725.35	 11
  5	 F 	   331.69	   632.31	 10
  6	 E 	   396.21	   558.77	  9
  7	 P 	   444.73	   494.25	  8
  8	 L 	   501.28	   445.72	  7
  9	 V 	   550.81	   389.18	  6
 10	 E 	   615.33	   339.65	  5
 11	 D 	   672.85	   275.13	  4
 12	 M 	   738.37	   217.61	  3
 13	 Q 	   802.39	   152.09	  2
 14	 R 	        -  	    88.06	  1  _
```

**59. IPI00010214 Protein S100-A14**

| Sequence | Charge | M+H+ | XCorr | DeltaCn |
| --- | --- | --- | --- | --- |
| R.SFWELIGEAAK.S | 2 | 1251.412 | 2.996 | 0.478 |

+1 charge

```
		      B   	     Y
  1	 S 	    88.04	        -  	 11
  2	 F 	   235.11	  1163.61	 10
  3	 W 	   421.19	  1016.54	  9
  4	 E 	   550.23	   830.46	  8
  5	 L 	   663.31	   701.42	  7
  6	 I 	   776.40	   588.34	  6
  7	 G 	   833.42	   475.25	  5
  8	 E 	   962.46	   418.23	  4
  9	 A 	  1033.50	   289.19	  3
 10	 A 	  1104.54	   218.15	  2
 11	 K 	        -  	   147.11	  1  _
+2 charge
		      B   	     Y
  1	 S 	    44.52	        -  	 11
  2	 F 	   118.06	   582.31	 10
  3	 W 	   211.10	   508.77	  9
  4	 E 	   275.62	   415.73	  8
  5	 L 	   332.16	   351.21	  7
  6	 I 	   388.70	   294.67	  6
  7	 G 	   417.21	   238.13	  5
  8	 E 	   481.73	   209.62	  4
  9	 A 	   517.25	   145.10	  3
 10	 A 	   552.77	   109.58	  2
 11	 K 	        -  	    74.06	  1  _
```

**60. IPI00021263 14-3-3 protein zeta/delta**

| Sequence | Charge | M+H+ | XCorr | DeltaCn |
| --- | --- | --- | --- | --- |
| K.SVTEQGAELSNEERNLLSVAYKNVVGAR.R | 3 | 3035.316 | 4.285 | 0.459 |

+1 charge

```
		      B   	     Y
  1	 S 	    88.04	        -  	 28
  2	 V 	   187.11	  2946.53	 27
  3	 T 	   288.16	  2847.46	 26
  4	 E 	   417.20	  2746.41	 25
  5	 Q 	   545.26	  2617.37	 24
  6	 G 	   602.28	  2489.31	 23
  7	 A 	   673.32	  2432.29	 22
  8	 E 	   802.36	  2361.25	 21
  9	 L 	   915.44	  2232.21	 20
 10	 S 	  1002.47	  2119.13	 19
 11	 N 	  1116.52	  2032.09	 18
 12	 E 	  1245.56	  1918.05	 17
 13	 E 	  1374.60	  1789.01	 16
 14	 R 	  1530.70	  1659.97	 15
 15	 N 	  1644.75	  1503.86	 14
 16	 L 	  1757.83	  1389.82	 13
 17	 L 	  1870.91	  1276.74	 12
 18	 S 	  1957.95	  1163.65	 11
 19	 V 	  2057.01	  1076.62	 10
 20	 A 	  2128.05	   977.55	  9
 21	 Y 	  2291.11	   906.52	  8
 22	 K 	  2419.21	   743.45	  7
 23	 N 	  2533.25	   615.36	  6
 24	 V 	  2632.32	   501.31	  5
 25	 V 	  2731.39	   402.25	  4
 26	 G 	  2788.41	   303.18	  3
 27	 A 	  2859.45	   246.16	  2
 28	 R 	        -  	   175.12	  1  _


+2 charge
		      B   	     Y
  1	 S 	    44.52	        -  	 28
  2	 V 	    94.06	  1473.77	 27
  3	 T 	   144.58	  1424.23	 26
  4	 E 	   209.10	  1373.71	 25
  5	 Q 	   273.13	  1309.19	 24
  6	 G 	   301.64	  1245.16	 23
  7	 A 	   337.16	  1216.65	 22
  8	 E 	   401.68	  1181.13	 21
  9	 L 	   458.22	  1116.61	 20
 10	 S 	   501.74	  1060.07	 19
 11	 N 	   558.76	  1016.55	 18
 12	 E 	   623.28	   959.53	 17
 13	 E 	   687.80	   895.01	 16
 14	 R 	   765.86	   830.49	 15
 15	 N 	   822.88	   752.44	 14
 16	 L 	   879.42	   695.41	 13
 17	 L 	   935.96	   638.87	 12
 18	 S 	   979.48	   582.33	 11
 19	 V 	  1029.01	   538.81	 10
 20	 A 	  1064.53	   489.28	  9
 21	 Y 	  1146.06	   453.76	  8
 22	 K 	  1210.11	   372.23	  7
 23	 N 	  1267.13	   308.18	  6
 24	 V 	  1316.66	   251.16	  5
 25	 V 	  1366.20	   201.63	  4
 26	 G 	  1394.71	   152.09	  3
 27	 A 	  1430.23	   123.58	  2
 28	 R 	        -  	    88.06	  1  _


+3 charge
		      B   	     Y
  1	 S 	    30.02	        -  	 28
  2	 V 	    63.04	   982.85	 27
  3	 T 	    96.72	   949.82	 26
  4	 E 	   139.74	   916.14	 25
  5	 Q 	   182.42	   873.13	 24
  6	 G 	   201.43	   830.44	 23
  7	 A 	   225.11	   811.43	 22
  8	 E 	   268.12	   787.76	 21
  9	 L 	   305.82	   744.74	 20
 10	 S 	   334.83	   707.05	 19
 11	 N 	   372.84	   678.04	 18
 12	 E 	   415.86	   640.02	 17
 13	 E 	   458.87	   597.01	 16
 14	 R 	   510.91	   553.99	 15
 15	 N 	   548.92	   501.96	 14
 16	 L 	   586.61	   463.95	 13
 17	 L 	   624.31	   426.25	 12
 18	 S 	   653.32	   388.56	 11
 19	 V 	   686.34	   359.55	 10
 20	 A 	   710.02	   326.52	  9
 21	 Y 	   764.38	   302.84	  8
 22	 K 	   807.07	   248.49	  7
 23	 N 	   845.09	   205.79	  6
 24	 V 	   878.11	   167.78	  5
 25	 V 	   911.13	   134.75	  4
 26	 G 	   930.14	   101.73	  3
 27	 A 	   953.82	    82.72	  2
 28	 R 	        -  	    59.04	  1  _
```

**61. IPI00000828 Proenkephalin-A**

| Sequence | Charge | M+H+ | XCorr | DeltaCn |
| --- | --- | --- | --- | --- |
| K.DAEEDDSLANSSDLLKELLETGDNRER.S | 3 | 3036.123 | 4.308 | 0.495 |

+1 charge

```
		      B   	     Y
  1	 D 	   116.03	        -  	 27
  2	 A 	   187.07	  2919.38	 26
  3	 E 	   316.11	  2848.34	 25
  4	 E 	   445.16	  2719.30	 24
  5	 D 	   560.18	  2590.26	 23
  6	 D 	   675.21	  2475.23	 22
  7	 S 	   762.24	  2360.21	 21
  8	 L 	   875.33	  2273.17	 20
  9	 A 	   946.36	  2160.09	 19
 10	 N 	  1060.41	  2089.05	 18
 11	 S 	  1147.44	  1975.01	 17
 12	 S 	  1234.47	  1887.98	 16
 13	 D 	  1349.50	  1800.95	 15
 14	 L 	  1462.58	  1685.92	 14
 15	 L 	  1575.67	  1572.83	 13
 16	 K 	  1703.76	  1459.75	 12
 17	 E 	  1832.80	  1331.65	 11
 18	 L 	  1945.89	  1202.61	 10
 19	 L 	  2058.97	  1089.53	  9
 20	 E 	  2188.01	   976.44	  8
 21	 T 	  2289.06	   847.40	  7
 22	 G 	  2346.08	   746.35	  6
 23	 D 	  2461.11	   689.33	  5
 24	 N 	  2575.15	   574.31	  4
 25	 R 	  2731.25	   460.26	  3
 26	 E 	  2860.30	   304.16	  2
 27	 R 	        -  	   175.12	  1  _


+2 charge
		      B   	     Y
  1	 D 	    58.52	        -  	 27
  2	 A 	    94.04	  1460.19	 26
  3	 E 	   158.56	  1424.68	 25
  4	 E 	   223.08	  1360.15	 24
  5	 D 	   280.60	  1295.63	 23
  6	 D 	   338.11	  1238.12	 22
  7	 S 	   381.62	  1180.61	 21
  8	 L 	   438.17	  1137.09	 20
  9	 A 	   473.69	  1080.55	 19
 10	 N 	   530.71	  1045.03	 18
 11	 S 	   574.22	   988.01	 17
 12	 S 	   617.74	   944.49	 16
 13	 D 	   675.25	   900.98	 15
 14	 L 	   731.79	   843.46	 14
 15	 L 	   788.34	   786.92	 13
 16	 K 	   852.38	   730.38	 12
 17	 E 	   916.91	   666.33	 11
 18	 L 	   973.45	   601.81	 10
 19	 L 	  1029.99	   545.27	  9
 20	 E 	  1094.51	   488.73	  8
 21	 T 	  1145.03	   424.20	  7
 22	 G 	  1173.55	   373.68	  6
 23	 D 	  1231.06	   345.17	  5
 24	 N 	  1288.08	   287.66	  4
 25	 R 	  1366.13	   230.63	  3
 26	 E 	  1430.65	   152.58	  2
 27	 R 	        -  	    88.06	  1  _


+3 charge
		      B   	     Y
  1	 D 	    39.35	        -  	 27
  2	 A 	    63.03	   973.80	 26
  3	 E 	   106.04	   950.12	 25
  4	 E 	   149.06	   907.11	 24
  5	 D 	   187.40	   864.09	 23
  6	 D 	   225.74	   825.75	 22
  7	 S 	   254.75	   787.41	 21
  8	 L 	   292.45	   758.40	 20
  9	 A 	   316.13	   720.70	 19
 10	 N 	   354.14	   697.02	 18
 11	 S 	   383.15	   659.01	 17
 12	 S 	   412.16	   630.00	 16
 13	 D 	   450.50	   600.99	 15
 14	 L 	   488.20	   562.64	 14
 15	 L 	   525.89	   524.95	 13
 16	 K 	   568.59	   487.25	 12
 17	 E 	   611.61	   444.56	 11
 18	 L 	   649.30	   401.54	 10
 19	 L 	   687.00	   363.85	  9
 20	 E 	   730.01	   326.15	  8
 21	 T 	   763.69	   283.14	  7
 22	 G 	   782.70	   249.46	  6
 23	 D 	   821.04	   230.45	  5
 24	 N 	   859.06	   192.11	  4
 25	 R 	   911.09	   154.09	  3
 26	 E 	   954.10	   102.06	  2
 27	 R 	        -  	    59.04	  1  _
```

**62. IPI00658073 Coiled-coil domain-containing protein 126**

| Sequence | Charge | M+H+ | XCorr | DeltaCn |
| --- | --- | --- | --- | --- |
| R.TIAVLLDDILQR.L | 2 | 1370.62 | 3.309 | 0.268 |

+1 charge

```
		      B   	     Y
  1	 T 	   102.05	        -  	 12
  2	 I 	   215.14	  1268.76	 11
  3	 A 	   286.18	  1155.67	 10
  4	 V 	   385.24	  1084.64	  9
  5	 L 	   498.33	   985.57	  8
  6	 L 	   611.41	   872.48	  7
  7	 D 	   726.44	   759.40	  6
  8	 D 	   841.47	   644.37	  5
  9	 I 	   954.55	   529.35	  4
 10	 L 	  1067.63	   416.26	  3
 11	 Q 	  1195.69	   303.18	  2
 12	 R 	        -  	   175.12	  1  _
+2 charge
		      B   	     Y
  1	 T 	    51.53	        -  	 12
  2	 I 	   108.07	   634.88	 11
  3	 A 	   143.59	   578.34	 10
  4	 V 	   193.13	   542.82	  9
  5	 L 	   249.67	   493.29	  8
  6	 L 	   306.21	   436.75	  7
  7	 D 	   363.72	   380.20	  6
  8	 D 	   421.24	   322.69	  5
  9	 I 	   477.78	   265.18	  4
 10	 L 	   534.32	   208.63	  3
 11	 Q 	   598.35	   152.09	  2
 12	 R 	        -  	    88.06	  1  _
```

**63. IPI00940960 Epididymal secretory protein E1**

| Sequence | Charge | M+H+ | XCorr | DeltaCn |
| --- | --- | --- | --- | --- |
| K.EVNVSPCPTQPCQLSK.G | 2 | 1845.031 | 3.057 | 0.39 |

+1 charge

```
		      B   	     Y
  1	 E 	   130.05	        -  	 16
  2	 V 	   229.12	  1715.09	 15
  3	 N 	   343.16	  1616.03	 14
  4	 V 	   442.23	  1501.98	 13
  5	 S 	   529.26	  1402.91	 12
  6	 P 	   626.31	  1315.88	 11
  7	 C 	   786.48	  1218.83	 10
  8	 P 	   883.53	  1058.66	  9
  9	 T 	   984.58	   961.61	  8
 10	 Q 	  1112.64	   860.56	  7
 11	 P 	  1209.69	   732.51	  6
 12	 C 	  1369.86	   635.45	  5
 13	 Q 	  1497.92	   475.29	  4
 14	 L 	  1611.00	   347.23	  3
 15	 S 	  1698.03	   234.14	  2
 16	 K 	        -  	   147.11	  1  _
+2 charge
		      B   	     Y
  1	 E 	    65.53	        -  	 16
  2	 V 	   115.06	   858.05	 15
  3	 N 	   172.08	   808.52	 14
  4	 V 	   221.62	   751.50	 13
  5	 S 	   265.13	   701.96	 12
  6	 P 	   313.66	   658.44	 11
  7	 C 	   393.74	   609.92	 10
  8	 P 	   442.27	   529.84	  9
  9	 T 	   492.79	   481.31	  8
 10	 Q 	   556.82	   430.79	  7
 11	 P 	   605.35	   366.76	  6
 12	 C 	   685.43	   318.23	  5
 13	 Q 	   749.46	   238.15	  4
 14	 L 	   806.00	   174.12	  3
 15	 S 	   849.52	   117.58	  2
 16	 K 	        -  	    74.06	  1  _
```

**64. IPI00892695 Putative uncharacterized protein BRE**

| Sequence | Charge | M+H+ | XCorr | DeltaCn |
| --- | --- | --- | --- | --- |
| R.IEQLSLSPCHTDFLLTR.F | 2 | 2031.292 | 2.811 | 0.205 |

+1 charge

```
		      B   	     Y
  1	 I 	   114.09	        -  	 17
  2	 E 	   243.13	  1917.09	 16
  3	 Q 	   371.19	  1788.05	 15
  4	 L 	   484.28	  1659.99	 14
  5	 S 	   571.31	  1546.90	 13
  6	 L 	   684.39	  1459.87	 12
  7	 S 	   771.42	  1346.79	 11
  8	 P 	   868.48	  1259.75	 10
  9	 C 	  1028.64	  1162.70	  9
 10	 H 	  1165.70	  1002.54	  8
 11	 T 	  1266.75	   865.48	  7
 12	 D 	  1381.78	   764.43	  6
 13	 F 	  1528.84	   649.40	  5
 14	 L 	  1641.93	   502.33	  4
 15	 L 	  1755.01	   389.25	  3
 16	 T 	  1856.06	   276.17	  2
 17	 R 	        -  	   175.12	  1  _
+2 charge
		      B   	     Y
  1	 I 	    57.55	        -  	 17
  2	 E 	   122.07	   959.05	 16
  3	 Q 	   186.10	   894.53	 15
  4	 L 	   242.64	   830.50	 14
  5	 S 	   286.16	   773.96	 13
  6	 L 	   342.70	   730.44	 12
  7	 S 	   386.22	   673.90	 11
  8	 P 	   434.74	   630.38	 10
  9	 C 	   514.82	   581.85	  9
 10	 H 	   583.35	   501.77	  8
 11	 T 	   633.88	   433.24	  7
 12	 D 	   691.39	   382.72	  6
 13	 F 	   764.93	   325.21	  5
 14	 L 	   821.47	   251.67	  4
 15	 L 	   878.01	   195.13	  3
 16	 T 	   928.53	   138.59	  2
 17	 R 	        -  	    88.06	  1  _
```

**65. IPI00009477 Intercellular adhesion molecule 2**

| Sequence | Charge | M+H+ | XCorr | DeltaCn |
| --- | --- | --- | --- | --- |
| R.QVILTLQPTLVAVGK.S | 2 | 1580.937 | 3.59 | 0.501 |

+1 charge

```
		      B   	     Y
  1	 Q 	   129.07	        -  	 15
  2	 V 	   228.13	  1451.92	 14
  3	 I 	   341.22	  1352.85	 13
  4	 L 	   454.30	  1239.77	 12
  5	 T 	   555.35	  1126.68	 11
  6	 L 	   668.43	  1025.64	 10
  7	 Q 	   796.49	   912.55	  9
  8	 P 	   893.55	   784.49	  8
  9	 T 	   994.59	   687.44	  7
 10	 L 	  1107.68	   586.39	  6
 11	 V 	  1206.75	   473.31	  5
 12	 A 	  1277.78	   374.24	  4
 13	 V 	  1376.85	   303.20	  3
 14	 G 	  1433.87	   204.13	  2
 15	 K 	        -  	   147.11	  1  _
+2 charge
		      B   	     Y
  1	 Q 	    65.04	        -  	 15
  2	 V 	   114.57	   726.46	 14
  3	 I 	   171.11	   676.93	 13
  4	 L 	   227.65	   620.39	 12
  5	 T 	   278.18	   563.85	 11
  6	 L 	   334.72	   513.32	 10
  7	 Q 	   398.75	   456.78	  9
  8	 P 	   447.28	   392.75	  8
  9	 T 	   497.80	   344.22	  7
 10	 L 	   554.34	   293.70	  6
 11	 V 	   603.88	   237.16	  5
 12	 A 	   639.40	   187.62	  4
 13	 V 	   688.93	   152.10	  3
 14	 G 	   717.44	   102.57	  2
 15	 K 	        -  	    74.06	  1  _
```

**66. IPI00940353 Isoform 1 of Gamma-glutamyltransferase 6**

| Sequence | Charge | M+H+ | XCorr | DeltaCn |
| --- | --- | --- | --- | --- |
| R.LLVGPTTLAQEGFLVDTPLAR.A | 2 | 2212.574 | 4.665 | 0.603 |

+1 charge

```
		      B   	     Y
  1	 L 	   114.09	        -  	 21
  2	 L 	   227.18	  2098.15	 20
  3	 V 	   326.24	  1985.07	 19
  4	 G 	   383.27	  1886.00	 18
  5	 P 	   480.32	  1828.98	 17
  6	 T 	   581.37	  1731.93	 16
  7	 T 	   682.41	  1630.88	 15
  8	 L 	   795.50	  1529.83	 14
  9	 A 	   866.53	  1416.75	 13
 10	 Q 	   994.59	  1345.71	 12
 11	 E 	  1123.64	  1217.65	 11
 12	 G 	  1180.66	  1088.61	 10
 13	 F 	  1327.73	  1031.59	  9
 14	 L 	  1440.81	   884.52	  8
 15	 V 	  1539.88	   771.44	  7
 16	 D 	  1654.91	   672.37	  6
 17	 T 	  1755.95	   557.34	  5
 18	 P 	  1853.01	   456.29	  4
 19	 L 	  1966.09	   359.24	  3
 20	 A 	  2037.13	   246.16	  2
 21	 R 	        -  	   175.12	  1  _
+2 charge
		      B   	     Y
  1	 L 	    57.55	        -  	 21
  2	 L 	   114.09	  1049.58	 20
  3	 V 	   163.63	   993.04	 19
  4	 G 	   192.14	   943.50	 18
  5	 P 	   240.66	   914.99	 17
  6	 T 	   291.19	   866.47	 16
  7	 T 	   341.71	   815.94	 15
  8	 L 	   398.25	   765.42	 14
  9	 A 	   433.77	   708.88	 13
 10	 Q 	   497.80	   673.36	 12
 11	 E 	   562.32	   609.33	 11
 12	 G 	   590.83	   544.81	 10
 13	 F 	   664.37	   516.30	  9
 14	 L 	   720.91	   442.76	  8
 15	 V 	   770.44	   386.22	  7
 16	 D 	   827.96	   336.69	  6
 17	 T 	   878.48	   279.17	  5
 18	 P 	   927.01	   228.65	  4
 19	 L 	   983.55	   180.12	  3
 20	 A 	  1019.07	   123.58	  2
 21	 R 	        -  	    88.06	  1  _
```

**67. IPI00005181 Phospholipid scramblase 1**

| Sequence | Charge | M+H+ | XCorr | DeltaCn |
| --- | --- | --- | --- | --- |
| R.VYFAAEDTDCCTR.N | 2 | 1608.675 | 3.666 | 0.602 |

+1 charge

```
		      B   	     Y
  1	 V 	   100.08	        -  	 13
  2	 Y 	   263.14	  1508.85	 12
  3	 F 	   410.21	  1345.78	 11
  4	 A 	   481.24	  1198.72	 10
  5	 A 	   552.28	  1127.68	  9
  6	 E 	   681.32	  1056.64	  8
  7	 D 	   796.35	   927.60	  7
  8	 T 	   897.40	   812.57	  6
  9	 D 	  1012.43	   711.52	  5
 10	 C 	  1172.59	   596.50	  4
 11	 C 	  1332.76	   436.33	  3
 12	 T 	  1433.80	   276.17	  2
 13	 R 	        -  	   175.12	  1  _
+2 charge
		      B   	     Y
  1	 V 	    50.54	        -  	 13
  2	 Y 	   132.07	   754.93	 12
  3	 F 	   205.61	   673.40	 11
  4	 A 	   241.13	   599.86	 10
  5	 A 	   276.64	   564.34	  9
  6	 E 	   341.17	   528.82	  8
  7	 D 	   398.68	   464.30	  7
  8	 T 	   449.20	   406.79	  6
  9	 D 	   506.72	   356.27	  5
 10	 C 	   586.80	   298.75	  4
 11	 C 	   666.88	   218.67	  3
 12	 T 	   717.41	   138.59	  2
 13	 R 	        -  	    88.06	  1  _
```

**68. IPI00873371 Isoform 1 of Latent-transforming growth factor beta-binding protein 4**

| Sequence | Charge | M+H+ | XCorr | DeltaCn |
| --- | --- | --- | --- | --- |
| R.CVRVPEGFTCR.C | 3 | 1381.55 | 3.401 | 0.436 |

+1 charge

```
		      B   	     Y
  1	 C 	   161.17	        -  	 11
  2	 V 	   260.24	  1220.76	 10
  3	 R 	   416.34	  1121.69	  9
  4	 V 	   515.41	   965.59	  8
  5	 P 	   612.46	   866.52	  7
  6	 E 	   741.51	   769.46	  6
  7	 G 	   798.53	   640.42	  5
  8	 F 	   945.60	   583.40	  4
  9	 T 	  1046.64	   436.33	  3
 10	 C 	  1206.81	   335.28	  2
 11	 R 	        -  	   175.12	  1  _
+2 charge
		      B   	     Y
  1	 C 	    81.09	        -  	 11
  2	 V 	   130.62	   610.88	 10
  3	 R 	   208.67	   561.35	  9
  4	 V 	   258.21	   483.30	  8
  5	 P 	   306.74	   433.76	  7
  6	 E 	   371.26	   385.24	  6
  7	 G 	   399.77	   320.71	  5
  8	 F 	   473.30	   292.20	  4
  9	 T 	   523.83	   218.67	  3
 10	 C 	   603.91	   168.15	  2
 11	 R 	        -  	    88.06	  1  _
+3 charge
		      B   	     Y
  1	 C 	    54.40	        -  	 11
  2	 V 	    87.42	   407.59	 10
  3	 R 	   139.45	   374.57	  9
  4	 V 	   172.47	   322.53	  8
  5	 P 	   204.83	   289.51	  7
  6	 E 	   247.84	   257.16	  6
  7	 G 	   266.85	   214.15	  5
  8	 F 	   315.87	   195.14	  4
  9	 T 	   349.55	   146.12	  3
 10	 C 	   402.94	   112.43	  2
 11	 R 	        -  	    59.04	  1  _
```

**69. IPI00465248 Isoform alpha-enolase of Alpha-enolase**

| Sequence | Charge | M+H+ | XCorr | DeltaCn |
| --- | --- | --- | --- | --- |
| K.LAQANGWGVMVSHR.S | 2 | 1526.748 | 2.885 | 0.294 |

+1 charge

```
		      B   	     Y
  1	 L 	   114.09	        -  	 14
  2	 A 	   185.13	  1412.69	 13
  3	 Q 	   313.19	  1341.65	 12
  4	 A 	   384.22	  1213.59	 11
  5	 N 	   498.27	  1142.55	 10
  6	 G 	   555.29	  1028.51	  9
  7	 W 	   741.37	   971.49	  8
  8	 G 	   798.39	   785.41	  7
  9	 V 	   897.46	   728.39	  6
 10	 M 	  1028.50	   629.32	  5
 11	 V 	  1127.57	   498.28	  4
 12	 S 	  1214.60	   399.21	  3
 13	 H 	  1351.66	   312.18	  2
 14	 R 	        -  	   175.12	  1  _
+2 charge
		      B   	     Y
  1	 L 	    57.55	        -  	 14
  2	 A 	    93.07	   706.85	 13
  3	 Q 	   157.10	   671.33	 12
  4	 A 	   192.62	   607.30	 11
  5	 N 	   249.64	   571.78	 10
  6	 G 	   278.15	   514.76	  9
  7	 W 	   371.19	   486.25	  8
  8	 G 	   399.70	   393.21	  7
  9	 V 	   449.23	   364.70	  6
 10	 M 	   514.75	   315.16	  5
 11	 V 	   564.29	   249.64	  4
 12	 S 	   607.80	   200.11	  3
 13	 H 	   676.33	   156.59	  2
 14	 R 	        -  	    88.06	  1  _
```

**70. IPI00297714 Gamma-synuclein**

| Sequence | Charge | M+H+ | XCorr | DeltaCn |
| --- | --- | --- | --- | --- |
| K.TVEEAENIAVTSGVVR.K | 2 | 1674.834 | 3.141 | 0.425 |

+1 charge

```
		      B   	     Y
  1	 T 	   102.05	        -  	 16
  2	 V 	   201.12	  1572.82	 15
  3	 E 	   330.17	  1473.75	 14
  4	 E 	   459.21	  1344.71	 13
  5	 A 	   530.25	  1215.67	 12
  6	 E 	   659.29	  1144.63	 11
  7	 N 	   773.33	  1015.59	 10
  8	 I 	   886.42	   901.55	  9
  9	 A 	   957.45	   788.46	  8
 10	 V 	  1056.52	   717.43	  7
 11	 T 	  1157.57	   618.36	  6
 12	 S 	  1244.60	   517.31	  5
 13	 G 	  1301.62	   430.28	  4
 14	 V 	  1400.69	   373.26	  3
 15	 V 	  1499.76	   274.19	  2
 16	 R 	        -  	   175.12	  1  _
+2 charge
		      B   	     Y
  1	 T 	    51.53	        -  	 16
  2	 V 	   101.07	   786.92	 15
  3	 E 	   165.59	   737.38	 14
  4	 E 	   230.11	   672.86	 13
  5	 A 	   265.63	   608.34	 12
  6	 E 	   330.15	   572.82	 11
  7	 N 	   387.17	   508.30	 10
  8	 I 	   443.71	   451.28	  9
  9	 A 	   479.23	   394.73	  8
 10	 V 	   528.76	   359.22	  7
 11	 T 	   579.29	   309.68	  6
 12	 S 	   622.80	   259.16	  5
 13	 G 	   651.31	   215.64	  4
 14	 V 	   700.85	   187.13	  3
 15	 V 	   750.38	   137.60	  2
 16	 R 	        -  	    88.06	  1  _
```

**71. IPI00011694 Trypsin-1**

| Sequence | Charge | M+H+ | XCorr | DeltaCn |
| --- | --- | --- | --- | --- |
| K.TLNNDIMLIK.L | 2 | 1175.424 | 3.632 | 0.462 |

+1 charge

```
		      B   	     Y
  1	 T 	   102.05	        -  	 10
  2	 L 	   215.14	  1073.60	  9
  3	 N 	   329.18	   960.52	  8
  4	 N 	   443.22	   846.48	  7
  5	 D 	   558.25	   732.43	  6
  6	 I 	   671.34	   617.41	  5
  7	 M 	   802.38	   504.32	  4
  8	 L 	   915.46	   373.28	  3
  9	 I 	  1028.54	   260.20	  2
 10	 K 	        -  	   147.11	  1  _
+2 charge
		      B   	     Y
  1	 T 	    51.53	        -  	 10
  2	 L 	   108.07	   537.30	  9
  3	 N 	   165.09	   480.76	  8
  4	 N 	   222.12	   423.74	  7
  5	 D 	   279.63	   366.72	  6
  6	 I 	   336.17	   309.21	  5
  7	 M 	   401.69	   252.66	  4
  8	 L 	   458.23	   187.14	  3
  9	 I 	   514.78	   130.60	  2
 10	 K 	        -  	    74.06	  1  _
```

**72. IPI00382436 Ig lambda chain V-III region SH**

| Sequence | Charge | M+H+ | XCorr | DeltaCn |
| --- | --- | --- | --- | --- |
| - .SELTQDPAVSVALGQTVR.I | 2 | 1872.07 | 3.293 | 0.272 |

+1 charge

```
		      B   	     Y
  1	 S 	    88.04	        -  	 18
  2	 E 	   217.08	  1783.95	 17
  3	 L 	   330.17	  1654.91	 16
  4	 T 	   431.21	  1541.83	 15
  5	 Q 	   559.27	  1440.78	 14
  6	 D 	   674.30	  1312.72	 13
  7	 P 	   771.35	  1197.69	 12
  8	 A 	   842.39	  1100.64	 11
  9	 V 	   941.46	  1029.61	 10
 10	 S 	  1028.49	   930.54	  9
 11	 V 	  1127.56	   843.50	  8
 12	 A 	  1198.60	   744.44	  7
 13	 L 	  1311.68	   673.40	  6
 14	 G 	  1368.70	   560.32	  5
 15	 Q 	  1496.76	   503.29	  4
 16	 T 	  1597.81	   375.24	  3
 17	 V 	  1696.88	   274.19	  2
 18	 R 	        -  	   175.12	  1  _
+2 charge
		      B   	     Y
  1	 S 	    44.52	        -  	 18
  2	 E 	   109.04	   892.48	 17
  3	 L 	   165.59	   827.96	 16
  4	 T 	   216.11	   771.42	 15
  5	 Q 	   280.14	   720.89	 14
  6	 D 	   337.65	   656.86	 13
  7	 P 	   386.18	   599.35	 12
  8	 A 	   421.70	   550.82	 11
  9	 V 	   471.23	   515.31	 10
 10	 S 	   514.75	   465.77	  9
 11	 V 	   564.28	   422.26	  8
 12	 A 	   599.80	   372.72	  7
 13	 L 	   656.34	   337.20	  6
 14	 G 	   684.85	   280.66	  5
 15	 Q 	   748.88	   252.15	  4
 16	 T 	   799.41	   188.12	  3
 17	 V 	   848.94	   137.60	  2
 18	 R 	        -  	    88.06	  1  _
```

**73. IPI00024046 cDNA FLJ52398, highly similar to Cadherin-13**

| Sequence | Charge | M+H+ | XCorr | DeltaCn |
| --- | --- | --- | --- | --- |
| R.MTAFDADDPATDNALLR.Y | 2 | 1837.989 | 5.225 | 0.555 |

+1 charge

```
		      B   	     Y
  1	 M 	   132.05	        -  	 17
  2	 T 	   233.10	  1705.80	 16
  3	 A 	   304.13	  1604.76	 15
  4	 F 	   451.20	  1533.72	 14
  5	 D 	   566.23	  1386.65	 13
  6	 A 	   637.27	  1271.62	 12
  7	 D 	   752.29	  1200.59	 11
  8	 D 	   867.32	  1085.56	 10
  9	 P 	   964.37	   970.53	  9
 10	 A 	  1035.41	   873.48	  8
 11	 T 	  1136.46	   802.44	  7
 12	 D 	  1251.48	   701.39	  6
 13	 N 	  1365.53	   586.37	  5
 14	 A 	  1436.56	   472.32	  4
 15	 L 	  1549.65	   401.29	  3
 16	 L 	  1662.73	   288.20	  2
 17	 R 	        -  	   175.12	  1  _
+2 charge
		      B   	     Y
  1	 M 	    66.53	        -  	 17
  2	 T 	   117.05	   853.41	 16
  3	 A 	   152.57	   802.88	 15
  4	 F 	   226.10	   767.36	 14
  5	 D 	   283.62	   693.83	 13
  6	 A 	   319.14	   636.31	 12
  7	 D 	   376.65	   600.80	 11
  8	 D 	   434.16	   543.28	 10
  9	 P 	   482.69	   485.77	  9
 10	 A 	   518.21	   437.24	  8
 11	 T 	   568.73	   401.72	  7
 12	 D 	   626.25	   351.20	  6
 13	 N 	   683.27	   293.69	  5
 14	 A 	   718.79	   236.67	  4
 15	 L 	   775.33	   201.15	  3
 16	 L 	   831.87	   144.61	  2
 17	 R 	        -  	    88.06	  1  _
```

**74. IPI00658038 Isoform 3 of Leukocyte immunoglobulin-like receptor subfamily B member 4**

| Sequence | Charge | M+H+ | XCorr | DeltaCn |
| --- | --- | --- | --- | --- |
| R.SPMDTFLLIKER.A | 2 | 1450.728 | 3.322 | 0.335 |

+1 charge

```
		      B   	     Y
  1	 S 	    88.04	        -  	 12
  2	 P 	   185.09	  1362.74	 11
  3	 M 	   316.13	  1265.69	 10
  4	 D 	   431.16	  1134.65	  9
  5	 T 	   532.21	  1019.62	  8
  6	 F 	   679.28	   918.58	  7
  7	 L 	   792.36	   771.51	  6
  8	 L 	   905.44	   658.42	  5
  9	 I 	  1018.53	   545.34	  4
 10	 K 	  1146.62	   432.26	  3
 11	 E 	  1275.67	   304.16	  2
 12	 R 	        -  	   175.12	  1  _
+2 charge
		      B   	     Y
  1	 S 	    44.52	        -  	 12
  2	 P 	    93.05	   681.88	 11
  3	 M 	   158.57	   633.35	 10
  4	 D 	   216.08	   567.83	  9
  5	 T 	   266.61	   510.32	  8
  6	 F 	   340.14	   459.79	  7
  7	 L 	   396.68	   386.26	  6
  8	 L 	   453.23	   329.72	  5
  9	 I 	   509.77	   273.17	  4
 10	 K 	   573.82	   216.63	  3
 11	 E 	   638.34	   152.58	  2
 12	 R 	        -  	    88.06	  1  _
```

**75. IPI00029275 Isoform 1 of Melanotransferrin**

| Sequence | Charge | M+H+ | XCorr | DeltaCn |
| --- | --- | --- | --- | --- |
| R.SCHAGFGSPAGWDVPVGALIQR.G | 3 | 2283.524 | 3.495 | 0.359 |

+1 charge

```
		      B   	     Y
  1	 S 	    88.04	        -  	 22
  2	 C 	   248.20	  2195.22	 21
  3	 H 	   385.26	  2035.05	 20
  4	 A 	   456.30	  1897.99	 19
  5	 G 	   513.32	  1826.95	 18
  6	 F 	   660.39	  1769.93	 17
  7	 G 	   717.41	  1622.86	 16
  8	 S 	   804.44	  1565.84	 15
  9	 P 	   901.50	  1478.81	 14
 10	 A 	   972.53	  1381.76	 13
 11	 G 	  1029.56	  1310.72	 12
 12	 W 	  1215.63	  1253.70	 11
 13	 D 	  1330.66	  1067.62	 10
 14	 V 	  1429.73	   952.59	  9
 15	 P 	  1526.78	   853.53	  8
 16	 V 	  1625.85	   756.47	  7
 17	 G 	  1682.87	   657.40	  6
 18	 A 	  1753.91	   600.38	  5
 19	 L 	  1866.99	   529.35	  4
 20	 I 	  1980.08	   416.26	  3
 21	 Q 	  2108.14	   303.18	  2
 22	 R 	        -  	   175.12	  1  _
+2 charge
		      B   	     Y
  1	 S 	    44.52	        -  	 22
  2	 C 	   124.61	  1098.11	 21
  3	 H 	   193.14	  1018.03	 20
  4	 A 	   228.65	   949.50	 19
  5	 G 	   257.16	   913.98	 18
  6	 F 	   330.70	   885.47	 17
  7	 G 	   359.21	   811.94	 16
  8	 S 	   402.73	   783.43	 15
  9	 P 	   451.25	   739.91	 14
 10	 A 	   486.77	   691.38	 13
 11	 G 	   515.28	   655.86	 12
 12	 W 	   608.32	   627.35	 11
 13	 D 	   665.83	   534.31	 10
 14	 V 	   715.37	   476.80	  9
 15	 P 	   763.89	   427.27	  8
 16	 V 	   813.43	   378.74	  7
 17	 G 	   841.94	   329.21	  6
 18	 A 	   877.46	   300.70	  5
 19	 L 	   934.00	   265.18	  4
 20	 I 	   990.54	   208.63	  3
 21	 Q 	  1054.57	   152.09	  2
 22	 R 	        -  	    88.06	  1  _
+3 charge
		      B   	     Y
  1	 S 	    30.02	        -  	 22
  2	 C 	    83.41	   732.41	 21
  3	 H 	   129.09	   679.02	 20
  4	 A 	   152.77	   633.34	 19
  5	 G 	   171.78	   609.66	 18
  6	 F 	   220.80	   590.65	 17
  7	 G 	   239.81	   541.63	 16
  8	 S 	   268.82	   522.62	 15
  9	 P 	   301.17	   493.61	 14
 10	 A 	   324.85	   461.26	 13
 11	 G 	   343.86	   437.58	 12
 12	 W 	   405.88	   418.57	 11
 13	 D 	   444.23	   356.55	 10
 14	 V 	   477.25	   318.20	  9
 15	 P 	   509.60	   285.18	  8
 16	 V 	   542.62	   252.83	  7
 17	 G 	   561.63	   219.81	  6
 18	 A 	   585.31	   200.80	  5
 19	 L 	   623.00	   177.12	  4
 20	 I 	   660.70	   139.43	  3
 21	 Q 	   703.38	   101.73	  2
 22	 R 	        -  	    59.04	  1  _
```

**76. IPI00025846 Isoform 2A of Desmocollin-2**

| Sequence | Charge | M+H+ | XCorr | DeltaCn |
| --- | --- | --- | --- | --- |
| R.LSYQNDPPFGSYVVPITVR.D | 2 | 2153.422 | 3.86 | 0.592 |

+1 charge

```
		      B   	     Y
  1	 L 	   114.09	        -  	 19
  2	 S 	   201.12	  2039.02	 18
  3	 Y 	   364.19	  1951.99	 17
  4	 Q 	   492.25	  1788.93	 16
  5	 N 	   606.29	  1660.87	 15
  6	 D 	   721.32	  1546.83	 14
  7	 P 	   818.37	  1431.80	 13
  8	 P 	   915.42	  1334.75	 12
  9	 F 	  1062.49	  1237.69	 11
 10	 G 	  1119.51	  1090.63	 10
 11	 S 	  1206.54	  1033.60	  9
 12	 Y 	  1369.61	   946.57	  8
 13	 V 	  1468.67	   783.51	  7
 14	 V 	  1567.74	   684.44	  6
 15	 P 	  1664.80	   585.37	  5
 16	 I 	  1777.88	   488.32	  4
 17	 T 	  1878.93	   375.24	  3
 18	 V 	  1978.00	   274.19	  2
 19	 R 	        -  	   175.12	  1  _
+2 charge
		      B   	     Y
  1	 L 	    57.55	        -  	 19
  2	 S 	   101.07	  1020.02	 18
  3	 Y 	   182.60	   976.50	 17
  4	 Q 	   246.63	   894.97	 16
  5	 N 	   303.65	   830.94	 15
  6	 D 	   361.16	   773.92	 14
  7	 P 	   409.69	   716.40	 13
  8	 P 	   458.21	   667.88	 12
  9	 F 	   531.75	   619.35	 11
 10	 G 	   560.26	   545.82	 10
 11	 S 	   603.77	   517.31	  9
 12	 Y 	   685.31	   473.79	  8
 13	 V 	   734.84	   392.26	  7
 14	 V 	   784.38	   342.72	  6
 15	 P 	   832.90	   293.19	  5
 16	 I 	   889.44	   244.66	  4
 17	 T 	   939.97	   188.12	  3
 18	 V 	   989.50	   137.60	  2
 19	 R 	        -  	    88.06	  1  _
```

**77. IPI00936772 Isoform 1 of Golgi membrane protein 1**

| Sequence | Charge | M+H+ | XCorr | DeltaCn |
| --- | --- | --- | --- | --- |
| R.LQAAGLPHTEVPQGK.G | 2 | 1546.753 | 3.251 | 0.456 |

+1 charge

```
		      B   	     Y
  1	 L 	   114.09	        -  	 15
  2	 Q 	   242.15	  1432.75	 14
  3	 A 	   313.19	  1304.70	 13
  4	 A 	   384.22	  1233.66	 12
  5	 G 	   441.25	  1162.62	 11
  6	 L 	   554.33	  1105.60	 10
  7	 P 	   651.38	   992.52	  9
  8	 H 	   788.44	   895.46	  8
  9	 T 	   889.49	   758.40	  7
 10	 E 	  1018.53	   657.36	  6
 11	 V 	  1117.60	   528.31	  5
 12	 P 	  1214.65	   429.25	  4
 13	 Q 	  1342.71	   332.19	  3
 14	 G 	  1399.73	   204.13	  2
 15	 K 	        -  	   147.11	  1  _
+2 charge
		      B   	     Y
  1	 L 	    57.55	        -  	 15
  2	 Q 	   121.58	   716.88	 14
  3	 A 	   157.10	   652.85	 13
  4	 A 	   192.62	   617.33	 12
  5	 G 	   221.13	   581.81	 11
  6	 L 	   277.67	   553.30	 10
  7	 P 	   326.19	   496.76	  9
  8	 H 	   394.72	   448.24	  8
  9	 T 	   445.25	   379.71	  7
 10	 E 	   509.77	   329.18	  6
 11	 V 	   559.30	   264.66	  5
 12	 P 	   607.83	   215.13	  4
 13	 Q 	   671.86	   166.60	  3
 14	 G 	   700.37	   102.57	  2
 15	 K 	        -  	    74.06	  1  _
```

**78. IPI00657936 Isoform 1 of Collagen alpha-1(XXVIII) chain**

| Sequence | Charge | M+H+ | XCorr | DeltaCn |
| --- | --- | --- | --- | --- |
| R.WYYDKQVNSCAR.F | 2 | 1590.714 | 2.973 | 0.44 |

+1 charge

```
		      B   	     Y
  1	 W 	   187.09	        -  	 12
  2	 Y 	   350.15	  1403.77	 11
  3	 Y 	   513.21	  1240.71	 10
  4	 D 	   628.24	  1077.65	  9
  5	 K 	   756.34	   962.62	  8
  6	 Q 	   884.39	   834.52	  7
  7	 V 	   983.46	   706.46	  6
  8	 N 	  1097.51	   607.40	  5
  9	 S 	  1184.54	   493.35	  4
 10	 C 	  1344.70	   406.32	  3
 11	 A 	  1415.74	   246.16	  2
 12	 R 	        -  	   175.12	  1  _
+2 charge
		      B   	     Y
  1	 W 	    94.05	        -  	 12
  2	 Y 	   175.58	   702.39	 11
  3	 Y 	   257.11	   620.86	 10
  4	 D 	   314.62	   539.33	  9
  5	 K 	   378.67	   481.81	  8
  6	 Q 	   442.70	   417.77	  7
  7	 V 	   492.23	   353.74	  6
  8	 N 	   549.26	   304.20	  5
  9	 S 	   592.77	   247.18	  4
 10	 C 	   672.85	   203.66	  3
 11	 A 	   708.37	   123.58	  2
 12	 R 	        -  	    88.06	  1  _
```

**79. IPI00032325 Cystatin-A**

| Sequence | Charge | M+H+ | XCorr | DeltaCn |
| --- | --- | --- | --- | --- |
| K.TNETYGKLEAVQYK.T | 2 | 1644.807 | 2.886 | 0.403 |

+1 charge

```
		      B   	     Y
  1	 T 	   102.05	        -  	 14
  2	 N 	   216.10	  1542.78	 13
  3	 E 	   345.14	  1428.74	 12
  4	 T 	   446.19	  1299.69	 11
  5	 Y 	   609.25	  1198.65	 10
  6	 G 	   666.27	  1035.58	  9
  7	 K 	   794.37	   978.56	  8
  8	 L 	   907.45	   850.47	  7
  9	 E 	  1036.49	   737.38	  6
 10	 A 	  1107.53	   608.34	  5
 11	 V 	  1206.60	   537.30	  4
 12	 Q 	  1334.66	   438.23	  3
 13	 Y 	  1497.72	   310.18	  2
 14	 K 	        -  	   147.11	  1  _
+2 charge
		      B   	     Y
  1	 T 	    51.53	        -  	 14
  2	 N 	   108.55	   771.89	 13
  3	 E 	   173.07	   714.87	 12
  4	 T 	   223.60	   650.35	 11
  5	 Y 	   305.13	   599.83	 10
  6	 G 	   333.64	   518.30	  9
  7	 K 	   397.69	   489.78	  8
  8	 L 	   454.23	   425.74	  7
  9	 E 	   518.75	   369.20	  6
 10	 A 	   554.27	   304.67	  5
 11	 V 	   603.80	   269.16	  4
 12	 Q 	   667.83	   219.62	  3
 13	 Y 	   749.36	   155.59	  2
 14	 K 	        -  	    74.06	  1  _
```

80. **IPI00009997 N-acetyllactosaminide beta-1,3-N-acetylglucosaminyltransferase**

| Sequence | Charge | M+H+ | XCorr | DeltaCn |
| --- | --- | --- | --- | --- |
| R.SCQEVFDKLAR.V | 2 | 1353.5 | 3.197 | 0.423 |

+1 charge

```
		      B   	     Y
  1	 S 	    88.04	        -  	 11
  2	 C 	   248.20	  1265.77	 10
  3	 Q 	   376.26	  1105.60	  9
  4	 E 	   505.31	   977.54	  8
  5	 V 	   604.37	   848.50	  7
  6	 F 	   751.44	   749.43	  6
  7	 D 	   866.47	   602.36	  5
  8	 K 	   994.56	   487.34	  4
  9	 L 	  1107.65	   359.24	  3
 10	 A 	  1178.69	   246.16	  2
 11	 R 	        -  	   175.12	  1  _
+2 charge
		      B   	     Y
  1	 S 	    44.52	        -  	 11
  2	 C 	   124.61	   633.39	 10
  3	 Q 	   188.64	   553.30	  9
  4	 E 	   253.16	   489.27	  8
  5	 V 	   302.69	   424.75	  7
  6	 F 	   376.22	   375.22	  6
  7	 D 	   433.74	   301.68	  5
  8	 K 	   497.79	   244.17	  4
  9	 L 	   554.33	   180.12	  3
 10	 A 	   589.85	   123.58	  2
 11	 R 	        -  	    88.06	  1  _
```

**81. IPI00026944 Isoform 1 of Nidogen-1**

| Sequence | Charge | M+H+ | XCorr | DeltaCn |
| --- | --- | --- | --- | --- |
| K.VYYREDLSPSITQR.A | 2 | 1727.899 | 4.804 | 0.496 |

+1 charge

```
		      B   	     Y
  1	 V 	   100.08	        -  	 14
  2	 Y 	   263.14	  1627.81	 13
  3	 Y 	   426.20	  1464.74	 12
  4	 R 	   582.30	  1301.68	 11
  5	 E 	   711.35	  1145.58	 10
  6	 D 	   826.37	  1016.54	  9
  7	 L 	   939.46	   901.51	  8
  8	 S 	  1026.49	   788.43	  7
  9	 P 	  1123.54	   701.39	  6
 10	 S 	  1210.57	   604.34	  5
 11	 I 	  1323.66	   517.31	  4
 12	 T 	  1424.71	   404.23	  3
 13	 Q 	  1552.76	   303.18	  2
 14	 R 	        -  	   175.12	  1  _
+2 charge
		      B   	     Y
  1	 V 	    50.54	        -  	 14
  2	 Y 	   132.07	   814.41	 13
  3	 Y 	   213.60	   732.88	 12
  4	 R 	   291.66	   651.34	 11
  5	 E 	   356.18	   573.29	 10
  6	 D 	   413.69	   508.77	  9
  7	 L 	   470.23	   451.26	  8
  8	 S 	   513.75	   394.72	  7
  9	 P 	   562.27	   351.20	  6
 10	 S 	   605.79	   302.67	  5
 11	 I 	   662.33	   259.16	  4
 12	 T 	   712.86	   202.62	  3
 13	 Q 	   776.89	   152.09	  2
 14	 R 	        -  	    88.06	  1  _
```

**82. IPI00294713 Isoform 1 of Mannan-binding lectin serine protease 2**

| Sequence | Charge | M+H+ | XCorr | DeltaCn |
| --- | --- | --- | --- | --- |
| R.LASPGFPGEYANDQER.R | 2 | 1751.834 | 4.134 | 0.539 |

+1 charge

```
		      B   	     Y
  1	 L 	   114.09	        -  	 16
  2	 A 	   185.13	  1637.72	 15
  3	 S 	   272.16	  1566.68	 14
  4	 P 	   369.21	  1479.65	 13
  5	 G 	   426.23	  1382.60	 12
  6	 F 	   573.30	  1325.58	 11
  7	 P 	   670.36	  1178.51	 10
  8	 G 	   727.38	  1081.45	  9
  9	 E 	   856.42	  1024.43	  8
 10	 Y 	  1019.48	   895.39	  7
 11	 A 	  1090.52	   732.33	  6
 12	 N 	  1204.56	   661.29	  5
 13	 D 	  1319.59	   547.25	  4
 14	 Q 	  1447.65	   432.22	  3
 15	 E 	  1576.69	   304.16	  2
 16	 R 	        -  	   175.12	  1  _
+2 charge
		      B   	     Y
  1	 L 	    57.55	        -  	 16
  2	 A 	    93.07	   819.36	 15
  3	 S 	   136.58	   783.84	 14
  4	 P 	   185.11	   740.33	 13
  5	 G 	   213.62	   691.80	 12
  6	 F 	   287.16	   663.29	 11
  7	 P 	   335.68	   589.76	 10
  8	 G 	   364.19	   541.23	  9
  9	 E 	   428.71	   512.72	  8
 10	 Y 	   510.25	   448.20	  7
 11	 A 	   545.76	   366.67	  6
 12	 N 	   602.79	   331.15	  5
 13	 D 	   660.30	   274.13	  4
 14	 Q 	   724.33	   216.61	  3
 15	 E 	   788.85	   152.58	  2
 16	 R 	        -  	    88.06	  1  _
```

**83. IPI00885179 Isoform 1 of Mucin-20**

| Sequence | Charge | M+H+ | XCorr | DeltaCn |
| --- | --- | --- | --- | --- |
| R.LSVASPEDLTDPR.V | 2 | 1400.516 | 2.92 | 0.295 |

+1 charge

```
		      B   	     Y
  1	 L 	   114.09	        -  	 13
  2	 S 	   201.12	  1286.62	 12
  3	 V 	   300.19	  1199.59	 11
  4	 A 	   371.23	  1100.52	 10
  5	 S 	   458.26	  1029.48	  9
  6	 P 	   555.31	   942.45	  8
  7	 E 	   684.36	   845.40	  7
  8	 D 	   799.38	   716.36	  6
  9	 L 	   912.47	   601.33	  5
 10	 T 	  1013.51	   488.25	  4
 11	 D 	  1128.54	   387.20	  3
 12	 P 	  1225.59	   272.17	  2
 13	 R 	        -  	   175.12	  1  _
+2 charge
		      B   	     Y
  1	 L 	    57.55	        -  	 13
  2	 S 	   101.07	   643.81	 12
  3	 V 	   150.60	   600.30	 11
  4	 A 	   186.12	   550.76	 10
  5	 S 	   229.63	   515.25	  9
  6	 P 	   278.16	   471.73	  8
  7	 E 	   342.68	   423.20	  7
  8	 D 	   400.20	   358.68	  6
  9	 L 	   456.74	   301.17	  5
 10	 T 	   507.26	   244.63	  4
 11	 D 	   564.77	   194.10	  3
 12	 P 	   613.30	   136.59	  2
 13	 R 	        -  	    88.06	  1  _
```

**84. IPI00021434 Elafin**

| Sequence | Charge | M+H+ | XCorr | DeltaCn |
| --- | --- | --- | --- | --- |
| K.AQEPVKGPVSTKPGSCPIILIR.C | 3 | 2348.764 | 4.228 | 0.485 |

+1 charge

```
		      B   	     Y
  1	 A 	    72.04	        -  	 22
  2	 Q 	   200.10	  2276.41	 21
  3	 E 	   329.15	  2148.36	 20
  4	 P 	   426.20	  2019.31	 19
  5	 V 	   525.27	  1922.26	 18
  6	 K 	   653.36	  1823.19	 17
  7	 G 	   710.38	  1695.10	 16
  8	 P 	   807.44	  1638.08	 15
  9	 V 	   906.50	  1541.02	 14
 10	 S 	   993.54	  1441.95	 13
 11	 T 	  1094.58	  1354.92	 12
 12	 K 	  1222.68	  1253.87	 11
 13	 P 	  1319.73	  1125.78	 10
 14	 G 	  1376.75	  1028.73	  9
 15	 S 	  1463.79	   971.71	  8
 16	 C 	  1623.95	   884.67	  7
 17	 P 	  1721.00	   724.51	  6
 18	 I 	  1834.09	   627.46	  5
 19	 I 	  1947.17	   514.37	  4
 20	 L 	  2060.26	   401.29	  3
 21	 I 	  2173.34	   288.20	  2
 22	 R 	        -  	   175.12	  1  _
+2 charge
		      B   	     Y
  1	 A 	    36.53	        -  	 22
  2	 Q 	   100.56	  1138.71	 21
  3	 E 	   165.08	  1074.68	 20
  4	 P 	   213.60	  1010.16	 19
  5	 V 	   263.14	   961.63	 18
  6	 K 	   327.18	   912.10	 17
  7	 G 	   355.70	   848.05	 16
  8	 P 	   404.22	   819.54	 15
  9	 V 	   453.76	   771.01	 14
 10	 S 	   497.27	   721.48	 13
 11	 T 	   547.80	   677.96	 12
 12	 K 	   611.84	   627.44	 11
 13	 P 	   660.37	   563.39	 10
 14	 G 	   688.88	   514.87	  9
 15	 S 	   732.40	   486.36	  8
 16	 C 	   812.48	   442.84	  7
 17	 P 	   861.01	   362.76	  6
 18	 I 	   917.55	   314.23	  5
 19	 I 	   974.09	   257.69	  4
 20	 L 	  1030.63	   201.15	  3
 21	 I 	  1087.17	   144.61	  2
 22	 R 	        -  	    88.06	  1  _


+3 charge
		      B   	     Y
  1	 A 	    24.69	        -  	 22
  2	 Q 	    67.37	   759.48	 21
  3	 E 	   110.39	   716.79	 20
  4	 P 	   142.74	   673.78	 19
  5	 V 	   175.76	   641.42	 18
  6	 K 	   218.46	   608.40	 17
  7	 G 	   237.47	   565.70	 16
  8	 P 	   269.82	   546.70	 15
  9	 V 	   302.84	   514.35	 14
 10	 S 	   331.85	   481.32	 13
 11	 T 	   365.53	   452.31	 12
 12	 K 	   408.23	   418.63	 11
 13	 P 	   440.58	   375.93	 10
 14	 G 	   459.59	   343.58	  9
 15	 S 	   488.60	   324.57	  8
 16	 C 	   541.99	   295.56	  7
 17	 P 	   574.34	   242.17	  6
 18	 I 	   612.03	   209.82	  5
 19	 I 	   649.73	   172.13	  4
 20	 L 	   687.42	   134.43	  3
 21	 I 	   725.12	    96.74	  2
 22	 R 	        -  	    59.04	  1  _
```

**85. IPI00409566 Isoform 1 of Polycystin-1**

| Sequence | Charge | M+H+ | XCorr | DeltaCn |
| --- | --- | --- | --- | --- |
| R.TLGPALRIPADATALDVSHNLLR.A | 3 | 2415.777 | 4.422 | 0.438 |

+1 charge

```
		      B   	     Y
  1	 T 	   102.05	        -  	 23
  2	 L 	   215.14	  2313.30	 22
  3	 G 	   272.16	  2200.22	 21
  4	 P 	   369.21	  2143.20	 20
  5	 A 	   440.25	  2046.15	 19
  6	 L 	   553.33	  1975.11	 18
  7	 R 	   709.44	  1862.02	 17
  8	 I 	   822.52	  1705.92	 16
  9	 P 	   919.57	  1592.84	 15
 10	 A 	   990.61	  1495.79	 14
 11	 D 	  1105.64	  1424.75	 13
 12	 A 	  1176.67	  1309.72	 12
 13	 T 	  1277.72	  1238.69	 11
 14	 A 	  1348.76	  1137.64	 10
 15	 L 	  1461.84	  1066.60	  9
 16	 D 	  1576.87	   953.52	  8
 17	 V 	  1675.94	   838.49	  7
 18	 S 	  1762.97	   739.42	  6
 19	 H 	  1900.03	   652.39	  5
 20	 N 	  2014.07	   515.33	  4
 21	 L 	  2127.16	   401.29	  3
 22	 L 	  2240.24	   288.20	  2
 23	 R 	        -  	   175.12	  1  _
+2 charge
		      B   	     Y
  1	 T 	    51.53	        -  	 23
  2	 L 	   108.07	  1157.16	 22
  3	 G 	   136.58	  1100.61	 21
  4	 P 	   185.11	  1072.10	 20
  5	 A 	   220.63	  1023.58	 19
  6	 L 	   277.17	   988.06	 18
  7	 R 	   355.22	   931.52	 17
  8	 I 	   411.76	   853.47	 16
  9	 P 	   460.29	   796.92	 15
 10	 A 	   495.81	   748.40	 14
 11	 D 	   553.32	   712.88	 13
 12	 A 	   588.84	   655.36	 12
 13	 T 	   639.36	   619.85	 11
 14	 A 	   674.88	   569.32	 10
 15	 L 	   731.42	   533.80	  9
 16	 D 	   788.94	   477.26	  8
 17	 V 	   838.47	   419.75	  7
 18	 S 	   881.99	   370.21	  6
 19	 H 	   950.52	   326.70	  5
 20	 N 	  1007.54	   258.17	  4
 21	 L 	  1064.08	   201.15	  3
 22	 L 	  1120.62	   144.61	  2
 23	 R 	        -  	    88.06	  1  _


+3 charge
		      B   	     Y
  1	 T 	    34.69	        -  	 23
  2	 L 	    72.38	   771.77	 22
  3	 G 	    91.39	   734.08	 21
  4	 P 	   123.74	   715.07	 20
  5	 A 	   147.42	   682.72	 19
  6	 L 	   185.12	   659.04	 18
  7	 R 	   237.15	   621.35	 17
  8	 I 	   274.84	   569.31	 16
  9	 P 	   307.20	   531.62	 15
 10	 A 	   330.87	   499.27	 14
 11	 D 	   369.22	   475.59	 13
 12	 A 	   392.90	   437.25	 12
 13	 T 	   426.58	   413.57	 11
 14	 A 	   450.26	   379.88	 10
 15	 L 	   487.95	   356.20	  9
 16	 D 	   526.29	   318.51	  8
 17	 V 	   559.32	   280.17	  7
 18	 S 	   588.33	   247.15	  6
 19	 H 	   634.01	   218.13	  5
 20	 N 	   672.03	   172.45	  4
 21	 L 	   709.72	   134.43	  3
 22	 L 	   747.42	    96.74	  2
 23	 R 	        -  	    59.04	  1  _
```

**86. IPI00167710 Isoform 1 of Fibulin-7**

| Sequence | Charge | M+H+ | XCorr | DeltaCn |
| --- | --- | --- | --- | --- |
| K.TISFHYLSLPSNLKTPITLFR.M | 3 | 2449.875 | 5.413 | 0.547 |

+1 charge

```
		      B   	     Y
  1	 T 	   102.05	        -  	 21
  2	 I 	   215.14	  2347.32	 20
  3	 S 	   302.17	  2234.23	 19
  4	 F 	   449.24	  2147.20	 18
  5	 H 	   586.30	  2000.13	 17
  6	 Y 	   749.36	  1863.07	 16
  7	 L 	   862.45	  1700.01	 15
  8	 S 	   949.48	  1586.93	 14
  9	 L 	  1062.56	  1499.89	 13
 10	 P 	  1159.61	  1386.81	 12
 11	 S 	  1246.65	  1289.76	 11
 12	 N 	  1360.69	  1202.73	 10
 13	 L 	  1473.77	  1088.68	  9
 14	 K 	  1601.87	   975.60	  8
 15	 T 	  1702.92	   847.50	  7
 16	 P 	  1799.97	   746.46	  6
 17	 I 	  1913.05	   649.40	  5
 18	 T 	  2014.10	   536.32	  4
 19	 L 	  2127.18	   435.27	  3
 20	 F 	  2274.25	   322.19	  2
 21	 R 	        -  	   175.12	  1  _
+2 charge
		      B   	     Y
  1	 T 	    51.53	        -  	 21
  2	 I 	   108.07	  1174.16	 20
  3	 S 	   151.59	  1117.62	 19
  4	 F 	   225.12	  1074.10	 18
  5	 H 	   293.65	  1000.57	 17
  6	 Y 	   375.18	   932.04	 16
  7	 L 	   431.73	   850.51	 15
  8	 S 	   475.24	   793.97	 14
  9	 L 	   531.78	   750.45	 13
 10	 P 	   580.31	   693.91	 12
 11	 S 	   623.83	   645.38	 11
 12	 N 	   680.85	   601.87	 10
 13	 L 	   737.39	   544.84	  9
 14	 K 	   801.44	   488.30	  8
 15	 T 	   851.96	   424.26	  7
 16	 P 	   900.49	   373.73	  6
 17	 I 	   957.03	   325.21	  5
 18	 T 	  1007.55	   268.66	  4
 19	 L 	  1064.10	   218.14	  3
 20	 F 	  1137.63	   161.60	  2
 21	 R 	        -  	    88.06	  1  _


+3 charge
		      B   	     Y
  1	 T 	    34.69	        -  	 21
  2	 I 	    72.38	   783.11	 20
  3	 S 	   101.40	   745.42	 19
  4	 F 	   150.42	   716.41	 18
  5	 H 	   196.10	   667.38	 17
  6	 Y 	   250.46	   621.70	 16
  7	 L 	   288.15	   567.34	 15
  8	 S 	   317.16	   529.65	 14
  9	 L 	   354.86	   500.64	 13
 10	 P 	   387.21	   462.94	 12
 11	 S 	   416.22	   430.59	 11
 12	 N 	   454.23	   401.58	 10
 13	 L 	   491.93	   363.57	  9
 14	 K 	   534.63	   325.87	  8
 15	 T 	   568.31	   283.17	  7
 16	 P 	   600.66	   249.49	  6
 17	 I 	   638.36	   217.14	  5
 18	 T 	   672.04	   179.44	  4
 19	 L 	   709.73	   145.76	  3
 20	 F 	   758.76	   108.07	  2
 21	 R 	        -  	    59.04	  1  _
```

**87. IPI00004656 Beta-2-microglobulin**

| Sequence | Charge | M+H+ | XCorr | DeltaCn |
| --- | --- | --- | --- | --- |
| K.SNFLNCYVSGFHPSDIEVDLLK.N | 3 | 2555.817 | 3.59 | 0.555 |

+1 charge

```
		      B   	     Y
  1	 S 	    88.04	        -  	 22
  2	 N 	   202.08	  2467.33	 21
  3	 F 	   349.15	  2353.29	 20
  4	 L 	   462.23	  2206.22	 19
  5	 N 	   576.28	  2093.14	 18
  6	 C 	   736.44	  1979.09	 17
  7	 Y 	   899.51	  1818.93	 16
  8	 V 	   998.57	  1655.86	 15
  9	 S 	  1085.61	  1556.80	 14
 10	 G 	  1142.63	  1469.76	 13
 11	 F 	  1289.70	  1412.74	 12
 12	 H 	  1426.76	  1265.67	 11
 13	 P 	  1523.81	  1128.61	 10
 14	 S 	  1610.84	  1031.56	  9
 15	 D 	  1725.87	   944.53	  8
 16	 I 	  1838.95	   829.50	  7
 17	 E 	  1967.99	   716.42	  6
 18	 V 	  2067.06	   587.38	  5
 19	 D 	  2182.09	   488.31	  4
 20	 L 	  2295.17	   373.28	  3
 21	 L 	  2408.26	   260.20	  2
 22	 K 	        -  	   147.11	  1  _
+2 charge
		      B   	     Y
  1	 S 	    44.52	        -  	 22
  2	 N 	   101.54	  1234.17	 21
  3	 F 	   175.08	  1177.15	 20
  4	 L 	   231.62	  1103.61	 19
  5	 N 	   288.64	  1047.07	 18
  6	 C 	   368.73	   990.05	 17
  7	 Y 	   450.26	   909.97	 16
  8	 V 	   499.79	   828.44	 15
  9	 S 	   543.31	   778.90	 14
 10	 G 	   571.82	   735.39	 13
 11	 F 	   645.35	   706.87	 12
 12	 H 	   713.88	   633.34	 11
 13	 P 	   762.41	   564.81	 10
 14	 S 	   805.92	   516.28	  9
 15	 D 	   863.44	   472.77	  8
 16	 I 	   919.98	   415.26	  7
 17	 E 	   984.50	   358.71	  6
 18	 V 	  1034.03	   294.19	  5
 19	 D 	  1091.55	   244.66	  4
 20	 L 	  1148.09	   187.14	  3
 21	 L 	  1204.63	   130.60	  2
 22	 K 	        -  	    74.06	  1  _
+3 charge
		      B   	     Y
  1	 S 	    30.02	        -  	 22
  2	 N 	    68.03	   823.12	 21
  3	 F 	   117.06	   785.10	 20
  4	 L 	   154.75	   736.08	 19
  5	 N 	   192.76	   698.38	 18
  6	 C 	   246.15	   660.37	 17
  7	 Y 	   300.51	   606.98	 16
  8	 V 	   333.53	   552.63	 15
  9	 S 	   362.54	   519.60	 14
 10	 G 	   381.55	   490.59	 13
 11	 F 	   430.57	   471.59	 12
 12	 H 	   476.26	   422.56	 11
 13	 P 	   508.61	   376.88	 10
 14	 S 	   537.62	   344.53	  9
 15	 D 	   575.96	   315.51	  8
 16	 I 	   613.66	   277.17	  7
 17	 E 	   656.67	   239.48	  6
 18	 V 	   689.69	   196.46	  5
 19	 D 	   728.03	   163.44	  4
 20	 L 	   765.73	   125.10	  3
 21	 L 	   803.42	    87.40	  2
 22	 K 	        -  	    49.71	  1  _
```

**88. IPI00021885 Isoform 1 of Fibrinogen alpha chain**

| Sequence | Charge | M+H+ | XCorr | DeltaCn |
| --- | --- | --- | --- | --- |
| R.GFGSLNDEGEGEFWLGNDYLHLLTQR.G | 3 | 2969.17 | 5.312 | 0.645 |

+1 charge

```
		      B   	     Y
  1	 G 	    58.03	        -  	 26
  2	 F 	   205.10	  2910.37	 25
  3	 G 	   262.12	  2763.30	 24
  4	 S 	   349.15	  2706.28	 23
  5	 L 	   462.23	  2619.25	 22
  6	 N 	   576.28	  2506.16	 21
  7	 D 	   691.30	  2392.12	 20
  8	 E 	   820.35	  2277.09	 19
  9	 G 	   877.37	  2148.05	 18
 10	 E 	  1006.41	  2091.03	 17
 11	 G 	  1063.43	  1961.99	 16
 12	 E 	  1192.48	  1904.97	 15
 13	 F 	  1339.54	  1775.92	 14
 14	 W 	  1525.62	  1628.85	 13
 15	 L 	  1638.71	  1442.78	 12
 16	 G 	  1695.73	  1329.69	 11
 17	 N 	  1809.77	  1272.67	 10
 18	 D 	  1924.80	  1158.63	  9
 19	 Y 	  2087.86	  1043.60	  8
 20	 L 	  2200.95	   880.54	  7
 21	 H 	  2338.00	   767.45	  6
 22	 L 	  2451.09	   630.39	  5
 23	 L 	  2564.17	   517.31	  4
 24	 T 	  2665.22	   404.23	  3
 25	 Q 	  2793.28	   303.18	  2
 26	 R 	        -  	   175.12	  1  _


+2 charge
		      B   	     Y
  1	 G 	    29.52	        -  	 26
  2	 F 	   103.05	  1455.69	 25
  3	 G 	   131.56	  1382.15	 24
  4	 S 	   175.08	  1353.64	 23
  5	 L 	   231.62	  1310.13	 22
  6	 N 	   288.64	  1253.59	 21
  7	 D 	   346.16	  1196.56	 20
  8	 E 	   410.68	  1139.05	 19
  9	 G 	   439.19	  1074.53	 18
 10	 E 	   503.71	  1046.02	 17
 11	 G 	   532.22	   981.50	 16
 12	 E 	   596.74	   952.99	 15
 13	 F 	   670.28	   888.47	 14
 14	 W 	   763.32	   814.93	 13
 15	 L 	   819.86	   721.89	 12
 16	 G 	   848.37	   665.35	 11
 17	 N 	   905.39	   636.84	 10
 18	 D 	   962.90	   579.82	  9
 19	 Y 	  1044.43	   522.30	  8
 20	 L 	  1100.98	   440.77	  7
 21	 H 	  1169.51	   384.23	  6
 22	 L 	  1226.05	   315.70	  5
 23	 L 	  1282.59	   259.16	  4
 24	 T 	  1333.11	   202.62	  3
 25	 Q 	  1397.14	   152.09	  2
 26	 R 	        -  	    88.06	  1  _


+3 charge
		      B   	     Y
  1	 G 	    20.01	        -  	 26
  2	 F 	    69.04	   970.79	 25
  3	 G 	    88.04	   921.77	 24
  4	 S 	   117.06	   902.76	 23
  5	 L 	   154.75	   873.75	 22
  6	 N 	   192.76	   836.06	 21
  7	 D 	   231.11	   798.04	 20
  8	 E 	   274.12	   759.70	 19
  9	 G 	   293.13	   716.69	 18
 10	 E 	   336.14	   697.68	 17
 11	 G 	   355.15	   654.67	 16
 12	 E 	   398.16	   635.66	 15
 13	 F 	   447.19	   592.65	 14
 14	 W 	   509.21	   543.62	 13
 15	 L 	   546.91	   481.60	 12
 16	 G 	   565.91	   443.90	 11
 17	 N 	   603.93	   424.89	 10
 18	 D 	   642.27	   386.88	  9
 19	 Y 	   696.63	   348.54	  8
 20	 L 	   734.32	   294.18	  7
 21	 H 	   780.01	   256.49	  6
 22	 L 	   817.70	   210.80	  5
 23	 L 	   855.40	   173.11	  4
 24	 T 	   889.08	   135.41	  3
 25	 Q 	   931.76	   101.73	  2
 26	 R 	        -  	    59.04	  1  _
```

**89. IPI00296713 Isoform 1 of Granulins**

| Sequence | Charge | M+H+ | XCorr | DeltaCn |
| --- | --- | --- | --- | --- |
| K.CDMEVSCPDGYTCCR.L | 2 | 1911.012 | 5.128 | 0.673 |

+1 charge

```
		      B   	     Y
  1	 C 	   161.17	        -  	 15
  2	 D 	   276.20	  1750.04	 14
  3	 M 	   407.24	  1635.01	 13
  4	 E 	   536.28	  1503.97	 12
  5	 V 	   635.35	  1374.93	 11
  6	 S 	   722.38	  1275.86	 10
  7	 C 	   882.55	  1188.83	  9
  8	 P 	   979.60	  1028.66	  8
  9	 D 	  1094.63	   931.61	  7
 10	 G 	  1151.65	   816.58	  6
 11	 Y 	  1314.71	   759.56	  5
 12	 T 	  1415.76	   596.50	  4
 13	 C 	  1575.93	   495.45	  3
 14	 C 	  1736.09	   335.28	  2
 15	 R 	        -  	   175.12	  1  _
+2 charge
		      B   	     Y
  1	 C 	    81.09	        -  	 15
  2	 D 	   138.60	   875.52	 14
  3	 M 	   204.12	   818.01	 13
  4	 E 	   268.64	   752.49	 12
  5	 V 	   318.18	   687.97	 11
  6	 S 	   361.70	   638.43	 10
  7	 C 	   441.78	   594.92	  9
  8	 P 	   490.30	   514.83	  8
  9	 D 	   547.82	   466.31	  7
 10	 G 	   576.33	   408.79	  6
 11	 Y 	   657.86	   380.28	  5
 12	 T 	   708.38	   298.75	  4
 13	 C 	   788.47	   248.23	  3
 14	 C 	   868.55	   168.15	  2
 15	 R 	        -  	    88.06	  1  _
```

**90. IPI00386839 Amyloid lambda 6 light chain variable region SAR (Fragment)**

| Sequence | Charge | M+H+ | XCorr | DeltaCn |
| --- | --- | --- | --- | --- |
| - .NFMLTQPHSVSESPGK.T | 2 | 1759.965 | 3.493 | 0.496 |

+1 charge

```
		      B   	     Y
  1	 N 	   115.05	        -  	 16
  2	 F 	   262.12	  1644.81	 15
  3	 M 	   393.16	  1497.74	 14
  4	 L 	   506.24	  1366.70	 13
  5	 T 	   607.29	  1253.61	 12
  6	 Q 	   735.35	  1152.56	 11
  7	 P 	   832.40	  1024.51	 10
  8	 H 	   969.46	   927.45	  9
  9	 S 	  1056.49	   790.39	  8
 10	 V 	  1155.56	   703.36	  7
 11	 S 	  1242.59	   604.29	  6
 12	 E 	  1371.64	   517.26	  5
 13	 S 	  1458.67	   388.22	  4
 14	 P 	  1555.72	   301.19	  3
 15	 G 	  1612.74	   204.13	  2
 16	 K 	        -  	   147.11	  1  _
+2 charge
		      B   	     Y
  1	 N 	    58.03	        -  	 16
  2	 F 	   131.56	   822.91	 15
  3	 M 	   197.08	   749.37	 14
  4	 L 	   253.63	   683.85	 13
  5	 T 	   304.15	   627.31	 12
  6	 Q 	   368.18	   576.79	 11
  7	 P 	   416.70	   512.76	 10
  8	 H 	   485.23	   464.23	  9
  9	 S 	   528.75	   395.70	  8
 10	 V 	   578.28	   352.18	  7
 11	 S 	   621.80	   302.65	  6
 12	 E 	   686.32	   259.13	  5
 13	 S 	   729.84	   194.61	  4
 14	 P 	   778.36	   151.10	  3
 15	 G 	   806.87	   102.57	  2
 16	 K 	        -  	    74.06	  1  _
```

**91. IPI00298388 Isoform 1 of Phosphoinositide-3-kinase-interacting protein 1**

| Sequence | Charge | M+H+ | XCorr | DeltaCn |
| --- | --- | --- | --- | --- |
| R.SEAAAVQPVIGISQR.V | 2 | 1526.72 | 4.603 | 0.553 |

+1 charge

```
		      B   	     Y
  1	 S 	    88.04	        -  	 15
  2	 E 	   217.08	  1438.80	 14
  3	 A 	   288.12	  1309.76	 13
  4	 A 	   359.16	  1238.72	 12
  5	 A 	   430.19	  1167.68	 11
  6	 V 	   529.26	  1096.65	 10
  7	 Q 	   657.32	   997.58	  9
  8	 P 	   754.37	   869.52	  8
  9	 V 	   853.44	   772.47	  7
 10	 I 	   966.53	   673.40	  6
 11	 G 	  1023.55	   560.32	  5
 12	 I 	  1136.63	   503.29	  4
 13	 S 	  1223.66	   390.21	  3
 14	 Q 	  1351.72	   303.18	  2
 15	 R 	        -  	   175.12	  1  _
+2 charge
		      B   	     Y
  1	 S 	    44.52	        -  	 15
  2	 E 	   109.04	   719.90	 14
  3	 A 	   144.56	   655.38	 13
  4	 A 	   180.08	   619.86	 12
  5	 A 	   215.60	   584.35	 11
  6	 V 	   265.13	   548.83	 10
  7	 Q 	   329.16	   499.29	  9
  8	 P 	   377.69	   435.26	  8
  9	 V 	   427.22	   386.74	  7
 10	 I 	   483.77	   337.20	  6
 11	 G 	   512.28	   280.66	  5
 12	 I 	   568.82	   252.15	  4
 13	 S 	   612.34	   195.61	  3
 14	 Q 	   676.36	   152.09	  2
 15	 R 	        -  	    88.06	  1  _
```

**92. IPI00022974 Prolactin-inducible protein**

| Sequence | Charge | M+H+ | XCorr | DeltaCn |
| --- | --- | --- | --- | --- |
| R.KIIIKNFDIPK.S | 2 | 1329.656 | 3.23 | 0.29 |

+1 charge

```
		      B   	     Y
  1	 K 	   129.10	        -  	 11
  2	 I 	   242.19	  1200.74	 10
  3	 I 	   355.27	  1087.65	  9
  4	 I 	   468.35	   974.57	  8
  5	 K 	   596.45	   861.48	  7
  6	 N 	   710.49	   733.39	  6
  7	 F 	   857.56	   619.34	  5
  8	 D 	   972.59	   472.28	  4
  9	 I 	  1085.67	   357.25	  3
 10	 P 	  1182.72	   244.17	  2
 11	 K 	        -  	   147.11	  1  _
+2 charge
		      B   	     Y
  1	 K 	    65.05	        -  	 11
  2	 I 	   121.60	   600.87	 10
  3	 I 	   178.14	   544.33	  9
  4	 I 	   234.68	   487.79	  8
  5	 K 	   298.73	   431.25	  7
  6	 N 	   355.75	   367.20	  6
  7	 F 	   429.28	   310.18	  5
  8	 D 	   486.80	   236.64	  4
  9	 I 	   543.34	   179.13	  3
 10	 P 	   591.87	   122.59	  2
 11	 K 	        -  	    74.06	  1  _
```

**93. IPI00414717 Isoform 2 of Golgi apparatus protein 1**

| Sequence | Charge | M+H+ | XCorr | DeltaCn |
| --- | --- | --- | --- | --- |
| K.AKDDSELEGQVISCLK.L | 2 | 1792.96 | 2.994 | 0.478 |

**+1 charge**

```
		      B   	     Y
  1	 A 	    72.04	        -  	 16
  2	 K 	   200.14	  1720.98	 15
  3	 D 	   315.17	  1592.88	 14
  4	 D 	   430.19	  1477.85	 13
  5	 S 	   517.23	  1362.83	 12
  6	 E 	   646.27	  1275.80	 11
  7	 L 	   759.35	  1146.75	 10
  8	 E 	   888.39	  1033.67	  9
  9	 G 	   945.42	   904.63	  8
 10	 Q 	  1073.47	   847.61	  7
 11	 V 	  1172.54	   719.55	  6
 12	 I 	  1285.63	   620.48	  5
 13	 S 	  1372.66	   507.39	  4
 14	 C 	  1532.82	   420.36	  3
 15	 L 	  1645.91	   260.20	  2
 16	 K 	        -  	   147.11	  1  _
+2 charge
		      B   	     Y
  1	 A 	    36.53	        -  	 16
  2	 K 	   100.57	   860.99	 15
  3	 D 	   158.09	   796.94	 14
  4	 D 	   215.60	   739.43	 13
  5	 S 	   259.12	   681.92	 12
  6	 E 	   323.64	   638.40	 11
  7	 L 	   380.18	   573.88	 10
  8	 E 	   444.70	   517.34	  9
  9	 G 	   473.21	   452.82	  8
 10	 Q 	   537.24	   424.31	  7
 11	 V 	   586.78	   360.28	  6
 12	 I 	   643.32	   310.74	  5
 13	 S 	   686.83	   254.20	  4
 14	 C 	   766.92	   210.68	  3
 15	 L 	   823.46	   130.60	  2
 16	 K 	        -  	    74.06	  1  _
```

**94. IPI00178140 Isoform 1 of Hyaluronidase-1**

| Sequence | Charge | M+H+ | XCorr | DeltaCn |
| --- | --- | --- | --- | --- |
| R.AWMAGTLQLGR.A | 2 | 1204.427 | 3.357 | 0.387 |

+1 charge

```
		      B   	     Y
  1	 A 	    72.04	        -  	 11
  2	 W 	   258.12	  1132.59	 10
  3	 M 	   389.16	   946.51	  9
  4	 A 	   460.20	   815.47	  8
  5	 G 	   517.22	   744.44	  7
  6	 T 	   618.27	   687.41	  6
  7	 L 	   731.35	   586.37	  5
  8	 Q 	   859.41	   473.28	  4
  9	 L 	   972.50	   345.22	  3
 10	 G 	  1029.52	   232.14	  2
 11	 R 	        -  	   175.12	  1  _
+2 charge
		      B   	     Y
  1	 A 	    36.53	        -  	 11
  2	 W 	   129.57	   566.80	 10
  3	 M 	   195.09	   473.76	  9
  4	 A 	   230.60	   408.24	  8
  5	 G 	   259.12	   372.72	  7
  6	 T 	   309.64	   344.21	  6
  7	 L 	   366.18	   293.69	  5
  8	 Q 	   430.21	   237.15	  4
  9	 L 	   486.75	   173.12	  3
 10	 G 	   515.26	   116.57	  2
 11	 R 	        -  	    88.06	  1  _
```

**95. IPI00015199 T-cell antigen CD7**

| Sequence | Charge | M+H+ | XCorr | DeltaCn |
| --- | --- | --- | --- | --- |
| R.QLGPQPQDIIYYEDGVVPTTDRR.F | 3 | 2661.908 | 3.383 | 0.444 |

+1 charge

```
		      B   	     Y
  1	 Q 	   129.07	        -  	 23
  2	 L 	   242.15	  2532.27	 22
  3	 G 	   299.17	  2419.19	 21
  4	 P 	   396.22	  2362.17	 20
  5	 Q 	   524.28	  2265.11	 19
  6	 P 	   621.34	  2137.06	 18
  7	 Q 	   749.39	  2040.00	 17
  8	 D 	   864.42	  1911.94	 16
  9	 I 	   977.51	  1796.92	 15
 10	 I 	  1090.59	  1683.83	 14
 11	 Y 	  1253.65	  1570.75	 13
 12	 Y 	  1416.72	  1407.69	 12
 13	 E 	  1545.76	  1244.62	 11
 14	 D 	  1660.79	  1115.58	 10
 15	 G 	  1717.81	  1000.55	  9
 16	 V 	  1816.88	   943.53	  8
 17	 V 	  1915.94	   844.46	  7
 18	 P 	  2013.00	   745.40	  6
 19	 T 	  2114.04	   648.34	  5
 20	 T 	  2215.09	   547.29	  4
 21	 D 	  2330.12	   446.25	  3
 22	 R 	  2486.22	   331.22	  2
 23	 R 	        -  	   175.12	  1  _
+2 charge
		      B   	     Y
  1	 Q 	    65.04	        -  	 23
  2	 L 	   121.58	  1266.64	 22
  3	 G 	   150.09	  1210.10	 21
  4	 P 	   198.62	  1181.59	 20
  5	 Q 	   262.64	  1133.06	 19
  6	 P 	   311.17	  1069.03	 18
  7	 Q 	   375.20	  1020.51	 17
  8	 D 	   432.71	   956.48	 16
  9	 I 	   489.26	   898.96	 15
 10	 I 	   545.80	   842.42	 14
 11	 Y 	   627.33	   785.88	 13
 12	 Y 	   708.86	   704.35	 12
 13	 E 	   773.38	   622.82	 11
 14	 D 	   830.90	   558.29	 10
 15	 G 	   859.41	   500.78	  9
 16	 V 	   908.94	   472.27	  8
 17	 V 	   958.48	   422.74	  7
 18	 P 	  1007.00	   373.20	  6
 19	 T 	  1057.53	   324.67	  5
 20	 T 	  1108.05	   274.15	  4
 21	 D 	  1165.56	   223.63	  3
 22	 R 	  1243.61	   166.11	  2
 23	 R 	        -  	    88.06	  1  _


+3 charge
		      B   	     Y
  1	 Q 	    43.69	        -  	 23
  2	 L 	    81.39	   844.76	 22
  3	 G 	   100.40	   807.07	 21
  4	 P 	   132.75	   788.06	 20
  5	 Q 	   175.43	   755.71	 19
  6	 P 	   207.78	   713.02	 18
  7	 Q 	   250.47	   680.67	 17
  8	 D 	   288.81	   637.99	 16
  9	 I 	   326.51	   599.64	 15
 10	 I 	   364.20	   561.95	 14
 11	 Y 	   418.56	   524.25	 13
 12	 Y 	   472.91	   469.90	 12
 13	 E 	   515.92	   415.55	 11
 14	 D 	   554.27	   372.53	 10
 15	 G 	   573.27	   334.19	  9
 16	 V 	   606.30	   315.18	  8
 17	 V 	   639.32	   282.16	  7
 18	 P 	   671.67	   249.14	  6
 19	 T 	   705.35	   216.79	  5
 20	 T 	   739.04	   183.10	  4
 21	 D 	   777.38	   149.42	  3
 22	 R 	   829.41	   111.08	  2
 23	 R 	        -  	    59.04	  1  _
```

**96. IPI00025861 Cadherin-1**

| Sequence | Charge | M+H+ | XCorr | DeltaCn |
| --- | --- | --- | --- | --- |
| K.MALEVGDYKINLK.L | 2 | 1494.781 | 3.819 | 0.47 |

+1 charge

```
		      B   	     Y
  1	 M 	   132.05	        -  	 13
  2	 A 	   203.08	  1362.76	 12
  3	 L 	   316.17	  1291.73	 11
  4	 E 	   445.21	  1178.64	 10
  5	 V 	   544.28	  1049.60	  9
  6	 G 	   601.30	   950.53	  8
  7	 D 	   716.33	   893.51	  7
  8	 Y 	   879.39	   778.48	  6
  9	 K 	  1007.49	   615.42	  5
 10	 I 	  1120.57	   487.32	  4
 11	 N 	  1234.61	   374.24	  3
 12	 L 	  1347.70	   260.20	  2
 13	 K 	        -  	   147.11	  1  _
+2 charge
		      B   	     Y
  1	 M 	    66.53	        -  	 13
  2	 A 	   102.05	   681.89	 12
  3	 L 	   158.59	   646.37	 11
  4	 E 	   223.11	   589.82	 10
  5	 V 	   272.64	   525.30	  9
  6	 G 	   301.15	   475.77	  8
  7	 D 	   358.67	   447.26	  7
  8	 Y 	   440.20	   389.74	  6
  9	 K 	   504.25	   308.21	  5
 10	 I 	   560.79	   244.17	  4
 11	 N 	   617.81	   187.62	  3
 12	 L 	   674.35	   130.60	  2
 13	 K 	        -  	    74.06	  1  _
```

**97. IPI00297420 Frizzled-4 precursor**

| Sequence | Charge | M+H+ | XCorr | DeltaCn |
| --- | --- | --- | --- | --- |
| K.INIPIGPCGGMCLSVK.R | 2 | 1717.054 | 2.906 | 0.304 |

+1 charge

```
		      B   	     Y
  1	 I 	   114.09	        -  	 16
  2	 N 	   228.13	  1603.05	 15
  3	 I 	   341.22	  1489.01	 14
  4	 P 	   438.27	  1375.92	 13
  5	 I 	   551.36	  1278.87	 12
  6	 G 	   608.38	  1165.79	 11
  7	 P 	   705.43	  1108.76	 10
  8	 C 	   865.59	  1011.71	  9
  9	 G 	   922.62	   851.55	  8
 10	 G 	   979.64	   794.52	  7
 11	 M 	  1110.68	   737.50	  6
 12	 C 	  1270.84	   606.46	  5
 13	 L 	  1383.93	   446.30	  4
 14	 S 	  1470.96	   333.21	  3
 15	 V 	  1570.03	   246.18	  2
 16	 K 	        -  	   147.11	  1  _
+2 charge
		      B   	     Y
  1	 I 	    57.55	        -  	 16
  2	 N 	   114.57	   802.03	 15
  3	 I 	   171.11	   745.01	 14
  4	 P 	   219.64	   688.46	 13
  5	 I 	   276.18	   639.94	 12
  6	 G 	   304.69	   583.40	 11
  7	 P 	   353.22	   554.89	 10
  8	 C 	   433.30	   506.36	  9
  9	 G 	   461.81	   426.28	  8
 10	 G 	   490.32	   397.77	  7
 11	 M 	   555.84	   369.26	  6
 12	 C 	   635.93	   303.73	  5
 13	 L 	   692.47	   223.65	  4
 14	 S 	   735.98	   167.11	  3
 15	 V 	   785.52	   123.59	  2
 16	 K 	        -  	    74.06	  1  _
```

**98. IPI00022620 Secreted Ly-6/uPAR-related protein 1**

| Sequence | Charge | M+H+ | XCorr | DeltaCn |
| --- | --- | --- | --- | --- |
| R.SCSSSCVATDPDSIGAAHLIFCCFR.D | 3 | 2820.036 | 6.083 | 0.65 |

+1 charge

```
		      B   	     Y
  1	 S 	    88.04	        -  	 25
  2	 C 	   248.20	  2731.71	 24
  3	 S 	   335.24	  2571.55	 23
  4	 S 	   422.27	  2484.51	 22
  5	 S 	   509.30	  2397.48	 21
  6	 C 	   669.47	  2310.45	 20
  7	 V 	   768.53	  2150.28	 19
  8	 A 	   839.57	  2051.22	 18
  9	 T 	   940.62	  1980.18	 17
 10	 D 	  1055.65	  1879.13	 16
 11	 P 	  1152.70	  1764.10	 15
 12	 D 	  1267.73	  1667.05	 14
 13	 S 	  1354.76	  1552.03	 13
 14	 I 	  1467.84	  1464.99	 12
 15	 G 	  1524.86	  1351.91	 11
 16	 A 	  1595.90	  1294.89	 10
 17	 A 	  1666.94	  1223.85	  9
 18	 H 	  1804.00	  1152.81	  8
 19	 L 	  1917.08	  1015.75	  7
 20	 I 	  2030.16	   902.67	  6
 21	 F 	  2177.23	   789.59	  5
 22	 C 	  2337.40	   642.52	  4
 23	 C 	  2497.56	   482.35	  3
 24	 F 	  2644.63	   322.19	  2
 25	 R 	        -  	   175.12	  1  _


+2 charge
		      B   	     Y
  1	 S 	    44.52	        -  	 25
  2	 C 	   124.61	  1366.36	 24
  3	 S 	   168.12	  1286.28	 23
  4	 S 	   211.64	  1242.76	 22
  5	 S 	   255.15	  1199.24	 21
  6	 C 	   335.24	  1155.73	 20
  7	 V 	   384.77	  1075.65	 19
  8	 A 	   420.29	  1026.11	 18
  9	 T 	   470.81	   990.59	 17
 10	 D 	   528.33	   940.07	 16
 11	 P 	   576.85	   882.56	 15
 12	 D 	   634.37	   834.03	 14
 13	 S 	   677.88	   776.52	 13
 14	 I 	   734.42	   733.00	 12
 15	 G 	   762.94	   676.46	 11
 16	 A 	   798.45	   647.95	 10
 17	 A 	   833.97	   612.43	  9
 18	 H 	   902.50	   576.91	  8
 19	 L 	   959.04	   508.38	  7
 20	 I 	  1015.59	   451.84	  6
 21	 F 	  1089.12	   395.30	  5
 22	 C 	  1169.20	   321.76	  4
 23	 C 	  1249.29	   241.68	  3
 24	 F 	  1322.82	   161.60	  2
 25	 R 	        -  	    88.06	  1  _


+3 charge
		      B   	     Y
  1	 S 	    30.02	        -  	 25
  2	 C 	    83.41	   911.24	 24
  3	 S 	   112.42	   857.85	 23
  4	 S 	   141.43	   828.84	 22
  5	 S 	   170.44	   799.83	 21
  6	 C 	   223.83	   770.82	 20
  7	 V 	   256.85	   717.43	 19
  8	 A 	   280.53	   684.41	 18
  9	 T 	   314.21	   660.73	 17
 10	 D 	   352.55	   627.05	 16
 11	 P 	   384.90	   588.71	 15
 12	 D 	   423.25	   556.36	 14
 13	 S 	   452.26	   518.01	 13
 14	 I 	   489.95	   489.00	 12
 15	 G 	   508.96	   451.31	 11
 16	 A 	   532.64	   432.30	 10
 17	 A 	   556.32	   408.62	  9
 18	 H 	   602.00	   384.94	  8
 19	 L 	   639.70	   339.26	  7
 20	 I 	   677.39	   301.56	  6
 21	 F 	   726.42	   263.87	  5
 22	 C 	   779.80	   214.84	  4
 23	 C 	   833.19	   161.46	  3
 24	 F 	   882.22	   108.07	  2
 25	 R 	        -  	    59.04	  1  _
```

**99. IPI00297263 Isoform 1 of Protein HEG homolog 1**

| Sequence | Charge | M+H+ | XCorr | DeltaCn |
| --- | --- | --- | --- | --- |
| R.ALSLAPLAGAGLELQLER.R | 2 | 1823.127 | 3.986 | 0.595 |

+1 charge

```
		      B   	     Y
  1	 A 	    72.04	        -  	 18
  2	 L 	   185.13	  1751.01	 17
  3	 S 	   272.16	  1637.92	 16
  4	 L 	   385.24	  1550.89	 15
  5	 A 	   456.28	  1437.81	 14
  6	 P 	   553.33	  1366.77	 13
  7	 L 	   666.42	  1269.72	 12
  8	 A 	   737.46	  1156.63	 11
  9	 G 	   794.48	  1085.59	 10
 10	 A 	   865.51	  1028.57	  9
 11	 G 	   922.54	   957.54	  8
 12	 L 	  1035.62	   900.51	  7
 13	 E 	  1164.66	   787.43	  6
 14	 L 	  1277.75	   658.39	  5
 15	 Q 	  1405.80	   545.30	  4
 16	 L 	  1518.89	   417.25	  3
 17	 E 	  1647.93	   304.16	  2
 18	 R 	        -  	   175.12	  1  _
+2 charge
		      B   	     Y
  1	 A 	    36.53	        -  	 18
  2	 L 	    93.07	   876.01	 17
  3	 S 	   136.58	   819.46	 16
  4	 L 	   193.13	   775.95	 15
  5	 A 	   228.64	   719.41	 14
  6	 P 	   277.17	   683.89	 13
  7	 L 	   333.71	   635.36	 12
  8	 A 	   369.23	   578.82	 11
  9	 G 	   397.74	   543.30	 10
 10	 A 	   433.26	   514.79	  9
 11	 G 	   461.77	   479.27	  8
 12	 L 	   518.31	   450.76	  7
 13	 E 	   582.83	   394.22	  6
 14	 L 	   639.38	   329.70	  5
 15	 Q 	   703.41	   273.16	  4
 16	 L 	   759.95	   209.13	  3
 17	 E 	   824.47	   152.58	  2
 18	 R 	        -  	    88.06	  1  _
```

**100. IPI00555812 Isoform 1 of Vitamin D-binding protein**

| Sequence | Charge | M+H+ | XCorr | DeltaCn |
| --- | --- | --- | --- | --- |
| K.LAQKVPTADLEDVLPLAEDITNILSK.C | 3 | 2808.217 | 5.412 | 0.619 |

+1 charge

```
		      B   	     Y
  1	 L 	   114.09	        -  	 26
  2	 A 	   185.13	  2693.46	 25
  3	 Q 	   313.19	  2622.42	 24
  4	 K 	   441.28	  2494.37	 23
  5	 V 	   540.35	  2366.27	 22
  6	 P 	   637.40	  2267.20	 21
  7	 T 	   738.45	  2170.15	 20
  8	 A 	   809.49	  2069.10	 19
  9	 D 	   924.51	  1998.06	 18
 10	 L 	  1037.60	  1883.04	 17
 11	 E 	  1166.64	  1769.95	 16
 12	 D 	  1281.67	  1640.91	 15
 13	 V 	  1380.74	  1525.88	 14
 14	 L 	  1493.82	  1426.82	 13
 15	 P 	  1590.87	  1313.73	 12
 16	 L 	  1703.96	  1216.68	 11
 17	 A 	  1774.99	  1103.59	 10
 18	 E 	  1904.04	  1032.56	  9
 19	 D 	  2019.06	   903.51	  8
 20	 I 	  2132.15	   788.49	  7
 21	 T 	  2233.20	   675.40	  6
 22	 N 	  2347.24	   574.36	  5
 23	 I 	  2460.32	   460.31	  4
 24	 L 	  2573.41	   347.23	  3
 25	 S 	  2660.44	   234.14	  2
 26	 K 	        -  	   147.11	  1  _


+2 charge
		      B   	     Y
  1	 L 	    57.55	        -  	 26
  2	 A 	    93.07	  1347.23	 25
  3	 Q 	   157.10	  1311.72	 24
  4	 K 	   221.14	  1247.69	 23
  5	 V 	   270.68	  1183.64	 22
  6	 P 	   319.21	  1134.10	 21
  7	 T 	   369.73	  1085.58	 20
  8	 A 	   405.25	  1035.05	 19
  9	 D 	   462.76	   999.54	 18
 10	 L 	   519.30	   942.02	 17
 11	 E 	   583.82	   885.48	 16
 12	 D 	   641.34	   820.96	 15
 13	 V 	   690.87	   763.45	 14
 14	 L 	   747.41	   713.91	 13
 15	 P 	   795.94	   657.37	 12
 16	 L 	   852.48	   608.84	 11
 17	 A 	   888.00	   552.30	 10
 18	 E 	   952.52	   516.78	  9
 19	 D 	  1010.04	   452.26	  8
 20	 I 	  1066.58	   394.75	  7
 21	 T 	  1117.10	   338.21	  6
 22	 N 	  1174.12	   287.68	  5
 23	 I 	  1230.67	   230.66	  4
 24	 L 	  1287.21	   174.12	  3
 25	 S 	  1330.72	   117.58	  2
 26	 K 	        -  	    74.06	  1  _


+3 charge
		      B   	     Y
  1	 L 	    38.70	        -  	 26
  2	 A 	    62.38	   898.49	 25
  3	 Q 	   105.07	   874.81	 24
  4	 K 	   147.77	   832.13	 23
  5	 V 	   180.79	   789.43	 22
  6	 P 	   213.14	   756.41	 21
  7	 T 	   246.82	   724.05	 20
  8	 A 	   270.50	   690.37	 19
  9	 D 	   308.84	   666.69	 18
 10	 L 	   346.54	   628.35	 17
 11	 E 	   389.55	   590.66	 16
 12	 D 	   427.89	   547.64	 15
 13	 V 	   460.92	   509.30	 14
 14	 L 	   498.61	   476.28	 13
 15	 P 	   530.96	   438.58	 12
 16	 L 	   568.66	   406.23	 11
 17	 A 	   592.34	   368.54	 10
 18	 E 	   635.35	   344.86	  9
 19	 D 	   673.69	   301.84	  8
 20	 I 	   711.39	   263.50	  7
 21	 T 	   745.07	   225.81	  6
 22	 N 	   783.08	   192.12	  5
 23	 I 	   820.78	   154.11	  4
 24	 L 	   858.47	   116.41	  3
 25	 S 	   887.48	    78.72	  2
 26	 K 	        -  	    49.71	  1  _
```

**101. IPI00304999 Tyrosine-protein kinase transmembrane receptor ROR2**

| Sequence | Charge | M+H+ | XCorr | DeltaCn |
| --- | --- | --- | --- | --- |
| R.WLKNDAPVVQEPR.R | 2 | 1552.759 | 3.119 | 0.229 |

+1 charge

```
		      B   	     Y
  1	 W 	   187.09	        -  	 13
  2	 L 	   300.17	  1365.75	 12
  3	 K 	   428.27	  1252.66	 11
  4	 N 	   542.31	  1124.57	 10
  5	 D 	   657.34	  1010.53	  9
  6	 A 	   728.37	   895.50	  8
  7	 P 	   825.43	   824.46	  7
  8	 V 	   924.49	   727.41	  6
  9	 V 	  1023.56	   628.34	  5
 10	 Q 	  1151.62	   529.27	  4
 11	 E 	  1280.66	   401.21	  3
 12	 P 	  1377.72	   272.17	  2
 13	 R 	        -  	   175.12	  1  _
+2 charge
		      B   	     Y
  1	 W 	    94.05	        -  	 13
  2	 L 	   150.59	   683.38	 12
  3	 K 	   214.64	   626.84	 11
  4	 N 	   271.66	   562.79	 10
  5	 D 	   329.17	   505.77	  9
  6	 A 	   364.69	   448.25	  8
  7	 P 	   413.22	   412.73	  7
  8	 V 	   462.75	   364.21	  6
  9	 V 	   512.28	   314.67	  5
 10	 Q 	   576.31	   265.14	  4
 11	 E 	   640.84	   201.11	  3
 12	 P 	   689.36	   136.59	  2
 13	 R 	        -  	    88.06	  1  _
```

**102. IPI00064262 Protocadherin-16**

| Sequence | Charge | M+H+ | XCorr | DeltaCn |
| --- | --- | --- | --- | --- |
| R.VADINDHAPAFPQAR.A | 2 | 1622.766 | 3.574 | 0.548 |

+1 charge

```
		      B   	     Y
  1	 V 	   100.08	        -  	 15
  2	 A 	   171.11	  1522.74	 14
  3	 D 	   286.14	  1451.70	 13
  4	 I 	   399.22	  1336.68	 12
  5	 N 	   513.27	  1223.59	 11
  6	 D 	   628.29	  1109.55	 10
  7	 H 	   765.35	   994.52	  9
  8	 A 	   836.39	   857.46	  8
  9	 P 	   933.44	   786.43	  7
 10	 A 	  1004.48	   689.37	  6
 11	 F 	  1151.55	   618.34	  5
 12	 P 	  1248.60	   471.27	  4
 13	 Q 	  1376.66	   374.21	  3
 14	 A 	  1447.70	   246.16	  2
 15	 R 	        -  	   175.12	  1  _
+2 charge
		      B   	     Y
  1	 V 	    50.54	        -  	 15
  2	 A 	    86.06	   761.87	 14
  3	 D 	   143.57	   726.35	 13
  4	 I 	   200.12	   668.84	 12
  5	 N 	   257.14	   612.30	 11
  6	 D 	   314.65	   555.28	 10
  7	 H 	   383.18	   497.76	  9
  8	 A 	   418.70	   429.24	  8
  9	 P 	   467.22	   393.72	  7
 10	 A 	   502.74	   345.19	  6
 11	 F 	   576.28	   309.67	  5
 12	 P 	   624.80	   236.14	  4
 13	 Q 	   688.83	   187.61	  3
 14	 A 	   724.35	   123.58	  2
 15	 R 	        -  	    88.06	  1  _
```

**103. IPI00002689 Isoform Alpha of Short transient receptor potential channel 4**

| Sequence | Charge | M+H+ | XCorr | DeltaCn |
| --- | --- | --- | --- | --- |
| K.IVAFVKYSALNPR.E | 2 | 1478.763 | 2.801 | 0.202 |

+1 charge

```
		      B   	     Y
  1	 I 	   114.09	        -  	 13
  2	 V 	   213.16	  1364.77	 12
  3	 A 	   284.20	  1265.70	 11
  4	 F 	   431.27	  1194.66	 10
  5	 V 	   530.33	  1047.59	  9
  6	 K 	   658.43	   948.53	  8
  7	 Y 	   821.49	   820.43	  7
  8	 S 	   908.52	   657.37	  6
  9	 A 	   979.56	   570.34	  5
 10	 L 	  1092.65	   499.30	  4
 11	 N 	  1206.69	   386.21	  3
 12	 P 	  1303.74	   272.17	  2
 13	 R 	        -  	   175.12	  1  _
+2 charge
		      B   	     Y
  1	 I 	    57.55	        -  	 13
  2	 V 	   107.08	   682.89	 12
  3	 A 	   142.60	   633.35	 11
  4	 F 	   216.14	   597.84	 10
  5	 V 	   265.67	   524.30	  9
  6	 K 	   329.72	   474.77	  8
  7	 Y 	   411.25	   410.72	  7
  8	 S 	   454.77	   329.19	  6
  9	 A 	   490.28	   285.67	  5
 10	 L 	   546.83	   250.15	  4
 11	 N 	   603.85	   193.61	  3
 12	 P 	   652.37	   136.59	  2
 13	 R 	        -  	    88.06	  1  _
```

**104. IPI00382478 Ig heavy chain V-III region TIL**

| Sequence | Charge | M+H+ | XCorr | DeltaCn |
| --- | --- | --- | --- | --- |
| - .EVQLLESGGGLVQPGGSLR.L | 2 | 1897.122 | 3.766 | 0.405 |

+1 charge

```
		      B   	     Y
  1	 E 	   130.05	        -  	 19
  2	 V 	   229.12	  1766.98	 18
  3	 Q 	   357.18	  1667.91	 17
  4	 L 	   470.26	  1539.85	 16
  5	 L 	   583.34	  1426.76	 15
  6	 E 	   712.39	  1313.68	 14
  7	 S 	   799.42	  1184.64	 13
  8	 G 	   856.44	  1097.61	 12
  9	 G 	   913.46	  1040.58	 11
 10	 G 	   970.48	   983.56	 10
 11	 L 	  1083.57	   926.54	  9
 12	 V 	  1182.64	   813.46	  8
 13	 Q 	  1310.70	   714.39	  7
 14	 P 	  1407.75	   586.33	  6
 15	 G 	  1464.77	   489.28	  5
 16	 G 	  1521.79	   432.26	  4
 17	 S 	  1608.82	   375.24	  3
 18	 L 	  1721.91	   288.20	  2
 19	 R 	        -  	   175.12	  1  _
+2 charge
		      B   	     Y
  1	 E 	    65.53	        -  	 19
  2	 V 	   115.06	   883.99	 18
  3	 Q 	   179.09	   834.46	 17
  4	 L 	   235.63	   770.43	 16
  5	 L 	   292.18	   713.89	 15
  6	 E 	   356.70	   657.34	 14
  7	 S 	   400.21	   592.82	 13
  8	 G 	   428.72	   549.31	 12
  9	 G 	   457.23	   520.80	 11
 10	 G 	   485.75	   492.29	 10
 11	 L 	   542.29	   463.77	  9
 12	 V 	   591.82	   407.23	  8
 13	 Q 	   655.85	   357.70	  7
 14	 P 	   704.38	   293.67	  6
 15	 G 	   732.89	   245.14	  5
 16	 G 	   761.40	   216.63	  4
 17	 S 	   804.92	   188.12	  3
 18	 L 	   861.46	   144.61	  2
 19	 R 	        -  	    88.06	  1  _
```

**105. IPI00019359 Keratin, type I cytoskeletal 9**

| Sequence | Charge | M+H+ | XCorr | DeltaCn |
| --- | --- | --- | --- | --- |
| K.TLNDMRQEYEQLIAK.N | 2 | 1853.09 | 2.824 | 0.308 |

+1 charge

```
		      B   	     Y
  1	 T 	   102.05	        -  	 15
  2	 L 	   215.14	  1750.88	 14
  3	 N 	   329.18	  1637.80	 13
  4	 D 	   444.21	  1523.75	 12
  5	 M 	   575.25	  1408.73	 11
  6	 R 	   731.35	  1277.68	 10
  7	 Q 	   859.41	  1121.58	  9
  8	 E 	   988.45	   993.53	  8
  9	 Y 	  1151.51	   864.48	  7
 10	 E 	  1280.56	   701.42	  6
 11	 Q 	  1408.62	   572.38	  5
 12	 L 	  1521.70	   444.32	  4
 13	 I 	  1634.78	   331.23	  3
 14	 A 	  1705.82	   218.15	  2
 15	 K 	        -  	   147.11	  1  _
+2 charge
		      B   	     Y
  1	 T 	    51.53	        -  	 15
  2	 L 	   108.07	   875.94	 14
  3	 N 	   165.09	   819.40	 13
  4	 D 	   222.61	   762.38	 12
  5	 M 	   288.13	   704.87	 11
  6	 R 	   366.18	   639.35	 10
  7	 Q 	   430.21	   561.30	  9
  8	 E 	   494.73	   497.27	  8
  9	 Y 	   576.26	   432.74	  7
 10	 E 	   640.78	   351.21	  6
 11	 Q 	   704.81	   286.69	  5
 12	 L 	   761.35	   222.66	  4
 13	 I 	   817.90	   166.12	  3
 14	 A 	   853.41	   109.58	  2
 15	 K 	        -  	    74.06	  1  _
```

**106. IPI00453473 Histone H4**

| Sequence | Charge | M+H+ | XCorr | DeltaCn |
| --- | --- | --- | --- | --- |
| K.TVTAMDVVYALKR.Q | 2 | 1467.759 | 3.017 | 0.434 |

+1 charge

```
		      B   	     Y
  1	 T 	   102.05	        -  	 13
  2	 V 	   201.12	  1365.76	 12
  3	 T 	   302.17	  1266.69	 11
  4	 A 	   373.21	  1165.64	 10
  5	 M 	   504.25	  1094.60	  9
  6	 D 	   619.28	   963.56	  8
  7	 V 	   718.34	   848.54	  7
  8	 V 	   817.41	   749.47	  6
  9	 Y 	   980.48	   650.40	  5
 10	 A 	  1051.51	   487.34	  4
 11	 L 	  1164.60	   416.30	  3
 12	 K 	  1292.69	   303.21	  2
 13	 R 	        -  	   175.12	  1  _
+2 charge
		      B   	     Y
  1	 T 	    51.53	        -  	 13
  2	 V 	   101.07	   683.38	 12
  3	 T 	   151.59	   633.85	 11
  4	 A 	   187.11	   583.32	 10
  5	 M 	   252.63	   547.80	  9
  6	 D 	   310.14	   482.28	  8
  7	 V 	   359.68	   424.77	  7
  8	 V 	   409.21	   375.24	  6
  9	 Y 	   490.74	   325.70	  5
 10	 A 	   526.26	   244.17	  4
 11	 L 	   582.80	   208.65	  3
 12	 K 	   646.85	   152.11	  2
 13	 R 	        -  	    88.06	  1  _
```

**107. IPI00022420 Retinol-binding protein 4**

| Sequence | Charge | M+H+ | XCorr | DeltaCn |
| --- | --- | --- | --- | --- |
| R.LLNLDGTCADSYSFVFSRDPNGLPPEAQK.I | 3 | 3213.493 | 3.544 | 0.514 |

+1 charge

```
		      B   	     Y
  1	 L 	   114.09	        -  	 29
  2	 L 	   227.18	  3098.59	 28
  3	 N 	   341.22	  2985.50	 27
  4	 L 	   454.30	  2871.46	 26
  5	 D 	   569.33	  2758.38	 25
  6	 G 	   626.35	  2643.35	 24
  7	 T 	   727.40	  2586.33	 23
  8	 C 	   887.56	  2485.28	 22
  9	 A 	   958.60	  2325.11	 21
 10	 D 	  1073.63	  2254.08	 20
 11	 S 	  1160.66	  2139.05	 19
 12	 Y 	  1323.72	  2052.02	 18
 13	 S 	  1410.76	  1888.96	 17
 14	 F 	  1557.82	  1801.92	 16
 15	 V 	  1656.89	  1654.85	 15
 16	 F 	  1803.96	  1555.79	 14
 17	 S 	  1890.99	  1408.72	 13
 18	 R 	  2047.09	  1321.69	 12
 19	 D 	  2162.12	  1165.58	 11
 20	 P 	  2259.17	  1050.56	 10
 21	 N 	  2373.22	   953.51	  9
 22	 G 	  2430.24	   839.46	  8
 23	 L 	  2543.32	   782.44	  7
 24	 P 	  2640.37	   669.36	  6
 25	 P 	  2737.43	   572.30	  5
 26	 E 	  2866.47	   475.25	  4
 27	 A 	  2937.51	   346.21	  3
 28	 Q 	  3065.57	   275.17	  2
 29	 K 	        -  	   147.11	  1  _


+2 charge
		      B   	     Y
  1	 L 	    57.55	        -  	 29
  2	 L 	   114.09	  1549.80	 28
  3	 N 	   171.11	  1493.26	 27
  4	 L 	   227.65	  1436.23	 26
  5	 D 	   285.17	  1379.69	 25
  6	 G 	   313.68	  1322.18	 24
  7	 T 	   364.20	  1293.67	 23
  8	 C 	   444.29	  1243.14	 22
  9	 A 	   479.80	  1163.06	 21
 10	 D 	   537.32	  1127.54	 20
 11	 S 	   580.83	  1070.03	 19
 12	 Y 	   662.37	  1026.51	 18
 13	 S 	   705.88	   944.98	 17
 14	 F 	   779.42	   901.47	 16
 15	 V 	   828.95	   827.93	 15
 16	 F 	   902.48	   778.40	 14
 17	 S 	   946.00	   704.86	 13
 18	 R 	  1024.05	   661.35	 12
 19	 D 	  1081.56	   583.30	 11
 20	 P 	  1130.09	   525.78	 10
 21	 N 	  1187.11	   477.26	  9
 22	 G 	  1215.62	   420.23	  8
 23	 L 	  1272.16	   391.72	  7
 24	 P 	  1320.69	   335.18	  6
 25	 P 	  1369.22	   286.66	  5
 26	 E 	  1433.74	   238.13	  4
 27	 A 	  1469.26	   173.61	  3
 28	 Q 	  1533.29	   138.09	  2
 29	 K 	        -  	    74.06	  1  _


+3 charge
		      B   	     Y
  1	 L 	    38.70	        -  	 29
  2	 L 	    76.40	  1033.53	 28
  3	 N 	   114.41	   995.84	 27
  4	 L 	   152.11	   957.82	 26
  5	 D 	   190.45	   920.13	 25
  6	 G 	   209.46	   881.79	 24
  7	 T 	   243.14	   862.78	 23
  8	 C 	   296.53	   829.10	 22
  9	 A 	   320.21	   775.71	 21
 10	 D 	   358.55	   752.03	 20
 11	 S 	   387.56	   713.69	 19
 12	 Y 	   441.91	   684.68	 18
 13	 S 	   470.92	   630.32	 17
 14	 F 	   519.95	   601.31	 16
 15	 V 	   552.97	   552.29	 15
 16	 F 	   601.99	   519.27	 14
 17	 S 	   631.00	   470.24	 13
 18	 R 	   683.04	   441.23	 12
 19	 D 	   721.38	   389.20	 11
 20	 P 	   753.73	   350.86	 10
 21	 N 	   791.74	   318.51	  9
 22	 G 	   810.75	   280.49	  8
 23	 L 	   848.45	   261.49	  7
 24	 P 	   880.80	   223.79	  6
 25	 P 	   913.15	   191.44	  5
 26	 E 	   956.16	   159.09	  4
 27	 A 	   979.84	   116.07	  3
 28	 Q 	  1022.53	    92.40	  2
 29	 K 	        -  	    49.71	  1  _
```

**108. IPI00056309 Liver-expressed antimicrobial peptide 2**

| Sequence | Charge | M+H+ | XCorr | DeltaCn |
| --- | --- | --- | --- | --- |
| R.GVSLRPIGASCRDDSECITR.L | 3 | 2250.443 | 4.818 | 0.431 |

+1 charge

```
		      B   	     Y
  1	 G 	    58.03	        -  	 20
  2	 V 	   157.10	  2192.32	 19
  3	 S 	   244.13	  2093.26	 18
  4	 L 	   357.21	  2006.22	 17
  5	 R 	   513.31	  1893.14	 16
  6	 P 	   610.37	  1737.04	 15
  7	 I 	   723.45	  1639.99	 14
  8	 G 	   780.47	  1526.90	 13
  9	 A 	   851.51	  1469.88	 12
 10	 S 	   938.54	  1398.84	 11
 11	 C 	  1098.71	  1311.81	 10
 12	 R 	  1254.81	  1151.65	  9
 13	 D 	  1369.84	   995.54	  8
 14	 D 	  1484.86	   880.52	  7
 15	 S 	  1571.89	   765.49	  6
 16	 E 	  1700.94	   678.46	  5
 17	 C 	  1861.10	   549.42	  4
 18	 I 	  1974.19	   389.25	  3
 19	 T 	  2075.23	   276.17	  2
 20	 R 	        -  	   175.12	  1  _
+2 charge
		      B   	     Y
  1	 G 	    29.52	        -  	 20
  2	 V 	    79.05	  1096.67	 19
  3	 S 	   122.57	  1047.13	 18
  4	 L 	   179.11	  1003.62	 17
  5	 R 	   257.16	   947.07	 16
  6	 P 	   305.69	   869.02	 15
  7	 I 	   362.23	   820.50	 14
  8	 G 	   390.74	   763.95	 13
  9	 A 	   426.26	   735.44	 12
 10	 S 	   469.77	   699.93	 11
 11	 C 	   549.86	   656.41	 10
 12	 R 	   627.91	   576.33	  9
 13	 D 	   685.42	   498.28	  8
 14	 D 	   742.93	   440.76	  7
 15	 S 	   786.45	   383.25	  6
 16	 E 	   850.97	   339.73	  5
 17	 C 	   931.05	   275.21	  4
 18	 I 	   987.60	   195.13	  3
 19	 T 	  1038.12	   138.59	  2
 20	 R 	        -  	    88.06	  1  _

+3 charge
		      B   	     Y
  1	 G 	    20.01	        -  	 20
  2	 V 	    53.04	   731.45	 19
  3	 S 	    82.05	   698.42	 18
  4	 L 	   119.74	   669.41	 17
  5	 R 	   171.78	   631.72	 16
  6	 P 	   204.13	   579.68	 15
  7	 I 	   241.82	   547.33	 14
  8	 G 	   260.83	   509.64	 13
  9	 A 	   284.51	   490.63	 12
 10	 S 	   313.52	   466.95	 11
 11	 C 	   366.91	   437.94	 10
 12	 R 	   418.94	   384.55	  9
 13	 D 	   457.28	   332.52	  8
 14	 D 	   495.63	   294.18	  7
 15	 S 	   524.64	   255.84	  6
 16	 E 	   567.65	   226.82	  5
 17	 C 	   621.04	   183.81	  4
 18	 I 	   658.73	   130.42	  3
 19	 T 	   692.42	    92.73	  2
 20	 R 	        -  	    59.04	  1  _
```

**109. IPI00219827 Secreted Ly-6/uPAR-related protein 2**

| Sequence | Charge | M+H+ | XCorr | DeltaCn |
| --- | --- | --- | --- | --- |
| R.VLSNTEDLPLVTK.M | 2 | 1429.641 | 4.384 | 0.569 |

+1 charge

```
		      B   	     Y
  1	 V 	   100.08	        -  	 13
  2	 L 	   213.16	  1329.73	 12
  3	 S 	   300.19	  1216.64	 11
  4	 N 	   414.23	  1129.61	 10
  5	 T 	   515.28	  1015.57	  9
  6	 E 	   644.32	   914.52	  8
  7	 D 	   759.35	   785.48	  7
  8	 L 	   872.44	   670.45	  6
  9	 P 	   969.49	   557.37	  5
 10	 L 	  1082.57	   460.31	  4
 11	 V 	  1181.64	   347.23	  3
 12	 T 	  1282.69	   248.16	  2
 13	 K 	        -  	   147.11	  1  _
+2 charge
		      B   	     Y
  1	 V 	    50.54	        -  	 13
  2	 L 	   107.08	   665.37	 12
  3	 S 	   150.60	   608.82	 11
  4	 N 	   207.62	   565.31	 10
  5	 T 	   258.14	   508.29	  9
  6	 E 	   322.67	   457.76	  8
  7	 D 	   380.18	   393.24	  7
  8	 L 	   436.72	   335.73	  6
  9	 P 	   485.25	   279.19	  5
 10	 L 	   541.79	   230.66	  4
 11	 V 	   591.32	   174.12	  3
 12	 T 	   641.85	   124.58	  2
 13	 K 	        -  	    74.06	  1  _
```

**110. IPI00013446 Prostate stem cell antigen**

| Sequence | Charge | M+H+ | XCorr | DeltaCn |
| --- | --- | --- | --- | --- |
| R.AVGLLTVISK.G | 2 | 1001.245 | 2.853 | 0.373 |

+1 charge

```
		      B   	     Y
  1	 A 	    72.04	        -  	 10
  2	 V 	   171.11	   929.60	  9
  3	 G 	   228.13	   830.53	  8
  4	 L 	   341.22	   773.51	  7
  5	 L 	   454.30	   660.43	  6
  6	 T 	   555.35	   547.34	  5
  7	 V 	   654.42	   446.30	  4
  8	 I 	   767.50	   347.23	  3
  9	 S 	   854.53	   234.14	  2
 10	 K 	        -  	   147.11	  1  _
+2 charge
		      B   	     Y
  1	 A 	    36.53	        -  	 10
  2	 V 	    86.06	   465.31	  9
  3	 G 	   114.57	   415.77	  8
  4	 L 	   171.11	   387.26	  7
  5	 L 	   227.65	   330.72	  6
  6	 T 	   278.18	   274.18	  5
  7	 V 	   327.71	   223.65	  4
  8	 I 	   384.25	   174.12	  3
  9	 S 	   427.77	   117.58	  2
 10	 K 	        -  	    74.06	  1  _
```

**111. IPI00026259 N(4)-(beta-N-acetylglucosaminyl)-L-asparaginase**

| Sequence | Charge | M+H+ | XCorr | DeltaCn |
| --- | --- | --- | --- | --- |
| R.FLPSYQAVEYMR.R | 2 | 1504.735 | 3.386 | 0.561 |

+1 charge

```
		      B   	     Y
  1	 F 	   148.08	        -  	 12
  2	 L 	   261.16	  1356.66	 11
  3	 P 	   358.21	  1243.58	 10
  4	 S 	   445.24	  1146.52	  9
  5	 Y 	   608.31	  1059.49	  8
  6	 Q 	   736.37	   896.43	  7
  7	 A 	   807.40	   768.37	  6
  8	 V 	   906.47	   697.33	  5
  9	 E 	  1035.51	   598.27	  4
 10	 Y 	  1198.58	   469.22	  3
 11	 M 	  1329.62	   306.16	  2
 12	 R 	        -  	   175.12	  1  _
+2 charge
		      B   	     Y
  1	 F 	    74.54	        -  	 12
  2	 L 	   131.08	   678.83	 11
  3	 P 	   179.61	   622.29	 10
  4	 S 	   223.13	   573.77	  9
  5	 Y 	   304.66	   530.25	  8
  6	 Q 	   368.69	   448.72	  7
  7	 A 	   404.21	   384.69	  6
  8	 V 	   453.74	   349.17	  5
  9	 E 	   518.26	   299.64	  4
 10	 Y 	   599.79	   235.12	  3
 11	 M 	   665.31	   153.58	  2
 12	 R 	        -  	    88.06	  1  _
```

**112. IPI00004416 Charged multivesicular body protein 2a**

| Sequence | Charge | M+H+ | XCorr | DeltaCn |
| --- | --- | --- | --- | --- |
| K.AEAAASALADADADLEERLK.N | 2 | 2031.167 | 3.617 | 0.485 |

+1 charge

```
		      B   	     Y
  1	 A 	    72.04	        -  	 20
  2	 E 	   201.09	  1958.97	 19
  3	 A 	   272.12	  1829.92	 18
  4	 A 	   343.16	  1758.89	 17
  5	 A 	   414.20	  1687.85	 16
  6	 S 	   501.23	  1616.81	 15
  7	 A 	   572.27	  1529.78	 14
  8	 L 	   685.35	  1458.74	 13
  9	 A 	   756.39	  1345.66	 12
 10	 D 	   871.42	  1274.62	 11
 11	 A 	   942.45	  1159.60	 10
 12	 D 	  1057.48	  1088.56	  9
 13	 A 	  1128.52	   973.53	  8
 14	 D 	  1243.54	   902.49	  7
 15	 L 	  1356.63	   787.47	  6
 16	 E 	  1485.67	   674.38	  5
 17	 E 	  1614.71	   545.34	  4
 18	 R 	  1770.81	   416.30	  3
 19	 L 	  1883.90	   260.20	  2
 20	 K 	        -  	   147.11	  1  _
+2 charge
		      B   	     Y
  1	 A 	    36.53	        -  	 20
  2	 E 	   101.05	   979.99	 19
  3	 A 	   136.57	   915.47	 18
  4	 A 	   172.08	   879.95	 17
  5	 A 	   207.60	   844.43	 16
  6	 S 	   251.12	   808.91	 15
  7	 A 	   286.64	   765.39	 14
  8	 L 	   343.18	   729.88	 13
  9	 A 	   378.70	   673.33	 12
 10	 D 	   436.21	   637.81	 11
 11	 A 	   471.73	   580.30	 10
 12	 D 	   529.24	   544.78	  9
 13	 A 	   564.76	   487.27	  8
 14	 D 	   622.28	   451.75	  7
 15	 L 	   678.82	   394.24	  6
 16	 E 	   743.34	   337.70	  5
 17	 E 	   807.86	   273.17	  4
 18	 R 	   885.91	   208.65	  3
 19	 L 	   942.45	   130.60	  2
 20	 K 	        -  	    74.06	  1  _
```

**113. IPI00000760 N(G),N(G)-dimethylarginine dimethylaminohydrolase 2**

| Sequence | Charge | M+H+ | XCorr | DeltaCn |
| --- | --- | --- | --- | --- |
| R.GVPESLASGEGAGAGLPALDLAK.A | 2 | 2081.313 | 4.096 | 0.528 |

+1 charge

```
		      B   	     Y
  1	 G 	    58.03	        -  	 23
  2	 V 	   157.10	  2023.07	 22
  3	 P 	   254.15	  1924.00	 21
  4	 E 	   383.19	  1826.95	 20
  5	 S 	   470.22	  1697.91	 19
  6	 L 	   583.31	  1610.87	 18
  7	 A 	   654.35	  1497.79	 17
  8	 S 	   741.38	  1426.75	 16
  9	 G 	   798.40	  1339.72	 15
 10	 E 	   927.44	  1282.70	 14
 11	 G 	   984.46	  1153.66	 13
 12	 A 	  1055.50	  1096.64	 12
 13	 G 	  1112.52	  1025.60	 11
 14	 A 	  1183.56	   968.58	 10
 15	 G 	  1240.58	   897.54	  9
 16	 L 	  1353.66	   840.52	  8
 17	 P 	  1450.72	   727.43	  7
 18	 A 	  1521.75	   630.38	  6
 19	 L 	  1634.84	   559.34	  5
 20	 D 	  1749.87	   446.26	  4
 21	 L 	  1862.95	   331.23	  3
 22	 A 	  1933.99	   218.15	  2
 23	 K 	        -  	   147.11	  1  _
+2 charge
		      B   	     Y
  1	 G 	    29.52	        -  	 23
  2	 V 	    79.05	  1012.04	 22
  3	 P 	   127.58	   962.50	 21
  4	 E 	   192.10	   913.98	 20
  5	 S 	   235.62	   849.46	 19
  6	 L 	   292.16	   805.94	 18
  7	 A 	   327.68	   749.40	 17
  8	 S 	   371.19	   713.88	 16
  9	 G 	   399.70	   670.36	 15
 10	 E 	   464.22	   641.85	 14
 11	 G 	   492.74	   577.33	 13
 12	 A 	   528.25	   548.82	 12
 13	 G 	   556.76	   513.30	 11
 14	 A 	   592.28	   484.79	 10
 15	 G 	   620.79	   449.27	  9
 16	 L 	   677.34	   420.76	  8
 17	 P 	   725.86	   364.22	  7
 18	 A 	   761.38	   315.69	  6
 19	 L 	   817.92	   280.18	  5
 20	 D 	   875.44	   223.63	  4
 21	 L 	   931.98	   166.12	  3
 22	 A 	   967.50	   109.58	  2
 23	 K 	        -  	    74.06	  1  _
```

**114. IPI00176776 Uncharacterized protein C11orf85**

| Sequence | Charge | M+H+ | XCorr | DeltaCn |
| --- | --- | --- | --- | --- |
| K.WFHENLSPGKPISDSPLGLVPVEKK.A | 3 | 2776.182 | 4.453 | 0.232 |

+1 charge

```
		      B   	     Y
  1	 W 	   187.09	        -  	 25
  2	 F 	   334.16	  2588.41	 24
  3	 H 	   471.21	  2441.34	 23
  4	 E 	   600.26	  2304.28	 22
  5	 N 	   714.30	  2175.24	 21
  6	 L 	   827.38	  2061.20	 20
  7	 S 	   914.42	  1948.11	 19
  8	 P 	  1011.47	  1861.08	 18
  9	 G 	  1068.49	  1764.03	 17
 10	 K 	  1196.58	  1707.01	 16
 11	 P 	  1293.64	  1578.91	 15
 12	 I 	  1406.72	  1481.86	 14
 13	 S 	  1493.75	  1368.77	 13
 14	 D 	  1608.78	  1281.74	 12
 15	 S 	  1695.81	  1166.71	 11
 16	 P 	  1792.87	  1079.68	 10
 17	 L 	  1905.95	   982.63	  9
 18	 G 	  1962.97	   869.55	  8
 19	 L 	  2076.05	   812.52	  7
 20	 V 	  2175.12	   699.44	  6
 21	 P 	  2272.18	   600.37	  5
 22	 V 	  2371.24	   503.32	  4
 23	 E 	  2500.29	   404.25	  3
 24	 K 	  2628.38	   275.21	  2
 25	 K 	        -  	   147.11	  1  _


+2 charge
		      B   	     Y
  1	 W 	    94.05	        -  	 25
  2	 F 	   167.58	  1294.71	 24
  3	 H 	   236.11	  1221.17	 23
  4	 E 	   300.63	  1152.64	 22
  5	 N 	   357.65	  1088.12	 21
  6	 L 	   414.20	  1031.10	 20
  7	 S 	   457.71	   974.56	 19
  8	 P 	   506.24	   931.04	 18
  9	 G 	   534.75	   882.52	 17
 10	 K 	   598.80	   854.01	 16
 11	 P 	   647.32	   789.96	 15
 12	 I 	   703.86	   741.43	 14
 13	 S 	   747.38	   684.89	 13
 14	 D 	   804.89	   641.37	 12
 15	 S 	   848.41	   583.86	 11
 16	 P 	   896.94	   540.34	 10
 17	 L 	   953.48	   491.82	  9
 18	 G 	   981.99	   435.28	  8
 19	 L 	  1038.53	   406.77	  7
 20	 V 	  1088.07	   350.22	  6
 21	 P 	  1136.59	   300.69	  5
 22	 V 	  1186.13	   252.16	  4
 23	 E 	  1250.65	   202.63	  3
 24	 K 	  1314.69	   138.11	  2
 25	 K 	        -  	    74.06	  1  _
+3 charge
		      B   	     Y
  1	 W 	    63.03	        -  	 25
  2	 F 	   112.06	   863.47	 24
  3	 H 	   157.74	   814.45	 23
  4	 E 	   200.76	   768.77	 22
  5	 N 	   238.77	   725.75	 21
  6	 L 	   276.47	   687.74	 20
  7	 S 	   305.48	   650.04	 19
  8	 P 	   337.83	   621.03	 18
  9	 G 	   356.83	   588.68	 17
 10	 K 	   399.53	   569.67	 16
 11	 P 	   431.88	   526.97	 15
 12	 I 	   469.58	   494.62	 14
 13	 S 	   498.59	   456.93	 13
 14	 D 	   536.93	   427.92	 12
 15	 S 	   565.94	   389.58	 11
 16	 P 	   598.29	   360.57	 10
 17	 L 	   635.99	   328.21	  9
 18	 G 	   655.00	   290.52	  8
 19	 L 	   692.69	   271.51	  7
 20	 V 	   725.71	   233.82	  6
 21	 P 	   758.06	   200.80	  5
 22	 V 	   791.09	   168.44	  4
 23	 E 	   834.10	   135.42	  3
 24	 K 	   876.80	    92.41	  2
 25	 K 	        -  	    49.71	  1  _
```

**115. IPI00409698 Isoform 1 of Transportin-2**

| Sequence | Charge | M+H+ | XCorr | DeltaCn |
| --- | --- | --- | --- | --- |
| K.QECLNNIGDASSLIR.A | 2 | 1690.831 | 3.353 | 0.211 |

+1 charge

```
		      B   	     Y
  1	 Q 	   129.07	        -  	 15
  2	 E 	   258.11	  1561.90	 14
  3	 C 	   418.27	  1432.86	 13
  4	 L 	   531.36	  1272.69	 12
  5	 N 	   645.40	  1159.61	 11
  6	 N 	   759.44	  1045.56	 10
  7	 I 	   872.53	   931.52	  9
  8	 G 	   929.55	   818.44	  8
  9	 D 	  1044.58	   761.42	  7
 10	 A 	  1115.61	   646.39	  6
 11	 S 	  1202.65	   575.35	  5
 12	 S 	  1289.68	   488.32	  4
 13	 L 	  1402.76	   401.29	  3
 14	 I 	  1515.85	   288.20	  2
 15	 R 	        -  	   175.12	  1  _
+2 charge
		      B   	     Y
  1	 Q 	    65.04	        -  	 15
  2	 E 	   129.56	   781.45	 14
  3	 C 	   209.64	   716.93	 13
  4	 L 	   266.18	   636.85	 12
  5	 N 	   323.20	   580.31	 11
  6	 N 	   380.23	   523.29	 10
  7	 I 	   436.77	   466.26	  9
  8	 G 	   465.28	   409.72	  8
  9	 D 	   522.79	   381.21	  7
 10	 A 	   558.31	   323.70	  6
 11	 S 	   601.83	   288.18	  5
 12	 S 	   645.34	   244.66	  4
 13	 L 	   701.88	   201.15	  3
 14	 I 	   758.43	   144.61	  2
 15	 R 	        -  	    88.06	  1  _
```

**116. IPI00018901 Isoform 1 of Gamma-glutamyltranspeptidase 1**

| Sequence | Charge | M+H+ | XCorr | DeltaCn |
| --- | --- | --- | --- | --- |
| R.TVIEQQPVLCEVFCR.D | 2 | 1879.134 | 3.513 | 0.505 |

+1 charge

```
		      B   	     Y
  1	 T 	   102.05	        -  	 15
  2	 V 	   201.12	  1777.15	 14
  3	 I 	   314.21	  1678.08	 13
  4	 E 	   443.25	  1564.99	 12
  5	 Q 	   571.31	  1435.95	 11
  6	 Q 	   699.37	  1307.89	 10
  7	 P 	   796.42	  1179.83	  9
  8	 V 	   895.49	  1082.78	  8
  9	 L 	  1008.57	   983.71	  7
 10	 C 	  1168.74	   870.63	  6
 11	 E 	  1297.78	   710.46	  5
 12	 V 	  1396.85	   581.42	  4
 13	 F 	  1543.92	   482.35	  3
 14	 C 	  1704.08	   335.28	  2
 15	 R 	        -  	   175.12	  1  _
+2 charge
		      B   	     Y
  1	 T 	    51.53	        -  	 15
  2	 V 	   101.07	   889.08	 14
  3	 I 	   157.61	   839.54	 13
  4	 E 	   222.13	   783.00	 12
  5	 Q 	   286.16	   718.48	 11
  6	 Q 	   350.19	   654.45	 10
  7	 P 	   398.71	   590.42	  9
  8	 V 	   448.25	   541.89	  8
  9	 L 	   504.79	   492.36	  7
 10	 C 	   584.87	   435.82	  6
 11	 E 	   649.39	   355.74	  5
 12	 V 	   698.93	   291.21	  4
 13	 F 	   772.46	   241.68	  3
 14	 C 	   852.54	   168.15	  2
 15	 R 	        -  	    88.06	  1  _
```

**117. IPI00290085 Cadherin-2**

| Sequence | Charge | M+H+ | XCorr | DeltaCn |
| --- | --- | --- | --- | --- |
| R.FAIQTDPNSNDGLVTVVKPIDFETNR.M | 3 | 2892.17 | 4.764 | 0.582 |

+1 charge

```
		      B   	     Y
  1	 F 	   148.08	        -  	 26
  2	 A 	   219.11	  2743.39	 25
  3	 I 	   332.20	  2672.35	 24
  4	 Q 	   460.26	  2559.27	 23
  5	 T 	   561.30	  2431.21	 22
  6	 D 	   676.33	  2330.16	 21
  7	 P 	   773.38	  2215.14	 20
  8	 N 	   887.43	  2118.08	 19
  9	 S 	   974.46	  2004.04	 18
 10	 N 	  1088.50	  1917.01	 17
 11	 D 	  1203.53	  1802.96	 16
 12	 G 	  1260.55	  1687.94	 15
 13	 L 	  1373.63	  1630.92	 14
 14	 V 	  1472.70	  1517.83	 13
 15	 T 	  1573.75	  1418.76	 12
 16	 V 	  1672.82	  1317.72	 11
 17	 V 	  1771.89	  1218.65	 10
 18	 K 	  1899.98	  1119.58	  9
 19	 P 	  1997.03	   991.48	  8
 20	 I 	  2110.12	   894.43	  7
 21	 D 	  2225.14	   781.35	  6
 22	 F 	  2372.21	   666.32	  5
 23	 E 	  2501.26	   519.25	  4
 24	 T 	  2602.30	   390.21	  3
 25	 N 	  2716.35	   289.16	  2
 26	 R 	        -  	   175.12	  1  _


+2 charge
		      B   	     Y
  1	 F 	    74.54	        -  	 26
  2	 A 	   110.06	  1372.20	 25
  3	 I 	   166.60	  1336.68	 24
  4	 Q 	   230.63	  1280.14	 23
  5	 T 	   281.16	  1216.11	 22
  6	 D 	   338.67	  1165.58	 21
  7	 P 	   387.20	  1108.07	 20
  8	 N 	   444.22	  1059.54	 19
  9	 S 	   487.73	  1002.52	 18
 10	 N 	   544.75	   959.01	 17
 11	 D 	   602.27	   901.99	 16
 12	 G 	   630.78	   844.47	 15
 13	 L 	   687.32	   815.96	 14
 14	 V 	   736.85	   759.42	 13
 15	 T 	   787.38	   709.89	 12
 16	 V 	   836.91	   659.36	 11
 17	 V 	   886.45	   609.83	 10
 18	 K 	   950.49	   560.29	  9
 19	 P 	   999.02	   496.25	  8
 20	 I 	  1055.56	   447.72	  7
 21	 D 	  1113.08	   391.18	  6
 22	 F 	  1186.61	   333.66	  5
 23	 E 	  1251.13	   260.13	  4
 24	 T 	  1301.66	   195.61	  3
 25	 N 	  1358.68	   145.08	  2
 26	 R 	        -  	    88.06	  1  _


+3 charge
		      B   	     Y
  1	 F 	    50.03	        -  	 26
  2	 A 	    73.71	   915.13	 25
  3	 I 	   111.40	   891.46	 24
  4	 Q 	   154.09	   853.76	 23
  5	 T 	   187.77	   811.07	 22
  6	 D 	   226.11	   777.39	 21
  7	 P 	   258.47	   739.05	 20
  8	 N 	   296.48	   706.70	 19
  9	 S 	   325.49	   668.68	 18
 10	 N 	   363.51	   639.67	 17
 11	 D 	   401.85	   601.66	 16
 12	 G 	   420.85	   563.32	 15
 13	 L 	   458.55	   544.31	 14
 14	 V 	   491.57	   506.62	 13
 15	 T 	   525.25	   473.59	 12
 16	 V 	   558.28	   439.91	 11
 17	 V 	   591.30	   406.89	 10
 18	 K 	   634.00	   373.86	  9
 19	 P 	   666.35	   331.17	  8
 20	 I 	   704.04	   298.82	  7
 21	 D 	   742.39	   261.12	  6
 22	 F 	   791.41	   222.78	  5
 23	 E 	   834.42	   173.76	  4
 24	 T 	   868.11	   130.74	  3
 25	 N 	   906.12	    97.06	  2
 26	 R 	        -  	    59.04	  1  _
```

**118. IPI00410585 Isoform 1 of Crumbs homolog 2**

| Sequence | Charge | M+H+ | XCorr | DeltaCn |
| --- | --- | --- | --- | --- |
| R.GLASDLGFLQGPGAVR.I | 2 | 1558.764 | 3.073 | 0.461 |

+1 charge

```
		      B   	     Y
  1	 G 	    58.03	        -  	 16
  2	 L 	   171.11	  1500.82	 15
  3	 A 	   242.15	  1387.73	 14
  4	 S 	   329.18	  1316.70	 13
  5	 D 	   444.21	  1229.66	 12
  6	 L 	   557.29	  1114.64	 11
  7	 G 	   614.31	  1001.55	 10
  8	 F 	   761.38	   944.53	  9
  9	 L 	   874.47	   797.46	  8
 10	 Q 	  1002.53	   684.38	  7
 11	 G 	  1059.55	   556.32	  6
 12	 P 	  1156.60	   499.30	  5
 13	 G 	  1213.62	   402.25	  4
 14	 A 	  1284.66	   345.22	  3
 15	 V 	  1383.73	   274.19	  2
 16	 R 	        -  	   175.12	  1  _
+2 charge
		      B   	     Y
  1	 G 	    29.52	        -  	 16
  2	 L 	    86.06	   750.91	 15
  3	 A 	   121.58	   694.37	 14
  4	 S 	   165.09	   658.85	 13
  5	 D 	   222.61	   615.34	 12
  6	 L 	   279.15	   557.82	 11
  7	 G 	   307.66	   501.28	 10
  8	 F 	   381.20	   472.77	  9
  9	 L 	   437.74	   399.24	  8
 10	 Q 	   501.77	   342.69	  7
 11	 G 	   530.28	   278.66	  6
 12	 P 	   578.80	   250.15	  5
 13	 G 	   607.31	   201.63	  4
 14	 A 	   642.83	   173.12	  3
 15	 V 	   692.37	   137.60	  2
 16	 R 	        -  	    88.06	  1  _
```

**119. IPI00418169 Isoform 2 of Annexin A2**

| Sequence | Charge | M+H+ | XCorr | DeltaCn |
| --- | --- | --- | --- | --- |
| K.GLGTDEDSLIEIICSR.T | 2 | 1778.933 | 2.865 | 0.483 |

+1 charge

```
		      B   	     Y
  1	 G 	    58.03	        -  	 16
  2	 L 	   171.11	  1720.98	 15
  3	 G 	   228.13	  1607.89	 14
  4	 T 	   329.18	  1550.87	 13
  5	 D 	   444.21	  1449.82	 12
  6	 E 	   573.25	  1334.80	 11
  7	 D 	   688.28	  1205.75	 10
  8	 S 	   775.31	  1090.73	  9
  9	 L 	   888.39	  1003.70	  8
 10	 I 	  1001.48	   890.61	  7
 11	 E 	  1130.52	   777.53	  6
 12	 I 	  1243.61	   648.48	  5
 13	 I 	  1356.69	   535.40	  4
 14	 C 	  1516.85	   422.32	  3
 15	 S 	  1603.89	   262.15	  2
 16	 R 	        -  	   175.12	  1  _
+2 charge
		      B   	     Y
  1	 G 	    29.52	        -  	 16
  2	 L 	    86.06	   860.99	 15
  3	 G 	   114.57	   804.45	 14
  4	 T 	   165.09	   775.94	 13
  5	 D 	   222.61	   725.42	 12
  6	 E 	   287.13	   667.90	 11
  7	 D 	   344.64	   603.38	 10
  8	 S 	   388.16	   545.87	  9
  9	 L 	   444.70	   502.35	  8
 10	 I 	   501.24	   445.81	  7
 11	 E 	   565.76	   389.27	  6
 12	 I 	   622.31	   324.75	  5
 13	 I 	   678.85	   268.20	  4
 14	 C 	   758.93	   211.66	  3
 15	 S 	   802.45	   131.58	  2
 16	 R 	        -  	    88.06	  1  _
```

**120. IPI00394992 Isoform 2 of N-acetylmuramoyl-L-alanine amidase**

| Sequence | Charge | M+H+ | XCorr | DeltaCn |
| --- | --- | --- | --- | --- |
| R.TDCPGDALFDLLR.T | 2 | 1493.637 | 3.404 | 0.491 |

+1 charge

```
		      B   	     Y
  1	 T 	   102.05	        -  	 13
  2	 D 	   217.08	  1391.80	 12
  3	 C 	   377.25	  1276.77	 11
  4	 P 	   474.30	  1116.60	 10
  5	 G 	   531.32	  1019.55	  9
  6	 D 	   646.35	   962.53	  8
  7	 A 	   717.39	   847.50	  7
  8	 L 	   830.47	   776.47	  6
  9	 F 	   977.54	   663.38	  5
 10	 D 	  1092.56	   516.31	  4
 11	 L 	  1205.65	   401.29	  3
 12	 L 	  1318.73	   288.20	  2
 13	 R 	        -  	   175.12	  1  _
+2 charge
		      B   	     Y
  1	 T 	    51.53	        -  	 13
  2	 D 	   109.04	   696.40	 12
  3	 C 	   189.13	   638.89	 11
  4	 P 	   237.65	   558.81	 10
  5	 G 	   266.16	   510.28	  9
  6	 D 	   323.68	   481.77	  8
  7	 A 	   359.20	   424.26	  7
  8	 L 	   415.74	   388.74	  6
  9	 F 	   489.27	   332.19	  5
 10	 D 	   546.79	   258.66	  4
 11	 L 	   603.33	   201.15	  3
 12	 L 	   659.87	   144.61	  2
 13	 R 	        -  	    88.06	  1  _
```

**121. IPI00794461 Histone H2B type 1-N**

| Sequence | Charge | M+H+ | XCorr | DeltaCn |
| --- | --- | --- | --- | --- |
| K.AMGIMNSFVNDIFER.I | 2 | 1745.017 | 4.16 | 0.524 |

+1 charge

```
		      B   	     Y
  1	 A 	    72.04	        -  	 15
  2	 M 	   203.08	  1672.78	 14
  3	 G 	   260.11	  1541.74	 13
  4	 I 	   373.19	  1484.72	 12
  5	 M 	   504.23	  1371.64	 11
  6	 N 	   618.27	  1240.60	 10
  7	 S 	   705.31	  1126.55	  9
  8	 F 	   852.37	  1039.52	  8
  9	 V 	   951.44	   892.45	  7
 10	 N 	  1065.49	   793.38	  6
 11	 D 	  1180.51	   679.34	  5
 12	 I 	  1293.60	   564.31	  4
 13	 F 	  1440.67	   451.23	  3
 14	 E 	  1569.71	   304.16	  2
 15	 R 	        -  	   175.12	  1  _
+2 charge
		      B   	     Y
  1	 A 	    36.53	        -  	 15
  2	 M 	   102.05	   836.89	 14
  3	 G 	   130.56	   771.37	 13
  4	 I 	   187.10	   742.86	 12
  5	 M 	   252.62	   686.32	 11
  6	 N 	   309.64	   620.80	 10
  7	 S 	   353.16	   563.78	  9
  8	 F 	   426.69	   520.26	  8
  9	 V 	   476.22	   446.73	  7
 10	 N 	   533.25	   397.20	  6
 11	 D 	   590.76	   340.17	  5
 12	 I 	   647.30	   282.66	  4
 13	 F 	   720.84	   226.12	  3
 14	 E 	   785.36	   152.58	  2
 15	 R 	        -  	    88.06	  1  _
```

**122. IPI00219018 Glyceraldehyde-3-phosphate dehydrogenase**

| Sequence | Charge | M+H+ | XCorr | DeltaCn |
| --- | --- | --- | --- | --- |
| K.VIHDNFGIVEGLMTTVHAITATQK.T | 3 | 2596.987 | 5.97 | 0.579 |

+1 charge

```
		      B   	     Y
  1	 V 	   100.08	        -  	 24
  2	 I 	   213.16	  2496.29	 23
  3	 H 	   350.22	  2383.21	 22
  4	 D 	   465.25	  2246.15	 21
  5	 N 	   579.29	  2131.12	 20
  6	 F 	   726.36	  2017.08	 19
  7	 G 	   783.38	  1870.01	 18
  8	 I 	   896.46	  1812.99	 17
  9	 V 	   995.53	  1699.90	 16
 10	 E 	  1124.57	  1600.84	 15
 11	 G 	  1181.59	  1471.79	 14
 12	 L 	  1294.68	  1414.77	 13
 13	 M 	  1425.72	  1301.69	 12
 14	 T 	  1526.77	  1170.65	 11
 15	 T 	  1627.81	  1069.60	 10
 16	 V 	  1726.88	   968.55	  9
 17	 H 	  1863.94	   869.48	  8
 18	 A 	  1934.98	   732.43	  7
 19	 I 	  2048.06	   661.39	  6
 20	 T 	  2149.11	   548.30	  5
 21	 A 	  2220.15	   447.26	  4
 22	 T 	  2321.20	   376.22	  3
 23	 Q 	  2449.25	   275.17	  2
 24	 K 	        -  	   147.11	  1  _


+2 charge
		      B   	     Y
  1	 V 	    50.54	        -  	 24
  2	 I 	   107.08	  1248.65	 23
  3	 H 	   175.61	  1192.11	 22
  4	 D 	   233.13	  1123.58	 21
  5	 N 	   290.15	  1066.06	 20
  6	 F 	   363.68	  1009.04	 19
  7	 G 	   392.19	   935.51	 18
  8	 I 	   448.73	   907.00	 17
  9	 V 	   498.27	   850.46	 16
 10	 E 	   562.79	   800.92	 15
 11	 G 	   591.30	   736.40	 14
 12	 L 	   647.84	   707.89	 13
 13	 M 	   713.36	   651.35	 12
 14	 T 	   763.89	   585.83	 11
 15	 T 	   814.41	   535.30	 10
 16	 V 	   863.95	   484.78	  9
 17	 H 	   932.47	   435.25	  8
 18	 A 	   967.99	   366.72	  7
 19	 I 	  1024.54	   331.20	  6
 20	 T 	  1075.06	   274.66	  5
 21	 A 	  1110.58	   224.13	  4
 22	 T 	  1161.10	   188.61	  3
 23	 Q 	  1225.13	   138.09	  2
 24	 K 	        -  	    74.06	  1  _
+3 charge
		      B   	     Y
  1	 V 	    34.03	        -  	 24
  2	 I 	    71.72	   832.77	 23
  3	 H 	   117.41	   795.07	 22
  4	 D 	   155.75	   749.39	 21
  5	 N 	   193.77	   711.05	 20
  6	 F 	   242.79	   673.03	 19
  7	 G 	   261.80	   624.01	 18
  8	 I 	   299.49	   605.00	 17
  9	 V 	   332.52	   567.31	 16
 10	 E 	   375.53	   534.28	 15
 11	 G 	   394.54	   491.27	 14
 12	 L 	   432.23	   472.26	 13
 13	 M 	   475.91	   434.57	 12
 14	 T 	   509.59	   390.89	 11
 15	 T 	   543.28	   357.20	 10
 16	 V 	   576.30	   323.52	  9
 17	 H 	   621.99	   290.50	  8
 18	 A 	   645.66	   244.81	  7
 19	 I 	   683.36	   221.13	  6
 20	 T 	   717.04	   183.44	  5
 21	 A 	   740.72	   149.76	  4
 22	 T 	   774.40	   126.08	  3
 23	 Q 	   817.09	    92.40	  2
 24	 K 	        -  	    49.71	  1  _
```

**123. IPI00939667 Isoform 1 of Tripeptidyl-peptidase 1**

| Sequence | Charge | M+H+ | XCorr | DeltaCn |
| --- | --- | --- | --- | --- |
| R.LFGGNFAHQASVAR.V | 2 | 1475.636 | 4.66 | 0.528 |

+1 charge

```
		      B   	     Y
  1	 L 	   114.09	        -  	 14
  2	 F 	   261.16	  1361.67	 13
  3	 G 	   318.18	  1214.60	 12
  4	 G 	   375.20	  1157.58	 11
  5	 N 	   489.25	  1100.56	 10
  6	 F 	   636.31	   986.52	  9
  7	 A 	   707.35	   839.45	  8
  8	 H 	   844.41	   768.41	  7
  9	 Q 	   972.47	   631.35	  6
 10	 A 	  1043.51	   503.29	  5
 11	 S 	  1130.54	   432.26	  4
 12	 V 	  1229.61	   345.22	  3
 13	 A 	  1300.64	   246.16	  2
 14	 R 	        -  	   175.12	  1  _
+2 charge
		      B   	     Y
  1	 L 	    57.55	        -  	 14
  2	 F 	   131.08	   681.34	 13
  3	 G 	   159.59	   607.80	 12
  4	 G 	   188.10	   579.29	 11
  5	 N 	   245.13	   550.78	 10
  6	 F 	   318.66	   493.76	  9
  7	 A 	   354.18	   420.23	  8
  8	 H 	   422.71	   384.71	  7
  9	 Q 	   486.74	   316.18	  6
 10	 A 	   522.26	   252.15	  5
 11	 S 	   565.77	   216.63	  4
 12	 V 	   615.31	   173.12	  3
 13	 A 	   650.83	   123.58	  2
 14	 R 	        -  	    88.06	  1  _
```

**124. IPI00066856 Ly6/PLAUR domain-containing protein 2**

| Sequence | Charge | M+H+ | XCorr | DeltaCn |
| --- | --- | --- | --- | --- |
| R.EIVYPFQGDSTVTK.S | 2 | 1584.751 | 3.159 | 0.456 |

+1 charge

```
		      B   	     Y
  1	 E 	   130.05	        -  	 14
  2	 I 	   243.13	  1454.75	 13
  3	 V 	   342.20	  1341.67	 12
  4	 Y 	   505.27	  1242.60	 11
  5	 P 	   602.32	  1079.54	 10
  6	 F 	   749.39	   982.48	  9
  7	 Q 	   877.45	   835.42	  8
  8	 G 	   934.47	   707.36	  7
  9	 D 	  1049.49	   650.34	  6
 10	 S 	  1136.53	   535.31	  5
 11	 T 	  1237.57	   448.28	  4
 12	 V 	  1336.64	   347.23	  3
 13	 T 	  1437.69	   248.16	  2
 14	 K 	        -  	   147.11	  1  _
+2 charge
		      B   	     Y
  1	 E 	    65.53	        -  	 14
  2	 I 	   122.07	   727.88	 13
  3	 V 	   171.60	   671.34	 12
  4	 Y 	   253.14	   621.80	 11
  5	 P 	   301.66	   540.27	 10
  6	 F 	   375.20	   491.75	  9
  7	 Q 	   439.23	   418.21	  8
  8	 G 	   467.74	   354.18	  7
  9	 D 	   525.25	   325.67	  6
 10	 S 	   568.77	   268.16	  5
 11	 T 	   619.29	   224.64	  4
 12	 V 	   668.82	   174.12	  3
 13	 T 	   719.35	   124.58	  2
 14	 K 	        -  	    74.06	  1  _
```

**125. IPI00025753 Desmoglein-1**

| Sequence | Charge | M+H+ | XCorr | DeltaCn |
| --- | --- | --- | --- | --- |
| R.YVMGNNPADLLAVDSR.T | 2 | 1735.943 | 3.42 | 0.34 |

+1 charge

```
		      B   	     Y
  1	 Y 	   164.07	        -  	 16
  2	 V 	   263.14	  1571.78	 15
  3	 M 	   394.18	  1472.72	 14
  4	 G 	   451.20	  1341.68	 13
  5	 N 	   565.24	  1284.65	 12
  6	 N 	   679.29	  1170.61	 11
  7	 P 	   776.34	  1056.57	 10
  8	 A 	   847.38	   959.52	  9
  9	 D 	   962.40	   888.48	  8
 10	 L 	  1075.49	   773.45	  7
 11	 L 	  1188.57	   660.37	  6
 12	 A 	  1259.61	   547.28	  5
 13	 V 	  1358.68	   476.25	  4
 14	 D 	  1473.70	   377.18	  3
 15	 S 	  1560.74	   262.15	  2
 16	 R 	        -  	   175.12	  1  _
+2 charge
		      B   	     Y
  1	 Y 	    82.54	        -  	 16
  2	 V 	   132.07	   786.40	 15
  3	 M 	   197.59	   736.86	 14
  4	 G 	   226.10	   671.34	 13
  5	 N 	   283.13	   642.83	 12
  6	 N 	   340.15	   585.81	 11
  7	 P 	   388.67	   528.79	 10
  8	 A 	   424.19	   480.26	  9
  9	 D 	   481.71	   444.74	  8
 10	 L 	   538.25	   387.23	  7
 11	 L 	   594.79	   330.69	  6
 12	 A 	   630.31	   274.15	  5
 13	 V 	   679.84	   238.63	  4
 14	 D 	   737.36	   189.09	  3
 15	 S 	   780.87	   131.58	  2
 16	 R 	        -  	    88.06	  1  _
```

**126. IPI00217471 Hemoglobin subunit epsilon**

| Sequence | Charge | M+H+ | XCorr | DeltaCn |
| --- | --- | --- | --- | --- |
| R.LLVVYPWTQR.F | 2 | 1275.523 | 2.889 | 0.226 |

+1 charge

```
		      B   	     Y
  1	 L 	   114.09	        -  	 10
  2	 L 	   227.18	  1161.64	  9
  3	 V 	   326.24	  1048.56	  8
  4	 V 	   425.31	   949.49	  7
  5	 Y 	   588.38	   850.42	  6
  6	 P 	   685.43	   687.36	  5
  7	 W 	   871.51	   590.30	  4
  8	 T 	   972.56	   404.23	  3
  9	 Q 	  1100.61	   303.18	  2
 10	 R 	        -  	   175.12	  1  _
+2 charge
		      B   	     Y
  1	 L 	    57.55	        -  	 10
  2	 L 	   114.09	   581.32	  9
  3	 V 	   163.63	   524.78	  8
  4	 V 	   213.16	   475.25	  7
  5	 Y 	   294.69	   425.71	  6
  6	 P 	   343.22	   344.18	  5
  7	 W 	   436.26	   295.66	  4
  8	 T 	   486.78	   202.62	  3
  9	 Q 	   550.81	   152.09	  2
 10	 R 	        -  	    88.06	  1  _
```

**127. IPI00006608 Isoform APP770 of Amyloid beta A4 protein (Fragment)**

| Sequence | Charge | M+H+ | XCorr | DeltaCn |
| --- | --- | --- | --- | --- |
| R.CLVGEFVSDALLVPDKCK.F | 2 | 2051.356 | 3.219 | 0.556 |

+1 charge

```
		      B   	     Y
  1	 C 	   161.17	        -  	 18
  2	 L 	   274.26	  1890.14	 17
  3	 V 	   373.32	  1777.05	 16
  4	 G 	   430.35	  1677.99	 15
  5	 E 	   559.39	  1620.96	 14
  6	 F 	   706.46	  1491.92	 13
  7	 V 	   805.53	  1344.85	 12
  8	 S 	   892.56	  1245.79	 11
  9	 D 	  1007.58	  1158.75	 10
 10	 A 	  1078.62	  1043.73	  9
 11	 L 	  1191.71	   972.69	  8
 12	 L 	  1304.79	   859.61	  7
 13	 V 	  1403.86	   746.52	  6
 14	 P 	  1500.91	   647.45	  5
 15	 D 	  1615.94	   550.40	  4
 16	 K 	  1744.03	   435.37	  3
 17	 C 	  1904.20	   307.28	  2
 18	 K 	        -  	   147.11	  1  _
+2 charge
		      B   	     Y
  1	 C 	    81.09	        -  	 18
  2	 L 	   137.63	   945.57	 17
  3	 V 	   187.17	   889.03	 16
  4	 G 	   215.68	   839.50	 15
  5	 E 	   280.20	   810.99	 14
  6	 F 	   353.73	   746.46	 13
  7	 V 	   403.27	   672.93	 12
  8	 S 	   446.78	   623.40	 11
  9	 D 	   504.30	   579.88	 10
 10	 A 	   539.81	   522.37	  9
 11	 L 	   596.36	   486.85	  8
 12	 L 	   652.90	   430.31	  7
 13	 V 	   702.43	   373.76	  6
 14	 P 	   750.96	   324.23	  5
 15	 D 	   808.47	   275.70	  4
 16	 K 	   872.52	   218.19	  3
 17	 C 	   952.60	   154.14	  2
 18	 K 	        -  	    74.06	  1  _
```

**128. IPI00936444 Mucin-5B**

| Sequence | Charge | M+H+ | XCorr | DeltaCn |
| --- | --- | --- | --- | --- |
| R.AQAQPGVPLRELGQVVECSLDFGLVCR.N | 3 | 3000.386 | 4.297 | 0.54 |

+1 charge

```
		      B   	     Y
  1	 A 	    72.04	        -  	 27
  2	 Q 	   200.10	  2927.76	 26
  3	 A 	   271.14	  2799.70	 25
  4	 Q 	   399.20	  2728.66	 24
  5	 P 	   496.25	  2600.60	 23
  6	 G 	   553.27	  2503.55	 22
  7	 V 	   652.34	  2446.53	 21
  8	 P 	   749.39	  2347.46	 20
  9	 L 	   862.48	  2250.41	 19
 10	 R 	  1018.58	  2137.32	 18
 11	 E 	  1147.62	  1981.22	 17
 12	 L 	  1260.71	  1852.18	 16
 13	 G 	  1317.73	  1739.09	 15
 14	 Q 	  1445.79	  1682.07	 14
 15	 V 	  1544.85	  1554.01	 13
 16	 V 	  1643.92	  1454.95	 12
 17	 E 	  1772.97	  1355.88	 11
 18	 C 	  1933.13	  1226.83	 10
 19	 S 	  2020.16	  1066.67	  9
 20	 L 	  2133.25	   979.64	  8
 21	 D 	  2248.27	   866.55	  7
 22	 F 	  2395.34	   751.53	  6
 23	 G 	  2452.36	   604.46	  5
 24	 L 	  2565.45	   547.44	  4
 25	 V 	  2664.52	   434.35	  3
 26	 C 	  2824.68	   335.28	  2
 27	 R 	        -  	   175.12	  1  _


+2 charge
		      B   	     Y
  1	 A 	    36.53	        -  	 27
  2	 Q 	   100.56	  1464.38	 26
  3	 A 	   136.07	  1400.35	 25
  4	 Q 	   200.10	  1364.83	 24
  5	 P 	   248.63	  1300.80	 23
  6	 G 	   277.14	  1252.28	 22
  7	 V 	   326.67	  1223.77	 21
  8	 P 	   375.20	  1174.23	 20
  9	 L 	   431.74	  1125.71	 19
 10	 R 	   509.79	  1069.16	 18
 11	 E 	   574.31	   991.11	 17
 12	 L 	   630.86	   926.59	 16
 13	 G 	   659.37	   870.05	 15
 14	 Q 	   723.40	   841.54	 14
 15	 V 	   772.93	   777.51	 13
 16	 V 	   822.47	   727.98	 12
 17	 E 	   886.99	   678.44	 11
 18	 C 	   967.07	   613.92	 10
 19	 S 	  1010.58	   533.84	  9
 20	 L 	  1067.13	   490.32	  8
 21	 D 	  1124.64	   433.78	  7
 22	 F 	  1198.17	   376.27	  6
 23	 G 	  1226.69	   302.73	  5
 24	 L 	  1283.23	   274.22	  4
 25	 V 	  1332.76	   217.68	  3
 26	 C 	  1412.84	   168.15	  2
 27	 R 	        -  	    88.06	  1  _


+3 charge
		      B   	     Y
  1	 A 	    24.69	        -  	 27
  2	 Q 	    67.37	   976.59	 26
  3	 A 	    91.05	   933.90	 25
  4	 Q 	   133.74	   910.22	 24
  5	 P 	   166.09	   867.54	 23
  6	 G 	   185.10	   835.19	 22
  7	 V 	   218.12	   816.18	 21
  8	 P 	   250.47	   783.16	 20
  9	 L 	   288.16	   750.81	 19
 10	 R 	   340.20	   713.11	 18
 11	 E 	   383.21	   661.08	 17
 12	 L 	   420.91	   618.06	 16
 13	 G 	   439.91	   580.37	 15
 14	 Q 	   482.60	   561.36	 14
 15	 V 	   515.62	   518.68	 13
 16	 V 	   548.65	   485.65	 12
 17	 E 	   591.66	   452.63	 11
 18	 C 	   645.05	   409.62	 10
 19	 S 	   674.06	   356.23	  9
 20	 L 	   711.75	   327.22	  8
 21	 D 	   750.10	   289.52	  7
 22	 F 	   799.12	   251.18	  6
 23	 G 	   818.13	   202.16	  5
 24	 L 	   855.82	   183.15	  4
 25	 V 	   888.84	   145.46	  3
 26	 C 	   942.23	   112.43	  2
 27	 R 	        -  	    59.04	  1  _
```

**129. IPI00297252 Isoform 1 of Extracellular sulfatase Sulf-2**

| Sequence | Charge | M+H+ | XCorr | DeltaCn |
| --- | --- | --- | --- | --- |
| R.VYHVGLGDAAQPR.N | 2 | 1383.537 | 3.553 | 0.365 |

+1 charge

```
		      B   	     Y
  1	 V 	   100.08	        -  	 13
  2	 Y 	   263.14	  1283.65	 12
  3	 H 	   400.20	  1120.59	 11
  4	 V 	   499.27	   983.53	 10
  5	 G 	   556.29	   884.46	  9
  6	 L 	   669.37	   827.44	  8
  7	 G 	   726.39	   714.35	  7
  8	 D 	   841.42	   657.33	  6
  9	 A 	   912.46	   542.30	  5
 10	 A 	   983.49	   471.27	  4
 11	 Q 	  1111.55	   400.23	  3
 12	 P 	  1208.61	   272.17	  2
 13	 R 	        -  	   175.12	  1  _
+2 charge
		      B   	     Y
  1	 V 	    50.54	        -  	 13
  2	 Y 	   132.07	   642.33	 12
  3	 H 	   200.60	   560.80	 11
  4	 V 	   250.14	   492.27	 10
  5	 G 	   278.65	   442.73	  9
  6	 L 	   335.19	   414.22	  8
  7	 G 	   363.70	   357.68	  7
  8	 D 	   421.21	   329.17	  6
  9	 A 	   456.73	   271.66	  5
 10	 A 	   492.25	   236.14	  4
 11	 Q 	   556.28	   200.62	  3
 12	 P 	   604.81	   136.59	  2
 13	 R 	        -  	    88.06	  1  _
```

**130. IPI00216914 Vitelline membrane outer layer protein 1 homolog**

| Sequence | Charge | M+H+ | XCorr | DeltaCn |
| --- | --- | --- | --- | --- |
| R.VEAPTTLGDNTAANNVR.F | 2 | 1743.857 | 4.484 | 0.652 |

+1 charge

```
		      B   	     Y
  1	 V 	   100.08	        -  	 17
  2	 E 	   229.12	  1643.80	 16
  3	 A 	   300.16	  1514.76	 15
  4	 P 	   397.21	  1443.72	 14
  5	 T 	   498.26	  1346.67	 13
  6	 T 	   599.30	  1245.62	 12
  7	 L 	   712.39	  1144.57	 11
  8	 G 	   769.41	  1031.49	 10
  9	 D 	   884.44	   974.46	  9
 10	 N 	   998.48	   859.44	  8
 11	 T 	  1099.53	   745.40	  7
 12	 A 	  1170.56	   644.35	  6
 13	 A 	  1241.60	   573.31	  5
 14	 N 	  1355.64	   502.27	  4
 15	 N 	  1469.69	   388.23	  3
 16	 V 	  1568.76	   274.19	  2
 17	 R 	        -  	   175.12	  1  _
+2 charge
		      B   	     Y
  1	 V 	    50.54	        -  	 17
  2	 E 	   115.06	   822.40	 16
  3	 A 	   150.58	   757.88	 15
  4	 P 	   199.11	   722.36	 14
  5	 T 	   249.63	   673.84	 13
  6	 T 	   300.16	   623.31	 12
  7	 L 	   356.70	   572.79	 11
  8	 G 	   385.21	   516.25	 10
  9	 D 	   442.72	   487.74	  9
 10	 N 	   499.74	   430.22	  8
 11	 T 	   550.27	   373.20	  7
 12	 A 	   585.79	   322.68	  6
 13	 A 	   621.30	   287.16	  5
 14	 N 	   678.33	   251.64	  4
 15	 N 	   735.35	   194.62	  3
 16	 V 	   784.88	   137.60	  2
 17	 R 	        -  	    88.06	  1  _
```

**131. IPI00002280 ProSAAS**

| Sequence | Charge | M+H+ | XCorr | DeltaCn |
| --- | --- | --- | --- | --- |
| R.SVPRGEAAGAVQELAR.A | 2 | 1611.785 | 3.672 | 0.403 |

+1 charge

```
		      B   	     Y
  1	 S 	    88.04	        -  	 16
  2	 V 	   187.11	  1523.83	 15
  3	 P 	   284.16	  1424.76	 14
  4	 R 	   440.26	  1327.71	 13
  5	 G 	   497.28	  1171.61	 12
  6	 E 	   626.33	  1114.59	 11
  7	 A 	   697.36	   985.54	 10
  8	 A 	   768.40	   914.51	  9
  9	 G 	   825.42	   843.47	  8
 10	 A 	   896.46	   786.45	  7
 11	 V 	   995.53	   715.41	  6
 12	 Q 	  1123.59	   616.34	  5
 13	 E 	  1252.63	   488.28	  4
 14	 L 	  1365.71	   359.24	  3
 15	 A 	  1436.75	   246.16	  2
 16	 R 	        -  	   175.12	  1  _
+2 charge
		      B   	     Y
  1	 S 	    44.52	        -  	 16
  2	 V 	    94.06	   762.42	 15
  3	 P 	   142.58	   712.88	 14
  4	 R 	   220.63	   664.36	 13
  5	 G 	   249.15	   586.31	 12
  6	 E 	   313.67	   557.80	 11
  7	 A 	   349.19	   493.27	 10
  8	 A 	   384.70	   457.76	  9
  9	 G 	   413.21	   422.24	  8
 10	 A 	   448.73	   393.73	  7
 11	 V 	   498.27	   358.21	  6
 12	 Q 	   562.30	   308.67	  5
 13	 E 	   626.82	   244.64	  4
 14	 L 	   683.36	   180.12	  3
 15	 A 	   718.88	   123.58	  2
 16	 R 	        -  	    88.06	  1  _
```

**132. IPI00100796 Charged multivesicular body protein 5**

| Sequence | Charge | M+H+ | XCorr | DeltaCn |
| --- | --- | --- | --- | --- |
| K.AKPKAPPPSLTDCIGTVDSR.A | 3 | 2111.378 | 4.421 | 0.495 |

+1 charge

```
		      B   	     Y
  1	 A 	    72.04	        -  	 20
  2	 K 	   200.14	  2039.19	 19
  3	 P 	   297.19	  1911.10	 18
  4	 K 	   425.29	  1814.05	 17
  5	 A 	   496.32	  1685.95	 16
  6	 P 	   593.38	  1614.91	 15
  7	 P 	   690.43	  1517.86	 14
  8	 P 	   787.48	  1420.81	 13
  9	 S 	   874.51	  1323.76	 12
 10	 L 	   987.60	  1236.72	 11
 11	 T 	  1088.65	  1123.64	 10
 12	 D 	  1203.67	  1022.59	  9
 13	 C 	  1363.84	   907.56	  8
 14	 I 	  1476.92	   747.40	  7
 15	 G 	  1533.94	   634.32	  6
 16	 T 	  1634.99	   577.29	  5
 17	 V 	  1734.06	   476.25	  4
 18	 D 	  1849.09	   377.18	  3
 19	 S 	  1936.12	   262.15	  2
 20	 R 	        -  	   175.12	  1  _
+2 charge
		      B   	     Y
  1	 A 	    36.53	        -  	 20
  2	 K 	   100.57	  1020.10	 19
  3	 P 	   149.10	   956.05	 18
  4	 K 	   213.15	   907.53	 17
  5	 A 	   248.67	   843.48	 16
  6	 P 	   297.19	   807.96	 15
  7	 P 	   345.72	   759.43	 14
  8	 P 	   394.24	   710.91	 13
  9	 S 	   437.76	   662.38	 12
 10	 L 	   494.30	   618.87	 11
 11	 T 	   544.83	   562.32	 10
 12	 D 	   602.34	   511.80	  9
 13	 C 	   682.42	   454.29	  8
 14	 I 	   738.96	   374.20	  7
 15	 G 	   767.48	   317.66	  6
 16	 T 	   818.00	   289.15	  5
 17	 V 	   867.53	   238.63	  4
 18	 D 	   925.05	   189.09	  3
 19	 S 	   968.56	   131.58	  2
 20	 R 	        -  	    88.06	  1  _

+3 charge
		      B   	     Y
  1	 A 	    24.69	        -  	 20
  2	 K 	    67.38	   680.40	 19
  3	 P 	    99.74	   637.70	 18
  4	 K 	   142.43	   605.35	 17
  5	 A 	   166.11	   562.66	 16
  6	 P 	   198.46	   538.98	 15
  7	 P 	   230.81	   506.63	 14
  8	 P 	   263.17	   474.27	 13
  9	 S 	   292.18	   441.92	 12
 10	 L 	   329.87	   412.91	 11
 11	 T 	   363.55	   375.22	 10
 12	 D 	   401.90	   341.54	  9
 13	 C 	   455.28	   303.19	  8
 14	 I 	   492.98	   249.80	  7
 15	 G 	   511.99	   212.11	  6
 16	 T 	   545.67	   193.10	  5
 17	 V 	   578.69	   159.42	  4
 18	 D 	   617.03	   126.40	  3
 19	 S 	   646.04	    88.06	  2
 20	 R 	        -  	    59.04	  1  _
```

**133. IPI00027235 Isoform 1 of Attractin**

| Sequence | Charge | M+H+ | XCorr | DeltaCn |
| --- | --- | --- | --- | --- |
| R.SCALDQNCQWEPR.N | 2 | 1664.745 | 3.556 | 0.354 |

+1 charge

```
		      B   	     Y
  1	 S 	    88.04	        -  	 13
  2	 C 	   248.20	  1576.93	 12
  3	 A 	   319.24	  1416.77	 11
  4	 L 	   432.33	  1345.73	 10
  5	 D 	   547.35	  1232.65	  9
  6	 Q 	   675.41	  1117.62	  8
  7	 N 	   789.45	   989.56	  7
  8	 C 	   949.62	   875.52	  6
  9	 Q 	  1077.68	   715.35	  5
 10	 W 	  1263.76	   587.29	  4
 11	 E 	  1392.80	   401.21	  3
 12	 P 	  1489.85	   272.17	  2
 13	 R 	        -  	   175.12	  1  _
+2 charge
		      B   	     Y
  1	 S 	    44.52	        -  	 13
  2	 C 	   124.61	   788.97	 12
  3	 A 	   160.12	   708.89	 11
  4	 L 	   216.67	   673.37	 10
  5	 D 	   274.18	   616.83	  9
  6	 Q 	   338.21	   559.31	  8
  7	 N 	   395.23	   495.28	  7
  8	 C 	   475.31	   438.26	  6
  9	 Q 	   539.34	   358.18	  5
 10	 W 	   632.38	   294.15	  4
 11	 E 	   696.90	   201.11	  3
 12	 P 	   745.43	   136.59	  2
 13	 R 	        -  	    88.06	  1  _
```

**134. IPI00553215 V1-5 protein**

| Sequence | Charge | M+H+ | XCorr | DeltaCn |
| --- | --- | --- | --- | --- |
| R.VSWYQQPPGTAPK.L | 2 | 1459.63 | 3.652 | 0.43 |

+1 charge

```
		      B   	     Y
  1	 V 	   100.08	        -  	 13
  2	 S 	   187.11	  1359.67	 12
  3	 W 	   373.19	  1272.64	 11
  4	 Y 	   536.25	  1086.56	 10
  5	 Q 	   664.31	   923.49	  9
  6	 Q 	   792.37	   795.44	  8
  7	 P 	   889.42	   667.38	  7
  8	 P 	   986.47	   570.32	  6
  9	 G 	  1043.49	   473.27	  5
 10	 T 	  1144.54	   416.25	  4
 11	 A 	  1215.58	   315.20	  3
 12	 P 	  1312.63	   244.17	  2
 13	 K 	        -  	   147.11	  1  _
+2 charge
		      B   	     Y
  1	 V 	    50.54	        -  	 13
  2	 S 	    94.06	   680.34	 12
  3	 W 	   187.10	   636.82	 11
  4	 Y 	   268.63	   543.78	 10
  5	 Q 	   332.66	   462.25	  9
  6	 Q 	   396.69	   398.22	  8
  7	 P 	   445.21	   334.19	  7
  8	 P 	   493.74	   285.67	  6
  9	 G 	   522.25	   237.14	  5
 10	 T 	   572.77	   208.63	  4
 11	 A 	   608.29	   158.10	  3
 12	 P 	   656.82	   122.59	  2
 13	 K 	        -  	    74.06	  1  _
```

**135. IPI00082931 Small proline-rich protein 3**

| Sequence | Charge | M+H+ | XCorr | DeltaCn |
| --- | --- | --- | --- | --- |
| K.QTFTPPPQLQQQQVK.Q | 2 | 1768.994 | 3.479 | 0.444 |

+1 charge

```
		      B   	     Y
  1	 Q 	   129.07	        -  	 15
  2	 T 	   230.11	  1639.88	 14
  3	 F 	   377.18	  1538.83	 13
  4	 T 	   478.23	  1391.76	 12
  5	 P 	   575.28	  1290.72	 11
  6	 P 	   672.34	  1193.66	 10
  7	 P 	   769.39	  1096.61	  9
  8	 Q 	   897.45	   999.56	  8
  9	 L 	  1010.53	   871.50	  7
 10	 Q 	  1138.59	   758.42	  6
 11	 Q 	  1266.65	   630.36	  5
 12	 Q 	  1394.71	   502.30	  4
 13	 Q 	  1522.76	   374.24	  3
 14	 V 	  1621.83	   246.18	  2
 15	 K 	        -  	   147.11	  1  _
+2 charge
		      B   	     Y
  1	 Q 	    65.04	        -  	 15
  2	 T 	   115.56	   820.44	 14
  3	 F 	   189.09	   769.92	 13
  4	 T 	   239.62	   696.39	 12
  5	 P 	   288.14	   645.86	 11
  6	 P 	   336.67	   597.34	 10
  7	 P 	   385.20	   548.81	  9
  8	 Q 	   449.23	   500.28	  8
  9	 L 	   505.77	   436.25	  7
 10	 Q 	   569.80	   379.71	  6
 11	 Q 	   633.83	   315.68	  5
 12	 Q 	   697.86	   251.65	  4
 13	 Q 	   761.89	   187.62	  3
 14	 V 	   811.42	   123.59	  2
 15	 K 	        -  	    74.06	  1  _
```

**136. IPI00011140 Protein NOV homolog**

| Sequence | Charge | M+H+ | XCorr | DeltaCn |
| --- | --- | --- | --- | --- |
| R.AVLDGCSCCLVCAR.Q | 2 | 1641.823 | 4.236 | 0.512 |

+1 charge

```
		      B   	     Y
  1	 A 	    72.04	        -  	 14
  2	 V 	   171.11	  1570.20	 13
  3	 L 	   284.20	  1471.13	 12
  4	 D 	   399.22	  1358.05	 11
  5	 G 	   456.25	  1243.02	 10
  6	 C 	   616.41	  1186.00	  9
  7	 S 	   703.44	  1025.84	  8
  8	 C 	   863.61	   938.80	  7
  9	 C 	  1023.77	   778.64	  6
 10	 L 	  1136.86	   618.47	  5
 11	 V 	  1235.93	   505.39	  4
 12	 C 	  1396.09	   406.32	  3
 13	 A 	  1467.13	   246.16	  2
 14	 R 	        -  	   175.12	  1  _
+2 charge
		      B   	     Y
  1	 A 	    36.53	        -  	 14
  2	 V 	    86.06	   785.60	 13
  3	 L 	   142.60	   736.07	 12
  4	 D 	   200.12	   679.53	 11
  5	 G 	   228.63	   622.02	 10
  6	 C 	   308.71	   593.50	  9
  7	 S 	   352.22	   513.42	  8
  8	 C 	   432.31	   469.91	  7
  9	 C 	   512.39	   389.82	  6
 10	 L 	   568.93	   309.74	  5
 11	 V 	   618.47	   253.20	  4
 12	 C 	   698.55	   203.66	  3
 13	 A 	   734.07	   123.58	  2
 14	 R 	        -  	    88.06	  1  _
```

**137. IPI00003865 Isoform 1 of Heat shock cognate 71 kDa protein**

| Sequence | Charge | M+H+ | XCorr | DeltaCn |
| --- | --- | --- | --- | --- |
| R.IINEPTAAAIAYGLDKK.V | 2 | 1789.066 | 3.355 | 0.376 |

+1 charge

```
		      B   	     Y
  1	 I 	   114.09	        -  	 17
  2	 I 	   227.18	  1674.91	 16
  3	 N 	   341.22	  1561.82	 15
  4	 E 	   470.26	  1447.78	 14
  5	 P 	   567.31	  1318.74	 13
  6	 T 	   668.36	  1221.68	 12
  7	 A 	   739.40	  1120.64	 11
  8	 A 	   810.44	  1049.60	 10
  9	 A 	   881.47	   978.56	  9
 10	 I 	   994.56	   907.52	  8
 11	 A 	  1065.59	   794.44	  7
 12	 Y 	  1228.66	   723.40	  6
 13	 G 	  1285.68	   560.34	  5
 14	 L 	  1398.76	   503.32	  4
 15	 D 	  1513.79	   390.23	  3
 16	 K 	  1641.88	   275.21	  2
 17	 K 	        -  	   147.11	  1  _
+2 charge
		      B   	     Y
  1	 I 	    57.55	        -  	 17
  2	 I 	   114.09	   837.96	 16
  3	 N 	   171.11	   781.41	 15
  4	 E 	   235.63	   724.39	 14
  5	 P 	   284.16	   659.87	 13
  6	 T 	   334.68	   611.35	 12
  7	 A 	   370.20	   560.82	 11
  8	 A 	   405.72	   525.30	 10
  9	 A 	   441.24	   489.78	  9
 10	 I 	   497.78	   454.27	  8
 11	 A 	   533.30	   397.72	  7
 12	 Y 	   614.83	   362.21	  6
 13	 G 	   643.34	   280.67	  5
 14	 L 	   699.89	   252.16	  4
 15	 D 	   757.40	   195.62	  3
 16	 K 	   821.45	   138.11	  2
 17	 K 	        -  	    74.06	  1  _
```

**138. IPI00651692 Stromal cell-derived factor 1 theta**

| Sequence | Charge | M+H+ | XCorr | DeltaCn |
| --- | --- | --- | --- | --- |
| K.ILNTPNCALQIVAR.L | 2 | 1583.851 | 4.102 | 0.439 |

+1 charge

```
		      B   	     Y
  1	 I 	   114.09	        -  	 14
  2	 L 	   227.18	  1469.92	 13
  3	 N 	   341.22	  1356.84	 12
  4	 T 	   442.27	  1242.80	 11
  5	 P 	   539.32	  1141.75	 10
  6	 N 	   653.36	  1044.70	  9
  7	 C 	   813.53	   930.65	  8
  8	 A 	   884.56	   770.49	  7
  9	 L 	   997.65	   699.45	  6
 10	 Q 	  1125.71	   586.37	  5
 11	 I 	  1238.79	   458.31	  4
 12	 V 	  1337.86	   345.22	  3
 13	 A 	  1408.90	   246.16	  2
 14	 R 	        -  	   175.12	  1  _
+2 charge
		      B   	     Y
  1	 I 	    57.55	        -  	 14
  2	 L 	   114.09	   735.47	 13
  3	 N 	   171.11	   678.92	 12
  4	 T 	   221.64	   621.90	 11
  5	 P 	   270.16	   571.38	 10
  6	 N 	   327.18	   522.85	  9
  7	 C 	   407.27	   465.83	  8
  8	 A 	   442.79	   385.75	  7
  9	 L 	   499.33	   350.23	  6
 10	 Q 	   563.36	   293.69	  5
 11	 I 	   619.90	   229.66	  4
 12	 V 	   669.43	   173.12	  3
 13	 A 	   704.95	   123.58	  2
 14	 R 	        -  	    88.06	  1  _
```

**139. IPI00414542 Isoform 1 of Programmed cell death 1 ligand 2**

| Sequence | Charge | M+H+ | XCorr | DeltaCn |
| --- | --- | --- | --- | --- |
| R.ATLLEEQLPLGK.A | 2 | 1312.537 | 2.826 | 0.299 |

+1 charge

```
		      B   	     Y
  1	 A 	    72.04	        -  	 12
  2	 T 	   173.09	  1240.71	 11
  3	 L 	   286.18	  1139.67	 10
  4	 L 	   399.26	  1026.58	  9
  5	 E 	   528.30	   913.50	  8
  6	 E 	   657.35	   784.46	  7
  7	 Q 	   785.40	   655.41	  6
  8	 L 	   898.49	   527.36	  5
  9	 P 	   995.54	   414.27	  4
 10	 L 	  1108.62	   317.22	  3
 11	 G 	  1165.65	   204.13	  2
 12	 K 	        -  	   147.11	  1  _
+2 charge
		      B   	     Y
  1	 A 	    36.53	        -  	 12
  2	 T 	    87.05	   620.86	 11
  3	 L 	   143.59	   570.34	 10
  4	 L 	   200.13	   513.80	  9
  5	 E 	   264.66	   457.25	  8
  6	 E 	   329.18	   392.73	  7
  7	 Q 	   393.21	   328.21	  6
  8	 L 	   449.75	   264.18	  5
  9	 P 	   498.27	   207.64	  4
 10	 L 	   554.82	   159.11	  3
 11	 G 	   583.33	   102.57	  2
 12	 K 	        -  	    74.06	  1  _
```

**140. IPI00816799 Rheumatoid factor D5 light chain (Fragment)**

| Sequence | Charge | M+H+ | XCorr | DeltaCn |
| --- | --- | --- | --- | --- |
| - .EIVLTQSPATLSLSPGER.A | 2 | 1899.135 | 4.988 | 0.479 |

+1 charge

```
		      B   	     Y
  1	 E 	   130.05	        -  	 18
  2	 I 	   243.13	  1768.98	 17
  3	 V 	   342.20	  1655.90	 16
  4	 L 	   455.29	  1556.83	 15
  5	 T 	   556.33	  1443.74	 14
  6	 Q 	   684.39	  1342.70	 13
  7	 S 	   771.42	  1214.64	 12
  8	 P 	   868.48	  1127.61	 11
  9	 A 	   939.51	  1030.55	 10
 10	 T 	  1040.56	   959.52	  9
 11	 L 	  1153.65	   858.47	  8
 12	 S 	  1240.68	   745.38	  7
 13	 L 	  1353.76	   658.35	  6
 14	 S 	  1440.79	   545.27	  5
 15	 P 	  1537.85	   458.24	  4
 16	 G 	  1594.87	   361.18	  3
 17	 E 	  1723.91	   304.16	  2
 18	 R 	        -  	   175.12	  1  _
+2 charge
		      B   	     Y
  1	 E 	    65.53	        -  	 18
  2	 I 	   122.07	   884.99	 17
  3	 V 	   171.60	   828.45	 16
  4	 L 	   228.15	   778.92	 15
  5	 T 	   278.67	   722.38	 14
  6	 Q 	   342.70	   671.85	 13
  7	 S 	   386.22	   607.82	 12
  8	 P 	   434.74	   564.31	 11
  9	 A 	   470.26	   515.78	 10
 10	 T 	   520.78	   480.26	  9
 11	 L 	   577.33	   429.74	  8
 12	 S 	   620.84	   373.20	  7
 13	 L 	   677.38	   329.68	  6
 14	 S 	   720.90	   273.14	  5
 15	 P 	   769.43	   229.62	  4
 16	 G 	   797.94	   181.10	  3
 17	 E 	   862.46	   152.58	  2
 18	 R 	        -  	    88.06	  1  _
```

**141. IPI00018136 Isoform 1 of Vascular cell adhesion protein 1**

| Sequence | Charge | M+H+ | XCorr | DeltaCn |
| --- | --- | --- | --- | --- |
| K.SQEFLEDADRKSLETK.S | 2 | 1897.033 | 3.488 | 0.238 |

+1 charge

```
		      B   	     Y
  1	 S 	    88.04	        -  	 16
  2	 Q 	   216.10	  1808.90	 15
  3	 E 	   345.14	  1680.84	 14
  4	 F 	   492.21	  1551.80	 13
  5	 L 	   605.29	  1404.73	 12
  6	 E 	   734.34	  1291.65	 11
  7	 D 	   849.36	  1162.61	 10
  8	 A 	   920.40	  1047.58	  9
  9	 D 	  1035.43	   976.54	  8
 10	 R 	  1191.53	   861.52	  7
 11	 K 	  1319.62	   705.41	  6
 12	 S 	  1406.65	   577.32	  5
 13	 L 	  1519.74	   490.29	  4
 14	 E 	  1648.78	   377.20	  3
 15	 T 	  1749.83	   248.16	  2
 16	 K 	        -  	   147.11	  1  _
+2 charge
		      B   	     Y
  1	 S 	    44.52	        -  	 16
  2	 Q 	   108.55	   904.95	 15
  3	 E 	   173.07	   840.93	 14
  4	 F 	   246.61	   776.40	 13
  5	 L 	   303.15	   702.87	 12
  6	 E 	   367.67	   646.33	 11
  7	 D 	   425.18	   581.81	 10
  8	 A 	   460.70	   524.29	  9
  9	 D 	   518.22	   488.77	  8
 10	 R 	   596.27	   431.26	  7
 11	 K 	   660.31	   353.21	  6
 12	 S 	   703.83	   289.16	  5
 13	 L 	   760.37	   245.65	  4
 14	 E 	   824.89	   189.11	  3
 15	 T 	   875.42	   124.58	  2
 16	 K 	        -  	    74.06	  1  _
```

**142. IPI00783931 Isoform 1 of Collagen alpha-1(XVIII) chain**

| Sequence | Charge | M+H+ | XCorr | DeltaCn |
| --- | --- | --- | --- | --- |
| R.DFQPVLHLVALNSPLSGGMR.G | 2 | 2152.505 | 3.269 | 0.498 |

+1 charge

```
		      B   	     Y
  1	 D 	   116.03	        -  	 20
  2	 F 	   263.10	  2036.11	 19
  3	 Q 	   391.16	  1889.04	 18
  4	 P 	   488.21	  1760.98	 17
  5	 V 	   587.28	  1663.93	 16
  6	 L 	   700.37	  1564.86	 15
  7	 H 	   837.43	  1451.78	 14
  8	 L 	   950.51	  1314.72	 13
  9	 V 	  1049.58	  1201.64	 12
 10	 A 	  1120.61	  1102.57	 11
 11	 L 	  1233.70	  1031.53	 10
 12	 N 	  1347.74	   918.45	  9
 13	 S 	  1434.77	   804.40	  8
 14	 P 	  1531.83	   717.37	  7
 15	 L 	  1644.91	   620.32	  6
 16	 S 	  1731.94	   507.23	  5
 17	 G 	  1788.96	   420.20	  4
 18	 G 	  1845.99	   363.18	  3
 19	 M 	  1977.03	   306.16	  2
 20	 R 	        -  	   175.12	  1  _
+2 charge
		      B   	     Y
  1	 D 	    58.52	        -  	 20
  2	 F 	   132.05	  1018.56	 19
  3	 Q 	   196.08	   945.02	 18
  4	 P 	   244.61	   881.00	 17
  5	 V 	   294.14	   832.47	 16
  6	 L 	   350.69	   782.94	 15
  7	 H 	   419.22	   726.39	 14
  8	 L 	   475.76	   657.86	 13
  9	 V 	   525.29	   601.32	 12
 10	 A 	   560.81	   551.79	 11
 11	 L 	   617.35	   516.27	 10
 12	 N 	   674.37	   459.73	  9
 13	 S 	   717.89	   402.71	  8
 14	 P 	   766.42	   359.19	  7
 15	 L 	   822.96	   310.66	  6
 16	 S 	   866.48	   254.12	  5
 17	 G 	   894.99	   210.60	  4
 18	 G 	   923.50	   182.09	  3
 19	 M 	   989.02	   153.58	  2
 20	 R 	        -  	    88.06	  1  _
```

**143. IPI00073772 Fructose-1,6-bisphosphatase 1**

| Sequence | Charge | M+H+ | XCorr | DeltaCn |
| --- | --- | --- | --- | --- |
| R.APVILGSPDDVLEFLK.V | 2 | 1713.995 | 3.2 | 0.436 |

+1 charge

```
		      B   	     Y
  1	 A 	    72.04	        -  	 16
  2	 P 	   169.10	  1641.91	 15
  3	 V 	   268.17	  1544.86	 14
  4	 I 	   381.25	  1445.79	 13
  5	 L 	   494.33	  1332.70	 12
  6	 G 	   551.36	  1219.62	 11
  7	 S 	   638.39	  1162.60	 10
  8	 P 	   735.44	  1075.57	  9
  9	 D 	   850.47	   978.51	  8
 10	 D 	   965.49	   863.49	  7
 11	 V 	  1064.56	   748.46	  6
 12	 L 	  1177.65	   649.39	  5
 13	 E 	  1306.69	   536.31	  4
 14	 F 	  1453.76	   407.27	  3
 15	 L 	  1566.84	   260.20	  2
 16	 K 	        -  	   147.11	  1  _
+2 charge
		      B   	     Y
  1	 A 	    36.53	        -  	 16
  2	 P 	    85.05	   821.46	 15
  3	 V 	   134.59	   772.93	 14
  4	 I 	   191.13	   723.40	 13
  5	 L 	   247.67	   666.86	 12
  6	 G 	   276.18	   610.31	 11
  7	 S 	   319.70	   581.80	 10
  8	 P 	   368.22	   538.29	  9
  9	 D 	   425.74	   489.76	  8
 10	 D 	   483.25	   432.25	  7
 11	 V 	   532.78	   374.73	  6
 12	 L 	   589.33	   325.20	  5
 13	 E 	   653.85	   268.66	  4
 14	 F 	   727.38	   204.14	  3
 15	 L 	   783.92	   130.60	  2
 16	 K 	        -  	    74.06	  1  _
```

**144. IPI00303161 Endothelial cell-selective adhesion molecule**

| Sequence | Charge | M+H+ | XCorr | DeltaCn |
| --- | --- | --- | --- | --- |
| R.QLPSFQTFFAPALDVIR.G | 2 | 1951.257 | 3.125 | 0.364 |

+1 charge

```
		      B   	     Y
  1	 Q 	   129.07	        -  	 17
  2	 L 	   242.15	  1821.99	 16
  3	 P 	   339.20	  1708.91	 15
  4	 S 	   426.23	  1611.85	 14
  5	 F 	   573.30	  1524.82	 13
  6	 Q 	   701.36	  1377.75	 12
  7	 T 	   802.41	  1249.69	 11
  8	 F 	   949.48	  1148.65	 10
  9	 F 	  1096.55	  1001.58	  9
 10	 A 	  1167.58	   854.51	  8
 11	 P 	  1264.64	   783.47	  7
 12	 A 	  1335.67	   686.42	  6
 13	 L 	  1448.76	   615.38	  5
 14	 D 	  1563.78	   502.30	  4
 15	 V 	  1662.85	   387.27	  3
 16	 I 	  1775.94	   288.20	  2
 17	 R 	        -  	   175.12	  1  _
+2 charge
		      B   	     Y
  1	 Q 	    65.04	        -  	 17
  2	 L 	   121.58	   911.50	 16
  3	 P 	   170.10	   854.96	 15
  4	 S 	   213.62	   806.43	 14
  5	 F 	   287.16	   762.91	 13
  6	 Q 	   351.18	   689.38	 12
  7	 T 	   401.71	   625.35	 11
  8	 F 	   475.24	   574.83	 10
  9	 F 	   548.78	   501.29	  9
 10	 A 	   584.30	   427.76	  8
 11	 P 	   632.82	   392.24	  7
 12	 A 	   668.34	   343.71	  6
 13	 L 	   724.88	   308.19	  5
 14	 D 	   782.40	   251.65	  4
 15	 V 	   831.93	   194.14	  3
 16	 I 	   888.47	   144.61	  2
 17	 R 	        -  	    88.06	  1  _
```

**145. IPI00604773 PODXL protein**

| Sequence | Charge | M+H+ | XCorr | DeltaCn |
| --- | --- | --- | --- | --- |
| K.ATFNPAQDKCGIR.L | 2 | 1478.629 | 2.843 | 0.418 |

+1 charge

```
		      B   	     Y
  1	 A 	    72.04	        -  	 13
  2	 T 	   173.09	  1406.82	 12
  3	 F 	   320.16	  1305.77	 11
  4	 N 	   434.20	  1158.70	 10
  5	 P 	   531.26	  1044.66	  9
  6	 A 	   602.29	   947.61	  8
  7	 Q 	   730.35	   876.57	  7
  8	 D 	   845.38	   748.51	  6
  9	 K 	   973.47	   633.48	  5
 10	 C 	  1133.64	   505.39	  4
 11	 G 	  1190.66	   345.22	  3
 12	 I 	  1303.74	   288.20	  2
 13	 R 	        -  	   175.12	  1  _
+2 charge
		      B   	     Y
  1	 A 	    36.53	        -  	 13
  2	 T 	    87.05	   703.91	 12
  3	 F 	   160.58	   653.39	 11
  4	 N 	   217.61	   579.86	 10
  5	 P 	   266.13	   522.83	  9
  6	 A 	   301.65	   474.31	  8
  7	 Q 	   365.68	   438.79	  7
  8	 D 	   423.19	   374.76	  6
  9	 K 	   487.24	   317.25	  5
 10	 C 	   567.32	   253.20	  4
 11	 G 	   595.83	   173.12	  3
 12	 I 	   652.38	   144.61	  2
 13	 R 	        -  	    88.06	  1  _
```

**146.IPI00003590 Isoform 1 of Sulfhydryl oxidase 1**

| Sequence | Charge | M+H+ | XCorr | DeltaCn |
| --- | --- | --- | --- | --- |
| R.AAPGQEPPEHMAELQR.N | 2 | 1761.941 | 3.524 | 0.488 |

+1 charge

```
		      B   	     Y
  1	 A 	    72.04	        -  	 16
  2	 A 	   143.08	  1689.80	 15
  3	 P 	   240.13	  1618.76	 14
  4	 G 	   297.16	  1521.71	 13
  5	 Q 	   425.21	  1464.69	 12
  6	 E 	   554.26	  1336.63	 11
  7	 P 	   651.31	  1207.59	 10
  8	 P 	   748.36	  1110.54	  9
  9	 E 	   877.41	  1013.48	  8
 10	 H 	  1014.46	   884.44	  7
 11	 M 	  1145.50	   747.38	  6
 12	 A 	  1216.54	   616.34	  5
 13	 E 	  1345.58	   545.30	  4
 14	 L 	  1458.67	   416.26	  3
 15	 Q 	  1586.73	   303.18	  2
 16	 R 	        -  	   175.12	  1  _
+2 charge
		      B   	     Y
  1	 A 	    36.53	        -  	 16
  2	 A 	    72.04	   845.40	 15
  3	 P 	   120.57	   809.89	 14
  4	 G 	   149.08	   761.36	 13
  5	 Q 	   213.11	   732.85	 12
  6	 E 	   277.63	   668.82	 11
  7	 P 	   326.16	   604.30	 10
  8	 P 	   374.68	   555.77	  9
  9	 E 	   439.21	   507.25	  8
 10	 H 	   507.74	   442.72	  7
 11	 M 	   573.26	   374.19	  6
 12	 A 	   608.77	   308.67	  5
 13	 E 	   673.30	   273.16	  4
 14	 L 	   729.84	   208.63	  3
 15	 Q 	   793.87	   152.09	  2
 16	 R 	        -  	    88.06	  1  _
```

**147. IPI00220642 14-3-3 protein gamma**

| Sequence | Charge | M+H+ | XCorr | DeltaCn |
| --- | --- | --- | --- | --- |
| K.LAEQAERYDDMAAAMK.A | 2 | 1814.034 | 3.536 | 0.432 |

+1 charge

```
		      B   	     Y
  1	 L 	   114.09	        -  	 16
  2	 A 	   185.13	  1699.74	 15
  3	 E 	   314.17	  1628.70	 14
  4	 Q 	   442.23	  1499.66	 13
  5	 A 	   513.27	  1371.60	 12
  6	 E 	   642.31	  1300.57	 11
  7	 R 	   798.41	  1171.52	 10
  8	 Y 	   961.47	  1015.42	  9
  9	 D 	  1076.50	   852.36	  8
 10	 D 	  1191.53	   737.33	  7
 11	 M 	  1322.57	   622.31	  6
 12	 A 	  1393.61	   491.26	  5
 13	 A 	  1464.64	   420.23	  4
 14	 A 	  1535.68	   349.19	  3
 15	 M 	  1666.72	   278.15	  2
 16	 K 	        -  	   147.11	  1  _
+2 charge
		      B   	     Y
  1	 L 	    57.55	        -  	 16
  2	 A 	    93.07	   850.37	 15
  3	 E 	   157.59	   814.86	 14
  4	 Q 	   221.62	   750.33	 13
  5	 A 	   257.14	   686.31	 12
  6	 E 	   321.66	   650.79	 11
  7	 R 	   399.71	   586.27	 10
  8	 Y 	   481.24	   508.21	  9
  9	 D 	   538.75	   426.68	  8
 10	 D 	   596.27	   369.17	  7
 11	 M 	   661.79	   311.66	  6
 12	 A 	   697.31	   246.14	  5
 13	 A 	   732.82	   210.62	  4
 14	 A 	   768.34	   175.10	  3
 15	 M 	   833.86	   139.58	  2
 16	 K 	        -  	    74.06	  1  _
```

**148. IPI00022284 Major prion protein**

| Sequence | Charge | M+H+ | XCorr | DeltaCn |
| --- | --- | --- | --- | --- |
| R.VVEQMCITQYER.E | 2 | 1556.76 | 4.15 | 0.554 |

+1 charge

```
		      B   	     Y
  1	 V 	   100.08	        -  	 12
  2	 V 	   199.14	  1456.79	 11
  3	 E 	   328.19	  1357.72	 10
  4	 Q 	   456.25	  1228.68	  9
  5	 M 	   587.29	  1100.62	  8
  6	 C 	   747.45	   969.58	  7
  7	 I 	   860.54	   809.42	  6
  8	 T 	   961.58	   696.33	  5
  9	 Q 	  1089.64	   595.28	  4
 10	 Y 	  1252.70	   467.22	  3
 11	 E 	  1381.75	   304.16	  2
 12	 R 	        -  	   175.12	  1  _
+2 charge
		      B   	     Y
  1	 V 	    50.54	        -  	 12
  2	 V 	   100.08	   728.90	 11
  3	 E 	   164.60	   679.36	 10
  4	 Q 	   228.63	   614.84	  9
  5	 M 	   294.15	   550.81	  8
  6	 C 	   374.23	   485.29	  7
  7	 I 	   430.77	   405.21	  6
  8	 T 	   481.30	   348.67	  5
  9	 Q 	   545.32	   298.15	  4
 10	 Y 	   626.86	   234.12	  3
 11	 E 	   691.38	   152.58	  2
 12	 R 	        -  	    88.06	  1  _
```

**149. IPI00829834 Ig kappa chain V-III region VH (Fragment)**

| Sequence | Charge | M+H+ | XCorr | DeltaCn |
| --- | --- | --- | --- | --- |
| R.EIVMTQSPPTLSLSPGER.V | 2 | 1943.211 | 3.522 | 0.295 |

+1 charge

```
		      B   	     Y
  1	 E 	   130.05	        -  	 18
  2	 I 	   243.13	  1812.95	 17
  3	 V 	   342.20	  1699.87	 16
  4	 M 	   473.24	  1600.80	 15
  5	 T 	   574.29	  1469.76	 14
  6	 Q 	   702.35	  1368.71	 13
  7	 S 	   789.38	  1240.65	 12
  8	 P 	   886.43	  1153.62	 11
  9	 P 	   983.49	  1056.57	 10
 10	 T 	  1084.53	   959.52	  9
 11	 L 	  1197.62	   858.47	  8
 12	 S 	  1284.65	   745.38	  7
 13	 L 	  1397.73	   658.35	  6
 14	 S 	  1484.77	   545.27	  5
 15	 P 	  1581.82	   458.24	  4
 16	 G 	  1638.84	   361.18	  3
 17	 E 	  1767.88	   304.16	  2
 18	 R 	        -  	   175.12	  1  _
+2 charge
		      B   	     Y
  1	 E 	    65.53	        -  	 18
  2	 I 	   122.07	   906.98	 17
  3	 V 	   171.60	   850.44	 16
  4	 M 	   237.13	   800.90	 15
  5	 T 	   287.65	   735.38	 14
  6	 Q 	   351.68	   684.86	 13
  7	 S 	   395.19	   620.83	 12
  8	 P 	   443.72	   577.31	 11
  9	 P 	   492.25	   528.79	 10
 10	 T 	   542.77	   480.26	  9
 11	 L 	   599.31	   429.74	  8
 12	 S 	   642.83	   373.20	  7
 13	 L 	   699.37	   329.68	  6
 14	 S 	   742.89	   273.14	  5
 15	 P 	   791.41	   229.62	  4
 16	 G 	   819.92	   181.10	  3
 17	 E 	   884.45	   152.58	  2
 18	 R 	        -  	    88.06	  1  _
```

**150. IPI00385252 Ig kappa chain V-III region GOL**

| Sequence | Charge | M+H+ | XCorr | DeltaCn |
| --- | --- | --- | --- | --- |
| - .EIVLTQSPGTLSLSPGER.A | 2 | 1885.108 | 5.42 | 0.559 |

+1 charge

```
		      B   	     Y
  1	 E 	   130.05	        -  	 18
  2	 I 	   243.13	  1754.96	 17
  3	 V 	   342.20	  1641.88	 16
  4	 L 	   455.29	  1542.81	 15
  5	 T 	   556.33	  1429.73	 14
  6	 Q 	   684.39	  1328.68	 13
  7	 S 	   771.42	  1200.62	 12
  8	 P 	   868.48	  1113.59	 11
  9	 G 	   925.50	  1016.54	 10
 10	 T 	  1026.55	   959.52	  9
 11	 L 	  1139.63	   858.47	  8
 12	 S 	  1226.66	   745.38	  7
 13	 L 	  1339.75	   658.35	  6
 14	 S 	  1426.78	   545.27	  5
 15	 P 	  1523.83	   458.24	  4
 16	 G 	  1580.85	   361.18	  3
 17	 E 	  1709.90	   304.16	  2
 18	 R 	        -  	   175.12	  1  _
+2 charge
		      B   	     Y
  1	 E 	    65.53	        -  	 18
  2	 I 	   122.07	   877.99	 17
  3	 V 	   171.60	   821.44	 16
  4	 L 	   228.15	   771.91	 15
  5	 T 	   278.67	   715.37	 14
  6	 Q 	   342.70	   664.84	 13
  7	 S 	   386.22	   600.81	 12
  8	 P 	   434.74	   557.30	 11
  9	 G 	   463.25	   508.77	 10
 10	 T 	   513.78	   480.26	  9
 11	 L 	   570.32	   429.74	  8
 12	 S 	   613.83	   373.20	  7
 13	 L 	   670.38	   329.68	  6
 14	 S 	   713.89	   273.14	  5
 15	 P 	   762.42	   229.62	  4
 16	 G 	   790.93	   181.10	  3
 17	 E 	   855.45	   152.58	  2
 18	 R 	        -  	    88.06	  1  _
```

**151. IPI00009901 Nuclear transport factor 2**

| Sequence | Charge | M+H+ | XCorr | DeltaCn |
| --- | --- | --- | --- | --- |
| K.AAIVEKLSSLPFQK.I | 2 | 1531.821 | 3.661 | 0.401 |

+1 charge

```
		      B   	     Y
  1	 A 	    72.04	        -  	 14
  2	 A 	   143.08	  1459.85	 13
  3	 I 	   256.17	  1388.81	 12
  4	 V 	   355.23	  1275.73	 11
  5	 E 	   484.28	  1176.66	 10
  6	 K 	   612.37	  1047.62	  9
  7	 L 	   725.46	   919.52	  8
  8	 S 	   812.49	   806.44	  7
  9	 S 	   899.52	   719.41	  6
 10	 L 	  1012.60	   632.38	  5
 11	 P 	  1109.66	   519.29	  4
 12	 F 	  1256.72	   422.24	  3
 13	 Q 	  1384.78	   275.17	  2
 14	 K 	        -  	   147.11	  1  _
+2 charge
		      B   	     Y
  1	 A 	    36.53	        -  	 14
  2	 A 	    72.04	   730.43	 13
  3	 I 	   128.59	   694.91	 12
  4	 V 	   178.12	   638.37	 11
  5	 E 	   242.64	   588.83	 10
  6	 K 	   306.69	   524.31	  9
  7	 L 	   363.23	   460.27	  8
  8	 S 	   406.75	   403.72	  7
  9	 S 	   450.26	   360.21	  6
 10	 L 	   506.81	   316.69	  5
 11	 P 	   555.33	   260.15	  4
 12	 F 	   628.87	   211.62	  3
 13	 Q 	   692.90	   138.09	  2
 14	 K 	        -  	    74.06	  1  _
```

**152. IPI00169383 Phosphoglycerate kinase 1**

| Sequence | Charge | M+H+ | XCorr | DeltaCn |
| --- | --- | --- | --- | --- |
| K.SLLGKDVLFLKDCVGPEVEK.A | 3 | 2247.61 | 4.244 | 0.426 |

+1 charge

```
		      B   	     Y
  1	 S 	    88.04	        -  	 20
  2	 L 	   201.12	  2159.31	 19
  3	 L 	   314.21	  2046.23	 18
  4	 G 	   371.23	  1933.14	 17
  5	 K 	   499.32	  1876.12	 16
  6	 D 	   614.35	  1748.03	 15
  7	 V 	   713.42	  1633.00	 14
  8	 L 	   826.50	  1533.93	 13
  9	 F 	   973.57	  1420.85	 12
 10	 L 	  1086.66	  1273.78	 11
 11	 K 	  1214.75	  1160.70	 10
 12	 D 	  1329.78	  1032.60	  9
 13	 C 	  1489.94	   917.57	  8
 14	 V 	  1589.01	   757.41	  7
 15	 G 	  1646.03	   658.34	  6
 16	 P 	  1743.09	   601.32	  5
 17	 E 	  1872.13	   504.27	  4
 18	 V 	  1971.20	   375.22	  3
 19	 E 	  2100.24	   276.16	  2
 20	 K 	        -  	   147.11	  1  _
+2 charge
		      B   	     Y
  1	 S 	    44.52	        -  	 20
  2	 L 	   101.07	  1080.16	 19
  3	 L 	   157.61	  1023.62	 18
  4	 G 	   186.12	   967.08	 17
  5	 K 	   250.17	   938.57	 16
  6	 D 	   307.68	   874.52	 15
  7	 V 	   357.21	   817.00	 14
  8	 L 	   413.76	   767.47	 13
  9	 F 	   487.29	   710.93	 12
 10	 L 	   543.83	   637.39	 11
 11	 K 	   607.88	   580.85	 10
 12	 D 	   665.39	   516.80	  9
 13	 C 	   745.48	   459.29	  8
 14	 V 	   795.01	   379.21	  7
 15	 G 	   823.52	   329.67	  6
 16	 P 	   872.05	   301.16	  5
 17	 E 	   936.57	   252.64	  4
 18	 V 	   986.10	   188.12	  3
 19	 E 	  1050.62	   138.58	  2
 20	 K 	        -  	    74.06	  1  _


+3 charge
		      B   	     Y
  1	 S 	    30.02	        -  	 20
  2	 L 	    67.71	   720.44	 19
  3	 L 	   105.41	   682.75	 18
  4	 G 	   124.41	   645.05	 17
  5	 K 	   167.11	   626.05	 16
  6	 D 	   205.46	   583.35	 15
  7	 V 	   238.48	   545.01	 14
  8	 L 	   276.17	   511.98	 13
  9	 F 	   325.20	   474.29	 12
 10	 L 	   362.89	   425.26	 11
 11	 K 	   405.59	   387.57	 10
 12	 D 	   443.93	   344.87	  9
 13	 C 	   497.32	   306.53	  8
 14	 V 	   530.34	   253.14	  7
 15	 G 	   549.35	   220.12	  6
 16	 P 	   581.70	   201.11	  5
 17	 E 	   624.71	   168.76	  4
 18	 V 	   657.74	   125.75	  3
 19	 E 	   700.75	    92.72	  2
 20	 K 	        -  	    49.71	  1  _
```

**153. IPI00736885 Ig kappa chain V-II region TEW**

| Sequence | Charge | M+H+ | XCorr | DeltaCn |
| --- | --- | --- | --- | --- |
| - .DIVMTQSPLSLPVTPGEPASISCR.S | 3 | 2556.911 | 5.881 | 0.505 |

+1 charge

```
		      B   	     Y
  1	 D 	   116.03	        -  	 24
  2	 I 	   229.12	  2440.39	 23
  3	 V 	   328.19	  2327.31	 22
  4	 M 	   459.23	  2228.24	 21
  5	 T 	   560.27	  2097.20	 20
  6	 Q 	   688.33	  1996.15	 19
  7	 S 	   775.37	  1868.09	 18
  8	 P 	   872.42	  1781.06	 17
  9	 L 	   985.50	  1684.01	 16
 10	 S 	  1072.53	  1570.92	 15
 11	 L 	  1185.62	  1483.89	 14
 12	 P 	  1282.67	  1370.81	 13
 13	 V 	  1381.74	  1273.76	 12
 14	 T 	  1482.79	  1174.69	 11
 15	 P 	  1579.84	  1073.64	 10
 16	 G 	  1636.86	   976.59	  9
 17	 E 	  1765.90	   919.56	  8
 18	 P 	  1862.96	   790.52	  7
 19	 A 	  1933.99	   693.47	  6
 20	 S 	  2021.03	   622.43	  5
 21	 I 	  2134.11	   535.40	  4
 22	 S 	  2221.14	   422.32	  3
 23	 C 	  2381.31	   335.28	  2
 24	 R 	        -  	   175.12	  1  _


+2 charge
		      B   	     Y
  1	 D 	    58.52	        -  	 24
  2	 I 	   115.06	  1220.70	 23
  3	 V 	   164.60	  1164.16	 22
  4	 M 	   230.12	  1114.62	 21
  5	 T 	   280.64	  1049.10	 20
  6	 Q 	   344.67	   998.58	 19
  7	 S 	   388.19	   934.55	 18
  8	 P 	   436.71	   891.03	 17
  9	 L 	   493.25	   842.51	 16
 10	 S 	   536.77	   785.97	 15
 11	 L 	   593.31	   742.45	 14
 12	 P 	   641.84	   685.91	 13
 13	 V 	   691.37	   637.38	 12
 14	 T 	   741.90	   587.85	 11
 15	 P 	   790.42	   537.32	 10
 16	 G 	   818.93	   488.80	  9
 17	 E 	   883.46	   460.29	  8
 18	 P 	   931.98	   395.76	  7
 19	 A 	   967.50	   347.24	  6
 20	 S 	  1011.02	   311.72	  5
 21	 I 	  1067.56	   268.20	  4
 22	 S 	  1111.07	   211.66	  3
 23	 C 	  1191.16	   168.15	  2
 24	 R 	        -  	    88.06	  1  _
+3 charge
		      B   	     Y
  1	 D 	    39.35	        -  	 24
  2	 I 	    77.04	   814.14	 23
  3	 V 	   110.07	   776.44	 22
  4	 M 	   153.75	   743.42	 21
  5	 T 	   187.43	   699.74	 20
  6	 Q 	   230.12	   666.06	 19
  7	 S 	   259.13	   623.37	 18
  8	 P 	   291.48	   594.36	 17
  9	 L 	   329.17	   562.01	 16
 10	 S 	   358.18	   524.31	 15
 11	 L 	   395.88	   495.30	 14
 12	 P 	   428.23	   457.61	 13
 13	 V 	   461.25	   425.26	 12
 14	 T 	   494.93	   392.23	 11
 15	 P 	   527.28	   358.55	 10
 16	 G 	   546.29	   326.20	  9
 17	 E 	   589.31	   307.19	  8
 18	 P 	   621.66	   264.18	  7
 19	 A 	   645.34	   231.83	  6
 20	 S 	   674.35	   208.15	  5
 21	 I 	   712.04	   179.14	  4
 22	 S 	   741.05	   141.44	  3
 23	 C 	   794.44	   112.43	  2
 24	 R 	        -  	    59.04	  1  _
```

**154. IPI00291006 Malate dehydrogenase, mitochondrial**

| Sequence | Charge | M+H+ | XCorr | DeltaCn |
| --- | --- | --- | --- | --- |
| R.LTLYDIAHTPGVAADLSHIETK.A | 3 | 2366.655 | 3.939 | 0.406 |

+1 charge

```
		      B   	     Y
  1	 L 	   114.09	        -  	 22
  2	 T 	   215.14	  2252.16	 21
  3	 L 	   328.22	  2151.11	 20
  4	 Y 	   491.29	  2038.02	 19
  5	 D 	   606.31	  1874.96	 18
  6	 I 	   719.40	  1759.93	 17
  7	 A 	   790.43	  1646.85	 16
  8	 H 	   927.49	  1575.81	 15
  9	 T 	  1028.54	  1438.75	 14
 10	 P 	  1125.59	  1337.71	 13
 11	 G 	  1182.62	  1240.65	 12
 12	 V 	  1281.68	  1183.63	 11
 13	 A 	  1352.72	  1084.56	 10
 14	 A 	  1423.76	  1013.53	  9
 15	 D 	  1538.78	   942.49	  8
 16	 L 	  1651.87	   827.46	  7
 17	 S 	  1738.90	   714.38	  6
 18	 H 	  1875.96	   627.35	  5
 19	 I 	  1989.04	   490.29	  4
 20	 E 	  2118.09	   377.20	  3
 21	 T 	  2219.13	   248.16	  2
 22	 K 	        -  	   147.11	  1  _
+2 charge
		      B   	     Y
  1	 L 	    57.55	        -  	 22
  2	 T 	   108.07	  1126.58	 21
  3	 L 	   164.62	  1076.06	 20
  4	 Y 	   246.15	  1019.52	 19
  5	 D 	   303.66	   937.98	 18
  6	 I 	   360.20	   880.47	 17
  7	 A 	   395.72	   823.93	 16
  8	 H 	   464.25	   788.41	 15
  9	 T 	   514.77	   719.88	 14
 10	 P 	   563.30	   669.36	 13
 11	 G 	   591.81	   620.83	 12
 12	 V 	   641.35	   592.32	 11
 13	 A 	   676.86	   542.79	 10
 14	 A 	   712.38	   507.27	  9
 15	 D 	   769.90	   471.75	  8
 16	 L 	   826.44	   414.23	  7
 17	 S 	   869.95	   357.69	  6
 18	 H 	   938.48	   314.18	  5
 19	 I 	   995.03	   245.65	  4
 20	 E 	  1059.55	   189.11	  3
 21	 T 	  1110.07	   124.58	  2
 22	 K 	        -  	    74.06	  1  _
+3 charge
		      B   	     Y
  1	 L 	    38.70	        -  	 22
  2	 T 	    72.38	   751.39	 21
  3	 L 	   110.08	   717.71	 20
  4	 Y 	   164.43	   680.01	 19
  5	 D 	   202.78	   625.66	 18
  6	 I 	   240.47	   587.32	 17
  7	 A 	   264.15	   549.62	 16
  8	 H 	   309.84	   525.94	 15
  9	 T 	   343.52	   480.26	 14
 10	 P 	   375.87	   446.57	 13
 11	 G 	   394.88	   414.22	 12
 12	 V 	   427.90	   395.22	 11
 13	 A 	   451.58	   362.19	 10
 14	 A 	   475.26	   338.51	  9
 15	 D 	   513.60	   314.83	  8
 16	 L 	   551.29	   276.49	  7
 17	 S 	   580.31	   238.80	  6
 18	 H 	   625.99	   209.79	  5
 19	 I 	   663.69	   164.10	  4
 20	 E 	   706.70	   126.41	  3
 21	 T 	   740.38	    83.39	  2
 22	 K 	        -  	    49.71	  1  _
```

**155. IPI00451401 Isoform 2 of Triosephosphate isomerase**

| Sequence | Charge | M+H+ | XCorr | DeltaCn |
| --- | --- | --- | --- | --- |
| K.VVLAYEPVWAIGTGK.T | 2 | 1603.886 | 4.287 | 0.482 |

+1 charge

```
		      B   	     Y
  1	 V 	   100.08	        -  	 15
  2	 V 	   199.14	  1503.82	 14
  3	 L 	   312.23	  1404.75	 13
  4	 A 	   383.27	  1291.67	 12
  5	 Y 	   546.33	  1220.63	 11
  6	 E 	   675.37	  1057.57	 10
  7	 P 	   772.42	   928.53	  9
  8	 V 	   871.49	   831.47	  8
  9	 W 	  1057.57	   732.40	  7
 10	 A 	  1128.61	   546.32	  6
 11	 I 	  1241.69	   475.29	  5
 12	 G 	  1298.71	   362.20	  4
 13	 T 	  1399.76	   305.18	  3
 14	 G 	  1456.78	   204.13	  2
 15	 K 	        -  	   147.11	  1  _
+2 charge
		      B   	     Y
  1	 V 	    50.54	        -  	 15
  2	 V 	   100.08	   752.41	 14
  3	 L 	   156.62	   702.88	 13
  4	 A 	   192.14	   646.34	 12
  5	 Y 	   273.67	   610.82	 11
  6	 E 	   338.19	   529.29	 10
  7	 P 	   386.72	   464.77	  9
  8	 V 	   436.25	   416.24	  8
  9	 W 	   529.29	   366.71	  7
 10	 A 	   564.81	   273.67	  6
 11	 I 	   621.35	   238.15	  5
 12	 G 	   649.86	   181.61	  4
 13	 T 	   700.38	   153.09	  3
 14	 G 	   728.90	   102.57	  2
 15	 K 	        -  	    74.06	  1  _
```

**156. IPI00006547 T-cell immunomodulatory protein**

| Sequence | Charge | M+H+ | XCorr | DeltaCn |
| --- | --- | --- | --- | --- |
| R.ERNDLIVFLADQNAPYFKPK.V | 3 | 2379.699 | 3.397 | 0.432 |

+1 charge

```
		      B   	     Y
  1	 E 	   130.05	        -  	 20
  2	 R 	   286.15	  2249.21	 19
  3	 N 	   400.19	  2093.11	 18
  4	 D 	   515.22	  1979.06	 17
  5	 L 	   628.30	  1864.04	 16
  6	 I 	   741.39	  1750.95	 15
  7	 V 	   840.46	  1637.87	 14
  8	 F 	   987.53	  1538.80	 13
  9	 L 	  1100.61	  1391.73	 12
 10	 A 	  1171.65	  1278.65	 11
 11	 D 	  1286.67	  1207.61	 10
 12	 Q 	  1414.73	  1092.58	  9
 13	 N 	  1528.78	   964.53	  8
 14	 A 	  1599.81	   850.48	  7
 15	 P 	  1696.87	   779.45	  6
 16	 Y 	  1859.93	   682.39	  5
 17	 F 	  2007.00	   519.33	  4
 18	 K 	  2135.09	   372.26	  3
 19	 P 	  2232.14	   244.17	  2
 20	 K 	        -  	   147.11	  1  _
+2 charge
		      B   	     Y
  1	 E 	    65.53	        -  	 20
  2	 R 	   143.58	  1125.11	 19
  3	 N 	   200.60	  1047.06	 18
  4	 D 	   258.11	   990.04	 17
  5	 L 	   314.66	   932.52	 16
  6	 I 	   371.20	   875.98	 15
  7	 V 	   420.73	   819.44	 14
  8	 F 	   494.27	   769.90	 13
  9	 L 	   550.81	   696.37	 12
 10	 A 	   586.33	   639.83	 11
 11	 D 	   643.84	   604.31	 10
 12	 Q 	   707.87	   546.80	  9
 13	 N 	   764.89	   482.77	  8
 14	 A 	   800.41	   425.74	  7
 15	 P 	   848.94	   390.23	  6
 16	 Y 	   930.47	   341.70	  5
 17	 F 	  1004.00	   260.17	  4
 18	 K 	  1068.05	   186.63	  3
 19	 P 	  1116.58	   122.59	  2
 20	 K 	        -  	    74.06	  1  _


+3 charge
		      B   	     Y
  1	 E 	    44.02	        -  	 20
  2	 R 	    96.06	   750.41	 19
  3	 N 	   134.07	   698.37	 18
  4	 D 	   172.41	   660.36	 17
  5	 L 	   210.11	   622.02	 16
  6	 I 	   247.80	   584.32	 15
  7	 V 	   280.82	   546.63	 14
  8	 F 	   329.85	   513.60	 13
  9	 L 	   367.54	   464.58	 12
 10	 A 	   391.22	   426.89	 11
 11	 D 	   429.56	   403.21	 10
 12	 Q 	   472.25	   364.87	  9
 13	 N 	   510.26	   322.18	  8
 14	 A 	   533.94	   284.17	  7
 15	 P 	   566.29	   260.49	  6
 16	 Y 	   620.65	   228.14	  5
 17	 F 	   669.67	   173.78	  4
 18	 K 	   712.37	   124.76	  3
 19	 P 	   744.72	    82.06	  2
 20	 K 	        -  	    49.71	  1  _
```

**157. IPI00480159 Isoform 1 of Phosphatidylinositide phosphatase SAC2**

| Sequence | Charge | M+H+ | XCorr | DeltaCn |
| --- | --- | --- | --- | --- |
| R.IYQIMWANNGDSISR.Q | 2 | 1768.975 | 3.04 | 0.281 |

+1 charge

```
		      B   	     Y
  1	 I 	   114.09	        -  	 15
  2	 Y 	   277.15	  1654.76	 14
  3	 Q 	   405.21	  1491.70	 13
  4	 I 	   518.30	  1363.64	 12
  5	 M 	   649.34	  1250.56	 11
  6	 W 	   835.42	  1119.52	 10
  7	 A 	   906.45	   933.44	  9
  8	 N 	  1020.50	   862.40	  8
  9	 N 	  1134.54	   748.36	  7
 10	 G 	  1191.56	   634.32	  6
 11	 D 	  1306.59	   577.29	  5
 12	 S 	  1393.62	   462.27	  4
 13	 I 	  1506.70	   375.24	  3
 14	 S 	  1593.74	   262.15	  2
 15	 R 	        -  	   175.12	  1  _
+2 charge
		      B   	     Y
  1	 I 	    57.55	        -  	 15
  2	 Y 	   139.08	   827.89	 14
  3	 Q 	   203.11	   746.35	 13
  4	 I 	   259.65	   682.32	 12
  5	 M 	   325.17	   625.78	 11
  6	 W 	   418.21	   560.26	 10
  7	 A 	   453.73	   467.22	  9
  8	 N 	   510.75	   431.70	  8
  9	 N 	   567.77	   374.68	  7
 10	 G 	   596.28	   317.66	  6
 11	 D 	   653.80	   289.15	  5
 12	 S 	   697.31	   231.64	  4
 13	 I 	   753.86	   188.12	  3
 14	 S 	   797.37	   131.58	  2
 15	 R 	        -  	    88.06	  1  _
```

**158. IPI00029997 6-phosphogluconolactonase**

| Sequence | Charge | M+H+ | XCorr | DeltaCn |
| --- | --- | --- | --- | --- |
| R.FALGLSGGSLVSMLAR.E | 2 | 1579.889 | 2.813 | 0.383 |

+1 charge

```
		      B   	     Y
  1	 F 	   148.08	        -  	 16
  2	 A 	   219.11	  1431.80	 15
  3	 L 	   332.20	  1360.76	 14
  4	 G 	   389.22	  1247.68	 13
  5	 L 	   502.30	  1190.66	 12
  6	 S 	   589.33	  1077.57	 11
  7	 G 	   646.36	   990.54	 10
  8	 G 	   703.38	   933.52	  9
  9	 S 	   790.41	   876.50	  8
 10	 L 	   903.49	   789.47	  7
 11	 V 	  1002.56	   676.38	  6
 12	 S 	  1089.59	   577.31	  5
 13	 M 	  1220.63	   490.28	  4
 14	 L 	  1333.72	   359.24	  3
 15	 A 	  1404.76	   246.16	  2
 16	 R 	        -  	   175.12	  1  _
+2 charge
		      B   	     Y
  1	 F 	    74.54	        -  	 16
  2	 A 	   110.06	   716.40	 15
  3	 L 	   166.60	   680.88	 14
  4	 G 	   195.11	   624.34	 13
  5	 L 	   251.65	   595.83	 12
  6	 S 	   295.17	   539.29	 11
  7	 G 	   323.68	   495.77	 10
  8	 G 	   352.19	   467.26	  9
  9	 S 	   395.71	   438.75	  8
 10	 L 	   452.25	   395.24	  7
 11	 V 	   501.78	   338.69	  6
 12	 S 	   545.30	   289.16	  5
 13	 M 	   610.82	   245.64	  4
 14	 L 	   667.36	   180.12	  3
 15	 A 	   702.88	   123.58	  2
 16	 R 	        -  	    88.06	  1  _
```

**159. IPI00017557 Secreted frizzled-related protein 4**

| Sequence | Charge | M+H+ | XCorr | DeltaCn |
| --- | --- | --- | --- | --- |
| R.FFLCAMYAPICTLEFLHDPIKPCK.S | 3 | 2973.501 | 3.545 | 0.463 |

+1 charge

```
		      B   	     Y
  1	 F 	   148.08	        -  	 24
  2	 F 	   295.14	  2824.77	 23
  3	 L 	   408.23	  2677.70	 22
  4	 C 	   568.39	  2564.62	 21
  5	 A 	   639.43	  2404.46	 20
  6	 M 	   770.47	  2333.42	 19
  7	 Y 	   933.53	  2202.38	 18
  8	 A 	  1004.57	  2039.31	 17
  9	 P 	  1101.62	  1968.28	 16
 10	 I 	  1214.71	  1871.22	 15
 11	 C 	  1374.87	  1758.14	 14
 12	 T 	  1475.92	  1597.98	 13
 13	 L 	  1589.01	  1496.93	 12
 14	 E 	  1718.05	  1383.84	 11
 15	 F 	  1865.12	  1254.80	 10
 16	 L 	  1978.20	  1107.73	  9
 17	 H 	  2115.26	   994.65	  8
 18	 D 	  2230.29	   857.59	  7
 19	 P 	  2327.34	   742.56	  6
 20	 I 	  2440.42	   645.51	  5
 21	 K 	  2568.52	   532.43	  4
 22	 P 	  2665.57	   404.33	  3
 23	 C 	  2825.74	   307.28	  2
 24	 K 	        -  	   147.11	  1  _


+2 charge
		      B   	     Y
  1	 F 	    74.54	        -  	 24
  2	 F 	   148.08	  1412.89	 23
  3	 L 	   204.62	  1339.36	 22
  4	 C 	   284.70	  1282.81	 21
  5	 A 	   320.22	  1202.73	 20
  6	 M 	   385.74	  1167.21	 19
  7	 Y 	   467.27	  1101.69	 18
  8	 A 	   502.79	  1020.16	 17
  9	 P 	   551.32	   984.64	 16
 10	 I 	   607.86	   936.12	 15
 11	 C 	   687.94	   879.57	 14
 12	 T 	   738.46	   799.49	 13
 13	 L 	   795.01	   748.97	 12
 14	 E 	   859.53	   692.43	 11
 15	 F 	   933.06	   627.90	 10
 16	 L 	   989.60	   554.37	  9
 17	 H 	  1058.13	   497.83	  8
 18	 D 	  1115.65	   429.30	  7
 19	 P 	  1164.17	   371.78	  6
 20	 I 	  1220.72	   323.26	  5
 21	 K 	  1284.76	   266.72	  4
 22	 P 	  1333.29	   202.67	  3
 23	 C 	  1413.37	   154.14	  2
 24	 K 	        -  	    74.06	  1  _
+3 charge
		      B   	     Y
  1	 F 	    50.03	        -  	 24
  2	 F 	    99.05	   942.26	 23
  3	 L 	   136.75	   893.24	 22
  4	 C 	   190.14	   855.55	 21
  5	 A 	   213.82	   802.16	 20
  6	 M 	   257.50	   778.48	 19
  7	 Y 	   311.85	   734.80	 18
  8	 A 	   335.53	   680.44	 17
  9	 P 	   367.88	   656.76	 16
 10	 I 	   405.57	   624.41	 15
 11	 C 	   458.96	   586.72	 14
 12	 T 	   492.65	   533.33	 13
 13	 L 	   530.34	   499.65	 12
 14	 E 	   573.35	   461.95	 11
 15	 F 	   622.38	   418.94	 10
 16	 L 	   660.07	   369.92	  9
 17	 H 	   705.76	   332.22	  8
 18	 D 	   744.10	   286.53	  7
 19	 P 	   776.45	   248.19	  6
 20	 I 	   814.15	   215.84	  5
 21	 K 	   856.84	   178.15	  4
 22	 P 	   889.20	   135.45	  3
 23	 C 	   942.58	   103.10	  2
 24	 K 	        -  	    49.71	  1  _
```

**Female 9**

**1. IPI00304962 Collagen alpha-2(I) chain**

| Sequence | Charge | M+H+ | XCorr | DeltaCn |
| --- | --- | --- | --- | --- |
| R.GETGPSGPVGPAGAVGPR.G | 2 | 1563.697 | 3.154 | 0.383 |

+1 charge

```
		      B   	       Y
  1	 G 	    58.03	        -  	 18
  2	 E 	   187.07	  1505.77	 17
  3	 T 	   288.12	  1376.73	 16
  4	 G 	   345.14	  1275.68	 15
  5	 P 	   442.19	  1218.66	 14
  6	 S 	   529.23	  1121.61	 13
  7	 G 	   586.25	  1034.57	 12
  8	 P 	   683.30	   977.55	 11
  9	 V 	   782.37	   880.50	 10
 10	 G 	   839.39	   781.43	  9
 11	 P 	   936.44	   724.41	  8
 12	 A 	  1007.48	   627.36	  7
 13	 G 	  1064.50	   556.32	  6
 14	 A 	  1135.54	   499.30	  5
 15	 V 	  1234.61	   428.26	  4
 16	 G 	  1291.63	   329.19	  3
 17	 P 	  1388.68	   272.17	  2
 18	 R 	        -  	   175.12	  1  _
+2 charge
		      B   	       Y
  1	 G 	    29.52	        -  	 18
  2	 E 	    94.04	   753.39	 17
  3	 T 	   144.56	   688.87	 16
  4	 G 	   173.07	   638.34	 15
  5	 P 	   221.60	   609.83	 14
  6	 S 	   265.12	   561.31	 13
  7	 G 	   293.63	   517.79	 12
  8	 P 	   342.15	   489.28	 11
  9	 V 	   391.69	   440.75	 10
 10	 G 	   420.20	   391.22	  9
 11	 P 	   468.72	   362.71	  8
 12	 A 	   504.24	   314.18	  7
 13	 G 	   532.75	   278.66	  6
 14	 A 	   568.27	   250.15	  5
 15	 V 	   617.81	   214.63	  4
 16	 G 	   646.32	   165.10	  3
 17	 P 	   694.84	   136.59	  2
 18	 R 	        -  	    88.06	  1  _
```

**2. IPI00299724 Isoform 1 of Signal-regulatory protein beta-1**

| Sequence | Charge | M+H+ | XCorr | DeltaCn |
| --- | --- | --- | --- | --- |
| R.VPPTLEVTQQPMR.V | 2 | 1496.757 | 2.996 | 0.469 |

+1 charge

```
		      B   	       Y
  1	 V 	   100.08	        -  	 13
  2	 P 	   197.13	  1396.73	 12
  3	 P 	   294.18	  1299.67	 11
  4	 T 	   395.23	  1202.62	 10
  5	 L 	   508.31	  1101.57	  9
  6	 E 	   637.36	   988.49	  8
  7	 V 	   736.42	   859.45	  7
  8	 T 	   837.47	   760.38	  6
  9	 Q 	   965.53	   659.33	  5
 10	 Q 	  1093.59	   531.27	  4
 11	 P 	  1190.64	   403.21	  3
 12	 M 	  1321.68	   306.16	  2
 13	 R 	        -  	   175.12	  1  _
+2 charge
		      B   	       Y
  1	 V 	    50.54	        -  	 13
  2	 P 	    99.07	   698.87	 12
  3	 P 	   147.59	   650.34	 11
  4	 T 	   198.12	   601.81	 10
  5	 L 	   254.66	   551.29	  9
  6	 E 	   319.18	   494.75	  8
  7	 V 	   368.72	   430.23	  7
  8	 T 	   419.24	   380.69	  6
  9	 Q 	   483.27	   330.17	  5
 10	 Q 	   547.30	   266.14	  4
 11	 P 	   595.82	   202.11	  3
 12	 M 	   661.34	   153.58	  2
 13	 R 	        -  	    88.06	  1  _
```

**3. IPI00029997 6-phosphogluconolactonase**

| Sequence | Charge | M+H+ | XCorr | DeltaCn |
| --- | --- | --- | --- | --- |
| R.ELPAAVAPAGPASLAR.W | 2 | 1491.717 | 3.22 | 0.544 |

+1 charge

```
		      B   	       Y
  1	 E 	   130.05	        -  	 16
  2	 L 	   243.13	  1361.79	 15
  3	 P 	   340.19	  1248.71	 14
  4	 A 	   411.22	  1151.65	 13
  5	 A 	   482.26	  1080.62	 12
  6	 V 	   581.33	  1009.58	 11
  7	 A 	   652.37	   910.51	 10
  8	 P 	   749.42	   839.47	  9
  9	 A 	   820.46	   742.42	  8
 10	 G 	   877.48	   671.38	  7
 11	 P 	   974.53	   614.36	  6
 12	 A 	  1045.57	   517.31	  5
 13	 S 	  1132.60	   446.27	  4
 14	 L 	  1245.68	   359.24	  3
 15	 A 	  1316.72	   246.16	  2
 16	 R 	        -  	   175.12	  1  _
+2 charge
		      B   	       Y
  1	 E 	    65.53	        -  	 16
  2	 L 	   122.07	   681.40	 15
  3	 P 	   170.60	   624.86	 14
  4	 A 	   206.12	   576.33	 13
  5	 A 	   241.63	   540.81	 12
  6	 V 	   291.17	   505.29	 11
  7	 A 	   326.69	   455.76	 10
  8	 P 	   375.21	   420.24	  9
  9	 A 	   410.73	   371.71	  8
 10	 G 	   439.24	   336.20	  7
 11	 P 	   487.77	   307.68	  6
 12	 A 	   523.29	   259.16	  5
 13	 S 	   566.80	   223.64	  4
 14	 L 	   623.35	   180.12	  3
 15	 A 	   658.86	   123.58	  2
 16	 R 	        -  	    88.06	  1  _
```

**4. IPI00026314 Isoform 1 of Gelsolin**

| Sequence | Charge | M+H+ | XCorr | DeltaCn |
| --- | --- | --- | --- | --- |
| R.VPFDAATLHTSTAMAAQHGMDDDGTGQK.Q | 3 | 2875.101 | 7.029 | 0.671 |

+1 charge

```
		      B   	       Y
  1	 V 	   100.08	        -  	 28
  2	 P 	   197.13	  2774.21	 27
  3	 F 	   344.20	  2677.16	 26
  4	 D 	   459.22	  2530.09	 25
  5	 A 	   530.26	  2415.07	 24
  6	 A 	   601.30	  2344.03	 23
  7	 T 	   702.35	  2272.99	 22
  8	 L 	   815.43	  2171.94	 21
  9	 H 	   952.49	  2058.86	 20
 10	 T 	  1053.54	  1921.80	 19
 11	 S 	  1140.57	  1820.75	 18
 12	 T 	  1241.62	  1733.72	 17
 13	 A 	  1312.65	  1632.67	 16
 14	 M 	  1443.69	  1561.64	 15
 15	 A 	  1514.73	  1430.60	 14
 16	 A 	  1585.77	  1359.56	 13
 17	 Q 	  1713.83	  1288.52	 12
 18	 H 	  1850.89	  1160.46	 11
 19	 G 	  1907.91	  1023.40	 10
 20	 M 	  2038.95	   966.38	  9
 21	 D 	  2153.97	   835.34	  8
 22	 D 	  2269.00	   720.32	  7
 23	 D 	  2384.03	   605.29	  6
 24	 G 	  2441.05	   490.26	  5
 25	 T 	  2542.10	   433.24	  4
 26	 G 	  2599.12	   332.19	  3
 27	 Q 	  2727.18	   275.17	  2
 28	 K 	        -  	   147.11	  1  _


+2 charge
		      B   	       Y
  1	 V 	    50.54	        -  	 28
  2	 P 	    99.07	  1387.61	 27
  3	 F 	   172.60	  1339.08	 26
  4	 D 	   230.12	  1265.55	 25
  5	 A 	   265.63	  1208.04	 24
  6	 A 	   301.15	  1172.52	 23
  7	 T 	   351.68	  1137.00	 22
  8	 L 	   408.22	  1086.48	 21
  9	 H 	   476.75	  1029.93	 20
 10	 T 	   527.27	   961.40	 19
 11	 S 	   570.79	   910.88	 18
 12	 T 	   621.31	   867.36	 17
 13	 A 	   656.83	   816.84	 16
 14	 M 	   722.35	   781.32	 15
 15	 A 	   757.87	   715.80	 14
 16	 A 	   793.39	   680.28	 13
 17	 Q 	   857.42	   644.76	 12
 18	 H 	   925.95	   580.74	 11
 19	 G 	   954.46	   512.21	 10
 20	 M 	  1019.98	   483.70	  9
 21	 D 	  1077.49	   418.18	  8
 22	 D 	  1135.00	   360.66	  7
 23	 D 	  1192.52	   303.15	  6
 24	 G 	  1221.03	   245.63	  5
 25	 T 	  1271.55	   217.12	  4
 26	 G 	  1300.06	   166.60	  3
 27	 Q 	  1364.09	   138.09	  2
 28	 K 	        -  	    74.06	  1  _


+3 charge
		      B   	       Y
  1	 V 	    34.03	        -  	 28
  2	 P 	    66.38	   925.41	 27
  3	 F 	   115.40	   893.06	 26
  4	 D 	   153.75	   844.04	 25
  5	 A 	   177.43	   805.69	 24
  6	 A 	   201.10	   782.01	 23
  7	 T 	   234.79	   758.34	 22
  8	 L 	   272.48	   724.65	 21
  9	 H 	   318.17	   686.96	 20
 10	 T 	   351.85	   641.27	 19
 11	 S 	   380.86	   607.59	 18
 12	 T 	   414.54	   578.58	 17
 13	 A 	   438.22	   544.90	 16
 14	 M 	   481.90	   521.22	 15
 15	 A 	   505.58	   477.54	 14
 16	 A 	   529.26	   453.86	 13
 17	 Q 	   571.95	   430.18	 12
 18	 H 	   617.63	   387.49	 11
 19	 G 	   636.64	   341.81	 10
 20	 M 	   680.32	   322.80	  9
 21	 D 	   718.66	   279.12	  8
 22	 D 	   757.01	   240.78	  7
 23	 D 	   795.35	   202.43	  6
 24	 G 	   814.35	   164.09	  5
 25	 T 	   848.04	   145.09	  4
 26	 G 	   867.04	   111.40	  3
 27	 Q 	   909.73	    92.40	  2
 28	 K 	        -  	    49.71	  1  _
```

**5. IPI00291867 Complement factor I**

| Sequence | Charge | M+H+ | XCorr | DeltaCn |
| --- | --- | --- | --- | --- |
| R.GLETSLAECTFTK.R | 2 | 1457.601 | 3.724 | 0.551 |

+1 charge

```
		      B   	       Y
  1	 G 	    58.03	        -  	 13
  2	 L 	   171.11	  1399.81	 12
  3	 E 	   300.16	  1286.73	 11
  4	 T 	   401.20	  1157.69	 10
  5	 S 	   488.24	  1056.64	  9
  6	 L 	   601.32	   969.61	  8
  7	 A 	   672.36	   856.52	  7
  8	 E 	   801.40	   785.48	  6
  9	 C 	   961.56	   656.44	  5
 10	 T 	  1062.61	   496.28	  4
 11	 F 	  1209.68	   395.23	  3
 12	 T 	  1310.73	   248.16	  2
 13	 K 	        -  	   147.11	  1  _
+2 charge
		      B   	       Y
  1	 G 	    29.52	        -  	 13
  2	 L 	    86.06	   700.41	 12
  3	 E 	   150.58	   643.87	 11
  4	 T 	   201.11	   579.35	 10
  5	 S 	   244.62	   528.82	  9
  6	 L 	   301.16	   485.31	  8
  7	 A 	   336.68	   428.76	  7
  8	 E 	   401.20	   393.25	  6
  9	 C 	   481.29	   328.72	  5
 10	 T 	   531.81	   248.64	  4
 11	 F 	   605.34	   198.12	  3
 12	 T 	   655.87	   124.58	  2
 13	 K 	        -  	    74.06	  1  _
```

**6. IPI00328113 Fibrillin-1**

| Sequence | Charge | M+H+ | XCorr | DeltaCn |
| --- | --- | --- | --- | --- |
| R.TCVDINECLLEPR.K | 2 | 1619.786 | 3.513 | 0.455 |

+1 charge

```
		      B   	       Y
  1	 T 	   102.05	        -  	 13
  2	 C 	   262.22	  1517.98	 12
  3	 V 	   361.29	  1357.81	 11
  4	 D 	   476.32	  1258.74	 10
  5	 I 	   589.40	  1143.72	  9
  6	 N 	   703.44	  1030.63	  8
  7	 E 	   832.49	   916.59	  7
  8	 C 	   992.65	   787.55	  6
  9	 L 	  1105.73	   627.38	  5
 10	 L 	  1218.82	   514.30	  4
 11	 E 	  1347.86	   401.21	  3
 12	 P 	  1444.91	   272.17	  2
 13	 R 	        -  	   175.12	  1  _
+2 charge
		      B   	       Y
  1	 T 	    51.53	        -  	 13
  2	 C 	   131.61	   759.49	 12
  3	 V 	   181.15	   679.41	 11
  4	 D 	   238.66	   629.88	 10
  5	 I 	   295.20	   572.36	  9
  6	 N 	   352.22	   515.82	  8
  7	 E 	   416.75	   458.80	  7
  8	 C 	   496.83	   394.28	  6
  9	 L 	   553.37	   314.19	  5
 10	 L 	   609.91	   257.65	  4
 11	 E 	   674.43	   201.11	  3
 12	 P 	   722.96	   136.59	  2
 13	 R 	        -  	    88.06	  1  _
```

**7. IPI00011694 Trypsin-1**

| Sequence | Charge | M+H+ | XCorr | DeltaCn |
| --- | --- | --- | --- | --- |
| K.TLNNDIMLIK.L | 2 | 1175.424 | 3.459 | 0.286 |

+1 charge

```
		      B   	       Y
  1	 T 	   102.05	        -  	 10
  2	 L 	   215.14	  1073.60	  9
  3	 N 	   329.18	   960.52	  8
  4	 N 	   443.22	   846.48	  7
  5	 D 	   558.25	   732.43	  6
  6	 I 	   671.34	   617.41	  5
  7	 M 	   802.38	   504.32	  4
  8	 L 	   915.46	   373.28	  3
  9	 I 	  1028.54	   260.20	  2
 10	 K 	        -  	   147.11	  1  _
+2 charge
		      B   	       Y
  1	 T 	    51.53	        -  	 10
  2	 L 	   108.07	   537.30	  9
  3	 N 	   165.09	   480.76	  8
  4	 N 	   222.12	   423.74	  7
  5	 D 	   279.63	   366.72	  6
  6	 I 	   336.17	   309.21	  5
  7	 M 	   401.69	   252.66	  4
  8	 L 	   458.23	   187.14	  3
  9	 I 	   514.78	   130.60	  2
 10	 K 	        -  	    74.06	  1  _
```

**8. IPI00410487 Isoform 1 of Twisted gastrulation protein homolog 1**

| Sequence | Charge | M+H+ | XCorr | DeltaCn |
| --- | --- | --- | --- | --- |
| K.STVEELHEPIPSLFR.A | 2 | 1754.964 | 4.276 | 0.5 |

+1 charge

```
		      B   	       Y
  1	 S 	    88.04	        -  	 15
  2	 T 	   189.09	  1666.88	 14
  3	 V 	   288.16	  1565.83	 13
  4	 E 	   417.20	  1466.76	 12
  5	 E 	   546.24	  1337.72	 11
  6	 L 	   659.32	  1208.68	 10
  7	 H 	   796.38	  1095.59	  9
  8	 E 	   925.43	   958.54	  8
  9	 P 	  1022.48	   829.49	  7
 10	 I 	  1135.56	   732.44	  6
 11	 P 	  1232.62	   619.36	  5
 12	 S 	  1319.65	   522.30	  4
 13	 L 	  1432.73	   435.27	  3
 14	 F 	  1579.80	   322.19	  2
 15	 R 	        -  	   175.12	  1  _
+2 charge
		      B   	       Y
  1	 S 	    44.52	        -  	 15
  2	 T 	    95.05	   833.94	 14
  3	 V 	   144.58	   783.42	 13
  4	 E 	   209.10	   733.89	 12
  5	 E 	   273.62	   669.36	 11
  6	 L 	   330.17	   604.84	 10
  7	 H 	   398.70	   548.30	  9
  8	 E 	   463.22	   479.77	  8
  9	 P 	   511.74	   415.25	  7
 10	 I 	   568.29	   366.72	  6
 11	 P 	   616.81	   310.18	  5
 12	 S 	   660.33	   261.66	  4
 13	 L 	   716.87	   218.14	  3
 14	 F 	   790.40	   161.60	  2
 15	 R 	        -  	    88.06	  1  _
```

**9. IPI00021536 Calmodulin-like protein 5**

| Sequence | Charge | M+H+ | XCorr | DeltaCn |
| --- | --- | --- | --- | --- |
| R.AGLEDLQVAFR.A | 2 | 1219.372 | 3.398 | 0.326 |

+1 charge

```
		      B   	       Y
  1	 A 	    72.04	        -  	 11
  2	 G 	   129.07	  1147.61	 10
  3	 L 	   242.15	  1090.59	  9
  4	 E 	   371.19	   977.51	  8
  5	 D 	   486.22	   848.46	  7
  6	 L 	   599.30	   733.44	  6
  7	 Q 	   727.36	   620.35	  5
  8	 V 	   826.43	   492.29	  4
  9	 A 	   897.47	   393.22	  3
 10	 F 	  1044.54	   322.19	  2
 11	 R 	        -  	   175.12	  1  _
+2 charge
		      B   	       Y
  1	 A 	    36.53	        -  	 11
  2	 G 	    65.04	   574.31	 10
  3	 L 	   121.58	   545.80	  9
  4	 E 	   186.10	   489.26	  8
  5	 D 	   243.61	   424.73	  7
  6	 L 	   300.16	   367.22	  6
  7	 Q 	   364.18	   310.68	  5
  8	 V 	   413.72	   246.65	  4
  9	 A 	   449.24	   197.12	  3
 10	 F 	   522.77	   161.60	  2
 11	 R 	        -  	    88.06	  1  _
```

**11. IPI00414320 cDNA FLJ55482, highly similar to Annexin A11**

| Sequence | Charge | M+H+ | XCorr | DeltaCn |
| --- | --- | --- | --- | --- |
| K.GVGTDEACLIEILASR.S | 2 | 1704.897 | 3.6 | 0.368 |

+1 charge

```
		      B   	       Y
  1	 G 	    58.03	        -  	 16
  2	 V 	   157.10	  1646.98	 15
  3	 G 	   214.12	  1547.91	 14
  4	 T 	   315.17	  1490.89	 13
  5	 D 	   430.19	  1389.84	 12
  6	 E 	   559.24	  1274.81	 11
  7	 A 	   630.27	  1145.77	 10
  8	 C 	   790.44	  1074.73	  9
  9	 L 	   903.52	   914.57	  8
 10	 I 	  1016.61	   801.48	  7
 11	 E 	  1145.65	   688.40	  6
 12	 I 	  1258.73	   559.36	  5
 13	 L 	  1371.82	   446.27	  4
 14	 A 	  1442.85	   333.19	  3
 15	 S 	  1529.89	   262.15	  2
 16	 R 	        -  	   175.12	  1  _
+2 charge
		      B   	       Y
  1	 G 	    29.52	        -  	 16
  2	 V 	    79.05	   823.99	 15
  3	 G 	   107.56	   774.46	 14
  4	 T 	   158.09	   745.95	 13
  5	 D 	   215.60	   695.42	 12
  6	 E 	   280.12	   637.91	 11
  7	 A 	   315.64	   573.39	 10
  8	 C 	   395.72	   537.87	  9
  9	 L 	   452.26	   457.79	  8
 10	 I 	   508.81	   401.25	  7
 11	 E 	   573.33	   344.70	  6
 12	 I 	   629.87	   280.18	  5
 13	 L 	   686.41	   223.64	  4
 14	 A 	   721.93	   167.10	  3
 15	 S 	   765.45	   131.58	  2
 16	 R 	        -  	    88.06	  1  _
```

**12. IPI00073772 Fructose-1,6-bisphosphatase 1**

| Sequence | Charge | M+H+ | XCorr | DeltaCn |
| --- | --- | --- | --- | --- |
| R.APVILGSPDDVLEFLK.V | 2 | 1713.995 | 3.702 | 0.501 |

+1 charge

```
		      B   	       Y
  1	 A 	    72.04	        -  	 16
  2	 P 	   169.10	  1641.91	 15
  3	 V 	   268.17	  1544.86	 14
  4	 I 	   381.25	  1445.79	 13
  5	 L 	   494.33	  1332.70	 12
  6	 G 	   551.36	  1219.62	 11
  7	 S 	   638.39	  1162.60	 10
  8	 P 	   735.44	  1075.57	  9
  9	 D 	   850.47	   978.51	  8
 10	 D 	   965.49	   863.49	  7
 11	 V 	  1064.56	   748.46	  6
 12	 L 	  1177.65	   649.39	  5
 13	 E 	  1306.69	   536.31	  4
 14	 F 	  1453.76	   407.27	  3
 15	 L 	  1566.84	   260.20	  2
 16	 K 	        -  	   147.11	  1  _
+2 charge
		      B   	       Y
  1	 A 	    36.53	        -  	 16
  2	 P 	    85.05	   821.46	 15
  3	 V 	   134.59	   772.93	 14
  4	 I 	   191.13	   723.40	 13
  5	 L 	   247.67	   666.86	 12
  6	 G 	   276.18	   610.31	 11
  7	 S 	   319.70	   581.80	 10
  8	 P 	   368.22	   538.29	  9
  9	 D 	   425.74	   489.76	  8
 10	 D 	   483.25	   432.25	  7
 11	 V 	   532.78	   374.73	  6
 12	 L 	   589.33	   325.20	  5
 13	 E 	   653.85	   268.66	  4
 14	 F 	   727.38	   204.14	  3
 15	 L 	   783.92	   130.60	  2
 16	 K 	        -  	    74.06	  1  _
```

**13. IPI00843975 Ezrin**

| Sequence | Charge | M+H+ | XCorr | DeltaCn |
| --- | --- | --- | --- | --- |
| K.FYPEDVAEELIQDITQK.L | 2 | 2039.228 | 3.117 | 0.387 |

+1 charge

```
		      B   	       Y
  1	 F 	   148.08	        -  	 17
  2	 Y 	   311.14	  1890.93	 16
  3	 P 	   408.19	  1727.87	 15
  4	 E 	   537.23	  1630.82	 14
  5	 D 	   652.26	  1501.77	 13
  6	 V 	   751.33	  1386.75	 12
  7	 A 	   822.37	  1287.68	 11
  8	 E 	   951.41	  1216.64	 10
  9	 E 	  1080.45	  1087.60	  9
 10	 L 	  1193.54	   958.56	  8
 11	 I 	  1306.62	   845.47	  7
 12	 Q 	  1434.68	   732.39	  6
 13	 D 	  1549.71	   604.33	  5
 14	 I 	  1662.79	   489.30	  4
 15	 T 	  1763.84	   376.22	  3
 16	 Q 	  1891.90	   275.17	  2
 17	 K 	        -  	   147.11	  1  _
+2 charge
		      B   	       Y
  1	 F 	    74.54	        -  	 17
  2	 Y 	   156.07	   945.97	 16
  3	 P 	   204.60	   864.44	 15
  4	 E 	   269.12	   815.91	 14
  5	 D 	   326.63	   751.39	 13
  6	 V 	   376.17	   693.88	 12
  7	 A 	   411.69	   644.34	 11
  8	 E 	   476.21	   608.82	 10
  9	 E 	   540.73	   544.30	  9
 10	 L 	   597.27	   479.78	  8
 11	 I 	   653.81	   423.24	  7
 12	 Q 	   717.84	   366.70	  6
 13	 D 	   775.36	   302.67	  5
 14	 I 	   831.90	   245.16	  4
 15	 T 	   882.42	   188.61	  3
 16	 Q 	   946.45	   138.09	  2
 17	 K 	        -  	    74.06	  1  _
```

**14. IPI00027827 Extracellular superoxide dismutase [Cu-Zn]**

| Sequence | Charge | M+H+ | XCorr | DeltaCn |
| --- | --- | --- | --- | --- |
| R.AGLAASLAGPHSIVGR.A | 2 | 1477.694 | 4.353 | 0.582 |

+1 charge

```
		      B   	       Y
  1	 A 	    72.04	        -  	 16
  2	 G 	   129.07	  1405.79	 15
  3	 L 	   242.15	  1348.77	 14
  4	 A 	   313.19	  1235.69	 13
  5	 A 	   384.22	  1164.65	 12
  6	 S 	   471.26	  1093.61	 11
  7	 L 	   584.34	  1006.58	 10
  8	 A 	   655.38	   893.50	  9
  9	 G 	   712.40	   822.46	  8
 10	 P 	   809.45	   765.44	  7
 11	 H 	   946.51	   668.38	  6
 12	 S 	  1033.54	   531.32	  5
 13	 I 	  1146.63	   444.29	  4
 14	 V 	  1245.69	   331.21	  3
 15	 G 	  1302.72	   232.14	  2
 16	 R 	        -  	   175.12	  1  _
+2 charge
		      B   	       Y
  1	 A 	    36.53	        -  	 16
  2	 G 	    65.04	   703.40	 15
  3	 L 	   121.58	   674.89	 14
  4	 A 	   157.10	   618.35	 13
  5	 A 	   192.62	   582.83	 12
  6	 S 	   236.13	   547.31	 11
  7	 L 	   292.67	   503.79	 10
  8	 A 	   328.19	   447.25	  9
  9	 G 	   356.70	   411.73	  8
 10	 P 	   405.23	   383.22	  7
 11	 H 	   473.76	   334.70	  6
 12	 S 	   517.27	   266.17	  5
 13	 I 	   573.82	   222.65	  4
 14	 V 	   623.35	   166.11	  3
 15	 G 	   651.86	   116.57	  2
 16	 R 	        -  	    88.06	  1  _
```

**15. IPI00024331 Tumor necrosis factor receptor superfamily member 14**

| Sequence | Charge | M+H+ | XCorr | DeltaCn |
| --- | --- | --- | --- | --- |
| K.CLQCQMCDPAMGLR.A | 2 | 1741.008 | 4.163 | 0.545 |

+1 charge

```
		      B   	       Y
  1	 C 	   161.17	        -  	 14
  2	 L 	   274.26	  1579.95	 13
  3	 Q 	   402.32	  1466.87	 12
  4	 C 	   562.48	  1338.81	 11
  5	 Q 	   690.54	  1178.65	 10
  6	 M 	   821.58	  1050.59	  9
  7	 C 	   981.74	   919.55	  8
  8	 D 	  1096.77	   759.38	  7
  9	 P 	  1193.82	   644.35	  6
 10	 A 	  1264.86	   547.30	  5
 11	 M 	  1395.90	   476.26	  4
 12	 G 	  1452.92	   345.22	  3
 13	 L 	  1566.01	   288.20	  2
 14	 R 	        -  	   175.12	  1  _
+2 charge
		      B   	       Y
  1	 C 	    81.09	        -  	 14
  2	 L 	   137.63	   790.48	 13
  3	 Q 	   201.66	   733.94	 12
  4	 C 	   281.74	   669.91	 11
  5	 Q 	   345.77	   589.83	 10
  6	 M 	   411.29	   525.80	  9
  7	 C 	   491.38	   460.28	  8
  8	 D 	   548.89	   380.19	  7
  9	 P 	   597.42	   322.68	  6
 10	 A 	   632.93	   274.15	  5
 11	 M 	   698.45	   238.64	  4
 12	 G 	   726.97	   173.12	  3
 13	 L 	   783.51	   144.61	  2
 14	 R 	        -  	    88.06	  1  _
```

**16. IPI00219827 Secreted Ly-6/uPAR-related protein 2**

| Sequence | Charge | M+H+ | XCorr | DeltaCn |
| --- | --- | --- | --- | --- |
| R.VLSNTEDLPLVTK.M | 2 | 1429.641 | 4.108 | 0.554 |

+1 charge

```
		      B   	       Y
  1	 V 	   100.08	        -  	 13
  2	 L 	   213.16	  1329.73	 12
  3	 S 	   300.19	  1216.64	 11
  4	 N 	   414.23	  1129.61	 10
  5	 T 	   515.28	  1015.57	  9
  6	 E 	   644.32	   914.52	  8
  7	 D 	   759.35	   785.48	  7
  8	 L 	   872.44	   670.45	  6
  9	 P 	   969.49	   557.37	  5
 10	 L 	  1082.57	   460.31	  4
 11	 V 	  1181.64	   347.23	  3
 12	 T 	  1282.69	   248.16	  2
 13	 K 	        -  	   147.11	  1  _
+2 charge
		      B   	       Y
  1	 V 	    50.54	        -  	 13
  2	 L 	   107.08	   665.37	 12
  3	 S 	   150.60	   608.82	 11
  4	 N 	   207.62	   565.31	 10
  5	 T 	   258.14	   508.29	  9
  6	 E 	   322.67	   457.76	  8
  7	 D 	   380.18	   393.24	  7
  8	 L 	   436.72	   335.73	  6
  9	 P 	   485.25	   279.19	  5
 10	 L 	   541.79	   230.66	  4
 11	 V 	   591.32	   174.12	  3
 12	 T 	   641.85	   124.58	  2
 13	 K 	        -  	    74.06	  1  _
```

**17. IPI00002435 CD27 antigen**

| Sequence | Charge | M+H+ | XCorr | DeltaCn |
| --- | --- | --- | --- | --- |
| K.LCCQMCEPGTFLVK.D | 2 | 1744.027 | 4.25 | 0.43 |

+1 charge

```
		      B   	       Y
  1	 L 	   114.09	        -  	 14
  2	 C 	   274.26	  1630.09	 13
  3	 C 	   434.42	  1469.93	 12
  4	 Q 	   562.48	  1309.76	 11
  5	 M 	   693.52	  1181.70	 10
  6	 C 	   853.69	  1050.66	  9
  7	 E 	   982.73	   890.50	  8
  8	 P 	  1079.78	   761.46	  7
  9	 G 	  1136.80	   664.40	  6
 10	 T 	  1237.85	   607.38	  5
 11	 F 	  1384.92	   506.33	  4
 12	 L 	  1498.00	   359.27	  3
 13	 V 	  1597.07	   246.18	  2
 14	 K 	        -  	   147.11	  1  _
+2 charge
		      B   	       Y
  1	 L 	    57.55	        -  	 14
  2	 C 	   137.63	   815.55	 13
  3	 C 	   217.71	   735.47	 12
  4	 Q 	   281.74	   655.38	 11
  5	 M 	   347.26	   591.36	 10
  6	 C 	   427.35	   525.84	  9
  7	 E 	   491.87	   445.75	  8
  8	 P 	   540.39	   381.23	  7
  9	 G 	   568.91	   332.71	  6
 10	 T 	   619.43	   304.19	  5
 11	 F 	   692.96	   253.67	  4
 12	 L 	   749.51	   180.14	  3
 13	 V 	   799.04	   123.59	  2
 14	 K 	        -  	    74.06	  1  _
```

**18. IPI00010949 Isoform 1 of Sialate O-acetylesterase**

| Sequence | Charge | M+H+ | XCorr | DeltaCn |
| --- | --- | --- | --- | --- |
| R.FFPFGLVQLSSDLSK.K | 2 | 1685.944 | 3.037 | 0.534 |

+1 charge

```
		      B   	       Y
  1	 F 	   148.08	        -  	 15
  2	 F 	   295.14	  1537.83	 14
  3	 P 	   392.20	  1390.76	 13
  4	 F 	   539.27	  1293.70	 12
  5	 G 	   596.29	  1146.64	 11
  6	 L 	   709.37	  1089.62	 10
  7	 V 	   808.44	   976.53	  9
  8	 Q 	   936.50	   877.46	  8
  9	 L 	  1049.58	   749.40	  7
 10	 S 	  1136.61	   636.32	  6
 11	 S 	  1223.65	   549.29	  5
 12	 D 	  1338.67	   462.26	  4
 13	 L 	  1451.76	   347.23	  3
 14	 S 	  1538.79	   234.14	  2
 15	 K 	        -  	   147.11	  1  _
+2 charge
		      B   	       Y
  1	 F 	    74.54	        -  	 15
  2	 F 	   148.08	   769.42	 14
  3	 P 	   196.60	   695.88	 13
  4	 F 	   270.14	   647.36	 12
  5	 G 	   298.65	   573.82	 11
  6	 L 	   355.19	   545.31	 10
  7	 V 	   404.72	   488.77	  9
  8	 Q 	   468.75	   439.23	  8
  9	 L 	   525.29	   375.21	  7
 10	 S 	   568.81	   318.66	  6
 11	 S 	   612.33	   275.15	  5
 12	 D 	   669.84	   231.63	  4
 13	 L 	   726.38	   174.12	  3
 14	 S 	   769.90	   117.58	  2
 15	 K 	        -  	    74.06	  1  _
```

**19. IPI00000914 Isoform 1 of Calcitonin**

| Sequence | Charge | M+H+ | XCorr | DeltaCn |
| --- | --- | --- | --- | --- |
| R.LLLAALVQDYVQMK.A | 2 | 1605.967 | 3.403 | 0.348 |

+1 charge

```
		      B   	       Y
  1	 L 	   114.09	        -  	 14
  2	 L 	   227.18	  1491.82	 13
  3	 L 	   340.26	  1378.74	 12
  4	 A 	   411.30	  1265.66	 11
  5	 A 	   482.33	  1194.62	 10
  6	 L 	   595.42	  1123.58	  9
  7	 V 	   694.49	  1010.50	  8
  8	 Q 	   822.54	   911.43	  7
  9	 D 	   937.57	   783.37	  6
 10	 Y 	  1100.64	   668.34	  5
 11	 V 	  1199.70	   505.28	  4
 12	 Q 	  1327.76	   406.21	  3
 13	 M 	  1458.80	   278.15	  2
 14	 K 	        -  	   147.11	  1  _
+2 charge
		      B   	       Y
  1	 L 	    57.55	        -  	 14
  2	 L 	   114.09	   746.42	 13
  3	 L 	   170.63	   689.87	 12
  4	 A 	   206.15	   633.33	 11
  5	 A 	   241.67	   597.81	 10
  6	 L 	   298.21	   562.29	  9
  7	 V 	   347.75	   505.75	  8
  8	 Q 	   411.78	   456.22	  7
  9	 D 	   469.29	   392.19	  6
 10	 Y 	   550.82	   334.68	  5
 11	 V 	   600.36	   253.14	  4
 12	 Q 	   664.38	   203.61	  3
 13	 M 	   729.90	   139.58	  2
 14	 K 	        -  	    74.06	  1  _
```

**20. IPI00006705 Uteroglobin**

| Sequence | Charge | M+H+ | XCorr | DeltaCn |
| --- | --- | --- | --- | --- |
| R.VIETLLMDTPSSYEAAMELFSPDQDMR.E | 3 | 3091.48 | 4.612 | 0.468 |

+1 charge

```
		      B   	       Y
  1	 V 	   100.08	        -  	 27
  2	 I 	   213.16	  2990.35	 26
  3	 E 	   342.20	  2877.26	 25
  4	 T 	   443.25	  2748.22	 24
  5	 L 	   556.33	  2647.17	 23
  6	 L 	   669.42	  2534.09	 22
  7	 M 	   800.46	  2421.00	 21
  8	 D 	   915.49	  2289.96	 20
  9	 T 	  1016.53	  2174.94	 19
 10	 P 	  1113.59	  2073.89	 18
 11	 S 	  1200.62	  1976.84	 17
 12	 S 	  1287.65	  1889.80	 16
 13	 Y 	  1450.71	  1802.77	 15
 14	 E 	  1579.76	  1639.71	 14
 15	 A 	  1650.79	  1510.67	 13
 16	 A 	  1721.83	  1439.63	 12
 17	 M 	  1852.87	  1368.59	 11
 18	 E 	  1981.91	  1237.55	 10
 19	 L 	  2095.00	  1108.51	  9
 20	 F 	  2242.07	   995.43	  8
 21	 S 	  2329.10	   848.36	  7
 22	 P 	  2426.15	   761.32	  6
 23	 D 	  2541.18	   664.27	  5
 24	 Q 	  2669.24	   549.24	  4
 25	 D 	  2784.26	   421.19	  3
 26	 M 	  2915.30	   306.16	  2
 27	 R 	        -  	   175.12	  1  _


+2 charge
		      B   	       Y
  1	 V 	    50.54	        -  	 27
  2	 I 	   107.08	  1495.68	 26
  3	 E 	   171.60	  1439.13	 25
  4	 T 	   222.13	  1374.61	 24
  5	 L 	   278.67	  1324.09	 23
  6	 L 	   335.21	  1267.55	 22
  7	 M 	   400.73	  1211.01	 21
  8	 D 	   458.25	  1145.49	 20
  9	 T 	   508.77	  1087.97	 19
 10	 P 	   557.30	  1037.45	 18
 11	 S 	   600.81	   988.92	 17
 12	 S 	   644.33	   945.41	 16
 13	 Y 	   725.86	   901.89	 15
 14	 E 	   790.38	   820.36	 14
 15	 A 	   825.90	   755.84	 13
 16	 A 	   861.42	   720.32	 12
 17	 M 	   926.94	   684.80	 11
 18	 E 	   991.46	   619.28	 10
 19	 L 	  1048.00	   554.76	  9
 20	 F 	  1121.54	   498.22	  8
 21	 S 	  1165.05	   424.68	  7
 22	 P 	  1213.58	   381.17	  6
 23	 D 	  1271.09	   332.64	  5
 24	 Q 	  1335.12	   275.13	  4
 25	 D 	  1392.64	   211.10	  3
 26	 M 	  1458.16	   153.58	  2
 27	 R 	        -  	    88.06	  1  _


+3 charge
		      B   	       Y
  1	 V 	    34.03	        -  	 27
  2	 I 	    71.72	   997.45	 26
  3	 E 	   114.74	   959.76	 25
  4	 T 	   148.42	   916.74	 24
  5	 L 	   186.12	   883.06	 23
  6	 L 	   223.81	   845.37	 22
  7	 M 	   267.49	   807.67	 21
  8	 D 	   305.83	   763.99	 20
  9	 T 	   339.52	   725.65	 19
 10	 P 	   371.87	   691.97	 18
 11	 S 	   400.88	   659.62	 17
 12	 S 	   429.89	   630.61	 16
 13	 Y 	   484.24	   601.60	 15
 14	 E 	   527.26	   547.24	 14
 15	 A 	   550.94	   504.23	 13
 16	 A 	   574.61	   480.55	 12
 17	 M 	   618.30	   456.87	 11
 18	 E 	   661.31	   413.19	 10
 19	 L 	   699.00	   370.17	  9
 20	 F 	   748.03	   332.48	  8
 21	 S 	   777.04	   283.46	  7
 22	 P 	   809.39	   254.45	  6
 23	 D 	   847.73	   222.10	  5
 24	 Q 	   890.42	   183.75	  4
 25	 D 	   928.76	   141.07	  3
 26	 M 	   972.44	   102.72	  2
 27	 R 	        -  	    59.04	  1  _
```

**21. IPI00639874 Isoform 2 of Serine/threonine-protein kinase SMG1**

| Sequence | Charge | M+H+ | XCorr | DeltaCn |
| --- | --- | --- | --- | --- |
| K.LLMTWALEAAVLMRK.S | 3 | 1747.205 | 3.318 | 0.257 |

+1 charge

```
		      B   	       Y
  1	 L 	   114.09	        -  	 15
  2	 L 	   227.18	  1632.90	 14
  3	 M 	   358.22	  1519.81	 13
  4	 T 	   459.26	  1388.77	 12
  5	 W 	   645.34	  1287.72	 11
  6	 A 	   716.38	  1101.64	 10
  7	 L 	   829.46	  1030.61	  9
  8	 E 	   958.51	   917.52	  8
  9	 A 	  1029.54	   788.48	  7
 10	 A 	  1100.58	   717.44	  6
 11	 V 	  1199.65	   646.41	  5
 12	 L 	  1312.73	   547.34	  4
 13	 M 	  1443.77	   434.25	  3
 14	 R 	  1599.87	   303.21	  2
 15	 K 	        -  	   147.11	  1  _
+2 charge
		      B   	       Y
  1	 L 	    57.55	        -  	 15
  2	 L 	   114.09	   816.95	 14
  3	 M 	   179.61	   760.41	 13
  4	 T 	   230.14	   694.89	 12
  5	 W 	   323.18	   644.37	 11
  6	 A 	   358.69	   551.33	 10
  7	 L 	   415.24	   515.81	  9
  8	 E 	   479.76	   459.27	  8
  9	 A 	   515.28	   394.74	  7
 10	 A 	   550.79	   359.23	  6
 11	 V 	   600.33	   323.71	  5
 12	 L 	   656.87	   274.17	  4
 13	 M 	   722.39	   217.63	  3
 14	 R 	   800.44	   152.11	  2
 15	 K 	        -  	    74.06	  1  _
+3 charge
		      B   	       Y
  1	 L 	    38.70	        -  	 15
  2	 L 	    76.40	   544.97	 14
  3	 M 	   120.08	   507.28	 13
  4	 T 	   153.76	   463.60	 12
  5	 W 	   215.79	   429.91	 11
  6	 A 	   239.46	   367.89	 10
  7	 L 	   277.16	   344.21	  9
  8	 E 	   320.17	   306.51	  8
  9	 A 	   343.85	   263.50	  7
 10	 A 	   367.53	   239.82	  6
 11	 V 	   400.55	   216.14	  5
 12	 L 	   438.25	   183.12	  4
 13	 M 	   481.93	   145.42	  3
 14	 R 	   533.96	   101.74	  2
 15	 K 	        -  	    49.71	  1  _
```

**22. IPI00555812 Isoform 1 of Vitamin D-binding protein**

| Sequence | Charge | M+H+ | XCorr | DeltaCn |
| --- | --- | --- | --- | --- |
| K.LAQKVPTADLEDVLPLAEDITNILSK.C | 3 | 2808.217 | 5.077 | 0.501 |

+1 charge

```
		      B   	       Y
  1	 L 	   114.09	        -  	 26
  2	 A 	   185.13	  2693.46	 25
  3	 Q 	   313.19	  2622.42	 24
  4	 K 	   441.28	  2494.37	 23
  5	 V 	   540.35	  2366.27	 22
  6	 P 	   637.40	  2267.20	 21
  7	 T 	   738.45	  2170.15	 20
  8	 A 	   809.49	  2069.10	 19
  9	 D 	   924.51	  1998.06	 18
 10	 L 	  1037.60	  1883.04	 17
 11	 E 	  1166.64	  1769.95	 16
 12	 D 	  1281.67	  1640.91	 15
 13	 V 	  1380.74	  1525.88	 14
 14	 L 	  1493.82	  1426.82	 13
 15	 P 	  1590.87	  1313.73	 12
 16	 L 	  1703.96	  1216.68	 11
 17	 A 	  1774.99	  1103.59	 10
 18	 E 	  1904.04	  1032.56	  9
 19	 D 	  2019.06	   903.51	  8
 20	 I 	  2132.15	   788.49	  7
 21	 T 	  2233.20	   675.40	  6
 22	 N 	  2347.24	   574.36	  5
 23	 I 	  2460.32	   460.31	  4
 24	 L 	  2573.41	   347.23	  3
 25	 S 	  2660.44	   234.14	  2
 26	 K 	        -  	   147.11	  1  _


+2 charge
		      B   	       Y
  1	 L 	    57.55	        -  	 26
  2	 A 	    93.07	  1347.23	 25
  3	 Q 	   157.10	  1311.72	 24
  4	 K 	   221.14	  1247.69	 23
  5	 V 	   270.68	  1183.64	 22
  6	 P 	   319.21	  1134.10	 21
  7	 T 	   369.73	  1085.58	 20
  8	 A 	   405.25	  1035.05	 19
  9	 D 	   462.76	   999.54	 18
 10	 L 	   519.30	   942.02	 17
 11	 E 	   583.82	   885.48	 16
 12	 D 	   641.34	   820.96	 15
 13	 V 	   690.87	   763.45	 14
 14	 L 	   747.41	   713.91	 13
 15	 P 	   795.94	   657.37	 12
 16	 L 	   852.48	   608.84	 11
 17	 A 	   888.00	   552.30	 10
 18	 E 	   952.52	   516.78	  9
 19	 D 	  1010.04	   452.26	  8
 20	 I 	  1066.58	   394.75	  7
 21	 T 	  1117.10	   338.21	  6
 22	 N 	  1174.12	   287.68	  5
 23	 I 	  1230.67	   230.66	  4
 24	 L 	  1287.21	   174.12	  3
 25	 S 	  1330.72	   117.58	  2
 26	 K 	        -  	    74.06	  1  _
+3 charge
		      B   	       Y
  1	 L 	    38.70	        -  	 26
  2	 A 	    62.38	   898.49	 25
  3	 Q 	   105.07	   874.81	 24
  4	 K 	   147.77	   832.13	 23
  5	 V 	   180.79	   789.43	 22
  6	 P 	   213.14	   756.41	 21
  7	 T 	   246.82	   724.05	 20
  8	 A 	   270.50	   690.37	 19
  9	 D 	   308.84	   666.69	 18
 10	 L 	   346.54	   628.35	 17
 11	 E 	   389.55	   590.66	 16
 12	 D 	   427.89	   547.64	 15
 13	 V 	   460.92	   509.30	 14
 14	 L 	   498.61	   476.28	 13
 15	 P 	   530.96	   438.58	 12
 16	 L 	   568.66	   406.23	 11
 17	 A 	   592.34	   368.54	 10
 18	 E 	   635.35	   344.86	  9
 19	 D 	   673.69	   301.84	  8
 20	 I 	   711.39	   263.50	  7
 21	 T 	   745.07	   225.81	  6
 22	 N 	   783.08	   192.12	  5
 23	 I 	   820.78	   154.11	  4
 24	 L 	   858.47	   116.41	  3
 25	 S 	   887.48	    78.72	  2
 26	 K 	        -  	    49.71	  1  _
```

**23. IPI00022283 Trefoil factor 1**

| Sequence | Charge | M+H+ | XCorr | DeltaCn |
| --- | --- | --- | --- | --- |
| R.QNCGFPGVTPSQCANK.G | 2 | 1765.893 | 4.01 | 0.512 |

+1 charge

```
		      B   	       Y
  1	 Q 	   129.07	        -  	 16
  2	 N 	   243.11	  1636.99	 15
  3	 C 	   403.27	  1522.95	 14
  4	 G 	   460.30	  1362.78	 13
  5	 F 	   607.36	  1305.76	 12
  6	 P 	   704.42	  1158.69	 11
  7	 G 	   761.44	  1061.64	 10
  8	 V 	   860.51	  1004.62	  9
  9	 T 	   961.55	   905.55	  8
 10	 P 	  1058.61	   804.50	  7
 11	 S 	  1145.64	   707.45	  6
 12	 Q 	  1273.70	   620.42	  5
 13	 C 	  1433.86	   492.36	  4
 14	 A 	  1504.90	   332.19	  3
 15	 N 	  1618.94	   261.16	  2
 16	 K 	        -  	   147.11	  1  _
+2 charge
		      B   	       Y
  1	 Q 	    65.04	        -  	 16
  2	 N 	   122.06	   819.00	 15
  3	 C 	   202.14	   761.98	 14
  4	 G 	   230.65	   681.89	 13
  5	 F 	   304.19	   653.38	 12
  6	 P 	   352.71	   579.85	 11
  7	 G 	   381.22	   531.32	 10
  8	 V 	   430.76	   502.81	  9
  9	 T 	   481.28	   453.28	  8
 10	 P 	   529.81	   402.75	  7
 11	 S 	   573.32	   354.23	  6
 12	 Q 	   637.35	   310.71	  5
 13	 C 	   717.44	   246.68	  4
 14	 A 	   752.95	   166.60	  3
 15	 N 	   809.98	   131.08	  2
 16	 K 	        -  	    74.06	  1  _
```

**24. IPI00027409 Myeloblastin**

| Sequence | Charge | M+H+ | XCorr | DeltaCn |
| --- | --- | --- | --- | --- |
| R.LVNVVLGAHNVR.T | 2 | 1291.528 | 3.589 | 0.411 |

+1 charge

```
		      B   	       Y
  1	 L 	   114.09	        -  	 12
  2	 V 	   213.16	  1177.68	 11
  3	 N 	   327.20	  1078.61	 10
  4	 V 	   426.27	   964.57	  9
  5	 V 	   525.34	   865.50	  8
  6	 L 	   638.42	   766.43	  7
  7	 G 	   695.45	   653.35	  6
  8	 A 	   766.48	   596.33	  5
  9	 H 	   903.54	   525.29	  4
 10	 N 	  1017.58	   388.23	  3
 11	 V 	  1116.65	   274.19	  2
 12	 R 	        -  	   175.12	  1  _
+2 charge
		      B   	       Y
  1	 L 	    57.55	        -  	 12
  2	 V 	   107.08	   589.34	 11
  3	 N 	   164.10	   539.81	 10
  4	 V 	   213.64	   482.79	  9
  5	 V 	   263.17	   433.25	  8
  6	 L 	   319.72	   383.72	  7
  7	 G 	   348.23	   327.18	  6
  8	 A 	   383.74	   298.67	  5
  9	 H 	   452.27	   263.15	  4
 10	 N 	   509.30	   194.62	  3
 11	 V 	   558.83	   137.60	  2
 12	 R 	        -  	    88.06	  1  _
```

**25. IPI00007664 Plasma glutamate carboxypeptidase**

| Sequence | Charge | M+H+ | XCorr | DeltaCn |
| --- | --- | --- | --- | --- |
| K.AIINLAVYGK.A | 1 | 1062.287 | 2.268 | 0.441 |

+1 charge

```
		      B   	       Y
  1	 A 	    72.04	        -  	 10
  2	 I 	   185.13	   990.60	  9
  3	 I 	   298.21	   877.51	  8
  4	 N 	   412.26	   764.43	  7
  5	 L 	   525.34	   650.39	  6
  6	 A 	   596.38	   537.30	  5
  7	 V 	   695.45	   466.27	  4
  8	 Y 	   858.51	   367.20	  3
  9	 G 	   915.53	   204.13	  2
 10	 K 	        -  	   147.11	  1  _
```

**26. IPI00816799 Rheumatoid factor D5 light chain (Fragment)**

| Sequence | Charge | M+H+ | XCorr | DeltaCn |
| --- | --- | --- | --- | --- |
| - .EIVLTQSPATLSLSPGER.A | 2 | 1899.135 | 3.465 | 0.412 |

+1 charge

```
		      B   	       Y
  1	 E 	   130.05	        -  	 18
  2	 I 	   243.13	  1768.98	 17
  3	 V 	   342.20	  1655.90	 16
  4	 L 	   455.29	  1556.83	 15
  5	 T 	   556.33	  1443.74	 14
  6	 Q 	   684.39	  1342.70	 13
  7	 S 	   771.42	  1214.64	 12
  8	 P 	   868.48	  1127.61	 11
  9	 A 	   939.51	  1030.55	 10
 10	 T 	  1040.56	   959.52	  9
 11	 L 	  1153.65	   858.47	  8
 12	 S 	  1240.68	   745.38	  7
 13	 L 	  1353.76	   658.35	  6
 14	 S 	  1440.79	   545.27	  5
 15	 P 	  1537.85	   458.24	  4
 16	 G 	  1594.87	   361.18	  3
 17	 E 	  1723.91	   304.16	  2
 18	 R 	        -  	   175.12	  1  _
+2 charge
		      B   	       Y
  1	 E 	    65.53	        -  	 18
  2	 I 	   122.07	   884.99	 17
  3	 V 	   171.60	   828.45	 16
  4	 L 	   228.15	   778.92	 15
  5	 T 	   278.67	   722.38	 14
  6	 Q 	   342.70	   671.85	 13
  7	 S 	   386.22	   607.82	 12
  8	 P 	   434.74	   564.31	 11
  9	 A 	   470.26	   515.78	 10
 10	 T 	   520.78	   480.26	  9
 11	 L 	   577.33	   429.74	  8
 12	 S 	   620.84	   373.20	  7
 13	 L 	   677.38	   329.68	  6
 14	 S 	   720.90	   273.14	  5
 15	 P 	   769.43	   229.62	  4
 16	 G 	   797.94	   181.10	  3
 17	 E 	   862.46	   152.58	  2
 18	 R 	        -  	    88.06	  1  _
```

**27. IPI00014048 Ribonuclease pancreatic**

| Sequence | Charge | M+H+ | XCorr | DeltaCn |
| --- | --- | --- | --- | --- |
| R.CKPVNTFVHEPLVDVQNVCFQEK.V | 3 | 2789.123 | 5.748 | 0.628 |

+1 charge

```
		      B   	       Y
  1	 C 	   161.17	        -  	 23
  2	 K 	   289.27	  2627.46	 22
  3	 P 	   386.32	  2499.37	 21
  4	 V 	   485.39	  2402.32	 20
  5	 N 	   599.43	  2303.25	 19
  6	 T 	   700.48	  2189.20	 18
  7	 F 	   847.55	  2088.16	 17
  8	 V 	   946.62	  1941.09	 16
  9	 H 	  1083.67	  1842.02	 15
 10	 E 	  1212.72	  1704.96	 14
 11	 P 	  1309.77	  1575.92	 13
 12	 L 	  1422.85	  1478.87	 12
 13	 V 	  1521.92	  1365.78	 11
 14	 D 	  1636.95	  1266.71	 10
 15	 V 	  1736.02	  1151.69	  9
 16	 Q 	  1864.08	  1052.62	  8
 17	 N 	  1978.12	   924.56	  7
 18	 V 	  2077.19	   810.52	  6
 19	 C 	  2237.35	   711.45	  5
 20	 F 	  2384.42	   551.28	  4
 21	 Q 	  2512.48	   404.21	  3
 22	 E 	  2641.52	   276.16	  2
 23	 K 	        -  	   147.11	  1  _


+2 charge
		      B   	       Y
  1	 C 	    81.09	        -  	 23
  2	 K 	   145.14	  1314.24	 22
  3	 P 	   193.66	  1250.19	 21
  4	 V 	   243.20	  1201.66	 20
  5	 N 	   300.22	  1152.13	 19
  6	 T 	   350.74	  1095.11	 18
  7	 F 	   424.28	  1044.58	 17
  8	 V 	   473.81	   971.05	 16
  9	 H 	   542.34	   921.51	 15
 10	 E 	   606.86	   852.98	 14
 11	 P 	   655.39	   788.46	 13
 12	 L 	   711.93	   739.94	 12
 13	 V 	   761.47	   683.39	 11
 14	 D 	   818.98	   633.86	 10
 15	 V 	   868.51	   576.35	  9
 16	 Q 	   932.54	   526.81	  8
 17	 N 	   989.56	   462.78	  7
 18	 V 	  1039.10	   405.76	  6
 19	 C 	  1119.18	   356.23	  5
 20	 F 	  1192.71	   276.14	  4
 21	 Q 	  1256.74	   202.61	  3
 22	 E 	  1321.27	   138.58	  2
 23	 K 	        -  	    74.06	  1  _
+3 charge
		      B   	       Y
  1	 C 	    54.40	        -  	 23
  2	 K 	    97.09	   876.49	 22
  3	 P 	   129.44	   833.79	 21
  4	 V 	   162.47	   801.44	 20
  5	 N 	   200.48	   768.42	 19
  6	 T 	   234.16	   730.41	 18
  7	 F 	   283.19	   696.72	 17
  8	 V 	   316.21	   647.70	 16
  9	 H 	   361.90	   614.68	 15
 10	 E 	   404.91	   568.99	 14
 11	 P 	   437.26	   525.98	 13
 12	 L 	   474.96	   493.63	 12
 13	 V 	   507.98	   455.93	 11
 14	 D 	   546.32	   422.91	 10
 15	 V 	   579.34	   384.57	  9
 16	 Q 	   622.03	   351.54	  8
 17	 N 	   660.04	   308.86	  7
 18	 V 	   693.07	   270.84	  6
 19	 C 	   746.46	   237.82	  5
 20	 F 	   795.48	   184.43	  4
 21	 Q 	   838.16	   135.41	  3
 22	 E 	   881.18	    92.72	  2
 23	 K 	        -  	    49.71	  1  _
```

**28. IPI00292530 Inter-alpha-trypsin inhibitor heavy chain H1**

| Sequence | Charge | M+H+ | XCorr | DeltaCn |
| --- | --- | --- | --- | --- |
| R.GFSLDEATNLNGGLLR.G | 2 | 1677.84 | 3.068 | 0.328 |

+1 charge

```
		      B   	       Y
  1	 G 	    58.03	        -  	 16
  2	 F 	   205.10	  1619.84	 15
  3	 S 	   292.13	  1472.77	 14
  4	 L 	   405.21	  1385.74	 13
  5	 D 	   520.24	  1272.65	 12
  6	 E 	   649.28	  1157.63	 11
  7	 A 	   720.32	  1028.58	 10
  8	 T 	   821.37	   957.55	  9
  9	 N 	   935.41	   856.50	  8
 10	 L 	  1048.49	   742.46	  7
 11	 N 	  1162.54	   629.37	  6
 12	 G 	  1219.56	   515.33	  5
 13	 G 	  1276.58	   458.31	  4
 14	 L 	  1389.66	   401.29	  3
 15	 L 	  1502.75	   288.20	  2
 16	 R 	        -  	   175.12	  1  _
+2 charge
		      B   	       Y
  1	 G 	    29.52	        -  	 16
  2	 F 	   103.05	   810.42	 15
  3	 S 	   146.57	   736.89	 14
  4	 L 	   203.11	   693.37	 13
  5	 D 	   260.62	   636.83	 12
  6	 E 	   325.15	   579.32	 11
  7	 A 	   360.66	   514.80	 10
  8	 T 	   411.19	   479.28	  9
  9	 N 	   468.21	   428.75	  8
 10	 L 	   524.75	   371.73	  7
 11	 N 	   581.77	   315.19	  6
 12	 G 	   610.28	   258.17	  5
 13	 G 	   638.79	   229.66	  4
 14	 L 	   695.34	   201.15	  3
 15	 L 	   751.88	   144.61	  2
 16	 R 	        -  	    88.06	  1  _
```

**29. IPI00336112 Isoform 1 of Mucin and cadherin-like protein**

| Sequence | Charge | M+H+ | XCorr | DeltaCn |
| --- | --- | --- | --- | --- |
| R.VFVSVLDVNDNAPEFPFK.T | 2 | 2038.288 | 3.29 | 0.544 |

+1 charge

```
		      B   	       Y
  1	 V 	   100.08	        -  	 18
  2	 F 	   247.14	  1937.96	 17
  3	 V 	   346.21	  1790.90	 16
  4	 S 	   433.24	  1691.83	 15
  5	 V 	   532.31	  1604.80	 14
  6	 L 	   645.40	  1505.73	 13
  7	 D 	   760.42	  1392.64	 12
  8	 V 	   859.49	  1277.62	 11
  9	 N 	   973.54	  1178.55	 10
 10	 D 	  1088.56	  1064.50	  9
 11	 N 	  1202.61	   949.48	  8
 12	 A 	  1273.64	   835.43	  7
 13	 P 	  1370.70	   764.40	  6
 14	 E 	  1499.74	   667.34	  5
 15	 F 	  1646.81	   538.30	  4
 16	 P 	  1743.86	   391.23	  3
 17	 F 	  1890.93	   294.18	  2
 18	 K 	        -  	   147.11	  1  _
+2 charge
		      B   	       Y
  1	 V 	    50.54	        -  	 18
  2	 F 	   124.08	   969.49	 17
  3	 V 	   173.61	   895.95	 16
  4	 S 	   217.13	   846.42	 15
  5	 V 	   266.66	   802.90	 14
  6	 L 	   323.20	   753.37	 13
  7	 D 	   380.72	   696.83	 12
  8	 V 	   430.25	   639.31	 11
  9	 N 	   487.27	   589.78	 10
 10	 D 	   544.78	   532.76	  9
 11	 N 	   601.81	   475.24	  8
 12	 A 	   637.32	   418.22	  7
 13	 P 	   685.85	   382.70	  6
 14	 E 	   750.37	   334.18	  5
 15	 F 	   823.91	   269.65	  4
 16	 P 	   872.43	   196.12	  3
 17	 F 	   945.97	   147.59	  2
 18	 K 	        -  	    74.06	  1  _
```

**30. IPI00419215 Alpha-2-macroglobulin-like protein 1**

| Sequence | Charge | M+H+ | XCorr | DeltaCn |
| --- | --- | --- | --- | --- |
| K.KVEFGTDTLNIYLDELIK.N | 2 | 2112.408 | 2.997 | 0.482 |

+1 charge

```
		      B   	       Y
  1	 K 	   129.10	        -  	 18
  2	 V 	   228.17	  1983.03	 17
  3	 E 	   357.21	  1883.96	 16
  4	 F 	   504.28	  1754.92	 15
  5	 G 	   561.30	  1607.85	 14
  6	 T 	   662.35	  1550.83	 13
  7	 D 	   777.38	  1449.78	 12
  8	 T 	   878.43	  1334.76	 11
  9	 L 	   991.51	  1233.71	 10
 10	 N 	  1105.55	  1120.62	  9
 11	 I 	  1218.64	  1006.58	  8
 12	 Y 	  1381.70	   893.50	  7
 13	 L 	  1494.78	   730.43	  6
 14	 D 	  1609.81	   617.35	  5
 15	 E 	  1738.85	   502.32	  4
 16	 L 	  1851.94	   373.28	  3
 17	 I 	  1965.02	   260.20	  2
 18	 K 	        -  	   147.11	  1  _
+2 charge
		      B   	       Y
  1	 K 	    65.05	        -  	 18
  2	 V 	   114.59	   992.02	 17
  3	 E 	   179.11	   942.49	 16
  4	 F 	   252.64	   877.96	 15
  5	 G 	   281.16	   804.43	 14
  6	 T 	   331.68	   775.92	 13
  7	 D 	   389.19	   725.40	 12
  8	 T 	   439.72	   667.88	 11
  9	 L 	   496.26	   617.36	 10
 10	 N 	   553.28	   560.82	  9
 11	 I 	   609.82	   503.79	  8
 12	 Y 	   691.35	   447.25	  7
 13	 L 	   747.90	   365.72	  6
 14	 D 	   805.41	   309.18	  5
 15	 E 	   869.93	   251.67	  4
 16	 L 	   926.47	   187.14	  3
 17	 I 	   983.01	   130.60	  2
 18	 K 	        -  	    74.06	  1  _
```

**31. IPI00030786 Isoform 1 of Matrilin-4**

| Sequence | Charge | M+H+ | XCorr | DeltaCn |
| --- | --- | --- | --- | --- |
| R.TLGALESLTLNLAQLTAR.L | 2 | 1886.183 | 4.05 | 0.524 |

+1 charge

```
		      B   	       Y
  1	 T 	   102.05	        -  	 18
  2	 L 	   215.14	  1784.03	 17
  3	 G 	   272.16	  1670.94	 16
  4	 A 	   343.20	  1613.92	 15
  5	 L 	   456.28	  1542.88	 14
  6	 E 	   585.32	  1429.80	 13
  7	 S 	   672.36	  1300.76	 12
  8	 L 	   785.44	  1213.73	 11
  9	 T 	   886.49	  1100.64	 10
 10	 L 	   999.57	   999.59	  9
 11	 N 	  1113.62	   886.51	  8
 12	 L 	  1226.70	   772.47	  7
 13	 A 	  1297.74	   659.38	  6
 14	 Q 	  1425.79	   588.35	  5
 15	 L 	  1538.88	   460.29	  4
 16	 T 	  1639.93	   347.20	  3
 17	 A 	  1710.96	   246.16	  2
 18	 R 	        -  	   175.12	  1  _
+2 charge
		      B   	       Y
  1	 T 	    51.53	        -  	 18
  2	 L 	   108.07	   892.52	 17
  3	 G 	   136.58	   835.98	 16
  4	 A 	   172.10	   807.46	 15
  5	 L 	   228.64	   771.95	 14
  6	 E 	   293.17	   715.40	 13
  7	 S 	   336.68	   650.88	 12
  8	 L 	   393.22	   607.37	 11
  9	 T 	   443.75	   550.82	 10
 10	 L 	   500.29	   500.30	  9
 11	 N 	   557.31	   443.76	  8
 12	 L 	   613.85	   386.74	  7
 13	 A 	   649.37	   330.20	  6
 14	 Q 	   713.40	   294.68	  5
 15	 L 	   769.94	   230.65	  4
 16	 T 	   820.47	   174.11	  3
 17	 A 	   855.99	   123.58	  2
 18	 R 	        -  	    88.06	  1  _
```

**32. IPI00297252 Isoform 1 of Extracellular sulfatase Sulf-2**

| Sequence | Charge | M+H+ | XCorr | DeltaCn |
| --- | --- | --- | --- | --- |
| R.VYHVGLGDAAQPR.N | 2 | 1383.537 | 3.593 | 0.384 |

+1 charge

```
		      B   	       Y
  1	 V 	   100.08	        -  	 13
  2	 Y 	   263.14	  1283.65	 12
  3	 H 	   400.20	  1120.59	 11
  4	 V 	   499.27	   983.53	 10
  5	 G 	   556.29	   884.46	  9
  6	 L 	   669.37	   827.44	  8
  7	 G 	   726.39	   714.35	  7
  8	 D 	   841.42	   657.33	  6
  9	 A 	   912.46	   542.30	  5
 10	 A 	   983.49	   471.27	  4
 11	 Q 	  1111.55	   400.23	  3
 12	 P 	  1208.61	   272.17	  2
 13	 R 	        -  	   175.12	  1  _
+2 charge
		      B   	       Y
  1	 V 	    50.54	        -  	 13
  2	 Y 	   132.07	   642.33	 12
  3	 H 	   200.60	   560.80	 11
  4	 V 	   250.14	   492.27	 10
  5	 G 	   278.65	   442.73	  9
  6	 L 	   335.19	   414.22	  8
  7	 G 	   363.70	   357.68	  7
  8	 D 	   421.21	   329.17	  6
  9	 A 	   456.73	   271.66	  5
 10	 A 	   492.25	   236.14	  4
 11	 Q 	   556.28	   200.62	  3
 12	 P 	   604.81	   136.59	  2
 13	 R 	        -  	    88.06	  1  _
```

**33. IPI00028030 Cartilage oligomeric matrix protein**

| Sequence | Charge | M+H+ | XCorr | DeltaCn |
| --- | --- | --- | --- | --- |
| K.NTVMECDACGMQQSVR.T | 2 | 1887.073 | 2.989 | 0.446 |

+1 charge

```
		      B   	       Y
  1	 N 	   115.05	        -  	 16
  2	 T 	   216.10	  1771.99	 15
  3	 V 	   315.17	  1670.94	 14
  4	 M 	   446.21	  1571.88	 13
  5	 E 	   575.25	  1440.84	 12
  6	 C 	   735.41	  1311.79	 11
  7	 D 	   850.44	  1151.63	 10
  8	 A 	   921.48	  1036.60	  9
  9	 C 	  1081.64	   965.56	  8
 10	 G 	  1138.67	   805.40	  7
 11	 M 	  1269.71	   748.38	  6
 12	 Q 	  1397.76	   617.34	  5
 13	 Q 	  1525.82	   489.28	  4
 14	 S 	  1612.86	   361.22	  3
 15	 V 	  1711.92	   274.19	  2
 16	 R 	        -  	   175.12	  1  _
+2 charge
		      B   	       Y
  1	 N 	    58.03	        -  	 16
  2	 T 	   108.55	   886.50	 15
  3	 V 	   158.09	   835.98	 14
  4	 M 	   223.61	   786.44	 13
  5	 E 	   288.13	   720.92	 12
  6	 C 	   368.21	   656.40	 11
  7	 D 	   425.72	   576.32	 10
  8	 A 	   461.24	   518.80	  9
  9	 C 	   541.33	   483.29	  8
 10	 G 	   569.84	   403.20	  7
 11	 M 	   635.36	   374.69	  6
 12	 Q 	   699.39	   309.17	  5
 13	 Q 	   763.42	   245.14	  4
 14	 S 	   806.93	   181.11	  3
 15	 V 	   856.47	   137.60	  2
 16	 R 	        -  	    88.06	  1  _
```

**34. IPI00298388 Isoform 1 of Phosphoinositide-3-kinase-interacting protein 1**

| Sequence | Charge | M+H+ | XCorr | DeltaCn |
| --- | --- | --- | --- | --- |
| R.SEAAAVQPVIGISQR.V | 2 | 1526.72 | 4.24 | 0.458 |

+1 charge

```
		      B   	       Y
  1	 S 	    88.04	        -  	 15
  2	 E 	   217.08	  1438.80	 14
  3	 A 	   288.12	  1309.76	 13
  4	 A 	   359.16	  1238.72	 12
  5	 A 	   430.19	  1167.68	 11
  6	 V 	   529.26	  1096.65	 10
  7	 Q 	   657.32	   997.58	  9
  8	 P 	   754.37	   869.52	  8
  9	 V 	   853.44	   772.47	  7
 10	 I 	   966.53	   673.40	  6
 11	 G 	  1023.55	   560.32	  5
 12	 I 	  1136.63	   503.29	  4
 13	 S 	  1223.66	   390.21	  3
 14	 Q 	  1351.72	   303.18	  2
 15	 R 	        -  	   175.12	  1  _
+2 charge
		      B   	       Y
  1	 S 	    44.52	        -  	 15
  2	 E 	   109.04	   719.90	 14
  3	 A 	   144.56	   655.38	 13
  4	 A 	   180.08	   619.86	 12
  5	 A 	   215.60	   584.35	 11
  6	 V 	   265.13	   548.83	 10
  7	 Q 	   329.16	   499.29	  9
  8	 P 	   377.69	   435.26	  8
  9	 V 	   427.22	   386.74	  7
 10	 I 	   483.77	   337.20	  6
 11	 G 	   512.28	   280.66	  5
 12	 I 	   568.82	   252.15	  4
 13	 S 	   612.34	   195.61	  3
 14	 Q 	   676.36	   152.09	  2
 15	 R 	        -  	    88.06	  1  _
```

**35. IPI00022284 Major prion protein**

| Sequence | Charge | M+H+ | XCorr | DeltaCn |
| --- | --- | --- | --- | --- |
| R.VVEQMCITQYER.E | 2 | 1556.76 | 3.8 | 0.491 |

+1 charge

```
		      B   	       Y
  1	 V 	   100.08	        -  	 12
  2	 V 	   199.14	  1456.79	 11
  3	 E 	   328.19	  1357.72	 10
  4	 Q 	   456.25	  1228.68	  9
  5	 M 	   587.29	  1100.62	  8
  6	 C 	   747.45	   969.58	  7
  7	 I 	   860.54	   809.42	  6
  8	 T 	   961.58	   696.33	  5
  9	 Q 	  1089.64	   595.28	  4
 10	 Y 	  1252.70	   467.22	  3
 11	 E 	  1381.75	   304.16	  2
 12	 R 	        -  	   175.12	  1  _
+2 charge
		      B   	       Y
  1	 V 	    50.54	        -  	 12
  2	 V 	   100.08	   728.90	 11
  3	 E 	   164.60	   679.36	 10
  4	 Q 	   228.63	   614.84	  9
  5	 M 	   294.15	   550.81	  8
  6	 C 	   374.23	   485.29	  7
  7	 I 	   430.77	   405.21	  6
  8	 T 	   481.30	   348.67	  5
  9	 Q 	   545.32	   298.15	  4
 10	 Y 	   626.86	   234.12	  3
 11	 E 	   691.38	   152.58	  2
 12	 R 	        -  	    88.06	  1  _
```

**36. IPI00066856 Ly6/PLAUR domain-containing protein 2**

| Sequence | Charge | M+H+ | XCorr | DeltaCn |
| --- | --- | --- | --- | --- |
| R.EIVYPFQGDSTVTK.S | 2 | 1584.751 | 3.162 | 0.309 |

+1 charge

```
		      B   	       Y
  1	 E 	   130.05	        -  	 14
  2	 I 	   243.13	  1454.75	 13
  3	 V 	   342.20	  1341.67	 12
  4	 Y 	   505.27	  1242.60	 11
  5	 P 	   602.32	  1079.54	 10
  6	 F 	   749.39	   982.48	  9
  7	 Q 	   877.45	   835.42	  8
  8	 G 	   934.47	   707.36	  7
  9	 D 	  1049.49	   650.34	  6
 10	 S 	  1136.53	   535.31	  5
 11	 T 	  1237.57	   448.28	  4
 12	 V 	  1336.64	   347.23	  3
 13	 T 	  1437.69	   248.16	  2
 14	 K 	        -  	   147.11	  1  _
+2 charge
		      B   	       Y
  1	 E 	    65.53	        -  	 14
  2	 I 	   122.07	   727.88	 13
  3	 V 	   171.60	   671.34	 12
  4	 Y 	   253.14	   621.80	 11
  5	 P 	   301.66	   540.27	 10
  6	 F 	   375.20	   491.75	  9
  7	 Q 	   439.23	   418.21	  8
  8	 G 	   467.74	   354.18	  7
  9	 D 	   525.25	   325.67	  6
 10	 S 	   568.77	   268.16	  5
 11	 T 	   619.29	   224.64	  4
 12	 V 	   668.82	   174.12	  3
 13	 T 	   719.35	   124.58	  2
 14	 K 	        -  	    74.06	  1  _
```

**37. IPI00651768 Isoform 1 of Hepatitis A virus cellular receptor 2**

| Sequence | Charge | M+H+ | XCorr | DeltaCn |
| --- | --- | --- | --- | --- |
| K.GACPVFECGNVVLR.T | 2 | 1578.782 | 4.108 | 0.286 |

+1 charge

```
		      B   	       Y
  1	 G 	    58.03	        -  	 14
  2	 A 	   129.07	  1521.00	 13
  3	 C 	   289.23	  1449.97	 12
  4	 P 	   386.28	  1289.80	 11
  5	 V 	   485.35	  1192.75	 10
  6	 F 	   632.42	  1093.68	  9
  7	 E 	   761.46	   946.61	  8
  8	 C 	   921.63	   817.57	  7
  9	 G 	   978.65	   657.40	  6
 10	 N 	  1092.69	   600.38	  5
 11	 V 	  1191.76	   486.34	  4
 12	 V 	  1290.83	   387.27	  3
 13	 L 	  1403.91	   288.20	  2
 14	 R 	        -  	   175.12	  1  _
+2 charge
		      B   	       Y
  1	 G 	    29.52	        -  	 14
  2	 A 	    65.04	   761.01	 13
  3	 C 	   145.12	   725.49	 12
  4	 P 	   193.65	   645.40	 11
  5	 V 	   243.18	   596.88	 10
  6	 F 	   316.71	   547.34	  9
  7	 E 	   381.24	   473.81	  8
  8	 C 	   461.32	   409.29	  7
  9	 G 	   489.83	   329.21	  6
 10	 N 	   546.85	   300.70	  5
 11	 V 	   596.38	   243.67	  4
 12	 V 	   645.92	   194.14	  3
 13	 L 	   702.46	   144.61	  2
 14	 R 	        -  	    88.06	  1  _
```

**38. IPI00387113 Ig kappa chain V-III region B6**

| Sequence | Charge | M+H+ | XCorr | DeltaCn |
| --- | --- | --- | --- | --- |
| - .ZIVLTZSPGTLSLSPGZR.A | 2 | 1884.616 | 3.939 | 0.601 |

+1 charge

```
		      B   	       Y
  1	 Z 	   129.56	        -  	 18
  2	 I 	   242.64	  1754.96	 17
  3	 V 	   341.71	  1641.88	 16
  4	 L 	   454.79	  1542.81	 15
  5	 T 	   555.84	  1429.73	 14
  6	 Z 	   684.39	  1328.68	 13
  7	 S 	   771.42	  1200.13	 12
  8	 P 	   868.48	  1113.10	 11
  9	 G 	   925.50	  1016.05	 10
 10	 T 	  1026.55	   959.02	  9
 11	 L 	  1139.63	   857.98	  8
 12	 S 	  1226.66	   744.89	  7
 13	 L 	  1339.75	   657.86	  6
 14	 S 	  1426.78	   544.78	  5
 15	 P 	  1523.83	   457.74	  4
 16	 G 	  1580.85	   360.69	  3
 17	 Z 	  1709.40	   303.67	  2
 18	 R 	        -  	   175.12	  1  _
+2 charge
		      B   	       Y
  1	 Z 	    65.28	        -  	 18
  2	 I 	   121.82	   877.99	 17
  3	 V 	   171.36	   821.44	 16
  4	 L 	   227.90	   771.91	 15
  5	 T 	   278.42	   715.37	 14
  6	 Z 	   342.70	   664.84	 13
  7	 S 	   386.22	   600.57	 12
  8	 P 	   434.74	   557.05	 11
  9	 G 	   463.25	   508.53	 10
 10	 T 	   513.78	   480.02	  9
 11	 L 	   570.32	   429.49	  8
 12	 S 	   613.83	   372.95	  7
 13	 L 	   670.38	   329.43	  6
 14	 S 	   713.89	   272.89	  5
 15	 P 	   762.42	   229.38	  4
 16	 G 	   790.93	   180.85	  3
 17	 Z 	   855.21	   152.34	  2
 18	 R 	        -  	    88.06	  1  _
```

**39. IPI00013682 Isoform 3 of Ecto-ADP-ribosyltransferase 3**

| Sequence | Charge | M+H+ | XCorr | DeltaCn |
| --- | --- | --- | --- | --- |
| K.IPEPFPLPEDK.S | 2 | 1282.466 | 3.425 | 0.384 |

+1 charge

```
		      B   	       Y
  1	 I 	   114.09	        -  	 11
  2	 P 	   211.14	  1168.59	 10
  3	 E 	   340.19	  1071.54	  9
  4	 P 	   437.24	   942.49	  8
  5	 F 	   584.31	   845.44	  7
  6	 P 	   681.36	   698.37	  6
  7	 L 	   794.44	   601.32	  5
  8	 P 	   891.50	   488.24	  4
  9	 E 	  1020.54	   391.18	  3
 10	 D 	  1135.57	   262.14	  2
 11	 K 	        -  	   147.11	  1  _
+2 charge
		      B   	       Y
  1	 I 	    57.55	        -  	 11
  2	 P 	   106.08	   584.80	 10
  3	 E 	   170.60	   536.27	  9
  4	 P 	   219.12	   471.75	  8
  5	 F 	   292.66	   423.22	  7
  6	 P 	   341.18	   349.69	  6
  7	 L 	   397.73	   301.16	  5
  8	 P 	   446.25	   244.62	  4
  9	 E 	   510.77	   196.09	  3
 10	 D 	   568.29	   131.57	  2
 11	 K 	        -  	    74.06	  1  _
```

**40. IPI00879999 ATP-dependent RNA helicase DDX54**

| Sequence | Charge | M+H+ | XCorr | DeltaCn |
| --- | --- | --- | --- | --- |
| R.DQEFYIPYRPKDFDSER.G | 2 | 2206.355 | 4.037 | 0.272 |

+1 charge

```
		      B   	       Y
  1	 D 	   116.03	        -  	 17
  2	 Q 	   244.09	  2090.00	 16
  3	 E 	   373.14	  1961.94	 15
  4	 F 	   520.20	  1832.90	 14
  5	 Y 	   683.27	  1685.83	 13
  6	 I 	   796.35	  1522.76	 12
  7	 P 	   893.40	  1409.68	 11
  8	 Y 	  1056.47	  1312.63	 10
  9	 R 	  1212.57	  1149.56	  9
 10	 P 	  1309.62	   993.46	  8
 11	 K 	  1437.72	   896.41	  7
 12	 D 	  1552.74	   768.32	  6
 13	 F 	  1699.81	   653.29	  5
 14	 D 	  1814.84	   506.22	  4
 15	 S 	  1901.87	   391.19	  3
 16	 E 	  2030.91	   304.16	  2
 17	 R 	        -  	   175.12	  1  _
+2 charge
		      B   	       Y
  1	 D 	    58.52	        -  	 17
  2	 Q 	   122.55	  1045.50	 16
  3	 E 	   187.07	   981.47	 15
  4	 F 	   260.61	   916.95	 14
  5	 Y 	   342.14	   843.42	 13
  6	 I 	   398.68	   761.89	 12
  7	 P 	   447.21	   705.34	 11
  8	 Y 	   528.74	   656.82	 10
  9	 R 	   606.79	   575.29	  9
 10	 P 	   655.31	   497.24	  8
 11	 K 	   719.36	   448.71	  7
 12	 D 	   776.88	   384.66	  6
 13	 F 	   850.41	   327.15	  5
 14	 D 	   907.92	   253.61	  4
 15	 S 	   951.44	   196.10	  3
 16	 E 	  1015.96	   152.58	  2
 17	 R 	        -  	    88.06	  1  _
```

**41. IPI00006030 Dynein intermediate chain 1, axonemal**

| Sequence | Charge | M+H+ | XCorr | DeltaCn |
| --- | --- | --- | --- | --- |
| R.DLFAVGYGSYDFMK.Q | 2 | 1613.815 | 3.315 | 0.269 |

+1 charge

```
		      B   	       Y
  1	 D 	   116.03	        -  	 14
  2	 L 	   229.12	  1497.71	 13
  3	 F 	   376.19	  1384.62	 12
  4	 A 	   447.22	  1237.56	 11
  5	 V 	   546.29	  1166.52	 10
  6	 G 	   603.31	  1067.45	  9
  7	 Y 	   766.38	  1010.43	  8
  8	 G 	   823.40	   847.37	  7
  9	 S 	   910.43	   790.34	  6
 10	 Y 	  1073.49	   703.31	  5
 11	 D 	  1188.52	   540.25	  4
 12	 F 	  1335.59	   425.22	  3
 13	 M 	  1466.63	   278.15	  2
 14	 K 	        -  	   147.11	  1  _
+2 charge
		      B   	       Y
  1	 D 	    58.52	        -  	 14
  2	 L 	   115.06	   749.36	 13
  3	 F 	   188.60	   692.82	 12
  4	 A 	   224.12	   619.28	 11
  5	 V 	   273.65	   583.76	 10
  6	 G 	   302.16	   534.23	  9
  7	 Y 	   383.69	   505.72	  8
  8	 G 	   412.20	   424.19	  7
  9	 S 	   455.72	   395.68	  6
 10	 Y 	   537.25	   352.16	  5
 11	 D 	   594.76	   270.63	  4
 12	 F 	   668.30	   213.11	  3
 13	 M 	   733.82	   139.58	  2
 14	 K 	        -  	    74.06	  1  _
```

**42. IPI00009477 Intercellular adhesion molecule 2**

| Sequence | Charge | M+H+ | XCorr | DeltaCn |
| --- | --- | --- | --- | --- |
| R.QVILTLQPTLVAVGK.S | 2 | 1580.937 | 3.16 | 0.395 |

+1 charge

```
		      B   	       Y
  1	 Q 	   129.07	        -  	 15
  2	 V 	   228.13	  1451.92	 14
  3	 I 	   341.22	  1352.85	 13
  4	 L 	   454.30	  1239.77	 12
  5	 T 	   555.35	  1126.68	 11
  6	 L 	   668.43	  1025.64	 10
  7	 Q 	   796.49	   912.55	  9
  8	 P 	   893.55	   784.49	  8
  9	 T 	   994.59	   687.44	  7
 10	 L 	  1107.68	   586.39	  6
 11	 V 	  1206.75	   473.31	  5
 12	 A 	  1277.78	   374.24	  4
 13	 V 	  1376.85	   303.20	  3
 14	 G 	  1433.87	   204.13	  2
 15	 K 	        -  	   147.11	  1  _
+2 charge
		      B   	       Y
  1	 Q 	    65.04	        -  	 15
  2	 V 	   114.57	   726.46	 14
  3	 I 	   171.11	   676.93	 13
  4	 L 	   227.65	   620.39	 12
  5	 T 	   278.18	   563.85	 11
  6	 L 	   334.72	   513.32	 10
  7	 Q 	   398.75	   456.78	  9
  8	 P 	   447.28	   392.75	  8
  9	 T 	   497.80	   344.22	  7
 10	 L 	   554.34	   293.70	  6
 11	 V 	   603.88	   237.16	  5
 12	 A 	   639.40	   187.62	  4
 13	 V 	   688.93	   152.10	  3
 14	 G 	   717.44	   102.57	  2
 15	 K 	        -  	    74.06	  1  _
```

**43. IPI00297263 Isoform 1 of Protein HEG homolog 1**

| Sequence | Charge | M+H+ | XCorr | DeltaCn |
| --- | --- | --- | --- | --- |
| R.ALSLAPLAGAGLELQLER.R | 2 | 1823.127 | 5.285 | 0.636 |

+1 charge

```
		      B   	       Y
  1	 A 	    72.04	        -  	 18
  2	 L 	   185.13	  1751.01	 17
  3	 S 	   272.16	  1637.92	 16
  4	 L 	   385.24	  1550.89	 15
  5	 A 	   456.28	  1437.81	 14
  6	 P 	   553.33	  1366.77	 13
  7	 L 	   666.42	  1269.72	 12
  8	 A 	   737.46	  1156.63	 11
  9	 G 	   794.48	  1085.59	 10
 10	 A 	   865.51	  1028.57	  9
 11	 G 	   922.54	   957.54	  8
 12	 L 	  1035.62	   900.51	  7
 13	 E 	  1164.66	   787.43	  6
 14	 L 	  1277.75	   658.39	  5
 15	 Q 	  1405.80	   545.30	  4
 16	 L 	  1518.89	   417.25	  3
 17	 E 	  1647.93	   304.16	  2
 18	 R 	        -  	   175.12	  1  _
+2 charge
		      B   	       Y
  1	 A 	    36.53	        -  	 18
  2	 L 	    93.07	   876.01	 17
  3	 S 	   136.58	   819.46	 16
  4	 L 	   193.13	   775.95	 15
  5	 A 	   228.64	   719.41	 14
  6	 P 	   277.17	   683.89	 13
  7	 L 	   333.71	   635.36	 12
  8	 A 	   369.23	   578.82	 11
  9	 G 	   397.74	   543.30	 10
 10	 A 	   433.26	   514.79	  9
 11	 G 	   461.77	   479.27	  8
 12	 L 	   518.31	   450.76	  7
 13	 E 	   582.83	   394.22	  6
 14	 L 	   639.38	   329.70	  5
 15	 Q 	   703.41	   273.16	  4
 16	 L 	   759.95	   209.13	  3
 17	 E 	   824.47	   152.58	  2
 18	 R 	        -  	    88.06	  1  _
```

**44. IPI00940960 Epididymal secretory protein E1**

| Sequence | Charge | M+H+ | XCorr | DeltaCn |
| --- | --- | --- | --- | --- |
| K.AVVHGILMGVPVPFPIPEPDGCK.S | 3 | 2430.887 | 3.898 | 0.503 |

+1 charge

```
		      B   	       Y
  1	 A 	    72.04	        -  	 23
  2	 V 	   171.11	  2358.37	 22
  3	 V 	   270.18	  2259.30	 21
  4	 H 	   407.24	  2160.23	 20
  5	 G 	   464.26	  2023.17	 19
  6	 I 	   577.35	  1966.15	 18
  7	 L 	   690.43	  1853.07	 17
  8	 M 	   821.47	  1739.98	 16
  9	 G 	   878.49	  1608.94	 15
 10	 V 	   977.56	  1551.92	 14
 11	 P 	  1074.61	  1452.85	 13
 12	 V 	  1173.68	  1355.80	 12
 13	 P 	  1270.73	  1256.73	 11
 14	 F 	  1417.80	  1159.68	 10
 15	 P 	  1514.86	  1012.61	  9
 16	 I 	  1627.94	   915.56	  8
 17	 P 	  1724.99	   802.47	  7
 18	 E 	  1854.03	   705.42	  6
 19	 P 	  1951.09	   576.38	  5
 20	 D 	  2066.11	   479.33	  4
 21	 G 	  2123.14	   364.30	  3
 22	 C 	  2283.30	   307.28	  2
 23	 K 	        -  	   147.11	  1  _


+2 charge
		      B   	       Y
  1	 A 	    36.53	        -  	 23
  2	 V 	    86.06	  1179.69	 22
  3	 V 	   135.59	  1130.15	 21
  4	 H 	   204.12	  1080.62	 20
  5	 G 	   232.63	  1012.09	 19
  6	 I 	   289.18	   983.58	 18
  7	 L 	   345.72	   927.04	 17
  8	 M 	   411.24	   870.50	 16
  9	 G 	   439.75	   804.98	 15
 10	 V 	   489.28	   776.46	 14
 11	 P 	   537.81	   726.93	 13
 12	 V 	   587.34	   678.40	 12
 13	 P 	   635.87	   628.87	 11
 14	 F 	   709.40	   580.34	 10
 15	 P 	   757.93	   506.81	  9
 16	 I 	   814.47	   458.28	  8
 17	 P 	   863.00	   401.74	  7
 18	 E 	   927.52	   353.21	  6
 19	 P 	   976.05	   288.69	  5
 20	 D 	  1033.56	   240.17	  4
 21	 G 	  1062.07	   182.65	  3
 22	 C 	  1142.15	   154.14	  2
 23	 K 	        -  	    74.06	  1  _
+3 charge
		      B   	       Y
  1	 A 	    24.69	        -  	 23
  2	 V 	    57.71	   786.79	 22
  3	 V 	    90.73	   753.77	 21
  4	 H 	   136.42	   720.75	 20
  5	 G 	   155.43	   675.06	 19
  6	 I 	   193.12	   656.06	 18
  7	 L 	   230.81	   618.36	 17
  8	 M 	   274.49	   580.67	 16
  9	 G 	   293.50	   536.99	 15
 10	 V 	   326.52	   517.98	 14
 11	 P 	   358.88	   484.96	 13
 12	 V 	   391.90	   452.61	 12
 13	 P 	   424.25	   419.58	 11
 14	 F 	   473.27	   387.23	 10
 15	 P 	   505.62	   338.21	  9
 16	 I 	   543.32	   305.86	  8
 17	 P 	   575.67	   268.16	  7
 18	 E 	   618.68	   235.81	  6
 19	 P 	   651.03	   192.80	  5
 20	 D 	   689.38	   160.45	  4
 21	 G 	   708.38	   122.10	  3
 22	 C 	   761.77	   103.10	  2
 23	 K 	        -  	    49.71	  1  _
```

**45. IPI00027769 Neutrophil elastase**

| Sequence | Charge | M+H+ | XCorr | DeltaCn |
| --- | --- | --- | --- | --- |
| R.QAGVCFGDSGSPLVCNGLIHGIASFVR.G | 3 | 2820.14 | 4.26 | 0.44 |

+1 charge

```
		      B   	       Y
  1	 Q 	   129.07	        -  	 27
  2	 A 	   200.10	  2690.59	 26
  3	 G 	   257.12	  2619.55	 25
  4	 V 	   356.19	  2562.53	 24
  5	 C 	   516.36	  2463.46	 23
  6	 F 	   663.43	  2303.29	 22
  7	 G 	   720.45	  2156.23	 21
  8	 D 	   835.47	  2099.20	 20
  9	 S 	   922.51	  1984.18	 19
 10	 G 	   979.53	  1897.15	 18
 11	 S 	  1066.56	  1840.12	 17
 12	 P 	  1163.61	  1753.09	 16
 13	 L 	  1276.70	  1656.04	 15
 14	 V 	  1375.77	  1542.96	 14
 15	 C 	  1535.93	  1443.89	 13
 16	 N 	  1649.97	  1283.72	 12
 17	 G 	  1707.00	  1169.68	 11
 18	 L 	  1820.08	  1112.66	 10
 19	 I 	  1933.16	   999.57	  9
 20	 H 	  2070.22	   886.49	  8
 21	 G 	  2127.24	   749.43	  7
 22	 I 	  2240.33	   692.41	  6
 23	 A 	  2311.36	   579.32	  5
 24	 S 	  2398.40	   508.29	  4
 25	 F 	  2545.47	   421.26	  3
 26	 V 	  2644.53	   274.19	  2
 27	 R 	        -  	   175.12	  1  _


+2 charge
		      B   	       Y
  1	 Q 	    65.04	        -  	 27
  2	 A 	   100.56	  1345.80	 26
  3	 G 	   129.07	  1310.28	 25
  4	 V 	   178.60	  1281.77	 24
  5	 C 	   258.68	  1232.23	 23
  6	 F 	   332.22	  1152.15	 22
  7	 G 	   360.73	  1078.62	 21
  8	 D 	   418.24	  1050.11	 20
  9	 S 	   461.76	   992.59	 19
 10	 G 	   490.27	   949.08	 18
 11	 S 	   533.78	   920.57	 17
 12	 P 	   582.31	   877.05	 16
 13	 L 	   638.85	   828.52	 15
 14	 V 	   688.39	   771.98	 14
 15	 C 	   768.47	   722.45	 13
 16	 N 	   825.49	   642.36	 12
 17	 G 	   854.00	   585.34	 11
 18	 L 	   910.54	   556.83	 10
 19	 I 	   967.09	   500.29	  9
 20	 H 	  1035.61	   443.75	  8
 21	 G 	  1064.13	   375.22	  7
 22	 I 	  1120.67	   346.71	  6
 23	 A 	  1156.19	   290.17	  5
 24	 S 	  1199.70	   254.65	  4
 25	 F 	  1273.24	   211.13	  3
 26	 V 	  1322.77	   137.60	  2
 27	 R 	        -  	    88.06	  1  _


+3 charge
		      B   	       Y
  1	 Q 	    43.69	        -  	 27
  2	 A 	    67.37	   897.53	 26
  3	 G 	    86.38	   873.85	 25
  4	 V 	   119.40	   854.85	 24
  5	 C 	   172.79	   821.82	 23
  6	 F 	   221.81	   768.44	 22
  7	 G 	   240.82	   719.41	 21
  8	 D 	   279.16	   700.41	 20
  9	 S 	   308.17	   662.06	 19
 10	 G 	   327.18	   633.05	 18
 11	 S 	   356.19	   614.05	 17
 12	 P 	   388.54	   585.04	 16
 13	 L 	   426.24	   552.68	 15
 14	 V 	   459.26	   514.99	 14
 15	 C 	   512.65	   481.97	 13
 16	 N 	   550.66	   428.58	 12
 17	 G 	   569.67	   390.56	 11
 18	 L 	   607.36	   371.56	 10
 19	 I 	   645.06	   333.86	  9
 20	 H 	   690.75	   296.17	  8
 21	 G 	   709.75	   250.48	  7
 22	 I 	   747.45	   231.47	  6
 23	 A 	   771.13	   193.78	  5
 24	 S 	   800.14	   170.10	  4
 25	 F 	   849.16	   141.09	  3
 26	 V 	   882.18	    92.07	  2
 27	 R 	        -  	    59.04	  1  _
```

**46. IPI00914890 Isoform 1 of Microtubule-associated serine/threonine-protein kinase 4**

| Sequence | Charge | M+H+ | XCorr | DeltaCn |
| --- | --- | --- | --- | --- |
| R.DCLDKSHQGLITSRYFLELQHK.L | 3 | 2690.001 | 3.541 | 0.205 |

+1 charge

```
		      B   	       Y
  1	 D 	   116.03	        -  	 22
  2	 C 	   276.20	  2573.46	 21
  3	 L 	   389.28	  2413.30	 20
  4	 D 	   504.31	  2300.21	 19
  5	 K 	   632.41	  2185.19	 18
  6	 S 	   719.44	  2057.09	 17
  7	 H 	   856.50	  1970.06	 16
  8	 Q 	   984.55	  1833.00	 15
  9	 G 	  1041.58	  1704.94	 14
 10	 L 	  1154.66	  1647.92	 13
 11	 I 	  1267.74	  1534.84	 12
 12	 T 	  1368.79	  1421.75	 11
 13	 S 	  1455.82	  1320.71	 10
 14	 R 	  1611.93	  1233.67	  9
 15	 Y 	  1774.99	  1077.57	  8
 16	 F 	  1922.06	   914.51	  7
 17	 L 	  2035.14	   767.44	  6
 18	 E 	  2164.18	   654.36	  5
 19	 L 	  2277.27	   525.31	  4
 20	 Q 	  2405.33	   412.23	  3
 21	 H 	  2542.39	   284.17	  2
 22	 K 	        -  	   147.11	  1  _


+2 charge
		      B   	       Y
  1	 D 	    58.52	        -  	 22
  2	 C 	   138.60	  1287.24	 21
  3	 L 	   195.15	  1207.15	 20
  4	 D 	   252.66	  1150.61	 19
  5	 K 	   316.71	  1093.10	 18
  6	 S 	   360.22	  1029.05	 17
  7	 H 	   428.75	   985.53	 16
  8	 Q 	   492.78	   917.00	 15
  9	 G 	   521.29	   852.98	 14
 10	 L 	   577.83	   824.46	 13
 11	 I 	   634.38	   767.92	 12
 12	 T 	   684.90	   711.38	 11
 13	 S 	   728.42	   660.86	 10
 14	 R 	   806.47	   617.34	  9
 15	 Y 	   888.00	   539.29	  8
 16	 F 	   961.53	   457.76	  7
 17	 L 	  1018.07	   384.22	  6
 18	 E 	  1082.60	   327.68	  5
 19	 L 	  1139.14	   263.16	  4
 20	 Q 	  1203.17	   206.62	  3
 21	 H 	  1271.70	   142.59	  2
 22	 K 	        -  	    74.06	  1  _
+3 charge
		      B   	       Y
  1	 D 	    39.35	        -  	 22
  2	 C 	    92.74	   858.49	 21
  3	 L 	   130.43	   805.10	 20
  4	 D 	   168.78	   767.41	 19
  5	 K 	   211.47	   729.07	 18
  6	 S 	   240.48	   686.37	 17
  7	 H 	   286.17	   657.36	 16
  8	 Q 	   328.86	   611.67	 15
  9	 G 	   347.86	   568.99	 14
 10	 L 	   385.56	   549.98	 13
 11	 I 	   423.25	   512.28	 12
 12	 T 	   456.94	   474.59	 11
 13	 S 	   485.95	   440.91	 10
 14	 R 	   537.98	   411.90	  9
 15	 Y 	   592.33	   359.86	  8
 16	 F 	   641.36	   305.51	  7
 17	 L 	   679.05	   256.49	  6
 18	 E 	   722.07	   218.79	  5
 19	 L 	   759.76	   175.78	  4
 20	 Q 	   802.45	   138.08	  3
 21	 H 	   848.13	    95.40	  2
 22	 K 	        -  	    49.71	  1  _
```

**47. IPI00013890 Isoform 1 of 14-3-3 protein sigma**

| Sequence | Charge | M+H+ | XCorr | DeltaCn |
| --- | --- | --- | --- | --- |
| R.EKVETELQGVCDTVLGLLDSHLIK.E | 3 | 2698.056 | 4.23 | 0.354 |

+1 charge

```
		      B   	       Y
  1	 E 	   130.05	        -  	 24
  2	 K 	   258.14	  2567.51	 23
  3	 V 	   357.21	  2439.41	 22
  4	 E 	   486.26	  2340.35	 21
  5	 T 	   587.30	  2211.30	 20
  6	 E 	   716.35	  2110.26	 19
  7	 L 	   829.43	  1981.21	 18
  8	 Q 	   957.49	  1868.13	 17
  9	 G 	  1014.51	  1740.07	 16
 10	 V 	  1113.58	  1683.05	 15
 11	 C 	  1273.74	  1583.98	 14
 12	 D 	  1388.77	  1423.82	 13
 13	 T 	  1489.82	  1308.79	 12
 14	 V 	  1588.89	  1207.74	 11
 15	 L 	  1701.97	  1108.67	 10
 16	 G 	  1758.99	   995.59	  9
 17	 L 	  1872.08	   938.57	  8
 18	 L 	  1985.16	   825.48	  7
 19	 D 	  2100.19	   712.40	  6
 20	 S 	  2187.22	   597.37	  5
 21	 H 	  2324.28	   510.34	  4
 22	 L 	  2437.36	   373.28	  3
 23	 I 	  2550.45	   260.20	  2
 24	 K 	        -  	   147.11	  1  _


+2 charge
		      B   	       Y
  1	 E 	    65.53	        -  	 24
  2	 K 	   129.58	  1284.26	 23
  3	 V 	   179.11	  1220.21	 22
  4	 E 	   243.63	  1170.68	 21
  5	 T 	   294.16	  1106.16	 20
  6	 E 	   358.68	  1055.63	 19
  7	 L 	   415.22	   991.11	 18
  8	 Q 	   479.25	   934.57	 17
  9	 G 	   507.76	   870.54	 16
 10	 V 	   557.29	   842.03	 15
 11	 C 	   637.38	   792.49	 14
 12	 D 	   694.89	   712.41	 13
 13	 T 	   745.41	   654.90	 12
 14	 V 	   794.95	   604.37	 11
 15	 L 	   851.49	   554.84	 10
 16	 G 	   880.00	   498.30	  9
 17	 L 	   936.54	   469.79	  8
 18	 L 	   993.08	   413.25	  7
 19	 D 	  1050.60	   356.70	  6
 20	 S 	  1094.11	   299.19	  5
 21	 H 	  1162.64	   255.67	  4
 22	 L 	  1219.18	   187.14	  3
 23	 I 	  1275.73	   130.60	  2
 24	 K 	        -  	    74.06	  1  _


+3 charge
		      B   	       Y
  1	 E 	    44.02	        -  	 24
  2	 K 	    86.72	   856.51	 23
  3	 V 	   119.74	   813.81	 22
  4	 E 	   162.76	   780.79	 21
  5	 T 	   196.44	   737.77	 20
  6	 E 	   239.45	   704.09	 19
  7	 L 	   277.15	   661.08	 18
  8	 Q 	   319.83	   623.38	 17
  9	 G 	   338.84	   580.70	 16
 10	 V 	   371.86	   561.69	 15
 11	 C 	   425.25	   528.67	 14
 12	 D 	   463.60	   475.28	 13
 13	 T 	   497.28	   436.93	 12
 14	 V 	   530.30	   403.25	 11
 15	 L 	   568.00	   370.23	 10
 16	 G 	   587.00	   332.53	  9
 17	 L 	   624.70	   313.53	  8
 18	 L 	   662.39	   275.83	  7
 19	 D 	   700.73	   238.14	  6
 20	 S 	   729.74	   199.80	  5
 21	 H 	   775.43	   170.78	  4
 22	 L 	   813.13	   125.10	  3
 23	 I 	   850.82	    87.40	  2
 24	 K 	        -  	    49.71	  1  _
```

**48. IPI00010675 Trefoil factor 2**

| Sequence | Charge | M+H+ | XCorr | DeltaCn |
| --- | --- | --- | --- | --- |
| R.NCGYPGISPEECASR.K | 2 | 1697.772 | 3.701 | 0.486 |

+1 charge

```
		      B   	       Y
  1	 N 	   115.05	        -  	 15
  2	 C 	   275.22	  1582.93	 14
  3	 G 	   332.24	  1422.77	 13
  4	 Y 	   495.30	  1365.74	 12
  5	 P 	   592.35	  1202.68	 11
  6	 G 	   649.37	  1105.63	 10
  7	 I 	   762.46	  1048.61	  9
  8	 S 	   849.49	   935.52	  8
  9	 P 	   946.54	   848.49	  7
 10	 E 	  1075.59	   751.44	  6
 11	 E 	  1204.63	   622.40	  5
 12	 C 	  1364.79	   493.35	  4
 13	 A 	  1435.83	   333.19	  3
 14	 S 	  1522.86	   262.15	  2
 15	 R 	        -  	   175.12	  1  _
+2 charge
		      B   	       Y
  1	 N 	    58.03	        -  	 15
  2	 C 	   138.11	   791.97	 14
  3	 G 	   166.62	   711.89	 13
  4	 Y 	   248.15	   683.38	 12
  5	 P 	   296.68	   601.84	 11
  6	 G 	   325.19	   553.32	 10
  7	 I 	   381.73	   524.81	  9
  8	 S 	   425.25	   468.27	  8
  9	 P 	   473.78	   424.75	  7
 10	 E 	   538.30	   376.22	  6
 11	 E 	   602.82	   311.70	  5
 12	 C 	   682.90	   247.18	  4
 13	 A 	   718.42	   167.10	  3
 14	 S 	   761.94	   131.58	  2
 15	 R 	        -  	    88.06	  1  _
```

**49. IPI00301143 Isoform 1 of Peptidase inhibitor 16**

| Sequence | Charge | M+H+ | XCorr | DeltaCn |
| --- | --- | --- | --- | --- |
| R.LMVELHNLYR.A | 2 | 1288.544 | 3.383 | 0.335 |

+1 charge

```
		      B   	       Y
  1	 L 	   114.09	        -  	 10
  2	 M 	   245.13	  1174.60	  9
  3	 V 	   344.20	  1043.56	  8
  4	 E 	   473.24	   944.49	  7
  5	 L 	   586.33	   815.45	  6
  6	 H 	   723.39	   702.37	  5
  7	 N 	   837.43	   565.31	  4
  8	 L 	   950.51	   451.27	  3
  9	 Y 	  1113.58	   338.18	  2
 10	 R 	        -  	   175.12	  1  _
+2 charge
		      B   	       Y
  1	 L 	    57.55	        -  	 10
  2	 M 	   123.07	   587.81	  9
  3	 V 	   172.60	   522.29	  8
  4	 E 	   237.13	   472.75	  7
  5	 L 	   293.67	   408.23	  6
  6	 H 	   362.20	   351.69	  5
  7	 N 	   419.22	   283.16	  4
  8	 L 	   475.76	   226.14	  3
  9	 Y 	   557.29	   169.59	  2
 10	 R 	        -  	    88.06	  1  _
```

+

**50. IPI00006988 Resistin**

| Sequence | Charge | M+H+ | XCorr | DeltaCn |
| --- | --- | --- | --- | --- |
| R.IQEVAGSLIFR.A | 2 | 1233.442 | 4.169 | 0.492 |

+1 charge

```
		      B   	       Y
  1	 I 	   114.09	        -  	 11
  2	 Q 	   242.15	  1119.62	 10
  3	 E 	   371.19	   991.56	  9
  4	 V 	   470.26	   862.51	  8
  5	 A 	   541.30	   763.45	  7
  6	 G 	   598.32	   692.41	  6
  7	 S 	   685.35	   635.39	  5
  8	 L 	   798.44	   548.36	  4
  9	 I 	   911.52	   435.27	  3
 10	 F 	  1058.59	   322.19	  2
 11	 R 	        -  	   175.12	  1  _
+2 charge
		      B   	       Y
  1	 I 	    57.55	        -  	 11
  2	 Q 	   121.58	   560.31	 10
  3	 E 	   186.10	   496.28	  9
  4	 V 	   235.63	   431.76	  8
  5	 A 	   271.15	   382.23	  7
  6	 G 	   299.66	   346.71	  6
  7	 S 	   343.18	   318.20	  5
  8	 L 	   399.72	   274.68	  4
  9	 I 	   456.26	   218.14	  3
 10	 F 	   529.80	   161.60	  2
 11	 R 	        -  	    88.06	  1  _
```

**51. IPI00022892 Thy-1 membrane glycoprotein**

| Sequence | Charge | M+H+ | XCorr | DeltaCn |
| --- | --- | --- | --- | --- |
| K.VTSLTACLVDQSLR.L | 2 | 1563.772 | 4.179 | 0.412 |

+1 charge

```
		      B   	       Y
  1	 V 	   100.08	        -  	 14
  2	 T 	   201.12	  1463.89	 13
  3	 S 	   288.16	  1362.84	 12
  4	 L 	   401.24	  1275.81	 11
  5	 T 	   502.29	  1162.72	 10
  6	 A 	   573.32	  1061.68	  9
  7	 C 	   733.49	   990.64	  8
  8	 L 	   846.57	   830.47	  7
  9	 V 	   945.64	   717.39	  6
 10	 D 	  1060.67	   618.32	  5
 11	 Q 	  1188.73	   503.29	  4
 12	 S 	  1275.76	   375.24	  3
 13	 L 	  1388.84	   288.20	  2
 14	 R 	        -  	   175.12	  1  _
+2 charge
		      B   	       Y
  1	 V 	    50.54	        -  	 14
  2	 T 	   101.07	   732.45	 13
  3	 S 	   144.58	   681.92	 12
  4	 L 	   201.12	   638.41	 11
  5	 T 	   251.65	   581.87	 10
  6	 A 	   287.17	   531.34	  9
  7	 C 	   367.25	   495.82	  8
  8	 L 	   423.79	   415.74	  7
  9	 V 	   473.32	   359.20	  6
 10	 D 	   530.84	   309.66	  5
 11	 Q 	   594.87	   252.15	  4
 12	 S 	   638.38	   188.12	  3
 13	 L 	   694.93	   144.61	  2
 14	 R 	        -  	    88.06	  1  _
```

**52. IPI00413865 Arylsulfatase J**

| Sequence | Charge | M+H+ | XCorr | DeltaCn |
| --- | --- | --- | --- | --- |
| R.SNPRLNGGVWGPWYKEETK.K | 2 | 2219.444 | 3.702 | 0.348 |

+1 charge

```
		      B   	       Y
  1	 S 	    88.04	        -  	 19
  2	 N 	   202.08	  2131.07	 18
  3	 P 	   299.13	  2017.03	 17
  4	 R 	   455.24	  1919.98	 16
  5	 L 	   568.32	  1763.88	 15
  6	 N 	   682.36	  1650.79	 14
  7	 G 	   739.38	  1536.75	 13
  8	 G 	   796.41	  1479.73	 12
  9	 V 	   895.47	  1422.71	 11
 10	 W 	  1081.55	  1323.64	 10
 11	 G 	  1138.58	  1137.56	  9
 12	 P 	  1235.63	  1080.54	  8
 13	 W 	  1421.71	   983.48	  7
 14	 Y 	  1584.77	   797.40	  6
 15	 K 	  1712.87	   634.34	  5
 16	 E 	  1841.91	   506.25	  4
 17	 E 	  1970.95	   377.20	  3
 18	 T 	  2072.00	   248.16	  2
 19	 K 	        -  	   147.11	  1  _
+2 charge
		      B   	       Y
  1	 S 	    44.52	        -  	 19
  2	 N 	   101.54	  1066.04	 18
  3	 P 	   150.07	  1009.02	 17
  4	 R 	   228.12	   960.49	 16
  5	 L 	   284.66	   882.44	 15
  6	 N 	   341.69	   825.90	 14
  7	 G 	   370.20	   768.88	 13
  8	 G 	   398.71	   740.37	 12
  9	 V 	   448.24	   711.86	 11
 10	 W 	   541.28	   662.32	 10
 11	 G 	   569.79	   569.28	  9
 12	 P 	   618.32	   540.77	  8
 13	 W 	   711.36	   492.25	  7
 14	 Y 	   792.89	   399.21	  6
 15	 K 	   856.94	   317.67	  5
 16	 E 	   921.46	   253.63	  4
 17	 E 	   985.98	   189.11	  3
 18	 T 	  1036.50	   124.58	  2
 19	 K 	        -  	    74.06	  1  _
```

**52. IPI00296992 Isoform Long of Tyrosine-protein kinase receptor UFO**

| Sequence | Charge | M+H+ | XCorr | DeltaCn |
| --- | --- | --- | --- | --- |
| R.LAYQGQDTPEVLMDIGLR.Q | 2 | 2020.296 | 4.84 | 0.543 |

+1 charge

```
		      B   	       Y
  1	 L 	   114.09	        -  	 18
  2	 A 	   185.13	  1905.94	 17
  3	 Y 	   348.19	  1834.90	 16
  4	 Q 	   476.25	  1671.84	 15
  5	 G 	   533.27	  1543.78	 14
  6	 Q 	   661.33	  1486.76	 13
  7	 D 	   776.36	  1358.70	 12
  8	 T 	   877.41	  1243.67	 11
  9	 P 	   974.46	  1142.62	 10
 10	 E 	  1103.50	  1045.57	  9
 11	 V 	  1202.57	   916.53	  8
 12	 L 	  1315.65	   817.46	  7
 13	 M 	  1446.69	   704.38	  6
 14	 D 	  1561.72	   573.34	  5
 15	 I 	  1674.80	   458.31	  4
 16	 G 	  1731.83	   345.22	  3
 17	 L 	  1844.91	   288.20	  2
 18	 R 	        -  	   175.12	  1  _
+2 charge
		      B   	       Y
  1	 L 	    57.55	        -  	 18
  2	 A 	    93.07	   953.47	 17
  3	 Y 	   174.60	   917.95	 16
  4	 Q 	   238.63	   836.42	 15
  5	 G 	   267.14	   772.39	 14
  6	 Q 	   331.17	   743.88	 13
  7	 D 	   388.68	   679.85	 12
  8	 T 	   439.21	   622.34	 11
  9	 P 	   487.73	   571.82	 10
 10	 E 	   552.25	   523.29	  9
 11	 V 	   601.79	   458.77	  8
 12	 L 	   658.33	   409.23	  7
 13	 M 	   723.85	   352.69	  6
 14	 D 	   781.36	   287.17	  5
 15	 I 	   837.91	   229.66	  4
 16	 G 	   866.42	   173.12	  3
 17	 L 	   922.96	   144.61	  2
 18	 R 	        -  	    88.06	  1  _
```

**53. IPI00005707 C-type mannose receptor 2**

| Sequence | Charge | M+H+ | XCorr | DeltaCn |
| --- | --- | --- | --- | --- |
| R.TLGDQLSLLLGAR.T | 2 | 1357.581 | 3.11 | 0.287 |

+1 charge

```
		      B   	       Y
  1	 T 	   102.05	        -  	 13
  2	 L 	   215.14	  1255.74	 12
  3	 G 	   272.16	  1142.65	 11
  4	 D 	   387.19	  1085.63	 10
  5	 Q 	   515.25	   970.60	  9
  6	 L 	   628.33	   842.55	  8
  7	 S 	   715.36	   729.46	  7
  8	 L 	   828.45	   642.43	  6
  9	 L 	   941.53	   529.35	  5
 10	 L 	  1054.61	   416.26	  4
 11	 G 	  1111.64	   303.18	  3
 12	 A 	  1182.67	   246.16	  2
 13	 R 	        -  	   175.12	  1  _
+2 charge
		      B   	       Y
  1	 T 	    51.53	        -  	 13
  2	 L 	   108.07	   628.37	 12
  3	 G 	   136.58	   571.83	 11
  4	 D 	   194.10	   543.32	 10
  5	 Q 	   258.13	   485.81	  9
  6	 L 	   314.67	   421.78	  8
  7	 S 	   358.18	   365.23	  7
  8	 L 	   414.73	   321.72	  6
  9	 L 	   471.27	   265.18	  5
 10	 L 	   527.81	   208.63	  4
 11	 G 	   556.32	   152.09	  3
 12	 A 	   591.84	   123.58	  2
 13	 R 	        -  	    88.06	  1  _
```

**54．IPI00018236 Ganglioside GM2 activator**

| Sequence | Charge | M+H+ | XCorr | DeltaCn |
| --- | --- | --- | --- | --- |
| K.SEFVVPDLELPSWLTTGNYR.I | 2 | 2324.573 | 2.891 | 0.487 |

+1 charge

```
		      B   	       Y
  1	 S 	    88.04	        -  	 20
  2	 E 	   217.08	  2236.13	 19
  3	 F 	   364.15	  2107.09	 18
  4	 V 	   463.22	  1960.02	 17
  5	 V 	   562.29	  1860.95	 16
  6	 P 	   659.34	  1761.88	 15
  7	 D 	   774.37	  1664.83	 14
  8	 L 	   887.45	  1549.80	 13
  9	 E 	  1016.49	  1436.72	 12
 10	 L 	  1129.58	  1307.67	 11
 11	 P 	  1226.63	  1194.59	 10
 12	 S 	  1313.66	  1097.54	  9
 13	 W 	  1499.74	  1010.51	  8
 14	 L 	  1612.83	   824.43	  7
 15	 T 	  1713.87	   711.34	  6
 16	 T 	  1814.92	   610.29	  5
 17	 G 	  1871.94	   509.25	  4
 18	 N 	  1985.99	   452.23	  3
 19	 Y 	  2149.05	   338.18	  2
 20	 R 	        -  	   175.12	  1  _
+2 charge
		      B   	       Y
  1	 S 	    44.52	        -  	 20
  2	 E 	   109.04	  1118.57	 19
  3	 F 	   182.58	  1054.05	 18
  4	 V 	   232.11	   980.51	 17
  5	 V 	   281.65	   930.98	 16
  6	 P 	   330.17	   881.44	 15
  7	 D 	   387.69	   832.92	 14
  8	 L 	   444.23	   775.40	 13
  9	 E 	   508.75	   718.86	 12
 10	 L 	   565.29	   654.34	 11
 11	 P 	   613.82	   597.80	 10
 12	 S 	   657.33	   549.27	  9
 13	 W 	   750.37	   505.76	  8
 14	 L 	   806.92	   412.72	  7
 15	 T 	   857.44	   356.17	  6
 16	 T 	   907.96	   305.65	  5
 17	 G 	   936.47	   255.13	  4
 18	 N 	   993.50	   226.62	  3
 19	 Y 	  1075.03	   169.59	  2
 20	 R 	        -  	    88.06	  1  _
```

**55. IPI00001593 Lysosomal Pro-X carboxypeptidase**

| Sequence | Charge | M+H+ | XCorr | DeltaCn |
| --- | --- | --- | --- | --- |
| R.HLNFLTSEQALADFAELIK.H | 2 | 2161.443 | 4.495 | 0.578 |

+1 charge

```
		      B   	       Y
  1	 H 	   138.07	        -  	 19
  2	 L 	   251.15	  2023.07	 18
  3	 N 	   365.19	  1909.99	 17
  4	 F 	   512.26	  1795.95	 16
  5	 L 	   625.35	  1648.88	 15
  6	 T 	   726.39	  1535.80	 14
  7	 S 	   813.43	  1434.75	 13
  8	 E 	   942.47	  1347.72	 12
  9	 Q 	  1070.53	  1218.67	 11
 10	 A 	  1141.56	  1090.61	 10
 11	 L 	  1254.65	  1019.58	  9
 12	 A 	  1325.68	   906.49	  8
 13	 D 	  1440.71	   835.46	  7
 14	 F 	  1587.78	   720.43	  6
 15	 A 	  1658.82	   573.36	  5
 16	 E 	  1787.86	   502.32	  4
 17	 L 	  1900.94	   373.28	  3
 18	 I 	  2014.03	   260.20	  2
 19	 K 	        -  	   147.11	  1  _
+2 charge
		      B   	       Y
  1	 H 	    69.54	        -  	 19
  2	 L 	   126.08	  1012.04	 18
  3	 N 	   183.10	   955.50	 17
  4	 F 	   256.63	   898.48	 16
  5	 L 	   313.18	   824.94	 15
  6	 T 	   363.70	   768.40	 14
  7	 S 	   407.22	   717.88	 13
  8	 E 	   471.74	   674.36	 12
  9	 Q 	   535.77	   609.84	 11
 10	 A 	   571.29	   545.81	 10
 11	 L 	   627.83	   510.29	  9
 12	 A 	   663.35	   453.75	  8
 13	 D 	   720.86	   418.23	  7
 14	 F 	   794.39	   360.72	  6
 15	 A 	   829.91	   287.18	  5
 16	 E 	   894.43	   251.67	  4
 17	 L 	   950.98	   187.14	  3
 18	 I 	  1007.52	   130.60	  2
 19	 K 	        -  	    74.06	  1  _
```

**56. IPI00009027 Lithostathine-1-alpha**

| Sequence | Charge | M+H+ | XCorr | DeltaCn |
| --- | --- | --- | --- | --- |
| K.DVPCEDKFSFVCK.F | 2 | 1631.795 | 3.205 | 0.434 |

+1 charge

```
		      B   	       Y
  1	 D 	   116.03	        -  	 13
  2	 V 	   215.10	  1515.97	 12
  3	 P 	   312.16	  1416.90	 11
  4	 C 	   472.32	  1319.85	 10
  5	 E 	   601.36	  1159.68	  9
  6	 D 	   716.39	  1030.64	  8
  7	 K 	   844.49	   915.61	  7
  8	 F 	   991.55	   787.52	  6
  9	 S 	  1078.59	   640.45	  5
 10	 F 	  1225.65	   553.41	  4
 11	 V 	  1324.72	   406.35	  3
 12	 C 	  1484.89	   307.28	  2
 13	 K 	        -  	   147.11	  1  _
+2 charge
		      B   	       Y
  1	 D 	    58.52	        -  	 13
  2	 V 	   108.05	   758.49	 12
  3	 P 	   156.58	   708.95	 11
  4	 C 	   236.66	   660.43	 10
  5	 E 	   301.19	   580.34	  9
  6	 D 	   358.70	   515.82	  8
  7	 K 	   422.75	   458.31	  7
  8	 F 	   496.28	   394.26	  6
  9	 S 	   539.80	   320.73	  5
 10	 F 	   613.33	   277.21	  4
 11	 V 	   662.86	   203.68	  3
 12	 C 	   742.95	   154.14	  2
 13	 K 	        -  	    74.06	  1  _
```

**57. IPI00298281 Laminin subunit gamma-1**

| Sequence | Charge | M+H+ | XCorr | DeltaCn |
| --- | --- | --- | --- | --- |
| R.LNTFGDEVFNDPK.V | 2 | 1496.603 | 3.024 | 0.44 |

+1 charge

```
		      B   	       Y
  1	 L 	   114.09	        -  	 13
  2	 N 	   228.13	  1382.62	 12
  3	 T 	   329.18	  1268.58	 11
  4	 F 	   476.25	  1167.53	 10
  5	 G 	   533.27	  1020.46	  9
  6	 D 	   648.30	   963.44	  8
  7	 E 	   777.34	   848.41	  7
  8	 V 	   876.41	   719.37	  6
  9	 F 	  1023.48	   620.30	  5
 10	 N 	  1137.52	   473.24	  4
 11	 D 	  1252.55	   359.19	  3
 12	 P 	  1349.60	   244.17	  2
 13	 K 	        -  	   147.11	  1  _
+2 charge
		      B   	       Y
  1	 L 	    57.55	        -  	 13
  2	 N 	   114.57	   691.81	 12
  3	 T 	   165.09	   634.79	 11
  4	 F 	   238.63	   584.27	 10
  5	 G 	   267.14	   510.74	  9
  6	 D 	   324.65	   482.22	  8
  7	 E 	   389.17	   424.71	  7
  8	 V 	   438.71	   360.19	  6
  9	 F 	   512.24	   310.66	  5
 10	 N 	   569.26	   237.12	  4
 11	 D 	   626.78	   180.10	  3
 12	 P 	   675.30	   122.59	  2
 13	 K 	        -  	    74.06	  1  _
```

**58. IPI00102300 Isoform 3 of Platelet glycoprotein VI**

| Sequence | Charge | M+H+ | XCorr | DeltaCn |
| --- | --- | --- | --- | --- |
| R.YQDQAVLFIPAMK.R | 2 | 1524.809 | 3.606 | 0.525 |

+1 charge

```
		      B   	       Y
  1	 Y 	   164.07	        -  	 13
  2	 Q 	   292.13	  1360.73	 12
  3	 D 	   407.16	  1232.67	 11
  4	 Q 	   535.21	  1117.64	 10
  5	 A 	   606.25	   989.59	  9
  6	 V 	   705.32	   918.55	  8
  7	 L 	   818.40	   819.48	  7
  8	 F 	   965.47	   706.40	  6
  9	 I 	  1078.56	   559.33	  5
 10	 P 	  1175.61	   446.24	  4
 11	 A 	  1246.65	   349.19	  3
 12	 M 	  1377.69	   278.15	  2
 13	 K 	        -  	   147.11	  1  _
+2 charge
		      B   	       Y
  1	 Y 	    82.54	        -  	 13
  2	 Q 	   146.57	   680.87	 12
  3	 D 	   204.08	   616.84	 11
  4	 Q 	   268.11	   559.33	 10
  5	 A 	   303.63	   495.30	  9
  6	 V 	   353.16	   459.78	  8
  7	 L 	   409.71	   410.24	  7
  8	 F 	   483.24	   353.70	  6
  9	 I 	   539.78	   280.17	  5
 10	 P 	   588.31	   223.63	  4
 11	 A 	   623.83	   175.10	  3
 12	 M 	   689.35	   139.58	  2
 13	 K 	        -  	    74.06	  1  _
```

**59. IPI00028911 Dystroglycan**

| Sequence | Charge | M+H+ | XCorr | DeltaCn |
| --- | --- | --- | --- | --- |
| R.VTIPTDLIASSGDIIK.V | 2 | 1643.903 | 3.754 | 0.513 |

+1 charge

```
		      B   	       Y
  1	 V 	   100.08	        -  	 16
  2	 T 	   201.12	  1543.86	 15
  3	 I 	   314.21	  1442.81	 14
  4	 P 	   411.26	  1329.73	 13
  5	 T 	   512.31	  1232.67	 12
  6	 D 	   627.33	  1131.63	 11
  7	 L 	   740.42	  1016.60	 10
  8	 I 	   853.50	   903.51	  9
  9	 A 	   924.54	   790.43	  8
 10	 S 	  1011.57	   719.39	  7
 11	 S 	  1098.60	   632.36	  6
 12	 G 	  1155.63	   545.33	  5
 13	 D 	  1270.65	   488.31	  4
 14	 I 	  1383.74	   373.28	  3
 15	 I 	  1496.82	   260.20	  2
 16	 K 	        -  	   147.11	  1  _
+2 charge
		      B   	       Y
  1	 V 	    50.54	        -  	 16
  2	 T 	   101.07	   772.43	 15
  3	 I 	   157.61	   721.91	 14
  4	 P 	   206.13	   665.37	 13
  5	 T 	   256.66	   616.84	 12
  6	 D 	   314.17	   566.32	 11
  7	 L 	   370.71	   508.80	 10
  8	 I 	   427.26	   452.26	  9
  9	 A 	   462.77	   395.72	  8
 10	 S 	   506.29	   360.20	  7
 11	 S 	   549.81	   316.68	  6
 12	 G 	   578.32	   273.17	  5
 13	 D 	   635.83	   244.66	  4
 14	 I 	   692.37	   187.14	  3
 15	 I 	   748.91	   130.60	  2
 16	 K 	        -  	    74.06	  1  _
```

**60. IPI00304273 Apolipoprotein A-IV**

| Sequence | Charge | M+H+ | XCorr | DeltaCn |
| --- | --- | --- | --- | --- |
| K.SELTQQLNALFQDK.L | 2 | 1635.8 | 3.238 | 0.436 |

+1 charge

```
		      B   	       Y
  1	 S 	    88.04	        -  	 14
  2	 E 	   217.08	  1547.81	 13
  3	 L 	   330.17	  1418.76	 12
  4	 T 	   431.21	  1305.68	 11
  5	 Q 	   559.27	  1204.63	 10
  6	 Q 	   687.33	  1076.57	  9
  7	 L 	   800.41	   948.51	  8
  8	 N 	   914.46	   835.43	  7
  9	 A 	   985.49	   721.39	  6
 10	 L 	  1098.58	   650.35	  5
 11	 F 	  1245.65	   537.27	  4
 12	 Q 	  1373.71	   390.20	  3
 13	 D 	  1488.73	   262.14	  2
 14	 K 	        -  	   147.11	  1  _
+2 charge
		      B   	       Y
  1	 S 	    44.52	        -  	 14
  2	 E 	   109.04	   774.41	 13
  3	 L 	   165.59	   709.89	 12
  4	 T 	   216.11	   653.34	 11
  5	 Q 	   280.14	   602.82	 10
  6	 Q 	   344.17	   538.79	  9
  7	 L 	   400.71	   474.76	  8
  8	 N 	   457.73	   418.22	  7
  9	 A 	   493.25	   361.20	  6
 10	 L 	   549.79	   325.68	  5
 11	 F 	   623.33	   269.14	  4
 12	 Q 	   687.36	   195.60	  3
 13	 D 	   744.87	   131.57	  2
 14	 K 	        -  	    74.06	  1  _
```

**61. IPI00021091 Isoform 1 of Leucine-rich glioma-inactivated protein 1**

| Sequence | Charge | M+H+ | XCorr | DeltaCn |
| --- | --- | --- | --- | --- |
| K.CPAVCTCTKDNALCENAR.S | 2 | 2141.3 | 3.799 | 0.204 |

+1 charge

```
		      B   	       Y
  1	 C 	   161.17	        -  	 18
  2	 P 	   258.23	  1980.28	 17
  3	 A 	   329.26	  1883.22	 16
  4	 V 	   428.33	  1812.19	 15
  5	 C 	   588.50	  1713.12	 14
  6	 T 	   689.54	  1552.95	 13
  7	 C 	   849.71	  1451.91	 12
  8	 T 	   950.76	  1291.74	 11
  9	 K 	  1078.85	  1190.69	 10
 10	 D 	  1193.88	  1062.60	  9
 11	 N 	  1307.92	   947.57	  8
 12	 A 	  1378.96	   833.53	  7
 13	 L 	  1492.04	   762.49	  6
 14	 C 	  1652.21	   649.41	  5
 15	 E 	  1781.25	   489.24	  4
 16	 N 	  1895.29	   360.20	  3
 17	 A 	  1966.33	   246.16	  2
 18	 R 	        -  	   175.12	  1  _
+2 charge
		      B   	       Y
  1	 C 	    81.09	        -  	 18
  2	 P 	   129.62	   990.64	 17
  3	 A 	   165.13	   942.12	 16
  4	 V 	   214.67	   906.60	 15
  5	 C 	   294.75	   857.06	 14
  6	 T 	   345.28	   776.98	 13
  7	 C 	   425.36	   726.46	 12
  8	 T 	   475.88	   646.37	 11
  9	 K 	   539.93	   595.85	 10
 10	 D 	   597.44	   531.80	  9
 11	 N 	   654.46	   474.29	  8
 12	 A 	   689.98	   417.27	  7
 13	 L 	   746.52	   381.75	  6
 14	 C 	   826.61	   325.21	  5
 15	 E 	   891.13	   245.12	  4
 16	 N 	   948.15	   180.60	  3
 17	 A 	   983.67	   123.58	  2
 18	 R 	        -  	    88.06	  1  _
```

**62. IPI00307446 Protocadherin-24**

| Sequence | Charge | M+H+ | XCorr | DeltaCn |
| --- | --- | --- | --- | --- |
| K.TFVIIPELVLPNR.E | 2 | 1511.833 | 2.817 | 0.493 |

+1 charge

```
		      B   	       Y
  1	 T 	   102.05	        -  	 13
  2	 F 	   249.12	  1409.85	 12
  3	 V 	   348.19	  1262.78	 11
  4	 I 	   461.28	  1163.71	 10
  5	 I 	   574.36	  1050.63	  9
  6	 P 	   671.41	   937.55	  8
  7	 E 	   800.46	   840.49	  7
  8	 L 	   913.54	   711.45	  6
  9	 V 	  1012.61	   598.37	  5
 10	 L 	  1125.69	   499.30	  4
 11	 P 	  1222.74	   386.21	  3
 12	 N 	  1336.79	   289.16	  2
 13	 R 	        -  	   175.12	  1  _
+2 charge
		      B   	       Y
  1	 T 	    51.53	        -  	 13
  2	 F 	   125.07	   705.43	 12
  3	 V 	   174.60	   631.90	 11
  4	 I 	   231.14	   582.36	 10
  5	 I 	   287.68	   525.82	  9
  6	 P 	   336.21	   469.28	  8
  7	 E 	   400.73	   420.75	  7
  8	 L 	   457.27	   356.23	  6
  9	 V 	   506.81	   299.69	  5
 10	 L 	   563.35	   250.15	  4
 11	 P 	   611.88	   193.61	  3
 12	 N 	   668.90	   145.08	  2
 13	 R 	        -  	    88.06	  1  _
```

**63. IPI00298547 Protein DJ-1**

| Sequence | Charge | M+H+ | XCorr | DeltaCn |
| --- | --- | --- | --- | --- |
| R.GPGTSFEFALAIVEALNGK.E | 2 | 1922.17 | 3.736 | 0.53 |

+1 charge

```
		      B   	       Y
  1	 G 	    58.03	        -  	 19
  2	 P 	   155.08	  1863.99	 18
  3	 G 	   212.10	  1766.93	 17
  4	 T 	   313.15	  1709.91	 16
  5	 S 	   400.18	  1608.86	 15
  6	 F 	   547.25	  1521.83	 14
  7	 E 	   676.29	  1374.76	 13
  8	 F 	   823.36	  1245.72	 12
  9	 A 	   894.40	  1098.65	 11
 10	 L 	  1007.48	  1027.61	 10
 11	 A 	  1078.52	   914.53	  9
 12	 I 	  1191.60	   843.49	  8
 13	 V 	  1290.67	   730.41	  7
 14	 E 	  1419.72	   631.34	  6
 15	 A 	  1490.75	   502.30	  5
 16	 L 	  1603.84	   431.26	  4
 17	 N 	  1717.88	   318.18	  3
 18	 G 	  1774.90	   204.13	  2
 19	 K 	        -  	   147.11	  1  _
+2 charge
		      B   	       Y
  1	 G 	    29.52	        -  	 19
  2	 P 	    78.04	   932.50	 18
  3	 G 	   106.56	   883.97	 17
  4	 T 	   157.08	   855.46	 16
  5	 S 	   200.59	   804.94	 15
  6	 F 	   274.13	   761.42	 14
  7	 E 	   338.65	   687.89	 13
  8	 F 	   412.18	   623.36	 12
  9	 A 	   447.70	   549.83	 11
 10	 L 	   504.25	   514.31	 10
 11	 A 	   539.76	   457.77	  9
 12	 I 	   596.31	   422.25	  8
 13	 V 	   645.84	   365.71	  7
 14	 E 	   710.36	   316.17	  6
 15	 A 	   745.88	   251.65	  5
 16	 L 	   802.42	   216.13	  4
 17	 N 	   859.44	   159.59	  3
 18	 G 	   887.95	   102.57	  2
 19	 K 	        -  	    74.06	  1  _
```

**64. IPI00305064 Isoform 1 of CD44 antigen**

| Sequence | Charge | M+H+ | XCorr | DeltaCn |
| --- | --- | --- | --- | --- |
| R.YGFIEGHVVIPR.I | 2 | 1387.611 | 4.207 | 0.537 |

+1 charge

```
		      B   	       Y
  1	 Y 	   164.07	        -  	 12
  2	 G 	   221.09	  1223.69	 11
  3	 F 	   368.16	  1166.67	 10
  4	 I 	   481.24	  1019.60	  9
  5	 E 	   610.29	   906.52	  8
  6	 G 	   667.31	   777.47	  7
  7	 H 	   804.37	   720.45	  6
  8	 V 	   903.44	   583.39	  5
  9	 V 	  1002.50	   484.32	  4
 10	 I 	  1115.59	   385.26	  3
 11	 P 	  1212.64	   272.17	  2
 12	 R 	        -  	   175.12	  1  _
+2 charge
		      B   	       Y
  1	 Y 	    82.54	        -  	 12
  2	 G 	   111.05	   612.35	 11
  3	 F 	   184.58	   583.84	 10
  4	 I 	   241.13	   510.30	  9
  5	 E 	   305.65	   453.76	  8
  6	 G 	   334.16	   389.24	  7
  7	 H 	   402.69	   360.73	  6
  8	 V 	   452.22	   292.20	  5
  9	 V 	   501.76	   242.67	  4
 10	 I 	   558.30	   193.13	  3
 11	 P 	   606.82	   136.59	  2
 12	 R 	        -  	    88.06	  1  _
```

65. IPI00026199 Glutathione peroxidase 3

| Sequence | Charge | M+H+ | XCorr | DeltaCn |
| --- | --- | --- | --- | --- |
| K.FLVGPDGIPIMR.W | 2 | 1315.609 | 2.881 | 0.222 |

+1 charge

```
		      B   	       Y
  1	 F 	   148.08	        -  	 12
  2	 L 	   261.16	  1167.66	 11
  3	 V 	   360.23	  1054.57	 10
  4	 G 	   417.25	   955.50	  9
  5	 P 	   514.30	   898.48	  8
  6	 D 	   629.33	   801.43	  7
  7	 G 	   686.35	   686.40	  6
  8	 I 	   799.43	   629.38	  5
  9	 P 	   896.49	   516.30	  4
 10	 I 	  1009.57	   419.24	  3
 11	 M 	  1140.61	   306.16	  2
 12	 R 	        -  	   175.12	  1  _
+2 charge
		      B   	       Y
  1	 F 	    74.54	        -  	 12
  2	 L 	   131.08	   584.33	 11
  3	 V 	   180.62	   527.79	 10
  4	 G 	   209.13	   478.26	  9
  5	 P 	   257.65	   449.74	  8
  6	 D 	   315.17	   401.22	  7
  7	 G 	   343.68	   343.70	  6
  8	 I 	   400.22	   315.19	  5
  9	 P 	   448.75	   258.65	  4
 10	 I 	   505.29	   210.13	  3
 11	 M 	   570.81	   153.58	  2
 12	 R 	        -  	    88.06	  1  _
```

66. IPI00215983 Carbonic anhydrase 1

| Sequence | Charge | M+H+ | XCorr | DeltaCn |
| --- | --- | --- | --- | --- |
| K.ADGLAVIGVLMK.V | 2 | 1187.478 | 3.986 | 0.49 |

+1 charge

```
		      B   	       Y
  1	 A 	    72.04	        -  	 12
  2	 D 	   187.07	  1115.65	 11
  3	 G 	   244.09	  1000.62	 10
  4	 L 	   357.18	   943.60	  9
  5	 A 	   428.21	   830.52	  8
  6	 V 	   527.28	   759.48	  7
  7	 I 	   640.37	   660.41	  6
  8	 G 	   697.39	   547.33	  5
  9	 V 	   796.46	   490.31	  4
 10	 L 	   909.54	   391.24	  3
 11	 M 	  1040.58	   278.15	  2
 12	 K 	        -  	   147.11	  1  _
+2 charge
		      B   	       Y
  1	 A 	    36.53	        -  	 12
  2	 D 	    94.04	   558.33	 11
  3	 G 	   122.55	   500.81	 10
  4	 L 	   179.09	   472.30	  9
  5	 A 	   214.61	   415.76	  8
  6	 V 	   264.14	   380.24	  7
  7	 I 	   320.69	   330.71	  6
  8	 G 	   349.20	   274.17	  5
  9	 V 	   398.73	   245.66	  4
 10	 L 	   455.27	   196.12	  3
 11	 M 	   520.79	   139.58	  2
 12	 K 	        -  	    74.06	  1  _
```

**67. IPI00020996 Insulin-like growth factor-binding protein complex acid labile subunit**

| Sequence | Charge | M+H+ | XCorr | DeltaCn |
| --- | --- | --- | --- | --- |
| R.VAGLLEDTFPGLLGLR.V | 2 | 1671.962 | 3.853 | 0.518 |

+1 charge

```
		      B   	       Y
  1	 V 	   100.08	        -  	 16
  2	 A 	   171.11	  1571.88	 15
  3	 G 	   228.13	  1500.84	 14
  4	 L 	   341.22	  1443.82	 13
  5	 L 	   454.30	  1330.74	 12
  6	 E 	   583.34	  1217.65	 11
  7	 D 	   698.37	  1088.61	 10
  8	 T 	   799.42	   973.58	  9
  9	 F 	   946.49	   872.54	  8
 10	 P 	  1043.54	   725.47	  7
 11	 G 	  1100.56	   628.41	  6
 12	 L 	  1213.65	   571.39	  5
 13	 L 	  1326.73	   458.31	  4
 14	 G 	  1383.75	   345.22	  3
 15	 L 	  1496.84	   288.20	  2
 16	 R 	        -  	   175.12	  1  _
+2 charge
		      B   	       Y
  1	 V 	    50.54	        -  	 16
  2	 A 	    86.06	   786.44	 15
  3	 G 	   114.57	   750.92	 14
  4	 L 	   171.11	   722.41	 13
  5	 L 	   227.65	   665.87	 12
  6	 E 	   292.18	   609.33	 11
  7	 D 	   349.69	   544.81	 10
  8	 T 	   400.21	   487.30	  9
  9	 F 	   473.75	   436.77	  8
 10	 P 	   522.27	   363.24	  7
 11	 G 	   550.78	   314.71	  6
 12	 L 	   607.33	   286.20	  5
 13	 L 	   663.87	   229.66	  4
 14	 G 	   692.38	   173.12	  3
 15	 L 	   748.92	   144.61	  2
 16	 R 	        -  	    88.06	  1  _
```

**68. IPI00001759 Oxidized low-density lipoprotein receptor 1**

| Sequence | Charge | M+H+ | XCorr | DeltaCn |
| --- | --- | --- | --- | --- |
| R.GAVSQTYPSGTCAYIQR.G | 2 | 1860.011 | 3.037 | 0.286 |

+1 charge

```
		      B   	       Y
  1	 G 	    58.03	        -  	 17
  2	 A 	   129.07	  1801.99	 16
  3	 V 	   228.13	  1730.95	 15
  4	 S 	   315.17	  1631.88	 14
  5	 Q 	   443.22	  1544.85	 13
  6	 T 	   544.27	  1416.79	 12
  7	 Y 	   707.34	  1315.74	 11
  8	 P 	   804.39	  1152.68	 10
  9	 S 	   891.42	  1055.63	  9
 10	 G 	   948.44	   968.60	  8
 11	 T 	  1049.49	   911.57	  7
 12	 C 	  1209.66	   810.53	  6
 13	 A 	  1280.69	   650.36	  5
 14	 Y 	  1443.76	   579.32	  4
 15	 I 	  1556.84	   416.26	  3
 16	 Q 	  1684.90	   303.18	  2
 17	 R 	        -  	   175.12	  1  _
+2 charge
		      B   	       Y
  1	 G 	    29.52	        -  	 17
  2	 A 	    65.04	   901.50	 16
  3	 V 	   114.57	   865.98	 15
  4	 S 	   158.09	   816.45	 14
  5	 Q 	   222.12	   772.93	 13
  6	 T 	   272.64	   708.90	 12
  7	 Y 	   354.17	   658.38	 11
  8	 P 	   402.70	   576.84	 10
  9	 S 	   446.21	   528.32	  9
 10	 G 	   474.72	   484.80	  8
 11	 T 	   525.25	   456.29	  7
 12	 C 	   605.33	   405.77	  6
 13	 A 	   640.85	   325.68	  5
 14	 Y 	   722.38	   290.17	  4
 15	 I 	   778.92	   208.63	  3
 16	 Q 	   842.95	   152.09	  2
 17	 R 	        -  	    88.06	  1  _
```

**69. IPI00304903 Cornifin-B**

| Sequence | Charge | M+H+ | XCorr | DeltaCn |
| --- | --- | --- | --- | --- |
| K.VPEPCPSIVTPAPAQQK.T | 2 | 1820.073 | 5.3 | 0.631 |

+1 charge

```
		      B   	       Y
  1	 V 	   100.08	        -  	 17
  2	 P 	   197.13	  1720.01	 16
  3	 E 	   326.17	  1622.96	 15
  4	 P 	   423.22	  1493.91	 14
  5	 C 	   583.39	  1396.86	 13
  6	 P 	   680.44	  1236.69	 12
  7	 S 	   767.47	  1139.64	 11
  8	 I 	   880.56	  1052.61	 10
  9	 V 	   979.63	   939.53	  9
 10	 T 	  1080.67	   840.46	  8
 11	 P 	  1177.73	   739.41	  7
 12	 A 	  1248.76	   642.36	  6
 13	 P 	  1345.82	   571.32	  5
 14	 A 	  1416.85	   474.27	  4
 15	 Q 	  1544.91	   403.23	  3
 16	 Q 	  1672.97	   275.17	  2
 17	 K 	        -  	   147.11	  1  _

		      B   	       Y
  1	 V 	    50.54	        -  	 17
  2	 P 	    99.07	   860.51	 16
  3	 E 	   163.59	   811.98	 15
  4	 P 	   212.12	   747.46	 14
  5	 C 	   292.20	   698.93	 13
  6	 P 	   340.72	   618.85	 12
  7	 S 	   384.24	   570.32	 11
  8	 I 	   440.78	   526.81	 10
  9	 V 	   490.32	   470.27	  9
 10	 T 	   540.84	   420.73	  8
 11	 P 	   589.37	   370.21	  7
 12	 A 	   624.89	   321.68	  6
 13	 P 	   673.41	   286.16	  5
 14	 A 	   708.93	   237.64	  4
 15	 Q 	   772.96	   202.12	  3
 16	 Q 	   836.99	   138.09	  2
 17	 K 	        -  	    74.06	  1  _
```

**70. IPI00329688 Protein YIPF3**

| Sequence | Charge | M+H+ | XCorr | DeltaCn |
| --- | --- | --- | --- | --- |
| R.DIPAMLPAAR.L | 1 | 1055.276 | 2.902 | 0.249 |

+1 charge

```
		      B   	       Y
  1	 D 	   116.03	        -  	 10
  2	 I 	   229.12	   939.54	  9
  3	 P 	   326.17	   826.46	  8
  4	 A 	   397.21	   729.41	  7
  5	 M 	   528.25	   658.37	  6
  6	 L 	   641.33	   527.33	  5
  7	 P 	   738.39	   414.25	  4
  8	 A 	   809.42	   317.19	  3
  9	 A 	   880.46	   246.16	  2
 10	 R 	        -  	   175.12	  1  _
```

**71. IPI00297284 insulin-like growth factor binding protein 2, 36kDa precursor**

| Sequence | Charge | M+H+ | XCorr | DeltaCn |
| --- | --- | --- | --- | --- |
| R.LAACGPPPVAPPAAVAAVAGGAR.M | 2 | 2042.364 | 4.221 | 0.591 |

+1 charge

```
		      B   	       Y
  1	 L 	   114.09	        -  	 23
  2	 A 	   185.13	  1928.15	 22
  3	 A 	   256.17	  1857.11	 21
  4	 C 	   416.33	  1786.08	 20
  5	 G 	   473.35	  1625.91	 19
  6	 P 	   570.41	  1568.89	 18
  7	 P 	   667.46	  1471.84	 17
  8	 P 	   764.51	  1374.79	 16
  9	 V 	   863.58	  1277.73	 15
 10	 A 	   934.62	  1178.66	 14
 11	 P 	  1031.67	  1107.63	 13
 12	 P 	  1128.72	  1010.57	 12
 13	 A 	  1199.76	   913.52	 11
 14	 A 	  1270.80	   842.48	 10
 15	 V 	  1369.86	   771.45	  9
 16	 A 	  1440.90	   672.38	  8
 17	 A 	  1511.94	   601.34	  7
 18	 V 	  1611.01	   530.30	  6
 19	 A 	  1682.04	   431.24	  5
 20	 G 	  1739.07	   360.20	  4
 21	 G 	  1796.09	   303.18	  3
 22	 A 	  1867.12	   246.16	  2
 23	 R 	        -  	   175.12	  1  _


+2 charge
		      B   	       Y
  1	 L 	    57.55	        -  	 23
  2	 A 	    93.07	   964.58	 22
  3	 A 	   128.59	   929.06	 21
  4	 C 	   208.67	   893.54	 20
  5	 G 	   237.18	   813.46	 19
  6	 P 	   285.71	   784.95	 18
  7	 P 	   334.23	   736.42	 17
  8	 P 	   382.76	   687.90	 16
  9	 V 	   432.29	   639.37	 15
 10	 A 	   467.81	   589.84	 14
 11	 P 	   516.34	   554.32	 13
 12	 P 	   564.86	   505.79	 12
 13	 A 	   600.38	   457.26	 11
 14	 A 	   635.90	   421.75	 10
 15	 V 	   685.44	   386.23	  9
 16	 A 	   720.95	   336.69	  8
 17	 A 	   756.47	   301.17	  7
 18	 V 	   806.01	   265.66	  6
 19	 A 	   841.53	   216.12	  5
 20	 G 	   870.04	   180.60	  4
 21	 G 	   898.55	   152.09	  3
 22	 A 	   934.07	   123.58	  2
 23	 R 	        -  	    88.06	  1  _
```

**72. IPI00025512 Heat shock protein beta-1**

| Sequence | Charge | M+H+ | XCorr | DeltaCn |
| --- | --- | --- | --- | --- |
| R.VSLDVNHFAPDELTVK.T | 2 | 1784.991 | 4.453 | 0.455 |

+1 charge

```
		      B   	       Y
  1	 V 	   100.08	        -  	 16
  2	 S 	   187.11	  1684.85	 15
  3	 L 	   300.19	  1597.82	 14
  4	 D 	   415.22	  1484.74	 13
  5	 V 	   514.29	  1369.71	 12
  6	 N 	   628.33	  1270.64	 11
  7	 H 	   765.39	  1156.60	 10
  8	 F 	   912.46	  1019.54	  9
  9	 A 	   983.49	   872.47	  8
 10	 P 	  1080.55	   801.44	  7
 11	 D 	  1195.57	   704.38	  6
 12	 E 	  1324.62	   589.36	  5
 13	 L 	  1437.70	   460.31	  4
 14	 T 	  1538.75	   347.23	  3
 15	 V 	  1637.82	   246.18	  2
 16	 K 	        -  	   147.11	  1  _
+2 charge
		      B   	       Y
  1	 V 	    50.54	        -  	 16
  2	 S 	    94.06	   842.93	 15
  3	 L 	   150.60	   799.41	 14
  4	 D 	   208.11	   742.87	 13
  5	 V 	   257.65	   685.36	 12
  6	 N 	   314.67	   635.82	 11
  7	 H 	   383.20	   578.80	 10
  8	 F 	   456.73	   510.27	  9
  9	 A 	   492.25	   436.74	  8
 10	 P 	   540.78	   401.22	  7
 11	 D 	   598.29	   352.69	  6
 12	 E 	   662.81	   295.18	  5
 13	 L 	   719.35	   230.66	  4
 14	 T 	   769.88	   174.12	  3
 15	 V 	   819.41	   123.59	  2
 16	 K 	        -  	    74.06	  1  _
```

**73. IPI00176222 Isoform 1 of Zinc finger protein 549**

| Sequence | Charge | M+H+ | XCorr | DeltaCn |
| --- | --- | --- | --- | --- |
| K.WFSFGSNLQQHQNQDSGEKHIRK.E | 3 | 2772.975 | 3.766 | 0.356 |

+1 charge

```
		      B   	       Y
  1	 W 	   187.09	        -  	 23
  2	 F 	   334.16	  2585.26	 22
  3	 S 	   421.19	  2438.19	 21
  4	 F 	   568.26	  2351.16	 20
  5	 G 	   625.28	  2204.09	 19
  6	 S 	   712.31	  2147.07	 18
  7	 N 	   826.35	  2060.04	 17
  8	 L 	   939.44	  1946.00	 16
  9	 Q 	  1067.49	  1832.91	 15
 10	 Q 	  1195.55	  1704.85	 14
 11	 H 	  1332.61	  1576.79	 13
 12	 Q 	  1460.67	  1439.73	 12
 13	 N 	  1574.71	  1311.68	 11
 14	 Q 	  1702.77	  1197.63	 10
 15	 D 	  1817.80	  1069.57	  9
 16	 S 	  1904.83	   954.55	  8
 17	 G 	  1961.85	   867.52	  7
 18	 E 	  2090.90	   810.49	  6
 19	 K 	  2218.99	   681.45	  5
 20	 H 	  2356.05	   553.36	  4
 21	 I 	  2469.13	   416.30	  3
 22	 R 	  2625.23	   303.21	  2
 23	 K 	        -  	   147.11	  1  _


+2 charge
		      B   	       Y
  1	 W 	    94.05	        -  	 23
  2	 F 	   167.58	  1293.13	 22
  3	 S 	   211.10	  1219.60	 21
  4	 F 	   284.63	  1176.08	 20
  5	 G 	   313.14	  1102.55	 19
  6	 S 	   356.66	  1074.04	 18
  7	 N 	   413.68	  1030.52	 17
  8	 L 	   470.22	   973.50	 16
  9	 Q 	   534.25	   916.96	 15
 10	 Q 	   598.28	   852.93	 14
 11	 H 	   666.81	   788.90	 13
 12	 Q 	   730.84	   720.37	 12
 13	 N 	   787.86	   656.34	 11
 14	 Q 	   851.89	   599.32	 10
 15	 D 	   909.40	   535.29	  9
 16	 S 	   952.92	   477.78	  8
 17	 G 	   981.43	   434.26	  7
 18	 E 	  1045.95	   405.75	  6
 19	 K 	  1110.00	   341.23	  5
 20	 H 	  1178.53	   277.18	  4
 21	 I 	  1235.07	   208.65	  3
 22	 R 	  1313.12	   152.11	  2
 23	 K 	        -  	    74.06	  1  _

		      B   	       Y
  1	 W 	    63.03	        -  	 23
  2	 F 	   112.06	   862.42	 22
  3	 S 	   141.07	   813.40	 21
  4	 F 	   190.09	   784.39	 20
  5	 G 	   209.10	   735.37	 19
  6	 S 	   238.11	   716.36	 18
  7	 N 	   276.12	   687.35	 17
  8	 L 	   313.82	   649.34	 16
  9	 Q 	   356.50	   611.64	 15
 10	 Q 	   399.19	   568.96	 14
 11	 H 	   444.88	   526.27	 13
 12	 Q 	   487.56	   480.58	 12
 13	 N 	   525.58	   437.90	 11
 14	 Q 	   568.26	   399.88	 10
 15	 D 	   606.60	   357.20	  9
 16	 S 	   635.62	   318.85	  8
 17	 G 	   654.62	   289.84	  7
 18	 E 	   697.64	   270.84	  6
 19	 K 	   740.33	   227.82	  5
 20	 H 	   786.02	   185.12	  4
 21	 I 	   823.72	   139.44	  3
 22	 R 	   875.75	   101.74	  2
 23	 K 	        -  	    49.71	  1  _
```

**74. IPI00647019 12 kDa protein**

| Sequence | Charge | M+H+ | XCorr | DeltaCn |
| --- | --- | --- | --- | --- |
| R.PLGPADHQAALLGPRLLR.A | 2 | 1896.229 | 2.833 | 0.222 |

+1 charge

```
		      B   	       Y
  1	 P 	    98.06	        -  	 18
  2	 L 	   211.14	  1798.04	 17
  3	 G 	   268.17	  1684.96	 16
  4	 P 	   365.22	  1627.94	 15
  5	 A 	   436.26	  1530.89	 14
  6	 D 	   551.28	  1459.85	 13
  7	 H 	   688.34	  1344.82	 12
  8	 Q 	   816.40	  1207.76	 11
  9	 A 	   887.44	  1079.70	 10
 10	 A 	   958.47	  1008.67	  9
 11	 L 	  1071.56	   937.63	  8
 12	 L 	  1184.64	   824.55	  7
 13	 G 	  1241.66	   711.46	  6
 14	 P 	  1338.72	   654.44	  5
 15	 R 	  1494.82	   557.39	  4
 16	 L 	  1607.90	   401.29	  3
 17	 L 	  1720.99	   288.20	  2
 18	 R 	        -  	   175.12	  1  _
+2 charge
		      B   	       Y
  1	 P 	    49.53	        -  	 18
  2	 L 	   106.08	   899.53	 17
  3	 G 	   134.59	   842.98	 16
  4	 P 	   183.11	   814.47	 15
  5	 A 	   218.63	   765.95	 14
  6	 D 	   276.14	   730.43	 13
  7	 H 	   344.67	   672.91	 12
  8	 Q 	   408.70	   604.39	 11
  9	 A 	   444.22	   540.36	 10
 10	 A 	   479.74	   504.84	  9
 11	 L 	   536.28	   469.32	  8
 12	 L 	   592.82	   412.78	  7
 13	 G 	   621.34	   356.23	  6
 14	 P 	   669.86	   327.72	  5
 15	 R 	   747.91	   279.20	  4
 16	 L 	   804.45	   201.15	  3
 17	 L 	   861.00	   144.61	  2
 18	 R 	        -  	    88.06	  1  _
```

**75. IPI00794461 Histone H2B type 1-N**

| Sequence | Charge | M+H+ | XCorr | DeltaCn |
| --- | --- | --- | --- | --- |
| K.AMGIMNSFVNDIFER.I | 2 | 1745.017 | 4.528 | 0.531 |

+1 charge

```
		      B   	       Y
  1	 A 	    72.04	        -  	 15
  2	 M 	   203.08	  1672.78	 14
  3	 G 	   260.11	  1541.74	 13
  4	 I 	   373.19	  1484.72	 12
  5	 M 	   504.23	  1371.64	 11
  6	 N 	   618.27	  1240.60	 10
  7	 S 	   705.31	  1126.55	  9
  8	 F 	   852.37	  1039.52	  8
  9	 V 	   951.44	   892.45	  7
 10	 N 	  1065.49	   793.38	  6
 11	 D 	  1180.51	   679.34	  5
 12	 I 	  1293.60	   564.31	  4
 13	 F 	  1440.67	   451.23	  3
 14	 E 	  1569.71	   304.16	  2
 15	 R 	        -  	   175.12	  1  _
+2 charge
		      B   	       Y
  1	 A 	    36.53	        -  	 15
  2	 M 	   102.05	   836.89	 14
  3	 G 	   130.56	   771.37	 13
  4	 I 	   187.10	   742.86	 12
  5	 M 	   252.62	   686.32	 11
  6	 N 	   309.64	   620.80	 10
  7	 S 	   353.16	   563.78	  9
  8	 F 	   426.69	   520.26	  8
  9	 V 	   476.22	   446.73	  7
 10	 N 	   533.25	   397.20	  6
 11	 D 	   590.76	   340.17	  5
 12	 I 	   647.30	   282.66	  4
 13	 F 	   720.84	   226.12	  3
 14	 E 	   785.36	   152.58	  2
 15	 R 	        -  	    88.06	  1  _
```

**76. IPI00384401 Myosin-reactive immunoglobulin kappa chain variable region (Fragment)**

| Sequence | Charge | M+H+ | XCorr | DeltaCn |
| --- | --- | --- | --- | --- |
| - .EIVMTQSPATLSVSPGER.A | 2 | 1903.147 | 4.061 | 0.462 |

+1 charge

```
		      B   	       Y
  1	 E 	   130.05	        -  	 18
  2	 I 	   243.13	  1772.92	 17
  3	 V 	   342.20	  1659.84	 16
  4	 M 	   473.24	  1560.77	 15
  5	 T 	   574.29	  1429.73	 14
  6	 Q 	   702.35	  1328.68	 13
  7	 S 	   789.38	  1200.62	 12
  8	 P 	   886.43	  1113.59	 11
  9	 A 	   957.47	  1016.54	 10
 10	 T 	  1058.52	   945.50	  9
 11	 L 	  1171.60	   844.45	  8
 12	 S 	  1258.63	   731.37	  7
 13	 V 	  1357.70	   644.34	  6
 14	 S 	  1444.74	   545.27	  5
 15	 P 	  1541.79	   458.24	  4
 16	 G 	  1598.81	   361.18	  3
 17	 E 	  1727.85	   304.16	  2
 18	 R 	        -  	   175.12	  1  _
+2 charge
		      B   	       Y
  1	 E 	    65.53	        -  	 18
  2	 I 	   122.07	   886.96	 17
  3	 V 	   171.60	   830.42	 16
  4	 M 	   237.13	   780.89	 15
  5	 T 	   287.65	   715.37	 14
  6	 Q 	   351.68	   664.84	 13
  7	 S 	   395.19	   600.81	 12
  8	 P 	   443.72	   557.30	 11
  9	 A 	   479.24	   508.77	 10
 10	 T 	   529.76	   473.25	  9
 11	 L 	   586.31	   422.73	  8
 12	 S 	   629.82	   366.19	  7
 13	 V 	   679.36	   322.67	  6
 14	 S 	   722.87	   273.14	  5
 15	 P 	   771.40	   229.62	  4
 16	 G 	   799.91	   181.10	  3
 17	 E 	   864.43	   152.58	  2
 18	 R 	        -  	    88.06	  1  _
```

**77. IPI00216984 Calmodulin-like protein 3**

| Sequence | Charge | M+H+ | XCorr | DeltaCn |
| --- | --- | --- | --- | --- |
| R.AADTDGDGQVNYEEFVR.V | 2 | 1886.91 | 3.07 | 0.402 |

+1 charge

```
		      B   	       Y
  1	 A 	    72.04	        -  	 17
  2	 A 	   143.08	  1814.78	 16
  3	 D 	   258.11	  1743.75	 15
  4	 T 	   359.16	  1628.72	 14
  5	 D 	   474.18	  1527.67	 13
  6	 G 	   531.20	  1412.64	 12
  7	 D 	   646.23	  1355.62	 11
  8	 G 	   703.25	  1240.60	 10
  9	 Q 	   831.31	  1183.57	  9
 10	 V 	   930.38	  1055.52	  8
 11	 N 	  1044.42	   956.45	  7
 12	 Y 	  1207.49	   842.40	  6
 13	 E 	  1336.53	   679.34	  5
 14	 E 	  1465.57	   550.30	  4
 15	 F 	  1612.64	   421.26	  3
 16	 V 	  1711.71	   274.19	  2
 17	 R 	        -  	   175.12	  1  _
+2 charge
		      B   	       Y
  1	 A 	    36.53	        -  	 17
  2	 A 	    72.04	   907.90	 16
  3	 D 	   129.56	   872.38	 15
  4	 T 	   180.08	   814.86	 14
  5	 D 	   237.60	   764.34	 13
  6	 G 	   266.11	   706.83	 12
  7	 D 	   323.62	   678.31	 11
  8	 G 	   352.13	   620.80	 10
  9	 Q 	   416.16	   592.29	  9
 10	 V 	   465.69	   528.26	  8
 11	 N 	   522.72	   478.73	  7
 12	 Y 	   604.25	   421.71	  6
 13	 E 	   668.77	   340.17	  5
 14	 E 	   733.29	   275.65	  4
 15	 F 	   806.82	   211.13	  3
 16	 V 	   856.36	   137.60	  2
 17	 R 	        -  	    88.06	  1  _
```

**78. IPI00021841 Apolipoprotein A-I**

| Sequence | Charge | M+H+ | XCorr | DeltaCn |
| --- | --- | --- | --- | --- |
| K.DSGRDYVSQFEGSALGK.Q | 2 | 1816.906 | 2.898 | 0.379 |

+1 charge

```
		      B   	       Y
  1	 D 	   116.03	        -  	 17
  2	 S 	   203.07	  1700.82	 16
  3	 G 	   260.09	  1613.79	 15
  4	 R 	   416.19	  1556.77	 14
  5	 D 	   531.22	  1400.67	 13
  6	 Y 	   694.28	  1285.64	 12
  7	 V 	   793.35	  1122.58	 11
  8	 S 	   880.38	  1023.51	 10
  9	 Q 	  1008.44	   936.48	  9
 10	 F 	  1155.51	   808.42	  8
 11	 E 	  1284.55	   661.35	  7
 12	 G 	  1341.57	   532.31	  6
 13	 S 	  1428.60	   475.29	  5
 14	 A 	  1499.64	   388.26	  4
 15	 L 	  1612.72	   317.22	  3
 16	 G 	  1669.75	   204.13	  2
 17	 K 	        -  	   147.11	  1  _
+2 charge
		      B   	       Y
  1	 D 	    58.52	        -  	 17
  2	 S 	   102.04	   850.92	 16
  3	 G 	   130.55	   807.40	 15
  4	 R 	   208.60	   778.89	 14
  5	 D 	   266.11	   700.84	 13
  6	 Y 	   347.64	   643.32	 12
  7	 V 	   397.18	   561.79	 11
  8	 S 	   440.69	   512.26	 10
  9	 Q 	   504.72	   468.74	  9
 10	 F 	   578.26	   404.71	  8
 11	 E 	   642.78	   331.18	  7
 12	 G 	   671.29	   266.66	  6
 13	 S 	   714.80	   238.15	  5
 14	 A 	   750.32	   194.63	  4
 15	 L 	   806.87	   159.11	  3
 16	 G 	   835.38	   102.57	  2
 17	 K 	        -  	    74.06	  1  _
```

**79. IPI00651692 Stromal cell-derived factor 1 theta**

| Sequence | Charge | M+H+ | XCorr | DeltaCn |
| --- | --- | --- | --- | --- |
| K.ILNTPNCALQIVAR.L | 2 | 1583.851 | 3.828 | 0.44 |

+1 charge

```
		      B   	       Y
  1	 I 	   114.09	        -  	 14
  2	 L 	   227.18	  1469.92	 13
  3	 N 	   341.22	  1356.84	 12
  4	 T 	   442.27	  1242.80	 11
  5	 P 	   539.32	  1141.75	 10
  6	 N 	   653.36	  1044.70	  9
  7	 C 	   813.53	   930.65	  8
  8	 A 	   884.56	   770.49	  7
  9	 L 	   997.65	   699.45	  6
 10	 Q 	  1125.71	   586.37	  5
 11	 I 	  1238.79	   458.31	  4
 12	 V 	  1337.86	   345.22	  3
 13	 A 	  1408.90	   246.16	  2
 14	 R 	        -  	   175.12	  1  _
+2 charge
		      B   	       Y
  1	 I 	    57.55	        -  	 14
  2	 L 	   114.09	   735.47	 13
  3	 N 	   171.11	   678.92	 12
  4	 T 	   221.64	   621.90	 11
  5	 P 	   270.16	   571.38	 10
  6	 N 	   327.18	   522.85	  9
  7	 C 	   407.27	   465.83	  8
  8	 A 	   442.79	   385.75	  7
  9	 L 	   499.33	   350.23	  6
 10	 Q 	   563.36	   293.69	  5
 11	 I 	   619.90	   229.66	  4
 12	 V 	   669.43	   173.12	  3
 13	 A 	   704.95	   123.58	  2
 14	 R 	        -  	    88.06	  1  _
```

**80. IPI00297056 Cornulin**

| Sequence | Charge | M+H+ | XCorr | DeltaCn |
| --- | --- | --- | --- | --- |
| R.LLEQEFADVIVKPHDPATVDEVLR.L | 3 | 2735.085 | 3.682 | 0.388 |

+1 charge

```
		      B   	       Y
  1	 L 	   114.09	        -  	 24
  2	 L 	   227.18	  2620.36	 23
  3	 E 	   356.22	  2507.28	 22
  4	 Q 	   484.28	  2378.24	 21
  5	 E 	   613.32	  2250.18	 20
  6	 F 	   760.39	  2121.13	 19
  7	 A 	   831.42	  1974.07	 18
  8	 D 	   946.45	  1903.03	 17
  9	 V 	  1045.52	  1788.00	 16
 10	 I 	  1158.60	  1688.93	 15
 11	 V 	  1257.67	  1575.85	 14
 12	 K 	  1385.77	  1476.78	 13
 13	 P 	  1482.82	  1348.69	 12
 14	 H 	  1619.88	  1251.63	 11
 15	 D 	  1734.91	  1114.57	 10
 16	 P 	  1831.96	   999.55	  9
 17	 A 	  1903.00	   902.49	  8
 18	 T 	  2004.04	   831.46	  7
 19	 V 	  2103.11	   730.41	  6
 20	 D 	  2218.14	   631.34	  5
 21	 E 	  2347.18	   516.31	  4
 22	 V 	  2446.25	   387.27	  3
 23	 L 	  2559.33	   288.20	  2
 24	 R 	        -  	   175.12	  1  _


+2 charge
		      B   	       Y
  1	 L 	    57.55	        -  	 24
  2	 L 	   114.09	  1310.68	 23
  3	 E 	   178.61	  1254.14	 22
  4	 Q 	   242.64	  1189.62	 21
  5	 E 	   307.16	  1125.59	 20
  6	 F 	   380.70	  1061.07	 19
  7	 A 	   416.22	   987.54	 18
  8	 D 	   473.73	   952.02	 17
  9	 V 	   523.26	   894.50	 16
 10	 I 	   579.81	   844.97	 15
 11	 V 	   629.34	   788.43	 14
 12	 K 	   693.39	   738.89	 13
 13	 P 	   741.91	   674.85	 12
 14	 H 	   810.44	   626.32	 11
 15	 D 	   867.96	   557.79	 10
 16	 P 	   916.48	   500.28	  9
 17	 A 	   952.00	   451.75	  8
 18	 T 	  1002.53	   416.23	  7
 19	 V 	  1052.06	   365.71	  6
 20	 D 	  1109.57	   316.17	  5
 21	 E 	  1174.09	   258.66	  4
 22	 V 	  1223.63	   194.14	  3
 23	 L 	  1280.17	   144.61	  2
 24	 R 	        -  	    88.06	  1  _


+3 charge
		      B   	       Y
  1	 L 	    38.70	        -  	 24
  2	 L 	    76.40	   874.13	 23
  3	 E 	   119.41	   836.43	 22
  4	 Q 	   162.10	   793.42	 21
  5	 E 	   205.11	   750.73	 20
  6	 F 	   254.13	   707.72	 19
  7	 A 	   277.81	   658.69	 18
  8	 D 	   316.16	   635.01	 17
  9	 V 	   349.18	   596.67	 16
 10	 I 	   386.87	   563.65	 15
 11	 V 	   419.90	   525.95	 14
 12	 K 	   462.59	   492.93	 13
 13	 P 	   494.94	   450.23	 12
 14	 H 	   540.63	   417.88	 11
 15	 D 	   578.97	   372.20	 10
 16	 P 	   611.32	   333.85	  9
 17	 A 	   635.00	   301.50	  8
 18	 T 	   668.69	   277.82	  7
 19	 V 	   701.71	   244.14	  6
 20	 D 	   740.05	   211.12	  5
 21	 E 	   783.07	   172.78	  4
 22	 V 	   816.09	   129.76	  3
 23	 L 	   853.78	    96.74	  2
 24	 R 	        -  	    59.04	  1  _
```

**81. IPI00940353 Isoform 1 of Gamma-glutamyltransferase 6**

| Sequence | Charge | M+H+ | XCorr | DeltaCn |
| --- | --- | --- | --- | --- |
| R.LLVGPTTLAQEGFLVDTPLAR.A | 2 | 2212.574 | 3.828 | 0.501 |

+1 charge

```
		      B   	       Y
  1	 L 	   114.09	        -  	 21
  2	 L 	   227.18	  2098.15	 20
  3	 V 	   326.24	  1985.07	 19
  4	 G 	   383.27	  1886.00	 18
  5	 P 	   480.32	  1828.98	 17
  6	 T 	   581.37	  1731.93	 16
  7	 T 	   682.41	  1630.88	 15
  8	 L 	   795.50	  1529.83	 14
  9	 A 	   866.53	  1416.75	 13
 10	 Q 	   994.59	  1345.71	 12
 11	 E 	  1123.64	  1217.65	 11
 12	 G 	  1180.66	  1088.61	 10
 13	 F 	  1327.73	  1031.59	  9
 14	 L 	  1440.81	   884.52	  8
 15	 V 	  1539.88	   771.44	  7
 16	 D 	  1654.91	   672.37	  6
 17	 T 	  1755.95	   557.34	  5
 18	 P 	  1853.01	   456.29	  4
 19	 L 	  1966.09	   359.24	  3
 20	 A 	  2037.13	   246.16	  2
 21	 R 	        -  	   175.12	  1  _
+2 charge
		      B   	       Y
  1	 L 	    57.55	        -  	 21
  2	 L 	   114.09	  1049.58	 20
  3	 V 	   163.63	   993.04	 19
  4	 G 	   192.14	   943.50	 18
  5	 P 	   240.66	   914.99	 17
  6	 T 	   291.19	   866.47	 16
  7	 T 	   341.71	   815.94	 15
  8	 L 	   398.25	   765.42	 14
  9	 A 	   433.77	   708.88	 13
 10	 Q 	   497.80	   673.36	 12
 11	 E 	   562.32	   609.33	 11
 12	 G 	   590.83	   544.81	 10
 13	 F 	   664.37	   516.30	  9
 14	 L 	   720.91	   442.76	  8
 15	 V 	   770.44	   386.22	  7
 16	 D 	   827.96	   336.69	  6
 17	 T 	   878.48	   279.17	  5
 18	 P 	   927.01	   228.65	  4
 19	 L 	   983.55	   180.12	  3
 20	 A 	  1019.07	   123.58	  2
 21	 R 	        -  	    88.06	  1  _
```

**82. IPI00409698 Isoform 1 of Transportin-2**

| Sequence | Charge | M+H+ | XCorr | DeltaCn |
| --- | --- | --- | --- | --- |
| K.QECLNNIGDASSLIR.A | 2 | 1690.831 | 3.396 | 0.306 |

+1 charge

```
		      B   	       Y
  1	 Q 	   129.07	        -  	 15
  2	 E 	   258.11	  1561.90	 14
  3	 C 	   418.27	  1432.86	 13
  4	 L 	   531.36	  1272.69	 12
  5	 N 	   645.40	  1159.61	 11
  6	 N 	   759.44	  1045.56	 10
  7	 I 	   872.53	   931.52	  9
  8	 G 	   929.55	   818.44	  8
  9	 D 	  1044.58	   761.42	  7
 10	 A 	  1115.61	   646.39	  6
 11	 S 	  1202.65	   575.35	  5
 12	 S 	  1289.68	   488.32	  4
 13	 L 	  1402.76	   401.29	  3
 14	 I 	  1515.85	   288.20	  2
 15	 R 	        -  	   175.12	  1  _
+2 charge
		      B   	       Y
  1	 Q 	    65.04	        -  	 15
  2	 E 	   129.56	   781.45	 14
  3	 C 	   209.64	   716.93	 13
  4	 L 	   266.18	   636.85	 12
  5	 N 	   323.20	   580.31	 11
  6	 N 	   380.23	   523.29	 10
  7	 I 	   436.77	   466.26	  9
  8	 G 	   465.28	   409.72	  8
  9	 D 	   522.79	   381.21	  7
 10	 A 	   558.31	   323.70	  6
 11	 S 	   601.83	   288.18	  5
 12	 S 	   645.34	   244.66	  4
 13	 L 	   701.88	   201.15	  3
 14	 I 	   758.43	   144.61	  2
 15	 R 	        -  	    88.06	  1  _
```

**83. IPI00218414 Carbonic anhydrase 2**

| Sequence | Charge | M+H+ | XCorr | DeltaCn |
| --- | --- | --- | --- | --- |
| K.AVQQPDGLAVLGIFLK.V | 2 | 1669.989 | 3.831 | 0.397 |

+1 charge

```
		      B   	       Y
  1	 A 	    72.04	        -  	 16
  2	 V 	   171.11	  1597.93	 15
  3	 Q 	   299.17	  1498.86	 14
  4	 Q 	   427.23	  1370.80	 13
  5	 P 	   524.28	  1242.75	 12
  6	 D 	   639.31	  1145.69	 11
  7	 G 	   696.33	  1030.67	 10
  8	 L 	   809.42	   973.64	  9
  9	 A 	   880.45	   860.56	  8
 10	 V 	   979.52	   789.52	  7
 11	 L 	  1092.60	   690.45	  6
 12	 G 	  1149.63	   577.37	  5
 13	 I 	  1262.71	   520.35	  4
 14	 F 	  1409.78	   407.27	  3
 15	 L 	  1522.86	   260.20	  2
 16	 K 	        -  	   147.11	  1  _
+2 charge
		      B   	       Y
  1	 A 	    36.53	        -  	 16
  2	 V 	    86.06	   799.47	 15
  3	 Q 	   150.09	   749.94	 14
  4	 Q 	   214.12	   685.91	 13
  5	 P 	   262.64	   621.88	 12
  6	 D 	   320.16	   573.35	 11
  7	 G 	   348.67	   515.84	 10
  8	 L 	   405.21	   487.33	  9
  9	 A 	   440.73	   430.78	  8
 10	 V 	   490.26	   395.27	  7
 11	 L 	   546.81	   345.73	  6
 12	 G 	   575.32	   289.19	  5
 13	 I 	   631.86	   260.68	  4
 14	 F 	   705.39	   204.14	  3
 15	 L 	   761.94	   130.60	  2
 16	 K 	        -  	    74.06	  1  _
```

**84. IPI00216298 Thioredoxin**

| Sequence | Charge | M+H+ | XCorr | DeltaCn |
| --- | --- | --- | --- | --- |
| K.TAFQEALDAAGDK.L | 2 | 1337.417 | 2.918 | 0.402 |

+1 charge

```
		      B   	       Y
  1	 T 	   102.05	        -  	 13
  2	 A 	   173.09	  1235.59	 12
  3	 F 	   320.16	  1164.55	 11
  4	 Q 	   448.22	  1017.48	 10
  5	 E 	   577.26	   889.43	  9
  6	 A 	   648.30	   760.38	  8
  7	 L 	   761.38	   689.35	  7
  8	 D 	   876.41	   576.26	  6
  9	 A 	   947.45	   461.24	  5
 10	 A 	  1018.48	   390.20	  4
 11	 G 	  1075.51	   319.16	  3
 12	 D 	  1190.53	   262.14	  2
 13	 K 	        -  	   147.11	  1  _
+2 charge
		      B   	       Y
  1	 T 	    51.53	        -  	 13
  2	 A 	    87.05	   618.30	 12
  3	 F 	   160.58	   582.78	 11
  4	 Q 	   224.61	   509.25	 10
  5	 E 	   289.13	   445.22	  9
  6	 A 	   324.65	   380.70	  8
  7	 L 	   381.20	   345.18	  7
  8	 D 	   438.71	   288.63	  6
  9	 A 	   474.23	   231.12	  5
 10	 A 	   509.75	   195.60	  4
 11	 G 	   538.26	   160.08	  3
 12	 D 	   595.77	   131.57	  2
 13	 K 	        -  	    74.06	  1  _
```

**85. IPI00795918 Isoform 1 of Neural cell adhesion molecule 1**

| Sequence | Charge | M+H+ | XCorr | DeltaCn |
| --- | --- | --- | --- | --- |
| K.FFLCQVAGDAK.D | 2 | 1256.426 | 3.529 | 0.439 |

+1 charge

```
		      B   	       Y
  1	 F 	   148.08	        -  	 11
  2	 F 	   295.14	  1108.68	 10
  3	 L 	   408.23	   961.61	  9
  4	 C 	   568.39	   848.53	  8
  5	 Q 	   696.45	   688.36	  7
  6	 V 	   795.52	   560.30	  6
  7	 A 	   866.56	   461.24	  5
  8	 G 	   923.58	   390.20	  4
  9	 D 	  1038.61	   333.18	  3
 10	 A 	  1109.64	   218.15	  2
 11	 K 	        -  	   147.11	  1  _
+2 charge
		      B   	       Y
  1	 F 	    74.54	        -  	 11
  2	 F 	   148.08	   554.84	 10
  3	 L 	   204.62	   481.31	  9
  4	 C 	   284.70	   424.77	  8
  5	 Q 	   348.73	   344.68	  7
  6	 V 	   398.26	   280.66	  6
  7	 A 	   433.78	   231.12	  5
  8	 G 	   462.29	   195.60	  4
  9	 D 	   519.81	   167.09	  3
 10	 A 	   555.33	   109.58	  2
 11	 K 	        -  	    74.06	  1  _
```

**86. IPI00179330 ubiquitin and ribosomal protein S27a precursor**

| Sequence | Charge | M+H+ | XCorr | DeltaCn |
| --- | --- | --- | --- | --- |
| K.TITLEVEPSDTIENVK.A | 2 | 1788.974 | 3.088 | 0.462 |

+1 charge

```
		      B   	       Y
  1	 T 	   102.05	        -  	 16
  2	 I 	   215.14	  1686.88	 15
  3	 T 	   316.19	  1573.80	 14
  4	 L 	   429.27	  1472.75	 13
  5	 E 	   558.31	  1359.66	 12
  6	 V 	   657.38	  1230.62	 11
  7	 E 	   786.42	  1131.55	 10
  8	 P 	   883.48	  1002.51	  9
  9	 S 	   970.51	   905.46	  8
 10	 D 	  1085.54	   818.43	  7
 11	 T 	  1186.58	   703.40	  6
 12	 I 	  1299.67	   602.35	  5
 13	 E 	  1428.71	   489.27	  4
 14	 N 	  1542.75	   360.22	  3
 15	 V 	  1641.82	   246.18	  2
 16	 K 	        -  	   147.11	  1  _
+2 charge
		      B   	       Y
  1	 T 	    51.53	        -  	 16
  2	 I 	   108.07	   843.94	 15
  3	 T 	   158.60	   787.40	 14
  4	 L 	   215.14	   736.88	 13
  5	 E 	   279.66	   680.34	 12
  6	 V 	   329.19	   615.81	 11
  7	 E 	   393.72	   566.28	 10
  8	 P 	   442.24	   501.76	  9
  9	 S 	   485.76	   453.23	  8
 10	 D 	   543.27	   409.72	  7
 11	 T 	   593.80	   352.20	  6
 12	 I 	   650.34	   301.68	  5
 13	 E 	   714.86	   245.14	  4
 14	 N 	   771.88	   180.62	  3
 15	 V 	   821.41	   123.59	  2
 16	 K 	        -  	    74.06	  1  _
```

**87. IPI00382478 Ig heavy chain V-III region TIL**

| Sequence | Charge | M+H+ | XCorr | DeltaCn |
| --- | --- | --- | --- | --- |
| - .EVQLLESGGGLVQPGGSLR.L | 2 | 1897.122 | 3.032 | 0.236 |

+1 charge

```
		      B   	       Y
  1	 E 	   130.05	        -  	 19
  2	 V 	   229.12	  1766.98	 18
  3	 Q 	   357.18	  1667.91	 17
  4	 L 	   470.26	  1539.85	 16
  5	 L 	   583.34	  1426.76	 15
  6	 E 	   712.39	  1313.68	 14
  7	 S 	   799.42	  1184.64	 13
  8	 G 	   856.44	  1097.61	 12
  9	 G 	   913.46	  1040.58	 11
 10	 G 	   970.48	   983.56	 10
 11	 L 	  1083.57	   926.54	  9
 12	 V 	  1182.64	   813.46	  8
 13	 Q 	  1310.70	   714.39	  7
 14	 P 	  1407.75	   586.33	  6
 15	 G 	  1464.77	   489.28	  5
 16	 G 	  1521.79	   432.26	  4
 17	 S 	  1608.82	   375.24	  3
 18	 L 	  1721.91	   288.20	  2
 19	 R 	        -  	   175.12	  1  _
+2 charge
		      B   	       Y
  1	 E 	    65.53	        -  	 19
  2	 V 	   115.06	   883.99	 18
  3	 Q 	   179.09	   834.46	 17
  4	 L 	   235.63	   770.43	 16
  5	 L 	   292.18	   713.89	 15
  6	 E 	   356.70	   657.34	 14
  7	 S 	   400.21	   592.82	 13
  8	 G 	   428.72	   549.31	 12
  9	 G 	   457.23	   520.80	 11
 10	 G 	   485.75	   492.29	 10
 11	 L 	   542.29	   463.77	  9
 12	 V 	   591.82	   407.23	  8
 13	 Q 	   655.85	   357.70	  7
 14	 P 	   704.38	   293.67	  6
 15	 G 	   732.89	   245.14	  5
 16	 G 	   761.40	   216.63	  4
 17	 S 	   804.92	   188.12	  3
 18	 L 	   861.46	   144.61	  2
 19	 R 	        -  	    88.06	  1  _
```

**88. IPI00170635 Secreted and transmembrane protein 1**

| Sequence | Charge | M+H+ | XCorr | DeltaCn |
| --- | --- | --- | --- | --- |
| R.AHGQESAIFNEVAPGYFSR.D | 2 | 2081.233 | 5.109 | 0.595 |

+1 charge

```
		      B   	       Y
  1	 A 	    72.04	        -  	 19
  2	 H 	   209.10	  2008.95	 18
  3	 G 	   266.12	  1871.89	 17
  4	 Q 	   394.18	  1814.87	 16
  5	 E 	   523.23	  1686.81	 15
  6	 S 	   610.26	  1557.77	 14
  7	 A 	   681.30	  1470.74	 13
  8	 I 	   794.38	  1399.70	 12
  9	 F 	   941.45	  1286.62	 11
 10	 N 	  1055.49	  1139.55	 10
 11	 E 	  1184.53	  1025.51	  9
 12	 V 	  1283.60	   896.46	  8
 13	 A 	  1354.64	   797.39	  7
 14	 P 	  1451.69	   726.36	  6
 15	 G 	  1508.71	   629.30	  5
 16	 Y 	  1671.78	   572.28	  4
 17	 F 	  1818.84	   409.22	  3
 18	 S 	  1905.88	   262.15	  2
 19	 R 	        -  	   175.12	  1  _
+2 charge
		      B   	       Y
  1	 A 	    36.53	        -  	 19
  2	 H 	   105.06	  1004.98	 18
  3	 G 	   133.57	   936.45	 17
  4	 Q 	   197.60	   907.94	 16
  5	 E 	   262.12	   843.91	 15
  6	 S 	   305.63	   779.39	 14
  7	 A 	   341.15	   735.87	 13
  8	 I 	   397.69	   700.35	 12
  9	 F 	   471.23	   643.81	 11
 10	 N 	   528.25	   570.28	 10
 11	 E 	   592.77	   513.26	  9
 12	 V 	   642.30	   448.73	  8
 13	 A 	   677.82	   399.20	  7
 14	 P 	   726.35	   363.68	  6
 15	 G 	   754.86	   315.16	  5
 16	 Y 	   836.39	   286.64	  4
 17	 F 	   909.93	   205.11	  3
 18	 S 	   953.44	   131.58	  2
 19	 R 	        -  	    88.06	  1  _
```

**89. IPI00018136 Isoform 1 of Vascular cell adhesion protein 1**

| Sequence | Charge | M+H+ | XCorr | DeltaCn |
| --- | --- | --- | --- | --- |
| K.SQEFLEDADRKSLETK.S | 2 | 1897.033 | 3.226 | 0.246 |

+1 charge

```
		      B   	       Y
  1	 S 	    88.04	        -  	 16
  2	 Q 	   216.10	  1808.90	 15
  3	 E 	   345.14	  1680.84	 14
  4	 F 	   492.21	  1551.80	 13
  5	 L 	   605.29	  1404.73	 12
  6	 E 	   734.34	  1291.65	 11
  7	 D 	   849.36	  1162.61	 10
  8	 A 	   920.40	  1047.58	  9
  9	 D 	  1035.43	   976.54	  8
 10	 R 	  1191.53	   861.52	  7
 11	 K 	  1319.62	   705.41	  6
 12	 S 	  1406.65	   577.32	  5
 13	 L 	  1519.74	   490.29	  4
 14	 E 	  1648.78	   377.20	  3
 15	 T 	  1749.83	   248.16	  2
 16	 K 	        -  	   147.11	  1  _
+2 charge
		      B   	       Y
  1	 S 	    44.52	        -  	 16
  2	 Q 	   108.55	   904.95	 15
  3	 E 	   173.07	   840.93	 14
  4	 F 	   246.61	   776.40	 13
  5	 L 	   303.15	   702.87	 12
  6	 E 	   367.67	   646.33	 11
  7	 D 	   425.18	   581.81	 10
  8	 A 	   460.70	   524.29	  9
  9	 D 	   518.22	   488.77	  8
 10	 R 	   596.27	   431.26	  7
 11	 K 	   660.31	   353.21	  6
 12	 S 	   703.83	   289.16	  5
 13	 L 	   760.37	   245.65	  4
 14	 E 	   824.89	   189.11	  3
 15	 T 	   875.42	   124.58	  2
 16	 K 	        -  	    74.06	  1  _
```

**90. IPI00289831 Isoform PTPS of Receptor-type tyrosine-protein phosphatase S**

| Sequence | Charge | M+H+ | XCorr | DeltaCn |
| --- | --- | --- | --- | --- |
| R.VLAFTSVGDGPLSDPIQVK.T | 2 | 1944.218 | 3.226 | 0.411 |

+1 charge

```
		      B   	       Y
  1	 V 	   100.08	        -  	 19
  2	 L 	   213.16	  1843.98	 18
  3	 A 	   284.20	  1730.90	 17
  4	 F 	   431.27	  1659.86	 16
  5	 T 	   532.31	  1512.79	 15
  6	 S 	   619.34	  1411.74	 14
  7	 V 	   718.41	  1324.71	 13
  8	 G 	   775.43	  1225.64	 12
  9	 D 	   890.46	  1168.62	 11
 10	 G 	   947.48	  1053.59	 10
 11	 P 	  1044.54	   996.57	  9
 12	 L 	  1157.62	   899.52	  8
 13	 S 	  1244.65	   786.44	  7
 14	 D 	  1359.68	   699.40	  6
 15	 P 	  1456.73	   584.38	  5
 16	 I 	  1569.82	   487.32	  4
 17	 Q 	  1697.87	   374.24	  3
 18	 V 	  1796.94	   246.18	  2
 19	 K 	        -  	   147.11	  1  _
+2 charge
		      B   	       Y
  1	 V 	    50.54	        -  	 19
  2	 L 	   107.08	   922.49	 18
  3	 A 	   142.60	   865.95	 17
  4	 F 	   216.14	   830.43	 16
  5	 T 	   266.66	   756.90	 15
  6	 S 	   310.18	   706.38	 14
  7	 V 	   359.71	   662.86	 13
  8	 G 	   388.22	   613.32	 12
  9	 D 	   445.73	   584.81	 11
 10	 G 	   474.25	   527.30	 10
 11	 P 	   522.77	   498.79	  9
 12	 L 	   579.31	   450.26	  8
 13	 S 	   622.83	   393.72	  7
 14	 D 	   680.34	   350.21	  6
 15	 P 	   728.87	   292.69	  5
 16	 I 	   785.41	   244.17	  4
 17	 Q 	   849.44	   187.62	  3
 18	 V 	   898.98	   123.59	  2
 19	 K 	        -  	    74.06	  1  _
```

**91. IPI00219037 Histone H2A.x**

| Sequence | Charge | M+H+ | XCorr | DeltaCn |
| --- | --- | --- | --- | --- |
| R.VTIAQGGVLPNIQAVLLPK.K | 2 | 1932.339 | 4.721 | 0.537 |

+1 charge

```
		      B   	       Y
  1	 V 	   100.08	        -  	 19
  2	 T 	   201.12	  1832.10	 18
  3	 I 	   314.21	  1731.05	 17
  4	 A 	   385.24	  1617.97	 16
  5	 Q 	   513.30	  1546.93	 15
  6	 G 	   570.32	  1418.87	 14
  7	 G 	   627.35	  1361.85	 13
  8	 V 	   726.41	  1304.83	 12
  9	 L 	   839.50	  1205.76	 11
 10	 P 	   936.55	  1092.68	 10
 11	 N 	  1050.59	   995.62	  9
 12	 I 	  1163.68	   881.58	  8
 13	 Q 	  1291.74	   768.50	  7
 14	 A 	  1362.77	   640.44	  6
 15	 V 	  1461.84	   569.40	  5
 16	 L 	  1574.93	   470.33	  4
 17	 L 	  1688.01	   357.25	  3
 18	 P 	  1785.06	   244.17	  2
 19	 K 	        -  	   147.11	  1  _
+2 charge
		      B   	       Y
  1	 V 	    50.54	        -  	 19
  2	 T 	   101.07	   916.55	 18
  3	 I 	   157.61	   866.03	 17
  4	 A 	   193.13	   809.49	 16
  5	 Q 	   257.16	   773.97	 15
  6	 G 	   285.67	   709.94	 14
  7	 G 	   314.18	   681.43	 13
  8	 V 	   363.71	   652.92	 12
  9	 L 	   420.25	   603.38	 11
 10	 P 	   468.78	   546.84	 10
 11	 N 	   525.80	   498.32	  9
 12	 I 	   582.34	   441.29	  8
 13	 Q 	   646.37	   384.75	  7
 14	 A 	   681.89	   320.72	  6
 15	 V 	   731.42	   285.20	  5
 16	 L 	   787.97	   235.67	  4
 17	 L 	   844.51	   179.13	  3
 18	 P 	   893.04	   122.59	  2
 19	 K 	        -  	    74.06	  1  _
```

**92. IPI00032258 Complement C4-A**

| Sequence | Charge | M+H+ | XCorr | DeltaCn |
| --- | --- | --- | --- | --- |
| R.VTASDPLDTLGSEGALSPGGVASLLR.L | 3 | 2484.745 | 5.162 | 0.429 |

+1 charge

```
		      B   	       Y
  1	 V 	   100.08	        -  	 26
  2	 T 	   201.12	  2384.23	 25
  3	 A 	   272.16	  2283.18	 24
  4	 S 	   359.19	  2212.15	 23
  5	 D 	   474.22	  2125.11	 22
  6	 P 	   571.27	  2010.09	 21
  7	 L 	   684.36	  1913.03	 20
  8	 D 	   799.38	  1799.95	 19
  9	 T 	   900.43	  1684.92	 18
 10	 L 	  1013.51	  1583.88	 17
 11	 G 	  1070.54	  1470.79	 16
 12	 S 	  1157.57	  1413.77	 15
 13	 E 	  1286.61	  1326.74	 14
 14	 G 	  1343.63	  1197.69	 13
 15	 A 	  1414.67	  1140.67	 12
 16	 L 	  1527.75	  1069.64	 11
 17	 S 	  1614.79	   956.55	 10
 18	 P 	  1711.84	   869.52	  9
 19	 G 	  1768.86	   772.47	  8
 20	 G 	  1825.88	   715.45	  7
 21	 V 	  1924.95	   658.42	  6
 22	 A 	  1995.99	   559.36	  5
 23	 S 	  2083.02	   488.32	  4
 24	 L 	  2196.10	   401.29	  3
 25	 L 	  2309.19	   288.20	  2
 26	 R 	        -  	   175.12	  1  _


+2 charge
		      B   	       Y
  1	 V 	    50.54	        -  	 26
  2	 T 	   101.07	  1192.62	 25
  3	 A 	   136.58	  1142.09	 24
  4	 S 	   180.10	  1106.58	 23
  5	 D 	   237.61	  1063.06	 22
  6	 P 	   286.14	  1005.55	 21
  7	 L 	   342.68	   957.02	 20
  8	 D 	   400.20	   900.48	 19
  9	 T 	   450.72	   842.97	 18
 10	 L 	   507.26	   792.44	 17
 11	 G 	   535.77	   735.90	 16
 12	 S 	   579.29	   707.39	 15
 13	 E 	   643.81	   663.87	 14
 14	 G 	   672.32	   599.35	 13
 15	 A 	   707.84	   570.84	 12
 16	 L 	   764.38	   535.32	 11
 17	 S 	   807.90	   478.78	 10
 18	 P 	   856.42	   435.26	  9
 19	 G 	   884.93	   386.74	  8
 20	 G 	   913.44	   358.23	  7
 21	 V 	   962.98	   329.72	  6
 22	 A 	   998.50	   280.18	  5
 23	 S 	  1042.01	   244.66	  4
 24	 L 	  1098.56	   201.15	  3
 25	 L 	  1155.10	   144.61	  2
 26	 R 	        -  	    88.06	  1  _


+3 charge
		      B   	       Y
  1	 V 	    34.03	        -  	 26
  2	 T 	    67.71	   795.41	 25
  3	 A 	    91.39	   761.73	 24
  4	 S 	   120.40	   738.05	 23
  5	 D 	   158.74	   709.04	 22
  6	 P 	   191.10	   670.70	 21
  7	 L 	   228.79	   638.35	 20
  8	 D 	   267.13	   600.65	 19
  9	 T 	   300.82	   562.31	 18
 10	 L 	   338.51	   528.63	 17
 11	 G 	   357.52	   490.94	 16
 12	 S 	   386.53	   471.93	 15
 13	 E 	   429.54	   442.92	 14
 14	 G 	   448.55	   399.90	 13
 15	 A 	   472.23	   380.90	 12
 16	 L 	   509.92	   357.22	 11
 17	 S 	   538.93	   319.52	 10
 18	 P 	   571.28	   290.51	  9
 19	 G 	   590.29	   258.16	  8
 20	 G 	   609.30	   239.15	  7
 21	 V 	   642.32	   220.15	  6
 22	 A 	   666.00	   187.12	  5
 23	 S 	   695.01	   163.44	  4
 24	 L 	   732.71	   134.43	  3
 25	 L 	   770.40	    96.74	  2
 26	 R 	        -  	    59.04	  1  _
```

**93. IPI00913871 Lymphocyte antigen 6H**

| Sequence | Charge | M+H+ | XCorr | DeltaCn |
| --- | --- | --- | --- | --- |
| R.HFFSDYLMGFINSGILK.V | 2 | 1990.313 | 3.131 | 0.376 |

+1 charge

```
		      B   	       Y
  1	 H 	   138.07	        -  	 17
  2	 F 	   285.13	  1851.93	 16
  3	 F 	   432.20	  1704.87	 15
  4	 S 	   519.24	  1557.80	 14
  5	 D 	   634.26	  1470.77	 13
  6	 Y 	   797.33	  1355.74	 12
  7	 L 	   910.41	  1192.68	 11
  8	 M 	  1041.45	  1079.59	 10
  9	 G 	  1098.47	   948.55	  9
 10	 F 	  1245.54	   891.53	  8
 11	 I 	  1358.62	   744.46	  7
 12	 N 	  1472.67	   631.38	  6
 13	 S 	  1559.70	   517.33	  5
 14	 G 	  1616.72	   430.30	  4
 15	 I 	  1729.80	   373.28	  3
 16	 L 	  1842.89	   260.20	  2
 17	 K 	        -  	   147.11	  1  _
+2 charge
		      B   	       Y
  1	 H 	    69.54	        -  	 17
  2	 F 	   143.07	   926.47	 16
  3	 F 	   216.61	   852.94	 15
  4	 S 	   260.12	   779.40	 14
  5	 D 	   317.63	   735.89	 13
  6	 Y 	   399.17	   678.37	 12
  7	 L 	   455.71	   596.84	 11
  8	 M 	   521.23	   540.30	 10
  9	 G 	   549.74	   474.78	  9
 10	 F 	   623.27	   446.27	  8
 11	 I 	   679.82	   372.73	  7
 12	 N 	   736.84	   316.19	  6
 13	 S 	   780.35	   259.17	  5
 14	 G 	   808.86	   215.65	  4
 15	 I 	   865.41	   187.14	  3
 16	 L 	   921.95	   130.60	  2
 17	 K 	        -  	    74.06	  1  _
```

**94．IPI00385252 Ig kappa chain V-III region GOL**

| Sequence | Charge | M+H+ | XCorr | DeltaCn |
| --- | --- | --- | --- | --- |
| - .EIVLTQSPGTLSLSPGER.A | 2 | 1885.108 | 5.669 | 0.524 |

+1 charge

```
		      B   	       Y
  1	 E 	   130.05	        -  	 18
  2	 I 	   243.13	  1754.96	 17
  3	 V 	   342.20	  1641.88	 16
  4	 L 	   455.29	  1542.81	 15
  5	 T 	   556.33	  1429.73	 14
  6	 Q 	   684.39	  1328.68	 13
  7	 S 	   771.42	  1200.62	 12
  8	 P 	   868.48	  1113.59	 11
  9	 G 	   925.50	  1016.54	 10
 10	 T 	  1026.55	   959.52	  9
 11	 L 	  1139.63	   858.47	  8
 12	 S 	  1226.66	   745.38	  7
 13	 L 	  1339.75	   658.35	  6
 14	 S 	  1426.78	   545.27	  5
 15	 P 	  1523.83	   458.24	  4
 16	 G 	  1580.85	   361.18	  3
 17	 E 	  1709.90	   304.16	  2
 18	 R 	        -  	   175.12	  1  _
+2 charge
		      B   	       Y
  1	 E 	    65.53	        -  	 18
  2	 I 	   122.07	   877.99	 17
  3	 V 	   171.60	   821.44	 16
  4	 L 	   228.15	   771.91	 15
  5	 T 	   278.67	   715.37	 14
  6	 Q 	   342.70	   664.84	 13
  7	 S 	   386.22	   600.81	 12
  8	 P 	   434.74	   557.30	 11
  9	 G 	   463.25	   508.77	 10
 10	 T 	   513.78	   480.26	  9
 11	 L 	   570.32	   429.74	  8
 12	 S 	   613.83	   373.20	  7
 13	 L 	   670.38	   329.68	  6
 14	 S 	   713.89	   273.14	  5
 15	 P 	   762.42	   229.62	  4
 16	 G 	   790.93	   181.10	  3
 17	 E 	   855.45	   152.58	  2
 18	 R 	        -  	    88.06	  1  _
```

**95. IPI00004372 MEP1A protein**

| Sequence | Charge | M+H+ | XCorr | DeltaCn |
| --- | --- | --- | --- | --- |
| K.AMLEEALPVSLSQGQPSR.Q | 2 | 1914.173 | 3.511 | 0.396 |

+1 charge

```
		      B   	       Y
  1	 A 	    72.04	        -  	 18
  2	 M 	   203.08	  1841.94	 17
  3	 L 	   316.17	  1710.90	 16
  4	 E 	   445.21	  1597.82	 15
  5	 E 	   574.25	  1468.78	 14
  6	 A 	   645.29	  1339.73	 13
  7	 L 	   758.38	  1268.70	 12
  8	 P 	   855.43	  1155.61	 11
  9	 V 	   954.50	  1058.56	 10
 10	 S 	  1041.53	   959.49	  9
 11	 L 	  1154.61	   872.46	  8
 12	 S 	  1241.64	   759.37	  7
 13	 Q 	  1369.70	   672.34	  6
 14	 G 	  1426.72	   544.28	  5
 15	 Q 	  1554.78	   487.26	  4
 16	 P 	  1651.84	   359.20	  3
 17	 S 	  1738.87	   262.15	  2
 18	 R 	        -  	   175.12	  1  _
+2 charge
		      B   	       Y
  1	 A 	    36.53	        -  	 18
  2	 M 	   102.05	   921.47	 17
  3	 L 	   158.59	   855.95	 16
  4	 E 	   223.11	   799.41	 15
  5	 E 	   287.63	   734.89	 14
  6	 A 	   323.15	   670.37	 13
  7	 L 	   379.69	   634.85	 12
  8	 P 	   428.22	   578.31	 11
  9	 V 	   477.75	   529.78	 10
 10	 S 	   521.27	   480.25	  9
 11	 L 	   577.81	   436.73	  8
 12	 S 	   621.33	   380.19	  7
 13	 Q 	   685.36	   336.67	  6
 14	 G 	   713.87	   272.65	  5
 15	 Q 	   777.90	   244.13	  4
 16	 P 	   826.42	   180.11	  3
 17	 S 	   869.94	   131.58	  2
 18	 R 	        -  	    88.06	  1  _
```

**96. IPI0038712 Ig kappa chain V-IV region Len**

| Sequence | Charge | M+H+ | XCorr | DeltaCn |
| --- | --- | --- | --- | --- |
| - .DIVMTQSPDSLAVSLGER.A | 2 | 1919.147 | 5.881 | 0.528 |

+1 charge

```
		      B   	       Y
  1	 D 	   116.03	        -  	 18
  2	 I 	   229.12	  1802.93	 17
  3	 V 	   328.19	  1689.85	 16
  4	 M 	   459.23	  1590.78	 15
  5	 T 	   560.27	  1459.74	 14
  6	 Q 	   688.33	  1358.69	 13
  7	 S 	   775.37	  1230.63	 12
  8	 P 	   872.42	  1143.60	 11
  9	 D 	   987.45	  1046.55	 10
 10	 S 	  1074.48	   931.52	  9
 11	 L 	  1187.56	   844.49	  8
 12	 A 	  1258.60	   731.40	  7
 13	 V 	  1357.67	   660.37	  6
 14	 S 	  1444.70	   561.30	  5
 15	 L 	  1557.78	   474.27	  4
 16	 G 	  1614.80	   361.18	  3
 17	 E 	  1743.85	   304.16	  2
 18	 R 	        -  	   175.12	  1  _
+2 charge
		      B   	       Y
  1	 D 	    58.52	        -  	 18
  2	 I 	   115.06	   901.97	 17
  3	 V 	   164.60	   845.43	 16
  4	 M 	   230.12	   795.89	 15
  5	 T 	   280.64	   730.37	 14
  6	 Q 	   344.67	   679.85	 13
  7	 S 	   388.19	   615.82	 12
  8	 P 	   436.71	   572.30	 11
  9	 D 	   494.23	   523.78	 10
 10	 S 	   537.74	   466.26	  9
 11	 L 	   594.28	   422.75	  8
 12	 A 	   629.80	   366.21	  7
 13	 V 	   679.34	   330.69	  6
 14	 S 	   722.85	   281.15	  5
 15	 L 	   779.40	   237.64	  4
 16	 G 	   807.91	   181.10	  3
 17	 E 	   872.43	   152.58	  2
 18	 R 	        -  	    88.06	  1  _
```

**97. IPI00418169 Isoform 2 of Annexin A2**

| Sequence | Charge | M+H+ | XCorr | DeltaCn |
| --- | --- | --- | --- | --- |
| K.SALSGHLETVILGLLK.T | 2 | 1651.972 | 5.331 | 0.648 |

+1 charge

```
		      B   	       Y
  1	 S 	    88.04	        -  	 16
  2	 A 	   159.08	  1563.95	 15
  3	 L 	   272.16	  1492.91	 14
  4	 S 	   359.19	  1379.83	 13
  5	 G 	   416.21	  1292.79	 12
  6	 H 	   553.27	  1235.77	 11
  7	 L 	   666.36	  1098.71	 10
  8	 E 	   795.40	   985.63	  9
  9	 T 	   896.45	   856.59	  8
 10	 V 	   995.52	   755.54	  7
 11	 I 	  1108.60	   656.47	  6
 12	 L 	  1221.68	   543.39	  5
 13	 G 	  1278.71	   430.30	  4
 14	 L 	  1391.79	   373.28	  3
 15	 L 	  1504.87	   260.20	  2
 16	 K 	        -  	   147.11	  1  _
+2 charge
		      B   	       Y
  1	 S 	    44.52	        -  	 16
  2	 A 	    80.04	   782.48	 15
  3	 L 	   136.58	   746.96	 14
  4	 S 	   180.10	   690.42	 13
  5	 G 	   208.61	   646.90	 12
  6	 H 	   277.14	   618.39	 11
  7	 L 	   333.68	   549.86	 10
  8	 E 	   398.20	   493.32	  9
  9	 T 	   448.73	   428.80	  8
 10	 V 	   498.26	   378.27	  7
 11	 I 	   554.80	   328.74	  6
 12	 L 	   611.35	   272.20	  5
 13	 G 	   639.86	   215.65	  4
 14	 L 	   696.40	   187.14	  3
 15	 L 	   752.94	   130.60	  2
 16	 K 	        -  	    74.06	  1  _
```

**98. IPI00021263 14-3-3 protein zeta/delta**

| Sequence | Charge | M+H+ | XCorr | DeltaCn |
| --- | --- | --- | --- | --- |
| K.TAFDEAIAELDTLSEESYK.D | 2 | 2133.252 | 3.708 | 0.315 |

+1 charge

```
		      B   	       Y
  1	 T 	   102.05	        -  	 19
  2	 A 	   173.09	  2030.94	 18
  3	 F 	   320.16	  1959.91	 17
  4	 D 	   435.19	  1812.84	 16
  5	 E 	   564.23	  1697.81	 15
  6	 A 	   635.27	  1568.77	 14
  7	 I 	   748.35	  1497.73	 13
  8	 A 	   819.39	  1384.65	 12
  9	 E 	   948.43	  1313.61	 11
 10	 L 	  1061.51	  1184.57	 10
 11	 D 	  1176.54	  1071.48	  9
 12	 T 	  1277.59	   956.46	  8
 13	 L 	  1390.67	   855.41	  7
 14	 S 	  1477.71	   742.33	  6
 15	 E 	  1606.75	   655.29	  5
 16	 E 	  1735.79	   526.25	  4
 17	 S 	  1822.82	   397.21	  3
 18	 Y 	  1985.89	   310.18	  2
 19	 K 	        -  	   147.11	  1  _
+2 charge
		      B   	       Y
  1	 T 	    51.53	        -  	 19
  2	 A 	    87.05	  1015.98	 18
  3	 F 	   160.58	   980.46	 17
  4	 D 	   218.10	   906.92	 16
  5	 E 	   282.62	   849.41	 15
  6	 A 	   318.14	   784.89	 14
  7	 I 	   374.68	   749.37	 13
  8	 A 	   410.20	   692.83	 12
  9	 E 	   474.72	   657.31	 11
 10	 L 	   531.26	   592.79	 10
 11	 D 	   588.77	   536.25	  9
 12	 T 	   639.30	   478.73	  8
 13	 L 	   695.84	   428.21	  7
 14	 S 	   739.36	   371.67	  6
 15	 E 	   803.88	   328.15	  5
 16	 E 	   868.40	   263.63	  4
 17	 S 	   911.92	   199.11	  3
 18	 Y 	   993.45	   155.59	  2
 19	 K 	        -  	    74.06	  1  _
```

**99. IPI00169087 Olfactory receptor 1P1**

| Sequence | Charge | M+H+ | XCorr | DeltaCn |
| --- | --- | --- | --- | --- |
| K.EDTAAAVMYTVVTPLLNPFIYSMR.N | 3 | 2704.158 | 3.459 | 0.22 |

+1 charge

```
		      B   	       Y
  1	 E 	   130.05	        -  	 24
  2	 D 	   245.08	  2573.31	 23
  3	 T 	   346.12	  2458.29	 22
  4	 A 	   417.16	  2357.24	 21
  5	 A 	   488.20	  2286.20	 20
  6	 A 	   559.24	  2215.17	 19
  7	 V 	   658.30	  2144.13	 18
  8	 M 	   789.34	  2045.06	 17
  9	 Y 	   952.41	  1914.02	 16
 10	 T 	  1053.46	  1750.96	 15
 11	 V 	  1152.52	  1649.91	 14
 12	 V 	  1251.59	  1550.84	 13
 13	 T 	  1352.64	  1451.77	 12
 14	 P 	  1449.69	  1350.72	 11
 15	 L 	  1562.78	  1253.67	 10
 16	 L 	  1675.86	  1140.59	  9
 17	 N 	  1789.90	  1027.50	  8
 18	 P 	  1886.96	   913.46	  7
 19	 F 	  2034.03	   816.41	  6
 20	 I 	  2147.11	   669.34	  5
 21	 Y 	  2310.17	   556.25	  4
 22	 S 	  2397.20	   393.19	  3
 23	 M 	  2528.25	   306.16	  2
 24	 R 	        -  	   175.12	  1  _


+2 charge
		      B   	       Y
  1	 E 	    65.53	        -  	 24
  2	 D 	   123.04	  1287.16	 23
  3	 T 	   173.57	  1229.65	 22
  4	 A 	   209.08	  1179.12	 21
  5	 A 	   244.60	  1143.60	 20
  6	 A 	   280.12	  1108.09	 19
  7	 V 	   329.66	  1072.57	 18
  8	 M 	   395.18	  1023.03	 17
  9	 Y 	   476.71	   957.51	 16
 10	 T 	   527.23	   875.98	 15
 11	 V 	   576.77	   825.46	 14
 12	 V 	   626.30	   775.92	 13
 13	 T 	   676.82	   726.39	 12
 14	 P 	   725.35	   675.87	 11
 15	 L 	   781.89	   627.34	 10
 16	 L 	   838.43	   570.80	  9
 17	 N 	   895.46	   514.26	  8
 18	 P 	   943.98	   457.23	  7
 19	 F 	  1017.52	   408.71	  6
 20	 I 	  1074.06	   335.17	  5
 21	 Y 	  1155.59	   278.63	  4
 22	 S 	  1199.11	   197.10	  3
 23	 M 	  1264.63	   153.58	  2
 24	 R 	        -  	    88.06	  1  _


+3 charge
		      B   	       Y
  1	 E 	    44.02	        -  	 24
  2	 D 	    82.36	   858.44	 23
  3	 T 	   116.05	   820.10	 22
  4	 A 	   139.73	   786.42	 21
  5	 A 	   163.40	   762.74	 20
  6	 A 	   187.08	   739.06	 19
  7	 V 	   220.11	   715.38	 18
  8	 M 	   263.79	   682.36	 17
  9	 Y 	   318.14	   638.68	 16
 10	 T 	   351.82	   584.32	 15
 11	 V 	   384.85	   550.64	 14
 12	 V 	   417.87	   517.62	 13
 13	 T 	   451.55	   484.60	 12
 14	 P 	   483.90	   450.91	 11
 15	 L 	   521.60	   418.56	 10
 16	 L 	   559.29	   380.87	  9
 17	 N 	   597.31	   343.17	  8
 18	 P 	   629.66	   305.16	  7
 19	 F 	   678.68	   272.81	  6
 20	 I 	   716.37	   223.78	  5
 21	 Y 	   770.73	   186.09	  4
 22	 S 	   799.74	   131.74	  3
 23	 M 	   843.42	   102.72	  2
 24	 R 	        -  	    59.04	  1  _
```

**100. IPI00153049 Isoform 2 of Matrix-remodeling-associated protein 8**

| Sequence | Charge | M+H+ | XCorr | DeltaCn |
| --- | --- | --- | --- | --- |
| R.LLDLYSAGEQR.V | 2 | 1265.397 | 3.28 | 0.384 |

+1 charge

```
		      B   	       Y
  1	 L 	   114.09	        -  	 11
  2	 L 	   227.18	  1151.57	 10
  3	 D 	   342.20	  1038.49	  9
  4	 L 	   455.29	   923.46	  8
  5	 Y 	   618.35	   810.37	  7
  6	 S 	   705.38	   647.31	  6
  7	 A 	   776.42	   560.28	  5
  8	 G 	   833.44	   489.24	  4
  9	 E 	   962.48	   432.22	  3
 10	 Q 	  1090.54	   303.18	  2
 11	 R 	        -  	   175.12	  1  _
+2 charge
		      B   	       Y
  1	 L 	    57.55	        -  	 11
  2	 L 	   114.09	   576.29	 10
  3	 D 	   171.60	   519.75	  9
  4	 L 	   228.15	   462.23	  8
  5	 Y 	   309.68	   405.69	  7
  6	 S 	   353.19	   324.16	  6
  7	 A 	   388.71	   280.64	  5
  8	 G 	   417.22	   245.12	  4
  9	 E 	   481.75	   216.61	  3
 10	 Q 	   545.77	   152.09	  2
 11	 R 	        -  	    88.06	  1  _
```

**101. IPI00023648 Immunoglobulin superfamily containing leucine-rich repeat protein**

| Sequence | Charge | M+H+ | XCorr | DeltaCn |
| --- | --- | --- | --- | --- |
| R.TVAAGALASLSHLK.S | 2 | 1339.566 | 4.025 | 0.526 |

+1 charge

```
		      B   	       Y
  1	 T 	   102.05	        -  	 14
  2	 V 	   201.12	  1237.73	 13
  3	 A 	   272.16	  1138.66	 12
  4	 A 	   343.20	  1067.62	 11
  5	 G 	   400.22	   996.58	 10
  6	 A 	   471.26	   939.56	  9
  7	 L 	   584.34	   868.53	  8
  8	 A 	   655.38	   755.44	  7
  9	 S 	   742.41	   684.40	  6
 10	 L 	   855.49	   597.37	  5
 11	 S 	   942.53	   484.29	  4
 12	 H 	  1079.58	   397.26	  3
 13	 L 	  1192.67	   260.20	  2
 14	 K 	        -  	   147.11	  1  _
+2 charge
		      B   	       Y
  1	 T 	    51.53	        -  	 14
  2	 V 	   101.07	   619.37	 13
  3	 A 	   136.58	   569.83	 12
  4	 A 	   172.10	   534.31	 11
  5	 G 	   200.61	   498.80	 10
  6	 A 	   236.13	   470.28	  9
  7	 L 	   292.67	   434.77	  8
  8	 A 	   328.19	   378.22	  7
  9	 S 	   371.71	   342.71	  6
 10	 L 	   428.25	   299.19	  5
 11	 S 	   471.77	   242.65	  4
 12	 H 	   540.30	   199.13	  3
 13	 L 	   596.84	   130.60	  2
 14	 K 	        -  	    74.06	  1  _
```

**102. IPI00022661 Isoform Delta of Poliovirus receptor-related protein 2**

| Sequence | Charge | M+H+ | XCorr | DeltaCn |
| --- | --- | --- | --- | --- |
| K.VTFSQDPTTVALCISK.E | 2 | 1767.995 | 3.066 | 0.369 |

+1 charge

```
		      B   	       Y
  1	 V 	   100.08	        -  	 16
  2	 T 	   201.12	  1667.97	 15
  3	 F 	   348.19	  1566.92	 14
  4	 S 	   435.22	  1419.85	 13
  5	 Q 	   563.28	  1332.82	 12
  6	 D 	   678.31	  1204.76	 11
  7	 P 	   775.36	  1089.73	 10
  8	 T 	   876.41	   992.68	  9
  9	 T 	   977.46	   891.63	  8
 10	 V 	  1076.53	   790.58	  7
 11	 A 	  1147.56	   691.52	  6
 12	 L 	  1260.65	   620.48	  5
 13	 C 	  1420.81	   507.39	  4
 14	 I 	  1533.90	   347.23	  3
 15	 S 	  1620.93	   234.14	  2
 16	 K 	        -  	   147.11	  1  _
+2 charge
		      B   	       Y
  1	 V 	    50.54	        -  	 16
  2	 T 	   101.07	   834.49	 15
  3	 F 	   174.60	   783.96	 14
  4	 S 	   218.12	   710.43	 13
  5	 Q 	   282.14	   666.91	 12
  6	 D 	   339.66	   602.88	 11
  7	 P 	   388.18	   545.37	 10
  8	 T 	   438.71	   496.84	  9
  9	 T 	   489.23	   446.32	  8
 10	 V 	   538.77	   395.80	  7
 11	 A 	   574.29	   346.26	  6
 12	 L 	   630.83	   310.74	  5
 13	 C 	   710.91	   254.20	  4
 14	 I 	   767.45	   174.12	  3
 15	 S 	   810.97	   117.58	  2
 16	 K 	        -  	    74.06	  1  _
```

**103. IPI00002441 Syndecan-1**

| Sequence | Charge | M+H+ | XCorr | DeltaCn |
| --- | --- | --- | --- | --- |
| K.EGEAVVLPEVEPGLTAR.E | 2 | 1766.974 | 4.019 | 0.464 |

+1 charge

```
		      B   	       Y
  1	 E 	   130.05	        -  	 17
  2	 G 	   187.07	  1636.89	 16
  3	 E 	   316.11	  1579.87	 15
  4	 A 	   387.15	  1450.83	 14
  5	 V 	   486.22	  1379.79	 13
  6	 V 	   585.29	  1280.72	 12
  7	 L 	   698.37	  1181.65	 11
  8	 P 	   795.42	  1068.57	 10
  9	 E 	   924.47	   971.52	  9
 10	 V 	  1023.54	   842.47	  8
 11	 E 	  1152.58	   743.40	  7
 12	 P 	  1249.63	   614.36	  6
 13	 G 	  1306.65	   517.31	  5
 14	 L 	  1419.74	   460.29	  4
 15	 T 	  1520.78	   347.20	  3
 16	 A 	  1591.82	   246.16	  2
 17	 R 	        -  	   175.12	  1  _
+2 charge
		      B   	       Y
  1	 E 	    65.53	        -  	 17
  2	 G 	    94.04	   818.95	 16
  3	 E 	   158.56	   790.44	 15
  4	 A 	   194.08	   725.92	 14
  5	 V 	   243.61	   690.40	 13
  6	 V 	   293.15	   640.86	 12
  7	 L 	   349.69	   591.33	 11
  8	 P 	   398.22	   534.79	 10
  9	 E 	   462.74	   486.26	  9
 10	 V 	   512.27	   421.74	  8
 11	 E 	   576.79	   372.21	  7
 12	 P 	   625.32	   307.68	  6
 13	 G 	   653.83	   259.16	  5
 14	 L 	   710.37	   230.65	  4
 15	 T 	   760.90	   174.11	  3
 16	 A 	   796.41	   123.58	  2
 17	 R 	        -  	    88.06	  1  _
```

**104. IPI00247063 Neprilysin**

| Sequence | Charge | M+H+ | XCorr | DeltaCn |
| --- | --- | --- | --- | --- |
| R.LIQNMDATTEPCTDFFK.Y | 2 | 2032.254 | 3.154 | 0.44 |

+1 charge

```
		      B   	       Y
  1	 L 	   114.09	        -  	 17
  2	 I 	   227.18	  1917.97	 16
  3	 Q 	   355.23	  1804.89	 15
  4	 N 	   469.28	  1676.83	 14
  5	 M 	   600.32	  1562.78	 13
  6	 D 	   715.34	  1431.74	 12
  7	 A 	   786.38	  1316.72	 11
  8	 T 	   887.43	  1245.68	 10
  9	 T 	   988.48	  1144.63	  9
 10	 E 	  1117.52	  1043.58	  8
 11	 P 	  1214.57	   914.54	  7
 12	 C 	  1374.74	   817.49	  6
 13	 T 	  1475.79	   657.32	  5
 14	 D 	  1590.81	   556.28	  4
 15	 F 	  1737.88	   441.25	  3
 16	 F 	  1884.95	   294.18	  2
 17	 K 	        -  	   147.11	  1  _
+2 charge
		      B   	       Y
  1	 L 	    57.55	        -  	 17
  2	 I 	   114.09	   959.49	 16
  3	 Q 	   178.12	   902.95	 15
  4	 N 	   235.14	   838.92	 14
  5	 M 	   300.66	   781.90	 13
  6	 D 	   358.18	   716.38	 12
  7	 A 	   393.69	   658.86	 11
  8	 T 	   444.22	   623.34	 10
  9	 T 	   494.74	   572.82	  9
 10	 E 	   559.26	   522.30	  8
 11	 P 	   607.79	   457.77	  7
 12	 C 	   687.87	   409.25	  6
 13	 T 	   738.40	   329.17	  5
 14	 D 	   795.91	   278.64	  4
 15	 F 	   869.44	   221.13	  3
 16	 F 	   942.98	   147.59	  2
 17	 K 	        -  	    74.06	  1  _
```

**105. IPI00012540 Prominin-1**

| Sequence | Charge | M+H+ | XCorr | DeltaCn |
| --- | --- | --- | --- | --- |
| K.AFTDLNSINSVLGGGILDR.L | 2 | 1963.181 | 3.573 | 0.389 |

+1 charge

```
		      B   	       Y
  1	 A 	    72.04	        -  	 19
  2	 F 	   219.11	  1890.99	 18
  3	 T 	   320.16	  1743.92	 17
  4	 D 	   435.19	  1642.88	 16
  5	 L 	   548.27	  1527.85	 15
  6	 N 	   662.31	  1414.76	 14
  7	 S 	   749.35	  1300.72	 13
  8	 I 	   862.43	  1213.69	 12
  9	 N 	   976.47	  1100.61	 11
 10	 S 	  1063.51	   986.56	 10
 11	 V 	  1162.57	   899.53	  9
 12	 L 	  1275.66	   800.46	  8
 13	 G 	  1332.68	   687.38	  7
 14	 G 	  1389.70	   630.36	  6
 15	 G 	  1446.72	   573.34	  5
 16	 I 	  1559.81	   516.31	  4
 17	 L 	  1672.89	   403.23	  3
 18	 D 	  1787.92	   290.15	  2
 19	 R 	        -  	   175.12	  1  _
+2 charge
		      B   	       Y
  1	 A 	    36.53	        -  	 19
  2	 F 	   110.06	   946.00	 18
  3	 T 	   160.58	   872.47	 17
  4	 D 	   218.10	   821.94	 16
  5	 L 	   274.64	   764.43	 15
  6	 N 	   331.66	   707.89	 14
  7	 S 	   375.18	   650.86	 13
  8	 I 	   431.72	   607.35	 12
  9	 N 	   488.74	   550.81	 11
 10	 S 	   532.26	   493.79	 10
 11	 V 	   581.79	   450.27	  9
 12	 L 	   638.33	   400.73	  8
 13	 G 	   666.84	   344.19	  7
 14	 G 	   695.35	   315.68	  6
 15	 G 	   723.86	   287.17	  5
 16	 I 	   780.41	   258.66	  4
 17	 L 	   836.95	   202.12	  3
 18	 D 	   894.46	   145.58	  2
 19	 R 	        -  	    88.06	  1  _
```

**106. IPI00940046 cDNA FLJ40980 fis, clone UTERU2014464, highly similar to ACID CERAMIDASE**

| Sequence | Charge | M+H+ | XCorr | DeltaCn |
| --- | --- | --- | --- | --- |
| K.VMQVVDEKLPGLLGNFPGPFEEEMK.G | 3 | 2805.262 | 4.051 | 0.447 |

+1 charge

```
		      B   	       Y
  1	 V 	   100.08	        -  	 25
  2	 M 	   231.12	  2704.34	 24
  3	 Q 	   359.17	  2573.30	 23
  4	 V 	   458.24	  2445.24	 22
  5	 V 	   557.31	  2346.17	 21
  6	 D 	   672.34	  2247.10	 20
  7	 E 	   801.38	  2132.07	 19
  8	 K 	   929.48	  2003.03	 18
  9	 L 	  1042.56	  1874.94	 17
 10	 P 	  1139.61	  1761.85	 16
 11	 G 	  1196.63	  1664.80	 15
 12	 L 	  1309.72	  1607.78	 14
 13	 L 	  1422.80	  1494.69	 13
 14	 G 	  1479.82	  1381.61	 12
 15	 N 	  1593.87	  1324.59	 11
 16	 F 	  1740.94	  1210.54	 10
 17	 P 	  1837.99	  1063.48	  9
 18	 G 	  1895.01	   966.42	  8
 19	 P 	  1992.06	   909.40	  7
 20	 F 	  2139.13	   812.35	  6
 21	 E 	  2268.17	   665.28	  5
 22	 E 	  2397.22	   536.24	  4
 23	 E 	  2526.26	   407.20	  3
 24	 M 	  2657.30	   278.15	  2
 25	 K 	        -  	   147.11	  1  _


+2 charge
		      B   	       Y
  1	 V 	    50.54	        -  	 25
  2	 M 	   116.06	  1352.67	 24
  3	 Q 	   180.09	  1287.15	 23
  4	 V 	   229.63	  1223.12	 22
  5	 V 	   279.16	  1173.59	 21
  6	 D 	   336.67	  1124.05	 20
  7	 E 	   401.19	  1066.54	 19
  8	 K 	   465.24	  1002.02	 18
  9	 L 	   521.78	   937.97	 17
 10	 P 	   570.31	   881.43	 16
 11	 G 	   598.82	   832.90	 15
 12	 L 	   655.36	   804.39	 14
 13	 L 	   711.90	   747.85	 13
 14	 G 	   740.42	   691.31	 12
 15	 N 	   797.44	   662.80	 11
 16	 F 	   870.97	   605.78	 10
 17	 P 	   919.50	   532.24	  9
 18	 G 	   948.01	   483.72	  8
 19	 P 	   996.53	   455.20	  7
 20	 F 	  1070.07	   406.68	  6
 21	 E 	  1134.59	   333.14	  5
 22	 E 	  1199.11	   268.62	  4
 23	 E 	  1263.63	   204.10	  3
 24	 M 	  1329.15	   139.58	  2
 25	 K 	        -  	    74.06	  1  _


+3 charge
		      B   	       Y
  1	 V 	    34.03	        -  	 25
  2	 M 	    77.71	   902.12	 24
  3	 Q 	   120.40	   858.44	 23
  4	 V 	   153.42	   815.75	 22
  5	 V 	   186.44	   782.73	 21
  6	 D 	   224.78	   749.70	 20
  7	 E 	   267.80	   711.36	 19
  8	 K 	   310.50	   668.35	 18
  9	 L 	   348.19	   625.65	 17
 10	 P 	   380.54	   587.96	 16
 11	 G 	   399.55	   555.60	 15
 12	 L 	   437.24	   536.60	 14
 13	 L 	   474.94	   498.90	 13
 14	 G 	   493.95	   461.21	 12
 15	 N 	   531.96	   442.20	 11
 16	 F 	   580.98	   404.19	 10
 17	 P 	   613.33	   355.16	  9
 18	 G 	   632.34	   322.81	  8
 19	 P 	   664.69	   303.81	  7
 20	 F 	   713.72	   271.45	  6
 21	 E 	   756.73	   222.43	  5
 22	 E 	   799.74	   179.42	  4
 23	 E 	   842.76	   136.40	  3
 24	 M 	   886.44	    93.39	  2
 25	 K 	        -  	    49.71	  1  _
```

**107. IPI00001872 Isoform 1 of Protocadherin gamma-C3**

| Sequence | Charge | M+H+ | XCorr | DeltaCn |
| --- | --- | --- | --- | --- |
| K.GFAVGNVVANLGLDLGSLSAR.R | 2 | 2031.302 | 3.085 | 0.267 |

+1 charge

```
		      B   	       Y
  1	 G 	    58.03	        -  	 21
  2	 F 	   205.10	  1973.08	 20
  3	 A 	   276.13	  1826.01	 19
  4	 V 	   375.20	  1754.98	 18
  5	 G 	   432.22	  1655.91	 17
  6	 N 	   546.27	  1598.89	 16
  7	 V 	   645.34	  1484.84	 15
  8	 V 	   744.40	  1385.77	 14
  9	 A 	   815.44	  1286.71	 13
 10	 N 	   929.48	  1215.67	 12
 11	 L 	  1042.57	  1101.63	 11
 12	 G 	  1099.59	   988.54	 10
 13	 L 	  1212.67	   931.52	  9
 14	 D 	  1327.70	   818.44	  8
 15	 L 	  1440.78	   703.41	  7
 16	 G 	  1497.81	   590.33	  6
 17	 S 	  1584.84	   533.30	  5
 18	 L 	  1697.92	   446.27	  4
 19	 S 	  1784.95	   333.19	  3
 20	 A 	  1855.99	   246.16	  2
 21	 R 	        -  	   175.12	  1  _


+2 charge
		      B   	       Y
  1	 G 	    29.52	        -  	 21
  2	 F 	   103.05	   987.04	 20
  3	 A 	   138.57	   913.51	 19
  4	 V 	   188.10	   877.99	 18
  5	 G 	   216.62	   828.46	 17
  6	 N 	   273.64	   799.95	 16
  7	 V 	   323.17	   742.93	 15
  8	 V 	   372.71	   693.39	 14
  9	 A 	   408.22	   643.86	 13
 10	 N 	   465.25	   608.34	 12
 11	 L 	   521.79	   551.32	 11
 12	 G 	   550.30	   494.77	 10
 13	 L 	   606.84	   466.26	  9
 14	 D 	   664.35	   409.72	  8
 15	 L 	   720.90	   352.21	  7
 16	 G 	   749.41	   295.67	  6
 17	 S 	   792.92	   267.16	  5
 18	 L 	   849.46	   223.64	  4
 19	 S 	   892.98	   167.10	  3
 20	 A 	   928.50	   123.58	  2
 21	 R 	        -  	    88.06	  1  _
```

**108. IPI00013895 Protein S100-A11**

| Sequence | Charge | M+H+ | XCorr | DeltaCn |
| --- | --- | --- | --- | --- |
| K.TEFLSFMNTELAAFTK.N | 2 | 1851.112 | 3.676 | 0.62 |

```
+1 charge
		      B   	       Y
  1	 T 	   102.05	        -  	 16
  2	 E 	   231.10	  1748.86	 15
  3	 F 	   378.17	  1619.81	 14
  4	 L 	   491.25	  1472.75	 13
  5	 S 	   578.28	  1359.66	 12
  6	 F 	   725.35	  1272.63	 11
  7	 M 	   856.39	  1125.56	 10
  8	 N 	   970.43	   994.52	  9
  9	 T 	  1071.48	   880.48	  8
 10	 E 	  1200.52	   779.43	  7
 11	 L 	  1313.61	   650.39	  6
 12	 A 	  1384.65	   537.30	  5
 13	 A 	  1455.68	   466.27	  4
 14	 F 	  1602.75	   395.23	  3
 15	 T 	  1703.80	   248.16	  2
 16	 K 	        -  	   147.11	  1  _
+2 charge
		      B   	       Y
  1	 T 	    51.53	        -  	 16
  2	 E 	   116.05	   874.93	 15
  3	 F 	   189.59	   810.41	 14
  4	 L 	   246.13	   736.88	 13
  5	 S 	   289.64	   680.33	 12
  6	 F 	   363.18	   636.82	 11
  7	 M 	   428.70	   563.28	 10
  8	 N 	   485.72	   497.76	  9
  9	 T 	   536.24	   440.74	  8
 10	 E 	   600.77	   390.22	  7
 11	 L 	   657.31	   325.70	  6
 12	 A 	   692.83	   269.16	  5
 13	 A 	   728.34	   233.64	  4
 14	 F 	   801.88	   198.12	  3
 15	 T 	   852.40	   124.58	  2
 16	 K 	        -  	    74.06	  1  _
```

**109. IPI00382488 Ig heavy chain V-III region HIL**

| Sequence | Charge | M+H+ | XCorr | DeltaCn |
| --- | --- | --- | --- | --- |
| - .QVKLVQAGGGVVQPGR.S | 2 | 1593.856 | 2.83 | 0.376 |

+1 charge

```
		      B   	       Y
  1	 Q 	   129.07	        -  	 16
  2	 V 	   228.13	  1464.86	 15
  3	 K 	   356.23	  1365.80	 14
  4	 L 	   469.31	  1237.70	 13
  5	 V 	   568.38	  1124.62	 12
  6	 Q 	   696.44	  1025.55	 11
  7	 A 	   767.48	   897.49	 10
  8	 G 	   824.50	   826.45	  9
  9	 G 	   881.52	   769.43	  8
 10	 G 	   938.54	   712.41	  7
 11	 V 	  1037.61	   655.39	  6
 12	 V 	  1136.68	   556.32	  5
 13	 Q 	  1264.74	   457.25	  4
 14	 P 	  1361.79	   329.19	  3
 15	 G 	  1418.81	   232.14	  2
 16	 R 	        -  	   175.12	  1  _
+2 charge
		      B   	       Y
  1	 Q 	    65.04	        -  	 16
  2	 V 	   114.57	   732.94	 15
  3	 K 	   178.62	   683.40	 14
  4	 L 	   235.16	   619.35	 13
  5	 V 	   284.69	   562.81	 12
  6	 Q 	   348.72	   513.28	 11
  7	 A 	   384.24	   449.25	 10
  8	 G 	   412.75	   413.73	  9
  9	 G 	   441.26	   385.22	  8
 10	 G 	   469.77	   356.71	  7
 11	 V 	   519.31	   328.20	  6
 12	 V 	   568.84	   278.66	  5
 13	 Q 	   632.87	   229.13	  4
 14	 P 	   681.40	   165.10	  3
 15	 G 	   709.91	   116.57	  2
 16	 R 	        -  	    88.06	  1  _
```

**110. IPI00029168 Apolipoprotein(a)**

| Sequence | Charge | M+H+ | XCorr | DeltaCn |
| --- | --- | --- | --- | --- |
| R.TPEYYPNAGLIMNYCR.N | 2 | 1963.196 | 3.432 | 0.574 |

+1 charge

```
		      B   	       Y
  1	 T 	   102.05	        -  	 16
  2	 P 	   199.11	  1860.98	 15
  3	 E 	   328.15	  1763.92	 14
  4	 Y 	   491.21	  1634.88	 13
  5	 Y 	   654.28	  1471.82	 12
  6	 P 	   751.33	  1308.75	 11
  7	 N 	   865.37	  1211.70	 10
  8	 A 	   936.41	  1097.66	  9
  9	 G 	   993.43	  1026.62	  8
 10	 L 	  1106.52	   969.60	  7
 11	 I 	  1219.60	   856.51	  6
 12	 M 	  1350.64	   743.43	  5
 13	 N 	  1464.68	   612.39	  4
 14	 Y 	  1627.75	   498.35	  3
 15	 C 	  1787.91	   335.28	  2
 16	 R 	        -  	   175.12	  1  _
+2 charge
		      B   	       Y
  1	 T 	    51.53	        -  	 16
  2	 P 	   100.06	   930.99	 15
  3	 E 	   164.58	   882.46	 14
  4	 Y 	   246.11	   817.94	 13
  5	 Y 	   327.64	   736.41	 12
  6	 P 	   376.17	   654.88	 11
  7	 N 	   433.19	   606.35	 10
  8	 A 	   468.71	   549.33	  9
  9	 G 	   497.22	   513.81	  8
 10	 L 	   553.76	   485.30	  7
 11	 I 	   610.30	   428.76	  6
 12	 M 	   675.82	   372.22	  5
 13	 N 	   732.85	   306.70	  4
 14	 Y 	   814.38	   249.68	  3
 15	 C 	   894.46	   168.15	  2
 16	 R 	        -  	    88.06	  1  _
```

**111. IPI00176427 Cell adhesion molecule 4**

| Sequence | Charge | M+H+ | XCorr | DeltaCn |
| --- | --- | --- | --- | --- |
| R.EQAVEGGEVELSCLVPR.S | 2 | 1873.048 | 3.877 | 0.515 |

+1 charge

```
		      B   	       Y
  1	 E 	   130.05	        -  	 17
  2	 Q 	   258.11	  1743.01	 16
  3	 A 	   329.15	  1614.95	 15
  4	 V 	   428.21	  1543.91	 14
  5	 E 	   557.26	  1444.84	 13
  6	 G 	   614.28	  1315.80	 12
  7	 G 	   671.30	  1258.78	 11
  8	 E 	   800.34	  1201.76	 10
  9	 V 	   899.41	  1072.72	  9
 10	 E 	  1028.45	   973.65	  8
 11	 L 	  1141.54	   844.61	  7
 12	 S 	  1228.57	   731.52	  6
 13	 C 	  1388.73	   644.49	  5
 14	 L 	  1501.82	   484.32	  4
 15	 V 	  1600.89	   371.24	  3
 16	 P 	  1697.94	   272.17	  2
 17	 R 	        -  	   175.12	  1  _
+2 charge
		      B   	       Y
  1	 E 	    65.53	        -  	 17
  2	 Q 	   129.56	   872.01	 16
  3	 A 	   165.08	   807.98	 15
  4	 V 	   214.61	   772.46	 14
  5	 E 	   279.13	   722.93	 13
  6	 G 	   307.64	   658.40	 12
  7	 G 	   336.15	   629.89	 11
  8	 E 	   400.67	   601.38	 10
  9	 V 	   450.21	   536.86	  9
 10	 E 	   514.73	   487.33	  8
 11	 L 	   571.27	   422.81	  7
 12	 S 	   614.79	   366.26	  6
 13	 C 	   694.87	   322.75	  5
 14	 L 	   751.41	   242.67	  4
 15	 V 	   800.95	   186.12	  3
 16	 P 	   849.47	   136.59	  2
 17	 R 	        -  	    88.06	  1  _
```

**112. IPI00016007 Myosin-Vc**

| Sequence | Charge | M+H+ | XCorr | DeltaCn |
| --- | --- | --- | --- | --- |
| R.MSNTSFVIQHFADKVEYKCEGFLEK.N | 3 | 3009.375 | 3.727 | 0.45 |

+1 charge

```
		      B   	       Y
  1	 M 	   132.05	        -  	 25
  2	 S 	   219.08	  2876.53	 24
  3	 N 	   333.12	  2789.49	 23
  4	 T 	   434.17	  2675.45	 22
  5	 S 	   521.20	  2574.40	 21
  6	 F 	   668.27	  2487.37	 20
  7	 V 	   767.34	  2340.30	 19
  8	 I 	   880.42	  2241.24	 18
  9	 Q 	  1008.48	  2128.15	 17
 10	 H 	  1145.54	  2000.09	 16
 11	 F 	  1292.61	  1863.03	 15
 12	 A 	  1363.65	  1715.97	 14
 13	 D 	  1478.67	  1644.93	 13
 14	 K 	  1606.77	  1529.90	 12
 15	 V 	  1705.84	  1401.81	 11
 16	 E 	  1834.88	  1302.74	 10
 17	 Y 	  1997.94	  1173.70	  9
 18	 K 	  2126.04	  1010.63	  8
 19	 C 	  2286.20	   882.54	  7
 20	 E 	  2415.25	   722.37	  6
 21	 G 	  2472.27	   593.33	  5
 22	 F 	  2619.34	   536.31	  4
 23	 L 	  2732.42	   389.24	  3
 24	 E 	  2861.46	   276.16	  2
 25	 K 	        -  	   147.11	  1  _


+2 charge
		      B   	       Y
  1	 M 	    66.53	        -  	 25
  2	 S 	   110.04	  1438.77	 24
  3	 N 	   167.06	  1395.25	 23
  4	 T 	   217.59	  1338.23	 22
  5	 S 	   261.10	  1287.71	 21
  6	 F 	   334.64	  1244.19	 20
  7	 V 	   384.17	  1170.66	 19
  8	 I 	   440.72	  1121.12	 18
  9	 Q 	   504.74	  1064.58	 17
 10	 H 	   573.27	  1000.55	 16
 11	 F 	   646.81	   932.02	 15
 12	 A 	   682.33	   858.49	 14
 13	 D 	   739.84	   822.97	 13
 14	 K 	   803.89	   765.45	 12
 15	 V 	   853.42	   701.41	 11
 16	 E 	   917.94	   651.87	 10
 17	 Y 	   999.47	   587.35	  9
 18	 K 	  1063.52	   505.82	  8
 19	 C 	  1143.61	   441.77	  7
 20	 E 	  1208.13	   361.69	  6
 21	 G 	  1236.64	   297.17	  5
 22	 F 	  1310.17	   268.66	  4
 23	 L 	  1366.71	   195.12	  3
 24	 E 	  1431.23	   138.58	  2
 25	 K 	        -  	    74.06	  1  _


+3 charge
		      B   	       Y
  1	 M 	    44.69	        -  	 25
  2	 S 	    73.70	   959.51	 24
  3	 N 	   111.71	   930.50	 23
  4	 T 	   145.39	   892.49	 22
  5	 S 	   174.41	   858.81	 21
  6	 F 	   223.43	   829.80	 20
  7	 V 	   256.45	   780.77	 19
  8	 I 	   294.15	   747.75	 18
  9	 Q 	   336.83	   710.06	 17
 10	 H 	   382.52	   667.37	 16
 11	 F 	   431.54	   621.68	 15
 12	 A 	   455.22	   572.66	 14
 13	 D 	   493.56	   548.98	 13
 14	 K 	   536.26	   510.64	 12
 15	 V 	   569.28	   467.94	 11
 16	 E 	   612.30	   434.92	 10
 17	 Y 	   666.65	   391.90	  9
 18	 K 	   709.35	   337.55	  8
 19	 C 	   762.74	   294.85	  7
 20	 E 	   805.75	   241.46	  6
 21	 G 	   824.76	   198.45	  5
 22	 F 	   873.78	   179.44	  4
 23	 L 	   911.48	   130.42	  3
 24	 E 	   954.49	    92.72	  2
 25	 K 	        -  	    49.71	  1  _
```

**113. IPI00290315 Chromogranin-A**

| Sequence | Charge | M+H+ | XCorr | DeltaCn |
| --- | --- | --- | --- | --- |
| R.RPEDQELESLSAIEAELEK.V | 2 | 2187.347 | 5.824 | 0.559 |

+1 charge

```
		      B   	       Y
  1	 R 	   157.11	        -  	 19
  2	 P 	   254.16	  2029.98	 18
  3	 E 	   383.20	  1932.93	 17
  4	 D 	   498.23	  1803.89	 16
  5	 Q 	   626.29	  1688.86	 15
  6	 E 	   755.33	  1560.80	 14
  7	 L 	   868.42	  1431.76	 13
  8	 E 	   997.46	  1318.67	 12
  9	 S 	  1084.49	  1189.63	 11
 10	 L 	  1197.57	  1102.60	 10
 11	 S 	  1284.61	   989.51	  9
 12	 A 	  1355.64	   902.48	  8
 13	 I 	  1468.73	   831.45	  7
 14	 E 	  1597.77	   718.36	  6
 15	 A 	  1668.81	   589.32	  5
 16	 E 	  1797.85	   518.28	  4
 17	 L 	  1910.93	   389.24	  3
 18	 E 	  2039.98	   276.16	  2
 19	 K 	        -  	   147.11	  1  _
+2 charge
		      B   	       Y
  1	 R 	    79.06	        -  	 19
  2	 P 	   127.58	  1015.49	 18
  3	 E 	   192.11	   966.97	 17
  4	 D 	   249.62	   902.45	 16
  5	 Q 	   313.65	   844.93	 15
  6	 E 	   378.17	   780.90	 14
  7	 L 	   434.71	   716.38	 13
  8	 E 	   499.23	   659.84	 12
  9	 S 	   542.75	   595.32	 11
 10	 L 	   599.29	   551.80	 10
 11	 S 	   642.81	   495.26	  9
 12	 A 	   678.33	   451.75	  8
 13	 I 	   734.87	   416.23	  7
 14	 E 	   799.39	   359.68	  6
 15	 A 	   834.91	   295.16	  5
 16	 E 	   899.43	   259.64	  4
 17	 L 	   955.97	   195.12	  3
 18	 E 	  1020.49	   138.58	  2
 19	 K 	        -  	    74.06	  1  _
```

**114. IPI00220301 Peroxiredoxin-6**

| Sequence | Charge | M+H+ | XCorr | DeltaCn |
| --- | --- | --- | --- | --- |
| R.ELAILLGMLDPAEKDEK.G | 2 | 1886.2 | 3.183 | 0.479 |

+1 charge

```
		      B   	       Y
  1	 E 	   130.05	        -  	 17
  2	 L 	   243.13	  1755.96	 16
  3	 A 	   314.17	  1642.87	 15
  4	 I 	   427.26	  1571.83	 14
  5	 L 	   540.34	  1458.75	 13
  6	 L 	   653.42	  1345.67	 12
  7	 G 	   710.44	  1232.58	 11
  8	 M 	   841.49	  1175.56	 10
  9	 L 	   954.57	  1044.52	  9
 10	 D 	  1069.60	   931.44	  8
 11	 P 	  1166.65	   816.41	  7
 12	 A 	  1237.69	   719.36	  6
 13	 E 	  1366.73	   648.32	  5
 14	 K 	  1494.82	   519.28	  4
 15	 D 	  1609.85	   391.18	  3
 16	 E 	  1738.89	   276.16	  2
 17	 K 	        -  	   147.11	  1  _
+2 charge
		      B   	       Y
  1	 E 	    65.53	        -  	 17
  2	 L 	   122.07	   878.48	 16
  3	 A 	   157.59	   821.94	 15
  4	 I 	   214.13	   786.42	 14
  5	 L 	   270.67	   729.88	 13
  6	 L 	   327.22	   673.34	 12
  7	 G 	   355.73	   616.80	 11
  8	 M 	   421.25	   588.28	 10
  9	 L 	   477.79	   522.76	  9
 10	 D 	   535.30	   466.22	  8
 11	 P 	   583.83	   408.71	  7
 12	 A 	   619.35	   360.18	  6
 13	 E 	   683.87	   324.66	  5
 14	 K 	   747.92	   260.14	  4
 15	 D 	   805.43	   196.09	  3
 16	 E 	   869.95	   138.58	  2
 17	 K 	        -  	    74.06	  1  _
```

**115. IPI00744184 Arylsulfatase A**

| Sequence | Charge | M+H+ | XCorr | DeltaCn |
| --- | --- | --- | --- | --- |
| R.GGLPLEEVTVAEVLAAR.G | 2 | 1724.98 | 3.675 | 0.517 |

+1 charge

```
		      B   	       Y
  1	 G 	    58.03	        -  	 17
  2	 G 	   115.05	  1666.94	 16
  3	 L 	   228.13	  1609.92	 15
  4	 P 	   325.19	  1496.83	 14
  5	 L 	   438.27	  1399.78	 13
  6	 E 	   567.31	  1286.70	 12
  7	 E 	   696.36	  1157.65	 11
  8	 V 	   795.42	  1028.61	 10
  9	 T 	   896.47	   929.54	  9
 10	 V 	   995.54	   828.49	  8
 11	 A 	  1066.58	   729.43	  7
 12	 E 	  1195.62	   658.39	  6
 13	 V 	  1294.69	   529.35	  5
 14	 L 	  1407.77	   430.28	  4
 15	 A 	  1478.81	   317.19	  3
 16	 A 	  1549.85	   246.16	  2
 17	 R 	        -  	   175.12	  1  _
+2 charge
		      B   	       Y
  1	 G 	    29.52	        -  	 17
  2	 G 	    58.03	   833.97	 16
  3	 L 	   114.57	   805.46	 15
  4	 P 	   163.10	   748.92	 14
  5	 L 	   219.64	   700.39	 13
  6	 E 	   284.16	   643.85	 12
  7	 E 	   348.68	   579.33	 11
  8	 V 	   398.22	   514.81	 10
  9	 T 	   448.74	   465.27	  9
 10	 V 	   498.27	   414.75	  8
 11	 A 	   533.79	   365.22	  7
 12	 E 	   598.31	   329.70	  6
 13	 V 	   647.85	   265.18	  5
 14	 L 	   704.39	   215.64	  4
 15	 A 	   739.91	   159.10	  3
 16	 A 	   775.43	   123.58	  2
 17	 R 	        -  	    88.06	  1  _
```

**116. IPI00020091 Alpha-1-acid glycoprotein 2**

| Sequence | Charge | M+H+ | XCorr | DeltaCn |
| --- | --- | --- | --- | --- |
| K.EQLGEFYEALDCLCIPR.S | 2 | 2114.328 | 4.682 | 0.603 |

+1 charge

```
		      B   	       Y
  1	 E 	   130.05	        -  	 17
  2	 Q 	   258.11	  1984.20	 16
  3	 L 	   371.19	  1856.14	 15
  4	 G 	   428.21	  1743.06	 14
  5	 E 	   557.26	  1686.04	 13
  6	 F 	   704.32	  1556.99	 12
  7	 Y 	   867.39	  1409.92	 11
  8	 E 	   996.43	  1246.86	 10
  9	 A 	  1067.47	  1117.82	  9
 10	 L 	  1180.55	  1046.78	  8
 11	 D 	  1295.58	   933.70	  7
 12	 C 	  1455.74	   818.67	  6
 13	 L 	  1568.83	   658.51	  5
 14	 C 	  1728.99	   545.42	  4
 15	 I 	  1842.08	   385.26	  3
 16	 P 	  1939.13	   272.17	  2
 17	 R 	        -  	   175.12	  1  _
+2 charge
		      B   	       Y
  1	 E 	    65.53	        -  	 17
  2	 Q 	   129.56	   992.60	 16
  3	 L 	   186.10	   928.57	 15
  4	 G 	   214.61	   872.03	 14
  5	 E 	   279.13	   843.52	 13
  6	 F 	   352.67	   779.00	 12
  7	 Y 	   434.20	   705.47	 11
  8	 E 	   498.72	   623.93	 10
  9	 A 	   534.24	   559.41	  9
 10	 L 	   590.78	   523.89	  8
 11	 D 	   648.29	   467.35	  7
 12	 C 	   728.38	   409.84	  6
 13	 L 	   784.92	   329.76	  5
 14	 C 	   865.00	   273.21	  4
 15	 I 	   921.54	   193.13	  3
 16	 P 	   970.07	   136.59	  2
 17	 R 	        -  	    88.06	  1  _
```

**117. IPI00303161 Endothelial cell-selective adhesion molecule**

| Sequence | Charge | M+H+ | XCorr | DeltaCn |
| --- | --- | --- | --- | --- |
| R.QLPSFQTFFAPALDVIR.G | 2 | 1951.257 | 4.408 | 0.551 |

+1 charge

```
		      B   	       Y
  1	 Q 	   129.07	        -  	 17
  2	 L 	   242.15	  1821.99	 16
  3	 P 	   339.20	  1708.91	 15
  4	 S 	   426.23	  1611.85	 14
  5	 F 	   573.30	  1524.82	 13
  6	 Q 	   701.36	  1377.75	 12
  7	 T 	   802.41	  1249.69	 11
  8	 F 	   949.48	  1148.65	 10
  9	 F 	  1096.55	  1001.58	  9
 10	 A 	  1167.58	   854.51	  8
 11	 P 	  1264.64	   783.47	  7
 12	 A 	  1335.67	   686.42	  6
 13	 L 	  1448.76	   615.38	  5
 14	 D 	  1563.78	   502.30	  4
 15	 V 	  1662.85	   387.27	  3
 16	 I 	  1775.94	   288.20	  2
 17	 R 	        -  	   175.12	  1  _
+2 charge
		      B   	       Y
  1	 Q 	    65.04	        -  	 17
  2	 L 	   121.58	   911.50	 16
  3	 P 	   170.10	   854.96	 15
  4	 S 	   213.62	   806.43	 14
  5	 F 	   287.16	   762.91	 13
  6	 Q 	   351.18	   689.38	 12
  7	 T 	   401.71	   625.35	 11
  8	 F 	   475.24	   574.83	 10
  9	 F 	   548.78	   501.29	  9
 10	 A 	   584.30	   427.76	  8
 11	 P 	   632.82	   392.24	  7
 12	 A 	   668.34	   343.71	  6
 13	 L 	   724.88	   308.19	  5
 14	 D 	   782.40	   251.65	  4
 15	 V 	   831.93	   194.14	  3
 16	 I 	   888.47	   144.61	  2
 17	 R 	        -  	    88.06	  1  _
```

**118. IPI00005573 Isoform 1 of 5'(3')-deoxyribonucleotidase, cytosolic type**

| Sequence | Charge | M+H+ | XCorr | DeltaCn |
| --- | --- | --- | --- | --- |
| R.VLVDMDGVLADFEAGLLR.G | 2 | 1934.246 | 4.337 | 0.568 |

+1 charge

```
		      B   	       Y
  1	 V 	   100.08	        -  	 18
  2	 L 	   213.16	  1833.94	 17
  3	 V 	   312.23	  1720.86	 16
  4	 D 	   427.26	  1621.79	 15
  5	 M 	   558.30	  1506.76	 14
  6	 D 	   673.32	  1375.72	 13
  7	 G 	   730.34	  1260.69	 12
  8	 V 	   829.41	  1203.67	 11
  9	 L 	   942.50	  1104.60	 10
 10	 A 	  1013.53	   991.52	  9
 11	 D 	  1128.56	   920.48	  8
 12	 F 	  1275.63	   805.46	  7
 13	 E 	  1404.67	   658.39	  6
 14	 A 	  1475.71	   529.35	  5
 15	 G 	  1532.73	   458.31	  4
 16	 L 	  1645.81	   401.29	  3
 17	 L 	  1758.90	   288.20	  2
 18	 R 	        -  	   175.12	  1  _
+2 charge
		      B   	       Y
  1	 V 	    50.54	        -  	 18
  2	 L 	   107.08	   917.47	 17
  3	 V 	   156.62	   860.93	 16
  4	 D 	   214.13	   811.40	 15
  5	 M 	   279.65	   753.88	 14
  6	 D 	   337.16	   688.36	 13
  7	 G 	   365.68	   630.85	 12
  8	 V 	   415.21	   602.34	 11
  9	 L 	   471.75	   552.81	 10
 10	 A 	   507.27	   496.26	  9
 11	 D 	   564.78	   460.75	  8
 12	 F 	   638.32	   403.23	  7
 13	 E 	   702.84	   329.70	  6
 14	 A 	   738.36	   265.18	  5
 15	 G 	   766.87	   229.66	  4
 16	 L 	   823.41	   201.15	  3
 17	 L 	   879.95	   144.61	  2
 18	 R 	        -  	    88.06	  1  _
```

**119. IPI00219806 Protein S100-A7**

| Sequence | Charge | M+H+ | XCorr | DeltaCn |
| --- | --- | --- | --- | --- |
| K.KIDFSEFLSLLGDIATDYHK.Q | 3 | 2313.591 | 4.595 | 0.472 |

+1 charge

```
		      B   	       Y
  1	 K 	   129.10	        -  	 20
  2	 I 	   242.19	  2184.09	 19
  3	 D 	   357.21	  2071.00	 18
  4	 F 	   504.28	  1955.97	 17
  5	 S 	   591.31	  1808.91	 16
  6	 E 	   720.36	  1721.87	 15
  7	 F 	   867.42	  1592.83	 14
  8	 L 	   980.51	  1445.76	 13
  9	 S 	  1067.54	  1332.68	 12
 10	 L 	  1180.62	  1245.65	 11
 11	 L 	  1293.71	  1132.56	 10
 12	 G 	  1350.73	  1019.48	  9
 13	 D 	  1465.76	   962.46	  8
 14	 I 	  1578.84	   847.43	  7
 15	 A 	  1649.88	   734.35	  6
 16	 T 	  1750.93	   663.31	  5
 17	 D 	  1865.95	   562.26	  4
 18	 Y 	  2029.02	   447.24	  3
 19	 H 	  2166.08	   284.17	  2
 20	 K 	        -  	   147.11	  1  _
+2 charge
		      B   	       Y
  1	 K 	    65.05	        -  	 20
  2	 I 	   121.60	  1092.55	 19
  3	 D 	   179.11	  1036.00	 18
  4	 F 	   252.64	   978.49	 17
  5	 S 	   296.16	   904.96	 16
  6	 E 	   360.68	   861.44	 15
  7	 F 	   434.22	   796.92	 14
  8	 L 	   490.76	   723.39	 13
  9	 S 	   534.27	   666.84	 12
 10	 L 	   590.82	   623.33	 11
 11	 L 	   647.36	   566.79	 10
 12	 G 	   675.87	   510.24	  9
 13	 D 	   733.38	   481.73	  8
 14	 I 	   789.92	   424.22	  7
 15	 A 	   825.44	   367.68	  6
 16	 T 	   875.97	   332.16	  5
 17	 D 	   933.48	   281.63	  4
 18	 Y 	  1015.01	   224.12	  3
 19	 H 	  1083.54	   142.59	  2
 20	 K 	        -  	    74.06	  1  _

+3 charge
		      B   	       Y
  1	 K 	    43.71	        -  	 20
  2	 I 	    81.40	   728.70	 19
  3	 D 	   119.74	   691.01	 18
  4	 F 	   168.77	   652.66	 17
  5	 S 	   197.78	   603.64	 16
  6	 E 	   240.79	   574.63	 15
  7	 F 	   289.81	   531.62	 14
  8	 L 	   327.51	   482.59	 13
  9	 S 	   356.52	   444.90	 12
 10	 L 	   394.21	   415.89	 11
 11	 L 	   431.91	   378.19	 10
 12	 G 	   450.91	   340.50	  9
 13	 D 	   489.26	   321.49	  8
 14	 I 	   526.95	   283.15	  7
 15	 A 	   550.63	   245.45	  6
 16	 T 	   584.31	   221.77	  5
 17	 D 	   622.66	   188.09	  4
 18	 Y 	   677.01	   149.75	  3
 19	 H 	   722.70	    95.40	  2
 20	 K 	        -  	    49.71	  1  _
```

**Female 10.**

**1. IPI00292150 Latent-transforming growth factor beta-binding protein 2**

| Sequence | Charge | M+H+ | XCorr | DeltaCn |
| --- | --- | --- | --- | --- |
| R.CHCSPGYVAEAGPPHCTAKE.- | 3 | 2229.373 | 5.292 | 0.365 |

+1 charge

```
		      B   	       Y
  1	 C 	   161.17	        -  	 20
  2	 H 	   298.23	  2068.17	 19
  3	 C 	   458.40	  1931.11	 18
  4	 S 	   545.43	  1770.95	 17
  5	 P 	   642.48	  1683.91	 16
  6	 G 	   699.50	  1586.86	 15
  7	 Y 	   862.57	  1529.84	 14
  8	 V 	   961.63	  1366.78	 13
  9	 A 	  1032.67	  1267.71	 12
 10	 E 	  1161.71	  1196.67	 11
 11	 A 	  1232.75	  1067.63	 10
 12	 G 	  1289.77	   996.59	  9
 13	 P 	  1386.83	   939.57	  8
 14	 P 	  1483.88	   842.52	  7
 15	 H 	  1620.94	   745.46	  6
 16	 C 	  1781.10	   608.41	  5
 17	 T 	  1882.15	   448.24	  4
 18	 A 	  1953.19	   347.19	  3
 19	 K 	  2081.28	   276.16	  2
 20	 E 	        -  	   148.06	  1  _
+2 charge
		      B   	       Y
  1	 C 	    81.09	        -  	 20
  2	 H 	   149.62	  1034.59	 19
  3	 C 	   229.70	   966.06	 18
  4	 S 	   273.22	   885.98	 17
  5	 P 	   321.74	   842.46	 16
  6	 G 	   350.26	   793.93	 15
  7	 Y 	   431.79	   765.42	 14
  8	 V 	   481.32	   683.89	 13
  9	 A 	   516.84	   634.36	 12
 10	 E 	   581.36	   598.84	 11
 11	 A 	   616.88	   534.32	 10
 12	 G 	   645.39	   498.80	  9
 13	 P 	   693.92	   470.29	  8
 14	 P 	   742.44	   421.76	  7
 15	 H 	   810.97	   373.24	  6
 16	 C 	   891.05	   304.71	  5
 17	 T 	   941.58	   224.62	  4
 18	 A 	   977.10	   174.10	  3
 19	 K 	  1041.14	   138.58	  2
 20	 E 	        -  	    74.53	  1  _


+3 charge
		      B   	       Y
  1	 C 	    54.40	        -  	 20
  2	 H 	   100.08	   690.06	 19
  3	 C 	   153.47	   644.38	 18
  4	 S 	   182.48	   590.99	 17
  5	 P 	   214.83	   561.98	 16
  6	 G 	   233.84	   529.63	 15
  7	 Y 	   288.19	   510.62	 14
  8	 V 	   321.22	   456.26	 13
  9	 A 	   344.90	   423.24	 12
 10	 E 	   387.91	   399.56	 11
 11	 A 	   411.59	   356.55	 10
 12	 G 	   430.60	   332.87	  9
 13	 P 	   462.95	   313.86	  8
 14	 P 	   495.30	   281.51	  7
 15	 H 	   540.98	   249.16	  6
 16	 C 	   594.37	   203.47	  5
 17	 T 	   628.05	   150.08	  4
 18	 A 	   651.73	   116.40	  3
 19	 K 	   694.43	    92.72	  2
 20	 E 	        -  	    50.02	  1  _
```

**2. IPI00005142 Isoform 1 of Basic fibroblast growth factor receptor 1**

| Sequence | Charge | M+H+ | XCorr | DeltaCn |
| --- | --- | --- | --- | --- |
| K.FKCPSSGTPNPTLR.W | 2 | 1562.746 | 3.051 | 0.319 |

+1 charge

```
		      B   	       Y
  1	 F 	   148.08	        -  	 14
  2	 K 	   276.17	  1414.85	 13
  3	 C 	   436.34	  1286.75	 12
  4	 P 	   533.39	  1126.59	 11
  5	 S 	   620.42	  1029.53	 10
  6	 S 	   707.45	   942.50	  9
  7	 G 	   764.47	   855.47	  8
  8	 T 	   865.52	   798.45	  7
  9	 P 	   962.57	   697.40	  6
 10	 N 	  1076.62	   600.35	  5
 11	 P 	  1173.67	   486.30	  4
 12	 T 	  1274.72	   389.25	  3
 13	 L 	  1387.80	   288.20	  2
 14	 R 	        -  	   175.12	  1  _
+2 charge
		      B   	       Y
  1	 F 	    74.54	        -  	 14
  2	 K 	   138.59	   707.93	 13
  3	 C 	   218.67	   643.88	 12
  4	 P 	   267.20	   563.80	 11
  5	 S 	   310.71	   515.27	 10
  6	 S 	   354.23	   471.75	  9
  7	 G 	   382.74	   428.24	  8
  8	 T 	   433.26	   399.73	  7
  9	 P 	   481.79	   349.20	  6
 10	 N 	   538.81	   300.68	  5
 11	 P 	   587.34	   243.66	  4
 12	 T 	   637.86	   195.13	  3
 13	 L 	   694.40	   144.61	  2
 14	 R 	        -  	    88.06	  1  _
```

**3. IPI00152018 Isoform 1 of CMRF35-like molecule 9**

| Sequence | Charge | M+H+ | XCorr | DeltaCn |
| --- | --- | --- | --- | --- |
| R.KGGILFSR.C | 2 | 878.0538 | 3.018 | 0.34 |

+1 charge

```
		      B   	       Y
  1	 K 	   129.10	        -  	  8
  2	 G 	   186.12	   749.43	  7
  3	 G 	   243.15	   692.41	  6
  4	 I 	   356.23	   635.39	  5
  5	 L 	   469.31	   522.30	  4
  6	 F 	   616.38	   409.22	  3
  7	 S 	   703.41	   262.15	  2
  8	 R 	        -  	   175.12	  1  _
+2 charge
		      B   	       Y
  1	 K 	    65.05	        -  	  8
  2	 G 	    93.57	   375.22	  7
  3	 G 	   122.08	   346.71	  6
  4	 I 	   178.62	   318.20	  5
  5	 L 	   235.16	   261.66	  4
  6	 F 	   308.69	   205.11	  3
  7	 S 	   352.21	   131.58	  2
  8	 R 	        -  	    88.06	  1  _
```

**4. IPI00219827 Secreted Ly-6/uPAR-related protein 2**

| Sequence | Charge | M+H+ | XCorr | DeltaCn |
| --- | --- | --- | --- | --- |
| R.VLSNTEDLPLVTK.M | 2 | 1429.641 | 4.18 | 0.574 |

+1 charge

```
		      B   	       Y
  1	 V 	   100.08	        -  	 13
  2	 L 	   213.16	  1329.73	 12
  3	 S 	   300.19	  1216.64	 11
  4	 N 	   414.23	  1129.61	 10
  5	 T 	   515.28	  1015.57	  9
  6	 E 	   644.32	   914.52	  8
  7	 D 	   759.35	   785.48	  7
  8	 L 	   872.44	   670.45	  6
  9	 P 	   969.49	   557.37	  5
 10	 L 	  1082.57	   460.31	  4
 11	 V 	  1181.64	   347.23	  3
 12	 T 	  1282.69	   248.16	  2
 13	 K 	        -  	   147.11	  1  _
+2 charge
		      B   	       Y
  1	 V 	    50.54	        -  	 13
  2	 L 	   107.08	   665.37	 12
  3	 S 	   150.60	   608.82	 11
  4	 N 	   207.62	   565.31	 10
  5	 T 	   258.14	   508.29	  9
  6	 E 	   322.67	   457.76	  8
  7	 D 	   380.18	   393.24	  7
  8	 L 	   436.72	   335.73	  6
  9	 P 	   485.25	   279.19	  5
 10	 L 	   541.79	   230.66	  4
 11	 V 	   591.32	   174.12	  3
 12	 T 	   641.85	   124.58	  2
 13	 K 	        -  	    74.06	  1  _
```

**5. IPI00295777 Glycerol-3-phosphate dehydrogenase [NAD+], cytoplasmic**

| Sequence | Charge | M+H+ | XCorr | DeltaCn |
| --- | --- | --- | --- | --- |
| K.IVGGNAAQLAQFDPR.V | 2 | 1557.736 | 3.049 | 0.377 |

+1 charge

```
		      B   	       Y
  1	 I 	   114.09	        -  	 15
  2	 V 	   213.16	  1443.73	 14
  3	 G 	   270.18	  1344.67	 13
  4	 G 	   327.20	  1287.64	 12
  5	 N 	   441.25	  1230.62	 11
  6	 A 	   512.28	  1116.58	 10
  7	 A 	   583.32	  1045.54	  9
  8	 Q 	   711.38	   974.51	  8
  9	 L 	   824.46	   846.45	  7
 10	 A 	   895.50	   733.36	  6
 11	 Q 	  1023.56	   662.33	  5
 12	 F 	  1170.63	   534.27	  4
 13	 D 	  1285.65	   387.20	  3
 14	 P 	  1382.71	   272.17	  2
 15	 R 	        -  	   175.12	  1  _
+2 charge
		      B   	       Y
  1	 I 	    57.55	        -  	 15
  2	 V 	   107.08	   722.37	 14
  3	 G 	   135.59	   672.84	 13
  4	 G 	   164.10	   644.33	 12
  5	 N 	   221.13	   615.81	 11
  6	 A 	   256.64	   558.79	 10
  7	 A 	   292.16	   523.27	  9
  8	 Q 	   356.19	   487.76	  8
  9	 L 	   412.73	   423.73	  7
 10	 A 	   448.25	   367.19	  6
 11	 Q 	   512.28	   331.67	  5
 12	 F 	   585.82	   267.64	  4
 13	 D 	   643.33	   194.10	  3
 14	 P 	   691.86	   136.59	  2
 15	 R 	        -  	    88.06	  1  _
```

**6. IPI00382436 Ig lambda chain V-III region SH**

| Sequence | Charge | M+H+ | XCorr | DeltaCn |
| --- | --- | --- | --- | --- |
| - .SELTQDPAVSVALGQTVR.I | 2 | 1872.07 | 4.385 | 0.437 |

+1 charge

```
		      B   	       Y
  1	 S 	    88.04	        -  	 18
  2	 E 	   217.08	  1783.95	 17
  3	 L 	   330.17	  1654.91	 16
  4	 T 	   431.21	  1541.83	 15
  5	 Q 	   559.27	  1440.78	 14
  6	 D 	   674.30	  1312.72	 13
  7	 P 	   771.35	  1197.69	 12
  8	 A 	   842.39	  1100.64	 11
  9	 V 	   941.46	  1029.61	 10
 10	 S 	  1028.49	   930.54	  9
 11	 V 	  1127.56	   843.50	  8
 12	 A 	  1198.60	   744.44	  7
 13	 L 	  1311.68	   673.40	  6
 14	 G 	  1368.70	   560.32	  5
 15	 Q 	  1496.76	   503.29	  4
 16	 T 	  1597.81	   375.24	  3
 17	 V 	  1696.88	   274.19	  2
 18	 R 	        -  	   175.12	  1  _
+2 charge
		      B   	       Y
  1	 S 	    44.52	        -  	 18
  2	 E 	   109.04	   892.48	 17
  3	 L 	   165.59	   827.96	 16
  4	 T 	   216.11	   771.42	 15
  5	 Q 	   280.14	   720.89	 14
  6	 D 	   337.65	   656.86	 13
  7	 P 	   386.18	   599.35	 12
  8	 A 	   421.70	   550.82	 11
  9	 V 	   471.23	   515.31	 10
 10	 S 	   514.75	   465.77	  9
 11	 V 	   564.28	   422.26	  8
 12	 A 	   599.80	   372.72	  7
 13	 L 	   656.34	   337.20	  6
 14	 G 	   684.85	   280.66	  5
 15	 Q 	   748.88	   252.15	  4
 16	 T 	   799.41	   188.12	  3
 17	 V 	   848.94	   137.60	  2
 18	 R 	        -  	    88.06	  1  _
```

**7. IPI00009901 Nuclear transport factor 2**

| Sequence | Charge | M+H+ | XCorr | DeltaCn |
| --- | --- | --- | --- | --- |
| K.AAIVEKLSSLPFQK.I | 2 | 1531.821 | 3.011 | 0.464 |

+1 charge

```
		      B   	       Y
  1	 A 	    72.04	        -  	 14
  2	 A 	   143.08	  1459.85	 13
  3	 I 	   256.17	  1388.81	 12
  4	 V 	   355.23	  1275.73	 11
  5	 E 	   484.28	  1176.66	 10
  6	 K 	   612.37	  1047.62	  9
  7	 L 	   725.46	   919.52	  8
  8	 S 	   812.49	   806.44	  7
  9	 S 	   899.52	   719.41	  6
 10	 L 	  1012.60	   632.38	  5
 11	 P 	  1109.66	   519.29	  4
 12	 F 	  1256.72	   422.24	  3
 13	 Q 	  1384.78	   275.17	  2
 14	 K 	        -  	   147.11	  1  _
+2 charge
		      B   	       Y
  1	 A 	    36.53	        -  	 14
  2	 A 	    72.04	   730.43	 13
  3	 I 	   128.59	   694.91	 12
  4	 V 	   178.12	   638.37	 11
  5	 E 	   242.64	   588.83	 10
  6	 K 	   306.69	   524.31	  9
  7	 L 	   363.23	   460.27	  8
  8	 S 	   406.75	   403.72	  7
  9	 S 	   450.26	   360.21	  6
 10	 L 	   506.81	   316.69	  5
 11	 P 	   555.33	   260.15	  4
 12	 F 	   628.87	   211.62	  3
 13	 Q 	   692.90	   138.09	  2
 14	 K 	        -  	    74.06	  1  _
```

**8. IPI00304273 Apolipoprotein A-IV**

| Sequence | Charge | M+H+ | XCorr | DeltaCn |
| --- | --- | --- | --- | --- |
| K.SLAELGGHLDQQVEEFR.R | 2 | 1929.08 | 3.363 | 0.611 |

+1 charge

```
		      B   	       Y
  1	 S 	    88.04	        -  	 17
  2	 L 	   201.12	  1840.92	 16
  3	 A 	   272.16	  1727.83	 15
  4	 E 	   401.20	  1656.80	 14
  5	 L 	   514.29	  1527.76	 13
  6	 G 	   571.31	  1414.67	 12
  7	 G 	   628.33	  1357.65	 11
  8	 H 	   765.39	  1300.63	 10
  9	 L 	   878.47	  1163.57	  9
 10	 D 	   993.50	  1050.49	  8
 11	 Q 	  1121.56	   935.46	  7
 12	 Q 	  1249.62	   807.40	  6
 13	 V 	  1348.69	   679.34	  5
 14	 E 	  1477.73	   580.27	  4
 15	 E 	  1606.77	   451.23	  3
 16	 F 	  1753.84	   322.19	  2
 17	 R 	        -  	   175.12	  1  _
+2 charge
		      B   	       Y
  1	 S 	    44.52	        -  	 17
  2	 L 	   101.07	   920.96	 16
  3	 A 	   136.58	   864.42	 15
  4	 E 	   201.11	   828.90	 14
  5	 L 	   257.65	   764.38	 13
  6	 G 	   286.16	   707.84	 12
  7	 G 	   314.67	   679.33	 11
  8	 H 	   383.20	   650.82	 10
  9	 L 	   439.74	   582.29	  9
 10	 D 	   497.25	   525.75	  8
 11	 Q 	   561.28	   468.23	  7
 12	 Q 	   625.31	   404.20	  6
 13	 V 	   674.85	   340.17	  5
 14	 E 	   739.37	   290.64	  4
 15	 E 	   803.89	   226.12	  3
 16	 F 	   877.42	   161.60	  2
 17	 R 	        -  	    88.06	  1  _
```

**9. IPI00418169 Isoform 2 of Annexin A2**

| Sequence | Charge | M+H+ | XCorr | DeltaCn |
| --- | --- | --- | --- | --- |
| K.GVDEVTIVNILTNR.S | 2 | 1543.747 | 3.145 | 0.321 |

+1 charge

```
		      B   	       Y
  1	 G 	    58.03	        -  	 14
  2	 V 	   157.10	  1485.83	 13
  3	 D 	   272.12	  1386.76	 12
  4	 E 	   401.17	  1271.73	 11
  5	 V 	   500.24	  1142.69	 10
  6	 T 	   601.28	  1043.62	  9
  7	 I 	   714.37	   942.57	  8
  8	 V 	   813.44	   829.49	  7
  9	 N 	   927.48	   730.42	  6
 10	 I 	  1040.56	   616.38	  5
 11	 L 	  1153.65	   503.29	  4
 12	 T 	  1254.69	   390.21	  3
 13	 N 	  1368.74	   289.16	  2
 14	 R 	        -  	   175.12	  1  _
+2 charge
		      B   	       Y
  1	 G 	    29.52	        -  	 14
  2	 V 	    79.05	   743.42	 13
  3	 D 	   136.57	   693.88	 12
  4	 E 	   201.09	   636.37	 11
  5	 V 	   250.62	   571.85	 10
  6	 T 	   301.15	   522.31	  9
  7	 I 	   357.69	   471.79	  8
  8	 V 	   407.22	   415.25	  7
  9	 N 	   464.24	   365.71	  6
 10	 I 	   520.78	   308.69	  5
 11	 L 	   577.33	   252.15	  4
 12	 T 	   627.85	   195.61	  3
 13	 N 	   684.87	   145.08	  2
 14	 R 	        -  	    88.06	  1  _
```

**10. IPI00010949 Isoform 1 of Sialate O-acetylesterase**

| Sequence | Charge | M+H+ | XCorr | DeltaCn |
| --- | --- | --- | --- | --- |
| R.FFPFGLVQLSSDLSK.K | 2 | 1685.944 | 3.279 | 0.547 |

+1 charge

```
		      B   	       Y
  1	 F 	   148.08	        -  	 15
  2	 F 	   295.14	  1537.83	 14
  3	 P 	   392.20	  1390.76	 13
  4	 F 	   539.27	  1293.70	 12
  5	 G 	   596.29	  1146.64	 11
  6	 L 	   709.37	  1089.62	 10
  7	 V 	   808.44	   976.53	  9
  8	 Q 	   936.50	   877.46	  8
  9	 L 	  1049.58	   749.40	  7
 10	 S 	  1136.61	   636.32	  6
 11	 S 	  1223.65	   549.29	  5
 12	 D 	  1338.67	   462.26	  4
 13	 L 	  1451.76	   347.23	  3
 14	 S 	  1538.79	   234.14	  2
 15	 K 	        -  	   147.11	  1  _
+2 charge
		      B   	       Y
  1	 F 	    74.54	        -  	 15
  2	 F 	   148.08	   769.42	 14
  3	 P 	   196.60	   695.88	 13
  4	 F 	   270.14	   647.36	 12
  5	 G 	   298.65	   573.82	 11
  6	 L 	   355.19	   545.31	 10
  7	 V 	   404.72	   488.77	  9
  8	 Q 	   468.75	   439.23	  8
  9	 L 	   525.29	   375.21	  7
 10	 S 	   568.81	   318.66	  6
 11	 S 	   612.33	   275.15	  5
 12	 D 	   669.84	   231.63	  4
 13	 L 	   726.38	   174.12	  3
 14	 S 	   769.90	   117.58	  2
 15	 K 	        -  	    74.06	  1  _
```

**11. IPI00921523 Isoform 1 of Complement factor B (Fragment)**

| Sequence | Charge | M+H+ | XCorr | DeltaCn |
| --- | --- | --- | --- | --- |
| R.STGSWSTLKTQDQK.T | 2 | 1567.683 | 3.325 | 0.313 |

+1 charge

```
		      B   	       Y
  1	 S 	    88.04	        -  	 14
  2	 T 	   189.09	  1479.74	 13
  3	 G 	   246.11	  1378.70	 12
  4	 S 	   333.14	  1321.67	 11
  5	 W 	   519.22	  1234.64	 10
  6	 S 	   606.25	  1048.56	  9
  7	 T 	   707.30	   961.53	  8
  8	 L 	   820.38	   860.48	  7
  9	 K 	   948.48	   747.40	  6
 10	 T 	  1049.53	   619.30	  5
 11	 Q 	  1177.58	   518.26	  4
 12	 D 	  1292.61	   390.20	  3
 13	 Q 	  1420.67	   275.17	  2
 14	 K 	        -  	   147.11	  1  _
+2 charge
		      B   	       Y
  1	 S 	    44.52	        -  	 14
  2	 T 	    95.05	   740.38	 13
  3	 G 	   123.56	   689.85	 12
  4	 S 	   167.07	   661.34	 11
  5	 W 	   260.11	   617.82	 10
  6	 S 	   303.63	   524.79	  9
  7	 T 	   354.15	   481.27	  8
  8	 L 	   410.70	   430.75	  7
  9	 K 	   474.74	   374.20	  6
 10	 T 	   525.27	   310.16	  5
 11	 Q 	   589.30	   259.63	  4
 12	 D 	   646.81	   195.60	  3
 13	 Q 	   710.84	   138.09	  2
 14	 K 	        -  	    74.06	  1  _
```

**12. IPI00023501 Isoform 1 of Tumor necrosis factor receptor superfamily member 1B**

| Sequence | Charge | M+H+ | XCorr | DeltaCn |
| --- | --- | --- | --- | --- |
| R.LREYYDQTAQMCCSK.C | 2 | 1954.138 | 4.026 | 0.484 |

+1 charge

```
		      B   	       Y
  1	 L 	   114.09	        -  	 15
  2	 R 	   270.19	  1840.02	 14
  3	 E 	   399.24	  1683.91	 13
  4	 Y 	   562.30	  1554.87	 12
  5	 Y 	   725.36	  1391.81	 11
  6	 D 	   840.39	  1228.74	 10
  7	 Q 	   968.45	  1113.72	  9
  8	 T 	  1069.49	   985.66	  8
  9	 A 	  1140.53	   884.61	  7
 10	 Q 	  1268.59	   813.57	  6
 11	 M 	  1399.63	   685.52	  5
 12	 C 	  1559.80	   554.48	  4
 13	 C 	  1719.96	   394.31	  3
 14	 S 	  1806.99	   234.14	  2
 15	 K 	        -  	   147.11	  1  _
+2 charge
		      B   	       Y
  1	 L 	    57.55	        -  	 15
  2	 R 	   135.60	   920.51	 14
  3	 E 	   200.12	   842.46	 13
  4	 Y 	   281.65	   777.94	 12
  5	 Y 	   363.18	   696.41	 11
  6	 D 	   420.70	   614.88	 10
  7	 Q 	   484.73	   557.36	  9
  8	 T 	   535.25	   493.33	  8
  9	 A 	   570.77	   442.81	  7
 10	 Q 	   634.80	   407.29	  6
 11	 M 	   700.32	   343.26	  5
 12	 C 	   780.40	   277.74	  4
 13	 C 	   860.48	   197.66	  3
 14	 S 	   904.00	   117.58	  2
 15	 K 	        -  	    74.06	  1  _
```

**13. IPI00383048 Isoform 4 of Protein very KIND**

| Sequence | Charge | M+H+ | XCorr | DeltaCn |
| --- | --- | --- | --- | --- |
| R.DRLVCTGAPGGSTSEDR.E | 2 | 1778.853 | 3.131 | 0.257 |

+1 charge

```
		      B   	       Y
  1	 D 	   116.03	        -  	 17
  2	 R 	   272.14	  1662.92	 16
  3	 L 	   385.22	  1506.82	 15
  4	 V 	   484.29	  1393.74	 14
  5	 C 	   644.45	  1294.67	 13
  6	 T 	   745.50	  1134.50	 12
  7	 G 	   802.52	  1033.45	 11
  8	 A 	   873.56	   976.43	 10
  9	 P 	   970.61	   905.40	  9
 10	 G 	  1027.63	   808.34	  8
 11	 G 	  1084.65	   751.32	  7
 12	 S 	  1171.69	   694.30	  6
 13	 T 	  1272.73	   607.27	  5
 14	 S 	  1359.77	   506.22	  4
 15	 E 	  1488.81	   419.19	  3
 16	 D 	  1603.84	   290.15	  2
 17	 R 	        -  	   175.12	  1  _
+2 charge
		      B   	       Y
  1	 D 	    58.52	        -  	 17
  2	 R 	   136.57	   831.96	 16
  3	 L 	   193.11	   753.91	 15
  4	 V 	   242.65	   697.37	 14
  5	 C 	   322.73	   647.84	 13
  6	 T 	   373.25	   567.75	 12
  7	 G 	   401.76	   517.23	 11
  8	 A 	   437.28	   488.72	 10
  9	 P 	   485.81	   453.20	  9
 10	 G 	   514.32	   404.68	  8
 11	 G 	   542.83	   376.16	  7
 12	 S 	   586.35	   347.65	  6
 13	 T 	   636.87	   304.14	  5
 14	 S 	   680.39	   253.61	  4
 15	 E 	   744.91	   210.10	  3
 16	 D 	   802.42	   145.58	  2
 17	 R 	        -  	    88.06	  1  _
```

**14. IPI00304903 Cornifin-B**

| Sequence | Charge | M+H+ | XCorr | DeltaCn |
| --- | --- | --- | --- | --- |
| K.VPEPCPSIVTPAPAQQK.T | 2 | 1820.073 | 3.169 | 0.491 |

+1 charge

```
		      B   	       Y
  1	 V 	   100.08	        -  	 17
  2	 P 	   197.13	  1720.01	 16
  3	 E 	   326.17	  1622.96	 15
  4	 P 	   423.22	  1493.91	 14
  5	 C 	   583.39	  1396.86	 13
  6	 P 	   680.44	  1236.69	 12
  7	 S 	   767.47	  1139.64	 11
  8	 I 	   880.56	  1052.61	 10
  9	 V 	   979.63	   939.53	  9
 10	 T 	  1080.67	   840.46	  8
 11	 P 	  1177.73	   739.41	  7
 12	 A 	  1248.76	   642.36	  6
 13	 P 	  1345.82	   571.32	  5
 14	 A 	  1416.85	   474.27	  4
 15	 Q 	  1544.91	   403.23	  3
 16	 Q 	  1672.97	   275.17	  2
 17	 K 	        -  	   147.11	  1  _
+2 charge
		      B   	       Y
  1	 V 	    50.54	        -  	 17
  2	 P 	    99.07	   860.51	 16
  3	 E 	   163.59	   811.98	 15
  4	 P 	   212.12	   747.46	 14
  5	 C 	   292.20	   698.93	 13
  6	 P 	   340.72	   618.85	 12
  7	 S 	   384.24	   570.32	 11
  8	 I 	   440.78	   526.81	 10
  9	 V 	   490.32	   470.27	  9
 10	 T 	   540.84	   420.73	  8
 11	 P 	   589.37	   370.21	  7
 12	 A 	   624.89	   321.68	  6
 13	 P 	   673.41	   286.16	  5
 14	 A 	   708.93	   237.64	  4
 15	 Q 	   772.96	   202.12	  3
 16	 Q 	   836.99	   138.09	  2
 17	 K 	        -  	    74.06	  1  _
```

**15. IPI00939667 Isoform 1 of Tripeptidyl-peptidase 1**

| Sequence | Charge | M+H+ | XCorr | DeltaCn |
| --- | --- | --- | --- | --- |
| R.LFGGNFAHQASVAR.V | 2 | 1475.636 | 4.67 | 0.546 |

+1 charge

```
		      B   	       Y
  1	 L 	   114.09	        -  	 14
  2	 F 	   261.16	  1361.67	 13
  3	 G 	   318.18	  1214.60	 12
  4	 G 	   375.20	  1157.58	 11
  5	 N 	   489.25	  1100.56	 10
  6	 F 	   636.31	   986.52	  9
  7	 A 	   707.35	   839.45	  8
  8	 H 	   844.41	   768.41	  7
  9	 Q 	   972.47	   631.35	  6
 10	 A 	  1043.51	   503.29	  5
 11	 S 	  1130.54	   432.26	  4
 12	 V 	  1229.61	   345.22	  3
 13	 A 	  1300.64	   246.16	  2
 14	 R 	        -  	   175.12	  1  _
+2 charge
		      B   	       Y
  1	 L 	    57.55	        -  	 14
  2	 F 	   131.08	   681.34	 13
  3	 G 	   159.59	   607.80	 12
  4	 G 	   188.10	   579.29	 11
  5	 N 	   245.13	   550.78	 10
  6	 F 	   318.66	   493.76	  9
  7	 A 	   354.18	   420.23	  8
  8	 H 	   422.71	   384.71	  7
  9	 Q 	   486.74	   316.18	  6
 10	 A 	   522.26	   252.15	  5
 11	 S 	   565.77	   216.63	  4
 12	 V 	   615.31	   173.12	  3
 13	 A 	   650.83	   123.58	  2
 14	 R 	        -  	    88.06	  1  _
```

**16. IPI00012102 N-acetylglucosamine-6-sulfatase**

| Sequence | Charge | M+H+ | XCorr | DeltaCn |
| --- | --- | --- | --- | --- |
| R.LMMLQSCSGPTCR.T | 2 | 1541.807 | 3.992 | 0.493 |

+1 charge

```
		      B   	       Y
  1	 L 	   114.09	        -  	 13
  2	 M 	   245.13	  1427.86	 12
  3	 M 	   376.17	  1296.82	 11
  4	 L 	   489.26	  1165.78	 10
  5	 Q 	   617.31	  1052.69	  9
  6	 S 	   704.35	   924.64	  8
  7	 C 	   864.51	   837.60	  7
  8	 S 	   951.54	   677.44	  6
  9	 G 	  1008.57	   590.41	  5
 10	 P 	  1105.62	   533.38	  4
 11	 T 	  1206.67	   436.33	  3
 12	 C 	  1366.83	   335.28	  2
 13	 R 	        -  	   175.12	  1  _
+2 charge
		      B   	       Y
  1	 L 	    57.55	        -  	 13
  2	 M 	   123.07	   714.43	 12
  3	 M 	   188.59	   648.91	 11
  4	 L 	   245.13	   583.39	 10
  5	 Q 	   309.16	   526.85	  9
  6	 S 	   352.68	   462.82	  8
  7	 C 	   432.76	   419.31	  7
  8	 S 	   476.28	   339.22	  6
  9	 G 	   504.79	   295.71	  5
 10	 P 	   553.31	   267.20	  4
 11	 T 	   603.84	   218.67	  3
 12	 C 	   683.92	   168.15	  2
 13	 R 	        -  	    88.06	  1  _
```

**17. IPI00641737 Haptoglobin**

| Sequence | Charge | M+H+ | XCorr | DeltaCn |
| --- | --- | --- | --- | --- |
| K.LRTEGDGVYTLNNEKQWINK.A | 3 | 2379.614 | 3.661 | 0.383 |

+1 charge

```
		      B   	       Y
  1	 L 	   114.09	        -  	 20
  2	 R 	   270.19	  2265.13	 19
  3	 T 	   371.24	  2109.02	 18
  4	 E 	   500.28	  2007.98	 17
  5	 G 	   557.30	  1878.93	 16
  6	 D 	   672.33	  1821.91	 15
  7	 G 	   729.35	  1706.89	 14
  8	 V 	   828.42	  1649.86	 13
  9	 Y 	   991.48	  1550.80	 12
 10	 T 	  1092.53	  1387.73	 11
 11	 L 	  1205.62	  1286.69	 10
 12	 N 	  1319.66	  1173.60	  9
 13	 N 	  1433.70	  1059.56	  8
 14	 E 	  1562.74	   945.52	  7
 15	 K 	  1690.84	   816.47	  6
 16	 Q 	  1818.90	   688.38	  5
 17	 W 	  2004.98	   560.32	  4
 18	 I 	  2118.06	   374.24	  3
 19	 N 	  2232.10	   261.16	  2
 20	 K 	        -  	   147.11	  1  _
+2 charge
		      B   	       Y
  1	 L 	    57.55	        -  	 20
  2	 R 	   135.60	  1133.07	 19
  3	 T 	   186.12	  1055.02	 18
  4	 E 	   250.64	  1004.49	 17
  5	 G 	   279.16	   939.97	 16
  6	 D 	   336.67	   911.46	 15
  7	 G 	   365.18	   853.95	 14
  8	 V 	   414.71	   825.44	 13
  9	 Y 	   496.25	   775.90	 12
 10	 T 	   546.77	   694.37	 11
 11	 L 	   603.31	   643.85	 10
 12	 N 	   660.33	   587.30	  9
 13	 N 	   717.35	   530.28	  8
 14	 E 	   781.88	   473.26	  7
 15	 K 	   845.92	   408.74	  6
 16	 Q 	   909.95	   344.69	  5
 17	 W 	  1002.99	   280.66	  4
 18	 I 	  1059.53	   187.62	  3
 19	 N 	  1116.56	   131.08	  2
 20	 K 	        -  	    74.06	  1  _

+3 charge
		      B   	       Y
  1	 L 	    38.70	        -  	 20
  2	 R 	    90.74	   755.71	 19
  3	 T 	   124.42	   703.68	 18
  4	 E 	   167.43	   670.00	 17
  5	 G 	   186.44	   626.98	 16
  6	 D 	   224.78	   607.98	 15
  7	 G 	   243.79	   569.63	 14
  8	 V 	   276.81	   550.63	 13
  9	 Y 	   331.17	   517.60	 12
 10	 T 	   364.85	   463.25	 11
 11	 L 	   402.54	   429.57	 10
 12	 N 	   440.56	   391.87	  9
 13	 N 	   478.57	   353.86	  8
 14	 E 	   521.59	   315.84	  7
 15	 K 	   564.28	   272.83	  6
 16	 Q 	   606.97	   230.13	  5
 17	 W 	   669.00	   187.44	  4
 18	 I 	   706.69	   125.42	  3
 19	 N 	   744.71	    87.72	  2
 20	 K 	        -  	    49.71	  1  _
```

**18. IPI00651692 Stromal cell-derived factor 1 theta**

| Sequence | Charge | M+H+ | XCorr | DeltaCn |
| --- | --- | --- | --- | --- |
| K.ILNTPNCALQIVAR.L | 2 | 1583.851 | 4.331 | 0.518 |

+1 charge

```
		      B   	       Y
  1	 I 	   114.09	        -  	 14
  2	 L 	   227.18	  1469.92	 13
  3	 N 	   341.22	  1356.84	 12
  4	 T 	   442.27	  1242.80	 11
  5	 P 	   539.32	  1141.75	 10
  6	 N 	   653.36	  1044.70	  9
  7	 C 	   813.53	   930.65	  8
  8	 A 	   884.56	   770.49	  7
  9	 L 	   997.65	   699.45	  6
 10	 Q 	  1125.71	   586.37	  5
 11	 I 	  1238.79	   458.31	  4
 12	 V 	  1337.86	   345.22	  3
 13	 A 	  1408.90	   246.16	  2
 14	 R 	        -  	   175.12	  1  _
+2 charge
		      B   	       Y
  1	 I 	    57.55	        -  	 14
  2	 L 	   114.09	   735.47	 13
  3	 N 	   171.11	   678.92	 12
  4	 T 	   221.64	   621.90	 11
  5	 P 	   270.16	   571.38	 10
  6	 N 	   327.18	   522.85	  9
  7	 C 	   407.27	   465.83	  8
  8	 A 	   442.79	   385.75	  7
  9	 L 	   499.33	   350.23	  6
 10	 Q 	   563.36	   293.69	  5
 11	 I 	   619.90	   229.66	  4
 12	 V 	   669.43	   173.12	  3
 13	 A 	   704.95	   123.58	  2
 14	 R 	        -  	    88.06	  1  _
```

**19. IPI00022488 Hemopexin**

| Sequence | Charge | M+H+ | XCorr | DeltaCn |
| --- | --- | --- | --- | --- |
| R.YYCFQGNQFLR.F | 2 | 1496.644 | 2.986 | 0.405 |

+1 charge

```
		      B   	       Y
  1	 Y 	   164.07	        -  	 11
  2	 Y 	   327.13	  1332.75	 10
  3	 C 	   487.30	  1169.69	  9
  4	 F 	   634.37	  1009.52	  8
  5	 Q 	   762.43	   862.45	  7
  6	 G 	   819.45	   734.39	  6
  7	 N 	   933.49	   677.37	  5
  8	 Q 	  1061.55	   563.33	  4
  9	 F 	  1208.62	   435.27	  3
 10	 L 	  1321.70	   288.20	  2
 11	 R 	        -  	   175.12	  1  _
+2 charge
		      B   	       Y
  1	 Y 	    82.54	        -  	 11
  2	 Y 	   164.07	   666.88	 10
  3	 C 	   244.15	   585.35	  9
  4	 F 	   317.69	   505.26	  8
  5	 Q 	   381.72	   431.73	  7
  6	 G 	   410.23	   367.70	  6
  7	 N 	   467.25	   339.19	  5
  8	 Q 	   531.28	   282.17	  4
  9	 F 	   604.81	   218.14	  3
 10	 L 	   661.35	   144.61	  2
 11	 R 	        -  	    88.06	  1  _
```

**20. IPI00334282 Protein FAM3C**

| Sequence | Charge | M+H+ | XCorr | DeltaCn |
| --- | --- | --- | --- | --- |
| K.ICLEDNVLMSGVK.N | 2 | 1478.73 | 3.363 | 0.224 |

+1 charge

```
		      B   	       Y
  1	 I 	   114.09	        -  	 13
  2	 C 	   274.26	  1364.79	 12
  3	 L 	   387.34	  1204.62	 11
  4	 E 	   516.38	  1091.54	 10
  5	 D 	   631.41	   962.50	  9
  6	 N 	   745.45	   847.47	  8
  7	 V 	   844.52	   733.43	  7
  8	 L 	   957.61	   634.36	  6
  9	 M 	  1088.65	   521.28	  5
 10	 S 	  1175.68	   390.23	  4
 11	 G 	  1232.70	   303.20	  3
 12	 V 	  1331.77	   246.18	  2
 13	 K 	        -  	   147.11	  1  _
+2 charge
		      B   	       Y
  1	 I 	    57.55	        -  	 13
  2	 C 	   137.63	   682.90	 12
  3	 L 	   194.17	   602.82	 11
  4	 E 	   258.70	   546.27	 10
  5	 D 	   316.21	   481.75	  9
  6	 N 	   373.23	   424.24	  8
  7	 V 	   422.76	   367.22	  7
  8	 L 	   479.31	   317.68	  6
  9	 M 	   544.83	   261.14	  5
 10	 S 	   588.34	   195.62	  4
 11	 G 	   616.85	   152.10	  3
 12	 V 	   666.39	   123.59	  2
 13	 K 	        -  	    74.06	  1  _
```

**21．IPI00009477 Intercellular adhesion molecule 2**

| Sequence | Charge | M+H+ | XCorr | DeltaCn |
| --- | --- | --- | --- | --- |
| R.QVILTLQPTLVAVGK.S | 2 | 1580.937 | 3.87 | 0.495 |

+1 charge

```
		      B   	       Y
  1	 Q 	   129.07	        -  	 15
  2	 V 	   228.13	  1451.92	 14
  3	 I 	   341.22	  1352.85	 13
  4	 L 	   454.30	  1239.77	 12
  5	 T 	   555.35	  1126.68	 11
  6	 L 	   668.43	  1025.64	 10
  7	 Q 	   796.49	   912.55	  9
  8	 P 	   893.55	   784.49	  8
  9	 T 	   994.59	   687.44	  7
 10	 L 	  1107.68	   586.39	  6
 11	 V 	  1206.75	   473.31	  5
 12	 A 	  1277.78	   374.24	  4
 13	 V 	  1376.85	   303.20	  3
 14	 G 	  1433.87	   204.13	  2
 15	 K 	        -  	   147.11	  1  _
+2 charge
		      B   	       Y
  1	 Q 	    65.04	        -  	 15
  2	 V 	   114.57	   726.46	 14
  3	 I 	   171.11	   676.93	 13
  4	 L 	   227.65	   620.39	 12
  5	 T 	   278.18	   563.85	 11
  6	 L 	   334.72	   513.32	 10
  7	 Q 	   398.75	   456.78	  9
  8	 P 	   447.28	   392.75	  8
  9	 T 	   497.80	   344.22	  7
 10	 L 	   554.34	   293.70	  6
 11	 V 	   603.88	   237.16	  5
 12	 A 	   639.40	   187.62	  4
 13	 V 	   688.93	   152.10	  3
 14	 G 	   717.44	   102.57	  2
 15	 K 	        -  	    74.06	  1  _
```

**22. IPI00414888 Isoform 2 of ICOS ligand**

| Sequence | Charge | M+H+ | XCorr | DeltaCn |
| --- | --- | --- | --- | --- |
| R.GLYDVVSVLR.I | 2 | 1121.311 | 3.791 | 0.354 |

+1 charge

```
		      B   	       Y
  1	 G 	    58.03	        -  	 10
  2	 L 	   171.11	  1063.61	  9
  3	 Y 	   334.18	   950.53	  8
  4	 D 	   449.20	   787.47	  7
  5	 V 	   548.27	   672.44	  6
  6	 V 	   647.34	   573.37	  5
  7	 S 	   734.37	   474.30	  4
  8	 V 	   833.44	   387.27	  3
  9	 L 	   946.52	   288.20	  2
 10	 R 	        -  	   175.12	  1  _
+2 charge
		      B   	       Y
  1	 G 	    29.52	        -  	 10
  2	 L 	    86.06	   532.31	  9
  3	 Y 	   167.59	   475.77	  8
  4	 D 	   225.11	   394.24	  7
  5	 V 	   274.64	   336.72	  6
  6	 V 	   324.17	   287.19	  5
  7	 S 	   367.69	   237.66	  4
  8	 V 	   417.22	   194.14	  3
  9	 L 	   473.77	   144.61	  2
 10	 R 	        -  	    88.06	  1  _
```

**23. IPI00010737 Thrombomodulin**

| Sequence | Charge | M+H+ | XCorr | DeltaCn |
| --- | --- | --- | --- | --- |
| R.SSVAADVISLLLNGDGGVGR.R | 2 | 1901.111 | 3.595 | 0.589 |

+1 charge

```
		      B   	       Y
  1	 S 	    88.04	        -  	 20
  2	 S 	   175.07	  1812.98	 19
  3	 V 	   274.14	  1725.95	 18
  4	 A 	   345.18	  1626.88	 17
  5	 A 	   416.21	  1555.84	 16
  6	 D 	   531.24	  1484.81	 15
  7	 V 	   630.31	  1369.78	 14
  8	 I 	   743.39	  1270.71	 13
  9	 S 	   830.43	  1157.63	 12
 10	 L 	   943.51	  1070.60	 11
 11	 L 	  1056.59	   957.51	 10
 12	 L 	  1169.68	   844.43	  9
 13	 N 	  1283.72	   731.34	  8
 14	 G 	  1340.74	   617.30	  7
 15	 D 	  1455.77	   560.28	  6
 16	 G 	  1512.79	   445.25	  5
 17	 G 	  1569.81	   388.23	  4
 18	 V 	  1668.88	   331.21	  3
 19	 G 	  1725.90	   232.14	  2
 20	 R 	        -  	   175.12	  1  _
+2 charge
		      B   	       Y
  1	 S 	    44.52	        -  	 20
  2	 S 	    88.04	   906.99	 19
  3	 V 	   137.57	   863.48	 18
  4	 A 	   173.09	   813.94	 17
  5	 A 	   208.61	   778.43	 16
  6	 D 	   266.12	   742.91	 15
  7	 V 	   315.66	   685.39	 14
  8	 I 	   372.20	   635.86	 13
  9	 S 	   415.72	   579.32	 12
 10	 L 	   472.26	   535.80	 11
 11	 L 	   528.80	   479.26	 10
 12	 L 	   585.34	   422.72	  9
 13	 N 	   642.36	   366.18	  8
 14	 G 	   670.87	   309.15	  7
 15	 D 	   728.39	   280.64	  6
 16	 G 	   756.90	   223.13	  5
 17	 G 	   785.41	   194.62	  4
 18	 V 	   834.94	   166.11	  3
 19	 G 	   863.45	   116.57	  2
 20	 R 	        -  	    88.06	  1  _
```

**24. IPI00299724 Isoform 1 of Signal-regulatory protein beta-1**

| Sequence | Charge | M+H+ | XCorr | DeltaCn |
| --- | --- | --- | --- | --- |
| R.AKPSAPVVSGPAVR.A | 2 | 1336.565 | 3.04 | 0.49 |

+1 charge

```
		      B   	       Y
  1	 A 	    72.04	        -  	 14
  2	 K 	   200.14	  1264.74	 13
  3	 P 	   297.19	  1136.64	 12
  4	 S 	   384.22	  1039.59	 11
  5	 A 	   455.26	   952.56	 10
  6	 P 	   552.31	   881.52	  9
  7	 V 	   651.38	   784.47	  8
  8	 V 	   750.45	   685.40	  7
  9	 S 	   837.48	   586.33	  6
 10	 G 	   894.50	   499.30	  5
 11	 P 	   991.56	   442.28	  4
 12	 A 	  1062.59	   345.22	  3
 13	 V 	  1161.66	   274.19	  2
 14	 R 	        -  	   175.12	  1  _
+2 charge
		      B   	       Y
  1	 A 	    36.53	        -  	 14
  2	 K 	   100.57	   632.87	 13
  3	 P 	   149.10	   568.82	 12
  4	 S 	   192.62	   520.30	 11
  5	 A 	   228.13	   476.78	 10
  6	 P 	   276.66	   441.26	  9
  7	 V 	   326.19	   392.74	  8
  8	 V 	   375.73	   343.20	  7
  9	 S 	   419.25	   293.67	  6
 10	 G 	   447.76	   250.15	  5
 11	 P 	   496.28	   221.64	  4
 12	 A 	   531.80	   173.12	  3
 13	 V 	   581.33	   137.60	  2
 14	 R 	        -  	    88.06	  1  _
```

**25. IPI00005733 TGF-beta receptor type-1**

| Sequence | Charge | M+H+ | XCorr | DeltaCn |
| --- | --- | --- | --- | --- |
| R.DRPFVCAPSSK.T | 2 | 1264.406 | 3.125 | 0.308 |

+1 charge

```
		      B   	       Y
  1	 D 	   116.03	        -  	 11
  2	 R 	   272.14	  1148.72	 10
  3	 P 	   369.19	   992.62	  9
  4	 F 	   516.26	   895.57	  8
  5	 V 	   615.32	   748.50	  7
  6	 C 	   775.49	   649.43	  6
  7	 A 	   846.53	   489.27	  5
  8	 P 	   943.58	   418.23	  4
  9	 S 	  1030.61	   321.18	  3
 10	 S 	  1117.64	   234.14	  2
 11	 K 	        -  	   147.11	  1  _
+2 charge
		      B   	       Y
  1	 D 	    58.52	        -  	 11
  2	 R 	   136.57	   574.86	 10
  3	 P 	   185.10	   496.81	  9
  4	 F 	   258.63	   448.29	  8
  5	 V 	   308.17	   374.75	  7
  6	 C 	   388.25	   325.22	  6
  7	 A 	   423.77	   245.14	  5
  8	 P 	   472.29	   209.62	  4
  9	 S 	   515.81	   161.09	  3
 10	 S 	   559.33	   117.58	  2
 11	 K 	        -  	    74.06	  1  _
```

**26. IPI00026926 Guanylin**

| Sequence | Charge | M+H+ | XCorr | DeltaCn |
| --- | --- | --- | --- | --- |
| K.LKDLQEPQEPR.V | 3 | 1353.506 | 3.653 | 0.207 |

+1 charge

```
		      B   	       Y
  1	 L 	   114.09	        -  	 11
  2	 K 	   242.19	  1239.63	 10
  3	 D 	   357.21	  1111.54	  9
  4	 L 	   470.30	   996.51	  8
  5	 Q 	   598.36	   883.43	  7
  6	 E 	   727.40	   755.37	  6
  7	 P 	   824.45	   626.33	  5
  8	 Q 	   952.51	   529.27	  4
  9	 E 	  1081.55	   401.21	  3
 10	 P 	  1178.61	   272.17	  2
 11	 R 	        -  	   175.12	  1  _
+2 charge
		      B   	       Y
  1	 L 	    57.55	        -  	 11
  2	 K 	   121.60	   620.32	 10
  3	 D 	   179.11	   556.27	  9
  4	 L 	   235.65	   498.76	  8
  5	 Q 	   299.68	   442.22	  7
  6	 E 	   364.20	   378.19	  6
  7	 P 	   412.73	   313.67	  5
  8	 Q 	   476.76	   265.14	  4
  9	 E 	   541.28	   201.11	  3
 10	 P 	   589.81	   136.59	  2
 11	 R 	        -  	    88.06	  1  _
+3 charge
		      B   	       Y
  1	 L 	    38.70	        -  	 11
  2	 K 	    81.40	   413.88	 10
  3	 D 	   119.74	   371.18	  9
  4	 L 	   157.44	   332.84	  8
  5	 Q 	   200.12	   295.15	  7
  6	 E 	   243.14	   252.46	  6
  7	 P 	   275.49	   209.45	  5
  8	 Q 	   318.17	   177.10	  4
  9	 E 	   361.19	   134.41	  3
 10	 P 	   393.54	    91.40	  2
 11	 R 	        -  	    59.04	  1  _
```

**27. IPI00940960 Epididymal secretory protein E1**

| Sequence | Charge | M+H+ | XCorr | DeltaCn |
| --- | --- | --- | --- | --- |
| K.EVNVSPCPTQPCQLSK.G | 2 | 1845.031 | 3.364 | 0.352 |

+1 charge

```
		      B   	       Y
  1	 E 	   130.05	        -  	 16
  2	 V 	   229.12	  1715.09	 15
  3	 N 	   343.16	  1616.03	 14
  4	 V 	   442.23	  1501.98	 13
  5	 S 	   529.26	  1402.91	 12
  6	 P 	   626.31	  1315.88	 11
  7	 C 	   786.48	  1218.83	 10
  8	 P 	   883.53	  1058.66	  9
  9	 T 	   984.58	   961.61	  8
 10	 Q 	  1112.64	   860.56	  7
 11	 P 	  1209.69	   732.51	  6
 12	 C 	  1369.86	   635.45	  5
 13	 Q 	  1497.92	   475.29	  4
 14	 L 	  1611.00	   347.23	  3
 15	 S 	  1698.03	   234.14	  2
 16	 K 	        -  	   147.11	  1  _
+2 charge
		      B   	       Y
  1	 E 	    65.53	        -  	 16
  2	 V 	   115.06	   858.05	 15
  3	 N 	   172.08	   808.52	 14
  4	 V 	   221.62	   751.50	 13
  5	 S 	   265.13	   701.96	 12
  6	 P 	   313.66	   658.44	 11
  7	 C 	   393.74	   609.92	 10
  8	 P 	   442.27	   529.84	  9
  9	 T 	   492.79	   481.31	  8
 10	 Q 	   556.82	   430.79	  7
 11	 P 	   605.35	   366.76	  6
 12	 C 	   685.43	   318.23	  5
 13	 Q 	   749.46	   238.15	  4
 14	 L 	   806.00	   174.12	  3
 15	 S 	   849.52	   117.58	  2
 16	 K 	        -  	    74.06	  1  _
```

**28. IPI00374563 Agrin**

| Sequence | Charge | M+H+ | XCorr | DeltaCn |
| --- | --- | --- | --- | --- |
| R.VLPEGGAQCECPLGR.E | 2 | 1643.811 | 3.217 | 0.47 |

+1 charge

```
		      B   	       Y
  1	 V 	   100.08	        -  	 15
  2	 L 	   213.16	  1543.97	 14
  3	 P 	   310.21	  1430.88	 13
  4	 E 	   439.26	  1333.83	 12
  5	 G 	   496.28	  1204.79	 11
  6	 G 	   553.30	  1147.77	 10
  7	 A 	   624.34	  1090.75	  9
  8	 Q 	   752.39	  1019.71	  8
  9	 C 	   912.56	   891.65	  7
 10	 E 	  1041.60	   731.49	  6
 11	 C 	  1201.77	   602.44	  5
 12	 P 	  1298.82	   442.28	  4
 13	 L 	  1411.90	   345.22	  3
 14	 G 	  1468.93	   232.14	  2
 15	 R 	        -  	   175.12	  1  _
+2 charge
		      B   	       Y
  1	 V 	    50.54	        -  	 15
  2	 L 	   107.08	   772.49	 14
  3	 P 	   155.61	   715.95	 13
  4	 E 	   220.13	   667.42	 12
  5	 G 	   248.64	   602.90	 11
  6	 G 	   277.15	   574.39	 10
  7	 A 	   312.67	   545.88	  9
  8	 Q 	   376.70	   510.36	  8
  9	 C 	   456.78	   446.33	  7
 10	 E 	   521.30	   366.25	  6
 11	 C 	   601.39	   301.72	  5
 12	 P 	   649.91	   221.64	  4
 13	 L 	   706.46	   173.12	  3
 14	 G 	   734.97	   116.57	  2
 15	 R 	        -  	    88.06	  1  _
```

**29. IPI00061116 Mucin-like protein 1**

| Sequence | Charge | M+H+ | XCorr | DeltaCn |
| --- | --- | --- | --- | --- |
| K.WVGDLPNGR.V | 2 | 1014.119 | 2.831 | 0.327 |

+1 charge

```
		      B   	       Y
  1	 W 	   187.09	        -  	  9
  2	 V 	   286.16	   827.44	  8
  3	 G 	   343.18	   728.37	  7
  4	 D 	   458.20	   671.35	  6
  5	 L 	   571.29	   556.32	  5
  6	 P 	   668.34	   443.24	  4
  7	 N 	   782.38	   346.18	  3
  8	 G 	   839.40	   232.14	  2
  9	 R 	        -  	   175.12	  1  _
+2 charge
		      B   	       Y
  1	 W 	    94.05	        -  	  9
  2	 V 	   143.58	   414.22	  8
  3	 G 	   172.09	   364.69	  7
  4	 D 	   229.61	   336.18	  6
  5	 L 	   286.15	   278.66	  5
  6	 P 	   334.67	   222.12	  4
  7	 N 	   391.70	   173.60	  3
  8	 G 	   420.21	   116.57	  2
  9	 R 	        -  	    88.06	  1  _
```

**30. IPI00289931 MADCAM1 protein**

| Sequence | Charge | M+H+ | XCorr | DeltaCn |
| --- | --- | --- | --- | --- |
| R.GLDTSLGAVQSDTGR.S | 2 | 1477.559 | 4.739 | 0.57 |

+1 charge

```
		      B   	       Y
  1	 G 	    58.03	        -  	 15
  2	 L 	   171.11	  1419.71	 14
  3	 D 	   286.14	  1306.62	 13
  4	 T 	   387.19	  1191.60	 12
  5	 S 	   474.22	  1090.55	 11
  6	 L 	   587.30	  1003.52	 10
  7	 G 	   644.32	   890.43	  9
  8	 A 	   715.36	   833.41	  8
  9	 V 	   814.43	   762.37	  7
 10	 Q 	   942.49	   663.31	  6
 11	 S 	  1029.52	   535.25	  5
 12	 D 	  1144.55	   448.22	  4
 13	 T 	  1245.60	   333.19	  3
 14	 G 	  1302.62	   232.14	  2
 15	 R 	        -  	   175.12	  1  _
+2 charge
		      B   	       Y
  1	 G 	    29.52	        -  	 15
  2	 L 	    86.06	   710.36	 14
  3	 D 	   143.57	   653.82	 13
  4	 T 	   194.10	   596.30	 12
  5	 S 	   237.61	   545.78	 11
  6	 L 	   294.16	   502.26	 10
  7	 G 	   322.67	   445.72	  9
  8	 A 	   358.18	   417.21	  8
  9	 V 	   407.72	   381.69	  7
 10	 Q 	   471.75	   332.16	  6
 11	 S 	   515.26	   268.13	  5
 12	 D 	   572.78	   224.61	  4
 13	 T 	   623.30	   167.10	  3
 14	 G 	   651.81	   116.57	  2
 15	 R 	        -  	    88.06	  1  _
```

**31. IPI00299158 Isoform Alpha of Poliovirus receptor**

| Sequence | Charge | M+H+ | XCorr | DeltaCn |
| --- | --- | --- | --- | --- |
| R.VLAKPQNTAEVQKVQLTGEPVPMAR.C | 3 | 2706.158 | 5.236 | 0.501 |

+1 charge

```
		      B   	       Y
  1	 V 	   100.08	        -  	 25
  2	 L 	   213.16	  2605.41	 24
  3	 A 	   284.20	  2492.33	 23
  4	 K 	   412.29	  2421.29	 22
  5	 P 	   509.34	  2293.20	 21
  6	 Q 	   637.40	  2196.14	 20
  7	 N 	   751.45	  2068.09	 19
  8	 T 	   852.49	  1954.04	 18
  9	 A 	   923.53	  1852.99	 17
 10	 E 	  1052.57	  1781.96	 16
 11	 V 	  1151.64	  1652.92	 15
 12	 Q 	  1279.70	  1553.85	 14
 13	 K 	  1407.80	  1425.79	 13
 14	 V 	  1506.86	  1297.69	 12
 15	 Q 	  1634.92	  1198.62	 11
 16	 L 	  1748.01	  1070.57	 10
 17	 T 	  1849.05	   957.48	  9
 18	 G 	  1906.08	   856.43	  8
 19	 E 	  2035.12	   799.41	  7
 20	 P 	  2132.17	   670.37	  6
 21	 V 	  2231.24	   573.32	  5
 22	 P 	  2328.29	   474.25	  4
 23	 M 	  2459.33	   377.20	  3
 24	 A 	  2530.37	   246.16	  2
 25	 R 	        -  	   175.12	  1  _


+2 charge
		      B   	       Y
  1	 V 	    50.54	        -  	 25
  2	 L 	   107.08	  1303.21	 24
  3	 A 	   142.60	  1246.67	 23
  4	 K 	   206.65	  1211.15	 22
  5	 P 	   255.18	  1147.10	 21
  6	 Q 	   319.21	  1098.58	 20
  7	 N 	   376.23	  1034.55	 19
  8	 T 	   426.75	   977.52	 18
  9	 A 	   462.27	   927.00	 17
 10	 E 	   526.79	   891.48	 16
 11	 V 	   576.32	   826.96	 15
 12	 Q 	   640.35	   777.43	 14
 13	 K 	   704.40	   713.40	 13
 14	 V 	   753.94	   649.35	 12
 15	 Q 	   817.96	   599.82	 11
 16	 L 	   874.51	   535.79	 10
 17	 T 	   925.03	   479.24	  9
 18	 G 	   953.54	   428.72	  8
 19	 E 	  1018.06	   400.21	  7
 20	 P 	  1066.59	   335.69	  6
 21	 V 	  1116.12	   287.16	  5
 22	 P 	  1164.65	   237.63	  4
 23	 M 	  1230.17	   189.10	  3
 24	 A 	  1265.69	   123.58	  2
 25	 R 	        -  	    88.06	  1  _


+3 charge
		      B   	       Y
  1	 V 	    34.03	        -  	 25
  2	 L 	    71.72	   869.14	 24
  3	 A 	    95.40	   831.45	 23
  4	 K 	   138.10	   807.77	 22
  5	 P 	   170.45	   765.07	 21
  6	 Q 	   213.14	   732.72	 20
  7	 N 	   251.15	   690.03	 19
  8	 T 	   284.84	   652.02	 18
  9	 A 	   308.52	   618.34	 17
 10	 E 	   351.53	   594.66	 16
 11	 V 	   384.55	   551.64	 15
 12	 Q 	   427.24	   518.62	 14
 13	 K 	   469.94	   475.93	 13
 14	 V 	   502.96	   433.24	 12
 15	 Q 	   545.65	   400.21	 11
 16	 L 	   583.34	   357.53	 10
 17	 T 	   617.02	   319.83	  9
 18	 G 	   636.03	   286.15	  8
 19	 E 	   679.04	   267.14	  7
 20	 P 	   711.40	   224.13	  6
 21	 V 	   744.42	   191.78	  5
 22	 P 	   776.77	   158.75	  4
 23	 M 	   820.45	   126.40	  3
 24	 A 	   844.13	    82.72	  2
 25	 R 	        -  	    59.04	  1  _
```

**32. IPI00004310 Ly6/PLAUR domain-containing protein 3**

| Sequence | Charge | M+H+ | XCorr | DeltaCn |
| --- | --- | --- | --- | --- |
| R.DGVTGPGFTLSGSCCQGSR.C | 2 | 1944.037 | 2.934 | 0.455 |

+1 charge

```
		      B   	       Y
  1	 D 	   116.03	        -  	 19
  2	 G 	   173.06	  1828.08	 18
  3	 V 	   272.12	  1771.06	 17
  4	 T 	   373.17	  1671.99	 16
  5	 G 	   430.19	  1570.94	 15
  6	 P 	   527.25	  1513.92	 14
  7	 G 	   584.27	  1416.87	 13
  8	 F 	   731.34	  1359.85	 12
  9	 T 	   832.38	  1212.78	 11
 10	 L 	   945.47	  1111.73	 10
 11	 S 	  1032.50	   998.65	  9
 12	 G 	  1089.52	   911.61	  8
 13	 S 	  1176.55	   854.59	  7
 14	 C 	  1336.72	   767.56	  6
 15	 C 	  1496.88	   607.40	  5
 16	 Q 	  1624.94	   447.23	  4
 17	 G 	  1681.96	   319.17	  3
 18	 S 	  1769.00	   262.15	  2
 19	 R 	        -  	   175.12	  1  _
+2 charge
		      B   	       Y
  1	 D 	    58.52	        -  	 19
  2	 G 	    87.03	   914.54	 18
  3	 V 	   136.57	   886.03	 17
  4	 T 	   187.09	   836.50	 16
  5	 G 	   215.60	   785.98	 15
  6	 P 	   264.13	   757.46	 14
  7	 G 	   292.64	   708.94	 13
  8	 F 	   366.17	   680.43	 12
  9	 T 	   416.70	   606.89	 11
 10	 L 	   473.24	   556.37	 10
 11	 S 	   516.75	   499.83	  9
 12	 G 	   545.26	   456.31	  8
 13	 S 	   588.78	   427.80	  7
 14	 C 	   668.86	   384.28	  6
 15	 C 	   748.95	   304.20	  5
 16	 Q 	   812.97	   224.12	  4
 17	 G 	   841.49	   160.09	  3
 18	 S 	   885.00	   131.58	  2
 19	 R 	        -  	    88.06	  1  _
```

**33. IPI00043740 Isoform 1 of Sodium channel and clathrin linker 1**

| Sequence | Charge | M+H+ | XCorr | DeltaCn |
| --- | --- | --- | --- | --- |
| K.LHTQELLSQLEMANEK.V | 2 | 1885.132 | 3.547 | 0.223 |

+1 charge

```
		      B   	       Y
  1	 L 	   114.09	        -  	 16
  2	 H 	   251.15	  1770.87	 15
  3	 T 	   352.20	  1633.81	 14
  4	 Q 	   480.26	  1532.76	 13
  5	 E 	   609.30	  1404.70	 12
  6	 L 	   722.38	  1275.66	 11
  7	 L 	   835.47	  1162.58	 10
  8	 S 	   922.50	  1049.49	  9
  9	 Q 	  1050.56	   962.46	  8
 10	 L 	  1163.64	   834.40	  7
 11	 E 	  1292.68	   721.32	  6
 12	 M 	  1423.72	   592.28	  5
 13	 A 	  1494.76	   461.24	  4
 14	 N 	  1608.81	   390.20	  3
 15	 E 	  1737.85	   276.16	  2
 16	 K 	        -  	   147.11	  1  _
+2 charge
		      B   	       Y
  1	 L 	    57.55	        -  	 16
  2	 H 	   126.08	   885.94	 15
  3	 T 	   176.60	   817.41	 14
  4	 Q 	   240.63	   766.88	 13
  5	 E 	   305.15	   702.86	 12
  6	 L 	   361.70	   638.33	 11
  7	 L 	   418.24	   581.79	 10
  8	 S 	   461.75	   525.25	  9
  9	 Q 	   525.78	   481.73	  8
 10	 L 	   582.32	   417.70	  7
 11	 E 	   646.85	   361.16	  6
 12	 M 	   712.37	   296.64	  5
 13	 A 	   747.88	   231.12	  4
 14	 N 	   804.91	   195.60	  3
 15	 E 	   869.43	   138.58	  2
 16	 K 	        -  	    74.06	  1  _
```

**34. IPI00376403 Isoform 1 of Kunitz-type protease inhibitor 1**

| Sequence | Charge | M+H+ | XCorr | DeltaCn |
| --- | --- | --- | --- | --- |
| R.RHPVCSGTCQPTQFR.C | 3 | 1832.001 | 3.625 | 0.483 |

+1 charge

```
		      B   	       Y
  1	 R 	   157.11	        -  	 15
  2	 H 	   294.17	  1675.02	 14
  3	 P 	   391.22	  1537.96	 13
  4	 V 	   490.29	  1440.91	 12
  5	 C 	   650.45	  1341.84	 11
  6	 S 	   737.49	  1181.67	 10
  7	 G 	   794.51	  1094.64	  9
  8	 T 	   895.55	  1037.62	  8
  9	 C 	  1055.72	   936.57	  7
 10	 Q 	  1183.78	   776.40	  6
 11	 P 	  1280.83	   648.35	  5
 12	 T 	  1381.88	   551.29	  4
 13	 Q 	  1509.94	   450.25	  3
 14	 F 	  1657.01	   322.19	  2
 15	 R 	        -  	   175.12	  1  _
+2 charge
		      B   	       Y
  1	 R 	    79.06	        -  	 15
  2	 H 	   147.59	   838.01	 14
  3	 P 	   196.11	   769.48	 13
  4	 V 	   245.65	   720.96	 12
  5	 C 	   325.73	   671.42	 11
  6	 S 	   369.25	   591.34	 10
  7	 G 	   397.76	   547.82	  9
  8	 T 	   448.28	   519.31	  8
  9	 C 	   528.36	   468.79	  7
 10	 Q 	   592.39	   388.71	  6
 11	 P 	   640.92	   324.68	  5
 12	 T 	   691.44	   276.15	  4
 13	 Q 	   755.47	   225.63	  3
 14	 F 	   829.01	   161.60	  2
 15	 R 	        -  	    88.06	  1  _


+3 charge
		      B   	       Y
  1	 R 	    53.04	        -  	 15
  2	 H 	    98.73	   559.01	 14
  3	 P 	   131.08	   513.32	 13
  4	 V 	   164.10	   480.97	 12
  5	 C 	   217.49	   447.95	 11
  6	 S 	   246.50	   394.56	 10
  7	 G 	   265.51	   365.55	  9
  8	 T 	   299.19	   346.54	  8
  9	 C 	   352.58	   312.86	  7
 10	 Q 	   395.26	   259.47	  6
 11	 P 	   427.62	   216.79	  5
 12	 T 	   461.30	   184.44	  4
 13	 Q 	   503.98	   150.75	  3
 14	 F 	   553.01	   108.07	  2
 15	 R 	        -  	    59.04	  1  _
```

**35. IPI00296777 SPARC-like protein 1**

| Sequence | Charge | M+H+ | XCorr | DeltaCn |
| --- | --- | --- | --- | --- |
| R.VHAVDSCMSFQCK.R | 2 | 1569.752 | 2.809 | 0.555 |

+1 charge

```
		      B   	       Y
  1	 V 	   100.08	        -  	 13
  2	 H 	   237.13	  1469.87	 12
  3	 A 	   308.17	  1332.81	 11
  4	 V 	   407.24	  1261.77	 10
  5	 D 	   522.27	  1162.70	  9
  6	 S 	   609.30	  1047.67	  8
  7	 C 	   769.46	   960.64	  7
  8	 M 	   900.50	   800.48	  6
  9	 S 	   987.54	   669.44	  5
 10	 F 	  1134.61	   582.41	  4
 11	 Q 	  1262.66	   435.34	  3
 12	 C 	  1422.83	   307.28	  2
 13	 K 	        -  	   147.11	  1  _
+2 charge
		      B   	       Y
  1	 V 	    50.54	        -  	 13
  2	 H 	   119.07	   735.44	 12
  3	 A 	   154.59	   666.91	 11
  4	 V 	   204.12	   631.39	 10
  5	 D 	   261.64	   581.85	  9
  6	 S 	   305.15	   524.34	  8
  7	 C 	   385.24	   480.83	  7
  8	 M 	   450.76	   400.74	  6
  9	 S 	   494.27	   335.22	  5
 10	 F 	   567.81	   291.71	  4
 11	 Q 	   631.84	   218.17	  3
 12	 C 	   711.92	   154.14	  2
 13	 K 	        -  	    74.06	  1  _
```

**36. IPI00007221 Plasma serine protease inhibitor**

| Sequence | Charge | M+H+ | XCorr | DeltaCn |
| --- | --- | --- | --- | --- |
| K.MQQVENGLSEKTLR.K | 2 | 1633.852 | 3.293 | 0.228 |

+1 charge

```
		      B   	       Y
  1	 M 	   132.05	        -  	 14
  2	 Q 	   260.11	  1501.80	 13
  3	 Q 	   388.16	  1373.74	 12
  4	 V 	   487.23	  1245.68	 11
  5	 E 	   616.28	  1146.61	 10
  6	 N 	   730.32	  1017.57	  9
  7	 G 	   787.34	   903.53	  8
  8	 L 	   900.42	   846.50	  7
  9	 S 	   987.46	   733.42	  6
 10	 E 	  1116.50	   646.39	  5
 11	 K 	  1244.59	   517.35	  4
 12	 T 	  1345.64	   389.25	  3
 13	 L 	  1458.73	   288.20	  2
 14	 R 	        -  	   175.12	  1  _
+2 charge
		      B   	       Y
  1	 M 	    66.53	        -  	 14
  2	 Q 	   130.56	   751.40	 13
  3	 Q 	   194.59	   687.37	 12
  4	 V 	   244.12	   623.34	 11
  5	 E 	   308.64	   573.81	 10
  6	 N 	   365.66	   509.29	  9
  7	 G 	   394.17	   452.27	  8
  8	 L 	   450.72	   423.76	  7
  9	 S 	   494.23	   367.21	  6
 10	 E 	   558.75	   323.70	  5
 11	 K 	   622.80	   259.18	  4
 12	 T 	   673.32	   195.13	  3
 13	 L 	   729.87	   144.61	  2
 14	 R 	        -  	    88.06	  1  _
```

**37. IPI00020091 Alpha-1-acid glycoprotein 2**

| Sequence | Charge | M+H+ | XCorr | DeltaCn |
| --- | --- | --- | --- | --- |
| R.SDVMYTDWKK.D | 2 | 1273.44 | 2.991 | 0.561 |

+1 charge

```
		      B   	       Y
  1	 S 	    88.04	        -  	 10
  2	 D 	   203.07	  1185.56	  9
  3	 V 	   302.13	  1070.53	  8
  4	 M 	   433.18	   971.47	  7
  5	 Y 	   596.24	   840.43	  6
  6	 T 	   697.29	   677.36	  5
  7	 D 	   812.31	   576.31	  4
  8	 W 	   998.39	   461.29	  3
  9	 K 	  1126.49	   275.21	  2
 10	 K 	        -  	   147.11	  1  _3
+2 charge
		      B   	       Y
  1	 S 	    44.52	        -  	 10
  2	 D 	   102.04	   593.28	  9
  3	 V 	   151.57	   535.77	  8
  4	 M 	   217.09	   486.24	  7
  5	 Y 	   298.62	   420.72	  6
  6	 T 	   349.15	   339.18	  5
  7	 D 	   406.66	   288.66	  4
  8	 W 	   499.70	   231.15	  3
  9	 K 	   563.75	   138.11	  2
 10	 K 	        -  	    74.06	  1  _
```

**38. IPI00442297 Isoform 2 of Neurotrimin**

| Sequence | Charge | M+H+ | XCorr | DeltaCn |
| --- | --- | --- | --- | --- |
| R.EQSGDYECSASNDVAAPVVR.R | 2 | 2155.216 | 3.803 | 0.672 |

+1 charge

```
		      B   	       Y
  1	 E 	   130.05	        -  	 20
  2	 Q 	   258.11	  2025.03	 19
  3	 S 	   345.14	  1896.97	 18
  4	 G 	   402.16	  1809.94	 17
  5	 D 	   517.19	  1752.92	 16
  6	 Y 	   680.25	  1637.89	 15
  7	 E 	   809.29	  1474.83	 14
  8	 C 	   969.46	  1345.79	 13
  9	 S 	  1056.49	  1185.62	 12
 10	 A 	  1127.53	  1098.59	 11
 11	 S 	  1214.56	  1027.55	 10
 12	 N 	  1328.60	   940.52	  9
 13	 D 	  1443.63	   826.48	  8
 14	 V 	  1542.70	   711.45	  7
 15	 A 	  1613.74	   612.38	  6
 16	 A 	  1684.77	   541.35	  5
 17	 P 	  1781.83	   470.31	  4
 18	 V 	  1880.89	   373.26	  3
 19	 V 	  1979.96	   274.19	  2
 20	 R 	        -  	   175.12	  1  _
+2 charge
		      B   	       Y
  1	 E 	    65.53	        -  	 20
  2	 Q 	   129.56	  1013.02	 19
  3	 S 	   173.07	   948.99	 18
  4	 G 	   201.58	   905.47	 17
  5	 D 	   259.10	   876.96	 16
  6	 Y 	   340.63	   819.45	 15
  7	 E 	   405.15	   737.92	 14
  8	 C 	   485.23	   673.40	 13
  9	 S 	   528.75	   593.31	 12
 10	 A 	   564.27	   549.80	 11
 11	 S 	   607.78	   514.28	 10
 12	 N 	   664.81	   470.76	  9
 13	 D 	   722.32	   413.74	  8
 14	 V 	   771.85	   356.23	  7
 15	 A 	   807.37	   306.70	  6
 16	 A 	   842.89	   271.18	  5
 17	 P 	   891.42	   235.66	  4
 18	 V 	   940.95	   187.13	  3
 19	 V 	   990.49	   137.60	  2
 20	 R 	        -  	    88.06	  1  _
```

**39. IPI00029739 Isoform 1 of Complement factor H**

| Sequence | Charge | M+H+ | XCorr | DeltaCn |
| --- | --- | --- | --- | --- |
| K.SIDVACHPGYALPK.A | 2 | 1528.728 | 3.387 | 0.369 |

+1 charge

```
		      B   	       Y
  1	 S 	    88.04	        -  	 14
  2	 I 	   201.12	  1440.86	 13
  3	 D 	   316.15	  1327.78	 12
  4	 V 	   415.22	  1212.75	 11
  5	 A 	   486.26	  1113.69	 10
  6	 C 	   646.42	  1042.65	  9
  7	 H 	   783.48	   882.48	  8
  8	 P 	   880.53	   745.42	  7
  9	 G 	   937.55	   648.37	  6
 10	 Y 	  1100.62	   591.35	  5
 11	 A 	  1171.65	   428.29	  4
 12	 L 	  1284.74	   357.25	  3
 13	 P 	  1381.79	   244.17	  2
 14	 K 	        -  	   147.11	  1  _
+2 charge
		      B   	       Y
  1	 S 	    44.52	        -  	 14
  2	 I 	   101.07	   720.94	 13
  3	 D 	   158.58	   664.39	 12
  4	 V 	   208.11	   606.88	 11
  5	 A 	   243.63	   557.35	 10
  6	 C 	   323.71	   521.83	  9
  7	 H 	   392.24	   441.75	  8
  8	 P 	   440.77	   373.22	  7
  9	 G 	   469.28	   324.69	  6
 10	 Y 	   550.81	   296.18	  5
 11	 A 	   586.33	   214.65	  4
 12	 L 	   642.87	   179.13	  3
 13	 P 	   691.40	   122.59	  2
 14	 K 	        -  	    74.06	  1  _
```

**40. IPI00011140 Protein NOV homolog**

| Sequence | Charge | M+H+ | XCorr | DeltaCn |
| --- | --- | --- | --- | --- |
| R.AVLDGCSCCLVCAR.Q | 2 | 1641.823 | 3.815 | 0.496 |

+1 charge

```
		      B   	       Y
  1	 A 	    72.04	        -  	 14
  2	 V 	   171.11	  1570.20	 13
  3	 L 	   284.20	  1471.13	 12
  4	 D 	   399.22	  1358.05	 11
  5	 G 	   456.25	  1243.02	 10
  6	 C 	   616.41	  1186.00	  9
  7	 S 	   703.44	  1025.84	  8
  8	 C 	   863.61	   938.80	  7
  9	 C 	  1023.77	   778.64	  6
 10	 L 	  1136.86	   618.47	  5
 11	 V 	  1235.93	   505.39	  4
 12	 C 	  1396.09	   406.32	  3
 13	 A 	  1467.13	   246.16	  2
 14	 R 	        -  	   175.12	  1  _
+2 charge
		      B   	       Y
  1	 A 	    36.53	        -  	 14
  2	 V 	    86.06	   785.60	 13
  3	 L 	   142.60	   736.07	 12
  4	 D 	   200.12	   679.53	 11
  5	 G 	   228.63	   622.02	 10
  6	 C 	   308.71	   593.50	  9
  7	 S 	   352.22	   513.42	  8
  8	 C 	   432.31	   469.91	  7
  9	 C 	   512.39	   389.82	  6
 10	 L 	   568.93	   309.74	  5
 11	 V 	   618.47	   253.20	  4
 12	 C 	   698.55	   203.66	  3
 13	 A 	   734.07	   123.58	  2
 14	 R 	        -  	    88.06	  1  _
```

**41. IPI00180707 Isoform 1 of FRAS1-related extracellular matrix protein 2**

| Sequence | Charge | M+H+ | XCorr | DeltaCn |
| --- | --- | --- | --- | --- |
| R.VPGVVGAEPFSAK.L | 2 | 1258.448 | 3.035 | 0.397 |

+1 charge

```
		      B   	       Y
  1	 V 	   100.08	        -  	 13
  2	 P 	   197.13	  1158.62	 12
  3	 G 	   254.15	  1061.56	 11
  4	 V 	   353.22	  1004.54	 10
  5	 V 	   452.29	   905.47	  9
  6	 G 	   509.31	   806.40	  8
  7	 A 	   580.35	   749.38	  7
  8	 E 	   709.39	   678.35	  6
  9	 P 	   806.44	   549.30	  5
 10	 F 	   953.51	   452.25	  4
 11	 S 	  1040.54	   305.18	  3
 12	 A 	  1111.58	   218.15	  2
 13	 K 	        -  	   147.11	  1  _
+2 charge
		      B   	       Y
  1	 V 	    50.54	        -  	 13
  2	 P 	    99.07	   579.81	 12
  3	 G 	   127.58	   531.28	 11
  4	 V 	   177.11	   502.77	 10
  5	 V 	   226.65	   453.24	  9
  6	 G 	   255.16	   403.71	  8
  7	 A 	   290.68	   375.20	  7
  8	 E 	   355.20	   339.68	  6
  9	 P 	   403.72	   275.16	  5
 10	 F 	   477.26	   226.63	  4
 11	 S 	   520.77	   153.09	  3
 12	 A 	   556.29	   109.58	  2
 13	 K 	        -  	    74.06	  1  _
```

**42．IPI00295414 Collagen alpha-1(XV) chain**

| Sequence | Charge | M+H+ | XCorr | DeltaCn |
| --- | --- | --- | --- | --- |
| R.FTGSLQQLTVHPDPR.T | 2 | 1696.888 | 2.871 | 0.419 |

+1 charge

```
		      B   	       Y
  1	 F 	   148.08	        -  	 15
  2	 T 	   249.12	  1548.81	 14
  3	 G 	   306.14	  1447.77	 13
  4	 S 	   393.18	  1390.74	 12
  5	 L 	   506.26	  1303.71	 11
  6	 Q 	   634.32	  1190.63	 10
  7	 Q 	   762.38	  1062.57	  9
  8	 L 	   875.46	   934.51	  8
  9	 T 	   976.51	   821.43	  7
 10	 V 	  1075.58	   720.38	  6
 11	 H 	  1212.64	   621.31	  5
 12	 P 	  1309.69	   484.25	  4
 13	 D 	  1424.72	   387.20	  3
 14	 P 	  1521.77	   272.17	  2
 15	 R 	        -  	   175.12	  1  _
+2 charge
		      B   	       Y
  1	 F 	    74.54	        -  	 15
  2	 T 	   125.07	   774.91	 14
  3	 G 	   153.58	   724.39	 13
  4	 S 	   197.09	   695.88	 12
  5	 L 	   253.63	   652.36	 11
  6	 Q 	   317.66	   595.82	 10
  7	 Q 	   381.69	   531.79	  9
  8	 L 	   438.23	   467.76	  8
  9	 T 	   488.76	   411.22	  7
 10	 V 	   538.29	   360.69	  6
 11	 H 	   606.82	   311.16	  5
 12	 P 	   655.35	   242.63	  4
 13	 D 	   712.86	   194.10	  3
 14	 P 	   761.39	   136.59	  2
 15	 R 	        -  	    88.06	  1  _
```

**43. IPI00412298 Isoform 1 of Serine/threonine-protein kinase ATR**

| Sequence | Charge | M+H+ | XCorr | DeltaCn |
| --- | --- | --- | --- | --- |
| R.NVMGHAVEWPVVMSR.F | 2 | 1713.021 | 2.835 | 0.33 |

+1 charge

```
		      B   	       Y
  1	 N 	   115.05	        -  	 15
  2	 V 	   214.12	  1597.80	 14
  3	 M 	   345.16	  1498.73	 13
  4	 G 	   402.18	  1367.69	 12
  5	 H 	   539.24	  1310.67	 11
  6	 A 	   610.28	  1173.61	 10
  7	 V 	   709.35	  1102.57	  9
  8	 E 	   838.39	  1003.50	  8
  9	 W 	  1024.47	   874.46	  7
 10	 P 	  1121.52	   688.38	  6
 11	 V 	  1220.59	   591.33	  5
 12	 V 	  1319.66	   492.26	  4
 13	 M 	  1450.70	   393.19	  3
 14	 S 	  1537.73	   262.15	  2
 15	 R 	        -  	   175.12	  1  _
+2 charge
		      B   	       Y
  1	 N 	    58.03	        -  	 15
  2	 V 	   107.56	   799.40	 14
  3	 M 	   173.08	   749.87	 13
  4	 G 	   201.59	   684.35	 12
  5	 H 	   270.12	   655.84	 11
  6	 A 	   305.64	   587.31	 10
  7	 V 	   355.18	   551.79	  9
  8	 E 	   419.70	   502.26	  8
  9	 W 	   512.74	   437.73	  7
 10	 P 	   561.26	   344.69	  6
 11	 V 	   610.80	   296.17	  5
 12	 V 	   660.33	   246.63	  4
 13	 M 	   725.85	   197.10	  3
 14	 S 	   769.37	   131.58	  2
 15	 R 	        -  	    88.06	  1  _
```

**44. IPI00301143 Isoform 1 of Peptidase inhibitor 16**

| Sequence | Charge | M+H+ | XCorr | DeltaCn |
| --- | --- | --- | --- | --- |
| R.LMVELHNLYR.A | 2 | 1288.544 | 3.333 | 0.338 |

+1 charge

```
		      B   	       Y
  1	 L 	   114.09	        -  	 10
  2	 M 	   245.13	  1174.60	  9
  3	 V 	   344.20	  1043.56	  8
  4	 E 	   473.24	   944.49	  7
  5	 L 	   586.33	   815.45	  6
  6	 H 	   723.39	   702.37	  5
  7	 N 	   837.43	   565.31	  4
  8	 L 	   950.51	   451.27	  3
  9	 Y 	  1113.58	   338.18	  2
 10	 R 	        -  	   175.12	  1  _
+2 charge
		      B   	       Y
  1	 L 	    57.55	        -  	 10
  2	 M 	   123.07	   587.81	  9
  3	 V 	   172.60	   522.29	  8
  4	 E 	   237.13	   472.75	  7
  5	 L 	   293.67	   408.23	  6
  6	 H 	   362.20	   351.69	  5
  7	 N 	   419.22	   283.16	  4
  8	 L 	   475.76	   226.14	  3
  9	 Y 	   557.29	   169.59	  2
 10	 R 	        -  	    88.06	  1  _
```

**45. IPI00385252 Ig kappa chain V-III region GOL**

| Sequence | Charge | M+H+ | XCorr | DeltaCn |
| --- | --- | --- | --- | --- |
| - .EIVLTQSPGTLSLSPGER.A | 2 | 1885.108 | 4.954 | 0.533 |

+1 charge

```
		      B   	       Y
  1	 E 	   130.05	        -  	 18
  2	 I 	   243.13	  1754.96	 17
  3	 V 	   342.20	  1641.88	 16
  4	 L 	   455.29	  1542.81	 15
  5	 T 	   556.33	  1429.73	 14
  6	 Q 	   684.39	  1328.68	 13
  7	 S 	   771.42	  1200.62	 12
  8	 P 	   868.48	  1113.59	 11
  9	 G 	   925.50	  1016.54	 10
 10	 T 	  1026.55	   959.52	  9
 11	 L 	  1139.63	   858.47	  8
 12	 S 	  1226.66	   745.38	  7
 13	 L 	  1339.75	   658.35	  6
 14	 S 	  1426.78	   545.27	  5
 15	 P 	  1523.83	   458.24	  4
 16	 G 	  1580.85	   361.18	  3
 17	 E 	  1709.90	   304.16	  2
 18	 R 	        -  	   175.12	  1  _
+2 charge
		      B   	       Y
  1	 E 	    65.53	        -  	 18
  2	 I 	   122.07	   877.99	 17
  3	 V 	   171.60	   821.44	 16
  4	 L 	   228.15	   771.91	 15
  5	 T 	   278.67	   715.37	 14
  6	 Q 	   342.70	   664.84	 13
  7	 S 	   386.22	   600.81	 12
  8	 P 	   434.74	   557.30	 11
  9	 G 	   463.25	   508.77	 10
 10	 T 	   513.78	   480.26	  9
 11	 L 	   570.32	   429.74	  8
 12	 S 	   613.83	   373.20	  7
 13	 L 	   670.38	   329.68	  6
 14	 S 	   713.89	   273.14	  5
 15	 P 	   762.42	   229.62	  4
 16	 G 	   790.93	   181.10	  3
 17	 E 	   855.45	   152.58	  2
 18	 R 	        -  	    88.06	  1  _
```

**46. IPI00170635 Secreted and transmembrane protein 1**

| Sequence | Charge | M+H+ | XCorr | DeltaCn |
| --- | --- | --- | --- | --- |
| R.AHGQESAIFNEVAPGYFSR.D | 2 | 2081.233 | 5.116 | 0.647 |

+1 charge

```
		      B   	       Y
  1	 A 	    72.04	        -  	 19
  2	 H 	   209.10	  2008.95	 18
  3	 G 	   266.12	  1871.89	 17
  4	 Q 	   394.18	  1814.87	 16
  5	 E 	   523.23	  1686.81	 15
  6	 S 	   610.26	  1557.77	 14
  7	 A 	   681.30	  1470.74	 13
  8	 I 	   794.38	  1399.70	 12
  9	 F 	   941.45	  1286.62	 11
 10	 N 	  1055.49	  1139.55	 10
 11	 E 	  1184.53	  1025.51	  9
 12	 V 	  1283.60	   896.46	  8
 13	 A 	  1354.64	   797.39	  7
 14	 P 	  1451.69	   726.36	  6
 15	 G 	  1508.71	   629.30	  5
 16	 Y 	  1671.78	   572.28	  4
 17	 F 	  1818.84	   409.22	  3
 18	 S 	  1905.88	   262.15	  2
 19	 R 	        -  	   175.12	  1  _
+2 charge
		      B   	       Y
  1	 A 	    36.53	        -  	 19
  2	 H 	   105.06	  1004.98	 18
  3	 G 	   133.57	   936.45	 17
  4	 Q 	   197.60	   907.94	 16
  5	 E 	   262.12	   843.91	 15
  6	 S 	   305.63	   779.39	 14
  7	 A 	   341.15	   735.87	 13
  8	 I 	   397.69	   700.35	 12
  9	 F 	   471.23	   643.81	 11
 10	 N 	   528.25	   570.28	 10
 11	 E 	   592.77	   513.26	  9
 12	 V 	   642.30	   448.73	  8
 13	 A 	   677.82	   399.20	  7
 14	 P 	   726.35	   363.68	  6
 15	 G 	   754.86	   315.16	  5
 16	 Y 	   836.39	   286.64	  4
 17	 F 	   909.93	   205.11	  3
 18	 S 	   953.44	   131.58	  2
 19	 R 	        -  	    88.06	  1  _
```

**47. IPI00025753 Desmoglein-1**

| Sequence | Charge | M+H+ | XCorr | DeltaCn |
| --- | --- | --- | --- | --- |
| R.YVMGNNPADLLAVDSR.T | 2 | 1735.943 | 3.229 | 0.5 |

+1 charge

```
		      B   	       Y
  1	 Y 	   164.07	        -  	 16
  2	 V 	   263.14	  1571.78	 15
  3	 M 	   394.18	  1472.72	 14
  4	 G 	   451.20	  1341.68	 13
  5	 N 	   565.24	  1284.65	 12
  6	 N 	   679.29	  1170.61	 11
  7	 P 	   776.34	  1056.57	 10
  8	 A 	   847.38	   959.52	  9
  9	 D 	   962.40	   888.48	  8
 10	 L 	  1075.49	   773.45	  7
 11	 L 	  1188.57	   660.37	  6
 12	 A 	  1259.61	   547.28	  5
 13	 V 	  1358.68	   476.25	  4
 14	 D 	  1473.70	   377.18	  3
 15	 S 	  1560.74	   262.15	  2
 16	 R 	        -  	   175.12	  1  _
+2 charge
		      B   	       Y
  1	 Y 	    82.54	        -  	 16
  2	 V 	   132.07	   786.40	 15
  3	 M 	   197.59	   736.86	 14
  4	 G 	   226.10	   671.34	 13
  5	 N 	   283.13	   642.83	 12
  6	 N 	   340.15	   585.81	 11
  7	 P 	   388.67	   528.79	 10
  8	 A 	   424.19	   480.26	  9
  9	 D 	   481.71	   444.74	  8
 10	 L 	   538.25	   387.23	  7
 11	 L 	   594.79	   330.69	  6
 12	 A 	   630.31	   274.15	  5
 13	 V 	   679.84	   238.63	  4
 14	 D 	   737.36	   189.09	  3
 15	 S 	   780.87	   131.58	  2
 16	 R 	        -  	    88.06	  1  _
```

**48. IPI00783313 Glycogen phosphorylase, liver form**

| Sequence | Charge | M+H+ | XCorr | DeltaCn |
| --- | --- | --- | --- | --- |
| R.RWLLLCNPGLAELIAEKIGEDYVK.D | 3 | 2802.254 | 3.63 | 0.269 |

+1 charge

```
		      B   	       Y
  1	 R 	   157.11	        -  	 24
  2	 W 	   343.19	  2644.54	 23
  3	 L 	   456.27	  2458.46	 22
  4	 L 	   569.36	  2345.38	 21
  5	 L 	   682.44	  2232.29	 20
  6	 C 	   842.61	  2119.21	 19
  7	 N 	   956.65	  1959.04	 18
  8	 P 	  1053.70	  1845.00	 17
  9	 G 	  1110.72	  1747.95	 16
 10	 L 	  1223.81	  1690.93	 15
 11	 A 	  1294.84	  1577.84	 14
 12	 E 	  1423.89	  1506.81	 13
 13	 L 	  1536.97	  1377.76	 12
 14	 I 	  1650.05	  1264.68	 11
 15	 A 	  1721.09	  1151.59	 10
 16	 E 	  1850.13	  1080.56	  9
 17	 K 	  1978.23	   951.51	  8
 18	 I 	  2091.31	   823.42	  7
 19	 G 	  2148.33	   710.34	  6
 20	 E 	  2277.38	   653.31	  5
 21	 D 	  2392.40	   524.27	  4
 22	 Y 	  2555.47	   409.24	  3
 23	 V 	  2654.54	   246.18	  2
 24	 K 	        -  	   147.11	  1  _


+2 charge
		      B   	       Y
  1	 R 	    79.06	        -  	 24
  2	 W 	   172.10	  1322.77	 23
  3	 L 	   228.64	  1229.73	 22
  4	 L 	   285.18	  1173.19	 21
  5	 L 	   341.72	  1116.65	 20
  6	 C 	   421.81	  1060.11	 19
  7	 N 	   478.83	   980.03	 18
  8	 P 	   527.35	   923.00	 17
  9	 G 	   555.86	   874.48	 16
 10	 L 	   612.41	   845.97	 15
 11	 A 	   647.93	   789.42	 14
 12	 E 	   712.45	   753.91	 13
 13	 L 	   768.99	   689.38	 12
 14	 I 	   825.53	   632.84	 11
 15	 A 	   861.05	   576.30	 10
 16	 E 	   925.57	   540.78	  9
 17	 K 	   989.62	   476.26	  8
 18	 I 	  1046.16	   412.21	  7
 19	 G 	  1074.67	   355.67	  6
 20	 E 	  1139.19	   327.16	  5
 21	 D 	  1196.71	   262.64	  4
 22	 Y 	  1278.24	   205.13	  3
 23	 V 	  1327.77	   123.59	  2
 24	 K 	        -  	    74.06	  1  _


+3 charge
		      B   	       Y
  1	 R 	    53.04	        -  	 24
  2	 W 	   115.07	   882.18	 23
  3	 L 	   152.76	   820.16	 22
  4	 L 	   190.46	   782.46	 21
  5	 L 	   228.15	   744.77	 20
  6	 C 	   281.54	   707.07	 19
  7	 N 	   319.55	   653.69	 18
  8	 P 	   351.91	   615.67	 17
  9	 G 	   370.91	   583.32	 16
 10	 L 	   408.61	   564.31	 15
 11	 A 	   432.29	   526.62	 14
 12	 E 	   475.30	   502.94	 13
 13	 L 	   512.99	   459.93	 12
 14	 I 	   550.69	   422.23	 11
 15	 A 	   574.37	   384.54	 10
 16	 E 	   617.38	   360.86	  9
 17	 K 	   660.08	   317.84	  8
 18	 I 	   697.78	   275.14	  7
 19	 G 	   716.78	   237.45	  6
 20	 E 	   759.80	   218.44	  5
 21	 D 	   798.14	   175.43	  4
 22	 Y 	   852.49	   137.09	  3
 23	 V 	   885.52	    82.73	  2
 24	 K 	        -  	    49.71	  1  _
```

**49. IPI00034319 Isoform A of Protein CutA**

| Sequence | Charge | M+H+ | XCorr | DeltaCn |
| --- | --- | --- | --- | --- |
| K.TQSSLVPALTDFVR.S | 2 | 1534.739 | 2.951 | 0.423 |

+1 charge

```
		      B   	       Y
  1	 T 	   102.05	        -  	 14
  2	 Q 	   230.11	  1432.78	 13
  3	 S 	   317.15	  1304.72	 12
  4	 S 	   404.18	  1217.69	 11
  5	 L 	   517.26	  1130.66	 10
  6	 V 	   616.33	  1017.57	  9
  7	 P 	   713.38	   918.50	  8
  8	 A 	   784.42	   821.45	  7
  9	 L 	   897.50	   750.41	  6
 10	 T 	   998.55	   637.33	  5
 11	 D 	  1113.58	   536.28	  4
 12	 F 	  1260.65	   421.26	  3
 13	 V 	  1359.72	   274.19	  2
 14	 R 	        -  	   175.12	  1  _
+2 charge
		      B   	       Y
  1	 T 	    51.53	        -  	 14
  2	 Q 	   115.56	   716.89	 13
  3	 S 	   159.08	   652.86	 12
  4	 S 	   202.59	   609.35	 11
  5	 L 	   259.13	   565.83	 10
  6	 V 	   308.67	   509.29	  9
  7	 P 	   357.20	   459.76	  8
  8	 A 	   392.71	   411.23	  7
  9	 L 	   449.26	   375.71	  6
 10	 T 	   499.78	   319.17	  5
 11	 D 	   557.29	   268.64	  4
 12	 F 	   630.83	   211.13	  3
 13	 V 	   680.36	   137.60	  2
 14	 R 	        -  	    88.06	  1  _
```

**50. IPI00000914 Isoform 1 of Calcitonin**

| Sequence | Charge | M+H+ | XCorr | DeltaCn |
| --- | --- | --- | --- | --- |
| R.LLLAALVQDYVQMK.A | 2 | 1605.967 | 4.917 | 0.383 |

+1 charge

```
		      B   	       Y
  1	 L 	   114.09	        -  	 14
  2	 L 	   227.18	  1491.82	 13
  3	 L 	   340.26	  1378.74	 12
  4	 A 	   411.30	  1265.66	 11
  5	 A 	   482.33	  1194.62	 10
  6	 L 	   595.42	  1123.58	  9
  7	 V 	   694.49	  1010.50	  8
  8	 Q 	   822.54	   911.43	  7
  9	 D 	   937.57	   783.37	  6
 10	 Y 	  1100.64	   668.34	  5
 11	 V 	  1199.70	   505.28	  4
 12	 Q 	  1327.76	   406.21	  3
 13	 M 	  1458.80	   278.15	  2
 14	 K 	        -  	   147.11	  1  _
+2 charge
		      B   	       Y
  1	 L 	    57.55	        -  	 14
  2	 L 	   114.09	   746.42	 13
  3	 L 	   170.63	   689.87	 12
  4	 A 	   206.15	   633.33	 11
  5	 A 	   241.67	   597.81	 10
  6	 L 	   298.21	   562.29	  9
  7	 V 	   347.75	   505.75	  8
  8	 Q 	   411.78	   456.22	  7
  9	 D 	   469.29	   392.19	  6
 10	 Y 	   550.82	   334.68	  5
 11	 V 	   600.36	   253.14	  4
 12	 Q 	   664.38	   203.61	  3
 13	 M 	   729.90	   139.58	  2
 14	 K 	        -  	    74.06	  1  _
```

**51. IPI00009027 Lithostathine-1-alpha**

| Sequence | Charge | M+H+ | XCorr | DeltaCn |
| --- | --- | --- | --- | --- |
| R.ISCPEGTNAYR.S | 2 | 1268.352 | 3.202 | 0.491 |

+1 charge

```
		      B   	       Y
  1	 I 	   114.09	        -  	 11
  2	 S 	   201.12	  1154.62	 10
  3	 C 	   361.29	  1067.59	  9
  4	 P 	   458.34	   907.43	  8
  5	 E 	   587.38	   810.37	  7
  6	 G 	   644.41	   681.33	  6
  7	 T 	   745.45	   624.31	  5
  8	 N 	   859.50	   523.26	  4
  9	 A 	   930.53	   409.22	  3
 10	 Y 	  1093.60	   338.18	  2
 11	 R 	        -  	   175.12	  1  _
+2 charge
		      B   	       Y
  1	 I 	    57.55	        -  	 11
  2	 S 	   101.07	   577.82	 10
  3	 C 	   181.15	   534.30	  9
  4	 P 	   229.67	   454.22	  8
  5	 E 	   294.20	   405.69	  7
  6	 G 	   322.71	   341.17	  6
  7	 T 	   373.23	   312.66	  5
  8	 N 	   430.25	   262.13	  4
  9	 A 	   465.77	   205.11	  3
 10	 Y 	   547.30	   169.59	  2
 11	 R 	        -  	    88.06	  1  _
```

**52. IPI00021275 Isoform 1 of Ephrin type-B receptor 2**

| Sequence | Charge | M+H+ | XCorr | DeltaCn |
| --- | --- | --- | --- | --- |
| R.GCPSGTFKANQGDEACTHCPINSR.T | 3 | 2665.795 | 3.362 | 0.441 |

+1 charge

```
		      B   	       Y
  1	 G 	    58.03	        -  	 24
  2	 C 	   218.19	  2607.52	 23
  3	 P 	   315.25	  2447.35	 22
  4	 S 	   402.28	  2350.30	 21
  5	 G 	   459.30	  2263.27	 20
  6	 T 	   560.35	  2206.25	 19
  7	 F 	   707.42	  2105.20	 18
  8	 K 	   835.51	  1958.13	 17
  9	 A 	   906.55	  1830.03	 16
 10	 N 	  1020.59	  1759.00	 15
 11	 Q 	  1148.65	  1644.95	 14
 12	 G 	  1205.67	  1516.90	 13
 13	 D 	  1320.70	  1459.87	 12
 14	 E 	  1449.74	  1344.85	 11
 15	 A 	  1520.78	  1215.80	 10
 16	 C 	  1680.94	  1144.77	  9
 17	 T 	  1781.99	   984.60	  8
 18	 H 	  1919.05	   883.55	  7
 19	 C 	  2079.22	   746.50	  6
 20	 P 	  2176.27	   586.33	  5
 21	 I 	  2289.35	   489.28	  4
 22	 N 	  2403.39	   376.19	  3
 23	 S 	  2490.43	   262.15	  2
 24	 R 	        -  	   175.12	  1  _


+2 charge
		      B   	       Y
  1	 G 	    29.52	        -  	 24
  2	 C 	   109.60	  1304.26	 23
  3	 P 	   158.13	  1224.18	 22
  4	 S 	   201.64	  1175.65	 21
  5	 G 	   230.15	  1132.14	 20
  6	 T 	   280.68	  1103.63	 19
  7	 F 	   354.21	  1053.10	 18
  8	 K 	   418.26	   979.57	 17
  9	 A 	   453.78	   915.52	 16
 10	 N 	   510.80	   880.00	 15
 11	 Q 	   574.83	   822.98	 14
 12	 G 	   603.34	   758.95	 13
 13	 D 	   660.85	   730.44	 12
 14	 E 	   725.37	   672.93	 11
 15	 A 	   760.89	   608.41	 10
 16	 C 	   840.98	   572.89	  9
 17	 T 	   891.50	   492.80	  8
 18	 H 	   960.03	   442.28	  7
 19	 C 	  1040.11	   373.75	  6
 20	 P 	  1088.64	   293.67	  5
 21	 I 	  1145.18	   245.14	  4
 22	 N 	  1202.20	   188.60	  3
 23	 S 	  1245.72	   131.58	  2
 24	 R 	        -  	    88.06	  1  _


+3 charge
		      B   	       Y
  1	 G 	    20.01	        -  	 24
  2	 C 	    73.40	   869.84	 23
  3	 P 	   105.75	   816.46	 22
  4	 S 	   134.76	   784.10	 21
  5	 G 	   153.77	   755.09	 20
  6	 T 	   187.45	   736.09	 19
  7	 F 	   236.48	   702.40	 18
  8	 K 	   279.18	   653.38	 17
  9	 A 	   302.85	   610.68	 16
 10	 N 	   340.87	   587.00	 15
 11	 Q 	   383.55	   548.99	 14
 12	 G 	   402.56	   506.30	 13
 13	 D 	   440.90	   487.30	 12
 14	 E 	   483.92	   448.95	 11
 15	 A 	   507.60	   405.94	 10
 16	 C 	   560.99	   382.26	  9
 17	 T 	   594.67	   328.87	  8
 18	 H 	   640.35	   295.19	  7
 19	 C 	   693.74	   249.50	  6
 20	 P 	   726.09	   196.12	  5
 21	 I 	   763.79	   163.76	  4
 22	 N 	   801.80	   126.07	  3
 23	 S 	   830.81	    88.06	  2
 24	 R 	        -  	    59.04	  1  _
```

**53. IPI00029751 Isoform 1 of Contactin-1**

| Sequence | Charge | M+H+ | XCorr | DeltaCn |
| --- | --- | --- | --- | --- |
| R.IKTDGAAPNVAPSDVGGGGGR.N | 2 | 1897.039 | 3.294 | 0.474 |

+1 charge

```
		      B   	       Y
  1	 I 	   114.09	        -  	 21
  2	 K 	   242.19	  1782.87	 20
  3	 T 	   343.23	  1654.78	 19
  4	 D 	   458.26	  1553.73	 18
  5	 G 	   515.28	  1438.70	 17
  6	 A 	   586.32	  1381.68	 16
  7	 A 	   657.36	  1310.64	 15
  8	 P 	   754.41	  1239.61	 14
  9	 N 	   868.45	  1142.55	 13
 10	 V 	   967.52	  1028.51	 12
 11	 A 	  1038.56	   929.44	 11
 12	 P 	  1135.61	   858.41	 10
 13	 S 	  1222.64	   761.35	  9
 14	 D 	  1337.67	   674.32	  8
 15	 V 	  1436.74	   559.29	  7
 16	 G 	  1493.76	   460.23	  6
 17	 G 	  1550.78	   403.20	  5
 18	 G 	  1607.80	   346.18	  4
 19	 G 	  1664.82	   289.16	  3
 20	 G 	  1721.85	   232.14	  2
 21	 R 	        -  	   175.12	  1  _
+2 charge
		      B   	       Y
  1	 I 	    57.55	        -  	 21
  2	 K 	   121.60	   891.94	 20
  3	 T 	   172.12	   827.89	 19
  4	 D 	   229.63	   777.37	 18
  5	 G 	   258.14	   719.86	 17
  6	 A 	   293.66	   691.34	 16
  7	 A 	   329.18	   655.83	 15
  8	 P 	   377.71	   620.31	 14
  9	 N 	   434.73	   571.78	 13
 10	 V 	   484.26	   514.76	 12
 11	 A 	   519.78	   465.23	 11
 12	 P 	   568.31	   429.71	 10
 13	 S 	   611.82	   381.18	  9
 14	 D 	   669.34	   337.66	  8
 15	 V 	   718.87	   280.15	  7
 16	 G 	   747.38	   230.62	  6
 17	 G 	   775.89	   202.11	  5
 18	 G 	   804.40	   173.60	  4
 19	 G 	   832.92	   145.08	  3
 20	 G 	   861.43	   116.57	  2
 21	 R 	        -  	    88.06	  1  _
```

**54. IPI00021970 TNFRSF10C protein**

| Sequence | Charge | M+H+ | XCorr | DeltaCn |
| --- | --- | --- | --- | --- |
| R.DTVCQCKEGTFRNENSPEMCR.K | 3 | 2619.788 | 3.61 | 0.44 |

+1 charge

```
		      B   	       Y
  1	 D 	   116.03	        -  	 21
  2	 T 	   217.08	  2503.46	 20
  3	 V 	   316.15	  2402.41	 19
  4	 C 	   476.32	  2303.35	 18
  5	 Q 	   604.37	  2143.18	 17
  6	 C 	   764.54	  2015.12	 16
  7	 K 	   892.63	  1854.96	 15
  8	 E 	  1021.68	  1726.86	 14
  9	 G 	  1078.70	  1597.82	 13
 10	 T 	  1179.75	  1540.80	 12
 11	 F 	  1326.81	  1439.75	 11
 12	 R 	  1482.92	  1292.68	 10
 13	 N 	  1596.96	  1136.58	  9
 14	 E 	  1726.00	  1022.54	  8
 15	 N 	  1840.04	   893.49	  7
 16	 S 	  1927.08	   779.45	  6
 17	 P 	  2024.13	   692.42	  5
 18	 E 	  2153.17	   595.37	  4
 19	 M 	  2284.21	   466.32	  3
 20	 C 	  2444.38	   335.28	  2
 21	 R 	        -  	   175.12	  1  _
+2 charge
		      B   	       Y
  1	 D 	    58.52	        -  	 21
  2	 T 	   109.04	  1252.23	 20
  3	 V 	   158.58	  1201.71	 19
  4	 C 	   238.66	  1152.18	 18
  5	 Q 	   302.69	  1072.09	 17
  6	 C 	   382.77	  1008.06	 16
  7	 K 	   446.82	   927.98	 15
  8	 E 	   511.34	   863.93	 14
  9	 G 	   539.85	   799.41	 13
 10	 T 	   590.38	   770.90	 12
 11	 F 	   663.91	   720.38	 11
 12	 R 	   741.96	   646.84	 10
 13	 N 	   798.98	   568.79	  9
 14	 E 	   863.50	   511.77	  8
 15	 N 	   920.53	   447.25	  7
 16	 S 	   964.04	   390.23	  6
 17	 P 	  1012.57	   346.71	  5
 18	 E 	  1077.09	   298.19	  4
 19	 M 	  1142.61	   233.67	  3
 20	 C 	  1222.69	   168.15	  2
 21	 R 	        -  	    88.06	  1  _


+3 charge
		      B   	       Y
  1	 D 	    39.35	        -  	 21
  2	 T 	    73.03	   835.16	 20
  3	 V 	   106.05	   801.48	 19
  4	 C 	   159.44	   768.45	 18
  5	 Q 	   202.13	   715.07	 17
  6	 C 	   255.52	   672.38	 16
  7	 K 	   298.22	   618.99	 15
  8	 E 	   341.23	   576.29	 14
  9	 G 	   360.24	   533.28	 13
 10	 T 	   393.92	   514.27	 12
 11	 F 	   442.94	   480.59	 11
 12	 R 	   494.98	   431.57	 10
 13	 N 	   532.99	   379.53	  9
 14	 E 	   576.01	   341.52	  8
 15	 N 	   614.02	   298.50	  7
 16	 S 	   643.03	   260.49	  6
 17	 P 	   675.38	   231.48	  5
 18	 E 	   718.40	   199.13	  4
 19	 M 	   762.08	   156.11	  3
 20	 C 	   815.46	   112.43	  2
 21	 R 	        -  	    59.04	  1  _
```

**55. IPI00011662 Kunitz-type protease inhibitor 2**

| Sequence | Charge | M+H+ | XCorr | DeltaCn |
| --- | --- | --- | --- | --- |
| R.SIHDFCLVSK.V | 2 | 1206.367 | 2.961 | 0.359 |

+1 charge

```
		      B   	       Y
  1	 S 	    88.04	        -  	 10
  2	 I 	   201.12	  1118.70	  9
  3	 H 	   338.18	  1005.62	  8
  4	 D 	   453.21	   868.56	  7
  5	 F 	   600.28	   753.53	  6
  6	 C 	   760.44	   606.46	  5
  7	 L 	   873.53	   446.30	  4
  8	 V 	   972.60	   333.21	  3
  9	 S 	  1059.63	   234.14	  2
 10	 K 	        -  	   147.11	  1  _
+2 charge
		      B   	       Y
  1	 S 	    44.52	        -  	 10
  2	 I 	   101.07	   559.85	  9
  3	 H 	   169.59	   503.31	  8
  4	 D 	   227.11	   434.78	  7
  5	 F 	   300.64	   377.27	  6
  6	 C 	   380.73	   303.73	  5
  7	 L 	   437.27	   223.65	  4
  8	 V 	   486.80	   167.11	  3
  9	 S 	   530.32	   117.58	  2
 10	 K 	        -  	    74.06	  1  _
```

**56. IPI00433028 Isoform 1 of Leukocyte-associated immunoglobulin-like receptor 1**

| Sequence | Charge | M+H+ | XCorr | DeltaCn |
| --- | --- | --- | --- | --- |
| R.FRIDSVSEGNAGPYR.C | 2 | 1668.792 | 4.658 | 0.411 |

+1 charge

```
		      B   	       Y
  1	 F 	   148.08	        -  	 15
  2	 R 	   304.18	  1520.75	 14
  3	 I 	   417.26	  1364.64	 13
  4	 D 	   532.29	  1251.56	 12
  5	 S 	   619.32	  1136.53	 11
  6	 V 	   718.39	  1049.50	 10
  7	 S 	   805.42	   950.43	  9
  8	 E 	   934.46	   863.40	  8
  9	 G 	   991.48	   734.36	  7
 10	 N 	  1105.53	   677.34	  6
 11	 A 	  1176.56	   563.29	  5
 12	 G 	  1233.59	   492.26	  4
 13	 P 	  1330.64	   435.24	  3
 14	 Y 	  1493.70	   338.18	  2
 15	 R 	        -  	   175.12	  1  _
+2 charge
		      B   	       Y
  1	 F 	    74.54	        -  	 15
  2	 R 	   152.59	   760.88	 14
  3	 I 	   209.13	   682.83	 13
  4	 D 	   266.65	   626.28	 12
  5	 S 	   310.16	   568.77	 11
  6	 V 	   359.70	   525.25	 10
  7	 S 	   403.21	   475.72	  9
  8	 E 	   467.74	   432.20	  8
  9	 G 	   496.25	   367.68	  7
 10	 N 	   553.27	   339.17	  6
 11	 A 	   588.79	   282.15	  5
 12	 G 	   617.30	   246.63	  4
 13	 P 	   665.82	   218.12	  3
 14	 Y 	   747.35	   169.59	  2
 15	 R 	        -  	    88.06	  1  _
```

**57. IPI00022284 Major prion protein**

| Sequence | Charge | M+H+ | XCorr | DeltaCn |
| --- | --- | --- | --- | --- |
| R.VVEQMCITQYER.E | 2 | 1556.76 | 4.478 | 0.571 |

+1 charge

```
		      B   	       Y
  1	 V 	   100.08	        -  	 12
  2	 V 	   199.14	  1456.79	 11
  3	 E 	   328.19	  1357.72	 10
  4	 Q 	   456.25	  1228.68	  9
  5	 M 	   587.29	  1100.62	  8
  6	 C 	   747.45	   969.58	  7
  7	 I 	   860.54	   809.42	  6
  8	 T 	   961.58	   696.33	  5
  9	 Q 	  1089.64	   595.28	  4
 10	 Y 	  1252.70	   467.22	  3
 11	 E 	  1381.75	   304.16	  2
 12	 R 	        -  	   175.12	  1  _
+2 charge
		      B   	       Y
  1	 V 	    50.54	        -  	 12
  2	 V 	   100.08	   728.90	 11
  3	 E 	   164.60	   679.36	 10
  4	 Q 	   228.63	   614.84	  9
  5	 M 	   294.15	   550.81	  8
  6	 C 	   374.23	   485.29	  7
  7	 I 	   430.77	   405.21	  6
  8	 T 	   481.30	   348.67	  5
  9	 Q 	   545.32	   298.15	  4
 10	 Y 	   626.86	   234.12	  3
 11	 E 	   691.38	   152.58	  2
 12	 R 	        -  	    88.06	  1  _
```

**58. IPI00953695 Ig kappa chain V-II region GM607 (Fragment)**

| Sequence | Charge | M+H+ | XCorr | DeltaCn |
| --- | --- | --- | --- | --- |
| R.ASGVPDRFSGSGSGTDFTLK.I | 2 | 1987.116 | 4.217 | 0.486 |

+1 charge

```
		      B   	       Y
  1	 A 	    72.04	        -  	 20
  2	 S 	   159.08	  1914.92	 19
  3	 G 	   216.10	  1827.89	 18
  4	 V 	   315.17	  1770.87	 17
  5	 P 	   412.22	  1671.80	 16
  6	 D 	   527.25	  1574.74	 15
  7	 R 	   683.35	  1459.72	 14
  8	 F 	   830.42	  1303.62	 13
  9	 S 	   917.45	  1156.55	 12
 10	 G 	   974.47	  1069.52	 11
 11	 S 	  1061.50	  1012.49	 10
 12	 G 	  1118.52	   925.46	  9
 13	 S 	  1205.55	   868.44	  8
 14	 G 	  1262.58	   781.41	  7
 15	 T 	  1363.62	   724.39	  6
 16	 D 	  1478.65	   623.34	  5
 17	 F 	  1625.72	   508.31	  4
 18	 T 	  1726.77	   361.24	  3
 19	 L 	  1839.85	   260.20	  2
 20	 K 	        -  	   147.11	  1  _
+2 charge
		      B   	       Y
  1	 A 	    36.53	        -  	 20
  2	 S 	    80.04	   957.96	 19
  3	 G 	   108.55	   914.45	 18
  4	 V 	   158.09	   885.94	 17
  5	 P 	   206.61	   836.40	 16
  6	 D 	   264.13	   787.88	 15
  7	 R 	   342.18	   730.36	 14
  8	 F 	   415.71	   652.31	 13
  9	 S 	   459.23	   578.78	 12
 10	 G 	   487.74	   535.26	 11
 11	 S 	   531.25	   506.75	 10
 12	 G 	   559.76	   463.23	  9
 13	 S 	   603.28	   434.72	  8
 14	 G 	   631.79	   391.21	  7
 15	 T 	   682.32	   362.70	  6
 16	 D 	   739.83	   312.17	  5
 17	 F 	   813.36	   254.66	  4
 18	 T 	   863.89	   181.13	  3
 19	 L 	   920.43	   130.60	  2
 20	 K 	        -  	    74.06	  1  _
```

**59. IPI00216780 Cartilage intermediate layer protein 2 precursor**

| Sequence | Charge | M+H+ | XCorr | DeltaCn |
| --- | --- | --- | --- | --- |
| R.INLAKPRPGDPAEANGPVYPWR.S | 3 | 2419.726 | 4.084 | 0.378 |

+1 charge

```
		      B   	       Y
  1	 I 	   114.09	        -  	 22
  2	 N 	   228.13	  2305.18	 21
  3	 L 	   341.22	  2191.14	 20
  4	 A 	   412.26	  2078.06	 19
  5	 K 	   540.35	  2007.02	 18
  6	 P 	   637.40	  1878.92	 17
  7	 R 	   793.50	  1781.87	 16
  8	 P 	   890.56	  1625.77	 15
  9	 G 	   947.58	  1528.72	 14
 10	 D 	  1062.61	  1471.70	 13
 11	 P 	  1159.66	  1356.67	 12
 12	 A 	  1230.70	  1259.62	 11
 13	 E 	  1359.74	  1188.58	 10
 14	 A 	  1430.78	  1059.54	  9
 15	 N 	  1544.82	   988.50	  8
 16	 G 	  1601.84	   874.46	  7
 17	 P 	  1698.89	   817.44	  6
 18	 V 	  1797.96	   720.38	  5
 19	 Y 	  1961.02	   621.31	  4
 20	 P 	  2058.08	   458.25	  3
 21	 W 	  2244.16	   361.20	  2
 22	 R 	        -  	   175.12	  1  _
+2 charge
		      B   	       Y
  1	 I 	    57.55	        -  	 22
  2	 N 	   114.57	  1153.10	 21
  3	 L 	   171.11	  1096.07	 20
  4	 A 	   206.63	  1039.53	 19
  5	 K 	   270.68	  1004.01	 18
  6	 P 	   319.21	   939.97	 17
  7	 R 	   397.26	   891.44	 16
  8	 P 	   445.78	   813.39	 15
  9	 G 	   474.29	   764.86	 14
 10	 D 	   531.81	   736.35	 13
 11	 P 	   580.33	   678.84	 12
 12	 A 	   615.85	   630.31	 11
 13	 E 	   680.37	   594.79	 10
 14	 A 	   715.89	   530.27	  9
 15	 N 	   772.91	   494.75	  8
 16	 G 	   801.42	   437.73	  7
 17	 P 	   849.95	   409.22	  6
 18	 V 	   899.48	   360.70	  5
 19	 Y 	   981.02	   311.16	  4
 20	 P 	  1029.54	   229.63	  3
 21	 W 	  1122.58	   181.10	  2
 22	 R 	        -  	    88.06	  1  _
+3 charge
		      B   	       Y
  1	 I 	    38.70	        -  	 22
  2	 N 	    76.72	   769.07	 21
  3	 L 	   114.41	   731.05	 20
  4	 A 	   138.09	   693.36	 19
  5	 K 	   180.79	   669.68	 18
  6	 P 	   213.14	   626.98	 17
  7	 R 	   265.17	   594.63	 16
  8	 P 	   297.52	   542.60	 15
  9	 G 	   316.53	   510.24	 14
 10	 D 	   354.87	   491.24	 13
 11	 P 	   387.22	   452.89	 12
 12	 A 	   410.90	   420.54	 11
 13	 E 	   453.92	   396.86	 10
 14	 A 	   477.60	   353.85	  9
 15	 N 	   515.61	   330.17	  8
 16	 G 	   534.62	   292.16	  7
 17	 P 	   566.97	   273.15	  6
 18	 V 	   599.99	   240.80	  5
 19	 Y 	   654.35	   207.78	  4
 20	 P 	   686.70	   153.42	  3
 21	 W 	   748.72	   121.07	  2
 22	 R 	        -  	    59.04	  1  _
```

**60. IPI00024035 Isoform 1 of Cadherin-6**

| Sequence | Charge | M+H+ | XCorr | DeltaCn |
| --- | --- | --- | --- | --- |
| R.IVVEDVDEPPVFSK.L | 2 | 1573.769 | 2.975 | 0.546 |

+1 charge

```
		      B   	       Y
  1	 I 	   114.09	        -  	 14
  2	 V 	   213.16	  1459.73	 13
  3	 V 	   312.23	  1360.66	 12
  4	 E 	   441.27	  1261.59	 11
  5	 D 	   556.30	  1132.55	 10
  6	 V 	   655.37	  1017.53	  9
  7	 D 	   770.39	   918.46	  8
  8	 E 	   899.44	   803.43	  7
  9	 P 	   996.49	   674.39	  6
 10	 P 	  1093.54	   577.33	  5
 11	 V 	  1192.61	   480.28	  4
 12	 F 	  1339.68	   381.21	  3
 13	 S 	  1426.71	   234.14	  2
 14	 K 	        -  	   147.11	  1  _
+2 charge
		      B   	       Y
  1	 I 	    57.55	        -  	 14
  2	 V 	   107.08	   730.37	 13
  3	 V 	   156.62	   680.84	 12
  4	 E 	   221.14	   631.30	 11
  5	 D 	   278.65	   566.78	 10
  6	 V 	   328.19	   509.27	  9
  7	 D 	   385.70	   459.73	  8
  8	 E 	   450.22	   402.22	  7
  9	 P 	   498.75	   337.70	  6
 10	 P 	   547.27	   289.17	  5
 11	 V 	   596.81	   240.64	  4
 12	 F 	   670.34	   191.11	  3
 13	 S 	   713.86	   117.58	  2
 14	 K 	        -  	    74.06	  1  _
```

**61. IPI00387113 Ig kappa chain V-III region B6**

| Sequence | Charge | M+H+ | XCorr | DeltaCn |
| --- | --- | --- | --- | --- |
| - .ZIVLTZSPGTLSLSPGZR.A | 2 | 1884.616 | 4.454 | 0.604 |

+1 charge

```
		      B   	       Y
  1	 Z 	   129.56	        -  	 18
[truncated: 2,000,410 more chars]
